# MicroRNA-26b-5p Inhibits Mouse Liver Fibrogenesis and Angiogenesis by Targeting PDGF Receptor-Beta

**DOI:** 10.1016/j.omtn.2019.02.014

**Published:** 2019-02-26

**Authors:** Le Yang, Chengbin Dong, Jingjing Yang, Lin Yang, Na Chang, Changbo Qi, Liying Li

**Affiliations:** 1 Department of Cell Biology, Municipal Laboratory for Liver Protection and Regulation of Regeneration, Capital Medical University, Beijing 100069, China

**Keywords:** microRNA-26b-5p, long non-coding RNA maternally expressed gene 3, bone marrow mesenchymal stromal cell, platelet-derived growth factor receptor-beta, angiogenesis, liver fibrosis

## Abstract

Here microRNAs (miRNAs) with potentially therapeutic effects were screened and explored during liver fibrogenesis and angiogenesis via targeting the important mediators. Chimera mice with EGFP^+^ bone marrow mesenchymal stromal cells (BMSCs) were fed with methionine-choline-deficient and high-fat (MCDHF) diet to induce liver injury. Increased expression of platelet-derived growth factor receptor-beta (PDGFR-β) was detected in MCDHF mice, with a positive correlation to fibrosis and angiogenesis markers. BMSCs contributed to the significant proportion of PDGFR-β^+^ cells in the fibrotic liver. MicroRNA-26b-5p (miR-26b-5p) was predicted to target PDGFR-β from three databases. The hepatic expression of miR-26b-5p was decreased in the fibrotic liver, with a negative correlation to PDGFR-β and fibrosis and angiogenesis markers. miR-26b-5p directly targeted PDGFR-β in TGF-β1-treated BMSCs by pull-down and lucifer reporter assays, which can be sponged by long non-coding RNA (lncRNA) maternally expressed gene 3 (lncMEG3). Microarray analysis revealed that miR-26b-5p overexpression affected a list of genes associated with fibrosis and angiogenesis. *In vivo* miR-26b-5p negatively regulated PDGFR-β expression and attenuated liver fibrosis and angiogenesis. Together, miR-26b-5p inhibits liver fibrogenesis and angiogenesis via directly targeting PDGFR-β and interacting with lncMEG3, which may represent an effective therapeutic strategy for liver fibrosis.

## Introduction

MicroRNAs (miRNAs) are single-stranded, small (21- to 25-nt), non-coding RNAs that target mRNAs with complementary sequences, and they inhibit the expression of target genes at the post-transcriptional level.^1^ The binding of miRNA in the form of the RNA-induced silencing complex with the 3′ UTR) of its target mRNA represses protein synthesis by translational inhibition and mRNA cleavage or decay. miRNAs are involved in a variety of physiological and pathological processes, and they are regarded as a new diagnostic tool. miR-29b mediates the lung mesenchymal-epithelial transition and prevents lung fibrosis in the silicosis model.^2^ miR-202-3p controls human Sertoli cell functions and spermatogenesis via targeting LRP6 and Cyclin D1 of the Wnt, β-catenin-signaling pathway.^3^ Especially, miRNAs have recently attracted increasing attention in liver disorder. miR-122 stimulates the expression of 24 hepatocyte-specific genes during hepatocyte maturation, and it regulates hepatocyte proliferation and differentiation in liver regeneration.^4^, ^5^, ^6^ miR-34a, which is increased in the serum of patients with non-alcoholic fatty liver disease, targets peroxisome proliferator-activated receptor gamma and regulates lipid transport and metabolism, playing a key role in the mitochondrial β-oxidation pathway.^7^, ^8^ However, key miRNAs associated with liver fibrosis and accompanied angiogenesis have not been fully identified and deciphered yet.

Liver fibrosis is a common feature of almost all chronic liver diseases, which eventually leads to the development of cirrhosis and related complications, including hepatocellular carcinoma.^9^ Angiogenesis is intrinsically associated with the progression of chronic liver diseases,^10^, ^11^ and our previous work has demonstrated that the inhibition of angiogenesis resulted in the attenuation of hepatic fibrosis in carbon tetrachloride and bile duct ligation mouse fibrotic models.^12^ During liver fibrogenesis, many key fibrotic genes, such as transforming growth factor-β1 (TGF-β1), α-smooth muscle actin (α-SMA), procollagen α1(I) (Col α1[I]), and procollagen α1(III) (Col α1[III]), and angiogenic genes, such as angiopoietin 1 (Angpt1), CD31, and vascular cell adhesion molecule-1 (VCAM-1), are activated. Therefore, targeting the critical regulation factors in the development of hepatic fibrosis would be viewed as a promising strategy for the treatment of liver disease.

Platelet-derived growth factor receptor-beta (PDGFR-β) is a cell surface receptor tyrosine kinase, and the biological effect of it is initiated via binding to its ligand platelet-derived growth factor-B or platelet-derived growth factor-D.^13^, ^14^, ^15^, ^16^ PDGFR-β is best known as a regulator of vascular morphogenesis and function during various development, homeostasis, and disease processes.^17^, ^18^, ^19^, ^20^

For instance, during angiogenesis, endothelial cell-derived platelet-derived growth factor B recruits perivascular cells onto angiogenic blood vessels through the activation of PDGFR-β.^21^, ^22^, ^23^ Additionally, PDGFR-β plays a crucial role in the pathological processes of chronic liver diseases, and inhibition of PDGFR-β signaling by destruxin A5 represents an appealing target for the treatment of bile duct ligation-induced liver fibrosis.^24^ However, the origin of numerous PDGFR-β^+^ cells in the fibrotic liver, especially in fat liver disease and associated angiogenesis, and the exact molecular mechanism governing this process have not been clearly deciphered yet.

Here we perform a large-scale screen for miRNAs targeting PDGFR-β by bioinformatics analysis, and we investigate the potential regulatory mechanism of candidate miRNAs in liver fibrogenesis and angiogenesis. These data expand our understanding of miRNA and PDGFR-β during chronic liver injury, which may represent an effective therapeutic strategy for liver disease.

## Results

### Increased Expression of PDGFR-β Was Detected in Methionine-Choline-Deficient and High-Fat Mice, with a Positive Correlation to Fibrosis and Angiogenesis Markers

**Figure 1:**
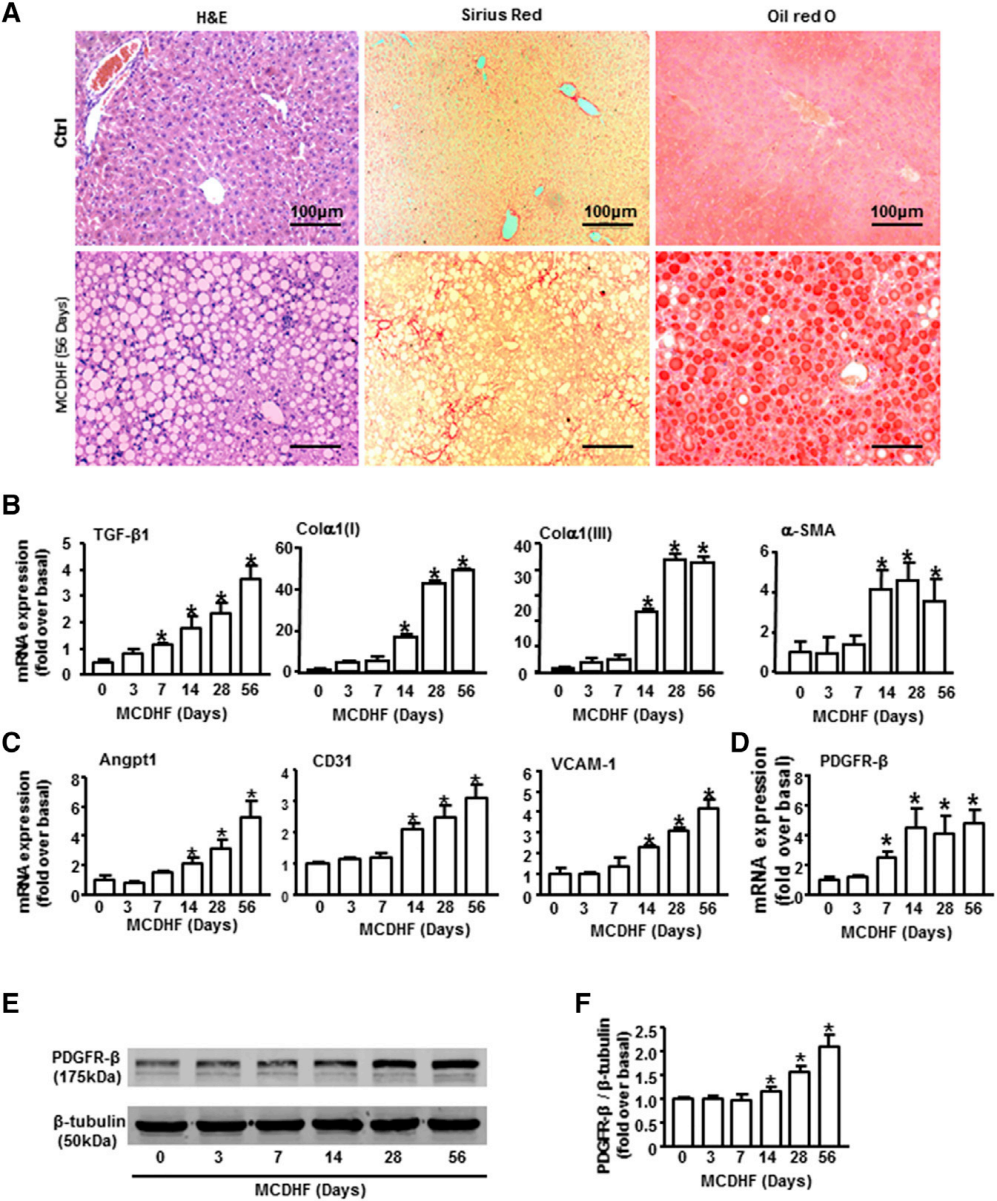
The Expression Pattern of PDGFR-β in the Fibrotic Liver of MCDHF Mice and the Correlation between PDGFR-β and Fibrosis and Angiogenesis Markers (A) Representative H&E, Sirius Red, and oil red O stainings in the fibrotic liver of MCDHF mice. The mRNA expressions of fibrosis markers (B), angiogenesis markers (C), and PDGFR-β (D) were examined by qRT-PCR in the fibrotic liver of MCDHF mice. PDGFR-β protein expression was examined (E) and quantified (F) by western blot in the fibrotic liver. Data are presented as the mean ± SEM. n = 6 per group. *p < 0.05 versus control.

Since fat liver injury has become a rising cause of chronic liver disease worldwide,^25^, ^26^ methionine-choline-deficient and high-fat (MCDHF) diet was fed to mice for different times to induce hepatic fibrosis. Liver histology, excessive collagen deposition, and lipid accumulation were demonstrated by H&E, Sirius Red, and oil red O staining in the fibrotic liver of 56-day MCDHF mice (Figure 1A). In line with this, the increased mRNA expression of fibrosis markers, including TGF-β1, α-SMA, Col α1(I), and Col α1(III), was observed in the fibrotic liver (Figure 1B), indicating the successful establishment of mouse fibrotic models by feeding an MCDHF diet. The mRNA levels of angiogenesis markers, including Angpt1, CD31, and VCAM-1, were also markedly upregulated in the damaged liver, which was consistent with the progression of liver fibrosis (Figure 1C). Notably, the expression of PDGFR-β increased nearly 2.5-fold at 7 days of MCDHF diet and continuously increased throughout the entire stage of chronic liver injury, reaching its peak around 56 days (4.8-fold) (Figure 1D); thus, we chose 56 days of MCDHF diet in the following studies. In addition, a positive correlation between PDGFR-β and fibrosis or angiogenesis markers was observed (Table 1). The protein levels of PDGFR-β in MCDHF mouse liver also increased significantly, with the maximal increase (2.62-fold) at 56 days after MCDHF diet feeding (Figures 1E and 1F). These results showed remarkable upregulation of PDGFR-β in MCDHF mouse liver, which was closely correlated with liver fibrogenesis and angiogenesis.

**Table 1:** The Correlation between PDGFR-β or miR-26b-5p and Fibrosis or Angiogenesis Markers in the Liver

Parameter		PDGFR-β		miR-26b-5p	
		Correlation Coefficient (r)	p Value	Correlation Coefficient (r)	p Value
Fibrosis markers	TGF-β1	0.897	<0.01	−0.831	<0.01
	Col α1(I)	0.835	<0.01	−0.808	<0.01
	Col α1(III)	0.883	<0.01	−0.881	<0.01
	α-SMA	0.823	<0.01	−0.854	<0.01
Angiogenesis markers	Angpt1	0.862	<0.01	−0.794	<0.01
	CD31	0.785	<0.01	−0.807	<0.01
	VCAM-1	0.850	<0.01	−0.868	<0.01

The relative expression of PDGFR-β and miR-26b-5p and fibrosis and angiogenesis markers in the liver of MCDHF mice was quantified using qRT-PCR. The relationship between PDGFR-β and miR-26b-5p and fibrosis and angiogenesis markers was analyzed by regression analysis.

### Bone Marrow Mesenchymal Stromal Cells Contributed to the Significant Proportion of PDGFR-β^+^ Cells in the Mouse Fibrotic Liver

**Figure 2:**
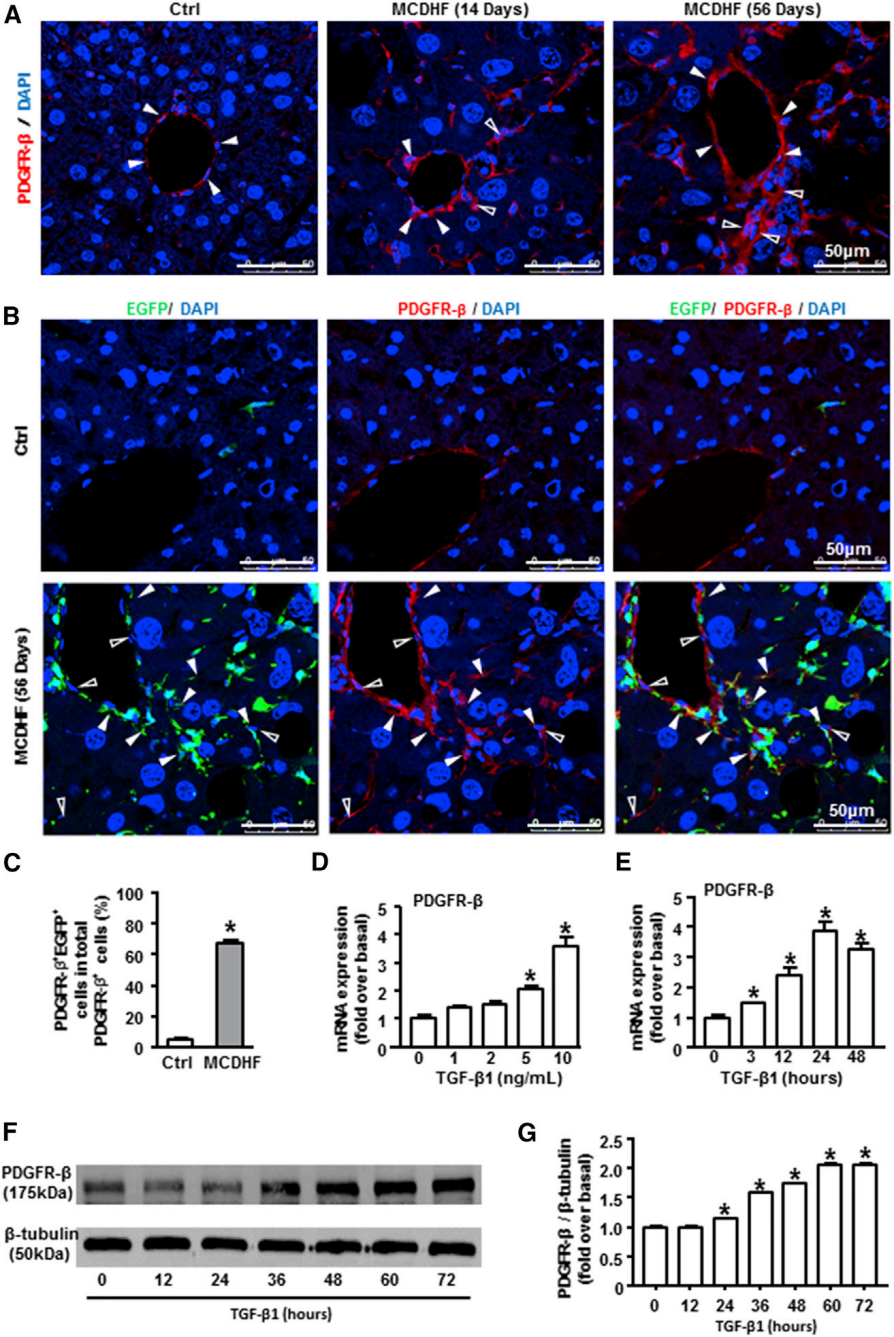
The Origin of PDGFR-β^+^ Cells in MCDHF Fibrotic Mice and the Expression of PDGFR-β in TGF-β1-Treated BMSCs *In Vivo* (A) Representative images of immunofluorescence analysis to track PDGFR-β (green) expression in the fibrotic liver. Hollow arrows indicate PDGFR-β expression around the newly formed vessels, while solid arrows indicate PDGFR-β expression around the existing mature vessels. (B) Immunofluorescence staining for PDGFR-β (red) to track PDGFR-β^+^ cells following 56 days of MCDHF diet. (C) The proportion of PDGFR-β^+^EGFP^+^ cells accounting for total PDGFR-β^+^ cells was measured by Image-Pro Plus software. (D) PDGFR-β mRNA expression was examined by qRT-PCR in BMSCs treated with the indicated concentrations of TGF-β1 for 24 h. (E) PDGFR-β mRNA expression in BMSCs treated with 10 ng/mL TGF-β1 at different times. (F and G) PDGFR-β protein expression was examined (F) and quantified (G) by western blot in TGF-β1-treated BMSCs. Data are presented as the mean ± SEM. n = 6 per group. *p < 0.05 versus control.

Immunofluorescence staining showed that PDGFR-β was only expressed in the vascular area of control liver with a weak immunoreactivity. However, there was strong immunoreactivity for PDGFR-β not only around mature vessels (solid arrows) but also around the area of nascent blood vessels (hollow arrows, Figure 2A) in the liver of MCDHF mice.

Previous studies have revealed that bone marrow mesenchymal stromal cells (BMSCs) can migrate to the damaged liver and distribute in the fibrotic area (angiogenic area).^27^, ^28^, ^29^ To explore whether PDGFR-β was highly expressed on these recruited BMSCs in the fibrotic liver, chimera mice with EGFP^+^ BMSCs were fed an MCDHF diet for 56 days to induce liver fibrosis, then the hepatic expression of PDGFR-β was examined by immunofluorescence staining. In control liver, few EGFP^+^ cells were detected and no co-localization was observed between PDGFR-β^+^ and EGFP^+^ cells (Figure 2B). In contrast, a large proportion of PDGFR-β^+^ cells were positive for EGFP, with the percentage of PDGFR-β^+^EGFP^+^ cells accounting for 72.5% of total PDGFR-β^+^ cells (Figure 2C), indicating there was a large proportion of PDGFR-β^+^ cells in mouse fibrotic liver derived from BMSCs.

To validate the expression of PDGFR-β *in vitro*, we treated primary BMSCs with TGF-β1, which is an important regulator of liver fibrosis and angiogenesis. qRT-PCR analysis revealed a pronounced increase in PDGFR-β mRNA expression stimulated by TGF-β1 in a dose-dependent manner, with the maximal upregulation at a concentration of 10 ng/mL TGF-β1 (Figure 2D). Then BMSCs were stimulated by 10 ng/mL TGF-β1 for different times, showing a progressive increase in PDGFR-β from 3 to 24 h, with the highest expression at 24 h (Figure 2E). Afterward, PDGFR-β protein levels were examined by western blot. TGF-β1 treatment markedly upregulated PDGFR-β protein amounts from 12 h, and this elevated level of PDGFR-β persisted for at least 72 h (Figures 2F and 2G), further confirming the upregulation of PDGFR-β in BMSCs under the treatment of TGF-β1 *in vitro*.

### miR-26b-5p Directly Targeted PDGFR-β in TGF-β1-Treated BMSCs

To identify the miRNAs targeting PDGFR-β, we intersected the predicted miRNAs from three databases (www.microrna.org/microrna/home.do, www.targetscan.org/mmu_72, and https://cm.jefferson.edu/rna22/), and we got five putative miRNAs for target gene PDGFR-β. Among them, one was unchanged (miR-1934-3p), one was not detected (miR-192-3p), and the other three were downregulated (miR-30b-5p, miR-26b-5p, and miR-101b-3p) after TGF-β1 treatment in BMSCs. Based on the fact that miR-26b-5p had the relatively lower expression in TGF-β1-treated BMSCs and the better binding scores with PDGFR-β, we chose miR-26b-5p to focus on its regulation of PDGFR-β in the follow-up studies.

**Figure 3:**
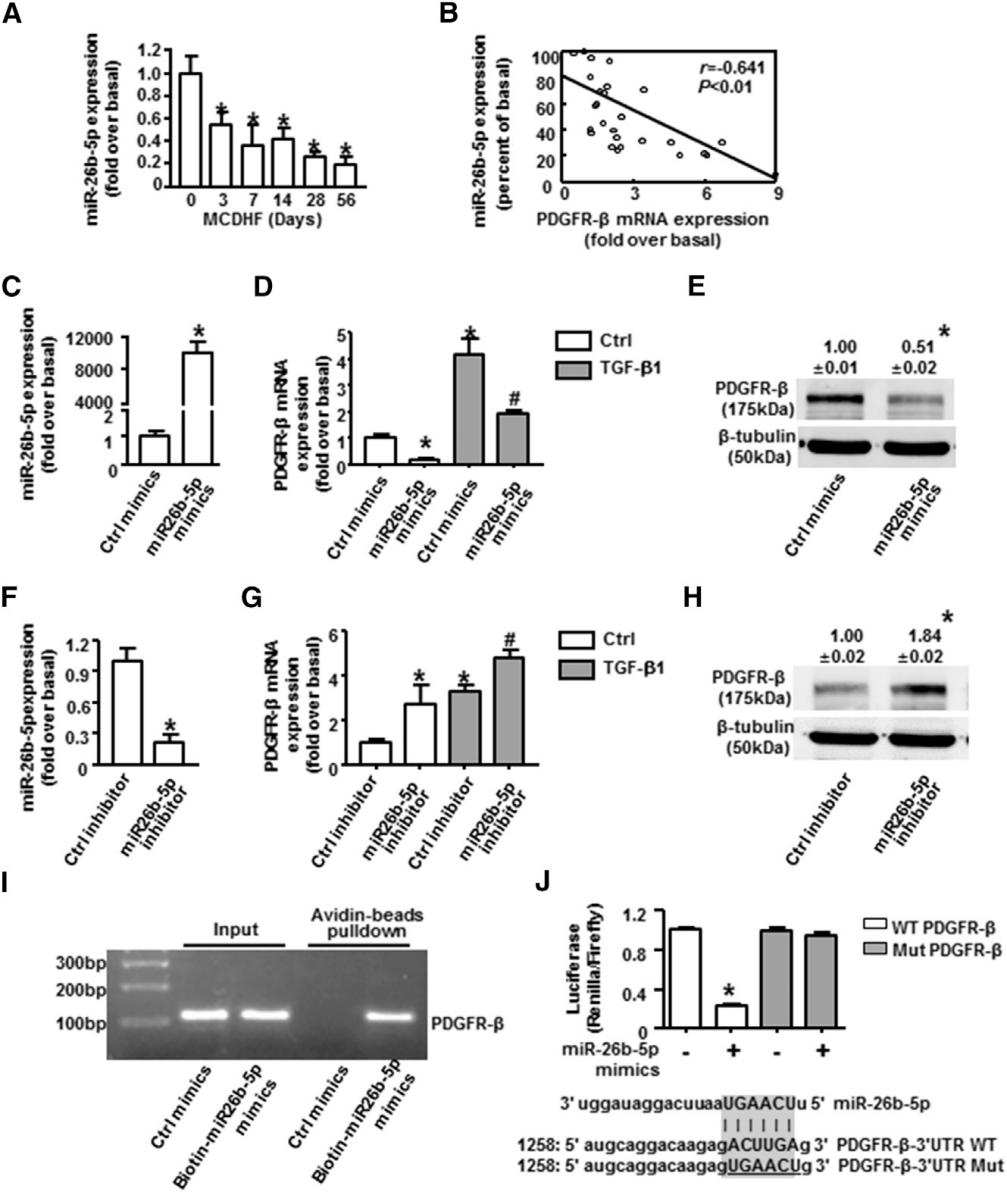
Downregulation of PDGFR-β Expression by miR-26b-5p in TGF-β1-Treated BMSCs (A) miR-26b-5p expression in the fibrotic liver induced by MCDHF. (B) The correlation between miR-26b-5p and PDGFR-β in liver tissue. Transfection efficiency of miR-26b-5p mimic (C) or inhibitor (F) in BMSCs is shown. PDGFR-β mRNA expression was examined by qRT-PCR with or without miR-26b-5p mimic (D) or inhibitor (G) transfection in BMSCs. Protein expression was examined by western blot with miR-26b-5p mimic (E) or inhibitor (H) transfection in BMSCs. Biotin-avidin pull-down assay (I) and luciferase reporter assay (J) demonstrated PDGFR-β was a target of miR-26b-5p. Data are presented as the mean ± SEM. n = 6 per group. *p < 0.05 versus control. #p < 0.05 versus TGF-β1 treated alone.

The hepatic expression of miR-26b-5p was decreased in the fibrotic liver of MCDHF mice (Figure 3A), with a negative correlation to PDGFR-β (Figure 3B) and fibrosis and angiogenesis markers (Table 1). As we know, miRNAs usually exert their effects through transcriptional regulation of downstream target genes.^30^ To confirm PDGFR-β was directly targeted and regulated by miR-26b-5p, BMSCs were transfected with miR-26b-5p mimic or inhibitor to alter its expression, and the transfected efficiency was verified by qRT-PCR analysis (Figures 3C and 3F). Overexpression of miR-26b-5p with its mimic inhibited the TGF-β1-induced increase of PDGFR-β both in mRNA (Figure 3D) and protein levels (Figure 3E), while miR-26b-5p inhibition resulted in the upregulation of PDGFR-β mRNA (Figure 3G) and protein expressions (Figure 3H). A biotin-avidin pull-down assay uncovered that PDGFR-β could be pulled down by wild-type bio-miR-26b-5p, whereas it could not be pulled down by the bio-control (Figure 3I). Lucifer reporter assays were used to further validate that PDGFR-β was a direct target of miR-26b-5p. Luciferase reporter genes were constructed using the PDGFR-β 3′ UTR and the mutant counterpart at the miR-26b-5p-binding regions, and miR-26b-5p mimic was co-transfected into the cells. Overexpression of miR-26b-5p significantly inhibited the luciferase activity of PDGFR-β with the wild-type 3′ UTR, but not with mutant 3′ UTR (Figure 3J), providing clear evidence that PDGFR-β was a direct and functional downstream target of miR-26b-5p.

### miR-26b-5p Was Sponged by lncMEG3 in TGF-β1-Treated BMSCs

**Figure 4:**
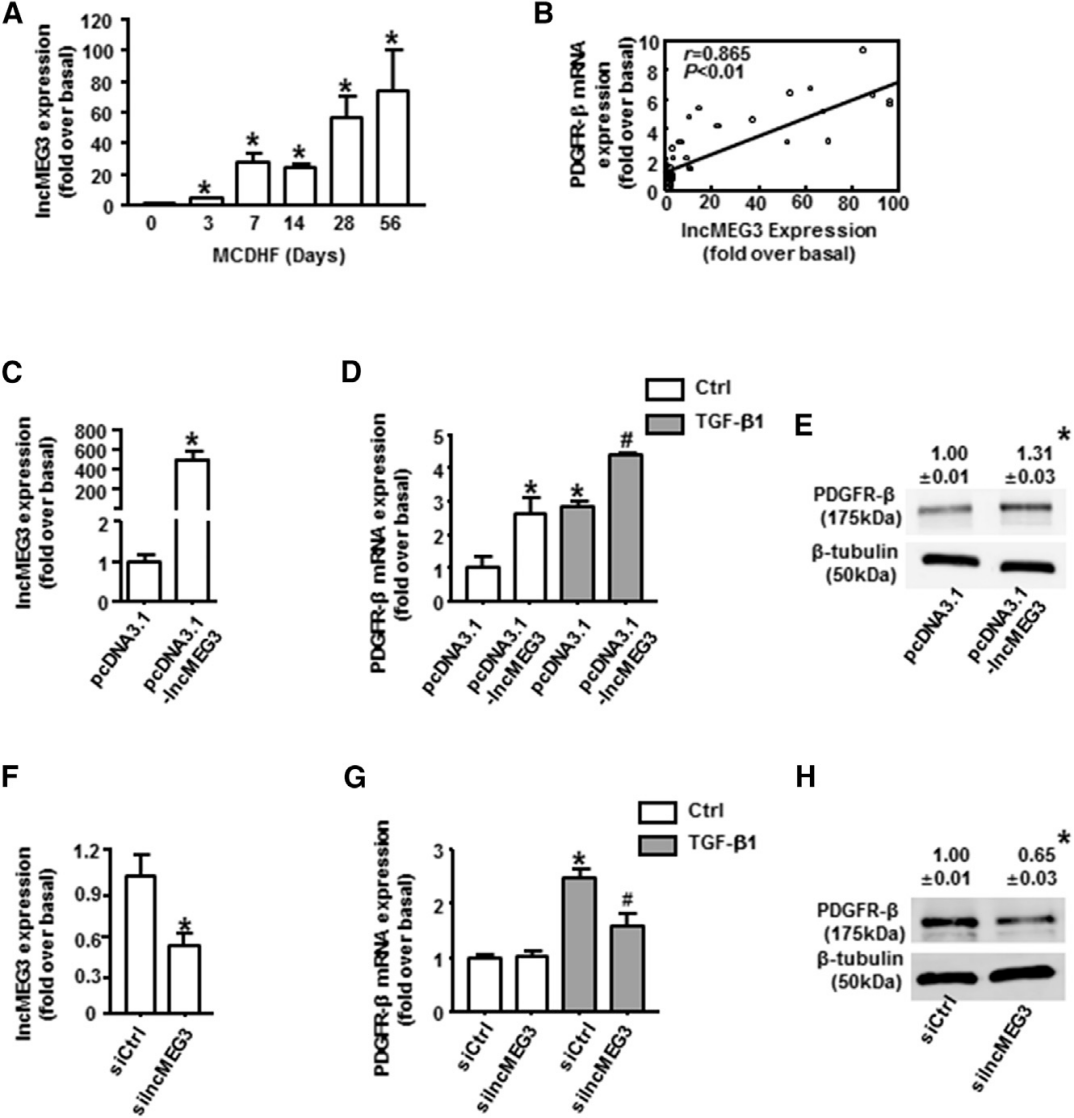
Regulation of lncMEG3 on PDGFR-β in TGF-β1-Treated BMSCs (A) lncMEG3 expression was examined by qRT-PCR in the fibrotic liver induced by MCDHF. (B) The correlation between lncMEG3 and PDGFR-β in liver tissue. Transfection efficiency of lncMEG3 overexpression plasmid (C) or siRNA (F) in BMSCs is shown. PDGFR-β mRNA expression was examined by qRT-PCR with lncMEG3 overexpression plasmid (D) or siRNA (G) in BMSCs. PDGFR-β protein expression was examined by western blot with lncMEG3 overexpression plasmid (E) or siRNA (H) in BMSCs. Data are presented as the mean ± SEM. n = 6 per group. *p < 0.05 versus control. #p < 0.05 versus TGF-β1 treated alone.

Recently, many efforts have been focused on the effects of long non-coding RNA maternally expressed gene 3 (lncMEG3) on angiogenesis,^31^, ^32^, ^33^ but whether lncMEG3 was involved in the modulation of PDGFR-β in BMSCs and liver fibrosis-associated angiogenesis was still unclear. To investigate lncMEG3 expression levels in liver fibrosis, we performed qRT-PCR analysis, and we observed strongly increased expression of lncMEG3 in the fibrotic liver of MCDHF mice (Figure 4A), indicating the importance of lncMEG3 during liver fibrogenesis. Moreover, the correlation between lncMEG3 and PDGFR-β was analyzed, with the Pearson correlation coefficient (r) of 0.865 (Figure 4B).

To explore the potential role of lncMEG3 in the regulation of PDGFR-β *in vitro*, BMSCs were transfected with lncMEG3 overexpression plasmid or specific small interfering RNA (siRNA) to alter its expression, and the transfected efficiency was verified by qRT-PCR analysis (Figures 4C and 4F). lncMEG3 overexpression significantly attenuated the TGF-β1-induced increase in PDGFR-β both in mRNA (Figure 4D) and protein levels (Figure 4E), as demonstrated by qRT-PCR and western blot analysis. In a parallel approach, lncMEG3 knockdown with its specific siRNA caused a significant reduction in PDGFR-β mRNA (Figure 4G) and protein expressions (Figure 4H) in TGF-β1-treated BMSCs. Altogether, these results substantiated the presumptive role of lncMEG3 on the regulation of PDGFR-β expression in BMSCs.

**Figure 5:**
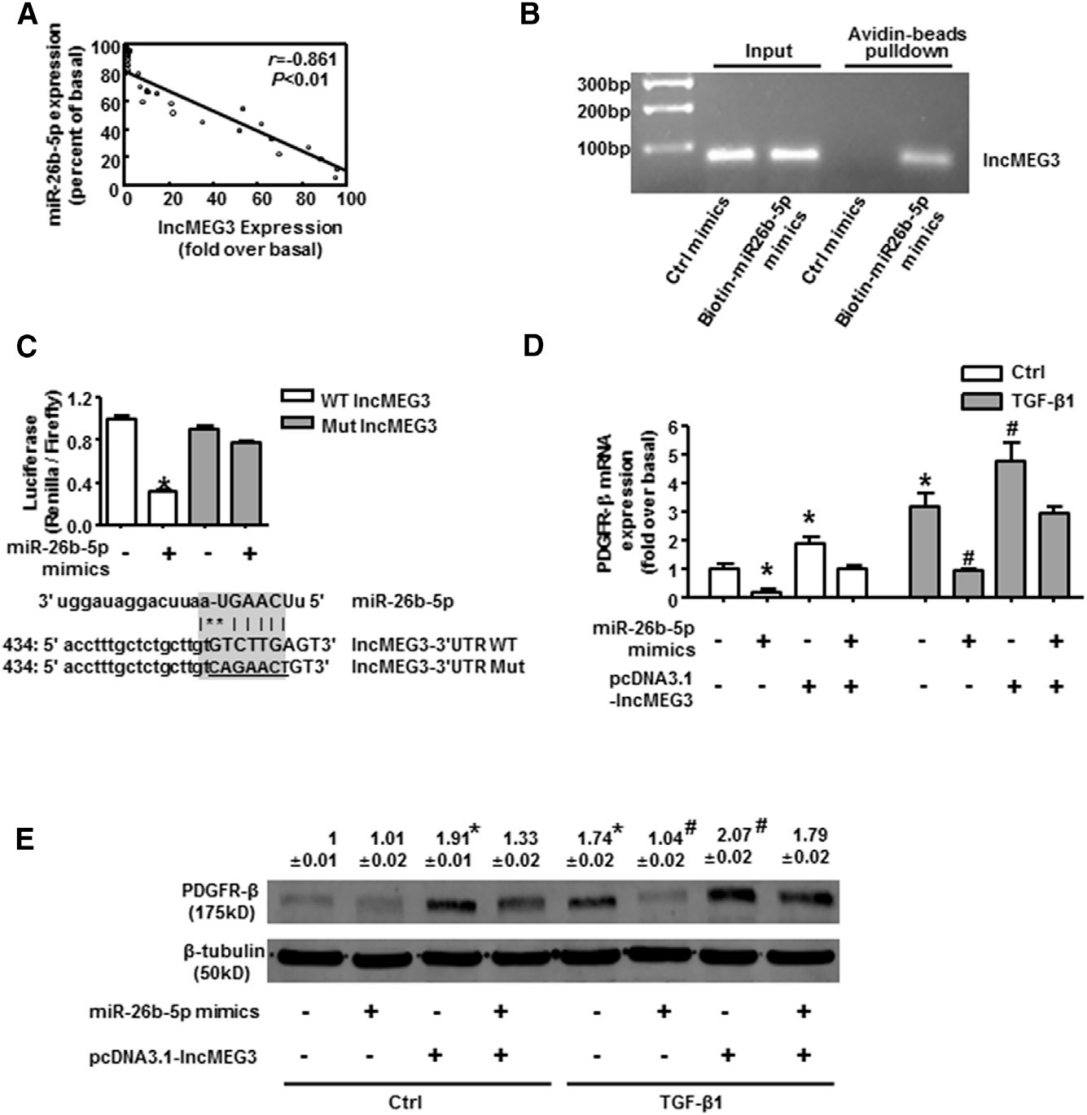
miR-26b-5p Was Sponged by lncMEG3 in TGF-β1-Treated BMSCs (A) The correlation between lncMEG3 and miR-26b-5p in liver tissue. Biotin-avidin pull-down assay (B) and luciferase reporter assay (C) demonstrated that lncMEG3 was a target of miR-26b-5p. (D) PDGFR-β mRNA expression with or without lncMEG3 overexpression plasmids in miR-26b-5p mimic-treated BMSCs. (E) PDGFR-β protein expression with or without lncMEG3 overexpression plasmids in miR-26b-5p mimic-treated BMSCs. Data are presented as the mean ± SEM. n = 6 per group. *p < 0.05 versus control. #p < 0.05 versus miR-26b-5p mimic treated alone.

Long non-coding RNA (lncRNA) can bind to miRNA and serve as a sponge to suppress miRNA action.^34^ Microarray analysis compared the change of lncRNA expression pattern, showing lncMEG3 ranked high among the downregulated lncRNAs in the microarray data of miR-26b-5p mimic-treated BMSCs (Table S1), indicating the possible interaction between lncMEG3 and miR-26b-5p. Consistently, there was a negative correlation between lncMEG3 and miR-26b-5p expression in liver, with the Pearson correlation coefficient (r) of −0.861 (Figure 5A). The biotin-avidin pull-down assay demonstrated that lncMEG3 could be pulled down by wild-type bio-miR-26b-5p, whereas it could not be pulled down by the bio-control (Figure 5B). Lucifer reporter assays suggested that miR-26b-5p mimic suppressed the luciferase activity of lncMEG3 with the wild-type 3′ UTR, but not with its mutant 3′ UTR (Figure 5C), further confirming that lncMEG3 could be directly bound by miR-26b-5p in BMSCs. Rescue experiments showed that co-transfection of lncMEG3 overexpression plasmid with miR-26b-5p mimic partially rescued the mRNA (Figure 5D) and protein levels (Figure 5E) of PDGFR-β, which was significantly decreased by individual miR-26b-5p mimics in TGF-β1-treatd BMSCs. Collectively, these results demonstrated that miR-26b-5p was sponged by lncMEG3 in TGF-β1-treated BMSCs.

### Microarray Analysis Was Performed in TGF-β1-Treated BMSCs with or without miR-26b-5p Mimics

**Figure 6:**
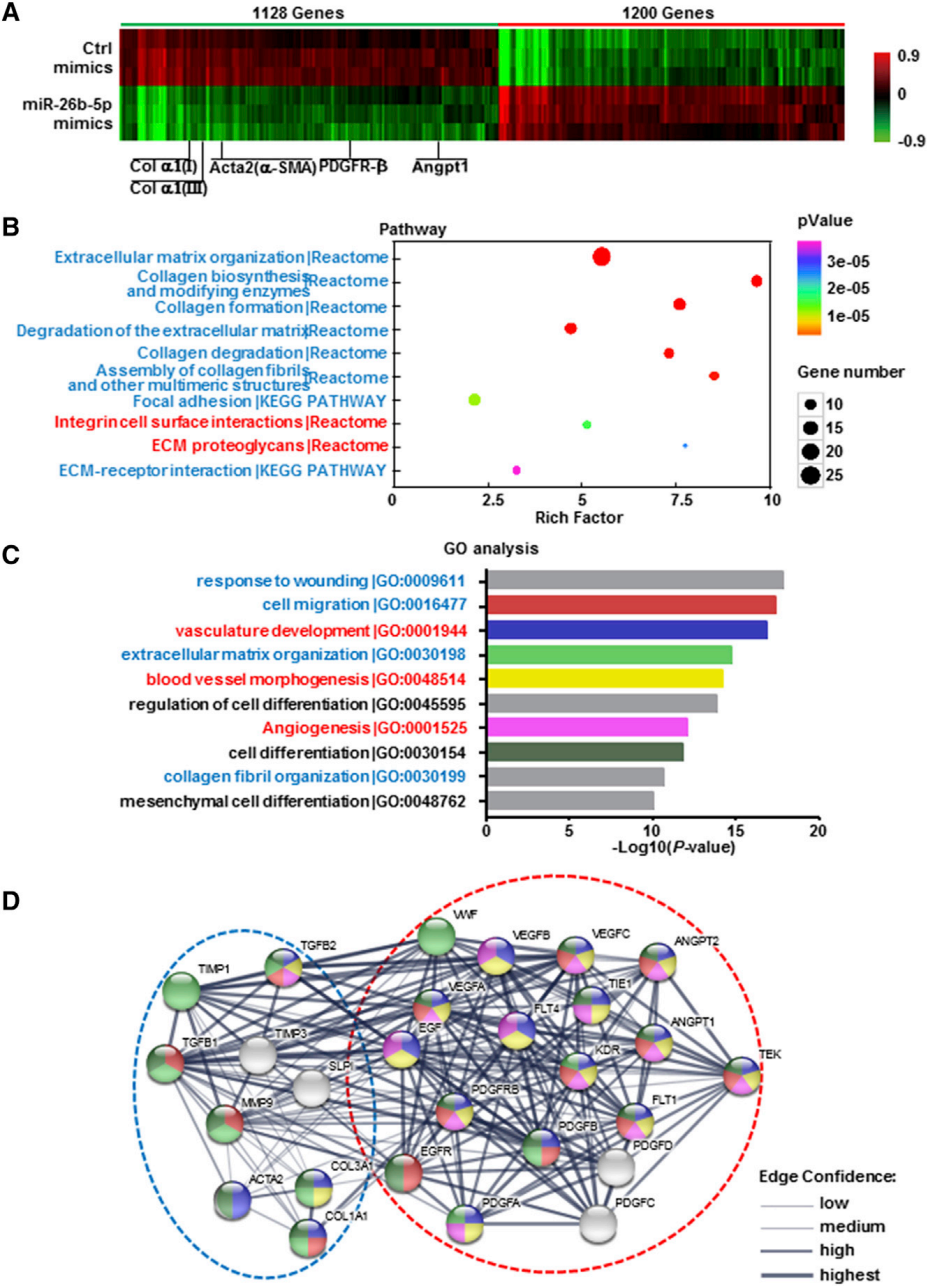
Microarray Analysis Was Performed in TGF-β1-Treated BMSCs with or without miR-26b-5p Mimics BMSCs were treated with 10 ng/mL TGF-β1 for 24 h with or without miR-26b-5p mimics. Microarray analysis for mRNA was performed with RNA extracted from BMSCs. Hierarchical cluster analysis of significantly differentially expressed mRNA is shown as follows: bright green, underexpression; black, no change; bright red, overexpression. n = 3 per group. (A) Microarray heatmap comparing the signatures of PDGFR-β and angiogenesis marker and fibrosis marker genes in TGF-β1-treated BMSCs with or without miR-26b-5p mimics. KEGG and Reactome enrichment analyses (B) and GO enrichment analysis for biological process terms (C) are for the differentially expressed genes with miR-26b-5p mimics. n = 3 per group. (D) Predicted protein network visualization with STRING. The network view predicted the associations between proteins from the regulated genes involved in angiogenesis and extracellular matrix organization in TGF-β1-treated BMSCs. The network nodes were proteins. These proteins were clustered using k-means clustering algorithms.

To gain further insights into the mechanism by which miR-26b-5p regulated PDGFR-β, we performed a microarray experiment using the RNA of TGF-β1-treated BMSCs with or without miR-26b-5p mimic, and we explored mRNA expression profiles. Hierarchical clustering showed systematic variations in transcript expression levels between miR-26b-5p mimic-treated and untreated BMSCs, with 1,200 mRNAs upregulated and 1,128 mRNAs downregulated after the transfection of miR-26b-5p mimic. The expression of PDGFR-β was distinctly decreased after miR-26b-5p overexpression in TGF-β1-treatd BMSCs in the microarray data (Figure 6A; Table S2), which was consistent with previous qRT-PCR and western blot results. In addition, the expression of angiogenic markers (Angpt1) and fibrotic markers (α-SMA, Col α1[I], and Col α1[III]) also declined with the transfection of miR-26b-5p mimic in TGF-β1-treated BMSCs (Figure 6A).

Kyoto Encyclopedia of Genes and Genomes (KEGG) and Reactome enrichment analyses indicated that the differentially expressed genes were closely related to fibrosis, including collagen formation and extracellular matrix organization (Figure 6B; Table S3). Gene Ontology (GO) enrichment analysis for biological process terms revealed that miR-26b-5p overexpression affected a list of genes associated with fibrosis and angiogenesis (Figure 6C; Table S4). Altogether, these results verified that miR-26b-5p directly targeted and downregulated PDGFR-β and the differentially expressed genes affected by miR-26b-5p were associated with fibrosis and angiogenesis by microarray analysis.

To better visualize the functional interactions between proteins from the regulated genes involved in miR-26b-5p-mediated PDGFR-β expression in BMSCs, the candidate genes were submitted to functional network reconstruction using STRING (https://string-db.org/cgi/input.pl). 27 genes were clustered together. Within this functional cluster, vascular endothelial growth factor (VEGF) A, B, and C; Angpt1; Angpt2; their tyrosine kinase receptor (TIE1); and von Willebrand factor (vWF) were involved in angiogenesis; and TGF-β1, α-SMA, Col α1(I), Col α1(III), matrix metalloproteinase 9 (MMP9), and tissue inhibitor of metalloproteinase (TIMP) 1 and 3 participated in collagen deposition, extracellular matrix remodeling, liver fibrosis, and repair (Figure 6D).

### miR-26b-5p Agomir Targeted PDGFR-β and Attenuated Liver Fibrosis and Angiogenesis *In Vivo*

**Figure 7:**
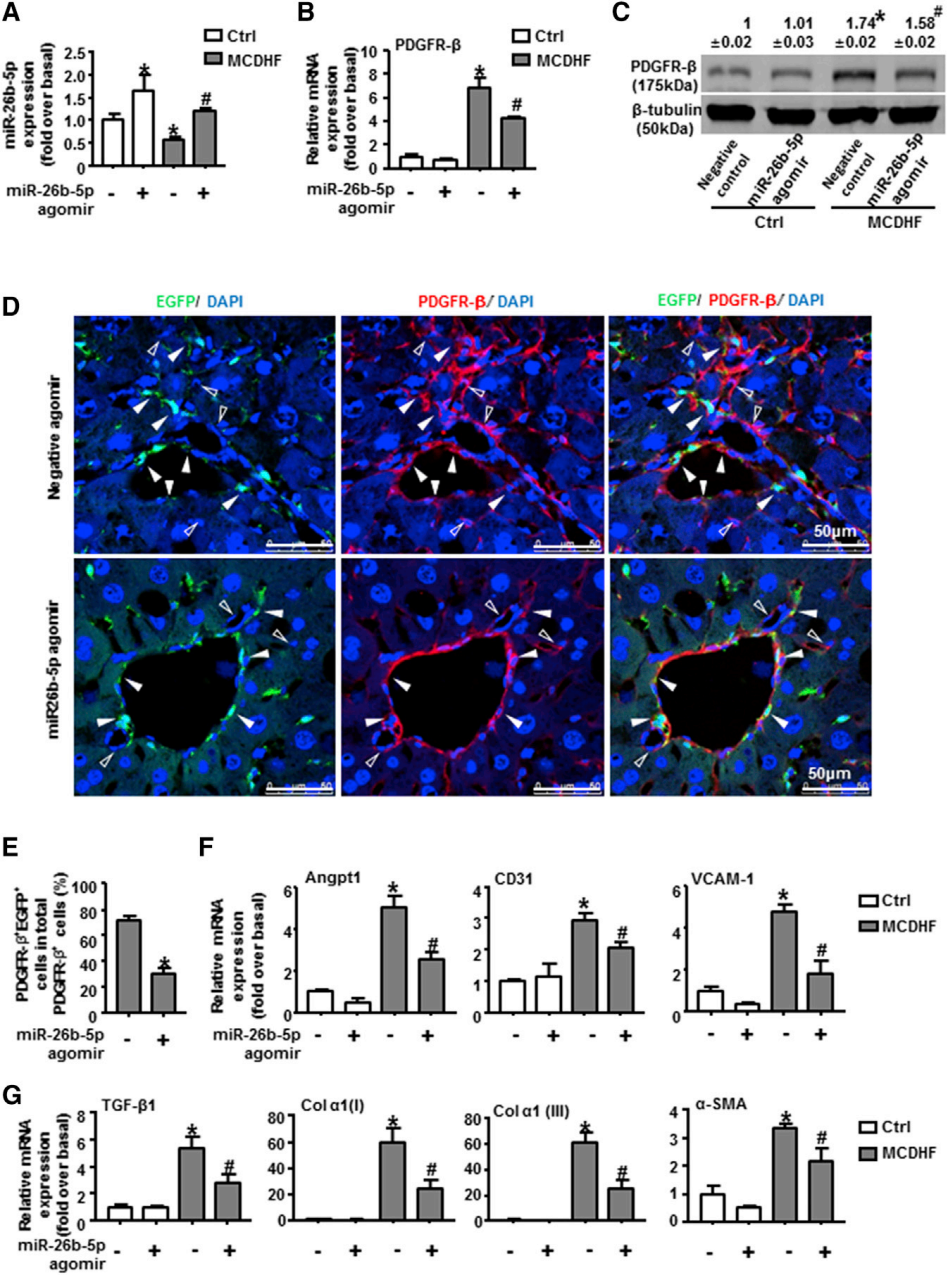
The Regulation of miR-26b-5p Agomir on PDGFR-β Expression, Liver Fibrosis, and Angiogenesis *In Vivo* (A) Transfection efficiency of 50 nM miR-26b-5p agomir (mimic *in vivo*) in the fibrotic liver of MCDHF mice. (B) PDGFR-β mRNA levels in liver tissue were measured by qRT-PCR with or without miR-26b-5p agomir injection in MCDHF mice. (C) PDGFR-β protein levels in liver tissue were measured by western blot. (D) Representative images of immunofluorescence analysis to track PDGFR-β (red) expression in BMSCs (green) in the fibrotic liver. (E) The proportion of PDGFR-β^+^EGFP^+^ cells of total PDGFR-β^+^ cells was measured by Image-Pro Plus software. Hollow arrows indicate PDGFR-β^−^EGFP^+^ cells, while solid arrows indicate PDGFR-β^+^EGFP^+^ cells. DAPI was used to visualize nuclei (blue). The mRNA levels of angiogenesis markers (F) and fibrosis markers (G) in the fibrotic liver are shown. Data are presented as the mean ± SEM. n = 6 per group. *p < 0.05 versus control. #p < 0.05 versus MCDHF treated alone.

In the end, we verified the regulation of miR-26b-5p on PDGFR-β during liver fibrogenesis *in vivo*, through the injection of miR-26b-5p agomir into MCDHF mice for 56 days. First, the hepatic expression of miR-26b-5p was examined by qRT-PCR, verifying the effectiveness and specificity of miR-26b-5p agomir *in vivo* (Figure 7A). Moreover, the expression of PDGFR-β was explored by qRT-PCR, western blot, and immunofluorescence staining. The results showed that PDGFR-β mRNA levels were markedly attenuated after the injection of miR-26b-5p agomir in MCDHF mice (Figure 7B). The protein expression of PDGFR-β (Figure 7C) and the proportion of PDGFR-β^+^EGFP^+^ cells of total PDGFR-β^+^ cells also presented a significant drop in the presence of miR-26b-5p agomir in MCDHF mice (Figures 7D and 7E). Furthermore, the mRNA expression of angiogenesis markers (Figure 7F) and fibrosis markers (Figure 7G) was markedly declined after miR-26b-5p agomir administration in MCDHF mice. Taken together, these results validated that miR-26b-5p targeted PDGFR-β and attenuated liver fibrosis and angiogenesis *in vivo*.

## Discussion

In this study, we investigated the potential role of miR-26b-5p in liver fibrogenesis and angiogenesis via directly regulating PDGFR-β expression and interacting with lncMEG3. Our work provides several new findings as follows: (1) the expression of miR-26b-5p, predicted to target PDGFR-β by bioinformatics analysis, is decreased in the fibrotic liver of MCDHF mice, with a negative correlation to PDGFR-β, fibrosis, and angiogenesis markers; (2) miR-26b-5p directly targets PDGFR-β in TGF-β1-treated BMSCs by pull-down and lucifer reporter assays, which can be sponged by lncMEG3; (3) microarray data and KEGG, Reactome, and GO enrichment analyses revealed that miR-26b-5p overexpression affects a variety of genes associated with fibrosis and angiogenesis; and (4) *in vivo* miR-26b-5p agomir negatively regulates PDGFR-β expression and attenuates liver fibrosis and angiogenesis.

The miR-26 family is one of the most extensively studied miRNAs. In the previous studies, miR-26b-5p has been characterized in a variety of pathophysiological processes, including proliferation, angiogenesis, inflammation, and injury-related processes. For instance, miR-26b-5p was identified as a negative regulator of proliferation and apoptosis in hepatocellular carcinoma.^35^ The lncMalat1, miR-26b-5p, ULK2 axis regulated brain microvascular endothelial cell autophagy and survival under oxygen-glucose deprivation and reoxygenation condition.^36^ A phenotypic miRNA screen identifies that miR-26b-5p can promote endothelial cell growth, survival, and angiogenesis following acute ischemia.^37^ In addition, increased miR-26b-5p expression could inhibit the activation of microglia and the production of interleukin-6 in hypoxia-ischemia, thus alleviating the cognitive impairment.^38^ The miR-26a and/or miR-26b, Cyclooxgenase-2-macrophage inhibitory protein-2 loop regulated a positive feedback between allergic inflammation and tumor metastasis.^39^ The present study first documents the role of miR-26b-5p in liver fibrogenesis and angiogenesis via targeting PDGFR-β, displaying that miR-26b-5p overexpression remarkably inhibits the upregulation of PDGFR-β mRNA and protein levels and attenuates liver fibrosis and angiogenesis *in vivo*, which can be used as a novel target for the treatment of liver fibrosis.

A common action mechanism for lncRNA-miRNA interaction is referred to as “sponge,” in which lncRNA functions as miRNA sponges competing with mRNA for miRNA binding.^40^ lncMEG3 was first identified as a tumor suppressor in various cancers,^41^, ^42^, ^43^ and recent studies have highlighted the importance of lncMEG3 in angiogenesis and the maintenance of normal function of vascular endothelial cells.^31^, ^32^, ^33^ Gain-of-function studies have confirmed that lncMEG3 acts as an endogenous sponge to suppress the biological function of miR-26b-5p by a sequence-complementary mechanism and to increase PDGFR-β expression in BMSCs. Thus, the balancing effects of lncMEG3 on miR-26b-5p action should be taken into account when using miR-26b-5p-based therapy.

The current study shows that BMSCs contribute to the vast number of PDGFR-β^+^ cells in mouse fibrotic liver, as demonstrated by the fact that PDGFR-β^+^EGFP^+^ cells accounted for 72.5% of total PDGFR-β^+^ cells in the fibrotic liver of MCDHF mice. Previous studies have revealed that BMSCs can migrate to the injured liver and differentiate to α-SMA^+^ myofibroblasts in liver fibrosis.^27^, ^28^, ^29^ Here we document the high expression of PDGFR-β on BMSCs in the fibrotic liver of MCDHF mice, and we identify the function of these PDGFR-β^+^ BMSCs, which is closely associated with liver fibrogenesis and angiogenesis. Consistently, PDGFR-β^+^ cells have been described as perivasular cells that are essential for the regulation of angiogenesis and blood vessel stabilization, integrity, and remodeling.^44^, ^45^, ^46^, ^47^, ^48^ Stromal cells have been shown to have two-sided effects in a range of clinical settings, including liver disease. On one hand, many clinical studies have been conducted on the treatment of liver fibrosis or cirrhosis via the transplantation of stromal cells.^49^ On the other hand, BMSCs can differentiate to myofibroblasts in previous studies or to PDGFR-β^+^ cells in this study, thus contributing to the progression of hepatic fibrogenesis in different mouse models. This could be partly attributed to the marked heterogeneity (different subtypes and probably different functions) of the stromal cell population in its respective niche.

Fat liver injury has become a rising cause of chronic liver disease worldwide;^25^, ^26^ therefore, here we used an MCDHF diet to induce liver fibrosis in mice. Combining methionine-choline deficiency with high-fat diet, which can both contribute to the abnormal accumulation of fat within liver, MCDHF mice showed a rapid progression of extensive liver fibrogenesis, with excessive collagen deposition, lipid accumulation, and a significant increase in fibrosis and inflammation markers starting from 14 until 56 days, indicating the successful establishment of liver fibrosis models in mice.

In conclusion, we identified the critical role of miR-26b-5p in chronic liver injury and angiogenesis, and we explored the underlying mechanism associated with the negative regulation of miR-26b-5p on its target PDGFR-β and interaction with lncMEG3, which may open new perspectives for pharmacological treatment of liver fibrosis.

## Materials and Methods

### Mouse Models

Liver fibrosis models were induced by MCDHF diet. In detail, mice were fed either a control diet or an MCDHF diet (A06071309, Research Diet) containing 46 kcal% fat, 18 kcal% protein, and 36 kcal% carbohydrate. Mice were sacrificed at days 3, 7, 14, 28, and 56. All animals received human care and all animal protocols conformed to the Ethics Committee of Capital Medical University and were in accordance with the approved guidelines (approval AEEI-2014-131).

To trace BMSC differentiation *in vivo*, adult Institute of Cancer Research (ICR) mice (post-natal day [P]50–60) received irradiation (8 Gy in a divided dose 4 h apart), and immediately they received transplantation by a tail vein injection of the cell mixture (0.10*10^7^ BMSCs from EGFP transgenic mice and 1.10*10^7^ whole bone marrow [BM] cells from wild-type mice, in which BMSCs had been removed). Anti-CD146 microbeads (Miltenyi Biotec, Germany) were used to purify BMSCs or remove BMSCs from whole bone marrow cells by immunomagnetic cell sorting. 4 weeks later, the bone marrow was reconstructed, and the chimera mice with EGFP-labeled BMSCs were fed with MCDHF diet to build the liver injury model.

miR-26b-5p agomir (miR-26b-5p mimic *in vivo*) was purchased from Guangzhou RiboBio, and it was delivered *in vivo* using a hydrodynamic transfection method, by which 50 nM miR-26b-5p agomir was rapidly injected into the tail vein. Control mice were injected with an equal volume of control agomir dissolved in PBS. These miRNA agomirs were injected twice per week in MCDHF diet-eating mice for 8 weeks.

### BMSC Isolation and Culture

ICR mice were anesthetized, and whole bone marrow cells were extracted from the tibias and femurs by using a 25G needle to flush with culture medium. The cells were filtered through a 70-μm nylon mesh and washed with PBS containing 2% fetal bovine serum (FBS). Then BMSCs were cultured and used for experiments from passages 3 to 6. Characterization of BMSCs was performed by flow cytometry analysis.

### Immunofluorescence Staining

BMSCs were fixed in 4% paraformaldehyde in PBS for 20 min and permeabilized in 0.5% Triton X-100 in PBS for 15 min. Liver samples were fixed in 4% paraformaldehyde and embedded in Tissue Tek optimal cutting temperature (OCT) compound. 5 μm frozen section was used for immunofluorescence. BMSCs or the liver sections were blocked with 2% BSA, and then they were incubated with anti-PDGFR-β monoclonal antibody (1:100, Abcam, Cambridge, UK) and Cy3-AffiniPure goat anti-rabbit immunoglobulin G (IgG) (1:100, Jackson ImmunoResearch Laboratories, West Grove, PA, USA) as secondary antibodies. The samples were covered with Vectashield mounting medium containing DAPI and observed under a confocal microscope (LSM510, Carl Zeiss MicroImaging, Germany). The percentage of PDGFR-β^+^EGFP^+^ cells accounting for total PDGFR-β^+^ cells was measured by the software Image-Pro Plus.

### Histology Analysis

Liver tissues were fixed in 4% paraformaldehyde. Liver tissue sections (5 μm) were stained with H&E for the assessment of inflammation and injury, Sirus Red for the extent of collagen deposition, and oil red O for lipid accumulation.

### Immunomagnetic Cell Sorting

CD146^+^ cells were enriched and depleted from EGFP^+^ and EGFP^−^ bone marrow, respectively, by using CD146 MicroBeads (Miltenyi Biotec, Bergisch Gladbach, Germany), according to the manufacturer’s instructions. About 0.35% CD146^+^ cells were separated from EGFP^+^ bone marrow and 92.44% CD146^−^ cells were separated from EGFP^−^ bone marrow complementarily. Cell sorting efficiency was further verified by flow cytometry. Immunomagnetic cell-sorted EGFP^+^/CD146^+^ cells and EGFP^−^/CD146^−^ cells were mixed at the original ratio for transplantation.

### qRT-PCR

Extraction of total RNA from mice liver frozen specimen and qRT-PCR were performed. Primers were as follows: 18S rRNA sense 5′-GTA ACC CGT TGA ACC CCA TT-3′, antisense 5′-CCA TCC AAT CGG TAG TAG CG-3′; PDGFR-β sense 5′-TTC CAG GAG TGA TAC CAG CTT-3′, antisense 5′-AGG GGG CGT GAT GAC TAG G-3′; TGF-β1 sense 5′-TGC GCT TGC AGA GAT TAA AA-3′, antisense 5′-TCA CTG GAG TTG TAC GGC AG-3′; α-SMA sense 5′-ATG CTC CCA GGG CTG TTT T-3′, antisense 5′-TTC CAA CCA TTA CTC CCT GAT GT-3′; Col α1(I) sense 5′-AGG GCG AGT GCT GTG CTT T-3′, antisense 5′-CCC TCG ACT CCT ACA TCT TCT GA-3′; Col α1(III) sense 5′-TGA AAC CCC AGC AAA ACA AAA-3′, antisense 5′-TCA CTT GCA CTG GTT GAT AAG ATT AA-3′; CD31 sense 5′-GAC GAT GCG ATG GTG TAT AAC G-3′, antisense 5′-GAG CCT GAG GAA TGA CGT AGC T-3′; and VCAM-1 sense 5′-TGC GAG TCA CCA TTG TTC TCA T-3′, antisense 5′-CCC CTC CGT CCT CAC CTT-3′. Probes (Applied Biosystems) used for real time RT-PCR were Angpt1 and Mm00456503_m1.

### Western Blot

Western blot for PDGFR-β was performed with 50 μg protein extract using monoclonal antibodies to PDGFR-β (1:1,000, Abcam, Cambridge, UK) and the appropriate IRDyeTM 800-conjugated secondary antibody (1:10,000). Signals were detected using the Odyssey Imaging System (LI-COR Biosciences, Lincoln, NE, USA) and analyzed with Odyssey software. Results were normalized relative to β-tubulin (mouse anti-β-tubulin monoclonal antibody, 1:2,000, Transgen Biotech, China) expression to correct for variations in protein loading and transfer.

### Pull-down Assay

Biotinylated miR-26b-5p mimics and negative control mimics (Beijing AuGCT Biotechnology, China) were respectively transfected into BMSCs. Cells lysate were then incubated with streptavidin-coupled magnetic beads (Dynabeads M-280 Streptavidin, Thermo Fisher Scientific, Waltham, MA, USA) for purifying target RNA, in accordance with the manufacturer’s instructions. The RNA amplification product quantified and analyzed by qRT-PCR was used for DNA agarose gel electrophoresis.

### Luciferase Reporter Assays

BMSCs were seeded in 96-well plates 24 h prior to transfection. Subsequently, the cells were transiently co-transfected with 5 ng wild-type or mutant reporter plasmid (Guangzhou RiboBio, China) and 50 nM miR-26b-5p mimic or miR-control using Lipofectamine 2000. Firefly and Renilla luciferase activities were measured 48 h subsequent to transfection using the Dual Luciferase Assay (Promega, Madison, WI, USA), according to the manufacturer’s protocol. Firefly luciferase activity was normalized to Renilla, and the value of the ratio of firefly luciferase activity to renilla luciferase activity was analyzed.

### Microarray and Computational Analyses

Briefly, samples (BMSCs treated with TGF-β1 with or without miR-26b-5p mimic) were used to synthesize double-stranded cDNA, and double-stranded cDNA was labeled and hybridized to an Affymetrix Mouse Gene MTA 1.0 array (Affymetrix, Santa Clara, CA, USA). To select the differentially expressed genes, we used threshold values of ≥1.3-fold change and corrected p value < 0.05. KEGG, Reactome, and GO analyses were performed to explore the function and pathways based on differentially expressed genes. mRNA microarray experiments and technical assistance in bioinformatic analysis were handled by Capitalbio Technology (Beijing, China).

### Statistical Analysis

The results are expressed as mean ± SEM. Statistical significance was assessed by Student’s t test or one-way ANOVA when appropriate. Correlation coefficients were calculated by Pearson test. p < 0.05 was considered to be significant. All results were verified in at least three independent experiments.

## Author Contributions

Study Concept and Design, L.L., Le Yang, and C.D.; Data Acquisition, Le Yang, C.D., and Lin Yang; Data Analysis and Interpretation, Le Yang, C.D., J.Y., Lin Yang, N.C., C.Q., and L.L.; Drafting of the Manuscript, Le Yang, C.D., and L.L.; Study Supervision, L.L.

## Conflicts of Interest

The authors indicate no potential conflicts of interest.

## Supplemental Information

Table S1. The Preliminary Probe Signal Values of Noncoding Genes by Microarray Analysis in TGF-b1-Treated BMSCs with or without miR-26b-5p Mimics

Table S2. The Preliminary Probe Signal Values of Coding Genes by Microarray Analysis in TGF-b1-Treated BMSCs with or without miR-26b-5p Mimics

Table S3. KEGG and Reactome Pathways in TGF-b1-Treated BMSCs with or without miR-26b-5p Mimics

Table S4. GO Terms (Biological Process) in TGF-b1-Treated BMSCs with or without miR-26b-5p Mimics

Document S2. Article plus Supplemental Information
