# Supplementary material for: MicroRNA-26b-5p Inhibits Mouse Liver Fibrogenesis and Angiogenesis by Targeting PDGF Receptor-Beta
Source: Mol Ther Nucleic Acids. 2019 Feb 26;16:206–17. doi: 10.1016/j.omtn.2019.02.014 (PMC6426711; doi:10.1016/j.omtn.2019.02.014)
Supplement: Table S1. The Preliminary Probe Signal Values of Noncoding Genes by Microarray Analysis in TGF-b1-Treated BMSCs with or without miR-26b-5p Mimics [file mmc1.pdf]

OMTN, Volume *16*

Supplemental Information

MicroRNA-26b-5p Inhibits Mouse Liver Fibrogenesis and Angiogenesis by Targeting PDGF Receptor-Beta

Le Yang, Chengbin Dong, Jingjing Yang, Lin Yang, Na Chang, Changbo Qi, and Liying Li

MicroRNA-26b-5p Inhibits Mouse Liver Fibrogenesis and Angiogenesis by Targeting PDGF Receptor-beta

Le Yang#, Chengbin Dong#, Jingjing Yang, Lin Yang, Na Chang, Changbo Qi, Liying Li*

Supplementary table 1. The preliminary probe signal values of noncoding genes by microarray analysis in TGF-b1-treated BMSCs with or without miR-26b-5p mimics

Probe Set ID	Gene Symbol	Control mimics			miR-26b-5p mimics		
		1	2	3	4	5	6
TC1500001514.mm.1		230.69	250.19	247.64	70.37	76.40	78.10
TC0100000997.mm.1		297.11	297.11	311.24	97.50	86.92	97.61
TC0500000573.mm.1		297.11	297.11	311.24	97.50	86.92	97.61
TC0100001693.mm.1	Gm24405	419.99	411.72	429.19	207.22	210.06	222.38
TC1200001192.mm.1	Mirg; Mir134; Mir377; Mir382; Mir410; Mir412; Mir485; Mir369; Mir668;	77.83	80.06	74.45	42.75	40.62	41.20
TC0900000895.mm.1	9530091C08Rik	64.33	68.73	67.72	30.32	31.79	28.82
TC0900000037.mm.1		24.30	25.61	24.76	15.59	14.81	15.49
TC1300000114.mm.1		5402.02	6162.90	5284.89	1531.62	1397.55	1624.13
TC1900000001.mm.1		705.19	746.21	797.75	322.89	295.37	314.01
TC1200001140.mm.1		1595.87	1853.22	1518.92	419.92	435.33	472.59
TC1000000100.mm.1	Hivep2; Gm20114	227.56	221.53	209.93	126.04	131.92	132.95
TC1200001142.mm.1	Rian; AF357355; AF357359; AF357425; DQ267100; DQ267101	434.72	443.68	405.31	159.07	158.44	182.66
TC1200001147.mm.1	Gm24564	7080.00	9285.74	7676.71	1607.60	1286.07	1491.24
TC0400001968.mm.1	Mir34a	110.29	109.95	110.61	78.12	72.03	74.69
TC1200001127.mm.1	Meg3; Mir770; Mir1906-1; Mir1906-2	100.49	109.20	94.60	49.75	50.49	51.74
TC0100002623.mm.1	Gm26263	50.98	58.13	54.71	29.75	28.84	29.12
TC1400001518.mm.1		70.77	69.18	70.45	43.08	44.49	40.11
TC1700000133.mm.1		225.37	235.43	200.93	95.76	86.21	91.19
TC0X00003297.mm.1		37.44	40.44	40.99	23.43	21.92	23.20
TC0100002692.mm.1		253.47	290.16	268.96	137.16	135.94	127.28
TC0400002815.mm.1		1922.80	1990.24	1784.36	526.89	463.25	603.38
TC0400002648.mm.1		24060.48	23960.61	24807.40	16088.47	15517.19	14641.79
TC0200003225.mm.1	Gm13371; RP23-475B13.4	78.28	79.72	78.80	55.49	52.72	57.34
TC1300002482.mm.1		296.21	299.38	285.51	183.85	167.79	185.13
TC1100001296.mm.1		43.83	39.60	39.29	24.43	23.19	23.55
TC1700002272.mm.1		13716.81	13730.62	13957.80	8431.19	8492.53	7580.17
TC0800002911.mm.1		14012.21	14997.21	15199.95	9867.14	9356.19	9754.39
TC0X00000514.mm.1	1600025M17Rik	19.05	19.38	18.49	13.76	14.31	13.47
TC0600002863.mm.1		95.56	108.35	105.21	30.48	38.77	32.93
TC0X00002854.mm.1	Gm23322	88.37	96.86	93.01	63.90	61.76	62.92
TC0400002965.mm.1		64.49	71.07	78.45	27.53	29.78	26.02
TC1200000136.mm.1		413.67	478.85	440.42	214.78	235.18	224.70
TC0100001475.mm.1	Dnm3os; Mir214; Mir199a-2	543.23	638.68	565.42	259.14	225.31	249.34
TC1800000033.mm.1		70.61	69.42	81.68	26.49	21.03	23.84
TC1300000169.mm.1		30852.82	31506.72	28817.49	19832.40	19429.05	18451.71
TC0400002967.mm.1		113.51	120.12	105.75	35.14	40.50	44.72
TC0700000086.mm.1	Peg3os	59.73	59.97	61.99	45.41	42.21	44.29
TC1800000752.mm.1		48.31	51.66	51.03	29.66	27.09	30.60
TC1400002790.mm.1	2610042L04Rik	38.07	36.76	37.88	28.26	28.05	26.43
TC0X00000785.mm.1	Gm14749	737.84	658.73	637.60	367.58	342.91	338.88
TC0800000940.mm.1		211.70	219.90	230.70	129.86	139.95	125.90
TC1800000524.mm.1		308.16	359.97	328.82	163.21	161.55	177.37
TC1800000745.mm.1		28.73	29.89	30.12	24.26	23.63	23.64
TC1100000241.mm.1		59.94	65.04	62.39	44.23	42.37	44.56
TC1100002345.mm.1		25.66	24.23	25.68	17.69	16.35	17.39
TC0100000398.mm.1	Gm10561	96.46	93.90	97.61	60.30	54.19	61.06
TC0200001941.mm.1		26.60	26.50	27.23	20.18	18.54	19.32
TC1200000732.mm.1		26.39	26.09	27.57	20.67	19.74	19.73
TC0600000333.mm.1		92.31	86.05	83.00	36.68	40.92	44.05
TC0200001793.mm.1	Gm14087; RP23-158L6.1	832.04	754.04	704.62	404.65	415.67	418.35
TC1500001292.mm.1		45.05	44.02	41.66	21.94	22.43	18.89
TC0900002256.mm.1		79.86	87.74	91.86	29.79	37.33	32.66
TC0100003023.mm.1		82.13	78.47	76.95	56.51	59.52	58.86
TC1500001517.mm.1		135.21	159.71	161.30	71.39	63.53	66.69
TC0900000898.mm.1	F830001A07Rik	188.48	262.53	209.40	50.86	61.33	55.39
TC0300003210.mm.1	Schip1; Gm21949	164.04	167.99	166.09	112.24	123.13	114.44
TC0300001589.mm.1	Wls	4151.20	4218.31	3953.19	2671.50	2426.76	2677.41
TC1700001305.mm.1		133.84	150.12	134.79	85.98	84.71	88.55
TC0100000065.mm.1		176.46	177.99	201.03	67.26	50.01	55.05
TC1300002720.mm.1		22758.57	22848.36	21123.59	14518.77	15237.41	14058.07
TC0200001610.mm.1		26159.70	26944.01	24810.32	15348.40	14678.51	16550.52
TC0200003052.mm.1		34.02	33.49	33.53	26.96	25.11	26.17
TC0500003696.mm.1		38.00	35.06	40.11	20.19	17.72	19.70
TC0X00002290.mm.1	Gm14889; RP23-303A21.2	439.81	405.13	427.79	257.48	278.07	281.81
TC0X00002908.mm.1	Gm14929; RP23-79L4.1	60.75	60.86	57.81	45.83	45.61	47.58

TC1100003458.mm.1		171.37	172.08	158.51	62.02	78.33	65.31
TC0200005100.mm.1	Gm14265; RP23-161B3.4	835.09	838.23	823.53	598.12	569.27	626.04
TC1500001801.mm.1		23.23	22.11	21.69	15.01	15.58	16.22
TC1100003621.mm.1	2010300F17Rik	164.90	151.24	131.66	63.00	55.81	62.85
TC0X00002541.mm.1	Gm14737; RP23-461P10.1	2673.64	2639.65	2397.64	1515.26	1374.72	1521.05
TC0400000474.mm.1		117.68	141.67	119.92	45.17	51.96	41.82
TC1400000035.mm.1	Gm10410	40.01	39.61	39.13	28.78	28.79	26.36
TC0700004076.mm.1	St5	539.11	513.76	508.49	317.49	289.32	331.93
TC0600002441.mm.1	Rprl1	45.07	46.50	44.97	36.23	35.28	33.84
TC0200003757.mm.1		64.22	57.48	56.85	32.96	32.00	28.81
TC0600002004.mm.1		114.30	115.51	111.75	82.28	73.75	78.18
TC1500000454.mm.1	Pvt1	61.72	66.37	63.15	50.00	49.24	49.73
TC0900000103.mm.1		2029.88	2132.65	2060.93	1166.33	1177.51	1336.78
TC1100001298.mm.1		19.14	21.28	20.18	13.62	14.19	13.66
TC0700004432.mm.1		47.17	47.60	46.49	31.29	27.04	29.74
TC0800002077.mm.1		121.27	131.96	134.66	64.60	53.60	51.77
TC0800002352.mm.1		75.54	76.47	61.80	31.39	32.19	33.64
TC1000002312.mm.1		123.10	145.27	123.57	70.44	66.26	70.74
TC0100003495.mm.1		52.57	68.43	57.25	24.83	24.83	22.63
TC0100003492.mm.1		32.58	31.88	35.12	22.41	23.62	22.10
TC1200001163.mm.1	Gm22205	301.06	410.04	287.58	86.64	72.75	90.77
TC1200002055.mm.1		638.64	651.61	706.64	437.61	412.15	451.07
TC0200003714.mm.1		88.65	95.59	90.90	59.47	63.12	65.65
TC0200004609.mm.1	Gm14043; RP23-358G23.3	3022.81	3086.53	2910.22	1626.56	1636.28	1899.66
TC0600000011.mm.1		14972.22	15699.23	13926.29	4319.52	3344.05	4993.45
TC1300001916.mm.1		47.06	55.84	51.04	30.77	30.12	30.57
TC1200000191.mm.1		32.99	29.95	29.62	20.89	19.27	19.89
TC0600003013.mm.1		59.97	53.61	53.18	30.79	31.99	34.67
TC1200001150.mm.1	Gm23600	372.04	391.39	376.69	124.25	121.99	165.55
TC1000001342.mm.1		2436.85	2413.65	2363.78	1505.64	1270.77	1277.40
TC1700000011.mm.1		40.09	44.97	41.76	28.68	29.90	29.36
TC0200004345.mm.1		359.35	295.55	298.05	81.55	114.45	96.72
TC0400000848.mm.1	Gm12415; RP23-229A22.3	38.08	34.25	34.29	21.92	22.82	20.34
TC0200005293.mm.1		346.36	353.04	296.26	183.31	174.31	163.98
TC1100002093.mm.1		87.32	74.78	84.48	46.42	49.57	44.71
TC1700002390.mm.1	4930405O22Rik	43.41	47.05	40.60	27.89	28.70	27.91
TC0X00003095.mm.1	Gm5762	100.15	102.12	92.66	63.31	69.30	65.75
TC1300001172.mm.1		5693.97	8504.95	6473.52	1818.52	1707.28	2050.05
TC0100000432.mm.1		6028.10	6232.70	5292.36	3382.94	3080.46	3355.73
TC0600000098.mm.1		23091.12	22983.56	22102.19	15106.07	12921.21	14338.66
TC0100002366.mm.1	Gm23943	25461.78	28351.28	29138.19	17136.23	17121.93	18215.65
TC0600002766.mm.1		38.12	42.37	37.62	20.41	16.17	18.55
TC0400000200.mm.1		14118.35	15428.98	14730.02	10624.54	11121.18	10397.85
TC0900001160.mm.1	Gm22866	12140.99	11174.31	13715.38	5605.33	4383.93	4749.17
TC0200001689.mm.1		53.23	53.03	58.17	40.16	38.59	40.11
TC1300001772.mm.1	Gm6093	82.55	86.05	74.65	40.30	33.53	40.93
TC0900000594.mm.1	Gm11149	36.13	34.92	33.65	27.35	25.60	26.51
TC0800000442.mm.1		51.84	58.82	55.84	38.90	37.18	37.73
TC1900001690.mm.1		3994.29	4231.43	3804.16	2347.01	2158.73	1926.08
TC0600002832.mm.1	Gm20705; RP23-333F8.1	45.54	53.73	41.64	22.87	22.70	21.89
TC1600002113.mm.1		32.48	30.63	30.84	24.38	22.44	23.34
TC0100001499.mm.1	Gm16588	334.00	377.05	332.18	183.80	169.57	147.21
TC0400000475.mm.1		107.68	137.97	129.12	51.08	53.31	59.30
TC1300001236.mm.1		30.21	37.13	35.70	18.91	18.68	18.83
TC1500000092.mm.1		121.29	135.96	126.37	53.09	47.86	63.82
TC0X00001711.mm.1		74.81	78.87	91.15	47.16	46.04	46.26
TC0X00000746.mm.1	Gm15384; RP23-252K7.9	1314.09	1419.99	1398.90	1042.59	973.95	957.55
TC0300001277.mm.1		88.41	99.21	90.48	66.99	64.20	64.90
TC0300000911.mm.1		14.29	13.96	15.34	9.56	10.11	10.51
TC1500002068.mm.1		25.21	25.54	26.02	19.04	17.14	18.97
TC1200001152.mm.1	Gm25357	308.21	290.33	213.99	89.94	80.06	72.13
TC1100000447.mm.1		111.49	132.59	112.37	61.35	64.31	68.78
TC1500001209.mm.1		73.63	63.66	69.15	45.00	40.47	42.15
TC0900000087.mm.1		62.43	75.02	74.22	41.02	39.66	40.55
TC1200001185.mm.1	Mir376a	94.17	88.60	105.11	40.86	50.58	47.11
TC0X00000583.mm.1	Gm24598	5755.15	4652.04	5125.98	1596.16	976.25	1270.55
TC0400002734.mm.1		180.37	169.30	151.57	82.52	78.56	94.14
TC1100001699.mm.1	Gm22683	28.92	27.84	29.28	21.47	21.94	19.85
TC1500000456.mm.1		84.92	83.74	80.37	53.18	60.45	53.96
TC0200001270.mm.1		22106.90	21873.74	22305.20	14508.99	12615.49	11907.08
TC0300001384.mm.1		42.42	47.52	38.59	25.01	24.66	24.33
TC1300001559.mm.1		7463.48	7415.57	7951.38	5710.79	5674.91	5249.23
TC0700001104.mm.1		2459.37	2685.55	2828.73	1591.75	1387.48	1595.40
TC1500001157.mm.1		108363.10	119628.90	104092.80	73339.27	66591.32	66400.46
TC0400004056.mm.1		27.52	30.84	27.04	16.68	15.61	18.05
TC0300002359.mm.1	Gm24046	747.99	690.10	807.45	399.86	365.04	438.19
TC0600000305.mm.1	Plxna4os1	17.88	17.86	16.20	11.53	10.77	12.04
TC0500002961.mm.1	Gm5871	727.66	760.55	711.88	566.05	596.25	560.82
TC0800000524.mm.1	Gm15878	286.60	291.37	329.16	168.45	136.54	157.10
TC1300001904.mm.1		2276.20	2235.82	2235.10	1568.42	1329.15	1365.44

TC0900000557.mm.1		23.40	21.39	22.62	16.33	17.40	16.95
TC0300002757.mm.1	Gm12521; RP23-316M11.3	1497.12	1418.01	1393.70	889.82	803.32	956.04
TC0X00002593.mm.1	Mir1906-1; Mir1906-2	84.10	84.47	94.95	53.03	44.96	51.22
TC1200001709.mm.1		52.23	51.69	47.55	32.31	28.91	33.43
TC0300000388.mm.1		47.92	61.90	61.95	24.86	20.09	22.25
TC1500001498.mm.1		497.15	693.02	555.30	220.77	245.62	230.04
TC0400000974.mm.1		33.38	37.77	35.84	19.11	21.08	17.07
TC1900000087.mm.1		157.56	137.53	118.03	34.44	44.35	48.71
TC0700001905.mm.1		1467.79	1563.86	1538.42	1015.08	893.30	843.47
TC1100000162.mm.1		111.45	92.21	103.82	60.54	59.79	54.66
TC0X00002686.mm.1	Gm14801; RP23-418O17.1	191.85	183.87	175.11	125.52	108.51	109.10
TC0600000332.mm.1		103.21	102.45	88.55	49.67	43.78	54.18
TC0300001276.mm.1		56.65	68.11	64.30	32.52	37.45	34.62
TC0200003040.mm.1		99.48	111.33	96.70	57.95	62.93	65.99
TC0900003213.mm.1		107.87	140.68	128.12	58.52	51.85	46.94
TC1700002605.mm.1		129.34	146.61	131.52	53.94	52.06	69.33
TC1800000600.mm.1		296.75	350.48	236.57	78.05	59.17	85.91
TC1800000031.mm.1		68.52	80.54	54.07	17.89	17.06	22.70
TC0100001696.mm.1		265.74	281.14	247.32	143.90	123.68	153.49
TC1100000810.mm.1	Gm12281	30.83	31.06	29.09	21.84	23.81	22.01
TC1500000857.mm.1	Gm23737	205.04	194.43	222.36	107.10	103.57	83.39
TC0600001583.mm.1		119.80	118.30	122.86	84.91	83.21	93.50
TC1300000437.mm.1		111.28	126.83	105.47	70.80	66.94	64.15
TC0900002258.mm.1		91.12	105.49	97.03	29.07	39.96	40.51
TC0300002981.mm.1	Gm25042	45460.18	52096.08	48480.46	31863.93	28509.33	31794.86
TC0X00002744.mm.1		24.34	27.10	25.31	18.68	16.87	17.47
TC0300001762.mm.1		106.01	106.62	90.14	53.14	48.22	57.70
TC0600003170.mm.1	Gm7308	13334.18	12351.26	13709.41	9643.03	8917.62	9544.99
TC0600001726.mm.1		3097.43	3010.93	2917.35	1910.23	1974.83	1649.57
TC1600001534.mm.1		269.35	320.66	280.01	187.91	180.82	181.99
TC1300002345.mm.1	Gm24519	31.38	34.76	34.55	25.37	25.31	24.63
TC0X00002483.mm.1		29.60	31.36	28.21	21.04	19.07	21.08
TC0900003202.mm.1		489.87	595.34	494.34	312.28	295.36	303.20
TC1100000209.mm.1		171.62	217.00	194.65	110.20	102.58	98.71
TC1700002606.mm.1		35.33	40.81	36.93	20.48	23.49	23.47
TC1300000079.mm.1		48.38	50.81	50.46	33.35	37.57	33.03
TC1000002513.mm.1	Gm17151; RP24-121A12.3	382.56	396.83	396.47	303.76	269.37	292.31
TC1100002927.mm.1		158.88	162.60	171.13	114.58	114.47	126.50
TC0X00002206.mm.1		710.12	635.43	594.61	371.43	300.58	312.19
TC0X00000841.mm.1	Gm14763; RP23-311G7.2	800.60	882.84	780.35	535.98	521.63	581.64
TC1500001513.mm.1		190.14	281.15	183.25	58.15	53.43	69.16
TC0X00001177.mm.1	Gm14928	717.98	702.35	739.29	526.94	452.89	499.76
TC1900000303.mm.1		289.40	299.83	285.85	163.45	173.80	199.53
TC1100002239.mm.1		17212.51	17005.76	16324.98	11786.41	10334.50	11948.57
TC0300000941.mm.1	Gm24136	3155.58	2685.48	3193.53	1535.69	1211.73	1499.05
TC1500002178.mm.1	Gm19664	68.67	68.53	77.51	49.79	46.11	49.71
TC0300000390.mm.1		25.15	25.61	28.58	14.43	17.04	16.19
TC1300000945.mm.1		54.33	45.45	40.57	22.96	21.25	23.47
TC0400000637.mm.1		89.46	100.05	78.03	49.77	49.27	46.12
TC0800002476.mm.1		64.52	82.98	60.38	30.45	26.49	24.23
TC1400002343.mm.1	Bmp1	8875.67	9414.19	8020.84	5469.55	4980.69	5694.36
TC1100000122.mm.1		4625.91	5660.95	4613.72	2747.41	2607.13	2889.34
TC0400002814.mm.1		284.74	355.09	261.15	76.90	83.61	114.12
TC0300003112.mm.1		4692.71	5332.93	5182.02	3498.33	3185.65	3041.12
TC1500000149.mm.1		13113.53	13708.52	13010.87	8832.27	7182.68	8276.82
TC0800001304.mm.1	Gm16349; AC122807.3	65.97	75.40	73.54	45.78	38.05	38.88
TC0900000901.mm.1	Gm19304	41.21	41.65	40.61	26.43	29.79	30.53
TC0100001323.mm.1		131.31	138.87	122.03	83.70	68.61	76.88
TC1100000089.mm.1	Gm11402; RP23-198N14.4	170.86	197.22	170.29	111.83	117.63	121.64
TC1300001304.mm.1		97.99	108.36	83.39	53.49	52.34	51.20
TC1300001310.mm.1		38.75	34.02	40.71	21.66	23.48	20.02
TC0200003855.mm.1	Gm13685	2108.75	2271.51	1996.13	1514.00	1354.41	1452.37
TC1200000196.mm.1		148.25	135.37	157.09	87.78	99.58	94.96
TC0700003882.mm.1	Gm25171	64910.45	72899.04	74425.13	50470.83	49682.68	47539.17
TC0800000949.mm.1	Gm25110	64910.45	72899.04	74425.13	50470.83	49682.68	47539.17
TC0800001606.mm.1	Gm25775	64910.45	72899.04	74425.13	50470.83	49682.68	47539.17
TC1600000569.mm.1	Gm24795	64910.45	72899.04	74425.13	50470.83	49682.68	47539.17
TC0600000138.mm.1	ST7-OT4_3	121.90	110.01	122.38	81.31	88.46	83.15
TC1200000213.mm.1		37.47	35.75	33.63	25.95	26.40	27.71
TC0500002514.mm.1		93.17	108.76	93.64	59.20	65.64	61.59
TC0700003533.mm.1		18074.41	19272.57	20921.03	11073.85	8435.74	8931.63
TC1700002604.mm.1		126.24	152.48	111.52	62.14	55.02	62.05
TC0300002663.mm.1		35.05	39.68	39.22	24.30	24.41	20.98
TC1100004162.mm.1	Gm11747	104.58	112.46	114.01	85.80	78.97	84.26
TC1700001443.mm.1	D17Ertd648e	32.73	34.25	34.23	25.24	27.61	26.99
TC1500000015.mm.1		8018.48	8246.26	8546.57	5827.03	4964.00	5692.72
TC0700001010.mm.1		59.59	65.75	67.73	33.04	39.81	39.12
TC1900000558.mm.1		141.83	133.74	144.79	96.10	86.71	101.12
TC0900000085.mm.1		2750.14	4063.20	4025.16	1150.98	1343.97	1253.01
TC1100000444.mm.1		177.80	214.17	173.99	63.35	62.83	87.55

TC1600000636.mm.1		72.59	87.05	84.64	36.77	41.31	46.42
TC0900000084.mm.1		114.27	133.95	104.36	51.60	56.78	63.19
TC0200000796.mm.1		104.82	117.93	101.92	68.56	57.93	56.64
TC0200000789.mm.1		29.80	31.27	26.94	19.59	20.05	17.95
TC1000001777.mm.1		72.46	65.36	69.12	31.87	23.70	33.43
TC0900002665.mm.1		4768.70	4840.51	5007.52	3168.90	2484.55	2835.30
TC0800002478.mm.1		76.58	100.01	68.94	34.79	32.01	34.86
TC0600002836.mm.1		97.71	113.72	108.60	45.01	60.18	50.62
TC0600002598.mm.1		18.07	22.74	20.17	12.20	12.11	12.35
TC1400002190.mm.1		61.40	56.09	65.15	30.62	36.22	37.14
TC0300000100.mm.1		44.57	47.49	47.57	25.94	28.30	31.98
TC0300000568.mm.1	Gm10040	850.73	948.77	863.16	590.62	573.71	649.97
TC1000001682.mm.1		318.56	330.61	278.45	170.28	136.86	169.88
TC0900002536.mm.1		89.38	99.35	82.81	60.78	55.23	57.57
TC0100003705.mm.1		123.35	141.42	116.86	65.09	71.97	77.89
TC0500002510.mm.1	Gm23183	46.95	54.82	45.87	29.91	27.96	31.74
TC1700002609.mm.1		110.71	117.88	102.84	67.18	59.50	72.21
TC0700004331.mm.1		35.81	34.71	35.38	26.31	22.89	26.13
TC0900000905.mm.1		44.10	34.40	38.73	21.04	22.92	20.18
TC1100003198.mm.1	Pafah1b1	541.80	502.71	517.21	385.65	411.44	419.95
TC0900000086.mm.1	Phxr4	547.50	656.15	490.34	186.28	136.83	213.64
TC1300000436.mm.1		234.19	271.66	239.83	160.32	165.47	143.88
TC1000001855.mm.1		196.35	180.38	147.26	62.55	83.51	66.92
TC1600000003.mm.1	Gm23215	6730.67	8227.23	6503.87	4194.48	3814.42	4001.16
TC0600002817.mm.1		25.05	27.27	27.99	20.19	19.18	20.61
TC0600003436.mm.1	Gm15720	21408.82	17844.95	21020.40	12745.46	13318.19	12610.91
TC0600002867.mm.1		31.55	23.87	31.62	14.46	14.64	14.56
TC0500000338.mm.1	Gm5864	52197.53	53667.73	50359.19	40454.28	36247.88	40159.38
TC0200001425.mm.1	Gm13815	321.24	342.99	355.44	256.46	245.97	267.78
TC1700002448.mm.1		28.06	25.06	26.33	15.61	18.55	17.32
TC0400002751.mm.1		312.61	284.84	340.99	200.06	209.26	214.69
TC1200001839.mm.1		73.99	69.57	71.53	49.62	54.16	46.06
TC0200003105.mm.1		42.26	43.87	44.21	31.36	30.87	26.71
TC0X00001066.mm.1		82.36	72.22	94.35	46.39	47.25	43.17
TC0300003072.mm.1		29.56	33.42	31.55	23.01	20.56	22.51
TC0X00002757.mm.1	Gm9115	929.60	813.86	763.67	546.07	538.38	552.78
TC1200001148.mm.1	Gm24899; Gm26945; RP23-204I16.2	32.01	37.45	38.19	23.80	24.13	23.36
TC1200000363.mm.1		56.01	61.89	46.71	26.75	29.60	24.73
TC1800000941.mm.1	Gm16147	253.46	269.17	279.06	158.60	134.36	170.97
TC0500000264.mm.1		63.52	66.91	60.71	48.04	46.46	50.75
TC1800000485.mm.1		235.25	231.95	219.53	167.50	175.87	185.57
TC0100001434.mm.1	Sec16b	69.98	71.49	62.12	41.00	35.99	43.88
TC1000001857.mm.1	Gm26528	82.69	83.47	75.99	61.80	56.09	54.85
TC1900000245.mm.1		26841.30	27860.82	28116.31	19255.66	15858.81	18571.42
TC1400001955.mm.1	Gm23470	142.84	143.68	133.23	83.94	61.07	69.02
TC0100003755.mm.1		58.12	72.56	65.93	34.97	38.61	40.22
TC0200000233.mm.1		9143.88	10192.84	9184.60	6907.67	6949.25	7388.00
TC1200001318.mm.1		84.24	89.28	73.21	53.78	49.13	47.32
TC0600000334.mm.1	Gm19966	50.57	54.16	42.73	23.53	19.68	25.99
TC0800001308.mm.1		26.73	24.90	26.96	19.85	17.25	18.91
TC1400001258.mm.1		69.63	81.41	64.37	39.55	32.86	39.68
TC0X00001357.mm.1		16263.06	16557.67	16436.45	11060.05	9047.51	10996.57
TC0600002827.mm.1		155.64	167.06	154.51	57.30	55.83	82.76
TC0300000389.mm.1		402.41	626.70	600.50	182.29	151.51	123.07
TC0200002730.mm.1	Gm16356	132.28	138.81	135.25	108.63	98.45	94.86
TC0100003555.mm.1		3173.56	3458.75	3055.56	2073.58	2379.54	2196.22
TC1300002393.mm.1		46.88	59.53	63.93	28.15	29.48	29.63
TC0700004436.mm.1	Gm19514	60.53	79.24	76.17	31.39	36.51	27.41
TC0700004439.mm.1		41.38	34.94	42.59	23.38	23.52	25.94
TC1100003243.mm.1		26.25	25.64	23.75	18.24	19.86	18.01
TC0800003064.mm.1	Gm17709	735.87	632.47	642.21	360.51	439.61	403.15
TC0300000251.mm.1		12221.10	12787.64	11899.95	9192.62	7831.87	7789.77
TC0800000591.mm.1	Gm15758; AC109200.1	750.77	792.12	893.77	550.65	482.40	528.38
TC0100002694.mm.1		95.04	120.06	92.78	46.32	56.86	49.41
TC0300000562.mm.1		49.53	46.30	45.45	34.59	37.78	34.09
TC1100000123.mm.1	Gm11995; RP23-138G17.1	2880.74	3048.79	2982.16	1812.95	1525.57	1966.33
TC0800002076.mm.1		230.33	233.51	192.07	87.15	77.99	113.11
TC0200000402.mm.1		25.57	23.28	25.22	19.20	18.83	20.14
TC1300002726.mm.1		101.47	101.96	110.77	72.68	71.58	61.00
TC1300001426.mm.1		75.39	78.64	71.39	59.84	56.88	60.98
TC1800000381.mm.1	Gm24401	25.92	28.01	26.96	19.88	21.27	18.61
TC1700002670.mm.1		35.00	37.93	38.70	29.00	30.14	29.97
TC0300002512.mm.1	Terc	126.75	117.51	125.10	98.10	94.73	87.68
TC0900003265.mm.1		16.08	18.82	18.71	11.99	12.22	10.93
TC1500001328.mm.1	Snhg18	821.49	669.68	686.57	388.98	331.29	420.82
TC0X00000588.mm.1	Gm14670	152.63	159.20	154.48	127.51	120.49	112.58
TC1700000127.mm.1	Airn	48.10	55.08	51.91	39.50	36.21	37.06
TC1900001083.mm.1	Gm24543	368.29	394.11	403.34	230.49	234.19	279.00
TC0300001343.mm.1		37.28	31.28	35.37	23.23	21.98	24.27

TC1400000345.mm.1		6538.90	6927.20	6286.90	4334.19	3383.53	3412.91
TC0800000678.mm.1	Gm25403	138.25	117.11	107.62	63.35	45.57	52.97
TC0400000983.mm.1		4259.97	4158.39	4133.11	2984.63	2549.94	3045.97
TC1900000986.mm.1	Neat1	932.08	994.29	829.64	409.07	327.05	480.02
TC0900002948.mm.1		43.36	34.69	37.96	25.37	24.18	23.67
TC0200000277.mm.1	Gm20038	96.14	86.91	90.73	27.94	39.12	43.65
TC0400002886.mm.1		18.56	19.63	20.10	15.98	14.92	14.49
TC1200001191.mm.1	Mir544	40.48	46.49	47.12	24.37	29.73	24.99
TC0700000216.mm.1		617.48	560.31	508.65	356.78	286.55	315.13
TC0800002073.mm.1		23.13	28.17	26.34	14.34	15.26	17.00
TC1800000717.mm.1		63.46	55.35	62.91	30.74	24.16	33.71
TC1400000983.mm.1		34.58	39.48	39.69	25.89	24.72	21.87
TC0300001274.mm.1		99.25	141.74	109.90	55.57	57.85	57.08
TC0600002094.mm.1		19.23	20.51	18.86	14.91	16.11	14.82
TC0300002821.mm.1		33187.50	34542.32	32366.61	24540.44	20370.69	23197.31
TC0100003357.mm.1		20.68	20.54	17.85	13.93	14.32	14.33
TC0100003331.mm.1		154.63	172.36	159.14	97.82	80.00	103.01
TC0900000091.mm.1		81.33	76.38	65.53	44.06	34.12	38.90
TC1200002021.mm.1		88.65	94.09	86.21	62.72	50.29	57.66
TC0100002691.mm.1		45.53	55.70	45.58	23.72	28.95	27.88
TC0200002001.mm.1		20.87	21.76	19.87	16.41	16.79	15.29
TC1700000210.mm.1	Mirlet7e	270.36	320.94	241.70	138.35	120.82	152.39
TC1100003676.mm.1	Gm25048	28.70	25.48	23.94	15.61	16.17	17.99
TC0900000978.mm.1	Gm7265	62.27	59.71	52.78	37.20	40.79	40.77
TC1200000845.mm.1	LOC100861892	89.23	94.06	92.47	68.28	56.31	57.18
TC1300001215.mm.1		53.49	64.94	59.53	37.46	34.03	39.59
TC1200001508.mm.1		368.11	358.41	313.11	178.66	141.84	195.19
TC0X00002944.mm.1		21456.79	25635.78	19872.06	13362.21	11667.81	11115.78
TC0X00002822.mm.1		45.99	50.28	53.57	33.07	36.03	36.29
TC1800000383.mm.1		102.38	129.62	99.68	52.02	62.16	58.76
TC0100003100.mm.1	Gm19487	22350.43	24731.87	20544.05	13200.66	9897.41	12094.01
TC0200004526.mm.1		52.99	54.08	54.98	31.05	29.34	37.82
TC0800001065.mm.1		23.01	25.07	23.67	18.63	18.74	16.83
TC0700004437.mm.1		35.87	32.77	36.89	21.73	18.67	15.85
TC0900001233.mm.1		9994.06	12141.00	10587.65	6943.37	5562.51	5728.66
TC0300000592.mm.1	Gm10780	12858.60	13534.18	16250.70	8068.41	6289.99	7535.17
TC0600002223.mm.1	Gm7887	3189.30	3379.91	2980.80	2444.43	2494.18	2531.33
TC0300000130.mm.1	Gm23330	30792.04	28520.27	34310.07	20025.86	15769.16	17372.48
TC1200001161.mm.1	Gm23787	138.28	130.66	86.42	44.46	44.90	39.49
TC1000001837.mm.1		26.65	29.08	29.88	21.31	18.12	19.03
TC0200000029.mm.1		50.27	64.47	49.60	29.88	32.11	30.42
TC0600000383.mm.1	Gm20022	120.02	130.46	137.65	80.56	80.02	93.84
TC0200003094.mm.1	Gm13356	55.40	67.64	57.64	40.15	36.01	39.06
TC1800000056.mm.1		46.03	51.14	50.90	38.88	35.76	37.94
TC0900000227.mm.1		56.71	69.89	59.64	40.46	34.10	35.19
TC1700001566.mm.1	Gm7809	213.29	200.43	221.51	161.97	155.08	140.52
TC0600000312.mm.1		60.03	77.39	63.00	40.75	37.94	35.86
TC0100003702.mm.1		47.48	55.61	39.44	25.30	24.71	23.62
TC1100003216.mm.1		35.20	30.86	30.72	22.16	24.05	21.61
TC1200001181.mm.1		32.03	34.50	32.67	22.50	25.30	21.15
TC0400000197.mm.1	Gm11883; RP23-25B10.2	512.98	524.52	606.31	362.21	362.43	394.52
TC0X00003162.mm.1		48.92	47.29	43.07	33.50	36.34	34.53
TC1400000028.mm.1	Gm26898	38.70	42.55	34.91	26.46	27.11	25.66
TC0900000899.mm.1		71.95	93.02	70.31	29.14	40.31	32.00
TC0900003208.mm.1		47.03	63.26	55.21	31.08	31.89	28.71
TC1300001900.mm.1		2591.84	2830.64	2904.42	1888.57	1882.38	2134.55
TC0X00002958.mm.1	Gm26475	25117.42	28626.15	27401.52	20840.09	19692.49	20953.26
TC0500001900.mm.1		75.43	80.16	69.70	37.86	36.33	48.14
TC0100000797.mm.1		17.95	19.58	19.79	11.34	13.88	12.47
TC1400002839.mm.1	Gm3500; Gm3373	52.04	51.21	52.98	39.22	35.85	41.81
TC0700003443.mm.1		1163.88	1422.22	1076.64	522.07	418.29	606.07
TC0400001297.mm.1	Gm12840; RP23-99G21.3	1639.77	1712.33	1444.41	1121.14	985.71	958.33
TC1500001496.mm.1		77.46	107.21	84.30	40.93	37.81	47.54
TC1800000066.mm.1	Gm7497	368.65	368.83	350.03	271.67	229.97	221.85
TC0200001408.mm.1	Gm13785	55.41	73.19	58.79	31.56	25.52	32.92
TC1600001710.mm.1		19.70	22.59	19.28	14.37	12.22	12.29
TC0Y00000128.mm.1		17.92	16.55	15.36	11.66	12.17	10.73
TC1400001903.mm.1	Gm22637	152.14	161.28	131.90	63.31	62.19	85.59
TC1600001099.mm.1	Gm22862	7196.74	8161.02	8085.03	5763.57	5346.94	4939.65
TC1500001516.mm.1	Gm19551	184.64	256.49	177.93	77.35	97.09	73.48
TC1100002033.mm.1	Gm20014	377.51	444.39	420.65	302.09	271.12	291.20
TC0200001666.mm.1		125.95	145.22	141.72	93.66	81.31	95.66
TC0700002756.mm.1		49.04	44.79	45.48	31.61	26.86	32.84
TC0200003396.mm.1		3716.36	3646.42	3728.08	2602.64	2380.24	2023.67
TC1500001916.mm.1		78.83	91.27	79.16	61.15	61.26	58.25
TC1700002602.mm.1		80.15	81.74	66.38	37.44	30.68	42.58
TC0700001089.mm.1		30.20	27.30	30.56	22.51	23.65	22.89
TC1700000140.mm.1		189.61	211.21	165.77	83.48	73.35	104.34
TC0200002969.mm.1		362.46	389.00	372.81	236.44	260.95	285.47
TC1100000446.mm.1		86.13	92.85	89.67	38.46	53.51	36.92

TC0600002489.mm.1		45.17	40.80	40.00	28.81	29.72	32.47
TC1000001860.mm.1		204.51	180.43	140.58	62.44	84.50	67.15
TC1600001119.mm.1		199.49	225.79	207.77	134.08	113.12	142.50
TC0600002862.mm.1		79.03	111.57	87.07	46.91	47.01	40.17
TC1600001726.mm.1		279.34	337.42	261.05	152.66	161.01	180.67
TC0200004718.mm.1	Gm14063; RP23-109J20.1	879.33	912.91	896.73	668.99	638.98	738.46
TC0100003774.mm.1		40.01	38.53	40.21	28.29	31.97	31.63
TC0400004128.mm.1		54.56	50.69	57.20	39.53	43.12	41.38
TC0X00002130.mm.1	Gm14604; RP23-260P9.1	332.41	364.70	381.90	253.78	205.63	225.99
TC0900002534.mm.1		82.19	85.03	89.38	53.39	56.53	64.80
TC1300001491.mm.1		22.77	24.04	24.39	17.79	18.94	19.79
TC1800000601.mm.1		56.14	86.68	66.26	28.36	29.51	32.58
TC0100001342.mm.1		719.58	524.74	587.70	296.67	209.13	269.83
TC1200000820.mm.1	D030025P21Rik	75.77	73.87	64.70	36.44	24.06	33.43
TC0400002197.mm.1		630.67	721.80	672.04	430.05	341.18	427.35
TC1500000457.mm.1		163.66	124.77	119.12	66.56	51.64	65.44
TC0800001166.mm.1		221.62	267.19	268.20	164.98	134.96	142.20
TC1200001365.mm.1		306.58	327.73	361.24	245.39	248.66	236.77
TC0700000623.mm.1	Gm12757	60.14	58.05	58.09	44.38	49.59	44.07
TC0200002926.mm.1		29272.71	28876.22	27621.64	19764.74	17436.89	21425.92
TC1300001849.mm.1		88677.43	98027.07	86878.62	64125.51	51484.88	52207.36
TC1800000034.mm.1		66.09	94.32	70.41	23.02	20.60	33.18
TC0400002884.mm.1		69.97	91.60	85.55	36.47	39.29	26.52
TC1700001664.mm.1		66.53	86.62	73.57	46.92	45.89	47.40
TC1000002861.mm.1		40.65	41.54	38.12	31.06	33.71	31.30
TC1100000545.mm.1	Gm12197; RP23-121P11.4	620.14	645.11	647.17	460.80	373.66	445.29
TC1400002784.mm.1	Gm3020; Gm10409	79.43	82.45	80.93	57.71	53.91	64.43
TC0200005206.mm.1		27.99	29.33	31.72	21.77	23.23	23.41
TC0500000509.mm.1	Gm10025	82411.65	94754.58	83886.53	62647.65	54787.86	61998.63
TC1300000122.mm.1		57.39	49.55	56.42	29.75	29.45	36.97
TC0900002099.mm.1		86.27	95.25	82.31	38.57	54.33	42.03
TC1800001703.mm.1		18.43	19.46	19.26	13.82	14.48	12.05
TC1300001223.mm.1		25.09	26.93	21.27	14.44	12.53	15.19
TC0600001385.mm.1		171.66	210.57	211.81	82.17	73.95	108.84
TC0200001686.mm.1		103.13	113.55	108.21	76.54	67.47	81.04
TC0900002098.mm.1		56.87	64.74	61.54	29.49	38.47	27.50
TC1700002683.mm.1		36.46	38.96	28.11	18.01	18.84	18.82
TC1100000626.mm.1		61.95	58.06	61.02	47.46	41.81	47.46
TC0100000722.mm.1	Gm25754	136591.10	160746.70	155402.80	108620.10	111258.00	101872.20
TC0100001888.mm.1	Gm24802	136591.10	160746.70	155402.80	108620.10	111258.00	101872.20
TC0100002294.mm.1	Gm22419	136591.10	160746.70	155402.80	108620.10	111258.00	101872.20
TC0100002671.mm.1	Gm22112	136591.10	160746.70	155402.80	108620.10	111258.00	101872.20
TC0300001225.mm.1	Gm24744	136591.10	160746.70	155402.80	108620.10	111258.00	101872.20
TC0300001226.mm.1	Gm26137	136591.10	160746.70	155402.80	108620.10	111258.00	101872.20
TC0300001993.mm.1	Gm22029	136591.10	160746.70	155402.80	108620.10	111258.00	101872.20
TC0300002791.mm.1	Gm25347	136591.10	160746.70	155402.80	108620.10	111258.00	101872.20
TC0400000137.mm.1	Gm24567	136591.10	160746.70	155402.80	108620.10	111258.00	101872.20
TC0400002099.mm.1	Gm22991	136591.10	160746.70	155402.80	108620.10	111258.00	101872.20
TC0400002377.mm.1	Gm23304	136591.10	160746.70	155402.80	108620.10	111258.00	101872.20
TC0700001493.mm.1	Gm25774	136591.10	160746.70	155402.80	108620.10	111258.00	101872.20
TC0700001894.mm.1	Gm25778	136591.10	160746.70	155402.80	108620.10	111258.00	101872.20
TC0700002324.mm.1	Gm26248	136591.10	160746.70	155402.80	108620.10	111258.00	101872.20
TC0700003452.mm.1	Gm23844	136591.10	160746.70	155402.80	108620.10	111258.00	101872.20
TC0800002026.mm.1	Gm23177	136591.10	160746.70	155402.80	108620.10	111258.00	101872.20
TC0900001937.mm.1	Gm26190	136591.10	160746.70	155402.80	108620.10	111258.00	101872.20
TC0X00001078.mm.1	Gm22102	136591.10	160746.70	155402.80	108620.10	111258.00	101872.20
TC1300002157.mm.1	Gm24193	136591.10	160746.70	155402.80	108620.10	111258.00	101872.20
TC1400001825.mm.1	Gm25208	136591.10	160746.70	155402.80	108620.10	111258.00	101872.20
TC1400001835.mm.1	Gm25228	136591.10	160746.70	155402.80	108620.10	111258.00	101872.20
TC1700000334.mm.1	Gm24427	136591.10	160746.70	155402.80	108620.10	111258.00	101872.20
TC1700002684.mm.1	Gm22346	136591.10	160746.70	155402.80	108620.10	111258.00	101872.20
TC1800000198.mm.1	Gm23606	136591.10	160746.70	155402.80	108620.10	111258.00	101872.20
TC1800000478.mm.1	Gm24617	136591.10	160746.70	155402.80	108620.10	111258.00	101872.20
TC1900000180.mm.1	Gm24484	136591.10	160746.70	155402.80	108620.10	111258.00	101872.20
TC0200005292.mm.1		164.95	164.87	130.21	77.51	87.04	65.28
TC1200002051.mm.1		592.10	575.35	514.31	429.47	397.71	422.66
TC1500001495.mm.1		116.33	176.86	110.95	55.02	43.26	49.45
TC1600001969.mm.1		97.90	121.02	96.60	63.57	67.70	58.94
TC0800001367.mm.1		37.47	32.10	31.80	20.88	20.69	24.05
TC0600000217.mm.1		58.37	62.45	62.75	51.55	46.76	48.96
TC0900002535.mm.1	Gm19279	370.47	424.73	415.05	285.87	245.22	284.50
TC1700000129.mm.1		154.85	169.45	169.51	92.15	90.12	115.58
TC1700002254.mm.1		81.67	87.75	86.56	61.06	49.71	47.33
TC0400002813.mm.1		96.11	66.23	76.10	28.46	26.49	38.27
TC0600002864.mm.1		80.05	130.98	88.36	35.48	40.02	39.74
TC1100000438.mm.1	Gm22121	29.80	31.58	32.90	25.29	23.43	22.20
TC0100002535.mm.1		43.23	38.32	38.62	28.32	30.49	26.33
TC0700004434.mm.1		98.38	159.33	111.48	50.77	51.61	46.19
TC0X00003370.mm.1	Gm23197	21.51	19.88	17.28	11.95	13.40	11.73
TC0400000638.mm.1		78.31	107.32	107.51	52.94	48.70	45.74

TC0200003756.mm.1		52.24	59.17	53.74	39.90	34.59	40.09
TC0500002914.mm.1		25.30	30.19	28.84	20.02	17.22	17.15
TC0900000474.mm.1		3851.21	5027.67	4085.82	2634.90	2551.58	2695.60
TC0100002699.mm.1		1821.69	2238.57	1817.35	953.98	881.87	1185.86
TC0400000729.mm.1		117.39	143.78	112.79	58.05	39.79	58.81
TC1100002143.mm.1		601.30	733.17	671.41	448.51	357.00	377.86
TC0400001065.mm.1		90.48	104.91	99.94	69.82	72.99	63.23
TC1700002538.mm.1	Gm26749	59.17	62.09	55.53	46.14	44.46	40.46
TC0300001393.mm.1	Gm22468	133044.80	156187.60	153368.70	107606.10	107279.40	98727.97
TC0200003055.mm.1		121.35	128.53	114.43	52.53	58.61	76.03
TC1300001175.mm.1		121.88	128.47	91.64	36.79	53.09	49.00
TC0700001895.mm.1		30128.76	29629.93	27722.41	20735.42	15844.31	17254.73
TC1500001497.mm.1		120.86	174.60	148.06	57.35	56.72	76.14
TC0400001522.mm.1	Gm24621	143.90	168.47	152.57	105.65	103.00	117.07
TC1400000508.mm.1	Gm15562; RP23-36P21.4	86.01	117.07	86.25	49.21	53.92	46.88
TC0600000123.mm.1		74.87	68.35	70.27	51.49	54.37	58.28
TC1200001165.mm.1	B830012L14Rik; RP23-378G4.2	38.02	47.83	43.68	27.43	28.16	24.08
TC0X00000542.mm.1	Gm14888; RP23-150G4.2	2289.64	2194.15	2014.26	1241.48	896.87	1231.30
TC1700002138.mm.1	Runx2	185.34	177.65	154.12	125.68	117.55	121.44
TC0X00001260.mm.1		30.46	35.27	30.17	22.95	23.48	21.21
TC1000001251.mm.1	Gm25587	4350.51	5250.41	4857.51	3253.68	2587.54	2633.07
TC1200001502.mm.1		55.37	57.72	59.24	39.35	32.33	40.60
TC1200001517.mm.1		55.37	57.72	59.24	39.35	32.33	40.60
TC0600002866.mm.1		48.88	59.21	51.83	38.35	35.83	37.84
TC1400000208.mm.1		62.03	55.96	48.85	22.78	31.46	29.14
TC0200004717.mm.1	Gm14060; RP23-181O3.2	699.66	651.73	605.11	448.71	422.69	493.62
TC0400004172.mm.1		51.32	55.05	52.27	27.66	32.63	36.77
TC0200004197.mm.1		2272.05	2442.32	2227.30	1601.69	1291.70	1588.37
TC1300001108.mm.1	Gm5086	258.47	254.82	173.74	95.20	88.83	109.39
TC1700000131.mm.1		50.01	62.94	61.21	33.19	29.02	36.34
TC0200000709.mm.1	Nr6a1os; RP23-334M3.3	38.84	36.78	37.53	26.59	24.61	20.46
TC1200001141.mm.1		32.94	33.11	30.78	19.14	23.91	19.31
TC1300001351.mm.1		716.04	880.46	742.29	464.87	364.45	462.40
TC0400000472.mm.1		60.86	67.37	51.49	38.00	37.96	35.89
TC1600002047.mm.1		373.94	419.37	420.83	254.11	176.63	208.76
TC1500000091.mm.1		29.95	25.02	27.73	14.88	14.82	18.71
TC0600002908.mm.1		40.23	40.39	33.23	25.28	26.57	24.71
TC0200000734.mm.1		351.11	376.20	355.46	258.70	278.21	297.11
TC0300000739.mm.1	Gm23054	74.96	72.26	87.25	54.33	53.41	47.70
TC0600000828.mm.1		40.43	42.21	42.15	33.26	35.02	30.76
TC0400000477.mm.1		68.61	93.67	66.51	33.67	39.75	39.08
TC1600000878.mm.1	Mirlet7c-1	55.64	66.77	47.70	26.84	16.32	18.47
TC1300002570.mm.1		23.00	19.81	21.02	16.12	16.78	16.21
TC0500002365.mm.1	Gm10048	223.50	265.90	239.35	175.55	183.17	168.86
TC0400003916.mm.1		59.67	53.52	54.12	37.07	32.89	40.90
TC1100000603.mm.1	Mir6920	75.15	79.26	63.96	50.79	47.47	45.46
TC1000000104.mm.1		41.59	48.37	43.99	24.89	31.58	26.45
TC0200002941.mm.1		70.58	64.40	57.78	44.02	45.83	46.32
TC0900000914.mm.1		86.33	93.51	91.91	49.82	42.57	60.03
TC1500001515.mm.1		105.71	161.08	111.79	35.81	51.17	50.42
TC0300001273.mm.1		265.93	293.17	237.14	136.18	113.53	160.38
TC1400000836.mm.1		48.71	52.16	50.67	42.91	40.06	38.16
TC1300000154.mm.1		48.11	42.12	36.15	19.32	24.51	23.44
TC0300001387.mm.1		122.55	110.36	103.97	75.25	61.22	74.49
TC0200001688.mm.1		74.30	80.00	66.53	49.22	45.53	38.70
TC1500000458.mm.1		22.88	21.75	27.62	16.22	14.42	14.74
TC0600002807.mm.1	Gm23510	5843.99	5322.73	6295.83	3881.97	2922.68	3006.74
TC0900002096.mm.1		54.73	58.09	55.72	36.69	38.05	44.46
TC1600000876.mm.1		124.82	221.97	158.13	63.60	56.46	64.68
TC0400001991.mm.1		123.72	113.54	121.36	94.47	80.82	90.30
TC1000002183.mm.1		422.13	399.54	369.52	307.57	263.79	269.96
TC1500000941.mm.1		58.12	77.83	66.48	42.78	38.36	39.23
TC1600001668.mm.1		46.04	60.85	55.11	34.34	29.38	29.17
TC1000002790.mm.1		15249.46	16588.10	13371.66	10201.22	9198.49	10389.88
TC1000001909.mm.1	Mir680-3	201.20	224.83	243.20	135.65	107.46	139.61
TC0200002222.mm.1		31.82	27.23	29.04	20.98	22.37	22.61
TC0800003241.mm.1	2610005L07Rik	1876.51	1950.74	1691.21	1259.87	1237.85	1420.06
TC1700002391.mm.1		18.50	16.12	17.22	10.85	13.06	11.45
TC0100000982.mm.1		973.83	866.35	909.41	520.83	502.46	652.78
TC0600001225.mm.1		353.73	432.85	378.25	267.80	220.03	244.46
TC1600000632.mm.1		30.34	31.73	35.98	15.62	18.06	21.32
TC0700003948.mm.1	Gm16464	6687.70	7433.02	6602.10	3638.63	3809.58	4809.13
TC1500001140.mm.1		142.70	177.82	139.00	62.21	32.54	51.64
TC1200000364.mm.1		339.15	396.03	385.98	137.23	151.32	215.23
TC0400003484.mm.1		99.75	102.78	87.06	65.62	53.78	50.06
TC0X00001734.mm.1	Gm15253; RP23-22L6.8	6768.89	8430.13	7453.65	5210.89	5012.13	5332.58
TC0300001383.mm.1		163.72	187.74	172.10	79.68	96.76	112.30
TC0600000056.mm.1		36.44	42.07	41.29	29.91	30.76	31.21
TC0200005067.mm.1		68.01	71.34	58.98	46.46	48.67	45.27

TC0500003695.mm.1		142.20	160.87	106.54	65.44	55.74	46.07
TC1700002546.mm.1	Gm17538	15901.72	18417.31	17684.58	13501.34	13574.80	13522.43
TC1100001059.mm.1		31.54	34.65	27.56	18.31	21.42	19.91
TC0800002080.mm.1	Gm19274	18.46	20.38	19.11	13.89	14.65	15.84
TC1500001635.mm.1	9130004J05Rik	141.98	146.12	145.13	108.76	99.69	118.55
TC0900002595.mm.1		257.72	242.66	229.55	164.16	146.24	181.79
TC1300000156.mm.1		30.58	27.53	26.18	21.43	19.10	18.52
TC1300001403.mm.1		4731.79	4685.11	4342.52	3668.72	3110.57	3377.61
TC0300000355.mm.1		191.94	255.43	177.33	82.62	102.50	106.31
TC0300001258.mm.1	Gm15400	73.60	73.18	69.47	58.30	54.26	61.20
TC0400000657.mm.1	Gm12529	84.77	76.84	67.85	54.50	50.98	50.20
TC0600002831.mm.1		48.78	53.66	57.07	29.62	36.19	26.89
TC1000000225.mm.1		307.06	448.26	302.58	149.64	106.36	149.67
TC0200001667.mm.1		60.75	58.29	60.79	46.75	45.44	39.09
TC0400000516.mm.1	Gm12424	598.95	620.34	538.46	429.03	425.65	465.85
TC0800002081.mm.1		25.22	25.93	20.62	14.14	16.26	14.62
TC0100000221.mm.1	D630036G22Rik	174.25	182.47	156.82	126.18	128.25	112.71
TC1200000379.mm.1		27.41	32.41	27.64	21.67	20.99	19.86
TC1400000997.mm.1	Mir320	164.28	189.73	153.00	119.48	107.17	114.86
TC0X00000938.mm.1	Gm14814; RP23-437A4.2	1066.72	1117.40	998.27	769.81	796.24	868.20
TC0400002195.mm.1	Gm11815; RP23-172G14.2	275.68	302.27	252.20	204.58	198.81	209.57
TC0100000156.mm.1	Mir30c-2	40.47	46.97	42.34	33.69	30.66	32.78
TC1700001304.mm.1	Gm19773	27.69	25.50	25.43	19.93	17.47	20.42
TC0200001175.mm.1	Gm13688	782.53	766.03	642.08	506.56	428.38	475.71
TC0400000335.mm.1		394.07	361.18	388.55	309.00	293.65	322.75
TC1300001220.mm.1		21.45	22.04	22.55	14.41	17.01	16.98
TC1200001136.mm.1	6430411K18Rik; Mir127; Mir433	69.70	86.73	63.77	40.76	34.25	42.62
TC0X00000394.mm.1		190.21	297.05	190.37	78.49	80.35	102.05
TC0700003063.mm.1		1085.29	1145.85	941.32	439.93	247.04	424.90
TC0500003697.mm.1		110.76	101.81	81.83	43.05	27.84	43.83
TC0800001553.mm.1		43.39	34.14	35.75	25.38	25.08	24.54
TC0400001191.mm.1		54.82	72.40	63.24	34.93	32.91	40.67
TC0500000304.mm.1		40.52	39.33	34.69	28.42	29.76	28.97
TC0700001384.mm.1	LOC100861604	316.21	277.44	281.20	188.57	135.88	170.24
TC0200001500.mm.1	Gm13804	61.97	57.14	66.67	47.26	46.59	42.30
TC1900000671.mm.1		22.04	22.95	25.39	16.08	16.66	18.40
TC0100000518.mm.1	Gm20342	112.75	113.47	103.91	93.80	86.19	88.04
TC0200002046.mm.1	Gm25704	223.48	230.61	244.91	178.47	142.50	154.52
TC0600000086.mm.1		300.91	373.95	374.21	235.20	201.70	195.37
TC0300000327.mm.1		9578.43	11646.23	10317.78	7451.72	6048.40	6518.09
TC0200003615.mm.1		64.18	80.40	71.62	41.41	50.05	43.83
TC0400000223.mm.1	Gm25173	24853.69	23423.50	26193.91	20328.31	18313.44	19725.87
TC0500002689.mm.1	Gm25211	24853.69	23423.50	26193.91	20328.31	18313.44	19725.87
TC1100001603.mm.1	Gm26324	24853.69	23423.50	26193.91	20328.31	18313.44	19725.87
TC1200001300.mm.1	Gm23732	24853.69	23423.50	26193.91	20328.31	18313.44	19725.87
TC1200001425.mm.1	Tubb2a-ps2	338.38	337.28	313.30	203.04	163.71	224.24
TC0300001675.mm.1		63.06	83.98	77.30	40.70	33.19	26.60
TC0200003478.mm.1		398.97	570.77	428.41	198.15	227.71	254.77
TC1800000722.mm.1		54.49	83.04	70.38	34.81	36.09	33.58
TC0300000632.mm.1		101.33	119.63	65.97	37.02	32.34	34.88
TC0700000603.mm.1	Gm24766	22788.33	27742.43	24490.64	18072.95	15270.16	16489.57
TC0700000606.mm.1	Gm25817	22788.33	27742.43	24490.64	18072.95	15270.16	16489.57
TC0100000801.mm.1		35.38	32.58	30.11	24.56	25.76	24.83
TC0X00001167.mm.1		52.43	51.57	52.41	34.45	27.19	36.16
TC1200000445.mm.1	Gm22220	636.91	550.55	532.37	427.06	405.63	381.10
TC1800000913.mm.1		8987.89	8262.60	9147.28	5185.38	3179.71	3562.45
TC1300001319.mm.1	Gm10734	42.46	42.38	39.45	27.73	31.62	32.71
TC1200000366.mm.1		46.49	51.62	46.51	34.01	35.40	38.99
TC0100002279.mm.1		292.65	506.50	253.83	85.34	110.08	105.33
TC1200001160.mm.1	Gm25856	78.65	118.69	94.53	48.73	51.69	43.77
TC1200000637.mm.1		22.07	18.66	21.85	15.29	14.95	15.78
TC1900001087.mm.1		162.72	138.02	173.04	91.71	93.58	69.97
TC0500002318.mm.1	Gm5292	3949.76	3715.83	3819.02	3190.97	2725.71	2923.96
TC0200001469.mm.1	Gm13800; RP23-246E11.1	249.54	216.75	253.46	159.96	133.42	165.90
TC0800003221.mm.1	Gm21811; 2610005L07Rik	12654.92	13771.17	11645.20	8947.34	7565.86	8989.69
TC0600002778.mm.1		7157.04	6578.75	7431.52	5013.66	3742.44	4034.29
TC0X00000540.mm.1	Gm14646; RP23-44A17.1	397.19	350.60	361.66	237.58	176.82	231.84
TC0X00000239.mm.1		111.24	141.99	124.87	83.04	78.17	87.25
TC0100002280.mm.1		705.01	1312.61	747.91	198.51	163.63	297.41
TC1700000174.mm.1		99.22	87.26	75.90	42.69	55.15	41.89
TC0400002653.mm.1	Gm22042	33216.17	30453.32	33224.29	23457.74	17623.88	19008.38
TC1600000682.mm.1	Gm15640; RP23-244J23.1	153.17	132.84	115.37	83.23	66.14	77.33
TC1800000087.mm.1	Mir1b	179.67	178.25	146.86	107.88	117.14	98.53
TC0100001116.mm.1		197.57	178.30	189.99	131.16	118.93	146.36
TC0900002257.mm.1		23.80	16.41	19.07	10.67	11.10	11.01
TC0700000111.mm.1		28.36	29.49	29.65	23.29	22.78	25.54
TC1300000767.mm.1		1380.76	1652.91	1302.98	912.17	907.70	1007.45
TC1000001775.mm.1		89.33	102.28	77.30	51.49	43.74	55.71
TC1300000058.mm.1		21.75	31.74	28.03	11.82	14.21	14.54

TC0600002507.mm.1		19589.43	19534.28	17890.96	13918.95	11932.45	14432.91
TC0300002774.mm.1		305.98	322.49	366.28	242.44	212.32	199.40
TC1700001242.mm.1		144.16	152.86	135.39	100.85	98.90	115.30
TC0400003914.mm.1	Gm13050	11241.06	10638.62	10414.89	8788.15	7544.76	8388.62
TC0100000349.mm.1		23.32	24.31	20.27	16.19	16.93	16.90
TC1500000940.mm.1		209.29	242.30	247.26	154.60	169.88	173.71
TC0800000967.mm.1	Mir7069	146.53	145.73	127.04	107.49	109.72	102.60
TC0600002824.mm.1		60.73	53.53	62.19	33.72	34.05	42.58
TC1700000132.mm.1	Gm23833	44.25	51.53	50.70	33.91	26.31	31.37
TC0300001590.mm.1		136.62	123.27	100.31	75.79	70.01	66.25
TC0500002512.mm.1		84.35	79.75	92.53	50.76	63.56	53.01
TC1100000476.mm.1		3459.69	3324.88	3623.13	1981.29	1515.92	2221.00
TC0X00001615.mm.1		177.73	183.14	202.30	140.33	118.40	137.99
TC1200001943.mm.1	Gm29344; Gm7862	660.08	695.67	610.98	517.67	532.21	483.56
TC1400002786.mm.1	Gm3020; Gm10409	103.66	106.49	99.95	80.00	69.33	82.25
TC0X00001609.mm.1		167.51	184.17	185.24	149.32	133.96	135.77
TC0100000482.mm.1	Gm11581	189.39	128.76	151.06	84.39	88.49	78.99
TC1700001840.mm.1		38.44	38.66	34.47	30.83	29.57	28.64
TC0200000619.mm.1		428.51	586.46	465.44	274.84	281.51	310.23
TC0300002655.mm.1		428.51	586.46	465.44	274.84	281.51	310.23
TC0600001362.mm.1		428.51	586.46	465.44	274.84	281.51	310.23
TC1200000494.mm.1		428.51	586.46	465.44	274.84	281.51	310.23
TC1800001172.mm.1		428.51	586.46	465.44	274.84	281.51	310.23
TC1500000325.mm.1		18.47	21.58	16.90	12.73	13.30	12.65
TC0500001002.mm.1		62.76	62.97	56.89	30.16	24.26	37.99
TC0200002458.mm.1		56.62	54.62	49.17	23.19	34.39	28.58
TC1800000928.mm.1	Gm10557	29568.61	31883.70	28179.01	19437.99	19552.21	23357.30
TC1700002601.mm.1		67.59	89.26	76.87	52.11	48.65	45.69
TC1000002907.mm.1	Cnot2	2411.39	2539.85	2239.64	1731.95	1689.42	1942.69
TC0900000216.mm.1	Zfp809	779.62	718.93	607.77	407.44	296.83	397.59
TC0200000793.mm.1		67.75	79.96	71.05	53.61	48.95	43.55
TC0900001839.mm.1		45.08	40.47	41.80	31.30	33.62	35.13
TC0600001582.mm.1		68.47	91.75	70.65	43.43	48.63	45.33
TC1400000244.mm.1	4931406H21Rik	44.65	50.25	44.03	33.96	37.47	35.13
TC0900002481.mm.1		43.69	55.40	53.67	34.50	34.72	33.95
TC1400002787.mm.1	Gm3002	53.50	49.54	46.89	37.71	33.86	39.27
TC1100002337.mm.1		34.06	44.05	41.70	24.50	26.29	26.81
TC0300002789.mm.1		68.77	71.96	57.35	41.55	30.23	37.24
TC0100002795.mm.1	Gm26683	116.14	126.21	131.67	96.57	98.14	104.07
TC1700000126.mm.1	Gm19270	190.58	197.35	197.95	126.98	81.49	92.90
TC1600001297.mm.1		218.21	265.88	236.67	173.82	156.82	176.01
TC1700000211.mm.1	Spaca6; Mir125a	160.48	183.77	158.85	113.17	97.62	122.02
TC0900003315.mm.1		105.64	112.28	103.24	63.00	44.03	64.13
TC1100003901.mm.1	Gm11663	841.97	958.00	780.55	644.07	580.36	594.09
TC1900000086.mm.1		45.87	63.51	67.23	28.07	20.80	28.94
TC1700002141.mm.1		40.83	55.54	42.13	26.14	28.43	24.10
TC0400000845.mm.1		40.10	45.31	52.54	23.65	29.37	28.28
TC1800001481.mm.1	Mir694	21.24	27.62	31.22	11.06	12.95	14.72
TC1000001267.mm.1		33.81	33.96	31.48	23.27	26.81	26.41
TC0400002425.mm.1	Gm25581	1257.50	1170.92	1196.38	964.14	857.16	777.07
TC0200001961.mm.1		129.55	135.27	126.57	112.34	102.00	98.35
TC0600003456.mm.1	Far2os1; RP23-285C21.5	51.38	50.02	61.63	37.13	38.36	33.18
TC1800000934.mm.1	Gm26682	43.76	43.31	38.41	33.73	33.95	33.26
TC1900001046.mm.1		150.23	196.11	146.87	101.67	102.17	100.48
TC0500001652.mm.1		221.58	250.40	301.06	161.38	163.05	162.58
TC0200002650.mm.1		23.58	27.74	25.42	18.49	19.03	20.38
TC1800000725.mm.1		139.11	149.77	100.64	60.37	41.77	59.97
TC1100000443.mm.1		237.34	345.22	218.19	122.46	74.56	79.41
TC0100000155.mm.1		231.59	346.50	253.55	128.25	118.42	152.18
TSUnmapped00000171.mm.1	Rnu1b2	23427.77	19029.31	23321.93	13515.29	9736.69	12557.66
TSUnmapped00000185.mm.1	Rnu1b1	23427.77	19029.31	23321.93	13515.29	9736.69	12557.66
TC0100002872.mm.1		528.78	663.85	549.31	358.00	264.10	340.40
TC1800000032.mm.1		43.62	61.96	52.74	23.63	31.26	23.59
TC0100001194.mm.1		66.95	74.39	71.42	52.30	49.28	58.05
TC0200001893.mm.1		3100.23	3643.38	3402.62	2392.10	2644.23	2593.63
TC1200002378.mm.1		730.49	782.15	787.71	358.64	172.16	273.95
TC1800001646.mm.1		43.63	47.64	46.09	33.20	37.61	31.48
TC0X00000393.mm.1		222.06	248.12	207.29	68.36	44.40	99.69
TC0400000202.mm.1	Gm11886; RP23-335F19.1	817.65	863.91	776.26	549.19	463.20	604.85
TC1900000383.mm.1	Gm9938	203.78	261.40	176.61	123.87	102.94	106.00
TC1200000170.mm.1	Gm22189	13157.38	15024.61	17111.24	9442.58	10554.14	9641.55
TC0500003558.mm.1	Gm20635; RP23-372N19.1	14.93	15.10	13.82	12.65	11.55	11.79
TC1600000344.mm.1		42.83	51.59	48.05	35.79	35.08	36.54
TC1700000324.mm.1	Mir5125	2613.97	2712.72	2438.93	1552.65	1453.12	1930.37
TC1100002499.mm.1		61.71	84.74	70.77	39.10	36.00	45.81
TC1900001370.mm.1		23.53	27.35	21.98	16.52	13.59	16.25
TC0300002941.mm.1		36.49	32.68	35.92	23.55	26.53	27.77
TC1100002581.mm.1	Gm12158; RP23-103H9.4	51.24	70.04	49.18	26.15	26.64	33.14
TC0200004092.mm.1		98.75	116.26	88.46	61.74	62.43	69.71
TC0300001480.mm.1	Gm17501	97.49	109.33	93.99	71.81	66.31	78.21

TC1400002434.mm.1		66.36	73.95	66.24	56.20	48.46	50.95
TC0600002017.mm.1		61.67	54.28	59.73	41.24	43.55	47.59
TC0500000342.mm.1	E230008O15Rik	80.14	92.79	71.70	55.35	56.05	56.01
TC1200001193.mm.1	Mir453	25.38	21.26	23.30	15.05	17.80	15.03
TC0900000217.mm.1		39.42	35.03	38.67	27.18	30.78	29.47
TC0900000083.mm.1		209.12	203.82	182.04	124.96	116.34	149.16
TC0200001401.mm.1		117.32	150.61	159.91	80.38	86.63	90.17
TC1100002321.mm.1	Gm12040	185.35	212.96	193.67	137.35	123.61	152.19
TC0500001899.mm.1		55.15	63.47	61.29	32.09	28.47	41.18
TC0300001440.mm.1		20.20	21.50	17.62	14.92	13.72	14.45
TC1200000558.mm.1		26791.70	28573.74	25203.26	19221.48	15269.96	19005.59
TC0900002626.mm.1		34.52	33.98	29.82	17.72	18.20	23.59
TC1200001153.mm.1		28.44	34.70	37.33	15.51	21.40	17.41
TC0500000086.mm.1		40.37	41.56	40.19	29.32	22.73	20.44
TC0700001445.mm.1		142.18	145.09	123.33	93.13	79.55	99.30
TC0800002299.mm.1		9744.94	11418.92	9453.27	5645.28	6981.83	6916.84
TC0X00000324.mm.1	Gm6274	1881.19	1841.22	2206.69	1266.79	966.30	1270.55
TC0100000173.mm.1		13710.75	13472.22	13788.02	9198.54	6148.52	7871.20
TC0X00002477.mm.1	Gm8666	553.17	566.32	523.90	418.05	470.60	413.91
TC0900002210.mm.1	Gm24646	186.99	170.72	158.17	122.86	123.90	102.14
TC1800001374.mm.1		62.00	58.70	61.12	43.31	30.02	34.76
TC0500001119.mm.1	Gm8473	12.00	11.93	12.78	9.26	10.40	8.94
TC0300000650.mm.1		2674.75	2774.75	2798.77	1876.04	1415.67	1889.95
TC1500001627.mm.1		61.85	74.81	60.77	38.03	41.78	46.88
TC1400002321.mm.1		25.82	24.91	23.90	17.26	16.38	20.21
TC0100002545.mm.1		363.97	379.62	297.85	167.60	122.11	195.84
TC0X00000602.mm.1	Gm14693; RP23-24K4.3	70.19	79.60	84.50	50.69	53.99	59.57
TC0400002450.mm.1	Mir5123	1976.66	2345.94	1837.70	1053.63	993.68	1363.95
TC0700004139.mm.1		19.04	19.16	18.24	12.37	13.25	15.37
TC1400002250.mm.1		95.97	101.19	68.93	48.93	42.43	48.48
TC1000000115.mm.1		16021.84	17318.72	16874.65	11250.65	8150.46	10900.25
TC0X00001171.mm.1		12983.16	12462.14	12241.50	8920.40	7106.23	9201.02
TC0900001636.mm.1		27.42	29.23	29.15	19.99	18.46	23.10
TC1100000445.mm.1		45.82	63.74	54.71	26.63	34.24	30.35
TC0400002749.mm.1	Rpsa-ps11	223.66	196.52	205.38	173.24	159.93	154.32
TC0200005014.mm.1	Mir5111	67793.02	60342.27	58775.57	47999.94	41390.45	47259.96
TC1000001966.mm.1	Gm7909	134.86	148.73	143.96	115.71	103.10	95.03
TC0900000892.mm.1	Rora	71.35	73.35	67.96	55.27	53.92	61.37
TC0300001636.mm.1		53.57	65.45	58.29	43.04	34.93	39.79
TC0300002464.mm.1	Gm24920	75.38	62.49	78.50	39.70	44.49	50.25
TC1300001230.mm.1	Gm10737	233.14	332.55	183.32	86.32	112.44	87.39
TC0400001254.mm.1	Gm12953; RP23-233B9.5	45.65	53.20	51.70	36.90	34.29	29.14
TC0400000538.mm.1	Gm12437; RP23-141C15.4	244.25	218.74	206.95	158.23	142.26	171.11
TC0400001903.mm.1	Gm13135	23.15	26.42	25.61	20.94	20.23	19.98
TC1500001291.mm.1		18.67	19.74	21.92	15.56	15.99	14.50
TC0500002030.mm.1		47.65	52.85	50.72	37.34	40.29	42.42
TC0700000828.mm.1		100.93	78.63	95.04	64.53	56.45	53.89
TC0500001003.mm.1		393.14	607.95	378.94	219.50	217.82	201.28
TC0700002074.mm.1	Gm26143	251.52	234.75	166.50	100.06	100.10	122.00
TC0500002352.mm.1		78.29	102.02	84.01	59.77	46.57	49.70
TC1600001288.mm.1	Gm24927	20.67	19.98	22.69	17.61	15.80	16.41
TC0400003428.mm.1	Gm12956	14.58	16.08	17.34	11.63	12.76	11.62
TC1800000307.mm.1		41.87	57.17	44.63	24.94	27.89	30.53
TC1200001151.mm.1	DQ267102	9602.52	7301.84	9210.08	5544.22	4006.70	4643.29
TC1200000179.mm.1	Gm19960	167.97	181.81	148.72	117.63	96.60	115.52
TC0900000574.mm.1		129.94	146.21	107.10	79.47	81.34	83.45
TC0200001153.mm.1		23.17	20.43	23.14	18.44	17.85	17.52
TC1100003400.mm.1	Rpl9-ps1	914.94	943.03	1060.47	705.79	615.66	743.83
TC1300002635.mm.1		164.22	149.34	137.05	77.95	104.63	74.50
TC0X00003163.mm.1		35.68	41.25	40.65	31.34	29.35	31.26
TC1300001232.mm.1		29.54	32.07	26.44	22.89	20.80	20.71
TC0500001968.mm.1	Gm24054	46.18	54.83	56.24	34.23	34.51	39.51
TC0400002118.mm.1		345.14	379.99	353.53	230.71	156.73	217.03
TC0X00001346.mm.1	Gm8019	588.72	498.06	493.40	407.39	363.03	372.26
TC0100002268.mm.1	Gm24180	52.44	45.02	47.53	39.66	37.56	36.85
TC0100001325.mm.1		394.98	557.89	473.78	231.60	167.39	257.65
TC0100001326.mm.1		394.98	557.89	473.78	231.60	167.39	257.65
TC0100001327.mm.1		394.98	557.89	473.78	231.60	167.39	257.65
TC0200001569.mm.1		394.98	557.89	473.78	231.60	167.39	257.65
TC0200001615.mm.1		394.98	557.89	473.78	231.60	167.39	257.65
TC0800000456.mm.1		394.98	557.89	473.78	231.60	167.39	257.65
TC0X00000800.mm.1		394.98	557.89	473.78	231.60	167.39	257.65
TC0X00001094.mm.1		394.98	557.89	473.78	231.60	167.39	257.65
TC0X00001200.mm.1		394.98	557.89	473.78	231.60	167.39	257.65
TC1000001319.mm.1		394.98	557.89	473.78	231.60	167.39	257.65
TC1000002465.mm.1		394.98	557.89	473.78	231.60	167.39	257.65
TC1200000168.mm.1		394.98	557.89	473.78	231.60	167.39	257.65
TC1200001486.mm.1		394.98	557.89	473.78	231.60	167.39	257.65
TC1200001838.mm.1		394.98	557.89	473.78	231.60	167.39	257.65
TC1200002489.mm.1		394.98	557.89	473.78	231.60	167.39	257.65

TC1600000722.mm.1		394.98	557.89	473.78	231.60	167.39	257.65
TC1800001393.mm.1		394.98	557.89	473.78	231.60	167.39	257.65
TC1900001143.mm.1		394.98	557.89	473.78	231.60	167.39	257.65
TC0200003871.mm.1		98.10	119.14	107.24	70.31	74.58	82.87
TC1500001512.mm.1		133.85	126.13	114.60	87.99	65.28	80.80
TC0800001059.mm.1		28.81	25.45	30.76	22.18	20.47	20.83
TC0700003814.mm.1		75.89	75.97	76.26	52.65	47.96	61.54
TC0700001418.mm.1		150.32	209.40	164.72	84.87	78.39	108.06
TC0200000746.mm.1		34.90	42.51	38.64	25.98	29.23	24.09
TC0100000838.mm.1	Gm15372	68.44	68.78	66.43	56.08	44.94	48.12
TC0500000123.mm.1		40.53	40.22	34.09	26.60	29.53	25.44
TC0100000051.mm.1		8093.93	10080.92	9294.98	6418.03	4839.94	4867.53
TC0500002011.mm.1		87.72	116.96	88.54	63.53	54.72	59.53
TC1300000155.mm.1		51.04	82.29	70.11	36.08	32.05	30.60
TC0400000737.mm.1		27.56	44.40	28.73	15.67	15.77	16.13
TC0200003567.mm.1	Gm13532	2978.87	2611.21	3425.11	1933.35	1927.90	2131.39
TC1900000288.mm.1		54.26	67.25	48.08	34.21	33.54	36.39
TC0200002845.mm.1		18.19	16.57	15.22	12.83	11.17	12.45
TC0800002345.mm.1		16.65	14.12	16.70	11.58	10.40	12.02
TC1600001717.mm.1		25948.81	30397.81	29701.57	21345.87	16770.52	19398.67
TC1700001338.mm.1		585.29	755.96	586.81	422.18	383.42	328.85
TC1200000518.mm.1		292.87	393.73	291.63	206.11	181.75	199.28
TC0300000793.mm.1		178.40	204.74	215.39	156.30	145.27	143.34
TC1000002860.mm.1		24.76	31.87	25.13	15.71	18.87	16.80
TC1600001669.mm.1		471.65	755.38	530.88	212.42	170.00	285.28
TC1200000344.mm.1		31.99	27.16	29.16	22.37	23.99	22.63
TC0X00000162.mm.1	Gm15027	7881.24	8052.52	7337.62	6379.33	5289.95	5838.36
TC0X00000163.mm.1	Gm14536	7881.24	8052.52	7337.62	6379.33	5289.95	5838.36
TC1700002607.mm.1		440.90	567.56	456.55	166.68	185.03	284.79
TC1400001509.mm.1	Gm20699	67.29	72.90	59.70	45.43	51.39	44.42
TC0800002365.mm.1		188.86	182.45	162.95	131.15	121.09	142.38
TC0700004594.mm.1		25870.16	28346.22	27458.91	18254.75	11509.71	14091.98
TC0200000865.mm.1	Gm13519; RP23-35D6.1	781.42	819.30	641.40	536.81	454.69	472.42
TC1300000941.mm.1		23.85	22.71	24.08	18.08	16.00	19.45
TC1100000482.mm.1	Gm12174	11607.08	9968.13	11406.52	7819.58	7340.70	8700.48
TC0100000843.mm.1	Gm15368	105.21	101.59	103.22	74.09	61.10	79.60
TC0400003367.mm.1		5012.43	5096.13	5111.66	3451.42	2512.73	2159.84
TC0100001666.mm.1		32.64	32.00	35.45	25.38	28.29	24.58
TC0600000189.mm.1		25.67	27.35	24.39	20.69	20.09	17.53
TC1100001762.mm.1	Gm11652	75.58	63.72	67.96	54.20	55.39	52.64
TC0900002636.mm.1	BC065403	34.88	34.22	36.34	26.86	24.75	29.74
TC0800002479.mm.1		45.80	73.57	60.02	30.55	31.80	29.82
TC0100000781.mm.1		97.76	110.06	96.70	76.59	66.62	79.28
TC1600001670.mm.1		101.40	114.30	101.61	58.76	77.68	56.19
TC1200000827.mm.1	Prox2os; RP24-89J3.3; Gm19357	50.12	53.54	51.92	44.38	38.59	37.55
TC0X00003080.mm.1	Gm7091	1939.56	1799.83	1900.67	1427.28	1504.22	1222.00
TC0900001794.mm.1		20.27	25.94	20.25	15.26	13.09	12.26
TC1600000356.mm.1	Morf4l1-ps1	96.37	128.81	89.10	59.38	63.15	60.04
TC1800001644.mm.1		66.00	107.88	77.82	40.43	32.40	41.88
TC0100001695.mm.1		60.11	79.12	88.30	32.05	44.19	30.73
TC0X00002363.mm.1	Gm14689; RP23-24K4.2	575.88	709.03	540.33	354.67	289.37	392.54
TC0100000837.mm.1	Gm15373	86.66	104.62	83.38	56.18	63.43	65.45
TC0300000439.mm.1		250.53	248.09	262.37	139.10	110.90	173.02
TC0600001731.mm.1		250.53	248.09	262.37	139.10	110.90	173.02
TC0600002537.mm.1		250.53	248.09	262.37	139.10	110.90	173.02
TC0600003306.mm.1		250.53	248.09	262.37	139.10	110.90	173.02
TC0900002567.mm.1		250.53	248.09	262.37	139.10	110.90	173.02
TC0X00000379.mm.1		250.53	248.09	262.37	139.10	110.90	173.02
TC0X00003344.mm.1		250.53	248.09	262.37	139.10	110.90	173.02
TC1400001128.mm.1		250.53	248.09	262.37	139.10	110.90	173.02
TC1400002705.mm.1		250.53	248.09	262.37	139.10	110.90	173.02
TC1500000481.mm.1		250.53	248.09	262.37	139.10	110.90	173.02
TC1800000768.mm.1		250.53	248.09	262.37	139.10	110.90	173.02
TC1800001570.mm.1		250.53	248.09	262.37	139.10	110.90	173.02
TC1900001634.mm.1		250.53	248.09	262.37	139.10	110.90	173.02
TC0600002152.mm.1		22030.11	21241.13	22379.78	16633.40	11945.94	13340.63
TC0300000391.mm.1		61.98	93.65	61.15	37.54	32.79	26.87
TC1300000109.mm.1		24.97	26.10	22.94	20.14	17.25	18.94
TC0100001538.mm.1	Gm22992	37182.27	43898.46	41036.86	28838.65	30677.53	32699.49
TC0100001819.mm.1	Gm24092	37182.27	43898.46	41036.86	28838.65	30677.53	32699.49
TC0400003225.mm.1	Gm25123	37182.27	43898.46	41036.86	28838.65	30677.53	32699.49
TC0X00000510.mm.1	Gm22103	37182.27	43898.46	41036.86	28838.65	30677.53	32699.49
TC1400001826.mm.1	Gm25892	37182.27	43898.46	41036.86	28838.65	30677.53	32699.49
TC1400001836.mm.1	Gm23620	37182.27	43898.46	41036.86	28838.65	30677.53	32699.49
TC1400001901.mm.1	Gm25353	37182.27	43898.46	41036.86	28838.65	30677.53	32699.49
TC0100001544.mm.1		78.32	90.44	103.17	63.53	51.38	54.31
TC1300002591.mm.1		38.03	63.29	55.05	24.73	25.59	25.18
TC0600000589.mm.1		423.90	577.27	442.40	293.99	274.71	314.64
TC0700000253.mm.1		2055.36	2199.72	2212.44	1564.72	1109.60	1092.40

TC1200000333.mm.1		82.05	71.18	54.12	38.31	31.88	26.05
TC0X00000874.mm.1	Gm14778; RP23-335N15.2	476.24	439.96	464.63	325.34	252.12	332.20
TC1700000690.mm.1		5782.87	5516.52	5923.29	3496.78	2151.98	3160.82
TC0X00000232.mm.1	Gm25812	138.43	116.33	109.75	89.45	86.00	85.80
TC1600001958.mm.1		34.10	35.34	31.55	22.14	24.28	27.25
TC0300002588.mm.1		29.52	32.69	34.58	25.40	23.55	26.02
TC0200005374.mm.1		26.58	33.44	30.90	20.95	21.79	22.60
TC0900000886.mm.1		288.86	272.77	296.89	195.41	237.27	215.87
TC0500002511.mm.1		81.40	77.42	76.97	65.09	52.58	53.73
TC1800000716.mm.1		30.84	25.60	30.16	22.03	21.83	20.22
TC1300002480.mm.1		53.32	51.76	57.36	41.45	37.26	44.57
TC0100003267.mm.1	Gm7278	68.84	71.01	57.32	44.48	43.46	49.48
TC1600001614.mm.1	Gm15530	267.27	256.47	278.85	186.33	181.43	221.99
TC0400000182.mm.1		28.30	31.73	29.91	25.42	23.04	22.12
TC1000000070.mm.1	Gm25682	24345.99	23393.09	26748.34	17794.91	12467.58	15033.82
TC0600001240.mm.1		126.12	159.00	142.70	88.32	61.75	85.16
TC0100001709.mm.1		45.42	56.92	51.68	30.83	33.39	37.97
TC0800000636.mm.1	Gm26905	1982.83	2742.02	2058.85	1265.23	767.42	1005.37
TC0200003472.mm.1		71.82	96.08	69.07	49.61	45.76	48.28
TC1800000054.mm.1	Gm7502	365.88	344.51	377.08	312.90	272.93	277.55
TC0700003883.mm.1	Gm23550	46322.02	55267.10	49379.36	35232.19	39962.36	37660.28
TC0400002602.mm.1		5142.97	5523.00	5179.58	3497.21	3465.79	2479.06
TC0400004024.mm.1	Gm13204	126.79	130.15	120.09	109.99	98.38	97.03
TC1700002611.mm.1		133.42	144.84	131.89	88.96	72.17	100.13
TC0900000897.mm.1		54.59	54.69	50.30	24.70	36.66	32.17
TC0X00003094.mm.1	Gm15298; RP23-232B3.4	599.99	735.46	843.18	405.23	417.98	477.83
TC0700000455.mm.1		89.22	68.72	70.95	46.89	43.15	53.07
TC0200000774.mm.1	Gm13468; RP23-103G12.1	291.89	296.25	319.23	209.04	223.49	252.59
TC0400001834.mm.1	Gm13086; RP23-451D4.10	47.25	40.97	44.90	36.69	32.92	31.95
TC0200000638.mm.1	Gm13443	1891.57	1673.66	1308.52	1018.03	906.96	947.20
TC1700002704.mm.1		902.97	1067.78	703.85	487.10	518.93	501.70
TC1300000441.mm.1	Gm27387	292.61	297.58	289.20	253.73	214.26	215.94
TC0900002646.mm.1	LOC100861984	410.82	353.66	337.10	285.15	262.38	271.49
TC0400001511.mm.1		90.73	94.31	94.85	68.79	65.93	79.69
TC1400001304.mm.1		80.41	92.97	81.62	55.23	56.02	67.31
TC0800001364.mm.1		31.11	32.66	33.27	23.75	25.48	19.95
TC1700001324.mm.1		21.29	22.58	21.08	17.41	14.06	13.46
TC0300001305.mm.1		64.05	66.69	65.60	46.84	51.85	56.00
TC1300000419.mm.1		284.00	348.77	326.12	247.55	217.77	226.67
TC0100001575.mm.1	Gm26110	19928.76	16798.67	20696.59	11931.37	7074.06	7779.75
TC1200001143.mm.1	Mir341	53.42	65.47	62.75	35.65	23.27	34.41
TC0500002049.mm.1		43.24	34.97	41.71	29.68	29.31	29.66
TC1700001179.mm.1		195.42	142.73	132.39	81.97	83.70	94.90
TC0900001218.mm.1	C430002N11Rik	247.70	212.36	217.98	182.79	159.81	170.50
TC1200001669.mm.1		34.00	34.81	31.09	21.80	25.44	26.38
TC0300001804.mm.1	Gm15574	85.14	90.13	90.64	69.45	59.22	71.90
TC0900001676.mm.1		128.61	138.65	116.50	82.82	66.55	90.73
TC0100003358.mm.1		18.16	15.88	13.82	10.64	11.16	11.22
TC1300001454.mm.1		374.61	533.85	440.04	196.05	146.71	249.72
TC1700000135.mm.1		88.13	85.24	61.57	41.63	48.22	43.98
TC0900001994.mm.1		427.00	646.74	542.62	285.42	269.66	324.67
TC1200001509.mm.1	2410018L13Rik	56.71	59.63	58.82	44.94	36.67	45.60
TC1600000624.mm.1	Gm25873	88.13	74.41	67.32	54.17	51.69	46.73
TC0400002966.mm.1		66.51	75.71	59.95	42.68	26.93	34.81
TC0200004091.mm.1		208.95	265.69	208.13	125.16	158.26	140.13
TC0400000203.mm.1	Gm11887; RP23-335F19.2	474.55	547.17	431.14	335.43	351.54	358.46
TC0700003422.mm.1		31.75	33.02	32.32	24.77	21.54	18.00
TC0600002649.mm.1		35.54	37.02	32.43	26.82	26.50	29.57
TC0800001817.mm.1		158.12	133.26	143.84	110.17	113.30	118.02
TC0100002352.mm.1		44.87	55.27	46.04	34.02	36.39	30.88
TC0800002074.mm.1		88.15	82.87	59.23	44.63	43.25	40.98
TC0600000639.mm.1	Gm25818	3375.23	3552.76	3628.99	2282.39	1318.63	1437.91
TC1400000312.mm.1		84.63	85.80	75.39	57.02	46.08	60.44
TC0500000140.mm.1		53.51	56.81	41.95	30.42	28.92	34.59
TC0700003633.mm.1		1448.90	1778.25	1353.60	1021.32	749.52	896.56
TC1500002104.mm.1		25.75	32.64	28.17	19.00	19.32	14.83
TC0100000441.mm.1		60.72	70.43	68.58	47.57	48.31	54.68
TC0100000938.mm.1		70.20	68.22	62.08	41.44	44.72	53.21
TC1700002387.mm.1		30.09	40.32	33.25	23.45	20.49	23.27
TC0200001155.mm.1		3839.21	4693.98	3947.28	2913.99	2552.06	3093.07
TC1300001235.mm.1		15.96	25.38	20.17	11.14	9.89	11.39
TC1800000742.mm.1		85.58	73.60	64.24	47.07	36.55	47.72
TC0300002902.mm.1		23.49	18.38	22.72	15.34	14.82	15.38
TC0X00001568.mm.1	Gm8569	11.73	10.26	12.46	8.60	9.09	8.67
TC1000000999.mm.1		121.71	252.24	129.06	57.30	55.29	40.54
TC0X00000868.mm.1	Gm14789; RP23-57I15.2	2745.01	2978.94	2745.96	1652.90	1375.17	2050.88
TC0200003036.mm.1		109.21	128.96	97.10	59.48	49.71	73.28
TC1600001496.mm.1	Hmgb1-ps6	5240.59	5322.07	5203.68	4492.97	3707.42	3702.88
TC1800000012.mm.1		103.65	99.56	88.21	78.43	78.63	74.33
TC1600001535.mm.1		25.66	30.10	31.00	18.50	22.42	19.54

TC0400002226.mm.1		32.49	39.08	35.02	28.29	24.36	25.86
TC1700002024.mm.1	Lgals1-ps1	36.20	30.15	34.30	26.92	23.93	24.72
TC1200000362.mm.1		89.50	128.04	103.97	56.96	59.89	70.20
TC1000001854.mm.1	Gm26740; RP24-547D11.2	47.88	50.98	50.05	38.38	36.79	29.76
TC1000001080.mm.1		196.20	215.48	210.67	141.53	128.07	166.72
TC0200003477.mm.1		109.29	141.70	116.64	56.26	72.40	79.74
TC1600000668.mm.1		165.52	164.01	129.78	92.48	79.46	105.72
TC0700001096.mm.1		4289.25	4527.05	4464.64	3566.96	2824.07	3350.88
TC0900000576.mm.1		78.93	91.78	77.56	41.04	55.24	55.60
TC0500001489.mm.1	Gm17132	14066.66	14695.59	15274.16	10322.81	6540.44	8027.83
TC0600001817.mm.1	Gm8649	467.01	527.23	520.42	349.24	224.00	282.08
TC0800000999.mm.1		59.48	64.56	52.95	44.69	40.29	45.57
TC0400000605.mm.1	Gm19609	13.31	16.13	16.84	10.50	10.07	11.55
TC1200001316.mm.1	Gm24741	40.09	48.05	50.65	32.93	32.33	27.29
TC0800000886.mm.1		63.01	62.34	61.45	37.67	43.50	50.09
TC1800000743.mm.1		38.31	35.91	34.36	28.09	22.54	27.44
TC0700001322.mm.1		54.66	68.59	54.27	40.95	37.86	31.50
TC0500002261.mm.1		84.25	108.97	96.78	71.75	63.64	60.83
TC0700002681.mm.1		503.98	486.63	576.20	295.56	168.44	268.10
TC0600003051.mm.1		27.05	22.74	26.11	20.28	19.86	19.91
TC0200002098.mm.1	Gm14079; RP23-139H14.3	39.17	44.69	35.21	29.84	27.52	29.01
TC1100002115.mm.1		67.05	57.76	50.86	39.48	30.35	26.81
TC0600001911.mm.1	Gm20186	630.69	657.87	673.67	558.32	515.53	464.50
TC0900000787.mm.1		11.87	13.91	15.09	9.44	10.02	10.10
TC0800001270.mm.1		30.23	28.91	26.71	23.38	18.94	19.00
TC0900001505.mm.1		62.62	88.66	74.15	46.95	49.23	41.84
TC1200000374.mm.1		15.56	17.49	20.37	13.11	11.91	11.62
TC0100001045.mm.1		422.69	588.26	442.89	294.11	265.20	316.49
TC1200001720.mm.1		125.26	107.87	82.16	59.84	44.21	56.58
TC0400003947.mm.1	Gm13121	2958.94	2956.73	2967.22	2619.38	2224.61	2248.99
TC1000001126.mm.1		60.57	68.07	60.09	41.36	37.04	49.18
TC0200002831.mm.1		156.98	172.08	177.09	136.85	112.86	129.03
TC1000000106.mm.1		34.18	48.15	35.29	19.46	24.44	16.97
TC0600002833.mm.1		78.74	87.72	102.90	56.54	64.80	51.47
TC0200002061.mm.1		17.26	20.37	17.98	15.09	13.26	12.89
TC0800000819.mm.1		11524.00	12844.29	12233.85	10425.38	9178.75	8839.85
TC0900000020.mm.1		758.10	803.41	863.03	589.34	397.09	477.12
TC0500001811.mm.1		129.39	176.99	127.65	86.98	78.30	93.31
TC1600000633.mm.1		60.02	79.85	77.24	45.86	40.65	49.71
TC1300000036.mm.1		2530.09	2499.04	2214.82	1695.40	1693.32	1272.56
TC0100001321.mm.1		359.29	377.03	356.18	236.85	216.95	292.71
TC0300001517.mm.1		359.29	377.03	356.18	236.85	216.95	292.71
TC0800000071.mm.1		359.29	377.03	356.18	236.85	216.95	292.71
TC0800000165.mm.1		359.29	377.03	356.18	236.85	216.95	292.71
TC1000001265.mm.1		359.29	377.03	356.18	236.85	216.95	292.71
TC1100002198.mm.1		359.29	377.03	356.18	236.85	216.95	292.71
TC1300001262.mm.1		359.29	377.03	356.18	236.85	216.95	292.71
TC1400001168.mm.1		359.29	377.03	356.18	236.85	216.95	292.71
TC1500001159.mm.1		359.29	377.03	356.18	236.85	216.95	292.71
TC1600001649.mm.1		359.29	377.03	356.18	236.85	216.95	292.71
TC0700000993.mm.1		35.77	39.13	32.20	28.04	27.56	25.21
TC0900000880.mm.1		31.71	31.16	33.87	24.82	28.19	24.34
TC1300001181.mm.1		47423.25	51881.60	55097.22	34042.83	19843.48	21354.04
TC1600000798.mm.1		339.87	379.98	286.79	208.65	189.02	239.22
TC1800000751.mm.1		89.87	107.87	71.03	47.26	45.43	55.78
TC1200001936.mm.1		40.48	39.35	30.42	23.11	23.22	25.79
TC0400001449.mm.1	Csf3r	233.60	236.32	209.94	188.79	161.37	177.91
TC0900001911.mm.1	Gm2976	3512.43	3603.96	3289.59	2911.75	2396.87	2675.93
TC1800000720.mm.1		23.22	19.33	21.78	16.65	17.36	16.05
TC0200003838.mm.1		76.79	64.00	64.43	50.71	36.89	40.41
TC1600000910.mm.1		1146.86	1263.95	1188.34	945.03	981.96	812.12
TC0100002881.mm.1		243.81	243.20	259.17	181.80	150.45	196.55
TC1800000481.mm.1		225.03	253.59	217.66	174.75	137.42	168.14
TC0900003045.mm.1		67.20	91.19	70.72	38.61	39.69	51.73
TC1100002596.mm.1	Gm16033; Gm16034; RP23-29H5.7; RP23-29H5.6	242.99	215.19	203.36	144.42	98.50	138.25
TC0300001110.mm.1	AI504432	190.54	201.06	164.85	109.59	122.35	141.11
TC1800001604.mm.1		74.04	82.67	63.76	51.61	41.37	49.74
TC0500000501.mm.1		54.55	80.21	82.30	30.05	34.47	42.13
TC1500001960.mm.1	Tbrg3	53.34	69.77	54.64	33.24	33.88	42.04
TC0X00002196.mm.1	Gm7834	61.03	57.83	59.17	51.03	41.60	43.46
TC0100000340.mm.1		74.67	72.49	65.11	47.10	52.69	38.20
TC0300002753.mm.1		75.41	93.37	84.02	52.99	48.98	63.83
TC0700003115.mm.1	Gm6226	85.60	86.17	77.39	70.14	60.38	66.20
TC1900000686.mm.1		55.62	64.04	55.71	48.03	43.57	40.93
TC0600003009.mm.1		34.72	34.80	41.73	19.56	19.70	27.12
TC1600000363.mm.1		144.74	136.69	140.20	74.47	45.02	80.62
TC0100000864.mm.1		48.66	59.45	59.78	39.20	42.42	41.75
TC1000001976.mm.1		11724.43	13930.09	12559.86	9627.05	7045.93	8169.58
TC1200001521.mm.1		64.22	75.57	59.94	44.67	41.48	31.49

TC0100003701.mm.1		57.43	75.30	69.57	44.51	32.89	42.60
TC0500000891.mm.1		364.66	352.24	351.90	267.90	236.23	298.63
TC1000002214.mm.1		33.22	41.95	33.38	23.94	25.62	26.55
TC1600000361.mm.1		159.99	213.10	154.28	76.48	112.50	89.29
TC1600001065.mm.1	Gm15971	98.84	98.72	84.90	58.27	57.57	73.80
TC0200002858.mm.1	Gm13187	13.59	15.40	15.98	12.04	11.82	10.62
TC1300002367.mm.1		1415.53	1568.95	1949.68	1140.59	963.40	918.08
TC1300001502.mm.1		43.99	43.37	52.63	35.05	28.83	26.32
TC1800001373.mm.1		38.78	38.60	33.75	19.58	25.52	26.53
TC0800003240.mm.1	6820431F20Rik; 2610005L07Rik	1900.53	2033.48	1711.29	1385.59	1165.92	1443.94
TC1300002476.mm.1		5325.91	5447.29	4569.86	4140.98	3634.36	3661.82
TC0100001917.mm.1		6459.89	7348.58	7515.16	5786.69	4898.74	5379.25
TC1800000723.mm.1		372.75	315.89	246.75	186.16	152.81	184.30
TC0600000825.mm.1		424.25	534.35	438.78	312.92	263.53	335.52
TC0900001970.mm.1		15761.62	16331.11	13709.10	9895.97	7824.15	10961.33
TC0100003684.mm.1		191.12	198.76	161.40	117.53	135.34	138.13
TC0600002933.mm.1		73.93	69.40	65.71	49.95	42.47	55.29
TC1100001447.mm.1	Gm22456	41079.14	44148.99	48880.39	28858.48	17423.16	15955.86
TC0500002304.mm.1		112.02	136.20	101.61	80.85	64.49	75.68
TC0200002944.mm.1	Gm23608	73.69	83.70	90.77	65.46	61.42	62.91
TC1400002304.mm.1		1413.83	1724.55	1448.32	994.10	630.76	872.63
TC0500001094.mm.1		25.65	32.95	31.41	22.25	21.33	20.57
TC0300001728.mm.1		18.27	14.99	14.72	12.24	11.68	11.50
TC1800001690.mm.1		4349.06	5702.56	4675.22	3189.98	2450.88	3242.90
TC1700002388.mm.1		69.65	105.21	110.96	52.78	48.89	42.46
TC1200001247.mm.1		132.21	131.25	144.98	117.07	101.97	99.30
TC1700001434.mm.1		159.39	171.72	151.49	99.70	79.39	117.60
TC0200003614.mm.1		20.25	21.14	23.29	12.70	16.86	14.81
TC0700002401.mm.1	Mrip-ps	8108.88	7373.37	7986.10	5923.66	4761.53	6059.37
TC0300001437.mm.1		28.80	29.34	30.85	20.09	24.47	18.64
TC1700000130.mm.1		42.22	54.24	45.77	28.20	26.57	34.96
TC0600000464.mm.1		20.45	30.86	25.29	14.82	15.64	15.44
TC0300000737.mm.1	Gm17146; RP23-199B2.2	153.14	183.84	164.53	128.53	114.70	132.71
TC0400001689.mm.1		492.78	580.04	514.48	281.72	361.53	383.62
TC0200003476.mm.1		460.93	681.14	391.25	208.09	201.33	272.98
TC1100001420.mm.1		85.76	97.72	68.91	56.44	50.40	45.00
TC0200000255.mm.1		15.44	17.12	18.37	13.05	12.30	13.95
TC0800002247.mm.1		75619.49	89663.33	73775.03	56093.80	38720.05	48979.85
TC1200001710.mm.1		35.43	42.38	35.73	19.73	28.01	20.65
TC1700002532.mm.1		49.47	68.76	53.75	35.89	36.10	39.24
TC1300000577.mm.1		3157.32	3277.63	2858.51	2018.77	1660.38	2335.74
TC1200000706.mm.1		18.99	18.91	16.14	14.79	13.43	13.62
TC1100003244.mm.1		39.97	51.73	50.33	28.30	26.29	34.22
TC1700002466.mm.1	2410021H03Rik	35.68	34.55	31.18	24.42	28.11	27.07
TC0X00000388.mm.1	Gria3	649.07	806.49	630.61	464.98	409.40	506.46
TC1100003946.mm.1	Rps12-ps26	827.85	718.32	838.77	613.06	529.55	626.36
TC1600001666.mm.1		30.28	27.24	30.25	24.20	20.97	23.98
TC0500002761.mm.1		23.47	21.51	22.93	14.59	18.58	16.92
TC0200001402.mm.1		40.54	36.60	43.41	24.52	23.10	31.23
TC0500002726.mm.1		140.78	181.93	141.75	100.11	72.22	96.68
TC0200002899.mm.1		73.04	72.16	84.24	40.47	49.88	56.66
TC0200005256.mm.1		21.45	19.03	21.59	14.52	17.23	14.16
TC0700001209.mm.1		62.06	63.42	59.77	49.43	47.07	38.49
TC0300002646.mm.1		24.14	25.60	34.86	16.39	16.57	18.29
TC1800001010.mm.1	Gm24924	121758.90	112293.20	123891.20	95868.23	70879.41	78914.02
TC1100004153.mm.1	Gm24060	149.60	195.96	155.79	111.29	71.31	86.73
TC0300000286.mm.1		34.21	44.28	32.89	21.12	24.94	24.92
TC0100000840.mm.1	Gm15369	92.52	77.01	85.96	63.23	65.18	52.96
TC0700001346.mm.1		126.61	166.68	117.37	88.72	86.82	75.59
TC0600000364.mm.1		186.83	189.18	166.00	127.79	102.06	136.34
TC0X00002945.mm.1	Gm14969; RP23-318H22.2	503.78	520.01	424.00	289.27	210.95	321.00
TC0300003043.mm.1		36.90	56.90	34.79	21.89	22.69	21.73
TC0800001405.mm.1	Mir7077	17.82	16.52	16.90	14.70	12.81	11.97
TC0X00003148.mm.1		21.83	21.27	23.61	15.76	18.87	15.46
TC1000003182.mm.1	Gm23252	89.78	86.78	71.75	61.07	50.67	59.34
TC0700004438.mm.1		117.80	134.06	144.85	56.22	55.64	90.28
TC1300000994.mm.1	Gm24935	57.90	47.69	57.60	38.92	42.96	40.28
TC1000002747.mm.1		406.27	500.78	424.63	300.65	249.93	328.09
TC1000002922.mm.1		116.17	106.30	137.04	76.51	78.89	91.08
TC0300001386.mm.1		97.68	104.08	83.02	73.58	63.86	60.68
TC0900000902.mm.1		23.96	28.02	28.25	18.13	21.15	20.58
TC1500001301.mm.1		62.18	63.05	54.29	47.30	35.71	37.05
TC0600001864.mm.1		25.33	26.22	29.92	20.25	21.85	18.11
TC0200001785.mm.1		69.08	76.00	67.09	60.77	53.66	52.37
TC0500002553.mm.1		6776.17	7266.12	8230.11	5440.23	3966.04	3633.32
TC0400003238.mm.1		71.13	90.09	77.44	59.21	60.67	59.35
TC1800000958.mm.1		416.83	511.00	437.69	309.76	257.67	339.61
TC1100003660.mm.1	Gm22461	4352.58	4143.25	5106.02	3216.66	2853.52	2241.18
TC0600000336.mm.1		106.64	138.43	124.40	63.21	63.37	88.67

TC0900001189.mm.1		88.33	115.81	102.68	66.89	73.91	74.88
TC0600003011.mm.1		30.89	30.17	25.83	16.00	21.96	15.87
TC0400002964.mm.1		70.85	96.47	68.99	34.63	48.34	28.26
TC1200002018.mm.1		68.82	109.37	66.81	34.68	41.47	43.10
TC0200001696.mm.1	Gm13975	14.24	15.51	13.04	11.17	11.93	11.16
TSUnmapped00000169.mm.1	Rnu1a1	118852.80	112854.10	125049.90	95360.95	70098.11	80741.01
TC0300001174.mm.1		50.59	102.09	56.56	29.05	22.97	27.44
TC1100004012.mm.1		442.91	363.41	355.82	275.24	276.29	301.34
TC0900001762.mm.1		5116.49	8048.81	6558.14	2289.17	3723.81	3080.98
TC1900001376.mm.1		27.53	37.34	28.14	16.71	20.65	20.06
TC0500000143.mm.1		42.05	51.11	43.50	31.49	22.51	30.29
TC1600002100.mm.1		70.98	101.32	86.03	56.01	45.67	55.33
TC1400001263.mm.1		29.24	46.12	40.18	16.89	23.46	17.76
TC0600000259.mm.1		112.97	122.27	109.38	66.98	67.29	91.34
TC1000001280.mm.1		10318.40	10927.48	11001.25	7609.57	4547.70	5878.73
TC1800001032.mm.1		48.93	53.97	55.78	22.68	34.18	34.20
TC0X00001649.mm.1	Gm8737	207.57	199.60	247.35	122.61	157.90	106.23
TC0200004287.mm.1	Gm9864	826.73	868.06	789.16	638.64	519.03	657.31
TC1600000874.mm.1		60.94	64.77	55.99	23.20	25.02	41.17
TC0900000093.mm.1		108.66	142.48	123.09	74.32	64.46	89.74
TC0200003858.mm.1	Gm13684; RP23-141N19.2	40.68	52.78	54.55	32.81	32.69	34.82
TC0200004393.mm.1	Gm25514	6364.04	4542.32	6454.56	3487.81	1976.31	2182.35
TC0200003644.mm.1		11.37	12.27	12.04	7.39	7.97	9.84
TC1300002590.mm.1		77.35	115.76	84.56	38.15	50.30	54.89
TC1200000450.mm.1	Gm22513	109307.80	102393.10	113042.90	86095.04	61606.02	64118.98
TC0400000145.mm.1	Gm11863; RP23-415F19.1	697.24	794.50	661.15	459.17	316.66	471.61
TC1400001046.mm.1		16.60	16.47	21.83	12.97	11.08	12.04
TC0500003424.mm.1		171.25	201.56	144.46	113.86	120.48	109.97
TC1600000453.mm.1		103.55	94.19	98.91	59.55	78.04	54.42
TC0200001400.mm.1	Gm13786	42.65	43.26	45.74	35.94	29.45	26.46
TC1000002911.mm.1		59.53	60.51	54.24	51.43	46.86	47.40
TC0600000313.mm.1		61.68	96.07	90.40	45.99	38.02	47.74
TC0100000028.mm.1		46.65	58.87	60.76	39.92	36.76	39.94
TC1000000821.mm.1	Mir6910	150.07	180.98	163.53	125.64	136.04	128.64
TC0300002626.mm.1		820.74	921.46	910.66	655.96	453.01	586.24
TC0200004346.mm.1		53.20	44.42	45.96	32.56	25.79	35.30
TC1700002603.mm.1		92.67	137.76	85.03	52.30	43.52	59.75
TC0800002079.mm.1		40.37	57.83	40.74	20.67	25.46	28.99
TC0200005348.mm.1	Gm16362	46.74	47.85	42.78	37.22	39.36	33.92
TC0100001488.mm.1	Gm20471; RP24-485J8.2	28.54	36.32	32.34	24.92	21.28	23.48
TC0900000577.mm.1		29.63	31.92	31.41	27.79	24.22	24.47
TC1100003951.mm.1		23.98	23.31	23.57	16.91	15.89	20.32
TC1600000061.mm.1		72.71	68.81	75.18	54.19	58.15	44.68
TC0700001056.mm.1	Kansl2-ps	895.76	946.09	777.33	584.84	466.23	642.63
TC1100001745.mm.1		120.15	116.82	101.56	81.42	92.03	75.26
TC1000001199.mm.1		147.54	183.20	160.48	126.78	121.86	129.38
TC1200000701.mm.1		24.98	29.40	31.24	21.04	22.20	19.14
TC1800000905.mm.1		2014.44	2222.79	1905.47	1575.91	1219.79	1509.35
TC0200005260.mm.1	Gm14263	10046.64	9601.51	11442.02	8532.01	7271.01	7155.90
TC0900002413.mm.1	Gm20199	121.80	120.92	105.16	88.62	60.61	66.00
TC1400002269.mm.1		77.97	69.06	66.16	51.31	38.90	51.50
TC0X00001138.mm.1		2746.35	2983.63	3333.39	2334.42	1702.73	1927.16
TC0300002830.mm.1		1171.35	1902.07	1250.50	571.20	667.59	822.44
TC1900001040.mm.1		52.38	58.61	51.28	30.59	41.69	37.80
TC1700002389.mm.1		32.13	32.78	33.24	26.69	20.02	19.20
TC1400000026.mm.1	Gm3030	35.31	34.46	30.26	25.64	28.18	24.92
TC0700001648.mm.1		30.75	34.44	38.05	26.82	23.59	26.56
TC0600001732.mm.1		220.00	244.25	242.30	182.72	141.43	178.17
TC1200002019.mm.1		28.85	26.09	23.63	15.98	14.45	20.14
TC0500003501.mm.1		2116.82	2484.29	2421.93	1891.32	1463.15	1577.44
TC0600002028.mm.1		79.48	92.87	80.93	55.03	66.45	48.71
TC1200000157.mm.1		73.45	94.83	91.63	62.77	64.21	59.83
TC0600002202.mm.1		32.49	40.68	31.13	21.00	24.26	25.09
TC0X00001812.mm.1		217.44	259.99	221.11	138.18	77.06	125.88
TC1600000748.mm.1	Gm15693	66.35	63.12	54.03	49.28	44.67	42.88
TC0900002614.mm.1		70.54	72.28	57.81	40.49	36.39	50.12
TC0700004130.mm.1	Gm23700	1020.41	875.62	1061.30	743.59	805.16	742.28
TC1200001169.mm.1	Mir380	19.28	23.18	28.28	12.18	12.99	15.94
TC0900002732.mm.1		50.54	41.84	32.84	24.41	26.47	22.99
TC0300000936.mm.1	Gm24830	40746.44	32689.51	36994.74	25787.86	16190.50	20565.66
TC0300000942.mm.1	Gm26232	40746.44	32689.51	36994.74	25787.86	16190.50	20565.66
TC0300002517.mm.1	Rnu1b6	40746.44	32689.51	36994.74	25787.86	16190.50	20565.66
TSUnmapped00000172.mm.1	Rnu1b6	40746.44	32689.51	36994.74	25787.86	16190.50	20565.66
TC0800002072.mm.1		27.19	30.03	29.42	19.85	24.26	18.83
TC0600001623.mm.1		33.38	36.12	34.15	23.59	29.67	24.55
TC0200005153.mm.1	Gm11460	759.65	764.40	779.08	436.20	578.17	583.30
TC1300001308.mm.1		70.05	45.14	61.42	25.01	27.41	36.71
TC0900003209.mm.1		99.62	170.09	132.67	55.56	70.58	39.65
TC0200000837.mm.1		22.08	23.29	29.02	17.70	18.15	16.64
TC1900001164.mm.1		56.74	62.46	50.02	44.48	43.50	42.26

TC1400000143.mm.1	Gm22350	19.05	22.66	22.42	16.90	16.72	14.67
TC0600003025.mm.1		25.62	27.14	31.34	20.42	21.40	22.68
TC0100002145.mm.1	Gm23792	18.90	19.26	17.10	16.22	14.82	15.04
TC0300001432.mm.1		13.60	14.47	14.89	11.39	12.85	11.44
TC0X00001161.mm.1	Gm16373	929.19	829.91	1034.08	732.20	727.51	695.11
TC0400004102.mm.1	Gm13173; RP23-186F3.1	21.45	25.33	26.65	18.82	19.14	17.48
TC0500003421.mm.1		107.67	130.31	116.06	94.89	80.46	90.00
TC0600001692.mm.1		194.72	234.02	234.45	144.71	170.08	168.61
TC0200003120.mm.1		62.56	84.64	88.74	52.77	39.26	44.70
TC0100001867.mm.1	Gm15867	51.74	54.16	53.76	46.84	38.14	39.75
TC1400001347.mm.1		20.66	21.05	23.97	15.31	17.48	13.28
TC0400001549.mm.1		143.19	167.92	170.53	124.45	105.10	124.31
TC1100003347.mm.1		18.82	21.84	15.83	11.20	11.13	13.66
TC0200003037.mm.1		37.70	43.59	49.99	32.14	31.02	31.60
TC1600000355.mm.1		81.27	104.23	77.29	63.55	51.59	50.82
TC0200000732.mm.1	Gm13462	33.46	30.38	37.23	25.93	27.17	26.61
TC0200000745.mm.1		33.37	35.74	40.38	20.33	27.82	19.26
TC1200001154.mm.1	Gm23347	40.13	56.55	55.48	30.55	32.22	33.62
TC0X00003119.mm.1		48713.72	58837.10	53707.97	37988.45	28596.29	38601.37
TC1900000988.mm.1		16.64	20.07	22.21	12.58	14.05	14.21
TC1800000715.mm.1		75.48	68.84	78.91	44.11	59.24	52.25
TC0100001430.mm.1		24.18	26.05	26.30	19.31	20.83	22.53
TC1600000866.mm.1	Mir99ahg	128.71	157.25	126.43	85.34	80.57	105.68
TC0300000015.mm.1	Gm17308	41.35	30.83	37.52	26.91	23.00	24.85
TC0600001581.mm.1		108.66	121.42	136.26	82.58	75.28	95.52
TC0600001719.mm.1	Gm15499; RP23-373I18.2	26.04	30.99	30.07	21.90	24.10	20.98
TC0X00000773.mm.1		186.94	205.78	205.19	125.33	96.81	148.08
TC1200001107.mm.1		131.03	106.51	106.50	72.61	69.97	89.22
TC1300000065.mm.1	Gm26861	28.30	31.95	25.39	21.45	19.80	22.45
TC0100000341.mm.1		29.28	36.59	33.56	20.26	13.35	21.02
TC1700001325.mm.1		658.71	666.78	590.12	455.44	440.73	542.09
TC0400000122.mm.1	Gm11824	140.36	118.91	109.74	95.78	87.88	89.17
TC1200002259.mm.1		28.99	26.45	25.80	18.09	20.74	22.57
TC1300001000.mm.1		24.65	32.65	23.88	18.13	14.16	17.50
TC0900000890.mm.1	Gm22962	153.99	222.90	121.11	40.56	53.96	82.36
TC0300002163.mm.1		8078.17	8582.43	7901.98	5728.32	4629.07	6463.39
TC0200001687.mm.1		219.49	238.51	184.53	157.75	156.96	161.90
TC0800002977.mm.1	4933408N05Rik	87.14	78.97	74.37	62.08	58.71	67.97
TC1300001915.mm.1		54.11	50.63	61.83	34.12	33.30	43.88
TC0900002804.mm.1		73.72	96.33	82.01	54.04	64.29	57.39
TC0800002444.mm.1		32.58	35.47	31.72	22.06	25.55	27.64
TC0200000792.mm.1	Gm13510	46.84	63.50	59.78	39.92	32.67	37.99
TC1600001474.mm.1		266.79	332.90	242.32	180.83	181.10	203.32
TC0X00002499.mm.1		198.87	279.67	214.97	159.59	147.16	149.85
TC0600002130.mm.1		59.51	77.73	66.09	38.11	42.70	50.69
TC0600002826.mm.1		31.78	30.55	26.02	21.29	23.17	23.37
TC0X00003224.mm.1	Gm15146; RP23-228N14.2	106.56	132.23	99.50	71.27	66.00	83.61
TC1300000738.mm.1		33.46	38.31	39.78	27.10	25.88	19.59
TC0100000669.mm.1		635.80	678.84	647.53	505.17	408.70	528.98
TC1100002713.mm.1	Olfr1372-ps1	49.32	49.03	42.37	39.31	34.66	33.10
TC0900000552.mm.1		63.39	65.01	60.55	49.71	35.15	43.64
TC0700003444.mm.1		107.77	191.24	127.91	70.76	64.63	76.11
TC1100004154.mm.1		80.66	90.51	65.63	47.82	43.25	56.66
TC1400001511.mm.1		61.65	75.62	57.12	34.22	46.18	41.79
TC0700000454.mm.1		41.98	47.32	40.10	35.50	32.50	29.19
TC1100004210.mm.1	Gm11788; RP23-82I5.15	57.42	70.81	68.84	50.54	46.97	51.77
TC1700002421.mm.1		199.52	194.31	221.78	138.59	164.24	119.41
TC0200004347.mm.1		86.99	76.14	67.13	42.81	56.58	51.54
TC1600000869.mm.1		35.92	64.13	41.62	22.51	23.48	24.37
TC1900000933.mm.1		206.22	210.86	176.48	145.92	145.39	164.40
TC1200000166.mm.1		65.65	75.55	62.45	43.43	42.01	27.30
TC1200001323.mm.1		118.52	125.58	104.57	88.46	79.63	94.80
TC0500002204.mm.1		16.32	13.11	12.96	9.83	9.03	10.88
TC0300001433.mm.1		49.18	90.63	62.02	25.51	28.56	36.21
TC0300001101.mm.1		27678.81	29167.56	28687.87	20759.53	11910.02	13031.95
TC0200002160.mm.1	Gm14093; 2.145048962-146444327.1	15.32	17.37	17.49	14.28	12.59	13.59
TC1700000160.mm.1		27.37	32.40	30.12	23.71	20.85	24.50
TC1900001242.mm.1		325.13	367.60	404.92	210.25	139.93	235.66
TC0300000486.mm.1		2529.15	4493.87	3019.44	1161.72	1165.26	1853.36
TC0300002788.mm.1	Gm19420	97.48	101.14	134.83	70.10	78.55	67.36
TC1600000004.mm.1		18.07	19.87	20.08	16.04	13.74	12.14
TC0600000610.mm.1		67.37	70.37	72.10	58.66	44.30	44.14
TC0800002246.mm.1		164398.30	184854.00	185130.60	147146.00	114029.20	130136.10
TC1800001003.mm.1	Gm22251	87.54	71.82	86.51	62.05	65.34	63.61
TC0500002844.mm.1		1539.05	1668.22	1437.92	1015.83	543.31	800.13
TC0400002261.mm.1	Gm11851; RP23-323E6.1	576.51	508.16	471.40	353.33	244.83	349.67
TC0800002069.mm.1		24.57	28.98	22.21	14.81	17.09	18.95
TC0900000894.mm.1		29.70	32.59	31.85	25.37	19.06	17.72
TC0600000382.mm.1		988.92	1232.38	1229.35	738.20	543.75	793.19

TC0100000306.mm.1		15.28	14.77	15.52	10.24	13.04	11.69
TC1200002210.mm.1		99.99	96.56	103.44	80.06	55.79	67.27
TC1300001663.mm.1		50.35	50.96	66.56	41.39	35.06	36.04
TC0400000359.mm.1		170.97	163.40	137.23	98.28	116.75	119.43
TC0600003032.mm.1		138.48	154.82	151.82	114.17	76.73	95.70
TC0400000597.mm.1	Tpt1-ps2	41.11	36.58	38.11	32.84	26.19	26.31
TC0100001287.mm.1		109.46	140.05	132.59	96.54	73.62	70.16
TC1500001492.mm.1		256.54	418.15	233.18	109.32	116.07	163.66
TC0600002061.mm.1		162.57	266.86	184.16	85.65	118.28	107.14
TC1800001372.mm.1		57.07	65.70	75.34	30.69	44.86	41.69
TC0200003100.mm.1		369.59	409.64	367.24	311.71	233.76	226.26
TC1800001645.mm.1		38.53	32.44	35.78	28.04	23.66	20.21
TC0600001779.mm.1	Gm23551	17716.30	19552.21	21896.52	13913.53	8760.33	11704.51
TC0200001723.mm.1	Gm25189	62188.91	55610.11	68798.91	48117.23	34187.86	38358.88
TC1600001830.mm.1		42.70	29.51	31.25	23.36	17.48	19.27
TC1700000466.mm.1	Rps2-ps9	440.19	393.97	477.74	354.61	278.72	279.13
TC0100003628.mm.1		23.58	29.40	27.81	20.13	17.89	21.13
TC0X00002410.mm.1	Gm14734; RP23-272G10.3	198.68	207.60	195.55	144.71	95.27	134.82
TC0400002740.mm.1		17.90	20.41	26.55	13.68	14.14	11.79
TC1900000384.mm.1		493.26	538.15	355.34	301.78	248.20	263.83
TC1100001337.mm.1	Rnu1a1	117525.90	117132.80	118980.90	95835.28	67842.24	81857.31
TC1100001338.mm.1	Gm22068	117525.90	117132.80	118980.90	95835.28	67842.24	81857.31
TC1200000451.mm.1	Gm22634	117525.90	117132.80	118980.90	95835.28	67842.24	81857.31
TC1200000452.mm.1	Gm23804	117525.90	117132.80	118980.90	95835.28	67842.24	81857.31
TC1200000453.mm.1	Gm26444	117525.90	117132.80	118980.90	95835.28	67842.24	81857.31
TC1200001751.mm.1	Gm25679	117525.90	117132.80	118980.90	95835.28	67842.24	81857.31
TC1200001754.mm.1	Gm22317	117525.90	117132.80	118980.90	95835.28	67842.24	81857.31
TC1000002009.mm.1		4455.25	4721.09	4329.59	2996.41	1917.92	2964.93
TC0600003004.mm.1		147.16	161.13	125.92	89.80	92.30	113.39
TC0900001007.mm.1		48.49	55.68	48.58	27.33	33.85	39.23
TC1500001290.mm.1		44.65	43.53	31.69	19.43	27.26	18.66
TC0800002198.mm.1		21.20	18.65	22.33	17.17	14.97	16.25
TC0500000330.mm.1	Gm24436	29.86	30.48	28.54	25.88	21.96	20.54
TC1300001754.mm.1		28.80	21.81	23.37	17.94	18.07	17.71
TC0200002388.mm.1		103.25	96.81	105.09	88.56	72.51	71.24
TC0100000339.mm.1		25.72	25.81	21.60	16.10	19.88	17.00
TC1500000101.mm.1		128.92	146.52	136.17	97.34	97.76	118.17
TC1200000332.mm.1		17.99	19.12	14.85	12.09	12.23	13.51
TC0900001095.mm.1	Rps27a-ps2	2844.14	2889.48	3728.38	2244.94	2017.00	2299.88
TC0X00001212.mm.1		56.03	53.95	72.01	42.25	41.20	44.25
TC1400001265.mm.1		14.86	18.80	17.08	11.87	12.80	13.37
TC1700001168.mm.1	Gm6276	108.40	87.40	102.05	80.22	67.68	72.05
TC0100002662.mm.1		91.00	128.34	113.46	71.40	49.15	67.71
TC1400002402.mm.1		559.34	611.56	505.35	402.39	277.84	376.74
TC0500002105.mm.1		144.15	160.29	119.98	106.58	98.66	92.48
TC1400000379.mm.1		405.59	490.14	453.69	332.37	273.58	350.65
TC0700003445.mm.1		338.13	512.13	338.34	151.52	161.08	245.10
TC0800001986.mm.1		37.70	36.70	25.55	16.41	20.18	20.90
TC0X00003068.mm.1		418.04	567.85	447.13	297.10	242.52	335.76
TC0300000947.mm.1	Gm22614	38136.01	32218.28	36324.18	27195.64	18021.70	21616.78
TC0300002518.mm.1	Gm25890	38136.01	32218.28	36324.18	27195.64	18021.70	21616.78
TC1100001751.mm.1		49.86	52.55	49.62	36.53	44.91	37.88
TC0200000637.mm.1		61.89	81.45	58.44	41.58	40.12	48.19
TC1100004257.mm.1	Gm12590; RP23-342H16.5	470.20	491.80	502.33	301.05	226.73	363.83
TC0900002596.mm.1		108.10	124.16	98.90	78.10	83.50	88.32
TC0100002298.mm.1	Gm23240	61.47	61.03	57.46	49.17	43.24	52.24
TC0500002516.mm.1		80.86	92.99	99.27	73.85	59.24	63.87
TC0200004094.mm.1		63.80	87.21	73.45	47.91	52.79	54.40
TC0100001700.mm.1		36.12	42.46	46.58	30.62	29.62	32.62
TC0200003475.mm.1		89.81	112.45	91.41	51.12	60.67	73.28
TC0800001351.mm.1		44.17	55.38	48.63	39.23	37.00	33.24
TC1600002099.mm.1		168.66	208.43	194.13	151.07	113.86	113.94
TC1900000326.mm.1	Gm23238	58310.60	58252.50	55270.42	46519.52	31653.74	35871.50
TC0900002500.mm.1		42.57	52.80	47.47	35.77	36.09	29.00
TC1100003843.mm.1		29.38	31.64	32.01	23.06	27.30	25.47
TC0800001824.mm.1		116.62	139.93	96.07	71.97	74.05	82.06
TC0800001846.mm.1		15621.73	16591.04	17291.46	13173.25	9803.67	12296.54
TC1200000597.mm.1		20.67	17.70	19.23	13.19	13.57	16.29
TC1200000666.mm.1		23.81	25.62	24.26	18.83	15.96	20.68
TC1100003670.mm.1		18.92	20.68	16.67	14.29	15.19	13.15
TC0800001272.mm.1		1182.41	1193.00	929.71	599.82	579.01	823.93
TC0900001274.mm.1		35.12	36.60	37.50	19.09	22.98	28.92
TC1100001870.mm.1		14.14	10.80	12.81	8.51	9.55	9.35
TC0700004580.mm.1	Kcnq1ot1	40.00	43.49	38.88	29.57	31.26	35.72
TC0X00000475.mm.1	Gm14584	1402.97	1277.98	1154.30	896.07	808.14	1039.39
TC0200002984.mm.1		80.08	69.11	58.43	34.56	41.37	49.58
TC0400000691.mm.1	Gm12910; RP23-64F17.5	21.82	22.61	20.61	17.19	18.62	15.01
TC0X00001888.mm.1	Drr1	57032.05	65387.23	58969.68	39717.91	44017.39	50853.75
TC1900000302.mm.1		39.20	34.46	34.70	14.61	20.76	25.24
TC1200001170.mm.1	Mir1197	28.79	25.79	24.06	21.65	17.19	18.98

TC1600001828.mm.1		26.15	23.97	18.43	14.67	14.39	16.19
TC0600000335.mm.1		37.66	45.41	49.24	33.04	30.90	33.32
TC1100003939.mm.1		62.92	72.95	68.01	46.53	56.98	52.98
TC0600000314.mm.1		62.96	105.07	57.43	35.31	37.03	36.98
TC0300001388.mm.1		46.05	64.02	49.08	34.03	18.02	23.79
TC0800002776.mm.1		1252.10	1411.21	1512.83	926.39	954.12	633.24
TC1300001738.mm.1		23.12	28.13	21.48	17.47	17.93	18.28
TC1600000341.mm.1		36.27	44.26	38.74	30.03	32.59	27.41
TC0900002097.mm.1		59.61	63.60	56.34	36.05	38.86	49.65
TC0800000913.mm.1		29.93	40.54	35.99	26.52	24.83	24.19
TC1200001707.mm.1		33.28	40.27	47.29	22.94	24.26	29.18
TC0400003486.mm.1		132.87	143.40	150.10	75.11	96.27	110.09
TC1600000877.mm.1	Mir99a	24.19	28.98	21.95	10.83	13.59	17.68
TC0X00002200.mm.1		20.23	25.36	22.04	13.45	14.09	17.92
TC0600000116.mm.1		64.10	97.64	84.81	41.77	43.18	55.63
TC0600000139.mm.1	ST7-OT4_4	95.57	101.56	88.26	73.64	65.82	51.42
TC1300000406.mm.1	Gm22126	3690.14	3142.68	3576.98	2565.62	1506.44	1606.45
TC0100003332.mm.1		179.68	240.18	205.14	113.09	131.38	73.64
TC0500001870.mm.1	Gm24630	163.03	238.07	179.65	118.30	131.11	125.79
TC1300000653.mm.1		115.79	136.44	106.75	72.54	68.44	93.28
TC1800000283.mm.1		48.99	46.62	39.78	37.04	33.76	32.09
TC0300000575.mm.1	Mir15b	35.36	53.52	41.05	25.98	25.44	29.22
TC1000002309.mm.1		30.18	39.29	30.13	21.85	21.62	25.24
TC0400001174.mm.1		57.45	74.47	73.16	39.46	52.09	39.49
TC1600000727.mm.1		445.43	643.96	465.55	326.57	310.87	352.42
TC1000000516.mm.1		103.78	113.15	78.60	65.07	61.15	67.71
TC0700001386.mm.1		26.71	20.74	23.50	18.27	17.40	18.27
TC0900001993.mm.1		234.90	294.46	258.43	187.68	128.68	175.44
TC0200005361.mm.1		1031.39	1504.16	1203.31	673.03	469.38	783.59
TC0800002536.mm.1	Gm4899	518.94	552.37	482.26	396.38	264.40	343.25
TC0600002011.mm.1		144.15	178.60	121.58	91.84	89.14	103.96
TC0200004723.mm.1	Gm14071	17.72	18.43	15.44	12.97	14.18	11.53
TC0X00003169.mm.1	Gm24907	25.73	25.39	22.13	15.67	20.38	16.50
TC1600002046.mm.1		40.27	42.21	36.82	34.00	27.62	30.64
TC1000000667.mm.1		15.00	16.46	14.75	10.75	13.18	10.14
TC1200000691.mm.1		25.98	25.09	20.93	16.73	18.11	19.30
TC1200001317.mm.1		186.86	207.37	171.49	126.77	73.59	111.29
TC1500000428.mm.1	Gm5045	432.08	420.33	465.54	386.34	313.39	326.72
TC1900001041.mm.1		25.67	31.06	29.30	23.72	20.49	22.52
TC1800000402.mm.1		30.49	29.10	31.23	26.48	20.77	21.02
TC1200002168.mm.1	LOC100862089	33.71	37.88	26.84	23.96	21.00	20.54
TC1300002636.mm.1		503.60	674.26	487.05	316.52	285.59	398.18
TC1300001221.mm.1		59.27	48.92	43.55	35.52	34.27	37.82
TC0500000506.mm.1	5730480H06Rik; Pacrgl	26.07	34.54	27.31	18.65	21.72	21.09
TC0X00002690.mm.1		41.26	60.78	53.14	29.43	34.17	34.77
TC0400000471.mm.1		220.39	303.51	244.80	135.74	139.56	189.29
TC0X00000738.mm.1	Fam220-ps	30.20	26.24	30.66	24.00	22.29	24.56
TC0900000249.mm.1		16.12	19.21	18.77	12.96	14.74	14.60
TC1500001633.mm.1		32.57	27.33	29.23	22.77	23.09	25.50
TC0300003055.mm.1		26.92	32.78	27.99	23.88	20.99	18.97
TC1700001430.mm.1		130.68	152.84	113.02	89.60	75.17	97.63
TC1400002270.mm.1		139.13	177.92	104.16	74.05	56.57	81.90
TC0300001968.mm.1		32.06	31.33	24.94	18.80	22.83	20.70
TC1600000801.mm.1	Gm22448	64.24	56.14	75.63	43.49	40.84	50.21
TC0900003212.mm.1		50.18	61.56	69.29	38.90	45.11	40.58
TC0500000561.mm.1		31.50	32.49	26.20	20.86	24.61	20.78
TC0200001414.mm.1	Gm13814; RP23-165L23.2	25.90	22.50	23.80	21.16	18.16	18.48
TC1300001998.mm.1		3138.75	3129.90	2918.91	2591.39	1975.64	1866.10
TC0900000041.mm.1		155.13	166.57	141.10	94.11	125.92	89.37
TC0X00000769.mm.1	Gm5937	6747.55	6730.90	6361.44	6004.51	5065.66	5092.48
TC1000001871.mm.1		40.52	45.76	48.88	30.94	37.91	31.66
TC0700001383.mm.1	Map6	29.88	36.67	41.36	18.83	23.96	25.23
TC1600002127.mm.1		96.46	68.84	70.30	53.09	41.48	51.54
TC1700002401.mm.1		27.55	33.70	26.52	20.68	18.09	22.83
TC0100001235.mm.1		51.73	54.25	63.67	46.93	40.30	40.99
TC0400002124.mm.1	Gm11805	85.43	87.93	106.28	56.61	72.12	68.05
TC0200000275.mm.1		109.74	108.95	116.69	68.03	77.18	94.50
TC1700001809.mm.1	Gm10503	24.69	17.85	20.01	15.34	12.30	13.98
TC0500001154.mm.1		91.50	111.61	79.85	50.79	49.75	69.58
TC0100000841.mm.1	Gm15374	70.47	76.47	72.12	47.94	50.28	63.24
TC1000002854.mm.1		21.63	21.72	17.64	15.69	14.70	16.31
TC1600001120.mm.1		164.30	194.03	143.04	97.85	74.13	117.17
TC0100003704.mm.1		38.26	47.43	42.67	34.89	28.27	26.65
TC1600000354.mm.1		81.06	87.42	75.25	39.65	53.48	61.02
TC1300000435.mm.1		93.76	78.11	78.64	69.16	56.82	61.49
TC0X00003305.mm.1		108.01	86.15	104.55	70.31	46.48	64.86
TC1900001581.mm.1	Gm6807	12931.49	13162.47	11464.84	10097.67	7654.75	9419.52
TC0500001156.mm.1		23.03	22.25	21.63	14.55	16.61	19.24
TC1400001264.mm.1		11.91	14.76	13.23	9.95	11.07	10.09
TC0X00003225.mm.1	Gm15145; RP23-228N14.5	870.79	798.08	749.69	543.33	479.02	665.08

TC0500001810.mm.1		692.67	1170.03	855.26	367.18	531.34	489.17
TC1300001174.mm.1		48.11	78.59	56.04	31.55	33.40	38.68
TC0600002053.mm.1	Gm13862	10312.91	9348.32	10680.60	8919.53	7627.56	7703.49
TC1600001953.mm.1		44786.76	51938.90	45069.85	33035.25	21562.20	32009.18
TC1000001723.mm.1		30.32	26.46	28.93	25.19	21.90	21.48
TC1500001974.mm.1		111.44	124.00	107.76	97.59	87.47	78.21
TC1100000524.mm.1	Gm23813	5947.91	7017.51	6461.95	5493.84	4401.86	4329.44
TC1600000419.mm.1		34.44	40.78	39.91	30.86	32.01	32.08
TC0X00000991.mm.1	Gm16471	162.37	177.62	178.72	156.42	136.66	141.09
TC1000003033.mm.1	Gm15961; RP23-455J6.4	133.16	201.78	143.62	89.03	96.16	106.57
TC0700003812.mm.1		29.56	28.74	27.81	19.53	25.24	21.47
TC0700003052.mm.1		40.66	38.69	42.53	35.93	31.27	28.89
TC0600002488.mm.1		56.65	51.83	58.16	43.79	37.18	46.89
TC0X00002311.mm.1	Mir505	207.60	371.17	319.33	128.23	140.18	175.00
TC0400003153.mm.1	Gm12785	61.22	78.99	63.45	47.14	45.68	53.67
TC0100001977.mm.1		90.28	114.44	91.40	65.81	70.65	78.12
TC0900000094.mm.1		28.71	33.07	35.74	22.47	23.80	26.83
TC1600000728.mm.1		75.21	76.47	54.81	42.40	37.71	49.26
TC0700003530.mm.1	AU020206	929.65	1061.64	792.19	558.25	526.07	714.52
TC1500001636.mm.1		162.70	212.26	161.18	132.90	123.84	129.04
TC1800000536.mm.1		38.27	36.04	32.59	28.75	19.96	23.57
TC0900001263.mm.1		60.01	57.75	77.37	37.46	33.72	48.52
TC1300000077.mm.1		39.04	30.92	28.57	20.31	24.94	19.84
TC0200003041.mm.1		94.26	126.74	86.11	55.03	49.62	71.57
TC0100002896.mm.1		22.69	30.30	24.31	15.69	19.32	18.17
TC1600000743.mm.1		168.40	148.12	208.31	114.76	122.12	126.55
TC1600001227.mm.1		19.34	23.70	20.65	17.84	16.12	15.80
TC1200000463.mm.1		41.96	52.51	41.81	29.88	21.30	31.85
TC0X00000419.mm.1	Gm14654	798.11	796.04	765.76	556.80	394.61	592.36
TC0200003399.mm.1	Snord90	175.25	191.96	144.90	120.74	89.85	119.63
TC0200002726.mm.1	Gm16358	49.06	48.18	41.28	36.82	39.22	35.43
TC1700000560.mm.1	A530088E08Rik	130.85	97.75	137.63	86.84	71.08	80.82
TC1300002265.mm.1	A130040M12Rik	758.45	867.89	859.68	691.26	548.22	643.91
TC0200005329.mm.1	Gm14392	200.14	204.34	218.36	153.51	107.11	153.00
TC1100000212.mm.1		96.96	96.93	78.96	68.47	70.82	73.26
TC1100002338.mm.1		46.24	50.94	39.96	37.16	32.87	33.68
TC0200004274.mm.1		29.99	24.12	27.13	18.26	17.35	22.27
TC1200000952.mm.1		15.44	19.80	20.74	13.76	11.42	9.60
TC0100003390.mm.1		65.31	68.36	76.45	48.65	30.20	45.80
TC0100002380.mm.1		39.07	46.97	39.11	34.95	29.80	30.46
TC0X00000123.mm.1		98.27	131.07	80.43	67.25	50.98	55.84
TC0200003124.mm.1	Gm13369	165.72	184.85	133.58	109.93	87.50	114.59
TC1800000633.mm.1		120.28	170.29	142.71	81.42	78.46	107.19
TC1700001161.mm.1		62.45	60.73	51.60	43.72	32.10	42.48
TC1100001181.mm.1		21.25	19.42	21.42	18.69	16.35	16.10
TC0300000019.mm.1		46.56	73.15	48.05	31.79	27.27	35.09
TC0400000640.mm.1		58.88	66.83	59.41	50.32	35.55	42.75
TC0400002451.mm.1	Gm24112	2689.67	3804.60	2447.11	1170.55	1125.85	1943.15
TC1700002392.mm.1	Gm17228	95.91	82.02	81.80	65.54	42.24	55.73
TC0400003455.mm.1	Gm12881	149.14	97.15	113.57	77.61	62.73	77.17
TC0900000962.mm.1		105.71	137.67	140.78	92.09	62.71	79.80
TC0200004527.mm.1		84.99	131.46	95.09	55.25	66.88	65.37
TC0800000657.mm.1		561.70	659.76	514.41	419.26	289.98	392.04
TC0X00000109.mm.1		53.64	57.14	46.66	34.91	43.67	34.36
TC1300000055.mm.1		43.47	37.90	31.91	28.33	27.47	26.22
TC0X00000301.mm.1		37.53	51.66	40.75	25.25	23.47	32.52
TC0200003341.mm.1		53.71	61.00	66.63	47.35	43.23	49.64
TC0500000388.mm.1	Mir3097	266.06	346.26	344.18	209.48	233.67	240.92
TC1800001641.mm.1		57.96	89.55	73.19	38.94	41.58	50.91
TC1700002326.mm.1		178.47	160.06	168.35	116.58	84.60	128.62
TC1800000252.mm.1	Gm16345	519.72	413.32	471.09	369.93	296.77	352.18
TC0900002597.mm.1	Gm23730	645.93	558.94	605.95	441.18	370.95	500.12
TC0600003320.mm.1	LOC100861780	677.68	674.29	611.36	484.18	254.79	324.25
TC0400001046.mm.1		465.45	552.80	463.81	358.43	293.73	393.14
TC0400002368.mm.1		465.45	552.80	463.81	358.43	293.73	393.14
TC1800000079.mm.1		28.56	25.63	20.18	15.64	17.54	17.60
TC0400004038.mm.1		130.09	105.28	83.14	67.52	61.11	69.57
TC0300002004.mm.1		21.64	21.70	23.01	20.06	16.69	16.63
TC0700001414.mm.1		61.08	72.50	74.34	57.63	49.36	53.17
TC1600000441.mm.1	Gm20056	849.46	886.21	809.91	767.55	642.52	648.61
TC0100000438.mm.1	Gm20257	14.08	13.90	12.04	11.17	9.77	11.04
TC1600000259.mm.1	Gm23612	20.88	30.85	21.91	16.15	15.86	15.74
TC1900000968.mm.1		79.64	59.06	67.97	52.45	50.58	48.81
TC1800000246.mm.1		64.87	112.95	97.02	55.83	43.62	45.99
TC0200004247.mm.1		58.34	63.61	75.27	48.32	47.49	52.90
TC1500001579.mm.1		76.23	78.38	77.24	55.66	47.35	65.96
TC1000000029.mm.1	Gm22739	273.25	217.17	236.95	181.10	133.90	176.14
TC0800000492.mm.1		147.16	182.53	150.89	97.11	77.65	121.66
TC0200002079.mm.1		18.26	23.76	22.44	13.56	15.53	16.68
TC0900002527.mm.1		52.85	63.33	57.52	49.93	44.16	42.84

TC1800000947.mm.1		69.45	71.40	65.48	43.41	52.13	58.17
TC0200002740.mm.1	Gm14301	25.74	31.09	34.24	21.37	23.20	22.80
TC0800002366.mm.1		791.90	882.45	690.04	534.95	382.34	564.46
TC0X00002287.mm.1	Gm14650	1230.17	1229.34	1397.34	1050.99	727.47	867.28
TC1000000270.mm.1		1892.35	1780.48	1993.10	1637.30	1256.47	1249.85
TC0X00001290.mm.1		42.46	44.28	37.89	28.09	27.59	35.55
TC0900002456.mm.1		18.09	19.19	19.86	16.06	12.37	15.01
TC0300001793.mm.1		600.51	702.71	632.72	474.90	438.25	554.34
TC1600000619.mm.1		32.87	37.63	28.26	20.35	25.54	23.14
TC0X00002506.mm.1	Gm14742; RP23-91C19.8	23.23	30.34	25.91	19.44	21.07	19.46
TC0X00002208.mm.1		77.68	89.23	92.12	57.91	35.88	57.33
TC1100000275.mm.1	Gm12080	27.52	28.25	35.16	21.67	17.72	23.12
TC1100001749.mm.1		23.09	26.63	35.79	18.66	18.05	14.93
TC0500001313.mm.1		116.33	163.63	135.51	100.84	74.48	90.37
TC0200000125.mm.1	Mir467a-3	139.53	161.77	151.32	91.59	110.81	124.40
TC0200000143.mm.1	Mir467a-6	139.53	161.77	151.32	91.59	110.81	124.40
TC0200000173.mm.1	Mir467a-10	139.53	161.77	151.32	91.59	110.81	124.40
TC0400003658.mm.1	Gm13215	846.45	861.16	978.97	757.37	557.97	584.65
TC0600001261.mm.1	Gm26982; RP23-392K24.4	23.58	21.89	20.70	19.58	16.22	16.47
TC0200000788.mm.1		66.65	81.79	75.86	58.28	45.27	57.08
TC1400001316.mm.1		51.21	53.64	35.46	17.99	29.51	25.47
TC1600000200.mm.1		80.60	68.16	80.76	60.06	47.03	59.23
TC0200003613.mm.1		56.72	44.14	44.43	21.33	26.72	35.60
TC1300002727.mm.1		36.99	48.09	46.70	29.92	26.96	34.44
TC0600000844.mm.1		111.90	101.38	112.10	73.58	91.46	64.45
TC0500000563.mm.1		36.39	46.74	41.61	33.81	30.03	27.94
TC1200001149.mm.1	AF357426	270.02	271.19	335.46	209.12	228.40	173.12
TC1200001711.mm.1		33.11	39.35	38.31	20.81	19.34	29.69
TC1400001313.mm.1		32.84	49.39	40.48	24.44	27.54	18.58
TC0400000950.mm.1	Mir872	58.74	96.66	52.04	36.20	34.90	35.01
TC0200000624.mm.1	Gm13419	246.56	274.13	299.29	233.25	210.27	199.00
TC0500002208.mm.1		132.38	115.27	98.17	87.23	86.57	79.00
TC1700000867.mm.1	Gm25008	1412.93	1233.66	1452.41	1152.85	1001.90	1126.49
TC1100003123.mm.1	Gm12321	500.64	397.07	390.34	343.90	297.98	295.58
TC1600000420.mm.1		123.68	165.82	142.55	81.89	93.03	111.68
TC0800001978.mm.1	Gm8051	23.17	24.58	25.47	21.10	15.42	16.05
TC0300000599.mm.1	Gm24544	38.52	33.92	33.15	30.73	25.92	25.41
TC0400003907.mm.1	Gm13018; RP23-416J8.1	205.68	198.92	231.38	164.28	145.00	109.06
TC0900003214.mm.1		24.18	26.39	31.24	21.51	14.77	16.21
TC1800000225.mm.1		2573.70	2384.05	2655.49	1904.85	1101.59	1536.26
TC1700002748.mm.1	Gm15405; RP23-75D10.1	83.25	79.07	79.39	53.61	71.22	57.10
TC0400001702.mm.1	Gm25772	13050.07	10960.26	13059.85	10507.26	8710.37	9002.13
TC1500001913.mm.1		67.97	80.96	74.50	50.96	63.95	51.50
TC0X00001614.mm.1		63.99	83.06	63.83	46.86	36.31	51.47
TC0700003494.mm.1		35.75	39.02	38.16	27.78	33.91	27.79
TC0500000929.mm.1		264.43	340.94	279.66	203.19	177.58	233.40
TC0700001809.mm.1	Gm20367	231.84	202.60	222.73	186.67	145.25	168.88
TC1000001778.mm.1		37.59	41.72	30.24	22.67	21.20	28.02
TC0700001092.mm.1		32.59	25.59	33.45	22.43	21.15	23.73
TC0600002753.mm.1		41.99	33.27	35.26	30.32	27.40	27.58
TC0600002179.mm.1		43.57	38.50	44.53	29.91	31.95	36.79
TC0800001004.mm.1		35.38	43.99	43.12	26.85	31.00	32.71
TC0500000460.mm.1		173.96	277.08	259.81	104.00	121.31	155.68
TC1900000453.mm.1		689.18	846.10	664.36	459.82	535.63	578.51
TC0900000555.mm.1		103.42	85.49	83.96	74.57	56.19	61.79
TC0X00002552.mm.1		422.70	532.69	422.54	318.53	267.14	358.47
TC0200000687.mm.1		42.91	40.89	33.96	31.57	30.26	30.49
TC1700000212.mm.1		76.58	105.72	84.74	68.24	59.39	59.76
TC1400001254.mm.1	Gm19845	29.31	26.32	33.40	16.76	19.39	23.74
TC0800001368.mm.1		37.61	35.78	37.59	26.13	28.13	33.29
TC1000000429.mm.1	Gm27731	299.69	354.21	240.37	191.53	122.60	182.75
TC0600002897.mm.1	n-R5s165	57.82	45.07	62.72	38.22	36.00	41.31
TC0400000635.mm.1		21.58	22.20	20.85	19.08	14.52	16.13
TC0400003062.mm.1	Gm12651; RP23-3E2.2	447.40	380.48	377.20	294.35	222.42	309.73
TC0300000488.mm.1		93.87	118.98	97.38	55.90	76.37	74.70
TC1200001232.mm.1		38.71	31.97	41.76	30.17	26.21	26.72
TC0300000073.mm.1		291.92	241.85	164.48	130.29	136.53	115.30
TC0500000574.mm.1		291.92	241.85	164.48	130.29	136.53	115.30
TC0800001607.mm.1		291.92	241.85	164.48	130.29	136.53	115.30
TC1200000534.mm.1		291.92	241.85	164.48	130.29	136.53	115.30
TC1500000226.mm.1		29.25	30.86	30.78	25.91	19.93	24.29
TC1700001298.mm.1		18.93	20.36	19.61	17.84	14.70	14.57
TC1300001227.mm.1		19.95	16.00	16.90	12.83	14.77	12.84
TC1200001680.mm.1		364.63	346.12	296.78	247.65	201.72	267.79
TC1200000360.mm.1		111.48	136.75	118.57	71.61	75.14	100.70
TC0700000947.mm.1		19.90	19.83	19.37	15.35	17.45	13.39
TC0400001476.mm.1		186.42	203.59	186.35	156.58	100.17	122.85
TC1600000884.mm.1		74.13	62.10	64.29	42.42	27.26	46.53
TC0200000590.mm.1	Gm26236	1359.73	1442.22	1443.17	1138.75	870.85	1147.80
TC0400000892.mm.1	Gm13284	114.45	95.15	72.79	58.81	45.87	61.40

TC0400000482.mm.1		16.55	14.11	15.94	11.04	13.20	12.60
TC0700001095.mm.1		155.24	174.40	200.14	135.19	132.65	142.65
TC0500003701.mm.1		24.38	29.53	25.40	18.66	15.01	21.10
TC1100002483.mm.1	Rpsa-ps4	347.00	298.10	266.12	241.28	228.75	231.01
TC0X00003109.mm.1		22.99	26.89	32.52	19.44	17.95	20.31
TC0100001797.mm.1		38.36	32.74	37.08	29.75	21.15	25.32
TC0400002910.mm.1	Gm11261; RP23-189G9.1	295.23	296.95	260.33	215.75	182.84	239.99
TC0600002830.mm.1		38.22	46.75	47.43	34.16	31.48	24.86
TC0100003118.mm.1		102.72	129.32	116.37	77.38	45.52	73.91
TC0100002968.mm.1		33.25	34.16	26.90	20.96	25.71	21.70
TC0800002474.mm.1		50.87	69.30	51.29	32.97	42.00	38.89
TC0300002649.mm.1		54.06	71.07	45.06	34.95	36.53	36.91
TC1900000997.mm.1	Gm8034	108935.20	104160.00	135339.80	89304.27	59063.51	71408.67
TC0800002498.mm.1		202.02	207.21	197.48	123.98	92.75	158.41
TC0X00001966.mm.1	Gm27910	3124.83	3418.09	2558.69	1703.96	850.71	1705.34
TC0100003494.mm.1		31.45	26.68	24.79	16.88	22.43	19.17
TC1600000985.mm.1	1110008E08Rik	17.90	15.71	17.53	13.05	14.98	11.98
TC0X00001273.mm.1		41.37	55.18	46.77	34.76	32.69	37.86
TC1700000051.mm.1	n-R5s26	156.69	181.75	143.19	51.04	72.21	111.49
TC1500001493.mm.1		107.21	157.44	96.39	51.46	56.42	79.99
TC0500000741.mm.1		17.52	20.79	20.07	15.80	15.63	16.92
TC0300000603.mm.1		202.80	203.54	217.56	167.66	102.59	129.74
TC1700000849.mm.1	Runx2os1; AC165141.1	237.19	223.27	253.28	193.39	131.66	167.83
TC1200000764.mm.1		409.45	520.29	430.38	316.26	265.21	359.95
TC1300000533.mm.1		409.45	520.29	430.38	316.26	265.21	359.95
TC1500001235.mm.1		409.45	520.29	430.38	316.26	265.21	359.95
TC1800000149.mm.1		409.45	520.29	430.38	316.26	265.21	359.95
TC0900000331.mm.1		118.57	139.73	131.66	112.20	86.49	92.90
TC0600001157.mm.1		179.76	200.25	223.31	90.57	102.77	156.43
TC0500002463.mm.1	Gm23967	181820.70	174313.30	167942.20	161004.10	139285.20	135845.30
TC0200000068.mm.1		1229.84	1382.97	1019.17	898.22	607.08	782.67
TC1100000646.mm.1	LOC100862216	22775.68	22246.63	23004.60	18533.94	12588.89	16481.15
TC0X00001616.mm.1		58.95	46.38	51.95	36.84	38.45	43.68
TC0200002717.mm.1		82.27	66.40	60.83	51.93	43.44	52.05
TC0200003223.mm.1	Gm13372	48.18	35.62	44.32	30.45	23.62	31.20
TC0100000539.mm.1		54.56	80.44	68.34	43.31	28.57	43.33
TC0900002808.mm.1	9330159M07Rik	102.02	99.07	90.38	78.90	78.15	88.35
TC0100002317.mm.1		63.81	96.41	80.85	56.42	49.74	53.22
TC1200000405.mm.1		144.76	151.26	191.31	121.19	78.19	100.71
TC1500000178.mm.1		35.79	42.72	29.29	25.43	23.63	25.44
TC0600002297.mm.1	Gm15572	38.04	35.88	42.65	29.82	34.09	29.43
TC0800001929.mm.1		50.35	44.91	50.67	41.46	31.66	37.47
TC0300000023.mm.1		48.51	62.22	64.62	40.88	45.77	40.68
TC0X00001967.mm.1	Gm27839	2213.80	2296.47	1802.48	1218.80	595.46	1185.25
TC1200001171.mm.1	Mir323	16.04	11.46	12.23	9.99	8.83	8.21
TC0200003306.mm.1	Gm23369	686.23	709.32	813.74	634.14	491.79	506.28
TC0600001597.mm.1		129013.70	151480.30	151473.10	101992.40	120854.20	117197.60
TC0900003211.mm.1		25.34	27.84	28.99	23.45	18.01	21.12
TC1100004004.mm.1		15.62	19.67	17.84	11.64	12.22	14.82
TC1100003449.mm.1		3348.67	3391.20	3029.97	2614.93	2608.48	2965.87
TC1200001082.mm.1		57.31	51.47	58.31	49.97	41.81	42.63
TC1500000038.mm.1		116.96	135.96	105.05	59.22	77.19	90.86
TC0200004041.mm.1	Gm13765; RP23-309M6.3	617.68	774.09	640.82	433.09	298.69	490.47
TC0100002958.mm.1		22.70	17.74	22.38	16.68	15.74	15.54
TC1300002746.mm.1		66.17	56.34	52.90	41.31	41.14	49.31
TC0X00001145.mm.1	Gm14897	1724.83	1977.44	2081.80	1464.66	975.98	1350.89
TC1400001317.mm.1		19.76	16.26	21.11	12.24	15.38	11.26
TC1600000868.mm.1	Gm21816	49.60	70.83	66.23	26.21	41.34	38.68
TC1100000868.mm.1		49.00	46.81	41.47	40.23	34.04	35.09
TC0700001277.mm.1		155.31	246.65	203.45	130.23	119.06	133.71
TC1200001368.mm.1		86.98	109.73	70.72	63.94	44.11	44.77
TC1800000348.mm.1	Vaultrc5	34.61	44.30	36.48	31.29	26.76	24.08
TC1800001093.mm.1	Gm7847	8553.92	7034.70	6433.67	5897.14	4880.31	4615.23
TC0200000252.mm.1	Gm13312	100.94	79.88	93.41	69.66	40.54	52.27
TC0200000030.mm.1		137.27	151.05	130.29	81.78	77.72	116.87
TC1300002394.mm.1		26.88	32.93	30.44	19.06	24.83	22.53
TC1000000177.mm.1	Gm1972	82.73	100.06	97.64	80.28	67.32	67.16
TC1000000102.mm.1		37.33	51.03	30.22	22.41	25.39	22.57
TC0700003713.mm.1		47.24	40.14	51.66	36.72	26.16	31.69
TC0100000218.mm.1		67.59	89.22	75.77	53.86	46.27	61.44
TC0500000568.mm.1		22.62	31.61	38.34	18.54	19.33	16.38
TC1200000722.mm.1		18.60	19.10	17.91	11.94	16.40	12.87
TC1100001421.mm.1		146.10	155.59	137.96	89.65	48.03	92.77
TC1300002159.mm.1		35919.57	36521.63	34603.80	30953.57	23058.69	27727.51
TC1200002245.mm.1		101.01	145.97	116.48	91.49	65.93	71.24
TC1000002087.mm.1	LOC100862325	439.36	294.33	287.79	174.86	122.69	219.22
TC0X00000492.mm.1	Gm6591	14.56	13.49	14.00	12.74	10.22	10.25
TC1600000444.mm.1		78.12	86.31	72.37	66.23	45.52	47.25
TC1700000175.mm.1		191.45	180.97	189.40	126.85	143.36	166.91
TC1100002722.mm.1	Gm11187; RP23-388J21.1	142.20	154.06	150.33	130.21	92.82	101.96

TC0800002943.mm.1		163.98	191.11	168.54	75.06	120.94	120.86
TC0900000088.mm.1		67.37	65.51	83.17	30.94	52.75	43.53
TC1400001885.mm.1		332.43	353.12	423.52	255.90	172.05	261.50
TC1500002060.mm.1		21.90	18.97	23.25	18.26	16.33	15.40
TC0100000178.mm.1		464.88	361.40	322.20	247.65	183.84	267.64
TC1700002364.mm.1		464.88	361.40	322.20	247.65	183.84	267.64
TC1900001325.mm.1		43.91	49.56	40.82	29.59	20.04	32.83
TC1300002398.mm.1	Gm24498	54.04	44.13	40.86	34.99	36.15	34.73
TC1400001261.mm.1		69.96	103.25	73.60	57.37	47.04	54.51
TC0X00001706.mm.1		75.90	90.84	86.23	49.30	60.82	69.00
TC0900001947.mm.1		17.80	19.54	19.81	13.16	16.54	15.47
TC0200000700.mm.1	Gm13588	37312.64	31720.67	47178.24	27945.93	16253.47	18843.47
TC1800000876.mm.1		255.77	288.52	212.14	198.35	168.56	180.21
TC0600000391.mm.1		15.55	18.45	13.64	11.58	11.12	12.51
TC0500001563.mm.1		101.88	85.61	116.48	53.54	73.85	71.47
TC1900001047.mm.1		403.35	602.29	509.45	347.55	290.74	353.11
TC1200001695.mm.1		21.18	22.00	16.94	11.58	14.83	15.09
TC1500002013.mm.1		120.45	106.28	107.53	82.47	48.29	72.32
TC1000002662.mm.1	Gm26180	2061.50	1916.73	2336.42	1763.69	1388.88	1607.18
TC0900001799.mm.1		17.04	19.23	23.07	13.01	15.80	12.29
TC0400002816.mm.1		219.84	268.42	170.84	115.79	87.62	148.95
TC0600001262.mm.1	LOC100504971	367.64	410.74	383.34	232.11	278.76	329.33
TC1200002336.mm.1		16.39	18.30	19.38	15.77	13.83	12.88
TC1200000949.mm.1		831.58	876.31	949.18	783.04	605.77	610.99
TC0X00000498.mm.1	Gm16431	16.00	13.85	14.80	12.75	9.99	11.78
TC1600000669.mm.1	Gm10808	154.94	146.71	157.11	116.32	72.38	106.50
TC0500002787.mm.1		210.70	202.61	187.17	168.30	124.08	153.97
TC1900001061.mm.1		74.74	59.77	51.22	41.89	42.74	31.03
TC1200002258.mm.1		80.92	87.63	69.50	61.71	61.76	48.75
TC0400002393.mm.1		344.98	413.44	324.83	247.15	259.39	300.71
TC0900000900.mm.1		25.97	21.27	21.67	13.97	19.10	15.87
TC0X00000857.mm.1	Gm16412	12633.68	11305.71	13554.09	10785.35	8228.56	8753.51
TC1300001306.mm.1		26.33	29.36	24.52	16.26	19.86	22.12
TC0X00001256.mm.1		214.55	344.05	214.50	137.63	169.17	125.28
TC0200001016.mm.1		35.70	33.23	34.89	31.11	23.63	25.45
TC1500000176.mm.1		74.00	67.11	70.42	44.49	61.82	49.38
TC0300002651.mm.1		73.30	78.06	88.35	59.24	67.42	51.14
TC0X00001437.mm.1	Gm15057	58.03	51.74	40.49	38.33	31.01	31.14
TC0700001267.mm.1	Prss23os; RP24-64C6.1	121.44	109.14	102.31	81.46	64.30	90.43
TC0600001835.mm.1	Gm22155	7414.47	5403.92	5589.91	4540.03	2708.12	2487.65
TC1800000817.mm.1		43.44	39.96	55.38	24.65	19.17	33.90
TC1600001963.mm.1		27.49	21.93	25.61	19.49	19.85	20.84
TC1700002705.mm.1		40.99	35.77	33.01	29.64	23.33	28.32
TC1500000435.mm.1		66.90	85.11	78.73	47.89	60.93	58.68
TC0600003293.mm.1	Gm23375	4712.78	4902.13	4542.46	3311.08	2631.91	3948.30
TC1200000365.mm.1		118.93	137.56	135.91	111.24	80.24	93.23
TC1900000783.mm.1		57.59	80.48	76.46	52.73	51.19	45.15
TC1700000524.mm.1		52.81	48.83	49.09	42.12	27.53	33.91
TC0700001670.mm.1		24.57	21.62	21.88	10.64	15.95	16.83
TC0100003703.mm.1		49.65	74.63	63.33	25.75	28.84	45.18
TC0600000310.mm.1		35.04	28.72	26.55	20.72	21.09	24.50
TC1400001071.mm.1		34.15	47.95	39.81	24.72	31.03	28.23
TC1100002325.mm.1	Mir1933	58.57	72.90	60.09	53.45	42.73	46.27
TC1300002728.mm.1		63.10	59.99	67.64	40.15	32.69	52.42
TC1300001963.mm.1		48.09	79.01	57.63	39.66	36.89	27.91
TC1500000486.mm.1		37.49	46.85	45.15	30.23	36.39	31.54
TC1200001303.mm.1	Gm19349	99.99	131.80	114.31	85.00	63.07	85.40
TC1500000140.mm.1	Gm25101	7331.42	6593.87	7294.05	5940.60	4454.35	5570.40
TC1000001201.mm.1		206.92	296.29	239.65	164.71	150.31	188.62
TC1500001454.mm.1		18.84	20.02	16.95	16.43	13.52	13.42
TC1500002179.mm.1	Snora2b	89.53	95.43	80.83	70.99	63.30	76.94
TC0300001690.mm.1	Gm23686	95427.80	94142.35	105883.30	81319.49	52282.68	65186.72
TC0800001825.mm.1		42115.10	44097.80	40220.99	32271.84	29589.87	37982.59
TC0X00003296.mm.1		36.45	30.39	36.12	16.51	23.16	26.13
TC1700002302.mm.1		73.52	67.20	72.04	49.05	40.56	60.25
TC0100001922.mm.1	4732440D04Rik; AC139064.2	67.62	66.02	58.84	55.65	45.28	52.22
TC1300000999.mm.1		25.45	33.56	22.28	18.38	18.11	18.65
TC0300000315.mm.1		20.35	26.07	21.77	18.79	16.37	17.20
TC1400000129.mm.1		52.64	44.18	52.80	40.97	35.70	41.49
TC0700001142.mm.1		144.86	188.89	171.07	134.81	118.79	131.30
TC0400000054.mm.1		51.37	54.41	52.99	30.57	34.81	45.78
TC0700003840.mm.1	Gm7027	2498.14	1927.62	2330.27	1847.94	1542.31	1656.28
TC1200000221.mm.1		139.58	126.84	117.33	112.60	98.63	93.08
TC0200001060.mm.1	Ak3l2-ps	112.28	149.07	127.31	102.95	98.76	96.00
TC1600001291.mm.1		32.78	33.27	30.05	28.94	23.35	25.41
TC1100002500.mm.1		124.17	149.11	120.85	82.30	85.83	110.75
TC0600000219.mm.1		240.69	304.76	249.93	174.71	220.21	189.87
TC0400000478.mm.1		80.62	108.67	91.65	55.06	61.68	74.91
TC0100000586.mm.1		241.07	285.53	316.15	227.76	184.54	206.77
TC1000001588.mm.1		72.86	78.71	69.95	38.12	58.91	52.06

TC1200001636.mm.1		42.36	36.97	41.22	36.41	32.21	32.05
TC0500000831.mm.1	Eif5al3-ps	8657.98	8596.29	6953.72	6735.86	5228.44	5679.24
TC1500000219.mm.1		51.55	62.00	54.54	42.87	40.43	48.94
TC1700000687.mm.1	Gm8752	60.35	71.84	71.92	46.82	47.11	58.91
TC1000002617.mm.1		19.10	15.38	20.29	14.42	13.87	13.48
TC1200001951.mm.1	Mir5135	91.94	98.50	98.06	71.54	88.53	74.62
TC0200000910.mm.1		55.08	65.19	55.98	42.46	43.51	51.60
TC0500001463.mm.1		19.63	17.34	19.12	17.08	14.96	14.97
TC0800000914.mm.1		62.26	77.72	65.98	50.32	36.28	51.95
TC1400002404.mm.1		19.82	16.71	22.20	14.51	13.84	15.83
TC1500000817.mm.1		44.46	55.14	46.59	38.88	28.00	35.66
TC0500002513.mm.1		20.84	23.72	28.14	14.96	19.26	17.09
TC1300000108.mm.1		27.05	26.61	27.20	21.41	25.19	20.59
TC0400001704.mm.1		149.77	186.29	156.44	136.42	123.54	107.32
TC1700000848.mm.1	Runx2os2; RP24-535G5.2	12.16	14.66	14.32	11.23	11.01	9.08
TC0800002937.mm.1	Gm24103	1751.47	1348.25	1599.08	1304.75	1051.96	1035.44
TC0100000728.mm.1		37.23	36.36	37.39	24.06	25.68	33.22
TC0100000745.mm.1		37.23	36.36	37.39	24.06	25.68	33.22
TC0100000759.mm.1		37.23	36.36	37.39	24.06	25.68	33.22
TC0100002710.mm.1		37.23	36.36	37.39	24.06	25.68	33.22
TC0100002715.mm.1		37.23	36.36	37.39	24.06	25.68	33.22
TC0100002717.mm.1		37.23	36.36	37.39	24.06	25.68	33.22
TC0100002720.mm.1		37.23	36.36	37.39	24.06	25.68	33.22
TC1_GL456210_random00000001.mm.1		37.23	36.36	37.39	24.06	25.68	33.22
TC1_GL456211_random00000007.mm.1		37.23	36.36	37.39	24.06	25.68	33.22
TC1_GL456211_random00000029.mm.1		37.23	36.36	37.39	24.06	25.68	33.22
TC1_GL456212_random00000005.mm.1		37.23	36.36	37.39	24.06	25.68	33.22
TC1_GL456221_random00000011.mm.1		37.23	36.36	37.39	24.06	25.68	33.22
TC1_GL456221_random00000018.mm.1		37.23	36.36	37.39	24.06	25.68	33.22
TC1500000964.mm.1		17.20	16.50	13.68	11.03	11.43	13.34
TC0700004435.mm.1		16.34	12.55	12.96	11.42	9.20	8.84
TC0900002208.mm.1		166.32	163.82	147.98	129.79	117.75	143.02
TC0700000992.mm.1		63.58	69.79	55.00	48.43	48.65	52.59
TC1400000840.mm.1	Gm16261	155.71	130.11	137.71	121.18	99.48	88.91
TC1800001709.mm.1		41.62	43.43	42.94	24.72	35.85	31.94
TC0600001819.mm.1	Gm8652	964.91	873.84	1058.73	825.97	620.27	703.55
TC1200001867.mm.1	Gm23018	124.92	129.45	150.88	108.12	70.01	91.04
TC1900000688.mm.1		88.18	111.99	80.00	61.85	63.17	72.86
TC1300000057.mm.1		23.12	18.15	26.36	16.43	16.14	14.12
TC0400000034.mm.1		84.74	124.33	101.17	63.59	44.53	72.14
TC1500000042.mm.1		79.26	102.04	86.41	74.01	53.97	52.59
TC0900001550.mm.1	Gm17396	39.93	34.84	31.90	30.41	27.15	26.92
TC1800001706.mm.1		38.55	36.71	32.64	21.22	26.51	29.85
TC0400003431.mm.1	Gm12959	82.65	81.76	83.66	75.24	56.52	61.35
TC0X00002484.mm.1		26.67	33.13	22.81	16.47	20.95	18.19
TC0900001687.mm.1		20.37	23.10	23.39	20.01	16.72	17.56
TC0900000297.mm.1		43.35	48.36	41.84	37.45	27.17	33.69
TC0X00001984.mm.1	Gm27341	4952.97	5810.54	4151.88	2853.76	1558.28	3114.95
TC1100003994.mm.1		27.53	18.82	18.56	13.34	13.55	15.42
TC1000001757.mm.1		236.99	356.76	234.34	181.71	167.13	182.24
TC1200000160.mm.1		162.93	170.65	182.85	121.94	89.61	138.74
TC0100003115.mm.1		69.73	71.23	62.69	54.01	37.61	51.64
TC1100002501.mm.1		79.45	80.92	63.06	55.84	60.23	56.77
TC0700001075.mm.1	Gm27459	807.04	907.28	522.39	436.15	236.06	384.68
TC0100003359.mm.1		27.81	34.08	20.62	16.14	18.77	15.95
TC0100003336.mm.1		61.26	96.09	55.16	42.54	38.97	41.51
TC1600000990.mm.1	Gm7831	42.71	36.59	40.23	34.27	24.60	28.77
TC0400002968.mm.1		61.09	50.26	73.28	24.06	23.78	45.41
TC0100000187.mm.1	Gm23771	110.60	117.06	107.87	103.35	87.31	84.18
TC1300001219.mm.1		19.81	23.97	18.92	17.50	13.38	14.68
TC1300000413.mm.1		39.03	35.01	37.49	25.73	25.61	33.45
TC0700000294.mm.1	Gm25134	589.33	508.63	615.63	458.83	421.12	492.90
TC1300000254.mm.1	Gm22452	402.02	579.27	466.51	192.47	352.76	253.80
TC1000000181.mm.1	Gm16531	66.87	82.38	81.62	65.60	52.93	55.56
TC1300002381.mm.1		243.50	307.82	224.72	190.73	137.95	183.51
TC0900000961.mm.1	Gm19353	1622.03	1468.54	1537.52	1350.65	927.97	1046.57
TC0100003330.mm.1		96.54	108.10	92.37	73.90	88.66	73.84
TC0800002446.mm.1		83.10	108.07	99.24	71.71	58.20	76.86
TC0300003114.mm.1		39.57	50.45	43.46	30.52	32.39	37.64
TC0X00002642.mm.1		85.95	93.25	66.07	54.27	56.52	62.79
TC0100003685.mm.1		152.47	202.62	173.37	113.23	134.56	138.69
TC0200004299.mm.1		12.09	12.19	14.51	11.24	9.39	8.97
TC1400002249.mm.1		74639.35	69068.98	74966.00	66442.96	54310.84	51655.19
TC0600001893.mm.1	LOC100862080	292.17	237.50	193.92	168.84	117.48	158.10

TC1000000323.mm.1		41.27	44.98	33.58	31.69	29.04	30.17
TC0300000879.mm.1		20.32	18.40	19.55	14.60	13.64	17.63
TC0X00000877.mm.1		43.47	35.72	33.08	29.69	22.00	26.88
TC0X00001840.mm.1	Gm14473	472.26	449.39	507.14	420.88	346.83	307.77
TC0200004195.mm.1	Gm13805; RP23-392O16.1	234.70	245.68	188.29	163.74	114.38	161.34
TC0100002263.mm.1		186.49	151.74	150.44	119.06	97.52	132.51
TC0700003435.mm.1	Gm23957	52.91	44.11	51.99	41.81	29.45	27.16
TC0800001819.mm.1		278.54	341.20	273.98	219.39	129.89	194.45
TC0200002840.mm.1		84.35	100.47	78.86	73.91	59.89	64.98
TC0200003598.mm.1		84.96	99.66	98.73	65.95	81.14	57.72
TC1300001498.mm.1		16.60	22.61	21.51	14.41	13.41	15.94
TC1400002129.mm.1	C030013D06Rik; RP23-121M22.4	105.42	135.24	97.23	65.12	48.80	83.85
TC1100001000.mm.1		55.38	63.87	74.54	40.57	36.58	52.43
TC1600001724.mm.1		47.28	63.37	34.65	26.38	29.87	26.95
TC0400002881.mm.1		28.58	22.41	19.87	14.33	18.30	12.88
TC0200004572.mm.1	Gm14012; RP23-340F21.2	27.90	32.06	33.58	26.82	24.62	21.66
TC1100000628.mm.1	Gm24198	105.79	127.77	133.62	90.58	91.83	64.27
TC0900000932.mm.1		361.65	471.00	394.13	335.23	312.75	309.83
TC1000000189.mm.1		75.05	81.40	62.73	49.55	19.86	15.35
TC1000001875.mm.1		75.05	81.40	62.73	49.55	19.86	15.35
TC0X00001258.mm.1		42.59	41.26	59.84	25.41	26.33	36.36
TC0400003614.mm.1		39.11	39.61	33.15	27.15	28.68	32.63
TC1800001347.mm.1		52.89	57.58	45.12	43.12	29.98	34.07
TC1400001542.mm.1		67.55	78.65	56.53	49.13	52.03	49.67
TC0400000050.mm.1	Gm25355	75214.18	80631.34	79951.77	69124.98	48094.65	56338.54
TC0400001074.mm.1	Gm23064	75214.18	80631.34	79951.77	69124.98	48094.65	56338.54
TC0400001967.mm.1	Gm13067; RP23-169H13.4	58.43	54.90	50.57	40.88	24.80	38.12
TC0400002078.mm.1		117.50	159.63	137.85	111.55	99.37	91.38
TC1200000361.mm.1		141.98	160.89	113.00	81.63	59.55	101.65
TC1300001249.mm.1	Gm15289	105.18	99.25	109.30	82.96	83.35	97.34
TC1300002650.mm.1		11.59	13.20	12.91	9.92	10.05	11.48
TC1000002908.mm.1		85.87	77.38	104.19	58.06	65.03	71.60
TC0400000636.mm.1	Gm24277	41.92	43.49	71.85	31.76	30.37	23.23
TC0700003707.mm.1	Gm15661	531.59	614.86	710.11	506.33	377.15	347.98
TC1600000875.mm.1		286.91	454.82	235.08	116.13	111.13	204.66
TC1400001318.mm.1	B930095G15Rik	46.20	41.11	41.28	33.28	22.40	32.37
TC0800000875.mm.1		27.48	23.07	25.41	19.56	22.13	17.36
TC1800000264.mm.1	Gm26378	7955.49	6842.46	7723.91	6041.55	6060.53	6779.94
TC0600003535.mm.1	E130112N10Rik; Vamp1	86.67	75.09	94.60	73.11	64.10	65.76
TC1900000346.mm.1		96.88	98.66	88.89	74.76	63.49	83.64
TC0100003624.mm.1		161.85	224.32	137.04	120.44	104.38	102.05
TC1400001126.mm.1		41.93	26.56	27.87	22.48	18.09	18.63
TC1200002054.mm.1		131.02	163.39	149.87	122.47	91.18	110.99
TC0700002227.mm.1		6172.88	6326.55	5574.77	5518.16	4696.24	4751.13
TC1000002115.mm.1		221.56	246.57	250.39	197.04	151.38	197.15
TC0600001555.mm.1		29.99	26.89	34.54	20.17	23.76	24.89
TC0400000105.mm.1		43468.55	49207.21	48790.42	40856.18	31618.38	37957.67
TC1200001705.mm.1		25.17	41.94	30.76	21.05	18.08	21.36
TC0600003309.mm.1		34.89	29.99	35.38	18.49	26.07	25.78
TC0200000281.mm.1		98.90	84.44	65.09	56.89	53.58	37.65
TC0300001438.mm.1		29.96	54.69	39.24	21.14	26.86	21.06
TC1200001213.mm.1	LOC100861856	86.25	96.84	77.18	74.19	64.77	58.74
TC0100002115.mm.1	Gm27430	2205.97	2964.40	1739.29	1345.98	1040.17	1524.58
TC0900002824.mm.1	Gm16200	8080.49	7778.60	9120.25	7473.63	6282.08	6128.71
TC1300001312.mm.1		53.42	49.32	39.05	32.39	37.95	32.94
TC1300002630.mm.1	Mir325	12256.56	17537.21	15492.95	7293.93	11579.56	8888.92
TC0100001460.mm.1		262.55	369.97	237.04	185.59	172.29	204.81
TC0200000692.mm.1		64.17	52.02	46.31	34.25	34.72	43.79
TC0800001290.mm.1		166.11	188.37	171.84	94.04	125.22	144.39
TC1400001742.mm.1		75.90	116.16	82.45	53.61	61.64	64.07
TC0100003490.mm.1		28.07	42.53	41.62	25.52	22.11	25.29
TC1100003389.mm.1		272.28	299.98	321.28	222.69	135.13	208.43
TC1000000190.mm.1		2104.54	2212.22	2023.06	1777.36	1183.03	1542.37
TC1500000117.mm.1		2104.54	2212.22	2023.06	1777.36	1183.03	1542.37
TC0400000473.mm.1		77.61	143.38	88.38	49.50	30.35	59.56
TC0800000780.mm.1	Gm27628	283.59	455.86	266.24	203.58	137.99	195.82
TC0500000089.mm.1		52.18	64.81	47.71	41.89	39.50	42.86
TC1900000305.mm.1		19.72	25.70	21.71	16.58	16.78	18.67
TC1300002584.mm.1		145.64	166.40	159.99	100.36	110.88	139.41
TC0400001718.mm.1	Mir6399	34.90	41.79	39.07	28.61	17.35	26.94
TC1400000209.mm.1		20.84	26.61	24.82	14.15	13.63	20.19
TC1700000128.mm.1		54.41	62.87	51.26	39.46	33.80	47.66
TC1600000879.mm.1		115.67	154.38	117.37	58.32	58.11	101.40
TC0X00002693.mm.1		31.76	31.81	30.15	28.48	21.88	21.04
TC1100002137.mm.1		17.53	22.12	23.06	16.77	15.65	15.85
TC0400001860.mm.1	Gm13224	2467.88	2239.87	2703.66	2102.34	1455.54	1727.58
TC0200000925.mm.1		62.81	72.42	77.22	50.88	43.61	60.15
TC0400000290.mm.1		39.59	51.51	42.68	36.98	33.79	33.27
TC1500000943.mm.1		73.61	66.70	58.70	56.30	53.15	48.27

TC0100000833.mm.1	Gm25198	80.11	121.61	96.29	67.09	64.96	72.53
TC0800000377.mm.1		39.90	33.80	32.78	24.65	29.97	20.84
TC0400002452.mm.1	Gm25931	2458.68	3285.56	1864.97	1037.84	738.91	1652.85
TC0900001143.mm.1		108.39	113.72	97.97	94.89	70.41	76.20
TC1100000163.mm.1		28.48	38.08	35.71	18.72	27.17	22.81
TC1500001404.mm.1		66.22	52.60	48.76	39.88	38.45	45.08
TC0300001068.mm.1		62.42	65.27	51.55	48.48	48.92	46.88
TC1100004119.mm.1		101.39	123.60	112.91	68.45	77.44	97.00
TC0400000033.mm.1	Gm20021	51.09	61.48	61.40	48.42	47.37	49.04
TC0600001311.mm.1		175.00	126.35	182.66	75.96	82.78	123.82
TC1600000909.mm.1		175.00	126.35	182.66	75.96	82.78	123.82
TC0300000454.mm.1		191.41	323.93	222.88	142.70	73.71	135.05
TC1600000481.mm.1		51.17	44.98	48.45	42.10	27.73	28.59
TC0100001109.mm.1		94.11	112.64	94.45	73.16	39.97	64.43
TC0400001548.mm.1		57.23	74.73	73.69	54.97	52.44	48.74
TC1500000252.mm.1		80.81	100.81	78.79	54.36	54.27	73.12
TC1300001213.mm.1		25.64	32.19	33.46	18.44	24.90	21.34
TC1300002232.mm.1	Gm27501	2130.15	2609.09	2174.11	1612.92	1027.63	1679.86
TC1000002849.mm.1		21.31	32.17	25.21	19.00	12.85	16.85
TC0800002075.mm.1		29.68	25.69	29.35	25.22	20.58	19.21
TC1700002402.mm.1	Gm24194	240.44	344.40	243.21	156.77	155.90	213.31
TC1900001409.mm.1	Gm27440	820.89	958.79	800.07	606.73	379.43	625.49
TC1100001951.mm.1	Gm24323	3742.05	3653.82	3625.02	3358.62	2519.04	2449.78
TC0500002812.mm.1	Mir703	36292.47	36074.24	41069.50	25767.42	23596.72	33453.02
TC1500001914.mm.1		113.11	160.58	133.43	76.42	63.90	106.05
TC1600000871.mm.1		132.11	153.58	95.61	76.07	62.28	90.20
TC0900001412.mm.1		50.81	72.56	61.06	47.76	43.67	42.21
TC1400000814.mm.1		23.85	34.01	30.64	19.34	23.25	19.54
TC1500000061.mm.1		13.29	16.02	16.33	12.58	12.68	11.09
TC0100002695.mm.1		66.95	83.86	69.46	56.81	35.16	49.79
TC1100003242.mm.1		54.48	37.17	61.03	29.02	35.56	29.37
TC0400002173.mm.1	Gm11794	366.84	376.60	311.72	284.90	306.03	286.73
TC1900000472.mm.1	Pten	578.98	616.57	538.69	418.88	309.90	475.11
TC1000002412.mm.1		77.87	82.32	77.91	66.18	44.27	59.82
TC1100000795.mm.1		125.32	180.66	113.40	90.79	52.64	82.06
TC1800000972.mm.1	Gm26244	188.08	181.03	232.59	145.14	124.74	165.75
TC0300002175.mm.1		57.73	45.44	54.05	37.63	43.65	41.98
TC0700002737.mm.1		1019.90	1054.19	990.94	901.94	814.20	665.82
TC1300000933.mm.1		19.45	19.87	18.04	12.03	15.13	16.49
TC1500002287.mm.1		40.66	39.25	41.72	31.55	25.86	35.80
TC1300001906.mm.1	Hsp25-ps1	4201.87	3163.19	2927.63	2501.28	1742.35	2342.70
TC1100000154.mm.1		220.39	214.98	178.45	172.52	129.06	112.91
TC1800000602.mm.1		18.01	16.49	18.73	10.76	11.85	15.70
TC1800000053.mm.1		84.98	117.89	107.09	59.55	46.12	79.80
TC1600000545.mm.1		93.74	100.58	68.54	58.44	48.15	65.15
TC1600001463.mm.1		24.01	23.45	20.39	18.72	17.63	20.15
TC0800000508.mm.1		751.61	809.95	665.36	507.42	396.50	613.97
TC1600000947.mm.1		66.35	70.35	67.32	53.83	64.14	53.33
TC1800001705.mm.1		60.22	75.05	64.60	32.98	49.45	49.59
TC0500002507.mm.1	Gm15794; AC132284.1	35.73	28.22	33.24	26.05	27.30	24.30
TC1600001536.mm.1		30.96	27.01	23.83	19.44	21.64	22.48
TC0200000787.mm.1		72.60	114.21	96.96	55.08	48.67	70.09
TC1200001753.mm.1	Gm24305	46252.26	43916.71	43968.51	38720.02	25113.25	31319.15
TC0500002834.mm.1		268.31	206.38	189.50	122.77	175.47	137.38
TC0100002239.mm.1	Gm23722	380.33	483.39	480.52	356.40	280.24	347.36
TC1900001327.mm.1		29.99	47.96	36.59	19.83	25.05	26.55
TC0300002508.mm.1	Rnu1b1	17773.46	15095.19	17739.27	13261.57	8016.53	11528.38
TC0600003303.mm.1		25.80	24.47	27.66	23.10	19.18	16.64
TC0100000842.mm.1	Gm15371	30.44	23.11	26.37	22.43	18.58	17.26
TC0400002329.mm.1		4112.71	4149.27	3727.89	3416.45	2293.71	2909.11
TC0600000488.mm.1	Gm15550	18.84	18.60	19.54	16.85	12.45	15.20
TC0100002453.mm.1	Gm11605	3065.31	3234.34	2511.48	2353.45	1791.88	2243.14
TC0400000606.mm.1		16.27	15.82	12.51	10.71	12.35	9.85
TC0X00002413.mm.1	Gm14736	34.13	45.50	39.12	28.25	30.15	32.53
TC0500002191.mm.1	Mir3473e	35.63	36.43	37.82	25.34	16.99	28.99
TC1100000207.mm.1		56.27	70.59	65.68	45.58	42.45	55.54
TC0200001665.mm.1		147.50	179.70	122.71	106.97	88.73	112.37
TC0300003078.mm.1		296.62	331.22	316.01	230.85	243.04	290.11
TC1100003213.mm.1	Rps12-ps19	3412.50	2762.79	3391.51	2473.78	1679.64	2351.76
TC1700002608.mm.1		77.63	140.33	69.71	43.50	37.17	56.62
TC1800001403.mm.1		19.90	17.97	15.25	12.15	14.92	13.11
TC1800000911.mm.1	Gm22694	1902.52	2003.11	2444.18	1686.53	948.87	1237.02
TC1500001959.mm.1		46.48	63.83	61.28	41.99	44.73	39.50
TC0200003612.mm.1		24.46	30.47	28.51	20.39	24.09	18.61
TC0200002259.mm.1	Gm14148	53236.66	54215.78	56885.21	50069.88	37226.96	41656.10
TC1200000707.mm.1		46.27	47.32	36.70	28.57	35.76	24.14
TC1500000223.mm.1		23.65	25.14	22.64	18.27	17.88	22.09
TC0200005093.mm.1		46.37	56.22	41.37	38.54	34.04	36.43
TC1000002724.mm.1		35.81	36.91	32.04	31.59	28.16	25.83
TC0300002026.mm.1		21.80	28.44	21.86	15.14	19.90	13.98

TC1100002922.mm.1		32.30	34.00	33.10	30.73	23.66	23.48
TC1000000360.mm.1	Gm25526	40.82	46.79	60.68	34.11	34.17	36.62
TC0200001718.mm.1		207.22	219.11	165.84	141.01	131.93	164.34
TC0900002178.mm.1		136.37	142.02	154.38	102.52	91.23	128.91
TC0900000740.mm.1		33.73	44.17	33.59	22.31	29.57	27.01
TC0500001490.mm.1	Gm17131	5645.11	6763.68	8083.60	5139.32	2666.74	3667.66
TC0900002628.mm.1		39.19	33.19	41.76	29.80	23.00	30.53
TC0300002280.mm.1	Gm19382	27.19	39.82	32.14	22.34	21.83	25.70
TC0X00000016.mm.1	Gm15249; RP23-22L6.7	29.97	22.27	31.30	20.50	20.68	20.00
TC1900000085.mm.1	Gm19474	108.74	60.60	95.63	55.69	26.34	40.94
TC1500000955.mm.1	Gm22045	166.47	125.27	126.06	98.99	84.53	111.59
TC0400003643.mm.1		42.42	33.37	40.40	32.96	29.82	29.32
TC1200000934.mm.1		69.24	86.23	82.40	69.13	54.32	52.85
TC1700002530.mm.1		31.92	42.28	24.82	21.72	16.26	21.13
TC0300002661.mm.1		178.06	170.07	137.65	134.40	102.33	118.89
TC1300001184.mm.1		71.68	74.11	50.58	44.85	39.92	48.56
TC1600001823.mm.1		2094.16	2845.99	2507.75	2022.96	1247.59	1449.35
TC1000002027.mm.1		38.68	46.03	50.50	32.30	35.92	37.02
TC1100003772.mm.1	Gm27374	48.36	66.12	57.58	47.52	39.22	37.94
TC0400001264.mm.1	ZNFX1-AS1_2	12.43	15.25	14.67	9.90	12.01	11.45
TC1500001161.mm.1		2767.62	3132.71	2737.15	2542.75	1704.27	1884.66
TC0600002907.mm.1		25.59	29.23	28.41	19.52	21.15	25.20
TC0200003102.mm.1		275.46	377.45	306.97	265.22	200.09	196.72
TC1100004258.mm.1	Gm12587	1993.14	1972.36	1791.59	1724.90	1210.28	1341.65
TC0900002412.mm.1		55.12	58.26	53.33	36.53	38.24	50.73
TC0200001682.mm.1		504.48	542.19	604.28	483.61	329.61	337.24
TC0900002265.mm.1		297.67	312.23	455.05	265.41	190.72	164.31
TC1200000954.mm.1		3805.79	4602.16	4594.76	3692.72	2923.72	2469.96
TC0900000038.mm.1		13.12	10.60	11.58	10.36	9.03	8.59
TC0800003101.mm.1		1174.21	846.30	730.33	388492.10	229250.90	288281.60
TC1700001619.mm.1	Atp6v0c; Atp6v0c-ps2	4893.44	4802.17	4837.52	6486.43	6666.33	6379.08
TC0X00001712.mm.1		19.26	18.90	19.43	27.94	27.31	28.89
TC0900000702.mm.1	Gm6166	51872.00	50382.66	59099.32	164500.50	176247.60	186346.70
TC1200000195.mm.1		32704.27	33213.34	32810.89	48157.16	46281.32	49398.98
TC1800001587.mm.1	Gm14328	13606.86	12524.10	15199.70	50762.23	44599.04	47182.83
TC0200004759.mm.1	Snord17	633.09	647.68	652.68	1003.22	953.17	927.44
TC1600001433.mm.1	Gm20328	8.52	8.86	9.12	12.06	12.07	12.23
TC0800002027.mm.1	Gm6100	357.26	325.43	386.10	1071.80	959.81	1142.02
TC0700002138.mm.1	Gm15930	1387.53	1304.56	1168.88	3224.67	2912.66	3195.91
TC0900000541.mm.1	Fxyd2	62.25	59.00	52.94	350.91	253.32	269.87
TC0800000516.mm.1	Sorbs2	22.36	23.44	24.34	47.05	42.74	41.69
TC1800000050.mm.1	Fabp5l2	990.99	913.20	981.53	3693.21	2821.49	3546.01
TC0100000198.mm.1	Gm19680	157.05	149.39	152.67	248.17	280.61	254.44
TC0200000042.mm.1		442.41	410.83	463.32	1000.37	1046.78	1225.79
TC0200004450.mm.1	Gm13998	1314.49	1264.02	1514.41	5896.32	4304.70	5064.74
TC0700000689.mm.1		15.32	15.33	14.09	26.18	25.10	23.47
TC1100001243.mm.1	AI662270	205.12	209.16	192.08	330.24	302.99	309.13
TC0700000688.mm.1		26.35	28.43	28.12	36.43	36.63	37.09
TC0800000968.mm.1	Syce2	206.68	214.14	210.88	282.75	282.41	265.04
TC0200000372.mm.1	Gm13392	1322.65	1237.00	1153.86	2459.46	2186.14	2482.81
TC0300001634.mm.1	LOC100862399	1496.92	1496.80	1184.78	6948.25	5316.00	7830.62
TC0700002140.mm.1	Gm10693	177.93	182.38	177.41	381.90	312.67	348.14
TC1100002047.mm.1	Gm11771	184.91	157.88	184.55	515.29	441.45	405.98
TC1900001474.mm.1	Gm23800	46.43	45.97	45.41	62.53	70.18	65.48
TC0800000551.mm.1		13.37	12.16	12.28	17.10	17.59	17.29
TC0700001714.mm.1	Gm5601	25783.67	25540.90	23698.29	35047.69	34974.55	37602.91
TC0700002139.mm.1	Gm15925	2273.93	2315.05	2063.52	4226.00	3830.20	4602.65
TC0900000680.mm.1	Peak1os	3141.93	3137.01	3411.30	4729.64	5236.37	4785.21
TC1700001552.mm.1	Vmn1r-ps149	13.48	13.55	14.41	18.66	20.04	20.64
TC0500000010.mm.1		955.03	826.46	820.13	1578.53	1455.21	1631.39
TC0900001684.mm.1		15.64	14.70	14.33	19.27	19.71	20.25
TC1800001540.mm.1		13.56	13.30	14.37	18.36	18.74	17.58
TC1500000310.mm.1		9.68	9.15	9.32	12.92	11.89	12.30
TC1100000683.mm.1		46.43	42.73	42.87	71.85	63.51	66.15
TC1400001972.mm.1	Gm3601	37362.92	41103.34	38706.03	124430.60	89982.30	94939.79
TC0900001596.mm.1	Gm24044	190.57	179.99	184.97	240.59	265.99	261.12
TC0100003549.mm.1	Ly9	758.72	716.81	674.26	1805.03	1398.47	1803.03
TC0300000056.mm.1	C030034L19Rik	20.19	21.12	21.21	43.11	34.44	37.65
TC0100000928.mm.1		19.21	19.58	19.00	24.19	26.73	26.40
TC1500000192.mm.1		11.75	10.78	10.91	14.61	15.83	15.24
TC0700004005.mm.1		92.93	123.97	89.05	313.10	258.81	313.36
TC1100001258.mm.1		95.49	83.93	94.46	211.48	165.47	197.48
TC0600003220.mm.1	Gm15987; Mir7649	19.70	21.79	18.25	55.41	41.21	46.13
TC0900000119.mm.1	Gm24357	19445.70	20911.98	21853.33	30123.92	30732.44	28329.87
TC0100003438.mm.1	Gm16550	499.77	501.37	454.12	662.63	640.45	674.02
TC1600000747.mm.1		93.04	119.67	97.02	310.57	233.76	290.28
TC1100002845.mm.1	2610507I01Rik	68.34	67.67	67.18	84.08	93.19	92.07
TC1900000104.mm.1	Gm5510	1420.32	1283.92	1226.83	1932.73	1861.59	1926.84
TC1200001701.mm.1	Gm25015	15.46	14.28	14.16	21.45	20.66	19.40
TC1700000342.mm.1	Rab26os	136.23	116.27	132.42	190.42	191.50	192.81

TC1400001677.mm.1		34.91	36.09	40.27	54.03	54.37	51.95
TC0200001411.mm.1	Pex16	161.39	156.31	159.01	197.66	219.88	218.51
TC0300001021.mm.1		11.35	12.16	12.75	15.76	16.58	16.28
TC1100001807.mm.1	Gm23120	13.03	14.07	12.98	16.69	17.59	17.91
TC0700000181.mm.1	Gm26056	52.45	52.23	50.22	70.51	63.59	68.93
TC0200000384.mm.1		18.80	17.88	19.35	36.12	30.61	40.00
TC0X00000918.mm.1	Mir223; F630028O10Rik	2782.35	2655.46	2637.45	4387.56	3711.45	3943.63
TC1100003657.mm.1	Snora21	3421.65	3377.55	3439.05	5486.11	4602.89	5197.70
TC1100002951.mm.1	Tvp23bos; RP23-428I5.4	42.90	40.40	40.92	55.77	58.32	52.00
TC0500003396.mm.1		79.79	88.32	73.66	121.91	124.93	118.49
TC0600001444.mm.1		78.22	83.09	93.95	178.88	143.56	174.68
TC0X00000732.mm.1	Xlr4e-ps	68.12	54.51	66.30	113.29	110.72	128.06
TC0600003101.mm.1		161.38	144.90	142.72	217.78	257.65	244.20
TC0800000849.mm.1	Gm7984	7806.11	7398.12	6774.65	11979.66	11561.53	14016.50
TC1200002225.mm.1		701.93	694.95	658.15	910.83	1019.87	904.21
TC1800000540.mm.1		9.08	8.16	8.47	12.36	11.65	11.16
TC0500001882.mm.1		18.66	18.65	16.57	23.88	25.48	24.40
TC0X00002904.mm.1	Gm14910	7.78	9.00	8.15	12.28	12.38	14.15
TC0300002335.mm.1	Gm22935	379.85	391.01	366.42	555.22	606.24	700.16
TC1300000238.mm.1	Gm11333	9.16	9.22	8.18	13.27	15.49	13.13
TC0800002726.mm.1		15.73	15.25	15.84	21.59	21.40	25.18
TC0200000381.mm.1	Gm13411; RP23-218B17.1	16.41	19.17	24.18	41.82	47.01	43.75
TC0400001713.mm.1	LOC100862140	15.16	14.50	18.41	33.44	47.10	49.63
TC1000000958.mm.1		7.06	8.95	8.79	41.73	24.60	25.20
TC1100003004.mm.1	LOC100861608	28.39	30.21	34.87	64.09	55.28	75.02
TC0800001708.mm.1		92.11	89.75	83.84	124.34	117.32	136.28
TC0800002358.mm.1	LOC100861650	146.02	130.46	150.29	232.90	205.50	204.45
TC0200000458.mm.1	Gm23969	504.43	411.50	506.17	863.52	939.36	772.51
TC1_GL456210_random00000005.mm.1		2163.72	2256.30	1984.80	4079.34	4995.53	3623.38
TC1_GL456211_random00000005.mm.1		2163.72	2256.30	1984.80	4079.34	4995.53	3623.38
TC1_GL456211_random00000026.mm.1		2163.72	2256.30	1984.80	4079.34	4995.53	3623.38
TC1_GL456221_random00000004.mm.1		2163.72	2256.30	1984.80	4079.34	4995.53	3623.38
TC0X00000876.mm.1	Gm6982	10.94	11.21	11.19	16.54	14.14	14.93
TC1700002700.mm.1		1855.13	1602.98	1628.98	2852.69	2425.46	2809.69
TC1300001451.mm.1	Gpr137b-ps	7063.67	7214.32	6674.93	10309.25	9038.55	10577.06
TC0400002833.mm.1	Tcp1-ps1	16.98	15.87	17.15	23.01	23.60	20.81
TC0700000398.mm.1		41.82	52.33	52.48	89.07	89.64	78.64
TC0100000217.mm.1		24.16	28.92	26.67	38.46	39.49	43.63
TC0800001796.mm.1	Gm17149	6210.57	6208.72	6502.82	12221.11	9447.74	10115.94
TC0400003274.mm.1		16.08	15.54	17.76	21.56	21.70	21.01
TC1400002727.mm.1		10.72	10.57	11.59	14.73	13.47	13.96
TC0900002295.mm.1		367.76	411.04	392.92	524.85	534.21	604.03
TC1300002716.mm.1		21.49	22.08	23.28	31.68	29.21	27.83
TC0300000692.mm.1		17567.21	14375.95	13361.74	26150.00	24708.37	25465.87
TC1600000210.mm.1		17567.21	14375.95	13361.74	26150.00	24708.37	25465.87
TC0400003729.mm.1	Gm13131	185.19	190.11	180.87	251.72	222.32	246.38
TC0300000108.mm.1		13.03	11.45	11.91	15.63	17.28	17.46
TC1600001492.mm.1	0610012G03Rik	287.28	290.74	303.87	463.82	391.68	484.75
TC0M00000028.mm.1	mt-Te	205319.40	214993.60	188787.00	307172.40	284762.20	350021.90
TC0800001108.mm.1	Ap3s1-ps2	4216.93	3435.34	3858.82	7420.41	5958.08	6321.36
TC0800002356.mm.1	LOC100861638	13.11	15.60	16.51	52.09	39.17	31.39
TC0400003266.mm.1	Ttc39aos1; RP23-110H24.2; Gm12750	51.25	56.24	57.86	78.20	70.81	81.12
TC0400002845.mm.1	Gm11223	2419.89	2682.02	2358.79	3946.38	3632.38	4631.45
TC1600002002.mm.1		7.64	8.18	7.64	9.66	10.72	11.20
TC0X00002725.mm.1	Gm14845	1509.42	1514.03	1310.61	2059.60	2573.89	2350.42
TC0200004207.mm.1	Ldlrad3	95.68	82.99	92.62	141.89	122.22	127.27
TC0100002686.mm.1		9.01	10.28	9.42	12.48	13.57	12.31
TC0600000516.mm.1		9.86	9.85	10.41	13.62	14.28	12.26
TC1800000664.mm.1	Gm23016	12.96	13.31	11.21	16.76	17.56	17.22
TC0500003475.mm.1		27.20	30.98	23.99	48.56	41.44	45.81
TC1400000123.mm.1		13.78	15.50	14.67	19.06	19.88	17.96
TC0X00000362.mm.1	Zfp36l1-ps	939.06	905.71	785.98	1420.57	1223.77	1284.62
TC0200004918.mm.1		221.73	247.38	219.94	284.09	289.55	301.38
TC1300001730.mm.1	Gm23206	14.66	13.61	15.90	19.35	18.82	20.11
TC0X00003379.mm.1	G530011O06Rik	108.11	94.43	78.89	187.02	161.07	213.58
TC1600002178.mm.1	Litaf; Gm19955	202.63	202.56	211.11	272.89	255.72	305.13
TC0600001941.mm.1		11.30	11.17	12.47	14.91	16.88	15.12
TC0400001078.mm.1		11.42	11.11	11.10	15.98	15.27	13.52
TC0600000361.mm.1	Gm15487; Atp6v0c	109645.50	99959.00	90721.95	134784.60	134621.30	136959.80
TC0200003773.mm.1	Mrpl23-ps1	3843.54	2834.76	3087.97	6076.32	5171.36	6009.35
TC0X00000759.mm.1	Gm14709	9.25	10.37	10.02	12.72	13.97	15.29
TC0400002543.mm.1	Olfr29-ps1	16.73	16.16	17.63	23.71	23.96	20.58
TC1000001670.mm.1	Gm16149	101.00	90.68	92.05	136.42	122.03	119.56
TC1400002674.mm.1	Gm17366	38.46	38.74	30.35	71.30	86.44	60.54
TC0200005075.mm.1		8.31	8.40	8.09	10.18	12.43	11.56

TC0500003276.mm.1		344.99	310.79	236.85	644.75	543.18	752.35
TC0X00001214.mm.1		17.05	14.40	16.93	22.13	21.92	24.51
TC0X00002732.mm.1	Gm25828	14.74	17.09	17.50	23.81	23.02	21.48
TC1800001301.mm.1	Mospd4	29.19	26.09	28.11	37.89	40.16	34.32
TC1000001874.mm.1		5.88	5.91	6.24	8.54	11.23	8.92
TC0200001512.mm.1		10.67	10.01	10.13	12.96	16.11	14.63
TC0200000046.mm.1		169.33	213.72	217.39	337.05	298.11	312.02
TC0500000297.mm.1	Slc35f6; Mir5625	4846.93	4969.65	3863.35	6856.84	6770.08	7208.42
TC1700002823.mm.1	H2-T10	363.15	334.92	327.83	522.67	465.72	611.29
TC0100000730.mm.1		56.81	57.60	49.78	84.42	100.74	123.95
TC0100000739.mm.1		56.81	57.60	49.78	84.42	100.74	123.95
TC0100000748.mm.1		56.81	57.60	49.78	84.42	100.74	123.95
TC0100000753.mm.1		56.81	57.60	49.78	84.42	100.74	123.95
TC0100002706.mm.1		56.81	57.60	49.78	84.42	100.74	123.95
TC0500003459.mm.1		56.81	57.60	49.78	84.42	100.74	123.95
TC1_GL456211_random00000012.mm.1		56.81	57.60	49.78	84.42	100.74	123.95
TC1_GL456211_random00000017.mm.1		56.81	57.60	49.78	84.42	100.74	123.95
TC1_GL456212_random00000002.mm.1		56.81	57.60	49.78	84.42	100.74	123.95
TC1_GL456212_random00000012.mm.1		56.81	57.60	49.78	84.42	100.74	123.95
TC1_GL456221_random00000006.mm.1		56.81	57.60	49.78	84.42	100.74	123.95
TC1_GL456221_random00000023.mm.1		56.81	57.60	49.78	84.42	100.74	123.95
TC0700003304.mm.1	Gm22776	17.20	17.44	16.21	23.97	25.10	20.78
TC0700000117.mm.1		12.72	13.84	13.21	17.88	17.90	15.69
TC0100000134.mm.1		10.52	11.31	11.83	13.91	16.41	15.05
TC0700002991.mm.1	Snord35b	224.54	207.13	205.39	273.96	298.00	343.01
TC0300001635.mm.1		28.70	30.72	33.32	50.46	42.65	57.19
TC1300002669.mm.1	Gm9025	411.43	423.84	351.92	549.10	560.12	650.98
TC0900000650.mm.1	LOC100862116	8.54	10.24	9.79	12.64	13.38	12.33
TC0500002152.mm.1	Tyms	277.73	283.58	250.24	372.77	338.10	402.46
TC0X00001034.mm.1	FTX_1	20.39	21.62	20.29	26.00	31.75	27.49
TC0700001965.mm.1		15.31	15.90	15.49	20.45	26.56	22.12
TC0300002825.mm.1		10.18	10.18	9.59	11.98	14.42	13.44
TC0300002235.mm.1	Gm26771	13.36	16.26	19.04	25.56	27.95	27.51
TC1500002015.mm.1		151.57	164.27	139.57	201.09	196.77	227.53
TC0600001838.mm.1		11.33	10.90	11.86	15.69	15.49	13.44
TC0600002407.mm.1		14.57	14.18	14.68	19.01	19.02	16.57
TC0500001919.mm.1		9.15	11.87	9.44	14.73	16.65	15.23
TC0700003078.mm.1		239.35	248.32	226.30	440.30	345.66	332.00
TC1900000690.mm.1		118.79	108.58	102.16	172.69	142.36	150.74
TC0700003637.mm.1		8.73	10.23	9.88	13.99	12.07	13.08
TC1000003133.mm.1	Gm26847	22.72	21.00	21.48	33.64	37.29	28.05
TC1800000519.mm.1		9.16	9.38	9.32	12.42	10.65	12.23
TC0300003172.mm.1	Gm24494	285486.70	279650.20	304004.60	362382.90	447588.80	388293.90
TC0300000257.mm.1	Gm25696	42.46	35.58	41.71	71.87	55.71	60.04
TC0X00002304.mm.1	Gm5637	36117.64	29206.63	29641.94	46164.41	43010.06	43657.17
TC0600001523.mm.1		13.65	14.20	11.74	19.05	18.81	23.46
TC0X00002916.mm.1	Gm5648	48.76	40.69	44.26	58.26	57.23	63.93
TC1500000507.mm.1		10.96	12.61	12.46	16.36	19.99	16.39
TC0X00001470.mm.1	Gm15113	7.32	7.46	7.71	8.88	10.45	10.70
TC0200000327.mm.1	Gm23970	84.11	75.36	61.39	110.32	113.87	124.23
TC0X00000526.mm.1	Gm23258	7.51	7.93	7.71	10.19	14.11	13.36
TC0600003221.mm.1		9.71	9.23	10.32	14.57	15.47	12.21
TC0400002333.mm.1	Gng2-ps1	1433.28	1268.60	1260.48	1674.28	1709.80	1952.15
TC0800002751.mm.1	Gm26493	266.71	263.41	272.95	601.63	386.49	494.95
TC0X00000607.mm.1	Gm6806	9.25	10.59	8.87	13.16	12.03	13.66
TC1700001711.mm.1	Cuta	15723.01	16488.84	15153.49	23287.50	19126.78	20901.48
TC0600002384.mm.1		84.20	107.40	62.22	197.83	160.04	205.84
TC0300002108.mm.1		18.67	18.21	21.14	23.57	25.02	26.13
TC0600001230.mm.1		13.26	13.19	12.45	23.03	18.38	17.39
TC1100000292.mm.1	Gm23109	12.94	10.17	12.44	18.25	16.56	16.61
TC0200001533.mm.1	LOC100862111	184.89	205.28	149.64	318.22	263.02	304.04
TC0700000119.mm.1	Vmn1r-ps54	8.21	8.89	9.72	13.22	11.26	12.00
TC1700002744.mm.1		10.77	11.20	12.48	13.81	15.36	14.95
TC0800002151.mm.1	Gm25309	12.20	14.47	15.85	19.96	21.00	19.62
TC0800002291.mm.1		11.01	10.90	11.55	13.90	15.60	17.94
TC0200001783.mm.1		26.40	21.59	21.76	39.49	35.64	31.37
TC0200000488.mm.1	Gm22675	16.85	15.60	16.26	22.85	19.07	21.31
TC0X00003267.mm.1	Gm15190	3331.58	3268.90	3482.94	4663.87	3980.31	4870.15
TC0600000766.mm.1		23.04	20.07	26.33	33.44	32.00	33.27
TC0100002938.mm.1	2900060B14Rik	214.89	205.30	222.71	299.44	251.13	290.64
TC0900002019.mm.1	Gm6762	36.77	35.94	39.72	70.46	61.24	49.90
TC0X00002097.mm.1		14.17	13.33	10.84	18.55	17.84	18.68
TC0200003432.mm.1	Gm13496; RP23-244N6.7	8.41	7.75	8.48	11.10	9.62	11.26
TC0700003282.mm.1	Gm23767	19.36	16.64	18.19	37.62	31.24	25.40

TC1200002637.mm.1		10.55	11.31	12.03	14.76	13.31	15.11
TC0600001184.mm.1		9.65	10.02	9.92	13.15	12.41	15.83
TC0200000621.mm.1		198.56	153.78	168.92	302.37	248.20	252.11
TC1200000511.mm.1		17.02	13.71	13.76	20.31	21.58	19.85
TC0500000514.mm.1		8.92	10.22	8.34	11.99	11.72	11.90
TC1700000317.mm.1		19.04	19.69	18.94	21.72	25.08	25.20
TC1200001538.mm.1	LOC100861907	22.41	20.90	16.89	29.78	30.74	27.93
TC0700004450.mm.1	5830432E09Rik	47.20	50.33	40.56	59.82	60.97	65.36
TC1300000909.mm.1		15.05	16.36	16.47	21.02	27.19	21.71
TC1800001247.mm.1		7.82	9.62	8.97	11.63	12.15	11.16
TC0500000199.mm.1	2700038G22Rik	97.51	98.51	84.17	129.54	136.37	173.06
TC0600000510.mm.1		10.60	11.18	9.19	13.13	15.47	16.05
TC0700002225.mm.1		18.81	19.76	21.05	23.92	29.25	26.41
TC0100001940.mm.1		9.96	9.41	11.33	13.71	12.63	13.23
TC0X00000401.mm.1	Gm14619	10.41	10.17	13.24	18.04	20.72	15.98
TC1100001783.mm.1	Snord104	1559.98	1409.30	1195.83	2705.69	2180.28	2012.27
TC1300001598.mm.1	Vmn1r-ps111	20.01	18.51	23.25	27.35	27.32	27.14
TC0X00002058.mm.1	Snora69	832.57	920.96	873.92	1085.92	1381.83	1429.96
TC0X00000673.mm.1	Gm20455; RP23-11C16.11	18.07	15.49	15.95	21.17	21.62	19.69
TC0400002283.mm.1		10.03	7.74	8.60	12.64	12.43	11.64
TC1000001806.mm.1		12.08	12.77	12.56	14.61	16.78	18.17
TC0300001721.mm.1		13.51	12.71	15.09	18.49	20.93	17.29
TC0700003638.mm.1		8.29	7.97	8.85	9.60	11.06	10.77
TC1500001924.mm.1		40.97	39.38	36.40	70.25	50.17	66.31
TC1100003552.mm.1		8.75	9.94	9.10	12.35	12.02	15.21
TC1700002295.mm.1		10.87	11.89	11.68	14.61	20.44	18.44
TC0400003819.mm.1		10.37	12.30	11.68	16.23	15.67	13.90
TC1800000069.mm.1	Gm10554	108.08	79.41	81.94	192.59	132.85	176.64
TC0100000078.mm.1		15.93	18.19	15.38	22.28	21.30	26.36
TC1300001485.mm.1		8.23	9.21	10.14	12.34	11.42	12.62
TC1600000616.mm.1		9.67	11.57	10.00	14.26	19.12	15.11
TC1100003060.mm.1	Gm12307	13.18	12.28	13.81	16.05	20.61	18.15
TC1200002303.mm.1	Scarna13; Mir3069	81.37	82.70	77.20	117.46	120.97	95.39
TC0200004891.mm.1		9.79	10.03	10.88	13.40	13.65	11.76
TC0100001387.mm.1	Glul; Mir8114	266.20	239.03	227.90	314.51	291.23	333.75
TC0100002724.mm.1		27.89	33.89	30.18	40.71	43.70	53.03
TC0200002581.mm.1	Gm23201	107.21	91.95	97.70	217.64	151.53	148.29
TC1300002266.mm.1		14.68	16.89	14.70	21.00	19.32	24.21
TC0600000767.mm.1	Gm15490	26.05	34.48	25.58	43.22	44.47	40.62
TC0500000771.mm.1		7.18	7.67	7.23	9.51	8.38	10.03
TC1100001976.mm.1	Gm11726	11.36	13.18	11.91	16.81	21.92	16.53
TC1600001541.mm.1	LOC100862381	16.32	16.81	15.52	19.93	26.51	26.68
TC1500001100.mm.1	Gm24128	15.76	17.03	12.81	22.23	23.54	20.47
TC0X00000164.mm.1	Rpl3-ps1	873.60	887.88	736.73	1023.39	1169.92	1172.90
TC0400000101.mm.1		7.25	8.09	8.51	9.37	10.46	10.53
TC0100001213.mm.1	Gm15851; AC157924.3	20.71	28.40	26.09	67.30	43.09	43.25
TC0X00002119.mm.1		13.81	11.19	11.72	15.97	18.30	15.89
TC1800000530.mm.1		9.61	9.62	11.40	13.73	13.10	12.46
TC0500003460.mm.1	Gm15753	1299.48	1137.81	1095.96	1925.56	1581.54	1529.48
TC0700001963.mm.1		350.40	362.77	263.14	616.88	499.16	790.46
TC1200001670.mm.1		14.47	11.61	13.38	17.20	20.35	17.42
TC0X00002137.mm.1		11.23	10.49	11.31	13.53	16.12	18.49
TC1100003507.mm.1	Gm15698	46.54	53.92	47.59	64.93	62.19	77.55
TC0100002816.mm.1	Myeov2	97.23	84.88	103.95	133.02	116.38	129.70
TC1100002587.mm.1	Gm12162; RP23-122N23.1	7.37	7.64	9.64	11.94	10.91	11.90
TC1300002369.mm.1		47.41	45.89	46.20	70.09	54.40	62.95
TC0200000350.mm.1	Gm13338	21.28	21.20	21.81	32.11	24.94	29.21
TC0100002238.mm.1	Snord89	70145.57	54093.96	70724.88	109667.50	96458.90	88599.85
TC0600001641.mm.1	Gm30332	10.57	10.38	11.34	14.80	14.25	12.28
TC0500000958.mm.1		25.05	25.04	28.07	31.46	38.24	32.93
TC0400003363.mm.1	Gm22980	179.04	137.56	176.00	461.05	311.37	262.69
TC0400001884.mm.1		15.20	14.44	13.99	33.88	25.85	20.17
TC0700001078.mm.1		28.17	23.53	24.38	34.16	38.08	31.18
TC1900000353.mm.1	Gm5514	85.93	79.30	66.57	145.98	113.04	190.05
TC0600000176.mm.1		9.24	6.85	8.64	11.17	14.44	14.89
TC1000000426.mm.1	Gm27723	9.94	10.70	12.48	14.56	19.36	16.13
TC0500000233.mm.1	Gm22656	12.88	11.48	14.52	35.49	19.43	26.47
TC0800000622.mm.1		13.68	18.01	18.66	28.58	26.04	23.13
TC1400000201.mm.1		60.41	64.57	61.56	87.06	73.77	98.08
TC0800001542.mm.1	Mir1967	14.32	14.37	14.27	16.54	21.46	20.26
TC1000001383.mm.1		7.19	6.11	6.52	8.03	8.48	9.70
TC1000002975.mm.1		7.19	6.11	6.52	8.03	8.48	9.70
TC0300001753.mm.1		8.62	7.36	7.29	9.95	12.32	10.14
TC0100000750.mm.1		7.64	11.99	10.73	41.65	86.80	24.03
TC1_GL456211_random00000016.mm.1		7.64	11.99	10.73	41.65	86.80	24.03
TC1_GL456212_random00000003.mm.1		7.64	11.99	10.73	41.65	86.80	24.03
TC1_GL456212_random00000010.mm.1		7.64	11.99	10.73	41.65	86.80	24.03

TC1_GL456221_random00000021.mm.1		7.64	11.99	10.73	41.65	86.80	24.03
TC1600000859.mm.1		7.90	9.34	8.23	11.15	12.17	10.17
TC0300002771.mm.1	Gm26076	11.12	11.60	10.12	14.26	14.63	18.83
TC0700001200.mm.1	Gm20077	23534.52	22069.84	19608.51	26052.84	31378.95	31471.93
TC0700003717.mm.1		17.92	23.69	22.89	31.85	32.60	28.23
TC0900002538.mm.1	Gm24225	27.77	49.19	33.64	69.84	65.77	66.51
TC0100001322.mm.1		10.20	9.44	11.04	12.50	14.38	16.51
TC0400002126.mm.1	9430025C20Rik	15.87	16.19	15.13	20.38	20.72	17.48
TC1200002487.mm.1	Mir6388	17.78	19.63	18.81	34.26	27.32	23.80
TC0X00000431.mm.1	Gm22528	16.21	14.65	16.07	17.93	21.70	21.73
TC0700002260.mm.1	Vmn2r-ps46	16.00	15.87	16.77	17.97	21.36	21.09
TC1600001911.mm.1		9.39	8.14	7.74	13.37	10.78	11.03
TC0700003327.mm.1	Gm22188	24.08	18.13	24.83	35.51	30.24	34.81
TC1700000024.mm.1		12.94	11.90	13.12	18.08	19.15	14.78
TC1100002774.mm.1		24.81	29.21	25.31	34.07	33.29	30.95
TC1000001068.mm.1	Tyms-ps	140.91	115.07	125.17	185.56	154.13	193.50
TC4_GL456216_random00000005.mm.1		60.83	43.60	53.26	73.27	73.22	77.19
TC0700003291.mm.1	Gm25944	26.00	22.09	24.90	45.67	35.42	31.86
TC0X00002737.mm.1	Gm9103	344.88	268.53	335.90	471.01	405.71	516.15
TC1100001986.mm.1		16.33	16.53	20.37	22.71	23.77	22.65
TC0X00000194.mm.1	Gm6923	19.16	17.93	16.53	29.66	22.02	24.80
TC0300001662.mm.1		9.20	9.95	9.50	12.61	12.51	10.62
TC1000002011.mm.1		15.47	15.24	17.73	21.30	18.83	22.40
TC0800001261.mm.1		706.75	632.14	523.59	871.31	843.51	1041.44
TC1100002838.mm.1		224.78	232.40	205.24	422.25	295.11	510.16
TC0400004053.mm.1	Gm13064	10.51	12.25	9.66	13.91	14.62	13.50
TC0300000677.mm.1	Gm25188	324.63	257.88	300.19	400.42	358.54	409.46
TC0800002540.mm.1	Gm25267	8.08	10.54	8.51	11.85	16.33	17.13
TC0100000089.mm.1		7.51	7.79	9.21	12.17	9.95	12.63
TC1700001941.mm.1		35.16	39.73	51.02	69.61	58.21	78.23
TC1000000443.mm.1		12.29	10.15	12.95	16.02	18.42	15.10
TC1300000732.mm.1	Gm24999	87.05	109.84	90.00	129.61	130.02	166.56
TC1100000050.mm.1		11.50	13.46	13.50	18.42	18.68	15.17
TC0200000027.mm.1		9.22	8.40	9.21	10.36	13.43	12.41
TC1000002758.mm.1		27.96	25.89	26.34	33.64	39.18	31.08
TC1300001343.mm.1		8.31	8.38	8.85	9.69	12.44	11.80
TC1700002310.mm.1	Gm16276	81.37	86.02	78.67	108.64	91.13	106.09
TC1300002738.mm.1		14.75	12.37	14.40	26.08	17.49	25.21
TC1700001640.mm.1	Snhg9	20.62	18.54	18.52	24.15	28.15	22.67
TC1800001387.mm.1		7.30	9.21	7.89	11.68	11.22	9.89
TC1700001713.mm.1	Mir3083	21.32	23.27	22.03	35.75	40.85	27.18
TC0700003311.mm.1	Gm22631	26.18	22.64	27.74	41.23	37.55	31.13
TC0100002066.mm.1		17.55	21.94	19.60	28.28	23.66	27.08
TC1700002759.mm.1		9.62	10.54	11.08	11.99	14.68	14.88
TC0100002682.mm.1		12.50	12.38	13.62	17.67	21.01	15.37
TC0100003469.mm.1	Gm16418	300.36	283.78	282.89	419.61	333.35	447.36
TC1100003398.mm.1		28.56	42.44	25.06	50.57	72.02	59.47
TC0400000097.mm.1		24.77	24.16	29.60	35.05	33.12	31.70
TC1800000180.mm.1		7.67	7.71	7.79	8.61	10.76	10.72
TC0100001809.mm.1		6.63	7.07	7.79	8.23	9.76	9.64
TC1300001324.mm.1	Gm23198	36.86	34.53	39.25	42.59	53.45	47.96
TC1400001154.mm.1		9.55	9.70	10.75	12.46	12.56	11.13
TC1100000895.mm.1	Snord118	345.34	289.39	298.95	380.40	453.41	387.60
TC0100001767.mm.1		11.38	13.29	13.28	14.88	18.29	18.89
TC0200003864.mm.1	Gm13711	18.47	22.53	19.18	25.33	23.93	25.61
TC1500001070.mm.1		9.15	9.25	8.80	11.96	12.04	9.97
TC0600000279.mm.1	MESTIT1_3	23.84	16.57	24.45	30.26	41.67	40.61
TC0300001464.mm.1		8.07	8.20	7.66	9.39	10.31	12.39
TC0800001461.mm.1	Gm26784; RP23-76C13.3	12.58	12.98	15.40	16.53	17.32	16.95
TC1000001586.mm.1	Mir677	132.96	152.73	133.16	189.90	208.04	162.94
TC0800001935.mm.1	Gm22227	10.05	9.32	12.74	16.60	14.39	14.54
TC1900001441.mm.1		14.11	18.79	16.38	22.66	20.71	22.73
TC1400002559.mm.1		9.94	11.19	10.51	12.42	16.68	14.38
TC1700002383.mm.1		10.97	12.74	10.97	15.83	13.30	15.38
TC1100001244.mm.1	Gm11427	80.48	58.84	60.94	116.24	87.92	103.47
TC0500001105.mm.1		8.96	8.93	10.43	12.57	15.08	11.51
TC0400001002.mm.1	Gm12686	32.43	25.86	25.46	34.37	40.68	41.75
TC0700003627.mm.1		10.53	10.68	11.51	15.23	15.49	12.29
TC1400000878.mm.1		11.10	12.36	13.71	14.64	17.33	16.34
TC0X00001709.mm.1		9.93	9.19	11.73	14.27	12.51	13.65
TC1500001543.mm.1		47.13	57.24	48.66	98.19	65.23	77.90
TC1600001155.mm.1	Nagpa	142.34	151.67	129.11	194.51	162.11	201.88
TC1400001951.mm.1		12.83	10.82	10.74	15.63	15.96	13.38
TC0100003157.mm.1	Gm23763	20.28	18.05	19.09	30.66	28.25	22.28
TC0900000814.mm.1	Gm23136	125.39	101.58	112.24	157.72	133.54	147.38
TC0800001783.mm.1		9.54	10.68	12.03	13.96	19.28	14.91
TC1200002197.mm.1		15.92	15.88	17.92	20.95	28.07	21.35
TC1100002495.mm.1		8.70	8.52	8.82	12.19	9.61	11.90

TC0300000081.mm.1		11.19	13.40	12.03	17.47	14.12	16.54
TC1300000146.mm.1		17.00	17.20	17.62	23.33	25.72	19.43
TC0X00001073.mm.1	Mir6382	12.49	10.54	8.92	13.65	18.47	17.27
TC1600001088.mm.1		10.78	11.10	12.21	17.08	13.02	15.61
TC1800000611.mm.1	Gm6960	110.52	92.87	74.64	140.71	158.57	125.50
TC1500001962.mm.1		174.36	212.11	150.11	237.77	255.65	248.87
TC0800002034.mm.1		23.88	26.83	24.16	27.58	31.90	31.56
TC0100003213.mm.1		12.30	10.39	12.03	13.31	15.98	16.18
TC1300001356.mm.1	Gm23308	10.70	10.40	10.35	11.82	15.94	15.02
TC0800001378.mm.1	Gm15655; AC113301.2	927.82	741.45	991.67	1754.18	1269.65	1234.42
TC1900000259.mm.1	Gm5512	90.37	95.50	72.56	128.90	106.02	127.92
TC1300000870.mm.1		17.27	16.23	16.26	19.26	25.27	20.93
TC0500000558.mm.1		11.26	12.44	10.89	14.92	17.77	13.56
TC0600001631.mm.1		1163.89	1144.21	1021.27	1702.91	1284.25	1751.13
TC0800002176.mm.1		10.97	12.36	14.81	16.93	18.10	16.14
TC0500001349.mm.1	Oas1b	155.22	151.90	140.77	219.84	168.05	207.85
TC0200002065.mm.1		10.10	11.26	9.99	11.62	13.22	13.92
TC0X00000115.mm.1	LOC100862340	1506.92	1384.79	1576.64	2713.61	1820.40	2148.90
TC0200002370.mm.1		33.52	45.94	33.68	64.00	48.87	58.33
TC0400003403.mm.1	Gm12855	11.26	8.83	10.41	13.24	15.35	12.53
TC0800001785.mm.1		10.07	9.91	9.65	14.10	11.10	15.07
TC0100001424.mm.1	Gm15428	524.40	413.01	433.56	788.06	565.32	668.21
TC1000001297.mm.1		9.46	9.23	10.08	13.62	13.58	10.69
TC0200004705.mm.1		10.62	9.49	8.41	11.37	14.02	12.63
TC0800001779.mm.1		12.86	13.60	12.39	16.74	17.00	14.15
TC0X00003356.mm.1		12.78	9.85	11.43	15.93	13.67	15.39
TC0400002659.mm.1		17.98	12.90	14.09	19.29	22.57	21.27
TC0100002839.mm.1	Gm22089	21.51	26.22	21.75	27.92	30.63	28.13
TC1100000307.mm.1	Gm22588	17.08	20.29	22.13	23.89	26.91	27.68
TC1200000684.mm.1	Gm24010	12.73	11.72	13.92	19.81	15.11	21.79
TC0X00001279.mm.1	Gm23124	9.39	9.74	9.31	12.65	10.30	12.60
TC1000002864.mm.1		15.03	14.12	11.76	18.50	18.71	16.29
TC0400003703.mm.1		14.08	14.22	15.42	20.29	18.67	16.17
TC1000001217.mm.1		38.21	22.72	34.87	49.34	51.54	59.06
TC1100001554.mm.1	Gm11633	77.89	76.39	89.87	98.06	104.77	128.09
TC0100000458.mm.1	Gm26293	118.52	94.86	136.66	170.46	155.68	162.82
TC0500003362.mm.1	Gm7902	1481.79	1659.14	1138.77	2090.12	1946.89	2004.13
TC0200000182.mm.1	Mir466	19.00	18.53	20.68	25.52	35.27	24.70
TC1000001853.mm.1	Gm4895	1367.28	1221.31	953.38	1604.70	1633.25	1662.44
TC0300000141.mm.1		17.26	18.97	20.81	26.49	26.00	21.69
TC0X00000220.mm.1		109.63	105.64	102.00	166.32	120.81	143.06
TC0200001651.mm.1	Olfr1293-ps	11.66	11.66	15.58	20.38	17.51	16.95
TC0700003666.mm.1	Vmn2r-ps67	8.63	7.77	8.72	11.95	9.45	12.49
TC1800000765.mm.1		82.90	109.04	92.08	209.41	121.50	173.06
TC0600000285.mm.1		10.72	11.82	13.75	17.55	17.91	14.31
TC0600001529.mm.1	Gm26160	21.64	20.38	25.72	32.45	26.79	35.12
TC0700004116.mm.1	Gm23345	24.98	23.42	29.65	32.12	52.26	49.22
TC0400000953.mm.1		12.49	11.18	13.60	16.25	14.12	16.18
TC0900002650.mm.1		11.74	15.00	11.07	15.75	17.69	17.68
TC0300002844.mm.1		8.70	8.97	8.32	12.87	14.07	9.83
TC0400003069.mm.1	Gm26410	13.95	17.11	13.98	20.73	21.12	17.47
TC1700001349.mm.1		12.67	12.05	12.99	17.83	18.96	13.99
TC1600000691.mm.1	Gm7204	17755.90	16618.79	17322.54	26261.44	19569.42	22076.61
TC0600002088.mm.1		8.82	8.76	10.26	12.41	11.59	10.50
TC0100003517.mm.1		43.64	54.30	44.14	57.69	68.06	81.75
TC0600001323.mm.1		251.42	315.28	247.08	365.38	336.87	338.08
TC1500001317.mm.1		8.88	9.60	8.28	13.40	11.41	10.28
TC0X00002151.mm.1	Gm5636	9.83	8.58	11.99	13.21	13.55	13.73
TC0100001046.mm.1		9.18	10.02	11.06	13.75	17.02	12.10
TC0X00000969.mm.1	Gm22472	12.95	15.39	17.07	18.28	20.55	20.01
TC1400002077.mm.1	Mir208b	7.80	7.84	8.15	8.69	11.43	11.18
TC1500001659.mm.1		52.08	40.66	43.36	62.08	65.16	53.38
TC0700004381.mm.1		12.65	10.53	11.51	19.86	14.60	26.45
TC0800002355.mm.1		11.52	11.78	8.11	22.89	13.77	24.08
TC0100000877.mm.1		15.74	15.11	17.37	17.84	22.49	21.22
TC1800000002.mm.1		21.38	23.75	19.02	29.70	24.56	28.71
TC0100001121.mm.1		3084.19	3155.56	2465.05	3428.80	4214.63	4163.57
TC0900001958.mm.1		10.30	9.64	9.30	12.92	14.50	10.87
TC1800000921.mm.1	Vmn1r-ps152	13.77	11.53	13.70	18.07	17.87	14.74
TC0200000874.mm.1	Gm13534	31.09	31.12	31.25	43.03	42.64	33.65
TC0200001486.mm.1	Gm22957	34.71	44.97	44.29	49.85	66.77	59.94
TC0200003831.mm.1		13.06	13.17	12.00	18.01	19.98	14.30
TC1400001877.mm.1	Gm3287; RP23-78F6.4	20.26	18.54	18.49	21.14	28.23	26.45
TC0900002116.mm.1		12.66	12.47	14.91	16.54	17.24	15.14
TC1400001461.mm.1		16.47	17.09	16.03	18.22	24.60	22.42
TC1700002007.mm.1	Gm4246	153.74	159.25	155.84	237.72	172.38	218.99
TC0700004004.mm.1		18.61	15.61	13.76	25.28	20.23	32.35
TC0900000389.mm.1	Olfr892-ps1	9.16	10.11	11.21	16.97	15.78	11.76
TC0X00000374.mm.1		13.00	11.49	11.18	13.63	20.04	19.26
TC1500000311.mm.1	AU022793	72.68	70.82	63.36	113.49	83.16	142.66

TC0400003053.mm.1	Gm22605	9.28	9.85	10.92	11.36	13.88	15.51
TC0X00000711.mm.1	Mir7092	24.37	31.88	28.11	39.48	50.74	83.76
TC1900001655.mm.1		10.32	9.62	9.15	16.30	13.75	11.12
TC1600000724.mm.1		11.03	14.87	17.07	23.39	23.37	18.67
TC0200001440.mm.1	Mir7222	10.98	9.93	12.75	13.08	14.79	14.82
TC1400002654.mm.1		23.26	36.39	27.33	55.19	50.30	37.69
TC1000000535.mm.1		10.34	9.10	10.95	14.92	14.38	11.42
TC0200004517.mm.1	4930583P06Rik	6.94	7.13	7.13	12.07	9.55	8.18
TC1300002731.mm.1	LOC100861680	35.38	25.64	42.30	56.05	65.34	47.11
TC0700003352.mm.1		8.37	7.79	7.81	11.18	10.27	8.68
TC0800001248.mm.1		23.23	26.62	20.62	36.82	27.60	32.77
TC1500001310.mm.1		14.46	14.29	17.25	29.14	18.83	21.96
TC0500001040.mm.1		116.63	83.32	87.01	147.56	118.01	155.48
TC0200001037.mm.1		14.28	15.09	14.95	22.20	16.25	22.37
TC0100001521.mm.1		21.89	18.33	26.67	28.33	31.59	32.14
TC0100003741.mm.1	Gm23780	20.44	28.84	23.86	35.06	31.49	31.53
TC1600001685.mm.1		8.77	8.60	11.25	11.48	12.77	13.68
TC1700000679.mm.1	Gm25128	1533.87	1474.95	1993.11	5755.66	2711.46	2772.56
TC0200003867.mm.1	Gm13710	570.16	583.92	496.21	888.98	627.36	930.73
TC1600000289.mm.1	Snord66	41.08	30.99	40.91	72.73	46.42	60.28
TC0300001859.mm.1		15.39	16.99	17.15	24.42	18.19	24.21
TC1100002294.mm.1		16.23	15.13	15.45	18.45	20.39	27.01
TC0500001940.mm.1	Gm7332	737.17	713.12	597.39	1114.33	805.90	1291.55
TC1700002725.mm.1		9.89	10.01	13.93	13.98	18.28	18.10
TC0800003210.mm.1		11.44	12.11	12.44	16.27	21.76	14.28
TC0500000016.mm.1	Gm26238	22.18	23.02	26.86	27.56	40.64	37.03
TC0600001927.mm.1		17.17	14.37	14.96	20.68	20.27	17.21
TC0500000469.mm.1	Gm16014; AC164004.2	19.06	13.59	13.25	20.89	27.29	20.88
TC1600000886.mm.1		9.60	9.97	11.75	14.14	16.42	12.08
TC0500000127.mm.1		11.01	12.07	12.58	14.05	22.98	17.95
TC0700000185.mm.1	Gm24701	39.43	40.40	32.63	46.77	76.26	55.32
TC1200002592.mm.1	Ighv3-8	8.98	8.56	7.47	12.73	10.97	9.53
TC0600001607.mm.1	Gm25136	44.65	39.50	34.35	52.23	46.04	52.10
TC0500002545.mm.1		11.05	13.24	12.17	16.26	17.08	13.50
TC0700001744.mm.1	1700025J12Rik	12.59	13.46	15.70	22.47	20.58	15.97
TC0X00001184.mm.1		7.17	9.52	9.10	10.17	12.39	12.50
TC1500001443.mm.1		10.25	11.34	10.28	13.40	12.12	16.45
TC0200002951.mm.1	Gm26478	13.96	12.98	16.42	17.72	26.89	20.37
TC0800001890.mm.1		10.51	13.15	10.50	15.21	19.93	31.40
TC0100002108.mm.1		19.46	21.63	19.57	22.37	29.16	25.49
TC0400001368.mm.1	Gm12883	13.04	11.93	13.73	17.47	19.79	14.39
TC1100001805.mm.1		28.92	28.02	26.28	38.18	36.78	29.89
TC0X00000511.mm.1	Gm14638	26.55	18.65	17.50	27.85	30.70	37.69
TC0300002125.mm.1	Gm20691	280.76	235.85	247.74	322.95	328.86	281.10
TC1900000514.mm.1		37.42	34.71	28.60	64.06	43.56	45.22
