# Supplementary material for: MicroRNA-26b-5p Inhibits Mouse Liver Fibrogenesis and Angiogenesis by Targeting PDGF Receptor-Beta
Source: Mol Ther Nucleic Acids. 2019 Feb 26;16:206–17. doi: 10.1016/j.omtn.2019.02.014 (PMC6426711; doi:10.1016/j.omtn.2019.02.014)
Supplement: Table S2. The Preliminary Probe Signal Values of Coding Genes by Microarray Analysis in TGF-b1-Treated BMSCs with or without miR-26b-5p Mimics [file mmc2.pdf]

MicroRNA-26b-5p Inhibits Mouse Liver Fibrogenesis and Angiogenesis by Targeting PDGF Receptor-beta

Le Yang#, Chengbin Dong#, Jingjing Yang, Lin Yang, Na Chang, Changbo Qi, Liying Li*

Supplementary table 2. The preliminary probe signal values of coding genes by microarray analysis in TGF-b1-treated BMSCs with or without miR-26b-5p mimics

Probe Set ID	Gene Symbol	Control mimics			miR-26b-5p mimics		
		1	2	3	4	5	6
TC1500000300.mm.1	Cthrc1	4090.91	4093.06	3812.53	1231.72	1229.18	1240.54
TC0200004309.mm.1	Fibin	103.45	108.05	104.45	44.27	43.07	44.92
TC0400002812.mm.1	Tnc	128077.50	130825.50	124266.10	50032.72	47542.00	50120.11
TC0100002266.mm.1	Uxs1	516.98	514.46	502.86	314.90	312.60	307.37
TC0600002553.mm.1	Sema4f	38.49	38.52	38.22	24.91	24.70	24.00
TC0600002487.mm.1	Vamp5	534.22	524.22	530.17	388.50	398.94	390.24
TC0X00001271.mm.1	Srpx2	27591.62	28517.91	26865.28	10383.21	9986.66	11037.69
TC0900001175.mm.1	Plod2	5763.61	5831.73	5318.29	1594.69	1765.21	1737.97
TC0300000710.mm.1	Crabp2	1948.52	1904.05	1729.05	465.29	519.46	485.05
TC1300002143.mm.1	Ctla2a	313.22	297.50	313.29	142.73	151.85	141.85
TC0800002113.mm.1	Mtus1	83.91	84.36	81.89	55.27	54.72	57.05
TC1300002564.mm.1	Mast4	68.20	68.02	66.38	51.59	51.04	50.40
TC0800001480.mm.1	Gm22	4427.76	4105.78	4072.15	1283.83	1451.41	1383.08
TC1700001484.mm.1	Thbs2	91825.34	92438.75	85682.51	30733.92	29060.50	32923.16
TC0900002735.mm.1	Col12a1	18779.30	19951.68	18074.78	8166.34	7707.84	8175.93
TC1800001193.mm.1	Hbegf	105.24	103.90	100.56	51.27	53.11	55.58
TC1000001895.mm.1	Enpp3	130.30	117.36	126.62	58.07	56.29	57.04
TC0300003041.mm.1	Bmpr1b	95.64	92.28	98.18	49.05	50.68	52.45
TC0900002254.mm.1	Ncam1	1018.66	1056.63	938.02	289.06	267.91	306.78
TC0800001121.mm.1	Ccl17	1684.17	1645.80	1678.67	408.94	438.11	494.93
TC0800002447.mm.1	Cyp4f18	36.51	35.60	35.58	20.82	19.87	21.42
TC0100000064.mm.1	Sulf1	7919.29	7927.90	7423.10	2723.62	2624.05	2990.33
TC1300002460.mm.1	F2rl1	28.28	27.41	26.29	15.92	16.21	16.47
TC0700003437.mm.1	Aldh1a3	295.16	265.41	264.53	100.77	105.45	109.52
TC0200004133.mm.1	Creb3l1	6793.36	6150.19	6096.24	2844.90	2790.12	2695.67
TC0200004525.mm.1	Fbn1	21423.53	22628.03	20712.25	10014.86	9604.35	10462.48
TC1600000553.mm.1	Cd80	2050.93	2140.06	2013.10	1080.20	1018.51	1107.75
TC0300001173.mm.1	Col11a1	123.76	134.72	122.26	60.89	58.56	56.92
TC1300002079.mm.1	Cxcl14	17150.01	14873.23	17004.40	5469.98	5491.03	5836.29
TC0600002089.mm.1	Creb3l2	1426.86	1443.59	1339.00	750.13	737.26	782.58
TC1700000168.mm.1	Smoc2	639.54	588.19	554.73	213.52	226.04	230.74
TC0100001338.mm.1	Ptgs2	64190.78	64012.60	56967.92	16961.35	16595.86	19301.96
TC0100003240.mm.1	Prg4	697.94	642.85	658.23	188.32	168.90	205.83
TC0800000286.mm.1	Plat	504.32	504.26	457.45	163.05	174.39	186.61
TC1500000676.mm.1	Kdelr3	16995.82	17600.57	16572.20	7989.62	7305.99	8201.86
TC0700004431.mm.1	Adam12	424.78	412.15	383.42	195.58	193.76	206.91
TC1000001894.mm.1	Enpp1	9468.10	9274.72	8690.00	3887.84	3428.64	3845.89
TC0400001990.mm.1	Errfi1	1291.72	1300.56	1178.41	651.47	617.26	645.59
TC1200000142.mm.1	Odc1	1762.39	1728.13	1657.79	1114.66	1120.59	1161.05
TC0500001732.mm.1	Kdelr2	342948.90	331388.80	335565.20	203443.90	216513.60	218673.80
TC1300002392.mm.1	Vcan	1109.47	1238.32	1099.05	380.97	348.51	408.95
TC1000000817.mm.1	Fstl3	189.28	175.96	165.94	85.81	81.82	84.61
TC1200001500.mm.1	Gm20472; Gm4425	61.60	63.14	64.28	49.74	49.29	48.76
TC0900001254.mm.1	Dzip1l	90.07	88.45	87.50	66.21	62.69	63.98
TC0100001112.mm.1	Gpr39	87.42	79.83	81.52	50.26	49.04	50.22
TC0900001236.mm.1	Copb2	3743.67	3480.88	3481.15	2297.58	2271.64	2316.71
TC1600001655.mm.1	Cd200	1443.65	1338.64	1259.01	466.54	429.06	503.35
TC0700004593.mm.1	Tnfrsf23	3333.94	3655.37	3433.14	1420.27	1452.02	1616.50
TC1000000508.mm.1	Fabp7	1631.36	1700.46	1584.45	825.04	915.72	861.85
TC0600000711.mm.1	Abcg2	219.40	215.99	198.99	120.63	122.56	117.71
TC0300001482.mm.1	Ddah1	53946.30	54835.57	48588.68	24375.40	26080.29	24958.60
TC0900001846.mm.1	Col5a3	56.11	59.84	57.19	34.30	36.85	35.26
TC0X00002676.mm.1	Vsig4	5781.56	5668.73	4855.90	1644.44	1450.27	1385.16
TC1000001027.mm.1	Tmem263	298.88	287.23	277.27	136.44	135.29	151.20
TC1300002287.mm.1	Adamts16	53.89	51.60	49.21	28.46	30.21	28.18
TC1000003163.mm.1	Tmem198b	387.67	372.43	352.10	227.88	222.66	229.29
TC1600001752.mm.1	Tmem45a	835.76	823.26	883.56	434.63	388.41	384.86
TC0900000285.mm.1	Arhgap32	46.87	45.82	45.63	34.07	35.65	35.42
TC1400002723.mm.1	Uggt2	119.53	121.34	112.12	71.55	70.46	74.46
TC0400003629.mm.1	Serinc2	420.54	392.72	356.65	159.09	170.53	162.44
TC1600001395.mm.1	Masp1	762.91	718.25	666.08	365.08	354.67	369.88
TC0600003412.mm.1	Bcat1	365.91	342.47	340.11	201.43	182.90	192.94
TC1900000725.mm.1	Sfr1	13292.05	13644.59	13299.10	8762.40	9402.55	9282.85
TC0X00000065.mm.1	Praf2	8901.72	8816.79	8179.66	5161.67	5312.60	5010.31

TC0X00003038.mm.1	Morf4l2	309.87	309.31	300.60	241.66	233.55	242.11
TC1200000253.mm.1	Pxdn	1100.84	1016.85	951.74	404.04	394.38	444.51
TC0600000124.mm.1	Tes	247.89	266.37	253.17	178.47	174.00	173.04
TC0100001218.mm.1	Fmod	10201.62	9975.40	9238.85	5761.09	5472.83	5420.30
TC1000001284.mm.1	Csrp2	4639.46	4786.05	4255.64	1345.65	1449.99	1658.08
TC0100001234.mm.1	Kdm5b	186.13	185.24	182.51	125.28	114.26	119.15
TC0400003475.mm.1	Mfsd2a	108.86	109.65	102.87	72.93	73.89	75.78
TC0100002544.mm.1	Fn1	407862.70	408064.40	402772.60	312748.70	293920.30	304051.60
TC0100002736.mm.1	Htr2b	639.36	567.68	527.20	178.30	173.22	201.41
TC0400000893.mm.1	Gm13285; Gm13290; Gm13289	30.37	31.71	31.84	23.74	22.95	23.64
TC0500000173.mm.1	Lrrc17	190.11	176.95	165.79	61.35	52.88	64.38
TC1200001813.mm.1	Sec23a	7009.01	7384.85	6343.51	2881.80	2842.28	3153.21
TC0400002962.mm.1	Bnc2	284.30	294.09	264.21	121.13	111.49	130.32
TC1300002426.mm.1	Thbs4	1842.60	1663.65	1617.01	837.26	797.41	868.82
TC0300000627.mm.1	Fam198b	2723.02	2829.93	2436.22	1238.58	1150.57	1244.59
TC1500000086.mm.1	C1qtnf3	770.52	689.47	612.75	184.14	164.59	200.47
TC0700001252.mm.1	Nox4	120.37	122.57	111.32	62.07	55.85	61.60
TC1600001070.mm.1	Kcnj15	264.68	248.76	235.89	120.10	108.94	123.42
TC0600000010.mm.1	Col1a2	41996.37	44577.20	39308.51	16549.15	15032.70	17937.17
TC0200004511.mm.1	Gatm	97554.91	102259.30	88909.51	42744.93	39539.91	44825.32
TC0300003225.mm.1	Sirpb1a	767.31	849.51	712.05	311.99	314.56	341.75
TC0100002356.mm.1	Rftn2	134.46	131.38	119.44	72.62	69.41	72.63
TC1500001548.mm.1	Has2	739.80	879.57	866.97	290.25	304.11	329.71
TC1200001549.mm.1	Rnf144a	64.79	65.95	63.20	46.41	47.16	49.16
TC1100000526.mm.1	Gfpt2	349.43	353.61	307.46	165.37	152.51	162.86
TC1800000029.mm.1	Zeb1	730.93	719.40	641.94	369.14	359.35	379.63
TC0900002396.mm.1	Cd276	1505.68	1471.09	1364.77	941.86	916.99	924.45
TC0200002067.mm.1	Plcb4	86.69	88.31	89.14	65.01	62.16	66.51
TC1900001527.mm.1	Loxl4	98.68	95.36	87.14	54.00	51.53	51.39
TC1600001764.mm.1	Col8a1	1198.01	1216.22	1099.39	530.40	507.13	587.45
TC0400000736.mm.1	Pappa	5203.13	5461.28	4689.83	1437.86	1336.85	1715.09
TC1000000246.mm.1	Ptprk	185.11	186.24	173.76	97.31	94.37	106.49
TC1800001479.mm.1	Ccbe1	83.18	80.03	72.99	44.21	45.52	44.12
TC0500001327.mm.1	Tbx3	47.53	45.59	47.62	36.71	37.52	37.64
TC0300002295.mm.1	Bcan	38.01	36.98	35.98	28.19	27.28	28.38
TC1500001835.mm.1	C1qtnf6	256.69	250.09	225.67	101.36	96.53	113.10
TC0100003754.mm.1	Tgfb2	6044.47	5782.24	5440.03	2294.20	1809.62	1980.98
TC1100001448.mm.1	Col1a1	142831.90	146443.30	138341.80	71931.80	68046.87	79487.30
TC0300002173.mm.1	Golim4	471.51	491.59	473.49	281.94	299.79	314.47
TC0500003694.mm.1	Stard13	142.28	146.52	137.55	77.82	77.98	86.96
TC0900000226.mm.1	Bmper	149.54	155.03	147.67	83.42	74.03	84.91
TC1200002082.mm.1	Ltbp2	3927.70	3788.79	3258.76	1134.86	986.92	1234.12
TC0800000483.mm.1	Pdgfrl	223.16	221.03	189.61	102.95	102.18	101.17
TC1500000395.mm.1	Col14a1	167.31	180.41	162.15	102.25	103.92	107.90
TC0600003002.mm.1	Zfp9	229.81	236.39	233.06	110.48	94.07	113.01
TC0X00003066.mm.1	Morc4	104.16	108.22	98.59	70.79	70.45	71.96
TC0600001384.mm.1	Mfap5	73475.43	73970.75	68403.70	34203.48	37112.41	39656.89
TC0800000321.mm.1	Fgfr1	1177.44	1174.79	1033.14	571.66	544.57	596.81
TC0900000720.mm.1	Sema7a	1455.65	1293.63	1165.09	396.26	336.67	418.02
TC1600000760.mm.1	Dcbld2	627.00	614.82	596.37	351.03	321.95	369.37
TC0600001653.mm.1	Plekha5	80.10	77.08	74.42	51.71	52.79	55.01
TC0500000842.mm.1	Ppbp	426.09	411.84	368.77	118.71	93.15	122.42
TC1400000976.mm.1	Loxl2; Mir6950; Gm21451	6157.94	5989.28	5225.57	2221.93	2245.98	2559.61
TC0700002477.mm.1	Fosb	54.97	53.46	56.11	37.09	40.11	38.39
TC1600000536.mm.1	Fstl1	21750.00	22496.39	21310.10	11407.84	11914.93	13135.35
TC0900002541.mm.1	Tpm1	4195.03	4078.09	3893.93	2285.87	2122.80	2422.08
TC0600000914.mm.1	Loxl3	10240.40	10395.13	8638.36	4117.95	3905.65	4300.69
TC0700002495.mm.1	Apoc2; Apoc4	61.80	63.84	61.49	44.27	40.50	43.17
TC1300002540.mm.1	Cdk7	329.10	342.15	314.92	232.88	232.26	223.62
TC0500003722.mm.1	Medag	248.06	242.02	202.25	79.75	79.61	90.70
TC0700003804.mm.1	Slco2b1	173.99	163.96	152.62	98.40	96.60	99.43
TC0800001599.mm.1	Itgb1	31294.19	29423.88	29012.91	19717.47	19184.03	20622.34
TC0300003226.mm.1	Gm5150	389.30	338.77	367.54	201.78	213.22	202.62
TC0600001029.mm.1	Gata2	80.32	82.68	76.58	57.75	59.02	59.29
TC0X00000218.mm.1	Timp1	222235.30	205616.90	214167.50	145522.30	133826.70	139971.00
TC0200000478.mm.1	Col5a1	470.58	450.42	408.02	193.05	159.45	182.45
TC0100001373.mm.1	Nmnat2	73.34	73.42	76.47	53.59	56.13	56.89
TC0600002574.mm.1	Actg2	88376.29	82114.52	81484.15	57955.10	55134.24	54549.74
TC0200005291.mm.1	Pmepa1	7280.56	6823.37	6663.46	2999.79	2439.94	2951.45
TC0100000343.mm.1	Col3a1	4364.70	4774.14	3941.62	1211.84	949.93	1269.71

TC0200000603.mm.1	Angptl2	1158.87	1086.70	1094.49	636.24	562.76	641.68
TC1400002785.mm.1	Gm2897; Gm3005	71.20	70.86	68.36	56.43	54.98	57.24
TC1100003345.mm.1	Myo1d	1082.30	1158.71	1019.44	595.73	618.51	649.01
TC0200003689.mm.1	Stk39	144.09	143.86	132.08	97.66	98.05	97.26
TC0100001692.mm.1	Kif26b	173.07	168.29	162.25	96.45	82.22	91.09
TC0900003204.mm.1	Rbms3	369.50	372.93	328.38	199.64	198.67	211.12
TC1300000273.mm.1	Cmah	204.96	226.42	188.93	102.27	104.01	107.93
TC1900001266.mm.1	Mamdc2	467.33	442.21	398.72	169.68	148.48	183.70
TC0800000588.mm.1	Asb5	53.80	48.51	50.91	33.08	34.25	34.65
TC0700004288.mm.1	Nupr1	4411.94	4378.86	4476.51	2977.83	3029.03	3266.41
TC0700004403.mm.1	Cpxm2	888.28	885.44	772.00	309.68	310.84	373.69
TC1200002103.mm.1	Tgfb3	29174.87	29770.60	26897.07	11708.85	10118.58	12787.35
TC1500002162.mm.1	Col2a1	92.91	93.57	89.52	66.19	62.68	68.05
TC1100003688.mm.1	Nr1d1	600.35	566.56	514.49	314.25	301.01	318.85
TC0500001912.mm.1	Steap2	199.46	205.70	195.45	120.56	124.39	135.41
TC1200001990.mm.1	Actn1	29283.57	28354.02	26264.23	18732.05	18800.25	18995.62
TC1300000691.mm.1	Tgfbi	37815.79	35861.73	35572.34	21686.48	23022.38	24302.08
TC1400000368.mm.1	Anxa8	5775.57	5391.04	4662.53	1812.09	1488.44	1843.26
TC1300000113.mm.1	Inhba	2422.97	2062.44	2144.62	857.20	935.20	1025.97
TC0300001674.mm.1	Sirpb1a; Sirpb1b	461.11	394.35	383.77	192.72	190.60	205.15
TC1200000069.mm.1	Sdc1	133.09	128.98	121.93	90.42	86.47	90.79
TC0500000387.mm.1	Rgs12	57.44	54.91	53.25	39.97	40.84	41.67
TC0200000651.mm.1	Ptgs1	159.40	153.32	139.10	88.44	80.88	84.41
TC0300000408.mm.1	Postn	70892.34	70066.14	68046.42	29157.92	23044.96	29137.50
TC0900000824.mm.1	Dennd4a	906.32	883.81	815.58	427.86	385.99	459.76
TC0100002833.mm.1	Hdlbp	31294.85	32102.27	30678.02	20743.23	18945.14	21158.59
TC0X00000051.mm.1	Shroom4	113.41	122.41	106.75	60.20	51.55	53.76
TC1100001624.mm.1	Fkbp10	15984.18	15990.01	14049.95	7990.68	6969.25	7697.47
TC0100000895.mm.1	Fam132b	327.88	330.99	273.19	110.62	104.13	126.34
TC0400000654.mm.1	Ugcg	3157.53	2972.40	2687.38	1536.77	1402.57	1549.08
TC1000002583.mm.1	Aldh1l2	294.13	291.96	256.64	146.74	148.91	160.17
TC0400000602.mm.1	Zfp462	102.53	103.22	97.21	63.93	62.80	69.56
TC1800000646.mm.1	Apcdd1	50.48	51.58	50.74	35.66	33.35	37.01
TC1200000951.mm.1	Flrt2	310.34	282.96	249.25	101.15	83.92	103.01
TC0900002455.mm.1	Glce; Mir5133	199.66	198.92	182.91	133.74	125.25	129.23
TC1100003406.mm.1	Dusp14	197.12	182.71	181.96	117.21	125.24	126.86
TC0800002307.mm.1	Tll1	233.68	232.25	184.09	77.19	80.92	85.16
TC0900002542.mm.1	Tln2	333.93	340.76	310.60	180.24	156.84	181.20
TC1300002285.mm.1	Ice1	824.75	821.19	745.88	329.15	346.99	402.27
TC0300000752.mm.1	Thbs3	64.59	57.63	59.78	39.49	40.50	40.88
TC0800001143.mm.1	Ndrg4	124.69	117.55	114.82	80.13	77.92	84.21
TC0500003431.mm.1	Serpine1	125834.00	128730.90	121254.80	74218.59	73232.30	82733.80
TC1800001269.mm.1	Dpysl3	514.12	496.90	443.13	203.28	178.68	220.87
TC1000000171.mm.1	Sgk1	661.88	659.96	593.15	410.01	391.91	411.67
TC1300001722.mm.1	Sox4	130.37	129.70	136.95	98.78	100.94	104.14
TC1400001726.mm.1	Bmpr1a	1814.58	1762.23	1642.04	802.54	786.17	937.98
TC1000002240.mm.1	Adamts14	64.34	66.94	64.24	47.07	49.63	45.72
TC0800000814.mm.1	Tpm4	75797.85	79717.56	76821.34	60385.56	56657.41	59119.29
TC0800002349.mm.1	Csgalnact1	163.28	158.11	146.53	90.56	81.81	92.45
TC1300002270.mm.1	Adcy2	88.36	85.47	80.69	49.77	55.63	51.67
TC0900003248.mm.1	Cx3cr1	2104.79	1817.50	1664.09	854.93	818.82	819.13
TC1500001121.mm.1	Hoxc6	222.15	221.57	218.34	137.36	127.28	146.84
TC1100004222.mm.1	Pycr1	302.98	289.44	276.06	185.73	199.83	196.78
TC0100001330.mm.1	Brinp3	32.67	30.79	29.39	19.56	21.02	20.19
TC0700001646.mm.1	Parva	3858.60	3685.72	3492.12	2395.49	2553.92	2499.66
TC0400002018.mm.1	Gpr153	184.16	167.79	161.88	111.09	108.28	108.20
TC1500000791.mm.1	Prr5	96.83	92.17	93.07	74.09	69.52	71.03
TC1400000030.mm.1	Gm3239	74.24	74.36	79.42	58.48	58.31	59.19
TC0900000834.mm.1	Cilp	40.44	41.64	40.41	28.20	25.28	25.07
TC0500002254.mm.1	Sorcs2	101.04	90.79	87.79	48.93	47.87	53.60
TC0100000894.mm.1	Klhl30	97.15	98.25	84.03	52.86	50.62	51.58
TC0100000298.mm.1	Il1rl1	756.88	703.94	652.07	309.76	312.20	363.65
TC1000000531.mm.1	P4ha1	2289.76	2381.50	2083.98	1125.72	1104.45	1266.65
TC0600001035.mm.1	Mgll	82.21	80.94	78.58	59.18	57.74	53.92
TC1400000986.mm.1	Tnfrsf10b	762.48	741.90	751.10	371.48	389.69	446.60
TC0700001841.mm.1	Itgal	192.80	180.67	167.99	93.39	85.71	99.66
TC1100000161.mm.1	Egfr	1340.60	1335.59	1192.24	596.45	536.18	653.47
TC0600000487.mm.1	Arhgef5	67.34	66.21	62.76	48.66	50.48	48.12
TC0200001729.mm.1	Thbs1	658134.10	650853.60	638940.60	492675.50	451275.10	482743.60
TC0X00000963.mm.1	Dlg3	49.84	50.04	50.32	37.90	40.74	39.70
TC0900001732.mm.1	Arhgap42	278.45	265.36	245.45	170.23	160.40	168.27
TC0X00002982.mm.1	Pcdh19	980.62	1073.03	960.19	459.14	397.95	494.32

TC0900001056.mm.1	Cd109	797.89	796.25	689.22	323.65	262.34	319.34
TC0700002951.mm.1	Siglece	114.31	122.60	120.81	84.05	89.78	86.10
TC1400000599.mm.1	Arhgef40	51.97	51.62	50.52	40.17	40.61	42.61
TC1100004126.mm.1	Mxra7	755.00	771.65	663.83	376.85	339.31	387.40
TC0400000861.mm.1	Adamtsl1	32.03	33.64	32.05	22.49	24.09	24.28
TC1900001484.mm.1	Pdlim1	20495.22	20003.54	19414.24	16096.86	15393.11	15283.62
TC0600002861.mm.1	Pdzrn3	298.00	293.95	298.31	121.77	135.07	155.32
TC0100003507.mm.1	Uap1	149.16	143.95	135.58	103.61	98.34	99.40
TC1800001595.mm.1	Lipg	49.29	48.14	47.15	34.77	33.38	31.17
TC0300000711.mm.1	Nes	78.66	76.39	76.27	42.46	39.21	47.08
TC0500002252.mm.1	Htra3	832.07	755.93	708.43	369.95	323.13	382.35
TC0100003113.mm.1	Ppp1r12b	58.51	59.49	58.20	44.54	47.87	46.44
TC1100000015.mm.1	Selm	222.92	228.98	223.37	160.91	165.78	175.71
TC1700002162.mm.1	Vegfa	795.90	818.89	745.80	382.66	352.32	431.75
TC0500000084.mm.1	Sema3a	105.13	104.48	94.91	64.03	59.77	65.76
TC1600002039.mm.1	Tiam1	63.75	60.54	61.95	48.38	46.09	49.04
TC0700001064.mm.1	Rgma	47.24	48.13	47.05	37.21	37.45	39.54
TC0800000749.mm.1	Crlf1	1189.76	1025.64	970.30	453.43	376.82	445.69
TC0500001914.mm.1	Steap1	591.26	589.35	532.81	322.39	273.29	277.77
TC0400002782.mm.1	Zfp37	84.87	91.66	88.13	54.02	59.77	59.37
TC1600001548.mm.1	Pdia5	1631.00	1546.36	1407.62	660.53	703.70	797.30
TC1100003948.mm.1	Pitpnc1	187.74	190.06	183.48	129.62	131.24	142.26
TC1400001045.mm.1	Htr2a	787.69	725.28	607.86	259.17	238.90	286.33
TC1400002293.mm.1	Dock5	495.21	500.37	451.72	297.74	287.83	318.35
TC0800001984.mm.1	Nrg1	344.91	355.03	303.17	148.25	128.99	159.01
TC0400002549.mm.1	Gne	161.52	167.99	159.30	132.85	126.67	127.22
TC0600001019.mm.1	Copg1	2579.47	2475.35	2335.59	1618.64	1590.49	1725.38
TC1700002438.mm.1	Ptprm	128.63	122.16	115.79	78.07	73.71	81.71
TC0300001431.mm.1	Unc5c	79.21	77.86	71.80	48.20	49.94	52.60
TC1000000402.mm.1	Cd24a	1067.92	944.09	842.15	385.50	351.01	418.66
TC1100004003.mm.1	Abca5	84.78	85.70	80.25	62.88	61.55	65.21
TC0300000631.mm.1	Pdgfc	1239.68	1179.75	1076.82	557.35	549.65	645.00
TC0200002457.mm.1	Lpin3	58.75	55.52	56.17	40.90	43.17	43.86
TC1000000671.mm.1	Ccdc6	1546.85	1589.06	1585.81	1192.83	1252.28	1280.05
TC0900000976.mm.1	Nedd4	8994.98	8938.05	8809.70	7012.25	6663.56	7204.11
TC1500000090.mm.1	Adamts12	2342.71	2477.63	2122.17	1100.02	970.16	1179.64
TC0100003506.mm.1	Ddr2	1160.66	1218.07	1077.52	652.40	639.91	722.55
TC0X00002648.mm.1	Maged1	1921.18	1949.81	1817.76	1023.64	950.55	1144.78
TC0800001696.mm.1	Col4a1	96.75	96.48	89.60	64.73	60.17	66.08
TC0100002278.mm.1	Col5a2	1670.58	1816.46	1507.52	582.10	443.11	595.69
TC0X00002207.mm.1	Gpc4	8179.96	7931.57	6694.12	3228.67	3417.72	3744.76
TC0500000305.mm.1	Emilin1	128.94	127.93	118.06	78.00	68.99	77.93
TC1100002679.mm.1	Zfp354c	207.28	179.53	181.39	105.91	112.48	114.25
TC1500001285.mm.1	Fbxl7	76.74	71.94	77.20	54.95	54.35	57.73
TC1600000415.mm.1	Dlg1	762.56	779.20	734.46	561.09	564.54	598.66
TC1700002181.mm.1	Ptk7	552.52	515.10	441.69	229.16	216.58	245.63
TC0700002217.mm.1	Peg3	28.35	28.91	29.29	19.40	20.75	21.61
TC1600000834.mm.1	Robo1	109.25	105.77	100.70	70.20	63.25	70.48
TC0200000551.mm.1	Ass1; Gm5424	20924.05	20844.03	18537.75	13117.71	12948.04	12334.54
TC1400002791.mm.1	Gm3264; Gm3173	53.79	51.16	53.56	41.92	41.95	43.72
TC1000001626.mm.1	Itga7	51.63	53.68	48.94	36.84	33.72	35.21
TC0400002529.mm.1	Tpm2	1510.63	1416.71	1459.89	829.91	783.04	930.66
TC0600000128.mm.1	Cav1	2787.37	2616.10	2260.84	1258.24	1295.86	1361.01
TC0400002532.mm.1	Msmp	111.01	107.06	105.79	84.82	86.85	81.19
TC1300001209.mm.1	Pde4d; Mir1904	46.98	47.80	42.99	33.06	32.01	32.04
TC0100002534.mm.1	Ikzf2	61.37	68.34	59.08	40.60	40.20	40.68
TC1600001974.mm.1	Adamts1	2146.65	2082.01	1884.07	1241.41	1258.16	1343.04
TC0700001382.mm.1	Map6	383.32	355.84	339.88	238.13	249.63	248.62
TC1600000480.mm.1	Itgb5	20797.45	20487.22	19502.50	13819.38	14343.08	15203.11
TC0600002315.mm.1	Fkbp14	554.81	553.78	459.58	272.23	269.23	282.99
TC1000000211.mm.1	Moxd1	65.63	60.99	60.62	39.31	38.85	34.18
TC1400002788.mm.1	Gm5796; Gm3095; Gm8108	62.22	59.93	61.61	45.75	45.71	49.22
TC1600001643.mm.1	Boc	196.15	189.32	182.30	142.58	134.59	144.19
TC0100003478.mm.1	Uck2	5279.39	4935.49	4394.99	2666.94	2303.90	2417.04
TC0900001200.mm.1	Pcolce2	48.19	43.98	45.41	34.06	31.75	32.83
TC1400000290.mm.1	Wnt5a	168.02	168.72	142.47	82.52	73.06	83.67
TC1300001173.mm.1	Adamts6	672.09	851.13	646.67	213.76	200.38	262.64
TC0800001432.mm.1	Crispld2	43.07	42.97	44.24	30.11	33.36	32.24
TC0900000544.mm.1	Bace1	621.30	553.85	501.35	282.37	248.24	278.82
TC1500002318.mm.1	Itga5	17726.38	18252.60	16073.67	8284.90	7448.55	9317.33

TC0400000535.mm.1	Tmeff1	1035.96	926.01	985.50	473.93	424.82	535.27
TC1500000175.mm.1	Ank; Mir7117	1107.77	1059.10	962.89	520.54	480.34	583.50
TC1000001919.mm.1	Lama2	281.63	264.99	227.78	102.54	93.99	118.46
TC1100004215.mm.1	P4hb	19352.78	19072.86	18620.23	12746.16	12443.72	14042.49
TC1700001403.mm.1	Fndc1	45.01	43.66	39.48	23.70	26.45	26.28
TC0500002833.mm.1	Sec31a	6926.62	6675.00	6197.84	4511.56	4320.07	4663.12
TC1100003222.mm.1	Serpinf1	6663.32	6779.72	6305.07	4626.98	4299.18	4769.68
TC0600000374.mm.1	Ttc26	89.12	82.27	78.51	58.27	55.88	57.26
TC0600000229.mm.1	Calu	13234.46	12071.54	12356.32	8029.19	7463.27	8508.46
TC0900002040.mm.1	Pknox2	56.67	54.09	52.02	38.75	39.99	41.46
TC0700001644.mm.1	Mical2	837.22	793.77	763.42	503.53	457.94	527.70
TC0700000557.mm.1	Clip3	718.24	676.47	657.04	443.48	378.08	412.86
TC1300000566.mm.1	Aspn	3455.38	3875.18	3014.98	1073.56	738.69	1006.50
TC0700001640.mm.1	Usp47	643.17	666.02	611.97	459.48	430.33	469.90
TC0100003352.mm.1	Rabgap1l	118.21	118.63	110.86	87.93	90.58	92.25
TC1800001338.mm.1	Ppic	24942.11	23010.85	21837.89	13964.10	12038.98	13538.91
TC1900001131.mm.1	Ms4a7	86643.70	88180.56	83966.42	63801.80	59620.06	65912.24
TC1200000760.mm.1	Ttc9	89.70	81.95	76.01	42.11	36.56	44.15
TC1300002141.mm.1	Ctla2b	1132.91	983.29	1136.39	676.90	635.98	674.91
TC0300000990.mm.1	Tbx15	2596.38	2611.05	2182.69	1284.82	1401.06	1282.96
TC0600002178.mm.1	Tcaf1	94.48	96.23	86.02	65.64	64.47	61.99
TC1600000938.mm.1	Jam2	61.25	60.65	56.71	47.48	46.51	46.13
TC0400003568.mm.1	Gjb5	107.10	94.91	84.64	42.80	44.25	48.94
TC0600000948.mm.1	Dysf	431.36	404.91	380.03	244.29	225.29	259.18
TC1200001562.mm.1	Sox11	69.81	71.85	68.08	49.38	54.42	51.56
TC1400000071.mm.1	Flnb	2932.70	2740.11	2541.17	1526.30	1334.94	1580.38
TC0100003502.mm.1	Rgs4	436.97	375.84	381.58	227.48	198.40	225.69
TC0100002038.mm.1	Tram2	2870.07	2692.61	2436.89	1534.13	1388.86	1582.86
TC1100000041.mm.1	Lif	160.89	169.04	160.66	90.75	86.83	105.02
TC1400002794.mm.1	Gm3298	73.26	80.98	74.47	53.20	55.93	55.88
TC0600001740.mm.1	Ppfibp1	473.67	465.54	440.34	251.80	248.02	295.16
TC0900000336.mm.1	Cdon	112.23	107.03	106.16	66.56	75.12	75.19
TC0700001651.mm.1	Tead1	714.32	742.46	692.48	420.03	387.50	465.84
TC1500000489.mm.1	Wisp1	3853.81	3502.63	3098.01	1220.48	1232.07	1569.90
TC1500002159.mm.1	Vdr	259.56	236.26	198.23	100.66	108.33	110.06
TC1300002463.mm.1	Iqgap2	327.66	323.17	300.50	211.82	188.55	212.69
TC1100001748.mm.1	Mrc2	1749.83	1619.63	1361.24	686.60	623.77	744.62
TC0500000499.mm.1	Slit2	422.67	445.74	379.18	239.56	221.72	252.35
TC0800001077.mm.1	Mmp2	11414.29	10642.45	9006.57	5113.55	4927.31	5417.62
TC0X00003133.mm.1	Amot	98.07	92.25	85.72	59.29	55.04	60.97
TC0900002128.mm.1	Oaf	1874.47	1686.48	1650.98	890.26	769.99	958.87
TC1900000141.mm.1	Pla2g16	83.28	81.64	80.48	58.23	55.82	62.82
TC1500000897.mm.1	Lrrk2	222.93	233.33	197.64	119.97	111.30	128.94
TC0200001951.mm.1	Slc20a1	2682.81	2882.04	2579.17	1360.57	1279.92	1589.90
TC1100003966.mm.1	Prkca	198.80	191.52	185.11	121.03	106.81	125.92
TC0900000212.mm.1	Cnn1	12371.17	12137.00	10822.97	6808.01	7200.29	7662.16
TC1300000998.mm.1	Edil3	103.30	109.28	87.27	53.74	52.01	54.38
TC0700002171.mm.1	Il11	44.07	44.04	40.27	31.71	29.68	29.39
TC0100002305.mm.1	Nabp1	1153.86	1214.39	1036.05	435.50	322.17	448.81
TC0X00000122.mm.1	Sytl5	40.07	40.61	38.70	21.42	25.60	21.28
TC1100003993.mm.1	Fam20a	1127.16	1128.44	965.72	537.11	425.28	507.24
TC0400001329.mm.1	P3h1	461.73	425.81	380.99	234.34	199.79	224.71
TC1600001827.mm.1	Epha3	138.98	146.33	121.28	77.63	72.10	80.10
TC1000000215.mm.1	Ctgf	59955.32	57473.67	51685.11	31472.54	33496.51	35920.00
TC1900000205.mm.1	Ddb1	16988.42	16582.98	15773.35	11281.63	10829.59	12198.74
TC0X00003114.mm.1	Capn6	80.02	79.00	68.31	41.94	37.76	44.20
TC0X00003168.mm.1	Maged2	271.11	246.82	241.84	166.04	171.80	180.92
TC1700000092.mm.1	Pde10a	51.55	53.14	49.54	38.71	34.79	37.38
TC0700004134.mm.1	Copb1	4382.40	4438.13	4012.26	2771.09	2859.05	3072.62
TC1300000105.mm.1	Gli3	228.09	228.73	208.81	154.57	139.79	154.40
TC1600000797.mm.1	Vgll3	2361.71	2439.20	2137.88	1442.19	1520.06	1582.92
TC1100001730.mm.1	Itgb3	107.49	85.76	93.22	52.13	50.99	53.96
TC0400002918.mm.1	Mpdz	76.65	81.97	75.96	57.17	57.45	61.28
TC0300001117.mm.1	Slc16a4	109.96	117.01	105.63	54.05	52.28	65.48
TC1700002776.mm.1	Pram1	71.16	65.93	64.07	43.78	36.80	39.79
TC0600000661.mm.1	Gars	4874.55	4686.02	4565.86	3163.17	2942.97	3398.66
TC0700003743.mm.1	Ddias	157.57	175.05	155.28	112.19	117.28	115.10
TC0400003670.mm.1	Phactr4	606.96	585.79	590.04	464.71	414.98	425.38
TC1000000578.mm.1	Gm5424	23938.54	24395.98	20518.09	13905.45	14078.44	13082.18
TC0100003700.mm.1	Enah	749.25	709.42	765.84	560.68	524.19	569.97
TC1700001166.mm.1	Ltbp1	369.03	358.16	316.96	199.19	185.79	216.26
TC0600000459.mm.1	Zyx	3689.70	3368.89	3208.20	2393.80	2234.55	2305.22
TC0300002629.mm.1	Olfml3	166.63	158.59	156.50	103.99	118.77	111.01
TC0X00002983.mm.1	Tspan6	3325.08	3124.25	2884.27	1701.47	1786.09	1991.93

TC1800001334.mm.1	Lox	139929.60	136252.00	127740.70	87387.32	81391.66	94215.07
TC1900001437.mm.1	Ankrd1	7977.05	6799.29	5778.03	2943.49	2647.81	3005.23
TC1600001459.mm.1	Lrrc15	1395.77	1831.82	1183.93	454.31	387.87	470.51
TC0900002391.mm.1	Loxl1	43220.42	40497.06	37116.02	25643.93	23549.60	26254.29
TC0300000675.mm.1	Sh3d19	253.47	235.47	226.77	146.33	123.15	144.56
TC1100000464.mm.1	Adam19	2205.13	2079.07	1824.79	1239.21	1125.69	1205.44
TC0900001428.mm.1	Col7a1; Mir711	44.45	49.23	46.39	33.06	35.54	33.45
TC1500000503.mm.1	Khdrbs3	138.79	134.86	127.87	84.01	90.77	96.33
TC1700000523.mm.1	Glp1r	178.48	210.31	151.93	79.08	71.34	79.09
TC1900000467.mm.1	Papss2	1242.66	1197.22	1046.24	695.87	664.38	734.85
TC1300002520.mm.1	Map1b	320.45	304.58	289.11	188.49	178.65	208.85
TC0900000791.mm.1	Itga11	5105.34	4753.90	4205.58	2338.07	2044.19	2528.16
TC0100001703.mm.1	Cdc42bpa	348.06	386.80	322.38	200.33	183.35	212.03
TC0700003797.mm.1	Serpinh1	1283.21	1252.48	1025.69	604.46	613.03	656.50
TC1000002076.mm.1	Sobp	148.85	184.10	149.10	85.91	78.34	86.14
TC1200001302.mm.1	Esyt2	5969.34	6040.67	5231.42	3220.70	2928.50	3499.07
TC0100000338.mm.1	Gulp1	443.86	525.96	418.51	255.29	249.27	262.61
TC0100000541.mm.1	Ccnyl1	5048.05	5396.14	4583.71	3002.99	2873.00	3247.29
TC1100001304.mm.1	Tbx4	26.09	25.57	26.66	22.13	20.82	20.64
TC1700001798.mm.1	Btbd9	225.67	219.29	217.59	167.12	181.73	167.14
TC0600000714.mm.1	Herc3	117.67	110.41	107.63	84.31	83.40	88.56
TC1000002846.mm.1	Nav3	60.16	60.49	57.15	47.41	46.70	49.61
TC0700001186.mm.1	Adamtsl3	140.48	133.46	129.24	99.01	86.85	91.79
TC0100003119.mm.1	Lgr6	68.77	67.11	58.51	37.82	36.73	41.50
TC0900000200.mm.1	Carm1	818.40	827.96	802.15	662.54	597.70	633.60
TC1200001281.mm.1	Crip2	7055.31	6460.12	5988.87	4007.09	3402.39	3786.11
TC1600001236.mm.1	Pdxdc1	1197.36	1204.44	1156.11	832.53	828.58	927.15
TC0100002177.mm.1	Neurl3	849.72	755.46	752.04	303.68	321.28	410.82
TC0600000232.mm.1	Flnc	963.56	920.34	797.52	513.37	462.34	529.63
TC1300002725.mm.1	Parp8	1786.09	1801.87	1612.61	1026.19	913.53	1103.70
TC0600003005.mm.1	Cacna1c	171.03	171.16	162.86	106.18	99.78	119.01
TC0800002184.mm.1	Wwc2	379.57	389.00	355.51	236.62	223.34	262.38
TC1400001902.mm.1	Fermt2	4829.89	4680.77	4117.81	2596.78	2325.29	2762.60
TC1800001478.mm.1	Lman1	9422.95	9093.94	8306.39	5677.26	4917.02	5694.00
TC1000002458.mm.1	Syde1	1820.45	1893.73	1632.77	1238.10	1161.33	1208.61
TC0600001203.mm.1	Bhlhe40	285.95	263.12	266.50	168.02	131.96	142.93
TC0100003827.mm.1	Hhat	108.17	105.49	94.14	72.26	72.31	72.56
TC0500000602.mm.1	Fam114a1	1437.44	1393.63	1261.06	843.00	757.72	889.19
TC0X00002685.mm.1	Eda2r	210.80	208.34	178.98	117.14	100.22	115.55
TC1800001026.mm.1	Cdh2	303.65	290.74	277.98	191.28	172.22	202.46
TC1700001794.mm.1	Mdga1	90.22	80.58	77.21	55.74	50.64	53.67
TC1200000596.mm.1	Frmd6	370.01	342.93	322.05	216.13	183.28	211.70
TC0600000678.mm.1	Fkbp9	5872.90	5322.71	5275.03	3368.41	2869.43	3432.50
TC0200004251.mm.1	Prrg4	94.20	89.27	79.50	57.56	58.71	55.54
TC0800000653.mm.1	Sh3rf1	286.07	262.09	245.79	154.38	142.11	168.90
TC0300003077.mm.1	Sh3glb1	17729.10	18169.16	17303.66	14162.71	13445.08	14652.73
TC1400002783.mm.1	Gm2956	74.96	75.12	73.00	57.62	62.52	57.79
TC1000001949.mm.1	Hey2	38.92	38.86	39.48	25.51	25.95	29.53
TC1300002059.mm.1	Dbn1	450.27	475.75	411.99	303.79	282.34	309.24
TC1000000572.mm.1	Sar1a	9529.86	10109.78	9451.05	5944.01	6204.06	6991.11
TC0800000384.mm.1	Purg	76.24	77.23	77.62	56.33	56.42	62.54
TC1600000193.mm.1	Pkp2	88.38	92.06	75.29	52.88	49.06	49.89
TC0900001792.mm.1	Fat3	51.15	54.56	48.08	36.13	36.60	38.39
TC1600001922.mm.1	Nrip1	68.54	72.57	69.11	54.24	53.72	58.00
TC0300000228.mm.1	Usp13	41.33	41.59	38.43	29.68	31.79	31.62
TC0200001552.mm.1	Cd59a	2093.43	2017.01	1769.45	1124.36	1093.72	1258.68
TC0900002175.mm.1	Arcn1	1855.80	1997.40	1837.87	1445.50	1360.58	1478.13
TC1400001429.mm.1	Gm10128	57.19	52.16	55.05	41.04	43.17	43.44
TC0300000354.mm.1	Noct	103.26	103.33	100.87	71.30	64.44	75.80
TC1400002116.mm.1	Gzmd; Gzme	435.34	384.25	360.08	247.80	217.24	228.90
TC1100003240.mm.1	Nxn	1942.74	1843.26	1651.10	825.75	756.21	1001.39
TC0900002887.mm.1	Zbtb38; E030011O05Rik	235.10	239.26	224.33	145.26	132.10	160.89
TC0500000527.mm.1	Sod3	53.64	54.31	52.16	37.62	34.49	40.17
TC1600000439.mm.1	Ubxn7	230.82	235.41	251.19	183.21	170.73	185.82
TC0200003033.mm.1	Dnajc1	353.73	352.38	322.06	214.72	207.49	242.43
TC0100003830.mm.1	Sertad4	245.98	252.24	221.87	145.19	116.09	135.79
TC0200002549.mm.1	Slc2a10	104.45	93.36	89.73	62.29	66.76	65.64
TC1000000322.mm.1	Lama4	238.33	262.28	235.34	159.32	139.41	163.58
TC1300001242.mm.1	Plk2	2283.69	2391.45	1930.67	1096.56	967.42	1204.72
TC1000001743.mm.1	Adgb	45.16	49.55	47.98	27.41	32.92	28.63
TC0600000331.mm.1	Cald1	1136.17	1223.35	1076.03	740.26	714.60	814.13

TC1900000382.mm.1	Ptar1	284.38	299.10	265.03	180.25	154.12	182.57
TC1500000125.mm.1	Cdh10	29.57	32.17	30.68	24.07	23.17	21.76
TC0200000549.mm.1	Ncs1	413.16	390.38	369.83	245.53	217.48	261.94
TC0800000874.mm.1	Zfp827	143.32	141.95	137.02	101.12	89.95	104.25
TC1100003769.mm.1	Jup	216.91	196.83	180.20	127.15	110.38	113.93
TC1000001110.mm.1	Ikbip	384.61	407.51	362.19	266.30	232.71	262.61
TC0900001771.mm.1	Amotl1	141.79	143.55	137.82	96.83	80.30	93.78
TC1900000495.mm.1	Stambpl1	453.56	429.32	408.21	307.36	277.92	312.81
TC0100002789.mm.1	Col6a3	1177.80	1162.02	1084.06	658.07	533.23	679.14
TC1400000795.mm.1	Nfatc4	116.72	117.93	105.91	83.59	74.95	75.11
TC0900000033.mm.1	Casp12	274.95	274.29	243.15	188.63	173.56	186.74
TC1500002076.mm.1	Slc2a13	85.98	87.62	78.73	63.67	60.06	64.08
TC1700001783.mm.1	Mtch1	5022.57	4583.20	4373.86	3346.43	3138.05	3359.02
TC1200000263.mm.1	Lamb1	516.75	488.88	425.96	249.56	216.41	271.97
TC0700002988.mm.1	Rcn3	7843.79	7908.71	6122.55	3430.56	3207.09	3801.83
TC0600002079.mm.1	Ptn	34.03	30.69	33.10	24.81	22.51	22.49
TC0400002737.mm.1	Lpar1	113.42	100.39	97.68	73.40	68.31	71.12
TC1400002780.mm.1	Gm2897	65.51	57.68	60.39	45.57	43.21	45.68
TC0400003076.mm.1	Mysm1	119.97	127.26	126.72	100.44	101.13	106.26
TC0300001142.mm.1	Psrc1	217.29	212.01	200.43	151.70	144.47	163.04
TC1000002547.mm.1	Gna11	428.81	398.49	379.02	297.49	297.30	310.13
TC1500001819.mm.1	Myh9	4717.53	4677.00	4260.81	2921.89	2739.19	3228.33
TC1400002259.mm.1	Elp3	384.70	383.01	359.96	305.08	285.51	305.37
TC1500000351.mm.1	Eny2	279.16	278.62	263.36	219.56	213.94	230.12
TC0700002858.mm.1	Zfp507	63.95	64.25	62.48	50.40	46.89	52.22
TC1800001180.mm.1	Ecscr	487.98	497.95	488.65	377.83	355.94	401.55
TC0600001154.mm.1	Gxylt2	169.75	156.44	143.45	108.80	108.78	104.03
TC0600002024.mm.1	Plxna4	58.40	51.09	53.39	38.15	39.41	40.59
TC0600001602.mm.1	Gprc5a	104.00	93.47	85.78	51.71	38.47	42.90
TC0X00002482.mm.1	Pls3	793.12	748.94	700.73	575.86	549.54	543.32
TC0800001632.mm.1	Ctxn1	635.40	584.06	642.46	489.45	461.80	485.18
TC0700002417.mm.1	Ehd2	7028.39	6777.10	5768.98	3762.59	3468.03	4035.43
TC1200001241.mm.1	Mark3	394.83	415.75	373.98	289.02	307.05	307.48
TC1600000667.mm.1	Ccdc80	17734.15	16951.97	16556.82	11394.74	10722.23	12680.50
TC1800001012.mm.1	Kctd1	77.54	71.94	77.92	57.06	54.52	60.13
TC1800001296.mm.1	Ccdc112	42.75	44.41	45.80	34.67	33.99	36.87
TC0800001654.mm.1	Efnb2	302.84	276.75	260.73	156.93	140.10	175.91
TC0700000508.mm.1	Fbxo17	74.27	77.94	68.16	54.73	50.65	50.46
TC1100000880.mm.1	Myh10	110.89	112.34	109.07	79.85	71.37	83.67
TC0800002066.mm.1	Dlc1	71.43	68.80	63.53	45.94	41.96	48.64
TC0700001404.mm.1	P4ha3	776.00	867.63	710.65	443.09	404.45	485.59
TC1500001799.mm.1	Rbfox2	1540.97	1522.03	1348.65	954.67	862.74	1004.09
TC0200001551.mm.1	Cd59b	154.05	144.51	130.29	96.15	86.61	95.59
TC1800000714.mm.1	Ldlrad4	205.27	203.17	173.09	99.09	104.27	120.66
TC0X00001625.mm.1	Sh3kbp1	377.30	388.76	360.16	293.22	271.47	298.68
TC0X00000525.mm.1	Fhl1	195.79	187.41	163.06	114.39	121.64	121.02
TC0700001119.mm.1	Abhd2	2379.84	2149.81	1881.34	1080.28	1006.15	1236.47
TC0100002606.mm.1	Ptprn	214.49	212.11	178.64	117.78	108.24	126.73
TC0300002324.mm.1	Syt11	102.96	94.78	89.07	69.30	68.78	71.37
TC0300001081.mm.1	Rhoc	21182.24	20393.34	17809.67	13386.65	12233.03	13096.73
TC0100001252.mm.1	Csrp1	3599.69	3581.54	3259.17	2706.48	2777.52	2675.46
TC0900001293.mm.1	Amotl2	125.65	130.46	117.39	87.58	81.36	92.98
TC0200002811.mm.1	Arfgap1	223.23	231.19	202.14	159.62	164.14	163.79
TC0600002285.mm.1	Hoxa11	48.56	48.01	44.53	37.82	34.74	35.84
TC1900001464.mm.1	Rbp4	275.59	241.16	261.06	140.49	168.13	165.78
TC0400001708.mm.1	Hspg2	3217.26	3020.69	2532.53	1570.61	1409.37	1681.86
TC0200000783.mm.1	Mbd5	168.56	180.74	161.61	127.79	123.16	133.32
TC1100001115.mm.1	Ssh2	613.66	666.12	585.61	457.70	458.52	479.25
TC0X00000801.mm.1	Tmem47	1844.07	1912.10	1685.23	1148.89	1032.98	1242.62
TC1100001520.mm.1	Copz2	6657.39	6652.19	5691.04	3673.24	4234.25	3944.81
TC0900002676.mm.1	Mapk6	455.21	441.44	409.99	339.84	343.72	351.85
TC1000001868.mm.1	Eya4	561.65	578.77	481.10	329.14	296.87	344.79
TC0100001108.mm.1		367.32	403.50	381.55	297.24	261.99	271.79
TC0800000936.mm.1	Adgrl1	190.41	189.67	174.69	145.51	136.44	146.93
TC1400002795.mm.1	Gm8281	43.81	40.72	41.69	33.68	29.96	30.90
TC1200002089.mm.1	Pgf	133.96	115.38	102.87	68.49	68.14	67.50
TC0200004750.mm.1	Rrbp1	1531.46	1427.39	1341.88	909.31	783.40	948.71
TC1100002334.mm.1	Ehbp1	58.31	59.90	56.80	42.09	44.12	47.50
TC0400004055.mm.1	Spsb1	103.48	91.37	85.25	54.04	47.12	57.33
TC0100003350.mm.1	Tnn	520.87	494.01	451.21	264.29	203.48	268.68
TC1400002849.mm.1	Gm3696	39.81	38.07	38.92	30.27	30.03	32.95
TC1800000953.mm.1	Mkx	29.63	28.59	26.99	20.65	19.33	21.96
TC1800001397.mm.1	Smim3	412.00	350.23	345.68	205.90	213.76	241.64
TC0700001991.mm.1	Inpp5a	903.12	959.32	867.51	680.34	677.14	731.92

TC0X00001322.mm.1	Wbp5	5060.60	5012.56	5346.14	3455.06	2816.85	3388.75
TC1300000087.mm.1	Nid1	4169.98	4195.28	3515.87	2144.85	1817.96	2321.81
TC0400002095.mm.1	Mxra8	2296.94	2223.14	2044.54	1665.28	1681.73	1736.21
TC1300000565.mm.1	Ecm2	56.90	51.87	49.01	39.07	37.13	36.20
TC0800002953.mm.1	Bcar1	767.68	740.49	735.90	573.91	508.58	578.75
TC1000001531.mm.1	Lrig3	379.82	342.22	323.85	201.30	186.94	229.90
TC0600000362.mm.1	Trim24	226.90	223.24	214.16	155.27	142.26	168.45
TC1500000298.mm.1	Fzd6	86.51	74.79	68.65	45.95	48.93	45.50
TC1700000955.mm.1	Gm7334	2256.87	2209.37	2059.16	1324.08	1330.75	1575.58
TC0200003560.mm.1	Nr4a2	82.82	81.42	74.37	59.52	58.50	53.17
TC1600001528.mm.1	Kalrn	54.92	54.28	50.88	41.95	42.63	44.82
TC1400002848.mm.1	Gm10406	64.13	59.70	57.81	45.08	43.22	47.76
TC1100002301.mm.1	Slc1a4	326.24	302.26	274.90	193.62	168.87	197.58
TC0700000038.mm.1	Brsk1	59.96	55.42	52.06	41.98	38.56	40.22
TC1900001163.mm.1	Dtx4	248.63	271.61	256.29	173.82	147.04	177.88
TC0400001510.mm.1	Rnf19b	757.64	720.89	634.18	488.75	484.97	492.67
TC0100002690.mm.1	Pid1	126.15	134.80	134.69	104.32	99.59	109.17
TC0100003443.mm.1	Dcaf6	300.59	289.52	276.83	204.20	193.52	224.12
TC0400002854.mm.1	Tle1	93.17	100.12	87.92	69.18	66.77	72.35
TC0200003806.mm.1	Fkbp7	1955.34	2251.50	1933.16	1140.53	1088.13	1343.00
TC0100000364.mm.1	Tmeff2	58.24	58.22	48.54	36.08	35.43	36.86
TC0900002054.mm.1	Tbrg1	4475.15	4403.31	4013.50	2988.68	3008.49	3320.77
TC1500000647.mm.1	Kctd17	462.35	455.37	377.92	282.21	261.79	269.68
TC0100001821.mm.1	Ptpn14	466.59	455.31	414.84	318.29	310.09	343.66
TC1200002493.mm.1	Wdr60	51.93	49.82	51.55	43.81	39.85	40.98
TC0100001193.mm.1	Dstyk	104.71	106.24	94.03	77.87	73.45	77.33
TC0100002261.mm.1	Fhl2	380.02	360.00	343.57	289.17	271.78	262.04
TC1300002119.mm.1	Golm1	309.23	277.22	296.28	197.84	205.81	225.86
TC0900001900.mm.1	Acp5	271.39	253.28	240.12	184.06	186.91	199.90
TC0700003420.mm.1	Tjp1	325.99	332.22	305.33	213.91	169.25	202.22
TC0400002665.mm.1	Abca1	5357.50	5542.41	4853.74	3392.95	3248.23	3805.52
TC0300001958.mm.1	Setd7	294.60	297.72	246.27	155.06	137.31	171.49
TC0500003738.mm.1	Pcolce	1393.29	1321.24	1209.51	938.38	898.19	986.75
TC0200000518.mm.1	Sptan1	930.09	942.96	907.89	714.77	704.16	783.69
TC1100003063.mm.1	Chrnb1	46.98	47.11	42.40	36.15	35.07	33.96
TC0200001230.mm.1	Tnks1bp1	146.25	138.72	129.90	106.46	96.14	103.45
TC1400000263.mm.1	Arf4	780.08	740.87	647.01	460.83	450.57	500.97
TC1400000851.mm.1	Spata13	48.08	43.90	44.32	34.72	34.19	37.06
TC0700003434.mm.1	Lrrk1	240.17	232.07	214.31	151.84	136.87	164.52
TC1100000544.mm.1	Hnrnph1	681.18	725.90	663.49	487.57	484.68	546.00
TC0600000768.mm.1	Prdm5	94.55	98.86	91.90	73.39	71.84	79.19
TC1500000592.mm.1	Scx	81.67	76.95	68.81	48.20	53.31	52.34
TC0500000734.mm.1	Srp72	3481.58	3555.91	3188.20	2622.78	2469.64	2681.97
TC0600002799.mm.1	Uba3	2485.08	2442.35	2199.80	1820.56	1740.35	1843.31
TC0500003576.mm.1	Baiap2l1	180.41	194.07	171.95	132.22	110.36	118.31
TC1100003236.mm.1	Fam101b	985.72	888.79	767.85	394.54	352.04	482.20
TC1400001303.mm.1	Mbnl2	10721.98	11906.36	9422.36	6042.57	5714.56	6747.24
TC0100000916.mm.1	Gpc1	294.49	265.41	250.56	179.75	155.24	181.31
TC1700001805.mm.1	Glo1	536.39	534.07	438.29	313.03	324.05	327.42
TC0700002078.mm.1	Lsp1	223.14	236.39	207.56	150.36	127.74	151.95
TC0300001760.mm.1	Fndc3b	7365.57	7475.17	6539.93	4578.69	4117.46	4969.96
TC1300001500.mm.1	Cdk13	2126.99	2297.95	2046.48	1586.26	1691.94	1694.24
TC0100001351.mm.1	Ivns1abp	2150.33	2143.20	1925.02	1409.93	1331.67	1550.04
TC0700003552.mm.1	Mfge8	5346.14	5098.47	4631.22	3553.35	3144.90	3570.70
TC1600001319.mm.1	Gp1bb; Sept5	675.80	607.39	545.70	391.94	338.45	381.94
TC1900000186.mm.1	Fads3	15332.15	15479.40	13914.80	11774.47	11011.59	11713.65
TC1700000009.mm.1	Tiam2	79.48	73.88	70.77	56.09	47.86	51.56
TC1300001036.mm.1	Serinc5	512.58	498.05	437.00	282.81	298.59	336.40
TC0200002410.mm.1	Src	144.96	140.83	136.04	105.16	99.39	114.04
TC0600003264.mm.1	Klra2	160.10	154.27	134.94	101.61	103.49	91.48
TC1300002326.mm.1	Cast	1045.50	1046.38	994.48	802.82	720.78	819.42
TC1100002092.mm.1	Smtn	193.75	187.54	165.32	131.51	123.88	132.87
TC1200000110.mm.1	Nbas	438.24	436.64	378.79	304.65	281.69	300.70
TC0300002094.mm.1	Ssr3	2934.30	2742.71	2710.75	2074.46	1702.24	1870.22
TC0900000871.mm.1	Car12	33.34	29.28	29.76	23.42	23.26	21.84
TC0700004201.mm.1	Cdr2	412.12	420.05	362.24	278.25	277.38	298.34
TC0500002651.mm.1	Igfbp7	162211.50	156024.50	148128.20	113090.00	105817.30	122166.30
TC1300002061.mm.1	Pdlim7; Mir6945	542.36	520.86	439.99	323.24	286.43	320.01
TC0200004264.mm.1	Rcn1	718.02	683.16	591.77	372.81	346.18	430.63
TC1300002481.mm.1	Hexb	60879.91	56774.76	54980.69	39960.72	35256.10	42147.66
TC1500001181.mm.1	Gm2310	1085.58	1184.26	1341.27	631.61	701.29	772.82
TC1400002844.mm.1	Gm3591	89.38	79.61	80.50	60.04	50.12	55.90
TC0200003837.mm.1	Nckap1	700.98	648.64	620.83	435.44	387.35	470.05

TC0600001606.mm.1	Emp1	40625.39	39728.44	35870.41	24751.32	27482.08	28541.45
TC0800002300.mm.1	Palld	265.16	262.61	233.79	162.62	159.53	186.54
TC1000000937.mm.1	Chst11	2230.43	2065.27	1677.14	924.64	657.64	902.95
TC0300002023.mm.1	Tm4sf1	687.97	647.99	565.37	406.10	420.40	442.98
TC1500001656.mm.1	Sla	111.39	106.28	97.31	80.11	76.73	81.67
TC1500001511.mm.1	Ext1	2042.23	1886.94	1700.46	1131.42	1295.87	1253.25
TC0300000124.mm.1	Mtfr1	315.81	317.23	297.82	252.13	224.78	221.66
TC0500000534.mm.1	Anapc4	356.54	365.00	341.54	276.29	278.23	301.70
TC1900000749.mm.1	Mxi1	77.06	72.78	74.54	55.75	58.29	62.68
TC0900002944.mm.1	Ppp2r3a	64.60	66.76	60.29	50.64	50.24	53.08
TC0700004592.mm.1	Tnfrsf22	1215.63	1337.30	1272.79	878.12	840.50	986.68
TC1100002131.mm.1	Kremen1	249.90	223.89	219.45	163.11	142.82	165.12
TC1600000443.mm.1	Pcyt1a	312.60	285.71	315.10	242.46	220.30	236.50
TC1100001694.mm.1	Fzd2	314.44	324.95	283.65	215.05	190.73	221.79
TC1200001523.mm.1	Gm10330; LOC100861601	181.07	169.44	165.35	100.02	82.16	110.72
TC0200000741.mm.1	Arhgap15	75.01	80.07	69.45	56.40	58.51	55.64
TC0900001067.mm.1	Senp6	259.77	268.33	251.07	208.25	193.71	215.96
TC0300001521.mm.1	Adgrl4	65.70	70.65	62.45	50.33	46.92	51.77
TC1300001654.mm.1	Lrrc16a	93.18	92.94	84.12	64.33	68.05	71.45
TC1200000074.mm.1	Wdr35	141.88	147.90	119.20	86.56	84.87	91.84
TC0400000052.mm.1	Rab2a	37008.41	38034.65	37921.61	30146.61	26590.82	30117.57
TC0600001622.mm.1	Atf7ip	1958.95	2092.43	2145.27	1565.15	1549.61	1704.38
TC0600003601.mm.1	Foxp1	243.37	253.89	224.86	176.07	164.61	186.66
TC0400003581.mm.1	Azin2	94.73	98.24	86.89	74.29	68.41	71.17
TC1800000314.mm.1	Paip2	2314.10	2328.52	2360.44	1836.12	1590.04	1823.17
TC0200001881.mm.1	Dut	98.94	110.92	104.61	74.03	81.77	81.25
TC0700001684.mm.1	Nucb2	2119.12	2033.86	1966.07	1523.73	1353.61	1582.78
TC0300001229.mm.1	Alg14	1749.63	1730.83	1598.57	1170.00	1116.73	1315.27
TC0500002508.mm.1	Apbb2	1508.86	1524.01	1337.96	1115.21	1069.48	1129.66
TC1300001861.mm.1	Nedd9	301.79	286.69	289.51	173.17	143.98	194.90
TC0500001894.mm.1	Cyp51	495.04	496.02	437.54	287.41	270.78	336.60
TC0800000088.mm.1	Col4a2	95.55	95.43	85.20	66.50	62.68	71.10
TC1700001521.mm.1	Has1	61.25	62.53	61.26	44.02	51.11	46.14
TC1100002550.mm.1	Ccng1	83777.48	89389.80	70737.13	50196.70	48661.77	53952.15
TC0900000035.mm.1	Pdgfd	213.56	227.65	194.98	134.24	118.48	146.86
TC0900002613.mm.1	Tcf12	391.59	394.14	369.21	305.71	269.54	302.46
TC0900002921.mm.1	Mras	405.98	454.81	419.88	326.24	294.60	329.66
TC0200004219.mm.1	Cd44	2289.06	2276.46	2088.45	1578.36	1369.86	1636.98
TC0200003670.mm.1	Galnt3	76.64	77.08	70.00	54.12	55.66	60.37
TC0100001605.mm.1	Copa	18314.44	18186.16	17465.74	14412.24	13691.99	15342.57
TC0500001106.mm.1	Pcgf3	496.17	510.07	465.88	344.50	359.02	395.16
TC1100000494.mm.1	Trim7	46.77	42.87	47.13	34.31	37.09	33.26
TC1400000507.mm.1	Samd4	142.70	132.87	123.50	93.08	93.58	102.99
TC1000000224.mm.1	Epb41l2	1388.36	1505.02	1310.45	997.98	935.15	1070.11
TC1500001465.mm.1	Nudcd1	309.94	312.18	291.19	208.51	206.91	241.68
TC0100003096.mm.1	Atp2b4; Mir6903	213.89	225.85	198.97	153.17	135.61	158.28
TC1100003217.mm.1	Hic1	30.31	32.98	30.55	25.86	23.47	23.36
TC1100000081.mm.1	Aebp1	12656.21	12026.50	12029.09	10054.00	8709.39	9362.05
TC0X00000709.mm.1	Gdi1	919.67	886.91	866.60	632.16	645.86	730.19
TC1100000601.mm.1	Aff4	1965.77	2080.33	1863.92	1547.12	1413.08	1560.38
TC1900001130.mm.1	Ms4a14	793.97	1053.62	733.12	327.97	373.83	433.75
TC0700001660.mm.1	Arntl	284.04	275.25	266.78	199.28	162.19	194.06
TC0500003169.mm.1	Tpcn1	628.56	616.92	557.48	462.75	425.29	467.26
TC0900001295.mm.1	Ryk	4928.57	4902.76	4345.87	3143.06	2821.59	3425.14
TC0X00000169.mm.1	Gpr34	90.28	81.93	64.38	40.62	38.40	44.17
TC1500001570.mm.1	Fbxo32	127.08	111.36	110.30	86.48	82.79	76.07
TC1700002324.mm.1	Ptprs	285.99	289.03	252.13	187.19	174.71	205.00
TC1900000787.mm.1	Tcf7l2	251.74	254.45	230.91	198.83	180.90	194.77
TC0200002026.mm.1	Prnd; Prnp; Prn; PRND	142.85	146.68	146.21	125.91	112.91	113.97
TC1100001891.mm.1	Cdr2l	466.11	462.42	403.55	201.09	240.02	278.62
TC1700000274.mm.1	Zfp52	706.61	780.56	708.66	396.58	310.41	444.62
TC0900001675.mm.1	Ccr5	997.26	966.92	845.75	680.09	665.30	703.45
TC1400002689.mm.1	Slitrk6	72.41	65.99	52.93	34.10	35.36	37.57
TC0X00001056.mm.1	Magee1	51.39	50.41	44.14	36.57	34.14	36.24
TC0700004171.mm.1	Syt17	29.21	27.81	26.10	22.81	21.05	21.78
TC0300000017.mm.1	Zfhx4	108.19	123.11	98.41	71.93	69.11	75.99
TC0500001724.mm.1	Fscn1	5563.23	5282.57	4673.22	3221.12	2600.07	3286.19
TC0100000244.mm.1	Cnnm4	187.75	186.63	180.44	157.59	148.41	139.35
TC0X00001383.mm.1	Mid2	104.66	101.21	87.53	67.46	71.53	69.91
TC0900000858.mm.1	Csnk1g1	345.29	384.67	333.72	260.53	235.41	265.63

TC0700002759.mm.1	Aplp1	76.01	83.81	67.36	49.12	47.38	52.88
TC0500002486.mm.1	Ugdh	581.61	651.38	528.98	404.36	387.09	416.99
TC0900001349.mm.1	Pcbp4	729.13	653.02	590.92	457.16	402.08	437.48
TC0200004107.mm.1	Slc39a13	328.90	310.19	277.40	223.43	203.88	223.17
TC0100000216.mm.1	Dst	428.16	454.24	406.09	289.45	274.95	329.67
TC1100003667.mm.1	Fbxl20	155.28	147.96	143.81	118.76	110.04	123.70
TC0800001078.mm.1	Lpcat2	901.94	981.98	818.76	626.11	629.38	674.33
TC1600000353.mm.1	Lpp	843.74	930.63	749.50	518.13	530.54	589.79
TC1400001315.mm.1	Farp1	903.98	828.04	733.91	547.56	476.55	552.21
TC0200001006.mm.1	Dhrs9	439.72	418.36	364.50	263.62	242.88	287.73
TC1000001091.mm.1	Gm4184	135758.00	128151.40	137639.20	105445.90	93825.63	107057.10
TC0500003054.mm.1	Ssh1	346.11	367.90	327.61	268.71	264.73	286.61
TC0X00001372.mm.1	Tbc1d8b	254.06	282.99	229.57	154.17	126.80	161.58
TC1100000720.mm.1	Wnt9a	54.03	57.72	46.38	34.16	36.97	34.76
TC0300002822.mm.1	Snx7	521.68	581.10	507.10	342.93	318.41	392.57
TC1400001517.mm.1	Gm2237	57.90	59.17	60.95	45.50	38.36	44.92
TC1000001138.mm.1	Ntn4	53.97	52.56	46.40	35.23	37.99	38.56
TC1000002729.mm.1	Nudt4	2830.89	2772.08	2670.68	2090.99	1857.15	2193.39
TC0400002449.mm.1	B4galt1	7791.45	7427.56	6981.04	4830.52	4654.41	5649.56
TC0200004512.mm.1	Slc30a4	359.58	337.40	336.25	208.27	221.10	261.23
TC0100000351.mm.1	Wdr75	930.58	901.07	824.47	681.91	631.13	702.38
TC1900001505.mm.1	Tm9sf3	4271.40	4247.48	4151.96	3223.64	2982.87	3500.83
TC1100000730.mm.1	Mprip	1034.32	960.11	930.29	728.86	681.54	782.15
TC1400001487.mm.1	Rarb	31.14	31.34	33.36	24.00	24.66	20.82
TC1800000564.mm.1	Gramd3	89.71	87.30	78.09	63.47	60.95	66.93
TC0100001615.mm.1	Slamf9	240.22	230.00	188.47	143.01	137.97	144.34
TC1300001781.mm.1	Tubb2b	1476.59	1645.55	1329.90	948.54	798.38	969.48
TC0300000923.mm.1	Otud7b	74.56	82.73	80.62	54.88	56.55	63.48
TC1200001704.mm.1	Prkd1	225.56	260.99	219.79	117.53	116.31	154.18
TC1000002594.mm.1	Ckap4	818.69	786.70	764.95	528.83	544.53	632.01
TC0700002644.mm.1	Axl	4197.23	4071.76	3711.62	3055.94	2740.66	3083.48
TC1000000301.mm.1	Frk	177.20	206.12	156.59	88.94	77.83	106.57
TC1600001750.mm.1	Tfg	1845.44	1926.88	1689.52	1479.60	1415.82	1392.82
TC1200001436.mm.1	Ddx1	6696.47	7202.69	6448.41	4819.32	4862.74	5475.03
TC0500003482.mm.1	Pdgfa	170.03	158.97	149.21	124.27	122.64	130.30
TC0900000550.mm.1	Sik3	668.84	659.34	597.54	516.34	470.72	505.82
TC0600001793.mm.1	Tfpi2	169.34	125.13	111.81	62.33	45.91	59.43
TC1000003094.mm.1	Mettl21b	97.89	91.42	82.10	65.25	55.86	62.74
TC1700002541.mm.1	Fam98a	408.79	436.77	442.34	305.30	264.50	324.80
TC1600001053.mm.1	Sim2	73.01	68.78	75.81	58.40	50.56	55.15
TC0400002945.mm.1	Ttc39b	425.33	448.58	390.39	275.98	263.63	318.23
TC0700003714.mm.1	Prss23	251.75	249.27	211.58	144.49	161.28	168.77
TC0200001681.mm.1	Fmn1	542.41	607.57	585.26	436.64	395.36	455.77
TC1600002136.mm.1	Brwd1	120.47	126.22	117.14	92.29	89.62	101.99
TC1200002138.mm.1	Gm5662	19.78	20.51	17.77	14.98	15.25	15.28
TC0600003185.mm.1	Tspan9	105.00	99.51	86.58	68.95	62.02	68.87
TC0100001190.mm.1	Nuak2	128.37	129.89	133.50	95.84	95.44	109.89
TC1700000752.mm.1	Zfp57	54.80	61.19	52.99	41.81	44.74	42.95
TC0900001845.mm.1	Olfm2	23.89	21.18	23.37	17.58	17.22	18.68
TC0900002037.mm.1	Stt3a	31236.15	27954.30	27019.23	21809.17	21813.52	22881.31
TC0300001232.mm.1	Cnn3	30592.94	39338.02	31691.41	14250.24	18496.06	19176.44
TC0800001928.mm.1	Hook3	397.03	437.45	398.91	292.62	258.00	313.35
TC0100001581.mm.1	Adamts4	439.66	455.22	332.51	163.00	137.96	211.98
TC1300002609.mm.1	Rnf180	140.45	147.85	136.53	108.32	100.27	116.40
TC0200001203.mm.1	Itgav	2433.93	2396.78	2127.35	1552.62	1420.36	1732.89
TC0500000856.mm.1	Ereg	205.07	191.82	169.27	111.33	102.24	130.59
TC0600001002.mm.1	Gfpt1	305.86	280.96	256.47	215.31	208.27	209.90
TC0900002320.mm.1	Dmxl2	474.12	494.66	436.49	333.73	350.17	377.43
TC0700001368.mm.1	Lrrc32	7554.83	7383.08	6043.65	4521.38	4724.18	4103.86
TC1500002186.mm.1	Fkbp11	97.50	86.44	85.54	63.35	56.85	67.74
TC1200001877.mm.1	Map4k5	507.12	538.41	451.77	333.20	296.59	359.53
TC0400000519.mm.1	Tgfbr1	1114.94	1256.21	1070.57	740.34	720.44	858.22
TC0500000868.mm.1	Uso1	2015.25	2058.35	1739.74	1229.38	1159.33	1408.77
TC0700004604.mm.1	Cttn	782.07	787.37	699.10	513.60	448.37	557.04
TC0300000210.mm.1	Skil	957.66	930.89	942.72	520.17	407.25	604.25
TC0100000393.mm.1	Coq10b	285.16	303.30	247.33	185.34	185.88	204.16
TC1000000386.mm.1	Sec63	1375.47	1406.30	1320.95	1008.94	837.82	1021.50
TC0100003477.mm.1	Uck2	249.99	195.90	182.06	93.05	62.67	97.27
TC0700001372.mm.1	Prkrir	139.59	132.88	148.17	104.74	104.34	116.30
TC1200000462.mm.1	Srp54b; Srp54c	7108.90	7641.20	7048.54	4216.86	4297.70	5408.49
TC1400002072.mm.1	Efs	71.85	69.47	63.72	54.94	47.75	49.85
TC0X00000916.mm.1	Msn	15162.98	15808.91	14954.33	11383.92	11775.08	13046.99
TC1400002487.mm.1	Rgcc	43.94	36.03	37.04	26.56	23.78	27.37

TC0800002589.mm.1	Nfix	2650.55	2672.87	2444.95	2097.61	1853.63	2056.31
TC1400001393.mm.1	Gm3500	65.86	60.87	62.14	46.95	48.75	53.25
TC1100002314.mm.1	Lgalsl	1337.10	1226.19	1237.22	909.39	809.60	986.15
TC1100000794.mm.1	Ttc19	226.42	240.44	191.17	143.97	148.43	153.84
TC0800001481.mm.1	Zfpm1	141.22	125.34	115.19	85.88	80.35	92.54
TC0X00003108.mm.1	Ammecr1	2239.69	2293.90	2069.13	1324.70	986.90	1377.81
TC0200005089.mm.1	Jph2	55.08	54.48	49.00	42.86	41.97	43.59
TC1000002580.mm.1	Slc41a2	2418.85	2418.87	2097.41	1596.27	1668.60	1793.55
TC0900000686.mm.1	Cspg4	145.40	142.27	127.32	80.99	89.05	102.13
TC1400000517.mm.1	Ktn1	100.46	115.54	110.34	77.88	75.30	86.14
TC0400002717.mm.1	Ptpn3	38.48	37.79	37.30	29.80	29.48	33.15
TC0600002127.mm.1	Braf	248.78	292.47	246.10	183.87	173.25	195.25
TC0600000850.mm.1	Ggcx	483.50	450.69	387.72	298.74	291.83	316.59
TC0900001903.mm.1	Zfp599	74.88	69.81	68.32	55.83	46.85	52.98
TC1100000440.mm.1	Rnf145	626.16	650.29	571.19	428.67	357.94	442.84
TC1100000831.mm.1	Hs3st3a1	48.38	50.98	44.59	36.62	35.46	31.26
TC0600002773.mm.1	Lrig1	91.16	75.68	75.73	57.21	54.79	58.66
TC1100002227.mm.1	Grb10	564.73	611.80	535.71	413.35	392.73	453.93
TC0900001232.mm.1	Rbp1	320.65	246.36	235.00	157.36	133.35	153.04
TC0300001255.mm.1	Sec24d	2863.42	2697.57	2373.55	1686.64	1537.74	1895.93
TC1100001361.mm.1	Vezf1	2061.12	1890.53	1834.16	1421.51	1329.69	1545.43
TC0700001669.mm.1	Pde3b	323.79	330.18	311.78	199.31	148.75	206.90
TC1300000362.mm.1	Serpinb6b	88.83	79.09	92.43	68.34	62.04	62.66
TC0700002148.mm.1	Cdc42ep5	247.69	229.60	188.93	147.94	132.02	136.77
TC1900000082.mm.1	Ltbp3	175.73	161.59	148.40	119.13	104.68	119.37
TC0500001615.mm.1	Plod3; Mir702	2965.65	2898.70	2548.13	2039.31	1919.66	2175.00
TC1900000154.mm.1	Stx5a; Mir6992	215.91	214.55	195.31	170.89	156.12	169.77
TC1500001876.mm.1	Ddx17	2221.04	2423.42	2102.41	1744.56	1580.02	1754.70
TC0200005471.mm.1	Ak1	1613.61	1760.13	1677.53	1060.58	1201.91	1309.36
TC0800000916.mm.1	Tbc1d9	963.71	908.26	870.00	643.18	621.04	736.80
TC0300001342.mm.1	Dkk2	235.54	211.41	188.29	147.73	124.55	139.36
TC1700001158.mm.1	Slc30a6	570.99	607.72	504.86	413.89	401.14	426.37
TC1600001976.mm.1	Adamts5	6458.97	6462.11	5501.18	3861.35	3286.60	4246.41
TC0100003505.mm.1	Hsd17b7	283.49	259.62	212.05	161.76	148.27	158.28
TC0800001566.mm.1	Ntpcr	455.91	449.38	385.43	268.99	303.79	314.42
TC0700002830.mm.1	4931406P16Rik	517.16	447.29	416.24	315.80	269.67	313.24
TC0100003715.mm.1	Capn2	4668.24	4664.75	4091.90	3258.13	3262.13	3587.88
TC1500002289.mm.1	Rarg	97.52	98.62	88.07	73.42	74.60	79.20
TC0200005030.mm.1	Tgm2	1103.33	1086.08	938.41	765.94	745.06	809.00
TC0700004595.mm.1	Osbpl5	197.21	200.65	179.41	143.08	159.42	149.65
TC0500002760.mm.1	Scarb2	905.20	953.24	863.71	663.37	585.84	711.57
TC0700001879.mm.1	Tgfb1i1	663.16	633.22	590.45	503.70	435.81	482.38
TC1500000041.mm.1	Rictor	760.47	855.77	692.74	457.13	408.59	530.83
TC1000000193.mm.1	Vnn1	53.49	40.27	48.70	30.74	30.09	30.84
TC1300001835.mm.1	Txndc5	3538.36	3547.74	3329.98	2421.19	2167.55	2725.14
TC1400001233.mm.1	Gm6280	23.34	21.06	21.68	18.20	17.36	16.01
TC0600002935.mm.1	Sec13	1244.81	1240.58	1078.92	887.83	814.12	919.87
TC1600002168.mm.1	Vasn	165.48	165.95	160.75	139.74	119.14	127.83
TC0700001086.mm.1	Akap13	279.29	303.63	274.24	197.24	179.49	222.94
TC0200002589.mm.1	Snai1	154.85	127.52	131.44	82.82	98.00	94.61
TC0200000514.mm.1	Cercam	406.31	375.24	346.80	296.50	285.44	299.05
TC1200000156.mm.1	Gm3944	35.76	34.66	36.54	31.17	28.19	27.57
TC0300000765.mm.1	Pbxip1	96.64	94.02	85.88	73.79	77.81	74.07
TC1500000297.mm.1	Baalc	24.92	29.12	24.90	17.29	18.16	20.14
TC0800002169.mm.1	Stox2	73.00	66.73	58.46	46.82	42.26	46.99
TC1500001207.mm.1	Rai14	464.07	440.06	398.75	319.01	279.50	329.00
TC1600001679.mm.1	Pvrl3	107.91	111.51	98.05	78.70	74.03	85.40
TC1400002757.mm.1	Tmtc4	160.17	169.71	156.48	107.75	119.15	130.79
TC0100003233.mm.1	Pla2g4a	1368.75	1520.89	1418.22	1052.65	939.29	1136.14
TC0500000560.mm.1	Pcdh7	192.89	182.23	148.81	117.83	110.90	116.58
TC0300000802.mm.1	S100a16	187.18	180.31	152.71	128.45	120.55	115.87
TC1500001262.mm.1	Gm5803	172.85	178.12	211.45	118.85	124.13	137.84
TC0200002399.mm.1	Myl9	48992.77	44944.04	44845.30	34494.82	29484.78	35736.45
TC0600001679.mm.1	Golt1b	2477.07	2390.53	2184.07	1591.90	1572.21	1868.96
TC0900002346.mm.1	Tspan3	1667.69	1444.53	1378.69	982.29	991.58	1129.95
TC0200004922.mm.1	Pdrg1	185.77	205.27	189.24	146.57	144.86	163.08
TC1200001261.mm.1	Inf2	342.14	321.34	287.52	233.92	231.40	249.80
TC1400001741.mm.1	Ccser2	426.63	406.77	370.95	288.87	268.65	317.81
TC0900000562.mm.1	Zpr1	533.53	527.46	467.55	391.44	328.61	372.58
TC1600000558.mm.1	Tmem39a	628.22	594.42	546.06	400.21	379.61	461.15
TC0X00000512.mm.1	3830403N18Rik	246.47	235.05	204.89	153.43	168.36	171.81

TC0500002168.mm.1	Cgref1	215.05	166.30	164.69	117.32	95.65	107.49
TC0900000296.mm.1	Ets1	118.32	119.11	108.65	85.45	85.28	96.88
TC0900000492.mm.1	Pvrl1	523.27	542.30	440.82	335.28	263.85	323.45
TC0X00001859.mm.1	Med14	2126.39	2367.23	2000.33	1638.45	1664.11	1716.01
TC1500000417.mm.1	D15Ertd621e	3156.25	3108.07	2738.52	2339.29	2113.50	2338.30
TC1100003252.mm.1	Cpd	7999.12	8170.16	7585.37	6071.04	5904.61	6779.85
TC1200000460.mm.1	Srp54b; Srp54a	7923.32	8689.90	7808.40	4774.77	4790.40	6145.87
TC1500001167.mm.1	Osmr	2329.70	2505.32	2168.37	1529.29	1314.72	1713.71
TC0400000439.mm.1	Creb3	691.37	674.46	608.75	546.48	522.58	538.73
TC0400003361.mm.1	Plk3	556.46	523.10	474.23	346.55	325.38	397.92
TC0400000889.mm.1	Gm13271	60.23	63.29	55.82	43.58	46.39	49.31
TC1200000458.mm.1	Srp54a	9388.17	10370.31	9096.00	5245.95	5472.16	7031.61
TC0500000864.mm.1	Gm1045	32.68	41.05	38.35	21.74	26.46	24.19
TC1500001089.mm.1	Igfbp6	129.41	111.01	91.86	55.18	65.69	65.99
TC0600001383.mm.1	Gm8430	2021.61	1893.14	2225.18	1420.23	1216.22	1511.37
TC0900003233.mm.1	Plcd1	359.20	344.55	273.95	205.54	171.10	205.92
TC0400000529.mm.1	Nr4a3	32.57	25.85	28.86	20.36	21.13	20.80
TC1000000548.mm.1	Vsir	611.80	552.64	522.84	429.20	375.19	427.56
TC1700000899.mm.1	Cul7	140.73	141.22	125.11	104.94	100.70	111.95
TC1200000608.mm.1	Dact1	106.89	96.73	96.99	84.74	76.45	76.02
TC1600001742.mm.1	Nfkbiz	179.47	172.59	166.23	136.65	134.18	150.67
TC0400003241.mm.1	Gpx7	253.69	235.51	201.82	147.47	166.48	159.96
TC1700000496.mm.1	Cdkn1a	120705.00	123092.00	105145.90	91087.90	84338.35	90277.21
TC0300002664.mm.1	Wnt2b	52.62	59.68	46.76	34.72	38.18	36.51
TC0100003487.mm.1	Pbx1	129.28	117.39	108.89	91.16	86.57	93.71
TC1100002429.mm.1	Sptbn1	2395.52	2523.49	2242.14	1791.40	1905.45	2000.05
TC0400003483.mm.1	D830031N03 Rik; Macf1	1216.70	1413.48	1215.60	904.76	849.01	996.91
TC1300001812.mm.1	Nrn1	280.12	282.62	227.83	142.07	159.30	182.25
TC1800001640.mm.1	Setbp1	168.40	166.84	149.23	110.66	111.39	130.11
TC0900002480.mm.1	Smad3	176.15	188.46	167.33	133.89	129.31	147.88
TC1800000741.mm.1	Tcf4	943.92	958.86	807.90	636.37	530.53	643.04
TC1400002106.mm.1	Sdr39u1	106.91	88.71	93.27	69.53	74.59	71.57
TC0900002283.mm.1	Layn	2053.91	2041.46	1915.54	1686.46	1423.80	1510.77
TC1300002435.mm.1	Jmy	108.84	112.05	101.26	80.23	66.60	81.54
TC1800000015.mm.1	9430020K01Ri k	209.66	198.51	179.41	141.28	133.02	155.54
TC1300002471.mm.1	Polk	500.46	494.53	442.22	358.08	290.32	347.28
TC0100003093.mm.1	Zbed6; Zc3h11a	1498.31	1593.93	1456.75	1135.53	963.48	1183.42
TC1400001311.mm.1	Gm17613; LOC10086201 4	119.44	143.33	134.90	94.76	80.32	96.40
TC0800000192.mm.1	Gm6483	1449.97	1569.42	1508.87	1038.44	833.09	1103.75
TC0600001087.mm.1	Tmem43	2178.93	2092.38	1877.85	1444.40	1362.38	1624.57
TC0300003231.mm.1	Arfip1	291.31	251.21	250.18	189.35	170.92	203.19
TC0X00000012.mm.1	Gm3750; Gm3706	17.63	17.17	15.12	11.32	12.64	12.93
TC0200005164.mm.1	Sulf2	1988.13	1897.32	1739.95	1412.18	1211.59	1444.74
TC0900000210.mm.1	Prkcsh	2101.94	1916.51	1875.81	1477.99	1298.11	1557.39
TC0100000334.mm.1	Ercc5	73.44	72.32	64.85	57.02	55.22	58.78
TC0300003087.mm.1	Cyr61	61055.75	56713.21	54807.49	40779.31	39905.21	47367.33
TC0700003442.mm.1	Mef2a	5619.01	6298.24	5433.82	3531.88	3189.25	4270.17
TC1900000685.mm.1	Gbf1	723.22	744.56	665.62	549.15	520.89	596.62
TC0100001189.mm.1	Klhdc8a	74.91	69.01	68.12	53.20	59.89	52.12
TC1500000104.mm.1	Golph3	196.65	180.82	176.24	151.52	131.00	145.25
TC0900001486.mm.1	Epm2aip1	237.78	226.66	225.81	172.46	141.25	176.86
TC1400002841.mm.1	Gm3468	54.02	53.44	60.33	39.52	42.09	45.89
TC0200004924.mm.1	Gm20094	71653.33	66445.34	78751.95	56074.26	52865.77	47546.39
TC1400002103.mm.1	Ripk3	1441.88	1426.77	1138.88	940.15	888.98	918.17
TC1100000459.mm.1	Clint1	1252.21	1265.85	1132.72	905.50	971.17	1018.00
TC0900001674.mm.1	Ccr2	58.72	71.74	51.87	36.21	37.69	39.75
TC1100001660.mm.1	Nbr1	423.07	428.59	383.23	338.30	316.96	346.44
TC0900000131.mm.1	Gm5611	262.68	263.20	211.87	179.91	163.10	163.34
TC1500001403.mm.1	Ubr5	993.49	1082.62	930.87	673.09	653.16	793.34
TC0700000007.mm.1	Cacng7	280.64	284.94	259.36	215.02	215.19	238.23
TC1500002067.mm.1	Cpne8	169.89	194.24	175.43	112.77	92.82	128.80
TC0700003648.mm.1	Cemip	3835.07	3869.81	3513.58	2854.52	2688.94	3142.68
TC0700002507.mm.1	Pvr	746.11	681.88	600.67	485.36	461.29	512.24
TC0900002981.mm.1	Uba5	1444.65	1392.78	1377.33	1090.72	982.68	1183.33
TC1500001008.mm.1	Spats2	180.82	202.07	173.75	136.88	140.42	152.14
TC0700000720.mm.1	Vstm2b	72.49	63.81	67.57	56.55	52.29	49.23
TC0900002869.mm.1	Trpc1	55.33	58.92	52.42	46.07	39.13	39.74

TC0900001672.mm.1	Cxcr6	70.58	70.37	49.77	37.87	33.45	30.03
TC0500003116.mm.1	Hspb8	627.46	527.49	536.57	349.49	288.35	395.15
TC0800000871.mm.1	Slc10a7	372.08	369.44	310.90	243.53	239.28	271.90
TC0200002359.mm.1	Itch	565.11	509.43	508.01	393.96	363.20	432.76
TC1100000789.mm.1	Specc1	369.09	345.29	317.25	270.49	259.62	283.71
TC1600000097.mm.1	1810013L24Rik	3256.70	2673.72	2780.54	2200.82	1861.24	2014.72
TC0400003000.mm.1	Hacd4	3985.58	3723.97	3452.54	2915.85	2732.34	3066.30
TC0200001015.mm.1	Ubr3	411.94	408.12	371.25	296.17	304.91	337.43
TC0900001408.mm.1	Lamb2	463.83	443.33	383.80	299.72	278.10	330.69
TC1400001418.mm.1	Gm6356	52.44	47.52	59.89	38.77	34.15	30.95
TC1000000708.mm.1	Bcr	130.92	124.41	111.02	86.93	79.61	95.90
TC0800001050.mm.1	Chd9	487.37	559.21	473.36	364.24	339.18	399.04
TC0100000462.mm.1	Bmpr2	1803.68	2186.27	1671.95	1164.79	1216.07	1341.63
TC1800000543.mm.1	Csnk1g3	828.84	798.55	745.17	608.63	605.04	679.93
TC1200000751.mm.1	Smoc1	390.01	359.52	351.88	254.09	192.24	256.40
TC0500002386.mm.1	Adgra3; Adgra2	291.90	274.41	229.28	159.75	161.04	193.00
TC1300000958.mm.1	Arrdc3	165.33	153.92	137.50	103.97	89.28	113.15
TC1400002469.mm.1	Tnfsf11	63.02	47.80	54.75	33.61	37.07	38.61
TC1400002853.mm.1	Gm2897	60.29	52.99	52.20	44.58	37.97	38.89
TC1600000448.mm.1	Tnk2	157.72	167.59	139.73	120.14	122.23	113.10
TC1700002298.mm.1	Pot1b	74.41	69.81	71.43	63.28	56.16	55.29
TC1300002015.mm.1	Ror2	69.56	59.58	62.33	41.43	47.45	49.66
TC1100003538.mm.1	Tom1l1	115.87	106.45	91.68	70.85	60.37	74.31
TC1200001912.mm.1	Six1	103.36	115.10	101.24	81.48	72.47	85.67
TC0600001083.mm.1	Fbln2	4726.23	4665.77	4001.83	2962.54	2831.21	3469.70
TC0500003136.mm.1	Fbxw8	680.55	658.90	583.71	523.32	515.85	509.15
TC1200000134.mm.1	Rock2	1592.51	1791.28	1490.16	1122.65	989.54	1233.11
TC0200003579.mm.1	Acvr1	127.59	127.86	111.60	101.06	98.42	98.20
TC0700000005.mm.1	Myadm	41689.32	40366.82	35907.49	30827.86	27407.12	31188.24
TC1300000460.mm.1	Gcnt2	272.32	242.73	238.94	163.50	164.96	201.15
TC1000001328.mm.1	Zfc3h1	798.77	954.70	826.25	551.06	496.53	646.61
TC0900002276.mm.1	2310030G06Rik	68.25	49.52	54.63	31.92	38.72	31.83
TC0900000100.mm.1	Sesn3	170.09	176.38	134.75	107.79	99.66	111.35
TC0600002683.mm.1	Sec61a1	829.78	806.05	715.99	632.04	642.06	641.68
TC0500000402.mm.1	Afap1	94.55	89.72	86.46	68.25	60.33	74.19
TC1400002325.mm.1	R3hcc1	137.10	147.73	117.63	97.43	97.43	100.98
TC1600000440.mm.1	Tm4sf19	126.67	111.26	99.99	66.09	65.30	82.69
TC0400000458.mm.1	Glipr2	572.49	481.47	571.13	370.51	348.75	420.80
TC1200000996.mm.1	9030617O03Rik	105.41	112.71	100.36	79.50	76.92	89.88
TC0700001893.mm.1	Sec23ip	1663.70	1615.14	1464.99	1201.33	1037.26	1255.15
TC1600001879.mm.1	4930423O20Rik	315.19	360.84	326.81	265.57	209.41	230.25
TC1400000975.mm.1	Entpd4	215.15	207.41	183.13	161.55	156.29	164.34
TC0900002212.mm.1	Tagln	30167.29	30027.84	24557.48	19870.79	20722.36	21196.30
TC0500001026.mm.1	Pkd2	387.12	395.82	348.57	286.92	274.83	316.59
TC1100000475.mm.1	Havcr2	829.12	811.68	727.68	608.32	458.22	523.97
TC1600001939.mm.1	Btg3; Gm7334	1355.89	1270.96	1379.78	975.48	797.71	1032.43
TC0300001035.mm.1	Tspan2	38.67	41.55	34.30	29.51	28.28	30.21
TC1300002335.mm.1	Rhobtb3	257.57	264.56	215.69	186.24	158.63	174.69
TC0700001689.mm.1	Xylt1	474.98	461.84	426.11	325.28	316.31	380.39
TC1400002117.mm.1	Gzmg	82.68	67.28	84.20	55.60	57.16	56.72
TC0700001362.mm.1	Pak1	913.32	950.53	815.63	702.57	664.05	736.87
TC0700001767.mm.1	Gm20274	128.21	127.75	105.72	93.37	87.51	86.91
TC1400002336.mm.1	Slc39a14	204.85	199.42	189.03	158.68	131.76	157.17
TC0900001473.mm.1	Prss46	154.95	161.06	147.29	93.37	109.26	122.18
TC1700001178.mm.1	Crim1	1426.18	1311.99	1150.37	910.30	788.91	963.36
TC0400002016.mm.1	Acot7	468.79	426.81	393.56	308.49	292.79	345.65
TC0300001932.mm.1	Pcdh18	72.72	88.70	61.41	43.21	42.54	48.39
TC1100000937.mm.1	Dlg4	120.42	119.04	102.03	88.00	80.90	90.18
TC1700000474.mm.1	Ppard; 1810013A23Rik	234.81	240.14	203.76	172.95	177.40	183.79
TC1100001834.mm.1	Prkar1a	858.39	841.71	777.70	645.24	621.80	714.80
TC0X00001939.mm.1	Zfp300	35.36	35.92	31.33	28.58	28.22	27.61
TC0800001269.mm.1	Nfat5	469.76	532.05	490.53	364.54	296.66	378.42
TC1300000934.mm.1	2210408I21Rik	52.69	53.44	47.63	40.96	34.02	39.71
TC1200001757.mm.1	Cfl2	1768.37	1701.35	1395.49	1049.22	1179.81	1162.89
TC0900002284.mm.1	4833427G06Rik	59.46	57.77	60.90	43.91	35.08	45.70

TC1200001262.mm.1	Adssl1	291.46	269.37	266.18	197.34	211.24	235.09
TC0900002123.mm.1	Arhgef12	233.72	242.66	203.88	170.22	159.10	182.08
TC0X00003000.mm.1	Armcx2	110.65	107.85	90.28	79.85	73.05	75.45
TC1400001990.mm.1	Klhl33	20.87	16.95	20.68	13.91	13.70	14.93
TC1000002236.mm.1	Unc5b	553.32	499.57	477.16	377.07	290.83	363.89
TC1400001346.mm.1	Itgbl1	107.55	95.17	83.98	64.44	65.11	72.34
TC1400001900.mm.1	Gnpnat1	230.12	244.59	189.73	159.15	141.31	157.26
TC0400003329.mm.1	Nsun4	103.86	104.42	99.00	76.83	71.51	87.66
TC1100002449.mm.1	Erlec1	1140.14	1022.84	1077.31	827.98	658.52	816.06
TC0400004219.mm.1	B4galt2	154.20	138.76	131.98	116.26	98.27	102.28
TC0400000894.mm.1	Gm13272; Gm13285; Gm13290; Gm13289	32.00	29.44	31.73	26.62	23.09	25.55
TC0400002469.mm.1	AI464131	42.27	42.93	38.63	35.15	30.42	33.03
TC0400003537.mm.1	Ago3	376.06	395.23	332.94	273.23	263.43	300.51
TC1200000083.mm.1	Osr1	1288.13	1329.36	981.78	598.98	658.66	804.92
TC1500000805.mm.1	Atxn10	11711.93	11308.55	10722.06	8897.01	8684.54	9912.81
TC0900000019.mm.1	Gucy1a2	46.08	52.25	40.51	34.56	30.17	32.09
TC0300000972.mm.1	Sec22b	312.43	295.54	272.40	195.55	164.19	223.48
TC1000003032.mm.1	Msrb3	1224.42	1307.86	1061.42	865.86	852.61	945.01
TC0900000124.mm.1	Smco4	52.80	54.75	51.62	46.20	38.57	41.17
TC0800003222.mm.1	6820431F20Rik; Gm21092	3273.39	3756.94	3131.09	2292.26	1966.06	2560.05
TC1300000054.mm.1	Dip2c	230.44	229.08	215.03	182.73	164.60	193.55
TC0200000636.mm.1	Gsn	713.43	686.42	607.81	465.94	434.66	537.33
TC1600001461.mm.1	Atp13a3	1114.11	1156.20	1039.11	852.87	781.56	933.65
TC1300001789.mm.1	Pxdc1	161.44	166.21	134.58	113.77	90.68	105.79
TC1600001604.mm.1	Pla1a	99.75	90.89	82.93	71.60	58.87	66.16
TC1100003019.mm.1	Ndel1	399.91	415.27	355.33	307.64	284.92	320.76
TC1700000563.mm.1	Cyp4f17	145.52	145.99	139.60	102.62	101.79	123.77
TC1100000039.mm.1	Gatsl3	310.31	256.93	248.79	197.38	161.54	191.54
TC1400000980.mm.1	Entpd4; Gm21685	343.58	344.30	296.05	257.80	251.93	270.89
TC1400000958.mm.1	Ebf2	88.42	77.14	68.51	57.62	55.45	56.93
TC1000002612.mm.1	Rtcb	1996.21	1970.99	1676.05	1102.55	1141.10	1444.77
TC0900001155.mm.1	Ctsh	5137.80	4774.17	4534.35	3445.42	3021.04	3872.12
TC0500003048.mm.1	Tmem119	460.50	411.60	327.96	267.73	246.54	257.56
TC0600000308.mm.1	Exoc4	549.98	569.58	536.24	429.39	432.72	492.96
TC1100003072.mm.1	Kctd11	150.13	162.03	138.97	92.84	91.19	118.79
TC0200002293.mm.1	Rem1	104.32	105.42	91.57	75.47	65.51	79.87
TC0500000195.mm.1	Kmt2e	1562.65	1726.84	1510.27	1257.10	1139.68	1330.51
TC1500001971.mm.1	Arfgap3	310.90	295.06	261.64	218.51	195.25	230.83
TC0400001250.mm.1	Pik3r3	216.62	202.01	193.34	154.37	131.59	164.40
TC1100002288.mm.1	Actr2	8905.05	8828.37	8528.28	6990.76	7059.63	7937.12
TC0100000372.mm.1	Sdpr	681.83	687.03	466.26	221.45	207.58	349.85
TC1100000025.mm.1	Dusp18	156.36	149.55	124.53	105.68	108.06	96.80
TC0400003456.mm.1	Rlf	435.03	433.81	371.64	308.19	249.12	306.48
TC1300000476.mm.1	Hivep1	242.01	245.26	219.23	192.51	181.38	203.38
TC1400002832.mm.1	Pabpn1	682.97	667.95	617.91	523.85	518.18	579.39
TC0200001053.mm.1	Zak	237.87	226.38	223.59	172.05	174.91	202.22
TC0800001872.mm.1	Polb	200.91	205.53	187.47	160.70	143.03	168.27
TC0700000399.mm.1	Gm9844	28498.37	27718.20	31669.69	21997.44	16509.44	20729.96
TC0500000648.mm.1	Slc30a9	450.89	442.21	405.16	325.18	283.01	353.97
TC1200001715.mm.1	Strn3	897.88	1021.86	864.82	673.09	597.66	737.55
TC0100001611.mm.1	Kcnj10	959.31	853.23	742.65	618.92	541.90	619.87
TC0200000574.mm.1	Golga2	218.79	212.84	211.29	151.21	165.22	185.79
TC0500000112.mm.1	Cacna2d1	168.82	147.58	133.79	102.22	90.68	113.31
TC1300000924.mm.1	Ttc37	330.99	336.80	317.90	258.90	240.44	288.10
TC1300000364.mm.1	Serpinb9	113.77	109.51	100.99	93.85	84.76	82.38
TC0800000328.mm.1	Plpp5	442.90	439.94	363.96	314.99	262.99	302.46
TC0600001736.mm.1	Stk38l	501.09	504.49	495.75	383.56	304.41	393.02
TC1400002843.mm.1	Gm3411	45.64	39.04	44.52	31.42	34.32	35.29
TC1100004196.mm.1	Slc38a10	441.12	475.87	398.73	309.69	310.39	360.44
TC0800001031.mm.1	Adcy7	1276.70	1262.69	1139.42	897.20	860.30	1038.18
TC1500002059.mm.1	Rabl2	304.23	288.92	240.46	193.14	206.07	210.35
TC0500000896.mm.1	Ccng2	96.36	95.95	92.48	76.21	66.05	80.71
TC0400000613.mm.1	Rad23b	8886.23	8508.04	7774.79	6250.57	5309.91	6725.38
TC1500002209.mm.1	Fmnl3	522.46	478.63	470.32	359.75	307.85	397.75
TC0500001909.mm.1	Cldn12	70.87	81.98	81.15	64.09	62.21	65.40
TC0800000368.mm.1	Rnf122	71.72	60.63	56.41	42.21	33.67	44.71
TSUnmapped00000001.mm.1	Plppr2	354.24	308.03	320.70	238.62	230.74	276.24
TC0800002145.mm.1	Fam149a	495.65	458.68	419.18	345.82	284.52	352.60
TC0300002406.mm.1	Lce1f	47.55	49.90	46.06	22.35	35.06	22.27

TC0500002231.mm.1	Mxd4	683.70	624.26	558.73	455.08	456.73	506.24
TC0500002432.mm.1	LOC432823	582.48	678.55	562.42	462.55	378.69	453.20
TC0700001275.mm.1	Picalm	30732.23	31765.40	29081.71	23672.83	20935.16	25851.12
TC0600003020.mm.1	Erc1	190.39	204.36	186.03	148.35	139.13	167.69
TC0400001957.mm.1	Clstn1	188.42	171.94	167.79	136.12	131.58	152.78
TC0700004119.mm.1	Dkk3	932.26	929.04	840.55	741.94	618.01	720.75
TC0400000834.mm.1	Snapc3	344.06	388.00	334.77	259.66	218.63	280.42
TC0300000925.mm.1	Mtmr11	60.10	60.61	57.25	46.81	41.63	51.11
TC1200001106.mm.1	Hhipl1	193.81	187.41	144.19	112.25	83.93	112.08
TC1600000890.mm.1	Gm17333; LOC100862299	52.34	56.81	56.77	43.85	32.27	39.18
TC0300002025.mm.1	Wwtr1	151.50	131.83	117.88	82.79	62.54	91.42
TC1800000599.mm.1	Chsy3	60.47	47.91	45.83	35.60	37.47	34.32
TSUnmapped00000005.mm.1	Amot	116.97	103.25	102.63	70.13	59.15	83.27
TC0600000215.mm.1	Snd1	484.07	492.62	441.00	354.31	355.29	410.82
TC0900001905.mm.1	Zfp810	142.84	138.76	133.05	101.56	77.51	104.77
TC0700000963.mm.1	Atp5l-ps1	1102.68	874.77	1018.16	736.76	556.87	673.30
TC0500000289.mm.1	Ept1	609.39	622.93	516.06	400.17	412.13	471.45
TC0100000323.mm.1	Nck2	109.31	104.04	91.38	79.41	84.21	78.86
TC0800002471.mm.1	Large	892.10	840.00	745.53	625.35	567.21	672.18
TC0500001328.mm.1	Tbx5	32.50	27.04	31.68	21.66	24.82	22.65
TC0200000274.mm.1	Plxdc2	3784.25	3837.14	3206.74	2644.67	2363.09	2874.19
TC0200002136.mm.1	Dstn	23511.39	22807.33	20571.39	15625.47	15896.85	18878.80
TC0900000090.mm.1	Maml2	845.84	909.44	782.85	665.89	661.14	729.20
TC1000002265.mm.1	Ddx50	143.26	158.90	130.89	113.83	100.54	114.34
TC0800000441.mm.1	D8Ertd82e	75.75	65.99	64.57	52.03	47.38	56.56
TC0400003417.mm.1	Zfp691	140.55	121.20	137.46	112.33	109.71	109.84
TC0900001128.mm.1	Nt5e	172.73	163.82	144.24	118.34	107.33	130.28
TC0300002595.mm.1	Ptgfrn	311.80	298.73	259.48	172.42	145.92	217.00
TC1500000021.mm.1	Prkaa1	3816.27	3633.61	3200.10	2511.78	2255.04	2863.08
TC1100003770.mm.1	P3h4	1911.79	1817.29	1725.55	1372.45	1277.80	1569.67
TC0700003783.mm.1	Emsy	810.07	839.88	701.02	601.89	508.60	611.42
TC0500000021.mm.1	Akap9	110.34	137.58	113.71	87.20	85.16	95.39
TC0900002779.mm.1	Pgm3	196.91	221.91	184.53	138.95	134.92	165.21
TC0400000084.mm.1	Trp53inp1	675.21	712.51	621.37	508.32	418.44	533.36
TC0900001725.mm.1	9230110C19Rik	54.94	52.23	50.70	35.66	34.94	44.82
TC1800000805.mm.1	Smad7	76.95	80.72	70.12	62.37	48.41	55.00
TC0100003380.mm.1	Suco	435.39	484.41	387.67	285.69	266.24	343.45
TC0500000532.mm.1	Pi4k2b	168.28	173.58	152.53	109.49	100.45	134.93
TC1000002725.mm.1	Socs2	100.65	95.79	86.71	72.25	70.77	81.15
TC0100003302.mm.1	BC034090	46.21	40.79	38.29	32.60	32.22	34.68
TC1200001527.mm.1	9030624G23Rik	105.79	92.00	93.08	69.53	58.40	77.18
TC0400000248.mm.1	Map3k7	621.73	551.81	527.36	439.13	423.35	480.30
TC0X00000316.mm.1	Il13ra1	5674.09	5476.99	4706.35	4048.52	4008.54	4367.99
TC0800001289.mm.1	Zfhx3	1021.98	952.02	896.95	712.33	631.11	799.16
TC1100002921.mm.1	Ulk2	537.56	554.30	461.87	392.78	332.33	406.71
TC1400002866.mm.1	Gzmc	52.69	42.48	45.59	36.82	31.33	28.02
TC0900001724.mm.1	Yap1	151.39	140.61	131.87	101.27	96.98	120.18
TC0500002840.mm.1	Plac8	210.38	185.36	211.13	145.23	159.49	171.55
TC1600000392.mm.1	Hes1	133.31	120.78	129.50	95.68	91.40	111.65
TC1400000130.mm.1	Top2b	420.65	465.36	402.61	333.45	293.07	357.29
TC1600000335.mm.1	St6gal1	674.37	617.26	574.16	442.98	432.21	525.09
TC0200001072.mm.1	Scrn3	143.78	128.78	138.90	108.97	99.46	119.12
TC0X00003146.mm.1	Lrch2	65.62	62.78	53.93	41.99	38.71	49.07
TC1000002592.mm.1	Nuak1	2638.16	2601.34	2220.71	1928.62	1784.51	2054.41
TC1700001598.mm.1	Tnfrsf12a	441.73	429.77	347.74	178.68	182.24	288.84
TC0400000532.mm.1	Msantd3	49.35	55.39	58.78	46.02	39.86	41.46
TC0100000021.mm.1	Rb1cc1	255.42	274.84	223.12	184.89	154.47	194.42
TC1300002629.mm.1	Zswim6	4420.96	4195.28	3624.41	3145.42	2724.20	3236.17
TC0800001245.mm.1	Slc7a6	737.48	708.48	647.66	546.85	505.79	603.34
TC1300002474.mm.1	Hmgcr	715.10	722.06	605.73	448.40	457.96	559.37
TC1900001490.mm.1	Aldh18a1	674.19	655.90	580.92	443.04	456.26	541.04
TC1200000114.mm.1	Gm9847	1060.00	1108.51	802.18	614.37	436.19	629.90
TC0600002177.mm.1	Tcaf2	72.75	66.21	63.74	52.30	44.53	55.84
TC0300002800.mm.1	Slc30a7	1231.54	1196.08	1109.87	882.77	884.05	1042.70
TC1300000607.mm.1	Gadd45g	603.04	524.57	528.63	353.25	353.09	457.80
TC0500001134.mm.1	Golga3	120.54	125.48	119.83	90.43	83.02	106.53
TC0400002513.mm.1	Vcp	2545.34	2546.63	2407.24	1982.81	1822.30	2223.07
TC0800000440.mm.1	Rps12-ps24	6157.93	5633.88	6508.47	5260.48	4398.53	4662.24
TC0600002796.mm.1	Eogt	436.54	411.13	360.98	302.59	283.96	335.16
TC0900000662.mm.1	Crabp1	243.64	265.13	218.42	165.54	191.65	193.98

TC0200002648.mm.1	Dok5	180.23	170.89	157.32	93.28	111.99	134.75
TC0700000847.mm.1	Gys1	271.70	276.10	241.91	214.40	198.05	227.62
TC0100002337.mm.1	Stk17b	2998.72	2891.93	2428.56	1991.39	1749.92	2200.57
TC0400003822.mm.1	Cda	98.14	103.43	72.33	58.51	61.29	48.49
TC0100001574.mm.1	Fcgr4	366.91	319.09	283.67	235.80	200.46	245.49
TC0300002470.mm.1	Mllt11	275.53	257.21	220.42	181.39	140.39	184.99
TC0200003435.mm.1	Golga1	123.04	110.76	105.81	86.32	81.80	97.43
TC1600000618.mm.1	Zbtb20; Mir568	147.52	181.79	145.18	113.91	109.57	126.18
TC0200001025.mm.1	Gorasp2	272.39	263.98	229.79	207.59	207.46	215.33
TC1300000076.mm.1	Gm2399	3466.72	3575.81	2674.25	1739.78	1225.88	2122.77
TC0600003169.mm.1	Ccnd2	19061.69	18107.46	15179.21	13006.52	10424.60	13039.52
TC1500002339.mm.1	Hoxc10	517.71	527.30	541.99	363.61	239.08	368.65
TC1_GL456221_random00000010.mm.1		898.20	1087.79	1132.86	735.12	418.69	534.63
TC1600000188.mm.1	Gm21897	26020.76	29952.15	27622.34	19589.38	12418.97	18276.85
TC1700002699.mm.1	Kcnk12	35.88	46.80	40.72	29.15	32.49	27.94
TC0300001031.mm.1	Ngf	1557.11	1773.37	1554.99	1239.41	1201.68	1416.18
TC1400001958.mm.1	Exoc5	401.30	408.56	352.38	306.56	296.20	335.96
TC1800001139.mm.1	Nrep	93.23	112.04	117.79	77.96	86.24	80.44
TC0600002554.mm.1	Dok1	357.44	288.18	251.12	196.96	167.20	210.65
TC0100002312.mm.1	Gm8420	36447.83	33686.74	35196.09	30421.21	23238.32	25407.71
TC0300000453.mm.1	Tsc22d2	577.20	641.52	544.88	457.06	415.03	498.52
TC0100002314.mm.1	Gls	441.39	519.29	421.03	325.88	330.04	381.19
TC0300002828.mm.1	Ptbp2	194.80	246.56	191.58	144.35	121.73	159.00
TC1300002596.mm.1	Trappc13	562.06	616.93	494.49	418.90	365.81	446.93
TC1000002224.mm.1	Ddit4	205.73	143.50	161.76	112.63	86.93	114.21
TC1900000015.mm.1	1810055G02Rik	251.75	274.40	201.28	152.73	164.10	182.52
TC0900000848.mm.1	Rbpms2	631.22	631.88	590.51	453.30	554.37	473.38
TC0400000902.mm.1	Gm13285; Gm13287	98.16	84.03	74.07	60.78	56.09	66.36
TC0500000843.mm.1	Pf4	5802.30	4904.11	5500.85	4140.34	4665.61	4055.29
TC1100003071.mm.1	Tmem95	38.36	40.82	28.54	24.14	24.88	22.19
TC0700003978.mm.1	Prkcdbp	2327.95	1667.22	1485.32	1194.84	983.03	1067.93
TC0900001838.mm.1	Zfp266	758.11	742.02	703.45	584.92	413.03	536.66
TC0300001395.mm.1	Gm21962	40551.33	50620.02	48255.66	35107.37	23470.52	29609.41
TC0500002885.mm.1	LOC432823	410.28	418.67	441.07	296.06	221.53	329.78
TC0400001395.mm.1	Pabpc4	636.76	606.09	555.44	512.71	415.27	471.85
TC0200003398.mm.1	Rc3h2	1342.76	1610.21	1307.39	976.72	906.38	1159.31
TC0800000472.mm.1	Micu3	100.71	89.12	77.45	62.47	54.21	69.53
TC1700000414.mm.1	Gm8225	297.27	278.46	268.87	246.69	196.31	194.40
TC1700002660.mm.1	Prepl	539.72	506.19	437.60	380.49	322.15	393.78
TC1000001084.mm.1	Arl1	2838.76	2733.61	2363.97	2007.35	1935.87	2250.66
TC0700000247.mm.1	Ccdc8	28.70	27.40	24.16	22.61	17.80	17.83
TC0500001170.mm.1	Gm26897	46.06	54.93	60.09	37.40	42.82	38.06
TC0100001358.mm.1	Edem3	511.64	536.98	479.25	361.23	355.19	446.84
TC1200002243.mm.1	Trip11	276.00	312.09	253.09	227.48	191.84	219.64
TC0500003268.mm.1	Ncor2	525.17	493.56	443.25	414.63	354.99	391.99
TC1600000911.mm.1	1700066C05Rik	232.47	247.05	302.70	193.71	144.39	182.26
TC1200001306.mm.1	Ncapg2	267.75	290.07	244.02	198.50	182.93	227.73
TC0700000240.mm.1	Dact3	84.73	71.55	77.40	54.79	65.71	62.07
TC0500001153.mm.1	Ttc28	115.52	116.41	102.39	86.03	89.56	98.69
TC0700000946.mm.1	Atp10a	323.90	329.37	302.12	253.62	252.73	291.16
TC0500000125.mm.1	Hgf	347.66	366.05	319.59	269.46	211.53	275.43
TC0500001451.mm.1	Atp6v0a2	724.61	707.63	648.87	548.10	518.10	619.63
TC1300001353.mm.1	Hmgcs1	2297.02	2194.80	1927.20	1516.63	1510.17	1832.39
TC1000002464.mm.1	2610008E11Rik	178.79	222.92	183.29	136.86	130.53	159.70
TC0300000139.mm.1	Hltf	167.76	192.31	176.90	141.24	118.03	149.52
TC0100003620.mm.1	Cep170	977.48	1194.47	1000.99	748.68	625.56	842.51
TC1200000211.mm.1	Kidins220	776.93	803.83	748.54	610.95	563.28	697.91
TC1700001857.mm.1	Cyp4f13	184.11	180.92	156.93	149.73	140.30	144.47
TC1500000221.mm.1	Sdc2	4656.80	4663.81	4116.33	3411.82	2845.98	3731.09
TC1000003103.mm.1	Arhgef25	179.32	176.13	159.88	126.76	126.38	152.71
TC1300002380.mm.1	Rasa1	863.35	1003.69	839.85	660.32	533.20	719.66
TC1700000344.mm.1	Pkd1	493.16	471.96	409.62	368.24	335.19	385.45
TC0X00002158.mm.1	Zdhhc9	253.20	243.97	226.21	218.05	186.81	192.69
TC1400002059.mm.1	Ajuba	238.08	185.06	162.29	138.60	109.72	127.13
TC0X00002671.mm.1	Las1l	115.34	110.72	107.76	86.34	77.89	99.23
TC0600002672.mm.1	H1fx	17.51	19.54	16.80	13.74	15.86	13.45
TC0200005363.mm.1	Gm14409	236.12	219.95	246.60	207.75	164.31	176.22
TC1300002689.mm.1	Gpx8	125967.10	117189.30	94130.78	75308.51	82381.70	86797.10

TC1500002176.mm.1	Kansl2	491.85	469.64	449.69	363.36	326.41	417.49
TC1200000303.mm.1	Snx13	1053.01	1065.95	904.55	820.52	808.75	871.81
TC1000002195.mm.1	Serinc1	30570.69	29719.25	25512.94	22503.43	22751.18	24569.25
TC0700004353.mm.1	Armcx3	4460.29	4205.85	3935.44	3081.21	2894.12	3704.71
TC0100003102.mm.1	Btg2	247.58	230.47	216.40	162.96	183.53	202.12
TC0100002562.mm.1	Igfbp5	192.11	153.67	146.48	131.26	105.61	110.87
TC0100003409.mm.1	Prrx1	426.08	417.03	352.56	305.00	281.62	337.41
TC1100003992.mm.1	Wipi1	524.95	498.45	475.93	385.17	269.84	378.22
TC0300003013.mm.1	Dapp1	140.57	146.01	124.71	118.86	100.81	111.32
TC1400002524.mm.1	Gm10110	963.26	914.21	723.29	653.85	541.59	642.65
TC1600000499.mm.1	Gm10237	28.54	26.73	26.41	24.71	20.36	19.92
TC1400001672.mm.1	Nisch	1977.50	1966.13	1782.31	1554.69	1551.60	1747.11
TC0100002237.mm.1	Rnf149	521.15	489.61	424.09	376.27	332.57	398.17
TC0100000131.mm.1	Efhc1	30.09	34.42	26.99	22.59	25.17	23.50
TC1700000703.mm.1	Ier3	2091.10	1963.01	1741.73	1586.01	1141.84	1384.29
TC0900003310.mm.1	Lztfl1	279.00	283.09	243.02	211.31	183.05	228.40
TC0600003122.mm.1	P3h3	248.88	225.50	191.40	162.56	148.30	179.98
TC1900001265.mm.1	Smc5	387.76	438.60	384.96	288.72	250.37	344.10
TC0500003208.mm.1	Fam216a	268.90	255.40	256.89	204.66	177.54	231.06
TC1200000173.mm.1	BC024416; LOC100861711	202.75	177.44	204.54	157.86	128.59	160.95
TC0X00000373.mm.1	Mcts1	320.13	306.78	263.71	236.40	175.56	222.98
TC0700001413.mm.1	Rab6a	2490.74	2498.35	2085.43	2001.32	1847.59	1912.60
TC1800000623.mm.1	Pdgfrb	6881.74	6866.11	5886.56	5250.80	5260.08	5736.66
TC1200001913.mm.1	Six4	43.96	51.00	49.29	35.45	41.96	39.49
TC1300001270.mm.1	Plpp1	954.11	927.19	731.75	647.70	602.05	689.59
TC0400003149.mm.1	Gm10576	544.55	607.32	491.52	421.59	311.25	414.46
TC0500002889.mm.1	Gm10047	47.49	42.73	38.39	26.50	21.83	34.31
TC0100003146.mm.1	Camsap2	557.24	590.69	513.17	435.60	421.66	497.66
TC0200001896.mm.1	Gm17555	199.32	221.11	238.36	162.10	115.79	167.07
TC1500000705.mm.1	Tnrc6b; Mir5113	167.91	174.72	148.29	130.83	129.00	144.78
TC1900001588.mm.1	Mgea5	239.87	249.32	218.08	179.19	169.94	210.45
TC0X00000213.mm.1	Cdk16	988.44	1011.18	874.62	751.79	751.72	857.62
TC0400001517.mm.1	Sync	161.12	159.59	154.79	120.63	108.18	142.62
TC1000002775.mm.1	Tmtc3	557.11	622.39	480.71	397.65	342.43	447.60
TC0100002836.mm.1	Stk25	542.59	551.51	448.03	386.02	352.60	431.37
TC0200004713.mm.1	Jag1	355.56	333.24	289.91	252.42	237.65	280.28
TC0100000297.mm.1	Il1rl2	341.84	310.84	305.67	210.46	191.16	276.37
TC1200002020.mm.1	Adam4	105.59	105.38	105.82	76.27	93.57	66.86
TC0100003301.mm.1	Mr1	1054.67	933.32	959.90	665.47	742.31	868.05
TC0X00001285.mm.1	Armcx4	25.22	30.13	33.97	20.67	23.38	22.49
TC1500002346.mm.1	Plec	459.96	460.76	420.79	346.99	324.74	405.74
TC0300000974.mm.1	Notch2	1430.82	1492.55	1295.31	1077.11	1038.55	1261.77
TC1000000420.mm.1	Gm4795	462.81	474.23	459.57	420.69	361.59	320.46
TC0X00002751.mm.1	Phka1	142.76	135.51	111.81	84.86	92.51	107.97
TC1600000930.mm.1	Gm21833	2782.52	2672.19	3073.35	2011.83	888.13	1418.02
TC0100000870.mm.1	Ackr3	133.10	103.67	102.47	70.47	74.21	91.96
TC0100000602.mm.1	Xrcc5	82.97	84.08	75.35	65.41	63.16	74.03
TC0200002746.mm.1	Gm14401	405.20	396.99	421.12	315.00	233.43	332.85
TC1200000636.mm.1	Mnat1	108.09	120.37	96.91	65.38	70.69	91.67
TC1700001614.mm.1	Kctd5	451.79	371.50	441.54	333.09	330.18	362.38
TSUnmapped00000024.mm.1	Gm21992	269.70	263.91	214.82	147.81	164.39	207.06
TC0900000862.mm.1	Ppib	64619.59	67391.88	52682.41	46744.57	50150.90	48171.27
TC1200000197.mm.1	Taf1b	192.03	190.23	178.95	148.04	150.98	174.97
TC0X00001283.mm.1	Hnrnph2	231.14	231.91	217.82	201.86	154.88	182.90
TC1000003119.mm.1	Lrp1	26983.64	27411.12	24523.46	21268.84	21383.64	24294.73
TC0500002592.mm.1	Ociad2	51.35	53.43	52.96	35.92	42.04	47.22
TC1000000842.mm.1	Midn	375.46	388.11	319.09	305.06	246.51	285.12
TC0800001977.mm.1	Mak16	1196.06	981.29	1059.07	854.28	729.01	913.70
TC1400000843.mm.1	Mtmr6	1493.20	1437.22	1261.97	1035.20	857.61	1185.68
TC0900000391.mm.1	Olfr894	11.62	9.85	10.57	8.38	9.30	7.26
TC0100002071.mm.1	Fam135a	199.84	208.39	175.55	145.76	131.93	170.53
TC1900000373.mm.1	Klf9	472.02	456.39	383.83	364.35	357.86	359.80
TC0Y00000255.mm.1	Gm20815	22.40	18.51	24.69	17.08	16.89	16.80
TC0500003027.mm.1	2900026A02Rik	144.43	129.43	107.81	95.11	76.54	98.95
TC0700003476.mm.1	Nr2f2	46.90	43.50	35.77	34.45	29.68	31.92
TC1200000524.mm.1	Prpf39	240.25	279.53	215.15	176.59	200.92	193.10
TC1400001422.mm.1	Gm3715	65.75	56.27	58.85	44.13	54.11	43.47
TC0700000991.mm.1	Chsy1	6487.31	6274.76	5178.97	4625.50	3821.31	4878.73
TC1400000453.mm.1	Gm8113	47.04	35.61	38.30	26.94	33.22	28.97
TC1600002094.mm.1	Rcan1	2180.94	2060.79	1912.56	1594.55	1242.39	1722.16

TC1400001275.mm.1	Gpr180	166.27	167.97	148.60	126.00	108.22	141.78
TC1200000750.mm.1	Srsf5	490.40	500.40	490.40	366.89	323.58	446.71
TC1100001165.mm.1	Ift20	41127.18	43796.08	36923.43	27132.43	34703.10	32547.05
TC1400000072.mm.1	Abhd6	48.55	58.15	45.95	36.53	35.88	43.59
TC0100003877.mm.1	Kdelc1	99.24	103.73	89.42	69.60	66.43	87.29
TC0500003608.mm.1	Rnf6	127.04	124.51	109.23	95.95	95.38	108.73
TC1600000164.mm.1	A930007A09Rik	60.16	48.21	48.28	33.43	36.84	44.13
TC0400000133.mm.1	Tmem64	212.49	214.08	190.97	142.55	183.56	164.20
TC1900001323.mm.1	Glis3	200.19	201.48	167.19	164.22	146.21	154.29
TC1100001149.mm.1	Rab34	324.02	308.35	293.85	231.10	240.81	286.20
TC0X00002826.mm.1	Taf9b	332.34	329.94	280.96	276.42	241.14	259.10
TC0100002284.mm.1	Gm5269	59.20	47.18	51.90	40.24	24.99	36.76
TSUnmapped00000025.mm.1	Gm21992	205.87	203.80	158.64	115.77	124.09	157.67
TC0300000206.mm.1	Prkci	352.28	357.05	296.69	277.63	241.60	284.93
TC0200005347.mm.1	Gm11009	172.82	182.49	178.61	144.83	82.74	116.60
TC0800002908.mm.1	Ist1	491.42	474.63	432.27	358.10	363.83	429.11
TC1300002327.mm.1		168.49	222.62	217.21	145.18	90.17	139.14
TC1600001559.mm.1	Fam162a	162.06	155.99	151.23	118.83	113.00	145.27
TC0500003371.mm.1	Gtf2ird1	131.22	129.30	110.29	107.05	92.73	103.68
TC1200002652.mm.1	Ighv1-73	21.08	24.19	18.24	13.91	16.73	16.78
TC0800003015.mm.1	Mphosph6	1119.44	1317.94	972.25	740.29	925.56	832.21
TC0900000943.mm.1	Adam10	4469.96	4431.83	3881.07	3490.50	2636.65	3478.16
TC1700000777.mm.1	Olfr111	2255.28	2739.89	1992.97	1542.83	1461.59	1915.21
TC1900001029.mm.1	Nudt22	462.54	410.89	390.41	371.89	291.09	329.67
TC0600002983.mm.1	Rpl28-ps4	107.74	128.67	127.98	105.21	77.20	89.09
TC0500001896.mm.1	Fzd1	69.57	88.02	82.67	58.32	63.49	68.08
TC1200000416.mm.1	Scfd1	943.75	882.96	764.95	629.49	551.25	747.64
TC0600002423.mm.1	Il12rb2	20.44	20.44	20.52	30.59	30.11	29.80
TC1100001219.mm.1	Ccl2	34497.96	36997.16	35359.23	96511.84	98021.76	99701.23
TC0800001094.mm.1	Mt2	55342.99	58697.41	56068.81	211983.40	207004.90	227609.30
TC0100003280.mm.1	Npl	125.13	127.63	138.04	2874.05	2598.69	3348.89
TC0600002965.mm.1	Plxnd1	413.42	405.13	353.89	2820.35	2720.68	2944.91
TC0X00001892.mm.1	Maob	59.83	58.78	60.40	192.90	174.86	186.16
TC0200000382.mm.1	Il1rn	391.39	402.87	358.59	6404.79	5302.52	6656.19
TC1100003399.mm.1	Ccl6	728.57	621.27	678.84	8960.44	7409.79	8183.43
TC1400002162.mm.1	Phf11b	3015.27	2886.47	3143.04	7144.98	7101.37	7265.84
TC1100004229.mm.1	Cbr2	5717.06	5209.81	4962.55	22646.67	23214.75	22142.47
TC0600003124.mm.1	Cd4	36.18	35.07	39.51	124.39	124.42	127.10
TC1100001220.mm.1	Ccl7	78547.94	75466.38	72810.62	198197.40	198749.20	211197.40
TC1400002161.mm.1	Phf11a	247.08	257.13	246.51	500.69	478.52	501.11
TC1400001725.mm.1	Sncg	78.35	88.79	89.75	3675.22	2582.27	3316.89
TC0900002387.mm.1	Islr	58.28	50.98	54.77	358.52	424.47	382.38
TC0800002905.mm.1	Hp	54.07	48.19	65.39	1515.31	1915.58	1779.19
TC0600000570.mm.1	Tmem176a	92.95	94.52	86.84	249.98	243.35	233.55
TC1500000963.mm.1	Slc48a1	3216.22	3080.87	3203.66	5958.07	6294.85	6248.02
TC1100003573.mm.1	Gm11545	841.51	773.84	779.98	6682.11	5362.19	6190.04
TC0200001445.mm.1	Syt13	27.26	24.87	25.34	61.68	64.45	63.81
TC0700004631.mm.1	Pirb; Pira1	1653.13	1575.76	1572.17	3065.66	2969.90	3121.46
TC0800001095.mm.1	Mt1	14625.28	14274.00	14430.50	39546.73	40313.38	44640.25
TC0800000297.mm.1	Sfrp1	92.96	82.12	86.05	863.39	893.29	1114.06
TC0300000060.mm.1	Fabp5	216.36	209.78	232.60	644.51	661.04	708.51
TC1100003141.mm.1	Slc13a5	21.62	21.85	21.02	33.08	32.02	32.88
TC0800001737.mm.1	Gas6	26716.06	25698.37	24754.84	56181.01	54555.33	58433.65
TC0600001403.mm.1	Clec4n	773.53	750.83	667.36	5418.26	6546.70	5338.34
TC1000001694.mm.1	Cnksr3	116.34	119.11	119.24	171.85	168.40	175.49
TC0600003109.mm.1	C1s2	12.71	14.02	13.70	32.63	35.08	33.55
TC0X00001667.mm.1	Figf	209.02	198.51	192.72	484.85	449.12	484.48
TC0700001631.mm.1	Ampd3	688.09	648.74	642.28	1465.41	1360.06	1437.84
TC0X00002453.mm.1	G6pdx	3630.76	3689.76	3417.00	6846.11	6656.82	6927.93
TC0800000621.mm.1	Hpgd	274.57	270.93	254.97	2170.62	1879.20	2507.07
TC0700003570.mm.1	Anpep	9924.29	9942.40	9367.61	47970.76	39243.55	43132.48
TC0300001488.mm.1	Mcoln3	42.19	38.32	40.56	82.13	83.51	85.16
TC0700004165.mm.1	Arl6ip1	4910.70	4714.46	4713.73	7456.71	7765.76	7480.25
TC1100002195.mm.1	Igfbp3	49.85	52.78	52.28	103.81	110.65	112.99
TC0500002843.mm.1	Hpse	262.82	229.76	200.85	2443.06	2131.93	2637.38
TC1100003401.mm.1	Ccl3	17812.49	19500.29	17440.15	45066.48	43894.51	42877.46
TC1400000316.mm.1	Mustn1	1408.56	1333.24	1327.08	2748.46	2554.73	2609.11
TC0200003880.mm.1	Serping1	2183.54	2022.69	2046.66	7050.81	6185.54	7138.52
TC0300002725.mm.1	Gstm1	1956.77	1958.34	1682.44	6560.56	6207.40	6213.82
TC0300001639.mm.1	Fabp4	358.96	303.83	389.80	2638.05	2392.28	2865.36
TC1000003214.mm.1	Lilrb4a	187971.30	193661.70	181764.90	295815.30	292771.30	288269.00
TC1100001222.mm.1	Ccl12	253.80	186.95	225.18	1714.37	1608.50	1765.39
TC1700000839.mm.1	Rcan2	28.82	30.49	29.39	60.10	54.98	57.70

TC0100003099.mm.1	Prelp	6086.40	5584.17	5416.86	27071.29	23181.17	22565.43
TC1900001027.mm.1	Vegfb	354.91	349.30	350.90	790.85	752.82	858.08
TC0700003039.mm.1	Saa3	36.47	38.33	36.96	135.51	159.89	167.93
TC0700000396.mm.1	Ceacam10	43.15	38.29	37.36	106.15	111.49	112.48
TC0200002367.mm.1	Acss2	30.85	30.72	31.29	53.97	50.79	49.95
TC0300001236.mm.1	F3	1582.17	1484.71	1234.44	18129.59	13860.12	18328.54
TC1100000859.mm.1	Gas7	41.69	43.01	41.85	107.05	96.35	110.90
TC0300002972.mm.1	Npnt	60.79	65.12	58.92	143.52	132.36	134.77
TC1100001223.mm.1	Ccl8	55.77	52.24	54.34	804.75	535.71	617.84
TC0600002233.mm.1	Tmem176b	443.11	453.13	397.00	1164.68	1121.10	1226.85
TC0800000845.mm.1	Hmox1	1861.05	1658.77	1686.59	5279.42	4826.64	5574.56
TC1500001457.mm.1	Rspo2	57.62	59.35	56.95	214.35	216.36	264.50
TC0700004666.mm.1	Fgfr2	24.68	24.80	24.71	50.02	47.60	53.85
TC0400003814.mm.1	Alpl	39.13	38.91	37.84	110.75	94.34	98.43
TC0800000674.mm.1	Tmem192	1129.77	1096.23	1103.25	1450.40	1409.10	1448.22
TC1500001729.mm.1	Ly6c1	7968.97	8276.53	7543.83	14356.19	14649.19	13927.47
TC1000002642.mm.1	Dram1	783.53	756.42	734.73	1431.87	1593.64	1533.26
TC1600000487.mm.1	Mylk	44.84	46.15	44.09	83.66	76.29	81.03
TC1600000328.mm.1	Kng1	27.18	28.67	29.86	161.36	127.02	164.74
TC0300000500.mm.1	Mme	43.84	43.03	41.36	119.75	110.84	132.65
TC1400001914.mm.1	Bmp4	39.79	37.79	40.01	74.59	81.98	82.57
TC0300000137.mm.1	Cp	31.82	35.21	33.92	63.42	63.77	60.94
TC0200000245.mm.1	Mrc1	2831.78	2832.35	2544.86	10085.04	8993.53	11118.44
TC0500001023.mm.1	Ibsp	31.77	30.89	36.49	1091.46	647.88	1112.02
TC0600001313.mm.1	Cxcl12	120.58	105.87	109.10	1582.05	1028.13	1448.17
TC1100001661.mm.1	Tmem106a	4619.78	4594.20	4368.81	9905.37	8966.62	10203.21
TC1000001011.mm.1	1500009L16Rik	29.99	36.47	38.87	234.26	187.72	203.95
TC1400002641.mm.1	Ednrb	19.83	19.90	20.61	84.27	70.04	91.33
TC1700002352.mm.1	C3	107.84	101.49	95.99	337.05	286.19	340.32
TC0900003344.mm.1	Trf	590.99	521.62	539.40	1248.44	1159.94	1261.67
TC1000001066.mm.1	Igf1	3315.77	3677.55	3127.98	7797.96	7650.88	7747.76
TC0700004632.mm.1	Pira1	4098.69	3962.30	3524.68	11468.25	10415.96	11983.05
TC0400002603.mm.1	Coro2a	73.08	68.94	69.49	107.33	105.19	102.18
TC1000002383.mm.1	Gstt1	27.68	24.78	23.84	65.82	59.72	62.50
TC1900001419.mm.1	Lipa	35333.44	33519.43	32715.60	60560.57	56154.83	59712.43
TC0400000499.mm.1	Tmod1	18.48	20.29	20.05	33.79	33.35	34.92
TC0600001530.mm.1	Clec2d	7440.41	7018.78	7113.31	14724.97	16937.41	16666.21
TC0200004843.mm.1	Acss1	60.34	61.89	57.85	106.26	98.50	99.33
TC0700002696.mm.1	Zfp36	210.36	222.16	216.59	339.89	338.67	319.51
TC1700001993.mm.1	H2-T24	116.77	108.17	101.26	224.97	236.44	225.14
TC1000001133.mm.1	Hal	4910.36	5215.57	4698.25	10146.07	9848.45	10954.44
TC1_GL456211_random00000019.mm.1		2957.96	2710.23	2849.97	4898.99	4650.24	4942.53
TC0700000501.mm.1	Gmfg	142.88	135.57	147.17	280.78	263.04	294.46
TC0700004571.mm.1	Igf2	25.40	22.50	27.29	126.85	99.46	116.71
TC0600003337.mm.1	Arhgdib	480.75	475.80	463.51	872.74	781.78	832.09
TC0900000051.mm.1	Mmp27	48.74	43.71	48.88	87.59	90.74	87.48
TC1600000331.mm.1	Adipoq	21.32	20.88	20.34	57.80	57.44	48.52
TC0300000647.mm.1	Sfrp2	93.03	100.74	89.72	239.40	273.26	286.61
TC0300001489.mm.1	Mcoln2	100.01	100.99	108.31	290.99	273.37	336.87
TC1100002983.mm.1	Myocd	24.29	24.64	25.05	33.71	32.33	34.17
TC0200002089.mm.1	Ism1	19.05	18.72	20.44	31.46	33.47	32.96
TC0800001085.mm.1	Gnao1	40.04	40.59	41.78	61.49	57.63	61.18
TC0200003164.mm.1	Tmem141	576.87	572.19	593.76	1092.22	1020.68	1165.95
TC1200000796.mm.1	2410016O06Rik	21.55	23.41	22.53	34.15	35.17	33.64
TC1700002152.mm.1	Slc29a1	291.54	286.80	284.85	680.33	587.23	692.82
TC0100000927.mm.1	Sned1; Mir6901	34.65	30.64	35.23	86.27	82.01	95.47
TC0X00001682.mm.1	Gpm6b	38.77	39.82	46.23	100.48	107.90	110.49
TC0700002169.mm.1	Cox6b2	76.28	75.12	72.18	103.64	99.32	101.99
TC0700004634.mm.1	Gm14548; Pira11; Pira2; Pira4; Pira7; Pira1	4223.96	3809.64	3448.11	10616.74	9744.38	10792.14
TC0700000030.mm.1	Pira6	3999.00	3904.06	3394.60	10452.98	9516.87	11073.08
TC0700000979.mm.1	Mcee	3714.07	3421.46	3497.83	5289.44	5389.13	5550.95
TC0900002007.mm.1	Dcps	407.02	403.31	419.21	593.02	640.86	599.87
TC1100002003.mm.1	Rnf213	2018.59	2148.87	1912.33	3641.20	3501.92	3763.08
TC1600000447.mm.1	Tfrc	545.10	589.96	571.83	1238.03	1086.88	1249.16
TC0200004671.mm.1	Pcna	5438.75	5222.50	5018.52	9395.91	8878.92	9949.52
TC0100000718.mm.1	Ccl20	42.59	37.99	41.94	68.58	70.29	69.57
TC1700002151.mm.1	Hsp90ab1	43500.46	41474.56	42843.60	55974.38	54114.23	55901.65

TC1800001712.mm.1	Cndp2	288.59	295.48	262.57	473.60	480.67	480.93
TC0300000656.mm.1	Tmem154	344.54	337.23	375.93	1381.29	1125.01	1500.75
TC1400002836.mm.1	Dok2	77.55	78.88	73.86	191.84	162.78	194.37
TC0200001546.mm.1	Lmo2	130.75	142.31	138.91	203.40	201.37	209.62
TC0100002645.mm.1	Serpine2	286.50	308.73	281.17	663.73	582.03	676.53
TC1200000236.mm.1	Adi1	154.88	159.62	147.97	213.76	221.86	219.06
TC1100000284.mm.1	Efemp1	34.05	26.42	33.23	236.65	174.05	248.87
TC1700000402.mm.1	Tmem8	959.42	947.44	834.69	2263.81	2258.82	2646.38
TC0400001557.mm.1	Laptm5	8161.47	7828.66	7840.16	10321.69	10396.87	10823.62
TC1200001333.mm.1	Gm6768	516.54	482.42	526.43	821.41	886.21	898.50
TC0900000950.mm.1	Aldh1a2	47.42	54.96	53.00	105.06	98.53	105.59
TC1900000552.mm.1	Plce1	43.36	45.36	41.27	85.22	76.04	84.64
TC0Y00000551.mm.1	Gm21860	1102.99	1055.90	832.70	5654.14	7391.50	8208.71
TC0Y00000552.mm.1	Gm21748	1102.99	1055.90	832.70	5654.14	7391.50	8208.71
TC0900000046.mm.1	Mmp12	95.06	92.01	85.30	154.91	153.12	145.81
TC0200004245.mm.1	Tcp11l1	116.05	113.26	116.58	182.25	166.49	169.24
TC1000002380.mm.1	Susd2	24.68	25.23	28.57	75.41	62.97	67.02
TC0800003069.mm.1	Mvd	110.17	104.38	110.51	146.41	146.32	152.36
TC0200001523.mm.1	Pamr1	37.43	30.70	33.69	94.50	97.43	113.24
TC0900000044.mm.1	Mmp13	5351.20	5692.61	4566.34	17310.93	16476.97	20272.54
TC0300002374.mm.1	S100a1	7294.78	7822.95	7221.68	15090.51	15579.82	13525.65
TC1500001531.mm.1	Enpp2	18.27	16.85	18.49	29.83	28.02	28.44
TC1300001377.mm.1	Akr1c18	17.92	23.04	20.08	240.29	166.39	288.23
TC1500000344.mm.1	Emc2	29018.63	30806.60	26680.60	60238.57	57653.28	65196.68
TC0200001859.mm.1	Sqrdl	271.05	248.10	251.55	377.17	391.20	391.07
TC1000000895.mm.1	Mfsd12	1015.16	1036.71	893.01	3471.33	2886.83	3759.47
TC1300000147.mm.1	Sfrp4	36.21	37.41	36.30	72.47	69.27	82.28
TC1000000903.mm.1	Aes	9486.39	9209.54	8982.30	13256.99	12427.40	13008.08
TC0500001240.mm.1	Oasl1	146.32	134.51	135.09	357.97	297.04	351.58
TC0900001297.mm.1	Slco2a1	159.14	157.58	143.52	299.15	268.08	281.16
TC1700001954.mm.1	Tnf	1773.38	1755.51	1500.68	3417.16	3555.76	3558.04
TC1000000729.mm.1	Chchd10	46.24	44.86	44.01	95.60	82.25	96.28
TC0600001890.mm.1	Tfec	793.16	749.46	682.19	1941.61	1684.05	2017.84
TC0700002635.mm.1	Ceacam1	84.29	86.59	85.14	185.93	201.63	233.58
TC1400000513.mm.1	Lgals3	124.15	126.37	130.15	280.98	234.36	260.72
TC1000000718.mm.1	Ggt5	26.68	22.36	23.57	51.27	48.27	49.11
TC0700004351.mm.1	Pycard	52.67	49.10	53.73	83.59	79.70	86.64
TC0700004516.mm.1	Ifitm6	153.07	150.47	147.94	835.03	570.09	800.35
TC1000001189.mm.1	Dcn	48.53	47.03	45.75	80.62	78.52	89.57
TC0800000711.mm.1	Atp6v1b2	1993.71	2026.75	1837.81	3333.70	3072.22	3258.01
TC0300000419.mm.1	Dclk1	44.20	40.12	43.56	78.18	76.90	87.17
TC0500003219.mm.1	Camkk2	81.81	83.22	85.40	145.67	132.20	151.04
TC0700001475.mm.1	Olfr583	10.10	10.97	10.33	16.75	16.86	15.60
TC1600000746.mm.1	Abi3bp	112.83	119.64	102.02	343.54	274.85	310.58
TC1400002098.mm.1	Tgm1	38.59	42.27	40.98	73.77	66.62	74.41
TC0100001597.mm.1	Cd48	1957.40	1906.77	1927.96	3629.02	3283.24	3874.20
TC0500001584.mm.1	Por	254.12	242.16	232.04	474.36	434.25	508.00
TC1700002313.mm.1	Plin4	17.03	18.23	18.50	28.01	28.05	30.49
TC1200001602.mm.1	Prkar2b	143.89	140.73	131.30	362.21	321.65	413.00
TC1300001326.mm.1	Emb	479.59	412.30	404.92	1127.86	962.17	1041.24
TC1100003397.mm.1	Ccl9	2758.89	2748.91	2223.95	7313.62	6559.46	6636.44
TC1100004227.mm.1	Stra13	2418.72	2585.65	2416.26	3825.32	4036.30	3666.13
TC0500000850.mm.1	Cxcl2	128.69	125.67	114.03	200.14	206.63	195.87
TC0700004635.mm.1	Lilra6	337.91	285.33	297.69	653.31	592.62	630.81
TC0500001648.mm.1	Nxpe5	343.68	365.67	333.92	1057.27	816.52	997.53
TC1100003055.mm.1	Cd68	284925.30	288164.60	273898.90	407490.20	379769.20	408070.80
TC1400001785.mm.1	Fam213a	74.13	76.43	70.64	117.70	112.21	107.52
TC1100003779.mm.1	Dhx58	228.45	213.69	195.78	402.66	376.02	409.56
TC0500003180.mm.1	Oas1g	577.39	603.90	529.05	982.69	1052.29	1096.01
TC0600001639.mm.1	Mgst1	101.14	98.03	96.10	179.73	156.06	174.36
TC0500001251.mm.1	Pop5	68.67	68.26	70.91	89.45	90.86	95.77
TC0400001639.mm.1	Stmn1	177.99	181.58	176.64	259.55	241.18	265.28
TC1200001974.mm.1	Tmem229b	90.11	96.06	90.98	121.76	122.56	127.67
TC0600002996.mm.1	Ret	56.65	44.97	50.18	148.08	133.05	165.06
TC0700004285.mm.1	Cln3	288.50	286.23	264.77	404.75	396.33	418.68
TC1500001730.mm.1	Ly6c2	64474.05	61439.24	54010.53	111287.70	114231.70	116978.50
TC0700003942.mm.1	Trim30c	31.77	31.59	31.85	39.98	42.66	43.08
TC0700003464.mm.1	Arrdc4	45.99	50.26	50.23	84.00	77.77	87.33
TC0300000880.mm.1	Selenbp1	48.52	48.00	49.39	65.06	60.67	63.20
TC0500002895.mm.1	Gbp10; Gbp6	56.33	52.92	52.79	105.47	94.51	90.78
TC0800000707.mm.1	Lpl	61.94	56.52	49.83	342.51	228.60	323.82
TC0600001279.mm.1	Pparg	99.97	104.90	100.55	306.34	265.41	366.87
TC0200002869.mm.1	Mcm10	52.79	53.13	52.40	75.40	71.01	78.66
TC0200005290.mm.1	Zbp1	485.79	480.79	460.28	860.79	881.28	1022.15

TC1700001299.mm.1	Epas1	237.68	234.89	213.31	781.86	638.51	892.50
TC1300000838.mm.1	Fastkd3	80.34	84.74	79.94	104.68	109.66	109.14
TC1100001875.mm.1	Ttyh2	5156.21	5084.81	4536.89	7962.78	8150.79	7883.92
TC0500001382.mm.1	Hvcn1	54.05	48.09	45.19	129.76	109.01	130.48
TC1500000580.mm.1	Grina	10905.96	11445.36	9783.24	18507.93	18226.37	18943.33
TC0800003068.mm.1	Cyba	10333.51	9714.65	8908.57	18380.49	17121.23	19294.06
TC1100000822.mm.1	Pmp22	39903.70	37430.43	35509.57	55413.50	56524.91	57425.10
TC1100001156.mm.1	Aldoc	37.02	38.08	33.53	62.76	62.72	69.23
TC0700000242.mm.1	Ptgir	23.83	25.77	25.08	54.43	53.23	66.21
TC1700000652.mm.1	Neu1	44.82	44.17	41.89	57.67	60.34	61.24
TC0600003084.mm.1	Clec4e	634.84	660.15	610.08	1469.92	1245.40	1550.40
TC1500000002.mm.1	Sepp1	1222.30	1254.36	1231.42	2006.12	1776.50	1960.32
TC0400001677.mm.1	Id3	56.96	59.39	65.47	128.04	110.57	118.82
TC0600003108.mm.1	C1s1	16.08	17.20	17.76	50.61	47.78	65.52
TC1200001747.mm.1	Sptssa; Mir1892	1380.31	1291.08	1237.19	2080.20	2320.17	2299.79
TC0800000645.mm.1	Aadat	15.33	13.82	14.51	21.38	20.64	21.66
TC0500002919.mm.1	Tgfbr3	160.43	145.68	137.92	384.68	311.42	348.84
TC0500001796.mm.1	Rasl11a	26.05	27.53	30.53	51.86	59.10	57.90
TC0X00003113.mm.1	Chrdl1	49.67	48.21	50.19	67.22	64.82	70.67
TC1100001634.mm.1	Atp6v0a1	2202.75	2164.10	2150.48	4186.57	3723.44	4534.12
TC1900000767.mm.1	Adra2a	39.11	36.01	38.92	67.61	59.41	65.26
TC0600002644.mm.1	Anxa4	4963.88	4489.11	4477.51	7295.11	7275.09	7893.07
TC1200002411.mm.1	Ckb	137.91	138.40	133.42	201.06	189.29	211.99
TC1200001047.mm.1	Serpina3n	18.63	18.77	18.11	32.16	28.90	28.06
TC0200001645.mm.1	Olfr1284	11.66	12.14	11.90	16.73	15.39	15.90
TC0X00001934.mm.1	Cfp	10866.63	10177.36	9579.14	17170.61	16207.47	15874.72
TC0500003181.mm.1	Oas1a	2436.91	2576.03	2215.69	4883.67	4468.66	5247.54
TC1900001729.mm.1	Shtn1	161.86	153.94	154.26	276.83	239.18	263.27
TC0400004097.mm.1	Kcnab2	113.12	104.99	99.23	171.38	161.53	168.43
TC1900000501.mm.1	Ifit3	138.04	106.71	133.29	340.70	320.68	375.35
TC0600000392.mm.1	Rab19	36.72	34.34	34.90	57.77	65.11	68.34
TC0600001229.mm.1	Lmcd1	53.01	44.64	47.18	150.22	142.88	196.33
TC0600001092.mm.1	Slc6a6	18618.06	19054.49	17135.52	27600.63	27285.84	29395.90
TC0700000786.mm.1	Klk1b11	21.23	21.60	20.92	60.75	45.58	55.67
TC0700001630.mm.1	Adm	79.43	85.34	92.70	460.27	339.08	559.01
TC0700003943.mm.1	Trim30a	1362.87	1429.87	1218.44	2284.30	2253.67	2396.70
TC1400001054.mm.1	Lcp1	5938.32	6089.53	5911.61	8440.17	7732.04	8338.16
TC1300000157.mm.1	Aoah	670.45	814.91	657.26	2043.86	1732.66	2150.01
TC1200002525.mm.1	Serpina3g	36.89	35.69	45.75	99.21	90.85	93.52
TC1700000833.mm.1	Adgrf5	89.49	81.65	72.04	160.58	161.91	172.37
TC0800000096.mm.1	Arhgef7	360.35	343.38	350.12	549.99	496.26	557.30
TC0400001259.mm.1	Prdx1	1049.19	1034.43	971.48	1390.57	1349.67	1432.69
TC0800000172.mm.1	Agpat5	571.92	575.40	586.02	828.88	813.37	912.27
TC1100002649.mm.1	Tgtp2	99.01	117.52	102.88	253.64	246.86	308.62
TC0800001112.mm.1	Cpne2	765.63	705.71	676.53	1400.20	1207.34	1290.58
TC0200001851.mm.1	Gm14085	104.41	105.94	106.69	150.20	144.04	161.63
TC0X00000961.mm.1	Gdpd2	22.97	23.17	25.06	36.17	35.01	33.34
TC0700004610.mm.1	Ccnd1; Mir3962	1600.22	1573.93	1451.66	2711.20	2707.93	3148.82
TC0600002917.mm.1	Camk1	264.98	253.77	254.93	332.89	340.52	361.31
TC0100003743.mm.1	Mark1	96.81	91.30	90.73	244.42	195.22	258.49
TC0100000524.mm.1	Adam23	196.56	180.55	163.85	349.29	342.00	391.92
TC0700004633.mm.1	Gm15448	2914.36	2672.94	2410.64	6443.61	5359.24	6250.95
TC1000000784.mm.1	Cstb	40918.73	43648.63	44011.80	64018.78	60815.11	67921.94
TC0200000037.mm.1	Phyh	440.06	402.53	391.73	657.68	632.39	606.44
TC1400001709.mm.1	Wdfy4	143.98	139.40	130.16	248.05	219.08	253.96
TC0X00000126.mm.1	Tspan7	48.32	54.40	49.05	74.30	76.50	78.74
TC0500003395.mm.1	Hip1	341.73	335.52	291.83	631.46	590.76	689.69
TC0700003592.mm.1	Man2a2	206.43	188.27	180.64	410.33	347.92	418.33
TC1700002814.mm.1	C4b	51.67	56.68	51.59	99.41	85.18	93.59
TC1300001806.mm.1	Lyrm4	568.37	538.97	609.66	867.48	822.55	856.26
TC1100004282.mm.1	Mfsd11	643.05	643.17	585.65	897.38	859.46	906.49
TC0500003284.mm.1	Slc15a4	544.37	527.54	532.03	780.08	769.68	873.33
TC0500002411.mm.1	Lgi2	21.86	18.09	24.91	68.31	56.75	66.24
TC1500002246.mm.1	Smagp	52.90	49.45	49.50	73.53	77.13	69.50
TC1700000694.mm.1	Cdsn	63.12	72.92	71.45	135.18	169.30	150.79
TC1300002047.mm.1	Hk3	367.40	364.20	338.86	698.45	618.50	765.54
TC0500003176.mm.1	Oas3	208.93	194.80	162.34	488.51	415.17	476.77
TC0900003062.mm.1	Ndufaf3	181.29	184.99	173.29	279.24	249.94	277.36
TC1700000936.mm.1	B430306N03Rik	94.38	76.13	86.72	228.45	183.75	203.24
TC0800002824.mm.1	Atp6v0d1	16994.91	15394.26	14413.85	24682.17	25695.04	25581.53
TC1800001197.mm.1	Cd14	1362.47	1217.04	1132.36	2297.69	2148.81	2400.09

TC0600003335.mm.1	Mgp	6368.95	4969.65	6598.70	36534.67	25115.00	42110.59
TC1100001592.mm.1	Igfbp4	1812.56	1583.46	1498.41	3967.77	3281.73	3967.56
TC1200000225.mm.1	Cmpk2	87.53	104.40	101.22	204.38	183.51	178.90
TC1600001280.mm.1	Sdf2l1	3665.82	2855.60	2989.53	8738.84	7331.17	7548.07
TC1800001744.mm.1	Arhgap26	34.71	35.68	33.76	45.12	49.50	46.59
TC1100002155.mm.1	Pold2	245.50	238.55	230.35	310.67	300.36	322.80
TC1100001586.mm.1	Cdc6	41.48	40.90	41.77	58.90	53.88	60.18
TC1600001134.mm.1	Coro7	185.17	169.33	167.59	319.05	274.12	313.26
TC1700000351.mm.1	Msrb1	1775.21	1689.35	1587.65	2909.83	2554.15	2861.72
TC1200001110.mm.1	Evl	346.03	338.30	317.36	500.31	491.16	553.53
TC0100002324.mm.1	Mfsd6	300.56	333.64	284.95	543.34	491.55	540.76
TC1900000175.mm.1	Pcna-ps2	51818.63	45230.48	41434.75	89982.54	90489.31	100814.90
TC0900001243.mm.1	Faim	77.99	85.50	81.01	107.34	106.00	110.76
TC0900002113.mm.1	Sorl1	36.43	36.11	36.52	51.34	47.49	53.50
TC0200004363.mm.1	Grem1	115.45	96.26	78.88	269.82	310.72	296.48
TC1300000543.mm.1	Rnf144b	56.36	50.48	50.29	85.90	88.81	99.79
TC0200001036.mm.1	Hat1	213.53	198.41	204.29	351.64	302.48	340.12
TC0700002002.mm.1	Paox	71.99	67.94	72.82	95.52	104.29	96.53
TC1300001155.mm.1	Cd180	5792.56	5677.77	5350.12	9185.86	8428.32	9891.94
TC0200003146.mm.1	Dpp7	770.89	686.29	672.22	1212.45	1165.48	1337.99
TC0800002757.mm.1	Slc38a7	248.48	229.43	225.31	430.91	421.32	515.29
TC1700002815.mm.1	C4a	26.22	27.13	26.76	39.67	35.44	36.50
TC1500001431.mm.1	Lrp12	1207.07	1124.53	994.22	1913.09	1857.87	1938.66
TC0500001992.mm.1	Cd36	7007.11	6924.82	6333.20	10640.58	10049.59	11440.19
TC1000002453.mm.1	Pdxk	419.22	417.62	349.41	853.21	833.64	1024.62
TC0400002251.mm.1	Ripk2	1209.38	1248.85	1134.73	1638.97	1592.51	1701.70
TC0200002223.mm.1	Cst7	41.01	47.25	42.75	94.78	112.69	130.34
TC0300002716.mm.1	Ahcyl1	1857.40	2014.78	1764.93	2674.11	2697.13	2754.61
TC0500001380.mm.1	Ppp1cc	15095.44	14759.76	13687.05	19234.77	19000.14	19146.55
TC1200000838.mm.1	Batf	51.46	51.19	52.34	85.73	89.29	105.40
TC0700002617.mm.1	Atp1a3	281.22	259.60	242.68	545.37	452.29	489.25
TC0100003528.mm.1	Sdhc	1903.49	1752.76	1699.60	2917.97	2648.75	3000.38
TC0200001365.mm.1	Rapsn	27.83	23.67	27.17	50.22	56.65	47.41
TC0800000937.mm.1	Asf1b	1431.52	1525.55	1357.50	2385.49	2205.08	2557.60
TC0600003212.mm.1	Clec2j	46.56	49.31	47.53	77.20	69.38	81.41
TC0800002799.mm.1	Cmtm4	206.56	205.46	182.93	287.24	284.98	283.95
TC0300002722.mm.1	Gstm7	38.91	41.12	37.17	51.05	51.86	50.97
TC1_GL456221_random00000014.mm.1		3014.00	2670.37	2853.86	4932.95	4301.14	4755.67
TC0700004487.mm.1	Adam8	6513.21	6458.44	5702.93	9981.24	9745.09	10866.75
TC1100001264.mm.1	Wfdc17	48681.30	51577.43	54056.39	119932.60	94011.02	101579.40
TC0800001182.mm.1	Cmtm3	11809.29	11192.32	10191.24	17907.03	18020.20	20139.79
TC1500001596.mm.1	E430025E21Rik	6439.27	6586.43	5872.91	9250.05	9002.94	9834.31
TC1900001418.mm.1	Ch25h	54213.13	49076.66	46993.86	79897.03	77574.75	86620.56
TC1300002506.mm.1	Tmem171	69.51	64.83	70.01	141.20	139.12	114.34
TC1000001506.mm.1	Avpr1a	85.20	78.14	90.95	129.79	130.56	140.08
TC0300001409.mm.1	Adh1	19.76	18.47	20.35	32.13	36.32	39.69
TC0600000765.mm.1	Ndnf	45.65	42.17	39.97	76.31	66.27	75.68
TC0100000766.mm.1	Sp100	761.58	828.56	740.80	1158.39	1074.96	1161.86
TC0900002823.mm.1	Tbc1d2b	430.09	440.56	396.12	618.85	646.22	702.85
TC1100002567.mm.1	Pttg1	118.21	109.37	103.91	165.89	185.95	169.85
TC1300000016.mm.1	Akr1c14	17.78	16.80	16.65	21.71	21.77	20.77
TC0600003503.mm.1	Herc6	218.99	220.77	198.76	293.20	297.21	285.38
TC0200003354.mm.1	Phf19	71.14	65.68	67.78	88.23	90.29	95.48
TC1400002067.mm.1	Slc7a8	4880.06	5023.98	4206.83	8203.25	7596.59	8034.66
TC1100003574.mm.1	Tmem92	21.04	21.25	21.40	29.00	27.65	26.20
TC0600002056.mm.1	Wdr91	377.46	383.68	375.18	524.07	492.98	559.13
TC0100002827.mm.1	Kif1a	96.51	92.89	89.74	153.31	135.12	158.98
TC0100002285.mm.1	Slc40a1	25.26	25.48	23.86	54.10	43.83	57.02
TC1600001071.mm.1	Ets2	122.68	119.53	106.61	209.25	181.49	196.36
TC0200000477.mm.1	Rxra	416.19	393.16	380.16	521.78	517.06	551.91
TC0700003990.mm.1	Tpp1	9902.88	9368.83	7987.44	16292.25	16514.70	18051.12
TC1900001030.mm.1	Fermt3	400.40	391.10	372.25	620.82	542.92	617.74
TC0X00001533.mm.1	Hsd17b10	1309.96	1380.56	1189.42	2137.41	1930.59	2056.07
TC0100003738.mm.1	42430	22.65	20.16	23.40	47.27	49.44	39.35
TC1900000502.mm.1	Ifit3b	85.72	66.66	84.62	207.00	231.97	178.81
TC1600001387.mm.1	Kng2	71.66	79.26	62.57	157.65	144.80	181.57
TC0200000888.mm.1	Pkp4	203.78	199.69	190.50	329.66	281.79	320.33
TC1400000968.mm.1	Stc1	73.15	74.39	79.91	146.10	171.02	200.29
TC0600000577.mm.1	Gpnmb	145687.50	147499.40	127327.60	219215.30	238993.80	250499.80
TC0800001511.mm.1	Dpep1	182.55	182.43	164.81	283.25	257.79	297.22
TC0500003573.mm.1	Tecpr1	32.23	30.93	31.67	44.68	39.97	43.09
TC0400001856.mm.1	Dhrs3	4636.91	4546.09	4305.48	6086.68	5643.09	6019.29

TC1900000102.mm.1	Batf2	44.10	43.47	43.52	63.38	55.95	61.48
TC0800000564.mm.1	Dctd	64.89	65.58	64.47	90.62	81.07	85.38
TC0700002102.mm.1	Dhcr7	92.28	89.46	88.91	132.87	119.86	137.69
TC1100001071.mm.1	Scarf1	54.61	55.38	54.20	85.52	74.41	85.86
TC0200005181.mm.1	Znfx1	340.82	336.63	314.84	499.76	446.34	496.82
TC0500003404.mm.1	Tmem120a	142.43	142.14	144.10	193.85	187.97	213.58
TC0900001048.mm.1	Gsta4	124.31	142.73	122.87	271.07	226.46	280.16
TC1700000214.mm.1	Fpr2; Fpr3	38.71	44.37	43.18	65.81	77.13	77.09
TC1700001522.mm.1	Fpr1	29.79	26.85	31.90	66.60	52.81	57.83
TC0400002744.mm.1	Ptgr1	239.19	235.54	237.26	320.88	291.49	322.01
TC1700000657.mm.1	Lsm2	90.23	85.78	86.97	130.41	116.11	132.51
TC1000003213.mm.1	Lilr4b	74869.23	82578.41	74675.27	124391.50	109173.80	120754.00
TC1100003807.mm.1	Vat1	174.06	176.26	156.03	305.98	257.84	289.16
TC0100003889.mm.1	Mnda	2602.24	2718.34	2443.70	3910.60	3527.92	3916.89
TC0900002724.mm.1	Mb21d1	58.24	54.43	57.84	83.97	74.51	79.62
TC1600000367.mm.1	Tprg	12.84	12.68	12.94	18.41	21.35	18.36
TC0200001849.mm.1	Slc28a2	176.07	171.68	163.72	234.81	226.38	254.91
TC0700003831.mm.1	P2ry2	248.26	236.95	221.58	363.32	334.13	383.72
TC0700001624.mm.1	Wee1	298.08	279.85	266.76	375.25	384.33	405.58
TC1200001544.mm.1	Id2	6563.60	6590.09	5305.90	10961.40	10880.67	11216.55
TC0400002873.mm.1	Tmem261	1422.08	1427.42	1542.43	3269.83	2497.82	2846.53
TC1400002869.mm.1	Phf11d; Phf11c	480.10	422.05	426.46	1204.26	886.71	1210.44
TC0700000458.mm.1	Blvrb	490.34	428.33	392.88	994.20	805.74	967.79
TC1700002225.mm.1	Nfya	181.61	171.44	179.58	240.66	219.68	236.99
TC0700002143.mm.1	Lair1	512.06	508.95	473.39	757.74	671.85	756.34
TC1900001551.mm.1	Erlin1	471.13	475.06	449.90	653.63	586.20	622.46
TC0600001371.mm.1	Slc6a12	31.62	29.99	30.97	38.67	43.13	41.52
TC0700000853.mm.1	Bcat2	139.20	134.05	125.76	171.80	181.97	185.71
TC0300001244.mm.1	Gclm	1190.97	1096.69	1054.63	2243.60	1831.32	1896.77
TC1100000094.mm.1	Zmiz2	371.44	368.24	344.00	529.66	472.86	518.48
TC1600001770.mm.1	St3gal6	35.07	31.19	32.54	42.90	44.26	44.50
TC1000002714.mm.1	Plxnc1	1280.80	1265.38	1149.64	1938.41	1735.54	1982.50
TC1500000667.mm.1	Polr2f	58150.89	55365.24	56122.91	79645.09	71599.54	79894.39
TC1800001198.mm.1	Ndufa2	2713.35	2789.33	2490.76	4055.73	3671.54	4119.96
TC0900001781.mm.1	Panx1	54.21	60.61	60.13	83.63	77.88	81.27
TC1400002160.mm.1	Gm6904	134.31	149.80	168.98	259.28	275.81	254.52
TC0X00001371.mm.1	Rnf128	901.65	823.50	715.54	1584.86	1416.48	1660.86
TC1700001154.mm.1	Ehd3	64.31	66.27	60.31	95.02	84.99	88.21
TC1100002643.mm.1	Gm12185; Tgtp1	67.20	66.60	63.30	133.10	104.65	129.42
TC0400003900.mm.1	Kazn	27.82	25.94	27.46	38.35	36.52	34.46
TC1900001770.mm.1	Taf6l	58.05	54.05	53.72	71.00	69.70	75.06
TC1000001216.mm.1	Kitl	67.41	55.28	55.62	227.91	151.61	234.19
TC0500002280.mm.1	Nsg1	286.00	294.90	263.64	438.29	459.08	530.46
TC1800001307.mm.1	Cdo1	94.98	116.68	132.17	284.20	242.33	263.24
TC0100003531.mm.1	Pcp4l1	71.63	92.84	102.84	225.60	204.89	205.68
TC1400001906.mm.1	Ddhd1; Mir5131	166.36	171.25	167.65	221.74	202.28	220.62
TC1600001287.mm.1	Pi4ka	955.74	966.13	854.56	1450.56	1328.01	1523.23
TC0900000748.mm.1	Hexa	67568.25	60810.41	60343.95	87673.84	85682.05	93274.27
TC1100002046.mm.1	Hmga1-rs1	726.13	666.15	702.79	1737.43	1277.89	1714.11
TC0100003320.mm.1	Tor3a	260.13	245.19	220.61	421.02	370.97	431.00
TC0600003114.mm.1	Ptpn6	736.34	733.28	655.83	1030.14	955.88	1021.90
TC0100002965.mm.1	Steap3	79.57	77.34	71.33	125.36	113.58	108.42
TC0200002298.mm.1	Id1	74.33	64.57	67.06	94.43	95.41	97.10
TC0200000714.mm.1	Olfml2a	16.55	16.45	18.00	28.23	33.91	27.50
TC1300000336.mm.1	Dusp22	114.78	107.99	102.54	146.73	161.44	149.24
TC1300002606.mm.1	Rgs7bp	31.86	31.33	30.19	65.38	50.15	61.43
TC1400000340.mm.1	Ncoa4	283.15	282.22	242.48	446.31	402.97	426.13
TC0600000277.mm.1	Mest	17.96	17.86	19.13	25.72	23.42	26.08
TC0400001382.mm.1	Ppt1	2414.32	2430.32	2160.72	3316.70	3141.31	3376.16
TC0600002225.mm.1	Rarres2	35.56	35.66	31.73	54.31	58.57	66.78
TC0800000846.mm.1	Mcm5	436.17	423.26	339.71	764.19	722.67	799.58
TC1100000531.mm.1	Rnf130	539.95	568.46	527.75	777.83	716.46	818.47
TC1100003799.mm.1	Coa3	1051.14	1031.79	972.70	1524.60	1426.96	1684.79
TC1400001671.mm.1	Stab1	6877.69	6705.66	5938.10	10925.66	9898.19	11662.05
TC0400001039.mm.1	Lepr	17.96	18.71	19.48	24.55	22.92	24.35
TC0200005112.mm.1	Slpi; Mir7678	44.43	44.89	51.86	104.60	93.84	81.28
TC1700000842.mm.1	Enpp5	32.19	33.04	35.28	81.20	66.85	98.49
TC1900001769.mm.1	Tmem179b	3578.14	3366.35	3231.05	4814.41	4437.55	4917.62
TC0600001424.mm.1	C1rb	80.65	86.07	65.41	174.82	147.02	165.62
TC0500003236.mm.1	Vps33a	407.27	375.93	333.61	605.08	602.72	673.33
TC0800000955.mm.1	Cacna1a	227.34	225.94	209.04	367.86	319.12	384.97

TC0200004776.mm.1	Ralgapa2	68.91	71.84	64.96	93.54	97.06	106.29
TC1100001785.mm.1	Milr1	2283.84	2430.85	2077.77	3391.79	3307.51	3674.88
TC1500000787.mm.1	Parvg	220.69	204.38	190.81	318.22	299.19	344.19
TC1100004000.mm.1	Abca9	61.35	64.20	60.08	113.44	92.69	114.16
TC1100003466.mm.1	Gdpd1	844.32	717.95	680.05	1458.18	1294.75	1576.81
TC1500000797.mm.1	Nup50	1893.06	1850.01	1898.01	2360.01	2531.04	2691.06
TC0700001815.mm.1	Gdpd3	35.52	38.36	30.70	66.12	58.71	69.25
TC0200005097.mm.1	Ada	398.29	397.28	344.17	579.41	543.38	585.39
TC0200003147.mm.1	Uap1l1	223.85	207.95	188.04	388.07	349.42	441.76
TC1000003019.mm.1	Irak3	244.33	228.51	224.28	311.25	305.72	288.62
TC1600000662.mm.1	Cd200r1	150.83	141.32	133.10	250.08	224.01	282.02
TC0700000527.mm.1	Ppp1r14a	70.36	71.69	70.73	138.09	113.96	151.31
TC0400001091.mm.1	Plpp3	222.70	189.16	163.10	393.39	370.25	416.28
TC0600001369.mm.1	Usp18	3985.69	3772.92	3217.55	5818.47	6074.19	6096.24
TC1700000148.mm.1	Sod2	101.33	100.12	89.12	133.96	139.73	147.20
TC1000002467.mm.1	Plpp2	1925.38	1852.72	1627.04	2689.28	3043.35	2934.25
TC0500003064.mm.1	Gltp	2937.46	2977.92	2825.38	5194.23	4286.28	5294.47
TC1300001954.mm.1	1110007C09Rik	377.71	349.07	324.89	620.55	530.83	631.10
TC1100000989.mm.1	Xaf1	2026.86	1984.48	1940.05	2609.33	2414.84	2689.27
TC0600001397.mm.1	Clec4a1	455.57	476.96	399.26	1390.66	952.37	1375.57
TC0200002270.mm.1	Angpt4	95.78	84.69	86.01	118.59	126.65	122.42
TC1300001580.mm.1	Vmn1r189	12.39	12.37	14.01	18.72	19.94	18.02
TC0500003376.mm.1	Lat2	995.82	937.57	971.51	1436.82	1509.96	1763.53
TC0200005314.mm.1	Ctsz	96208.13	84942.61	84079.62	132156.00	141036.10	155873.20
TC1200001861.mm.1	Pole2	132.68	143.21	133.25	188.37	172.71	191.34
TC1800000301.mm.1	Egr1	108.30	97.61	95.60	140.87	133.74	135.57
TC0100003264.mm.1	Rgl1	97.09	94.13	89.53	130.88	117.96	124.13
TC1_GL456210_random00000004.mm.1		3507.40	3065.45	3186.69	5462.76	4689.74	5348.28
TC1100003106.mm.1	Slc25a11	2081.22	1937.47	1883.08	2626.43	2490.72	2697.99
TC1500000967.mm.1	Tmem106c	225.33	229.61	186.10	401.80	357.77	420.79
TC0100000892.mm.1	Scly	76.50	83.77	72.29	144.83	119.74	144.12
TC0500000785.mm.1	Stap1	297.37	307.36	259.79	448.77	436.64	492.96
TC0600001422.mm.1	C1ra	82.60	85.29	67.72	152.54	135.78	160.75
TC0200005015.mm.1	Rbl1	358.49	371.77	318.26	499.10	517.88	550.03
TC0200004623.mm.1	Cpxm1	69.77	73.87	73.92	108.17	95.45	110.65
TC1800000488.mm.1	Lvrn	16.79	16.34	18.90	26.19	24.14	25.00
TC0700003028.mm.1	Emp3	144804.70	136931.30	126278.80	189893.60	180204.60	187031.80
TC1700001740.mm.1	AI413582	128.58	129.24	114.56	210.90	193.15	179.18
TC0600000852.mm.1	Capg	1484.10	1373.73	1305.10	2014.51	1840.12	1954.39
TC0600003370.mm.1	Slco1a5	30.93	29.82	31.05	41.03	39.58	45.45
TC0100003439.mm.1	Sft2d2	603.49	593.05	498.27	1006.41	903.01	1060.90
TC0500000541.mm.1	Rbpj	2245.76	2316.08	2179.25	3058.02	3041.15	3489.95
TC0X00002269.mm.1	Arhgef6	314.37	315.31	289.52	444.76	396.14	435.46
TC0X00000656.mm.1	Vma21	70.22	67.43	70.26	92.10	83.44	91.31
TC0200002678.mm.1	Rbm38	38.05	36.74	39.00	53.53	50.11	47.43
TC0900001979.mm.1	Aplp2	2496.94	2617.48	2436.73	3655.68	3698.08	4355.46
TC1100003326.mm.1	Evi2a; Evi2b; Gm21975	1989.73	2025.67	1878.46	3250.53	2852.40	3557.65
TC0700001391.mm.1	Arrb1	448.76	449.24	383.30	669.41	622.19	688.93
TC0100003804.mm.1	Atf3	428.75	355.14	350.59	745.49	623.88	685.81
TC0600001634.mm.1	Ptpro	41.91	37.58	37.63	50.96	50.16	51.80
TC0100000632.mm.1	Slc11a1	1659.88	1702.01	1447.30	2718.16	2567.92	3136.08
TC0700002414.mm.1	Sepw1	1901.28	1843.52	1676.54	2814.35	2508.74	2895.55
TC1800000200.mm.1	Mocos	150.88	137.24	142.90	252.49	205.77	233.28
TC0100001844.mm.1	Tmem206	369.92	377.98	386.25	514.93	471.00	538.28
TC0900003241.mm.1	Myd88	476.28	476.66	421.26	681.05	619.04	654.71
TC1400000575.mm.1	Pnp2	1585.53	1506.27	1462.46	2037.63	2211.92	2400.08
TC0200003209.mm.1	Slc2a6	191.59	175.02	149.66	382.00	304.24	358.73
TC0500001844.mm.1	Alox5ap	91156.09	76534.85	82545.45	118040.80	123959.90	126775.40
TC0100001526.mm.1	Creg1	1689.56	1701.31	1577.87	2851.67	2409.48	3010.94
TC0100003341.mm.1	Pappa2; 1600012P17Rik	58.92	60.20	54.64	86.40	77.41	89.68
TC0500002756.mm.1	Cxcl10	30.04	27.68	26.86	53.52	43.71	44.63
TC1900000504.mm.1	Ifit1	821.11	742.19	577.67	1700.25	1663.24	2166.65
TC0100002967.mm.1	Marco	31.44	32.28	33.53	42.16	48.60	48.47
TC0700000234.mm.1	Ap2s1	1440.49	1403.33	1350.39	2254.55	1966.45	2430.98
TC0800001319.mm.1	Vac14	959.70	915.68	781.91	1557.84	1450.21	1749.29
TC0X00000871.mm.1	Gm14781	7.63	6.84	7.72	10.49	9.71	10.33
TC0800000538.mm.1	Acsl1	228.87	216.11	210.64	284.94	276.36	307.06
TC0200004227.mm.1	Cat	2975.26	3003.89	2526.84	4358.34	4129.66	4334.09
TC0900000410.mm.1	Olfr913	10.74	9.69	9.78	13.58	12.70	13.14

TC0600001404.mm.1	Clec4d	2080.13	2051.52	1964.97	3440.16	2850.77	3475.23
TC1400000306.mm.1	Tkt; Mir3076	182.72	174.93	159.94	229.13	229.85	243.05
TC1500001585.mm.1	Mtss1	204.95	189.90	186.95	297.51	256.75	284.96
TC0600003428.mm.1	Bhlhe41	728.40	614.06	700.80	1143.01	1032.33	1236.12
TC1300001285.mm.1	Esm1	13.55	13.14	15.13	25.78	34.84	26.66
TC0900000184.mm.1	Icam1	211.97	197.93	184.85	283.16	266.79	298.60
TC0900002369.mm.1	Scamp5	1237.17	1080.05	1071.66	1551.45	1594.13	1554.37
TC0800000947.mm.1	Podnl1	249.63	228.97	211.62	328.28	315.14	325.74
TC1700002135.mm.1	Enpp4	48.93	46.21	45.65	64.90	59.94	69.01
TC0700002636.mm.1	Ceacam2	61.76	57.12	58.39	87.41	111.87	99.80
TC0500003271.mm.1	Scarb1	161.82	149.31	142.84	264.63	220.66	268.85
TC1600000346.mm.1	Rtp4	576.62	631.32	518.08	864.20	913.75	984.16
TC0400001736.mm.1	Pla2g2e	59.06	58.51	55.80	108.11	84.73	103.73
TC1600001019.mm.1	Slc5a3; Mrps6	123.88	131.90	124.48	180.26	164.96	193.68
TC0600002543.mm.1	Mrpl19	491.05	481.93	412.34	684.49	693.98	756.73
TC0X00002504.mm.1	Prkx	1914.51	1968.57	1710.09	2813.12	2533.28	2812.18
TC0100001550.mm.1	Aldh9a1	378.11	398.15	337.12	725.56	604.00	793.58
TC0900002156.mm.1	C2cd2l	155.02	147.13	135.73	224.52	196.68	213.34
TC0700001756.mm.1	Prkcb	218.33	223.18	199.45	348.47	312.73	389.99
TC0400003702.mm.1	Trnp1	15.35	13.52	14.62	20.24	22.22	19.64
TC1000001596.mm.1	Stat2	1097.89	1046.64	918.17	1796.43	1623.98	2042.49
TC0500003476.mm.1	Gm454; AC159257.3	166.04	174.95	187.81	593.85	364.47	484.62
TC0200000850.mm.1	Galnt13	26.74	28.18	26.55	36.54	36.90	33.09
TC0100000112.mm.1	Pi15	20.78	22.94	21.11	30.05	27.67	27.72
TC1100001047.mm.1	Cluh	333.31	287.88	285.46	475.84	424.93	454.40
TC0700004522.mm.1	Rnh1	1199.05	1061.46	1043.56	1623.68	1651.11	1904.10
TC1100003396.mm.1	Ccl5	442.29	390.45	392.75	647.56	563.69	591.33
TC1700001021.mm.1	Alkbh7	168.61	151.42	151.50	210.43	207.38	198.25
TC0600002978.mm.1	Rassf4	1029.23	1045.75	904.27	1581.45	1648.47	1981.74
TC0700000028.mm.1	Lilra5	60.35	53.65	50.05	90.93	79.47	86.31
TC0300000491.mm.1	P2ry1	13.86	18.09	14.95	27.36	26.17	26.27
TC0500002458.mm.1	Arap2	66.73	69.41	63.92	106.44	95.76	120.77
TC0100003069.mm.1	Cdk18	964.40	1002.86	859.86	1514.26	1322.17	1514.31
TC0600003375.mm.1	Ldhb	57.97	51.96	58.11	85.09	75.81	76.74
TC0700004620.mm.1	Sbsn	59.50	56.52	51.74	85.51	78.06	76.29
TC0100001382.mm.1	Rnasel	205.51	187.77	198.22	326.08	278.48	347.55
TC1700001689.mm.1	Nme4	64.72	61.35	56.97	99.43	84.84	88.73
TC0700002422.mm.1	C5ar1	447.31	382.31	398.98	552.72	561.31	564.48
TC0900000442.mm.1	Olfr974	8.99	9.48	8.95	11.51	11.74	13.00
TC1200001324.mm.1	Cdca7l	104.06	111.45	101.85	130.86	135.86	144.09
TC1100003872.mm.1	Plekhm1	1084.21	1148.90	960.70	1662.61	1499.60	1595.81
TC0600001042.mm.1	Tpra1	376.18	357.87	315.93	554.31	496.17	561.90
TC1000002493.mm.1	Gamt	27.64	25.27	27.61	46.44	38.60	39.41
TC0700000410.mm.1	Cic	451.38	442.78	427.05	662.71	563.33	610.30
TC1700000533.mm.1	Slc37a1	34.80	33.18	34.21	47.96	42.05	44.06
TC1600000368.mm.1	Trp63	12.39	13.04	13.17	15.89	17.67	18.08
TC1000003164.mm.1	Ormdl2	88.39	81.17	86.21	160.09	124.61	153.31
TC0100002838.mm.1	Dtymk	179.42	169.56	154.45	227.75	241.09	257.25
TC0200000383.mm.1	Psd4	132.18	135.26	126.84	198.56	178.05	219.01
TC1300000082.mm.1	Prl2c5	14.11	14.17	13.01	19.32	19.76	23.07
TC0900000793.mm.1	Cln6	2060.74	1921.22	1895.99	3016.22	2559.29	2797.96
TC0100001859.mm.1	Slc30a1	160.34	154.86	168.25	202.04	212.52	229.26
TC1100004102.mm.1	Wbp2	260.05	257.69	258.93	368.38	339.48	318.12
TC0300001137.mm.1	Sort1	101.35	105.28	97.11	139.01	123.97	133.17
TC0300000537.mm.1	Ptx3	17.76	21.15	23.31	70.29	47.42	79.45
TC0400004023.mm.1	Angptl7	57.38	60.32	64.55	109.33	89.56	114.52
TC0200001245.mm.1	Olfr1009	9.17	8.94	9.57	12.29	12.83	11.30
TC1200000865.mm.1	Cipc	133.17	132.27	129.45	189.52	175.19	213.65
TC0400003627.mm.1	Tinagl1	36.08	37.03	31.99	56.59	48.61	55.35
TC1700000836.mm.1	Cyp39a1	30.01	27.13	28.03	37.01	36.61	40.80
TC1700000992.mm.1	Ebi3	53.81	46.60	46.93	80.98	69.07	79.08
TC0100002033.mm.1	Mcm3	147.56	131.96	129.62	193.97	192.29	221.45
TC1300001263.mm.1	Ankrd55	19.25	18.92	20.12	27.61	24.09	25.61
TC0700001810.mm.1	Sgf29	99.56	105.65	91.96	144.41	129.75	142.49
TC0200001222.mm.1	Ube2l6	420.22	453.46	402.45	618.92	601.08	715.72
TC1300000560.mm.1	Ninj1	114.34	99.89	103.49	214.55	168.20	226.22
TC0200004896.mm.1	Trib3	18.76	18.51	19.34	22.72	25.81	24.86
TC1500000183.mm.1	Ctnnd2	33.85	36.18	36.52	50.54	44.34	47.35
TC0100003888.mm.1	Ifi204	11605.78	12656.01	11029.32	17316.00	16247.02	19126.30
TC0400004012.mm.1	Agtrap	109.60	107.86	101.98	142.26	128.46	141.40
TSUnmapped00000008.mm.1	42430	10.36	9.15	11.31	18.46	25.90	20.98
TC1000002175.mm.1	Man1a	1106.87	1167.05	965.44	1815.09	1606.33	1933.66
TC1100004051.mm.1	Cd300lb	402.27	386.73	318.39	682.47	628.14	797.79

TC0700000285.mm.1	Dmpk	67.38	66.12	63.97	97.97	83.13	90.89
TC0100003483.mm.1	Mgst3	29.87	29.76	31.86	54.76	48.94	67.56
TC1100003570.mm.1	Xylt2	466.59	475.78	414.61	701.98	617.64	719.36
TC0100003319.mm.1	Soat1	8972.23	8991.07	8277.63	12217.25	10891.08	12167.45
TC0200001985.mm.1	Ptpra	7344.21	7168.86	6890.39	9476.82	8813.59	10136.41
TC1100003156.mm.1	Gsg2	35.20	35.73	29.30	50.74	49.91	49.46
TC0400001555.mm.1	Sdc3	550.65	518.31	503.56	780.64	670.53	754.84
TC0700002788.mm.1	Fxyd1	51.39	69.14	68.75	225.07	152.91	153.21
TC0100000829.mm.1	Dgkd	343.03	332.65	306.21	448.88	414.85	464.97
TC0900002982.mm.1	Ackr4	18.74	16.39	20.39	27.40	27.46	28.58
TC0200004811.mm.1	Cd93	5674.75	5702.56	5317.18	10021.71	7938.88	10017.41
TC0800003024.mm.1	Hsdl1	222.94	195.78	180.34	339.30	295.04	318.04
TC0200005145.mm.1	Slc35c2	365.45	332.14	337.16	450.40	417.45	439.99
TC0200002668.mm.1	Cass4	26.78	31.82	27.98	39.65	41.39	39.88
TC0800002398.mm.1	Ssbp4	222.58	198.54	193.70	300.24	276.08	318.97
TC1100000951.mm.1	Med11	945.17	1001.30	952.34	1241.31	1406.36	1485.41
TC1100004283.mm.1	Syngr2	8338.08	7974.80	7116.95	11276.61	10487.31	11720.24
TC0500001257.mm.1	Triap1	101.79	98.46	89.74	149.85	128.28	144.16
TC1100004228.mm.1	Dcxr	184.07	167.03	161.42	244.85	246.53	289.18
TC0400000460.mm.1	Clta	1856.44	1650.07	1774.48	2407.79	2204.00	2285.64
TC0900003266.mm.1	Cck	27.14	29.48	25.56	37.53	37.60	42.30
TC1700000461.mm.1	Hmga1; Hmga1-rs1	761.26	800.58	710.61	1303.20	1054.83	1259.35
TC0600002109.mm.1	Hipk2	854.62	882.06	812.28	1132.96	1053.80	1205.84
TC1100000955.mm.1	Psmb6	923.49	903.13	846.16	1219.65	1147.36	1340.70
TC1500000296.mm.1	Atp6v1c1	3793.50	4316.94	3780.02	5396.90	5694.72	6228.49
TC0400001160.mm.1	Rab3b	19.69	19.13	19.80	26.73	33.07	33.47
TC0900001182.mm.1	Slc9a9	1267.94	1250.05	1116.57	1820.93	1628.50	1916.21
TC0600002194.mm.1	Rbpsuh-rs3	26038.57	26580.17	24023.75	34266.61	33135.38	37979.36
TC0200004548.mm.1	Itpripl1	248.63	263.81	229.29	364.69	331.43	388.42
TC1100001256.mm.1	Ccl4	59.48	59.46	61.95	121.83	93.59	131.49
TC1100003054.mm.1	Mpdu1	294.03	279.83	271.56	375.60	338.19	370.60
TC1000000300.mm.1	Col10a1	62.01	48.50	53.75	136.54	98.16	110.70
TC1600001500.mm.1	Nrros	183.52	172.62	156.87	251.14	225.99	244.13
TC1400001931.mm.1	Wdhd1	124.48	126.63	121.68	186.63	162.58	200.41
TC1900000500.mm.1	Ifit2	79.51	85.19	65.65	151.05	127.62	162.41
TC0700004659.mm.1	Rnf141	640.83	602.01	560.40	805.59	767.87	847.48
TC1400002632.mm.1	Kctd12; Mir5130	477.38	453.71	409.07	803.42	651.97	818.15
TC0100003664.mm.1	Psen2	72.40	64.03	71.18	111.59	93.55	109.35
TC1400002746.mm.1	Gpr183	1235.67	997.09	941.45	1860.14	1714.63	1974.80
TC1600001919.mm.1	Samsn1	59.22	55.43	52.41	73.41	68.56	72.61
TC1500000045.mm.1	Lifr	97.08	100.13	93.03	161.48	129.60	150.57
TC0X00003437.mm.1	Il2rg	564.56	602.60	533.47	718.51	753.61	803.57
TC0300002506.mm.1	Fcgr1	1444.76	1491.93	1440.42	2006.31	1811.73	2145.35
TC0600001514.mm.1	Gm5884	4183.08	4174.59	4018.84	6174.08	6592.88	8160.25
TC0100003891.mm.1	Ifi203	666.67	717.23	642.20	1319.71	992.96	1203.64
TC0200002170.mm.1	Naa20	377.31	361.38	378.94	517.24	498.27	449.76
TC1000002586.mm.1	Appl2	136.31	132.20	127.71	193.61	164.72	180.27
TC1800001437.mm.1	Adrb2	74.80	61.39	74.64	133.68	109.51	116.19
TC0800000786.mm.1	Babam1	11067.78	10860.47	9256.64	14323.94	15239.72	15320.36
TC0X00002423.mm.1	Xlr4c	34.09	34.95	35.18	45.19	43.08	50.32
TC0500002343.mm.1	Qdpr	163.46	158.79	147.17	247.28	210.83	214.56
TC0700003894.mm.1	Olfr611	8.30	9.53	9.20	12.00	11.51	12.48
TC0500002805.mm.1	Antxr2	1145.48	1126.86	971.36	1453.31	1525.74	1536.08
TC0500001560.mm.1	Rfc2	174.91	164.68	145.34	223.23	223.81	224.80
TC0800000648.mm.1	2700029M09 Rik	255.09	256.56	256.46	334.06	339.08	299.68
TC1100004053.mm.1	Cd300ld	217.44	220.61	211.16	260.58	287.57	300.78
TC1400000784.mm.1	Psme1	263.52	245.91	223.43	321.56	327.55	336.03
TC0500001649.mm.1	Lamtor4	1373.85	1296.10	1351.70	1706.97	1827.63	2032.86
TC0500003472.mm.1	Mcm7; Mir93; Mir25	250.00	237.33	211.04	311.95	320.71	337.09
TC1100004115.mm.1	Foxj1; Rnf157	56.88	56.45	56.81	78.90	69.36	81.72
TC0800000959.mm.1	Lyl1	318.23	262.96	230.08	695.19	500.60	638.09
TC0200004502.mm.1	Spg11	172.58	174.33	155.05	241.04	219.09	256.86
TC0800003224.mm.1	Zdhhc2	535.33	486.94	473.16	675.38	620.54	663.46
TC0600001193.mm.1	Itpr1	60.82	63.17	60.62	79.18	72.27	81.62
TC0900001441.mm.1	Nme6	157.49	152.19	141.96	249.65	216.61	284.47
TC0300000092.mm.1	Car13	775.16	736.91	532.14	1419.54	1377.77	1684.61
TC1700002771.mm.1	Nme3	147.80	144.00	118.89	228.95	205.33	242.84
TC0300000555.mm.1	Mfsd1	8240.51	8481.72	7340.64	11459.44	11079.55	12994.90
TC1300000597.mm.1	S1pr3	34.53	31.65	28.69	43.93	43.21	46.67
TC1100002224.mm.1	Fignl1	60.41	58.87	55.59	80.96	71.02	77.19

TC1400002142.mm.1	Cryl1	216.98	187.65	177.36	289.81	271.63	300.98
TC1700001463.mm.1	Gpr31b; Gpr31c	81.30	75.28	78.28	118.52	105.87	100.06
TC1100002103.mm.1	Tcn2	4647.37	4471.64	4180.51	6022.15	6205.76	5479.41
TC0500003175.mm.1	Oas2	1007.80	1105.22	951.60	1563.64	1437.49	1775.68
TC1000002671.mm.1	Tmpo	215.69	198.15	191.11	272.48	252.79	284.48
TC0700000647.mm.1	Pepd	91.81	91.51	81.71	136.33	115.73	126.84
TC0300003215.mm.1	Hist2h2aa1; Hist2h2aa2	1964.76	1831.43	2025.08	2730.56	2568.61	2399.57
TC1700001902.mm.1	Psmb9	55.00	53.35	53.48	69.38	69.53	80.96
TC1100003575.mm.1	Gm11546	28.88	27.29	28.11	36.99	37.58	33.23
TC0600001877.mm.1	Gpr85	35.02	40.71	38.53	56.88	50.72	59.64
TC0X00002995.mm.1	Gla	3282.71	3347.25	2835.43	4357.11	5040.75	4613.66
TC1200001552.mm.1	Rsad2	125.16	109.17	129.66	242.34	192.13	193.10
TC0100001173.mm.1	Rab7b	1219.27	1184.07	1050.07	1600.88	1537.76	1752.54
TC1700002822.mm.1	H2-T22; H2-T9	379.06	388.87	323.26	523.04	499.02	538.81
TC0100001985.mm.1	Eya1	65.84	67.56	63.90	90.55	82.59	99.04
TC0800000844.mm.1	Tom1	432.78	431.28	385.26	590.15	628.48	737.80
TC0700000896.mm.1	Nav2; Gm2788	102.58	97.08	93.79	127.85	116.68	129.19
TC1100000078.mm.1	Dbnl	163.48	150.72	149.47	195.01	188.74	209.99
TC0500003063.mm.1	Trpv4	153.17	129.34	126.82	223.59	192.73	220.73
TC0600000344.mm.1	Nup205	351.61	370.35	323.68	448.64	463.78	504.55
TC1300000919.mm.1	Glrx	947.14	959.91	728.35	1457.72	1805.16	1694.69
TC0800002992.mm.1	Maf	4893.29	4798.33	4315.94	7166.83	6147.33	7275.61
TC1_GL456210_random00000011.mm.1		2325.82	1916.61	2176.71	2949.90	3028.28	2935.83
TC1200002524.mm.1	Serpina3f	17.68	20.29	17.39	25.52	26.76	24.24
TC0900001042.mm.1	Elovl5	858.70	792.94	771.43	1079.13	993.99	1122.72
TC1600001556.mm.1	Parp14	327.22	363.57	317.30	466.18	460.98	539.09
TC0600002690.mm.1	Mcm2	222.06	208.15	188.79	311.62	305.65	377.85
TC1300001265.mm.1	Il6st	4345.99	4316.16	3981.07	6689.10	5487.50	6478.04
TC0400003379.mm.1	Atp6v0b	3978.49	3739.01	3259.84	5883.29	5232.91	6346.58
TC0200003362.mm.1	Stom	7981.12	8122.07	6858.64	10801.63	10175.61	10697.74
TC0200005114.mm.1	Sdc4	121.93	111.23	109.54	193.42	154.50	173.45
TC1200001277.mm.1	Pacs2	435.71	421.83	367.79	567.69	545.31	601.18
TC1300000262.mm.1	Hist1h1a	56.84	51.77	60.78	80.20	88.56	100.88
TC0300002695.mm.1	Cd53	19464.83	19140.70	16560.19	24856.79	26963.47	28849.07
TC0600002697.mm.1	Plxna1	4545.56	4357.42	4086.52	5845.81	5327.26	6077.78
TC1100000726.mm.1	Nlrp3	318.68	333.21	283.15	443.38	414.32	475.35
TC0800000758.mm.1	Lrrc25	104.85	89.96	101.46	134.49	135.07	153.07
TC0200004537.mm.1	Atp8b4	204.13	217.07	185.99	289.23	264.24	305.24
TC0600002114.mm.1	Parp12	3709.85	3675.00	3162.23	5380.57	4760.99	5599.76
TC0800001247.mm.1	Prmt7	86.41	77.39	76.93	102.56	99.59	108.60
TC1100003086.mm.1	Slc16a13	50.60	56.97	58.92	81.01	74.51	72.73
TC1700000036.mm.1	Zdhhc14	214.32	197.28	174.45	310.39	278.70	338.82
TC0500001610.mm.1	Ift22	538.31	469.22	516.99	635.48	703.80	692.25
TC1300000638.mm.1	Tspan17	440.23	375.52	399.37	614.35	542.35	549.05
TC1300000174.mm.1	Hist1h3h	74.87	71.88	80.10	93.99	107.93	99.60
TC0X00000677.mm.1	Xlr4b	45.97	42.50	50.01	58.79	61.79	62.18
TC0X00001634.mm.1	Phka2	278.52	289.91	259.02	345.31	360.06	394.78
TC1200002224.mm.1	Ttc7b	305.55	277.79	244.46	417.24	385.59	433.57
TC1100003115.mm.1	Scimp	219.15	184.64	204.37	348.27	318.34	438.11
TC0200001761.mm.1	Gchfr	26.03	25.15	28.93	40.30	34.77	41.03
TC0300002492.mm.1	Plekho1	1118.32	1021.39	968.22	1426.51	1300.95	1353.92
TC0200005402.mm.1	Lama5	68.02	61.83	62.79	87.01	79.14	92.09
TC0X00001665.mm.1	Pir	58.25	54.14	53.72	88.38	71.81	78.35
TC0800003171.mm.1	Agt	27.32	23.26	24.89	41.95	35.07	35.85
TC0500003507.mm.1	Snx8	264.26	263.01	243.52	451.49	351.63	453.71
TC1100002069.mm.1	Metrnl	246.10	237.16	206.10	363.63	312.60	370.99
TC0500003742.mm.1	Tmem184a	12.22	12.95	12.71	16.18	19.59	16.97
TC0300001161.mm.1	Vav3	162.28	159.06	160.94	230.07	206.58	259.78
TC1900000127.mm.1	Ppp1r14b	731.40	682.65	703.71	936.76	846.56	984.72
TC1500001528.mm.1	Tnfrsf11b	665.69	588.89	555.82	1415.89	972.96	1383.16
TC0800002842.mm.1	Psmb10	203.17	176.55	165.07	294.47	252.18	294.20
TC1900000022.mm.1	Acy3	36.42	32.46	31.45	43.15	42.08	42.76
TC0Y00000223.mm.1	Erdr1	1979.51	1629.86	1657.47	3287.32	2568.68	3182.45
TC1700001209.mm.1	Galm	115.17	105.02	107.46	185.07	164.80	232.16
TC1900000693.mm.1	Tmem180	59.28	57.62	49.58	82.99	73.71	81.47
TC1500001838.mm.1	Rac2	8075.59	6973.53	5679.82	12604.75	11430.98	12950.32
TC1400000574.mm.1	Pnp	1673.03	1595.91	1576.12	2560.08	2233.97	2996.42
TC0600001474.mm.1	D6Wsu163e	500.34	475.14	418.28	635.51	625.25	698.86

TC1500000591.mm.1	Mroh1; Mir6954	220.17	212.93	185.15	326.14	283.38	350.48
TC1400002454.mm.1	Lacc1	287.39	294.85	261.38	374.96	357.05	414.46
TC0100003058.mm.1	Srgap2	551.65	536.42	491.99	770.43	709.81	892.53
TC1600000033.mm.1	Hmox2	723.58	687.64	687.15	941.19	859.14	1032.90
TC0100002420.mm.1	Gm11578	7.71	8.52	8.40	14.23	12.55	11.03
TC1100003836.mm.1	Slc25a39	373.96	339.17	336.40	447.40	415.33	441.29
TC1800000606.mm.1	Gm4951	237.37	288.27	222.91	429.09	402.30	526.01
TC1100000333.mm.1	Snrnp25	227.06	227.06	249.62	312.69	334.37	288.08
TC1300001449.mm.1	Edaradd	37.74	31.87	34.58	55.32	46.08	53.95
TC0400002097.mm.1	Cpsf3l	312.81	299.63	280.47	412.32	361.60	409.19
TC0500001239.mm.1	Oasl2	938.69	994.34	829.11	1376.66	1224.35	1438.95
TC1500001658.mm.1	St3gal1	478.05	466.66	425.54	628.90	588.25	705.64
TC0300002501.mm.1	Hist2h2aa1; Hist2h2aa2	1773.62	1771.02	1885.85	2299.41	2555.86	2198.05
TC0100000386.mm.1	Ormdl1	35.26	38.01	33.02	51.01	45.11	53.31
TC1100001999.mm.1	Gaa	1917.90	1828.39	1609.46	2710.32	2359.83	2765.54
TC0100003401.mm.1	Fmo1	14.22	14.46	15.00	17.54	18.99	20.76
TC0400004062.mm.1	Car6	31.60	27.90	34.49	61.77	46.83	51.85
TC0600001398.mm.1	Clec4a3	765.23	692.80	657.20	1052.10	925.59	1128.68
TC0400003708.mm.1	Zdhhc18	689.31	698.86	612.35	917.34	826.06	918.49
TC0200001894.mm.1	Fgf7	214.92	212.18	196.95	375.25	308.79	450.46
TC0800003076.mm.1	Aprt	442.34	396.17	354.41	567.02	542.06	561.65
TC1300001383.mm.1	Akr1e1	195.83	179.62	179.36	246.71	220.67	250.41
TC1000002727.mm.1	Mrpl42	22897.96	23067.70	25370.03	33610.64	29037.26	32588.96
TC1600000529.mm.1	Hcls1	3477.21	3201.65	3208.73	5636.28	4345.46	5253.34
TC0600003048.mm.1	Bid	90.81	84.35	82.26	112.86	115.95	136.92
TC1300001139.mm.1	Taf9; Ak6	564.51	495.80	510.37	687.98	633.07	678.53
TC1700002534.mm.1	Dpy30	39.17	36.56	37.53	57.55	47.02	56.57
TC1200000279.mm.1	Nampt	3730.35	3601.68	3243.61	5418.20	4853.63	6298.50
TC0100003124.mm.1	Rnpep	653.11	636.85	616.88	1062.73	826.30	924.85
TC0500000057.mm.1	Tmem243	50.22	48.91	53.59	76.90	64.39	79.60
TC0100000476.mm.1	Cd28	26.35	24.55	23.28	35.18	30.60	35.69
TC1300000191.mm.1	Hist1h2bk	144.12	119.95	145.03	194.22	203.40	183.66
TC0100003595.mm.1	Gm16340	1347.73	1408.78	1168.12	2211.43	1786.69	2074.60
TC1000000892.mm.1	Pip5k1c	178.00	171.30	164.13	234.20	204.16	236.39
TC0900003318.mm.1	Ppp2r3d; Ppp2r3a	61.02	57.41	56.30	78.90	68.80	77.25
TC0900000816.mm.1	Snapc5	286.18	268.54	315.05	362.40	379.87	394.37
TC1700002571.mm.1	Cyp1b1	277.43	271.51	224.14	484.69	414.18	602.53
TC1100000582.mm.1	Cdkn2aipnl	3423.22	3173.29	2583.41	4947.38	4517.99	4971.18
TC0100003051.mm.1	Mapkapk2	6271.30	6686.47	5836.20	8901.12	8177.16	10038.85
TC0100001170.mm.1	Fam72a	31.87	28.10	32.33	56.41	49.04	42.73
TC0100003736.mm.1	Hlx	103.30	110.80	98.00	135.78	154.91	171.34
TC0600000705.mm.1	Vmn1r28	20.23	18.93	19.53	25.46	26.28	22.84
TC0300000697.mm.1	Etv3	86.40	91.50	77.66	111.71	106.44	111.73
TC0400004034.mm.1	Pgd	743.85	692.30	617.55	937.63	897.43	1013.57
TC1500000389.mm.1	Nov	18.70	18.77	21.32	40.72	29.79	31.14
TC1400001559.mm.1	Comtd1	43.03	44.66	42.61	60.60	51.48	60.04
TC0700002750.mm.1	Sdhaf1	59.64	60.60	58.86	79.29	70.88	85.03
TC0X00001054.mm.1	Pbdc1	94.82	82.09	83.44	107.80	111.43	109.08
TC1500000423.mm.1	Ndufb9	6853.57	5759.46	5882.02	8181.32	8323.08	9202.05
TC0400003847.mm.1	Pqlc2	143.85	142.95	125.76	187.75	173.32	201.62
TC1100002045.mm.1	Rac3	125.25	125.07	112.67	223.49	166.38	219.72
TC0200004953.mm.1	Ahcy; Gm4737	365.49	336.63	297.66	488.53	440.42	503.42
TC0100000375.mm.1	Stat1	2520.11	2278.41	2176.81	3189.57	2860.68	3166.24
TC0200005496.mm.1	Fam78a	38.53	36.06	33.86	50.07	43.98	47.40
TC1800000890.mm.1	Cyb5a	904.05	842.94	857.45	1457.07	1132.69	1246.82
TC1900000211.mm.1	Slc15a3	1238.68	1132.45	1051.09	1779.97	1477.30	1736.75
TC0100001711.mm.1	Parp1; Mir6904	245.68	219.57	208.43	317.71	282.41	311.58
TC1500001754.mm.1	Tsta3	346.34	306.34	283.71	466.32	411.98	480.42
TC0100000103.mm.1	Ly96	68908.45	77919.91	68650.48	96735.52	89159.77	102805.00
TC1100001565.mm.1	Stard3	350.50	340.67	308.11	500.43	417.92	473.39
TC0800000756.mm.1	Isyna1	106.35	103.45	83.93	169.09	146.45	193.69
TC0X00002417.mm.1	Xlr4a	33.62	37.39	35.11	50.45	47.23	60.23
TC1100004264.mm.1	Mgat4b; Mir6919	102.83	87.98	82.46	144.99	125.84	153.99
TC1300002247.mm.1	Zfp459	45.81	43.05	44.98	59.53	51.75	58.20
TC0100000222.mm.1	Imp4	874.83	770.45	745.86	1055.50	995.54	1086.52
TC0300000722.mm.1	Glmp	1495.88	1446.83	1312.25	1966.16	1727.90	1899.17
TC1400001573.mm.1	Dlg5	103.04	101.52	94.11	132.71	117.61	134.20
TC0200004942.mm.1	E2f1	169.45	179.16	155.74	224.35	206.90	237.17

TC1100004301.mm.1	Trim47	82.10	69.99	71.80	111.40	95.43	104.93
TC1400000176.mm.1	Plau	7376.70	7515.71	6361.65	9630.87	9220.04	10528.22
TC0100003890.mm.1	Mndal	311.88	322.48	316.54	506.24	399.73	439.92
TC0200001368.mm.1	Spi1	4106.56	4166.80	3594.97	5534.50	5081.33	6041.86
TC0600001521.mm.1	Clec2i	20.47	20.79	23.59	26.89	28.07	26.82
TC0X00000724.mm.1	Xlr4d-ps	74.03	69.69	87.49	120.24	134.80	170.06
TC0700002718.mm.1	Kcnk6	430.89	457.60	330.26	646.00	679.25	661.07
TC1700000318.mm.1	Pkmyt1	37.76	35.56	33.33	50.09	43.48	46.02
TC1100000362.mm.1	Lcp2	5250.68	5241.19	4859.99	7683.45	6286.94	7052.68
TC0100001016.mm.1	Zcchc2	231.83	237.12	225.24	316.81	271.17	315.24
TC0800001407.mm.1	Plcg2	432.85	434.63	395.43	615.30	518.10	621.20
TC0500001389.mm.1	Vps29	501.81	487.72	474.40	640.55	564.10	650.97
TC0900001423.mm.1	Nckipsd	131.93	119.78	123.47	188.23	153.96	177.68
TC0300002297.mm.1	Apoa1bp	8042.57	8722.59	7408.63	10684.42	9940.83	10867.66
TC0600001243.mm.1	Mtmr14	266.60	267.18	225.99	372.00	327.78	380.33
TC1500000775.mm.1	Bik	45.00	48.43	42.74	59.48	55.92	65.32
TC1000000744.mm.1	Lss	143.74	134.31	121.93	187.88	166.23	181.08
TC0700002693.mm.1	Timm50	78.15	82.97	82.48	113.01	96.89	115.93
TC1200001768.mm.1	Nfkbia	369.09	314.30	312.87	423.35	437.85	446.79
TC0700003693.mm.1	Olfr305	13.99	13.91	16.46	19.16	19.70	19.19
TC0400003680.mm.1	Smpdl3b	260.34	230.08	227.52	300.60	290.58	310.69
TC1800000484.mm.1	Ap3s1	11143.38	10840.23	11077.16	17741.12	14098.73	18548.11
TC1400000501.mm.1	Cdkn3	1069.22	1160.38	1252.77	1815.59	1487.77	1755.70
TC0400001790.mm.1	Hspb7	44.58	35.99	40.58	154.61	77.88	109.83
TC0800000017.mm.1	Stxbp2	1120.49	1074.12	1068.96	1513.78	1285.81	1524.43
TC0900001348.mm.1	Abhd14b	42.56	38.75	38.87	57.73	52.43	67.53
TC0800001493.mm.1	Cdt1	68.05	58.86	65.59	103.86	83.29	92.83
TC1300001634.mm.1	Hist1h2ae	67.45	64.32	67.10	90.65	77.77	91.69
TC0300000897.mm.1	Cers2	498.84	445.53	437.87	598.99	556.36	622.23
TC0800003031.mm.1	Cotl1	2283.62	1821.23	1764.11	3343.90	2793.74	3260.06
TC1200001237.mm.1	Tnfaip2	388.70	402.25	356.74	508.53	462.08	537.51
TC0600003123.mm.1	Gpr162	28.77	24.80	23.99	45.88	35.76	47.48
TC1100003379.mm.1	Slfn9	297.10	294.43	265.35	414.05	351.71	419.54
TC0900000031.mm.1	Casp1	472.02	422.24	420.91	786.35	625.62	916.19
TC0100002151.mm.1	Ccdc115	78.40	92.90	78.07	137.24	113.21	147.03
TC1000000783.mm.1	Gm10146	1795.46	1463.08	1538.01	2235.89	2113.36	2408.21
TC0X00003276.mm.1	Ppef1	13.22	13.50	14.85	16.93	19.59	17.76
TC0400000436.mm.1	Tesk1	97.67	102.79	92.30	130.39	119.22	143.10
TC0200003165.mm.1	Fcna	54.46	53.57	53.89	65.42	69.45	79.59
TC0300002348.mm.1	Adam15	979.16	939.63	859.69	1322.15	1144.12	1372.89
TC1400001524.mm.1	Gng2	1013.23	871.44	828.67	1275.56	1188.31	1369.53
TC0300002728.mm.1	Gstm4	759.08	700.61	606.00	980.59	918.00	953.57
TC1_GL456212_random00000008.mm.1		225.78	223.27	212.65	306.45	293.53	258.44
TC1900000993.mm.1	Pola2	249.73	248.30	212.14	333.11	301.55	349.97
TC1600002080.mm.1	Tmem50b	4236.64	4514.65	3707.25	5533.34	5796.71	6528.47
TC1800000534.mm.1	Snx2	14502.67	14094.15	13740.41	18433.34	17065.83	20791.92
TC1100002176.mm.1	Myo1g	286.41	271.77	253.39	403.58	340.01	425.94
TC1700002288.mm.1	Mrps36-ps1	1952.25	1519.20	1725.56	2835.32	2400.99	2975.28
TC0200004970.mm.1	Edem2	296.03	266.37	254.34	367.77	338.39	395.44
TC1000001203.mm.1	Poc1b	263.31	233.40	221.48	354.03	304.68	362.74
TC1700001130.mm.1	Myom1	26.95	29.01	27.76	32.72	37.23	38.71
TC1800001048.mm.1	B4galt6	4479.57	4517.37	3938.86	6104.86	5557.55	6897.20
TC1800000063.mm.1	Colec12	3140.64	3073.89	2520.22	4613.26	4176.39	5433.52
TC1900001137.mm.1	Ms4a6d	27641.62	26494.06	24861.79	38526.88	31943.33	37684.59
TC1100001265.mm.1	Wfdc18	25.34	27.78	27.33	35.28	44.79	36.73
TC0400002275.mm.1	Atp6v0d2	258.73	227.84	223.16	385.24	326.18	451.58
TC0300001447.mm.1	Gbp2	243.10	202.11	180.88	390.28	308.40	409.37
TC0900001603.mm.1	Eif1b	1417.62	1314.27	1198.28	1883.34	1736.94	2191.89
TC1300001461.mm.1	Gpr137b	5464.41	5507.77	5155.34	7366.90	6586.79	8245.21
TC1900000889.mm.1	Ndufs8	18588.81	16483.55	14117.32	23037.71	25087.95	27145.96
TC0600003342.mm.1	Eps8	59.59	54.67	55.45	83.13	68.78	84.12
TC0800000737.mm.1	Nr2c2ap	203.72	181.80	171.88	241.24	234.20	263.63
TC1000002093.mm.1	Aim1	42.54	38.91	38.50	55.44	47.40	52.44
TC1800000682.mm.1	Sec11c	340.75	365.48	353.42	427.02	453.74	515.88
TC1500002250.mm.1	Galnt6	85.69	90.38	78.35	119.28	104.25	124.42
TC1900000888.mm.1	Tcirg1	3076.82	3077.79	2651.47	4146.95	3774.60	4618.26
TC1_GL456211_random00000030.mm.1		734.23	760.02	578.20	1296.13	1167.34	1008.98
TC0100003550.mm.1	Slamf7	161.26	190.78	156.42	252.42	224.11	273.43
TC1600001343.mm.1	Abcc5	120.01	118.58	110.67	158.76	139.10	168.78
TC1100003506.mm.1	Scpep1	4195.04	4079.84	3660.21	5614.48	4933.40	5994.35
TC1300001016.mm.1	Gm21726	16.15	14.12	13.84	20.25	18.34	21.59
TC0400003323.mm.1	Tex38	14.47	15.73	14.75	20.79	20.20	17.59

TC1700001938.mm.1	D17H6S56E-5	1107.13	1112.32	941.28	1518.33	1359.72	1650.38
TC1000001286.mm.1	Osbpl8	2844.59	3217.73	2721.15	4719.78	3943.13	5325.95
TC0700004309.mm.1	Maz	232.91	212.42	184.73	321.01	277.09	314.47
TC1700000362.mm.1	Clcn7	469.90	427.16	388.73	646.20	559.77	703.13
TC1500001657.mm.1	Ndrg1	111.39	97.70	98.19	157.35	127.96	148.66
TC0X00002556.mm.1	Gk	143.34	156.56	153.38	217.88	188.37	242.15
TC0800002599.mm.1	Asna1	5114.81	4565.05	3714.54	7462.91	6515.80	7773.71
TC0500002755.mm.1	Cxcl9	163.07	162.05	168.54	400.11	250.98	420.15
TC0100000711.mm.1	Agfg1	7653.82	7987.24	7081.02	9381.60	9891.44	11254.73
TC1700001322.mm.1	Ppp1r21	246.86	259.70	214.25	330.78	302.65	329.04
TC0800000759.mm.1	Lsm4	2839.42	2778.65	2184.81	3902.26	4378.46	3737.12
TC0500002234.mm.1	Gm5553	397.50	359.08	381.26	730.87	531.93	806.24
TC1400002464.mm.1	Dnajc15	1707.59	1595.38	1920.79	2651.17	2780.96	3772.19
TC0800001259.mm.1	Sntb2	372.33	372.79	359.43	502.68	440.10	549.62
TC0X00001795.mm.1	Was	418.58	405.14	396.01	586.42	479.70	544.95
TC0700000794.mm.1	Klk1b4	16.98	17.67	15.47	26.42	22.74	21.30
TC0100002349.mm.1	Pgap1	272.17	279.89	248.01	400.50	339.57	443.43
TC0600001205.mm.1	Arl8b	9328.85	8483.97	8255.49	13194.18	10802.21	13540.94
TC1300001640.mm.1	Hist1h4c	241.67	203.24	199.61	328.42	278.59	303.10
TC0700004320.mm.1	Dctpp1	1162.74	936.45	1085.51	1625.08	1492.56	1971.01
TC1300001069.mm.1	Wdr41	603.73	552.71	529.34	773.71	692.20	850.46
TC0100003670.mm.1	Mixl1	13.97	16.03	12.28	22.58	28.18	20.89
TC0100001244.mm.1	Ptpn7	256.92	257.48	221.73	417.41	363.09	547.06
TC0500000992.mm.1	Cds1	41.92	37.31	42.62	54.39	48.55	55.74
TC0500001401.mm.1	P2rx4	4099.81	4151.42	3576.01	5781.46	4908.12	5965.65
TC0800002925.mm.1	Il34	17.62	17.36	18.62	23.72	20.48	23.61
TC1800000610.mm.1	Iigp1	34.19	34.87	34.92	58.14	47.54	71.30
TC1700002224.mm.1	A530064D06Rik	61.65	52.82	44.13	125.69	82.80	109.76
TC0800001320.mm.1	Mtss1l	92.40	92.36	80.86	155.30	115.58	148.39
TC0700001178.mm.1	Tm6sf1	927.53	914.63	877.07	1177.74	1032.31	1207.77
TC0500000848.mm.1	Cxcl1	32.14	24.68	29.02	40.64	39.66	39.59
TC0900001377.mm.1	Ifrd2	144.30	131.06	128.44	171.48	155.90	172.48
TC0600001637.mm.1	Dera	144.26	126.91	125.30	169.16	157.26	168.55
TC1600001785.mm.1	Olfr181	11.24	11.18	13.47	18.19	16.04	15.77
TC0600002480.mm.1	Atoh8	32.60	34.36	38.71	45.92	43.75	49.90
TC0700002927.mm.1	Gm21028	20.66	18.72	23.44	34.89	27.69	32.40
TC1000002517.mm.1	Lsm7	335.64	302.62	300.02	384.89	420.71	474.39
TC1800000728.mm.1	Mc5r	11.24	11.65	14.11	16.33	19.79	18.95
TC0600000470.mm.1	Olfr449	15.66	16.21	20.43	24.55	27.09	24.97
TC0100003593.mm.1	AI607873	3302.91	3533.91	3246.60	5131.80	4070.88	4986.09
TC1200001245.mm.1	Klc1	425.59	454.50	418.26	549.96	492.93	565.53
TC1100004265.mm.1	Igtp	665.55	547.72	547.44	873.34	806.60	1042.02
TC0300003246.mm.1	Gstm3	67.65	65.14	56.38	97.49	109.59	83.05
TC1700001618.mm.1	Amdhd2	116.34	117.14	112.66	165.04	139.54	180.66
TC1000001190.mm.1	Lum	132.14	130.73	125.30	191.97	173.01	153.78
TC0200005013.mm.1	Samhd1	1419.91	1318.54	1353.36	1950.72	1628.60	2042.66
TC0600001810.mm.1	Pdk4	2212.99	2240.13	2072.75	2908.19	2649.38	3329.90
TC0200002308.mm.1	Hck	399.16	364.79	309.94	576.11	477.34	575.16
TC0700004002.mm.1	Gvin1; Gm4070	158.54	147.93	120.27	257.04	228.72	353.49
TC0300002920.mm.1	5730508B09Rik	497.10	529.45	447.30	819.60	631.86	725.55
TC0500002623.mm.1	Kdr	62.35	61.79	61.07	82.89	74.77	95.73
TC0200002698.mm.1	Npepl1	238.37	206.12	187.01	288.37	280.32	318.33
TC0700000522.mm.1	Rasgrp4	33.79	32.75	30.46	45.17	37.86	43.58
TC1300000385.mm.1	Bphl	37.32	35.31	34.57	48.87	41.68	50.62
TC1700001395.mm.1	Tagap1	818.60	814.02	772.18	960.90	1228.44	1172.10
TC0900000854.mm.1	2810417H13Rik	496.64	494.73	505.87	725.40	665.63	582.68
TC1700000706.mm.1	Nrm	149.59	148.28	132.64	218.90	175.04	202.45
TC1100001849.mm.1	Kcnj2	30.63	26.51	25.94	47.65	35.89	46.11
TC0700002049.mm.1	Taldo1	293.76	272.20	267.80	391.46	326.73	391.55
TC0400000869.mm.1	Dennd4c	763.78	839.54	717.17	1116.28	946.38	1154.08
TC0200001256.mm.1	Olfr1026	9.81	10.09	10.18	11.73	14.53	13.14
TC0400002796.mm.1	Ambp	78.15	68.49	63.17	88.81	91.85	92.07
TC1400001086.mm.1	Epsti1	167.71	162.30	153.05	218.46	192.71	241.53
TC1100003159.mm.1	Ctns	515.14	500.32	445.72	705.02	587.92	684.42
TC1900000184.mm.1	Rab3il1	379.40	357.84	340.96	478.26	430.20	530.39
TC1800000358.mm.1	Pcdhb4	9.89	10.38	10.84	14.84	15.86	12.46
TC1400001676.mm.1	Sh3bp5	59.71	64.75	62.60	108.05	78.77	93.43
TC1000000158.mm.1	Mtfr2	113.68	135.87	107.83	163.31	158.29	183.26
TC1100002504.mm.1	Spdl1	63.96	61.95	70.14	87.60	77.42	92.70

TC1400002186.mm.1	Ebpl	70.04	73.31	79.71	86.28	97.41	96.73
TC0700003593.mm.1	Fes	426.53	385.84	343.58	510.11	487.43	509.78
TC1900001026.mm.1	Fkbp2	806.63	661.50	659.87	1332.95	967.54	1141.88
TC0800001179.mm.1	Cklf; A730028G07Rik	272.98	290.54	249.81	397.01	328.03	389.70
TC1400000912.mm.1	Sox7	16.08	14.26	16.28	20.25	21.64	26.41
TC0800000015.mm.1	Pet100	114.88	114.98	134.56	154.98	200.32	198.64
TC0600002418.mm.1	Vmn1r38	13.60	12.10	11.91	14.84	16.83	15.69
TC0600002853.mm.1	Rybp	953.71	941.28	896.20	1434.61	1201.54	1714.56
TC0300002504.mm.1	Hist2h4	1598.45	1530.82	1506.43	1984.13	1804.49	2211.18
TC0700002096.mm.1	Slc22a18	37.13	37.10	43.28	51.33	54.05	47.47
TC0400003681.mm.1	Themis2	2285.84	2097.28	2053.14	3439.53	2668.22	3550.35
TC0900000333.mm.1	Rpusd4	39.06	38.34	37.96	54.44	47.16	63.08
TC1500001702.mm.1	Gm3244	6023.64	5658.60	6380.96	8190.81	6992.31	7894.98
TC0900001376.mm.1	Hyal1; Nat6; Hyal3	106.95	102.96	95.72	134.15	116.77	131.09
TSUnmapped00000050.mm.1	F630003A18Rik	227.12	214.90	182.10	430.07	288.79	385.19
TC0100003026.mm.1	Mcm6	406.38	376.02	346.23	586.65	463.56	553.25
TC1100001241.mm.1	Slfn4	96.68	81.90	80.43	129.03	115.78	156.11
TC0600001088.mm.1	Lsm3	70.65	56.17	64.94	98.73	82.04	91.39
TC0800002553.mm.1	Tecr	42813.82	42525.89	38094.54	57333.84	48271.14	55794.92
TC1000002036.mm.1	Slc16a10	486.64	547.66	411.75	739.15	657.78	829.78
TC1100003314.mm.1	Lgals9	27744.36	30558.02	24477.75	35255.05	35712.43	36435.64
TC1100002755.mm.1	Pdlim4	188.34	176.71	184.37	257.72	211.50	255.44
TC0100001957.mm.1	Cpa6	28.52	28.51	24.94	33.23	37.35	41.04
TC1500002031.mm.1	Alg12	94.20	83.36	82.68	109.26	100.50	108.45
TC0400001129.mm.1	Ndc1	366.48	349.14	339.89	490.91	404.98	481.33
TC1100000712.mm.1	Hist3h2ba	108.55	92.59	102.41	147.24	123.57	131.16
TC1100002030.mm.1	Slc25a10	134.82	130.88	117.95	170.12	157.85	197.38
TC1000000482.mm.1	Gm5423	1583.97	1258.96	1110.21	2791.36	1937.33	2607.78
TC1100002106.mm.1	Mtfp1	16.90	16.13	19.49	21.34	23.26	22.78
TC0700000561.mm.1	Tyrobp	241652.10	207825.40	217424.60	284574.00	259598.40	285502.20
TC0700004607.mm.1	Fadd	48.29	47.27	53.07	73.17	69.62	58.83
TC1300002751.mm.1	Tcstv1	11.92	11.31	14.23	16.57	16.67	16.20
TC1200000819.mm.1	Isca2	402.46	364.16	396.24	557.82	455.11	551.77
TC0200003865.mm.1	Calcrl	145.23	137.86	115.27	183.78	174.57	210.07
TC0700003946.mm.1	Trim30d	176.91	164.58	151.96	233.89	208.09	275.31
TC0600002555.mm.1	Htra2	607.59	597.86	509.09	810.51	713.35	888.57
TC1300000019.mm.1	Akr1c13	42.72	38.55	38.90	57.51	47.07	57.17
TC0600000772.mm.1	Mad2l1	456.38	414.43	369.73	605.94	520.80	654.54
TC1100004122.mm.1	Rhbdf2	241.77	211.78	218.39	303.10	261.35	306.14
TC0300001076.mm.1	Slc16a1	158.34	138.93	146.14	216.72	181.51	240.53
TC1100000049.mm.1	Zmat5	441.96	419.72	343.86	554.83	524.65	567.23
TC0600003532.mm.1	Arpc4	197.69	168.70	168.59	226.73	217.12	246.81
TC1000002392.mm.1	Mmp11	62.12	48.61	48.82	81.16	69.57	82.13
TC1700002368.mm.1	Efna5	73.32	63.43	66.03	89.76	79.14	92.62
TC1100000892.mm.1	Aurkb	203.52	189.46	167.33	269.97	227.62	260.71
TC0200001874.mm.1	Slc24a5	97.73	90.59	92.92	138.29	110.55	142.25
TC1300002541.mm.1	Mrps36	206.82	192.39	193.31	291.53	233.42	253.64
TC1700000879.mm.1	Mrps18a	119.76	117.07	113.25	184.53	140.48	157.28
TC0400002660.mm.1	Vma21-ps	386.35	331.77	329.37	468.93	516.53	659.37
TC0700000787.mm.1	Egfbp2; Klk1b26	14.72	13.76	15.66	18.84	21.25	17.49
TC1100001386.mm.1	Trim25	679.13	687.22	734.01	887.26	828.60	1027.14
TC0900001345.mm.1	Dusp7	1501.64	1497.55	1262.75	2125.86	1763.98	2256.00
TC0900000141.mm.1	Olfr832	9.10	7.85	7.78	10.22	9.87	10.91
TC1900000996.mm.1	Gm19505	81.89	72.36	71.18	95.06	114.95	94.82
TC0200000593.mm.1	Ptrh1	96.28	96.76	81.24	132.03	111.82	137.44
TC1300001902.mm.1	Dtnbp1	456.81	417.19	388.02	569.42	514.58	648.37
TC1700001027.mm.1	Tnfsf9	116.79	106.60	84.96	212.04	158.79	281.86
TC0400001543.mm.1	Fabp3	235.33	216.43	203.44	286.28	269.09	338.40
TC0400002576.mm.1	Exosc3	166.59	186.42	159.95	231.41	201.68	242.42
TC1800000708.mm.1	Psmg2	2192.53	2359.76	1991.34	2955.10	2563.00	2892.37
TC0500001932.mm.1	Rundc3b	15.25	15.73	16.32	21.00	20.90	17.67
TC1300002304.mm.1	Nkd2	161.52	155.24	138.48	224.66	187.30	252.30
TC1900001342.mm.1	Plgrkt	161.27	151.04	148.97	195.32	170.45	194.66
TC0300000159.mm.1	Nceh1	411.52	449.66	394.61	580.38	496.75	633.10
TC1000002497.mm.1	Adamtsl5	64.18	60.03	54.72	79.01	69.24	78.45
TC1900001340.mm.1	Insl6	46.92	43.22	43.24	72.12	55.38	81.01
TC1400001174.mm.1	Uchl3	1822.67	1582.20	1412.16	2553.17	2044.10	2365.03
TC1700001213.mm.1	Morn2	170.50	139.58	146.40	277.40	200.95	222.23
TC0200004019.mm.1	Olfr1180	11.29	13.08	13.65	17.16	19.63	15.73

TC0400001773.mm.1	Atp13a2	400.60	374.28	336.18	504.43	441.64	537.61
TC0500002434.mm.1	Gm8069	868.10	698.55	733.46	1286.08	988.31	1402.99
TC0700002167.mm.1	Hspbp1	270.69	242.90	203.81	331.72	318.89	321.02
TC0200001002.mm.1	Nostrin	97.03	92.31	88.09	132.87	106.38	130.51
TC0X00000131.mm.1	Mid1ip1	133.84	123.36	107.17	197.72	151.66	194.72
TC1600001277.mm.1	2610318N02Rik	133.82	123.75	84.03	197.00	179.70	220.82
TC0700000424.mm.1	Tgfb1	14988.84	14031.58	13180.17	20888.38	16588.60	21284.95
TC0X00003361.mm.1	Prps2	403.25	392.23	354.70	548.44	446.19	545.10
TC1000000097.mm.1	Adat2	542.95	487.67	429.36	651.09	607.79	628.17
TC0200005271.mm.1	Aurka	285.40	245.16	251.19	331.19	311.00	372.12
TC0100003210.mm.1	Rgs18	503.24	612.67	518.00	704.16	713.34	866.11
TC0200004737.mm.1	Kif16b	136.31	131.25	129.48	186.37	151.10	192.41
TC0400003894.mm.1	Efhd2	2059.23	2181.59	1763.33	2476.94	2782.09	3088.65
TC0500003729.mm.1	Gbp8	31.85	26.23	26.06	35.09	36.06	35.62
TC1300001378.mm.1	Akr1c12	16.89	17.06	15.26	22.63	19.50	25.56
TC0900001038.mm.1	Gclc	921.46	866.96	783.08	1174.55	1019.77	1285.22
TC1500001568.mm.1	Atad2	315.01	340.89	307.58	438.28	363.46	415.13
TC1800001118.mm.1	Sft2d3	68.68	70.74	66.56	77.88	98.02	95.54
TC0800002658.mm.1	Snx20	48.98	36.82	39.62	69.03	53.99	66.14
TC1900000090.mm.1	Slc25a45	112.44	117.96	98.25	148.01	132.31	164.75
TC0600000854.mm.1	Retsat	158.57	137.55	119.93	198.14	181.78	231.00
TC0500001152.mm.1	Chek2	134.33	110.68	116.77	170.25	144.29	159.47
TC1300001564.mm.1	Hist1h4n; Hist1h4m	113.37	102.08	115.28	173.86	131.77	151.57
TC0700000228.mm.1	Tmem160	7195.62	5874.12	6365.32	7849.92	8850.17	9705.73
TC1300000205.mm.1	Vmn1r200	12.75	16.03	13.96	17.42	18.67	19.15
TC0300000910.mm.1	Mcl1	1469.27	1642.05	1426.75	1949.23	1720.81	1908.55
TC0900001828.mm.1	Olfr867	11.56	9.05	10.40	12.73	14.10	14.28
TC1300000181.mm.1	Hist1h2ao; Hist1h2ap; Hist1h2ai; Hist1h2ah	984.34	766.35	813.59	1384.68	1111.40	1174.60
TC0300002352.mm.1	Cks1b	1272.39	1254.67	1230.59	1655.85	1379.04	1629.81
TC0300000787.mm.1	Dennd4b	203.03	182.74	164.80	259.81	221.42	274.64
TC1300000969.mm.1	Cetn3	254.72	199.57	218.67	299.78	279.37	324.36
TC0800002338.mm.1	Gm9755	411.73	395.41	379.97	510.41	468.34	606.23
TC0600000340.mm.1	Tmem140	101.24	112.85	93.69	141.51	123.64	157.30
TC1400000440.mm.1	Gm8126	10.76	10.47	11.81	14.21	16.40	13.00
TC1500000742.mm.1	Srebf2	2164.41	1997.14	1855.09	2767.36	2323.28	2830.12
TC0900002593.mm.1	Ccnb2	782.05	704.91	653.95	955.18	830.67	932.85
TC1900001004.mm.1	Arl2	1246.43	1391.81	1090.76	1734.71	1525.58	1679.64
TC0200001843.mm.1	Sord	159.73	130.44	116.45	299.66	186.23	287.16
TC0500000816.mm.1	Dck	193.81	200.30	180.46	294.59	222.81	275.45
TC0200003132.mm.1	Tor4a	209.13	206.86	177.56	298.13	234.93	292.41
TC0600001443.mm.1	Lpar5	183.12	163.57	210.73	303.04	332.11	236.09
TC1200002269.mm.1	Ifi27l2a	388.77	370.69	355.03	494.39	465.27	637.89
TC1700002490.mm.1	Emilin2	279.76	294.30	282.87	364.22	329.57	423.09
TC1800000444.mm.1	Gm3650	252.72	218.85	227.91	366.28	294.48	446.85
TC0400001890.mm.1	Gm13248	466.19	473.89	417.59	635.01	569.20	804.01
TC0700001589.mm.1	Olfr477	8.10	8.99	9.37	10.74	15.47	14.15
TC1000001936.mm.1	Rspo3	67.97	59.95	56.17	83.81	73.02	90.96
TC0X00000521.mm.1	Gm10477	9.35	9.61	9.72	12.23	17.10	12.70
TC0900002064.mm.1	Olfr919	17.36	16.38	15.76	22.44	18.44	21.71
TC0500000171.mm.1	Fgl2	19.73	23.51	20.94	28.52	29.08	39.43
TC1200000647.mm.1	Prkch	270.40	259.84	244.84	346.04	294.49	375.77
TC0700004630.mm.1	Hsd3b7	57.43	54.59	46.57	70.17	62.91	70.54
TC1600001722.mm.1	Alcam	1408.42	1453.03	1291.09	1862.77	1587.59	2018.48
TC1300001453.mm.1	Prl2c2	18.09	15.43	17.30	36.87	22.22	38.22
TC1000001270.mm.1	Pawr	175.08	161.16	169.89	278.55	198.18	259.74
TC1400001182.mm.1	Irg1	158.65	143.27	133.92	204.42	174.09	232.81
TC1700002095.mm.1	Gm21903	13.12	16.61	15.00	19.93	20.45	26.94
TC1100001236.mm.1	Slfn5	177.81	170.36	152.10	254.74	200.02	279.64
TC0700002648.mm.1	LOC101055953	901.12	894.48	888.30	1379.11	1029.82	1196.89
TC0200004649.mm.1	1700037H04Rik	309.99	325.43	278.84	397.32	358.92	452.63
TC1000003018.mm.1	Helb	99.06	96.74	90.92	124.10	105.42	125.76
TC1900000008.mm.1	Cpt1a	1114.17	1066.51	965.69	1368.21	1189.73	1422.20
TC0800001244.mm.1	Pla2g15	2024.92	1752.74	1708.73	2256.48	2125.61	2373.97
TC0500003243.mm.1	Hcar2	76.92	68.49	71.55	119.38	97.01	87.51
TC1600001419.mm.1	Cldn1	10.95	12.01	13.29	14.44	15.85	17.92
TC0X00000181.mm.1	Maoa	101.60	100.66	87.36	139.27	113.79	151.14
TC1900001118.mm.1	Ccdc86	191.46	170.94	172.66	236.10	199.31	235.36

TC0X00001799.mm.1	Ebp	110.93	95.61	96.22	133.56	115.05	132.81
TC1_GL456221_random00000002.mm.1		1047.81	836.54	1139.58	1323.07	1540.81	1380.22
TC1000002943.mm.1	Slc35e3	472.56	549.89	433.36	733.26	585.32	717.19
TC0100003599.mm.1	Ifi202b; Ifi205	463.00	473.66	407.52	792.65	552.14	850.60
TC0400002694.mm.1	Klf4	93.08	78.10	70.75	104.69	102.61	114.07
TC0200004902.mm.1	Defb26	13.02	11.08	11.33	14.95	13.58	15.84
TC1100001629.mm.1	Nkiras2; Gm19456	107.38	99.17	87.37	143.59	115.52	137.76
TC0400004127.mm.1	Fam213b	79.51	74.53	81.97	105.50	89.95	117.93
TC1900000401.mm.1	Gm3873	83242.34	75992.21	87514.50	114944.60	93337.55	108834.70
TC1400000255.mm.1	Gm2274	1880.75	1759.09	1511.12	2109.60	2229.21	2604.59
TC0600002405.mm.1	Gm7144	9.27	7.81	6.58	11.67	10.27	11.43
TC0400001107.mm.1	Dhcr24	2730.73	2553.54	2164.37	3294.85	3106.98	3935.81
TC0300002114.mm.1	Rarres1	63.40	54.05	51.70	69.06	75.77	89.62
TC0500003240.mm.1	Zcchc8	1069.23	1146.48	1076.79	1562.14	1232.57	1590.14
TC1400000524.mm.1	Peli2	100.53	88.50	86.40	150.75	108.41	138.72
TC0400000743.mm.1	Tlr4	406.52	386.22	348.22	542.80	435.44	544.50
TC0500001288.mm.1	Taok3	170.57	176.03	158.43	229.84	187.13	225.24
TC0200001261.mm.1	Olfr1030	12.12	12.40	14.31	19.73	15.09	18.98
TC0500002480.mm.1	Tlr1	221.39	227.79	187.97	349.57	270.88	436.58
TC1000003204.mm.1	Olfr825	9.60	10.92	12.31	16.26	15.10	13.20
TC1300000209.mm.1	Vmn1r201	18.55	16.17	18.42	21.55	26.85	32.24
TC1300000709.mm.1	Idnk	1922.70	1678.32	1571.27	2003.90	2238.60	2214.47
TC0200002588.mm.1	Rnf114	474.68	439.46	430.98	574.15	492.51	591.29
TC1400000252.mm.1	Gm2260	1687.72	1571.44	1353.55	1882.01	2003.26	2367.66
TC1000001886.mm.1	Taar9	10.17	8.74	8.86	11.60	11.09	13.97
TC1900001196.mm.1	Tle4	377.58	396.35	348.26	519.43	423.82	549.14
TSUnmapped00000030.mm.1	H60a	114.09	134.70	109.74	142.41	149.30	147.07
TC0500002708.mm.1	Gm11116	11.69	19.24	15.74	27.69	23.85	39.63
TC0600002369.mm.1	Vmn1r27	8.98	9.37	10.48	12.83	12.89	10.82
TSUnmapped00000055.mm.1	Mrgpra7	15.62	19.80	16.27	20.56	23.98	24.15
TC0600002362.mm.1	Vmn1r25	19.49	20.88	19.69	30.01	35.41	23.62
TC0100001167.mm.1	Il10	17.52	16.48	17.64	19.12	24.64	24.19
TC1100001892.mm.1	Ict1	180.16	162.26	155.26	217.34	185.75	222.33
TC0200001412.mm.1	1700029I15Rik	24.16	22.93	23.27	34.18	26.14	34.22
TC0900002060.mm.1	Olfr915	9.26	8.62	11.71	13.76	18.99	13.74
TC1300000330.mm.1	Mboat1	106.77	89.42	84.89	148.77	111.90	149.57
TC0700001594.mm.1	Olfr494	18.09	16.90	15.05	19.17	24.19	24.27
TC0400002271.mm.1	Wwp1	4217.89	4752.31	3921.08	5596.52	5046.55	6377.63
TC0X00000587.mm.1	Ctag2	9.13	9.90	11.29	13.24	11.73	13.04
TC0200003960.mm.1	Olfr1097	6.60	7.50	8.03	16.00	10.42	10.31
TC0100001317.mm.1	Uchl5	507.59	490.35	420.87	987.86	598.73	1089.81
TC0900001337.mm.1	Twf2	305.83	249.61	228.52	336.75	333.63	354.97
TC1200000675.mm.1	Mthfd1	224.24	218.26	207.82	293.79	238.15	302.11
TC1300001561.mm.1	Hist1h3i	35.93	29.76	26.49	43.04	41.13	38.59
TC1300002462.mm.1	F2r	243.81	297.69	208.63	332.94	346.57	343.97
TC0600003271.mm.1	Tas2r130	9.99	9.09	11.39	14.77	11.84	14.43
TC0600002348.mm.1	Vmn1r18	15.12	11.05	15.04	23.36	17.45	24.78
TC0500001459.mm.1	Gm19631	159.39	146.79	162.02	223.96	173.40	219.12
TC1000003117.mm.1	Shmt2	512.49	461.70	378.65	630.83	554.67	645.64
TC1200001988.mm.1	Zfp36l1	1979.16	1927.30	1699.12	3094.16	2219.95	2533.14
TC0200004039.mm.1	Olfr1219	11.34	11.45	13.07	19.31	22.38	14.22
TC1500001733.mm.1	Gm10238	59.99	53.90	49.65	76.92	77.99	61.96
TC1300001557.mm.1	Hist1h4k	405.47	396.20	462.38	598.55	473.35	585.34
TC0900003176.mm.1	Cmtm7	962.69	811.99	791.67	1159.50	1000.36	1312.41
TC0X00001715.mm.1	Gm21887	420.50	382.91	332.30	542.80	455.62	643.19
TC1400000623.mm.1	Trav6d-3	16.21	15.27	17.73	18.10	21.25	21.28
TC1800000794.mm.1	Acaa2	447.46	371.77	350.02	474.32	499.95	582.11
TC1300001669.mm.1	Acot13	2896.96	2463.82	3208.67	4280.79	3400.54	3930.90
TC1500002316.mm.1	Gpr84	702.55	543.06	489.68	999.23	736.56	878.70
TC0300002802.mm.1	Vcam1	213.03	231.35	195.83	249.59	276.20	332.83
TC1700000203.mm.1	Lix1	21.62	23.66	18.85	27.92	27.51	38.06
TC1900000266.mm.1	Olfr1461	11.88	12.97	12.32	14.42	21.92	17.98
TC0700000365.mm.1	Vmn1r183	15.76	15.19	17.30	22.24	26.47	18.57
TC0500001038.mm.1	Lrrc8b	436.25	341.27	341.31	572.99	444.83	604.42
TC0800000941.mm.1	Samd1	60.13	64.26	60.05	76.88	69.37	91.65
TC0100001031.mm.1	Serpinb8	93.98	81.47	76.14	110.81	98.66	129.69
TC1700000772.mm.1	Olfr107	11.27	9.08	9.84	14.03	15.13	11.59
TC0700000791.mm.1	Klk1b16	13.85	11.71	15.17	18.42	15.94	18.76
TC1300000612.mm.1	Syk	167.92	171.97	160.41	223.90	180.60	230.70
TC0700003907.mm.1	Olfr67	12.55	11.02	13.53	14.20	18.01	19.24
TC1100000168.mm.1	Cnrip1	429.28	355.16	312.67	585.28	461.75	734.56

TC1900000908.mm.1	Rad9a	216.96	188.17	172.43	286.84	223.81	259.09
TC0100001370.mm.1	Ncf2	511.02	491.13	454.72	660.43	534.35	695.24
TC0200002496.mm.1	Wisp2	3404.56	3078.43	2717.92	4242.63	3524.94	4516.52
TC1200001971.mm.1	Atp6v1d	3542.39	3192.59	3128.69	4343.54	3613.25	4570.69
TC0900000152.mm.1	Olfr855	15.72	15.75	13.68	21.78	21.03	16.77
TC0200000676.mm.1	Olfr357	8.08	7.88	9.62	13.16	10.44	10.49
TC0500003487.mm.1	Adap1	65.41	62.18	61.53	82.23	68.22	88.15
TC1600001641.mm.1	Atp6v1a	1495.68	1445.88	1333.01	1835.49	1544.52	1903.15
TC0500001764.mm.1	Arpc1b	3582.83	3176.47	3112.20	4179.80	3693.20	4766.83
TC0600002337.mm.1	Lsm5	24.46	20.42	20.99	25.41	35.45	30.00
TC0300002425.mm.1	Tdpoz1	8.48	9.90	11.44	13.13	13.21	12.03
TC0700003996.mm.1	Gvin1; Gm4070; Gm17757; Gm18853	85.43	80.67	63.50	107.28	107.34	169.01
TC1400000415.mm.1	Gm2832	10.63	10.08	11.62	11.79	15.01	14.17
TC0600003379.mm.1	Abcc9	14.69	14.51	15.34	14.39	16.08	14.14
TC0100001560.mm.1	Rgs5	20.93	22.13	20.52	22.20	18.66	19.50
