# Supplementary material for: MicroRNA-26b-5p Inhibits Mouse Liver Fibrogenesis and Angiogenesis by Targeting PDGF Receptor-Beta
Source: Mol Ther Nucleic Acids. 2019 Feb 26;16:206–17. doi: 10.1016/j.omtn.2019.02.014 (PMC6426711; doi:10.1016/j.omtn.2019.02.014)
Supplement: Table S3. KEGG and Reactome Pathways in TGF-b1-Treated BMSCs with or without miR-26b-5p Mimics [file mmc3.pdf]

MicroRNA-26b-5p Inhibits Mouse Liver Fibrogenesis and Angiogenesis by Targeting PDGF Receptor-beta

Le Yang#, Chengbin Dong#, Jingjing Yang, Lin Yang, Na Chang, Changbo Qi, Liying Li*

Supplementary table 3. KEGG and Reactome pathways in TGF-b1-treated BMSCs with or without miR-26b-5p mimics

<table>
<tr><th>Term</th><th>Database</th><th>ID</th><th>P-Value</th><th>Corrected P-Value</th><th>input_gene</th></tr>
<tr><td>Cytokine-cytokine receptor interaction</td><td>KEGG PATHWAY</td><td>mmu04060</td><td>8.663E-17</td><td>6.073E-14</td><td>Ppbp,57349:down|Ccl2,20296:up|Ccl17,20295:down|Ccl12,20293:up|Figf,14205:up|Cxcl12,20315:up|Tnf,21926:up|Cxcr6,80901:down|Kitl,17311:up|Pdgfc,54635:down|Tnfrsf11b,18383:up|Inhba,16323:down|Tnfsf9,21950:up|Cxcl14,57266:down|Vegfb,22340:up|Egfr,13649:down|Ccl9,20308:up|Ccl6,20305:up|Ccl7,20306:up|Ccl8,20307:up|Ccl3,20302:up|Ccl4,20303:up|Tnfrsf12a,27279:down|Cxcl9,17329:up|Bmpr1a,12166:down|Vegfa,22339:down|Tgfb2,21808:down|Tgfb3,21809:down|Cx3cr1,13051:down</td></tr>
<tr><td>Extracellular matrix organization</td><td>Reactome</td><td>R-MMU-1474244</td><td>1.622E-14</td><td>5.684E-12</td><td>Ibsp,15891:up|P4ha1,18451:down|Col5a2,12832:down|Col5a1,12831:down|Col12a1,12816:down|Efemp1,216616:up|Col10a1,12813:up|Col3a1,12825:down|Plod2,26432:down|Mmp13,17386:up|Itga11,319480:down|Serpinh1,12406:down|Tll1,21892:down|Bmp4,12159:up|Vcan,13003:down|Col1a2,12843:down|Lama2,16773:down|Klk1b11,16613:up|Mmp2,17390:down|Ceacam1,26365:up|Col11a1,12814:down|Col8a1,12837:down|Tnc,21923:down|Adamts4,240913:down|Tgfb2,21808:down|Tgfb3,21809:down</td></tr>
<tr><td>Collagen biosynthesis and modifying enzymes</td><td>Reactome</td><td>R-MMU-1650814</td><td>2.884E-10</td><td>6.740E-08</td><td>Col11a1,12814:down|P4ha1,18451:down|Serpinh1,12406:down|Tll1,21892:down|Col5a2,12832:down|Col5a1,12831:down|Col12a1,12816:down|Col8a1,12837:down|Col10a1,12813:up|Col1a2,12843:down|Col3a1,12825:down|Plod2,26432:down</td></tr>
<tr><td>Collagen formation</td><td>Reactome</td><td>R-MMU-1474290</td><td>1.119E-09</td><td>1.961E-07</td><td>Col11a1,12814:down|P4ha1,18451:down|Serpinh1,12406:down|Tll1,21892:down|Col5a2,12832:down|Col5a1,12831:down|Col12a1,12816:down|Col8a1,12837:down|Col10a1,12813:up|Col1a2,12843:down|Col3a1,12825:down|Plod2,26432:down|Mmp13,17386:up</td></tr>
<tr><td>Platelet degranulation</td><td>Reactome</td><td>R-MMU-114608</td><td>1.746E-09</td><td>2.246E-07</td><td>Igf2,16002:up|Ppbp,57349:down|Islr,26968:up|Kng1,16644:up|Kng2,385643:up|Vegfb,22340:up|Serping1,12258:up|Cd109,235505:down|Gas6,14456:up|Igf1,16000:up|Trf,22041:up|Vegfa,22339:down|Tgfb2,21808:down|Tgfb3,21809:down|Figf,14205:up</td></tr>
<tr><td>Osteoclast differentiation</td><td>KEGG PATHWAY</td><td>mmu04380</td><td>1.922E-09</td><td>2.246E-07</td><td>Gm15448,100041146:up|Pira11,18724:up|Sirpb1a,320832:down|Lilra6,18726:up|Pira7,18730:up|Pira1,18722:up|Pira4,18727:up|Pira6,18729:up|Pparg,19016:up|Pira2,18725:up|Tnf,21926:up|Sirpb1b,668101:down|Tgfb2,21808:down|Gm14548,100038909:up|Tnfrsf11b,18383:up</td></tr>
<tr><td>Response to elevated platelet cytosolic Ca2+</td><td>Reactome</td><td>R-MMU-76005</td><td>2.800E-09</td><td>2.804E-07</td><td>Igf2,16002:up|Ppbp,57349:down|Islr,26968:up|Kng1,16644:up|Kng2,385643:up|Vegfb,22340:up|Serping1,12258:up|Cd109,235505:down|Gas6,14456:up|Igf1,16000:up|Trf,22041:up|Vegfa,22339:down|Tgfb2,21808:down|Tgfb3,21809:down|Figf,14205:up</td></tr>
<tr><td>Peptide ligand-binding receptors</td><td>Reactome</td><td>R-MMU-375276</td><td>2.241E-08</td><td>1.821E-06</td><td>Ccl17,20295:down|Ccl9,20308:up|Cxcl12,20315:up|Ccl6,20305:up|Ccl7,20306:up|Kng1,16644:up|Kng2,385643:up|Ccl3,20302:up|Ccl4,20303:up|Cxcl9,17329:up|Ppbp,57349:down|Ccl2,20296:up|Ednrb,13618:up|Cx3cr1,13051:down|C3,12266:up|Cxcr6,80901:down</td></tr>
<tr><td>Chemokine receptors bind chemokines</td><td>Reactome</td><td>R-MMU-380108</td><td>2.338E-08</td><td>1.821E-06</td><td>Ccl17,20295:down|Cxcl12,20315:up|Ccl7,20306:up|Ccl3,20302:up|Ccl4,20303:up|Cxcl9,17329:up|Ppbp,57349:down|Ccl2,20296:up|Cx3cr1,13051:down|Cxcr6,80901:down</td></tr>
<tr><td>Degradation of the extracellular matrix</td><td>Reactome</td><td>R-MMU-1474228</td><td>4.638E-08</td><td>3.251E-06</td><td>Col11a1,12814:down|Tll1,21892:down|Col5a2,12832:down|Col5a1,12831:down|Col12a1,12816:down|Col8a1,12837:down|Adamts4,240913:down|Col10a1,12813:up|Mmp2,17390:down|Klk1b11,16613:up|Col3a1,12825:down|Col1a2,12843:down|Mmp13,17386:up</td></tr>
<tr><td>AGE-RAGE signaling pathway in diabetic complications</td><td>KEGG PATHWAY</td><td>mmu04933</td><td>6.176E-08</td><td>3.936E-06</td><td>Mmp2,17390:down|F3,14066:up|Ccl2,20296:up|Vegfb,22340:up|Tnf,21926:up|Vegfa,22339:down|Col3a1,12825:down|Tgfb2,21808:down|Tgfb3,21809:down|Col1a2,12843:down|Ccl12,20293:up|Figf,14205:up</td></tr>
</table>

Class A/1 (Rhodopsin-like receptors)	Reactome	R-MMU-373076	7.826E-08	4.572E-06	Ednrb,13618:up|Ccl17,20295:down|Ccl9,20308:up|Cxcl12,20315:up|Ccl4,20303:up|Ccl6,20305:up|Ccl7,20306:up|Kng1,16644:up|Kng2,385643:up|Ccl3,20302:up|Cxcr6,80901:down|Cxcl9,17329:up|Ppbp,57349:down|Ptgir,19222:up|Ccl2,20296:up|C3,12266:up|Cx3cr1,13051:down|Htr2b,15559:down|Htr2a,15558:down
Collagen degradation	Reactome	R-MMU-1442490	9.361E-08	5.048E-06	Col11a1,12814:down|Col5a2,12832:down|Col5a1,12831:down|Col12a1,12816:down|Col8a1,12837:down|Col10a1,12813:up|Mmp2,17390:down|Col3a1,12825:down|Col1a2,12843:down|Mmp13,17386:up
Hemostasis	Reactome	R-MMU-109582	1.984E-07	9.387E-06	Ppbp,57349:down|Islr,26968:up|Prkar2b,19088:up|Kng1,16644:up|Gas6,14456:up|Figf,14205:up|Sirpb1a,320832:down|Dok2,13449:up|Plat,18791:down|Serping1,12258:up|Igf2,16002:up|Igf1,16000:up|Serpine2,20720:up|Kng2,385643:up|Vegfb,22340:up|Sirpb1b,668101:down|Trf,22041:up|Col1a2,12843:down|Ceacam1,26365:up|Cd109,235505:down|Ptgir,19222:up|Vegfa,22339:down|Tgfb2,21808:down|Tgfb3,21809:down
Assembly of collagen fibrils and other multimeric structures	Reactome	R-MMU-2022090	2.009E-07	9.387E-06	Col11a1,12814:down|Tll1,21892:down|Col5a2,12832:down|Col5a1,12831:down|Col8a1,12837:down|Col10a1,12813:up|Col1a2,12843:down|Col3a1,12825:down|Mmp13,17386:up
Chemokine signaling pathway	KEGG PATHWAY	mmu04062	2.257E-07	9.887E-06	Ccl8,20307:up|Ccl17,20295:down|Ccl9,20308:up|Cxcl12,20315:up|Ccl4,20303:up|Ccl6,20305:up|Ccl7,20306:up|Cxcl14,57266:down|Ccl3,20302:up|Cxcr6,80901:down|Cxcl9,17329:up|Ppbp,57349:down|Ccl2,20296:up|Cx3cr1,13051:down|Ccl12,20293:up
GPCR ligand binding	Reactome	R-MMU-500792	2.907E-07	1.199E-05	Ednrb,13618:up|Ccl3,20302:up|Glp1r,14652:down|Ccl9,20308:up|Cxcl12,20315:up|Ccl4,20303:up|Ccl6,20305:up|Ccl7,20306:up|Kng1,16644:up|Kng2,385643:up|Adm,11535:up|Cxcr6,80901:down|Cxcl9,17329:up|Ppbp,57349:down|Ptgir,19222:up|Ccl17,20295:down|Ccl2,20296:up|C3,12266:up|Cx3cr1,13051:down|Htr2b,15559:down|Htr2a,15558:down
Regulation of Insulin-like Growth Factor (IGF) transport and uptake by Insulin-like Growth Factor Binding Proteins (IGFBPs)	Reactome	R-MMU-381426	5.303E-07	2.065E-05	Pappa,18491:down|Igf2,16002:up|Igfbp4,16010:up|Igf1,16000:up|Klk1b11,16613:up|Mmp2,17390:down|Igfbp3,16009:up
Mineral absorption	KEGG PATHWAY	mmu04978	6.255E-07	2.308E-05	Hmox1,15368:up|Fxyd2,11936:up|Steap1,70358:down|Mt1,17748:up|Trf,22041:up|Slc40a1,53945:up|Vdr,22337:down|Mt2,17750:up
Platelet activation, signaling and aggregation	Reactome	R-MMU-76002	1.193E-06	4.182E-05	Igf2,16002:up|Ppbp,57349:down|Islr,26968:up|Kng1,16644:up|Kng2,385643:up|Vegfb,22340:up|Serping1,12258:up|Cd109,235505:down|Gas6,14456:up|Igf1,16000:up|Trf,22041:up|Vegfa,22339:down|Col1a2,12843:down|Tgfb2,21808:down|Tgfb3,21809:down|Figf,14205:up
Pathways in cancer	KEGG PATHWAY	mmu05200	1.549E-06	5.169E-05	Pparg,19016:up|Lama2,16773:down|Ednrb,13618:up|Vegfa,22339:down|Cxcl12,20315:up|Ret,19713:up|Fgfr2,14183:up|Fgfr1,14182:down|Igf1,16000:up|Vegfb,22340:up|Kitl,17311:up|Mmp2,17390:down|Ptgs2,19225:down|Bmp4,12159:up|Wnt5a,22418:down|Epas1,13819:up|Tgfb2,21808:down|Tgfb3,21809:down|Egfr,13649:down|Figf,14205:up
Non-integrin membrane-ECM interactions	Reactome	R-MMU-3000171	4.678E-06	1.491E-04	Lama2,16773:down|Col5a2,12832:down|Col5a1,12831:down|Tnc,21923:down|Col10a1,12813:up|Col1a2,12843:down|Col3a1,12825:down
Complement and coagulation cascades	KEGG PATHWAY	mmu04610	7.382E-06	2.250E-04	C1s2,317677:up|Plat,18791:down|Kng1,16644:up|Kng2,385643:up|Serping1,12258:up|F3,14066:up|C1s1,50908:up|C3,12266:up|Vsig4,278180:down
Protein digestion and absorption	KEGG PATHWAY	mmu04974	9.506E-06	2.776E-04	Col11a1,12814:down|Fxyd2,11936:up|Col5a2,12832:down|Col5a1,12831:down|Col12a1,12816:down|Col10a1,12813:up|Col1a2,12843:down|Col3a1,12825:down|Mme,17380:up

<table>
<tr><td>Focal adhesion</td><td>KEGG PATHWAY</td><td>mmu04510</td><td>1.055E-05</td><td>2.959E-04</td><td>Ibsp,15891:up|Itga11,319480:down|Lama2,16773:down|Thbs2,21826:down|Igf1,16000:up|Vegfb,22340:up|Pdgfc,54635:down|Itga5,16402:down|Vegfa,22339:down|Col1a2,12843:down|Tnc,21923:down|Egfr,13649:down|Figf,14205:up</td></tr>
<tr><td>PI3K-Akt signaling pathway</td><td>KEGG PATHWAY</td><td>mmu04151</td><td>1.230E-05</td><td>3.317E-04</td><td>Ibsp,15891:up|Lama2,16773:down|Itga11,319480:down|Fgfr2,14183:up|Fgfr1,14182:down|Thbs2,21826:down|Igf1,16000:up|Vegfb,22340:up|Kitl,17311:up|Pdgfc,54635:down|Itga5,16402:down|Vegfa,22339:down|Col1a2,12843:down|Creb3l1,26427:down|Tnc,21923:down|Egfr,13649:down|Figf,14205:up</td></tr>
<tr><td>Integrin cell surface interactions</td><td>Reactome</td><td>R-MMU-216083</td><td>1.481E-05</td><td>3.844E-04</td><td>Ibsp,15891:up|Itga11,319480:down|Col5a2,12832:down|Col5a1,12831:down|Tnc,21923:down|Col3a1,12825:down|Col8a1,12837:down|Col1a2,12843:down</td></tr>
<tr><td>ECM proteoglycans</td><td>Reactome</td><td>R-MMU-3000178</td><td>2.618E-05</td><td>6.555E-04</td><td>Col5a2,12832:down|Col5a1,12831:down|Tnc,21923:down|Vcan,13003:down|Col1a2,12843:down|Col3a1,12825:down</td></tr>
<tr><td>Rheumatoid arthritis</td><td>KEGG PATHWAY</td><td>mmu05323</td><td>3.514E-05</td><td>8.493E-04</td><td>Cxcl12,20315:up|Ccl2,20296:up|Ccl3,20302:up|Tnf,21926:up|Vegfa,22339:down|Tgfb2,21808:down|Tgfb3,21809:down|Ccl12,20293:up</td></tr>
<tr><td>ECM-receptor interaction</td><td>KEGG PATHWAY</td><td>mmu04512</td><td>3.807E-05</td><td>8.895E-04</td><td>Ibsp,15891:up|Tnc,21923:down|Itga11,319480:down|Lama2,16773:down|Thbs2,21826:down|Itga5,16402:down|Npnt,114249:up|Col1a2,12843:down</td></tr>
<tr><td>Hematopoietic cell lineage</td><td>KEGG PATHWAY</td><td>mmu04640</td><td>4.120E-05</td><td>9.316E-04</td><td>Cd24a,12484:down|Anpep,16790:up|Cd4,12504:up|Kitl,17311:up|Tnf,21926:up|Itga5,16402:down|Tfrc,22042:up|Mme,17380:up</td></tr>
<tr><td>Proteoglycans in cancer</td><td>KEGG PATHWAY</td><td>mmu05205</td><td>5.752E-05</td><td>1.260E-03</td><td>Hpse,15442:up|Fgfr1,14182:down|Igf2,16002:up|Tnf,21926:up|Igf1,16000:up|Itga5,16402:down|Vegfa,22339:down|Wnt5a,22418:down|Col1a2,12843:down|Tgfb2,21808:down|Mmp2,17390:down|Egfr,13649:down</td></tr>
<tr><td>Signal Transduction</td><td>Reactome</td><td>R-MMU-162582</td><td>1.031E-04</td><td>2.190E-03</td><td>Glp1r,14652:down|Wnt5a,22418:down|Ret,19713:up|Prkar2b,19088:up|Kng1,16644:up|Col5a2,12832:down|Col5a1,12831:down|Ppbp,57349:down|Ccl3,20302:up|Adam12,11489:down|Nrg1,211323:down|Inhba,16323:down|Ccl17,20295:down|Htr2b,15559:down|Cxcr6,80901:down|Aldh1a3,56847:down|Kitl,17311:up|Ednrb,13618:up|Plat,18791:down|Cxcl12,20315:up|Cxcl9,17329:up|Ccl2,20296:up|Pdgfc,54635:down|Gpc4,14735:down|Igf2,16002:up|Igf1,16000:up|Prl2c2,18811:up|Figf,14205:up|Crabp2,12904:down|Htr2a,15558:down|Rspo2,239405:up|Thbs2,21826:down|Aldh1a2,19378:up|Vegfb,22340:up|Kng2,385643:up|Fabp5,16592:up|Flrt2,399558:down|Lpl,16956:up|Col3a1,12825:down|C3,12266:up|Egfr,13649:down|Ccl9,20308:up|Ccl6,20305:up|Ccl7,20306:up|Fgfr1,14182:down|Adm,11535:up|Ccl4,20303:up|Pmepa1,65112:down|Tnf,21926:up|Bmpr1a,12166:down|Ptgir,19222:up|Vegfa,22339:down|Fstl3,83554:down|Cx3cr1,13051:down|Akr1c18,105349:up|Fgfr2,14183:up</td></tr>
<tr><td>Malaria</td><td>KEGG PATHWAY</td><td>mmu05144</td><td>1.084E-04</td><td>2.234E-03</td><td>Ccl2,20296:up|Thbs2,21826:down|Tnf,21926:up|Tgfb2,21808:down|Tgfb3,21809:down|Ccl12,20293:up</td></tr>
<tr><td>Initial triggering of complement</td><td>Reactome</td><td>R-MMU-166663</td><td>1.172E-04</td><td>2.346E-03</td><td>C1s2,317677:up|C1s1,50908:up|C1rb,667277:up|C3,12266:up</td></tr>
<tr><td>Axon guidance</td><td>Reactome</td><td>R-MMU-422475</td><td>1.366E-04</td><td>2.660E-03</td><td>Sema7a,20361:down|Dok2,13449:up|Cd24a,12484:down|Col5a2,12832:down|Ret,19713:up|Ncam1,17967:down|Fgfr2,14183:up|Fgfr1,14182:down|Kitl,17311:up|Plxnd1,67784:up|Col5a1,12831:down|Vegfa,22339:down|Mmp2,17390:down|Nrg1,211323:down|Col3a1,12825:down|Egfr,13649:down|Dpysl3,22240:down</td></tr>
<tr><td>Developmental Biology</td><td>Reactome</td><td>R-MMU-1266738</td><td>2.162E-04</td><td>4.028E-03</td><td>Fgfr1,14182:down|Cd24a,12484:down|Ret,19713:up|Cdo1,12583:up|Plxnd1,67784:up|Col5a1,12831:down|Col3a1,12825:down|Nrg1,211323:down|Pparg,19016:up|Dok2,13449:up|Ncam1,17967:down|Kitl,17311:up|Egfr,13649:down|Dpysl3,22240:down|Mmp2,17390:down|Lgi2,246316:up|Col5a2,12832:down|Fgfr2,14183:up|Sema7a,20361:down|Tnf,21926:up|Vegfa,22339:down|Fabp4,11770:up</td></tr>
<tr><td>EGFR tyrosine kinase inhibitor resistance</td><td>KEGG PATHWAY</td><td>mmu01521</td><td>2.184E-04</td><td>4.028E-03</td><td>Fgfr2,14183:up|Igf1,16000:up|Gas6,14456:up|Pdgfc,54635:down|Vegfa,22339:down|Nrg1,211323:down|Egfr,13649:down</td></tr>
<tr><td>Hypertrophic cardiomyopathy (HCM)</td><td>KEGG PATHWAY</td><td>mmu05410</td><td>2.688E-04</td><td>4.831E-03</td><td>Itga11,319480:down|Lama2,16773:down|Tnf,21926:up|Igf1,16000:up|Itga5,16402:down|Tgfb2,21808:down|Tgfb3,21809:down</td></tr>
</table>

VEGF binds to VEGFR leading to receptor dimerization	Reactome	R-MMU-195399	2.907E-04	4.970E-03	Vegfb,22340:up|Vegfa,22339:down|Figf,14205:up
VEGF ligand-receptor interactions	Reactome	R-MMU-194313	2.907E-04	4.970E-03	Vegfb,22340:up|Vegfa,22339:down|Figf,14205:up
Dilated cardiomyopathy	KEGG PATHWAY	mmu05414	3.501E-04	5.708E-03	Itga11,319480:down|Lama2,16773:down|Tnf,21926:up|Igf1,16000:up|Itga5,16402:down|Tgfb2,21808:down|Tgfb3,21809:down
Creation of C4 and C2 activators	Reactome	R-MMU-166786	3.956E-04	6.163E-03	C1s2,317677:up|C1s1,50908:up|C1rb,667277:up
Classical antibody-mediated complement activation	Reactome	R-MMU-173623	3.956E-04	6.163E-03	C1s2,317677:up|C1s1,50908:up|C1rb,667277:up
Intrinsic Pathway of Fibrin Clot Formation	Reactome	R-MMU-140837	4.862E-04	7.410E-03	Serpine2,20720:up|Kng1,16644:up|Kng2,385643:up|Serping1,12258:up
Signaling by PDGF	Reactome	R-MMU-186797	5.827E-04	8.510E-03	Fgfr1,14182:down|Plat,18791:down|Ret,19713:up|Fgfr2,14183:up|Prkar2b,19088:up|Thbs2,21826:down|Col5a1,12831:down|Kitl,17311:up|Col5a2,12832:down|Pdgfc,54635:down|Nrg1,211323:down|Col3a1,12825:down|Egfr,13649:down
NCAM1 interactions	Reactome	R-MMU-419037	6.439E-04	9.212E-03	Col3a1,12825:down|Col5a1,12831:down|Col5a2,12832:down|Ncam1,17967:down
Pertussis	KEGG PATHWAY	mmu05133	8.419E-04	1.163E-02	C1s2,317677:up|Serping1,12258:up|Tnf,21926:up|Itga5,16402:down|C1s1,50908:up|C3,12266:up
Chagas disease (American trypanosomiasis)	KEGG PATHWAY	mmu05142	8.460E-04	1.163E-02	Ccl2,20296:up|Ccl3,20302:up|Tnf,21926:up|Tgfb2,21808:down|Tgfb3,21809:down|C3,12266:up|Ccl12,20293:up
Signaling by Retinoic Acid	Reactome	R-MMU-5362517	1.321E-03	1.715E-02	Fabp5,16592:up|Crabp2,12904:down|Akr1c18,105349:up|Aldh1a2,19378:up|Aldh1a3,56847:down
Molecules associated with elastic fibres	Reactome	R-MMU-2129379	1.480E-03	1.887E-02	Tgfb2,21808:down|Tgfb3,21809:down|Efemp1,216616:up|Bmp4,12159:up
TGF-beta signaling pathway	KEGG PATHWAY	mmu04350	1.646E-03	2.030E-02	Inhba,16323:down|Tnf,21926:up|Bmpr1a,12166:down|Bmp4,12159:up|Tgfb2,21808:down|Tgfb3,21809:down
Glutathione metabolism	KEGG PATHWAY	mmu00480	1.651E-03	2.030E-02	Gstm1,14862:up|Gstt1,14871:up|Gsta4,14860:up|Anpep,16790:up|Ggt5,23887:up
NCAM signaling for neurite out-growth	Reactome	R-MMU-375165	1.719E-03	2.068E-02	Ret,19713:up|Ncam1,17967:down|Fgfr2,14183:up|Fgfr1,14182:down|Kitl,17311:up|Col5a2,12832:down|Col5a1,12831:down|Nrg1,211323:down|Col3a1,12825:down|Egfr,13649:down
Cell surface interactions at the vascular wall	Reactome	R-MMU-202733	1.741E-03	2.068E-02	Sirpb1a,320832:down|Dok2,13449:up|Sirpb1b,668101:down|Gas6,14456:up|Col1a2,12843:down|Ceacam1,26365:up
Elastic fibre formation	Reactome	R-MMU-1566948	1.815E-03	2.121E-02	Tgfb2,21808:down|Tgfb3,21809:down|Efemp1,216616:up|Bmp4,12159:up
Antagonism of Activin by Follistatin	Reactome	R-MMU-2473224	1.846E-03	2.122E-02	Fstl3,83554:down|Inhba,16323:down
Complement cascade	Reactome	R-MMU-166658	2.001E-03	2.262E-02	C1s2,317677:up|C1s1,50908:up|C1rb,667277:up|C3,12266:up
Prostate cancer	KEGG PATHWAY	mmu05215	2.050E-03	2.281E-02	Fgfr2,14183:up|Fgfr1,14182:down|Igf1,16000:up|Pdgfc,54635:down|Creb3l1,26427:down|Egfr,13649:down
G alpha (i) signalling events	Reactome	R-MMU-418594	2.199E-03	2.408E-02	Ppbp,57349:down|Ccl9,20308:up|Cxcl12,20315:up|Ccl6,20305:up|Kng1,16644:up|Kng2,385643:up|Cxcl9,17329:up|C3,12266:up|Cxcr6,80901:down
Immune System	Reactome	R-MMU-168256	2.590E-03	2.761E-02	C1s2,317677:up|Cd200,17470:down|Prkar2b,19088:up|Col1a2,12843:down|Nrg1,211323:down|C1rb,667277:up|Sec23a,20334:down|Sirpb1a,320832:down|S100a1,20193:up|Cd4,12504:up|Kitl,17311:up|Ret,19713:up|C1s1,50908:up|Prl2c2,18811:up|Tnfrsf11b,18383:up|Crlf1,12931:down|H2-T24,15042:up|Clec4n,56620:up|Sirpb1b,668101:down|Il1rn,16181:up|Col3a1,12825:down|Egfr,13649:down|Fgfr2,14183:up|Fgfr1,14182:down|Tnfrsf12a,27279:down|Tnf,21926:up|Il1rl1,17082:down|Clec2d,93694:up|C3,12266:up
Metallothioneins bind metals	Reactome	R-MMU-5661231	2.744E-03	2.761E-02	Mt2,17750:up|Mt1,17748:up

Response to metal ions	Reactome	R-MMU-5660526	2.744E-03	2.761E-02	Mt2,17750:up|Mt1,17748:up
Gastrin-CREB signalling pathway via PKC and MAPK	Reactome	R-MMU-881907	2.807E-03	2.761E-02	Ccl9,20308:up|Ret,19713:up|Ccl6,20305:up|Fgfr2,14183:up|Fgfr1,14182:down|Kng1,16644:up|Kng2,385643:up|Kitl,17311:up|Nrg1,211323:down|Ednrb,13618:up|Htr2b,15559:down|Htr2a,15558:down|Egfr,13649:down
Leishmaniasis	KEGG PATHWAY	mmu05140	2.824E-03	2.761E-02	Tgfb2,21808:down|Tnf,21926:up|Ptgs2,19225:down|C3,12266:up|Tgfb3,21809:down
Metabolism of xenobiotics by cytochrome P450	KEGG PATHWAY	mmu00980	2.824E-03	2.761E-02	Gstm1,14862:up|Cbr2,12409:up|Gstt1,14871:up|Aldh1a3,56847:down|Gsta4,14860:up
Iron uptake and transport	Reactome	R-MMU-917937	2.875E-03	2.761E-02	Slc40a1,53945:up|Hmox1,15368:up|Trf,22041:up|Tfrc,22042:up
Formation of Fibrin Clot (Clotting Cascade)	Reactome	R-MMU-140877	2.875E-03	2.761E-02	Serpine2,20720:up|Kng1,16644:up|Kng2,385643:up|Serping1,12258:up
Drug metabolism - cytochrome P450	KEGG PATHWAY	mmu00982	3.192E-03	3.024E-02	Gstm1,14862:up|Gstt1,14871:up|Maob,109731:up|Gsta4,14860:up|Aldh1a3,56847:down
SHC-related events triggered by IGF1R	Reactome	R-MMU-2428933	3.807E-03	3.511E-02	Igf2,16002:up|Igf1,16000:up
Crosslinking of collagen fibrils	Reactome	R-MMU-2243919	3.807E-03	3.511E-02	Col1a2,12843:down|Tll1,21892:down
Rap1 signaling pathway	KEGG PATHWAY	mmu04015	3.971E-03	3.615E-02	Fgfr2,14183:up|Fgfr1,14182:down|Igf1,16000:up|Vegfb,22340:up|Kitl,17311:up|Pdgfc,54635:down|Vegfa,22339:down|Egfr,13649:down|Figf,14205:up
Tuberculosis	KEGG PATHWAY	mmu05152	4.158E-03	3.737E-02	Tgfb2,21808:down|Tnf,21926:up|Mrc1,17533:up|Mrc2,17534:down|Vdr,22337:down|Tgfb3,21809:down|C3,12266:up|Clec4e,56619:up
Signaling pathways regulating pluripotency of stem cells	KEGG PATHWAY	mmu04550	4.399E-03	3.850E-02	Inhba,16323:down|Fgfr2,14183:up|Fgfr1,14182:down|Igf1,16000:up|Bmpr1a,12166:down|Bmp4,12159:up|Wnt5a,22418:down
Immunoregulatory interactions between a Lymphoid and a non-Lymphoid cell	Reactome	R-MMU-198933	4.449E-03	3.850E-02	H2-T24,15042:up|Cd200,17470:down|Col1a2,12843:down|Clec2d,93694:up|Col3a1,12825:down|C3,12266:up
Cargo recognition for clathrin-mediated endocytosis	Reactome	R-MMU-8856825	4.756E-03	4.066E-02	Cd4,12504:up|Trf,22041:up|Egfr,13649:down|Wnt5a,22418:down|Tfrc,22042:up
HIF-1 signaling pathway	KEGG PATHWAY	mmu04066	4.852E-03	4.098E-02	Hmox1,15368:up|Igf1,16000:up|Trf,22041:up|Vegfa,22339:down|Tfrc,22042:up|Egfr,13649:down
Amoebiasis	KEGG PATHWAY	mmu05146	5.282E-03	4.306E-02	Lama2,16773:down|Tnf,21926:up|Col3a1,12825:down|Tgfb3,21809:down|Tgfb2,21808:down|Col1a2,12843:down
PI5P, PP2A and IER3 Regulate PI3K/AKT Signaling	Reactome	R-MMU-6811558	5.861E-03	4.722E-02	Nrg1,211323:down|Kitl,17311:up|Fgfr2,14183:up|Fgfr1,14182:down|Egfr,13649:down
Ras signaling pathway	KEGG PATHWAY	mmu04014	6.035E-03	4.807E-02	Fgfr2,14183:up|Fgfr1,14182:down|Igf1,16000:up|Vegfb,22340:up|Kitl,17311:up|Pdgfc,54635:down|Vegfa,22339:down|Egfr,13649:down|Figf,14205:up
@@Heme degradation	Reactome	R-MMU-189483	6.408E-03	5.047E-02	Hmox1,15368:up|Blvrb,233016:up
Histidine metabolism	KEGG PATHWAY	mmu00340	6.605E-03	5.144E-02	Maob,109731:up|Aldh1a3,56847:down|Hal,15109:up
MicroRNAs in cancer	KEGG PATHWAY	mmu05206	7.164E-03	5.519E-02	Mir199a-2,723821:down|Mirlet7e,387248:down|Mir214,387210:down|Mirlet7c-1,387246:down|Ptgs2,19225:down|Itga5,16402:down|Vegfa,22339:down|Tgfb2,21808:down|Tnc,21923:down|Egfr,13649:down
Negative regulation of the PI3K/AKT network	Reactome	R-MMU-199418	7.481E-03	5.700E-02	Nrg1,211323:down|Kitl,17311:up|Fgfr2,14183:up|Fgfr1,14182:down|Egfr,13649:down

PPAR signaling pathway	KEGG PATHWAY	mmu03320	8.208E-03	6.121E-02	Fabp4,11770:up|Fabp5,16592:up|Adipoq,11450:up|Pparg,19016:up|Lpl,16956:up
Cytokine Signaling in Immune system	Reactome	R-MMU-1280215	9.816E-03	7.243E-02	Tnfrsf12a,27279:down|Crlf1,12931:down|Cd4,12504:up|Ret,19713:up|Fgfr2,14183:up|Fgfr1,14182:down|Kitl,17311:up|Tnf,21926:up|Il1rn,16181:up|Il1rl1,17082:down|Nrg1,211323:down|Prl2c2,18811:up|Tnfrsf11b,18383:up|Egfr,13649:down
G alpha (q) signalling events	Reactome	R-MMU-416476	1.011E-02	7.381E-02	Ccl9,20308:up|Ccl6,20305:up|Kng1,16644:up|Kng2,385643:up|Ednrb,13618:up|Htr2b,15559:down|Htr2a,15558:down
Formyl peptide receptors bind formyl peptides and many other ligands	Reactome	R-MMU-444473	1.143E-02	8.177E-02	Ccl6,20305:up|Ccl9,20308:up
Chemical carcinogenesis	KEGG PATHWAY	mmu05204	1.161E-02	8.220E-02	Gstm1,14862:up|Ptgs2,19225:down|Gstt1,14871:up|Aldh1a3,56847:down|Gsta4,14860:up
Herpes simplex infection	KEGG PATHWAY	mmu05168	1.229E-02	8.618E-02	H2-T24,15042:up|Oas3,246727:up|Ifit1,15957:up|Tnf,21926:up|Ccl2,20296:up|Oas1a,246730:up|C3,12266:up|Ccl12,20293:up
Signaling by GPCR	Reactome	R-MMU-372790	1.277E-02	8.861E-02	Glp1r,14652:down|Ret,19713:up|Prkar2b,19088:up|Kng1,16644:up|Ccl2,20296:up|Ppbp,57349:down|Ccl3,20302:up|Nrg1,211323:down|Ccl17,20295:down|Htr2b,15559:down|Cxcr6,80901:down|Ednrb,13618:up|Cxcl12,20315:up|Htr2a,15558:down|Kitl,17311:up|Kng2,385643:up|Egfr,13649:down|Ccl9,20308:up|Ccl6,20305:up|Ccl7,20306:up|Fgfr1,14182:down|Adm,11535:up|Ccl4,20303:up|Cxcl9,17329:up|Ptgir,19222:up|Cx3cr1,13051:down|C3,12266:up|Fgfr2,14183:up
Taurine and hypotaurine metabolism	KEGG PATHWAY	mmu00430	1.339E-02	8.938E-02	Cdo1,12583:up|Ggt5,23887:up
SHC1 events in ERBB2 signaling	Reactome	R-MMU-1250196	1.339E-02	8.938E-02	Nrg1,211323:down|Egfr,13649:down
Serotonin receptors	Reactome	R-MMU-390666	1.339E-02	8.938E-02	Htr2b,15559:down|Htr2a,15558:down
RA biosynthesis pathway	Reactome	R-MMU-5365859	1.430E-02	9.459E-02	Akr1c18,105349:up|Aldh1a2,19378:up|Aldh1a3,56847:down
Renin-angiotensin system	KEGG PATHWAY	mmu04614	1.537E-02	9.552E-02	Klk1b11,16613:up|Mme,17380:up|Anpep,16790:up
Signal regulatory protein (SIRP) family interactions	Reactome	R-MMU-391160	1.548E-02	9.552E-02	Sirpb1a,320832:down|Sirpb1b,668101:down
Signaling by Activin	Reactome	R-MMU-1502540	1.548E-02	9.552E-02	Fstl3,83554:down|Inhba,16323:down
ERBB2 Activates PTK6 Signaling	Reactome	R-MMU-8847993	1.548E-02	9.552E-02	Nrg1,211323:down|Egfr,13649:down
Phagosome	KEGG PATHWAY	mmu04145	1.584E-02	9.552E-02	H2-T24,15042:up|Thbs2,21826:down|Itga5,16402:down|Tfrc,22042:up|Mrc2,17534:down|C3,12266:up|Mrc1,17533:up
Central carbon metabolism in cancer	KEGG PATHWAY	mmu05230	1.600E-02	9.552E-02	Ret,19713:up|Fgfr2,14183:up|Fgfr1,14182:down|Egfr,13649:down
Pancreatic cancer	KEGG PATHWAY	mmu05212	1.600E-02	9.552E-02	Tgfb2,21808:down|Tgfb3,21809:down|Egfr,13649:down|Vegfa,22339:down
PI-3K cascade:FGFR4	Reactome	R-MMU-5654720	1.646E-02	9.552E-02	Nrg1,211323:down|Kitl,17311:up|Fgfr2,14183:up|Fgfr1,14182:down|Egfr,13649:down
PI-3K cascade:FGFR3	Reactome	R-MMU-5654710	1.646E-02	9.552E-02	Nrg1,211323:down|Kitl,17311:up|Fgfr2,14183:up|Fgfr1,14182:down|Egfr,13649:down
PI-3K cascade:FGFR1	Reactome	R-MMU-5654689	1.646E-02	9.552E-02	Nrg1,211323:down|Kitl,17311:up|Fgfr2,14183:up|Fgfr1,14182:down|Egfr,13649:down
PI-3K cascade:FGFR2	Reactome	R-MMU-5654695	1.646E-02	9.552E-02	Nrg1,211323:down|Kitl,17311:up|Fgfr2,14183:up|Fgfr1,14182:down|Egfr,13649:down
PI3K events in ERBB4 signaling	Reactome	R-MMU-1250342	1.646E-02	9.552E-02	Nrg1,211323:down|Kitl,17311:up|Fgfr2,14183:up|Fgfr1,14182:down|Egfr,13649:down
PIP3 activates AKT signaling	Reactome	R-MMU-1257604	1.646E-02	9.552E-02	Nrg1,211323:down|Kitl,17311:up|Fgfr2,14183:up|Fgfr1,14182:down|Egfr,13649:down

Renal cell carcinoma	KEGG PATHWAY	mmu05211	1.677E-02	9.639E-02	Tgfb2,21808:down|Tgfb3,21809:down|Vegfa,22339:down|Epas1,13819:up
GAB1 signalosome	Reactome	R-MMU-180292	1.833E-02	1.036E-01	Nrg1,211323:down|Kitl,17311:up|Fgfr2,14183:up|Fgfr1,14182:down|Egfr,13649:down
PI3K/AKT activation	Reactome	R-MMU-198203	1.833E-02	1.036E-01	Nrg1,211323:down|Kitl,17311:up|Fgfr2,14183:up|Fgfr1,14182:down|Egfr,13649:down
Clathrin-mediated endocytosis	Reactome	R-MMU-8856828	1.966E-02	1.101E-01	Cd4,12504:up|Trf,22041:up|Egfr,13649:down|Wnt5a,22418:down|Tfrc,22042:up
Hormone-sensitive lipase (HSL)-mediated triacylglycerol hydrolysis	Reactome	R-MMU-163560	2.004E-02	1.101E-01	Fabp4,11770:up|Fabp5,16592:up
ERBB2 Regulates Cell Motility	Reactome	R-MMU-6785631	2.004E-02	1.101E-01	Nrg1,211323:down|Egfr,13649:down
Melanoma	KEGG PATHWAY	mmu05218	2.011E-02	1.101E-01	Pdgfc,54635:down|Fgfr1,14182:down|Igf1,16000:up|Egfr,13649:down
Signaling by VEGF	Reactome	R-MMU-194138	2.070E-02	1.125E-01	Ret,19713:up|Fgfr2,14183:up|Fgfr1,14182:down|Vegfb,22340:up|Kitl,17311:up|Vegfa,22339:down|Nrg1,211323:down|Egfr,13649:down|Figf,14205:up
TNF signaling pathway	KEGG PATHWAY	mmu04668	2.104E-02	1.135E-01	Creb3l1,26427:down|Tnf,21926:up|Ptgs2,19225:down|Ccl12,20293:up|Ccl2,20296:up
Glycosaminoglycan metabolism	Reactome	R-MMU-1630316	2.176E-02	1.155E-01	Chst11,58250:down|Prelp,116847:up|Hpse,15442:up|Vcan,13003:down|Gpc4,14735:down
Role of LAT2/NTAL/LAB on calcium mobilization	Reactome	R-MMU-2730905	2.176E-02	1.155E-01	Nrg1,211323:down|Kitl,17311:up|Fgfr2,14183:up|Fgfr1,14182:down|Egfr,13649:down
Anchoring fibril formation	Reactome	R-MMU-2214320	2.251E-02	1.178E-01	Col1a2,12843:down|Tll1,21892:down
PI3K events in ERBB2 signaling	Reactome	R-MMU-1963642	2.251E-02	1.178E-01	Nrg1,211323:down|Egfr,13649:down
Bladder cancer	KEGG PATHWAY	mmu05219	2.272E-02	1.180E-01	Mmp2,17390:down|Egfr,13649:down|Vegfa,22339:down
Arachidonic acid metabolism	Reactome	R-MMU-2142753	2.383E-02	1.228E-01	Hpgd,15446:up|Ptgs2,19225:down|Akr1c18,105349:up|Ggt5,23887:up
DAP12 interactions	Reactome	R-MMU-2172127	2.694E-02	1.334E-01	Sirpb1a,320832:down|Prkar2b,19088:up|Ret,19713:up|Fgfr2,14183:up|Fgfr1,14182:down|Sirpb1b,668101:down|Kitl,17311:up|Nrg1,211323:down|Egfr,13649:down
@@Disease	Reactome	R-MMU-1643685	2.740E-02	1.334E-01	Fgfr2,14183:up
Signaling by FGFR2 IIIa TM	Reactome	R-MMU-8851708	2.740E-02	1.334E-01	Fgfr2,14183:up
FGFR2 mutant receptor activation	Reactome	R-MMU-1839126	2.740E-02	1.334E-01	Fgfr2,14183:up
Signaling by FGFR2 in disease	Reactome	R-MMU-5655253	2.740E-02	1.334E-01	Fgfr2,14183:up
Signaling by FGFR in disease	Reactome	R-MMU-1226099	2.740E-02	1.334E-01	Fgfr2,14183:up
Diseases of signal transduction	Reactome	R-MMU-5663202	2.740E-02	1.334E-01	Fgfr2,14183:up
Innate Immune System	Reactome	R-MMU-168249	2.750E-02	1.334E-01	Sirpb1a,320832:down|Prkar2b,19088:up|S100a1,20193:up|Ret,19713:up|Fgfr2,14183:up|Fgfr1,14182:down|Sirpb1b,668101:down|Kitl,17311:up|C1s2,317677:up|Nrg1,211323:down|C1s1,50908:up|C1rb,667277:up|C3,12266:up|Clec4n,56620:up|Egfr,13649:down
Signaling by Type 1 Insulin-like Growth Factor 1 Receptor (IGF1R)	Reactome	R-MMU-2404192	2.797E-02	1.334E-01	Kitl,17311:up|Ret,19713:up|Fgfr2,14183:up|Fgfr1,14182:down|Igf2,16002:up|Igf1,16000:up|Nrg1,211323:down|Egfr,13649:down
IRS-related events triggered by IGF1R	Reactome	R-MMU-2428928	2.797E-02	1.334E-01	Kitl,17311:up|Ret,19713:up|Fgfr2,14183:up|Fgfr1,14182:down|Igf2,16002:up|Igf1,16000:up|Nrg1,211323:down|Egfr,13649:down
IGF1R signaling cascade	Reactome	R-MMU-2428924	2.797E-02	1.334E-01	Kitl,17311:up|Ret,19713:up|Fgfr2,14183:up|Fgfr1,14182:down|Igf2,16002:up|Igf1,16000:up|Nrg1,211323:down|Egfr,13649:down

Chondroitin sulfate/dermatan sulfate metabolism	Reactome	R-MMU-1793185	3.007E-02	1.403E-01	Chst11,58250:down|Vcan,13003:down|Gpc4,14735:down
Retinoid metabolism and transport	Reactome	R-MMU-975634	3.007E-02	1.403E-01	Akr1c18,105349:up|Lpl,16956:up|Gpc4,14735:down
VEGFR2 mediated cell proliferation	Reactome	R-MMU-5218921	3.019E-02	1.403E-01	Ret,19713:up|Fgfr2,14183:up|Fgfr1,14182:down|Kitl,17311:up|Vegfa,22339:down|Nrg1,211323:down|Egfr,13649:down
Pantothenate and CoA biosynthesis	KEGG PATHWAY	mmu00770	3.061E-02	1.403E-01	Enpp3,209558:down|Enpp1,18605:down
Other semaphorin interactions	Reactome	R-MMU-416700	3.061E-02	1.403E-01	Plxnd1,67784:up|Sema7a,20361:down
Metabolism of porphyrins	Reactome	R-MMU-189445	3.061E-02	1.403E-01	Hmox1,15368:up|Blvrb,233016:up
Activation of Matrix Metalloproteinases	Reactome	R-MMU-1592389	3.167E-02	1.432E-01	Klk1b11,16613:up|Mmp2,17390:down|Mmp13,17386:up
Heparan sulfate/heparin (HS-GAG) metabolism	Reactome	R-MMU-1638091	3.167E-02	1.432E-01	Hpse,15442:up|Vcan,13003:down|Gpc4,14735:down
Signal transduction by L1	Reactome	R-MMU-445144	3.353E-02	1.497E-01	Fgfr1,14182:down|Egfr,13649:down
Chondroitin sulfate biosynthesis	Reactome	R-MMU-2022870	3.353E-02	1.497E-01	Chst11,58250:down|Vcan,13003:down
Signaling by Interleukins	Reactome	R-MMU-449147	3.468E-02	1.539E-01	Crlf1,12931:down|Cd4,12504:up|Ret,19713:up|Fgfr2,14183:up|Fgfr1,14182:down|Kitl,17311:up|Il1rn,16181:up|Il1rl1,17082:down|Nrg1,211323:down|Egfr,13649:down
Transcriptional regulation of white adipocyte differentiation	Reactome	R-MMU-381340	3.500E-02	1.543E-01	Tnf,21926:up|Pparg,19016:up|Fabp4,11770:up
Gap junction	KEGG PATHWAY	mmu04540	3.608E-02	1.580E-01	Pdgfc,54635:down|Htr2b,15559:down|Htr2a,15558:down|Egfr,13649:down
HS-GAG degradation	Reactome	R-MMU-2024096	3.655E-02	1.580E-01	Hpse,15442:up|Gpc4,14735:down
Synthesis of Prostaglandins (PG) and Thromboxanes (TX)	Reactome	R-MMU-2162123	3.655E-02	1.580E-01	Ptgs2,19225:down|Akr1c18,105349:up
Arginine and proline metabolism	KEGG PATHWAY	mmu00330	3.674E-02	1.580E-01	P4ha1,18451:down|Gatm,67092:down|Maob,109731:up
RET signaling	Reactome	R-MMU-8853659	3.721E-02	1.590E-01	Dok2,13449:up|Ret,19713:up|Fgfr2,14183:up|Fgfr1,14182:down|Kitl,17311:up|Nrg1,211323:down|Egfr,13649:down
Staphylococcus aureus infection	KEGG PATHWAY	mmu05150	3.851E-02	1.636E-01	C1s1,50908:up|C1s2,317677:up|C3,12266:up
Fructose biosynthesis	Reactome	R-MMU-5652227	4.082E-02	1.654E-01	Sord,20322:up
Biogenic amines are oxidatively deaminated to aldehydes by MAOA and MAOB	Reactome	R-MMU-141333	4.082E-02	1.654E-01	Maob,109731:up
TP53 Regulates Transcription of Death Receptors and Ligands	Reactome	R-MMU-6803211	4.082E-02	1.654E-01	Igfbp3,16009:up
Transcriptional misregulation in cancer	KEGG PATHWAY	mmu05202	4.191E-02	1.688E-01	Hpgd,15446:up|Pparg,19016:up|Plat,18791:down|Igf1,16000:up|Lyl1,17095:up|Igfbp3,16009:up
Metabolism of fat-soluble vitamins	Reactome	R-MMU-6806667	4.219E-02	1.690E-01	Akr1c18,105349:up|Lpl,16956:up|Gpc4,14735:down

Downstream signaling of activated FGFR1	Reactome	R-MMU-5654687	4.351E-02	1.733E-01	Prkar2b,19088:up|Ret,19713:up|Fgfr2,14183:up|Fgfr1,14182:down|Kitl,17311:up|Flrt2,399558:down|Nrg1,211323:down|Egfr,13649:down
FoxO signaling pathway	KEGG PATHWAY	mmu04068	4.386E-02	1.737E-01	Tgfb2,21808:down|Tgfb3,21809:down|Igf1,16000:up|Egfr,13649:down|Plk2,20620:down
Endocrine and other factor-regulated calcium reabsorption	KEGG PATHWAY	mmu04961	4.410E-02	1.737E-01	Vdr,22337:down|Klk1b11,16613:up|Fxyd2,11936:up
Semaphorin interactions	Reactome	R-MMU-373755	4.604E-02	1.790E-01	Plxnd1,67784:up|Sema7a,20361:down|Dpysl3,22240:down
Hepatitis C	KEGG PATHWAY	mmu05160	4.615E-02	1.790E-01	Oas1a,246730:up|Tnf,21926:up|Oas3,246727:up|Ifit1,15957:up|Egfr,13649:down
Phenylalanine metabolism	KEGG PATHWAY	mmu00360	4.621E-02	1.790E-01	Maob,109731:up|Aldh1a3,56847:down
Signaling by FGFR1	Reactome	R-MMU-5654736	4.734E-02	1.823E-01	Prkar2b,19088:up|Ret,19713:up|Fgfr2,14183:up|Fgfr1,14182:down|Kitl,17311:up|Flrt2,399558:down|Nrg1,211323:down|Egfr,13649:down
Longevity regulating pathway	KEGG PATHWAY	mmu04211	4.837E-02	1.853E-01	Creb3l1,26427:down|Igf1,16000:up|Adipoq,11450:up|Pparg,19016:up
A tetrasaccharide linker sequence is required for GAG synthesis	Reactome	R-MMU-1971475	4.962E-02	1.890E-01	Vcan,13003:down|Gpc4,14735:down
NOD-like receptor signaling pathway	KEGG PATHWAY	mmu04621	5.213E-02	1.919E-01	Ccl2,20296:up|Tnf,21926:up|Ccl12,20293:up
Adaptive Immune System	Reactome	R-MMU-1280218	5.232E-02	1.919E-01	Sec23a,20334:down|H2-T24,15042:up|Cd200,17470:down|Fgfr2,14183:up|Fgfr1,14182:down|Kitl,17311:up|Clec2d,93694:up|Col1a2,12843:down|Nrg1,211323:down|Col3a1,12825:down|C3,12266:up|Cd4,12504:up|Egfr,13649:down
NF-kappa B signaling pathway	KEGG PATHWAY	mmu04064	5.291E-02	1.919E-01	Tnf,21926:up|Ptgs2,19225:down|Cxcl12,20315:up|Ccl4,20303:up
Oxygen-dependent asparagine hydroxylation of Hypoxia-inducible Factor Alpha	Reactome	R-MMU-1234162	5.405E-02	1.919E-01	Epas1,13819:up
WNT5A-dependent internalization of FZD2, FZD5 and ROR2	Reactome	R-MMU-5140745	5.405E-02	1.919E-01	Wnt5a,22418:down
Vitamins B6 activation to pyridoxal phosphate	Reactome	R-MMU-964975	5.405E-02	1.919E-01	Pdxk,216134:up
Alternative complement activation	Reactome	R-MMU-173736	5.405E-02	1.919E-01	C3,12266:up
FGFR1c and Klotho ligand binding and activation	Reactome	R-MMU-190374	5.405E-02	1.919E-01	Fgfr1,14182:down
Inflammatory bowel disease (IBD)	KEGG PATHWAY	mmu05321	5.425E-02	1.919E-01	Tgfb2,21808:down|Tnf,21926:up|Tgfb3,21809:down
Melanogenesis	KEGG PATHWAY	mmu04916	5.448E-02	1.919E-01	Kitl,17311:up|Creb3l1,26427:down|Ednrb,13618:up|Wnt5a,22418:down
Metabolism of vitamins and cofactors	Reactome	R-MMU-196854	5.598E-02	1.962E-01	Ptgs2,19225:down|Pdxk,216134:up|Akr1c18,105349:up|Lpl,16956:up|Gpc4,14735:down
Toll-like receptor signaling pathway	KEGG PATHWAY	mmu04620	5.769E-02	2.012E-01	Tnf,21926:up|Ccl4,20303:up|Ccl3,20302:up|Cxcl9,17329:up
L1CAM interactions	Reactome	R-MMU-373760	5.859E-02	2.024E-01	Fgfr1,14182:down|Cd24a,12484:down|Egfr,13649:down
Wnt signaling pathway	KEGG PATHWAY	mmu04310	5.861E-02	2.024E-01	Sfrp4,20379:up|Sfrp2,20319:up|Sfrp1,20377:up|Wnt5a,22418:down|Gpc4,14735:down

Signaling by EGFR	Reactome	R-MMU-177929	5.922E-02	2.035E-01	Prkar2b,19088:up|Ret,19713:up|Fgfr2,14183:up|Fgfr1,14182:down|Kit1,17311:up|Adam12,11489:down|Nrg1,211323:down|Egfr,13649:down
Transferrin endocytosis and recycling	Reactome	R-MMU-917977	6.035E-02	2.054E-01	Trf,22041:up|Tfrc,22042:up
TRP channels	Reactome	R-MMU-3295583	6.035E-02	2.054E-01	Mcoln3,171166:up|Mcoln2,68279:up
Signaling by ERBB4	Reactome	R-MMU-1236394	6.484E-02	2.081E-01	Cxcl12,20315:up|Ret,19713:up|Fgfr2,14183:up|Fgfr1,14182:down|Kit1,17311:up|Nrg1,211323:down|Prl2c2,18811:up|Egfr,13649:down
GRB2 events in EGFR signaling	Reactome	R-MMU-179812	6.547E-02	2.081E-01	Ret,19713:up|Fgfr2,14183:up|Fgfr1,14182:down|Kit1,17311:up|Nrg1,211323:down|Egfr,13649:down
Signalling to p38 via RIT and RIN	Reactome	R-MMU-187706	6.547E-02	2.081E-01	Ret,19713:up|Fgfr2,14183:up|Fgfr1,14182:down|Kit1,17311:up|Nrg1,211323:down|Egfr,13649:down
SHC1 events in ERBB4 signaling	Reactome	R-MMU-1250347	6.547E-02	2.081E-01	Ret,19713:up|Fgfr2,14183:up|Fgfr1,14182:down|Kit1,17311:up|Nrg1,211323:down|Egfr,13649:down
SOS-mediated signalling	Reactome	R-MMU-112412	6.547E-02	2.081E-01	Ret,19713:up|Fgfr2,14183:up|Fgfr1,14182:down|Kit1,17311:up|Nrg1,211323:down|Egfr,13649:down
RAF/MAP kinase cascade	Reactome	R-MMU-5673001	6.547E-02	2.081E-01	Ret,19713:up|Fgfr2,14183:up|Fgfr1,14182:down|Kit1,17311:up|Nrg1,211323:down|Egfr,13649:down
SHC1 events in EGFR signaling	Reactome	R-MMU-180336	6.547E-02	2.081E-01	Ret,19713:up|Fgfr2,14183:up|Fgfr1,14182:down|Kit1,17311:up|Nrg1,211323:down|Egfr,13649:down
FRS-mediated FGFR2 signaling	Reactome	R-MMU-5654700	6.669E-02	2.081E-01	Ret,19713:up|Fgfr2,14183:up|Fgfr1,14182:down|Kit1,17311:up|Nrg1,211323:down|Egfr,13649:down
FRS-mediated FGFR4 signaling	Reactome	R-MMU-5654712	6.669E-02	2.081E-01	Ret,19713:up|Fgfr2,14183:up|Fgfr1,14182:down|Kit1,17311:up|Nrg1,211323:down|Egfr,13649:down
FRS-mediated FGFR3 signaling	Reactome	R-MMU-5654706	6.669E-02	2.081E-01	Ret,19713:up|Fgfr2,14183:up|Fgfr1,14182:down|Kit1,17311:up|Nrg1,211323:down|Egfr,13649:down
FRS-mediated FGFR1 signaling	Reactome	R-MMU-5654693	6.669E-02	2.081E-01	Ret,19713:up|Fgfr2,14183:up|Fgfr1,14182:down|Kit1,17311:up|Nrg1,211323:down|Egfr,13649:down
Riboflavin metabolism	KEGG PATHWAY	mmu00740	6.711E-02	2.081E-01	Blvrb,233016:up
Amine Oxidase reactions	Reactome	R-MMU-140179	6.711E-02	2.081E-01	Maob,109731:up
Prostacyclin signalling through prostacyclin receptor	Reactome	R-MMU-392851	6.711E-02	2.081E-01	Ptgir,19222:up
Fibronectin matrix formation	Reactome	R-MMU-1566977	6.711E-02	2.081E-01	Ceacam1,26365:up
Dectin-2 family	Reactome	R-MMU-5621480	6.711E-02	2.081E-01	Clec4n,56620:up
Thyroid cancer	KEGG PATHWAY	mmu05216	6.790E-02	2.097E-01	Ret,19713:up|Pparg,19016:up
Hippo signaling pathway	KEGG PATHWAY	mmu04390	6.984E-02	2.147E-01	Tgfb2,21808:down|Tgfb3,21809:down|Bmpr1a,12166:down|Bmp4,12159:up|Wnt5a,22418:down
Frs2-mediated activation	Reactome	R-MMU-170968	7.171E-02	2.160E-01	Ret,19713:up|Fgfr2,14183:up|Fgfr1,14182:down|Kit1,17311:up|Nrg1,211323:down|Egfr,13649:down
MAPK1/MAPK3 signaling	Reactome	R-MMU-5684996	7.171E-02	2.160E-01	Ret,19713:up|Fgfr2,14183:up|Fgfr1,14182:down|Kit1,17311:up|Nrg1,211323:down|Egfr,13649:down
Signalling to RAS	Reactome	R-MMU-167044	7.171E-02	2.160E-01	Ret,19713:up|Fgfr2,14183:up|Fgfr1,14182:down|Kit1,17311:up|Nrg1,211323:down|Egfr,13649:down
ARMS-mediated activation	Reactome	R-MMU-170984	7.171E-02	2.160E-01	Ret,19713:up|Fgfr2,14183:up|Fgfr1,14182:down|Kit1,17311:up|Nrg1,211323:down|Egfr,13649:down
EGFR interacts with phospholipase C-gamma	Reactome	R-MMU-212718	7.179E-02	2.160E-01	Prkar2b,19088:up|Egfr,13649:down
Peptide hormone metabolism	Reactome	R-MMU-2980736	7.256E-02	2.172E-01	Igf1,16000:up|Inhba,16323:down|Mme,17380:up
@@Signaling by FGFR	Reactome	R-MMU-190236	7.283E-02	2.172E-01	Prkar2b,19088:up|Ret,19713:up|Fgfr2,14183:up|Fgfr1,14182:down|Kit1,17311:up|Flrt2,399558:down|Nrg1,211323:down|Egfr,13649:down
Signaling by Leptin	Reactome	R-MMU-2586552	7.431E-02	2.207E-01	Ret,19713:up|Fgfr2,14183:up|Fgfr1,14182:down|Kit1,17311:up|Nrg1,211323:down|Egfr,13649:down

Transmembrane transport of small molecules	Reactome	R-MMU-382551	7.500E-02	2.218E-01	Hmox1,15368:up|Ank,11732:down|Fxyd2,11936:up|Prkar2b,19088:up|Fxyd1,56188:up|Emb,13723:up|Trf,22041:up|Mcoln2,68279:up|Tfrc,22042:up|Slc40a1,53945:up|Mcoln3,171166:up|Slc29a1,63959:up|Slc2a6,227659:up
Prolonged ERK activation events	Reactome	R-MMU-169893	7.562E-02	2.218E-01	Ret,19713:up|Fgfr2,14183:up|Fgfr1,14182:down|Kitl,17311:up|Nrg1,211323:down|Egfr,13649:down
Interleukin receptor SHC signaling	Reactome	R-MMU-912526	7.562E-02	2.218E-01	Ret,19713:up|Fgfr2,14183:up|Fgfr1,14182:down|Kitl,17311:up|Nrg1,211323:down|Egfr,13649:down
Toxoplasmosis	KEGG PATHWAY	mmu05145	7.897E-02	2.270E-01	Tgfb2,21808:down|Tnf,21926:up|Lama2,16773:down|Tgfb3,21809:down
Catabolism of glucuronate to xylulose-5-phosphate	Reactome	R-MMU-5661270	7.998E-02	2.270E-01	Sord,20322:up
Activation of C3 and C5	Reactome	R-MMU-174577	7.998E-02	2.270E-01	C3,12266:up
GRB7 events in ERBB2 signaling	Reactome	R-MMU-1306955	7.998E-02	2.270E-01	Nrg1,211323:down
Synthesis of Lipoxins (LX)	Reactome	R-MMU-2142700	7.998E-02	2.270E-01	Hpgd,15446:up
Synthesis of 15-eicosatetraenoic acid derivatives	Reactome	R-MMU-2142770	7.998E-02	2.270E-01	Ptgs2,19225:down
Signalling to ERKs	Reactome	R-MMU-187687	8.240E-02	2.329E-01	Ret,19713:up|Fgfr2,14183:up|Fgfr1,14182:down|Kitl,17311:up|Nrg1,211323:down|Egfr,13649:down
Interleukin-2 signaling	Reactome	R-MMU-451927	8.520E-02	2.389E-01	Ret,19713:up|Fgfr2,14183:up|Fgfr1,14182:down|Kitl,17311:up|Nrg1,211323:down|Egfr,13649:down
Regulation of actin cytoskeleton	KEGG PATHWAY	mmu04810	8.950E-02	2.432E-01	Itga11,319480:down|Fgfr2,14183:up|Fgfr1,14182:down|Pdgfc,54635:down|Itga5,16402:down|Egfr,13649:down
Arrhythmogenic right ventricular cardiomyopathy (ARVC)	KEGG PATHWAY	mmu05412	9.054E-02	2.432E-01	Itga11,319480:down|Lama2,16773:down|Itga5,16402:down
Interleukin-3, 5 and GM-CSF signaling	Reactome	R-MMU-512988	9.096E-02	2.432E-01	Ret,19713:up|Fgfr2,14183:up|Fgfr1,14182:down|Kitl,17311:up|Nrg1,211323:down|Egfr,13649:down
Nicotinate and nicotinamide metabolism	KEGG PATHWAY	mmu00760	9.222E-02	2.432E-01	Enpp3,209558:down|Enpp1,18605:down
Amine ligand-binding receptors	Reactome	R-MMU-375280	9.222E-02	2.432E-01	Htr2b,15559:down|Htr2a,15558:down
Signaling by ERBB2	Reactome	R-MMU-1227986	9.222E-02	2.432E-01	Nrg1,211323:down|Egfr,13649:down
AMPK inhibits chREBP transcriptional activation activity	Reactome	R-MMU-163680	9.267E-02	2.432E-01	Adipoq,11450:up
Proton-coupled monocarboxylate transport	Reactome	R-MMU-433692	9.267E-02	2.432E-01	Emb,13723:up
PTK6 promotes HIF1A stabilization	Reactome	R-MMU-8857538	9.267E-02	2.432E-01	Egfr,13649:down
FGFR1b ligand binding and activation	Reactome	R-MMU-190370	9.267E-02	2.432E-01	Fgfr1,14182:down
TNFR1-mediated ceramide production	Reactome	R-MMU-5626978	9.267E-02	2.432E-01	Tnf,21926:up
Downstream signaling of activated FGFR4	Reactome	R-MMU-5654716	9.296E-02	2.432E-01	Prkar2b,19088:up|Ret,19713:up|Fgfr2,14183:up|Fgfr1,14182:down|Kitl,17311:up|Nrg1,211323:down|Egfr,13649:down
Downstream signaling of activated FGFR2	Reactome	R-MMU-5654696	9.296E-02	2.432E-01	Prkar2b,19088:up|Ret,19713:up|Fgfr2,14183:up|Fgfr1,14182:down|Kitl,17311:up|Nrg1,211323:down|Egfr,13649:down

Downstream signaling of activated FGFR3	Reactome	R-MMU-5654708	9.296E-02	2.432E-01	Prkar2b,19088:up|Ret,19713:up|Fgfr2,14183:up|Fgfr1,14182:down|Kitl,17311:up|Nrg1,211323:down|Egfr,13649:down
Downstream signaling events of B Cell Receptor (BCR)	Reactome	R-MMU-1168372	9.384E-02	2.445E-01	Nrg1,211323:down|Kitl,17311:up|Fgfr2,14183:up|Fgfr1,14182:down|Egfr,13649:down
FCERI mediated MAPK activation	Reactome	R-MMU-2871796	9.542E-02	2.472E-01	Ret,19713:up|Fgfr2,14183:up|Fgfr1,14182:down|Kitl,17311:up|Nrg1,211323:down|Egfr,13649:down
Influenza A	KEGG PATHWAY	mmu05164	9.557E-02	2.472E-01	Oas1a,246730:up|Tnf,21926:up|Ccl2,20296:up|Oas3,246727:up|Ccl12,20293:up
Signaling by FGFR4	Reactome	R-MMU-5654743	9.697E-02	2.499E-01	Prkar2b,19088:up|Ret,19713:up|Fgfr2,14183:up|Fgfr1,14182:down|Kitl,17311:up|Nrg1,211323:down|Egfr,13649:down
VEGFA-VEGFR2 Pathway	Reactome	R-MMU-4420097	9.832E-02	2.515E-01	Ret,19713:up|Fgfr2,14183:up|Fgfr1,14182:down|Kitl,17311:up|Vegfa,22339:down|Nrg1,211323:down|Egfr,13649:down
Signaling by FGFR3	Reactome	R-MMU-5654741	9.832E-02	2.515E-01	Prkar2b,19088:up|Ret,19713:up|Fgfr2,14183:up|Fgfr1,14182:down|Kitl,17311:up|Nrg1,211323:down|Egfr,13649:down
Platinum drug resistance	KEGG PATHWAY	mmu01524	1.016E-01	2.587E-01	Gstm1,14862:up|Gstt1,14871:up|Gsta4,14860:up
MAPK family signaling cascades	Reactome	R-MMU-5683057	1.047E-01	2.587E-01	Ret,19713:up|Fgfr2,14183:up|Fgfr1,14182:down|Kitl,17311:up|Nrg1,211323:down|Egfr,13649:down
Glycerophospholipid catabolism	Reactome	R-MMU-6814848	1.052E-01	2.587E-01	Gdpd1,66569:up
Regulation of gene expression by Hypoxia-inducible Factor	Reactome	R-MMU-1234158	1.052E-01	2.587E-01	Epas1,13819:up
Histidine catabolism	Reactome	R-MMU-70921	1.052E-01	2.587E-01	Hal,15109:up
Creatine metabolism	Reactome	R-MMU-71288	1.052E-01	2.587E-01	Gatm,67092:down
Degradation of cysteine and homocysteine	Reactome	R-MMU-1614558	1.052E-01	2.587E-01	Cdo1,12583:up
Nicotinamide salvaging	Reactome	R-MMU-197264	1.052E-01	2.587E-01	Ptgs2,19225:down
Fructose metabolism	Reactome	R-MMU-5652084	1.052E-01	2.587E-01	Sord,20322:up
Downstream signal transduction	Reactome	R-MMU-186763	1.095E-01	2.677E-01	Prkar2b,19088:up|Ret,19713:up|Fgfr2,14183:up|Fgfr1,14182:down|Kitl,17311:up|Nrg1,211323:down|Egfr,13649:down
Tyrosine metabolism	KEGG PATHWAY	mmu00350	1.096E-01	2.677E-01	Maob,109731:up|Aldh1a3,56847:down
Endocytosis	KEGG PATHWAY	mmu04144	1.110E-01	2.702E-01	H2-T24,15042:up|Ret,19713:up|Fgfr2,14183:up|Tfrc,22042:up|Tgfb2,21808:down|Tgfb3,21809:down|Egfr,13649:down
AMPK signaling pathway	KEGG PATHWAY	mmu04152	1.125E-01	2.729E-01	Creb3l1,26427:down|Igf1,16000:up|Adipoq,11450:up|Pparg,19016:up
Glycine, serine and threonine metabolism	KEGG PATHWAY	mmu00260	1.141E-01	2.748E-01	Gatm,67092:down|Maob,109731:up
Aldosterone-regulated sodium reabsorption	KEGG PATHWAY	mmu04960	1.141E-01	2.748E-01	Fxyd2,11936:up|Igf1,16000:up
DAP12 signaling	Reactome	R-MMU-2424491	1.169E-01	2.762E-01	Prkar2b,19088:up|Ret,19713:up|Fgfr2,14183:up|Fgfr1,14182:down|Kitl,17311:up|Nrg1,211323:down|Egfr,13649:down
TNFR1-induced proapoptotic signaling	Reactome	R-MMU-5357786	1.175E-01	2.762E-01	Tnf,21926:up

Transport of nucleosides and free purine and pyrimidine bases across the plasma membrane	Reactome	R-MMU-83936	1.175E-01	2.762E-01	Slc29a1,63959:up
FGFR2b ligand binding and activation	Reactome	R-MMU-190377	1.175E-01	2.762E-01	Fgfr2,14183:up
Negative regulation of TCF-dependent signaling by WNT ligand antagonists	Reactome	R-MMU-3772470	1.175E-01	2.762E-01	Wnt5a,22418:down
Synthesis of UDP-N-acetyl-glucosamine	Reactome	R-MMU-446210	1.175E-01	2.762E-01	Gfpt2,14584:down
Porphyrin and chlorophyll metabolism	KEGG PATHWAY	mmu00860	1.186E-01	2.762E-01	Hmox1,15368:up|Blvrb,233016:up
Antigen Presentation: Folding, assembly and peptide loading of class I MHC	Reactome	R-MMU-983170	1.186E-01	2.762E-01	Sec23a,20334:down|H2-T24,15042:up
Serotonergic synapse	KEGG PATHWAY	mmu04726	1.194E-01	2.772E-01	Ptgs2,19225:down|Htr2b,15559:down|Htr2a,15558:down|Maob,109731:up
Metabolism	Reactome	R-MMU-1430728	1.203E-01	2.782E-01	Glp1r,14652:down|Prkar2b,19088:up|Mt1,17748:up|Hal,15109:up|Cdo1,12583:up|Maob,109731:up|Hmox1,15368:up|Blvrb,233016:up|Gpc4,14735:down|Prelp,116847:up|Vcan,13003:down|Ddah1,69219:down|Gdpd1,66569:up|Hpgd,15446:up|Gatm,67092:down|Hpse,15442:up|Fabp5,16592:up|Chst11,58250:down|Lpl,16956:up|Uck2,80914:down|Mt2,17750:up|Plpp3,67916:up|Pdxk,216134:up|Sord,20322:up|Adipoq,11450:up|Ptgs2,19225:down|Ampd3,11717:up|Ggt5,23887:up|Fabp4,11770:up|Akr1c18,105349:up
Insulin secretion	KEGG PATHWAY	mmu04911	1.221E-01	2.815E-01	Creb3l1,26427:down|Fxyd2,11936:up|Glp1r,14652:down
Phospholipase C-mediated cascade: FGFR1	Reactome	R-MMU-5654219	1.278E-01	2.887E-01	Fgfr1,14182:down|Prkar2b,19088:up
@@Vitamin B6 metabolism	KEGG PATHWAY	mmu00750	1.297E-01	2.887E-01	Pdxk,216134:up
Glycoprotein hormones	Reactome	R-MMU-209822	1.297E-01	2.887E-01	Inhba,16323:down
Downregulation of ERBB2:ERBB3 signaling	Reactome	R-MMU-1358803	1.297E-01	2.887E-01	Nrg1,211323:down
Dissolution of Fibrin Clot	Reactome	R-MMU-75205	1.297E-01	2.887E-01	Plat,18791:down
Reactions specific to the complex N-glycan synthesis pathway	Reactome	R-MMU-975578	1.297E-01	2.887E-01	Man2a2,140481:up
Prostanoid ligand receptors	Reactome	R-MMU-391908	1.297E-01	2.887E-01	Ptgir,19222:up
EGFR Transactivation by Gastrin	Reactome	R-MMU-2179392	1.297E-01	2.887E-01	Egfr,13649:down
Lipid digestion, mobilization, and transport	Reactome	R-MMU-73923	1.313E-01	2.903E-01	Fabp4,11770:up|Fabp5,16592:up|Lpl,16956:up
Visual phototransduction	Reactome	R-MMU-2187338	1.313E-01	2.903E-01	Akr1c18,105349:up|Lpl,16956:up|Gpc4,14735:down
@@Arachidonic acid metabolism	KEGG PATHWAY	mmu00590	1.344E-01	2.962E-01	Ptgs2,19225:down|Cbr2,12409:up|Ggt5,23887:up

Signaling by the B Cell Receptor (BCR)	Reactome	R-MMU-983705	1.356E-01	2.979E-01	Nrg1,211323:down|Kitl,17311:up|Fgfr2,14183:up|Fgfr1,14182:down|Egfr,13649:down
Phospholipase C-mediated cascade; FGFR2	Reactome	R-MMU-5654221	1.371E-01	2.985E-01	Fgfr2,14183:up|Prkar2b,19088:up
Ion transport by P-type ATPases	Reactome	R-MMU-936837	1.371E-01	2.985E-01	Fxyd2,11936:up|Fxyd1,56188:up
@@Signaling by FGFR2	Reactome	R-MMU-5654738	1.371E-01	2.985E-01	Prkar2b,19088:up|Ret,19713:up|Fgfr2,14183:up|Fgfr1,14182:down|Kitl,17311:up|Nrg1,211323:down|Egfr,13649:down
Antigen processing and presentation	KEGG PATHWAY	mmu04612	1.375E-01	2.985E-01	Tnf,21926:up|Cd4,12504:up|H2-T24,15042:up
IRS-mediated signalling	Reactome	R-MMU-112399	1.387E-01	2.993E-01	Ret,19713:up|Fgfr2,14183:up|Fgfr1,14182:down|Kitl,17311:up|Nrg1,211323:down|Egfr,13649:down
NGF signalling via TRKA from the plasma membrane	Reactome	R-MMU-187037	1.388E-01	2.993E-01	Prkar2b,19088:up|Ret,19713:up|Fgfr2,14183:up|Fgfr1,14182:down|Kitl,17311:up|Nrg1,211323:down|Egfr,13649:down
Calcitonin-like ligand receptors	Reactome	R-MMU-419812	1.417E-01	3.020E-01	Adm,11535:up
Dermatan sulfate biosynthesis	Reactome	R-MMU-2022923	1.417E-01	3.020E-01	Vcan,13003:down
Sperm Motility And Taxes	Reactome	R-MMU-1300642	1.417E-01	3.020E-01	Hvcn1,74096:up
Pyrimidine salvage reactions	Reactome	R-MMU-73614	1.417E-01	3.020E-01	Uck2,80914:down
TNFR2 non-canonical NF-kB pathway	Reactome	R-MMU-5668541	1.439E-01	3.039E-01	Tnfrsf12a,27279:down|Tnf,21926:up|Tnfrsf11b,18383:up
Transport of glucose and other sugars, bile salts and organic acids, metal ions and amine compounds	Reactome	R-MMU-425366	1.439E-01	3.039E-01	Slc40a1,53945:up|Emb,13723:up|Slc2a6,227659:up
Insulin receptor signalling cascade	Reactome	R-MMU-74751	1.461E-01	3.075E-01	Ret,19713:up|Fgfr2,14183:up|Fgfr1,14182:down|Kitl,17311:up|Nrg1,211323:down|Egfr,13649:down
MAPK signaling pathway	KEGG PATHWAY	mmu04010	1.480E-01	3.086E-01	Fgfr2,14183:up|Fgfr1,14182:down|Tnf,21926:up|Tgfb2,21808:down|Tgfb3,21809:down|Egfr,13649:down
Phospholipase D signaling pathway	KEGG PATHWAY	mmu04072	1.487E-01	3.086E-01	Kitl,17311:up|Pdgfc,54635:down|Plpp3,67916:up|Egfr,13649:down
Systemic lupus erythematosus	KEGG PATHWAY	mmu05322	1.513E-01	3.086E-01	Tnf,21926:up|C1s2,317677:up|C3,12266:up|C1s1,50908:up
Hepatitis B	KEGG PATHWAY	mmu05161	1.513E-01	3.086E-01	Creb3l1,26427:down|Tnf,21926:up|Tgfb2,21808:down|Tgfb3,21809:down
Cocaine addiction	KEGG PATHWAY	mmu05030	1.514E-01	3.086E-01	Creb3l1,26427:down|Maob,109731:up
Transcriptional Regulation of Adipocyte Differentiation in 3T3-L1 Pre-adipocytes	Reactome	R-MMU-442533	1.514E-01	3.086E-01	Tnf,21926:up|Fabp4,11770:up
Metabolism of carbohydrates	Reactome	R-MMU-71387	1.517E-01	3.086E-01	Sord,20322:up|Hpse,15442:up|Gpc4,14735:down|Chst11,58250:down|Vcan,13003:down|Prelp,116847:up
FGFR1c ligand binding and activation	Reactome	R-MMU-190373	1.536E-01	3.086E-01	Fgfr1,14182:down
CS/DS degradation	Reactome	R-MMU-2024101	1.536E-01	3.086E-01	Vcan,13003:down

Transport of gamma-carboxylated protein precursors from the endoplasmic reticulum to the Golgi apparatus	Reactome	R-MMU-159763	1.536E-01	3.086E-01	Gas6,14456:up
Keratan sulfate degradation	Reactome	R-MMU-2022857	1.536E-01	3.086E-01	Prelp,116847:up
Peptide hormone biosynthesis	Reactome	R-MMU-209952	1.536E-01	3.086E-01	Inhba,16323:down
Platelet Adhesion to exposed collagen	Reactome	R-MMU-75892	1.536E-01	3.086E-01	Col1a2,12843:down
Endocrine resistance	KEGG PATHWAY	mmu01522	1.536E-01	3.086E-01	Mmp2,17390:down|Igf1,16000:up|Egfr,13649:down
Amino sugar and nucleotide sugar metabolism	KEGG PATHWAY	mmu00520	1.562E-01	3.129E-01	Gfpt2,14584:down|Npl,74091:up
Estrogen signaling pathway	KEGG PATHWAY	mmu04915	1.602E-01	3.198E-01	Creb3l1,26427:down|Mmp2,17390:down|Egfr,13649:down
Type II diabetes mellitus	KEGG PATHWAY	mmu04930	1.611E-01	3.198E-01	Tnf,21926:up|Adipoq,11450:up
Nuclear Receptor transcription pathway	Reactome	R-MMU-383280	1.611E-01	3.198E-01	Vdr,22337:down|Pparg,19016:up
Integration of energy metabolism	Reactome	R-MMU-163685	1.636E-01	3.200E-01	Adipoq,11450:up|Glp1r,14652:down|Prkar2b,19088:up
TP53 Regulates Transcription of Cell Death Genes	Reactome	R-MMU-5633008	1.653E-01	3.200E-01	Igfbp3,16009:up
Translocation of ZAP-70 to Immunological synapse	Reactome	R-MMU-202430	1.653E-01	3.200E-01	Cd4,12504:up
eNOS activation	Reactome	R-MMU-203615	1.653E-01	3.200E-01	Ddah1,69219:down
Facilitative Na+-independent glucose transporters	Reactome	R-MMU-428790	1.653E-01	3.200E-01	Slc2a6,227659:up
Metabolism of Angiotensinogen to Angiotensins	Reactome	R-MMU-2022377	1.653E-01	3.200E-01	Mme,17380:up
Removal of aminoterminal propeptides from gamma-carboxylated proteins	Reactome	R-MMU-159782	1.653E-01	3.200E-01	Gas6,14456:up
GRB2 events in ERBB2 signaling	Reactome	R-MMU-1963640	1.653E-01	3.200E-01	Nrg1,211323:down
Formation of Senescence-Associated Heterochromatin Foci (SAHF)	Reactome	R-MMU-2559584	1.653E-01	3.200E-01	Hmga1-rs1,111241:up
Ion homeostasis	Reactome	R-MMU-5578775	1.659E-01	3.204E-01	Fxyd2,11936:up|Fxyd1,56188:up
Class B/2 (Secretin family receptors)	Reactome	R-MMU-373080	1.708E-01	3.290E-01	Glp1r,14652:down|Adm,11535:up
Choline metabolism in cancer	KEGG PATHWAY	mmu05231	1.737E-01	3.331E-01	Pdgfc,54635:down|Plpp3,67916:up|Egfr,13649:down
Nuclear signaling by ERBB4	Reactome	R-MMU-1251985	1.758E-01	3.331E-01	Prl2c2,18811:up|Cxcl12,20315:up

Folate biosynthesis	KEGG PATHWAY	mmu00790	1.768E-01	3.331E-01	Alpl,11647:up
Nicotinate metabolism	Reactome	R-MMU-196807	1.768E-01	3.331E-01	Ptgs2,19225:down
FGFR2c ligand binding and activation	Reactome	R-MMU-190375	1.768E-01	3.331E-01	Fgfr2,14183:up
LGI-ADAM interactions	Reactome	R-MMU-5682910	1.768E-01	3.331E-01	Lgi2,246316:up
Gamma-carboxylation, transport, and amino-terminal cleavage of proteins	Reactome	R-MMU-159854	1.768E-01	3.331E-01	Gas6,14456:up
Starch and sucrose metabolism	KEGG PATHWAY	mmu00500	1.807E-01	3.396E-01	Enpp3,209558:down|Enpp1,18605:down
Basal cell carcinoma	KEGG PATHWAY	mmu05217	1.857E-01	3.453E-01	Bmp4,12159:up|Wnt5a,22418:down
Signaling by SCF-KIT	Reactome	R-MMU-1433557	1.875E-01	3.453E-01	Ret,19713:up|Fgfr2,14183:up|Fgfr1,14182:down|Kitl,17311:up|Nrg1,211323:down|Egfr,13649:down
Downregulation of TGF-beta receptor signaling	Reactome	R-MMU-2173788	1.881E-01	3.453E-01	Pmepa1,65112:down
Eicosanoid ligand-binding receptors	Reactome	R-MMU-391903	1.881E-01	3.453E-01	Ptgir,19222:up
Regulation of FZD by ubiquitination	Reactome	R-MMU-4641263	1.881E-01	3.453E-01	Rspo2,239405:up
Phosphorylation of CD3 and TCR zeta chains	Reactome	R-MMU-202427	1.881E-01	3.453E-01	Cd4,12504:up
Purine salvage	Reactome	R-MMU-74217	1.881E-01	3.453E-01	Ampd3,11717:up
Synthesis of Leukotrienes (LT) and Eoxins (EX)	Reactome	R-MMU-2142691	1.881E-01	3.453E-01	Ggt5,23887:up
Miscellaneous transport and binding events	Reactome	R-MMU-5223345	1.881E-01	3.453E-01	Ank,11732:down
Signaling by PTK6	Reactome	R-MMU-8848021	1.907E-01	3.490E-01	Nrg1,211323:down|Egfr,13649:down
Metabolic pathways	KEGG PATHWAY	mmu01100	1.923E-01	3.510E-01	Maob,109731:up|P4ha1,18451:down|Gatm,67092:down|Enpp1,18605:down|Hal,15109:up|Cdo1,12583:up|Aldh1a3,56847:down|Cbr2,12409:up|Man2a2,140481:up|Alpl,11647:up|Hpse,15442:up|Aldh1a2,19378:up|Uck2,80914:down|Gfpt2,14584:down|Plpp3,67916:up|Pdxk,216134:up|Sord,20322:up|Enpp3,209558:down|Anpep,16790:up|Ptgs2,19225:down|Ampd3,11717:up|Ggt5,23887:up
Signaling by Insulin receptor	Reactome	R-MMU-74752	1.937E-01	3.528E-01	Ret,19713:up|Fgfr2,14183:up|Fgfr1,14182:down|Kitl,17311:up|Nrg1,211323:down|Egfr,13649:down
Regulation of lipolysis in adipocytes	KEGG PATHWAY	mmu04923	1.957E-01	3.553E-01	Fabp4,11770:up|Ptgs2,19225:down
Regulation of KIT signaling	Reactome	R-MMU-1433559	1.993E-01	3.556E-01	Kitl,17311:up
Syndecan interactions	Reactome	R-MMU-3000170	1.993E-01	3.556E-01	Tnc,21923:down
WNT5A-dependent internalization of FZD4	Reactome	R-MMU-5099900	1.993E-01	3.556E-01	Wnt5a,22418:down
PKA activation	Reactome	R-MMU-163615	1.993E-01	3.556E-01	Prkar2b,19088:up
FGFR1 ligand binding and activation	Reactome	R-MMU-190242	1.993E-01	3.556E-01	Fgfr1,14182:down

PKA-mediated phosphorylation of CREB	Reactome	R-MMU-111931	1.993E-01	3.556E-01	Prkar2b,19088:up
Legionellosis	KEGG PATHWAY	mmu05134	2.007E-01	3.562E-01	Tnf,21926:up|C3,12266:up
Ovarian steroidogenesis	KEGG PATHWAY	mmu04913	2.007E-01	3.562E-01	Ptgs2,19225:down|Igf1,16000:up
Glycerolipid metabolism	KEGG PATHWAY	mmu00561	2.057E-01	3.623E-01	Plpp3,67916:up|Lpl,16956:up
Synthesis of substrates in N-glycan biosythesis	Reactome	R-MMU-446219	2.057E-01	3.623E-01	Gfpt2,14584:down|Npl,74091:up
Insulin resistance	KEGG PATHWAY	mmu04931	2.086E-01	3.639E-01	Creb3l1,26427:down|Gfpt2,14584:down|Tnf,21926:up
G alpha (s) signalling events	Reactome	R-MMU-418555	2.086E-01	3.639E-01	Glp1r,14652:down|Ptgir,19222:up|Adm,11535:up
Scavenging of heme from plasma	Reactome	R-MMU-2168880	2.104E-01	3.639E-01	Hp,15439:up
PD-1 signaling	Reactome	R-MMU-389948	2.104E-01	3.639E-01	Cd4,12504:up
Oxygen-dependent proline hydroxylation of Hypoxia-inducible Factor Alpha	Reactome	R-MMU-1234176	2.104E-01	3.639E-01	Epas1,13819:up
PKA activation in glucagon signalling	Reactome	R-MMU-164378	2.104E-01	3.639E-01	Prkar2b,19088:up
CRMPs in Sema3A signaling	Reactome	R-MMU-399956	2.104E-01	3.639E-01	Dpysl3,22240:down
VEGF signaling pathway	KEGG PATHWAY	mmu04370	2.108E-01	3.639E-01	Ptgs2,19225:down|Vegfa,22339:down
Cell adhesion molecules (CAMs)	KEGG PATHWAY	mmu04514	2.143E-01	3.690E-01	Ncam1,17967:down|Cd4,12504:up|Vcan,13003:down|H2-T24,15042:up
Allograft rejection	KEGG PATHWAY	mmu05330	2.209E-01	3.747E-01	Tnf,21926:up|H2-T24,15042:up
TP53 regulates transcription of additional cell cycle genes whose exact role in the p53 pathway remain uncertain	Reactome	R-MMU-6804115	2.213E-01	3.747E-01	Plk2,20620:down
TNF receptor superfamily (TNFSF) members mediating non-canonical NF-kB pathway	Reactome	R-MMU-5676594	2.213E-01	3.747E-01	Tnfrsf12a,27279:down
Synthesis of bile acids and bile salts via 24-hydroxycholesterol	Reactome	R-MMU-193775	2.213E-01	3.747E-01	Akr1c18,105349:up
Synthesis of bile acids and bile salts via 27-hydroxycholesterol	Reactome	R-MMU-193807	2.213E-01	3.747E-01	Akr1c18,105349:up
Colorectal cancer	KEGG PATHWAY	mmu05210	2.311E-01	3.846E-01	Tgfb2,21808:down|Tgfb3,21809:down
Graft-versus-host disease	KEGG PATHWAY	mmu05332	2.311E-01	3.846E-01	Tnf,21926:up|H2-T24,15042:up
FGFR2 ligand binding and activation	Reactome	R-MMU-190241	2.321E-01	3.846E-01	Fgfr2,14183:up

Synthesis, secretion, and deacylation of Ghrelin	Reactome	R-MMU-422085	2.321E-01	3.846E-01	Igf1,16000:up
DARPP-32 events	Reactome	R-MMU-180024	2.321E-01	3.846E-01	Prkar2b,19088:up
Tie2 Signaling	Reactome	R-MMU-210993	2.321E-01	3.846E-01	Dok2,13449:up
Glioma	KEGG PATHWAY	mmu05214	2.362E-01	3.898E-01	Igf1,16000:up|Egfr,13649:down
Metabolism of proteins	Reactome	R-MMU-392499	2.363E-01	3.898E-01	Sec23a,20334:down|Inhba,16323:down|Pappa,18491:down|Igfbp4,16010:up|Man2a2,140481:up|Lmcd1,30937:up|Npl,74091:up|Igf2,16002:up|Gas6,14456:up|Igf1,16000:up|Uchl5,56207:up|Mmp2,17390:down|Klk1b11,16613:up|Gfpt2,14584:down|Mme,17380:up|Igfbp3,16009:up
Regulation of TLR by endogenous ligand	Reactome	R-MMU-5686938	2.427E-01	3.984E-01	S100a1,20193:up
Amphetamine addiction	KEGG PATHWAY	mmu05031	2.464E-01	4.026E-01	Creb3l1,26427:down|Maob,109731:up
RNA polymerase II transcribes snRNA genes	Reactome	R-MMU-6807505	2.464E-01	4.026E-01	Ice1,218333:down|Nabp1,109019:down
Calcium signaling pathway	KEGG PATHWAY	mmu04020	2.496E-01	4.060E-01	Ednrb,13618:up|Htr2b,15559:down|Htr2a,15558:down|Egfr,13649:down
Fc epsilon receptor (FCERI) signaling	Reactome	R-MMU-2454202	2.510E-01	4.060E-01	Ret,19713:up|Fgfr2,14183:up|Fgfr1,14182:down|Kitl,17311:up|Nrg1,211323:down|Egfr,13649:down
p53 signaling pathway	KEGG PATHWAY	mmu04115	2.515E-01	4.060E-01	Igf1,16000:up|Igfbp3,16009:up
Platelet activation	KEGG PATHWAY	mmu04611	2.522E-01	4.060E-01	Col3a1,12825:down|Ptgir,19222:up|Col1a2,12843:down
Glycosaminoglycan biosynthesis - chondroitin sulfate / dermatan sulfate	KEGG PATHWAY	mmu00532	2.531E-01	4.060E-01	Chst11,58250:down
EGFR downregulation	Reactome	R-MMU-182971	2.531E-01	4.060E-01	Egfr,13649:down
SHC-mediated cascade:FGFR1	Reactome	R-MMU-5654688	2.531E-01	4.060E-01	Fgfr1,14182:down
Type I diabetes mellitus	KEGG PATHWAY	mmu04940	2.566E-01	4.098E-01	Tnf,21926:up|H2-T24,15042:up
Biosynthesis of the N-glycan precursor (dolichol lipid-linked oligosaccharide, LLO) and transfer to a nascent protein	Reactome	R-MMU-446193	2.566E-01	4.098E-01	Gfpt2,14584:down|Npl,74091:up
Ion channel transport	Reactome	R-MMU-983712	2.616E-01	4.122E-01	Fxyd2,11936:up|Mcoln3,171166:up|Mcoln2,68279:up|Fxyd1,56188:up
Thyroid hormone synthesis	KEGG PATHWAY	mmu04918	2.617E-01	4.122E-01	Creb3l1,26427:down|Fxyd2,11936:up
Inflammatory mediator regulation of TRP channels	KEGG PATHWAY	mmu04750	2.634E-01	4.122E-01	Igf1,16000:up|Htr2b,15559:down|Htr2a,15558:down
Glycosaminoglycan degradation	KEGG PATHWAY	mmu00531	2.634E-01	4.122E-01	Hpse,15442:up
Sulfur amino acid metabolism	Reactome	R-MMU-1614635	2.634E-01	4.122E-01	Cdo1,12583:up
Regulation of Hypoxia-inducible Factor (HIF) by oxygen	Reactome	R-MMU-1234174	2.634E-01	4.122E-01	Epas1,13819:up

IL-6-type cytokine receptor ligand interactions	Reactome	R-MMU-6788467	2.634E-01	4.122E-01	Crlf1,12931:down
Cellular response to hypoxia	Reactome	R-MMU-2262749	2.634E-01	4.122E-01	Epas1,13819:up
TGF-beta receptor signaling activates SMADs	Reactome	R-MMU-2173789	2.634E-01	4.122E-01	Pmepa1,65112:down
Adipocytokine signaling pathway	KEGG PATHWAY	mmu04920	2.720E-01	4.197E-01	Tnf,21926:up|Adipoq,11450:up
Proximal tubule bicarbonate reclamation	KEGG PATHWAY	mmu04964	2.736E-01	4.197E-01	Fxyd2,11936:up
Chylomicron-mediated lipid transport	Reactome	R-MMU-174800	2.736E-01	4.197E-01	Lpl,16956:up
Surfactant metabolism	Reactome	R-MMU-5683826	2.736E-01	4.197E-01	Lmcd1,30937:up
eNOS activation and regulation	Reactome	R-MMU-203765	2.736E-01	4.197E-01	Ddah1,69219:down
TNFs bind their physiological receptors	Reactome	R-MMU-5669034	2.736E-01	4.197E-01	Tnfrsf11b,18383:up
Metabolism of nitric oxide	Reactome	R-MMU-202131	2.736E-01	4.197E-01	Ddah1,69219:down
Common Pathway of Fibrin Clot Formation	Reactome	R-MMU-140875	2.736E-01	4.197E-01	Serpine2,20720:up
Chronic myeloid leukemia	KEGG PATHWAY	mmu05220	2.771E-01	4.241E-01	Tgfb2,21808:down|Tgfb3,21809:down
Adherens junction	KEGG PATHWAY	mmu04520	2.822E-01	4.257E-01	Fgfr1,14182:down|Egfr,13649:down
EPH-Ephrin signaling	Reactome	R-MMU-2682334	2.822E-01	4.257E-01	Mmp2,17390:down|Vegfa,22339:down
Fertilization	Reactome	R-MMU-1187000	2.836E-01	4.257E-01	Hvcn1,74096:up
Regulation of Complement cascade	Reactome	R-MMU-977606	2.836E-01	4.257E-01	C3,12266:up
Reproduction	Reactome	R-MMU-1474165	2.836E-01	4.257E-01	Hvcn1,74096:up
TNFR1-induced NFkappaB signaling pathway	Reactome	R-MMU-5357956	2.836E-01	4.257E-01	Tnf,21926:up
Metal ion SLC transporters	Reactome	R-MMU-425410	2.836E-01	4.257E-01	Slc40a1,53945:up
Signaling by BMP	Reactome	R-MMU-201451	2.836E-01	4.257E-01	Bmpr1a,12166:down
@@Pyrimidine metabolism	Reactome	R-MMU-73848	2.935E-01	4.359E-01	Uck2,80914:down
Asthma	KEGG PATHWAY	mmu05310	2.935E-01	4.359E-01	Tnf,21926:up
Myogenesis	Reactome	R-MMU-525793	2.935E-01	4.359E-01	Cdo1,12583:up
CDO in myogenesis	Reactome	R-MMU-375170	2.935E-01	4.359E-01	Cdo1,12583:up
PI3K Cascade	Reactome	R-MMU-109704	2.975E-01	4.409E-01	Fgfr2,14183:up|Fgfr1,14182:down
Signalling by NGF	Reactome	R-MMU-166520	2.982E-01	4.410E-01	Prkar2b,19088:up|Ret,19713:up|Fgfr2,14183:up|Fgfr1,14182:down|Kitl,17311:up|Nrg1,211323:down|Egfr,13649:down
Salmonella infection	KEGG PATHWAY	mmu05132	3.026E-01	4.438E-01	Ccl3,20302:up|Ccl4,20303:up
N-glycan antennae elongation in the medial/trans-Golgi	Reactome	R-MMU-975576	3.033E-01	4.438E-01	Man2a2,140481:up

HS-GAG biosynthesis	Reactome	R-MMU-2022928	3.033E-01	4.438E-01	Gpc4,14735:down
SHC-mediated cascade:FGFR2	Reactome	R-MMU-5654699	3.033E-01	4.438E-01	Fgfr2,14183:up
Keratan sulfate biosynthesis	Reactome	R-MMU-2022854	3.033E-01	4.438E-01	Prelp,116847:up
cAMP signaling pathway	KEGG PATHWAY	mmu04024	3.046E-01	4.448E-01	Creb3l1,26427:down|Fxyd2,11936:up|Glp1r,14652:down|Fxyd1,56188:up
Dorso-ventral axis formation	KEGG PATHWAY	mmu04320	3.129E-01	4.467E-01	Egfr,13649:down
G protein gated Potassium channels	Reactome	R-MMU-1296059	3.129E-01	4.467E-01	Kcnj15,16516:down
Inhibition of voltage gated Ca2+ channels via Gbeta/gamma subunits	Reactome	R-MMU-997272	3.129E-01	4.467E-01	Kcnj15,16516:down
Synthesis of bile acids and bile salts via 7alpha-hydroxycholesterol	Reactome	R-MMU-193368	3.129E-01	4.467E-01	Akr1c18,105349:up
Generation of second messenger molecules	Reactome	R-MMU-202433	3.129E-01	4.467E-01	Cd4,12504:up
Regulation of TNFR1 signaling	Reactome	R-MMU-5357905	3.129E-01	4.467E-01	Tnf,21926:up
Calmodulin induced events	Reactome	R-MMU-111933	3.129E-01	4.467E-01	Prkar2b,19088:up
WNT ligand biogenesis and trafficking	Reactome	R-MMU-3238698	3.129E-01	4.467E-01	Wnt5a,22418:down
CaM pathway	Reactome	R-MMU-111997	3.129E-01	4.467E-01	Prkar2b,19088:up
Laminin interactions	Reactome	R-MMU-3000157	3.129E-01	4.467E-01	Lama2,16773:down
Activation of G protein gated Potassium channels	Reactome	R-MMU-1296041	3.129E-01	4.467E-01	Kcnj15,16516:down
DAG and IP3 signaling	Reactome	R-MMU-1489509	3.224E-01	4.575E-01	Prkar2b,19088:up
PLC-gamma1 signalling	Reactome	R-MMU-167021	3.224E-01	4.575E-01	Prkar2b,19088:up
EPHA-mediated growth cone collapse	Reactome	R-MMU-3928663	3.224E-01	4.575E-01	Vegfa,22339:down
Vesicle-mediated transport	Reactome	R-MMU-5653656	3.236E-01	4.583E-01	Kdelr3,105785:down|Sec23a,20334:down|Cd4,12504:up|Trf,22041:up|Wnt5a,22418:down|Tfrc,22042:up|Egfr,13649:down|Hp,15439:up
Interleukin-6 family signaling	Reactome	R-MMU-6783589	3.317E-01	4.660E-01	Crlf1,12931:down
Cargo concentration in the ER	Reactome	R-MMU-5694530	3.317E-01	4.660E-01	Sec23a,20334:down
Ca-dependent events	Reactome	R-MMU-111996	3.317E-01	4.660E-01	Prkar2b,19088:up
Endosomal/Vacuolar pathway	Reactome	R-MMU-1236977	3.317E-01	4.660E-01	H2-T24,15042:up
Small cell lung cancer	KEGG PATHWAY	mmu05222	3.381E-01	4.740E-01	Ptgs2,19225:down|Lama2,16773:down
Ca2+ pathway	Reactome	R-MMU-4086398	3.410E-01	4.771E-01	Wnt5a,22418:down
Viral myocarditis	KEGG PATHWAY	mmu05416	3.431E-01	4.791E-01	H2-T24,15042:up|Lama2,16773:down
ErbB signaling pathway	KEGG PATHWAY	mmu04012	3.481E-01	4.840E-01	Nrg1,211323:down|Egfr,13649:down

Synthesis of bile acids and bile salts	Reactome	R-MMU-192105	3.501E-01	4.840E-01	Akr1c18,105349:up
Negative regulation of FGFR1 signaling	Reactome	R-MMU-5654726	3.501E-01	4.840E-01	Fgfr1,14182:down
Keratan sulfate/keratin metabolism	Reactome	R-MMU-1638074	3.501E-01	4.840E-01	Prelp,116847:up
GnRH signaling pathway	KEGG PATHWAY	mmu04912	3.531E-01	4.872E-01	Mmp2,17390:down|Egfr,13649:down
Glucagon-type ligand receptors	Reactome	R-MMU-420092	3.590E-01	4.926E-01	Glp1r,14652:down
Sialic acid metabolism	Reactome	R-MMU-4085001	3.590E-01	4.926E-01	Npl,74091:up
TP53 Regulates Transcription of Cell Cycle Genes	Reactome	R-MMU-6791312	3.590E-01	4.926E-01	Plk2,20620:down
Inwardly rectifying K+ channels	Reactome	R-MMU-1296065	3.679E-01	5.017E-01	Kcnj15,16516:down
Gamma carboxylation, hypusine formation and arylsulfatase activation	Reactome	R-MMU-163841	3.679E-01	5.017E-01	Gas6,14456:up
Negative regulation of FGFR2 signaling	Reactome	R-MMU-5654727	3.679E-01	5.017E-01	Fgfr2,14183:up
HTLV-I infection	KEGG PATHWAY	mmu05166	3.686E-01	5.017E-01	Tgfb2,21808:down|Tnf,21926:up|H2-T24,15042:up|Wnt5a,22418:down|Tgfb3,21809:down
Neuroactive ligand-receptor interaction	KEGG PATHWAY	mmu04080	3.713E-01	5.044E-01	Ednrb,13618:up|Glp1r,14652:down|Htr2b,15559:down|Htr2a,15558:down|Ptgir,19222:up
mTOR signaling pathway	KEGG PATHWAY	mmu04150	3.723E-01	5.047E-01	Tnf,21926:up|Igf1,16000:up|Wnt5a,22418:down
Metabolism of water-soluble vitamins and cofactors	Reactome	R-MMU-196849	3.730E-01	5.047E-01	Ptgs2,19225:down|Pdxk,216134:up
beta-Alanine metabolism	KEGG PATHWAY	mmu00410	3.766E-01	5.068E-01	Aldh1a3,56847:down
Glucagon signaling in metabolic regulation	Reactome	R-MMU-163359	3.766E-01	5.068E-01	Prkar2b,19088:up
Metabolism of nucleotides	Reactome	R-MMU-15869	3.828E-01	5.140E-01	Ampd3,11717:up|Uck2,80914:down
Prion diseases	KEGG PATHWAY	mmu05020	3.852E-01	5.154E-01	Ncam1,17967:down
Binding and Uptake of Ligands by Scavenger Receptors	Reactome	R-MMU-2173782	3.852E-01	5.154E-01	Hp,15439:up
Cell-Cell communication	Reactome	R-MMU-1500931	3.877E-01	5.176E-01	Sirpb1a,320832:down|Sirpb1b,668101:down
@@Purine metabolism	Reactome	R-MMU-73847	3.937E-01	5.198E-01	Ampd3,11717:up
Primary immunodeficiency	KEGG PATHWAY	mmu05340	3.937E-01	5.198E-01	Cd4,12504:up
Fructose and mannose metabolism	KEGG PATHWAY	mmu00051	3.937E-01	5.198E-01	Sord,20322:up
African trypanosomiasis	KEGG PATHWAY	mmu05143	3.937E-01	5.198E-01	Tnf,21926:up
ER-Phagosome pathway	Reactome	R-MMU-1236974	3.937E-01	5.198E-01	H2-T24,15042:up

Prolactin receptor signaling	Reactome	R-MMU-1170546	3.937E-01	5.198E-01	Prl2c2,18811:up
Membrane Trafficking	Reactome	R-MMU-199991	3.961E-01	5.219E-01	Kdelr3,105785:down|Sec23a,20334:down|Cd4,12504:up|Trf,22041:up|Wnt5a,22418:down|Tfrc,22042:up|Egfr,13649:down
Stimuli-sensing channels	Reactome	R-MMU-2672351	3.974E-01	5.227E-01	Mcoln3,171166:up|Mcoln2,68279:up
Pentose and glucuronate interconversions	KEGG PATHWAY	mmu00040	4.021E-01	5.251E-01	Sord,20322:up
Glucagon-like Peptide-1 (GLP1) regulates insulin secretion	Reactome	R-MMU-381676	4.021E-01	5.251E-01	Glp1r,14652:down
TNF signaling	Reactome	R-MMU-75893	4.021E-01	5.251E-01	Tnf,21926:up
Cardiac conduction	Reactome	R-MMU-5576891	4.023E-01	5.251E-01	Fxyd2,11936:up|Fxyd1,56188:up
Alanine, aspartate and glutamate metabolism	KEGG PATHWAY	mmu00250	4.104E-01	5.317E-01	Gfpt2,14584:down
Transport of vitamins, nucleosides, and related molecules	Reactome	R-MMU-425397	4.104E-01	5.317E-01	Slc29a1,63959:up
EPH-ephrin mediated repulsion of cells	Reactome	R-MMU-3928665	4.185E-01	5.383E-01	Mmp2,17390:down
Interleukin-1 signaling	Reactome	R-MMU-446652	4.185E-01	5.383E-01	Il1rn,16181:up
Histidine, lysine, phenylalanine, tyrosine, proline and tryptophan catabolism	Reactome	R-MMU-6788656	4.266E-01	5.466E-01	Hal,15109:up
Alzheimer's disease	KEGG PATHWAY	mmu05010	4.315E-01	5.488E-01	Tnf,21926:up|Mme,17380:up|Lpl,16956:up
cGMP-PKG signaling pathway	KEGG PATHWAY	mmu04022	4.315E-01	5.488E-01	Creb3l1,26427:down|Fxyd2,11936:up|Ednrb,13618:up
Fat digestion and absorption	KEGG PATHWAY	mmu04975	4.345E-01	5.488E-01	Plpp3,67916:up
PLC beta mediated events	Reactome	R-MMU-112043	4.345E-01	5.488E-01	Prkar2b,19088:up
Phospholipase C-mediated cascade; FGFR3	Reactome	R-MMU-5654227	4.345E-01	5.488E-01	Prkar2b,19088:up
GABA B receptor activation	Reactome	R-MMU-977444	4.345E-01	5.488E-01	Kcnj15,16516:down
Activation of GABAB receptors	Reactome	R-MMU-991365	4.345E-01	5.488E-01	Kcnj15,16516:down
T cell receptor signaling pathway	KEGG PATHWAY	mmu04660	4.356E-01	5.492E-01	Tnf,21926:up|Cd4,12504:up
Bile acid and bile salt metabolism	Reactome	R-MMU-194068	4.423E-01	5.527E-01	Akr1c18,105349:up
G-protein mediated events	Reactome	R-MMU-112040	4.423E-01	5.527E-01	Prkar2b,19088:up
Phospholipase C-mediated cascade; FGFR4	Reactome	R-MMU-5654228	4.423E-01	5.527E-01	Prkar2b,19088:up
Death Receptor Signalling	Reactome	R-MMU-73887	4.423E-01	5.527E-01	Tnf,21926:up
Asparagine N-linked glycosylation	Reactome	R-MMU-446203	4.443E-01	5.542E-01	Gfpt2,14584:down|Npl,74091:up|Man2a2,140481:up|Sec23a,20334:down

GPCR downstream signaling	Reactome	R-MMU-388396	4.493E-01	5.573E-01	Ednrb,13618:up|Glp1r,14652:down|Ccl9,20308:up|Cxcl12,20315:up|Ccl6,20305:up|Kng1,16644:up|Kng2,385643:up|Adm,11535:up|Cxcr6,80901:down|Cxcl9,17329:up|Ppbp,57349:down|Ptgir,19222:up|C3,12266:up|Htr2b,15559:down|Htr2a,15558:down
@@Axon guidance	KEGG PATHWAY	mmu04360	4.496E-01	5.573E-01	Sema7a,20361:down|Cxcl12,20315:up|Wnt5a,22418:down
Intestinal immune network for IgA production	KEGG PATHWAY	mmu04672	4.500E-01	5.573E-01	Cxcl12,20315:up
Vasopressin regulates renal water homeostasis via Aquaporins	Reactome	R-MMU-432040	4.500E-01	5.573E-01	Prkar2b,19088:up
@@Purine metabolism	KEGG PATHWAY	mmu00230	4.532E-01	5.603E-01	Ampd3,11717:up|Enpp3,209558:down|Enpp1,18605:down
Vasopressin-regulated water reabsorption	KEGG PATHWAY	mmu04962	4.576E-01	5.638E-01	Creb3l1,26427:down
Sphingolipid de novo biosynthesis	Reactome	R-MMU-1660661	4.576E-01	5.638E-01	Plpp3,67916:up
SLC-mediated transmembrane transport	Reactome	R-MMU-425407	4.625E-01	5.688E-01	Slc40a1,53945:up|Emb,13723:up|Slc29a1,63959:up|Slc2a6,227659:up
Ether lipid metabolism	KEGG PATHWAY	mmu00565	4.651E-01	5.690E-01	Plpp3,67916:up
DNA Damage/Telomere Stress Induced Senescence	Reactome	R-MMU-2559586	4.651E-01	5.690E-01	Hmga1-rs1,111241:up
Growth hormone receptor signaling	Reactome	R-MMU-982772	4.651E-01	5.690E-01	Prl2c2,18811:up
Carbohydrate digestion and absorption	KEGG PATHWAY	mmu04973	4.725E-01	5.770E-01	Fxyd2,11936:up
Tryptophan metabolism	KEGG PATHWAY	mmu00380	4.798E-01	5.849E-01	Maob,109731:up
Thyroid hormone signaling pathway	KEGG PATHWAY	mmu04919	4.903E-01	5.967E-01	Fxyd2,11936:up|Bmp4,12159:up
Sphingolipid metabolism	KEGG PATHWAY	mmu00600	4.941E-01	5.971E-01	Plpp3,67916:up
Cysteine and methionine metabolism	KEGG PATHWAY	mmu00270	4.941E-01	5.971E-01	Cdo1,12583:up
GPVI-mediated activation cascade	Reactome	R-MMU-114604	4.941E-01	5.971E-01	Col1a2,12843:down
Aquaporin-mediated transport	Reactome	R-MMU-445717	4.941E-01	5.971E-01	Prkar2b,19088:up
N-Glycan biosynthesis	KEGG PATHWAY	mmu00510	5.010E-01	6.045E-01	Man2a2,140481:up
Leukocyte transendothelial migration	KEGG PATHWAY	mmu04670	5.078E-01	6.108E-01	Mmp2,17390:down|Cxcl12,20315:up
Golgi Associated Vesicle Biogenesis	Reactome	R-MMU-432722	5.079E-01	6.108E-01	Tfrc,22042:up
Metabolism of lipids and lipoproteins	Reactome	R-MMU-556833	5.124E-01	6.147E-01	Hpgd,15446:up|Ggt5,23887:up|Fabp5,16592:up|Ptgs2,19225:down|Lpl,16956:up|Fabp4,11770:up|Plpp3,67916:up|Gdpd1,66569:up|Akr1c18,105349:up
Drug metabolism - other enzymes	KEGG PATHWAY	mmu00983	5.147E-01	6.147E-01	Uck2,80914:down
COPI-dependent Golgi-to-ER retrograde traffic	Reactome	R-MMU-6811434	5.147E-01	6.147E-01	Kdelr3,105785:down

Cell cycle	KEGG PATHWAY	mmu04110	5.207E-01	6.185E-01	Tgfb2,21808:down|Tgfb3,21809:down
Endometrial cancer	KEGG PATHWAY	mmu05213	5.215E-01	6.185E-01	Egfr,13649:down
Amyotrophic lateral sclerosis (ALS)	KEGG PATHWAY	mmu05014	5.215E-01	6.185E-01	Tnf,21926:up
Lysine degradation	KEGG PATHWAY	mmu00310	5.215E-01	6.185E-01	Plod2,26432:down
Platelet homeostasis	Reactome	R-MMU-418346	5.281E-01	6.253E-01	Ptgir,19222:up
Alcoholism	KEGG PATHWAY	mmu05034	5.327E-01	6.298E-01	Creb3l1,26427:down|Slc29a1,63959:up|Maob,109731:up
Non-small cell lung cancer	KEGG PATHWAY	mmu05223	5.474E-01	6.427E-01	Egfr,13649:down
Lipoprotein metabolism	Reactome	R-MMU-174824	5.474E-01	6.427E-01	Lpl,16956:up
GABA receptor activation	Reactome	R-MMU-977443	5.474E-01	6.427E-01	Kcnj15,16516:down
Asymmetric localization of PCP proteins	Reactome	R-MMU-4608870	5.474E-01	6.427E-01	Wnt5a,22418:down
COPII (Coat Protein 2) Mediated Vesicle Transport	Reactome	R-MMU-204005	5.536E-01	6.479E-01	Sec23a,20334:down
PI Metabolism	Reactome	R-MMU-1483255	5.536E-01	6.479E-01	Gdpd1,66569:up
Dopaminergic synapse	KEGG PATHWAY	mmu04728	5.579E-01	6.518E-01	Creb3l1,26427:down|Maob,109731:up
Opioid Signalling	Reactome	R-MMU-111885	5.598E-01	6.519E-01	Prkar2b,19088:up
Costimulation by the CD28 family	Reactome	R-MMU-388841	5.598E-01	6.519E-01	Cd4,12504:up
Signaling by TGF-beta Receptor Complex	Reactome	R-MMU-170834	5.719E-01	6.637E-01	Pmepa1,65112:down
Measles	KEGG PATHWAY	mmu05162	5.737E-01	6.648E-01	Oas1a,246730:up|Oas3,246727:up
Cytosolic DNA-sensing pathway	KEGG PATHWAY	mmu04623	5.778E-01	6.662E-01	Ccl4,20303:up
Long-term depression	KEGG PATHWAY	mmu04730	5.778E-01	6.662E-01	Igf1,16000:up
Metabolism of amino acids and derivatives	Reactome	R-MMU-71291	5.841E-01	6.723E-01	Cdo1,12583:up|Gatm,67092:down|Hal,15109:up
Transport to the Golgi and subsequent modification	Reactome	R-MMU-948021	5.892E-01	6.771E-01	Sec23a,20334:down|Man2a2,140481:up
Longevity regulating pathway - multiple species	KEGG PATHWAY	mmu04213	5.951E-01	6.827E-01	Igf1,16000:up
Glycolysis / Gluconeogenesis	KEGG PATHWAY	mmu00010	6.007E-01	6.858E-01	Aldh1a3,56847:down
Loss of proteins required for interphase microtubule organizationÂ from the centrosome	Reactome	R-MMU-380284	6.007E-01	6.858E-01	Prkar2b,19088:up
Loss of Nlp from mitotic centrosomes	Reactome	R-MMU-380259	6.007E-01	6.858E-01	Prkar2b,19088:up
trans-Golgi Network Vesicle Budding	Reactome	R-MMU-199992	6.062E-01	6.899E-01	Tfrc,22042:up

Clathrin derived vesicle budding	Reactome	R-MMU-421837	6.062E-01	6.899E-01	Tfrc,22042:up
Fc epsilon RI signaling pathway	KEGG PATHWAY	mmu04664	6.117E-01	6.949E-01	Tnf,21926:up
RIG-I-like receptor signaling pathway	KEGG PATHWAY	mmu04622	6.170E-01	6.988E-01	Tnf,21926:up
AURKA Activation by TPX2	Reactome	R-MMU-8854518	6.170E-01	6.988E-01	Prkar2b,19088:up
Adrenergic signaling in cardiomyocytes	KEGG PATHWAY	mmu04261	6.260E-01	7.077E-01	Creb3l1,26427:down|Fxyd2,11936:up
Bile secretion	KEGG PATHWAY	mmu04976	6.327E-01	7.122E-01	Fxyd2,11936:up
Non-alcoholic fatty liver disease (NAFLD)	KEGG PATHWAY	mmu04932	6.330E-01	7.122E-01	Tnf,21926:up|Adipoq,11450:up
Muscle contraction	Reactome	R-MMU-397014	6.365E-01	7.149E-01	Fxyd2,11936:up|Fxyd1,56188:up
Viral carcinogenesis	KEGG PATHWAY	mmu05203	6.374E-01	7.149E-01	Creb3l1,26427:down|H2-T24,15042:up|C3,12266:up
Gastric acid secretion	KEGG PATHWAY	mmu04971	6.428E-01	7.159E-01	Kcnj15,16516:down
Regulation of insulin secretion	Reactome	R-MMU-422356	6.428E-01	7.159E-01	Glp1r,14652:down
Centrosome maturation	Reactome	R-MMU-380287	6.428E-01	7.159E-01	Prkar2b,19088:up
Recruitment of mitotic centrosome proteins and complexes	Reactome	R-MMU-380270	6.428E-01	7.159E-01	Prkar2b,19088:up
TCF dependent signaling in response to WNT	Reactome	R-MMU-201681	6.434E-01	7.159E-01	Rspo2,239405:up|Wnt5a,22418:down
Oxytocin signaling pathway	KEGG PATHWAY	mmu04921	6.502E-01	7.223E-01	Ptgs2,19225:down|Egfr,13649:down
Autoimmune thyroid disease	KEGG PATHWAY	mmu05320	6.622E-01	7.327E-01	H2-T24,15042:up
Salivary secretion	KEGG PATHWAY	mmu04970	6.622E-01	7.327E-01	Fxyd2,11936:up
Bacterial invasion of epithelial cells	KEGG PATHWAY	mmu05100	6.668E-01	7.327E-01	Itga5,16402:down
Cardiac muscle contraction	KEGG PATHWAY	mmu04260	6.668E-01	7.327E-01	Fxyd2,11936:up
PCP/CE pathway	Reactome	R-MMU-4086400	6.668E-01	7.327E-01	Wnt5a,22418:down
Metabolism of polyamines	Reactome	R-MMU-351202	6.668E-01	7.327E-01	Gatm,67092:down
Golgi-to-ER retrograde transport	Reactome	R-MMU-8856688	6.668E-01	7.327E-01	Kdelr3,105785:down
@@Sphingolipid metabolism	Reactome	R-MMU-428157	6.714E-01	7.366E-01	Plpp3,67916:up
Regulation of PLK1 Activity at G2/M Transition	Reactome	R-MMU-2565942	6.760E-01	7.404E-01	Prkar2b,19088:up
Aldosterone synthesis and secretion	KEGG PATHWAY	mmu04925	6.978E-01	7.619E-01	Creb3l1,26427:down
Downstream TCR signaling	Reactome	R-MMU-202424	7.020E-01	7.653E-01	Cd4,12504:up
Steroid hormone biosynthesis	KEGG PATHWAY	mmu00140	7.061E-01	7.686E-01	Akr1c18,105349:up
Fc gamma R-mediated phagocytosis	KEGG PATHWAY	mmu04666	7.102E-01	7.718E-01	Plpp3,67916:up

Retinol metabolism	KEGG PATHWAY	mmu00830	7.142E-01	7.726E-01	Aldh1a2,19378:up
Progesterone-mediated oocyte maturation	KEGG PATHWAY	mmu04914	7.142E-01	7.726E-01	Igf1,16000:up
MHC class II antigen presentation	Reactome	R-MMU-2132295	7.142E-01	7.726E-01	Sec23a,20334:down
Antigen processing-Cross presentation	Reactome	R-MMU-1236975	7.181E-01	7.757E-01	H2-T24,15042:up
Anchoring of the basal body to the plasma membrane	Reactome	R-MMU-5620912	7.221E-01	7.775E-01	Prkar2b,19088:up
UCH proteinases	Reactome	R-MMU-5689603	7.221E-01	7.775E-01	Uchl5,56207:up
Glycerophospholipid metabolism	KEGG PATHWAY	mmu00564	7.334E-01	7.874E-01	Plpp3,67916:up
Potassium Channels	Reactome	R-MMU-1296071	7.334E-01	7.874E-01	Kcnj15,16516:down
Huntington's disease	KEGG PATHWAY	mmu05016	7.503E-01	8.042E-01	Creb3l1,26427:down|Pparg,19016:up
Glucagon signaling pathway	KEGG PATHWAY	mmu04922	7.582E-01	8.102E-01	Creb3l1,26427:down
Beta-catenin independent WNT signaling	Reactome	R-MMU-3858494	7.582E-01	8.102E-01	Wnt5a,22418:down
@@Pyrimidine metabolism	KEGG PATHWAY	mmu00240	7.616E-01	8.113E-01	Uck2,80914:down
Retrograde endocannabinoid signaling	KEGG PATHWAY	mmu04723	7.616E-01	8.113E-01	Ptgs2,19225:down
Pancreatic secretion	KEGG PATHWAY	mmu04972	7.649E-01	8.115E-01	Fxyd2,11936:up
Class I MHC mediated antigen processing & presentation	Reactome	R-MMU-983169	7.652E-01	8.115E-01	Sec23a,20334:down|H2-T24,15042:up
TCR signaling	Reactome	R-MMU-202403	7.745E-01	8.201E-01	Cd4,12504:up
Factors involved in megakaryocyte development and platelet production	Reactome	R-MMU-983231	7.776E-01	8.222E-01	Prkar2b,19088:up
Hedgehog 'off' state	Reactome	R-MMU-5610787	7.807E-01	8.241E-01	Prkar2b,19088:up
Generic Transcription Pathway	Reactome	R-MMU-212436	7.818E-01	8.241E-01	Vdr,22337:down|Pparg,19016:up|Igfbp3,16009:up|Plk2,20620:down
C-type lectin receptors (CLRs)	Reactome	R-MMU-5621481	7.897E-01	8.300E-01	Clec4n,56620:up
ER to Golgi Anterograde Transport	Reactome	R-MMU-199977	7.897E-01	8.300E-01	Sec23a,20334:down
Cholinergic synapse	KEGG PATHWAY	mmu04725	7.926E-01	8.318E-01	Creb3l1,26427:down
Oocyte meiosis	KEGG PATHWAY	mmu04114	8.011E-01	8.370E-01	Igf1,16000:up
Mus musculus biological processes	Reactome	R-MMU-5334727	8.012E-01	8.370E-01	Tnf,21926:up|Fabp4,11770:up
Natural killer cell mediated cytotoxicity	KEGG PATHWAY	mmu04650	8.119E-01	8.470E-01	Tnf,21926:up
Sphingolipid signaling pathway	KEGG PATHWAY	mmu04071	8.246E-01	8.576E-01	Tnf,21926:up

Toll-Like Receptors Cascades	Reactome	R-MMU-168898	8.246E-01	8.576E-01	S100a1,20193:up
Signaling by Wnt	Reactome	R-MMU-195721	8.267E-01	8.585E-01	Rspo2,239405:up|Wnt5a,22418:down
Cellular Senescence	Reactome	R-MMU-2559583	8.318E-01	8.626E-01	Hmga1-rs1,111241:up
Vascular smooth muscle contraction	KEGG PATHWAY	mmu04270	8.341E-01	8.637E-01	Ptgir,19222:up
Neurotransmitter Receptor Binding And Downstream Transmission In The Postsynaptic Cell	Reactome	R-MMU-112314	8.431E-01	8.717E-01	Kcnj15,16516:down
Intra-Golgi and retrograde Golgi-to-ER traffic	Reactome	R-MMU-6811442	8.475E-01	8.736E-01	Kdelr3,105785:down
Phase 1 - Functionalization of compounds	Reactome	R-MMU-211945	8.475E-01	8.736E-01	Maob,109731:up
Apoptosis	KEGG PATHWAY	mmu04210	8.557E-01	8.809E-01	Tnf,21926:up
Tight junction	KEGG PATHWAY	mmu04530	8.597E-01	8.837E-01	Shroom4,208431:down
Insulin signaling pathway	KEGG PATHWAY	mmu04910	8.617E-01	8.844E-01	Prkar2b,19088:up
Transcriptional Regulation by TP53	Reactome	R-MMU-3700989	8.662E-01	8.877E-01	Igfbp3,16009:up|Plk2,20620:down
Signaling by Hedgehog	Reactome	R-MMU-5358351	8.710E-01	8.913E-01	Prkar2b,19088:up
Phospholipid metabolism	Reactome	R-MMU-1483257	8.830E-01	9.023E-01	Gdpd1,66569:up
G2/M Transition	Reactome	R-MMU-69275	8.878E-01	9.059E-01	Prkar2b,19088:up
Mitotic G2-G2/M phases	Reactome	R-MMU-453274	8.909E-01	9.078E-01	Prkar2b,19088:up
Post-translational protein modification	Reactome	R-MMU-597592	8.991E-01	9.148E-01	Sec23a,20334:down|Npl,74091:up|Man2a2,140481:up|Gas6,14456:up|Uchl5,56207:up|Gfpt2,14584:down
Protein processing in endoplasmic reticulum	KEGG PATHWAY	mmu04141	9.025E-01	9.168E-01	Sec23a,20334:down
Assembly of the primary cilium	Reactome	R-MMU-5617833	9.116E-01	9.247E-01	Prkar2b,19088:up
Cellular responses to stress	Reactome	R-MMU-2262752	9.170E-01	9.290E-01	Hmga1-rs1,111241:up|Epas1,13819:up
Transmission across Chemical Synapses	Reactome	R-MMU-112315	9.322E-01	9.430E-01	Kcnj15,16516:down
Biological oxidations	Reactome	R-MMU-211859	9.488E-01	9.583E-01	Maob,109731:up
Epstein-Barr virus infection	KEGG PATHWAY	mmu05169	9.522E-01	9.605E-01	H2-T24,15042:up
Deubiquitination	Reactome	R-MMU-5688426	9.607E-01	9.676E-01	Uchl5,56207:up
Organelle biogenesis and maintenance	Reactome	R-MMU-1852241	9.800E-01	9.856E-01	Prkar2b,19088:up
Neuronal System	Reactome	R-MMU-112316	9.853E-01	9.895E-01	Kcnj15,16516:down
Cell Cycle, Mitotic	Reactome	R-MMU-69278	9.972E-01	9.993E-01	Prkar2b,19088:up

Gene Expression	Reactome	R-MMU-74160	9.979E-01	9.993E-01	Pparg,19016:up|Nabp1,109019:down|Plk2,20620:down|Ice1,218333:down|Vdr,22337:down|Igfbp3,16009:up
@@Cell Cycle	Reactome	R-MMU-1640170	9.997E-01	9.997E-01	Prkar2b,19088:up
