# Supplementary material for: MicroRNA-26b-5p Inhibits Mouse Liver Fibrogenesis and Angiogenesis by Targeting PDGF Receptor-Beta
Source: Mol Ther Nucleic Acids. 2019 Feb 26;16:206–17. doi: 10.1016/j.omtn.2019.02.014 (PMC6426711; doi:10.1016/j.omtn.2019.02.014)
Supplement: Table S4. GO Terms (Biological Process) in TGF-b1-Treated BMSCs with or without miR-26b-5p Mimics [file mmc4.pdf]

MicroRNA-26b-5p Inhibits Mouse Liver Fibrogenesis and Angiogenesis by Targeting PDGF Receptor-beta

Le Yang#, Chengbin Dong#, Jingjing Yang, Lin Yang, Na Chang, Changbo Qi, Liying Li*

Supplementary table 4. GO terms (Biological Process) in TGF-b1-treated BMSCs with or without miR-26b-5p mimics

Term	ID	P-Value	Corrected P-Value	input_gene
regulation of multicellular organismal process	GO:0051239	6.36E-22	6.43E-19	Fstl3,83554:down|Clec2d,93694:up|Adm,11535:up|Flrt2,399558:down|Tmem176b,65963:up|Plxnd1,67784:up|Sfrp4,20379:up|C3,12266:up|Ankrd1,107765:down|Cx3cr1,13051:down|Cd109,235505:down|Loxl2,94352:down|Plk2,20620:down|Trf,22041:up|Cd24a,12484:down|Wnt5a,22418:down|Stc1,20855:up|Il1rn,16181:up|Adipoq,11450:up|Bmp4,12159:up|Igf1,16000:up|Crabp2,12904:down|Tgfb2,21808:down|Hpse,15442:up|F3,14066:up|Fgfr2,14183:up|Thbs2,21826:down|Clec4e,56619:up|Cthrc1,68588:down|Htr2b,15559:down|Kng1,16644:up|Prl2c2,18811:up|Efemp1,216616:up|Dpysl3,22240:down|Aspn,66695:down|Vdr,22337:down|Cxcl12,20315:up|Htr2a,15558:down|Mmp2,17390:down|Gas6,14456:up|Egfr,13649:down|Sfrp1,20377:up|C1qtnf3,81799:down|Tgfbr3,21814:up|Tfrc,22042:up|Ceacam1,26365:up|Sfrp2,20319:up|Rspo2,239405:up|Npnt,114249:up|Tnfrsf11b,18383:up|S100a1,20193:up|Col5a2,12832:down|Ly9,17085:up|Ccl2,20296:up|Ednrb,13618:up|Figf,14205:up|Mgp,17313:up|Mmp13,17386:up|Sema7a,20361:down|Epas1,13819:up|Tnfrsf12a,27279:down|Gpm6b,14758:up|Ddah1,69219:down|Clec4n,56620:up|Postn,50706:down|Ncam1,17967:down|Bmpr1a,12166:down|Wisp1,22402:down|Ccl4,20303:up|Ank,11732:down|Ret,19713:up|Prg4,96875:down|Plpp3,67916:up|Creb3l1,26427:down|Il1rl1,17082:down|Kitl,17311:up|Fam101b,76566:down|Lrrc17,74511:down|Lama2,16773:down|Ptk7,71461:down|Inhba,16323:down|Plat,18791:down|Ccl3,20302:up|Ctla2a,13024:down|Srpx2,68792:down|Vegfa,22339:down|Serpine2,20720:up|Adamts12,239337:down|Igf2,16002:up|Anpep,16790:up|Pparg,19016:up|Tnf,21926:up|Tmem176a,66058:up|Glp1r,14652:down|Lmcd1,30937:up|Fxyd1,56188:up|Fgfr1,14182:down|Has2,15117:down|Lpl,16956:up|Hmox1,15368:up|Sulf1,240725:down|Ptgs2,19225:down|Grem1,23892:up|Col3a1,12825:down|Serping1,12258:up|Col5a1,12831:down|Errfi1,74155:down|Enpp1,18605:down|Tnfsf9,21950:up
response to wounding	GO:0009611	1.41E-18	1.22E-15	Adm,11535:up|Ccl7,20306:up|Ccl2,20296:up|C3,12266:up|Pdgfc,54635:down|Ednrb,13618:up|Sema7a,20361:down|Tnfrsf12a,27279:down|Cx3cr1,13051:down|Cd109,235505:down|Cd24a,12484:down|Wnt5a,22418:down|Ccl12,20293:up|Ccl4,20303:up|Adipoq,11450:up|Igf1,16000:up|Tnc,21923:down|Tgfb2,21808:down|Hpse,15442:up|Plpp3,67916:up|F3,14066:up|Il1rl1,17082:down|Kng1,16644:up|Ccl9,20308:up|Ccl6,20305:up|Ptk7,71461:down|Aoah,27052:up|Anxa8,11752:down|Ninj1,18081:up|Ccl3,20302:up|Tfpi2,21789:down|Ctla2a,13024:down|Fabp4,11770:up|Mmp2,17390:down|Serpine2,20720:up|Adamts12,239337:down|Pparg,19016:up|Gas6,14456:up|Tnf,21926:up|Egfr,13649:down|Hmox1,15368:up|C1qtnf3,81799:down|Ptgs2,19225:down|Col3a1,12825:down|Ccl8,20307:up|Ceacam1,26365:up|Serping1,12258:up|Col5a1,12831:down
cell migration	GO:0016477	3.91E-18	2.96E-15	Flrt2,399558:down|Plxnd1,67784:up|Cx3cr1,13051:down|Igfbp3,16009:up|Loxl2,94352:down|Cxcl14,57266:down|Cd24a,12484:down|Wnt5a,22418:down|Mark1,226778:up|Stc1,20855:up|Il1rn,16181:up|Adipoq,11450:up|Bmp4,12159:up|Igf1,16000:up|Cxcl9,17329:up|Tgfb2,21808:down|F3,14066:up|Cthrc1,68588:down|Htr2b,15559:down|Prl2c2,18811:up|Efemp1,216616:up|Ccl6,20305:up|Dpysl3,22240:down|Saa3,20210:up|Cxcl12,20315:up|Mmp2,17390:down|Gas6,14456:up|Egfr,13649:down|Sfrp1,20377:up|Tgfbr3,21814:up|Ccl8,20307:up|Ceacam1,26365:up|Sfrp2,20319:up|Ccl2,20296:up|Ccl7,20306:up|Ednrb,13618:up|Figf,14205:up|Pdgfc,54635:down|Sema7a,20361:down|Tnfrsf12a,27279:down|Postn,50706:down|Ccl12,20293:up|Bmpr1a,12166:down|Ccl4,20303:up|Ret,19713:up|Plpp3,67916:up|Kitl,17311:up|Ccl9,20308:up|Lama2,16773:down|Ptk7,71461:down|Ccl3,20302:up|Plat,18791:down|Srpx2,68792:down|Vegfa,22339:down|Lrrc15,74488:down|Adamts12,239337:down|Tnf,21926:up|Fgfr1,14182:down|Has2,15117:down|Sulf1,240725:down|Ptgs2,19225:down|Grem1,23892:up|Col3a1,12825:down|Col5a1,12831:down
cell motility	GO:0048870	4.98E-18	3.02E-15	Flrt2,399558:down|Plxnd1,67784:up|Cx3cr1,13051:down|Igfbp3,16009:up|Loxl2,94352:down|Trf,22041:up|Cxcl14,57266:down|Cd24a,12484:down|Ceacam10,26366:up|Wnt5a,22418:down|Mark1,226778:up|Stc1,20855:up|Il1rn,16181:up|Adipoq,11450:up|Bmp4,12159:up|Igf1,16000:up|Cxcl9,17329:up|Tgfb2,21808:down|F3,14066:up|Cthrc1,68588:down|Htr2b,15559:down|Prl2c2,18811:up|Efemp1,216616:up|Ccl6,20305:up|Dpysl3,22240:down|Saa3,20210:up|Cxcl12,20315:up|Mmp2,17390:down|Gas6,14456:up|Egfr,13649:down|Sfrp1,20377:up|Tgfbr3,21814:up|Ccl8,20307:up|Ceacam1,26365:up|Sfrp2,20319:up|Ccl2,20296:up|Ccl7,20306:up|Ednrb,13618:up|Figf,14205:up|Pdgfc,54635:down|Sema7a,20361:down|Sord,20322:up|Tnfrsf12a,27279:down|Postn,50706:down|Ccl12,20293:up|Bmpr1a,12166:down|Ccl4,20303:up|Ret,19713:up|Plpp3,67916:up|Kitl,17311:up|Ccl9,20308:up|Lama2,16773:down|Ptk7,71461:down|Ccl3,20302:up|Plat,18791:down|Srpx2,68792:down|Vegfa,22339:down|Adamts12,239337:down|Lrrc15,74488:down|Tnf,21926:up|Fgfr1,14182:down|Has2,15117:down|Sulf1,240725:down|Ptgs2,19225:down|Grem1,23892:up|Col3a1,12825:down|Col5a1,12831:down
localization of cell	GO:0051674	4.98E-18	3.02E-15	Flrt2,399558:down|Plxnd1,67784:up|Cx3cr1,13051:down|Igfbp3,16009:up|Loxl2,94352:down|Trf,22041:up|Cxcl14,57266:down|Cd24a,12484:down|Ceacam10,26366:up|Wnt5a,22418:down|Mark1,226778:up|Stc1,20855:up|Il1rn,16181:up|Adipoq,11450:up|Bmp4,12159:up|Igf1,16000:up|Cxcl9,17329:up|Tgfb2,21808:down|F3,14066:up|Cthrc1,68588:down|Htr2b,15559:down|Prl2c2,18811:up|Efemp1,216616:up|Ccl6,20305:up|Dpysl3,22240:down|Saa3,20210:up|Cxcl12,20315:up|Mmp2,17390:down|Gas6,14456:up|Egfr,13649:down|Sfrp1,20377:up|Tgfbr3,21814:up|Ccl8,20307:up|Ceacam1,26365:up|Sfrp2,20319:up|Ccl2,20296:up|Ccl7,20306:up|Ednrb,13618:up|Figf,14205:up|Pdgfc,54635:down|Sema7a,20361:down|Sord,20322:up|Tnfrsf12a,27279:down|Postn,50706:down|Ccl12,20293:up|Bmpr1a,12166:down|Ccl4,20303:up|Ret,19713:up|Plpp3,67916:up|Kitl,17311:up|Ccl9,20308:up|Lama2,16773:down|Ptk7,71461:down|Ccl3,20302:up|Plat,18791:down|Srpx2,68792:down|Vegfa,22339:down|Adamts12,239337:down|Lrrc15,74488:down|Tnf,21926:up|Fgfr1,14182:down|Has2,15117:down|Sulf1,240725:down|Ptgs2,19225:down|Grem1,23892:up|Col3a1,12825:down|Col5a1,12831:down
vasculature development	GO:0001944	1.38E-17	7.63E-15	Adm,11535:up|Hpgd,15446:up|Plxnd1,67784:up|Ccl2,20296:up|Aldh1a2,19378:up|C3,12266:up|Figf,14205:up|Epas1,13819:up|Tnfrsf12a,27279:down|Ddah1,69219:down|Lyl1,17095:up|Cx3cr1,13051:down|Loxl2,94352:down|Wnt5a,22418:down|Bmpr1a,12166:down|Ccl12,20293:up|Col8a1,12837:down|Bmp4,12159:up|Igf1,16000:up|Tgfb2,21808:down|Vegfb,22340:up|Hpse,15442:up|Plpp3,67916:up|Fgfr2,14183:up|F3,14066:up|Thbs2,21826:down|Prl2c2,18811:up|Ptk7,71461:down|Srpx2,68792:down|Cxcl12,20315:up|Vegfa,22339:down|Mmp2,17390:down|Anpep,16790:up|Col1a2,12843:down|Fgfr1,14182:down|Has2,15117:down|Hmox1,15368:up|Sulf1,240725:down|Tgfbr3,21814:up|Ptgs2,19225:down|Grem1,23892:up|Col3a1,12825:down|Ceacam1,26365:up|Sfrp2,20319:up|Col5a1,12831:down|Errfi1,74155:down

ossification	GO:0001503	4.24E-17	2.06E-14	Fstl3,83554:down|Mgp,17313:up|Mmp13,17386:up|Sema7a,20361:down|Gpm6b,14758:up|Ibsp,15891:up|Igfbp3,16009:up|Wnt5a,22418:down|Bmpr1a,12166:down|Stc1,20855:up|Bmp4,12159:up|Igf1,16000:up|Ank,11732:down|Tnc,21923:down|Creb3l1,26427:down|Fgfr2,14183:up|Mrc2,17534:down|Fam101b,76566:down|Cthrc1,68588:down|Lrrc17,74511:down|Aspn,66695:down|Vegfa,22339:down|Mmp2,17390:down|Igf2,16002:up|Tnf,21926:up|Alpl,11647:up|Fgfr1,14182:down|Sfrp1,20377:up|Tgfbr3,21814:up|Ptgs2,19225:down|Grem1,23892:up|Sfrp2,20319:up|Rspo2,239405:up|Npnt,114249:up|Enpp1,18605:down
organ development	GO:0048513	4.41E-17	2.06E-14	Fstl3,83554:down|Clec2d,93694:up|Adm,11535:up|Flrt2,399558:down|Tmem176b,65963:up|Plxnd1,67784:up|Ankrd1,107765:down|Serpinh1,12406:down|Lyl1,17095:up|Cx3cr1,13051:down|Ibsp,15891:up|Cd109,235505:down|Loxl2,94352:down|Loxl3,16950:down|Trf,22041:up|Cxcl14,57266:down|Cd24a,12484:down|Wnt5a,22418:down|Stc1,20855:up|Col8a1,12837:down|Adipoq,11450:up|Bmp4,12159:up|Igf1,16000:up|Tgfb2,21808:down|Hpse,15442:up|Fgfr2,14183:up|Clec4e,56619:up|Cthrc1,68588:down|Htr2b,15559:down|Efemp1,216616:up|Gjb5,14622:down|Aspn,66695:down|Ninj1,18081:up|Vdr,22337:down|Cxcl12,20315:up|Mmp2,17390:down|Slc40a1,53945:up|Gas6,14456:up|Egfr,13649:down|Col11a1,12814:down|Alpl,11647:up|Sfrp1,20377:up|Tgfbr3,21814:up|Tfrc,22042:up|Ceacam1,26365:up|Sfrp2,20319:up|Rspo2,239405:up|Fam20a,208659:down|Cdsn,386463:up|Npnt,114249:up|Tnfrsf11b,18383:up|Col5a2,12832:down|Ly9,17085:up|Aldh1a2,19378:up|Pdgfc,54635:down|Ednrb,13618:up|Mgp,17313:up|Mmp13,17386:up|Sema7a,20361:down|Epas1,13819:up|Uchl5,56207:up|Gpm6b,14758:up|Shroom4,208431:down|Cdo1,12583:up|Ncam1,17967:down|Bmpr1a,12166:down|Wisp1,22402:down|Chst11,58250:down|Ank,11732:down|Tnc,21923:down|Ret,19713:up|Prg4,96875:down|Vegfb,22340:up|Aldh1a3,56847:down|Kitl,17311:up|Fam101b,76566:down|Lrrc17,74511:down|Hp,15439:up|Ptk7,71461:down|Inhba,16323:down|Bnc2,242509:down|Ccl3,20302:up|Ctla2a,13024:down|Crlf1,12931:down|Vegfa,22339:down|Serpine2,20720:up|Adamts12,239337:down|Igf2,16002:up|Pparg,19016:up|Tnf,21926:up|Tmem176a,66058:up|Col1a2,12843:down|Fgfr1,14182:down|Has2,15117:down|Hmox1,15368:up|Sulf1,240725:down|Cd4,12504:up|Ptgs2,19225:down|Grem1,23892:up|Col3a1,12825:down|Pcdh19,279653:down|Tbx15,21384:down|Mcoln3,171166:up|Col5a1,12831:down|Errfi1,74155:down|Enpp1,18605:down|Tnfsf9,21950:up
regulation of developmental process	GO:0050793	5.68E-17	2.46E-14	Fstl3,83554:down|Clec2d,93694:up|Adm,11535:up|Flrt2,399558:down|Tmem176b,65963:up|Plxnd1,67784:up|Sfrp4,20379:up|C3,12266:up|Ankrd1,107765:down|Cx3cr1,13051:down|Igfbp3,16009:up|Cd109,235505:down|Loxl2,94352:down|Plk2,20620:down|Trf,22041:up|Cxcl14,57266:down|Cd24a,12484:down|Wnt5a,22418:down|Il1rn,16181:up|Adipoq,11450:up|Bmp4,12159:up|Igf1,16000:up|Crabp2,12904:down|Cxcl9,17329:up|Tgfb2,21808:down|Hpse,15442:up|Medag,70717:down|Fgfr2,14183:up|F3,14066:up|Thbs2,21826:down|Cthrc1,68588:down|Prl2c2,18811:up|Efemp1,216616:up|Dpysl3,22240:down|Aspn,66695:down|Vdr,22337:down|Cxcl12,20315:up|Htr2a,15558:down|Gas6,14456:up|Sfrp1,20377:up|Tgfbr3,21814:up|Tfrc,22042:up|Ceacam1,26365:up|Ccl8,20307:up|Sfrp2,20319:up|Rspo2,239405:up|Npnt,114249:up|Tnfrsf11b,18383:up|Col5a2,12832:down|Ccl2,20296:up|Ednrb,13618:up|Mgp,17313:up|Sema7a,20361:down|Tnfrsf12a,27279:down|Gpm6b,14758:up|Ddah1,69219:down|Postn,50706:down|Ncam1,17967:down|Ccl12,20293:up|Bmpr1a,12166:down|Wisp1,22402:down|Ank,11732:down|Ret,19713:up|Kitl,17311:up|Fam101b,76566:down|Lrrc17,74511:down|Ccl9,20308:up|Lama2,16773:down|Ptk7,71461:down|Inhba,16323:down|Ccl3,20302:up|Ctla2a,13024:down|Srpx2,68792:down|Vegfa,22339:down|Serpine2,20720:up|Adamts12,239337:down|Igf2,16002:up|Pparg,19016:up|Tnf,21926:up|Tmem176a,66058:up|Glp1r,14652:down|Fgfr1,14182:down|Hmox1,15368:up|Lpl,16956:up|Has2,15117:down|Sulf1,240725:down|Ptgs2,19225:down|Grem1,23892:up|Col3a1,12825:down|Col5a1,12831:down|Errfi1,74155:down|Enpp1,18605:down|Tnfsf9,21950:up
blood vessel development	GO:0001568	8.90E-17	3.60E-14	Adm,11535:up|Hpgd,15446:up|Plxnd1,67784:up|Ccl2,20296:up|Aldh1a2,19378:up|C3,12266:up|Figf,14205:up|Epas1,13819:up|Tnfrsf12a,27279:down|Ddah1,69219:down|Lyl1,17095:up|Cx3cr1,13051:down|Loxl2,94352:down|Wnt5a,22418:down|Bmpr1a,12166:down|Ccl12,20293:up|Col8a1,12837:down|Bmp4,12159:up|Tgfb2,21808:down|Vegfb,22340:up|Hpse,15442:up|Plpp3,67916:up|Fgfr2,14183:up|F3,14066:up|Thbs2,21826:down|Prl2c2,18811:up|Ptk7,71461:down|Srpx2,68792:down|Cxcl12,20315:up|Vegfa,22339:down|Mmp2,17390:down|Anpep,16790:up|Col1a2,12843:down|Fgfr1,14182:down|Hmox1,15368:up|Has2,15117:down|Sulf1,240725:down|Tgfbr3,21814:up|Ptgs2,19225:down|Grem1,23892:up|Col3a1,12825:down|Ceacam1,26365:up|Sfrp2,20319:up|Col5a1,12831:down
regulation of multicellular organismal development	GO:2000026	1.58E-16	5.98E-14	Fstl3,83554:down|Clec2d,93694:up|Adm,11535:up|Flrt2,399558:down|Tmem176b,65963:up|Plxnd1,67784:up|Sfrp4,20379:up|C3,12266:up|Ankrd1,107765:down|Cx3cr1,13051:down|Cd109,235505:down|Loxl2,94352:down|Plk2,20620:down|Trf,22041:up|Cd24a,12484:down|Wnt5a,22418:down|Il1rn,16181:up|Adipoq,11450:up|Bmp4,12159:up|Igf1,16000:up|Crabp2,12904:down|Tgfb2,21808:down|Hpse,15442:up|Fgfr2,14183:up|F3,14066:up|Thbs2,21826:down|Cthrc1,68588:down|Prl2c2,18811:up|Efemp1,216616:up|Dpysl3,22240:down|Aspn,66695:down|Vdr,22337:down|Cxcl12,20315:up|Gas6,14456:up|Sfrp1,20377:up|Tgfbr3,21814:up|Tfrc,22042:up|Ceacam1,26365:up|Sfrp2,20319:up|Rspo2,239405:up|Tnfrsf11b,18383:up|Col5a2,12832:down|Ccl2,20296:up|Ednrb,13618:up|Mgp,17313:up|Sema7a,20361:down|Tnfrsf12a,27279:down|Gpm6b,14758:up|Ddah1,69219:down|Ncam1,17967:down|Bmpr1a,12166:down|Wisp1,22402:down|Ank,11732:down|Ret,19713:up|Kitl,17311:up|Fam101b,76566:down|Lrrc17,74511:down|Lama2,16773:down|Ptk7,71461:down|Inhba,16323:down|Ccl3,20302:up|Ctla2a,13024:down|Srpx2,68792:down|Vegfa,22339:down|Serpine2,20720:up|Adamts12,239337:down|Tnf,21926:up|Pparg,19016:up|Tmem176a,66058:up|Fgfr1,14182:down|Hmox1,15368:up|Sulf1,240725:down|Ptgs2,19225:down|Grem1,23892:up|Col3a1,12825:down|Col5a1,12831:down|Errfi1,74155:down|Tnfsf9,21950:up
positive regulation of multicellular organismal process	GO:0051240	1.83E-16	6.55E-14	Adm,11535:up|Flrt2,399558:down|Sfrp4,20379:up|C3,12266:up|Ankrd1,107765:down|Cx3cr1,13051:down|Loxl2,94352:down|Trf,22041:up|Cd24a,12484:down|Wnt5a,22418:down|Adipoq,11450:up|Bmp4,12159:up|Igf1,16000:up|Crabp2,12904:down|Tgfb2,21808:down|Hpse,15442:up|Fgfr2,14183:up|F3,14066:up|Thbs2,21826:down|Clec4e,56619:up|Cthrc1,68588:down|Htr2b,15559:down|Prl2c2,18811:up|Dpysl3,22240:down|Vdr,22337:down|Cxcl12,20315:up|Htr2a,15558:down|Gas6,14456:up|Egfr,13649:down|C1qtnf3,81799:down|Tfrc,22042:up|Ceacam1,26365:up|Sfrp2,20319:up|Npnt,114249:up|Ly9,17085:up|Ccl2,20296:up|Ednrb,13618:up|Figf,14205:up|Mmp13,17386:up|Sema7a,20361:down|Tnfrsf12a,27279:down|Gpm6b,14758:up|Ddah1,69219:down|Clec4n,56620:up|Postn,50706:down|Ncam1,17967:down|Bmpr1a,12166:down|Wisp1,22402:down|Ccl4,20303:up|Ret,19713:up|Plpp3,67916:up|Kitl,17311:up|Il1rl1,17082:down|Ptk7,71461:down|Inhba,16323:down|Ccl3,20302:up|Plat,18791:down|Srpx2,68792:down|Vegfa,22339:down|Serpine2,20720:up|Igf2,16002:up|Tnf,21926:up|Pparg,19016:up|Glp1r,14652:down|Fgfr1,14182:down|Hmox1,15368:up|Lpl,16956:up|Has2,15117:down|Sulf1,240725:down|Ptgs2,19225:down|Grem1,23892:up|Tnfsf9,21950:up

system development	GO:0048731	3.61E-16	1.22E-13	Fstl3,83554:down|Clec2d,93694:up|Adm,11535:up|Flrt2,399558:down|Tmem176b,65963:up|Hpgd,15446:up|Plxnd1,67784:up|C3,12266:up|Ankrd1,107765:down|Serpinh1,12406:down|Lyl1,17095:up|Cx3cr1,13051:down|Ibsp,15891:up|Cd109,235505:down|Loxl2,94352:down|Plk2,20620:down|Loxl3,16950:down|Trf,22041:up|Cxcl14,57266:down|Cd24a,12484:down|Wnt5a,22418:down|Mark1,226778:up|Stc1,20855:up|Col8a1,12837:down|Igf1,16000:up|Adipoq,11450:up|Bmp4,12159:up|Crabp2,12904:down|Tgfb2,21808:down|Hpse,15442:up|F3,14066:up|Fgfr2,14183:up|Thbs2,21826:down|Clec4e,56619:up|Cthrc1,68588:down|Htr2b,15559:down|Prl2c2,18811:up|Efemp1,216616:up|Gjb5,14622:down|Dpysl3,22240:down|Aspn,66695:down|Ninj1,18081:up|Vdr,22337:down|Cxcl12,20315:up|Mmp2,17390:down|Slc40a1,53945:up|Gas6,14456:up|Egfr,13649:down|Col11a1,12814:down|Alpl,11647:up|Sfrp1,20377:up|Tgfbr3,21814:up|Tfrc,22042:up|Ceacam1,26365:up|Sfrp2,20319:up|Rspo2,239405:up|Fam20a,208659:down|Cdsn,386463:up|Npnt,114249:up|Tnfrsf11b,18383:up|Col5a2,12832:down|Ly9,17085:up|Aldh1a2,19378:up|Ccl2,20296:up|Pdgfc,54635:down|Ednrb,13618:up|Figf,14205:up|Mgp,17313:up|Mmp13,17386:up|Sema7a,20361:down|Epas1,13819:up|Tnfrsf12a,27279:down|Uchl5,56207:up|Gpm6b,14758:up|Ddah1,69219:down|Shroom4,208431:down|Postn,50706:down|Cdo1,12583:up|Ncam1,17967:down|Bmpr1a,12166:down|Ccl12,20293:up|Wisp1,22402:down|Chst11,58250:down|Ank,11732:down|Tnc,21923:down|Ret,19713:up|Prg4,96875:down|Vegfb,22340:up|Aldh1a3,56847:down|Plpp3,67916:up|Kitl,17311:up|Fam101b,76566:down|Lrrc17,74511:down|Lama2,16773:down|Hp,15439:up|Ptk7,71461:down|Inhba,16323:down|Bnc2,242509:down|Ccl3,20302:up|Ctla2a,13024:down|Srpx2,68792:down|Crlf1,12931:down|Vegfa,22339:down|Serpine2,20720:up|Adamts12,239337:down|Nxn,18230:down|Igf2,16002:up|Anpep,16790:up|Pparg,19016:up|Tnf,21926:up|Tmem176a,66058:up|Col1a2,12843:down|Fgfr1,14182:down|Has2,15117:down|Hmox1,15368:up|Sulf1,240725:down|Cd4,12504:up|Ptgs2,19225:down|Grem1,23892:up|Col3a1,12825:down|Pcdh19,279653:down|Tbx15,21384:down|Col5a1,12831:down|Mcoln3,171166:up|Errfi1,74155:down|Enpp1,18605:down|Tnfsf9,21950:up
positive regulation of intracellular signal transduction	GO:1902533	5.89E-16	1.86E-13	Igfbp4,16010:up|Ccl7,20306:up|Ccl2,20296:up|C3,12266:up|Pdgfc,54635:down|Sema7a,20361:down|Ankrd1,107765:down|Clec4n,56620:up|Igfbp3,16009:up|Plk2,20620:down|Akr1c18,105349:up|Trf,22041:up|Cd24a,12484:down|Ncam1,17967:down|Wnt5a,22418:down|Ccl12,20293:up|Il1rn,16181:up|Ccl4,20303:up|Adipoq,11450:up|Bmp4,12159:up|Igf1,16000:up|Tgfb2,21808:down|Hpse,15442:up|Ptgir,19222:up|Fgfr2,14183:up|F3,14066:up|Kitl,17311:up|Htr2b,15559:down|Ccl9,20308:up|Ccl6,20305:up|Ccl3,20302:up|Crlf1,12931:down|Vegfa,22339:down|Htr2a,15558:down|Igf2,16002:up|Gas6,14456:up|Tnf,21926:up|Lmcd1,30937:up|Fgfr1,14182:down|Egfr,13649:down|Hmox1,15368:up|C1qtnf3,81799:down|Cd4,12504:up|Ptgs2,19225:down|Grem1,23892:up|Col3a1,12825:down|Ccl8,20307:up|Ceacam1,26365:up|Npnt,114249:up|Tnfrsf11b,18383:up
regulation of cell migration	GO:0030334	6.14E-16	1.86E-13	Flrt2,399558:down|Plxnd1,67784:up|Ccl2,20296:up|Pdgfc,54635:down|Figf,14205:up|Sema7a,20361:down|Postn,50706:down|Cx3cr1,13051:down|Igfbp3,16009:up|Cxcl14,57266:down|Wnt5a,22418:down|Bmpr1a,12166:down|Ccl12,20293:up|Stc1,20855:up|Il1rn,16181:up|Ccl4,20303:up|Adipoq,11450:up|Bmp4,12159:up|Igf1,16000:up|Cxcl9,17329:up|Ret,19713:up|Tgfb2,21808:down|Plpp3,67916:up|F3,14066:up|Kitl,17311:up|Prl2c2,18811:up|Lama2,16773:down|Efemp1,216616:up|Dpysl3,22240:down|Srpx2,68792:down|Cxcl12,20315:up|Vegfa,22339:down|Mmp2,17390:down|Lrrc15,74488:down|Gas6,14456:up|Egfr,13649:down|Fgfr1,14182:down|Sfrp1,20377:up|Has2,15117:down|Sulf1,240725:down|Tgfbr3,21814:up|Ptgs2,19225:down|Grem1,23892:up|Col3a1,12825:down|Ceacam1,26365:up|Sfrp2,20319:up
cellular response to chemical stimulus	GO:0070887	6.60E-16	1.91E-13	Fstl3,83554:down|Flrt2,399558:down|Hpgd,15446:up|Ankrd1,107765:down|Gstt1,14871:up|Cx3cr1,13051:down|Ibsp,15891:up|Cd109,235505:down|Trf,22041:up|Cxcl14,57266:down|Cd24a,12484:down|Mme,17380:up|Wnt5a,22418:down|Il1rn,16181:up|Igf1,16000:up|Adipoq,11450:up|Bmp4,12159:up|Cxcl9,17329:up|Tgfb2,21808:down|Ptgir,19222:up|Fgfr2,14183:up|F3,14066:up|Htr2b,15559:down|Ccl6,20305:up|Aspn,66695:down|Saa3,20210:up|Vdr,22337:down|Fabp4,11770:up|Cxcl12,20315:up|Mmp2,17390:down|Slc40a1,53945:up|Gas6,14456:up|Egfr,13649:down|Alpl,11647:up|Sfrp1,20377:up|Tgfbr3,21814:up|Tfrc,22042:up|Ceacam1,26365:up|Ccl8,20307:up|Sfrp2,20319:up|Gstm1,14862:up|Npnt,114249:up|Mt1,17748:up|Col5a2,12832:down|Mrc1,17533:up|Aldh1a2,19378:up|Ccl2,20296:up|Ccl7,20306:up|Pdgfc,54635:down|Ednrb,13618:up|Figf,14205:up|Ifit3,15959:up|Epas1,13819:up|Htra3,78558:down|Postn,50706:down|Hvcn1,74096:up|Akr1c18,105349:up|Ccl12,20293:up|Bmpr1a,12166:down|Ccl4,20303:up|Chst11,58250:down|Ret,19713:up|Sdf2l1,64136:up|Ifit1,15957:up|Creb3l1,26427:down|Hp,15439:up|Ccl9,20308:up|Ptk7,71461:down|Inhba,16323:down|Ccl3,20302:up|Vegfa,22339:down|Adamts12,239337:down|Lrrc15,74488:down|Igf2,16002:up|Pparg,19016:up|Tnf,21926:up|Col1a2,12843:down|Fgfr1,14182:down|Hmox1,15368:up|Has2,15117:down|Sulf1,240725:down|Grem1,23892:up|Col3a1,12825:down|Enpp1,18605:down
regulation of cell motility	GO:2000145	9.10E-16	2.51E-13	Flrt2,399558:down|Plxnd1,67784:up|Ccl2,20296:up|Pdgfc,54635:down|Figf,14205:up|Sema7a,20361:down|Postn,50706:down|Cx3cr1,13051:down|Igfbp3,16009:up|Trf,22041:up|Cxcl14,57266:down|Wnt5a,22418:down|Bmpr1a,12166:down|Ccl12,20293:up|Stc1,20855:up|Il1rn,16181:up|Ccl4,20303:up|Adipoq,11450:up|Bmp4,12159:up|Igf1,16000:up|Cxcl9,17329:up|Ret,19713:up|Tgfb2,21808:down|Plpp3,67916:up|F3,14066:up|Kitl,17311:up|Prl2c2,18811:up|Lama2,16773:down|Efemp1,216616:up|Dpysl3,22240:down|Srpx2,68792:down|Cxcl12,20315:up|Vegfa,22339:down|Mmp2,17390:down|Lrrc15,74488:down|Gas6,14456:up|Egfr,13649:down|Fgfr1,14182:down|Sfrp1,20377:up|Has2,15117:down|Sulf1,240725:down|Tgfbr3,21814:up|Ptgs2,19225:down|Grem1,23892:up|Col3a1,12825:down|Ceacam1,26365:up|Sfrp2,20319:up
organ morphogenesis	GO:0009887	1.10E-15	2.78E-13	Col5a2,12832:down|Flrt2,399558:down|Plxnd1,67784:up|Aldh1a2,19378:up|Pdgfc,54635:down|Mmp13,17386:up|Ankrd1,107765:down|Serpinh1,12406:down|Uchl5,56207:up|Wnt5a,22418:down|Bmpr1a,12166:down|Stc1,20855:up|Col8a1,12837:down|Bmp4,12159:up|Igf1,16000:up|Chst11,58250:down|Tnc,21923:down|Tgfb2,21808:down|Aldh1a3,56847:down|Fgfr2,14183:up|Fam101b,76566:down|Cthrc1,68588:down|Htr2b,15559:down|Ptk7,71461:down|Inhba,16323:down|Bnc2,242509:down|Aspn,66695:down|Vdr,22337:down|Vegfa,22339:down|Mmp2,17390:down|Igf2,16002:up|Slc40a1,53945:up|Tnf,21926:up|Col1a2,12843:down|Alpl,11647:up|Col11a1,12814:down|Egfr,13649:down|Fgfr1,14182:down|Sfrp1,20377:up|Has2,15117:down|Sulf1,240725:down|Tgfbr3,21814:up|Grem1,23892:up|Col3a1,12825:down|Ceacam1,26365:up|Sfrp2,20319:up|Tbx15,21384:down|Col5a1,12831:down|Rspo2,239405:up|Fam20a,208659:down|Tnfrsf11b,18383:up|Npnt,114249:up|Cdsn,386463:up|Errfi1,74155:down

<table>
<tr><td>circulatory system development</td><td>GO:0072359</td><td>1.15E-15</td><td>2.78E-13</td><td>Adm,11535:up|Flrt2,399558:down|Hpgd,15446:up|Plxnd1,67784:up|Ccl2,20296:up|Aldh1a2,19378:up|C3,12266:up|Figf,14205:up|Mmp13,17386:up|Epas1,13819:up|Ankrd1,107765:down|Tnfrsf12a,27279:down|Ddah1,69219:down|Lyl1,17095:up|Cx3cr1,13051:down|Loxl2,94352:down|Ncam1,17967:down|Wnt5a,22418:down|Bmpr1a,12166:down|Ccl12,20293:up|Wisp1,22402:down|Col8a1,12837:down|Bmp4,12159:up|Igf1,16000:up|Tgfb2,21808:down|Vegfb,22340:up|Hpse,15442:up|Plpp3,67916:up|Fgfr2,14183:up|F3,14066:up|Thbs2,21826:down|Htr2b,15559:down|Prl2c2,18811:up|Ptk7,71461:down|Srpx2,68792:down|Cxcl12,20315:up|Vegfa,22339:down|Mmp2,17390:down|Nxn,18230:down|Anpep,16790:up|Col1a2,12843:down|Fgfr1,14182:down|Col11a1,12814:down|Has2,15117:down|Hmox1,15368:up|Sulf1,240725:down|Tgfbr3,21814:up|Ptgs2,19225:down|Grem1,23892:up|Col3a1,12825:down|Ceacam1,26365:up|Sfrp2,20319:up|Col5a1,12831:down|Errfi1,74155:down</td></tr>
<tr><td>cardiovascular system development</td><td>GO:0072358</td><td>1.15E-15</td><td>2.78E-13</td><td>Adm,11535:up|Flrt2,399558:down|Hpgd,15446:up|Plxnd1,67784:up|Ccl2,20296:up|Aldh1a2,19378:up|C3,12266:up|Figf,14205:up|Mmp13,17386:up|Epas1,13819:up|Ankrd1,107765:down|Tnfrsf12a,27279:down|Ddah1,69219:down|Lyl1,17095:up|Cx3cr1,13051:down|Loxl2,94352:down|Ncam1,17967:down|Wnt5a,22418:down|Bmpr1a,12166:down|Ccl12,20293:up|Wisp1,22402:down|Col8a1,12837:down|Bmp4,12159:up|Igf1,16000:up|Tgfb2,21808:down|Vegfb,22340:up|Hpse,15442:up|Plpp3,67916:up|Fgfr2,14183:up|F3,14066:up|Thbs2,21826:down|Htr2b,15559:down|Prl2c2,18811:up|Ptk7,71461:down|Srpx2,68792:down|Cxcl12,20315:up|Vegfa,22339:down|Mmp2,17390:down|Nxn,18230:down|Anpep,16790:up|Col1a2,12843:down|Fgfr1,14182:down|Col11a1,12814:down|Has2,15117:down|Hmox1,15368:up|Sulf1,240725:down|Tgfbr3,21814:up|Ptgs2,19225:down|Grem1,23892:up|Col3a1,12825:down|Ceacam1,26365:up|Sfrp2,20319:up|Col5a1,12831:down|Errfi1,74155:down</td></tr>
<tr><td>locomotion</td><td>GO:0040011</td><td>1.19E-15</td><td>2.78E-13</td><td>Flrt2,399558:down|Plxnd1,67784:up|Cx3cr1,13051:down|Igfbp3,16009:up|Loxl2,94352:down|Trf,22041:up|Cxcl14,57266:down|Cd24a,12484:down|Ceacam10,26366:up|Wnt5a,22418:down|Mark1,226778:up|Stc1,20855:up|Il1rn,16181:up|Adipoq,11450:up|Bmp4,12159:up|Igf1,16000:up|Cxcl9,17329:up|Tgfb2,21808:down|F3,14066:up|Cthrc1,68588:down|Htr2b,15559:down|Prl2c2,18811:up|Efemp1,216616:up|Ccl6,20305:up|Dpysl3,22240:down|Saa3,20210:up|Cxcr6,80901:down|Cxcl12,20315:up|Mmp2,17390:down|Gas6,14456:up|Egfr,13649:down|Sfrp1,20377:up|Tgfbr3,21814:up|Ceacam1,26365:up|Ccl8,20307:up|Sfrp2,20319:up|Ccl2,20296:up|Ccl7,20306:up|Ednrb,13618:up|Figf,14205:up|Pdgfc,54635:down|Sema7a,20361:down|Sord,20322:up|Tnfrsf12a,27279:down|Postn,50706:down|Ptx3,19288:up|Ccl12,20293:up|Bmpr1a,12166:down|Ccl4,20303:up|Ret,19713:up|Plpp3,67916:up|Kitl,17311:up|Ccl9,20308:up|Lama2,16773:down|Ptk7,71461:down|Ccl3,20302:up|Plat,18791:down|Srpx2,68792:down|Vegfa,22339:down|Adamts12,239337:down|Lrrc15,74488:down|Tnf,21926:up|Fgfr1,14182:down|Has2,15117:down|Sulf1,240725:down|Ptgs2,19225:down|Grem1,23892:up|Col3a1,12825:down|Col5a1,12831:down</td></tr>
<tr><td>extracellular matrix organization</td><td>GO:0030198</td><td>1.86E-15</td><td>4.18E-13</td><td>P4ha1,18451:down|Col5a2,12832:down|Flrt2,399558:down|Mmp13,17386:up|Ltbp2,16997:down|Serpinh1,12406:down|Gpm6b,14758:up|Postn,50706:down|Ibsp,15891:up|Smoc2,64074:down|Tnf,21926:up|Loxl2,94352:down|Col1a2,12843:down|Col11a1,12814:down|Has2,15117:down|Sulf1,240725:down|Grem1,23892:up|Col3a1,12825:down|Sfrp2,20319:up|Pxdn,69675:down|Col5a1,12831:down|Tgfb2,21808:down|Hpse,15442:up|Tnfrsf11b,18383:up|Npnt,114249:up|Creb3l1,26427:down</td></tr>
<tr><td>extracellular structure organization</td><td>GO:0043062</td><td>2.11E-15</td><td>4.57E-13</td><td>P4ha1,18451:down|Col5a2,12832:down|Flrt2,399558:down|Mmp13,17386:up|Ltbp2,16997:down|Serpinh1,12406:down|Gpm6b,14758:up|Postn,50706:down|Ibsp,15891:up|Smoc2,64074:down|Tnf,21926:up|Loxl2,94352:down|Col1a2,12843:down|Col11a1,12814:down|Has2,15117:down|Sulf1,240725:down|Grem1,23892:up|Col3a1,12825:down|Sfrp2,20319:up|Pxdn,69675:down|Col5a1,12831:down|Tgfb2,21808:down|Hpse,15442:up|Tnfrsf11b,18383:up|Npnt,114249:up|Creb3l1,26427:down</td></tr>
<tr><td>response to organic substance</td><td>GO:0010033</td><td>2.86E-15</td><td>5.99E-13</td><td>Fstl3,83554:down|Adm,11535:up|Flrt2,399558:down|Hpgd,15446:up|Ankrd1,107765:down|Cx3cr1,13051:down|Ibsp,15891:up|Cd109,235505:down|Cd24a,12484:down|Mme,17380:up|Wnt5a,22418:down|Il1rn,16181:up|Igf1,16000:up|Adipoq,11450:up|Bmp4,12159:up|Cxcl9,17329:up|Tgfb2,21808:down|Ptgir,19222:up|F3,14066:up|Fgfr2,14183:up|Htr2b,15559:down|Ccl6,20305:up|Slpi,20568:up|Aspn,66695:down|Saa3,20210:up|Vdr,22337:down|Fabp4,11770:up|Cxcl12,20315:up|Htr2a,15558:down|Mmp2,17390:down|Pappa,18491:down|Gas6,14456:up|Egfr,13649:down|Alpl,11647:up|Sfrp1,20377:up|Tgfbr3,21814:up|Ceacam1,26365:up|Ccl8,20307:up|Sfrp2,20319:up|Serpina3g,20715:up|Npnt,114249:up|Tnfrsf11b,18383:up|Col5a2,12832:down|Mrc1,17533:up|Aldh1a2,19378:up|Ccl2,20296:up|Ccl7,20306:up|Pdgfc,54635:down|Ednrb,13618:up|Figf,14205:up|Ifit3,15959:up|Htra3,78558:down|Sord,20322:up|Postn,50706:down|Cdo1,12583:up|Akr1c18,105349:up|Ncam1,17967:down|Ccl12,20293:up|Bmpr1a,12166:down|Ccl4,20303:up|Chst11,58250:down|Gatm,67092:down|Ret,19713:up|Sdf2l1,64136:up|Ifit1,15957:up|Creb3l1,26427:down|Ccl9,20308:up|Ptk7,71461:down|Inhba,16323:down|Ccl3,20302:up|Vegfa,22339:down|Adamts12,239337:down|Lrrc15,74488:down|Igf2,16002:up|Pparg,19016:up|Tnf,21926:up|Col1a2,12843:down|Glp1r,14652:down|Fgfr1,14182:down|Hmox1,15368:up|Has2,15117:down|Sulf1,240725:down|Ptgs2,19225:down|Grem1,23892:up|Col3a1,12825:down|Enpp1,18605:down</td></tr>
<tr><td>blood vessel morphogenesis</td><td>GO:0048514</td><td>6.18E-15</td><td>1.21E-12</td><td>Adm,11535:up|Hpgd,15446:up|Plxnd1,67784:up|Ccl2,20296:up|C3,12266:up|Figf,14205:up|Epas1,13819:up|Tnfrsf12a,27279:down|Ddah1,69219:down|Cx3cr1,13051:down|Loxl2,94352:down|Wnt5a,22418:down|Bmpr1a,12166:down|Ccl12,20293:up|Col8a1,12837:down|Bmp4,12159:up|Tgfb2,21808:down|Vegfb,22340:up|Hpse,15442:up|Fgfr2,14183:up|F3,14066:up|Thbs2,21826:down|Prl2c2,18811:up|Srpx2,68792:down|Cxcl12,20315:up|Vegfa,22339:down|Mmp2,17390:down|Anpep,16790:up|Fgfr1,14182:down|Hmox1,15368:up|Has2,15117:down|Sulf1,240725:down|Tgfbr3,21814:up|Ptgs2,19225:down|Grem1,23892:up|Col3a1,12825:down|Ceacam1,26365:up|Sfrp2,20319:up</td></tr>
<tr><td>regulation of locomotion</td><td>GO:0040012</td><td>6.75E-15</td><td>1.21E-12</td><td>Flrt2,399558:down|Plxnd1,67784:up|Ccl2,20296:up|Pdgfc,54635:down|Figf,14205:up|Sema7a,20361:down|Postn,50706:down|Cx3cr1,13051:down|Igfbp3,16009:up|Trf,22041:up|Ptx3,19288:up|Cxcl14,57266:down|Wnt5a,22418:down|Bmpr1a,12166:down|Ccl12,20293:up|Stc1,20855:up|Il1rn,16181:up|Ccl4,20303:up|Adipoq,11450:up|Bmp4,12159:up|Igf1,16000:up|Cxcl9,17329:up|Ret,19713:up|Tgfb2,21808:down|Plpp3,67916:up|F3,14066:up|Kitl,17311:up|Prl2c2,18811:up|Lama2,16773:down|Efemp1,216616:up|Dpysl3,22240:down|Srpx2,68792:down|Cxcl12,20315:up|Vegfa,22339:down|Mmp2,17390:down|Lrrc15,74488:down|Gas6,14456:up|Egfr,13649:down|Fgfr1,14182:down|Has2,15117:down|Sfrp1,20377:up|Sulf1,240725:down|Tgfbr3,21814:up|Ptgs2,19225:down|Grem1,23892:up|Col3a1,12825:down|Ceacam1,26365:up|Sfrp2,20319:up</td></tr>
</table>

positive regulation of cell proliferation	GO:0008284	6.77E-15	1.21E-12	Adm,11535:up|Ccl2,20296:up|Aldh1a2,19378:up|Pdgfc,54635:down|Figf,14205:up|Ednrb,13618:up|Cx3cr1,13051:down|Akr1c18,105349:up|Trf,22041:up|Cd24a,12484:down|Ncam1,17967:down|Wnt5a,22418:down|Bmpr1a,12166:down|Wisp1,22402:down|Bmp4,12159:up|Igf1,16000:up|Tnc,21923:down|Tgfb2,21808:down|Hpse,15442:up|Fgfr2,14183:up|F3,14066:up|Kitl,17311:up|Cthrc1,68588:down|Htr2b,15559:down|Prl2c2,18811:up|Efemp1,216616:up|Fabp4,11770:up|Crlf1,12931:down|Cxcl12,20315:up|Vegfa,22339:down|Htr2a,15558:down|Mmp2,17390:down|Igf2,16002:up|Gas6,14456:up|Tnf,21926:up|Glp1r,14652:down|Fgfr1,14182:down|Egfr,13649:down|Has2,15117:down|Hmox1,15368:up|Sfrp1,20377:up|Cd4,12504:up|Tgfbr3,21814:up|Ptgs2,19225:down|Tfrc,22042:up|Grem1,23892:up|Ceacam1,26365:up|Sfrp2,20319:up|Tnfsf9,21950:up
anatomical structure morphogenesis	GO:0009653	8.91E-15	1.55E-12	Adm,11535:up|Flrt2,399558:down|Hpgd,15446:up|Plxnd1,67784:up|C3,12266:up|Ankrd1,107765:down|Serpinh1,12406:down|Cx3cr1,13051:down|Cd109,235505:down|Loxl2,94352:down|Loxl3,16950:down|Wnt5a,22418:down|Stc1,20855:up|Il1rn,16181:up|Col8a1,12837:down|Adipoq,11450:up|Bmp4,12159:up|Igf1,16000:up|Crabp2,12904:down|Cxcl9,17329:up|Tgfb2,21808:down|Hpse,15442:up|Fgfr2,14183:up|F3,14066:up|Thbs2,21826:down|Cthrc1,68588:down|Htr2b,15559:down|Prl2c2,18811:up|Gjb5,14622:down|Aspn,66695:down|Ninj1,18081:up|Vdr,22337:down|Cxcl12,20315:up|Mmp2,17390:down|Slc40a1,53945:up|Egfr,13649:down|Col11a1,12814:down|Alpl,11647:up|Sfrp1,20377:up|Tgfbr3,21814:up|Ceacam1,26365:up|Ccl8,20307:up|Sfrp2,20319:up|Rspo2,239405:up|Fam20a,208659:down|Cdsn,386463:up|Npnt,114249:up|Tnfrsf11b,18383:up|Col5a2,12832:down|Aldh1a2,19378:up|Ccl2,20296:up|Pdgfc,54635:down|Figf,14205:up|Mgp,17313:up|Mmp13,17386:up|Sema7a,20361:down|Epas1,13819:up|Tnfrsf12a,27279:down|Uchl5,56207:up|Ddah1,69219:down|Postn,50706:down|Ccl12,20293:up|Bmpr1a,12166:down|Chst11,58250:down|Tnc,21923:down|Ret,19713:up|Vegfb,22340:up|Aldh1a3,56847:down|Plpp3,67916:up|Fam101b,76566:down|Lama2,16773:down|Ptk7,71461:down|Inhba,16323:down|Bnc2,242509:down|Ccl3,20302:up|Srpx2,68792:down|Vegfa,22339:down|Serpine2,20720:up|Adamts12,239337:down|Igf2,16002:up|Anpep,16790:up|Tnf,21926:up|Col1a2,12843:down|Fgfr1,14182:down|Hmox1,15368:up|Has2,15117:down|Sulf1,240725:down|Ptgs2,19225:down|Grem1,23892:up|Col3a1,12825:down|Tbx15,21384:down|Col5a1,12831:down|Errfi1,74155:down
positive regulation of response to stimulus	GO:0048584	9.66E-15	1.61E-12	Igfbp4,16010:up|Sfrp4,20379:up|C3,12266:up|Ankrd1,107765:down|Igfbp3,16009:up|Plk2,20620:down|Trf,22041:up|Cxcl14,57266:down|Cd24a,12484:down|Wnt5a,22418:down|Il1rn,16181:up|Adipoq,11450:up|Bmp4,12159:up|Igf1,16000:up|Cxcl9,17329:up|Tgfb2,21808:down|Hpse,15442:up|Ptgir,19222:up|Fgfr2,14183:up|F3,14066:up|Htr2b,15559:down|Ccl6,20305:up|Fabp4,11770:up|Cxcl12,20315:up|Htr2a,15558:down|Mmp2,17390:down|Gas6,14456:up|Egfr,13649:down|Sfrp1,20377:up|C1qtnf3,81799:down|Tgfbr3,21814:up|C1rb,667277:up|Tfrc,22042:up|Ceacam1,26365:up|Ccl8,20307:up|Sfrp2,20319:up|Rspo2,239405:up|Npnt,114249:up|Tnfrsf11b,18383:up|Ccl2,20296:up|Ccl7,20306:up|Pdgfc,54635:down|Figf,14205:up|Sema7a,20361:down|Tnfrsf12a,27279:down|Clec4n,56620:up|Akr1c18,105349:up|Ncam1,17967:down|Ccl12,20293:up|Bmpr1a,12166:down|Ccl4,20303:up|C1s2,317677:up|Aldh1a3,56847:down|Plpp3,67916:up|Kitl,17311:up|Il1rl1,17082:down|Ccl9,20308:up|Inhba,16323:down|Ccl3,20302:up|C1s1,50908:up|Crlf1,12931:down|Vegfa,22339:down|Igf2,16002:up|Tnf,21926:up|Lmcd1,30937:up|Fgfr1,14182:down|Hmox1,15368:up|Sulf1,240725:down|Cd4,12504:up|Ptgs2,19225:down|Grem1,23892:up|Col3a1,12825:down|Serping1,12258:up
tissue development	GO:0009888	9.81E-15	1.61E-12	Adm,11535:up|Plxnd1,67784:up|Sfrp4,20379:up|Ankrd1,107765:down|Serpinh1,12406:down|Ibsp,15891:up|Cd109,235505:down|Loxl2,94352:down|Loxl3,16950:down|Wnt5a,22418:down|Stc1,20855:up|Adipoq,11450:up|Bmp4,12159:up|Igf1,16000:up|Tgfb2,21808:down|Hpse,15442:up|Fgfr2,14183:up|Cthrc1,68588:down|Htr2b,15559:down|Efemp1,216616:up|Gjb5,14622:down|Aspn,66695:down|Ninj1,18081:up|Vdr,22337:down|Cxcl12,20315:up|Mmp2,17390:down|Slc40a1,53945:up|Gas6,14456:up|Egfr,13649:down|Col11a1,12814:down|Sfrp1,20377:up|Tgfbr3,21814:up|Ceacam1,26365:up|Sfrp2,20319:up|Rspo2,239405:up|Fam20a,208659:down|Npnt,114249:up|Col5a2,12832:down|Aldh1a2,19378:up|Ednrb,13618:up|Mgp,17313:up|Mmp13,17386:up|Sema7a,20361:down|Gpm6b,14758:up|Postn,50706:down|Ncam1,17967:down|Bmpr1a,12166:down|Wisp1,22402:down|Chst11,58250:down|Ank,11732:down|Tnc,21923:down|Ret,19713:up|Aldh1a3,56847:down|Kitl,17311:up|Fam101b,76566:down|Lrrc17,74511:down|Ptk7,71461:down|Inhba,16323:down|Bnc2,242509:down|Crlf1,12931:down|Vegfa,22339:down|Serpine2,20720:up|Adamts12,239337:down|Tnf,21926:up|Pparg,19016:up|Fgfr1,14182:down|Has2,15117:down|Sulf1,240725:down|Ptgs2,19225:down|Grem1,23892:up|Col3a1,12825:down|Mcoln3,171166:up|Col5a1,12831:down|Errfi1,74155:down|Enpp1,18605:down
regulation of cellular component movement	GO:0051270	1.02E-14	1.63E-12	Flrt2,399558:down|Plxnd1,67784:up|Ccl2,20296:up|Pdgfc,54635:down|Figf,14205:up|Sema7a,20361:down|Postn,50706:down|Cx3cr1,13051:down|Igfbp3,16009:up|Trf,22041:up|Cxcl14,57266:down|Wnt5a,22418:down|Bmpr1a,12166:down|Ccl12,20293:up|Stc1,20855:up|Il1rn,16181:up|Ccl4,20303:up|Adipoq,11450:up|Bmp4,12159:up|Igf1,16000:up|Cxcl9,17329:up|Ret,19713:up|Tgfb2,21808:down|Plpp3,67916:up|F3,14066:up|Kitl,17311:up|Prl2c2,18811:up|Lama2,16773:down|Efemp1,216616:up|Dpysl3,22240:down|Srpx2,68792:down|Cxcl12,20315:up|Vegfa,22339:down|Mmp2,17390:down|Lrrc15,74488:down|Gas6,14456:up|Fxyd1,56188:up|Egfr,13649:down|Fgfr1,14182:down|Has2,15117:down|Sfrp1,20377:up|Sulf1,240725:down|Tgfbr3,21814:up|Ptgs2,19225:down|Grem1,23892:up|Col3a1,12825:down|Ceacam1,26365:up|Sfrp2,20319:up
immune response	GO:0006955	1.07E-14	1.67E-12	Clec2d,93694:up|Adm,11535:up|Ly9,17085:up|Mrc1,17533:up|Ccl7,20306:up|Ccl2,20296:up|C3,12266:up|Ifit3,15959:up|Sema7a,20361:down|Clec4n,56620:up|Cx3cr1,13051:down|Enpp3,209558:down|Ptx3,19288:up|Cxcl14,57266:down|Cd24a,12484:down|Wnt5a,22418:down|Bmpr1a,12166:down|Ccl12,20293:up|Ccl4,20303:up|Cxcl9,17329:up|Oasl1,231655:up|Prg4,96875:down|Tgfb2,21808:down|C1s2,317677:up|Ifit1,15957:up|Il1rl1,17082:down|Clec4e,56619:up|Ccl9,20308:up|Ccl6,20305:up|Slpi,20568:up|Ccl3,20302:up|C1s1,50908:up|Cxcl12,20315:up|Vegfa,22339:down|Phf11a,219131:up|Mmp2,17390:down|Oas3,246727:up|Igf2,16002:up|Pparg,19016:up|Gas6,14456:up|Tnf,21926:up|Hmox1,15368:up|Susd2,71733:up|Cd4,12504:up|Tgfbr3,21814:up|C1rb,667277:up|Tfrc,22042:up|Col3a1,12825:down|Ccl8,20307:up|Ceacam1,26365:up|Serping1,12258:up|Serpina3g,20715:up|Tnfrsf11b,18383:up|Enpp1,18605:down|Tnfsf9,21950:up

immune system process	GO:0002376	1.16E-14	1.72E-12	Fstl3,83554:down|Clec2d,93694:up|Adm,11535:up|Tmem176b,65963:up|C3,12266:up|Lyl1,17095:up|Cx3cr1,13051:down|Cd109,235505:down|Trf,22041:up|Cxcl14,57266:down|Cd24a,12484:down|Wnt5a,22418:down|Igf1,16000:up|Adipoq,11450:up|Bmp4,12159:up|Cxcl9,17329:up|Oasl1,231655:up|Tgfb2,21808:down|Fgfr2,14183:up|Clec4e,56619:up|Ccl6,20305:up|Slpi,20568:up|Cxcl12,20315:up|Phf11a,219131:up|Mmp2,17390:down|Oas3,246727:up|Slc40a1,53945:up|Gas6,14456:up|Sfrp1,20377:up|Tgfbr3,21814:up|C1rb,667277:up|Tfrc,22042:up|Ceacam1,26365:up|Ccl8,20307:up|Sfrp2,20319:up|Serpina3g,20715:up|Tnfrsf11b,18383:up|Ly9,17085:up|Mrc1,17533:up|Ccl2,20296:up|Ccl7,20306:up|Ednrb,13618:up|Figf,14205:up|Ampd3,11717:up|Ifit3,15959:up|Sema7a,20361:down|Epas1,13819:up|Clec4n,56620:up|Enpp3,209558:down|Ptx3,19288:up|Ccl12,20293:up|Bmpr1a,12166:down|Ccl4,20303:up|Ret,19713:up|Prg4,96875:down|C1s2,317677:up|Ifit1,15957:up|Il1rl1,17082:down|Kitl,17311:up|Lrrc17,74511:down|Hp,15439:up|Ccl9,20308:up|Inhba,16323:down|Ccl3,20302:up|Ctla2a,13024:down|C1s1,50908:up|Vegfa,22339:down|Igf2,16002:up|Pparg,19016:up|Tnf,21926:up|Tmem176a,66058:up|Lmcd1,30937:up|Hmox1,15368:up|Cd4,12504:up|Susd2,71733:up|Grem1,23892:up|Col3a1,12825:down|Serping1,12258:up|Enpp1,18605:down|Tnfsf9,21950:up
multicellular organismal development	GO:0007275	1.43E-14	2.07E-12	Fstl3,83554:down|Adm,11535:up|Flrt2,399558:down|Tmem176b,65963:up|Plxnd1,67784:up|Sfrp4,20379:up|Serpinh1,12406:down|Lyl1,17095:up|Ibsp,15891:up|Plk2,20620:down|Cd24a,12484:down|Il1rn,16181:up|Bmp4,12159:up|Hpse,15442:up|Fgfr2,14183:up|Thbs2,21826:down|Htr2b,15559:down|Gjb5,14622:down|Aspn,66695:down|Mmp2,17390:down|Slc40a1,53945:up|Egfr,13649:down|Alpl,11647:up|Tfrc,22042:up|Rspo2,239405:up|Fam20a,208659:down|Cdsn,386463:up|Col5a2,12832:down|Aldh1a2,19378:up|Ccl2,20296:up|Ednrb,13618:up|Mmp13,17386:up|Tnfrsf12a,27279:down|Uchl5,56207:up|Shroom4,208431:down|Cdo1,12583:up|Csrp2,13008:down|Ncam1,17967:down|Chst11,58250:down|Tmeff1,230157:down|Vegfb,22340:up|Aldh1a3,56847:down|Plpp3,67916:up|Lrrc17,74511:down|Lama2,16773:down|Ptk7,71461:down|Inhba,16323:down|Bnc2,242509:down|Crlf1,12931:down|Vegfa,22339:down|Serpine2,20720:up|Nxn,18230:down|Igf2,16002:up|Tnf,21926:up|Tmem176a,66058:up|Col1a2,12843:down|Fgfr1,14182:down|Sulf1,240725:down|Cd4,12504:up|Col3a1,12825:down|Tbx15,21384:down|Enpp1,18605:down|Clec2d,93694:up|Hpgd,15446:up|C3,12266:up|Ankrd1,107765:down|Cx3cr1,13051:down|Cd109,235505:down|Trf,22041:up|Loxl3,16950:down|Loxl2,94352:down|Cxcl14,57266:down|Wnt5a,22418:down|Stc1,20855:up|Mark1,226778:up|Col8a1,12837:down|Adipoq,11450:up|Igf1,16000:up|Crabp2,12904:down|Tgfb2,21808:down|F3,14066:up|Clec4e,56619:up|Prl2c2,18811:up|Cthrc1,68588:down|Efemp1,216616:up|Dpysl3,22240:down|Ninj1,18081:up|Vdr,22337:down|Cxcl12,20315:up|Gas6,14456:up|Col11a1,12814:down|Sfrp1,20377:up|Tgfbr3,21814:up|Tll1,21892:down|Ceacam1,26365:up|Sfrp2,20319:up|Tnfrsf11b,18383:up|Npnt,114249:up|Ly9,17085:up|Mgp,17313:up|Figf,14205:up|Pdgfc,54635:down|Epas1,13819:up|Sema7a,20361:down|Gpm6b,14758:up|Ddah1,69219:down|Postn,50706:down|Ccl12,20293:up|Bmpr1a,12166:down|Wisp1,22402:down|Ank,11732:down|Tnc,21923:down|Ret,19713:up|Sdf2l1,64136:up|Prg4,96875:down|Creb3l1,26427:down|Kitl,17311:up|Fam101b,76566:down|Hp,15439:up|Ccl3,20302:up|Ctla2a,13024:down|Srpx2,68792:down|Adamts12,239337:down|Anpep,16790:up|Pparg,19016:up|Hmox1,15368:up|Has2,15117:down|Ptgs2,19225:down|Grem1,23892:up|Pcdh19,279653:down|Mcoln3,171166:up|Col5a1,12831:down|Errfi1,74155:down|Tnfsf9,21950:up
positive regulation of signal transduction	GO:0009967	1.47E-14	2.07E-12	Igfbp4,16010:up|Sfrp4,20379:up|Ccl7,20306:up|Ccl2,20296:up|C3,12266:up|Pdgfc,54635:down|Sema7a,20361:down|Ankrd1,107765:down|Tnfrsf12a,27279:down|Clec4n,56620:up|Igfbp3,16009:up|Plk2,20620:down|Akr1c18,105349:up|Trf,22041:up|Cd24a,12484:down|Ncam1,17967:down|Wnt5a,22418:down|Bmpr1a,12166:down|Ccl12,20293:up|Il1rn,16181:up|Ccl4,20303:up|Adipoq,11450:up|Bmp4,12159:up|Igf1,16000:up|Tgfb2,21808:down|Hpse,15442:up|Aldh1a3,56847:down|Ptgir,19222:up|Fgfr2,14183:up|F3,14066:up|Kitl,17311:up|Htr2b,15559:down|Ccl9,20308:up|Ccl6,20305:up|Inhba,16323:down|Ccl3,20302:up|Crlf1,12931:down|Vegfa,22339:down|Htr2a,15558:down|Igf2,16002:up|Gas6,14456:up|Tnf,21926:up|Lmcd1,30937:up|Fgfr1,14182:down|Egfr,13649:down|Hmox1,15368:up|Sfrp1,20377:up|Sulf1,240725:down|C1qtnf3,81799:down|Cd4,12504:up|Tgfbr3,21814:up|Ptgs2,19225:down|Grem1,23892:up|Col3a1,12825:down|Ceacam1,26365:up|Ccl8,20307:up|Sfrp2,20319:up|Rspo2,239405:up|Npnt,114249:up|Tnfrsf11b,18383:up
regulation of cell differentiation	GO:0045595	1.54E-14	2.13E-12	Fstl3,83554:down|Clec2d,93694:up|Adm,11535:up|Flrt2,399558:down|Tmem176b,65963:up|Plxnd1,67784:up|Sfrp4,20379:up|Ankrd1,107765:down|Cx3cr1,13051:down|Igfbp3,16009:up|Cd109,235505:down|Loxl2,94352:down|Plk2,20620:down|Trf,22041:up|Cxcl14,57266:down|Cd24a,12484:down|Wnt5a,22418:down|Adipoq,11450:up|Bmp4,12159:up|Igf1,16000:up|Crabp2,12904:down|Cxcl9,17329:up|Tgfb2,21808:down|Medag,70717:down|Fgfr2,14183:up|Cthrc1,68588:down|Prl2c2,18811:up|Efemp1,216616:up|Dpysl3,22240:down|Vdr,22337:down|Cxcl12,20315:up|Htr2a,15558:down|Gas6,14456:up|Sfrp1,20377:up|Tgfbr3,21814:up|Ccl8,20307:up|Ceacam1,26365:up|Sfrp2,20319:up|Npnt,114249:up|Col5a2,12832:down|Ednrb,13618:up|Sema7a,20361:down|Tnfrsf12a,27279:down|Postn,50706:down|Bmpr1a,12166:down|Wisp1,22402:down|Ret,19713:up|Kitl,17311:up|Fam101b,76566:down|Lrrc17,74511:down|Ccl9,20308:up|Inhba,16323:down|Ptk7,71461:down|Ccl3,20302:up|Ctla2a,13024:down|Vegfa,22339:down|Serpine2,20720:up|Adamts12,239337:down|Tmem176a,66058:up|Tnf,21926:up|Pparg,19016:up|Glp1r,14652:down|Fgfr1,14182:down|Lpl,16956:up|Has2,15117:down|Ptgs2,19225:down|Grem1,23892:up|Col3a1,12825:down|Col5a1,12831:down|Enpp1,18605:down|Errfi1,74155:down|Tnfsf9,21950:up
cellular response to organic substance	GO:0071310	1.76E-14	2.37E-12	Fstl3,83554:down|Flrt2,399558:down|Hpgd,15446:up|Ankrd1,107765:down|Cx3cr1,13051:down|Ibsp,15891:up|Cd109,235505:down|Cd24a,12484:down|Mme,17380:up|Wnt5a,22418:down|Il1rn,16181:up|Adipoq,11450:up|Bmp4,12159:up|Igf1,16000:up|Cxcl9,17329:up|Tgfb2,21808:down|Ptgir,19222:up|Fgfr2,14183:up|F3,14066:up|Htr2b,15559:down|Ccl6,20305:up|Aspn,66695:down|Saa3,20210:up|Vdr,22337:down|Fabp4,11770:up|Cxcl12,20315:up|Mmp2,17390:down|Gas6,14456:up|Egfr,13649:down|Alpl,11647:up|Sfrp1,20377:up|Tgfbr3,21814:up|Ceacam1,26365:up|Ccl8,20307:up|Sfrp2,20319:up|Npnt,114249:up|Col5a2,12832:down|Mrc1,17533:up|Aldh1a2,19378:up|Ccl2,20296:up|Ccl7,20306:up|Figf,14205:up|Pdgfc,54635:down|Ifit3,15959:up|Htra3,78558:down|Akr1c18,105349:up|Ccl12,20293:up|Bmpr1a,12166:down|Ccl4,20303:up|Chst11,58250:down|Ret,19713:up|Sdf2l1,64136:up|Ifit1,15957:up|Creb3l1,26427:down|Ccl9,20308:up|Inhba,16323:down|Ptk7,71461:down|Ccl3,20302:up|Vegfa,22339:down|Adamts12,239337:down|Igf2,16002:up|Lrrc15,74488:down|Pparg,19016:up|Tnf,21926:up|Col1a2,12843:down|Fgfr1,14182:down|Has2,15117:down|Sulf1,240725:down|Grem1,23892:up|Col3a1,12825:down|Enpp1,18605:down

regulation of cell proliferation	GO:0042127	1.95E-14	2.57E-12	Adm,11535:up|Sfrp4,20379:up|Cx3cr1,13051:down|Igfbp3,16009:up|Cd109,235505:down|Trf,22041:up|Cd24a,12484:down|Wnt5a,22418:down|Adipoq,11450:up|Bmp4,12159:up|Igf1,16000:up|Cxcl9,17329:up|Tgfb2,21808:down|Hpse,15442:up|Ptgir,19222:up|Fgfr2,14183:up|F3,14066:up|Cthrc1,68588:down|Htr2b,15559:down|Prl2c2,18811:up|Efemp1,216616:up|Vdr,22337:down|Fabp4,11770:up|Cxcl12,20315:up|Htr2a,15558:down|Mmp2,17390:down|Gas6,14456:up|Egfr,13649:down|Sfrp1,20377:up|Tgfbr3,21814:up|Tfrc,22042:up|Ceacam1,26365:up|Sfrp2,20319:up|Tnfrsf11b,18383:up|Aldh1a2,19378:up|Ccl2,20296:up|Ednrb,13618:up|Figf,14205:up|Pdgfc,54635:down|Akr1c18,105349:up|Ncam1,17967:down|Ccl12,20293:up|Bmpr1a,12166:down|Wisp1,22402:down|Chst11,58250:down|Tnc,21923:down|Prg4,96875:down|Kitl,17311:up|Il1rl1,17082:down|Inhba,16323:down|Crlf1,12931:down|Vegfa,22339:down|Serpine2,20720:up|Igf2,16002:up|Tnf,21926:up|Pparg,19016:up|Glp1r,14652:down|Fgfr1,14182:down|Hmox1,15368:up|Has2,15117:down|Sulf1,240725:down|Cd4,12504:up|Ptgs2,19225:down|Grem1,23892:up|Errfi1,74155:down|Tnfsf9,21950:up
response to cytokine	GO:0034097	2.24E-14	2.90E-12	Flrt2,399558:down|Mrc1,17533:up|Ccl7,20306:up|Ccl2,20296:up|Aldh1a2,19378:up|Ifit3,15959:up|Ankrd1,107765:down|Cx3cr1,13051:down|Cd24a,12484:down|Mme,17380:up|Wnt5a,22418:down|Ccl12,20293:up|Il1rn,16181:up|Ccl4,20303:up|Adipoq,11450:up|Cxcl9,17329:up|Ifit1,15957:up|F3,14066:up|Ccl9,20308:up|Ccl6,20305:up|Aspn,66695:down|Ccl3,20302:up|Saa3,20210:up|Fabp4,11770:up|Cxcl12,20315:up|Adamts12,239337:down|Lrrc15,74488:down|Pparg,19016:up|Gas6,14456:up|Tnf,21926:up|Sfrp1,20377:up|Ptgs2,19225:down|Col3a1,12825:down|Ccl8,20307:up|Ceacam1,26365:up|Serpina3g,20715:up|Npnt,114249:up
single-multicellular organism process	GO:0044707	2.89E-14	3.66E-12	Fstl3,83554:down|Adm,11535:up|Flrt2,399558:down|Tmem176b,65963:up|Plxnd1,67784:up|Sfrp4,20379:up|Serpinh1,12406:down|Lyl1,17095:up|Igfbp3,16009:up|Ibsp,15891:up|Plk2,20620:down|Cd24a,12484:down|Mme,17380:up|Il1rn,16181:up|Bmp4,12159:up|Hpse,15442:up|Fgfr2,14183:up|Thbs2,21826:down|Htr2b,15559:down|Gjb5,14622:down|Anxa8,11752:down|Aspn,66695:down|Mmp2,17390:down|Slc40a1,53945:up|Egfr,13649:down|Alpl,11647:up|Tfrc,22042:up|Sncg,20618:up|Rspo2,239405:up|Fam20a,208659:down|Cdsn,386463:up|Col5a2,12832:down|Aldh1a2,19378:up|Ccl2,20296:up|Ednrb,13618:up|Ampd3,11717:up|Mmp13,17386:up|Tnfrsf12a,27279:down|Uchl5,56207:up|Clec4n,56620:up|Shroom4,208431:down|Cdo1,12583:up|Csrp2,13008:down|Ncam1,17967:down|Ccl4,20303:up|Chst11,58250:down|Tmeff1,230157:down|Vegfb,22340:up|Aldh1a3,56847:down|Plpp3,67916:up|Mrc2,17534:down|Lrrc17,74511:down|Lama2,16773:down|Ptk7,71461:down|Inhba,16323:down|Bnc2,242509:down|Tfpi2,21789:down|Crlf1,12931:down|Vegfa,22339:down|Serpine2,20720:up|Nxn,18230:down|Igf2,16002:up|Tmem176a,66058:up|Tnf,21926:up|Col1a2,12843:down|Fxyd1,56188:up|Fgfr1,14182:down|Lpl,16956:up|Sulf1,240725:down|Cd4,12504:up|Col3a1,12825:down|Tbx15,21384:down|Serping1,12258:up|Enpp1,18605:down|Clec2d,93694:up|Hpgd,15446:up|C3,12266:up|Ankrd1,107765:down|Cx3cr1,13051:down|Cd109,235505:down|Loxl2,94352:down|Trf,22041:up|Loxl3,16950:down|Cxcl14,57266:down|Wnt5a,22418:down|Stc1,20855:up|Mark1,226778:up|Col8a1,12837:down|Adipoq,11450:up|Igf1,16000:up|Crabp2,12904:down|Tgfb2,21808:down|F3,14066:up|Clec4e,56619:up|Cthrc1,68588:down|Kng1,16644:up|Prl2c2,18811:up|Efemp1,216616:up|Dpysl3,22240:down|Ninj1,18081:up|Vdr,22337:down|Fabp4,11770:up|Cxcl12,20315:up|Htr2a,15558:down|Gas6,14456:up|Col11a1,12814:down|Sfrp1,20377:up|C1qtnf3,81799:down|Tgfbr3,21814:up|Tll1,21892:down|Ceacam1,26365:up|Sfrp2,20319:up|Tnfrsf11b,18383:up|Prkar2b,19088:up|Npnt,114249:up|S100a1,20193:up|Ly9,17085:up|Pdgfc,54635:down|Mgp,17313:up|Figf,14205:up|Epas1,13819:up|Slc29a1,63959:up|Sema7a,20361:down|Gpm6b,14758:up|Ddah1,69219:down|Postn,50706:down|Ccl12,20293:up|Bmpr1a,12166:down|Wisp1,22402:down|Ank,11732:down|Tnc,21923:down|Ret,19713:up|Prg4,96875:down|Sdf2l1,64136:up|Creb3l1,26427:down|Kitl,17311:up|Il1rl1,17082:down|Fam101b,76566:down|Hp,15439:up|Ccl3,20302:up|Plat,18791:down|Ctla2a,13024:down|Srpx2,68792:down|Adamts12,239337:down|Anpep,16790:up|Pparg,19016:up|Lmcd1,30937:up|Glp1r,14652:down|Hmox1,15368:up|Has2,15117:down|Ptgs2,19225:down|Grem1,23892:up|Pcdh19,279653:down|Mcoln3,171166:up|Col5a1,12831:down|Errfi1,74155:down|Tnfsf9,21950:up
positive regulation of signaling	GO:0023056	3.59E-14	4.36E-12	Igfbp4,16010:up|Sfrp4,20379:up|C3,12266:up|Ankrd1,107765:down|Cx3cr1,13051:down|Igfbp3,16009:up|Plk2,20620:down|Trf,22041:up|Cd24a,12484:down|Wnt5a,22418:down|Il1rn,16181:up|Adipoq,11450:up|Bmp4,12159:up|Igf1,16000:up|Tgfb2,21808:down|Hpse,15442:up|Ptgir,19222:up|Fgfr2,14183:up|F3,14066:up|Htr2b,15559:down|Ccl6,20305:up|Htr2a,15558:down|Gas6,14456:up|Egfr,13649:down|Sfrp1,20377:up|C1qtnf3,81799:down|Tgfbr3,21814:up|Ceacam1,26365:up|Ccl8,20307:up|Sfrp2,20319:up|Rspo2,239405:up|Npnt,114249:up|Tnfrsf11b,18383:up|Ccl2,20296:up|Ccl7,20306:up|Pdgfc,54635:down|Sema7a,20361:down|Tnfrsf12a,27279:down|Clec4n,56620:up|Akr1c18,105349:up|Ncam1,17967:down|Ccl12,20293:up|Bmpr1a,12166:down|Ccl4,20303:up|Aldh1a3,56847:down|Kitl,17311:up|Ccl9,20308:up|Lama2,16773:down|Inhba,16323:down|Ccl3,20302:up|Crlf1,12931:down|Vegfa,22339:down|Serpine2,20720:up|Igf2,16002:up|Tnf,21926:up|Lmcd1,30937:up|Glp1r,14652:down|Fgfr1,14182:down|Hmox1,15368:up|Cd4,12504:up|Sulf1,240725:down|Ptgs2,19225:down|Grem1,23892:up|Col3a1,12825:down
regulation of response to wounding	GO:1903034	3.88E-14	4.62E-12	Ccl7,20306:up|Ccl2,20296:up|C3,12266:up|Ednrb,13618:up|Sema7a,20361:down|Tnfrsf12a,27279:down|Cx3cr1,13051:down|Cd109,235505:down|Cd24a,12484:down|Wnt5a,22418:down|Ccl12,20293:up|Ccl4,20303:up|Adipoq,11450:up|Hpse,15442:up|Plpp3,67916:up|Il1rl1,17082:down|Kng1,16644:up|Ccl9,20308:up|Ccl6,20305:up|Aoah,27052:up|Ccl3,20302:up|Ctla2a,13024:down|Fabp4,11770:up|Serpine2,20720:up|Adamts12,239337:down|Pparg,19016:up|Tnf,21926:up|Egfr,13649:down|C1qtnf3,81799:down|Ptgs2,19225:down|Ccl8,20307:up|Ceacam1,26365:up|Serping1,12258:up
positive regulation of developmental process	GO:0051094	4.00E-14	4.66E-12	Adm,11535:up|Flrt2,399558:down|Sfrp4,20379:up|C3,12266:up|Sema7a,20361:down|Ankrd1,107765:down|Tnfrsf12a,27279:down|Gpm6b,14758:up|Ddah1,69219:down|Cx3cr1,13051:down|Igfbp3,16009:up|Loxl2,94352:down|Trf,22041:up|Cd24a,12484:down|Ncam1,17967:down|Wnt5a,22418:down|Bmpr1a,12166:down|Wisp1,22402:down|Adipoq,11450:up|Bmp4,12159:up|Igf1,16000:up|Crabp2,12904:down|Cxcl9,17329:up|Ret,19713:up|Tgfb2,21808:down|Hpse,15442:up|Medag,70717:down|Fgfr2,14183:up|F3,14066:up|Thbs2,21826:down|Kitl,17311:up|Cthrc1,68588:down|Prl2c2,18811:up|Ptk7,71461:down|Inhba,16323:down|Dpysl3,22240:down|Ccl3,20302:up|Vdr,22337:down|Srpx2,68792:down|Cxcl12,20315:up|Vegfa,22339:down|Htr2a,15558:down|Serpine2,20720:up|Igf2,16002:up|Gas6,14456:up|Pparg,19016:up|Tnf,21926:up|Glp1r,14652:down|Fgfr1,14182:down|Has2,15117:down|Lpl,16956:up|Hmox1,15368:up|Sfrp1,20377:up|Ptgs2,19225:down|Tfrc,22042:up|Grem1,23892:up|Ccl8,20307:up|Ceacam1,26365:up|Sfrp2,20319:up|Npnt,114249:up|Tnfsf9,21950:up

response to chemical	GO:0042221	4.49E-14	5.14E-12	Fstl3,83554:down|Adm,11535:up|Flrt2,399558:down|Hpgd,15446:up|Plxnd1,67784:up|Ankrd1,107765:down|Gstt1,14871:up|Cx3cr1,13051:down|Ibsp,15891:up|Cd109,235505:down|Loxl2,94352:down|Trf,22041:up|Cxcl14,57266:down|Cd24a,12484:down|Mme,17380:up|Wnt5a,22418:down|Il1rn,16181:up|Igf1,16000:up|Adipoq,11450:up|Bmp4,12159:up|Cxcl9,17329:up|Tgfb2,21808:down|Ptgir,19222:up|F3,14066:up|Fgfr2,14183:up|Htr2b,15559:down|Ccl6,20305:up|Slpi,20568:up|Aspn,66695:down|Mt2,17750:up|Saa3,20210:up|Vdr,22337:down|Cxcr6,80901:down|Fabp4,11770:up|Cxcl12,20315:up|Htr2a,15558:down|Mmp2,17390:down|Pappa,18491:down|Slc40a1,53945:up|Gas6,14456:up|Egfr,13649:down|Alpl,11647:up|Sfrp1,20377:up|Tgfbr3,21814:up|Tfrc,22042:up|Ceacam1,26365:up|Ccl8,20307:up|Sfrp2,20319:up|Gstm1,14862:up|Serpina3g,20715:up|Npnt,114249:up|Tnfrsf11b,18383:up|Mt1,17748:up|Col5a2,12832:down|Mrc1,17533:up|Aldh1a2,19378:up|Ccl2,20296:up|Ccl7,20306:up|Pdgfc,54635:down|Ednrb,13618:up|Figf,14205:up|Ifit3,15959:up|Epas1,13819:up|Htra3,78558:down|Sord,20322:up|Postn,50706:down|Cdo1,12583:up|Hvcn1,74096:up|Akr1c18,105349:up|Ncam1,17967:down|Ccl12,20293:up|Bmpr1a,12166:down|Ccl4,20303:up|Chst11,58250:down|Gatm,67092:down|Ret,19713:up|Sdf2l1,64136:up|Ifit1,15957:up|Creb3l1,26427:down|Lama2,16773:down|Hp,15439:up|Ccl9,20308:up|Ptk7,71461:down|Inhba,16323:down|Ccl3,20302:up|Vegfa,22339:down|Adamts12,239337:down|Lrrc15,74488:down|Igf2,16002:up|Pparg,19016:up|Tnf,21926:up|Glp1r,14652:down|Col1a2,12843:down|Fgfr1,14182:down|Has2,15117:down|Hmox1,15368:up|Sulf1,240725:down|Ptgs2,19225:down|Grem1,23892:up|Col3a1,12825:down|Enpp1,18605:down
cellular response to cytokine stimulus	GO:0071345	5.27E-14	5.92E-12	Flrt2,399558:down|Mrc1,17533:up|Ccl7,20306:up|Ccl2,20296:up|Ifit3,15959:up|Ankrd1,107765:down|Cx3cr1,13051:down|Cd24a,12484:down|Mme,17380:up|Wnt5a,22418:down|Ccl12,20293:up|Il1rn,16181:up|Ccl4,20303:up|Adipoq,11450:up|Cxcl9,17329:up|Ifit1,15957:up|F3,14066:up|Ccl9,20308:up|Ccl6,20305:up|Ccl3,20302:up|Aspn,66695:down|Saa3,20210:up|Fabp4,11770:up|Cxcl12,20315:up|Lrrc15,74488:down|Adamts12,239337:down|Pparg,19016:up|Gas6,14456:up|Tnf,21926:up|Sfrp1,20377:up|Ccl8,20307:up|Ceacam1,26365:up|Npnt,114249:up
positive regulation of MAPK cascade	GO:0043410	6.21E-14	6.85E-12	Igfbp4,16010:up|Ccl7,20306:up|Ccl2,20296:up|C3,12266:up|Pdgfc,54635:down|Sema7a,20361:down|Igfbp3,16009:up|Trf,22041:up|Cd24a,12484:down|Wnt5a,22418:down|Ccl12,20293:up|Il1rn,16181:up|Ccl4,20303:up|Bmp4,12159:up|Igf1,16000:up|Tgfb2,21808:down|Fgfr2,14183:up|Kitl,17311:up|Htr2b,15559:down|Ccl9,20308:up|Ccl6,20305:up|Ccl3,20302:up|Htr2a,15558:down|Vegfa,22339:down|Igf2,16002:up|Gas6,14456:up|Tnf,21926:up|Egfr,13649:down|Fgfr1,14182:down|C1qtnf3,81799:down|Ccl8,20307:up|Ceacam1,26365:up|Tnfrsf11b,18383:up|Npnt,114249:up
positive regulation of cell communication	GO:0010647	6.62E-14	7.17E-12	Igfbp4,16010:up|Sfrp4,20379:up|C3,12266:up|Ankrd1,107765:down|Cx3cr1,13051:down|Igfbp3,16009:up|Plk2,20620:down|Trf,22041:up|Cd24a,12484:down|Wnt5a,22418:down|Il1rn,16181:up|Adipoq,11450:up|Bmp4,12159:up|Igf1,16000:up|Tgfb2,21808:down|Hpse,15442:up|Ptgir,19222:up|Fgfr2,14183:up|F3,14066:up|Htr2b,15559:down|Ccl6,20305:up|Htr2a,15558:down|Gas6,14456:up|Egfr,13649:down|Sfrp1,20377:up|C1qtnf3,81799:down|Tgfbr3,21814:up|Ceacam1,26365:up|Ccl8,20307:up|Sfrp2,20319:up|Rspo2,239405:up|Npnt,114249:up|Tnfrsf11b,18383:up|Ccl2,20296:up|Ccl7,20306:up|Pdgfc,54635:down|Sema7a,20361:down|Tnfrsf12a,27279:down|Clec4n,56620:up|Akr1c18,105349:up|Ncam1,17967:down|Ccl12,20293:up|Bmpr1a,12166:down|Ccl4,20303:up|Aldh1a3,56847:down|Kitl,17311:up|Ccl9,20308:up|Lama2,16773:down|Inhba,16323:down|Ccl3,20302:up|Crlf1,12931:down|Vegfa,22339:down|Serpine2,20720:up|Igf2,16002:up|Tnf,21926:up|Lmcd1,30937:up|Glp1r,14652:down|Fgfr1,14182:down|Hmox1,15368:up|Cd4,12504:up|Sulf1,240725:down|Ptgs2,19225:down|Grem1,23892:up|Col3a1,12825:down
regulation of response to stimulus	GO:0048583	8.58E-14	9.06E-12	Fstl3,83554:down|Igfbp4,16010:up|Clec2d,93694:up|Adm,11535:up|Flrt2,399558:down|Plxnd1,67784:up|Sfrp4,20379:up|C3,12266:up|Ankrd1,107765:down|Cx3cr1,13051:down|Igfbp3,16009:up|Cd109,235505:down|Plk2,20620:down|Trf,22041:up|Cxcl14,57266:down|Cd24a,12484:down|Wnt5a,22418:down|Il1rn,16181:up|Igf1,16000:up|Adipoq,11450:up|Bmp4,12159:up|Crabp2,12904:down|Cxcl9,17329:up|Tgfb2,21808:down|Hpse,15442:up|Ptgir,19222:up|F3,14066:up|Fgfr2,14183:up|Clec4e,56619:up|Cthrc1,68588:down|Htr2b,15559:down|Kng1,16644:up|Ccl6,20305:up|Aspn,66695:down|Fabp4,11770:up|Cxcl12,20315:up|Htr2a,15558:down|Phf11a,219131:up|Mmp2,17390:down|Gas6,14456:up|Egfr,13649:down|Sfrp1,20377:up|C1qtnf3,81799:down|Tgfbr3,21814:up|C1rb,667277:up|Tfrc,22042:up|Ceacam1,26365:up|Ccl8,20307:up|Sfrp2,20319:up|Rspo2,239405:up|Npnt,114249:up|Tnfrsf11b,18383:up|Ccl2,20296:up|Ccl7,20306:up|Pdgfc,54635:down|Ednrb,13618:up|Figf,14205:up|Sema7a,20361:down|Htra3,78558:down|Tnfrsf12a,27279:down|Clec4n,56620:up|Postn,50706:down|Akr1c18,105349:up|Ncam1,17967:down|Ccl12,20293:up|Bmpr1a,12166:down|Wisp1,22402:down|Ccl4,20303:up|Chst11,58250:down|Ret,19713:up|Sdf2l1,64136:up|C1s2,317677:up|Aldh1a3,56847:down|Ifit1,15957:up|Plpp3,67916:up|Creb3l1,26427:down|Il1rl1,17082:down|Kitl,17311:up|Hp,15439:up|Ccl9,20308:up|Inhba,16323:down|Aoah,27052:up|Ccl3,20302:up|Ctla2a,13024:down|Crlf1,12931:down|C1s1,50908:up|Vegfa,22339:down|Serpine2,20720:up|Adamts12,239337:down|Lrrc15,74488:down|Nxn,18230:down|Igf2,16002:up|Pparg,19016:up|Tnf,21926:up|Lmcd1,30937:up|Tnfrsf23,79201:down|Fgfr1,14182:down|Hmox1,15368:up|Sulf1,240725:down|Cd4,12504:up|Ptgs2,19225:down|Grem1,23892:up|Col3a1,12825:down|Serping1,12258:up|Errfi1,74155:down|Enpp1,18605:down
response to stress	GO:0006950	8.65E-14	9.06E-12	Clec2d,93694:up|Adm,11535:up|Tfec,21426:up|Sfrp4,20379:up|C3,12266:up|Ankrd1,107765:down|Cx3cr1,13051:down|Cd109,235505:down|Ggt5,23887:up|Loxl2,94352:down|Trf,22041:up|Cd24a,12484:down|Wnt5a,22418:down|Il1rn,16181:up|Igf1,16000:up|Adipoq,11450:up|Bmp4,12159:up|Cxcl9,17329:up|Oasl1,231655:up|Tgfb2,21808:down|Hpse,15442:up|Ptgir,19222:up|F3,14066:up|Clec4e,56619:up|Adamts4,240913:down|Kng1,16644:up|Ccl6,20305:up|Anxa8,11752:down|Slpi,20568:up|Mt2,17750:up|Ninj1,18081:up|Saa3,20210:up|Cxcr6,80901:down|Pdia5,72599:down|Fabp4,11770:up|Cxcl12,20315:up|Nabp1,109019:down|Mmp2,17390:down|Oas3,246727:up|Gas6,14456:up|Egfr,13649:down|Sfrp1,20377:up|C1qtnf3,81799:down|C1rb,667277:up|Ceacam1,26365:up|Ccl8,20307:up|Sfrp2,20319:up|Pxdn,69675:down|Tnfrsf11b,18383:up|Mt1,17748:up|Ly9,17085:up|Mrc1,17533:up|Ccl2,20296:up|Ccl7,20306:up|Pdgfc,54635:down|Ednrb,13618:up|Ifit3,15959:up|Sema7a,20361:down|Epas1,13819:up|Tnfrsf12a,27279:down|Uchl5,56207:up|Clec4n,56620:up|Hspb7,29818:up|Ptx3,19288:up|Akr1c18,105349:up|Ncam1,17967:down|Bmpr1a,12166:down|Ccl12,20293:up|Ccl4,20303:up|Tnc,21923:down|Ret,19713:up|C1s2,317677:up|Sdf2l1,64136:up|Ifit1,15957:up|Plpp3,67916:up|Creb3l1,26427:down|Il1rl1,17082:down|Hp,15439:up|Ccl9,20308:up|Ptk7,71461:down|Aoah,27052:up|Plat,18791:down|Ccl3,20302:up|Tfpi2,21789:down|Ctla2a,13024:down|C1s1,50908:up|Plod2,26432:down|Vegfa,22339:down|Serpine2,20720:up|Adamts12,239337:down|Igf2,16002:up|Pparg,19016:up|Tnf,21926:up|Glp1r,14652:down|Lmcd1,30937:up|Hmox1,15368:up|Cd4,12504:up|Ptgs2,19225:down|Col3a1,12825:down|Col5a1,12831:down|Serping1,12258:up|Errfi1,74155:down

<table>
<tr><th></th><th></th><th></th><th></th><th></th></tr>
<tr><td>movement of cell or subcellular component</td><td>GO:0006928</td><td>1.36E-13</td><td>1.38E-11</td><td>Flrt2,399558:down|Plxnd1,67784:up|Cx3cr1,13051:down|Igfbp3,16009:up|Loxl2,94352:down|Trf,22041:up|Cxcl14,57266:down|Cd24a,12484:down|Ceacam10,26366:up|Wnt5a,22418:down|Mark1,226778:up|Stc1,20855:up|Il1rn,16181:up|Adipoq,11450:up|Bmp4,12159:up|Igf1,16000:up|Cxcl9,17329:up|Tgfb2,21808:down|F3,14066:up|Cthrc1,68588:down|Htr2b,15559:down|Prl2c2,18811:up|Efemp1,216616:up|Ccl6,20305:up|Dpysl3,22240:down|Saa3,20210:up|Cxcl12,20315:up|Mmp2,17390:down|Gas6,14456:up|Egfr,13649:down|Sfrp1,20377:up|Tgfbr3,21814:up|Ccl8,20307:up|Ceacam1,26365:up|Sfrp2,20319:up|Ccl2,20296:up|Ccl7,20306:up|Ednrb,13618:up|Figf,14205:up|Pdgfc,54635:down|Sema7a,20361:down|Sord,20322:up|Tnfrsf12a,27279:down|Postn,50706:down|Ccl12,20293:up|Bmpr1a,12166:down|Ccl4,20303:up|Ret,19713:up|Plpp3,67916:up|Kitl,17311:up|Ccl9,20308:up|Lama2,16773:down|Ptk7,71461:down|Ccl3,20302:up|Plat,18791:down|Srpx2,68792:down|Vegfa,22339:down|Adamts12,239337:down|Lrrc15,74488:down|Tnf,21926:up|Fxyd1,56188:up|Fgfr1,14182:down|Has2,15117:down|Sulf1,240725:down|Ptgs2,19225:down|Grem1,23892:up|Col3a1,12825:down|Col5a1,12831:down</td></tr>
<tr><td>response to external stimulus</td><td>GO:0009605</td><td>1.37E-13</td><td>1.38E-11</td><td>Adm,11535:up|Flrt2,399558:down|Plxnd1,67784:up|Sfrp4,20379:up|C3,12266:up|Ankrd1,107765:down|Cx3cr1,13051:down|Cd109,235505:down|Cxcl14,57266:down|Cd24a,12484:down|Wnt5a,22418:down|Adipoq,11450:up|Igf1,16000:up|Cxcl9,17329:up|Oasl1,231655:up|Tgfb2,21808:down|Hpse,15442:up|Ptgir,19222:up|Clec4e,56619:up|Adamts4,240913:down|Kng1,16644:up|Ccl6,20305:up|Slpi,20568:up|Saa3,20210:up|Vdr,22337:down|Cxcr6,80901:down|Fabp4,11770:up|Cxcl12,20315:up|Htr2a,15558:down|Mmp2,17390:down|Oas3,246727:up|Gas6,14456:up|Egfr,13649:down|Col11a1,12814:down|C1qtnf3,81799:down|Tfrc,22042:up|Ceacam1,26365:up|Ccl8,20307:up|Sfrp2,20319:up|Npnt,114249:up|Tnfrsf11b,18383:up|Mrc1,17533:up|Ccl2,20296:up|Ccl7,20306:up|Ednrb,13618:up|Figf,14205:up|Ifit3,15959:up|Sema7a,20361:down|Clec4n,56620:up|Postn,50706:down|Ptx3,19288:up|Ccl12,20293:up|Ccl4,20303:up|Gatm,67092:down|Aldh1a3,56847:down|Ifit1,15957:up|Il1rl1,17082:down|Ccl9,20308:up|Hp,15439:up|Lama2,16773:down|Aoah,27052:up|Ccl3,20302:up|Ctla2a,13024:down|Plod2,26432:down|Vegfa,22339:down|Serpine2,20720:up|Adamts12,239337:down|Pparg,19016:up|Tnf,21926:up|Lmcd1,30937:up|Fgfr1,14182:down|Hmox1,15368:up|Cd4,12504:up|Ptgs2,19225:down|Grem1,23892:up|Serping1,12258:up</td></tr>
<tr><td>MAPK cascade</td><td>GO:0000165</td><td>1.56E-13</td><td>1.55E-11</td><td>Igfbp4,16010:up|Sfrp4,20379:up|Ccl7,20306:up|Ccl2,20296:up|C3,12266:up|Pdgfc,54635:down|Sema7a,20361:down|Igfbp3,16009:up|Dok2,13449:up|Trf,22041:up|Cd24a,12484:down|Wnt5a,22418:down|Ccl12,20293:up|Il1rn,16181:up|Ccl4,20303:up|Adipoq,11450:up|Bmp4,12159:up|Igf1,16000:up|Ret,19713:up|Tgfb2,21808:down|Fgfr2,14183:up|Kitl,17311:up|Htr2b,15559:down|Ccl9,20308:up|Ccl6,20305:up|Inhba,16323:down|Ccl3,20302:up|Vegfa,22339:down|Htr2a,15558:down|Igf2,16002:up|Gas6,14456:up|Tnf,21926:up|Egfr,13649:down|Fgfr1,14182:down|Sfrp1,20377:up|C1qtnf3,81799:down|Ccl8,20307:up|Ceacam1,26365:up|Sfrp2,20319:up|Tnfrsf11b,18383:up|Npnt,114249:up|Errfi1,74155:down</td></tr>
<tr><td>signal transduction by protein phosphorylation</td><td>GO:0023014</td><td>1.74E-13</td><td>1.70E-11</td><td>Igfbp4,16010:up|Sfrp4,20379:up|Ccl7,20306:up|Ccl2,20296:up|C3,12266:up|Pdgfc,54635:down|Sema7a,20361:down|Igfbp3,16009:up|Dok2,13449:up|Trf,22041:up|Cd24a,12484:down|Wnt5a,22418:down|Ccl12,20293:up|Il1rn,16181:up|Ccl4,20303:up|Adipoq,11450:up|Bmp4,12159:up|Igf1,16000:up|Ret,19713:up|Tgfb2,21808:down|Fgfr2,14183:up|Kitl,17311:up|Htr2b,15559:down|Ccl9,20308:up|Ccl6,20305:up|Inhba,16323:down|Ccl3,20302:up|Vegfa,22339:down|Htr2a,15558:down|Igf2,16002:up|Gas6,14456:up|Tnf,21926:up|Egfr,13649:down|Fgfr1,14182:down|Sfrp1,20377:up|C1qtnf3,81799:down|Ccl8,20307:up|Ceacam1,26365:up|Sfrp2,20319:up|Tnfrsf11b,18383:up|Npnt,114249:up|Errfi1,74155:down</td></tr>
<tr><td>regulation of MAPK cascade</td><td>GO:0043408</td><td>2.11E-13</td><td>2.03E-11</td><td>Igfbp4,16010:up|Sfrp4,20379:up|Ccl7,20306:up|Ccl2,20296:up|C3,12266:up|Pdgfc,54635:down|Sema7a,20361:down|Igfbp3,16009:up|Trf,22041:up|Cd24a,12484:down|Wnt5a,22418:down|Ccl12,20293:up|Il1rn,16181:up|Ccl4,20303:up|Adipoq,11450:up|Bmp4,12159:up|Igf1,16000:up|Tgfb2,21808:down|Fgfr2,14183:up|Kitl,17311:up|Htr2b,15559:down|Ccl9,20308:up|Ccl6,20305:up|Inhba,16323:down|Ccl3,20302:up|Vegfa,22339:down|Htr2a,15558:down|Igf2,16002:up|Gas6,14456:up|Tnf,21926:up|Egfr,13649:down|Fgfr1,14182:down|Sfrp1,20377:up|C1qtnf3,81799:down|Ccl8,20307:up|Ceacam1,26365:up|Sfrp2,20319:up|Tnfrsf11b,18383:up|Npnt,114249:up|Errfi1,74155:down</td></tr>
<tr><td>defense response</td><td>GO:0006952</td><td>2.32E-13</td><td>2.20E-11</td><td>Clec2d,93694:up|Adm,11535:up|Ly9,17085:up|Mrc1,17533:up|Ccl7,20306:up|Ccl2,20296:up|C3,12266:up|Ednrb,13618:up|Ifit3,15959:up|Sema7a,20361:down|Clec4n,56620:up|Cx3cr1,13051:down|Ggt5,23887:up|Ptx3,19288:up|Cd24a,12484:down|Wnt5a,22418:down|Ccl12,20293:up|Il1rn,16181:up|Ccl4,20303:up|Adipoq,11450:up|Cxcl9,17329:up|Oasl1,231655:up|C1s2,317677:up|Ptgir,19222:up|Ifit1,15957:up|F3,14066:up|Il1rl1,17082:down|Clec4e,56619:up|Adamts4,240913:down|Kng1,16644:up|Hp,15439:up|Ccl9,20308:up|Ccl6,20305:up|Aoah,27052:up|Slpi,20568:up|Ccl3,20302:up|Saa3,20210:up|Ctla2a,13024:down|Cxcr6,80901:down|C1s1,50908:up|Fabp4,11770:up|Mmp2,17390:down|Adamts12,239337:down|Oas3,246727:up|Igf2,16002:up|Tnf,21926:up|Pparg,19016:up|Lmcd1,30937:up|Egfr,13649:down|Hmox1,15368:up|C1qtnf3,81799:down|Cd4,12504:up|Ptgs2,19225:down|C1rb,667277:up|Ceacam1,26365:up|Ccl8,20307:up|Serping1,12258:up|Tnfrsf11b,18383:up</td></tr>
<tr><td>negative regulation of multicellular organismal process</td><td>GO:0051241</td><td>2.42E-13</td><td>2.26E-11</td><td>Fstl3,83554:down|Col5a2,12832:down|Clec2d,93694:up|Adm,11535:up|Tmem176b,65963:up|Ccl2,20296:up|Ednrb,13618:up|Cx3cr1,13051:down|Plk2,20620:down|Cd24a,12484:down|Wnt5a,22418:down|Bmpr1a,12166:down|Stc1,20855:up|Adipoq,11450:up|Bmp4,12159:up|Igf1,16000:up|Prg4,96875:down|Tgfb2,21808:down|Thbs2,21826:down|Il1rl1,17082:down|Fam101b,76566:down|Kng1,16644:up|Lrrc17,74511:down|Efemp1,216616:up|Inhba,16323:down|Dpysl3,22240:down|Aspn,66695:down|Vegfa,22339:down|Mmp2,17390:down|Serpine2,20720:up|Adamts12,239337:down|Anpep,16790:up|Pparg,19016:up|Gas6,14456:up|Tnf,21926:up|Tmem176a,66058:up|Fgfr1,14182:down|Sfrp1,20377:up|Hmox1,15368:up|Sulf1,240725:down|C1qtnf3,81799:down|Tgfbr3,21814:up|Ptgs2,19225:down|Grem1,23892:up|Col3a1,12825:down|Ceacam1,26365:up|Sfrp2,20319:up|Serping1,12258:up|Col5a1,12831:down|Rspo2,239405:up|Tnfrsf11b,18383:up|Errfi1,74155:down|Enpp1,18605:down</td></tr>
</table>

multicellular organismal process	GO:0032501	2.54E-13	2.33E-11	Fstl3,83554:down|Adm,11535:up|Flrt2,399558:down|Tmem176b,65963:up|Plxnd1,67784:up|Sfrp4,20379:up|Serpinh1,12406:down|Lyl1,17095:up|Igfbp3,16009:up|Ibsp,15891:up|Plk2,20620:down|Cd24a,12484:down|Mme,17380:up|Il1rn,16181:up|Bmp4,12159:up|Hpse,15442:up|Fgfr2,14183:up|Thbs2,21826:down|Htr2b,15559:down|Gjb5,14622:down|Anxa8,11752:down|Aspn,66695:down|Mmp2,17390:down|Slc40a1,53945:up|Egfr,13649:down|Alpl,11647:up|Tfrc,22042:up|Sncg,20618:up|Rspo2,239405:up|Fam20a,208659:down|Cdsn,386463:up|Col5a2,12832:down|Aldh1a2,19378:up|Ccl2,20296:up|Ednrb,13618:up|Ampd3,11717:up|Mmp13,17386:up|Tnfrsf12a,27279:down|Uchl5,56207:up|Clec4n,56620:up|Shroom4,208431:down|Cdo1,12583:up|Csrp2,13008:down|Ncam1,17967:down|Ccl4,20303:up|Chst11,58250:down|Tmeff1,230157:down|Vegfb,22340:up|Aldh1a3,56847:down|Plpp3,67916:up|Mrc2,17534:down|Lrrc17,74511:down|Lama2,16773:down|Ptk7,71461:down|Inhba,16323:down|Bnc2,242509:down|Tfpi2,21789:down|Crlf1,12931:down|Vegfa,22339:down|Serpine2,20720:up|Nxn,18230:down|Igf2,16002:up|Tmem176a,66058:up|Tnf,21926:up|Col1a2,12843:down|Fxyd1,56188:up|Fgfr1,14182:down|Lpl,16956:up|Sulf1,240725:down|Cd4,12504:up|Col3a1,12825:down|Tbx15,21384:down|Serping1,12258:up|Enpp1,18605:down|Clec2d,93694:up|Hpgd,15446:up|C3,12266:up|Ankrd1,107765:down|Cx3cr1,13051:down|Cd109,235505:down|Loxl2,94352:down|Trf,22041:up|Loxl3,16950:down|Cxcl14,57266:down|Wnt5a,22418:down|Stc1,20855:up|Mark1,226778:up|Col8a1,12837:down|Adipoq,11450:up|Igf1,16000:up|Crabp2,12904:down|Tgfb2,21808:down|F3,14066:up|Clec4e,56619:up|Cthrc1,68588:down|Kng1,16644:up|Prl2c2,18811:up|Efemp1,216616:up|Dpysl3,22240:down|Ninj1,18081:up|Vdr,22337:down|Fabp4,11770:up|Cxcl12,20315:up|Htr2a,15558:down|Gas6,14456:up|Col11a1,12814:down|Sfrp1,20377:up|C1qtnf3,81799:down|Tgfbr3,21814:up|Tll1,21892:down|Ceacam1,26365:up|Sfrp2,20319:up|Tnfrsf11b,18383:up|Prkar2b,19088:up|Npnt,114249:up|S100a1,20193:up|Ly9,17085:up|Pdgfc,54635:down|Mgp,17313:up|Figf,14205:up|Epas1,13819:up|Slc29a1,63959:up|Sema7a,20361:down|Gpm6b,14758:up|Ddah1,69219:down|Postn,50706:down|Akr1c18,105349:up|Ccl12,20293:up|Bmpr1a,12166:down|Wisp1,22402:down|Ank,11732:down|Tnc,21923:down|Ret,19713:up|Prg4,96875:down|Sdf2l1,64136:up|Creb3l1,26427:down|Kitl,17311:up|Il1rl1,17082:down|Fam101b,76566:down|Hp,15439:up|Ccl3,20302:up|Plat,18791:down|Ctla2a,13024:down|Srpx2,68792:down|Adamts12,239337:down|Anpep,16790:up|Pparg,19016:up|Lmcd1,30937:up|Glp1r,14652:down|Hmox1,15368:up|Has2,15117:down|Ptgs2,19225:down|Grem1,23892:up|Pcdh19,279653:down|Mcoln3,171166:up|Col5a1,12831:down|Errfi1,74155:down|Tnfsf9,21950:up
single-organism developmental process	GO:0044767	3.14E-13	2.85E-11	Fstl3,83554:down|Adm,11535:up|Flrt2,399558:down|Tmem176b,65963:up|Plxnd1,67784:up|Sfrp4,20379:up|Serpinh1,12406:down|Lyl1,17095:up|Igfbp3,16009:up|Ibsp,15891:up|Plk2,20620:down|Cd24a,12484:down|Il1rn,16181:up|Bmp4,12159:up|Hpse,15442:up|Medag,70717:down|Fgfr2,14183:up|Thbs2,21826:down|Htr2b,15559:down|Gjb5,14622:down|Aspn,66695:down|Mmp2,17390:down|Slc40a1,53945:up|Egfr,13649:down|Alpl,11647:up|Tfrc,22042:up|Ccl8,20307:up|Rspo2,239405:up|Fam20a,208659:down|Cdsn,386463:up|Col5a2,12832:down|Aldh1a2,19378:up|Ccl2,20296:up|Ednrb,13618:up|Mmp13,17386:up|Tnfrsf12a,27279:down|Uchl5,56207:up|Shroom4,208431:down|Cdo1,12583:up|Csrp2,13008:down|Ncam1,17967:down|Chst11,58250:down|Tmeff1,230157:down|Vegfb,22340:up|Aldh1a3,56847:down|Plpp3,67916:up|Mrc2,17534:down|Lrrc17,74511:down|Lama2,16773:down|Ccl9,20308:up|Ptk7,71461:down|Inhba,16323:down|Bnc2,242509:down|Crlf1,12931:down|Vegfa,22339:down|Serpine2,20720:up|Nxn,18230:down|Igf2,16002:up|Tnf,21926:up|Tmem176a,66058:up|Col1a2,12843:down|Fgfr1,14182:down|Lpl,16956:up|Sulf1,240725:down|Cd4,12504:up|Col3a1,12825:down|Tbx15,21384:down|Enpp1,18605:down|Clec2d,93694:up|Hpgd,15446:up|C3,12266:up|Ankrd1,107765:down|Cx3cr1,13051:down|Cd109,235505:down|Loxl2,94352:down|Trf,22041:up|Loxl3,16950:down|Cxcl14,57266:down|Wnt5a,22418:down|Stc1,20855:up|Mark1,226778:up|Col8a1,12837:down|Adipoq,11450:up|Igf1,16000:up|Crabp2,12904:down|Cxcl9,17329:up|Tgfb2,21808:down|F3,14066:up|Clec4e,56619:up|Prl2c2,18811:up|Cthrc1,68588:down|Efemp1,216616:up|Dpysl3,22240:down|Ninj1,18081:up|Vdr,22337:down|Fabp4,11770:up|Cxcl12,20315:up|Htr2a,15558:down|Gas6,14456:up|Col11a1,12814:down|Sfrp1,20377:up|Tgfbr3,21814:up|Tll1,21892:down|Ceacam1,26365:up|Sfrp2,20319:up|Tnfrsf11b,18383:up|Npnt,114249:up|Ly9,17085:up|Mgp,17313:up|Figf,14205:up|Pdgfc,54635:down|Epas1,13819:up|Sema7a,20361:down|Gpm6b,14758:up|Ddah1,69219:down|Postn,50706:down|Ccl12,20293:up|Bmpr1a,12166:down|Wisp1,22402:down|Ank,11732:down|Tnc,21923:down|Ret,19713:up|Prg4,96875:down|Sdf2l1,64136:up|Creb3l1,26427:down|Kitl,17311:up|Fam101b,76566:down|Hp,15439:up|Ccl3,20302:up|Ctla2a,13024:down|Srpx2,68792:down|Prelp,116847:up|Adamts12,239337:down|Anpep,16790:up|Pparg,19016:up|Glp1r,14652:down|Hmox1,15368:up|Has2,15117:down|Ptgs2,19225:down|Grem1,23892:up|Pcdh19,279653:down|Mcoln3,171166:up|Col5a1,12831:down|Errfi1,74155:down|Tnfsf9,21950:up
developmental process	GO:0032502	7.43E-13	6.63E-11	Fstl3,83554:down|Adm,11535:up|Flrt2,399558:down|Tmem176b,65963:up|Plxnd1,67784:up|Sfrp4,20379:up|Serpinh1,12406:down|Lyl1,17095:up|Igfbp3,16009:up|Ibsp,15891:up|Plk2,20620:down|Cd24a,12484:down|Il1rn,16181:up|Bmp4,12159:up|Hpse,15442:up|Medag,70717:down|Fgfr2,14183:up|Thbs2,21826:down|Htr2b,15559:down|Gjb5,14622:down|Aspn,66695:down|Mmp2,17390:down|Slc40a1,53945:up|Egfr,13649:down|Alpl,11647:up|Tfrc,22042:up|Ccl8,20307:up|Rspo2,239405:up|Fam20a,208659:down|Cdsn,386463:up|Col5a2,12832:down|Aldh1a2,19378:up|Ccl2,20296:up|Ednrb,13618:up|Mmp13,17386:up|Tnfrsf12a,27279:down|Uchl5,56207:up|Shroom4,208431:down|Cdo1,12583:up|Csrp2,13008:down|Ncam1,17967:down|Chst11,58250:down|Tmeff1,230157:down|Vegfb,22340:up|Aldh1a3,56847:down|Plpp3,67916:up|Mrc2,17534:down|Lrrc17,74511:down|Lama2,16773:down|Ccl9,20308:up|Ptk7,71461:down|Inhba,16323:down|Bnc2,242509:down|Crlf1,12931:down|Vegfa,22339:down|Serpine2,20720:up|Nxn,18230:down|Igf2,16002:up|Tnf,21926:up|Tmem176a,66058:up|Col1a2,12843:down|Fgfr1,14182:down|Lpl,16956:up|Sulf1,240725:down|Cd4,12504:up|Col3a1,12825:down|Tbx15,21384:down|Enpp1,18605:down|Clec2d,93694:up|Hpgd,15446:up|C3,12266:up|Ankrd1,107765:down|Cx3cr1,13051:down|Cd109,235505:down|Loxl2,94352:down|Trf,22041:up|Loxl3,16950:down|Cxcl14,57266:down|Wnt5a,22418:down|Stc1,20855:up|Mark1,226778:up|Col8a1,12837:down|Adipoq,11450:up|Igf1,16000:up|Crabp2,12904:down|Cxcl9,17329:up|Tgfb2,21808:down|F3,14066:up|Clec4e,56619:up|Prl2c2,18811:up|Cthrc1,68588:down|Efemp1,216616:up|Dpysl3,22240:down|Ninj1,18081:up|Vdr,22337:down|Fabp4,11770:up|Cxcl12,20315:up|Htr2a,15558:down|Gas6,14456:up|Col11a1,12814:down|Sfrp1,20377:up|Tgfbr3,21814:up|Tll1,21892:down|Ceacam1,26365:up|Sfrp2,20319:up|Tnfrsf11b,18383:up|Npnt,114249:up|Ly9,17085:up|Mgp,17313:up|Figf,14205:up|Pdgfc,54635:down|Epas1,13819:up|Sema7a,20361:down|Gpm6b,14758:up|Ddah1,69219:down|Postn,50706:down|Ccl12,20293:up|Bmpr1a,12166:down|Wisp1,22402:down|Ank,11732:down|Tnc,21923:down|Ret,19713:up|Prg4,96875:down|Sdf2l1,64136:up|Creb3l1,26427:down|Kitl,17311:up|Fam101b,76566:down|Hp,15439:up|Ccl3,20302:up|Ctla2a,13024:down|Srpx2,68792:down|Prelp,116847:up|Adamts12,239337:down|Anpep,16790:up|Pparg,19016:up|Glp1r,14652:down|Hmox1,15368:up|Has2,15117:down|Ptgs2,19225:down|Grem1,23892:up|Pcdh19,279653:down|Mcoln3,171166:up|Col5a1,12831:down|Errfi1,74155:down|Tnfsf9,21950:up

cell proliferation	GO:0008283	7.85E-13	6.91E-11	Igfbp4,16010:up|Adm,11535:up|Sfrp4,20379:up|Cx3cr1,13051:down|Igfbp3,16009:up|Cd109,235505:down|Loxl2,94352:down|Trf,22041:up|Cd24a,12484:down|Wnt5a,22418:down|Stc1,20855:up|Col8a1,12837:down|Adipoq,11450:up|Bmp4,12159:up|Igf1,16000:up|Cxcl9,17329:up|Tgfb2,21808:down|Hpse,15442:up|Ptgir,19222:up|Fgfr2,14183:up|F3,14066:up|Cthrc1,68588:down|Htr2b,15559:down|Prl2c2,18811:up|Efemp1,216616:up|Vdr,22337:down|Fabp4,11770:up|Cxcl12,20315:up|Htr2a,15558:down|Mmp2,17390:down|Gas6,14456:up|Egfr,13649:down|Sfrp1,20377:up|Tgfbr3,21814:up|Tfrc,22042:up|Ceacam1,26365:up|Sfrp2,20319:up|Tnfrsf11b,18383:up|Aldh1a2,19378:up|Ccl2,20296:up|Ednrb,13618:up|Figf,14205:up|Pdgfc,54635:down|Akr1c18,105349:up|Ncam1,17967:down|Ccl12,20293:up|Bmpr1a,12166:down|Wisp1,22402:down|Chst11,58250:down|Tnc,21923:down|Prg4,96875:down|Pdxk,216134:up|Kitl,17311:up|Il1rl1,17082:down|Inhba,16323:down|Crlf1,12931:down|Vegfa,22339:down|Serpine2,20720:up|Igf2,16002:up|Pparg,19016:up|Tnf,21926:up|Glp1r,14652:down|Fgfr1,14182:down|Hmox1,15368:up|Has2,15117:down|Sulf1,240725:down|Cd4,12504:up|Ptgs2,19225:down|Grem1,23892:up|Errfi1,74155:down|Tnfsf9,21950:up
angiogenesis	GO:0001525	8.26E-13	7.06E-11	Adm,11535:up|Plxnd1,67784:up|Ccl2,20296:up|C3,12266:up|Figf,14205:up|Epas1,13819:up|Tnfrsf12a,27279:down|Ddah1,69219:down|Cx3cr1,13051:down|Loxl2,94352:down|Wnt5a,22418:down|Ccl12,20293:up|Col8a1,12837:down|Bmp4,12159:up|Hpse,15442:up|Vegfb,22340:up|Fgfr2,14183:up|F3,14066:up|Thbs2,21826:down|Prl2c2,18811:up|Srpx2,68792:down|Cxcl12,20315:up|Vegfa,22339:down|Mmp2,17390:down|Anpep,16790:up|Fgfr1,14182:down|Hmox1,15368:up|Sulf1,240725:down|Ptgs2,19225:down|Grem1,23892:up|Ceacam1,26365:up|Sfrp2,20319:up
enzyme linked receptor protein signaling pathway	GO:0007167	9.67E-13	8.15E-11	Fstl3,83554:down|Igfbp4,16010:up|Flrt2,399558:down|Hpgd,15446:up|Ccl2,20296:up|Pdgfc,54635:down|Figf,14205:up|Htra3,78558:down|Igfbp3,16009:up|Cd109,235505:down|Dok2,13449:up|Trf,22041:up|Wnt5a,22418:down|Bmpr1a,12166:down|Adipoq,11450:up|Bmp4,12159:up|Igf1,16000:up|Chst11,58250:down|Ret,19713:up|Tgfb2,21808:down|Ptgir,19222:up|Fgfr2,14183:up|F3,14066:up|Efemp1,216616:up|Inhba,16323:down|Aspn,66695:down|Plat,18791:down|Vegfa,22339:down|Adamts12,239337:down|Igf2,16002:up|Gas6,14456:up|Col1a2,12843:down|Egfr,13649:down|Fgfr1,14182:down|Sfrp1,20377:up|Sulf1,240725:down|Tgfbr3,21814:up|Grem1,23892:up|Col3a1,12825:down|Ceacam1,26365:up|Sfrp2,20319:up|Npnt,114249:up|Errfi1,74155:down|Enpp1,18605:down
regulation of signal transduction	GO:0009966	1.00E-12	8.34E-11	Fstl3,83554:down|Igfbp4,16010:up|Adm,11535:up|Flrt2,399558:down|Sfrp4,20379:up|C3,12266:up|Ankrd1,107765:down|Cx3cr1,13051:down|Igfbp3,16009:up|Cd109,235505:down|Plk2,20620:down|Trf,22041:up|Cd24a,12484:down|Wnt5a,22418:down|Il1rn,16181:up|Adipoq,11450:up|Bmp4,12159:up|Igf1,16000:up|Crabp2,12904:down|Tgfb2,21808:down|Hpse,15442:up|Ptgir,19222:up|Fgfr2,14183:up|F3,14066:up|Cthrc1,68588:down|Htr2b,15559:down|Ccl6,20305:up|Aspn,66695:down|Cxcl12,20315:up|Htr2a,15558:down|Gas6,14456:up|Egfr,13649:down|Sfrp1,20377:up|C1qtnf3,81799:down|Tgfbr3,21814:up|Ceacam1,26365:up|Ccl8,20307:up|Sfrp2,20319:up|Rspo2,239405:up|Npnt,114249:up|Tnfrsf11b,18383:up|Ccl2,20296:up|Ccl7,20306:up|Pdgfc,54635:down|Figf,14205:up|Sema7a,20361:down|Htra3,78558:down|Tnfrsf12a,27279:down|Clec4n,56620:up|Postn,50706:down|Akr1c18,105349:up|Ncam1,17967:down|Ccl12,20293:up|Bmpr1a,12166:down|Ccl4,20303:up|Chst11,58250:down|Ret,19713:up|Sdf2l1,64136:up|Aldh1a3,56847:down|Plpp3,67916:up|Creb3l1,26427:down|Kitl,17311:up|Il1rl1,17082:down|Ccl9,20308:up|Inhba,16323:down|Ccl3,20302:up|Crlf1,12931:down|Vegfa,22339:down|Serpine2,20720:up|Adamts12,239337:down|Lrrc15,74488:down|Igf2,16002:up|Nxn,18230:down|Pparg,19016:up|Tnf,21926:up|Lmcd1,30937:up|Tnfrsf23,79201:down|Fgfr1,14182:down|Hmox1,15368:up|Sulf1,240725:down|Cd4,12504:up|Ptgs2,19225:down|Grem1,23892:up|Col3a1,12825:down|Errfi1,74155:down|Enpp1,18605:down
regulation of inflammatory response	GO:0050727	1.09E-12	8.91E-11	Ccl9,20308:up|Ccl6,20305:up|Aoah,27052:up|Ccl7,20306:up|Ccl2,20296:up|C3,12266:up|Ednrb,13618:up|Ccl3,20302:up|Sema7a,20361:down|Ctla2a,13024:down|Fabp4,11770:up|Cx3cr1,13051:down|Adamts12,239337:down|Pparg,19016:up|Tnf,21926:up|Egfr,13649:down|Cd24a,12484:down|C1qtnf3,81799:down|Wnt5a,22418:down|Ccl12,20293:up|Ptgs2,19225:down|Ccl4,20303:up|Adipoq,11450:up|Ccl8,20307:up|Serping1,12258:up|Il1rl1,17082:down
skeletal system development	GO:0001501	1.62E-12	1.31E-10	Col5a2,12832:down|Pdgfc,54635:down|Mgp,17313:up|Mmp13,17386:up|Serpinh1,12406:down|Loxl2,94352:down|Wnt5a,22418:down|Bmpr1a,12166:down|Stc1,20855:up|Bmp4,12159:up|Igf1,16000:up|Chst11,58250:down|Tgfb2,21808:down|Fgfr2,14183:up|Fam101b,76566:down|Lrrc17,74511:down|Efemp1,216616:up|Bnc2,242509:down|Vdr,22337:down|Vegfa,22339:down|Mmp2,17390:down|Adamts12,239337:down|Col1a2,12843:down|Col11a1,12814:down|Alpl,11647:up|Fgfr1,14182:down|Sfrp1,20377:up|Has2,15117:down|Sulf1,240725:down|Grem1,23892:up|Sfrp2,20319:up|Tbx15,21384:down|Rspo2,239405:up
cell differentiation	GO:0030154	1.69E-12	1.35E-10	Fstl3,83554:down|Clec2d,93694:up|Adm,11535:up|Flrt2,399558:down|Tmem176b,65963:up|Plxnd1,67784:up|Sfrp4,20379:up|Ankrd1,107765:down|Serpinh1,12406:down|Lyl1,17095:up|Cx3cr1,13051:down|Igfbp3,16009:up|Ibsp,15891:up|Cd109,235505:down|Loxl2,94352:down|Plk2,20620:down|Loxl3,16950:down|Trf,22041:up|Cxcl14,57266:down|Cd24a,12484:down|Wnt5a,22418:down|Mark1,226778:up|Stc1,20855:up|Adipoq,11450:up|Bmp4,12159:up|Igf1,16000:up|Crabp2,12904:down|Cxcl9,17329:up|Tgfb2,21808:down|Medag,70717:down|Fgfr2,14183:up|Clec4e,56619:up|Cthrc1,68588:down|Htr2b,15559:down|Prl2c2,18811:up|Efemp1,216616:up|Gjb5,14622:down|Dpysl3,22240:down|Vdr,22337:down|Fabp4,11770:up|Cxcl12,20315:up|Htr2a,15558:down|Mmp2,17390:down|Gas6,14456:up|Egfr,13649:down|Col11a1,12814:down|Alpl,11647:up|Sfrp1,20377:up|Tgfbr3,21814:up|Tfrc,22042:up|Tll1,21892:down|Ceacam1,26365:up|Ccl8,20307:up|Sfrp2,20319:up|Rspo2,239405:up|Npnt,114249:up|Col5a2,12832:down|Ly9,17085:up|Aldh1a2,19378:up|Ccl2,20296:up|Ednrb,13618:up|Figf,14205:up|Mgp,17313:up|Sema7a,20361:down|Epas1,13819:up|Tnfrsf12a,27279:down|Postn,50706:down|Csrp2,13008:down|Ncam1,17967:down|Ccl12,20293:up|Bmpr1a,12166:down|Wisp1,22402:down|Chst11,58250:down|Tnc,21923:down|Ret,19713:up|Vegfb,22340:up|Plpp3,67916:up|Creb3l1,26427:down|Mrc2,17534:down|Kitl,17311:up|Fam101b,76566:down|Lrrc17,74511:down|Lama2,16773:down|Ccl9,20308:up|Ptk7,71461:down|Inhba,16323:down|Ccl3,20302:up|Ctla2a,13024:down|Vegfa,22339:down|Serpine2,20720:up|Adamts12,239337:down|Nxn,18230:down|Igf2,16002:up|Anpep,16790:up|Pparg,19016:up|Tnf,21926:up|Tmem176a,66058:up|Glp1r,14652:down|Fgfr1,14182:down|Has2,15117:down|Lpl,16956:up|Sulf1,240725:down|Cd4,12504:up|Ptgs2,19225:down|Grem1,23892:up|Col3a1,12825:down|Mcoln3,171166:up|Col5a1,12831:down|Errfi1,74155:down|Enpp1,18605:down|Tnfsf9,21950:up
inflammatory response	GO:0006954	1.89E-12	1.49E-10	Ccl7,20306:up|Ccl2,20296:up|C3,12266:up|Ednrb,13618:up|Sema7a,20361:down|Cx3cr1,13051:down|Ggt5,23887:up|Cd24a,12484:down|Wnt5a,22418:down|Ccl12,20293:up|Il1rn,16181:up|Ccl4,20303:up|Adipoq,11450:up|Cxcl9,17329:up|Ptgir,19222:up|F3,14066:up|Il1rl1,17082:down|Kng1,16644:up|Hp,15439:up|Ccl9,20308:up|Ccl6,20305:up|Aoah,27052:up|Ccl3,20302:up|Saa3,20210:up|Ctla2a,13024:down|Cxcr6,80901:down|Fabp4,11770:up|Adamts12,239337:down|Pparg,19016:up|Tnf,21926:up|Egfr,13649:down|Hmox1,15368:up|C1qtnf3,81799:down|Ptgs2,19225:down|Ccl8,20307:up|Serping1,12258:up|Tnfrsf11b,18383:up

<table>
<tr><th></th><th></th><th></th><th></th><th></th></tr>
<tr><td>positive regulation of protein phosphorylation</td><td>GO:0001934</td><td>2.20E-12</td><td>1.71E-10</td><td>Igfbp4,16010:up|Ccl7,20306:up|Ccl2,20296:up|C3,12266:up|Pdgfc,54635:down|Ednrb,13618:up|Sema7a,20361:down|Igfbp3,16009:up|Trf,22041:up|Cd24a,12484:down|Wnt5a,22418:down|Bmpr1a,12166:down|Ccl12,20293:up|Il1rn,16181:up|Ccl4,20303:up|Adipoq,11450:up|Bmp4,12159:up|Igf1,16000:up|Ret,19713:up|Tgfb2,21808:down|Plpp3,67916:up|Fgfr2,14183:up|Kitl,17311:up|Htr2b,15559:down|Ccl9,20308:up|Ccl6,20305:up|Inhba,16323:down|Ccl3,20302:up|Crlf1,12931:down|Vegfa,22339:down|Htr2a,15558:down|Igf2,16002:up|Gas6,14456:up|Tnf,21926:up|Fgfr1,14182:down|Egfr,13649:down|C1qtnf3,81799:down|Cd4,12504:up|Grem1,23892:up|Ccl8,20307:up|Ceacam1,26365:up|Sfrp2,20319:up|Fam20a,208659:down|Tnfrsf11b,18383:up|Npnt,114249:up</td></tr>
<tr><td>anatomical structure development</td><td>GO:0048856</td><td>2.50E-12</td><td>1.92E-10</td><td>Fstl3,83554:down|Adm,11535:up|Flrt2,399558:down|Tmem176b,65963:up|Plxnd1,67784:up|Sfrp4,20379:up|Serpinh1,12406:down|Lyl1,17095:up|Igfbp3,16009:up|Ibsp,15891:up|Plk2,20620:down|Cd24a,12484:down|Il1rn,16181:up|Bmp4,12159:up|Hpse,15442:up|Fgfr2,14183:up|Thbs2,21826:down|Htr2b,15559:down|Gjb5,14622:down|Aspn,66695:down|Mmp2,17390:down|Slc40a1,53945:up|Egfr,13649:down|Alpl,11647:up|Tfrc,22042:up|Ccl8,20307:up|Rspo2,239405:up|Fam20a,208659:down|Cdsn,386463:up|Col5a2,12832:down|Aldh1a2,19378:up|Ccl2,20296:up|Ednrb,13618:up|Mmp13,17386:up|Tnfrsf12a,27279:down|Uchl5,56207:up|Shroom4,208431:down|Cdo1,12583:up|Csrp2,13008:down|Ncam1,17967:down|Chst11,58250:down|Tmeff1,230157:down|Vegfb,22340:up|Aldh1a3,56847:down|Plpp3,67916:up|Lrrc17,74511:down|Lama2,16773:down|Ccl9,20308:up|Ptk7,71461:down|Inhba,16323:down|Bnc2,242509:down|Crlf1,12931:down|Vegfa,22339:down|Serpine2,20720:up|Nxn,18230:down|Igf2,16002:up|Tnf,21926:up|Tmem176a,66058:up|Col1a2,12843:down|Fgfr1,14182:down|Sulf1,240725:down|Cd4,12504:up|Col3a1,12825:down|Tbx15,21384:down|Enpp1,18605:down|Clec2d,93694:up|Hpgd,15446:up|C3,12266:up|Ankrd1,107765:down|Cx3cr1,13051:down|Cd109,235505:down|Trf,22041:up|Loxl3,16950:down|Loxl2,94352:down|Cxcl14,57266:down|Wnt5a,22418:down|Stc1,20855:up|Mark1,226778:up|Col8a1,12837:down|Adipoq,11450:up|Igf1,16000:up|Crabp2,12904:down|Cxcl9,17329:up|Tgfb2,21808:down|F3,14066:up|Clec4e,56619:up|Prl2c2,18811:up|Cthrc1,68588:down|Efemp1,216616:up|Dpysl3,22240:down|Ninj1,18081:up|Vdr,22337:down|Cxcl12,20315:up|Gas6,14456:up|Col11a1,12814:down|Sfrp1,20377:up|Tgfbr3,21814:up|Tll1,21892:down|Ceacam1,26365:up|Sfrp2,20319:up|Tnfrsf11b,18383:up|Npnt,114249:up|Ly9,17085:up|Mgp,17313:up|Figf,14205:up|Pdgfc,54635:down|Epas1,13819:up|Sema7a,20361:down|Gpm6b,14758:up|Ddah1,69219:down|Postn,50706:down|Ccl12,20293:up|Bmpr1a,12166:down|Wisp1,22402:down|Ank,11732:down|Tnc,21923:down|Ret,19713:up|Sdf2l1,64136:up|Prg4,96875:down|Creb3l1,26427:down|Kitl,17311:up|Fam101b,76566:down|Hp,15439:up|Ccl3,20302:up|Ctla2a,13024:down|Srpx2,68792:down|Adamts12,239337:down|Anpep,16790:up|Pparg,19016:up|Hmox1,15368:up|Has2,15117:down|Ptgs2,19225:down|Grem1,23892:up|Pcdh19,279653:down|Mcoln3,171166:up|Col5a1,12831:down|Errfi1,74155:down|Tnfsf9,21950:up</td></tr>
<tr><td>positive regulation of cell differentiation</td><td>GO:0045597</td><td>2.58E-12</td><td>1.96E-10</td><td>Adm,11535:up|Sfrp4,20379:up|Sema7a,20361:down|Ankrd1,107765:down|Tnfrsf12a,27279:down|Cx3cr1,13051:down|Igfbp3,16009:up|Loxl2,94352:down|Trf,22041:up|Cd24a,12484:down|Wnt5a,22418:down|Bmpr1a,12166:down|Wisp1,22402:down|Adipoq,11450:up|Bmp4,12159:up|Igf1,16000:up|Crabp2,12904:down|Cxcl9,17329:up|Ret,19713:up|Tgfb2,21808:down|Medag,70717:down|Kitl,17311:up|Cthrc1,68588:down|Prl2c2,18811:up|Ptk7,71461:down|Inhba,16323:down|Dpysl3,22240:down|Ccl3,20302:up|Vdr,22337:down|Cxcl12,20315:up|Vegfa,22339:down|Htr2a,15558:down|Serpine2,20720:up|Pparg,19016:up|Gas6,14456:up|Tnf,21926:up|Glp1r,14652:down|Fgfr1,14182:down|Lpl,16956:up|Sfrp1,20377:up|Has2,15117:down|Ptgs2,19225:down|Grem1,23892:up|Ccl8,20307:up|Ceacam1,26365:up|Sfrp2,20319:up|Npnt,114249:up|Tnfsf9,21950:up</td></tr>
<tr><td>regulation of response to external stimulus</td><td>GO:0032101</td><td>4.09E-12</td><td>3.03E-10</td><td>Plxnd1,67784:up|Ccl7,20306:up|Ccl2,20296:up|C3,12266:up|Figf,14205:up|Ednrb,13618:up|Sema7a,20361:down|Cx3cr1,13051:down|Cd109,235505:down|Cxcl14,57266:down|Cd24a,12484:down|Wnt5a,22418:down|Ccl12,20293:up|Ccl4,20303:up|Adipoq,11450:up|Cxcl9,17329:up|Hpse,15442:up|Ifit1,15957:up|Il1rl1,17082:down|Kng1,16644:up|Ccl9,20308:up|Ccl6,20305:up|Aoah,27052:up|Ccl3,20302:up|Ctla2a,13024:down|Fabp4,11770:up|Cxcl12,20315:up|Vegfa,22339:down|Serpine2,20720:up|Adamts12,239337:down|Pparg,19016:up|Gas6,14456:up|Tnf,21926:up|Lmcd1,30937:up|Fgfr1,14182:down|Egfr,13649:down|Hmox1,15368:up|C1qtnf3,81799:down|Ptgs2,19225:down|Grem1,23892:up|Ccl8,20307:up|Ceacam1,26365:up|Serping1,12258:up</td></tr>
<tr><td>regulation of protein phosphorylation</td><td>GO:0001932</td><td>5.23E-12</td><td>3.83E-10</td><td>Igfbp4,16010:up|Flrt2,399558:down|Sfrp4,20379:up|Ccl7,20306:up|Ccl2,20296:up|C3,12266:up|Pdgfc,54635:down|Ednrb,13618:up|Sema7a,20361:down|Igfbp3,16009:up|Cd109,235505:down|Trf,22041:up|Cd24a,12484:down|Wnt5a,22418:down|Bmpr1a,12166:down|Ccl12,20293:up|Il1rn,16181:up|Ccl4,20303:up|Adipoq,11450:up|Bmp4,12159:up|Igf1,16000:up|Ret,19713:up|Tgfb2,21808:down|Plpp3,67916:up|Fgfr2,14183:up|Kitl,17311:up|Htr2b,15559:down|Ccl9,20308:up|Ccl6,20305:up|Inhba,16323:down|Aspn,66695:down|Ccl3,20302:up|Crlf1,12931:down|Fabp4,11770:up|Vegfa,22339:down|Htr2a,15558:down|Lrrc15,74488:down|Igf2,16002:up|Gas6,14456:up|Tnf,21926:up|Fgfr1,14182:down|Egfr,13649:down|Sfrp1,20377:up|C1qtnf3,81799:down|Cd4,12504:up|Grem1,23892:up|Ccl8,20307:up|Ceacam1,26365:up|Sfrp2,20319:up|Fam20a,208659:down|Npnt,114249:up|Prkar2b,19088:up|Tnfrsf11b,18383:up|Errfi1,74155:down|Enpp1,18605:down</td></tr>
<tr><td>regulation of anatomical structure morphogenesis</td><td>GO:0022603</td><td>8.37E-12</td><td>5.98E-10</td><td>Col5a2,12832:down|Adm,11535:up|Plxnd1,67784:up|Ccl2,20296:up|C3,12266:up|Sema7a,20361:down|Tnfrsf12a,27279:down|Ddah1,69219:down|Postn,50706:down|Cx3cr1,13051:down|Wnt5a,22418:down|Bmpr1a,12166:down|Ccl12,20293:up|Il1rn,16181:up|Adipoq,11450:up|Bmp4,12159:up|Crabp2,12904:down|Cxcl9,17329:up|Ret,19713:up|Tgfb2,21808:down|Fgfr2,14183:up|F3,14066:up|Thbs2,21826:down|Cthrc1,68588:down|Prl2c2,18811:up|Ptk7,71461:down|Aspn,66695:down|Ccl3,20302:up|Vdr,22337:down|Srpx2,68792:down|Cxcl12,20315:up|Vegfa,22339:down|Adamts12,239337:down|Tnf,21926:up|Fgfr1,14182:down|Hmox1,15368:up|Sfrp1,20377:up|Has2,15117:down|Sulf1,240725:down|Tgfbr3,21814:up|Ptgs2,19225:down|Grem1,23892:up|Ccl8,20307:up|Ceacam1,26365:up|Sfrp2,20319:up|Col5a1,12831:down|Rspo2,239405:up|Tnfrsf11b,18383:up</td></tr>
<tr><td>regulation of intracellular signal transduction</td><td>GO:1902531</td><td>1.03E-11</td><td>7.30E-10</td><td>Igfbp4,16010:up|Flrt2,399558:down|Sfrp4,20379:up|Ccl7,20306:up|Ccl2,20296:up|C3,12266:up|Pdgfc,54635:down|Sema7a,20361:down|Ankrd1,107765:down|Clec4n,56620:up|Igfbp3,16009:up|Plk2,20620:down|Akr1c18,105349:up|Trf,22041:up|Cd24a,12484:down|Ncam1,17967:down|Wnt5a,22418:down|Ccl12,20293:up|Il1rn,16181:up|Ccl4,20303:up|Adipoq,11450:up|Bmp4,12159:up|Igf1,16000:up|Ret,19713:up|Tgfb2,21808:down|Hpse,15442:up|Ptgir,19222:up|Creb3l1,26427:down|Fgfr2,14183:up|F3,14066:up|Il1rl1,17082:down|Kitl,17311:up|Htr2b,15559:down|Ccl9,20308:up|Ccl6,20305:up|Inhba,16323:down|Aspn,66695:down|Ccl3,20302:up|Crlf1,12931:down|Cxcl12,20315:up|Vegfa,22339:down|Htr2a,15558:down|Serpine2,20720:up|Lrrc15,74488:down|Igf2,16002:up|Gas6,14456:up|Tnf,21926:up|Lmcd1,30937:up|Fgfr1,14182:down|Egfr,13649:down|Hmox1,15368:up|Sfrp1,20377:up|C1qtnf3,81799:down|Cd4,12504:up|Ptgs2,19225:down|Grem1,23892:up|Col3a1,12825:down|Ceacam1,26365:up|Ccl8,20307:up|Sfrp2,20319:up|Npnt,114249:up|Tnfrsf11b,18383:up|Errfi1,74155:down</td></tr>
</table>

<table>
<tr><td>regulation of response to stress</td><td>GO:0080134</td><td>1.10E-11</td><td>7.71E-10</td><td>Clec2d,93694:up|Sfrp4,20379:up|Ccl7,20306:up|Ccl2,20296:up|C3,12266:up|Ednrb,13618:up|Sema7a,20361:down|Ankrd1,107765:down|Tnfrsf12a,27279:down|Cx3cr1,13051:down|Cd109,235505:down|Trf,22041:up|Cd24a,12484:down|Wnt5a,22418:down|Bmpr1a,12166:down|Ccl12,20293:up|Il1rn,16181:up|Ccl4,20303:up|Adipoq,11450:up|Tgfb2,21808:down|Sdf2l1,64136:up|Hpse,15442:up|Plpp3,67916:up|Ifit1,15957:up|Creb3l1,26427:down|Il1rl1,17082:down|Kng1,16644:up|Hp,15439:up|Ccl9,20308:up|Ccl6,20305:up|Aoah,27052:up|Ccl3,20302:up|Ctla2a,13024:down|Fabp4,11770:up|Cxcl12,20315:up|Vegfa,22339:down|Mmp2,17390:down|Serpine2,20720:up|Adamts12,239337:down|Igf2,16002:up|Pparg,19016:up|Tnf,21926:up|Lmcd1,30937:up|Egfr,13649:down|Hmox1,15368:up|Sfrp1,20377:up|C1qtnf3,81799:down|Ptgs2,19225:down|Ccl8,20307:up|Ceacam1,26365:up|Sfrp2,20319:up|Serping1,12258:up|Errfi1,74155:down</td></tr>
<tr><td>positive regulation of phosphorylation</td><td>GO:0042327</td><td>1.19E-11</td><td>8.18E-10</td><td>Igfbp4,16010:up|Ccl7,20306:up|Ccl2,20296:up|C3,12266:up|Pdgfc,54635:down|Ednrb,13618:up|Sema7a,20361:down|Igfbp3,16009:up|Trf,22041:up|Cd24a,12484:down|Wnt5a,22418:down|Bmpr1a,12166:down|Ccl12,20293:up|Il1rn,16181:up|Ccl4,20303:up|Adipoq,11450:up|Bmp4,12159:up|Igf1,16000:up|Ret,19713:up|Tgfb2,21808:down|Plpp3,67916:up|Fgfr2,14183:up|Kitl,17311:up|Htr2b,15559:down|Ccl9,20308:up|Ccl6,20305:up|Inhba,16323:down|Ccl3,20302:up|Crlf1,12931:down|Vegfa,22339:down|Htr2a,15558:down|Igf2,16002:up|Gas6,14456:up|Tnf,21926:up|Fgfr1,14182:down|Egfr,13649:down|C1qtnf3,81799:down|Cd4,12504:up|Grem1,23892:up|Ccl8,20307:up|Ceacam1,26365:up|Sfrp2,20319:up|Fam20a,208659:down|Tnfrsf11b,18383:up|Npnt,114249:up</td></tr>
<tr><td>cellular response to tumor necrosis factor</td><td>GO:0071356</td><td>1.78E-11</td><td>1.22E-09</td><td>Ccl9,20308:up|Ccl6,20305:up|Ccl7,20306:up|Ccl2,20296:up|Ccl3,20302:up|Ankrd1,107765:down|Fabp4,11770:up|Adamts12,239337:down|Gas6,14456:up|Tnf,21926:up|Sfrp1,20377:up|Ccl12,20293:up|Ccl4,20303:up|Adipoq,11450:up|Ccl8,20307:up|Npnt,114249:up</td></tr>
<tr><td>regulation of localization</td><td>GO:0032879</td><td>1.99E-11</td><td>1.35E-09</td><td>Flrt2,399558:down|Plxnd1,67784:up|Sfrp4,20379:up|C3,12266:up|Ankrd1,107765:down|Cx3cr1,13051:down|Igfbp3,16009:up|Trf,22041:up|Cxcl14,57266:down|Wnt5a,22418:down|Stc1,20855:up|Il1rn,16181:up|Maob,109731:up|Adipoq,11450:up|Bmp4,12159:up|Igf1,16000:up|Cxcl9,17329:up|Tgfb2,21808:down|F3,14066:up|Clec4e,56619:up|Htr2b,15559:down|Prl2c2,18811:up|Efemp1,216616:up|Dpysl3,22240:down|Vdr,22337:down|Fam132b,227358:down|Cxcl12,20315:up|Htr2a,15558:down|Mmp2,17390:down|Gas6,14456:up|Egfr,13649:down|Sfrp1,20377:up|C1qtnf3,81799:down|Tgfbr3,21814:up|Sncg,20618:up|Fxyd2,11936:up|Ceacam1,26365:up|Sfrp2,20319:up|S100a1,20193:up|Ice1,218333:down|Ccl2,20296:up|Pdgfc,54635:down|Ednrb,13618:up|Figf,14205:up|Mmp13,17386:up|Sema7a,20361:down|Gpm6b,14758:up|Clec4n,56620:up|Postn,50706:down|Ptx3,19288:up|Hvcn1,74096:up|Ncam1,17967:down|Ccl12,20293:up|Bmpr1a,12166:down|Ccl4,20303:up|Ret,19713:up|Plpp3,67916:up|Kitl,17311:up|Il1rl1,17082:down|Lama2,16773:down|Inhba,16323:down|Ccl3,20302:up|Srpx2,68792:down|Vegfa,22339:down|Serpine2,20720:up|Lrrc15,74488:down|Anpep,16790:up|Kdelr3,105785:down|Pparg,19016:up|Tnf,21926:up|Fxyd1,56188:up|Glp1r,14652:down|Fgfr1,14182:down|Hmox1,15368:up|Lpl,16956:up|Has2,15117:down|Sulf1,240725:down|Cd4,12504:up|Ptgs2,19225:down|Grem1,23892:up|Kcnj15,16516:down|Col3a1,12825:down|Enpp1,18605:down</td></tr>
<tr><td>positive regulation of inflammatory response</td><td>GO:0050729</td><td>2.10E-11</td><td>1.39E-09</td><td>Ccl9,20308:up|Ccl6,20305:up|Ccl7,20306:up|Ccl2,20296:up|C3,12266:up|Ccl3,20302:up|Fabp4,11770:up|Tnf,21926:up|Cd24a,12484:down|Egfr,13649:down|Ccl12,20293:up|Wnt5a,22418:down|Ptgs2,19225:down|Ccl4,20303:up|Ccl8,20307:up|Il1rl1,17082:down</td></tr>
<tr><td>biological adhesion</td><td>GO:0022610</td><td>2.10E-11</td><td>1.39E-09</td><td>Fstl3,83554:down|Flrt2,399558:down|Ly9,17085:up|Ccl2,20296:up|Tnfrsf12a,27279:down|Gpm6b,14758:up|Postn,50706:down|Cx3cr1,13051:down|Ibsp,15891:up|Cd24a,12484:down|Ncam1,17967:down|Adam12,11489:down|Wnt5a,22418:down|Wisp1,22402:down|Il1rn,16181:up|Col8a1,12837:down|Adipoq,11450:up|Bmp4,12159:up|Igf1,16000:up|Tnc,21923:down|Col12a1,12816:down|Ret,19713:up|Tgfb2,21808:down|Hpse,15442:up|Plpp3,67916:up|Thbs2,21826:down|Clec4e,56619:up|Kitl,17311:up|Lama2,16773:down|Ptk7,71461:down|Ninj1,18081:up|Tmem8,60455:up|Ctla2a,13024:down|Srpx2,68792:down|Sned1,208777:up|Cxcl12,20315:up|Vegfa,22339:down|Mmp2,17390:down|Serpine2,20720:up|Adamts12,239337:down|Lrrc15,74488:down|Smoc2,64074:down|Igf2,16002:up|Gas6,14456:up|Tnf,21926:up|Egfr,13649:down|Has2,15117:down|Sfrp1,20377:up|Cd4,12504:up|Tfrc,22042:up|Grem1,23892:up|Col3a1,12825:down|Ceacam1,26365:up|Pcdh19,279653:down|Col5a1,12831:down|Emb,13723:up|Npnt,114249:up|Cdsn,386463:up|Tnfsf9,21950:up</td></tr>
<tr><td>collagen fibril organization</td><td>GO:0030199</td><td>2.21E-11</td><td>1.44E-09</td><td>P4ha1,18451:down|Col5a2,12832:down|Loxl2,94352:down|Col1a2,12843:down|Col11a1,12814:down|Grem1,23892:up|Col3a1,12825:down|Serpinh1,12406:down|Sfrp2,20319:up|Col5a1,12831:down|Tgfb2,21808:down</td></tr>
<tr><td>regulation of signaling</td><td>GO:0023051</td><td>2.25E-11</td><td>1.45E-09</td><td>Fstl3,83554:down|Igfbp4,16010:up|Adm,11535:up|Flrt2,399558:down|Sfrp4,20379:up|C3,12266:up|Ankrd1,107765:down|Cx3cr1,13051:down|Igfbp3,16009:up|Cd109,235505:down|Plk2,20620:down|Trf,22041:up|Cd24a,12484:down|Wnt5a,22418:down|Il1rn,16181:up|Adipoq,11450:up|Bmp4,12159:up|Igf1,16000:up|Crabp2,12904:down|Tgfb2,21808:down|Hpse,15442:up|Ptgir,19222:up|Fgfr2,14183:up|F3,14066:up|Cthrc1,68588:down|Htr2b,15559:down|Ccl6,20305:up|Aspn,66695:down|Cxcl12,20315:up|Htr2a,15558:down|Gas6,14456:up|Egfr,13649:down|Sfrp1,20377:up|C1qtnf3,81799:down|Tgfbr3,21814:up|Sncg,20618:up|Ceacam1,26365:up|Ccl8,20307:up|Sfrp2,20319:up|Rspo2,239405:up|Npnt,114249:up|Tnfrsf11b,18383:up|Ccl2,20296:up|Ccl7,20306:up|Pdgfc,54635:down|Figf,14205:up|Sema7a,20361:down|Htra3,78558:down|Tnfrsf12a,27279:down|Gpm6b,14758:up|Clec4n,56620:up|Postn,50706:down|Akr1c18,105349:up|Ncam1,17967:down|Ccl12,20293:up|Bmpr1a,12166:down|Ccl4,20303:up|Chst11,58250:down|Ret,19713:up|Sdf2l1,64136:up|Aldh1a3,56847:down|Plpp3,67916:up|Creb3l1,26427:down|Kitl,17311:up|Il1rl1,17082:down|Ccl9,20308:up|Lama2,16773:down|Inhba,16323:down|Ccl3,20302:up|Crlf1,12931:down|Vegfa,22339:down|Serpine2,20720:up|Adamts12,239337:down|Lrrc15,74488:down|Igf2,16002:up|Nxn,18230:down|Pparg,19016:up|Tnf,21926:up|Lmcd1,30937:up|Glp1r,14652:down|Tnfrsf23,79201:down|Fgfr1,14182:down|Hmox1,15368:up|Sulf1,240725:down|Cd4,12504:up|Ptgs2,19225:down|Grem1,23892:up|Col3a1,12825:down|Errfi1,74155:down|Enpp1,18605:down</td></tr>
<tr><td>epithelial cell proliferation</td><td>GO:0050673</td><td>2.26E-11</td><td>1.45E-09</td><td>Htr2b,15559:down|Igfbp4,16010:up|Ccl2,20296:up|Aldh1a2,19378:up|Ednrb,13618:up|Vdr,22337:down|Cxcl12,20315:up|Vegfa,22339:down|Igfbp3,16009:up|Cd109,235505:down|Tnf,21926:up|Loxl2,94352:down|Egfr,13649:down|Has2,15117:down|Sfrp1,20377:up|Sulf1,240725:down|Wnt5a,22418:down|Tgfbr3,21814:up|Bmpr1a,12166:down|Col8a1,12837:down|Bmp4,12159:up|Igf1,16000:up|Ceacam1,26365:up|Sfrp2,20319:up|Tgfb2,21808:down|Errfi1,74155:down|Fgfr2,14183:up|F3,14066:up</td></tr>
</table>

regulation of phosphorylation	GO:0042325	3.73E-11	2.36E-09	Igfbp4,16010:up|Flrt2,399558:down|Sfrp4,20379:up|Ccl7,20306:up|Ccl2,20296:up|C3,12266:up|Pdgfc,54635:down|Ednrb,13618:up|Sema7a,20361:down|Igfbp3,16009:up|Cd109,235505:down|Trf,22041:up|Cd24a,12484:down|Wnt5a,22418:down|Bmpr1a,12166:down|Ccl12,20293:up|Il1rn,16181:up|Ccl4,20303:up|Adipoq,11450:up|Bmp4,12159:up|Igf1,16000:up|Ret,19713:up|Tgfb2,21808:down|Plpp3,67916:up|Fgfr2,14183:up|Kitl,17311:up|Htr2b,15559:down|Ccl9,20308:up|Ccl6,20305:up|Inhba,16323:down|Aspn,66695:down|Ccl3,20302:up|Srpx2,68792:down|Crlf1,12931:down|Fabp4,11770:up|Vegfa,22339:down|Htr2a,15558:down|Lrrc15,74488:down|Igf2,16002:up|Gas6,14456:up|Tnf,21926:up|Fgfr1,14182:down|Egfr,13649:down|Sfrp1,20377:up|C1qtnf3,81799:down|Cd4,12504:up|Grem1,23892:up|Ccl8,20307:up|Ceacam1,26365:up|Sfrp2,20319:up|Fam20a,208659:down|Npnt,114249:up|Prkar2b,19088:up|Tnfrsf11b,18383:up|Errfi1,74155:down|Enpp1,18605:down
biomineral tissue development	GO:0031214	3.87E-11	2.38E-09	Fam101b,76566:down|Mgp,17313:up|Aspn,66695:down|Mmp13,17386:up|Gpm6b,14758:up|Ibsp,15891:up|Gas6,14456:up|Bmpr1a,12166:down|Ptgs2,19225:down|Grem1,23892:up|Bmp4,12159:up|Igf1,16000:up|Ank,11732:down|Rspo2,239405:up|Fam20a,208659:down|Fgfr2,14183:up|Enpp1,18605:down
regulation of cell communication	GO:0010646	3.88E-11	2.38E-09	Fstl3,83554:down|Igfbp4,16010:up|Adm,11535:up|Flrt2,399558:down|Sfrp4,20379:up|C3,12266:up|Ankrd1,107765:down|Cx3cr1,13051:down|Igfbp3,16009:up|Cd109,235505:down|Plk2,20620:down|Trf,22041:up|Cd24a,12484:down|Wnt5a,22418:down|Il1rn,16181:up|Adipoq,11450:up|Bmp4,12159:up|Igf1,16000:up|Crabp2,12904:down|Tgfb2,21808:down|Hpse,15442:up|Ptgir,19222:up|Fgfr2,14183:up|F3,14066:up|Cthrc1,68588:down|Htr2b,15559:down|Ccl6,20305:up|Aspn,66695:down|Cxcl12,20315:up|Htr2a,15558:down|Gas6,14456:up|Egfr,13649:down|Sfrp1,20377:up|C1qtnf3,81799:down|Tgfbr3,21814:up|Sncg,20618:up|Ceacam1,26365:up|Ccl8,20307:up|Sfrp2,20319:up|Rspo2,239405:up|Npnt,114249:up|Tnfrsf11b,18383:up|Ccl2,20296:up|Ccl7,20306:up|Pdgfc,54635:down|Figf,14205:up|Sema7a,20361:down|Htra3,78558:down|Tnfrsf12a,27279:down|Gpm6b,14758:up|Clec4n,56620:up|Postn,50706:down|Akr1c18,105349:up|Ncam1,17967:down|Ccl12,20293:up|Bmpr1a,12166:down|Ccl4,20303:up|Chst11,58250:down|Ret,19713:up|Sdf2l1,64136:up|Aldh1a3,56847:down|Plpp3,67916:up|Creb3l1,26427:down|Kitl,17311:up|Il1rl1,17082:down|Ccl9,20308:up|Lama2,16773:down|Inhba,16323:down|Ccl3,20302:up|Crlf1,12931:down|Vegfa,22339:down|Serpine2,20720:up|Adamts12,239337:down|Lrrc15,74488:down|Igf2,16002:up|Nxn,18230:down|Pparg,19016:up|Tnf,21926:up|Lmcd1,30937:up|Glp1r,14652:down|Tnfrsf23,79201:down|Fgfr1,14182:down|Hmox1,15368:up|Sulf1,240725:down|Cd4,12504:up|Ptgs2,19225:down|Grem1,23892:up|Col3a1,12825:down|Errfi1,74155:down|Enpp1,18605:down
lymphocyte chemotaxis	GO:0048247	4.29E-11	2.61E-09	Gas6,14456:up|Cxcl14,57266:down|Ccl9,20308:up|Ccl6,20305:up|Wnt5a,22418:down|Ccl7,20306:up|Ccl2,20296:up|Ccl12,20293:up|Ccl4,20303:up|Ccl3,20302:up|Ccl8,20307:up
cell surface receptor signaling pathway	GO:0007166	4.43E-11	2.66E-09	Fstl3,83554:down|Igfbp4,16010:up|Adm,11535:up|Flrt2,399558:down|Hpgd,15446:up|Plxnd1,67784:up|Sfrp4,20379:up|C3,12266:up|Cx3cr1,13051:down|Igfbp3,16009:up|Cd109,235505:down|Trf,22041:up|Cd24a,12484:down|Wnt5a,22418:down|Mark1,226778:up|Il1rn,16181:up|Adipoq,11450:up|Bmp4,12159:up|Igf1,16000:up|Cxcl9,17329:up|Tgfb2,21808:down|Ptgir,19222:up|F3,14066:up|Fgfr2,14183:up|Clec4e,56619:up|Cthrc1,68588:down|Htr2b,15559:down|Efemp1,216616:up|Ccl6,20305:up|Aspn,66695:down|Cxcr6,80901:down|Cxcl12,20315:up|Htr2a,15558:down|Gas6,14456:up|Egfr,13649:down|Sfrp1,20377:up|Tgfbr3,21814:up|Ceacam1,26365:up|Ccl8,20307:up|Sfrp2,20319:up|Rspo2,239405:up|Npnt,114249:up|Ccl2,20296:up|Ccl7,20306:up|Pdgfc,54635:down|Ednrb,13618:up|Figf,14205:up|Sema7a,20361:down|Slc29a1,63959:up|Htra3,78558:down|Tnfrsf12a,27279:down|Postn,50706:down|Dok2,13449:up|Akr1c18,105349:up|Ncam1,17967:down|Ccl12,20293:up|Bmpr1a,12166:down|Wisp1,22402:down|Ccl4,20303:up|Chst11,58250:down|Ret,19713:up|Plpp3,67916:up|Kitl,17311:up|Ccl9,20308:up|Hp,15439:up|Ptk7,71461:down|Inhba,16323:down|Plat,18791:down|Ccl3,20302:up|Vegfa,22339:down|Serpine2,20720:up|Adamts12,239337:down|Lrrc15,74488:down|Igf2,16002:up|Nxn,18230:down|Pparg,19016:up|Tnf,21926:up|Col1a2,12843:down|Glp1r,14652:down|Tnfrsf23,79201:down|Fgfr1,14182:down|Hmox1,15368:up|Sulf1,240725:down|Cd4,12504:up|Grem1,23892:up|Col3a1,12825:down|Errfi1,74155:down|Enpp1,18605:down
positive regulation of response to external stimulus	GO:0032103	4.99E-11	2.96E-09	Ccl9,20308:up|Ccl6,20305:up|Ccl7,20306:up|Ccl2,20296:up|C3,12266:up|Figf,14205:up|Ccl3,20302:up|Fabp4,11770:up|Cxcl12,20315:up|Vegfa,22339:down|Gas6,14456:up|Tnf,21926:up|Cxcl14,57266:down|Fgfr1,14182:down|Egfr,13649:down|Cd24a,12484:down|Hmox1,15368:up|Wnt5a,22418:down|Ccl12,20293:up|Ptgs2,19225:down|Ccl4,20303:up|Ccl8,20307:up|Cxcl9,17329:up|Il1rl1,17082:down
cell adhesion	GO:0007155	5.03E-11	2.96E-09	Fstl3,83554:down|Flrt2,399558:down|Ly9,17085:up|Ccl2,20296:up|Tnfrsf12a,27279:down|Gpm6b,14758:up|Postn,50706:down|Cx3cr1,13051:down|Ibsp,15891:up|Cd24a,12484:down|Ncam1,17967:down|Adam12,11489:down|Wnt5a,22418:down|Wisp1,22402:down|Il1rn,16181:up|Col8a1,12837:down|Adipoq,11450:up|Bmp4,12159:up|Igf1,16000:up|Tnc,21923:down|Col12a1,12816:down|Ret,19713:up|Tgfb2,21808:down|Hpse,15442:up|Plpp3,67916:up|Thbs2,21826:down|Clec4e,56619:up|Kitl,17311:up|Lama2,16773:down|Ptk7,71461:down|Ninj1,18081:up|Tmem8,60455:up|Ctla2a,13024:down|Srpx2,68792:down|Sned1,208777:up|Cxcl12,20315:up|Vegfa,22339:down|Mmp2,17390:down|Serpine2,20720:up|Adamts12,239337:down|Smoc2,64074:down|Igf2,16002:up|Gas6,14456:up|Tnf,21926:up|Egfr,13649:down|Has2,15117:down|Sfrp1,20377:up|Cd4,12504:up|Tfrc,22042:up|Grem1,23892:up|Col3a1,12825:down|Ceacam1,26365:up|Pcdh19,279653:down|Col5a1,12831:down|Emb,13723:up|Npnt,114249:up|Cdsn,386463:up|Tnfsf9,21950:up
positive regulation of phosphate metabolic process	GO:0045937	5.65E-11	3.27E-09	Igfbp4,16010:up|Adm,11535:up|Ccl7,20306:up|Ccl2,20296:up|C3,12266:up|Pdgfc,54635:down|Ednrb,13618:up|Sema7a,20361:down|Igfbp3,16009:up|Trf,22041:up|Cd24a,12484:down|Wnt5a,22418:down|Bmpr1a,12166:down|Ccl12,20293:up|Il1rn,16181:up|Ccl4,20303:up|Adipoq,11450:up|Bmp4,12159:up|Igf1,16000:up|Ret,19713:up|Tgfb2,21808:down|Plpp3,67916:up|Ptgir,19222:up|Fgfr2,14183:up|Kitl,17311:up|Htr2b,15559:down|Ccl9,20308:up|Ccl6,20305:up|Inhba,16323:down|Ccl3,20302:up|Crlf1,12931:down|Vegfa,22339:down|Htr2a,15558:down|Igf2,16002:up|Gas6,14456:up|Tnf,21926:up|Fgfr1,14182:down|Egfr,13649:down|C1qtnf3,81799:down|Cd4,12504:up|Grem1,23892:up|Ccl8,20307:up|Ceacam1,26365:up|Sfrp2,20319:up|Fam20a,208659:down|Tnfrsf11b,18383:up|Npnt,114249:up
positive regulation of phosphorus metabolic process	GO:0010562	5.65E-11	3.27E-09	Igfbp4,16010:up|Adm,11535:up|Ccl7,20306:up|Ccl2,20296:up|C3,12266:up|Pdgfc,54635:down|Ednrb,13618:up|Sema7a,20361:down|Igfbp3,16009:up|Trf,22041:up|Cd24a,12484:down|Wnt5a,22418:down|Bmpr1a,12166:down|Ccl12,20293:up|Il1rn,16181:up|Ccl4,20303:up|Adipoq,11450:up|Bmp4,12159:up|Igf1,16000:up|Ret,19713:up|Tgfb2,21808:down|Plpp3,67916:up|Ptgir,19222:up|Fgfr2,14183:up|Kitl,17311:up|Htr2b,15559:down|Ccl9,20308:up|Ccl6,20305:up|Inhba,16323:down|Ccl3,20302:up|Crlf1,12931:down|Vegfa,22339:down|Htr2a,15558:down|Igf2,16002:up|Gas6,14456:up|Tnf,21926:up|Fgfr1,14182:down|Egfr,13649:down|C1qtnf3,81799:down|Cd4,12504:up|Grem1,23892:up|Ccl8,20307:up|Ceacam1,26365:up|Sfrp2,20319:up|Fam20a,208659:down|Tnfrsf11b,18383:up|Npnt,114249:up

<table>
<tr><th></th><th></th><th></th><th></th><th></th></tr>
<tr><td>response to stimulus</td><td>GO:0050896</td><td>7.59E-11</td><td>4.30E-09</td><td>Fstl3,83554:down|Igfbp4,16010:up|Adm,11535:up|Flrt2,399558:down|Plxnd1,67784:up|Tfec,21426:up|Sfrp4,20379:up|Gstt1,14871:up|Igfbp3,16009:up|Ibsp,15891:up|Plk2,20620:down|Cd24a,12484:down|Mme,17380:up|Il1rn,16181:up|Bmp4,12159:up|Oasl1,231655:up|Hpse,15442:up|Ptgir,19222:up|Fgfr2,14183:up|Htr2b,15559:down|Ccl6,20305:up|Anxa8,11752:down|Slpi,20568:up|Aspn,66695:down|Mt2,17750:up|Cxcr6,80901:down|Nabp1,109019:down|Phf11a,219131:up|Mmp2,17390:down|Oas3,246727:up|Pappa,18491:down|Slc40a1,53945:up|Egfr,13649:down|Alpl,11647:up|C1rb,667277:up|Tfrc,22042:up|Ccl8,20307:up|Gstm1,14862:up|Serpina3g,20715:up|Rspo2,239405:up|Mt1,17748:up|Col5a2,12832:down|Mrc1,17533:up|Aldh1a2,19378:up|Ccl2,20296:up|Ednrb,13618:up|Htra3,78558:down|Tnfrsf12a,27279:down|Uchl5,56207:up|Sord,20322:up|Clec4n,56620:up|Enpp3,209558:down|Cdo1,12583:up|Dok2,13449:up|Hvcn1,74096:up|Ptx3,19288:up|Ncam1,17967:down|Ccl4,20303:up|Chst11,58250:down|Aldh1a3,56847:down|Plpp3,67916:up|Lama2,16773:down|Ccl9,20308:up|Ptk7,71461:down|Inhba,16323:down|Tfpi2,21789:down|Crlf1,12931:down|C1s1,50908:up|Plod2,26432:down|Vegfa,22339:down|Serpine2,20720:up|Nxn,18230:down|Igf2,16002:up|Tnf,21926:up|Col1a2,12843:down|Fgfr1,14182:down|Sulf1,240725:down|Susd2,71733:up|Cd4,12504:up|Col3a1,12825:down|Serping1,12258:up|Enpp1,18605:down|Clec2d,93694:up|Hpgd,15446:up|C3,12266:up|Ankrd1,107765:down|Cx3cr1,13051:down|Cd109,235505:down|Ggt5,23887:up|Loxl2,94352:down|Trf,22041:up|Cxcl14,57266:down|Wnt5a,22418:down|Mark1,226778:up|Adipoq,11450:up|Igf1,16000:up|Crabp2,12904:down|Cxcl9,17329:up|Tgfb2,21808:down|F3,14066:up|Clec4e,56619:up|Adamts4,240913:down|Cthrc1,68588:down|Kng1,16644:up|Efemp1,216616:up|Ninj1,18081:up|Saa3,20210:up|Vdr,22337:down|Pdia5,72599:down|Fabp4,11770:up|Cxcl12,20315:up|Htr2a,15558:down|Smoc2,64074:down|Gas6,14456:up|Col11a1,12814:down|Sfrp1,20377:up|C1qtnf3,81799:down|Tgfbr3,21814:up|Ceacam1,26365:up|Sfrp2,20319:up|Pxdn,69675:down|Tnfrsf11b,18383:up|Npnt,114249:up|Ly9,17085:up|Ccl7,20306:up|Pdgfc,54635:down|Figf,14205:up|Ifit3,15959:up|Epas1,13819:up|Slc29a1,63959:up|Sema7a,20361:down|Postn,50706:down|Hspb7,29818:up|Akr1c18,105349:up|Ccl12,20293:up|Bmpr1a,12166:down|Wisp1,22402:down|Tnc,21923:down|Gatm,67092:down|Ret,19713:up|Prg4,96875:down|Sdf2l1,64136:up|C1s2,317677:up|Ifit1,15957:up|Creb3l1,26427:down|Kitl,17311:up|Il1rl1,17082:down|Hp,15439:up|Aoah,27052:up|Rasl11a,68895:up|Ccl3,20302:up|Plat,18791:down|Ctla2a,13024:down|Adamts12,239337:down|Lrrc15,74488:down|Pparg,19016:up|Lmcd1,30937:up|Glp1r,14652:down|Tnfrsf23,79201:down|Hmox1,15368:up|Has2,15117:down|Ptgs2,19225:down|Grem1,23892:up|Col5a1,12831:down|Errfi1,74155:down|Tnfsf9,21950:up</td></tr>
<tr><td>ERK1 and ERK2 cascade</td><td>GO:0070371</td><td>7.65E-11</td><td>4.30E-09</td><td>Htr2b,15559:down|Ccl9,20308:up|Ccl6,20305:up|Ccl7,20306:up|Ccl2,20296:up|C3,12266:up|Pdgfc,54635:down|Ccl3,20302:up|Sema7a,20361:down|Htr2a,15558:down|Gas6,14456:up|Trf,22041:up|Egfr,13649:down|C1qtnf3,81799:down|Ccl12,20293:up|Ccl4,20303:up|Adipoq,11450:up|Bmp4,12159:up|Igf1,16000:up|Ccl8,20307:up|Npnt,114249:up|Errfi1,74155:down|Fgfr2,14183:up</td></tr>
<tr><td>mesenchymal cell differentiation</td><td>GO:0048762</td><td>8.18E-11</td><td>4.56E-09</td><td>Fam101b,76566:down|Htr2b,15559:down|Aldh1a2,19378:up|Ednrb,13618:up|Sema7a,20361:down|Loxl2,94352:down|Loxl3,16950:down|Fgfr1,14182:down|Sfrp1,20377:up|Has2,15117:down|Wnt5a,22418:down|Tgfbr3,21814:up|Bmpr1a,12166:down|Grem1,23892:up|Bmp4,12159:up|Sfrp2,20319:up|Ret,19713:up|Tgfb2,21808:down|Fgfr2,14183:up|Kitl,17311:up</td></tr>
<tr><td>positive regulation of ERK1 and ERK2 cascade</td><td>GO:0070374</td><td>8.51E-11</td><td>4.69E-09</td><td>Htr2b,15559:down|Ccl9,20308:up|Ccl6,20305:up|Ccl7,20306:up|Ccl2,20296:up|C3,12266:up|Pdgfc,54635:down|Ccl3,20302:up|Sema7a,20361:down|Htr2a,15558:down|Gas6,14456:up|Egfr,13649:down|C1qtnf3,81799:down|Ccl12,20293:up|Ccl4,20303:up|Bmp4,12159:up|Ccl8,20307:up|Npnt,114249:up|Fgfr2,14183:up</td></tr>
<tr><td>cartilage development</td><td>GO:0051216</td><td>9.49E-11</td><td>5.19E-09</td><td>Fam101b,76566:down|Efemp1,216616:up|Mgp,17313:up|Mmp13,17386:up|Serpinh1,12406:down|Adamts12,239337:down|Loxl2,94352:down|Col11a1,12814:down|Fgfr1,14182:down|Sulf1,240725:down|Bmpr1a,12166:down|Wnt5a,22418:down|Stc1,20855:up|Grem1,23892:up|Bmp4,12159:up|Chst11,58250:down|Sfrp2,20319:up|Rspo2,239405:up|Tgfb2,21808:down</td></tr>
<tr><td>positive regulation of cell motility</td><td>GO:2000147</td><td>1.14E-10</td><td>6.08E-09</td><td>Prl2c2,18811:up|Ccl2,20296:up|Pdgfc,54635:down|Figf,14205:up|Sema7a,20361:down|Srpx2,68792:down|Cxcl12,20315:up|Postn,50706:down|Vegfa,22339:down|Mmp2,17390:down|Lrrc15,74488:down|Gas6,14456:up|Trf,22041:up|Cxcl14,57266:down|Fgfr1,14182:down|Egfr,13649:down|Has2,15117:down|Wnt5a,22418:down|Ccl12,20293:up|Ptgs2,19225:down|Ccl4,20303:up|Bmp4,12159:up|Igf1,16000:up|Cxcl9,17329:up|Ret,19713:up|Tgfb2,21808:down|Plpp3,67916:up|F3,14066:up|Kitl,17311:up</td></tr>
<tr><td>positive regulation of response to wounding</td><td>GO:1903036</td><td>1.15E-10</td><td>6.08E-09</td><td>Ccl9,20308:up|Ccl6,20305:up|Ccl7,20306:up|Ccl2,20296:up|C3,12266:up|Ccl3,20302:up|Fabp4,11770:up|Tnf,21926:up|Cd24a,12484:down|Egfr,13649:down|Wnt5a,22418:down|Ccl12,20293:up|Ptgs2,19225:down|Ccl4,20303:up|Ccl8,20307:up|Hpse,15442:up|Plpp3,67916:up|Il1rl1,17082:down</td></tr>
<tr><td>mesenchyme development</td><td>GO:0060485</td><td>1.15E-10</td><td>6.08E-09</td><td>Fam101b,76566:down|Htr2b,15559:down|Ptk7,71461:down|Aldh1a2,19378:up|Ednrb,13618:up|Bnc2,242509:down|Sema7a,20361:down|Loxl2,94352:down|Loxl3,16950:down|Fgfr1,14182:down|Sfrp1,20377:up|Has2,15117:down|Wnt5a,22418:down|Tgfbr3,21814:up|Bmpr1a,12166:down|Grem1,23892:up|Bmp4,12159:up|Sfrp2,20319:up|Ret,19713:up|Tgfb2,21808:down|Fgfr2,14183:up|Kitl,17311:up</td></tr>
<tr><td>cellular developmental process</td><td>GO:0048869</td><td>1.37E-10</td><td>7.16E-09</td><td>Fstl3,83554:down|Clec2d,93694:up|Adm,11535:up|Flrt2,399558:down|Tmem176b,65963:up|Plxnd1,67784:up|Sfrp4,20379:up|Ankrd1,107765:down|Serpinh1,12406:down|Lyl1,17095:up|Cx3cr1,13051:down|Igfbp3,16009:up|Ibsp,15891:up|Cd109,235505:down|Loxl2,94352:down|Plk2,20620:down|Loxl3,16950:down|Trf,22041:up|Cxcl14,57266:down|Cd24a,12484:down|Wnt5a,22418:down|Mark1,226778:up|Stc1,20855:up|Adipoq,11450:up|Bmp4,12159:up|Igf1,16000:up|Crabp2,12904:down|Cxcl9,17329:up|Tgfb2,21808:down|Medag,70717:down|Fgfr2,14183:up|Clec4e,56619:up|Cthrc1,68588:down|Htr2b,15559:down|Prl2c2,18811:up|Efemp1,216616:up|Gjb5,14622:down|Dpysl3,22240:down|Vdr,22337:down|Fabp4,11770:up|Cxcl12,20315:up|Htr2a,15558:down|Mmp2,17390:down|Gas6,14456:up|Egfr,13649:down|Col11a1,12814:down|Alpl,11647:up|Sfrp1,20377:up|Tgfbr3,21814:up|Tfrc,22042:up|Tll1,21892:down|Ceacam1,26365:up|Ccl8,20307:up|Sfrp2,20319:up|Rspo2,239405:up|Npnt,114249:up|Col5a2,12832:down|Ly9,17085:up|Aldh1a2,19378:up|Ccl2,20296:up|Ednrb,13618:up|Figf,14205:up|Mgp,17313:up|Sema7a,20361:down|Epas1,13819:up|Tnfrsf12a,27279:down|Postn,50706:down|Csrp2,13008:down|Ncam1,17967:down|Ccl12,20293:up|Bmpr1a,12166:down|Wisp1,22402:down|Chst11,58250:down|Tnc,21923:down|Ret,19713:up|Vegfb,22340:up|Plpp3,67916:up|Creb3l1,26427:down|Mrc2,17534:down|Kitl,17311:up|Fam101b,76566:down|Lrrc17,74511:down|Lama2,16773:down|Ccl9,20308:up|Ptk7,71461:down|Inhba,16323:down|Ccl3,20302:up|Ctla2a,13024:down|Prelp,116847:up|Vegfa,22339:down|Serpine2,20720:up|Adamts12,239337:down|Nxn,18230:down|Igf2,16002:up|Anpep,16790:up|Pparg,19016:up|Tnf,21926:up|Tmem176a,66058:up|Glp1r,14652:down|Fgfr1,14182:down|Has2,15117:down|Lpl,16956:up|Sulf1,240725:down|Cd4,12504:up|Ptgs2,19225:down|Grem1,23892:up|Col3a1,12825:down|Mcoln3,171166:up|Col5a1,12831:down|Errfi1,74155:down|Enpp1,18605:down|Tnfsf9,21950:up</td></tr>
<tr><td>regulation of ERK1 and ERK2 cascade</td><td>GO:0070372</td><td>1.49E-10</td><td>7.74E-09</td><td>Htr2b,15559:down|Ccl9,20308:up|Ccl6,20305:up|Ccl7,20306:up|Ccl2,20296:up|C3,12266:up|Pdgfc,54635:down|Ccl3,20302:up|Sema7a,20361:down|Htr2a,15558:down|Gas6,14456:up|Egfr,13649:down|C1qtnf3,81799:down|Ccl12,20293:up|Ccl4,20303:up|Adipoq,11450:up|Bmp4,12159:up|Igf1,16000:up|Ccl8,20307:up|Npnt,114249:up|Errfi1,74155:down|Fgfr2,14183:up</td></tr>
</table>

response to tumor necrosis factor	GO:0034612	1.68E-10	8.64E-09	Ccl9,20308:up|Ccl6,20305:up|Ccl7,20306:up|Ccl2,20296:up|Ccl3,20302:up|Ankrd1,107765:down|Fabp4,11770:up|Adamts12,239337:down|Gas6,14456:up|Tnf,21926:up|Sfrp1,20377:up|Ccl12,20293:up|Ccl4,20303:up|Adipoq,11450:up|Ccl8,20307:up|Npnt,114249:up
cellular response to growth factor stimulus	GO:0071363	1.78E-10	9.07E-09	Fstl3,83554:down|Flrt2,399558:down|Hpgd,15446:up|Ccl2,20296:up|Figf,14205:up|Aspn,66695:down|Ankrd1,107765:down|Htra3,78558:down|Vegfa,22339:down|Ibsp,15891:up|Adamts12,239337:down|Cd109,235505:down|Gas6,14456:up|Akr1c18,105349:up|Col1a2,12843:down|Fgfr1,14182:down|Egfr,13649:down|Has2,15117:down|Sfrp1,20377:up|Sulf1,240725:down|Wnt5a,22418:down|Tgfbr3,21814:up|Bmpr1a,12166:down|Grem1,23892:up|Bmp4,12159:up|Col3a1,12825:down|Chst11,58250:down|Sfrp2,20319:up|Tgfb2,21808:down|Npnt,114249:up|Fgfr2,14183:up
osteoblast differentiation	GO:0001649	1.81E-10	9.16E-09	Cthrc1,68588:down|Sema7a,20361:down|Vegfa,22339:down|Ibsp,15891:up|Igfbp3,16009:up|Igf2,16002:up|Tnf,21926:up|Alpl,11647:up|Fgfr1,14182:down|Sfrp1,20377:up|Tgfbr3,21814:up|Bmpr1a,12166:down|Bmp4,12159:up|Igf1,16000:up|Sfrp2,20319:up|Tnc,21923:down|Rspo2,239405:up|Npnt,114249:up|Creb3l1,26427:down|Mrc2,17534:down
cytokine-mediated signaling pathway	GO:0019221	2.03E-10	1.02E-08	Flrt2,399558:down|Ccl9,20308:up|Ccl6,20305:up|Ccl7,20306:up|Ccl2,20296:up|Aspn,66695:down|Ccl3,20302:up|Cxcl12,20315:up|Cx3cr1,13051:down|Lrrc15,74488:down|Gas6,14456:up|Pparg,19016:up|Tnf,21926:up|Cd24a,12484:down|Wnt5a,22418:down|Ccl12,20293:up|Il1rn,16181:up|Ccl4,20303:up|Adipoq,11450:up|Ceacam1,26365:up|Ccl8,20307:up|Cxcl9,17329:up|F3,14066:up
positive regulation of cellular component movement	GO:0051272	2.20E-10	1.10E-08	Prl2c2,18811:up|Ccl2,20296:up|Pdgfc,54635:down|Figf,14205:up|Sema7a,20361:down|Srpx2,68792:down|Cxcl12,20315:up|Postn,50706:down|Vegfa,22339:down|Mmp2,17390:down|Lrrc15,74488:down|Gas6,14456:up|Trf,22041:up|Cxcl14,57266:down|Fgfr1,14182:down|Egfr,13649:down|Has2,15117:down|Wnt5a,22418:down|Ccl12,20293:up|Ptgs2,19225:down|Ccl4,20303:up|Bmp4,12159:up|Igf1,16000:up|Cxcl9,17329:up|Ret,19713:up|Tgfb2,21808:down|Plpp3,67916:up|F3,14066:up|Kitl,17311:up
positive regulation of cell migration	GO:0030335	2.36E-10	1.17E-08	Prl2c2,18811:up|Ccl2,20296:up|Pdgfc,54635:down|Figf,14205:up|Sema7a,20361:down|Srpx2,68792:down|Cxcl12,20315:up|Postn,50706:down|Vegfa,22339:down|Mmp2,17390:down|Lrrc15,74488:down|Gas6,14456:up|Cxcl14,57266:down|Egfr,13649:down|Fgfr1,14182:down|Has2,15117:down|Wnt5a,22418:down|Ccl12,20293:up|Ptgs2,19225:down|Ccl4,20303:up|Bmp4,12159:up|Igf1,16000:up|Cxcl9,17329:up|Ret,19713:up|Tgfb2,21808:down|Plpp3,67916:up|F3,14066:up|Kitl,17311:up
response to growth factor	GO:0070848	2.90E-10	1.42E-08	Fstl3,83554:down|Flrt2,399558:down|Hpgd,15446:up|Ccl2,20296:up|Figf,14205:up|Aspn,66695:down|Ankrd1,107765:down|Htra3,78558:down|Vegfa,22339:down|Ibsp,15891:up|Adamts12,239337:down|Cd109,235505:down|Gas6,14456:up|Akr1c18,105349:up|Col1a2,12843:down|Fgfr1,14182:down|Egfr,13649:down|Has2,15117:down|Sfrp1,20377:up|Sulf1,240725:down|Wnt5a,22418:down|Tgfbr3,21814:up|Bmpr1a,12166:down|Grem1,23892:up|Bmp4,12159:up|Col3a1,12825:down|Chst11,58250:down|Sfrp2,20319:up|Tgfb2,21808:down|Npnt,114249:up|Fgfr2,14183:up
positive regulation of locomotion	GO:0040017	3.12E-10	1.52E-08	Prl2c2,18811:up|Ccl2,20296:up|Pdgfc,54635:down|Figf,14205:up|Sema7a,20361:down|Srpx2,68792:down|Cxcl12,20315:up|Postn,50706:down|Vegfa,22339:down|Mmp2,17390:down|Lrrc15,74488:down|Gas6,14456:up|Trf,22041:up|Cxcl14,57266:down|Fgfr1,14182:down|Egfr,13649:down|Has2,15117:down|Wnt5a,22418:down|Ccl12,20293:up|Ptgs2,19225:down|Ccl4,20303:up|Bmp4,12159:up|Igf1,16000:up|Cxcl9,17329:up|Ret,19713:up|Tgfb2,21808:down|Plpp3,67916:up|F3,14066:up|Kitl,17311:up
tissue morphogenesis	GO:0048729	3.30E-10	1.59E-08	Adm,11535:up|Plxnd1,67784:up|Aldh1a2,19378:up|Mgp,17313:up|Ankrd1,107765:down|Wnt5a,22418:down|Bmpr1a,12166:down|Stc1,20855:up|Bmp4,12159:up|Igf1,16000:up|Tnc,21923:down|Ret,19713:up|Tgfb2,21808:down|Aldh1a3,56847:down|Fgfr2,14183:up|Cthrc1,68588:down|Ptk7,71461:down|Inhba,16323:down|Vdr,22337:down|Cxcl12,20315:up|Vegfa,22339:down|Mmp2,17390:down|Adamts12,239337:down|Tnf,21926:up|Col11a1,12814:down|Egfr,13649:down|Fgfr1,14182:down|Sfrp1,20377:up|Sulf1,240725:down|Tgfbr3,21814:up|Grem1,23892:up|Col3a1,12825:down|Sfrp2,20319:up|Col5a1,12831:down|Rspo2,239405:up|Npnt,114249:up
negative regulation of developmental process	GO:0051093	4.17E-10	1.99E-08	Fstl3,83554:down|Col5a2,12832:down|Clec2d,93694:up|Tmem176b,65963:up|Ccl2,20296:up|Ednrb,13618:up|Postn,50706:down|Cx3cr1,13051:down|Plk2,20620:down|Cxcl14,57266:down|Cd24a,12484:down|Wnt5a,22418:down|Bmpr1a,12166:down|Adipoq,11450:up|Bmp4,12159:up|Igf1,16000:up|Tgfb2,21808:down|Thbs2,21826:down|Fam101b,76566:down|Prl2c2,18811:up|Lrrc17,74511:down|Ccl9,20308:up|Efemp1,216616:up|Inhba,16323:down|Dpysl3,22240:down|Aspn,66695:down|Vegfa,22339:down|Adamts12,239337:down|Tnf,21926:up|Pparg,19016:up|Gas6,14456:up|Tmem176a,66058:up|Fgfr1,14182:down|Sfrp1,20377:up|Sulf1,240725:down|Tgfbr3,21814:up|Grem1,23892:up|Col3a1,12825:down|Ceacam1,26365:up|Sfrp2,20319:up|Col5a1,12831:down|Rspo2,239405:up|Tnfrsf11b,18383:up|Enpp1,18605:down
regulation of organ morphogenesis	GO:2000027	4.46E-10	2.12E-08	Cthrc1,68588:down|Ptk7,71461:down|Aspn,66695:down|Vdr,22337:down|Vegfa,22339:down|Tnf,21926:up|Fgfr1,14182:down|Sfrp1,20377:up|Sulf1,240725:down|Bmpr1a,12166:down|Wnt5a,22418:down|Grem1,23892:up|Bmp4,12159:up|Ceacam1,26365:up|Sfrp2,20319:up|Rspo2,239405:up|Tgfb2,21808:down|Tnfrsf11b,18383:up|Fgfr2,14183:up
response to endogenous stimulus	GO:0009719	4.91E-10	2.29E-08	Fstl3,83554:down|Col5a2,12832:down|Flrt2,399558:down|Hpgd,15446:up|Ccl2,20296:up|Pdgfc,54635:down|Ankrd1,107765:down|Htra3,78558:down|Sord,20322:up|Cd109,235505:down|Cdo1,12583:up|Akr1c18,105349:up|Cd24a,12484:down|Ncam1,17967:down|Wnt5a,22418:down|Bmpr1a,12166:down|Il1rn,16181:up|Adipoq,11450:up|Bmp4,12159:up|Igf1,16000:up|Chst11,58250:down|Gatm,67092:down|Tgfb2,21808:down|Ptgir,19222:up|Fgfr2,14183:up|Htr2b,15559:down|Inhba,16323:down|Aspn,66695:down|Vdr,22337:down|Htr2a,15558:down|Mmp2,17390:down|Adamts12,239337:down|Igf2,16002:up|Pappa,18491:down|Pparg,19016:up|Tnf,21926:up|Col1a2,12843:down|Alpl,11647:up|Egfr,13649:down|Fgfr1,14182:down|Sfrp1,20377:up|Hmox1,15368:up|Has2,15117:down|Sulf1,240725:down|Tgfbr3,21814:up|Grem1,23892:up|Col3a1,12825:down|Sfrp2,20319:up|Serpina3g,20715:up|Npnt,114249:up|Enpp1,18605:down
cellular response to interleukin-1	GO:0071347	5.02E-10	2.32E-08	Ccl9,20308:up|Ccl6,20305:up|Ccl7,20306:up|Ccl2,20296:up|Ccl12,20293:up|Il1rn,16181:up|Ccl4,20303:up|Ccl3,20302:up|Saa3,20210:up|Ankrd1,107765:down|Ccl8,20307:up|Adamts12,239337:down
cell chemotaxis	GO:0060326	5.05E-10	2.32E-08	Ccl9,20308:up|Ccl6,20305:up|Ccl7,20306:up|Ccl2,20296:up|Figf,14205:up|Ednrb,13618:up|Ccl3,20302:up|Saa3,20210:up|Cxcl12,20315:up|Vegfa,22339:down|Cx3cr1,13051:down|Gas6,14456:up|Cxcl14,57266:down|Fgfr1,14182:down|Wnt5a,22418:down|Ccl12,20293:up|Ccl4,20303:up|Grem1,23892:up|Ccl8,20307:up|Cxcl9,17329:up
connective tissue development	GO:0061448	6.21E-10	2.82E-08	Fam101b,76566:down|Efemp1,216616:up|Mgp,17313:up|Mmp13,17386:up|Serpinh1,12406:down|Adamts12,239337:down|Loxl2,94352:down|Col11a1,12814:down|Fgfr1,14182:down|Sulf1,240725:down|Bmpr1a,12166:down|Wnt5a,22418:down|Stc1,20855:up|Grem1,23892:up|Bmp4,12159:up|Igf1,16000:up|Chst11,58250:down|Sfrp2,20319:up|Col5a1,12831:down|Rspo2,239405:up|Tgfb2,21808:down

regulation of defense response	GO:0031347	6.23E-10	2.82E-08	Clec2d,93694:up|Ccl7,20306:up|Ccl2,20296:up|C3,12266:up|Ednrb,13618:up|Sema7a,20361:down|Cx3cr1,13051:down|Cd24a,12484:down|Wnt5a,22418:down|Ccl12,20293:up|Ccl4,20303:up|Adipoq,11450:up|Ifit1,15957:up|Il1rl1,17082:down|Ccl9,20308:up|Ccl6,20305:up|Aoah,27052:up|Ccl3,20302:up|Ctla2a,13024:down|Fabp4,11770:up|Mmp2,17390:down|Igf2,16002:up|Adamts12,239337:down|Pparg,19016:up|Tnf,21926:up|Lmcd1,30937:up|Egfr,13649:down|C1qtnf3,81799:down|Ptgs2,19225:down|Ccl8,20307:up|Ceacam1,26365:up|Serping1,12258:up
muscle cell proliferation	GO:0033002	8.70E-10	3.91E-08	Vegfa,22339:down|Mmp2,17390:down|Igfbp3,16009:up|Pparg,19016:up|Tnf,21926:up|Egfr,13649:down|Fgfr1,14182:down|Hmox1,15368:up|Ncam1,17967:down|Tgfbr3,21814:up|Bmpr1a,12166:down|Wisp1,22402:down|Ptgs2,19225:down|Adipoq,11450:up|Bmp4,12159:up|Igf1,16000:up|Tgfb2,21808:down|Ptgir,19222:up|Fgfr2,14183:up
protein phosphorylation	GO:0006468	9.19E-10	4.10E-08	Igfbp4,16010:up|Flrt2,399558:down|Sfrp4,20379:up|Ccl7,20306:up|Ccl2,20296:up|C3,12266:up|Pdgfc,54635:down|Ednrb,13618:up|Sema7a,20361:down|Igfbp3,16009:up|Cd109,235505:down|Dok2,13449:up|Plk2,20620:down|Trf,22041:up|Cd24a,12484:down|Wnt5a,22418:down|Bmpr1a,12166:down|Ccl12,20293:up|Mark1,226778:up|Il1rn,16181:up|Ccl4,20303:up|Adipoq,11450:up|Bmp4,12159:up|Igf1,16000:up|Ret,19713:up|Tgfb2,21808:down|Plpp3,67916:up|Fgfr2,14183:up|Kitl,17311:up|Htr2b,15559:down|Ccl9,20308:up|Efemp1,216616:up|Ccl6,20305:up|Inhba,16323:down|Aspn,66695:down|Ccl3,20302:up|Saa3,20210:up|Crlf1,12931:down|Fabp4,11770:up|Vegfa,22339:down|Htr2a,15558:down|Lrrc15,74488:down|Igf2,16002:up|Gas6,14456:up|Tnf,21926:up|Fgfr1,14182:down|Egfr,13649:down|Sfrp1,20377:up|C1qtnf3,81799:down|Cd4,12504:up|Tgfbr3,21814:up|Grem1,23892:up|Ceacam1,26365:up|Ccl8,20307:up|Sfrp2,20319:up|Fam20a,208659:down|Npnt,114249:up|Tnfrsf11b,18383:up|Prkar2b,19088:up|Errfi1,74155:down|Enpp1,18605:down
mesenchymal cell development	GO:0014031	9.93E-10	4.40E-08	Fam101b,76566:down|Htr2b,15559:down|Aldh1a2,19378:up|Ednrb,13618:up|Sema7a,20361:down|Loxl2,94352:down|Loxl3,16950:down|Sfrp1,20377:up|Has2,15117:down|Tgfbr3,21814:up|Bmpr1a,12166:down|Wnt5a,22418:down|Grem1,23892:up|Sfrp2,20319:up|Ret,19713:up|Tgfb2,21808:down|Fgfr2,14183:up|Kitl,17311:up
bone mineralization	GO:0030282	1.02E-09	4.47E-08	Fam101b,76566:down|Bmpr1a,12166:down|Ptgs2,19225:down|Mgp,17313:up|Aspn,66695:down|Grem1,23892:up|Mmp13,17386:up|Igf1,16000:up|Bmp4,12159:up|Ank,11732:down|Gpm6b,14758:up|Rspo2,239405:up|Ibsp,15891:up|Fgfr2,14183:up
negative regulation of biological process	GO:0048519	1.10E-09	4.81E-08	Fstl3,83554:down|Clec2d,93694:up|Adm,11535:up|Flrt2,399558:down|Tmem176b,65963:up|Hpgd,15446:up|Sfrp4,20379:up|Ankrd1,107765:down|Cx3cr1,13051:down|Igfbp3,16009:up|Cd109,235505:down|Loxl2,94352:down|Plk2,20620:down|Loxl3,16950:down|Cxcl14,57266:down|Cd24a,12484:down|Wnt5a,22418:down|Stc1,20855:up|Il1rn,16181:up|Maob,109731:up|Adipoq,11450:up|Bmp4,12159:up|Igf1,16000:up|Oasl1,231655:up|Tgfb2,21808:down|Ptgir,19222:up|Fgfr2,14183:up|Thbs2,21826:down|Cthrc1,68588:down|Htr2b,15559:down|Kng1,16644:up|Prl2c2,18811:up|Efemp1,216616:up|Dpysl3,22240:down|Slpi,20568:up|Aspn,66695:down|Vdr,22337:down|Fabp4,11770:up|Cxcl12,20315:up|Nabp1,109019:down|Htr2a,15558:down|Mmp2,17390:down|Oas3,246727:up|Slc40a1,53945:up|Gas6,14456:up|Egfr,13649:down|Sfrp1,20377:up|C1qtnf3,81799:down|Tgfbr3,21814:up|Ceacam1,26365:up|Sfrp2,20319:up|Serpina3g,20715:up|Rspo2,239405:up|Tnfrsf11b,18383:up|Prkar2b,19088:up|Mt1,17748:up|S100a1,20193:up|Col5a2,12832:down|Aldh1a2,19378:up|Ccl2,20296:up|Cst7,13011:up|Ednrb,13618:up|Epas1,13819:up|Htra3,78558:down|Gpm6b,14758:up|Postn,50706:down|Ptx3,19288:up|Akr1c18,105349:up|Ncam1,17967:down|Ccl12,20293:up|Bmpr1a,12166:down|Wisp1,22402:down|Ccl4,20303:up|Chst11,58250:down|Tnc,21923:down|Prg4,96875:down|Pdxk,216134:up|Plpp3,67916:up|Creb3l1,26427:down|Il1rl1,17082:down|Kitl,17311:up|Fam101b,76566:down|Lrrc17,74511:down|Hp,15439:up|Ccl9,20308:up|Inhba,16323:down|Aoah,27052:up|Plat,18791:down|Tfpi2,21789:down|Ctla2a,13024:down|Crlf1,12931:down|Vegfa,22339:down|Serpine2,20720:up|Adamts12,239337:down|Lrrc15,74488:down|Nxn,18230:down|Igf2,16002:up|Anpep,16790:up|Kdelr3,105785:down|Pparg,19016:up|Tnf,21926:up|Tmem176a,66058:up|Glp1r,14652:down|Lmcd1,30937:up|Tnfrsf23,79201:down|Fgfr1,14182:down|Hmox1,15368:up|Sulf1,240725:down|Susd2,71733:up|Ptgs2,19225:down|Grem1,23892:up|Col3a1,12825:down|Col5a1,12831:down|Serping1,12258:up|Errfi1,74155:down|Enpp1,18605:down
lymphocyte migration	GO:0072676	1.12E-09	4.86E-08	Gas6,14456:up|Cxcl14,57266:down|Ccl9,20308:up|Ccl6,20305:up|Wnt5a,22418:down|Ccl7,20306:up|Ccl2,20296:up|Ccl12,20293:up|Ccl4,20303:up|Ccl3,20302:up|Ccl8,20307:up|Cxcl12,20315:up
epithelium development	GO:0060429	1.22E-09	5.23E-08	Adm,11535:up|Plxnd1,67784:up|Sfrp4,20379:up|Aldh1a2,19378:up|Mgp,17313:up|Ednrb,13618:up|Cd109,235505:down|Wnt5a,22418:down|Bmpr1a,12166:down|Stc1,20855:up|Adipoq,11450:up|Bmp4,12159:up|Igf1,16000:up|Tnc,21923:down|Ret,19713:up|Tgfb2,21808:down|Hpse,15442:up|Aldh1a3,56847:down|Fgfr2,14183:up|Kitl,17311:up|Cthrc1,68588:down|Ptk7,71461:down|Inhba,16323:down|Vdr,22337:down|Crlf1,12931:down|Cxcl12,20315:up|Vegfa,22339:down|Mmp2,17390:down|Serpine2,20720:up|Adamts12,239337:down|Slc40a1,53945:up|Pparg,19016:up|Tnf,21926:up|Egfr,13649:down|Fgfr1,14182:down|Sfrp1,20377:up|Sulf1,240725:down|Tgfbr3,21814:up|Ptgs2,19225:down|Grem1,23892:up|Ceacam1,26365:up|Sfrp2,20319:up|Mcoln3,171166:up|Col5a1,12831:down|Rspo2,239405:up|Npnt,114249:up|Errfi1,74155:down
chemokine-mediated signaling pathway	GO:0070098	1.26E-09	5.36E-08	Ccl9,20308:up|Ccl6,20305:up|Ccl7,20306:up|Ccl2,20296:up|Ccl12,20293:up|Ccl4,20303:up|Ccl3,20302:up|Ccl8,20307:up|Cxcl9,17329:up|Cxcl12,20315:up
monocyte chemotaxis	GO:0002548	1.26E-09	5.36E-08	Ccl9,20308:up|Ccl6,20305:up|Ccl7,20306:up|Ccl2,20296:up|Ccl12,20293:up|Grem1,23892:up|Ccl4,20303:up|Ccl3,20302:up|Ccl8,20307:up|Cxcl12,20315:up
regulation of phosphate metabolic process	GO:0019220	1.30E-09	5.49E-08	Igfbp4,16010:up|Adm,11535:up|Flrt2,399558:down|Sfrp4,20379:up|Ccl7,20306:up|Ccl2,20296:up|C3,12266:up|Pdgfc,54635:down|Ednrb,13618:up|Sema7a,20361:down|Igfbp3,16009:up|Cd109,235505:down|Trf,22041:up|Cd24a,12484:down|Wnt5a,22418:down|Bmpr1a,12166:down|Ccl12,20293:up|Il1rn,16181:up|Ccl4,20303:up|Adipoq,11450:up|Bmp4,12159:up|Igf1,16000:up|Ret,19713:up|Tgfb2,21808:down|Plpp3,67916:up|Ptgir,19222:up|Fgfr2,14183:up|Kitl,17311:up|Htr2b,15559:down|Ccl9,20308:up|Ccl6,20305:up|Inhba,16323:down|Aspn,66695:down|Ccl3,20302:up|Srpx2,68792:down|Crlf1,12931:down|Fabp4,11770:up|Vegfa,22339:down|Htr2a,15558:down|Lrrc15,74488:down|Igf2,16002:up|Gas6,14456:up|Tnf,21926:up|Fgfr1,14182:down|Egfr,13649:down|Sfrp1,20377:up|C1qtnf3,81799:down|Cd4,12504:up|Grem1,23892:up|Ceacam1,26365:up|Ccl8,20307:up|Sfrp2,20319:up|Fam20a,208659:down|Npnt,114249:up|Tnfrsf11b,18383:up|Prkar2b,19088:up|Errfi1,74155:down|Enpp1,18605:down
positive regulation of protein modification process	GO:0031401	1.43E-09	6.00E-08	Igfbp4,16010:up|Ccl7,20306:up|Ccl2,20296:up|C3,12266:up|Pdgfc,54635:down|Ednrb,13618:up|Sema7a,20361:down|Igfbp3,16009:up|Trf,22041:up|Cd24a,12484:down|Wnt5a,22418:down|Bmpr1a,12166:down|Ccl12,20293:up|Il1rn,16181:up|Ccl4,20303:up|Adipoq,11450:up|Bmp4,12159:up|Igf1,16000:up|Ret,19713:up|Tgfb2,21808:down|Plpp3,67916:up|Fgfr2,14183:up|Kitl,17311:up|Htr2b,15559:down|Ccl9,20308:up|Ccl6,20305:up|Inhba,16323:down|Ccl3,20302:up|Crlf1,12931:down|Vegfa,22339:down|Htr2a,15558:down|Igf2,16002:up|Gas6,14456:up|Tnf,21926:up|Fgfr1,14182:down|Egfr,13649:down|C1qtnf3,81799:down|Cd4,12504:up|Grem1,23892:up|Ccl8,20307:up|Ceacam1,26365:up|Sfrp2,20319:up|Fam20a,208659:down|Tnfrsf11b,18383:up|Npnt,114249:up

regulation of phosphorus metabolic process	GO:0051174	1.49E-09	6.19E-08	Igfbp4,16010:up|Adm,11535:up|Flrt2,399558:down|Sfrp4,20379:up|Ccl7,20306:up|Ccl2,20296:up|C3,12266:up|Pdgfc,54635:down|Ednrb,13618:up|Sema7a,20361:down|Igfbp3,16009:up|Cd109,235505:down|Trf,22041:up|Cd24a,12484:down|Wnt5a,22418:down|Bmpr1a,12166:down|Ccl12,20293:up|Il1rn,16181:up|Ccl4,20303:up|Adipoq,11450:up|Bmp4,12159:up|Igf1,16000:up|Ret,19713:up|Tgfb2,21808:down|Plpp3,67916:up|Ptgir,19222:up|Fgfr2,14183:up|Kitl,17311:up|Htr2b,15559:down|Ccl9,20308:up|Ccl6,20305:up|Inhba,16323:down|Aspn,66695:down|Ccl3,20302:up|Srpx2,68792:down|Crlf1,12931:down|Fabp4,11770:up|Vegfa,22339:down|Htr2a,15558:down|Lrrc15,74488:down|Igf2,16002:up|Gas6,14456:up|Tnf,21926:up|Fgfr1,14182:down|Egfr,13649:down|Sfrp1,20377:up|C1qtnf3,81799:down|Cd4,12504:up|Grem1,23892:up|Ceacam1,26365:up|Ccl8,20307:up|Sfrp2,20319:up|Fam20a,208659:down|Npnt,114249:up|Tnfrsf11b,18383:up|Prkar2b,19088:up|Errfi1,74155:down|Enpp1,18605:down
leukocyte chemotaxis	GO:0030595	1.61E-09	6.64E-08	Ccl9,20308:up|Ccl6,20305:up|Ccl7,20306:up|Ccl2,20296:up|Figf,14205:up|Ednrb,13618:up|Ccl3,20302:up|Cxcl12,20315:up|Cx3cr1,13051:down|Gas6,14456:up|Cxcl14,57266:down|Ccl12,20293:up|Wnt5a,22418:down|Ccl4,20303:up|Grem1,23892:up|Ccl8,20307:up|Cxcl9,17329:up
regulation of epithelial cell proliferation	GO:0050678	1.72E-09	7.07E-08	Htr2b,15559:down|Ccl2,20296:up|Aldh1a2,19378:up|Ednrb,13618:up|Vdr,22337:down|Cxcl12,20315:up|Vegfa,22339:down|Cd109,235505:down|Egfr,13649:down|Sfrp1,20377:up|Has2,15117:down|Sulf1,240725:down|Wnt5a,22418:down|Tgfbr3,21814:up|Bmpr1a,12166:down|Bmp4,12159:up|Igf1,16000:up|Ceacam1,26365:up|Sfrp2,20319:up|Tgfb2,21808:down|Errfi1,74155:down|Fgfr2,14183:up|F3,14066:up
regulation of immune system process	GO:0002682	1.79E-09	7.29E-08	Fstl3,83554:down|Clec2d,93694:up|Tmem176b,65963:up|Ccl2,20296:up|C3,12266:up|Figf,14205:up|Sema7a,20361:down|Cxcl14,57266:down|Cd24a,12484:down|Wnt5a,22418:down|Ccl12,20293:up|Ccl4,20303:up|Adipoq,11450:up|Bmp4,12159:up|Igf1,16000:up|Cxcl9,17329:up|Tgfb2,21808:down|C1s2,317677:up|Ifit1,15957:up|Il1rl1,17082:down|Clec4e,56619:up|Kitl,17311:up|Lrrc17,74511:down|Inhba,16323:down|Ccl3,20302:up|Ctla2a,13024:down|C1s1,50908:up|Cxcl12,20315:up|Vegfa,22339:down|Phf11a,219131:up|Mmp2,17390:down|Igf2,16002:up|Pparg,19016:up|Gas6,14456:up|Tnf,21926:up|Tmem176a,66058:up|Lmcd1,30937:up|Hmox1,15368:up|Sfrp1,20377:up|Cd4,12504:up|C1rb,667277:up|Tfrc,22042:up|Grem1,23892:up|Col3a1,12825:down|Ceacam1,26365:up|Serping1,12258:up|Tnfsf9,21950:up
anatomical structure formation involved in morphogenesis	GO:0048646	1.99E-09	8.04E-08	Col5a2,12832:down|Adm,11535:up|Plxnd1,67784:up|Ccl2,20296:up|C3,12266:up|Figf,14205:up|Epas1,13819:up|Tnfrsf12a,27279:down|Ddah1,69219:down|Cx3cr1,13051:down|Cd109,235505:down|Loxl2,94352:down|Wnt5a,22418:down|Bmpr1a,12166:down|Ccl12,20293:up|Col8a1,12837:down|Bmp4,12159:up|Cxcl9,17329:up|Ret,19713:up|Tgfb2,21808:down|Vegfb,22340:up|Hpse,15442:up|Aldh1a3,56847:down|Fgfr2,14183:up|F3,14066:up|Thbs2,21826:down|Cthrc1,68588:down|Prl2c2,18811:up|Ptk7,71461:down|Inhba,16323:down|Srpx2,68792:down|Cxcl12,20315:up|Vegfa,22339:down|Mmp2,17390:down|Anpep,16790:up|Slc40a1,53945:up|Tnf,21926:up|Fgfr1,14182:down|Hmox1,15368:up|Sfrp1,20377:up|Sulf1,240725:down|Tgfbr3,21814:up|Ptgs2,19225:down|Grem1,23892:up|Ccl8,20307:up|Ceacam1,26365:up|Sfrp2,20319:up|Col5a1,12831:down|Fam20a,208659:down
tissue remodeling	GO:0048771	2.43E-09	9.75E-08	Cthrc1,68588:down|Epas1,13819:up|Vdr,22337:down|Vegfa,22339:down|Mmp2,17390:down|Trf,22041:up|Cd24a,12484:down|Egfr,13649:down|Sfrp1,20377:up|Tgfbr3,21814:up|Tfrc,22042:up|Grem1,23892:up|Igf1,16000:up|Ceacam1,26365:up|Tgfb2,21808:down|Tnfrsf11b,18383:up|Enpp1,18605:down
regulation of vasculature development	GO:1901342	2.98E-09	1.19E-07	Prl2c2,18811:up|Adm,11535:up|Plxnd1,67784:up|Ccl2,20296:up|C3,12266:up|Tnfrsf12a,27279:down|Srpx2,68792:down|Ddah1,69219:down|Vegfa,22339:down|Cx3cr1,13051:down|Hmox1,15368:up|Sulf1,240725:down|Wnt5a,22418:down|Ptgs2,19225:down|Grem1,23892:up|Bmp4,12159:up|Ceacam1,26365:up|Sfrp2,20319:up|F3,14066:up|Thbs2,21826:down
regulation of angiogenesis	GO:0045765	3.29E-09	1.31E-07	Prl2c2,18811:up|Adm,11535:up|Plxnd1,67784:up|Ccl2,20296:up|C3,12266:up|Tnfrsf12a,27279:down|Srpx2,68792:down|Ddah1,69219:down|Vegfa,22339:down|Cx3cr1,13051:down|Hmox1,15368:up|Sulf1,240725:down|Wnt5a,22418:down|Ptgs2,19225:down|Grem1,23892:up|Ceacam1,26365:up|Sfrp2,20319:up|F3,14066:up|Thbs2,21826:down
cytokine production	GO:0001816	3.57E-09	1.40E-07	Ly9,17085:up|Ccl2,20296:up|C3,12266:up|Figf,14205:up|Sema7a,20361:down|Clec4n,56620:up|Postn,50706:down|Cd24a,12484:down|Wnt5a,22418:down|Ccl4,20303:up|Adipoq,11450:up|Prg4,96875:down|Tgfb2,21808:down|Hpse,15442:up|Il1rl1,17082:down|Clec4e,56619:up|Htr2b,15559:down|Ccl3,20302:up|Fabp4,11770:up|Tnf,21926:up|Pparg,19016:up|Gas6,14456:up|Fgfr1,14182:down|Lpl,16956:up|Hmox1,15368:up|Cd4,12504:up|C1qtnf3,81799:down|Sulf1,240725:down|Ptgs2,19225:down|Ceacam1,26365:up|Errfi1,74155:down|Tnfsf9,21950:up
positive regulation of biological process	GO:0048518	3.57E-09	1.40E-07	Fstl3,83554:down|Igfbp4,16010:up|Adm,11535:up|Flrt2,399558:down|Sfrp4,20379:up|Lyl1,17095:up|Igfbp3,16009:up|Plk2,20620:down|Cd24a,12484:down|Il1rn,16181:up|Maob,109731:up|Bmp4,12159:up|Hpse,15442:up|Medag,70717:down|Ptgir,19222:up|Fgfr2,14183:up|Thbs2,21826:down|Htr2b,15559:down|Ccl6,20305:up|Fam132b,227358:down|Mmp2,17390:down|Slc40a1,53945:up|Egfr,13649:down|C1rb,667277:up|Tfrc,22042:up|Ccl8,20307:up|Rspo2,239405:up|Fam20a,208659:down|Aldh1a2,19378:up|Ccl2,20296:up|Ednrb,13618:up|Mmp13,17386:up|Tnfrsf12a,27279:down|Clec4n,56620:up|Ptx3,19288:up|Ncam1,17967:down|Ccl4,20303:up|Vegfb,22340:up|Aldh1a3,56847:down|Plpp3,67916:up|Lama2,16773:down|Ccl9,20308:up|Ptk7,71461:down|Inhba,16323:down|Crlf1,12931:down|C1s1,50908:up|Plod2,26432:down|Vegfa,22339:down|Serpine2,20720:up|Igf2,16002:up|Tnf,21926:up|Fxyd1,56188:up|Fgfr1,14182:down|Lpl,16956:up|Sulf1,240725:down|Cd4,12504:up|Col3a1,12825:down|Serping1,12258:up|C3,12266:up|Ankrd1,107765:down|Cx3cr1,13051:down|Trf,22041:up|Loxl2,94352:down|Cxcl14,57266:down|Wnt5a,22418:down|Stc1,20855:up|Col8a1,12837:down|Adipoq,11450:up|Igf1,16000:up|Crabp2,12904:down|Cxcl9,17329:up|Tgfb2,21808:down|F3,14066:up|Clec4e,56619:up|Prl2c2,18811:up|Cthrc1,68588:down|Efemp1,216616:up|Dpysl3,22240:down|Ninj1,18081:up|Vdr,22337:down|Fabp4,11770:up|Cxcl12,20315:up|Htr2a,15558:down|Smoc2,64074:down|Gas6,14456:up|Sfrp1,20377:up|Tgfbr3,21814:up|C1qtnf3,81799:down|Serinc2,230779:down|Ceacam1,26365:up|Sfrp2,20319:up|Tnfrsf11b,18383:up|Npnt,114249:up|S100a1,20193:up|Ice1,218333:down|Ly9,17085:up|Ccl7,20306:up|Figf,14205:up|Pdgfc,54635:down|Epas1,13819:up|Sema7a,20361:down|Gpm6b,14758:up|Ddah1,69219:down|Sdpr,20324:down|Postn,50706:down|Akr1c18,105349:up|Ccl12,20293:up|Bmpr1a,12166:down|Wisp1,22402:down|Tnc,21923:down|Ret,19713:up|C1s2,317677:up|Creb3l1,26427:down|Kitl,17311:up|Il1rl1,17082:down|Hp,15439:up|Rasl11a,68895:up|Ccl3,20302:up|Plat,18791:down|Srpx2,68792:down|Lrrc15,74488:down|Pparg,19016:up|Lmcd1,30937:up|Glp1r,14652:down|Hmox1,15368:up|Has2,15117:down|Ptgs2,19225:down|Grem1,23892:up|Tnfsf9,21950:up

positive regulation of cellular process	GO:0048522	4.26E-09	1.66E-07	Fstl3,83554:down|Igfbp4,16010:up|Adm,11535:up|Flrt2,399558:down|Sfrp4,20379:up|C3,12266:up|Ankrd1,107765:down|Lyl1,17095:up|Cx3cr1,13051:down|Igfbp3,16009:up|Loxl2,94352:down|Plk2,20620:down|Trf,22041:up|Cxcl14,57266:down|Cd24a,12484:down|Wnt5a,22418:down|Stc1,20855:up|Il1rn,16181:up|Col8a1,12837:down|Maob,109731:up|Adipoq,11450:up|Bmp4,12159:up|Igf1,16000:up|Crabp2,12904:down|Cxcl9,17329:up|Tgfb2,21808:down|Hpse,15442:up|Medag,70717:down|Ptgir,19222:up|F3,14066:up|Fgfr2,14183:up|Thbs2,21826:down|Clec4e,56619:up|Cthrc1,68588:down|Htr2b,15559:down|Prl2c2,18811:up|Efemp1,216616:up|Ccl6,20305:up|Dpysl3,22240:down|Ninj1,18081:up|Vdr,22337:down|Fabp4,11770:up|Cxcl12,20315:up|Htr2a,15558:down|Mmp2,17390:down|Smoc2,64074:down|Slc40a1,53945:up|Gas6,14456:up|Egfr,13649:down|Sfrp1,20377:up|C1qtnf3,81799:down|Tgfbr3,21814:up|Tfrc,22042:up|Ceacam1,26365:up|Ccl8,20307:up|Sfrp2,20319:up|Rspo2,239405:up|Fam20a,208659:down|Npnt,114249:up|Tnfrsf11b,18383:up|S100a1,20193:up|Ice1,218333:down|Aldh1a2,19378:up|Ccl2,20296:up|Ccl7,20306:up|Pdgfc,54635:down|Ednrb,13618:up|Figf,14205:up|Mmp13,17386:up|Sema7a,20361:down|Epas1,13819:up|Tnfrsf12a,27279:down|Ddah1,69219:down|Clec4n,56620:up|Sdpr,20324:down|Postn,50706:down|Ptx3,19288:up|Akr1c18,105349:up|Ncam1,17967:down|Bmpr1a,12166:down|Ccl12,20293:up|Wisp1,22402:down|Ccl4,20303:up|Tnc,21923:down|Ret,19713:up|Vegfb,22340:up|Aldh1a3,56847:down|Plpp3,67916:up|Creb3l1,26427:down|Il1rl1,17082:down|Kitl,17311:up|Lama2,16773:down|Hp,15439:up|Ccl9,20308:up|Ptk7,71461:down|Inhba,16323:down|Rasl11a,68895:up|Ccl3,20302:up|Srpx2,68792:down|Crlf1,12931:down|Plod2,26432:down|Vegfa,22339:down|Serpine2,20720:up|Lrrc15,74488:down|Igf2,16002:up|Pparg,19016:up|Tnf,21926:up|Glp1r,14652:down|Lmcd1,30937:up|Fxyd1,56188:up|Fgfr1,14182:down|Has2,15117:down|Lpl,16956:up|Hmox1,15368:up|Sulf1,240725:down|Cd4,12504:up|Ptgs2,19225:down|Grem1,23892:up|Col3a1,12825:down|Tnfsf9,21950:up
innate immune response	GO:0045087	4.30E-09	1.66E-07	Clec2d,93694:up|Ccl9,20308:up|Ly9,17085:up|Ccl6,20305:up|Mrc1,17533:up|Ccl7,20306:up|Ccl2,20296:up|C3,12266:up|Slpi,20568:up|Ccl3,20302:up|Ifit3,15959:up|Clec4n,56620:up|C1s1,50908:up|Mmp2,17390:down|Oas3,246727:up|Igf2,16002:up|Tnf,21926:up|Pparg,19016:up|Ptx3,19288:up|Wnt5a,22418:down|Ccl12,20293:up|C1rb,667277:up|Ccl4,20303:up|Ceacam1,26365:up|Ccl8,20307:up|Serping1,12258:up|Oasl1,231655:up|C1s2,317677:up|Ifit1,15957:up|Clec4e,56619:up
chondrocyte differentiation	GO:0002062	4.43E-09	1.70E-07	Fam101b,76566:down|Loxl2,94352:down|Fgfr1,14182:down|Col11a1,12814:down|Efemp1,216616:up|Sulf1,240725:down|Bmpr1a,12166:down|Grem1,23892:up|Bmp4,12159:up|Chst11,58250:down|Serpinh1,12406:down|Sfrp2,20319:up|Adamts12,239337:down
cellular response to interferon-gamma	GO:0071346	4.56E-09	1.74E-07	Pparg,19016:up|Ccl9,20308:up|Ccl6,20305:up|Mrc1,17533:up|Ccl7,20306:up|Ccl2,20296:up|Ccl12,20293:up|Ccl4,20303:up|Ccl3,20302:up|Ccl8,20307:up
regulation of ossification	GO:0030278	4.89E-09	1.86E-07	Fam101b,76566:down|Cthrc1,68588:down|Mgp,17313:up|Gpm6b,14758:up|Vegfa,22339:down|Tnf,21926:up|Fgfr1,14182:down|Sfrp1,20377:up|Bmpr1a,12166:down|Wnt5a,22418:down|Grem1,23892:up|Bmp4,12159:up|Igf1,16000:up|Sfrp2,20319:up|Ank,11732:down|Npnt,114249:up|Enpp1,18605:down|Creb3l1,26427:down
regulation of cytokine production	GO:0001817	4.96E-09	1.87E-07	Htr2b,15559:down|Ly9,17085:up|Ccl2,20296:up|C3,12266:up|Figf,14205:up|Ccl3,20302:up|Sema7a,20361:down|Clec4n,56620:up|Postn,50706:down|Gas6,14456:up|Pparg,19016:up|Tnf,21926:up|Fgfr1,14182:down|Cd24a,12484:down|Hmox1,15368:up|Lpl,16956:up|Sulf1,240725:down|C1qtnf3,81799:down|Wnt5a,22418:down|Ptgs2,19225:down|Ccl4,20303:up|Adipoq,11450:up|Ceacam1,26365:up|Prg4,96875:down|Tgfb2,21808:down|Hpse,15442:up|Errfi1,74155:down|Il1rl1,17082:down|Tnfsf9,21950:up|Clec4e,56619:up
morphogenesis of a branching epithelium	GO:0061138	4.99E-09	1.87E-07	Adm,11535:up|Plxnd1,67784:up|Mgp,17313:up|Vdr,22337:down|Cxcl12,20315:up|Vegfa,22339:down|Tnf,21926:up|Fgfr1,14182:down|Sfrp1,20377:up|Sulf1,240725:down|Wnt5a,22418:down|Grem1,23892:up|Bmp4,12159:up|Igf1,16000:up|Sfrp2,20319:up|Tnc,21923:down|Rspo2,239405:up|Npnt,114249:up|Fgfr2,14183:up
response to interleukin-1	GO:0070555	5.59E-09	2.08E-07	Ccl9,20308:up|Ccl6,20305:up|Ccl7,20306:up|Ccl2,20296:up|Ccl12,20293:up|Il1rn,16181:up|Ccl4,20303:up|Ccl3,20302:up|Saa3,20210:up|Ankrd1,107765:down|Ccl8,20307:up|Adamts12,239337:down
regulation of biological quality	GO:0065008	5.72E-09	2.12E-07	Adm,11535:up|Flrt2,399558:down|Plxnd1,67784:up|Sfrp4,20379:up|C3,12266:up|Cx3cr1,13051:down|Plk2,20620:down|Trf,22041:up|Cd24a,12484:down|Wnt5a,22418:down|Stc1,20855:up|Il1rn,16181:up|Maob,109731:up|Igf1,16000:up|Adipoq,11450:up|Bmp4,12159:up|Crabp2,12904:down|Tgfb2,21808:down|Hpse,15442:up|Ptgir,19222:up|F3,14066:up|Thbs2,21826:down|Htr2b,15559:down|Kng1,16644:up|Anxa8,11752:down|Mt2,17750:up|Vdr,22337:down|Pdia5,72599:down|Fabp4,11770:up|Fam132b,227358:down|Cxcl12,20315:up|Htr2a,15558:down|Mmp2,17390:down|Slc40a1,53945:up|Gas6,14456:up|Egfr,13649:down|Sfrp1,20377:up|C1qtnf3,81799:down|Tgfbr3,21814:up|Tfrc,22042:up|Sncg,20618:up|Ceacam1,26365:up|Fam20a,208659:down|Tnfrsf11b,18383:up|Syt13,80976:up|Mt1,17748:up|Aldh1a2,19378:up|Ccl2,20296:up|Pdgfc,54635:down|Ednrb,13618:up|Ampd3,11717:up|Mmp13,17386:up|Sema7a,20361:down|Epas1,13819:up|Slc29a1,63959:up|Tnfrsf12a,27279:down|Gpm6b,14758:up|Ddah1,69219:down|Steap1,70358:down|Postn,50706:down|Cdo1,12583:up|Ptx3,19288:up|Akr1c18,105349:up|Ncam1,17967:down|Ccl12,20293:up|Ccl4,20303:up|Ret,19713:up|Aldh1a3,56847:down|Plpp3,67916:up|Kitl,17311:up|Inhba,16323:down|Ccl3,20302:up|Tfpi2,21789:down|Srpx2,68792:down|Vegfa,22339:down|Serpine2,20720:up|Nxn,18230:down|Igf2,16002:up|Anpep,16790:up|Kdelr3,105785:down|Pparg,19016:up|Tnf,21926:up|Fxyd1,56188:up|Col1a2,12843:down|Glp1r,14652:down|Fgfr1,14182:down|Lpl,16956:up|Hmox1,15368:up|Cd4,12504:up|Ptgs2,19225:down|Serping1,12258:up|Enpp1,18605:down
regulation of cell adhesion	GO:0030155	6.63E-09	2.42E-07	Fstl3,83554:down|Ccl2,20296:up|Gpm6b,14758:up|Postn,50706:down|Cd24a,12484:down|Wnt5a,22418:down|Il1rn,16181:up|Col8a1,12837:down|Adipoq,11450:up|Bmp4,12159:up|Igf1,16000:up|Tnc,21923:down|Ret,19713:up|Tgfb2,21808:down|Plpp3,67916:up|Lama2,16773:down|Ninj1,18081:up|Ctla2a,13024:down|Cxcl12,20315:up|Vegfa,22339:down|Serpine2,20720:up|Mmp2,17390:down|Igf2,16002:up|Smoc2,64074:down|Tnf,21926:up|Has2,15117:down|Cd4,12504:up|Tfrc,22042:up|Grem1,23892:up|Ceacam1,26365:up|Npnt,114249:up|Tnfsf9,21950:up
regulation of smooth muscle cell proliferation	GO:0048660	6.68E-09	2.43E-07	Tnf,21926:up|Pparg,19016:up|Egfr,13649:down|Hmox1,15368:up|Bmpr1a,12166:down|Wisp1,22402:down|Ptgs2,19225:down|Igf1,16000:up|Bmp4,12159:up|Adipoq,11450:up|Vegfa,22339:down|Mmp2,17390:down|Igfbp3,16009:up|Ptgir,19222:up|Fgfr2,14183:up
positive regulation of transport	GO:0051050	7.05E-09	2.55E-07	Ice1,218333:down|Sfrp4,20379:up|Ccl2,20296:up|C3,12266:up|Ednrb,13618:up|Mmp13,17386:up|Ankrd1,107765:down|Clec4n,56620:up|Postn,50706:down|Trf,22041:up|Ptx3,19288:up|Wnt5a,22418:down|Bmpr1a,12166:down|Ccl12,20293:up|Stc1,20855:up|Ccl4,20303:up|Adipoq,11450:up|Bmp4,12159:up|Igf1,16000:up|Tgfb2,21808:down|Il1rl1,17082:down|Clec4e,56619:up|Htr2b,15559:down|Ccl3,20302:up|Fam132b,227358:down|Cxcl12,20315:up|Vegfa,22339:down|Pparg,19016:up|Gas6,14456:up|Tnf,21926:up|Fxyd1,56188:up|Glp1r,14652:down|Egfr,13649:down|Fgfr1,14182:down|Lpl,16956:up|C1qtnf3,81799:down|Cd4,12504:up|Ptgs2,19225:down|Grem1,23892:up|Ceacam1,26365:up|Sfrp2,20319:up|S100a1,20193:up

<table>
<tr><td>wound healing</td><td>GO:0042060</td><td>7.29E-09</td><td>2.62E-07</td><td>Kng1,16644:up|Ptk7,71461:down|C3,12266:up|Pdgfc,54635:down|Anxa8,11752:down|Ninj1,18081:up|Tfpi2,21789:down|Tnfrsf12a,27279:down|Serpine2,20720:up|Cd109,235505:down|Gas6,14456:up|Egfr,13649:down|Hmox1,15368:up|Wnt5a,22418:down|Igf1,16000:up|Col3a1,12825:down|Ceacam1,26365:up|Serping1,12258:up|Col5a1,12831:down|Tgfb2,21808:down|Hpse,15442:up|Plpp3,67916:up|F3,14066:up</td></tr>
<tr><td>blood circulation</td><td>GO:0008015</td><td>7.95E-09</td><td>2.81E-07</td><td>Htr2b,15559:down|Kng1,16644:up|Adm,11535:up|Ednrb,13618:up|Epas1,13819:up|Ddah1,69219:down|Postn,50706:down|Vegfa,22339:down|Htr2a,15558:down|Mmp2,17390:down|Gas6,14456:up|Pparg,19016:up|Glp1r,14652:down|Fxyd1,56188:up|Col1a2,12843:down|Egfr,13649:down|Hmox1,15368:up|Tgfbr3,21814:up|Ptgs2,19225:down|Stc1,20855:up|Ccl4,20303:up|Adipoq,11450:up|Ceacam1,26365:up|Tgfb2,21808:down|Vegfb,22340:up|S100a1,20193:up</td></tr>
<tr><td>regulation of protein modification process</td><td>GO:0031399</td><td>7.97E-09</td><td>2.81E-07</td><td>Igfbp4,16010:up|Flrt2,399558:down|Sfrp4,20379:up|Ccl7,20306:up|Ccl2,20296:up|C3,12266:up|Pdgfc,54635:down|Ednrb,13618:up|Sema7a,20361:down|Igfbp3,16009:up|Cd109,235505:down|Trf,22041:up|Cd24a,12484:down|Wnt5a,22418:down|Bmpr1a,12166:down|Ccl12,20293:up|Il1rn,16181:up|Ccl4,20303:up|Adipoq,11450:up|Bmp4,12159:up|Igf1,16000:up|Ret,19713:up|Tgfb2,21808:down|Plpp3,67916:up|Fgfr2,14183:up|Kitl,17311:up|Htr2b,15559:down|Ccl9,20308:up|Ccl6,20305:up|Inhba,16323:down|Aspn,66695:down|Ccl3,20302:up|Crlf1,12931:down|Fabp4,11770:up|Vegfa,22339:down|Htr2a,15558:down|Lrrc15,74488:down|Nxn,18230:down|Igf2,16002:up|Gas6,14456:up|Tnf,21926:up|Fgfr1,14182:down|Egfr,13649:down|Sfrp1,20377:up|C1qtnf3,81799:down|Cd4,12504:up|Grem1,23892:up|Ccl8,20307:up|Ceacam1,26365:up|Sfrp2,20319:up|Fam20a,208659:down|Npnt,114249:up|Prkar2b,19088:up|Tnfrsf11b,18383:up|Errfi1,74155:down|Enpp1,18605:down</td></tr>
<tr><td>negative regulation of cellular process</td><td>GO:0048523</td><td>8.55E-09</td><td>3.00E-07</td><td>Fstl3,83554:down|Clec2d,93694:up|Adm,11535:up|Flrt2,399558:down|Tmem176b,65963:up|Hpgd,15446:up|Sfrp4,20379:up|Ankrd1,107765:down|Cx3cr1,13051:down|Igfbp3,16009:up|Cd109,235505:down|Loxl2,94352:down|Plk2,20620:down|Loxl3,16950:down|Cxcl14,57266:down|Cd24a,12484:down|Wnt5a,22418:down|Stc1,20855:up|Il1rn,16181:up|Maob,109731:up|Adipoq,11450:up|Bmp4,12159:up|Igf1,16000:up|Oasl1,231655:up|Tgfb2,21808:down|Ptgir,19222:up|Fgfr2,14183:up|Cthrc1,68588:down|Htr2b,15559:down|Kng1,16644:up|Prl2c2,18811:up|Efemp1,216616:up|Dpysl3,22240:down|Slpi,20568:up|Aspn,66695:down|Vdr,22337:down|Fabp4,11770:up|Cxcl12,20315:up|Nabp1,109019:down|Htr2a,15558:down|Mmp2,17390:down|Oas3,246727:up|Slc40a1,53945:up|Gas6,14456:up|Egfr,13649:down|Sfrp1,20377:up|C1qtnf3,81799:down|Tgfbr3,21814:up|Ceacam1,26365:up|Sfrp2,20319:up|Serpina3g,20715:up|Prkar2b,19088:up|Mt1,17748:up|S100a1,20193:up|Col5a2,12832:down|Aldh1a2,19378:up|Cst7,13011:up|Ednrb,13618:up|Epas1,13819:up|Htra3,78558:down|Gpm6b,14758:up|Postn,50706:down|Ptx3,19288:up|Akr1c18,105349:up|Ncam1,17967:down|Ccl12,20293:up|Bmpr1a,12166:down|Wisp1,22402:down|Ccl4,20303:up|Chst11,58250:down|Tnc,21923:down|Prg4,96875:down|Pdxk,216134:up|Plpp3,67916:up|Creb3l1,26427:down|Il1rl1,17082:down|Kitl,17311:up|Fam101b,76566:down|Lrrc17,74511:down|Ccl9,20308:up|Hp,15439:up|Inhba,16323:down|Plat,18791:down|Tfpi2,21789:down|Ctla2a,13024:down|Crlf1,12931:down|Vegfa,22339:down|Serpine2,20720:up|Adamts12,239337:down|Lrrc15,74488:down|Nxn,18230:down|Pparg,19016:up|Tnf,21926:up|Tmem176a,66058:up|Glp1r,14652:down|Lmcd1,30937:up|Tnfrsf23,79201:down|Fgfr1,14182:down|Hmox1,15368:up|Sulf1,240725:down|Susd2,71733:up|Ptgs2,19225:down|Grem1,23892:up|Col3a1,12825:down|Serping1,12258:up|Col5a1,12831:down|Errfi1,74155:down|Enpp1,18605:down</td></tr>
<tr><td>circulatory system process</td><td>GO:0003013</td><td>9.34E-09</td><td>3.26E-07</td><td>Htr2b,15559:down|Kng1,16644:up|Adm,11535:up|Ednrb,13618:up|Epas1,13819:up|Ddah1,69219:down|Postn,50706:down|Vegfa,22339:down|Htr2a,15558:down|Mmp2,17390:down|Gas6,14456:up|Pparg,19016:up|Glp1r,14652:down|Fxyd1,56188:up|Col1a2,12843:down|Egfr,13649:down|Hmox1,15368:up|Tgfbr3,21814:up|Ptgs2,19225:down|Stc1,20855:up|Ccl4,20303:up|Adipoq,11450:up|Ceacam1,26365:up|Tgfb2,21808:down|Vegfb,22340:up|S100a1,20193:up</td></tr>
<tr><td>smooth muscle cell proliferation</td><td>GO:0048659</td><td>1.16E-08</td><td>4.01E-07</td><td>Tnf,21926:up|Pparg,19016:up|Egfr,13649:down|Hmox1,15368:up|Bmpr1a,12166:down|Wisp1,22402:down|Ptgs2,19225:down|Igf1,16000:up|Bmp4,12159:up|Adipoq,11450:up|Vegfa,22339:down|Mmp2,17390:down|Igfbp3,16009:up|Ptgir,19222:up|Fgfr2,14183:up</td></tr>
<tr><td>regulation of embryonic development</td><td>GO:0045995</td><td>1.25E-08</td><td>4.31E-07</td><td>Col5a2,12832:down|Lama2,16773:down|Fgfr1,14182:down|Sfrp1,20377:up|Sulf1,240725:down|Ptk7,71461:down|Wnt5a,22418:down|Bmpr1a,12166:down|Il1rn,16181:up|Igf1,16000:up|Bmp4,12159:up|Adipoq,11450:up|Sfrp2,20319:up|Col5a1,12831:down</td></tr>
<tr><td>response to oxygen-containing compound</td><td>GO:1901700</td><td>1.26E-08</td><td>4.31E-07</td><td>Col5a2,12832:down|Adm,11535:up|Mrc1,17533:up|Ccl7,20306:up|Ccl2,20296:up|Aldh1a2,19378:up|Pdgfc,54635:down|Ednrb,13618:up|Ankrd1,107765:down|Postn,50706:down|Cdo1,12583:up|Akr1c18,105349:up|Ncam1,17967:down|Ccl12,20293:up|Adipoq,11450:up|Bmp4,12159:up|Igf1,16000:up|Cxcl9,17329:up|Gatm,67092:down|Ret,19713:up|Tgfb2,21808:down|Ptgir,19222:up|Htr2b,15559:down|Hp,15439:up|Ptk7,71461:down|Inhba,16323:down|Slpi,20568:up|Vdr,22337:down|Htr2a,15558:down|Mmp2,17390:down|Igf2,16002:up|Pparg,19016:up|Gas6,14456:up|Tnf,21926:up|Col1a2,12843:down|Glp1r,14652:down|Egfr,13649:down|Hmox1,15368:up|Sfrp1,20377:up|Tgfbr3,21814:up|Ptgs2,19225:down|Col3a1,12825:down|Serpina3g,20715:up|Tnfrsf11b,18383:up|Enpp1,18605:down|Mt1,17748:up</td></tr>
<tr><td>regulation of cellular protein metabolic process</td><td>GO:0032268</td><td>1.33E-08</td><td>4.53E-07</td><td>Igfbp4,16010:up|Flrt2,399558:down|Sfrp4,20379:up|C3,12266:up|Igfbp3,16009:up|Cd109,235505:down|Plk2,20620:down|Trf,22041:up|Cd24a,12484:down|Wnt5a,22418:down|Il1rn,16181:up|Adipoq,11450:up|Bmp4,12159:up|Igf1,16000:up|Tgfb2,21808:down|F3,14066:up|Fgfr2,14183:up|Htr2b,15559:down|Kng1,16644:up|Ccl6,20305:up|Slpi,20568:up|Aspn,66695:down|Fabp4,11770:up|Htr2a,15558:down|Gas6,14456:up|Egfr,13649:down|Sfrp1,20377:up|C1qtnf3,81799:down|Ceacam1,26365:up|Ccl8,20307:up|Sfrp2,20319:up|Serpina3g,20715:up|Fam20a,208659:down|Npnt,114249:up|Tnfrsf11b,18383:up|Prkar2b,19088:up|Ccl2,20296:up|Ccl7,20306:up|Cst7,13011:up|Ednrb,13618:up|Pdgfc,54635:down|Sema7a,20361:down|Uchl5,56207:up|Ccl12,20293:up|Bmpr1a,12166:down|Ccl4,20303:up|Ret,19713:up|Plpp3,67916:up|Kitl,17311:up|Ccl9,20308:up|Inhba,16323:down|Ccl3,20302:up|Plat,18791:down|Tfpi2,21789:down|Ctla2a,13024:down|Crlf1,12931:down|Vegfa,22339:down|Serpine2,20720:up|Lrrc15,74488:down|Igf2,16002:up|Nxn,18230:down|Pparg,19016:up|Tnf,21926:up|Fgfr1,14182:down|Cd4,12504:up|Grem1,23892:up|Serping1,12258:up|Enpp1,18605:down|Errfi1,74155:down</td></tr>
<tr><td>morphogenesis of an epithelium</td><td>GO:0002009</td><td>1.41E-08</td><td>4.77E-07</td><td>Cthrc1,68588:down|Adm,11535:up|Plxnd1,67784:up|Ptk7,71461:down|Aldh1a2,19378:up|Mgp,17313:up|Vdr,22337:down|Cxcl12,20315:up|Vegfa,22339:down|Mmp2,17390:down|Adamts12,239337:down|Tnf,21926:up|Fgfr1,14182:down|Egfr,13649:down|Sfrp1,20377:up|Sulf1,240725:down|Wnt5a,22418:down|Grem1,23892:up|Bmp4,12159:up|Igf1,16000:up|Sfrp2,20319:up|Tnc,21923:down|Col5a1,12831:down|Ret,19713:up|Tgfb2,21808:down|Rspo2,239405:up|Aldh1a3,56847:down|Npnt,114249:up|Fgfr2,14183:up</td></tr>
<tr><td>leukocyte migration</td><td>GO:0050900</td><td>1.54E-08</td><td>5.20E-07</td><td>Ccl9,20308:up|Ccl6,20305:up|Ccl7,20306:up|Ccl2,20296:up|Figf,14205:up|Ednrb,13618:up|Ccl3,20302:up|Cxcl12,20315:up|Cx3cr1,13051:down|Gas6,14456:up|Tnf,21926:up|Cxcl14,57266:down|Cd24a,12484:down|Wnt5a,22418:down|Ccl12,20293:up|Ccl4,20303:up|Grem1,23892:up|Ccl8,20307:up|Cxcl9,17329:up|Kitl,17311:up</td></tr>
</table>

regulation of apoptotic process	GO:0042981	1.63E-08	5.48E-07	Adm,11535:up|Sfrp4,20379:up|Aldh1a2,19378:up|Ednrb,13618:up|Ankrd1,107765:down|Tnfrsf12a,27279:down|Cx3cr1,13051:down|Igfbp3,16009:up|Plk2,20620:down|Akr1c18,105349:up|Cd24a,12484:down|Wnt5a,22418:down|Ccl12,20293:up|Il1rn,16181:up|Adipoq,11450:up|Bmp4,12159:up|Igf1,16000:up|Chst11,58250:down|Tgfb2,21808:down|Pdxk,216134:up|Sdf2l1,64136:up|Aldh1a3,56847:down|Creb3l1,26427:down|Fgfr2,14183:up|F3,14066:up|Kitl,17311:up|Htr2b,15559:down|Inhba,16323:down|Ccl3,20302:up|Vdr,22337:down|Crlf1,12931:down|Cxcl12,20315:up|Vegfa,22339:down|Mmp2,17390:down|Slc40a1,53945:up|Pparg,19016:up|Gas6,14456:up|Tnf,21926:up|Glp1r,14652:down|Tnfrsf23,79201:down|Fgfr1,14182:down|Egfr,13649:down|Hmox1,15368:up|Sfrp1,20377:up|Tgfbr3,21814:up|Ptgs2,19225:down|Grem1,23892:up|Ceacam1,26365:up|Sfrp2,20319:up|Tnfrsf11b,18383:up|Mt1,17748:up
intracellular signal transduction	GO:0035556	1.66E-08	5.52E-07	Igfbp4,16010:up|Flrt2,399558:down|Sfrp4,20379:up|C3,12266:up|Ankrd1,107765:down|Igfbp3,16009:up|Plk2,20620:down|Trf,22041:up|Cd24a,12484:down|Wnt5a,22418:down|Mark1,226778:up|Il1rn,16181:up|Adipoq,11450:up|Bmp4,12159:up|Igf1,16000:up|Tgfb2,21808:down|Hpse,15442:up|Ptgir,19222:up|Fgfr2,14183:up|F3,14066:up|Htr2b,15559:down|Ccl6,20305:up|Aspn,66695:down|Mt2,17750:up|Saa3,20210:up|Cxcl12,20315:up|Htr2a,15558:down|Gas6,14456:up|Egfr,13649:down|Sfrp1,20377:up|C1qtnf3,81799:down|Tgfbr3,21814:up|Ceacam1,26365:up|Ccl8,20307:up|Sfrp2,20319:up|Npnt,114249:up|Tnfrsf11b,18383:up|Mt1,17748:up|Ccl2,20296:up|Ccl7,20306:up|Pdgfc,54635:down|Ednrb,13618:up|Sema7a,20361:down|Clec4n,56620:up|Dok2,13449:up|Akr1c18,105349:up|Ncam1,17967:down|Ccl12,20293:up|Ccl4,20303:up|Ret,19713:up|Creb3l1,26427:down|Kitl,17311:up|Il1rl1,17082:down|Ccl9,20308:up|Rasl11a,68895:up|Inhba,16323:down|Ccl3,20302:up|Crlf1,12931:down|Vegfa,22339:down|Serpine2,20720:up|Lrrc15,74488:down|Igf2,16002:up|Tnf,21926:up|Lmcd1,30937:up|Col1a2,12843:down|Glp1r,14652:down|Fgfr1,14182:down|Hmox1,15368:up|Cd4,12504:up|Ptgs2,19225:down|Grem1,23892:up|Col3a1,12825:down|Errfi1,74155:down
morphogenesis of a branching structure	GO:0001763	1.74E-08	5.77E-07	Adm,11535:up|Plxnd1,67784:up|Mgp,17313:up|Vdr,22337:down|Cxcl12,20315:up|Vegfa,22339:down|Tnf,21926:up|Fgfr1,14182:down|Sfrp1,20377:up|Sulf1,240725:down|Wnt5a,22418:down|Grem1,23892:up|Bmp4,12159:up|Igf1,16000:up|Sfrp2,20319:up|Tnc,21923:down|Rspo2,239405:up|Npnt,114249:up|Fgfr2,14183:up
regulation of cell death	GO:0010941	1.87E-08	6.18E-07	Adm,11535:up|Sfrp4,20379:up|Aldh1a2,19378:up|Ednrb,13618:up|Ankrd1,107765:down|Tnfrsf12a,27279:down|Cx3cr1,13051:down|Igfbp3,16009:up|Plk2,20620:down|Akr1c18,105349:up|Cd24a,12484:down|Ncam1,17967:down|Wnt5a,22418:down|Ccl12,20293:up|Wisp1,22402:down|Il1rn,16181:up|Adipoq,11450:up|Bmp4,12159:up|Igf1,16000:up|Chst11,58250:down|Tgfb2,21808:down|Pdxk,216134:up|Sdf2l1,64136:up|Aldh1a3,56847:down|Creb3l1,26427:down|Fgfr2,14183:up|F3,14066:up|Kitl,17311:up|Htr2b,15559:down|Hp,15439:up|Inhba,16323:down|Ccl3,20302:up|Vdr,22337:down|Crlf1,12931:down|Cxcl12,20315:up|Vegfa,22339:down|Mmp2,17390:down|Slc40a1,53945:up|Pparg,19016:up|Gas6,14456:up|Tnf,21926:up|Glp1r,14652:down|Tnfrsf23,79201:down|Fgfr1,14182:down|Egfr,13649:down|Hmox1,15368:up|Sfrp1,20377:up|Tgfbr3,21814:up|Ptgs2,19225:down|Grem1,23892:up|Ceacam1,26365:up|Sfrp2,20319:up|Tnfrsf11b,18383:up|Mt1,17748:up
negative regulation of cell differentiation	GO:0045596	1.93E-08	6.34E-07	Fstl3,83554:down|Col5a2,12832:down|Clec2d,93694:up|Tmem176b,65963:up|Ednrb,13618:up|Postn,50706:down|Plk2,20620:down|Cxcl14,57266:down|Cd24a,12484:down|Wnt5a,22418:down|Bmpr1a,12166:down|Adipoq,11450:up|Bmp4,12159:up|Igf1,16000:up|Tgfb2,21808:down|Fam101b,76566:down|Prl2c2,18811:up|Ccl9,20308:up|Lrrc17,74511:down|Efemp1,216616:up|Dpysl3,22240:down|Vegfa,22339:down|Adamts12,239337:down|Tmem176a,66058:up|Tnf,21926:up|Pparg,19016:up|Fgfr1,14182:down|Sfrp1,20377:up|Tgfbr3,21814:up|Grem1,23892:up|Col3a1,12825:down|Ceacam1,26365:up|Sfrp2,20319:up|Col5a1,12831:down|Enpp1,18605:down
peptidyl-tyrosine phosphorylation	GO:0018108	2.33E-08	7.62E-07	Efemp1,216616:up|Pdgfc,54635:down|Crlf1,12931:down|Vegfa,22339:down|Htr2a,15558:down|Igf2,16002:up|Gas6,14456:up|Cd24a,12484:down|Egfr,13649:down|Fgfr1,14182:down|Sfrp1,20377:up|Cd4,12504:up|Grem1,23892:up|Adipoq,11450:up|Igf1,16000:up|Sfrp2,20319:up|Plpp3,67916:up|Errfi1,74155:down|Fgfr2,14183:up|Kitl,17311:up
regulation of programmed cell death	GO:0043067	2.36E-08	7.67E-07	Adm,11535:up|Sfrp4,20379:up|Aldh1a2,19378:up|Ednrb,13618:up|Ankrd1,107765:down|Tnfrsf12a,27279:down|Cx3cr1,13051:down|Igfbp3,16009:up|Plk2,20620:down|Akr1c18,105349:up|Cd24a,12484:down|Wnt5a,22418:down|Ccl12,20293:up|Il1rn,16181:up|Adipoq,11450:up|Bmp4,12159:up|Igf1,16000:up|Chst11,58250:down|Tgfb2,21808:down|Pdxk,216134:up|Sdf2l1,64136:up|Aldh1a3,56847:down|Creb3l1,26427:down|Fgfr2,14183:up|F3,14066:up|Kitl,17311:up|Htr2b,15559:down|Inhba,16323:down|Ccl3,20302:up|Vdr,22337:down|Crlf1,12931:down|Cxcl12,20315:up|Vegfa,22339:down|Mmp2,17390:down|Slc40a1,53945:up|Pparg,19016:up|Gas6,14456:up|Tnf,21926:up|Glp1r,14652:down|Tnfrsf23,79201:down|Fgfr1,14182:down|Egfr,13649:down|Hmox1,15368:up|Sfrp1,20377:up|Tgfbr3,21814:up|Ptgs2,19225:down|Grem1,23892:up|Ceacam1,26365:up|Sfrp2,20319:up|Tnfrsf11b,18383:up|Mt1,17748:up
peptidyl-tyrosine modification	GO:0018212	2.50E-08	8.07E-07	Efemp1,216616:up|Pdgfc,54635:down|Crlf1,12931:down|Vegfa,22339:down|Htr2a,15558:down|Igf2,16002:up|Gas6,14456:up|Cd24a,12484:down|Egfr,13649:down|Fgfr1,14182:down|Sfrp1,20377:up|Cd4,12504:up|Grem1,23892:up|Adipoq,11450:up|Igf1,16000:up|Sfrp2,20319:up|Plpp3,67916:up|Errfi1,74155:down|Fgfr2,14183:up|Kitl,17311:up
regulation of system process	GO:0044057	2.52E-08	8.10E-07	Adm,11535:up|Inhba,16323:down|Ednrb,13618:up|Mmp13,17386:up|Epas1,13819:up|Htr2a,15558:down|Mmp2,17390:down|Anpep,16790:up|Gas6,14456:up|Tnf,21926:up|Glp1r,14652:down|Trf,22041:up|Lmcd1,30937:up|Fxyd1,56188:up|Fgfr1,14182:down|Egfr,13649:down|Ncam1,17967:down|C1qtnf3,81799:down|Wisp1,22402:down|Ptgs2,19225:down|Stc1,20855:up|Adipoq,11450:up|Igf1,16000:up|Tgfb2,21808:down|Npnt,114249:up|Errfi1,74155:down|S100a1,20193:up
response to lipid	GO:0033993	2.61E-08	8.35E-07	Adm,11535:up|Mrc1,17533:up|Ccl2,20296:up|Aldh1a2,19378:up|Ednrb,13618:up|Ankrd1,107765:down|Cdo1,12583:up|Akr1c18,105349:up|Cd24a,12484:down|Ccl12,20293:up|Il1rn,16181:up|Bmp4,12159:up|Igf1,16000:up|Cxcl9,17329:up|Ret,19713:up|Tgfb2,21808:down|Ptgir,19222:up|Inhba,16323:down|Ptk7,71461:down|Slpi,20568:up|Vdr,22337:down|Mmp2,17390:down|Pappa,18491:down|Pparg,19016:up|Tnf,21926:up|Alpl,11647:up|Egfr,13649:down|Hmox1,15368:up|Sfrp1,20377:up|Tgfbr3,21814:up|Ptgs2,19225:down|Tnfrsf11b,18383:up
tube development	GO:0035295	3.19E-08	1.01E-06	Adm,11535:up|Plxnd1,67784:up|Aldh1a2,19378:up|Pdgfc,54635:down|Mgp,17313:up|Ednrb,13618:up|Epas1,13819:up|Wnt5a,22418:down|Bmpr1a,12166:down|Bmp4,12159:up|Igf1,16000:up|Tnc,21923:down|Ret,19713:up|Fgfr2,14183:up|Cthrc1,68588:down|Ptk7,71461:down|Vdr,22337:down|Crlf1,12931:down|Cxcl12,20315:up|Vegfa,22339:down|Serpine2,20720:up|Adamts12,239337:down|Tnf,21926:up|Egfr,13649:down|Fgfr1,14182:down|Sfrp1,20377:up|Grem1,23892:up|Col3a1,12825:down|Sfrp2,20319:up|Rspo2,239405:up|Npnt,114249:up|Errfi1,74155:down

phosphorylation	GO:0016310	3.68E-08	1.16E-06	Igfbp4,16010:up|Flrt2,399558:down|Sfrp4,20379:up|C3,12266:up|Igfbp3,16009:up|Cd109,235505:down|Plk2,20620:down|Trf,22041:up|Cd24a,12484:down|Wnt5a,22418:down|Mark1,226778:up|Il1rn,16181:up|Adipoq,11450:up|Bmp4,12159:up|Igf1,16000:up|Tgfb2,21808:down|Fgfr2,14183:up|Htr2b,15559:down|Efemp1,216616:up|Ccl6,20305:up|Aspn,66695:down|Saa3,20210:up|Fabp4,11770:up|Htr2a,15558:down|Gas6,14456:up|Egfr,13649:down|Sfrp1,20377:up|C1qtnf3,81799:down|Tgfbr3,21814:up|Ceacam1,26365:up|Ccl8,20307:up|Sfrp2,20319:up|Fam20a,208659:down|Npnt,114249:up|Tnfrsf11b,18383:up|Prkar2b,19088:up|Ccl2,20296:up|Ccl7,20306:up|Ednrb,13618:up|Pdgfc,54635:down|Sema7a,20361:down|Dok2,13449:up|Ccl12,20293:up|Bmpr1a,12166:down|Ccl4,20303:up|Ret,19713:up|Pdxk,216134:up|Plpp3,67916:up|Kitl,17311:up|Ccl9,20308:up|Inhba,16323:down|Ccl3,20302:up|Srpx2,68792:down|Crlf1,12931:down|Vegfa,22339:down|Igf2,16002:up|Lrrc15,74488:down|Tnf,21926:up|Fgfr1,14182:down|Cd4,12504:up|Grem1,23892:up|Enpp1,18605:down|Errfi1,74155:down|Uck2,80914:down
transmembrane receptor protein serine/threonine kinase signaling pathway	GO:0007178	3.92E-08	1.23E-06	Fstl3,83554:down|Hpgd,15446:up|Inhba,16323:down|Ccl2,20296:up|Aspn,66695:down|Htra3,78558:down|Cd109,235505:down|Trf,22041:up|Col1a2,12843:down|Sfrp1,20377:up|Sulf1,240725:down|Tgfbr3,21814:up|Bmpr1a,12166:down|Wnt5a,22418:down|Grem1,23892:up|Bmp4,12159:up|Col3a1,12825:down|Chst11,58250:down|Sfrp2,20319:up|Tgfb2,21808:down|Npnt,114249:up
positive regulation of cellular protein metabolic process	GO:0032270	4.52E-08	1.41E-06	Igfbp4,16010:up|Ccl7,20306:up|Ccl2,20296:up|C3,12266:up|Pdgfc,54635:down|Ednrb,13618:up|Sema7a,20361:down|Igfbp3,16009:up|Plk2,20620:down|Trf,22041:up|Cd24a,12484:down|Wnt5a,22418:down|Bmpr1a,12166:down|Ccl12,20293:up|Il1rn,16181:up|Ccl4,20303:up|Adipoq,11450:up|Bmp4,12159:up|Igf1,16000:up|Ret,19713:up|Tgfb2,21808:down|Plpp3,67916:up|Fgfr2,14183:up|F3,14066:up|Kitl,17311:up|Htr2b,15559:down|Ccl9,20308:up|Ccl6,20305:up|Inhba,16323:down|Ccl3,20302:up|Crlf1,12931:down|Vegfa,22339:down|Htr2a,15558:down|Igf2,16002:up|Pparg,19016:up|Gas6,14456:up|Tnf,21926:up|Fgfr1,14182:down|Egfr,13649:down|C1qtnf3,81799:down|Cd4,12504:up|Grem1,23892:up|Ccl8,20307:up|Ceacam1,26365:up|Sfrp2,20319:up|Fam20a,208659:down|Tnfrsf11b,18383:up|Npnt,114249:up
regulation of cellular response to growth factor stimulus	GO:0090287	4.67E-08	1.46E-06	Fstl3,83554:down|Figf,14205:up|Aspn,66695:down|Htra3,78558:down|Vegfa,22339:down|Adamts12,239337:down|Cd109,235505:down|Fgfr1,14182:down|Sfrp1,20377:up|Sulf1,240725:down|Tgfbr3,21814:up|Wnt5a,22418:down|Grem1,23892:up|Bmp4,12159:up|Chst11,58250:down|Sfrp2,20319:up|Npnt,114249:up|Fgfr2,14183:up
metal ion homeostasis	GO:0055065	5.35E-08	1.66E-06	Htr2b,15559:down|Kng1,16644:up|Adm,11535:up|Ccl2,20296:up|Ednrb,13618:up|Ccl3,20302:up|Mt2,17750:up|Epas1,13819:up|Vdr,22337:down|Fam132b,227358:down|Steap1,70358:down|Htr2a,15558:down|Slc40a1,53945:up|Anpep,16790:up|Glp1r,14652:down|Fxyd1,56188:up|Trf,22041:up|Egfr,13649:down|Cd24a,12484:down|Hmox1,15368:up|Cd4,12504:up|Stc1,20855:up|Tfrc,22042:up|Bmp4,12159:up|Tgfb2,21808:down|Fam20a,208659:down|Ptgir,19222:up|Mt1,17748:up
regulation of transport	GO:0051049	5.66E-08	1.74E-06	Ice1,218333:down|Sfrp4,20379:up|Ccl2,20296:up|C3,12266:up|Ednrb,13618:up|Mmp13,17386:up|Ankrd1,107765:down|Gpm6b,14758:up|Clec4n,56620:up|Postn,50706:down|Hvcn1,74096:up|Trf,22041:up|Ptx3,19288:up|Ncam1,17967:down|Wnt5a,22418:down|Bmpr1a,12166:down|Ccl12,20293:up|Stc1,20855:up|Il1rn,16181:up|Ccl4,20303:up|Maob,109731:up|Adipoq,11450:up|Bmp4,12159:up|Igf1,16000:up|Tgfb2,21808:down|Il1rl1,17082:down|Clec4e,56619:up|Htr2b,15559:down|Inhba,16323:down|Ccl3,20302:up|Vdr,22337:down|Fam132b,227358:down|Cxcl12,20315:up|Vegfa,22339:down|Htr2a,15558:down|Serpine2,20720:up|Anpep,16790:up|Kdelr3,105785:down|Pparg,19016:up|Gas6,14456:up|Tnf,21926:up|Glp1r,14652:down|Fxyd1,56188:up|Fgfr1,14182:down|Egfr,13649:down|Lpl,16956:up|Hmox1,15368:up|Sfrp1,20377:up|C1qtnf3,81799:down|Cd4,12504:up|Ptgs2,19225:down|Kcnj15,16516:down|Fxyd2,11936:up|Sncg,20618:up|Grem1,23892:up|Ceacam1,26365:up|Sfrp2,20319:up|Enpp1,18605:down|S100a1,20193:up
ameboidal-type cell migration	GO:0001667	5.87E-08	1.79E-06	Htr2b,15559:down|Prl2c2,18811:up|Plxnd1,67784:up|Ednrb,13618:up|Sema7a,20361:down|Srpx2,68792:down|Cxcl12,20315:up|Vegfa,22339:down|Loxl2,94352:down|Fgfr1,14182:down|Has2,15117:down|Wnt5a,22418:down|Tgfbr3,21814:up|Stc1,20855:up|Ptgs2,19225:down|Grem1,23892:up|Bmp4,12159:up|Ceacam1,26365:up|Ret,19713:up|Tgfb2,21808:down|Plpp3,67916:up|Kitl,17311:up
cellular response to endogenous stimulus	GO:0071495	6.04E-08	1.83E-06	Fstl3,83554:down|Col5a2,12832:down|Flrt2,399558:down|Hpgd,15446:up|Ccl2,20296:up|Pdgfc,54635:down|Ankrd1,107765:down|Htra3,78558:down|Cd109,235505:down|Akr1c18,105349:up|Wnt5a,22418:down|Bmpr1a,12166:down|Adipoq,11450:up|Bmp4,12159:up|Igf1,16000:up|Chst11,58250:down|Tgfb2,21808:down|Ptgir,19222:up|Fgfr2,14183:up|Htr2b,15559:down|Inhba,16323:down|Aspn,66695:down|Vdr,22337:down|Mmp2,17390:down|Adamts12,239337:down|Igf2,16002:up|Tnf,21926:up|Pparg,19016:up|Col1a2,12843:down|Egfr,13649:down|Fgfr1,14182:down|Sfrp1,20377:up|Has2,15117:down|Sulf1,240725:down|Tgfbr3,21814:up|Grem1,23892:up|Col3a1,12825:down|Sfrp2,20319:up|Npnt,114249:up|Enpp1,18605:down
skeletal system morphogenesis	GO:0048705	6.72E-08	2.03E-06	Fam101b,76566:down|Bnc2,242509:down|Mmp13,17386:up|Serpinh1,12406:down|Vegfa,22339:down|Mmp2,17390:down|Alpl,11647:up|Col11a1,12814:down|Sfrp1,20377:up|Has2,15117:down|Stc1,20855:up|Grem1,23892:up|Bmp4,12159:up|Chst11,58250:down|Sfrp2,20319:up|Tbx15,21384:down|Tgfb2,21808:down|Fgfr2,14183:up
growth	GO:0040007	6.75E-08	2.03E-06	Igfbp4,16010:up|Adm,11535:up|Plxnd1,67784:up|C3,12266:up|Mmp13,17386:up|Sema7a,20361:down|Htra3,78558:down|Tnfrsf12a,27279:down|Postn,50706:down|Igfbp3,16009:up|Ncam1,17967:down|Wnt5a,22418:down|Bmpr1a,12166:down|Wisp1,22402:down|Stc1,20855:up|Bmp4,12159:up|Igf1,16000:up|Chst11,58250:down|Crabp2,12904:down|Tnc,21923:down|Tgfb2,21808:down|Fgfr2,14183:up|Ptk7,71461:down|Inhba,16323:down|Bnc2,242509:down|Ninj1,18081:up|Cxcl12,20315:up|Vegfa,22339:down|Serpine2,20720:up|Igf2,16002:up|Pparg,19016:up|Gas6,14456:up|Tnf,21926:up|Egfr,13649:down|Fgfr1,14182:down|Sfrp1,20377:up|Tgfbr3,21814:up|Grem1,23892:up|Sfrp2,20319:up|Rspo2,239405:up|Enpp1,18605:down
positive regulation of vasculature development	GO:1904018	7.85E-08	2.35E-06	Prl2c2,18811:up|Adm,11535:up|Hmox1,15368:up|Wnt5a,22418:down|C3,12266:up|Ptgs2,19225:down|Grem1,23892:up|Ceacam1,26365:up|Srpx2,68792:down|Sfrp2,20319:up|Ddah1,69219:down|Vegfa,22339:down|Cx3cr1,13051:down|F3,14066:up
regulation of protein metabolic process	GO:0051246	8.71E-08	2.59E-06	Igfbp4,16010:up|Flrt2,399558:down|Sfrp4,20379:up|C3,12266:up|Igfbp3,16009:up|Cd109,235505:down|Plk2,20620:down|Trf,22041:up|Cd24a,12484:down|Wnt5a,22418:down|Il1rn,16181:up|Adipoq,11450:up|Bmp4,12159:up|Igf1,16000:up|Tgfb2,21808:down|F3,14066:up|Fgfr2,14183:up|Htr2b,15559:down|Kng1,16644:up|Ccl6,20305:up|Slpi,20568:up|Aspn,66695:down|Fabp4,11770:up|Htr2a,15558:down|Gas6,14456:up|Egfr,13649:down|Sfrp1,20377:up|C1qtnf3,81799:down|Ceacam1,26365:up|Ccl8,20307:up|Sfrp2,20319:up|Serpina3g,20715:up|Fam20a,208659:down|Npnt,114249:up|Tnfrsf11b,18383:up|Prkar2b,19088:up|Ccl2,20296:up|Ccl7,20306:up|Cst7,13011:up|Ednrb,13618:up|Pdgfc,54635:down|Sema7a,20361:down|Uchl5,56207:up|Ptx3,19288:up|Ccl12,20293:up|Bmpr1a,12166:down|Ccl4,20303:up|Ret,19713:up|Prg4,96875:down|Plpp3,67916:up|Kitl,17311:up|Ccl9,20308:up|Inhba,16323:down|Ccl3,20302:up|Plat,18791:down|Tfpi2,21789:down|Ctla2a,13024:down|Crlf1,12931:down|Vegfa,22339:down|Serpine2,20720:up|Lrrc15,74488:down|Igf2,16002:up|Nxn,18230:down|Pparg,19016:up|Tnf,21926:up|Fgfr1,14182:down|Cd4,12504:up|Grem1,23892:up|Serping1,12258:up|Errfi1,74155:down|Enpp1,18605:down

<table>
<tr><td>regulation of peptidyl-tyrosine phosphorylation</td><td>GO:0050730</td><td>9.33E-08</td><td>2.76E-06</td><td>Pdgfc,54635:down|Crlf1,12931:down|Vegfa,22339:down|Htr2a,15558:down|Igf2,16002:up|Gas6,14456:up|Cd24a,12484:down|Egfr,13649:down|Sfrp1,20377:up|Cd4,12504:up|Grem1,23892:up|Adipoq,11450:up|Igf1,16000:up|Sfrp2,20319:up|Plpp3,67916:up|Errfi1,74155:down|Kitl,17311:up</td></tr>
<tr><td>prostate gland epithelium morphogenesis</td><td>GO:0060740</td><td>9.65E-08</td><td>2.84E-06</td><td>Sfrp1,20377:up|Sulf1,240725:down|Wnt5a,22418:down|Igf1,16000:up|Bmp4,12159:up|Tnc,21923:down|Mmp2,17390:down|Fgfr2,14183:up</td></tr>
<tr><td>inorganic ion homeostasis</td><td>GO:0098771</td><td>1.00E-07</td><td>2.94E-06</td><td>Htr2b,15559:down|Kng1,16644:up|Adm,11535:up|Sfrp4,20379:up|Ccl2,20296:up|Ednrb,13618:up|Ccl3,20302:up|Mt2,17750:up|Epas1,13819:up|Vdr,22337:down|Fam132b,227358:down|Steap1,70358:down|Htr2a,15558:down|Slc40a1,53945:up|Anpep,16790:up|Glp1r,14652:down|Fxyd1,56188:up|Trf,22041:up|Egfr,13649:down|Cd24a,12484:down|Hmox1,15368:up|Cd4,12504:up|Tfrc,22042:up|Stc1,20855:up|Bmp4,12159:up|Tgfb2,21808:down|Fam20a,208659:down|Ptgir,19222:up|Enpp1,18605:down|Mt1,17748:up</td></tr>
<tr><td>system process</td><td>GO:0003008</td><td>1.05E-07</td><td>3.05E-06</td><td>Adm,11535:up|Ednrb,13618:up|Mmp13,17386:up|Epas1,13819:up|Ddah1,69219:down|Shroom4,208431:down|Postn,50706:down|Cx3cr1,13051:down|Plk2,20620:down|Trf,22041:up|Ncam1,17967:down|Mme,17380:up|Wisp1,22402:down|Stc1,20855:up|Il1rn,16181:up|Ccl4,20303:up|Adipoq,11450:up|Bmp4,12159:up|Igf1,16000:up|Tgfb2,21808:down|Vegfb,22340:up|Aldh1a3,56847:down|Htr2b,15559:down|Kng1,16644:up|Inhba,16323:down|Vdr,22337:down|Vegfa,22339:down|Htr2a,15558:down|Mmp2,17390:down|Serpine2,20720:up|Igf2,16002:up|Anpep,16790:up|Pparg,19016:up|Gas6,14456:up|Tnf,21926:up|Glp1r,14652:down|Lmcd1,30937:up|Fxyd1,56188:up|Col1a2,12843:down|Fgfr1,14182:down|Egfr,13649:down|Col11a1,12814:down|Hmox1,15368:up|Sulf1,240725:down|C1qtnf3,81799:down|Tgfbr3,21814:up|Ptgs2,19225:down|Ceacam1,26365:up|Mcoln3,171166:up|Prkar2b,19088:up|Npnt,114249:up|Errfi1,74155:down|S100a1,20193:up</td></tr>
<tr><td>response to interferon-gamma</td><td>GO:0034341</td><td>1.07E-07</td><td>3.12E-06</td><td>Pparg,19016:up|Ccl9,20308:up|Ccl6,20305:up|Mrc1,17533:up|Ccl7,20306:up|Ccl2,20296:up|Ccl12,20293:up|Ccl4,20303:up|Ccl3,20302:up|Ccl8,20307:up</td></tr>
<tr><td>positive regulation of angiogenesis</td><td>GO:0045766</td><td>1.14E-07</td><td>3.29E-06</td><td>Prl2c2,18811:up|Adm,11535:up|Hmox1,15368:up|Wnt5a,22418:down|C3,12266:up|Ptgs2,19225:down|Grem1,23892:up|Srpx2,68792:down|Sfrp2,20319:up|Ddah1,69219:down|Vegfa,22339:down|Cx3cr1,13051:down|F3,14066:up</td></tr>
<tr><td>prostate gland morphogenesis</td><td>GO:0060512</td><td>1.24E-07</td><td>3.57E-06</td><td>Sfrp1,20377:up|Sulf1,240725:down|Wnt5a,22418:down|Igf1,16000:up|Bmp4,12159:up|Tnc,21923:down|Mmp2,17390:down|Fgfr2,14183:up</td></tr>
<tr><td>myoblast differentiation</td><td>GO:0045445</td><td>1.28E-07</td><td>3.66E-06</td><td>Tnf,21926:up|Prl2c2,18811:up|Cxcl14,57266:down|Ccl9,20308:up|Grem1,23892:up|Igf1,16000:up|Epas1,13819:up|Bmp4,12159:up|Ccl8,20307:up|Cxcl9,17329:up|Igfbp3,16009:up</td></tr>
<tr><td>negative regulation of immune system process</td><td>GO:0002683</td><td>1.41E-07</td><td>4.02E-06</td><td>Fstl3,83554:down|Clec2d,93694:up|Lrrc17,74511:down|Tmem176b,65963:up|Cxcl12,20315:up|Igf2,16002:up|Pparg,19016:up|Tnf,21926:up|Tmem176a,66058:up|Cd24a,12484:down|Hmox1,15368:up|Sfrp1,20377:up|Ccl12,20293:up|Grem1,23892:up|Col3a1,12825:down|Adipoq,11450:up|Bmp4,12159:up|Ceacam1,26365:up|Serping1,12258:up|Tgfb2,21808:down|Il1rl1,17082:down|Kitl,17311:up</td></tr>
<tr><td>myeloid leukocyte differentiation</td><td>GO:0002573</td><td>1.47E-07</td><td>4.18E-06</td><td>Fstl3,83554:down|Clec2d,93694:up|Lrrc17,74511:down|Ccl3,20302:up|Vegfa,22339:down|Cd109,235505:down|Pparg,19016:up|Tnf,21926:up|Trf,22041:up|Sfrp1,20377:up|Tfrc,22042:up|Adipoq,11450:up|Bmp4,12159:up|Ceacam1,26365:up|Tnfsf9,21950:up|Kitl,17311:up</td></tr>
<tr><td>epithelial tube morphogenesis</td><td>GO:0060562</td><td>1.49E-07</td><td>4.20E-06</td><td>Cthrc1,68588:down|Adm,11535:up|Plxnd1,67784:up|Ptk7,71461:down|Aldh1a2,19378:up|Mgp,17313:up|Vdr,22337:down|Cxcl12,20315:up|Vegfa,22339:down|Adamts12,239337:down|Tnf,21926:up|Sfrp1,20377:up|Wnt5a,22418:down|Grem1,23892:up|Bmp4,12159:up|Igf1,16000:up|Sfrp2,20319:up|Tnc,21923:down|Ret,19713:up|Rspo2,239405:up|Npnt,114249:up|Fgfr2,14183:up</td></tr>
<tr><td>regulation of secretion</td><td>GO:0051046</td><td>1.70E-07</td><td>4.78E-06</td><td>Ednrb,13618:up|Mmp13,17386:up|Ankrd1,107765:down|Clec4n,56620:up|Postn,50706:down|Ncam1,17967:down|Wnt5a,22418:down|Il1rn,16181:up|Stc1,20855:up|Maob,109731:up|Adipoq,11450:up|Igf1,16000:up|Tgfb2,21808:down|Il1rl1,17082:down|Clec4e,56619:up|Htr2b,15559:down|Inhba,16323:down|Ccl3,20302:up|Cxcl12,20315:up|Htr2a,15558:down|Anpep,16790:up|Tnf,21926:up|Pparg,19016:up|Gas6,14456:up|Glp1r,14652:down|Egfr,13649:down|Fgfr1,14182:down|Sfrp1,20377:up|Lpl,16956:up|Hmox1,15368:up|C1qtnf3,81799:down|Sncg,20618:up|Ceacam1,26365:up</td></tr>
<tr><td>hematopoietic or lymphoid organ development</td><td>GO:0048534</td><td>1.84E-07</td><td>5.15E-06</td><td>Fstl3,83554:down|Clec2d,93694:up|Tmem176b,65963:up|Ly9,17085:up|Epas1,13819:up|Lyl1,17095:up|Cd109,235505:down|Trf,22041:up|Cd24a,12484:down|Wnt5a,22418:down|Adipoq,11450:up|Bmp4,12159:up|Ret,19713:up|Prg4,96875:down|Tgfb2,21808:down|Fgfr2,14183:up|Clec4e,56619:up|Kitl,17311:up|Lrrc17,74511:down|Inhba,16323:down|Ccl3,20302:up|Ctla2a,13024:down|Vegfa,22339:down|Slc40a1,53945:up|Tnf,21926:up|Pparg,19016:up|Gas6,14456:up|Tmem176a,66058:up|Sfrp1,20377:up|Tgfbr3,21814:up|Cd4,12504:up|Tfrc,22042:up|Ceacam1,26365:up|Sfrp2,20319:up|Tnfsf9,21950:up</td></tr>
<tr><td>gland morphogenesis</td><td>GO:0022612</td><td>1.85E-07</td><td>5.15E-06</td><td>Tnf,21926:up|Fgfr1,14182:down|Egfr,13649:down|Plxnd1,67784:up|Sfrp1,20377:up|Sulf1,240725:down|Wnt5a,22418:down|Igf1,16000:up|Bmp4,12159:up|Vdr,22337:down|Ceacam1,26365:up|Tnc,21923:down|Mmp2,17390:down|Fgfr2,14183:up</td></tr>
<tr><td>heart development</td><td>GO:0007507</td><td>1.95E-07</td><td>5.42E-06</td><td>Htr2b,15559:down|Adm,11535:up|Flrt2,399558:down|Plxnd1,67784:up|Ptk7,71461:down|Aldh1a2,19378:up|Mmp13,17386:up|Ankrd1,107765:down|Vegfa,22339:down|Mmp2,17390:down|Col11a1,12814:down|Fgfr1,14182:down|Ncam1,17967:down|Has2,15117:down|Wnt5a,22418:down|Tgfbr3,21814:up|Bmpr1a,12166:down|Wisp1,22402:down|Grem1,23892:up|Bmp4,12159:up|Igf1,16000:up|Col3a1,12825:down|Sfrp2,20319:up|Col5a1,12831:down|Tgfb2,21808:down|Vegfb,22340:up|Fgfr2,14183:up</td></tr>
<tr><td>regulation of catalytic activity</td><td>GO:0050790</td><td>1.96E-07</td><td>5.42E-06</td><td>Flrt2,399558:down|Ccl7,20306:up|Ccl2,20296:up|Pdgfc,54635:down|Cst7,13011:up|Cd109,235505:down|Trf,22041:up|Ptx3,19288:up|Cd24a,12484:down|Wnt5a,22418:down|Ccl12,20293:up|Il1rn,16181:up|Ccl4,20303:up|Adipoq,11450:up|Bmp4,12159:up|Igf1,16000:up|Tgfb2,21808:down|Fgfr2,14183:up|F3,14066:up|Kitl,17311:up|Htr2b,15559:down|Kng1,16644:up|Hp,15439:up|Ccl9,20308:up|Ccl6,20305:up|Slpi,20568:up|Aspn,66695:down|Ccl3,20302:up|Tfpi2,21789:down|Vdr,22337:down|Fabp4,11770:up|Vegfa,22339:down|Htr2a,15558:down|Serpine2,20720:up|Lrrc15,74488:down|Oas3,246727:up|Igf2,16002:up|Pparg,19016:up|Gas6,14456:up|Tnf,21926:up|Fgfr1,14182:down|Egfr,13649:down|Sfrp1,20377:up|Cd4,12504:up|Grem1,23892:up|Serinc2,230779:down|Ceacam1,26365:up|Ccl8,20307:up|Sfrp2,20319:up|Serping1,12258:up|Serpina3g,20715:up|Npnt,114249:up|Prkar2b,19088:up|Errfi1,74155:down</td></tr>
<tr><td>transmembrane receptor protein tyrosine kinase signaling pathway</td><td>GO:0007169</td><td>2.18E-07</td><td>5.97E-06</td><td>Igfbp4,16010:up|Flrt2,399558:down|Efemp1,216616:up|Ccl2,20296:up|Pdgfc,54635:down|Figf,14205:up|Plat,18791:down|Vegfa,22339:down|Igfbp3,16009:up|Adamts12,239337:down|Igf2,16002:up|Dok2,13449:up|Fgfr1,14182:down|Egfr,13649:down|Sulf1,240725:down|Wnt5a,22418:down|Adipoq,11450:up|Bmp4,12159:up|Igf1,16000:up|Ceacam1,26365:up|Ret,19713:up|Ptgir,19222:up|Errfi1,74155:down|Enpp1,18605:down|Fgfr2,14183:up|F3,14066:up</td></tr>
</table>

<table>
<tr><td>negative regulation of response to stimulus</td><td>GO:0048585</td><td>2.25E-07</td><td>6.14E-06</td><td>Fstl3,83554:down|Clec2d,93694:up|Adm,11535:up|Flrt2,399558:down|Sfrp4,20379:up|Htra3,78558:down|Cx3cr1,13051:down|Cd109,235505:down|Wnt5a,22418:down|Ccl12,20293:up|Il1rn,16181:up|Adipoq,11450:up|Bmp4,12159:up|Igf1,16000:up|Chst11,58250:down|Tgfb2,21808:down|Ptgir,19222:up|Creb3l1,26427:down|Il1rl1,17082:down|Cthrc1,68588:down|Kng1,16644:up|Htr2b,15559:down|Hp,15439:up|Aoah,27052:up|Aspn,66695:down|Ctla2a,13024:down|Cxcl12,20315:up|Serpine2,20720:up|Adamts12,239337:down|Lrrc15,74488:down|Igf2,16002:up|Nxn,18230:down|Pparg,19016:up|Gas6,14456:up|Tnf,21926:up|Tnfrsf23,79201:down|Hmox1,15368:up|Sfrp1,20377:up|Sulf1,240725:down|C1qtnf3,81799:down|Tgfbr3,21814:up|Grem1,23892:up|Col3a1,12825:down|Ceacam1,26365:up|Sfrp2,20319:up|Serping1,12258:up|Errfi1,74155:down|Enpp1,18605:down</td></tr>
<tr><td>negative regulation of signaling</td><td>GO:0023057</td><td>2.29E-07</td><td>6.24E-06</td><td>Fstl3,83554:down|Adm,11535:up|Flrt2,399558:down|Sfrp4,20379:up|Htra3,78558:down|Gpm6b,14758:up|Cx3cr1,13051:down|Cd109,235505:down|Plk2,20620:down|Wnt5a,22418:down|Il1rn,16181:up|Adipoq,11450:up|Bmp4,12159:up|Igf1,16000:up|Chst11,58250:down|Tgfb2,21808:down|Ptgir,19222:up|Creb3l1,26427:down|Il1rl1,17082:down|Cthrc1,68588:down|Htr2b,15559:down|Aspn,66695:down|Cxcl12,20315:up|Htr2a,15558:down|Serpine2,20720:up|Adamts12,239337:down|Lrrc15,74488:down|Nxn,18230:down|Pparg,19016:up|Gas6,14456:up|Tnf,21926:up|Tnfrsf23,79201:down|Sfrp1,20377:up|Hmox1,15368:up|Sulf1,240725:down|C1qtnf3,81799:down|Tgfbr3,21814:up|Ptgs2,19225:down|Grem1,23892:up|Ceacam1,26365:up|Sfrp2,20319:up|Errfi1,74155:down|Enpp1,18605:down</td></tr>
<tr><td>tube morphogenesis</td><td>GO:0035239</td><td>2.33E-07</td><td>6.31E-06</td><td>Cthrc1,68588:down|Adm,11535:up|Plxnd1,67784:up|Ptk7,71461:down|Aldh1a2,19378:up|Mgp,17313:up|Vdr,22337:down|Cxcl12,20315:up|Vegfa,22339:down|Adamts12,239337:down|Tnf,21926:up|Egfr,13649:down|Sfrp1,20377:up|Wnt5a,22418:down|Grem1,23892:up|Bmp4,12159:up|Igf1,16000:up|Sfrp2,20319:up|Tnc,21923:down|Ret,19713:up|Rspo2,239405:up|Npnt,114249:up|Fgfr2,14183:up</td></tr>
<tr><td>regulation of tissue remodeling</td><td>GO:0034103</td><td>2.35E-07</td><td>6.35E-06</td><td>Trf,22041:up|Egfr,13649:down|Cd24a,12484:down|Sfrp1,20377:up|Tfrc,22042:up|Grem1,23892:up|Vdr,22337:down|Ceacam1,26365:up|Vegfa,22339:down|Tnfrsf11b,18383:up</td></tr>
<tr><td>positive regulation of protein transport</td><td>GO:0051222</td><td>2.38E-07</td><td>6.40E-06</td><td>Htr2b,15559:down|Ice1,218333:down|Ccl2,20296:up|Ccl3,20302:up|Mmp13,17386:up|Ankrd1,107765:down|Clec4n,56620:up|Postn,50706:down|Gas6,14456:up|Tnf,21926:up|Glp1r,14652:down|Egfr,13649:down|Lpl,16956:up|C1qtnf3,81799:down|Wnt5a,22418:down|Bmpr1a,12166:down|Ptgs2,19225:down|Grem1,23892:up|Bmp4,12159:up|Igf1,16000:up|Ceacam1,26365:up|Sfrp2,20319:up|Tgfb2,21808:down|Il1rl1,17082:down|Clec4e,56619:up</td></tr>
<tr><td>positive regulation of smooth muscle cell proliferation</td><td>GO:0048661</td><td>2.47E-07</td><td>6.61E-06</td><td>Tnf,21926:up|Egfr,13649:down|Hmox1,15368:up|Bmpr1a,12166:down|Wisp1,22402:down|Ptgs2,19225:down|Igf1,16000:up|Bmp4,12159:up|Vegfa,22339:down|Mmp2,17390:down|Fgfr2,14183:up</td></tr>
<tr><td>regulation of transmembrane receptor protein serine/threonine kinase signaling pathway</td><td>GO:0090092</td><td>2.70E-07</td><td>7.18E-06</td><td>Fstl3,83554:down|Inhba,16323:down|Aspn,66695:down|Htra3,78558:down|Cd109,235505:down|Sfrp1,20377:up|Tgfbr3,21814:up|Bmpr1a,12166:down|Wnt5a,22418:down|Sulf1,240725:down|Grem1,23892:up|Bmp4,12159:up|Chst11,58250:down|Sfrp2,20319:up|Tgfb2,21808:down|Npnt,114249:up</td></tr>
<tr><td>secretion</td><td>GO:0046903</td><td>2.85E-07</td><td>7.56E-06</td><td>Adm,11535:up|Ednrb,13618:up|Mmp13,17386:up|Ankrd1,107765:down|Clec4n,56620:up|Postn,50706:down|Cdo1,12583:up|Ncam1,17967:down|Wnt5a,22418:down|Stc1,20855:up|Il1rn,16181:up|Maob,109731:up|Adipoq,11450:up|Igf1,16000:up|Tgfb2,21808:down|Creb3l1,26427:down|Il1rl1,17082:down|Clec4e,56619:up|Htr2b,15559:down|Inhba,16323:down|Ccl3,20302:up|Vdr,22337:down|Cxcl12,20315:up|Vegfa,22339:down|Htr2a,15558:down|Serpine2,20720:up|Anpep,16790:up|Pparg,19016:up|Gas6,14456:up|Tnf,21926:up|Glp1r,14652:down|Egfr,13649:down|Fgfr1,14182:down|Sfrp1,20377:up|Lpl,16956:up|Hmox1,15368:up|C1qtnf3,81799:down|Sncg,20618:up|Ceacam1,26365:up|Syt13,80976:up</td></tr>
<tr><td>single-organism metabolic process</td><td>GO:0044710</td><td>2.91E-07</td><td>7.67E-06</td><td>Igfbp4,16010:up|Adm,11535:up|Hpgd,15446:up|Sfrp4,20379:up|C3,12266:up|Enpp5,83965:up|Serpinh1,12406:down|Gstt1,14871:up|Blvrb,233016:up|Igfbp3,16009:up|Ggt5,23887:up|Loxl2,94352:down|Loxl3,16950:down|Trf,22041:up|Cd24a,12484:down|Mme,17380:up|Wnt5a,22418:down|Il1rn,16181:up|Maob,109731:up|Igf1,16000:up|Adipoq,11450:up|Bmp4,12159:up|Hal,15109:up|Crabp2,12904:down|Tgfb2,21808:down|Hpse,15442:up|Ptgir,19222:up|Fgfr2,14183:up|Htr2b,15559:down|Ccl6,20305:up|Gdpd1,66569:up|Vdr,22337:down|Fabp4,11770:up|Fam132b,227358:down|Nabp1,109019:down|Htr2a,15558:down|Mmp2,17390:down|Gas6,14456:up|Egfr,13649:down|Sfrp1,20377:up|C1qtnf3,81799:down|Tgfbr3,21814:up|C1rb,667277:up|Tfrc,22042:up|Serinc2,230779:down|Ceacam1,26365:up|Ccl8,20307:up|Sfrp2,20319:up|Pxdn,69675:down|Npnt,114249:up|Tnfrsf11b,18383:up|Prkar2b,19088:up|Aldh1a2,19378:up|Ccl2,20296:up|Ccl7,20306:up|Pdgfc,54635:down|Ampd3,11717:up|Mmp13,17386:up|Sema7a,20361:down|Marc1,66112:up|Sord,20322:up|Uchl5,56207:up|Ddah1,69219:down|Steap1,70358:down|Npl,74091:up|Enpp3,209558:down|Man2a2,140481:up|Cdo1,12583:up|Dok2,13449:up|Mamdc2,71738:down|Akr1c18,105349:up|Fabp5,16592:up|Ccl12,20293:up|Ccl4,20303:up|Chst11,58250:down|Gatm,67092:down|Ret,19713:up|C1s2,317677:up|Pdxk,216134:up|Vegfb,22340:up|Sdf2l1,64136:up|Aldh1a3,56847:down|Plpp3,67916:up|Mrc2,17534:down|Kitl,17311:up|P4ha1,18451:down|Hp,15439:up|Ccl9,20308:up|Inhba,16323:down|Aoah,27052:up|Ccl3,20302:up|C1s1,50908:up|Plod2,26432:down|Vegfa,22339:down|Gfpt2,14584:down|Cbr2,12409:up|Nxn,18230:down|Igf2,16002:up|Pparg,19016:up|Tnf,21926:up|Lmcd1,30937:up|Fgfr1,14182:down|Has2,15117:down|Lpl,16956:up|Hmox1,15368:up|Sulf1,240725:down|Ptgs2,19225:down|Grem1,23892:up|Col5a1,12831:down|Serping1,12258:up|Uck2,80914:down|Errfi1,74155:down|Enpp1,18605:down</td></tr>
<tr><td>branching morphogenesis of an epithelial tube</td><td>GO:0048754</td><td>3.40E-07</td><td>8.89E-06</td><td>Tnf,21926:up|Plxnd1,67784:up|Wnt5a,22418:down|Mgp,17313:up|Grem1,23892:up|Igf1,16000:up|Bmp4,12159:up|Vdr,22337:down|Sfrp2,20319:up|Tnc,21923:down|Cxcl12,20315:up|Vegfa,22339:down|Rspo2,239405:up|Npnt,114249:up|Fgfr2,14183:up</td></tr>
<tr><td>developmental growth</td><td>GO:0048589</td><td>3.45E-07</td><td>8.99E-06</td><td>Adm,11535:up|Plxnd1,67784:up|Ptk7,71461:down|C3,12266:up|Bnc2,242509:down|Ninj1,18081:up|Mmp13,17386:up|Sema7a,20361:down|Tnfrsf12a,27279:down|Cxcl12,20315:up|Postn,50706:down|Vegfa,22339:down|Igf2,16002:up|Fgfr1,14182:down|Ncam1,17967:down|Sfrp1,20377:up|Wnt5a,22418:down|Tgfbr3,21814:up|Bmpr1a,12166:down|Wisp1,22402:down|Stc1,20855:up|Bmp4,12159:up|Igf1,16000:up|Chst11,58250:down|Crabp2,12904:down|Sfrp2,20319:up|Tnc,21923:down|Tgfb2,21808:down|Rspo2,239405:up|Fgfr2,14183:up</td></tr>
<tr><td>negative regulation of cell communication</td><td>GO:0010648</td><td>3.49E-07</td><td>9.05E-06</td><td>Fstl3,83554:down|Adm,11535:up|Flrt2,399558:down|Sfrp4,20379:up|Htra3,78558:down|Gpm6b,14758:up|Cx3cr1,13051:down|Cd109,235505:down|Plk2,20620:down|Wnt5a,22418:down|Il1rn,16181:up|Adipoq,11450:up|Bmp4,12159:up|Igf1,16000:up|Chst11,58250:down|Tgfb2,21808:down|Ptgir,19222:up|Creb3l1,26427:down|Il1rl1,17082:down|Cthrc1,68588:down|Htr2b,15559:down|Aspn,66695:down|Cxcl12,20315:up|Htr2a,15558:down|Serpine2,20720:up|Adamts12,239337:down|Lrrc15,74488:down|Nxn,18230:down|Pparg,19016:up|Gas6,14456:up|Tnf,21926:up|Tnfrsf23,79201:down|Sfrp1,20377:up|Hmox1,15368:up|Sulf1,240725:down|C1qtnf3,81799:down|Tgfbr3,21814:up|Ptgs2,19225:down|Grem1,23892:up|Ceacam1,26365:up|Sfrp2,20319:up|Errfi1,74155:down|Enpp1,18605:down</td></tr>
</table>

collagen metabolic process	GO:0032963	3.64E-07	9.41E-06	Pparg,19016:up|Tgfbr3,21814:up|Ccl2,20296:up|Mmp13,17386:up|Bmp4,12159:up|Serpinh1,12406:down|Col5a1,12831:down|Mmp2,17390:down|Errfi1,74155:down|Mrc2,17534:down
positive regulation of protein metabolic process	GO:0051247	3.99E-07	1.02E-05	Igfbp4,16010:up|Ccl7,20306:up|Ccl2,20296:up|C3,12266:up|Pdgfc,54635:down|Ednrb,13618:up|Sema7a,20361:down|Igfbp3,16009:up|Plk2,20620:down|Trf,22041:up|Cd24a,12484:down|Wnt5a,22418:down|Bmpr1a,12166:down|Ccl12,20293:up|Il1rn,16181:up|Ccl4,20303:up|Adipoq,11450:up|Bmp4,12159:up|Igf1,16000:up|Ret,19713:up|Tgfb2,21808:down|Plpp3,67916:up|Fgfr2,14183:up|F3,14066:up|Kitl,17311:up|Htr2b,15559:down|Ccl9,20308:up|Ccl6,20305:up|Inhba,16323:down|Ccl3,20302:up|Crlf1,12931:down|Vegfa,22339:down|Htr2a,15558:down|Igf2,16002:up|Pparg,19016:up|Gas6,14456:up|Tnf,21926:up|Fgfr1,14182:down|Egfr,13649:down|C1qtnf3,81799:down|Cd4,12504:up|Grem1,23892:up|Ccl8,20307:up|Ceacam1,26365:up|Sfrp2,20319:up|Fam20a,208659:down|Tnfrsf11b,18383:up|Npnt,114249:up
hemopoiesis	GO:0030097	4.19E-07	1.07E-05	Fstl3,83554:down|Clec2d,93694:up|Tmem176b,65963:up|Ly9,17085:up|Epas1,13819:up|Lyl1,17095:up|Cd109,235505:down|Trf,22041:up|Cd24a,12484:down|Wnt5a,22418:down|Adipoq,11450:up|Bmp4,12159:up|Prg4,96875:down|Tgfb2,21808:down|Fgfr2,14183:up|Clec4e,56619:up|Kitl,17311:up|Lrrc17,74511:down|Inhba,16323:down|Ccl3,20302:up|Ctla2a,13024:down|Vegfa,22339:down|Tnf,21926:up|Pparg,19016:up|Gas6,14456:up|Tmem176a,66058:up|Sfrp1,20377:up|Tgfbr3,21814:up|Cd4,12504:up|Tfrc,22042:up|Ceacam1,26365:up|Sfrp2,20319:up|Tnfsf9,21950:up
multicellular organismal macromolecule metabolic process	GO:0044259	4.20E-07	1.07E-05	Pparg,19016:up|Tgfbr3,21814:up|Ccl2,20296:up|Mmp13,17386:up|Bmp4,12159:up|Serpinh1,12406:down|Col5a1,12831:down|Mmp2,17390:down|Errfi1,74155:down|Mrc2,17534:down
embryonic morphogenesis	GO:0048598	4.22E-07	1.07E-05	Cthrc1,68588:down|Htr2b,15559:down|Col5a2,12832:down|Adm,11535:up|Ptk7,71461:down|Inhba,16323:down|Gjb5,14622:down|Aldh1a2,19378:up|Fgfr1,14182:down|Col11a1,12814:down|Sfrp1,20377:up|Sulf1,240725:down|Wnt5a,22418:down|Bmpr1a,12166:down|Il1rn,16181:up|Grem1,23892:up|Adipoq,11450:up|Bmp4,12159:up|Chst11,58250:down|Crabp2,12904:down|Sfrp2,20319:up|Tbx15,21384:down|Col5a1,12831:down|Ret,19713:up|Tgfb2,21808:down|Rspo2,239405:up|Aldh1a3,56847:down|Plpp3,67916:up|Fgfr2,14183:up
regulation of MAP kinase activity	GO:0043405	4.27E-07	1.07E-05	Htr2b,15559:down|Pdgfc,54635:down|Vegfa,22339:down|Htr2a,15558:down|Tnf,21926:up|Trf,22041:up|Cd24a,12484:down|Egfr,13649:down|Fgfr1,14182:down|Sfrp1,20377:up|Wnt5a,22418:down|Il1rn,16181:up|Adipoq,11450:up|Bmp4,12159:up|Igf1,16000:up|Ceacam1,26365:up|Sfrp2,20319:up|Kitl,17311:up
chemotaxis	GO:0006935	4.37E-07	1.09E-05	Flrt2,399558:down|Lama2,16773:down|Ccl9,20308:up|Plxnd1,67784:up|Ccl6,20305:up|Ccl7,20306:up|Ccl2,20296:up|Figf,14205:up|Ednrb,13618:up|Ccl3,20302:up|Saa3,20210:up|Cxcr6,80901:down|Cxcl12,20315:up|Vegfa,22339:down|Cx3cr1,13051:down|Gas6,14456:up|Cxcl14,57266:down|Fgfr1,14182:down|Wnt5a,22418:down|Ccl12,20293:up|Ccl4,20303:up|Grem1,23892:up|Ccl8,20307:up|Cxcl9,17329:up|Tgfb2,21808:down
regulation of growth	GO:0040008	4.43E-07	1.10E-05	Igfbp4,16010:up|Plxnd1,67784:up|Inhba,16323:down|C3,12266:up|Sema7a,20361:down|Htra3,78558:down|Tnfrsf12a,27279:down|Cxcl12,20315:up|Vegfa,22339:down|Serpine2,20720:up|Igfbp3,16009:up|Igf2,16002:up|Gas6,14456:up|Pparg,19016:up|Tnf,21926:up|Fgfr1,14182:down|Egfr,13649:down|Ncam1,17967:down|Sfrp1,20377:up|Wnt5a,22418:down|Bmpr1a,12166:down|Wisp1,22402:down|Grem1,23892:up|Bmp4,12159:up|Igf1,16000:up|Crabp2,12904:down|Sfrp2,20319:up|Tgfb2,21808:down|Enpp1,18605:down|Fgfr2,14183:up
leukocyte differentiation	GO:0002521	4.55E-07	1.12E-05	Fstl3,83554:down|Clec2d,93694:up|Lrrc17,74511:down|Tmem176b,65963:up|Ly9,17085:up|Ccl3,20302:up|Ctla2a,13024:down|Lyl1,17095:up|Vegfa,22339:down|Cd109,235505:down|Tmem176a,66058:up|Gas6,14456:up|Pparg,19016:up|Tnf,21926:up|Trf,22041:up|Cd24a,12484:down|Sfrp1,20377:up|Cd4,12504:up|Tfrc,22042:up|Adipoq,11450:up|Bmp4,12159:up|Ceacam1,26365:up|Kitl,17311:up|Tnfsf9,21950:up|Clec4e,56619:up
taxis	GO:0042330	4.75E-07	1.17E-05	Flrt2,399558:down|Lama2,16773:down|Ccl9,20308:up|Plxnd1,67784:up|Ccl6,20305:up|Ccl7,20306:up|Ccl2,20296:up|Figf,14205:up|Ednrb,13618:up|Ccl3,20302:up|Saa3,20210:up|Cxcr6,80901:down|Cxcl12,20315:up|Vegfa,22339:down|Cx3cr1,13051:down|Gas6,14456:up|Cxcl14,57266:down|Fgfr1,14182:down|Wnt5a,22418:down|Ccl12,20293:up|Ccl4,20303:up|Grem1,23892:up|Ccl8,20307:up|Cxcl9,17329:up|Tgfb2,21808:down
positive regulation of cytokine production	GO:0001819	4.83E-07	1.18E-05	Htr2b,15559:down|Ly9,17085:up|Ccl2,20296:up|C3,12266:up|Figf,14205:up|Ccl3,20302:up|Sema7a,20361:down|Clec4n,56620:up|Postn,50706:down|Tnf,21926:up|Lpl,16956:up|Sulf1,240725:down|C1qtnf3,81799:down|Wnt5a,22418:down|Ptgs2,19225:down|Ccl4,20303:up|Adipoq,11450:up|Hpse,15442:up|Tnfsf9,21950:up|Clec4e,56619:up|Il1rl1,17082:down
positive regulation of protein secretion	GO:0050714	4.94E-07	1.20E-05	Htr2b,15559:down|Ccl3,20302:up|Mmp13,17386:up|Ankrd1,107765:down|Clec4n,56620:up|Postn,50706:down|Tnf,21926:up|Glp1r,14652:down|Egfr,13649:down|Lpl,16956:up|Wnt5a,22418:down|C1qtnf3,81799:down|Igf1,16000:up|Ceacam1,26365:up|Tgfb2,21808:down|Clec4e,56619:up|Il1rl1,17082:down
bone development	GO:0060348	4.99E-07	1.21E-05	Fam101b,76566:down|Lrrc17,74511:down|Alpl,11647:up|Has2,15117:down|Sulf1,240725:down|Pdgfc,54635:down|Stc1,20855:up|Bnc2,242509:down|Grem1,23892:up|Mmp13,17386:up|Igf1,16000:up|Bmp4,12159:up|Serpinh1,12406:down|Sfrp2,20319:up|Fgfr2,14183:up
positive regulation of cell adhesion	GO:0045785	5.08E-07	1.23E-05	Fstl3,83554:down|Ccl2,20296:up|Ninj1,18081:up|Cxcl12,20315:up|Vegfa,22339:down|Smoc2,64074:down|Igf2,16002:up|Tnf,21926:up|Cd24a,12484:down|Has2,15117:down|Wnt5a,22418:down|Cd4,12504:up|Tfrc,22042:up|Col8a1,12837:down|Igf1,16000:up|Ceacam1,26365:up|Ret,19713:up|Tgfb2,21808:down|Plpp3,67916:up|Npnt,114249:up|Tnfsf9,21950:up
regulation of protein kinase activity	GO:0045859	5.13E-07	1.24E-05	Htr2b,15559:down|Flrt2,399558:down|Pdgfc,54635:down|Aspn,66695:down|Fabp4,11770:up|Vegfa,22339:down|Htr2a,15558:down|Lrrc15,74488:down|Gas6,14456:up|Tnf,21926:up|Trf,22041:up|Fgfr1,14182:down|Egfr,13649:down|Cd24a,12484:down|Sfrp1,20377:up|Wnt5a,22418:down|Cd4,12504:up|Il1rn,16181:up|Grem1,23892:up|Adipoq,11450:up|Bmp4,12159:up|Igf1,16000:up|Ceacam1,26365:up|Sfrp2,20319:up|Tgfb2,21808:down|Prkar2b,19088:up|Errfi1,74155:down|Kitl,17311:up
negative regulation of signal transduction	GO:0009968	5.45E-07	1.30E-05	Fstl3,83554:down|Adm,11535:up|Flrt2,399558:down|Sfrp4,20379:up|Htra3,78558:down|Cx3cr1,13051:down|Cd109,235505:down|Wnt5a,22418:down|Il1rn,16181:up|Adipoq,11450:up|Bmp4,12159:up|Igf1,16000:up|Chst11,58250:down|Tgfb2,21808:down|Ptgir,19222:up|Creb3l1,26427:down|Il1rl1,17082:down|Htr2b,15559:down|Cthrc1,68588:down|Aspn,66695:down|Cxcl12,20315:up|Serpine2,20720:up|Adamts12,239337:down|Nxn,18230:down|Lrrc15,74488:down|Tnf,21926:up|Pparg,19016:up|Gas6,14456:up|Tnfrsf23,79201:down|Sfrp1,20377:up|Hmox1,15368:up|Sulf1,240725:down|C1qtnf3,81799:down|Tgfbr3,21814:up|Grem1,23892:up|Ceacam1,26365:up|Sfrp2,20319:up|Enpp1,18605:down|Errfi1,74155:down

<table>
<tr><td>regulation of molecular function</td><td>GO:0065009</td><td>6.06E-07</td><td>1.44E-05</td><td>Flrt2,399558:down|Plxnd1,67784:up|Sfrp4,20379:up|Cd109,235505:down|Plk2,20620:down|Trf,22041:up|Cd24a,12484:down|Wnt5a,22418:down|Il1rn,16181:up|Adipoq,11450:up|Bmp4,12159:up|Igf1,16000:up|Tgfb2,21808:down|F3,14066:up|Fgfr2,14183:up|Cthrc1,68588:down|Htr2b,15559:down|Kng1,16644:up|Ccl6,20305:up|Slpi,20568:up|Aspn,66695:down|Vdr,22337:down|Fabp4,11770:up|Htr2a,15558:down|Oas3,246727:up|Gas6,14456:up|Egfr,13649:down|Sfrp1,20377:up|Tgfbr3,21814:up|Fxyd2,11936:up|Serinc2,230779:down|Ceacam1,26365:up|Ccl8,20307:up|Sfrp2,20319:up|Serpina3g,20715:up|Npnt,114249:up|Prkar2b,19088:up|S100a1,20193:up|Ccl2,20296:up|Ccl7,20306:up|Cst7,13011:up|Pdgfc,54635:down|Ptx3,19288:up|Ccl12,20293:up|Ccl4,20303:up|Plpp3,67916:up|Kitl,17311:up|Ccl9,20308:up|Hp,15439:up|Ccl3,20302:up|Tfpi2,21789:down|Vegfa,22339:down|Serpine2,20720:up|Igf2,16002:up|Lrrc15,74488:down|Pparg,19016:up|Tnf,21926:up|Fxyd1,56188:up|Fgfr1,14182:down|Hmox1,15368:up|Cd4,12504:up|Grem1,23892:up|Serping1,12258:up|Errfi1,74155:down</td></tr>
<tr><td>response to transition metal nanoparticle</td><td>GO:1990267</td><td>6.31E-07</td><td>1.49E-05</td><td>Loxl2,94352:down|Hvcn1,74096:up|Trf,22041:up|Hmox1,15368:up|Tfrc,22042:up|Mt2,17750:up|Sord,20322:up|Gatm,67092:down|Mt1,17748:up|Slc40a1,53945:up</td></tr>
<tr><td>myeloid leukocyte migration</td><td>GO:0097529</td><td>6.56E-07</td><td>1.54E-05</td><td>Ccl9,20308:up|Ccl6,20305:up|Ccl7,20306:up|Ccl2,20296:up|Ccl12,20293:up|Figf,14205:up|Ednrb,13618:up|Grem1,23892:up|Ccl4,20303:up|Ccl3,20302:up|Ccl8,20307:up|Cxcl12,20315:up|Cx3cr1,13051:down</td></tr>
<tr><td>muscle structure development</td><td>GO:0061061</td><td>6.65E-07</td><td>1.55E-05</td><td>Prl2c2,18811:up|Adm,11535:up|Ccl9,20308:up|Ednrb,13618:up|Epas1,13819:up|Ankrd1,107765:down|Cxcl12,20315:up|Vegfa,22339:down|Igfbp3,16009:up|Igf2,16002:up|Tnf,21926:up|Cxcl14,57266:down|Fgfr1,14182:down|Col11a1,12814:down|Ncam1,17967:down|Wnt5a,22418:down|Bmpr1a,12166:down|Tgfbr3,21814:up|Wisp1,22402:down|Grem1,23892:up|Col3a1,12825:down|Bmp4,12159:up|Igf1,16000:up|Ccl8,20307:up|Cxcl9,17329:up|Tgfb2,21808:down|Npnt,114249:up|Fgfr2,14183:up</td></tr>
<tr><td>negative regulation of cell proliferation</td><td>GO:0008285</td><td>6.65E-07</td><td>1.55E-05</td><td>Adm,11535:up|Inhba,16323:down|Sfrp4,20379:up|Aldh1a2,19378:up|Vdr,22337:down|Serpine2,20720:up|Igfbp3,16009:up|Cd109,235505:down|Tnf,21926:up|Pparg,19016:up|Cd24a,12484:down|Hmox1,15368:up|Sfrp1,20377:up|Sulf1,240725:down|Wnt5a,22418:down|Ccl12,20293:up|Tgfbr3,21814:up|Ptgs2,19225:down|Grem1,23892:up|Adipoq,11450:up|Bmp4,12159:up|Igf1,16000:up|Ceacam1,26365:up|Sfrp2,20319:up|Tgfb2,21808:down|Ptgir,19222:up|Fgfr2,14183:up|Il1rl1,17082:down</td></tr>
<tr><td>regulation of cell development</td><td>GO:0060284</td><td>6.87E-07</td><td>1.60E-05</td><td>Flrt2,399558:down|Plxnd1,67784:up|Ednrb,13618:up|Sema7a,20361:down|Ankrd1,107765:down|Tnfrsf12a,27279:down|Postn,50706:down|Cx3cr1,13051:down|Plk2,20620:down|Trf,22041:up|Cd24a,12484:down|Wnt5a,22418:down|Bmpr1a,12166:down|Wisp1,22402:down|Adipoq,11450:up|Bmp4,12159:up|Igf1,16000:up|Crabp2,12904:down|Ret,19713:up|Tgfb2,21808:down|Fam101b,76566:down|Prl2c2,18811:up|Efemp1,216616:up|Ptk7,71461:down|Dpysl3,22240:down|Vdr,22337:down|Cxcl12,20315:up|Vegfa,22339:down|Serpine2,20720:up|Tnf,21926:up|Pparg,19016:up|Fgfr1,14182:down|Sfrp1,20377:up|Has2,15117:down|Tgfbr3,21814:up|Grem1,23892:up|Col3a1,12825:down|Sfrp2,20319:up</td></tr>
<tr><td>cation homeostasis</td><td>GO:0055080</td><td>6.89E-07</td><td>1.60E-05</td><td>Htr2b,15559:down|Kng1,16644:up|Adm,11535:up|Ccl2,20296:up|Ednrb,13618:up|Ccl3,20302:up|Mt2,17750:up|Epas1,13819:up|Vdr,22337:down|Fam132b,227358:down|Steap1,70358:down|Htr2a,15558:down|Slc40a1,53945:up|Anpep,16790:up|Glp1r,14652:down|Fxyd1,56188:up|Trf,22041:up|Egfr,13649:down|Cd24a,12484:down|Hmox1,15368:up|Cd4,12504:up|Stc1,20855:up|Tfrc,22042:up|Bmp4,12159:up|Tgfb2,21808:down|Fam20a,208659:down|Ptgir,19222:up|Mt1,17748:up</td></tr>
<tr><td>immune system development</td><td>GO:0002520</td><td>6.99E-07</td><td>1.61E-05</td><td>Fstl3,83554:down|Clec2d,93694:up|Tmem176b,65963:up|Ly9,17085:up|Epas1,13819:up|Lyl1,17095:up|Cd109,235505:down|Trf,22041:up|Cd24a,12484:down|Wnt5a,22418:down|Adipoq,11450:up|Bmp4,12159:up|Ret,19713:up|Prg4,96875:down|Tgfb2,21808:down|Fgfr2,14183:up|Clec4e,56619:up|Kitl,17311:up|Lrrc17,74511:down|Inhba,16323:down|Ccl3,20302:up|Ctla2a,13024:down|Vegfa,22339:down|Slc40a1,53945:up|Tnf,21926:up|Pparg,19016:up|Gas6,14456:up|Tmem176a,66058:up|Sfrp1,20377:up|Tgfbr3,21814:up|Cd4,12504:up|Tfrc,22042:up|Ceacam1,26365:up|Sfrp2,20319:up|Tnfsf9,21950:up</td></tr>
<tr><td>positive regulation of cardiac muscle tissue development</td><td>GO:0055025</td><td>7.12E-07</td><td>1.64E-05</td><td>Fgfr1,14182:down|Ncam1,17967:down|Bmpr1a,12166:down|Wisp1,22402:down|Grem1,23892:up|Igf1,16000:up|Bmp4,12159:up|Fgfr2,14183:up</td></tr>
<tr><td>ion homeostasis</td><td>GO:0050801</td><td>7.19E-07</td><td>1.65E-05</td><td>Htr2b,15559:down|Kng1,16644:up|Adm,11535:up|Sfrp4,20379:up|Ccl2,20296:up|Ednrb,13618:up|Ccl3,20302:up|Mt2,17750:up|Epas1,13819:up|Vdr,22337:down|Fam132b,227358:down|Steap1,70358:down|Htr2a,15558:down|Slc40a1,53945:up|Anpep,16790:up|Glp1r,14652:down|Fxyd1,56188:up|Trf,22041:up|Egfr,13649:down|Cd24a,12484:down|Hmox1,15368:up|Cd4,12504:up|Tfrc,22042:up|Stc1,20855:up|Bmp4,12159:up|Tgfb2,21808:down|Fam20a,208659:down|Ptgir,19222:up|Enpp1,18605:down|Mt1,17748:up</td></tr>
<tr><td>cellular response to stimulus</td><td>GO:0051716</td><td>7.50E-07</td><td>1.71E-05</td><td>Fstl3,83554:down|Igfbp4,16010:up|Adm,11535:up|Flrt2,399558:down|Plxnd1,67784:up|Tfec,21426:up|Sfrp4,20379:up|Gstt1,14871:up|Igfbp3,16009:up|Ibsp,15891:up|Plk2,20620:down|Cd24a,12484:down|Mme,17380:up|Il1rn,16181:up|Bmp4,12159:up|Hpse,15442:up|Ptgir,19222:up|Fgfr2,14183:up|Htr2b,15559:down|Ccl6,20305:up|Aspn,66695:down|Mt2,17750:up|Cxcr6,80901:down|Nabp1,109019:down|Mmp2,17390:down|Slc40a1,53945:up|Egfr,13649:down|Alpl,11647:up|Tfrc,22042:up|Ccl8,20307:up|Gstm1,14862:up|Rspo2,239405:up|Mt1,17748:up|Col5a2,12832:down|Mrc1,17533:up|Aldh1a2,19378:up|Ccl2,20296:up|Ednrb,13618:up|Htra3,78558:down|Tnfrsf12a,27279:down|Uchl5,56207:up|Clec4n,56620:up|Dok2,13449:up|Hvcn1,74096:up|Ncam1,17967:down|Ccl4,20303:up|Chst11,58250:down|Aldh1a3,56847:down|Plpp3,67916:up|Ccl9,20308:up|Ptk7,71461:down|Inhba,16323:down|Crlf1,12931:down|Plod2,26432:down|Vegfa,22339:down|Serpine2,20720:up|Nxn,18230:down|Igf2,16002:up|Tnf,21926:up|Col1a2,12843:down|Fgfr1,14182:down|Sulf1,240725:down|Cd4,12504:up|Col3a1,12825:down|Enpp1,18605:down|Hpgd,15446:up|C3,12266:up|Ankrd1,107765:down|Cx3cr1,13051:down|Cd109,235505:down|Trf,22041:up|Cxcl14,57266:down|Wnt5a,22418:down|Mark1,226778:up|Adipoq,11450:up|Igf1,16000:up|Crabp2,12904:down|Cxcl9,17329:up|Tgfb2,21808:down|F3,14066:up|Clec4e,56619:up|Cthrc1,68588:down|Efemp1,216616:up|Saa3,20210:up|Vdr,22337:down|Pdia5,72599:down|Fabp4,11770:up|Cxcl12,20315:up|Htr2a,15558:down|Smoc2,64074:down|Gas6,14456:up|Sfrp1,20377:up|Tgfbr3,21814:up|C1qtnf3,81799:down|Ceacam1,26365:up|Sfrp2,20319:up|Tnfrsf11b,18383:up|Npnt,114249:up|Ccl7,20306:up|Figf,14205:up|Pdgfc,54635:down|Ifit3,15959:up|Epas1,13819:up|Slc29a1,63959:up|Sema7a,20361:down|Postn,50706:down|Akr1c18,105349:up|Ccl12,20293:up|Bmpr1a,12166:down|Wisp1,22402:down|Tnc,21923:down|Ret,19713:up|Sdf2l1,64136:up|Ifit1,15957:up|Creb3l1,26427:down|Kitl,17311:up|Il1rl1,17082:down|Hp,15439:up|Rasl11a,68895:up|Ccl3,20302:up|Plat,18791:down|Adamts12,239337:down|Lrrc15,74488:down|Pparg,19016:up|Lmcd1,30937:up|Glp1r,14652:down|Tnfrsf23,79201:down|Hmox1,15368:up|Has2,15117:down|Ptgs2,19225:down|Grem1,23892:up|Errfi1,74155:down</td></tr>
<tr><td>anatomical structure maturation</td><td>GO:0071695</td><td>7.60E-07</td><td>1.73E-05</td><td>Fam101b,76566:down|Aldh1a2,19378:up|Ptgs2,19225:down|Grem1,23892:up|Igf1,16000:up|Lyl1,17095:up|Ret,19713:up|Tgfb2,21808:down|Mmp2,17390:down</td></tr>
<tr><td>negative regulation of cellular response to growth factor stimulus</td><td>GO:0090288</td><td>7.68E-07</td><td>1.74E-05</td><td>Fstl3,83554:down|Sfrp1,20377:up|Sulf1,240725:down|Wnt5a,22418:down|Tgfbr3,21814:up|Aspn,66695:down|Grem1,23892:up|Chst11,58250:down|Htra3,78558:down|Sfrp2,20319:up|Adamts12,239337:down|Cd109,235505:down</td></tr>
</table>

positive regulation of catalytic activity	GO:0043085	8.18E-07	1.85E-05	Ccl7,20306:up|Ccl2,20296:up|Pdgfc,54635:down|Trf,22041:up|Cd24a,12484:down|Wnt5a,22418:down|Ccl12,20293:up|Il1rn,16181:up|Ccl4,20303:up|Adipoq,11450:up|Bmp4,12159:up|Igf1,16000:up|Tgfb2,21808:down|Fgfr2,14183:up|F3,14066:up|Kitl,17311:up|Htr2b,15559:down|Ccl9,20308:up|Ccl6,20305:up|Ccl3,20302:up|Vdr,22337:down|Vegfa,22339:down|Htr2a,15558:down|Igf2,16002:up|Pparg,19016:up|Gas6,14456:up|Tnf,21926:up|Egfr,13649:down|Fgfr1,14182:down|Cd4,12504:up|Grem1,23892:up|Serinc2,230779:down|Ccl8,20307:up|Ceacam1,26365:up|Sfrp2,20319:up|Npnt,114249:up
positive regulation of MAP kinase activity	GO:0043406	8.46E-07	1.90E-05	Htr2b,15559:down|Tnf,21926:up|Trf,22041:up|Fgfr1,14182:down|Egfr,13649:down|Cd24a,12484:down|Wnt5a,22418:down|Pdgfc,54635:down|Il1rn,16181:up|Igf1,16000:up|Ceacam1,26365:up|Vegfa,22339:down|Htr2a,15558:down|Kitl,17311:up
trabecula morphogenesis	GO:0061383	8.62E-07	1.93E-05	Sfrp1,20377:up|Bmpr1a,12166:down|Tgfbr3,21814:up|Grem1,23892:up|Tgfb2,21808:down|Vegfa,22339:down|Mmp2,17390:down|Slc40a1,53945:up
chemical homeostasis	GO:0048878	9.04E-07	2.02E-05	Adm,11535:up|Sfrp4,20379:up|Ccl2,20296:up|Ednrb,13618:up|Epas1,13819:up|Steap1,70358:down|Trf,22041:up|Cd24a,12484:down|Stc1,20855:up|Adipoq,11450:up|Bmp4,12159:up|Igf1,16000:up|Tgfb2,21808:down|Ptgir,19222:up|Kng1,16644:up|Htr2b,15559:down|Mt2,17750:up|Ccl3,20302:up|Vdr,22337:down|Fabp4,11770:up|Fam132b,227358:down|Vegfa,22339:down|Htr2a,15558:down|Anpep,16790:up|Slc40a1,53945:up|Pparg,19016:up|Gas6,14456:up|Fxyd1,56188:up|Glp1r,14652:down|Egfr,13649:down|Lpl,16956:up|Hmox1,15368:up|C1qtnf3,81799:down|Cd4,12504:up|Tfrc,22042:up|Fam20a,208659:down|Enpp1,18605:down|Mt1,17748:up
death	GO:0016265	1.03E-06	2.26E-05	Adm,11535:up|Sfrp4,20379:up|Aldh1a2,19378:up|Ednrb,13618:up|Ankrd1,107765:down|Tnfrsf12a,27279:down|Cx3cr1,13051:down|Igfbp3,16009:up|Plk2,20620:down|Akr1c18,105349:up|Cd24a,12484:down|Ncam1,17967:down|Wnt5a,22418:down|Ccl12,20293:up|Wisp1,22402:down|Il1rn,16181:up|Adipoq,11450:up|Bmp4,12159:up|Igf1,16000:up|Chst11,58250:down|Tgfb2,21808:down|Pdxk,216134:up|Sdf2l1,64136:up|Aldh1a3,56847:down|Creb3l1,26427:down|Fgfr2,14183:up|F3,14066:up|Kitl,17311:up|Htr2b,15559:down|Hp,15439:up|Dram1,71712:up|Inhba,16323:down|Ccl3,20302:up|Vdr,22337:down|Crlf1,12931:down|Cxcl12,20315:up|Vegfa,22339:down|Htr2a,15558:down|Mmp2,17390:down|Slc40a1,53945:up|Pparg,19016:up|Gas6,14456:up|Tnf,21926:up|Glp1r,14652:down|Tnfrsf23,79201:down|Fgfr1,14182:down|Egfr,13649:down|Hmox1,15368:up|Sfrp1,20377:up|Sulf1,240725:down|Tgfbr3,21814:up|Ptgs2,19225:down|Grem1,23892:up|Ceacam1,26365:up|Sfrp2,20319:up|Serpina3g,20715:up|Tnfrsf11b,18383:up|Mt1,17748:up
cell death	GO:0008219	1.03E-06	2.26E-05	Adm,11535:up|Sfrp4,20379:up|Aldh1a2,19378:up|Ednrb,13618:up|Ankrd1,107765:down|Tnfrsf12a,27279:down|Cx3cr1,13051:down|Igfbp3,16009:up|Plk2,20620:down|Akr1c18,105349:up|Cd24a,12484:down|Ncam1,17967:down|Wnt5a,22418:down|Ccl12,20293:up|Wisp1,22402:down|Il1rn,16181:up|Adipoq,11450:up|Bmp4,12159:up|Igf1,16000:up|Chst11,58250:down|Tgfb2,21808:down|Pdxk,216134:up|Sdf2l1,64136:up|Aldh1a3,56847:down|Creb3l1,26427:down|Fgfr2,14183:up|F3,14066:up|Kitl,17311:up|Htr2b,15559:down|Hp,15439:up|Dram1,71712:up|Inhba,16323:down|Ccl3,20302:up|Vdr,22337:down|Crlf1,12931:down|Cxcl12,20315:up|Vegfa,22339:down|Htr2a,15558:down|Mmp2,17390:down|Slc40a1,53945:up|Pparg,19016:up|Gas6,14456:up|Tnf,21926:up|Glp1r,14652:down|Tnfrsf23,79201:down|Fgfr1,14182:down|Egfr,13649:down|Hmox1,15368:up|Sfrp1,20377:up|Sulf1,240725:down|Tgfbr3,21814:up|Ptgs2,19225:down|Grem1,23892:up|Ceacam1,26365:up|Sfrp2,20319:up|Serpina3g,20715:up|Tnfrsf11b,18383:up|Mt1,17748:up
regulation of morphogenesis of a branching structure	GO:0060688	1.03E-06	2.26E-05	Tnf,21926:up|Fgfr1,14182:down|Sfrp1,20377:up|Sulf1,240725:down|Wnt5a,22418:down|Grem1,23892:up|Bmp4,12159:up|Vegfa,22339:down|Fgfr2,14183:up
response to metal ion	GO:0010038	1.11E-06	2.43E-05	Pparg,19016:up|Loxl2,94352:down|Akr1c18,105349:up|Hvcn1,74096:up|Trf,22041:up|Egfr,13649:down|Ncam1,17967:down|Hmox1,15368:up|Tfrc,22042:up|Mt2,17750:up|Sord,20322:up|Fabp4,11770:up|Gatm,67092:down|Slc40a1,53945:up|Mt1,17748:up
regulation of secretion by cell	GO:1903530	1.11E-06	2.43E-05	Htr2b,15559:down|Inhba,16323:down|Ccl3,20302:up|Mmp13,17386:up|Ankrd1,107765:down|Clec4n,56620:up|Cxcl12,20315:up|Postn,50706:down|Htr2a,15558:down|Gas6,14456:up|Pparg,19016:up|Tnf,21926:up|Glp1r,14652:down|Fgfr1,14182:down|Egfr,13649:down|Ncam1,17967:down|Hmox1,15368:up|Lpl,16956:up|Sfrp1,20377:up|C1qtnf3,81799:down|Wnt5a,22418:down|Il1rn,16181:up|Sncg,20618:up|Maob,109731:up|Adipoq,11450:up|Igf1,16000:up|Ceacam1,26365:up|Tgfb2,21808:down|Il1rl1,17082:down|Clec4e,56619:up
epithelial cell migration	GO:0010631	1.14E-06	2.48E-05	Prl2c2,18811:up|Plxnd1,67784:up|Srpx2,68792:down|Vegfa,22339:down|Loxl2,94352:down|Fgfr1,14182:down|Has2,15117:down|Tgfbr3,21814:up|Wnt5a,22418:down|Stc1,20855:up|Ptgs2,19225:down|Grem1,23892:up|Bmp4,12159:up|Ceacam1,26365:up|Tgfb2,21808:down|Plpp3,67916:up
developmental growth involved in morphogenesis	GO:0060560	1.18E-06	2.55E-05	Plxnd1,67784:up|Ptk7,71461:down|Bnc2,242509:down|Mmp13,17386:up|Sema7a,20361:down|Tnfrsf12a,27279:down|Cxcl12,20315:up|Postn,50706:down|Vegfa,22339:down|Sfrp1,20377:up|Wnt5a,22418:down|Stc1,20855:up|Bmp4,12159:up|Sfrp2,20319:up|Crabp2,12904:down|Tnc,21923:down|Fgfr2,14183:up
epithelium migration	GO:0090132	1.30E-06	2.80E-05	Prl2c2,18811:up|Plxnd1,67784:up|Srpx2,68792:down|Vegfa,22339:down|Loxl2,94352:down|Fgfr1,14182:down|Has2,15117:down|Tgfbr3,21814:up|Wnt5a,22418:down|Stc1,20855:up|Ptgs2,19225:down|Grem1,23892:up|Bmp4,12159:up|Ceacam1,26365:up|Tgfb2,21808:down|Plpp3,67916:up
positive regulation of growth	GO:0045927	1.40E-06	3.01E-05	C3,12266:up|Sema7a,20361:down|Tnfrsf12a,27279:down|Cxcl12,20315:up|Vegfa,22339:down|Igf2,16002:up|Egfr,13649:down|Fgfr1,14182:down|Sfrp1,20377:up|Ncam1,17967:down|Bmpr1a,12166:down|Wisp1,22402:down|Igf1,16000:up|Sfrp2,20319:up|Crabp2,12904:down|Tgfb2,21808:down|Fgfr2,14183:up
endothelial cell migration	GO:0043542	1.41E-06	3.02E-05	Prl2c2,18811:up|Loxl2,94352:down|Fgfr1,14182:down|Plxnd1,67784:up|Wnt5a,22418:down|Ptgs2,19225:down|Stc1,20855:up|Grem1,23892:up|Bmp4,12159:up|Ceacam1,26365:up|Srpx2,68792:down|Vegfa,22339:down|Plpp3,67916:up
negative regulation of tissue remodeling	GO:0034104	1.43E-06	3.04E-05	Ceacam1,26365:up|Cd24a,12484:down|Sfrp1,20377:up|Vegfa,22339:down|Tnfrsf11b,18383:up|Grem1,23892:up
tissue migration	GO:0090130	1.47E-06	3.12E-05	Prl2c2,18811:up|Plxnd1,67784:up|Srpx2,68792:down|Vegfa,22339:down|Loxl2,94352:down|Fgfr1,14182:down|Has2,15117:down|Tgfbr3,21814:up|Wnt5a,22418:down|Stc1,20855:up|Ptgs2,19225:down|Grem1,23892:up|Bmp4,12159:up|Ceacam1,26365:up|Tgfb2,21808:down|Plpp3,67916:up
apoptotic process	GO:0006915	1.54E-06	3.26E-05	Adm,11535:up|Sfrp4,20379:up|Aldh1a2,19378:up|Ednrb,13618:up|Ankrd1,107765:down|Tnfrsf12a,27279:down|Cx3cr1,13051:down|Igfbp3,16009:up|Plk2,20620:down|Akr1c18,105349:up|Cd24a,12484:down|Wnt5a,22418:down|Ccl12,20293:up|Il1rn,16181:up|Adipoq,11450:up|Bmp4,12159:up|Igf1,16000:up|Chst11,58250:down|Tgfb2,21808:down|Pdxk,216134:up|Sdf2l1,64136:up|Aldh1a3,56847:down|Creb3l1,26427:down|Fgfr2,14183:up|F3,14066:up|Kitl,17311:up|Htr2b,15559:down|Dram1,71712:up|Inhba,16323:down|Ccl3,20302:up|Vdr,22337:down|Crlf1,12931:down|Cxcl12,20315:up|Vegfa,22339:down|Mmp2,17390:down|Slc40a1,53945:up|Pparg,19016:up|Gas6,14456:up|Tnf,21926:up|Glp1r,14652:down|Tnfrsf23,79201:down|Fgfr1,14182:down|Egfr,13649:down|Hmox1,15368:up|Sfrp1,20377:up|Sulf1,240725:down|Tgfbr3,21814:up|Ptgs2,19225:down|Grem1,23892:up|Ceacam1,26365:up|Sfrp2,20319:up|Serpina3g,20715:up|Tnfrsf11b,18383:up|Mt1,17748:up

<table>
<tr><td></td><td></td><td></td><td></td><td></td></tr>
<tr><td>lung development</td><td>GO:0030324</td><td>1.56E-06</td><td>3.29E-05</td><td>Tnf,21926:up|Fgfr1,14182:down|Ptk7,71461:down|Wnt5a,22418:down|Bmpr1a,12166:down|Aldh1a2,19378:up|Mgp,17313:up|Igf1,16000:up|Epas1,13819:up|Bmp4,12159:up|Tnc,21923:down|Vegfa,22339:down|Rspo2,239405:up|Errfi1,74155:down|Fgfr2,14183:up</td></tr>
<tr><td>respiratory system development</td><td>GO:0060541</td><td>1.66E-06</td><td>3.49E-05</td><td>Ptk7,71461:down|Aldh1a2,19378:up|Mgp,17313:up|Epas1,13819:up|Vegfa,22339:down|Tnf,21926:up|Fgfr1,14182:down|Bmpr1a,12166:down|Wnt5a,22418:down|Bmp4,12159:up|Igf1,16000:up|Tnc,21923:down|Rspo2,239405:up|Aldh1a3,56847:down|Fgfr2,14183:up|Errfi1,74155:down</td></tr>
<tr><td>axis elongation</td><td>GO:0003401</td><td>1.67E-06</td><td>3.49E-05</td><td>Sfrp2,20319:up|Tnc,21923:down|Sfrp1,20377:up|Ptk7,71461:down|Wnt5a,22418:down|Fgfr2,14183:up|Bmp4,12159:up</td></tr>
<tr><td>regulation of cell growth</td><td>GO:0001558</td><td>1.68E-06</td><td>3.50E-05</td><td>Igfbp4,16010:up|Plxnd1,67784:up|Inhba,16323:down|Sema7a,20361:down|Htra3,78558:down|Tnfrsf12a,27279:down|Cxcl12,20315:up|Vegfa,22339:down|Igfbp3,16009:up|Serpine2,20720:up|Pparg,19016:up|Egfr,13649:down|Sfrp1,20377:up|Wnt5a,22418:down|Wisp1,22402:down|Grem1,23892:up|Igf1,16000:up|Sfrp2,20319:up|Crabp2,12904:down|Tgfb2,21808:down|Enpp1,18605:down</td></tr>
<tr><td>regulation of bone remodeling</td><td>GO:0046850</td><td>1.76E-06</td><td>3.65E-05</td><td>Trf,22041:up|Egfr,13649:down|Sfrp1,20377:up|Tfrc,22042:up|Grem1,23892:up|Ceacam1,26365:up|Vegfa,22339:down|Tnfrsf11b,18383:up</td></tr>
<tr><td>gland development</td><td>GO:0048732</td><td>1.77E-06</td><td>3.67E-05</td><td>Hp,15439:up|Plxnd1,67784:up|Vdr,22337:down|Vegfa,22339:down|Mmp2,17390:down|Serpine2,20720:up|Igf2,16002:up|Cdo1,12583:up|Tnf,21926:up|Egfr,13649:down|Fgfr1,14182:down|Hmox1,15368:up|Sfrp1,20377:up|Sulf1,240725:down|Wnt5a,22418:down|Tgfbr3,21814:up|Bmpr1a,12166:down|Bmp4,12159:up|Igf1,16000:up|Ceacam1,26365:up|Tnc,21923:down|Fgfr2,14183:up</td></tr>
<tr><td>stem cell development</td><td>GO:0048864</td><td>1.81E-06</td><td>3.73E-05</td><td>Fam101b,76566:down|Htr2b,15559:down|Aldh1a2,19378:up|Ednrb,13618:up|Sema7a,20361:down|Loxl2,94352:down|Loxl3,16950:down|Fgfr1,14182:down|Sfrp1,20377:up|Has2,15117:down|Wnt5a,22418:down|Tgfbr3,21814:up|Bmpr1a,12166:down|Grem1,23892:up|Sfrp2,20319:up|Ret,19713:up|Tgfb2,21808:down|Fgfr2,14183:up|Kitl,17311:up</td></tr>
<tr><td>localization</td><td>GO:0051179</td><td>1.82E-06</td><td>3.75E-05</td><td>Adm,11535:up|Flrt2,399558:down|Plxnd1,67784:up|Sfrp4,20379:up|C3,12266:up|Ankrd1,107765:down|Cx3cr1,13051:down|Igfbp3,16009:up|Loxl2,94352:down|Trf,22041:up|Cxcl14,57266:down|Cd24a,12484:down|Ceacam10,26366:up|Wnt5a,22418:down|Mark1,226778:up|Stc1,20855:up|Il1rn,16181:up|Maob,109731:up|Igf1,16000:up|Adipoq,11450:up|Bmp4,12159:up|Crabp2,12904:down|Mcoln2,68279:up|Cxcl9,17329:up|Tgfb2,21808:down|F3,14066:up|Fgfr2,14183:up|Clec4e,56619:up|Cthrc1,68588:down|Htr2b,15559:down|Mfsd12,73822:up|Prl2c2,18811:up|Efemp1,216616:up|Ccl6,20305:up|Dpysl3,22240:down|Saa3,20210:up|Vdr,22337:down|Fabp4,11770:up|Fam132b,227358:down|Cxcl12,20315:up|Htr2a,15558:down|Mmp2,17390:down|Slc16a4,229699:down|Slc40a1,53945:up|Gas6,14456:up|Egfr,13649:down|Sec23a,20334:down|Sfrp1,20377:up|C1qtnf3,81799:down|Tgfbr3,21814:up|Tfrc,22042:up|Sncg,20618:up|Fxyd2,11936:up|Serinc2,230779:down|Ceacam1,26365:up|Ccl8,20307:up|Sfrp2,20319:up|Npnt,114249:up|Syt13,80976:up|S100a1,20193:up|Ice1,218333:down|Mrc1,17533:up|Ccl2,20296:up|Ccl7,20306:up|Pdgfc,54635:down|Ednrb,13618:up|Figf,14205:up|Mmp13,17386:up|Sema7a,20361:down|Slc29a1,63959:up|Tnfrsf12a,27279:down|Sord,20322:up|Gpm6b,14758:up|Clec4n,56620:up|Steap1,70358:down|Postn,50706:down|Cdo1,12583:up|Akr1c18,105349:up|Hvcn1,74096:up|Ptx3,19288:up|Fabp5,16592:up|Ncam1,17967:down|Bmpr1a,12166:down|Ccl12,20293:up|Ccl4,20303:up|Chst11,58250:down|Ank,11732:down|Ret,19713:up|Plpp3,67916:up|Creb3l1,26427:down|Mrc2,17534:down|Il1rl1,17082:down|Kitl,17311:up|Lama2,16773:down|Ccl9,20308:up|Ptk7,71461:down|Inhba,16323:down|Plat,18791:down|Ccl3,20302:up|Srpx2,68792:down|Vegfa,22339:down|Serpine2,20720:up|Adamts12,239337:down|Lrrc15,74488:down|Anpep,16790:up|Kdelr3,105785:down|Pparg,19016:up|Tnf,21926:up|Glp1r,14652:down|Fxyd1,56188:up|Fgfr1,14182:down|Has2,15117:down|Lpl,16956:up|Hmox1,15368:up|Sulf1,240725:down|Cd4,12504:up|Ptgs2,19225:down|Kcnj15,16516:down|Grem1,23892:up|Col3a1,12825:down|Col5a1,12831:down|Mcoln3,171166:up|Emb,13723:up|Enpp1,18605:down</td></tr>
<tr><td>respiratory tube development</td><td>GO:0030323</td><td>1.90E-06</td><td>3.90E-05</td><td>Tnf,21926:up|Fgfr1,14182:down|Ptk7,71461:down|Wnt5a,22418:down|Bmpr1a,12166:down|Aldh1a2,19378:up|Mgp,17313:up|Igf1,16000:up|Epas1,13819:up|Bmp4,12159:up|Tnc,21923:down|Vegfa,22339:down|Rspo2,239405:up|Errfi1,74155:down|Fgfr2,14183:up</td></tr>
<tr><td>bone remodeling</td><td>GO:0046849</td><td>1.92E-06</td><td>3.91E-05</td><td>Cthrc1,68588:down|Trf,22041:up|Egfr,13649:down|Sfrp1,20377:up|Tfrc,22042:up|Grem1,23892:up|Ceacam1,26365:up|Vegfa,22339:down|Tnfrsf11b,18383:up|Enpp1,18605:down</td></tr>
<tr><td>heart morphogenesis</td><td>GO:0003007</td><td>2.00E-06</td><td>4.07E-05</td><td>Htr2b,15559:down|Flrt2,399558:down|Plxnd1,67784:up|Aldh1a2,19378:up|Ankrd1,107765:down|Vegfa,22339:down|Col11a1,12814:down|Has2,15117:down|Tgfbr3,21814:up|Bmpr1a,12166:down|Wnt5a,22418:down|Bmp4,12159:up|Sfrp2,20319:up|Col5a1,12831:down|Tgfb2,21808:down|Fgfr2,14183:up</td></tr>
<tr><td>single organism cell adhesion</td><td>GO:0098602</td><td>2.02E-06</td><td>4.09E-05</td><td>Fstl3,83554:down|Ly9,17085:up|Ccl2,20296:up|Ctla2a,13024:down|Srpx2,68792:down|Cxcl12,20315:up|Postn,50706:down|Vegfa,22339:down|Serpine2,20720:up|Igf2,16002:up|Tnf,21926:up|Egfr,13649:down|Cd24a,12484:down|Ncam1,17967:down|Has2,15117:down|Sfrp1,20377:up|Wnt5a,22418:down|Cd4,12504:up|Il1rn,16181:up|Tfrc,22042:up|Adipoq,11450:up|Bmp4,12159:up|Igf1,16000:up|Ceacam1,26365:up|Ret,19713:up|Tgfb2,21808:down|Cdsn,386463:up|Npnt,114249:up|Plpp3,67916:up|Clec4e,56619:up|Tnfsf9,21950:up</td></tr>
<tr><td>response to drug</td><td>GO:0042493</td><td>2.03E-06</td><td>4.11E-05</td><td>Htr2b,15559:down|Gas6,14456:up|Ncam1,17967:down|Sfrp1,20377:up|Inhba,16323:down|Tfrc,22042:up|Ccl4,20303:up|Adipoq,11450:up|Ankrd1,107765:down|Gstm1,14862:up|Sfrp2,20319:up|Tgfb2,21808:down|Htr2a,15558:down|Mmp2,17390:down|Igf2,16002:up</td></tr>
<tr><td>regulation of gastrulation</td><td>GO:0010470</td><td>2.07E-06</td><td>4.15E-05</td><td>Col5a2,12832:down|Sfrp2,20319:up|Fgfr1,14182:down|Col5a1,12831:down|Bmpr1a,12166:down|Il1rn,16181:up|Adipoq,11450:up</td></tr>
<tr><td>dopaminergic neuron differentiation</td><td>GO:0071542</td><td>2.07E-06</td><td>4.15E-05</td><td>Sfrp2,20319:up|Sfrp1,20377:up|Cxcl12,20315:up|Wnt5a,22418:down|Vegfa,22339:down|Rspo2,239405:up|Figf,14205:up</td></tr>
<tr><td>epithelial to mesenchymal transition</td><td>GO:0001837</td><td>2.07E-06</td><td>4.15E-05</td><td>Fam101b,76566:down|Loxl2,94352:down|Loxl3,16950:down|Has2,15117:down|Sfrp1,20377:up|Wnt5a,22418:down|Tgfbr3,21814:up|Grem1,23892:up|Sfrp2,20319:up|Tgfb2,21808:down|Fgfr2,14183:up</td></tr>
</table>

<table>
<tr><td>signal transduction</td><td>GO:0007165</td><td>2.14E-06</td><td>4.26E-05</td><td>Fstl3,83554:down|Igfbp4,16010:up|Adm,11535:up|Flrt2,399558:down|Hpgd,15446:up|Plxnd1,67784:up|Sfrp4,20379:up|C3,12266:up|Ankrd1,107765:down|Cx3cr1,13051:down|Igfbp3,16009:up|Cd109,235505:down|Plk2,20620:down|Trf,22041:up|Cd24a,12484:down|Wnt5a,22418:down|Mark1,226778:up|Il1rn,16181:up|Adipoq,11450:up|Bmp4,12159:up|Igf1,16000:up|Crabp2,12904:down|Cxcl9,17329:up|Tgfb2,21808:down|Hpse,15442:up|Ptgir,19222:up|F3,14066:up|Fgfr2,14183:up|Clec4e,56619:up|Cthrc1,68588:down|Htr2b,15559:down|Efemp1,216616:up|Ccl6,20305:up|Aspn,66695:down|Mt2,17750:up|Saa3,20210:up|Vdr,22337:down|Cxcr6,80901:down|Cxcl12,20315:up|Htr2a,15558:down|Smoc2,64074:down|Gas6,14456:up|Egfr,13649:down|Sfrp1,20377:up|C1qtnf3,81799:down|Tgfbr3,21814:up|Ceacam1,26365:up|Ccl8,20307:up|Sfrp2,20319:up|Rspo2,239405:up|Npnt,114249:up|Tnfrsf11b,18383:up|Mt1,17748:up|Aldh1a2,19378:up|Ccl2,20296:up|Ccl7,20306:up|Pdgfc,54635:down|Ednrb,13618:up|Figf,14205:up|Sema7a,20361:down|Slc29a1,63959:up|Htra3,78558:down|Tnfrsf12a,27279:down|Clec4n,56620:up|Postn,50706:down|Dok2,13449:up|Akr1c18,105349:up|Ncam1,17967:down|Ccl12,20293:up|Bmpr1a,12166:down|Wisp1,22402:down|Ccl4,20303:up|Chst11,58250:down|Ret,19713:up|Sdf2l1,64136:up|Aldh1a3,56847:down|Plpp3,67916:up|Creb3l1,26427:down|Il1rl1,17082:down|Kitl,17311:up|Hp,15439:up|Ccl9,20308:up|Ptk7,71461:down|Inhba,16323:down|Rasl11a,68895:up|Plat,18791:down|Ccl3,20302:up|Crlf1,12931:down|Vegfa,22339:down|Serpine2,20720:up|Adamts12,239337:down|Lrrc15,74488:down|Nxn,18230:down|Igf2,16002:up|Pparg,19016:up|Tnf,21926:up|Glp1r,14652:down|Col1a2,12843:down|Lmcd1,30937:up|Tnfrsf23,79201:down|Fgfr1,14182:down|Hmox1,15368:up|Sulf1,240725:down|Cd4,12504:up|Ptgs2,19225:down|Grem1,23892:up|Col3a1,12825:down|Errfi1,74155:down|Enpp1,18605:down</td></tr>
<tr><td>positive regulation of defense response</td><td>GO:0031349</td><td>2.22E-06</td><td>4.41E-05</td><td>Ccl9,20308:up|Ccl6,20305:up|Ccl7,20306:up|Ccl2,20296:up|C3,12266:up|Ccl3,20302:up|Fabp4,11770:up|Mmp2,17390:down|Tnf,21926:up|Cd24a,12484:down|Egfr,13649:down|Wnt5a,22418:down|Ccl12,20293:up|Ptgs2,19225:down|Ccl4,20303:up|Ccl8,20307:up|Il1rl1,17082:down</td></tr>
<tr><td>cell communication</td><td>GO:0007154</td><td>2.23E-06</td><td>4.43E-05</td><td>Fstl3,83554:down|Igfbp4,16010:up|Adm,11535:up|Flrt2,399558:down|Hpgd,15446:up|Plxnd1,67784:up|Sfrp4,20379:up|C3,12266:up|Enpp5,83965:up|Ankrd1,107765:down|Cx3cr1,13051:down|Igfbp3,16009:up|Cd109,235505:down|Plk2,20620:down|Trf,22041:up|Cxcl14,57266:down|Cd24a,12484:down|Wnt5a,22418:down|Mark1,226778:up|Il1rn,16181:up|Igf1,16000:up|Adipoq,11450:up|Bmp4,12159:up|Crabp2,12904:down|Cxcl9,17329:up|Tgfb2,21808:down|Hpse,15442:up|Ptgir,19222:up|F3,14066:up|Fgfr2,14183:up|Clec4e,56619:up|Cthrc1,68588:down|Htr2b,15559:down|Efemp1,216616:up|Ccl6,20305:up|Gjb5,14622:down|Aspn,66695:down|Mt2,17750:up|Saa3,20210:up|Vdr,22337:down|Cxcr6,80901:down|Cxcl12,20315:up|Htr2a,15558:down|Smoc2,64074:down|Gas6,14456:up|Egfr,13649:down|Sfrp1,20377:up|C1qtnf3,81799:down|Tgfbr3,21814:up|Tfrc,22042:up|Sncg,20618:up|Ceacam1,26365:up|Ccl8,20307:up|Sfrp2,20319:up|Rspo2,239405:up|Npnt,114249:up|Tnfrsf11b,18383:up|Syt13,80976:up|Mt1,17748:up|Aldh1a2,19378:up|Ccl2,20296:up|Ccl7,20306:up|Pdgfc,54635:down|Ednrb,13618:up|Figf,14205:up|Sema7a,20361:down|Slc29a1,63959:up|Htra3,78558:down|Tnfrsf12a,27279:down|Gpm6b,14758:up|Clec4n,56620:up|Postn,50706:down|Dok2,13449:up|Akr1c18,105349:up|Ncam1,17967:down|Bmpr1a,12166:down|Ccl12,20293:up|Wisp1,22402:down|Ccl4,20303:up|Chst11,58250:down|Tnc,21923:down|Ret,19713:up|Sdf2l1,64136:up|Aldh1a3,56847:down|Plpp3,67916:up|Creb3l1,26427:down|Il1rl1,17082:down|Kitl,17311:up|Lama2,16773:down|Hp,15439:up|Ccl9,20308:up|Ptk7,71461:down|Inhba,16323:down|Rasl11a,68895:up|Plat,18791:down|Ccl3,20302:up|Crlf1,12931:down|Plod2,26432:down|Vegfa,22339:down|Serpine2,20720:up|Adamts12,239337:down|Lrrc15,74488:down|Nxn,18230:down|Igf2,16002:up|Pparg,19016:up|Tnf,21926:up|Glp1r,14652:down|Col1a2,12843:down|Lmcd1,30937:up|Tnfrsf23,79201:down|Fgfr1,14182:down|Hmox1,15368:up|Sulf1,240725:down|Cd4,12504:up|Ptgs2,19225:down|Grem1,23892:up|Col3a1,12825:down|Errfi1,74155:down|Enpp1,18605:down|Tnfsf9,21950:up</td></tr>
<tr><td>cellular metal ion homeostasis</td><td>GO:0006875</td><td>2.41E-06</td><td>4.76E-05</td><td>Htr2b,15559:down|Kng1,16644:up|Adm,11535:up|Ccl2,20296:up|Ednrb,13618:up|Ccl3,20302:up|Mt2,17750:up|Vdr,22337:down|Fam132b,227358:down|Htr2a,15558:down|Slc40a1,53945:up|Glp1r,14652:down|Trf,22041:up|Fxyd1,56188:up|Cd24a,12484:down|Hmox1,15368:up|Cd4,12504:up|Stc1,20855:up|Tfrc,22042:up|Bmp4,12159:up|Tgfb2,21808:down|Ptgir,19222:up|Mt1,17748:up</td></tr>
<tr><td>single organism signaling</td><td>GO:0044700</td><td>2.41E-06</td><td>4.76E-05</td><td>Fstl3,83554:down|Igfbp4,16010:up|Adm,11535:up|Flrt2,399558:down|Hpgd,15446:up|Plxnd1,67784:up|Sfrp4,20379:up|C3,12266:up|Ankrd1,107765:down|Cx3cr1,13051:down|Igfbp3,16009:up|Cd109,235505:down|Plk2,20620:down|Trf,22041:up|Cxcl14,57266:down|Cd24a,12484:down|Wnt5a,22418:down|Mark1,226778:up|Il1rn,16181:up|Igf1,16000:up|Adipoq,11450:up|Bmp4,12159:up|Crabp2,12904:down|Cxcl9,17329:up|Tgfb2,21808:down|Hpse,15442:up|Ptgir,19222:up|F3,14066:up|Fgfr2,14183:up|Clec4e,56619:up|Cthrc1,68588:down|Htr2b,15559:down|Efemp1,216616:up|Ccl6,20305:up|Aspn,66695:down|Mt2,17750:up|Saa3,20210:up|Vdr,22337:down|Cxcr6,80901:down|Cxcl12,20315:up|Htr2a,15558:down|Smoc2,64074:down|Gas6,14456:up|Egfr,13649:down|Sfrp1,20377:up|C1qtnf3,81799:down|Tgfbr3,21814:up|Sncg,20618:up|Ceacam1,26365:up|Ccl8,20307:up|Sfrp2,20319:up|Rspo2,239405:up|Npnt,114249:up|Tnfrsf11b,18383:up|Syt13,80976:up|Mt1,17748:up|Aldh1a2,19378:up|Ccl2,20296:up|Ccl7,20306:up|Pdgfc,54635:down|Ednrb,13618:up|Figf,14205:up|Sema7a,20361:down|Slc29a1,63959:up|Htra3,78558:down|Tnfrsf12a,27279:down|Gpm6b,14758:up|Clec4n,56620:up|Postn,50706:down|Dok2,13449:up|Akr1c18,105349:up|Ncam1,17967:down|Bmpr1a,12166:down|Ccl12,20293:up|Wisp1,22402:down|Ccl4,20303:up|Chst11,58250:down|Tnc,21923:down|Ret,19713:up|Sdf2l1,64136:up|Aldh1a3,56847:down|Plpp3,67916:up|Creb3l1,26427:down|Il1rl1,17082:down|Kitl,17311:up|Lama2,16773:down|Hp,15439:up|Ccl9,20308:up|Ptk7,71461:down|Inhba,16323:down|Rasl11a,68895:up|Plat,18791:down|Ccl3,20302:up|Crlf1,12931:down|Vegfa,22339:down|Serpine2,20720:up|Adamts12,239337:down|Lrrc15,74488:down|Nxn,18230:down|Igf2,16002:up|Pparg,19016:up|Tnf,21926:up|Glp1r,14652:down|Col1a2,12843:down|Lmcd1,30937:up|Tnfrsf23,79201:down|Fgfr1,14182:down|Hmox1,15368:up|Sulf1,240725:down|Cd4,12504:up|Ptgs2,19225:down|Grem1,23892:up|Col3a1,12825:down|Errfi1,74155:down|Enpp1,18605:down|Tnfsf9,21950:up</td></tr>
<tr><td>prostate gland development</td><td>GO:0030850</td><td>2.44E-06</td><td>4.79E-05</td><td>Sfrp1,20377:up|Sulf1,240725:down|Wnt5a,22418:down|Igf1,16000:up|Bmp4,12159:up|Tnc,21923:down|Mmp2,17390:down|Fgfr2,14183:up</td></tr>
<tr><td>cardiac muscle tissue development</td><td>GO:0048738</td><td>2.47E-06</td><td>4.83E-05</td><td>Fgfr1,14182:down|Col11a1,12814:down|Ncam1,17967:down|Wnt5a,22418:down|Bmpr1a,12166:down|Tgfbr3,21814:up|Aldh1a2,19378:up|Wisp1,22402:down|Grem1,23892:up|Igf1,16000:up|Bmp4,12159:up|Ankrd1,107765:down|Vegfa,22339:down|Tgfb2,21808:down|Fgfr2,14183:up</td></tr>
<tr><td>regulation of transferase activity</td><td>GO:0051338</td><td>2.49E-06</td><td>4.86E-05</td><td>Htr2b,15559:down|Flrt2,399558:down|Pdgfc,54635:down|Aspn,66695:down|Fabp4,11770:up|Vegfa,22339:down|Htr2a,15558:down|Lrrc15,74488:down|Igf2,16002:up|Gas6,14456:up|Pparg,19016:up|Tnf,21926:up|Trf,22041:up|Fgfr1,14182:down|Egfr,13649:down|Cd24a,12484:down|Sfrp1,20377:up|Wnt5a,22418:down|Cd4,12504:up|Il1rn,16181:up|Grem1,23892:up|Serinc2,230779:down|Adipoq,11450:up|Bmp4,12159:up|Igf1,16000:up|Ceacam1,26365:up|Sfrp2,20319:up|Tgfb2,21808:down|Prkar2b,19088:up|Errfi1,74155:down|Kitl,17311:up</td></tr>
</table>

neurogenesis	GO:0022008	2.55E-06	4.97E-05	Adm,11535:up|Flrt2,399558:down|Plxnd1,67784:up|Ccl2,20296:up|Aldh1a2,19378:up|Figf,14205:up|Ednrb,13618:up|Sema7a,20361:down|Ankrd1,107765:down|Tnfrsf12a,27279:down|Postn,50706:down|Cx3cr1,13051:down|Plk2,20620:down|Trf,22041:up|Cd24a,12484:down|Ncam1,17967:down|Wnt5a,22418:down|Bmpr1a,12166:down|Ccl12,20293:up|Mark1,226778:up|Bmp4,12159:up|Igf1,16000:up|Crabp2,12904:down|Tnc,21923:down|Ret,19713:up|Tgfb2,21808:down|Plpp3,67916:up|Fgfr2,14183:up|Cthrc1,68588:down|Prl2c2,18811:up|Lama2,16773:down|Efemp1,216616:up|Ptk7,71461:down|Inhba,16323:down|Dpysl3,22240:down|Ccl3,20302:up|Cxcl12,20315:up|Vegfa,22339:down|Mmp2,17390:down|Serpine2,20720:up|Pparg,19016:up|Gas6,14456:up|Egfr,13649:down|Fgfr1,14182:down|Sfrp1,20377:up|Col3a1,12825:down|Sfrp2,20319:up|Mcoln3,171166:up|Rspo2,239405:up
negative regulation of cartilage development	GO:0061037	2.57E-06	4.98E-05	Fam101b,76566:down|Efemp1,216616:up|Tgfb2,21808:down|Adamts12,239337:down|Grem1,23892:up|Bmp4,12159:up
regulation of biological process	GO:0050789	2.57E-06	4.98E-05	Fstl3,83554:down|Igfbp4,16010:up|Adm,11535:up|Flrt2,399558:down|Tmem176b,65963:up|Plxnd1,67784:up|Tfec,21426:up|Sfrp4,20379:up|Lyl1,17095:up|Igfbp3,16009:up|Plk2,20620:down|Cd24a,12484:down|Il1rn,16181:up|Maob,109731:up|Bmp4,12159:up|Oasl1,231655:up|Hpse,15442:up|Medag,70717:down|Ptgir,19222:up|Fgfr2,14183:up|Thbs2,21826:down|Htr2b,15559:down|Dram1,71712:up|Ccl6,20305:up|Slpi,20568:up|Aspn,66695:down|Mt2,17750:up|Cxcr6,80901:down|Fam132b,227358:down|Nabp1,109019:down|Phf11a,219131:up|Mmp2,17390:down|Oas3,246727:up|Slc40a1,53945:up|Egfr,13649:down|C1rb,667277:up|Tfrc,22042:up|Sncg,20618:up|Ccl8,20307:up|Serpina3g,20715:up|Rspo2,239405:up|Fam20a,208659:down|Mt1,17748:up|Col5a2,12832:down|Aldh1a2,19378:up|Ccl2,20296:up|Ednrb,13618:up|Mmp13,17386:up|Htra3,78558:down|Tnfrsf12a,27279:down|Uchl5,56207:up|Clec4n,56620:up|Dok2,13449:up|Hvcn1,74096:up|Ptx3,19288:up|Ncam1,17967:down|Ccl4,20303:up|Chst11,58250:down|Vegfb,22340:up|Aldh1a3,56847:down|Plpp3,67916:up|Lrrc17,74511:down|Lama2,16773:down|Ccl9,20308:up|Ptk7,71461:down|Inhba,16323:down|Bnc2,242509:down|Tfpi2,21789:down|Crlf1,12931:down|C1s1,50908:up|Plod2,26432:down|Vegfa,22339:down|Serpine2,20720:up|Nxn,18230:down|Igf2,16002:up|Kdelr3,105785:down|Tmem176a,66058:up|Tnf,21926:up|Col1a2,12843:down|Fxyd1,56188:up|Fgfr1,14182:down|Lpl,16956:up|Sulf1,240725:down|Susd2,71733:up|Cd4,12504:up|Col3a1,12825:down|Tbx15,21384:down|Serping1,12258:up|Enpp1,18605:down|Clec2d,93694:up|Hpgd,15446:up|C3,12266:up|Ankrd1,107765:down|Cx3cr1,13051:down|Cd109,235505:down|Loxl2,94352:down|Loxl3,16950:down|Trf,22041:up|Cxcl14,57266:down|Wnt5a,22418:down|Stc1,20855:up|Mark1,226778:up|Col8a1,12837:down|Adipoq,11450:up|Igf1,16000:up|Crabp2,12904:down|Cxcl9,17329:up|Tgfb2,21808:down|F3,14066:up|Clec4e,56619:up|Cthrc1,68588:down|Kng1,16644:up|Prl2c2,18811:up|Efemp1,216616:up|Dpysl3,22240:down|Ninj1,18081:up|Saa3,20210:up|Vdr,22337:down|Pdia5,72599:down|Fabp4,11770:up|Cxcl12,20315:up|Htr2a,15558:down|Smoc2,64074:down|Gas6,14456:up|Sfrp1,20377:up|C1qtnf3,81799:down|Tgfbr3,21814:up|Fxyd2,11936:up|Serinc2,230779:down|Ceacam1,26365:up|Sfrp2,20319:up|Tnfrsf11b,18383:up|Prkar2b,19088:up|Npnt,114249:up|S100a1,20193:up|Ice1,218333:down|Ly9,17085:up|Ccl7,20306:up|Pdgfc,54635:down|Mgp,17313:up|Figf,14205:up|Cst7,13011:up|Sema7a,20361:down|Epas1,13819:up|Slc29a1,63959:up|Gpm6b,14758:up|Ddah1,69219:down|Sdpr,20324:down|Postn,50706:down|Akr1c18,105349:up|Ccl12,20293:up|Bmpr1a,12166:down|Wisp1,22402:down|Ank,11732:down|Tnc,21923:down|Ret,19713:up|Prg4,96875:down|Sdf2l1,64136:up|Pdxk,216134:up|C1s2,317677:up|Ifit1,15957:up|Creb3l1,26427:down|Il1rl1,17082:down|Kitl,17311:up|Fam101b,76566:down|Hp,15439:up|Aoah,27052:up|Rasl11a,68895:up|Plat,18791:down|Ccl3,20302:up|Ctla2a,13024:down|Srpx2,68792:down|Adamts12,239337:down|Lrrc15,74488:down|Anpep,16790:up|Pparg,19016:up|Lmcd1,30937:up|Glp1r,14652:down|Tnfrsf23,79201:down|Hmox1,15368:up|Has2,15117:down|Ptgs2,19225:down|Grem1,23892:up|Kcnj15,16516:down|Phf11d,219132:up|Col5a1,12831:down|Errfi1,74155:down|Tnfsf9,21950:up
programmed cell death	GO:0012501	2.66E-06	5.12E-05	Adm,11535:up|Sfrp4,20379:up|Aldh1a2,19378:up|Ednrb,13618:up|Ankrd1,107765:down|Tnfrsf12a,27279:down|Cx3cr1,13051:down|Igfbp3,16009:up|Plk2,20620:down|Akr1c18,105349:up|Cd24a,12484:down|Wnt5a,22418:down|Ccl12,20293:up|Il1rn,16181:up|Adipoq,11450:up|Bmp4,12159:up|Igf1,16000:up|Chst11,58250:down|Tgfb2,21808:down|Pdxk,216134:up|Sdf2l1,64136:up|Aldh1a3,56847:down|Creb3l1,26427:down|Fgfr2,14183:up|F3,14066:up|Kitl,17311:up|Htr2b,15559:down|Dram1,71712:up|Inhba,16323:down|Ccl3,20302:up|Vdr,22337:down|Crlf1,12931:down|Cxcl12,20315:up|Vegfa,22339:down|Mmp2,17390:down|Slc40a1,53945:up|Pparg,19016:up|Gas6,14456:up|Tnf,21926:up|Glp1r,14652:down|Tnfrsf23,79201:down|Fgfr1,14182:down|Egfr,13649:down|Hmox1,15368:up|Sfrp1,20377:up|Sulf1,240725:down|Tgfbr3,21814:up|Ptgs2,19225:down|Grem1,23892:up|Ceacam1,26365:up|Sfrp2,20319:up|Serpina3g,20715:up|Tnfrsf11b,18383:up|Mt1,17748:up
multicellular organismal metabolic process	GO:0044236	2.68E-06	5.13E-05	Pparg,19016:up|Tgfbr3,21814:up|Ccl2,20296:up|Mmp13,17386:up|Bmp4,12159:up|Serpinh1,12406:down|Col5a1,12831:down|Mmp2,17390:down|Errfi1,74155:down|Mrc2,17534:down
regulation of leukocyte chemotaxis	GO:0002688	2.68E-06	5.13E-05	Gas6,14456:up|Cxcl14,57266:down|Wnt5a,22418:down|Ccl2,20296:up|Ccl12,20293:up|Figf,14205:up|Grem1,23892:up|Ccl4,20303:up|Cxcl9,17329:up|Cxcl12,20315:up
signaling	GO:0023052	2.69E-06	5.13E-05	Fstl3,83554:down|Igfbp4,16010:up|Adm,11535:up|Flrt2,399558:down|Hpgd,15446:up|Plxnd1,67784:up|Sfrp4,20379:up|C3,12266:up|Ankrd1,107765:down|Cx3cr1,13051:down|Igfbp3,16009:up|Cd109,235505:down|Plk2,20620:down|Trf,22041:up|Cxcl14,57266:down|Cd24a,12484:down|Wnt5a,22418:down|Mark1,226778:up|Il1rn,16181:up|Igf1,16000:up|Adipoq,11450:up|Bmp4,12159:up|Crabp2,12904:down|Cxcl9,17329:up|Tgfb2,21808:down|Hpse,15442:up|Ptgir,19222:up|F3,14066:up|Fgfr2,14183:up|Clec4e,56619:up|Cthrc1,68588:down|Htr2b,15559:down|Efemp1,216616:up|Ccl6,20305:up|Aspn,66695:down|Mt2,17750:up|Saa3,20210:up|Vdr,22337:down|Cxcr6,80901:down|Cxcl12,20315:up|Htr2a,15558:down|Smoc2,64074:down|Gas6,14456:up|Egfr,13649:down|Sfrp1,20377:up|C1qtnf3,81799:down|Tgfbr3,21814:up|Sncg,20618:up|Ceacam1,26365:up|Ccl8,20307:up|Sfrp2,20319:up|Rspo2,239405:up|Npnt,114249:up|Tnfrsf11b,18383:up|Syt13,80976:up|Mt1,17748:up|Aldh1a2,19378:up|Ccl2,20296:up|Ccl7,20306:up|Pdgfc,54635:down|Ednrb,13618:up|Figf,14205:up|Sema7a,20361:down|Slc29a1,63959:up|Htra3,78558:down|Tnfrsf12a,27279:down|Gpm6b,14758:up|Clec4n,56620:up|Postn,50706:down|Dok2,13449:up|Akr1c18,105349:up|Ncam1,17967:down|Bmpr1a,12166:down|Ccl12,20293:up|Wisp1,22402:down|Ccl4,20303:up|Chst11,58250:down|Tnc,21923:down|Ret,19713:up|Sdf2l1,64136:up|Aldh1a3,56847:down|Plpp3,67916:up|Creb3l1,26427:down|Il1rl1,17082:down|Kitl,17311:up|Lama2,16773:down|Hp,15439:up|Ccl9,20308:up|Ptk7,71461:down|Inhba,16323:down|Rasl11a,68895:up|Plat,18791:down|Ccl3,20302:up|Crlf1,12931:down|Vegfa,22339:down|Serpine2,20720:up|Adamts12,239337:down|Lrrc15,74488:down|Nxn,18230:down|Igf2,16002:up|Pparg,19016:up|Tnf,21926:up|Glp1r,14652:down|Col1a2,12843:down|Lmcd1,30937:up|Tnfrsf23,79201:down|Fgfr1,14182:down|Hmox1,15368:up|Sulf1,240725:down|Cd4,12504:up|Ptgs2,19225:down|Grem1,23892:up|Col3a1,12825:down|Errfi1,74155:down|Enpp1,18605:down|Tnfsf9,21950:up
regulation of epidermis development	GO:0045682	2.71E-06	5.16E-05	Tnf,21926:up|Inhba,16323:down|Sfrp4,20379:up|Bmp4,12159:up|Vdr,22337:down|Hpse,15442:up|Tgfb2,21808:down|Cd109,235505:down|Errfi1,74155:down

regulation of cell-cell adhesion	GO:0022407	2.82E-06	5.33E-05	Fstl3,83554:down|Ccl2,20296:up|Ctla2a,13024:down|Cxcl12,20315:up|Vegfa,22339:down|Serpine2,20720:up|Igf2,16002:up|Tnf,21926:up|Cd24a,12484:down|Has2,15117:down|Wnt5a,22418:down|Cd4,12504:up|Il1rn,16181:up|Tfrc,22042:up|Adipoq,11450:up|Bmp4,12159:up|Igf1,16000:up|Ceacam1,26365:up|Plpp3,67916:up|Tnfsf9,21950:up
positive regulation of protein kinase activity	GO:0045860	2.82E-06	5.33E-05	Htr2b,15559:down|Pdgfc,54635:down|Vegfa,22339:down|Htr2a,15558:down|Gas6,14456:up|Tnf,21926:up|Trf,22041:up|Cd24a,12484:down|Egfr,13649:down|Fgfr1,14182:down|Wnt5a,22418:down|Cd4,12504:up|Il1rn,16181:up|Grem1,23892:up|Adipoq,11450:up|Bmp4,12159:up|Igf1,16000:up|Ceacam1,26365:up|Tgfb2,21808:down|Kitl,17311:up
positive regulation of molecular function	GO:0044093	2.93E-06	5.53E-05	Plxnd1,67784:up|Ccl7,20306:up|Ccl2,20296:up|Pdgfc,54635:down|Plk2,20620:down|Trf,22041:up|Cd24a,12484:down|Wnt5a,22418:down|Ccl12,20293:up|Il1rn,16181:up|Ccl4,20303:up|Adipoq,11450:up|Bmp4,12159:up|Igf1,16000:up|Tgfb2,21808:down|Plpp3,67916:up|Fgfr2,14183:up|F3,14066:up|Kitl,17311:up|Cthrc1,68588:down|Htr2b,15559:down|Ccl9,20308:up|Ccl6,20305:up|Ccl3,20302:up|Vdr,22337:down|Vegfa,22339:down|Htr2a,15558:down|Igf2,16002:up|Pparg,19016:up|Gas6,14456:up|Tnf,21926:up|Egfr,13649:down|Fgfr1,14182:down|Cd4,12504:up|Tgfbr3,21814:up|Grem1,23892:up|Serinc2,230779:down|Ccl8,20307:up|Ceacam1,26365:up|Sfrp2,20319:up|Npnt,114249:up|S100a1,20193:up
regulation of kinase activity	GO:0043549	3.03E-06	5.70E-05	Htr2b,15559:down|Flrt2,399558:down|Pdgfc,54635:down|Aspn,66695:down|Fabp4,11770:up|Vegfa,22339:down|Htr2a,15558:down|Lrrc15,74488:down|Gas6,14456:up|Tnf,21926:up|Trf,22041:up|Fgfr1,14182:down|Egfr,13649:down|Cd24a,12484:down|Sfrp1,20377:up|Wnt5a,22418:down|Cd4,12504:up|Il1rn,16181:up|Grem1,23892:up|Adipoq,11450:up|Bmp4,12159:up|Igf1,16000:up|Ceacam1,26365:up|Sfrp2,20319:up|Tgfb2,21808:down|Prkar2b,19088:up|Errfi1,74155:down|Kitl,17311:up
positive regulation of immune system process	GO:0002684	3.07E-06	5.75E-05	Inhba,16323:down|Ccl2,20296:up|C3,12266:up|Figf,14205:up|Ccl3,20302:up|Sema7a,20361:down|C1s1,50908:up|Cxcl12,20315:up|Mmp2,17390:down|Igf2,16002:up|Gas6,14456:up|Tnf,21926:up|Cxcl14,57266:down|Cd24a,12484:down|Hmox1,15368:up|Wnt5a,22418:down|Cd4,12504:up|Ccl12,20293:up|C1rb,667277:up|Tfrc,22042:up|Ccl4,20303:up|Igf1,16000:up|Ceacam1,26365:up|Serping1,12258:up|Cxcl9,17329:up|Tgfb2,21808:down|C1s2,317677:up|Il1rl1,17082:down|Tnfsf9,21950:up|Kitl,17311:up
negative regulation of cell migration	GO:0030336	3.18E-06	5.92E-05	Sfrp1,20377:up|Sulf1,240725:down|Bmpr1a,12166:down|Tgfbr3,21814:up|Ccl12,20293:up|Dpysl3,22240:down|Stc1,20855:up|Il1rn,16181:up|Grem1,23892:up|Col3a1,12825:down|Adipoq,11450:up|Sfrp2,20319:up|Cxcl12,20315:up|Cx3cr1,13051:down|Igfbp3,16009:up
osteoclast differentiation	GO:0030316	3.34E-06	6.17E-05	Fstl3,83554:down|Tnf,21926:up|Clec2d,93694:up|Trf,22041:up|Lrrc17,74511:down|Sfrp1,20377:up|Tfrc,22042:up|Ccl3,20302:up|Ceacam1,26365:up|Cd109,235505:down
single organismal cell-cell adhesion	GO:0016337	3.43E-06	6.31E-05	Fstl3,83554:down|Ly9,17085:up|Ccl2,20296:up|Ctla2a,13024:down|Srpx2,68792:down|Cxcl12,20315:up|Vegfa,22339:down|Serpine2,20720:up|Igf2,16002:up|Tnf,21926:up|Egfr,13649:down|Cd24a,12484:down|Ncam1,17967:down|Has2,15117:down|Wnt5a,22418:down|Cd4,12504:up|Il1rn,16181:up|Tfrc,22042:up|Adipoq,11450:up|Bmp4,12159:up|Igf1,16000:up|Ceacam1,26365:up|Ret,19713:up|Tgfb2,21808:down|Cdsn,386463:up|Npnt,114249:up|Plpp3,67916:up|Tnfsf9,21950:up|Clec4e,56619:up
phosphate-containing compound metabolic process	GO:0006796	3.52E-06	6.42E-05	Igfbp4,16010:up|Adm,11535:up|Flrt2,399558:down|Sfrp4,20379:up|C3,12266:up|Enpp5,83965:up|Igfbp3,16009:up|Cd109,235505:down|Plk2,20620:down|Trf,22041:up|Cd24a,12484:down|Wnt5a,22418:down|Mark1,226778:up|Il1rn,16181:up|Adipoq,11450:up|Bmp4,12159:up|Igf1,16000:up|Tgfb2,21808:down|Ptgir,19222:up|Fgfr2,14183:up|Htr2b,15559:down|Efemp1,216616:up|Ccl6,20305:up|Aspn,66695:down|Saa3,20210:up|Fabp4,11770:up|Htr2a,15558:down|Gas6,14456:up|Egfr,13649:down|Alpl,11647:up|Sfrp1,20377:up|C1qtnf3,81799:down|Tgfbr3,21814:up|Serinc2,230779:down|Ceacam1,26365:up|Ccl8,20307:up|Sfrp2,20319:up|Fam20a,208659:down|Npnt,114249:up|Tnfrsf11b,18383:up|Prkar2b,19088:up|Ccl2,20296:up|Ccl7,20306:up|Ednrb,13618:up|Pdgfc,54635:down|Ampd3,11717:up|Sema7a,20361:down|Enpp3,209558:down|Dok2,13449:up|Fabp5,16592:up|Ccl12,20293:up|Bmpr1a,12166:down|Ccl4,20303:up|Ret,19713:up|Pdxk,216134:up|Plpp3,67916:up|Kitl,17311:up|Ccl9,20308:up|Inhba,16323:down|Ccl3,20302:up|Srpx2,68792:down|Crlf1,12931:down|Gfpt2,14584:down|Vegfa,22339:down|Cbr2,12409:up|Lrrc15,74488:down|Igf2,16002:up|Tnf,21926:up|Fgfr1,14182:down|Cd4,12504:up|Grem1,23892:up|Uck2,80914:down|Errfi1,74155:down|Enpp1,18605:down
digestive system development	GO:0055123	3.52E-06	6.42E-05	Egfr,13649:down|Sfrp1,20377:up|Wnt5a,22418:down|Aldh1a2,19378:up|Pdgfc,54635:down|Ednrb,13618:up|Col3a1,12825:down|Igf1,16000:up|Bmp4,12159:up|Sfrp2,20319:up|Fgfr2,14183:up|Igf2,16002:up
negative regulation of transmembrane receptor protein serine/threonine kinase signaling pathway	GO:0090101	3.71E-06	6.74E-05	Fstl3,83554:down|Sfrp1,20377:up|Wnt5a,22418:down|Tgfbr3,21814:up|Aspn,66695:down|Grem1,23892:up|Chst11,58250:down|Htra3,78558:down|Sfrp2,20319:up|Cd109,235505:down
regulation of muscle cell differentiation	GO:0051147	3.86E-06	6.99E-05	Tnf,21926:up|Prl2c2,18811:up|Cxcl14,57266:down|Ccl9,20308:up|Wisp1,22402:down|Grem1,23892:up|Igf1,16000:up|Bmp4,12159:up|Ccl8,20307:up|Cxcl9,17329:up|Cxcl12,20315:up|Igfbp3,16009:up|Fgfr2,14183:up
regulation of myoblast differentiation	GO:0045661	3.89E-06	7.02E-05	Tnf,21926:up|Prl2c2,18811:up|Cxcl14,57266:down|Ccl9,20308:up|Bmp4,12159:up|Ccl8,20307:up|Cxcl9,17329:up|Igfbp3,16009:up
negative regulation of locomotion	GO:0040013	4.03E-06	7.25E-05	Dpysl3,22240:down|Cxcl12,20315:up|Cx3cr1,13051:down|Igfbp3,16009:up|Ptx3,19288:up|Sfrp1,20377:up|Sulf1,240725:down|Ccl12,20293:up|Tgfbr3,21814:up|Bmpr1a,12166:down|Wnt5a,22418:down|Il1rn,16181:up|Stc1,20855:up|Grem1,23892:up|Adipoq,11450:up|Col3a1,12825:down|Sfrp2,20319:up
cell-cell signaling	GO:0007267	4.11E-06	7.36E-05	Adm,11535:up|Sfrp4,20379:up|Ccl2,20296:up|Slc29a1,63959:up|Gpm6b,14758:up|Cx3cr1,13051:down|Plk2,20620:down|Cxcl14,57266:down|Cd24a,12484:down|Ncam1,17967:down|Wnt5a,22418:down|Wisp1,22402:down|Mark1,226778:up|Il1rn,16181:up|Adipoq,11450:up|Bmp4,12159:up|Tnc,21923:down|Plpp3,67916:up|Fgfr2,14183:up|Cthrc1,68588:down|Htr2b,15559:down|Lama2,16773:down|Ptk7,71461:down|Inhba,16323:down|Plat,18791:down|Htr2a,15558:down|Serpine2,20720:up|Nxn,18230:down|Tnf,21926:up|Pparg,19016:up|Glp1r,14652:down|Egfr,13649:down|Fgfr1,14182:down|Sfrp1,20377:up|Sulf1,240725:down|C1qtnf3,81799:down|Ptgs2,19225:down|Grem1,23892:up|Sncg,20618:up|Sfrp2,20319:up|Rspo2,239405:up|Syt13,80976:up|Tnfsf9,21950:up
myeloid cell differentiation	GO:0030099	4.11E-06	7.36E-05	Fstl3,83554:down|Clec2d,93694:up|Lrrc17,74511:down|Inhba,16323:down|Ccl3,20302:up|Epas1,13819:up|Vegfa,22339:down|Cd109,235505:down|Pparg,19016:up|Tnf,21926:up|Trf,22041:up|Sfrp1,20377:up|Tgfbr3,21814:up|Tfrc,22042:up|Adipoq,11450:up|Bmp4,12159:up|Ceacam1,26365:up|Kitl,17311:up|Tnfsf9,21950:up
positive regulation of epithelial cell proliferation	GO:0050679	4.15E-06	7.41E-05	Htr2b,15559:down|Egfr,13649:down|Has2,15117:down|Sfrp1,20377:up|Wnt5a,22418:down|Bmpr1a,12166:down|Ccl2,20296:up|Igf1,16000:up|Bmp4,12159:up|Cxcl12,20315:up|Vegfa,22339:down|F3,14066:up|Fgfr2,14183:up

regulation of epithelial cell migration	GO:0010632	4.46E-06	7.92E-05	Prl2c2,18811:up|Fgfr1,14182:down|Has2,15117:down|Wnt5a,22418:down|Tgfbr3,21814:up|Ptgs2,19225:down|Stc1,20855:up|Bmp4,12159:up|Ceacam1,26365:up|Srpx2,68792:down|Vegfa,22339:down|Tgfb2,21808:down|Plpp3,67916:up
muscle cell differentiation	GO:0042692	4.60E-06	8.15E-05	Prl2c2,18811:up|Adm,11535:up|Ccl9,20308:up|Ednrb,13618:up|Epas1,13819:up|Ankrd1,107765:down|Cxcl12,20315:up|Vegfa,22339:down|Igfbp3,16009:up|Igf2,16002:up|Tnf,21926:up|Cxcl14,57266:down|Wisp1,22402:down|Grem1,23892:up|Bmp4,12159:up|Igf1,16000:up|Ccl8,20307:up|Cxcl9,17329:up|Npnt,114249:up|Fgfr2,14183:up
response to transforming growth factor beta	GO:0071559	4.79E-06	8.43E-05	Col1a2,12843:down|Akr1c18,105349:up|Hpgd,15446:up|Tgfbr3,21814:up|Ccl2,20296:up|Aspn,66695:down|Col3a1,12825:down|Ankrd1,107765:down|Htra3,78558:down|Chst11,58250:down|Tgfb2,21808:down|Npnt,114249:up|Cd109,235505:down
cellular response to transforming growth factor beta stimulus	GO:0071560	4.79E-06	8.43E-05	Col1a2,12843:down|Akr1c18,105349:up|Hpgd,15446:up|Tgfbr3,21814:up|Ccl2,20296:up|Aspn,66695:down|Col3a1,12825:down|Ankrd1,107765:down|Htra3,78558:down|Chst11,58250:down|Tgfb2,21808:down|Npnt,114249:up|Cd109,235505:down
establishment of planar polarity involved in neural tube closure	GO:0090177	4.82E-06	8.43E-05	Cthrc1,68588:down|Sfrp2,20319:up|Sfrp1,20377:up|Ptk7,71461:down|Wnt5a,22418:down
positive regulation of protein kinase B signaling	GO:0051897	5.05E-06	8.80E-05	Tnf,21926:up|Gas6,14456:up|Akr1c18,105349:up|Egfr,13649:down|C1qtnf3,81799:down|Igf1,16000:up|Hpse,15442:up|F3,14066:up|Igf2,16002:up
homeostatic process	GO:0042592	5.17E-06	8.98E-05	Adm,11535:up|Sfrp4,20379:up|Ccl2,20296:up|Ednrb,13618:up|Ampd3,11717:up|Epas1,13819:up|Steap1,70358:down|Trf,22041:up|Cd24a,12484:down|Stc1,20855:up|Il1rn,16181:up|Adipoq,11450:up|Bmp4,12159:up|Igf1,16000:up|Tgfb2,21808:down|Ptgir,19222:up|Kitl,17311:up|Htr2b,15559:down|Kng1,16644:up|Inhba,16323:down|Mt2,17750:up|Ccl3,20302:up|Vdr,22337:down|Pdia5,72599:down|Fabp4,11770:up|Fam132b,227358:down|Vegfa,22339:down|Htr2a,15558:down|Nxn,18230:down|Anpep,16790:up|Slc40a1,53945:up|Gas6,14456:up|Pparg,19016:up|Tnf,21926:up|Glp1r,14652:down|Fxyd1,56188:up|Egfr,13649:down|Lpl,16956:up|Hmox1,15368:up|C1qtnf3,81799:down|Cd4,12504:up|Tgfbr3,21814:up|Ptgs2,19225:down|Tfrc,22042:up|Ceacam1,26365:up|Fam20a,208659:down|Tnfrsf11b,18383:up|Enpp1,18605:down|Mt1,17748:up
negative regulation of cell motility	GO:2000146	5.18E-06	8.98E-05	Sfrp1,20377:up|Sulf1,240725:down|Bmpr1a,12166:down|Tgfbr3,21814:up|Ccl12,20293:up|Dpysl3,22240:down|Stc1,20855:up|Il1rn,16181:up|Grem1,23892:up|Col3a1,12825:down|Adipoq,11450:up|Sfrp2,20319:up|Cxcl12,20315:up|Cx3cr1,13051:down|Igfbp3,16009:up
positive regulation of endocytosis	GO:0045807	5.26E-06	9.10E-05	Tnf,21926:up|Pparg,19016:up|Gas6,14456:up|Trf,22041:up|Ptx3,19288:up|Wnt5a,22418:down|Sfrp4,20379:up|Ccl2,20296:up|C3,12266:up|Grem1,23892:up|Vegfa,22339:down
positive regulation of apoptotic process	GO:0043065	5.43E-06	9.32E-05	Adm,11535:up|Inhba,16323:down|Sfrp4,20379:up|Aldh1a2,19378:up|Ccl3,20302:up|Ankrd1,107765:down|Tnfrsf12a,27279:down|Vdr,22337:down|Mmp2,17390:down|Igfbp3,16009:up|Pparg,19016:up|Tnf,21926:up|Akr1c18,105349:up|Cd24a,12484:down|Hmox1,15368:up|Sfrp1,20377:up|Wnt5a,22418:down|Ptgs2,19225:down|Adipoq,11450:up|Bmp4,12159:up|Ceacam1,26365:up|Sfrp2,20319:up|Tgfb2,21808:down|Aldh1a3,56847:down|F3,14066:up
cell growth	GO:0016049	5.43E-06	9.32E-05	Igfbp4,16010:up|Plxnd1,67784:up|Inhba,16323:down|Sema7a,20361:down|Htra3,78558:down|Tnfrsf12a,27279:down|Cxcl12,20315:up|Postn,50706:down|Vegfa,22339:down|Igfbp3,16009:up|Serpine2,20720:up|Pparg,19016:up|Egfr,13649:down|Sfrp1,20377:up|Wnt5a,22418:down|Tgfbr3,21814:up|Wisp1,22402:down|Grem1,23892:up|Igf1,16000:up|Sfrp2,20319:up|Crabp2,12904:down|Tgfb2,21808:down|Enpp1,18605:down
response to acid chemical	GO:0001101	5.49E-06	9.39E-05	Col5a2,12832:down|Tnf,21926:up|Pparg,19016:up|Col1a2,12843:down|Akr1c18,105349:up|Egfr,13649:down|Ptk7,71461:down|Tgfbr3,21814:up|Aldh1a2,19378:up|Pdgfc,54635:down|Col3a1,12825:down|Ret,19713:up|Mmp2,17390:down|Ptgir,19222:up|Cdo1,12583:up
trabecula formation	GO:0060343	5.61E-06	9.52E-05	Sfrp1,20377:up|Vegfa,22339:down|Tgfbr3,21814:up|Mmp2,17390:down|Grem1,23892:up|Slc40a1,53945:up
chondrocyte development	GO:0002063	5.61E-06	9.52E-05	Fam101b,76566:down|Chst11,58250:down|Serpinh1,12406:down|Sfrp2,20319:up|Col11a1,12814:down|Sulf1,240725:down
regulation of leukocyte differentiation	GO:1902105	5.63E-06	9.52E-05	Fstl3,83554:down|Clec2d,93694:up|Lrrc17,74511:down|Tmem176b,65963:up|Ccl3,20302:up|Ctla2a,13024:down|Gas6,14456:up|Tnf,21926:up|Tmem176a,66058:up|Cd24a,12484:down|Sfrp1,20377:up|Adipoq,11450:up|Bmp4,12159:up|Ceacam1,26365:up|Tnfsf9,21950:up|Kitl,17311:up
negative regulation of cellular component movement	GO:0051271	5.63E-06	9.52E-05	Dpysl3,22240:down|Cxcl12,20315:up|Cx3cr1,13051:down|Igfbp3,16009:up|Sfrp1,20377:up|Ccl12,20293:up|Tgfbr3,21814:up|Bmpr1a,12166:down|Wnt5a,22418:down|Sulf1,240725:down|Il1rn,16181:up|Stc1,20855:up|Grem1,23892:up|Adipoq,11450:up|Col3a1,12825:down|Sfrp2,20319:up
secretion by cell	GO:0032940	5.95E-06	1.00E-04	Adm,11535:up|Mmp13,17386:up|Ankrd1,107765:down|Clec4n,56620:up|Postn,50706:down|Ncam1,17967:down|Wnt5a,22418:down|Il1rn,16181:up|Maob,109731:up|Adipoq,11450:up|Igf1,16000:up|Tgfb2,21808:down|Creb3l1,26427:down|Il1rl1,17082:down|Clec4e,56619:up|Htr2b,15559:down|Inhba,16323:down|Ccl3,20302:up|Cxcl12,20315:up|Htr2a,15558:down|Serpine2,20720:up|Tnf,21926:up|Pparg,19016:up|Gas6,14456:up|Glp1r,14652:down|Egfr,13649:down|Fgfr1,14182:down|Sfrp1,20377:up|Lpl,16956:up|Hmox1,15368:up|C1qtnf3,81799:down|Sncg,20618:up|Ceacam1,26365:up|Syt13,80976:up
negative regulation of epithelial cell proliferation	GO:0050680	6.26E-06	1.05E-04	Sfrp1,20377:up|Sulf1,240725:down|Wnt5a,22418:down|Tgfbr3,21814:up|Bmp4,12159:up|Ceacam1,26365:up|Vdr,22337:down|Sfrp2,20319:up|Tgfb2,21808:down|Cd109,235505:down|Fgfr2,14183:up
regulation of biomineral tissue development	GO:0070167	6.38E-06	1.07E-04	Fam101b,76566:down|Gas6,14456:up|Bmpr1a,12166:down|Mgp,17313:up|Aspn,66695:down|Grem1,23892:up|Bmp4,12159:up|Gpm6b,14758:up|Ank,11732:down
positive regulation of programmed cell death	GO:0043068	6.44E-06	1.07E-04	Adm,11535:up|Inhba,16323:down|Sfrp4,20379:up|Aldh1a2,19378:up|Ccl3,20302:up|Ankrd1,107765:down|Tnfrsf12a,27279:down|Vdr,22337:down|Mmp2,17390:down|Igfbp3,16009:up|Pparg,19016:up|Tnf,21926:up|Akr1c18,105349:up|Cd24a,12484:down|Hmox1,15368:up|Sfrp1,20377:up|Wnt5a,22418:down|Ptgs2,19225:down|Adipoq,11450:up|Bmp4,12159:up|Ceacam1,26365:up|Sfrp2,20319:up|Tgfb2,21808:down|Aldh1a3,56847:down|F3,14066:up

phosphorus metabolic process	GO:0006793	6.57E-06	1.09E-04	Igfbp4,16010:up|Adm,11535:up|Flrt2,399558:down|Sfrp4,20379:up|C3,12266:up|Enpp5,83965:up|Igfbp3,16009:up|Cd109,235505:down|Plk2,20620:down|Trf,22041:up|Cd24a,12484:down|Wnt5a,22418:down|Mark1,226778:up|Il1rn,16181:up|Adipoq,11450:up|Bmp4,12159:up|Igf1,16000:up|Tgfb2,21808:down|Ptgir,19222:up|Fgfr2,14183:up|Htr2b,15559:down|Efemp1,216616:up|Ccl6,20305:up|Aspn,66695:down|Saa3,20210:up|Fabp4,11770:up|Htr2a,15558:down|Gas6,14456:up|Egfr,13649:down|Alpl,11647:up|Sfrp1,20377:up|C1qtnf3,81799:down|Tgfbr3,21814:up|Serinc2,230779:down|Ceacam1,26365:up|Ccl8,20307:up|Sfrp2,20319:up|Fam20a,208659:down|Npnt,114249:up|Tnfrsf11b,18383:up|Prkar2b,19088:up|Ccl2,20296:up|Ccl7,20306:up|Ednrb,13618:up|Pdgfc,54635:down|Ampd3,11717:up|Sema7a,20361:down|Enpp3,209558:down|Dok2,13449:up|Fabp5,16592:up|Ccl12,20293:up|Bmpr1a,12166:down|Ccl4,20303:up|Ret,19713:up|Pdxk,216134:up|Plpp3,67916:up|Kitl,17311:up|Ccl9,20308:up|Inhba,16323:down|Ccl3,20302:up|Srpx2,68792:down|Crlf1,12931:down|Gfpt2,14584:down|Vegfa,22339:down|Cbr2,12409:up|Lrrc15,74488:down|Igf2,16002:up|Tnf,21926:up|Fgfr1,14182:down|Cd4,12504:up|Grem1,23892:up|Uck2,80914:down|Errfi1,74155:down|Enpp1,18605:down
positive regulation of cell death	GO:0010942	6.77E-06	1.12E-04	Adm,11535:up|Hp,15439:up|Inhba,16323:down|Sfrp4,20379:up|Aldh1a2,19378:up|Ccl3,20302:up|Ankrd1,107765:down|Tnfrsf12a,27279:down|Vdr,22337:down|Mmp2,17390:down|Igfbp3,16009:up|Pparg,19016:up|Tnf,21926:up|Akr1c18,105349:up|Cd24a,12484:down|Hmox1,15368:up|Sfrp1,20377:up|Wnt5a,22418:down|Ptgs2,19225:down|Adipoq,11450:up|Bmp4,12159:up|Ceacam1,26365:up|Sfrp2,20319:up|Tgfb2,21808:down|Aldh1a3,56847:down|F3,14066:up
morphogenesis of embryonic epithelium	GO:0016331	6.80E-06	1.12E-04	Cthrc1,68588:down|Adm,11535:up|Sfrp1,20377:up|Sulf1,240725:down|Ptk7,71461:down|Wnt5a,22418:down|Aldh1a2,19378:up|Grem1,23892:up|Bmp4,12159:up|Sfrp2,20319:up|Ret,19713:up|Aldh1a3,56847:down|Fgfr2,14183:up
positive regulation of epithelial cell migration	GO:0010634	6.81E-06	1.12E-04	Prl2c2,18811:up|Fgfr1,14182:down|Has2,15117:down|Wnt5a,22418:down|Ptgs2,19225:down|Bmp4,12159:up|Srpx2,68792:down|Tgfb2,21808:down|Vegfa,22339:down|Plpp3,67916:up
regulation of hydrolase activity	GO:0051336	6.84E-06	1.12E-04	Ccl7,20306:up|Ccl2,20296:up|Cst7,13011:up|Cd109,235505:down|Ptx3,19288:up|Wnt5a,22418:down|Ccl12,20293:up|Ccl4,20303:up|Igf1,16000:up|Tgfb2,21808:down|Fgfr2,14183:up|F3,14066:up|Kng1,16644:up|Htr2b,15559:down|Ccl9,20308:up|Ccl6,20305:up|Slpi,20568:up|Ccl3,20302:up|Tfpi2,21789:down|Htr2a,15558:down|Vegfa,22339:down|Serpine2,20720:up|Oas3,246727:up|Pparg,19016:up|Gas6,14456:up|Tnf,21926:up|Fgfr1,14182:down|Ccl8,20307:up|Sfrp2,20319:up|Serping1,12258:up|Serpina3g,20715:up|Npnt,114249:up
positive regulation of endothelial cell migration	GO:0010595	6.89E-06	1.13E-04	Prl2c2,18811:up|Fgfr1,14182:down|Wnt5a,22418:down|Ptgs2,19225:down|Bmp4,12159:up|Srpx2,68792:down|Vegfa,22339:down|Plpp3,67916:up
regulation of cartilage development	GO:0061035	6.89E-06	1.13E-04	Fam101b,76566:down|Loxl2,94352:down|Efemp1,216616:up|Wnt5a,22418:down|Grem1,23892:up|Bmp4,12159:up|Tgfb2,21808:down|Adamts12,239337:down
negative regulation of bone remodeling	GO:0046851	6.90E-06	1.13E-04	Ceacam1,26365:up|Sfrp1,20377:up|Vegfa,22339:down|Tnfrsf11b,18383:up|Grem1,23892:up
negative regulation of embryonic development	GO:0045992	7.10E-06	1.16E-04	Col5a2,12832:down|Sfrp2,20319:up|Col5a1,12831:down|Sulf1,240725:down|Wnt5a,22418:down|Bmp4,12159:up
positive regulation of stem cell proliferation	GO:2000648	7.14E-06	1.16E-04	Prl2c2,18811:up|Fgfr1,14182:down|Wnt5a,22418:down|Bmpr1a,12166:down|Bmp4,12159:up|Vegfa,22339:down|Cx3cr1,13051:down|Fgfr2,14183:up|Kitl,17311:up
regulation of fat cell differentiation	GO:0045598	7.42E-06	1.20E-04	Pparg,19016:up|Sfrp1,20377:up|Wnt5a,22418:down|Ptgs2,19225:down|Igf1,16000:up|Adipoq,11450:up|Sfrp2,20319:up|Vegfa,22339:down|Htr2a,15558:down|Medag,70717:down|Enpp1,18605:down
positive regulation of cell division	GO:0051781	7.42E-06	1.20E-04	Htr2b,15559:down|Tnf,21926:up|Wnt5a,22418:down|Pdgfc,54635:down|Figf,14205:up|Igf1,16000:up|Vegfa,22339:down|Vegfb,22340:up|Tgfb2,21808:down|Fgfr2,14183:up|Igf2,16002:up
endothelial cell proliferation	GO:0001935	7.50E-06	1.21E-04	Htr2b,15559:down|Loxl2,94352:down|Sulf1,240725:down|Wnt5a,22418:down|Ccl2,20296:up|Aldh1a2,19378:up|Bmp4,12159:up|Cxcl12,20315:up|Vegfa,22339:down|F3,14066:up
negative regulation of protein metabolic process	GO:0051248	8.02E-06	1.28E-04	Flrt2,399558:down|Sfrp4,20379:up|Ednrb,13618:up|Cst7,13011:up|Igfbp3,16009:up|Cd109,235505:down|Ptx3,19288:up|Adipoq,11450:up|Bmp4,12159:up|Igf1,16000:up|Prg4,96875:down|Plpp3,67916:up|Kng1,16644:up|Slpi,20568:up|Plat,18791:down|Aspn,66695:down|Tfpi2,21789:down|Ctla2a,13024:down|Fabp4,11770:up|Vegfa,22339:down|Serpine2,20720:up|Nxn,18230:down|Lrrc15,74488:down|Gas6,14456:up|Tnf,21926:up|Egfr,13649:down|Sfrp1,20377:up|Grem1,23892:up|Ceacam1,26365:up|Sfrp2,20319:up|Serping1,12258:up|Serpina3g,20715:up|Prkar2b,19088:up|Enpp1,18605:down|Errfi1,74155:down

biological regulation	GO:0065007	8.13E-06	1.30E-04	Fstl3,83554:down|Igfbp4,16010:up|Adm,11535:up|Flrt2,399558:down|Tmem176b,65963:up|Plxnd1,67784:up|Tfec,21426:up|Sfrp4,20379:up|Lyl1,17095:up|Igfbp3,16009:up|Plk2,20620:down|Cd24a,12484:down|Il1rn,16181:up|Maob,109731:up|Bmp4,12159:up|Oasl1,231655:up|Hpse,15442:up|Medag,70717:down|Ptgir,19222:up|Fgfr2,14183:up|Thbs2,21826:down|Htr2b,15559:down|Dram1,71712:up|Ccl6,20305:up|Anxa8,11752:down|Slpi,20568:up|Aspn,66695:down|Mt2,17750:up|Cxcr6,80901:down|Fam132b,227358:down|Nabp1,109019:down|Phf11a,219131:up|Mmp2,17390:down|Oas3,246727:up|Slc40a1,53945:up|Egfr,13649:down|C1rb,667277:up|Tfrc,22042:up|Sncg,20618:up|Ccl8,20307:up|Serpina3g,20715:up|Rspo2,239405:up|Fam20a,208659:down|Syt13,80976:up|Mt1,17748:up|Col5a2,12832:down|Aldh1a2,19378:up|Ccl2,20296:up|Ednrb,13618:up|Ampd3,11717:up|Mmp13,17386:up|Htra3,78558:down|Tnfrsf12a,27279:down|Uchl5,56207:up|Clec4n,56620:up|Steap1,70358:down|Cdo1,12583:up|Dok2,13449:up|Hvcn1,74096:up|Ptx3,19288:up|Ncam1,17967:down|Ccl4,20303:up|Chst11,58250:down|Vegfb,22340:up|Aldh1a3,56847:down|Plpp3,67916:up|Lrrc17,74511:down|Lama2,16773:down|Ccl9,20308:up|Ptk7,71461:down|Inhba,16323:down|Bnc2,242509:down|Tfpi2,21789:down|Crlf1,12931:down|C1s1,50908:up|Plod2,26432:down|Vegfa,22339:down|Serpine2,20720:up|Nxn,18230:down|Igf2,16002:up|Kdelr3,105785:down|Tmem176a,66058:up|Tnf,21926:up|Col1a2,12843:down|Fxyd1,56188:up|Fgfr1,14182:down|Lpl,16956:up|Sulf1,240725:down|Susd2,71733:up|Cd4,12504:up|Col3a1,12825:down|Tbx15,21384:down|Serping1,12258:up|Enpp1,18605:down|Clec2d,93694:up|Hpgd,15446:up|C3,12266:up|Ankrd1,107765:down|Cx3cr1,13051:down|Cd109,235505:down|Loxl2,94352:down|Loxl3,16950:down|Trf,22041:up|Cxcl14,57266:down|Wnt5a,22418:down|Stc1,20855:up|Mark1,226778:up|Col8a1,12837:down|Adipoq,11450:up|Igf1,16000:up|Crabp2,12904:down|Cxcl9,17329:up|Tgfb2,21808:down|F3,14066:up|Clec4e,56619:up|Cthrc1,68588:down|Kng1,16644:up|Prl2c2,18811:up|Efemp1,216616:up|Dpysl3,22240:down|Ninj1,18081:up|Saa3,20210:up|Vdr,22337:down|Pdia5,72599:down|Fabp4,11770:up|Cxcl12,20315:up|Htr2a,15558:down|Smoc2,64074:down|Gas6,14456:up|Sfrp1,20377:up|C1qtnf3,81799:down|Tgfbr3,21814:up|Fxyd2,11936:up|Serinc2,230779:down|Ceacam1,26365:up|Sfrp2,20319:up|Tnfrsf11b,18383:up|Prkar2b,19088:up|Npnt,114249:up|S100a1,20193:up|Ice1,218333:down|Ly9,17085:up|Ccl7,20306:up|Pdgfc,54635:down|Mgp,17313:up|Figf,14205:up|Cst7,13011:up|Sema7a,20361:down|Epas1,13819:up|Slc29a1,63959:up|Gpm6b,14758:up|Ddah1,69219:down|Sdpr,20324:down|Postn,50706:down|Akr1c18,105349:up|Ccl12,20293:up|Bmpr1a,12166:down|Wisp1,22402:down|Ank,11732:down|Tnc,21923:down|Ret,19713:up|Prg4,96875:down|Sdf2l1,64136:up|Pdxk,216134:up|C1s2,317677:up|Ifit1,15957:up|Creb3l1,26427:down|Il1rl1,17082:down|Kitl,17311:up|Fam101b,76566:down|Hp,15439:up|Aoah,27052:up|Rasl11a,68895:up|Plat,18791:down|Ccl3,20302:up|Ctla2a,13024:down|Srpx2,68792:down|Adamts12,239337:down|Lrrc15,74488:down|Anpep,16790:up|Pparg,19016:up|Lmcd1,30937:up|Glp1r,14652:down|Tnfrsf23,79201:down|Hmox1,15368:up|Has2,15117:down|Ptgs2,19225:down|Kcnj15,16516:down|Grem1,23892:up|Phf11d,219132:up|Col5a1,12831:down|Errfi1,74155:down|Tnfsf9,21950:up
cellular response to acid chemical	GO:0071229	8.29E-06	1.32E-04	Col5a2,12832:down|Tnf,21926:up|Col1a2,12843:down|Akr1c18,105349:up|Egfr,13649:down|Ptk7,71461:down|Aldh1a2,19378:up|Pdgfc,54635:down|Col3a1,12825:down|Ret,19713:up|Mmp2,17390:down|Ptgir,19222:up
cellular response to oxygen-containing compound	GO:1901701	8.55E-06	1.36E-04	Htr2b,15559:down|Col5a2,12832:down|Mrc1,17533:up|Ptk7,71461:down|Inhba,16323:down|Ccl7,20306:up|Ccl2,20296:up|Aldh1a2,19378:up|Pdgfc,54635:down|Ankrd1,107765:down|Vdr,22337:down|Postn,50706:down|Mmp2,17390:down|Igf2,16002:up|Gas6,14456:up|Pparg,19016:up|Tnf,21926:up|Col1a2,12843:down|Akr1c18,105349:up|Egfr,13649:down|Sfrp1,20377:up|Ccl12,20293:up|Col3a1,12825:down|Adipoq,11450:up|Bmp4,12159:up|Igf1,16000:up|Ret,19713:up|Ptgir,19222:up|Enpp1,18605:down|Mt1,17748:up
cellular ion homeostasis	GO:0006873	8.80E-06	1.39E-04	Htr2b,15559:down|Kng1,16644:up|Adm,11535:up|Ccl2,20296:up|Ednrb,13618:up|Ccl3,20302:up|Mt2,17750:up|Vdr,22337:down|Fam132b,227358:down|Htr2a,15558:down|Slc40a1,53945:up|Glp1r,14652:down|Trf,22041:up|Fxyd1,56188:up|Cd24a,12484:down|Hmox1,15368:up|Cd4,12504:up|Stc1,20855:up|Tfrc,22042:up|Bmp4,12159:up|Tgfb2,21808:down|Ptgir,19222:up|Enpp1,18605:down|Mt1,17748:up
positive regulation of transferase activity	GO:0051347	8.81E-06	1.39E-04	Htr2b,15559:down|Pdgfc,54635:down|Vegfa,22339:down|Htr2a,15558:down|Igf2,16002:up|Gas6,14456:up|Tnf,21926:up|Trf,22041:up|Cd24a,12484:down|Egfr,13649:down|Fgfr1,14182:down|Wnt5a,22418:down|Cd4,12504:up|Il1rn,16181:up|Grem1,23892:up|Serinc2,230779:down|Adipoq,11450:up|Bmp4,12159:up|Igf1,16000:up|Ceacam1,26365:up|Tgfb2,21808:down|Kitl,17311:up
regulation of chemotaxis	GO:0050920	8.90E-06	1.40E-04	Gas6,14456:up|Cxcl14,57266:down|Fgfr1,14182:down|Plxnd1,67784:up|Wnt5a,22418:down|Ccl2,20296:up|Ccl12,20293:up|Figf,14205:up|Grem1,23892:up|Ccl4,20303:up|Cxcl9,17329:up|Cxcl12,20315:up|Vegfa,22339:down
protein metabolic process	GO:0019538	9.28E-06	1.46E-04	Igfbp4,16010:up|Flrt2,399558:down|Sfrp4,20379:up|C3,12266:up|Serpinh1,12406:down|Igfbp3,16009:up|Cd109,235505:down|Ggt5,23887:up|Loxl2,94352:down|Plk2,20620:down|Trf,22041:up|Cd24a,12484:down|Adam12,11489:down|Mme,17380:up|Fkbp14,231997:down|Wnt5a,22418:down|Mark1,226778:up|Il1rn,16181:up|Adipoq,11450:up|Bmp4,12159:up|Igf1,16000:up|Tgfb2,21808:down|Hpse,15442:up|F3,14066:up|Fgfr2,14183:up|Adamts4,240913:down|Htr2b,15559:down|Kng1,16644:up|Efemp1,216616:up|Ccl6,20305:up|Slpi,20568:up|Aspn,66695:down|Saa3,20210:up|Pdia5,72599:down|Fabp4,11770:up|Htr2a,15558:down|Mmp2,17390:down|Pappa,18491:down|Gas6,14456:up|Egfr,13649:down|Col11a1,12814:down|Sfrp1,20377:up|C1qtnf3,81799:down|Tgfbr3,21814:up|C1rb,667277:up|Tll1,21892:down|Ceacam1,26365:up|Ccl8,20307:up|Sfrp2,20319:up|Pamr1,210622:up|Serpina3g,20715:up|Fam20a,208659:down|Npnt,114249:up|Tnfrsf11b,18383:up|Prkar2b,19088:up|Neurl3,214854:down|Ccl2,20296:up|Ccl7,20306:up|Pdzrn3,55983:down|Pdgfc,54635:down|Cst7,13011:up|Ednrb,13618:up|Mmp13,17386:up|Sema7a,20361:down|Htra3,78558:down|Uchl5,56207:up|Ddah1,69219:down|Fkbp10,14230:down|Dok2,13449:up|Man2a2,140481:up|Mamdc2,71738:down|Ptx3,19288:up|Bmpr1a,12166:down|Ccl12,20293:up|Ccl4,20303:up|Klk1b11,16613:up|Chst11,58250:down|Ret,19713:up|Prg4,96875:down|Vegfb,22340:up|Sdf2l1,64136:up|C1s2,317677:up|Plpp3,67916:up|Kitl,17311:up|P4ha1,18451:down|Hp,15439:up|Ccl9,20308:up|Inhba,16323:down|Plat,18791:down|Ccl3,20302:up|Tfpi2,21789:down|Ctla2a,13024:down|Crlf1,12931:down|C1s1,50908:up|Vegfa,22339:down|Serpine2,20720:up|Adamts12,239337:down|Lrrc15,74488:down|Nxn,18230:down|Igf2,16002:up|Anpep,16790:up|Pparg,19016:up|Tnf,21926:up|Fgfr1,14182:down|Sulf1,240725:down|Cd4,12504:up|Grem1,23892:up|Col3a1,12825:down|Serping1,12258:up|Errfi1,74155:down|Enpp1,18605:down
positive regulation of kinase activity	GO:0033674	9.43E-06	1.48E-04	Htr2b,15559:down|Pdgfc,54635:down|Vegfa,22339:down|Htr2a,15558:down|Gas6,14456:up|Tnf,21926:up|Trf,22041:up|Cd24a,12484:down|Egfr,13649:down|Fgfr1,14182:down|Wnt5a,22418:down|Cd4,12504:up|Il1rn,16181:up|Grem1,23892:up|Adipoq,11450:up|Bmp4,12159:up|Igf1,16000:up|Ceacam1,26365:up|Tgfb2,21808:down|Kitl,17311:up
establishment of planar polarity of embryonic epithelium	GO:0042249	9.64E-06	1.51E-04	Cthrc1,68588:down|Sfrp2,20319:up|Sfrp1,20377:up|Ptk7,71461:down|Wnt5a,22418:down

positive regulation of developmental growth	GO:0048639	1.01E-05	1.58E-04	Fgfr1,14182:down|Ncam1,17967:down|Bmpr1a,12166:down|C3,12266:up|Wisp1,22402:down|Sema7a,20361:down|Igf1,16000:up|Tnfrsf12a,27279:down|Crabp2,12904:down|Cxcl12,20315:up|Vegfa,22339:down|Fgfr2,14183:up|Igf2,16002:up
positive regulation of cytokine secretion	GO:0050715	1.09E-05	1.70E-04	Htr2b,15559:down|Tnf,21926:up|Lpl,16956:up|C1qtnf3,81799:down|Wnt5a,22418:down|Ccl3,20302:up|Clec4n,56620:up|Postn,50706:down|Clec4e,56619:up|Il1rl1,17082:down
regulation of BMP signaling pathway	GO:0030510	1.11E-05	1.72E-04	Fstl3,83554:down|Sfrp1,20377:up|Sulf1,240725:down|Wnt5a,22418:down|Tgfbr3,21814:up|Grem1,23892:up|Bmp4,12159:up|Htra3,78558:down|Sfrp2,20319:up
negative regulation of cellular protein metabolic process	GO:0032269	1.13E-05	1.75E-04	Flrt2,399558:down|Sfrp4,20379:up|Ednrb,13618:up|Cst7,13011:up|Igfbp3,16009:up|Cd109,235505:down|Adipoq,11450:up|Bmp4,12159:up|Igf1,16000:up|Plpp3,67916:up|Kng1,16644:up|Slpi,20568:up|Plat,18791:down|Aspn,66695:down|Tfpi2,21789:down|Ctla2a,13024:down|Fabp4,11770:up|Vegfa,22339:down|Serpine2,20720:up|Nxn,18230:down|Lrrc15,74488:down|Gas6,14456:up|Tnf,21926:up|Egfr,13649:down|Sfrp1,20377:up|Grem1,23892:up|Ceacam1,26365:up|Sfrp2,20319:up|Serping1,12258:up|Serpina3g,20715:up|Prkar2b,19088:up|Enpp1,18605:down|Errfi1,74155:down
cellular chemical homeostasis	GO:0055082	1.18E-05	1.81E-04	Htr2b,15559:down|Kng1,16644:up|Adm,11535:up|Ccl2,20296:up|Ednrb,13618:up|Ccl3,20302:up|Mt2,17750:up|Vdr,22337:down|Fam132b,227358:down|Htr2a,15558:down|Slc40a1,53945:up|Gas6,14456:up|Glp1r,14652:down|Fxyd1,56188:up|Trf,22041:up|Cd24a,12484:down|Hmox1,15368:up|C1qtnf3,81799:down|Cd4,12504:up|Stc1,20855:up|Tfrc,22042:up|Bmp4,12159:up|Igf1,16000:up|Tgfb2,21808:down|Ptgir,19222:up|Enpp1,18605:down|Mt1,17748:up
negative regulation of catalytic activity	GO:0043086	1.18E-05	1.81E-04	Kng1,16644:up|Flrt2,399558:down|Hp,15439:up|Slpi,20568:up|Cst7,13011:up|Aspn,66695:down|Tfpi2,21789:down|Fabp4,11770:up|Vegfa,22339:down|Serpine2,20720:up|Cd109,235505:down|Lrrc15,74488:down|Tnf,21926:up|Gas6,14456:up|Pparg,19016:up|Ptx3,19288:up|Sfrp1,20377:up|Adipoq,11450:up|Bmp4,12159:up|Igf1,16000:up|Ceacam1,26365:up|Sfrp2,20319:up|Serping1,12258:up|Serpina3g,20715:up|Tgfb2,21808:down|Prkar2b,19088:up|Errfi1,74155:down
positive regulation of protein serine/threonine kinase activity	GO:0071902	1.21E-05	1.85E-04	Htr2b,15559:down|Tnf,21926:up|Trf,22041:up|Fgfr1,14182:down|Egfr,13649:down|Cd24a,12484:down|Wnt5a,22418:down|Pdgfc,54635:down|Il1rn,16181:up|Igf1,16000:up|Adipoq,11450:up|Ceacam1,26365:up|Vegfa,22339:down|Htr2a,15558:down|Kitl,17311:up
skin development	GO:0043588	1.28E-05	1.95E-04	Col5a2,12832:down|Tnf,21926:up|Col1a2,12843:down|Egfr,13649:down|Inhba,16323:down|Ptgs2,19225:down|Col3a1,12825:down|Vdr,22337:down|Col5a1,12831:down|Tgfb2,21808:down|Hpse,15442:up|Cdsn,386463:up|Cd109,235505:down|Errfi1,74155:down|Fgfr2,14183:up
positive regulation of peptidyl-tyrosine phosphorylation	GO:0050731	1.28E-05	1.95E-04	Gas6,14456:up|Cd24a,12484:down|Cd4,12504:up|Grem1,23892:up|Igf1,16000:up|Adipoq,11450:up|Crlf1,12931:down|Vegfa,22339:down|Htr2a,15558:down|Plpp3,67916:up|Igf2,16002:up|Kitl,17311:up
stem cell differentiation	GO:0048863	1.30E-05	1.98E-04	Fam101b,76566:down|Htr2b,15559:down|Aldh1a2,19378:up|Ednrb,13618:up|Sema7a,20361:down|Loxl2,94352:down|Loxl3,16950:down|Fgfr1,14182:down|Sfrp1,20377:up|Has2,15117:down|Wnt5a,22418:down|Tgfbr3,21814:up|Bmpr1a,12166:down|Grem1,23892:up|Bmp4,12159:up|Sfrp2,20319:up|Ret,19713:up|Tgfb2,21808:down|Fgfr2,14183:up|Kitl,17311:up
acute inflammatory response	GO:0002526	1.31E-05	1.98E-04	Tnf,21926:up|Pparg,19016:up|Hp,15439:up|C3,12266:up|Ptgs2,19225:down|Il1rn,16181:up|Ednrb,13618:up|Saa3,20210:up|Serping1,12258:up|F3,14066:up
negative regulation of chondrocyte differentiation	GO:0032331	1.31E-05	1.99E-04	Fam101b,76566:down|Efemp1,216616:up|Adamts12,239337:down|Grem1,23892:up|Bmp4,12159:up
sprouting angiogenesis	GO:0002040	1.32E-05	1.99E-04	Prl2c2,18811:up|Loxl2,94352:down|Ptgs2,19225:down|Grem1,23892:up|Bmp4,12159:up|Ceacam1,26365:up|Srpx2,68792:down|Vegfa,22339:down
cell-type specific apoptotic process	GO:0097285	1.33E-05	2.00E-04	Sfrp4,20379:up|Ccl3,20302:up|Crlf1,12931:down|Cxcl12,20315:up|Igfbp3,16009:up|Gas6,14456:up|Tnf,21926:up|Glp1r,14652:down|Akr1c18,105349:up|Cd24a,12484:down|Hmox1,15368:up|Sfrp1,20377:up|Wnt5a,22418:down|Ccl12,20293:up|Adipoq,11450:up|Bmp4,12159:up|Igf1,16000:up|Ceacam1,26365:up|Sfrp2,20319:up|Tgfb2,21808:down|Fgfr2,14183:up|Kitl,17311:up|Mt1,17748:up
positive regulation of epidermis development	GO:0045684	1.36E-05	2.04E-04	Vdr,22337:down|Tnf,21926:up|Tgfb2,21808:down|Sfrp4,20379:up|Hpse,15442:up|Bmp4,12159:up
negative regulation of leukocyte differentiation	GO:1902106	1.36E-05	2.04E-04	Fstl3,83554:down|Tmem176a,66058:up|Clec2d,93694:up|Lrrc17,74511:down|Tmem176b,65963:up|Sfrp1,20377:up|Bmp4,12159:up|Adipoq,11450:up|Ceacam1,26365:up
regulation of hemopoiesis	GO:1903706	1.42E-05	2.11E-04	Fstl3,83554:down|Clec2d,93694:up|Lrrc17,74511:down|Tmem176b,65963:up|Inhba,16323:down|Ccl3,20302:up|Ctla2a,13024:down|Vegfa,22339:down|Gas6,14456:up|Tnf,21926:up|Tmem176a,66058:up|Cd24a,12484:down|Sfrp1,20377:up|Adipoq,11450:up|Bmp4,12159:up|Ceacam1,26365:up|Tnfsf9,21950:up|Kitl,17311:up
regulation of developmental growth	GO:0048638	1.42E-05	2.11E-04	Plxnd1,67784:up|C3,12266:up|Sema7a,20361:down|Tnfrsf12a,27279:down|Cxcl12,20315:up|Vegfa,22339:down|Igf2,16002:up|Fgfr1,14182:down|Sfrp1,20377:up|Ncam1,17967:down|Wnt5a,22418:down|Bmpr1a,12166:down|Wisp1,22402:down|Bmp4,12159:up|Igf1,16000:up|Sfrp2,20319:up|Crabp2,12904:down|Fgfr2,14183:up
regulation of endothelial cell migration	GO:0010594	1.43E-05	2.12E-04	Prl2c2,18811:up|Fgfr1,14182:down|Wnt5a,22418:down|Ptgs2,19225:down|Stc1,20855:up|Bmp4,12159:up|Ceacam1,26365:up|Srpx2,68792:down|Vegfa,22339:down|Plpp3,67916:up
regulation of extrinsic apoptotic signaling pathway	GO:2001236	1.47E-05	2.17E-04	Tnf,21926:up|Tnfrsf23,79201:down|Fgfr1,14182:down|Sfrp1,20377:up|Hmox1,15368:up|Inhba,16323:down|Igf1,16000:up|Bmp4,12159:up|Tnfrsf12a,27279:down|Sfrp2,20319:up|Tgfb2,21808:down|Cx3cr1,13051:down
coronary vasculature development	GO:0060976	1.49E-05	2.21E-04	Fgfr1,14182:down|Plxnd1,67784:up|Ptk7,71461:down|Tgfbr3,21814:up|Bmp4,12159:up|Vegfb,22340:up|Vegfa,22339:down|Fgfr2,14183:up
regulation of endothelial cell proliferation	GO:0001936	1.51E-05	2.23E-04	Htr2b,15559:down|Sulf1,240725:down|Wnt5a,22418:down|Ccl2,20296:up|Aldh1a2,19378:up|Bmp4,12159:up|Cxcl12,20315:up|Vegfa,22339:down|F3,14066:up
divalent inorganic cation homeostasis	GO:0072507	1.64E-05	2.41E-04	Htr2b,15559:down|Kng1,16644:up|Adm,11535:up|Ccl2,20296:up|Ednrb,13618:up|Ccl3,20302:up|Mt2,17750:up|Vdr,22337:down|Htr2a,15558:down|Fxyd1,56188:up|Glp1r,14652:down|Cd24a,12484:down|Egfr,13649:down|Cd4,12504:up|Stc1,20855:up|Bmp4,12159:up|Tgfb2,21808:down|Fam20a,208659:down|Ptgir,19222:up|Mt1,17748:up
positive regulation of cardiac muscle tissue growth	GO:0055023	1.66E-05	2.43E-04	Fgfr1,14182:down|Ncam1,17967:down|Bmpr1a,12166:down|Wisp1,22402:down|Fgfr2,14183:up|Igf1,16000:up

positive regulation of calcium-mediated signaling	GO:0050850	1.66E-05	2.43E-04	Lmcd1,30937:up|Cd24a,12484:down|Ncam1,17967:down|Cd4,12504:up|Ccl4,20303:up|Igf1,16000:up
response to inorganic substance	GO:0010035	1.69E-05	2.46E-04	Hp,15439:up|Mt2,17750:up|Sord,20322:up|Fabp4,11770:up|Mmp2,17390:down|Slc40a1,53945:up|Pparg,19016:up|Tnf,21926:up|Loxl2,94352:down|Trf,22041:up|Hvcn1,74096:up|Akr1c18,105349:up|Egfr,13649:down|Hmox1,15368:up|Ncam1,17967:down|Tfrc,22042:up|Gatm,67092:down|Mt1,17748:up
positive regulation of nucleocytoplasmic transport	GO:0046824	1.70E-05	2.47E-04	Tnf,21926:up|Gas6,14456:up|Egfr,13649:down|Bmpr1a,12166:down|Ptgs2,19225:down|Grem1,23892:up|Igf1,16000:up|Bmp4,12159:up|Ceacam1,26365:up|Sfrp2,20319:up
cell-substrate adhesion	GO:0031589	1.74E-05	2.53E-04	Ninj1,18081:up|Tnfrsf12a,27279:down|Gpm6b,14758:up|Sned1,208777:up|Postn,50706:down|Vegfa,22339:down|Adamts12,239337:down|Smoc2,64074:down|Gas6,14456:up|Sfrp1,20377:up|Has2,15117:down|Grem1,23892:up|Col8a1,12837:down|Col3a1,12825:down|Hpse,15442:up|Npnt,114249:up
nervous system development	GO:0007399	1.76E-05	2.53E-04	Adm,11535:up|Flrt2,399558:down|Plxnd1,67784:up|Ccl2,20296:up|Aldh1a2,19378:up|Figf,14205:up|Ednrb,13618:up|Sema7a,20361:down|Ankrd1,107765:down|Tnfrsf12a,27279:down|Uchl5,56207:up|Shroom4,208431:down|Postn,50706:down|Cx3cr1,13051:down|Plk2,20620:down|Trf,22041:up|Cd24a,12484:down|Ncam1,17967:down|Wnt5a,22418:down|Bmpr1a,12166:down|Ccl12,20293:up|Mark1,226778:up|Bmp4,12159:up|Igf1,16000:up|Crabp2,12904:down|Tnc,21923:down|Ret,19713:up|Tgfb2,21808:down|Aldh1a3,56847:down|Plpp3,67916:up|Fgfr2,14183:up|Thbs2,21826:down|Cthrc1,68588:down|Prl2c2,18811:up|Lama2,16773:down|Efemp1,216616:up|Ptk7,71461:down|Inhba,16323:down|Dpysl3,22240:down|Ccl3,20302:up|Srpx2,68792:down|Cxcl12,20315:up|Vegfa,22339:down|Mmp2,17390:down|Serpine2,20720:up|Pparg,19016:up|Gas6,14456:up|Tnf,21926:up|Fgfr1,14182:down|Egfr,13649:down|Sfrp1,20377:up|Sulf1,240725:down|Col3a1,12825:down|Sfrp2,20319:up|Pcdh19,279653:down|Mcoln3,171166:up|Rspo2,239405:up
regulation of ion transport	GO:0043269	1.76E-05	2.53E-04	Sfrp4,20379:up|Vdr,22337:down|Gpm6b,14758:up|Fam132b,227358:down|Cxcl12,20315:up|Htr2a,15558:down|Serpine2,20720:up|Tnf,21926:up|Glp1r,14652:down|Hvcn1,74096:up|Fxyd1,56188:up|Cd4,12504:up|Ccl12,20293:up|Ptgs2,19225:down|Il1rn,16181:up|Stc1,20855:up|Kcnj15,16516:down|Fxyd2,11936:up|Ccl4,20303:up|Sncg,20618:up|Maob,109731:up|Bmp4,12159:up|Igf1,16000:up|Tgfb2,21808:down|S100a1,20193:up
regulation of anion transport	GO:0044070	1.78E-05	2.55E-04	Tnf,21926:up|Fxyd1,56188:up|Sfrp4,20379:up|Stc1,20855:up|Il1rn,16181:up|Sncg,20618:up|Maob,109731:up|Gpm6b,14758:up|Fam132b,227358:down|Cxcl12,20315:up|Htr2a,15558:down
cellular cation homeostasis	GO:0030003	1.81E-05	2.58E-04	Htr2b,15559:down|Kng1,16644:up|Adm,11535:up|Ccl2,20296:up|Ednrb,13618:up|Ccl3,20302:up|Mt2,17750:up|Vdr,22337:down|Fam132b,227358:down|Htr2a,15558:down|Slc40a1,53945:up|Glp1r,14652:down|Trf,22041:up|Fxyd1,56188:up|Cd24a,12484:down|Hmox1,15368:up|Cd4,12504:up|Stc1,20855:up|Tfrc,22042:up|Bmp4,12159:up|Tgfb2,21808:down|Ptgir,19222:up|Mt1,17748:up
positive regulation of ossification	GO:0045778	1.84E-05	2.63E-04	Cthrc1,68588:down|Wnt5a,22418:down|Bmpr1a,12166:down|Igf1,16000:up|Bmp4,12159:up|Gpm6b,14758:up|Sfrp2,20319:up|Vegfa,22339:down|Npnt,114249:up
positive regulation of leukocyte migration	GO:0002687	1.85E-05	2.63E-04	Gas6,14456:up|Cxcl14,57266:down|Wnt5a,22418:down|Ccl2,20296:up|Ccl12,20293:up|Figf,14205:up|Ccl4,20303:up|Cxcl9,17329:up|Cxcl12,20315:up|Kitl,17311:up
neutrophil chemotaxis	GO:0030593	1.89E-05	2.69E-04	Ccl9,20308:up|Ccl6,20305:up|Ccl7,20306:up|Ccl2,20296:up|Ccl12,20293:up|Ccl4,20303:up|Ccl3,20302:up|Ccl8,20307:up
regulation of epithelial cell differentiation	GO:0030856	1.92E-05	2.72E-04	Tnf,21926:up|Sfrp4,20379:up|Bmp4,12159:up|Adipoq,11450:up|Ceacam1,26365:up|Vdr,22337:down|Vegfa,22339:down|Tgfb2,21808:down|Cd109,235505:down|Errfi1,74155:down|Kitl,17311:up
gastrulation	GO:0007369	1.92E-05	2.72E-04	Col5a2,12832:down|Fgfr1,14182:down|Sfrp1,20377:up|Wnt5a,22418:down|Inhba,16323:down|Bmpr1a,12166:down|Il1rn,16181:up|Bmp4,12159:up|Adipoq,11450:up|Sfrp2,20319:up|Col5a1,12831:down|Plpp3,67916:up
embryo development	GO:0009790	1.92E-05	2.72E-04	Col5a2,12832:down|Adm,11535:up|Aldh1a2,19378:up|Pdgfc,54635:down|Epas1,13819:up|Wnt5a,22418:down|Bmpr1a,12166:down|Il1rn,16181:up|Adipoq,11450:up|Bmp4,12159:up|Igf1,16000:up|Chst11,58250:down|Crabp2,12904:down|Ret,19713:up|Tgfb2,21808:down|Aldh1a3,56847:down|Plpp3,67916:up|Fgfr2,14183:up|Kitl,17311:up|Cthrc1,68588:down|Htr2b,15559:down|Lama2,16773:down|Ptk7,71461:down|Gjb5,14622:down|Inhba,16323:down|Vegfa,22339:down|Col11a1,12814:down|Egfr,13649:down|Fgfr1,14182:down|Sfrp1,20377:up|Sulf1,240725:down|Tgfbr3,21814:up|Grem1,23892:up|Sfrp2,20319:up|Col5a1,12831:down|Tbx15,21384:down|Rspo2,239405:up
immune effector process	GO:0002252	2.01E-05	2.82E-04	Clec2d,93694:up|Ly9,17085:up|Ccl2,20296:up|C3,12266:up|Ifit3,15959:up|Sema7a,20361:down|C1s1,50908:up|Cx3cr1,13051:down|Oas3,246727:up|Igf2,16002:up|Tnf,21926:up|Ptx3,19288:up|Lmcd1,30937:up|Cd24a,12484:down|Hmox1,15368:up|Wnt5a,22418:down|C1rb,667277:up|Tfrc,22042:up|Ceacam1,26365:up|Cxcl9,17329:up|Serping1,12258:up|Oasl1,231655:up|Tgfb2,21808:down|C1s2,317677:up|Ifit1,15957:up|Clec4e,56619:up
positive regulation of chemotaxis	GO:0050921	2.01E-05	2.82E-04	Gas6,14456:up|Cxcl14,57266:down|Fgfr1,14182:down|Wnt5a,22418:down|Ccl2,20296:up|Figf,14205:up|Ccl4,20303:up|Cxcl9,17329:up|Cxcl12,20315:up|Vegfa,22339:down
cellular response to iron ion	GO:0071281	2.01E-05	2.82E-04	Trf,22041:up|Hmox1,15368:up|Tfrc,22042:up|Slc40a1,53945:up
regulation of protein autophosphorylation	GO:0031952	2.02E-05	2.82E-04	Vegfa,22339:down|Pdgfc,54635:down|Errfi1,74155:down|Enpp1,18605:down|Grem1,23892:up|Adipoq,11450:up
cellular response to metal ion	GO:0071248	2.03E-05	2.84E-04	Pparg,19016:up|Akr1c18,105349:up|Hvcn1,74096:up|Trf,22041:up|Hmox1,15368:up|Tfrc,22042:up|Fabp4,11770:up|Mt1,17748:up|Slc40a1,53945:up
small molecule metabolic process	GO:0044281	2.06E-05	2.86E-04	Igfbp4,16010:up|Adm,11535:up|Hpgd,15446:up|Aldh1a2,19378:up|C3,12266:up|Ampd3,11717:up|Enpp5,83965:up|Marc1,66112:up|Sord,20322:up|Ddah1,69219:down|Npl,74091:up|Igfbp3,16009:up|Enpp3,209558:down|Man2a2,140481:up|Cdo1,12583:up|Mamdc2,71738:down|Ggt5,23887:up|Akr1c18,105349:up|Fabp5,16592:up|Mme,17380:up|Wnt5a,22418:down|Adipoq,11450:up|Hal,15109:up|Igf1,16000:up|Chst11,58250:down|Crabp2,12904:down|Gatm,67092:down|Pdxk,216134:up|Hpse,15442:up|Aldh1a3,56847:down|Ptgir,19222:up|P4ha1,18451:down|Htr2b,15559:down|Vdr,22337:down|Fabp4,11770:up|Fam132b,227358:down|Vegfa,22339:down|Htr2a,15558:down|Gfpt2,14584:down|Cbr2,12409:up|Igf2,16002:up|Pparg,19016:up|Tnf,21926:up|Fgfr1,14182:down|Lpl,16956:up|Sulf1,240725:down|C1qtnf3,81799:down|Ptgs2,19225:down|Serinc2,230779:down|Prkar2b,19088:up|Uck2,80914:down|Enpp1,18605:down
negative regulation of prostatic bud formation	GO:0060686	2.31E-05	3.17E-04	Sulf1,240725:down|Wnt5a,22418:down|Bmp4,12159:up
negative regulation of odontogenesis	GO:0042483	2.31E-05	3.17E-04	Rspo2,239405:up|Tnfrsf11b,18383:up|Aspn,66695:down

regulation of protein serine/threonine kinase activity	GO:0071900	2.36E-05	3.24E-04	Htr2b,15559:down|Pdgfc,54635:down|Vegfa,22339:down|Htr2a,15558:down|Tnf,21926:up|Trf,22041:up|Cd24a,12484:down|Egfr,13649:down|Fgfr1,14182:down|Sfrp1,20377:up|Wnt5a,22418:down|Il1rn,16181:up|Adipoq,11450:up|Bmp4,12159:up|Igf1,16000:up|Ceacam1,26365:up|Sfrp2,20319:up|Prkar2b,19088:up|Kitl,17311:up
regulation of stem cell proliferation	GO:0072091	2.37E-05	3.24E-04	Prl2c2,18811:up|Fgfr1,14182:down|Cd24a,12484:down|Wnt5a,22418:down|Bmpr1a,12166:down|Bmp4,12159:up|Vegfa,22339:down|Cx3cr1,13051:down|Fgfr2,14183:up|Kitl,17311:up
positive regulation of muscle organ development	GO:0048636	2.38E-05	3.24E-04	Fgfr1,14182:down|Ncam1,17967:down|Bmpr1a,12166:down|Wisp1,22402:down|Grem1,23892:up|Igf1,16000:up|Bmp4,12159:up|Fgfr2,14183:up
positive regulation of striated muscle tissue development	GO:0045844	2.38E-05	3.24E-04	Fgfr1,14182:down|Ncam1,17967:down|Bmpr1a,12166:down|Wisp1,22402:down|Grem1,23892:up|Igf1,16000:up|Bmp4,12159:up|Fgfr2,14183:up
positive regulation of cellular metabolic process	GO:0031325	2.39E-05	3.25E-04	Fstl3,83554:down|Igfbp4,16010:up|Adm,11535:up|Sfrp4,20379:up|C3,12266:up|Lyl1,17095:up|Igfbp3,16009:up|Plk2,20620:down|Trf,22041:up|Cd24a,12484:down|Wnt5a,22418:down|Il1rn,16181:up|Maob,109731:up|Adipoq,11450:up|Bmp4,12159:up|Igf1,16000:up|Tgfb2,21808:down|Ptgir,19222:up|F3,14066:up|Fgfr2,14183:up|Htr2b,15559:down|Prl2c2,18811:up|Ccl6,20305:up|Vdr,22337:down|Htr2a,15558:down|Slc40a1,53945:up|Gas6,14456:up|Egfr,13649:down|Sfrp1,20377:up|C1qtnf3,81799:down|Tgfbr3,21814:up|Tfrc,22042:up|Ceacam1,26365:up|Ccl8,20307:up|Sfrp2,20319:up|Fam20a,208659:down|Npnt,114249:up|Tnfrsf11b,18383:up|Ice1,218333:down|Ccl2,20296:up|Ccl7,20306:up|Pdgfc,54635:down|Ednrb,13618:up|Epas1,13819:up|Sema7a,20361:down|Ddah1,69219:down|Sdpr,20324:down|Ptx3,19288:up|Akr1c18,105349:up|Ccl12,20293:up|Bmpr1a,12166:down|Ccl4,20303:up|Ret,19713:up|Plpp3,67916:up|Creb3l1,26427:down|Kitl,17311:up|Ccl9,20308:up|Rasl11a,68895:up|Inhba,16323:down|Ccl3,20302:up|Crlf1,12931:down|Plod2,26432:down|Vegfa,22339:down|Igf2,16002:up|Pparg,19016:up|Tnf,21926:up|Lmcd1,30937:up|Glp1r,14652:down|Fgfr1,14182:down|Hmox1,15368:up|Cd4,12504:up|Ptgs2,19225:down|Grem1,23892:up
positive regulation of heart growth	GO:0060421	2.43E-05	3.29E-04	Fgfr1,14182:down|Ncam1,17967:down|Bmpr1a,12166:down|Wisp1,22402:down|Fgfr2,14183:up|Igf1,16000:up
bone morphogenesis	GO:0060349	2.46E-05	3.33E-04	Alpl,11647:up|Has2,15117:down|Stc1,20855:up|Bnc2,242509:down|Mmp13,17386:up|Bmp4,12159:up|Serpinh1,12406:down|Sfrp2,20319:up|Fgfr2,14183:up
cellular homeostasis	GO:0019725	2.48E-05	3.35E-04	Htr2b,15559:down|Kng1,16644:up|Adm,11535:up|Ccl2,20296:up|Ednrb,13618:up|Ccl3,20302:up|Mt2,17750:up|Vdr,22337:down|Pdia5,72599:down|Fam132b,227358:down|Htr2a,15558:down|Nxn,18230:down|Slc40a1,53945:up|Gas6,14456:up|Glp1r,14652:down|Fxyd1,56188:up|Trf,22041:up|Cd24a,12484:down|Hmox1,15368:up|C1qtnf3,81799:down|Cd4,12504:up|Stc1,20855:up|Tfrc,22042:up|Bmp4,12159:up|Igf1,16000:up|Tgfb2,21808:down|Ptgir,19222:up|Enpp1,18605:down|Mt1,17748:up
organ growth	GO:0035265	2.50E-05	3.36E-04	Fgfr1,14182:down|Ncam1,17967:down|Bmpr1a,12166:down|Tgfbr3,21814:up|Wisp1,22402:down|Stc1,20855:up|Bnc2,242509:down|Mmp13,17386:up|Igf1,16000:up|Tgfb2,21808:down|Rspo2,239405:up|Fgfr2,14183:up
generation of neurons	GO:0048699	2.52E-05	3.39E-04	Adm,11535:up|Flrt2,399558:down|Plxnd1,67784:up|Aldh1a2,19378:up|Figf,14205:up|Ednrb,13618:up|Sema7a,20361:down|Ankrd1,107765:down|Tnfrsf12a,27279:down|Postn,50706:down|Cx3cr1,13051:down|Plk2,20620:down|Trf,22041:up|Cd24a,12484:down|Ncam1,17967:down|Wnt5a,22418:down|Bmpr1a,12166:down|Mark1,226778:up|Bmp4,12159:up|Crabp2,12904:down|Tnc,21923:down|Ret,19713:up|Tgfb2,21808:down|Fgfr2,14183:up|Cthrc1,68588:down|Prl2c2,18811:up|Lama2,16773:down|Efemp1,216616:up|Ptk7,71461:down|Inhba,16323:down|Dpysl3,22240:down|Cxcl12,20315:up|Vegfa,22339:down|Serpine2,20720:up|Mmp2,17390:down|Pparg,19016:up|Gas6,14456:up|Egfr,13649:down|Fgfr1,14182:down|Sfrp1,20377:up|Col3a1,12825:down|Sfrp2,20319:up|Mcoln3,171166:up|Rspo2,239405:up
negative regulation of cytokine production	GO:0001818	2.56E-05	3.42E-04	Tnf,21926:up|Pparg,19016:up|Gas6,14456:up|Fgfr1,14182:down|Cd24a,12484:down|Hmox1,15368:up|C1qtnf3,81799:down|Adipoq,11450:up|Ceacam1,26365:up|Prg4,96875:down|Tgfb2,21808:down|Errfi1,74155:down|Il1rl1,17082:down
second-messenger-mediated signaling	GO:0019932	2.56E-05	3.42E-04	Htr2b,15559:down|Tnf,21926:up|Glp1r,14652:down|Lmcd1,30937:up|Cd24a,12484:down|Ncam1,17967:down|Cd4,12504:up|Ednrb,13618:up|Ccl4,20303:up|Mt2,17750:up|Igf1,16000:up|Ptgir,19222:up|Mt1,17748:up
transforming growth factor beta receptor signaling pathway	GO:0007179	2.57E-05	3.42E-04	Col1a2,12843:down|Hpgd,15446:up|Tgfbr3,21814:up|Ccl2,20296:up|Aspn,66695:down|Col3a1,12825:down|Htra3,78558:down|Chst11,58250:down|Tgfb2,21808:down|Npnt,114249:up|Cd109,235505:down
regulation of osteoblast differentiation	GO:0045667	2.57E-05	3.43E-04	Cthrc1,68588:down|Tnf,21926:up|Fgfr1,14182:down|Sfrp1,20377:up|Bmpr1a,12166:down|Igf1,16000:up|Bmp4,12159:up|Sfrp2,20319:up|Vegfa,22339:down|Npnt,114249:up
positive regulation of muscle tissue development	GO:1901863	2.66E-05	3.53E-04	Fgfr1,14182:down|Ncam1,17967:down|Bmpr1a,12166:down|Wisp1,22402:down|Grem1,23892:up|Igf1,16000:up|Bmp4,12159:up|Fgfr2,14183:up
positive regulation of leukocyte chemotaxis	GO:0002690	2.66E-05	3.53E-04	Gas6,14456:up|Cxcl14,57266:down|Wnt5a,22418:down|Ccl2,20296:up|Figf,14205:up|Ccl4,20303:up|Cxcl9,17329:up|Cxcl12,20315:up
behavior	GO:0007610	2.70E-05	3.57E-04	Htr2b,15559:down|Plxnd1,67784:up|Ccl2,20296:up|Figf,14205:up|Srpx2,68792:down|Cxcl12,20315:up|Vegfa,22339:down|Htr2a,15558:down|Cx3cr1,13051:down|Serpine2,20720:up|Igf2,16002:up|Gas6,14456:up|Plk2,20620:down|Glp1r,14652:down|Cxcl14,57266:down|Fgfr1,14182:down|Egfr,13649:down|Ncam1,17967:down|Wnt5a,22418:down|Ccl12,20293:up|Ptgs2,19225:down|Il1rn,16181:up|Ccl4,20303:up|Sncg,20618:up|Grem1,23892:up|Igf1,16000:up|Cxcl9,17329:up|Mcoln3,171166:up|Aldh1a3,56847:down|Prkar2b,19088:up
positive regulation of protein import into nucleus	GO:0042307	2.70E-05	3.57E-04	Tnf,21926:up|Egfr,13649:down|Bmpr1a,12166:down|Ptgs2,19225:down|Grem1,23892:up|Igf1,16000:up|Bmp4,12159:up|Ceacam1,26365:up|Sfrp2,20319:up
stem cell proliferation	GO:0072089	2.84E-05	3.74E-04	Prl2c2,18811:up|Fgfr1,14182:down|Cd24a,12484:down|Wnt5a,22418:down|Bmpr1a,12166:down|Bmp4,12159:up|Sfrp2,20319:up|Prg4,96875:down|Vegfa,22339:down|Cx3cr1,13051:down|Fgfr2,14183:up|Kitl,17311:up
positive regulation of secretion	GO:0051047	2.88E-05	3.78E-04	Htr2b,15559:down|Ednrb,13618:up|Ccl3,20302:up|Mmp13,17386:up|Ankrd1,107765:down|Clec4n,56620:up|Cxcl12,20315:up|Postn,50706:down|Tnf,21926:up|Glp1r,14652:down|Egfr,13649:down|Fgfr1,14182:down|Lpl,16956:up|C1qtnf3,81799:down|Wnt5a,22418:down|Igf1,16000:up|Ceacam1,26365:up|Tgfb2,21808:down|Clec4e,56619:up|Il1rl1,17082:down

positive regulation of cell development	GO:0010720	2.92E-05	3.82E-04	Prl2c2,18811:up|Ptk7,71461:down|Dpysl3,22240:down|Sema7a,20361:down|Ankrd1,107765:down|Tnfrsf12a,27279:down|Vdr,22337:down|Cxcl12,20315:up|Vegfa,22339:down|Cx3cr1,13051:down|Serpine2,20720:up|Pparg,19016:up|Trf,22041:up|Cd24a,12484:down|Fgfr1,14182:down|Has2,15117:down|Wnt5a,22418:down|Wisp1,22402:down|Adipoq,11450:up|Bmp4,12159:up|Igf1,16000:up|Crabp2,12904:down|Ret,19713:up|Tgfb2,21808:down
regulation of cardiac muscle tissue development	GO:0055024	2.97E-05	3.88E-04	Fgfr1,14182:down|Ncam1,17967:down|Bmpr1a,12166:down|Wisp1,22402:down|Grem1,23892:up|Igf1,16000:up|Bmp4,12159:up|Fgfr2,14183:up
organ maturation	GO:0048799	2.99E-05	3.88E-04	Fam101b,76566:down|Ret,19713:up|Aldh1a2,19378:up|Grem1,23892:up|Igf1,16000:up
myeloid leukocyte cytokine production	GO:0061082	2.99E-05	3.88E-04	Gas6,14456:up|Hmox1,15368:up|Wnt5a,22418:down|Tgfb2,21808:down|Sema7a,20361:down
bone trabecula formation	GO:0060346	3.12E-05	4.04E-04	Sfrp1,20377:up|Vegfa,22339:down|Mmp2,17390:down|Grem1,23892:up
negative regulation of immune response	GO:0050777	3.27E-05	4.22E-04	Tnf,21926:up|Pparg,19016:up|Clec2d,93694:up|Hmox1,15368:up|Col3a1,12825:down|Ceacam1,26365:up|Serping1,12258:up|Tgfb2,21808:down|Igf2,16002:up|Il1rl1,17082:down
response to amino acid	GO:0043200	3.31E-05	4.27E-04	Col5a2,12832:down|Tnf,21926:up|Col1a2,12843:down|Egfr,13649:down|Pdgfc,54635:down|Col3a1,12825:down|Mmp2,17390:down|Cdo1,12583:up
regulation of protein secretion	GO:0050708	3.34E-05	4.30E-04	Htr2b,15559:down|Ccl3,20302:up|Mmp13,17386:up|Ankrd1,107765:down|Clec4n,56620:up|Postn,50706:down|Gas6,14456:up|Tnf,21926:up|Glp1r,14652:down|Egfr,13649:down|Lpl,16956:up|Sfrp1,20377:up|C1qtnf3,81799:down|Wnt5a,22418:down|Igf1,16000:up|Ceacam1,26365:up|Tgfb2,21808:down|Clec4e,56619:up|Il1rl1,17082:down
regulation of behavior	GO:0050795	3.35E-05	4.30E-04	Htr2b,15559:down|Gas6,14456:up|Cxcl14,57266:down|Fgfr1,14182:down|Plxnd1,67784:up|Wnt5a,22418:down|Ccl2,20296:up|Ccl12,20293:up|Figf,14205:up|Grem1,23892:up|Ccl4,20303:up|Cxcl9,17329:up|Cxcl12,20315:up|Vegfa,22339:down|Htr2a,15558:down
positive regulation of fat cell differentiation	GO:0045600	3.42E-05	4.39E-04	Pparg,19016:up|Sfrp2,20319:up|Sfrp1,20377:up|Htr2a,15558:down|Medag,70717:down|Ptgs2,19225:down|Igf1,16000:up
regulation of protein transport	GO:0051223	3.46E-05	4.42E-04	Htr2b,15559:down|Ice1,218333:down|Ccl2,20296:up|Ccl3,20302:up|Mmp13,17386:up|Ankrd1,107765:down|Clec4n,56620:up|Postn,50706:down|Kdelr3,105785:down|Gas6,14456:up|Tnf,21926:up|Glp1r,14652:down|Egfr,13649:down|Lpl,16956:up|Sfrp1,20377:up|C1qtnf3,81799:down|Wnt5a,22418:down|Bmpr1a,12166:down|Ptgs2,19225:down|Grem1,23892:up|Adipoq,11450:up|Bmp4,12159:up|Igf1,16000:up|Ceacam1,26365:up|Sfrp2,20319:up|Tgfb2,21808:down|Il1rl1,17082:down|Clec4e,56619:up
positive regulation of secretion by cell	GO:1903532	3.47E-05	4.42E-04	Htr2b,15559:down|Ccl3,20302:up|Mmp13,17386:up|Ankrd1,107765:down|Clec4n,56620:up|Cxcl12,20315:up|Postn,50706:down|Tnf,21926:up|Glp1r,14652:down|Egfr,13649:down|Fgfr1,14182:down|Lpl,16956:up|C1qtnf3,81799:down|Wnt5a,22418:down|Igf1,16000:up|Ceacam1,26365:up|Tgfb2,21808:down|Clec4e,56619:up|Il1rl1,17082:down
muscle tissue morphogenesis	GO:0060415	3.68E-05	4.67E-04	Col11a1,12814:down|Wnt5a,22418:down|Bmpr1a,12166:down|Tgfbr3,21814:up|Col3a1,12825:down|Ankrd1,107765:down|Tgfb2,21808:down|Fgfr2,14183:up
negative regulation of osteoclast differentiation	GO:0045671	3.81E-05	4.82E-04	Fstl3,83554:down|Ceacam1,26365:up|Clec2d,93694:up|Lrrc17,74511:down|Sfrp1,20377:up
mesodermal cell differentiation	GO:0048333	3.81E-05	4.82E-04	Sfrp2,20319:up|Fgfr1,14182:down|Inhba,16323:down|Bmpr1a,12166:down|Bmp4,12159:up
negative regulation of apoptotic process	GO:0043066	3.93E-05	4.95E-04	Htr2b,15559:down|Ednrb,13618:up|Crlf1,12931:down|Cxcl12,20315:up|Vegfa,22339:down|Cx3cr1,13051:down|Slc40a1,53945:up|Gas6,14456:up|Tnf,21926:up|Plk2,20620:down|Glp1r,14652:down|Tnfrsf23,79201:down|Egfr,13649:down|Hmox1,15368:up|Sfrp1,20377:up|Wnt5a,22418:down|Ccl12,20293:up|Tgfbr3,21814:up|Il1rn,16181:up|Grem1,23892:up|Bmp4,12159:up|Igf1,16000:up|Chst11,58250:down|Sfrp2,20319:up|Tgfb2,21808:down|Pdxk,216134:up|Creb3l1,26427:down|Fgfr2,14183:up|Kitl,17311:up|Mt1,17748:up
humoral immune response mediated by circulating immunoglobulin	GO:0002455	4.08E-05	5.12E-04	Tnf,21926:up|C1s1,50908:up|Serping1,12258:up|C1s2,317677:up|C3,12266:up|C1rb,667277:up
digestive tract development	GO:0048565	4.44E-05	5.54E-04	Egfr,13649:down|Sfrp1,20377:up|Wnt5a,22418:down|Aldh1a2,19378:up|Pdgfc,54635:down|Ednrb,13618:up|Col3a1,12825:down|Bmp4,12159:up|Sfrp2,20319:up|Fgfr2,14183:up

regulation of cellular process	GO:0050794	4.45E-05	5.54E-04	Fstl3,83554:down|Igfbp4,16010:up|Adm,11535:up|Flrt2,399558:down|Tmem176b,65963:up|Plxnd1,67784:up|Tfec,21426:up|Sfrp4,20379:up|Lyl1,17095:up|Igfbp3,16009:up|Plk2,20620:down|Cd24a,12484:down|Il1rn,16181:up|Maob,109731:up|Bmp4,12159:up|Oasl1,231655:up|Hpse,15442:up|Medag,70717:down|Ptgir,19222:up|Fgfr2,14183:up|Thbs2,21826:down|Htr2b,15559:down|Dram1,71712:up|Ccl6,20305:up|Slpi,20568:up|Aspn,66695:down|Mt2,17750:up|Cxcr6,80901:down|Fam132b,227358:down|Nabp1,109019:down|Mmp2,17390:down|Oas3,246727:up|Slc40a1,53945:up|Egfr,13649:down|Tfrc,22042:up|Sncg,20618:up|Ccl8,20307:up|Serpina3g,20715:up|Rspo2,239405:up|Fam20a,208659:down|Mt1,17748:up|Col5a2,12832:down|Aldh1a2,19378:up|Ccl2,20296:up|Ednrb,13618:up|Mmp13,17386:up|Htra3,78558:down|Tnfrsf12a,27279:down|Uchl5,56207:up|Clec4n,56620:up|Dok2,13449:up|Hvcn1,74096:up|Ptx3,19288:up|Ncam1,17967:down|Ccl4,20303:up|Chst11,58250:down|Vegfb,22340:up|Aldh1a3,56847:down|Plpp3,67916:up|Lrrc17,74511:down|Lama2,16773:down|Ccl9,20308:up|Ptk7,71461:down|Inhba,16323:down|Bnc2,242509:down|Tfpi2,21789:down|Crlf1,12931:down|Plod2,26432:down|Vegfa,22339:down|Serpine2,20720:up|Nxn,18230:down|Igf2,16002:up|Tmem176a,66058:up|Tnf,21926:up|Col1a2,12843:down|Fxyd1,56188:up|Fgfr1,14182:down|Lpl,16956:up|Sulf1,240725:down|Susd2,71733:up|Cd4,12504:up|Col3a1,12825:down|Tbx15,21384:down|Serping1,12258:up|Enpp1,18605:down|Clec2d,93694:up|Hpgd,15446:up|C3,12266:up|Ankrd1,107765:down|Cx3cr1,13051:down|Cd109,235505:down|Loxl2,94352:down|Trf,22041:up|Loxl3,16950:down|Cxcl14,57266:down|Wnt5a,22418:down|Stc1,20855:up|Mark1,226778:up|Col8a1,12837:down|Adipoq,11450:up|Igf1,16000:up|Crabp2,12904:down|Cxcl9,17329:up|Tgfb2,21808:down|F3,14066:up|Clec4e,56619:up|Cthrc1,68588:down|Kng1,16644:up|Prl2c2,18811:up|Efemp1,216616:up|Dpysl3,22240:down|Ninj1,18081:up|Saa3,20210:up|Vdr,22337:down|Pdia5,72599:down|Fabp4,11770:up|Cxcl12,20315:up|Htr2a,15558:down|Smoc2,64074:down|Gas6,14456:up|Sfrp1,20377:up|C1qtnf3,81799:down|Tgfbr3,21814:up|Fxyd2,11936:up|Ceacam1,26365:up|Sfrp2,20319:up|Tnfrsf11b,18383:up|Prkar2b,19088:up|Npnt,114249:up|S100a1,20193:up|Ice1,218333:down|Ccl7,20306:up|Pdgfc,54635:down|Figf,14205:up|Cst7,13011:up|Sema7a,20361:down|Epas1,13819:up|Slc29a1,63959:up|Gpm6b,14758:up|Ddah1,69219:down|Sdpr,20324:down|Postn,50706:down|Akr1c18,105349:up|Ccl12,20293:up|Bmpr1a,12166:down|Wisp1,22402:down|Tnc,21923:down|Ret,19713:up|Prg4,96875:down|Sdf2l1,64136:up|Pdxk,216134:up|Ifit1,15957:up|Creb3l1,26427:down|Kitl,17311:up|Il1rl1,17082:down|Fam101b,76566:down|Hp,15439:up|Rasl11a,68895:up|Ccl3,20302:up|Plat,18791:down|Ctla2a,13024:down|Srpx2,68792:down|Adamts12,239337:down|Lrrc15,74488:down|Pparg,19016:up|Lmcd1,30937:up|Glp1r,14652:down|Tnfrsf23,79201:down|Hmox1,15368:up|Has2,15117:down|Ptgs2,19225:down|Grem1,23892:up|Kcnj15,16516:down|Phf11d,219132:up|Col5a1,12831:down|Errfi1,74155:down|Tnfsf9,21950:up
response to BMP	GO:0071772	4.45E-05	5.54E-04	Fstl3,83554:down|Sfrp1,20377:up|Sulf1,240725:down|Wnt5a,22418:down|Bmpr1a,12166:down|Tgfbr3,21814:up|Grem1,23892:up|Bmp4,12159:up|Htra3,78558:down|Sfrp2,20319:up|Adamts12,239337:down
cellular response to BMP stimulus	GO:0071773	4.45E-05	5.54E-04	Fstl3,83554:down|Sfrp1,20377:up|Sulf1,240725:down|Wnt5a,22418:down|Bmpr1a,12166:down|Tgfbr3,21814:up|Grem1,23892:up|Bmp4,12159:up|Htra3,78558:down|Sfrp2,20319:up|Adamts12,239337:down
response to hormone	GO:0009725	4.55E-05	5.66E-04	Inhba,16323:down|Vdr,22337:down|Sord,20322:up|Mmp2,17390:down|Igf2,16002:up|Pappa,18491:down|Cdo1,12583:up|Pparg,19016:up|Tnf,21926:up|Akr1c18,105349:up|Egfr,13649:down|Cd24a,12484:down|Alpl,11647:up|Hmox1,15368:up|Sfrp1,20377:up|Tgfbr3,21814:up|Il1rn,16181:up|Adipoq,11450:up|Bmp4,12159:up|Igf1,16000:up|Gatm,67092:down|Serpina3g,20715:up|Tgfb2,21808:down|Ptgir,19222:up|Enpp1,18605:down
response to organic cyclic compound	GO:0014070	4.58E-05	5.68E-04	Htr2b,15559:down|Inhba,16323:down|Ifit3,15959:up|Saa3,20210:up|Vdr,22337:down|Htr2a,15558:down|Mmp2,17390:down|Igf2,16002:up|Pappa,18491:down|Cdo1,12583:up|Pparg,19016:up|Tnf,21926:up|Akr1c18,105349:up|Egfr,13649:down|Cd24a,12484:down|Alpl,11647:up|Ncam1,17967:down|Hmox1,15368:up|Sfrp1,20377:up|Ccl12,20293:up|Il1rn,16181:up|Adipoq,11450:up|Bmp4,12159:up|Igf1,16000:up|Tgfb2,21808:down|Ifit1,15957:up
fever generation	GO:0001660	4.61E-05	5.70E-04	Tnf,21926:up|Ptgs2,19225:down|Il1rn,16181:up|Ednrb,13618:up
protein secretion	GO:0009306	4.71E-05	5.81E-04	Htr2b,15559:down|Ccl3,20302:up|Mmp13,17386:up|Ankrd1,107765:down|Clec4n,56620:up|Postn,50706:down|Gas6,14456:up|Tnf,21926:up|Glp1r,14652:down|Egfr,13649:down|Lpl,16956:up|Sfrp1,20377:up|C1qtnf3,81799:down|Wnt5a,22418:down|Il1rn,16181:up|Sncg,20618:up|Igf1,16000:up|Ceacam1,26365:up|Tgfb2,21808:down|Clec4e,56619:up|Il1rl1,17082:down
positive regulation of cell-cell adhesion	GO:0022409	4.74E-05	5.84E-04	Fstl3,83554:down|Tnf,21926:up|Cd24a,12484:down|Has2,15117:down|Wnt5a,22418:down|Ccl2,20296:up|Cd4,12504:up|Tfrc,22042:up|Igf1,16000:up|Ceacam1,26365:up|Plpp3,67916:up|Igf2,16002:up|Tnfsf9,21950:up
collagen biosynthetic process	GO:0032964	4.79E-05	5.88E-04	Serpinh1,12406:down|Pparg,19016:up|Col5a1,12831:down|Ccl2,20296:up|Errfi1,74155:down|Bmp4,12159:up
regulation of nervous system development	GO:0051960	4.91E-05	6.01E-04	Prl2c2,18811:up|Flrt2,399558:down|Efemp1,216616:up|Plxnd1,67784:up|Ptk7,71461:down|Dpysl3,22240:down|Ednrb,13618:up|Sema7a,20361:down|Ankrd1,107765:down|Tnfrsf12a,27279:down|Srpx2,68792:down|Cxcl12,20315:up|Vegfa,22339:down|Cx3cr1,13051:down|Serpine2,20720:up|Pparg,19016:up|Tnf,21926:up|Plk2,20620:down|Trf,22041:up|Fgfr1,14182:down|Cd24a,12484:down|Sfrp1,20377:up|Wnt5a,22418:down|Bmpr1a,12166:down|Col3a1,12825:down|Bmp4,12159:up|Igf1,16000:up|Crabp2,12904:down|Sfrp2,20319:up|Ret,19713:up|Thbs2,21826:down
cellular response to amino acid stimulus	GO:0071230	5.00E-05	6.07E-04	Col5a2,12832:down|Tnf,21926:up|Col1a2,12843:down|Egfr,13649:down|Pdgfc,54635:down|Mmp2,17390:down|Col3a1,12825:down
cardiac muscle tissue growth	GO:0055017	5.00E-05	6.07E-04	Fgfr1,14182:down|Ncam1,17967:down|Bmpr1a,12166:down|Tgfbr3,21814:up|Wisp1,22402:down|Igf1,16000:up|Tgfb2,21808:down|Fgfr2,14183:up
reproductive structure development	GO:0048608	5.01E-05	6.07E-04	Adm,11535:up|Inhba,16323:down|Gjb5,14622:down|Epas1,13819:up|Vegfa,22339:down|Mmp2,17390:down|Serpine2,20720:up|Pparg,19016:up|Egfr,13649:down|Sfrp1,20377:up|Sulf1,240725:down|Wnt5a,22418:down|Ptgs2,19225:down|Bmp4,12159:up|Igf1,16000:up|Sfrp2,20319:up|Tnc,21923:down|Fgfr2,14183:up|Kitl,17311:up
mesonephric epithelium development	GO:0072163	5.02E-05	6.07E-04	Fgfr1,14182:down|Sfrp1,20377:up|Grem1,23892:up|Bmp4,12159:up|Crlf1,12931:down|Ret,19713:up|Vegfa,22339:down|Npnt,114249:up|Fgfr2,14183:up
ureteric bud development	GO:0001657	5.02E-05	6.07E-04	Fgfr1,14182:down|Sfrp1,20377:up|Grem1,23892:up|Bmp4,12159:up|Crlf1,12931:down|Ret,19713:up|Vegfa,22339:down|Npnt,114249:up|Fgfr2,14183:up
mesonephric tubule development	GO:0072164	5.02E-05	6.07E-04	Fgfr1,14182:down|Sfrp1,20377:up|Grem1,23892:up|Bmp4,12159:up|Crlf1,12931:down|Ret,19713:up|Vegfa,22339:down|Npnt,114249:up|Fgfr2,14183:up
positive regulation of cell growth	GO:0030307	5.07E-05	6.12E-04	Egfr,13649:down|Sfrp1,20377:up|Wisp1,22402:down|Sema7a,20361:down|Igf1,16000:up|Tnfrsf12a,27279:down|Sfrp2,20319:up|Crabp2,12904:down|Cxcl12,20315:up|Vegfa,22339:down|Tgfb2,21808:down

positive regulation of protein localization to nucleus	GO:1900182	5.14E-05	6.20E-04	Tnf,21926:up|Egfr,13649:down|Bmpr1a,12166:down|Ptgs2,19225:down|Grem1,23892:up|Igf1,16000:up|Bmp4,12159:up|Ceacam1,26365:up|Sfrp2,20319:up|Tgfb2,21808:down
regulation of cellular localization	GO:0060341	5.19E-05	6.24E-04	Ice1,218333:down|Ccl2,20296:up|Mmp13,17386:up|Ankrd1,107765:down|Gpm6b,14758:up|Clec4n,56620:up|Postn,50706:down|Ncam1,17967:down|Wnt5a,22418:down|Bmpr1a,12166:down|Il1rn,16181:up|Maob,109731:up|Adipoq,11450:up|Bmp4,12159:up|Igf1,16000:up|Tgfb2,21808:down|Il1rl1,17082:down|Clec4e,56619:up|Htr2b,15559:down|Inhba,16323:down|Ccl3,20302:up|Cxcl12,20315:up|Htr2a,15558:down|Lrrc15,74488:down|Pparg,19016:up|Gas6,14456:up|Tnf,21926:up|Glp1r,14652:down|Egfr,13649:down|Fgfr1,14182:down|Sfrp1,20377:up|Lpl,16956:up|Hmox1,15368:up|C1qtnf3,81799:down|Cd4,12504:up|Ptgs2,19225:down|Sncg,20618:up|Grem1,23892:up|Ceacam1,26365:up|Sfrp2,20319:up
regulation of establishment of protein localization	GO:0070201	5.20E-05	6.24E-04	Htr2b,15559:down|Ice1,218333:down|Ccl2,20296:up|Ccl3,20302:up|Mmp13,17386:up|Ankrd1,107765:down|Clec4n,56620:up|Postn,50706:down|Lrrc15,74488:down|Kdelr3,105785:down|Gas6,14456:up|Tnf,21926:up|Glp1r,14652:down|Egfr,13649:down|Lpl,16956:up|Sfrp1,20377:up|C1qtnf3,81799:down|Wnt5a,22418:down|Bmpr1a,12166:down|Ptgs2,19225:down|Grem1,23892:up|Adipoq,11450:up|Bmp4,12159:up|Igf1,16000:up|Ceacam1,26365:up|Sfrp2,20319:up|Tgfb2,21808:down|Il1rl1,17082:down|Clec4e,56619:up
negative regulation of programmed cell death	GO:0043069	5.39E-05	6.45E-04	Htr2b,15559:down|Ednrb,13618:up|Crlf1,12931:down|Cxcl12,20315:up|Vegfa,22339:down|Cx3cr1,13051:down|Slc40a1,53945:up|Gas6,14456:up|Tnf,21926:up|Plk2,20620:down|Glp1r,14652:down|Tnfrsf23,79201:down|Egfr,13649:down|Hmox1,15368:up|Sfrp1,20377:up|Wnt5a,22418:down|Ccl12,20293:up|Tgfbr3,21814:up|Il1rn,16181:up|Grem1,23892:up|Bmp4,12159:up|Igf1,16000:up|Chst11,58250:down|Sfrp2,20319:up|Tgfb2,21808:down|Pdxk,216134:up|Creb3l1,26427:down|Fgfr2,14183:up|Kitl,17311:up|Mt1,17748:up
regulation of leukocyte migration	GO:0002685	5.41E-05	6.47E-04	Gas6,14456:up|Cxcl14,57266:down|Wnt5a,22418:down|Ccl2,20296:up|Ccl12,20293:up|Figf,14205:up|Grem1,23892:up|Ccl4,20303:up|Cxcl9,17329:up|Cxcl12,20315:up|Kitl,17311:up
cell development	GO:0048468	5.50E-05	6.56E-04	Adm,11535:up|Flrt2,399558:down|Plxnd1,67784:up|Aldh1a2,19378:up|Ednrb,13618:up|Sema7a,20361:down|Epas1,13819:up|Ankrd1,107765:down|Tnfrsf12a,27279:down|Serpinh1,12406:down|Postn,50706:down|Cx3cr1,13051:down|Loxl2,94352:down|Plk2,20620:down|Loxl3,16950:down|Trf,22041:up|Cd24a,12484:down|Ncam1,17967:down|Wnt5a,22418:down|Bmpr1a,12166:down|Wisp1,22402:down|Stc1,20855:up|Adipoq,11450:up|Bmp4,12159:up|Igf1,16000:up|Chst11,58250:down|Crabp2,12904:down|Tnc,21923:down|Ret,19713:up|Tgfb2,21808:down|Fgfr2,14183:up|Kitl,17311:up|Fam101b,76566:down|Cthrc1,68588:down|Htr2b,15559:down|Prl2c2,18811:up|Lama2,16773:down|Efemp1,216616:up|Ptk7,71461:down|Inhba,16323:down|Dpysl3,22240:down|Vdr,22337:down|Cxcl12,20315:up|Vegfa,22339:down|Mmp2,17390:down|Serpine2,20720:up|Pparg,19016:up|Tnf,21926:up|Col11a1,12814:down|Egfr,13649:down|Fgfr1,14182:down|Has2,15117:down|Sfrp1,20377:up|Sulf1,240725:down|Tgfbr3,21814:up|Grem1,23892:up|Col3a1,12825:down|Sfrp2,20319:up
smooth muscle cell migration	GO:0014909	5.52E-05	6.56E-04	Has2,15117:down|Bmpr1a,12166:down|Plat,18791:down|Igf1,16000:up|Adipoq,11450:up|Postn,50706:down|Igfbp3,16009:up|F3,14066:up
vascular process in circulatory system	GO:0003018	5.53E-05	6.56E-04	Htr2b,15559:down|Kng1,16644:up|Adm,11535:up|Egfr,13649:down|Tgfbr3,21814:up|Ptgs2,19225:down|Ednrb,13618:up|Ccl4,20303:up|Ceacam1,26365:up|Vegfa,22339:down|Htr2a,15558:down|Mmp2,17390:down
negative regulation of ion transport	GO:0043271	5.53E-05	6.56E-04	Tnf,21926:up|Sfrp4,20379:up|Ptgs2,19225:down|Stc1,20855:up|Il1rn,16181:up|Maob,109731:up|Gpm6b,14758:up|Tgfb2,21808:down|Htr2a,15558:down|Serpine2,20720:up
reproductive system development	GO:0061458	5.58E-05	6.60E-04	Adm,11535:up|Inhba,16323:down|Gjb5,14622:down|Epas1,13819:up|Vegfa,22339:down|Mmp2,17390:down|Serpine2,20720:up|Pparg,19016:up|Egfr,13649:down|Sfrp1,20377:up|Sulf1,240725:down|Wnt5a,22418:down|Ptgs2,19225:down|Bmp4,12159:up|Igf1,16000:up|Sfrp2,20319:up|Tnc,21923:down|Fgfr2,14183:up|Kitl,17311:up
negative regulation of cell death	GO:0060548	5.60E-05	6.61E-04	Ednrb,13618:up|Cx3cr1,13051:down|Plk2,20620:down|Ncam1,17967:down|Wnt5a,22418:down|Ccl12,20293:up|Wisp1,22402:down|Il1rn,16181:up|Bmp4,12159:up|Igf1,16000:up|Chst11,58250:down|Tgfb2,21808:down|Pdxk,216134:up|Creb3l1,26427:down|Fgfr2,14183:up|Kitl,17311:up|Htr2b,15559:down|Crlf1,12931:down|Cxcl12,20315:up|Vegfa,22339:down|Slc40a1,53945:up|Tnf,21926:up|Gas6,14456:up|Glp1r,14652:down|Egfr,13649:down|Tnfrsf23,79201:down|Sfrp1,20377:up|Hmox1,15368:up|Tgfbr3,21814:up|Grem1,23892:up|Sfrp2,20319:up|Mt1,17748:up
response to steroid hormone	GO:0048545	5.63E-05	6.63E-04	Vdr,22337:down|Mmp2,17390:down|Pappa,18491:down|Cdo1,12583:up|Pparg,19016:up|Tnf,21926:up|Akr1c18,105349:up|Alpl,11647:up|Cd24a,12484:down|Egfr,13649:down|Hmox1,15368:up|Sfrp1,20377:up|Il1rn,16181:up|Bmp4,12159:up|Igf1,16000:up|Tgfb2,21808:down
exocrine system development	GO:0035272	5.64E-05	6.63E-04	Tnf,21926:up|Fgfr1,14182:down|Egfr,13649:down|Plxnd1,67784:up|Igf2,16002:up|Fgfr2,14183:up|Igf1,16000:up
natural killer cell chemotaxis	GO:0035747	5.69E-05	6.65E-04	Cxcl14,57266:down|Ccl12,20293:up|Ccl4,20303:up
regulation of natural killer cell chemotaxis	GO:2000501	5.69E-05	6.65E-04	Cxcl14,57266:down|Ccl12,20293:up|Ccl4,20303:up
regulation of cytokine secretion	GO:0050707	5.77E-05	6.74E-04	Htr2b,15559:down|Tnf,21926:up|Gas6,14456:up|Lpl,16956:up|C1qtnf3,81799:down|Wnt5a,22418:down|Ccl3,20302:up|Clec4n,56620:up|Postn,50706:down|Clec4e,56619:up|Il1rl1,17082:down
mesonephros development	GO:0001823	5.93E-05	6.91E-04	Fgfr1,14182:down|Sfrp1,20377:up|Grem1,23892:up|Bmp4,12159:up|Crlf1,12931:down|Ret,19713:up|Vegfa,22339:down|Npnt,114249:up|Fgfr2,14183:up
neutrophil migration	GO:1990266	6.09E-05	7.06E-04	Ccl9,20308:up|Ccl6,20305:up|Ccl7,20306:up|Ccl2,20296:up|Ccl12,20293:up|Ccl4,20303:up|Ccl3,20302:up|Ccl8,20307:up
positive regulation of endothelial cell proliferation	GO:0001938	6.35E-05	7.36E-04	Htr2b,15559:down|Cxcl12,20315:up|Wnt5a,22418:down|Vegfa,22339:down|Ccl2,20296:up|F3,14066:up|Bmp4,12159:up
regulation of bone resorption	GO:0045124	6.53E-05	7.55E-04	Ceacam1,26365:up|Trf,22041:up|Egfr,13649:down|Vegfa,22339:down|Tfrc,22042:up|Tnfrsf11b,18383:up
lateral sprouting from an epithelium	GO:0060601	6.57E-05	7.56E-04	Sulf1,240725:down|Wnt5a,22418:down|Fgfr2,14183:up|Bmp4,12159:up
positive regulation of nervous system development	GO:0051962	6.67E-05	7.67E-04	Prl2c2,18811:up|Flrt2,399558:down|Ptk7,71461:down|Dpysl3,22240:down|Sema7a,20361:down|Ankrd1,107765:down|Tnfrsf12a,27279:down|Srpx2,68792:down|Cxcl12,20315:up|Vegfa,22339:down|Cx3cr1,13051:down|Serpine2,20720:up|Pparg,19016:up|Trf,22041:up|Cd24a,12484:down|Fgfr1,14182:down|Wnt5a,22418:down|Bmp4,12159:up|Igf1,16000:up|Crabp2,12904:down|Ret,19713:up|Thbs2,21826:down
cellular response to inorganic substance	GO:0071241	6.98E-05	8.00E-04	Pparg,19016:up|Akr1c18,105349:up|Hvcn1,74096:up|Trf,22041:up|Hmox1,15368:up|Tfrc,22042:up|Fabp4,11770:up|Mt1,17748:up|Slc40a1,53945:up
proteoglycan metabolic process	GO:0006029	7.13E-05	8.17E-04	Mamdc2,71738:down|Chst11,58250:down|Col11a1,12814:down|Sulf1,240725:down|Hpse,15442:up|Adamts12,239337:down|Igf1,16000:up

regulation of cell fate commitment	GO:0010453	7.35E-05	8.37E-04	Sfrp2,20319:up|Fgfr1,14182:down|Bmpr1a,12166:down|Fgfr2,14183:up|Bmp4,12159:up
negative regulation of myoblast differentiation	GO:0045662	7.35E-05	8.37E-04	Tnf,21926:up|Prl2c2,18811:up|Cxcl14,57266:down|Ccl9,20308:up|Bmp4,12159:up
acute-phase response	GO:0006953	7.56E-05	8.56E-04	Tnf,21926:up|Hp,15439:up|Ptgs2,19225:down|Il1rn,16181:up|Ednrb,13618:up|Saa3,20210:up
negative regulation of myeloid leukocyte differentiation	GO:0002762	7.56E-05	8.56E-04	Fstl3,83554:down|Ceacam1,26365:up|Clec2d,93694:up|Lrrc17,74511:down|Sfrp1,20377:up|Adipoq,11450:up
negative regulation of protein phosphorylation	GO:0001933	7.81E-05	8.83E-04	Flrt2,399558:down|Sfrp4,20379:up|Aspn,66695:down|Fabp4,11770:up|Igfbp3,16009:up|Cd109,235505:down|Lrrc15,74488:down|Sfrp1,20377:up|Grem1,23892:up|Adipoq,11450:up|Bmp4,12159:up|Igf1,16000:up|Ceacam1,26365:up|Sfrp2,20319:up|Prkar2b,19088:up|Plpp3,67916:up|Enpp1,18605:down|Errfi1,74155:down
regulation of body fluid levels	GO:0050878	7.84E-05	8.83E-04	Kng1,16644:up|C3,12266:up|Pdgfc,54635:down|Anxa8,11752:down|Ednrb,13618:up|Mmp13,17386:up|Tfpi2,21789:down|Vdr,22337:down|Vegfa,22339:down|Serpine2,20720:up|Cdo1,12583:up|Gas6,14456:up|Ceacam1,26365:up|Serping1,12258:up|Hpse,15442:up|F3,14066:up
neural crest cell differentiation	GO:0014033	8.07E-05	9.04E-04	Htr2b,15559:down|Sfrp1,20377:up|Bmpr1a,12166:down|Aldh1a2,19378:up|Ednrb,13618:up|Sema7a,20361:down|Ret,19713:up|Kitl,17311:up
muscle organ morphogenesis	GO:0048644	8.07E-05	9.04E-04	Col11a1,12814:down|Wnt5a,22418:down|Bmpr1a,12166:down|Tgfbr3,21814:up|Col3a1,12825:down|Ankrd1,107765:down|Tgfb2,21808:down|Fgfr2,14183:up
heart growth	GO:0060419	8.07E-05	9.04E-04	Fgfr1,14182:down|Ncam1,17967:down|Bmpr1a,12166:down|Tgfbr3,21814:up|Wisp1,22402:down|Igf1,16000:up|Tgfb2,21808:down|Fgfr2,14183:up
cellular divalent inorganic cation homeostasis	GO:0072503	8.10E-05	9.05E-04	Htr2b,15559:down|Kng1,16644:up|Adm,11535:up|Ccl2,20296:up|Ednrb,13618:up|Ccl3,20302:up|Mt2,17750:up|Vdr,22337:down|Htr2a,15558:down|Fxyd1,56188:up|Glp1r,14652:down|Cd24a,12484:down|Cd4,12504:up|Stc1,20855:up|Bmp4,12159:up|Tgfb2,21808:down|Ptgir,19222:up|Mt1,17748:up
positive regulation of hydrolase activity	GO:0051345	8.19E-05	9.14E-04	Htr2b,15559:down|Ccl9,20308:up|Ccl6,20305:up|Ccl7,20306:up|Ccl2,20296:up|Ccl3,20302:up|Vegfa,22339:down|Htr2a,15558:down|Pparg,19016:up|Tnf,21926:up|Fgfr1,14182:down|Wnt5a,22418:down|Ccl12,20293:up|Ccl4,20303:up|Ccl8,20307:up|Sfrp2,20319:up|Npnt,114249:up|Fgfr2,14183:up|F3,14066:up
response to molecule of bacterial origin	GO:0002237	8.54E-05	9.51E-04	Tnf,21926:up|Adm,11535:up|Cd24a,12484:down|Mrc1,17533:up|Wnt5a,22418:down|Ccl2,20296:up|Ccl12,20293:up|Ptgs2,19225:down|Slpi,20568:up|Ednrb,13618:up|Ankrd1,107765:down|Cxcl9,17329:up|Tnfrsf11b,18383:up|Ptgir,19222:up
regulation of chondrocyte differentiation	GO:0032330	8.73E-05	9.68E-04	Fam101b,76566:down|Loxl2,94352:down|Efemp1,216616:up|Adamts12,239337:down|Grem1,23892:up|Bmp4,12159:up
regulation of mesenchymal cell proliferation	GO:0010464	8.73E-05	9.68E-04	Fgfr1,14182:down|Wnt5a,22418:down|Vegfa,22339:down|Bmpr1a,12166:down|Fgfr2,14183:up|Bmp4,12159:up
granulocyte chemotaxis	GO:0071621	8.84E-05	9.80E-04	Ccl9,20308:up|Ccl6,20305:up|Ccl7,20306:up|Ccl2,20296:up|Ccl12,20293:up|Ccl4,20303:up|Ccl3,20302:up|Ccl8,20307:up
complement activation, classical pathway	GO:0006958	8.97E-05	9.89E-04	C1s1,50908:up|Serping1,12258:up|C1s2,317677:up|C3,12266:up|C1rb,667277:up
morphogenesis of an epithelial fold	GO:0060571	8.97E-05	9.89E-04	Egfr,13649:down|Sulf1,240725:down|Wnt5a,22418:down|Fgfr2,14183:up|Bmp4,12159:up
bone trabecula morphogenesis	GO:0061430	9.06E-05	9.90E-04	Sfrp1,20377:up|Vegfa,22339:down|Mmp2,17390:down|Grem1,23892:up
regulation of establishment of planar polarity involved in neural tube closure	GO:0090178	9.06E-05	9.90E-04	Sfrp2,20319:up|Sfrp1,20377:up|Ptk7,71461:down|Wnt5a,22418:down
macrophage cytokine production	GO:0010934	9.06E-05	9.90E-04	Gas6,14456:up|Wnt5a,22418:down|Tgfb2,21808:down|Sema7a,20361:down
sequestering of triglyceride	GO:0030730	9.06E-05	9.90E-04	Tnf,21926:up|Pparg,19016:up|Lpl,16956:up|Enpp1,18605:down
planar cell polarity pathway involved in neural tube closure	GO:0090179	9.06E-05	9.90E-04	Sfrp2,20319:up|Sfrp1,20377:up|Ptk7,71461:down|Wnt5a,22418:down
regulation of protein import into nucleus	GO:0042306	9.45E-05	1.03E-03	Tnf,21926:up|Gas6,14456:up|Egfr,13649:down|C1qtnf3,81799:down|Bmpr1a,12166:down|Ptgs2,19225:down|Grem1,23892:up|Igf1,16000:up|Bmp4,12159:up|Ceacam1,26365:up|Sfrp2,20319:up
urogenital system development	GO:0001655	9.58E-05	1.04E-03	Aldh1a2,19378:up|Crlf1,12931:down|Vegfa,22339:down|Mmp2,17390:down|Fgfr1,14182:down|Sfrp1,20377:up|Wnt5a,22418:down|Sulf1,240725:down|Grem1,23892:up|Adipoq,11450:up|Bmp4,12159:up|Igf1,16000:up|Tnc,21923:down|Ret,19713:up|Npnt,114249:up|Fgfr2,14183:up
regulation of immune response	GO:0050776	9.84E-05	1.07E-03	Clec2d,93694:up|C3,12266:up|Sema7a,20361:down|C1s1,50908:up|Phf11a,219131:up|Mmp2,17390:down|Igf2,16002:up|Pparg,19016:up|Tnf,21926:up|Cd24a,12484:down|Hmox1,15368:up|Wnt5a,22418:down|Cd4,12504:up|Tfrc,22042:up|C1rb,667277:up|Col3a1,12825:down|Ceacam1,26365:up|Serping1,12258:up|Tgfb2,21808:down|C1s2,317677:up|Il1rl1,17082:down|Clec4e,56619:up
limb morphogenesis	GO:0035108	1.00E-04	1.08E-03	Fgfr1,14182:down|Wnt5a,22418:down|Bmpr1a,12166:down|Aldh1a2,19378:up|Grem1,23892:up|Bmp4,12159:up|Chst11,58250:down|Sfrp2,20319:up|Crabp2,12904:down|Rspo2,239405:up|Fgfr2,14183:up
appendage morphogenesis	GO:0035107	1.00E-04	1.08E-03	Fgfr1,14182:down|Wnt5a,22418:down|Bmpr1a,12166:down|Aldh1a2,19378:up|Grem1,23892:up|Bmp4,12159:up|Chst11,58250:down|Sfrp2,20319:up|Crabp2,12904:down|Rspo2,239405:up|Fgfr2,14183:up
negative regulation of BMP signaling pathway	GO:0030514	1.00E-04	1.08E-03	Fstl3,83554:down|Htra3,78558:down|Sfrp2,20319:up|Sfrp1,20377:up|Wnt5a,22418:down|Grem1,23892:up
transition metal ion homeostasis	GO:0055076	1.03E-04	1.11E-03	Trf,22041:up|Hmox1,15368:up|Tfrc,22042:up|Mt2,17750:up|Epas1,13819:up|Fam132b,227358:down|Steap1,70358:down|Mt1,17748:up|Slc40a1,53945:up

positive regulation of metabolic process	GO:0009893	1.05E-04	1.13E-03	Fstl3,83554:down|Igfbp4,16010:up|Adm,11535:up|Sfrp4,20379:up|C3,12266:up|Lyl1,17095:up|Igfbp3,16009:up|Plk2,20620:down|Trf,22041:up|Cd24a,12484:down|Wnt5a,22418:down|Il1rn,16181:up|Maob,109731:up|Adipoq,11450:up|Bmp4,12159:up|Igf1,16000:up|Tgfb2,21808:down|Ptgir,19222:up|F3,14066:up|Fgfr2,14183:up|Htr2b,15559:down|Prl2c2,18811:up|Ccl6,20305:up|Vdr,22337:down|Htr2a,15558:down|Slc40a1,53945:up|Gas6,14456:up|Egfr,13649:down|Sfrp1,20377:up|C1qtnf3,81799:down|Tgfbr3,21814:up|Tfrc,22042:up|Serinc2,230779:down|Ceacam1,26365:up|Ccl8,20307:up|Sfrp2,20319:up|Fam20a,208659:down|Npnt,114249:up|Tnfrsf11b,18383:up|Ice1,218333:down|Aldh1a2,19378:up|Ccl2,20296:up|Ccl7,20306:up|Pdgfc,54635:down|Ednrb,13618:up|Epas1,13819:up|Sema7a,20361:down|Ddah1,69219:down|Sdpr,20324:down|Ptx3,19288:up|Akr1c18,105349:up|Ccl12,20293:up|Bmpr1a,12166:down|Ccl4,20303:up|Tnc,21923:down|Ret,19713:up|Plpp3,67916:up|Creb3l1,26427:down|Kitl,17311:up|Ccl9,20308:up|Rasl11a,68895:up|Inhba,16323:down|Ccl3,20302:up|Crlf1,12931:down|Plod2,26432:down|Vegfa,22339:down|Igf2,16002:up|Pparg,19016:up|Tnf,21926:up|Lmcd1,30937:up|Glp1r,14652:down|Fgfr1,14182:down|Hmox1,15368:up|Has2,15117:down|Cd4,12504:up|Ptgs2,19225:down|Grem1,23892:up
regulation of leukocyte apoptotic process	GO:2000106	1.06E-04	1.13E-03	Gas6,14456:up|Cd24a,12484:down|Wnt5a,22418:down|Bmp4,12159:up|Ceacam1,26365:up|Cxcl12,20315:up|Tgfb2,21808:down|Kitl,17311:up
regulation of cell division	GO:0051302	1.07E-04	1.15E-03	Htr2b,15559:down|Tnf,21926:up|Wnt5a,22418:down|Susd2,71733:up|Pdgfc,54635:down|Figf,14205:up|Igf1,16000:up|Bmp4,12159:up|Sfrp2,20319:up|Vegfa,22339:down|Tgfb2,21808:down|Vegfb,22340:up|Fgfr2,14183:up|Igf2,16002:up
cardiac muscle tissue morphogenesis	GO:0055008	1.11E-04	1.18E-03	Ankrd1,107765:down|Col11a1,12814:down|Wnt5a,22418:down|Tgfb2,21808:down|Bmpr1a,12166:down|Tgfbr3,21814:up|Fgfr2,14183:up
positive regulation of osteoblast differentiation	GO:0045669	1.11E-04	1.18E-03	Cthrc1,68588:down|Sfrp2,20319:up|Vegfa,22339:down|Bmpr1a,12166:down|Npnt,114249:up|Igf1,16000:up|Bmp4,12159:up
regulation of osteoclast differentiation	GO:0045670	1.11E-04	1.18E-03	Fstl3,83554:down|Ceacam1,26365:up|Tnf,21926:up|Clec2d,93694:up|Lrrc17,74511:down|Sfrp1,20377:up|Ccl3,20302:up
negative regulation of response to external stimulus	GO:0032102	1.12E-04	1.18E-03	Pparg,19016:up|Kng1,16644:up|Hmox1,15368:up|C1qtnf3,81799:down|Wnt5a,22418:down|Aoah,27052:up|Ccl12,20293:up|Grem1,23892:up|Adipoq,11450:up|Ctla2a,13024:down|Ceacam1,26365:up|Serping1,12258:up|Cx3cr1,13051:down|Serpine2,20720:up|Cd109,235505:down
pharyngeal arch artery morphogenesis	GO:0061626	1.12E-04	1.18E-03	Tgfb2,21808:down|Bmpr1a,12166:down|Bmp4,12159:up
regulation of prostatic bud formation	GO:0060685	1.12E-04	1.18E-03	Sulf1,240725:down|Wnt5a,22418:down|Bmp4,12159:up
planar cell polarity pathway involved in axis elongation	GO:0003402	1.12E-04	1.18E-03	Sfrp2,20319:up|Sfrp1,20377:up|Wnt5a,22418:down
tendon development	GO:0035989	1.12E-04	1.18E-03	Col5a1,12831:down|Col11a1,12814:down|Bmp4,12159:up
head development	GO:0060322	1.19E-04	1.25E-03	Inhba,16323:down|Aldh1a2,19378:up|Sema7a,20361:down|Uchl5,56207:up|Shroom4,208431:down|Cxcl12,20315:up|Mmp2,17390:down|Cx3cr1,13051:down|Serpine2,20720:up|Egfr,13649:down|Fgfr1,14182:down|Ncam1,17967:down|Sfrp1,20377:up|Wnt5a,22418:down|Bmpr1a,12166:down|Bmp4,12159:up|Igf1,16000:up|Col3a1,12825:down|Sfrp2,20319:up|Pcdh19,279653:down|Tgfb2,21808:down|Aldh1a3,56847:down|Fgfr2,14183:up
single-organism process	GO:0044699	1.20E-04	1.26E-03	Fstl3,83554:down|Igfbp4,16010:up|Adm,11535:up|Flrt2,399558:down|Tmem176b,65963:up|Plxnd1,67784:up|Sfrp4,20379:up|Ltbp2,16997:down|Serpinh1,12406:down|Lyl1,17095:up|Gstt1,14871:up|Blvrb,233016:up|Igfbp3,16009:up|Ibsp,15891:up|Plk2,20620:down|Cd24a,12484:down|Mme,17380:up|Il1rn,16181:up|Maob,109731:up|Bmp4,12159:up|Hpse,15442:up|Medag,70717:down|Ptgir,19222:up|Fgfr2,14183:up|Thbs2,21826:down|Htr2b,15559:down|Dram1,71712:up|Ccl6,20305:up|Gjb5,14622:down|Anxa8,11752:down|Aspn,66695:down|Mt2,17750:up|Cxcr6,80901:down|Fam132b,227358:down|Nabp1,109019:down|Mmp2,17390:down|Slc40a1,53945:up|Egfr,13649:down|Alpl,11647:up|C1rb,667277:up|Tfrc,22042:up|Sncg,20618:up|Ccl8,20307:up|Serpina3g,20715:up|Rspo2,239405:up|Fam20a,208659:down|Cdsn,386463:up|Syt13,80976:up|Mt1,17748:up|Col5a2,12832:down|Aldh1a2,19378:up|Ccl2,20296:up|Ednrb,13618:up|Ampd3,11717:up|Mmp13,17386:up|Htra3,78558:down|Tnfrsf12a,27279:down|Uchl5,56207:up|Sord,20322:up|Clec4n,56620:up|Shroom4,208431:down|Steap1,70358:down|Enpp3,209558:down|Cdo1,12583:up|Dok2,13449:up|Mamdc2,71738:down|Csrp2,13008:down|Hvcn1,74096:up|Ptx3,19288:up|Ncam1,17967:down|Ccl4,20303:up|Chst11,58250:down|Tmeff1,230157:down|Vegfb,22340:up|Aldh1a3,56847:down|Plpp3,67916:up|Mrc2,17534:down|Lrrc17,74511:down|Lama2,16773:down|Ccl9,20308:up|Ptk7,71461:down|Inhba,16323:down|Bnc2,242509:down|Tfpi2,21789:down|Crlf1,12931:down|C1s1,50908:up|Plod2,26432:down|Vegfa,22339:down|Gfpt2,14584:down|Serpine2,20720:up|Cbr2,12409:up|Nxn,18230:down|Igf2,16002:up|Kdelr3,105785:down|Tmem176a,66058:up|Tnf,21926:up|Col1a2,12843:down|Fxyd1,56188:up|Fgfr1,14182:down|Lpl,16956:up|Sulf1,240725:down|Susd2,71733:up|Cd4,12504:up|Col3a1,12825:down|Tbx15,21384:down|Serping1,12258:up|Emb,13723:up|Uck2,80914:down|Enpp1,18605:down|Clec2d,93694:up|Hpgd,15446:up|C3,12266:up|Enpp5,83965:up|Ankrd1,107765:down|Cx3cr1,13051:down|Cd109,235505:down|Ggt5,23887:up|Loxl2,94352:down|Loxl3,16950:down|Trf,22041:up|Cxcl14,57266:down|Ceacam10,26366:up|Wnt5a,22418:down|Stc1,20855:up|Mark1,226778:up|Col8a1,12837:down|Adipoq,11450:up|Hal,15109:up|Igf1,16000:up|Crabp2,12904:down|Mcoln2,68279:up|Cxcl9,17329:up|Tgfb2,21808:down|F3,14066:up|Clec4e,56619:up|Cthrc1,68588:down|Kng1,16644:up|Prl2c2,18811:up|Efemp1,216616:up|Dpysl3,22240:down|Ninj1,18081:up|Saa3,20210:up|Gdpd1,66569:up|Vdr,22337:down|Pdia5,72599:down|Fabp4,11770:up|Cxcl12,20315:up|Htr2a,15558:down|Slc16a4,229699:down|Smoc2,64074:down|Gas6,14456:up|Col11a1,12814:down|Sec23a,20334:down|Sfrp1,20377:up|C1qtnf3,81799:down|Tgfbr3,21814:up|Fxyd2,11936:up|Serinc2,230779:down|Tll1,21892:down|Ceacam1,26365:up|Sfrp2,20319:up|Pxdn,69675:down|Tnfrsf11b,18383:up|Prkar2b,19088:up|Npnt,114249:up|S100a1,20193:up|Ly9,17085:up|Ccl7,20306:up|Pdzrn3,55983:down|Pdgfc,54635:down|Mgp,17313:up|Figf,14205:up|Sema7a,20361:down|Epas1,13819:up|Slc29a1,63959:up|Marc1,66112:up|Gpm6b,14758:up|Ddah1,69219:down|Postn,50706:down|Npl,74091:up|Man2a2,140481:up|Akr1c18,105349:up|Fabp5,16592:up|Ccl12,20293:up|Bmpr1a,12166:down|Wisp1,22402:down|Ank,11732:down|Tnc,21923:down|Gatm,67092:down|Ret,19713:up|Prg4,96875:down|Sdf2l1,64136:up|Pdxk,216134:up|C1s2,317677:up|Creb3l1,26427:down|Il1rl1,17082:down|Kitl,17311:up|Fam101b,76566:down|P4ha1,18451:down|Hp,15439:up|Aoah,27052:up|Rasl11a,68895:up|Plat,18791:down|Ccl3,20302:up|Ctla2a,13024:down|Srpx2,68792:down|Prelp,116847:up|Adamts12,239337:down|Lrrc15,74488:down|Anpep,16790:up|Pparg,19016:up|Glp1r,14652:down|Lmcd1,30937:up|Tnfrsf23,79201:down|Has2,15117:down|Hmox1,15368:up|Ptgs2,19225:down|Kcnj15,16516:down|Grem1,23892:up|Pcdh19,279653:down|Col5a1,12831:down|Mcoln3,171166:up|Errfi1,74155:down|Tnfsf9,21950:up

branching involved in prostate gland morphogenesis	GO:0060442	1.22E-04	1.28E-03	Sfrp1,20377:up|Fgfr2,14183:up|Igf1,16000:up|Bmp4,12159:up
convergent extension	GO:0060026	1.22E-04	1.28E-03	Sfrp2,20319:up|Sfrp1,20377:up|Ptk7,71461:down|Wnt5a,22418:down
positive regulation of striated muscle cell differentiation	GO:0051155	1.23E-04	1.29E-03	Ccl8,20307:up|Cxcl9,17329:up|Cxcl12,20315:up|Wisp1,22402:down|Grem1,23892:up|Igf1,16000:up|Bmp4,12159:up
extrinsic apoptotic signaling pathway	GO:0097191	1.24E-04	1.30E-03	Tnf,21926:up|Tnfrsf23,79201:down|Fgfr1,14182:down|Sfrp1,20377:up|Hmox1,15368:up|Inhba,16323:down|Igf1,16000:up|Bmp4,12159:up|Tnfrsf12a,27279:down|Sfrp2,20319:up|Tgfb2,21808:down|Cx3cr1,13051:down|Kitl,17311:up
regulation of myeloid leukocyte differentiation	GO:0002761	1.28E-04	1.34E-03	Fstl3,83554:down|Tnf,21926:up|Clec2d,93694:up|Lrrc17,74511:down|Sfrp1,20377:up|Ccl3,20302:up|Adipoq,11450:up|Ceacam1,26365:up|Kitl,17311:up
macrophage chemotaxis	GO:0048246	1.30E-04	1.35E-03	Ccl2,20296:up|Ccl12,20293:up|Cx3cr1,13051:down|Ednrb,13618:up|Ccl3,20302:up
regulation of vascular endothelial growth factor production	GO:0010574	1.30E-04	1.35E-03	Sulf1,240725:down|Ccl2,20296:up|Hpse,15442:up|C3,12266:up|Ptgs2,19225:down
midbrain development	GO:0030901	1.31E-04	1.35E-03	Uchl5,56207:up|Sfrp2,20319:up|Fgfr1,14182:down|Sfrp1,20377:up|Wnt5a,22418:down|Fgfr2,14183:up
positive regulation of organ growth	GO:0046622	1.31E-04	1.35E-03	Fgfr1,14182:down|Ncam1,17967:down|Bmpr1a,12166:down|Wisp1,22402:down|Fgfr2,14183:up|Igf1,16000:up
regulation of endocytosis	GO:0030100	1.32E-04	1.37E-03	Tnf,21926:up|Pparg,19016:up|Gas6,14456:up|Trf,22041:up|Ptx3,19288:up|Wnt5a,22418:down|Sfrp4,20379:up|Ccl2,20296:up|C3,12266:up|Grem1,23892:up|Adipoq,11450:up|Vegfa,22339:down
embryonic appendage morphogenesis	GO:0035113	1.33E-04	1.37E-03	Fgfr1,14182:down|Wnt5a,22418:down|Bmpr1a,12166:down|Aldh1a2,19378:up|Grem1,23892:up|Bmp4,12159:up|Chst11,58250:down|Sfrp2,20319:up|Crabp2,12904:down|Rspo2,239405:up
BMP signaling pathway	GO:0030509	1.33E-04	1.37E-03	Fstl3,83554:down|Sfrp1,20377:up|Sulf1,240725:down|Wnt5a,22418:down|Bmpr1a,12166:down|Tgfbr3,21814:up|Grem1,23892:up|Bmp4,12159:up|Htra3,78558:down|Sfrp2,20319:up
kidney epithelium development	GO:0072073	1.33E-04	1.37E-03	Fgfr1,14182:down|Sfrp1,20377:up|Grem1,23892:up|Bmp4,12159:up|Adipoq,11450:up|Crlf1,12931:down|Ret,19713:up|Vegfa,22339:down|Npnt,114249:up|Fgfr2,14183:up
embryonic limb morphogenesis	GO:0030326	1.33E-04	1.37E-03	Fgfr1,14182:down|Wnt5a,22418:down|Bmpr1a,12166:down|Aldh1a2,19378:up|Grem1,23892:up|Bmp4,12159:up|Chst11,58250:down|Sfrp2,20319:up|Crabp2,12904:down|Rspo2,239405:up
positive regulation of muscle cell differentiation	GO:0051149	1.37E-04	1.40E-03	Wisp1,22402:down|Grem1,23892:up|Igf1,16000:up|Bmp4,12159:up|Ccl8,20307:up|Cxcl9,17329:up|Cxcl12,20315:up|Igfbp3,16009:up
muscle cell migration	GO:0014812	1.49E-04	1.52E-03	Has2,15117:down|Bmpr1a,12166:down|Plat,18791:down|Igf1,16000:up|Adipoq,11450:up|Postn,50706:down|Igfbp3,16009:up|F3,14066:up
blood vessel remodeling	GO:0001974	1.49E-04	1.52E-03	Ceacam1,26365:up|Vegfa,22339:down|Tgfb2,21808:down|Tgfbr3,21814:up|Igf1,16000:up|Epas1,13819:up
response to corticosteroid	GO:0031960	1.50E-04	1.53E-03	Tnf,21926:up|Akr1c18,105349:up|Alpl,11647:up|Il1rn,16181:up|Pappa,18491:down|Cdo1,12583:up|Bmp4,12159:up
positive regulation of behavior	GO:0048520	1.51E-04	1.54E-03	Gas6,14456:up|Cxcl14,57266:down|Fgfr1,14182:down|Wnt5a,22418:down|Ccl2,20296:up|Figf,14205:up|Ccl4,20303:up|Cxcl9,17329:up|Cxcl12,20315:up|Vegfa,22339:down
oxoacid metabolic process	GO:0043436	1.53E-04	1.55E-03	P4ha1,18451:down|Hpgd,15446:up|Aldh1a2,19378:up|C3,12266:up|Marc1,66112:up|Ddah1,69219:down|Fabp4,11770:up|Fam132b,227358:down|Htr2a,15558:down|Gfpt2,14584:down|Npl,74091:up|Cdo1,12583:up|Mamdc2,71738:down|Pparg,19016:up|Ggt5,23887:up|Akr1c18,105349:up|Lpl,16956:up|Sulf1,240725:down|Ptgs2,19225:down|Serinc2,230779:down|Adipoq,11450:up|Hal,15109:up|Igf1,16000:up|Chst11,58250:down|Crabp2,12904:down|Gatm,67092:down|Hpse,15442:up|Aldh1a3,56847:down|Prkar2b,19088:up|Enpp1,18605:down
negative regulation of peptidase activity	GO:0010466	1.54E-04	1.56E-03	Kng1,16644:up|Gas6,14456:up|Slpi,20568:up|Cst7,13011:up|Tfpi2,21789:down|Igf1,16000:up|Sfrp2,20319:up|Serping1,12258:up|Serpina3g,20715:up|Vegfa,22339:down|Serpine2,20720:up|Cd109,235505:down
vascular endothelial growth factor production	GO:0010573	1.55E-04	1.57E-03	Sulf1,240725:down|Ccl2,20296:up|Hpse,15442:up|C3,12266:up|Ptgs2,19225:down
cytokine secretion	GO:0050663	1.58E-04	1.60E-03	Htr2b,15559:down|Tnf,21926:up|Gas6,14456:up|Lpl,16956:up|C1qtnf3,81799:down|Wnt5a,22418:down|Ccl3,20302:up|Clec4n,56620:up|Postn,50706:down|Clec4e,56619:up|Il1rl1,17082:down
regulation of lymphocyte chemotaxis	GO:1901623	1.60E-04	1.62E-03	Cxcl14,57266:down|Wnt5a,22418:down|Ccl12,20293:up|Ccl4,20303:up
outflow tract morphogenesis	GO:0003151	1.66E-04	1.67E-03	Sfrp2,20319:up|Plxnd1,67784:up|Wnt5a,22418:down|Tgfb2,21808:down|Bmpr1a,12166:down|Fgfr2,14183:up|Bmp4,12159:up
iron ion homeostasis	GO:0055072	1.66E-04	1.67E-03	Trf,22041:up|Fam132b,227358:down|Hmox1,15368:up|Steap1,70358:down|Tfrc,22042:up|Slc40a1,53945:up|Epas1,13819:up
reactive nitrogen species metabolic process	GO:2001057	1.66E-04	1.67E-03	Tnf,21926:up|Ptx3,19288:up|Ddah1,69219:down|Egfr,13649:down|Ptgs2,19225:down|Marc1,66112:up|Igf1,16000:up
regulation of epidermal cell differentiation	GO:0045604	1.69E-04	1.69E-03	Vdr,22337:down|Tgfb2,21808:down|Sfrp4,20379:up|Cd109,235505:down|Errfi1,74155:down|Bmp4,12159:up
myeloid cell homeostasis	GO:0002262	1.71E-04	1.71E-03	Cd24a,12484:down|Hmox1,15368:up|Inhba,16323:down|Tgfbr3,21814:up|Ccl2,20296:up|Ampd3,11717:up|Epas1,13819:up|Bmp4,12159:up|Vegfa,22339:down|Kitl,17311:up
positive regulation of neuron projection development	GO:0010976	1.73E-04	1.72E-03	Fgfr1,14182:down|Cd24a,12484:down|Ptk7,71461:down|Wnt5a,22418:down|Dpysl3,22240:down|Sema7a,20361:down|Bmp4,12159:up|Ankrd1,107765:down|Tnfrsf12a,27279:down|Crabp2,12904:down|Ret,19713:up|Cxcl12,20315:up|Vegfa,22339:down|Serpine2,20720:up
epithelial cell differentiation	GO:0030855	1.73E-04	1.72E-03	Ptk7,71461:down|Sfrp4,20379:up|Ednrb,13618:up|Vdr,22337:down|Vegfa,22339:down|Cd109,235505:down|Pparg,19016:up|Tnf,21926:up|Fgfr1,14182:down|Wnt5a,22418:down|Stc1,20855:up|Ptgs2,19225:down|Grem1,23892:up|Adipoq,11450:up|Bmp4,12159:up|Igf1,16000:up|Ceacam1,26365:up|Mcoln3,171166:up|Tgfb2,21808:down|Errfi1,74155:down|Fgfr2,14183:up|Kitl,17311:up
calcium ion homeostasis	GO:0055074	1.73E-04	1.72E-03	Htr2b,15559:down|Kng1,16644:up|Adm,11535:up|Ccl2,20296:up|Ednrb,13618:up|Ccl3,20302:up|Vdr,22337:down|Htr2a,15558:down|Fxyd1,56188:up|Glp1r,14652:down|Cd24a,12484:down|Cd4,12504:up|Stc1,20855:up|Bmp4,12159:up|Tgfb2,21808:down|Fam20a,208659:down|Ptgir,19222:up

negative regulation of cell development	GO:0010721	1.76E-04	1.75E-03	Fam101b,76566:down|Efemp1,216616:up|Dpysl3,22240:down|Ednrb,13618:up|Postn,50706:down|Plk2,20620:down|Cd24a,12484:down|Sfrp1,20377:up|Tgfbr3,21814:up|Bmpr1a,12166:down|Wnt5a,22418:down|Bmp4,12159:up|Igf1,16000:up|Col3a1,12825:down|Sfrp2,20319:up|Tgfb2,21808:down
negative regulation of response to wounding	GO:1903035	1.77E-04	1.75E-03	Pparg,19016:up|Kng1,16644:up|C1qtnf3,81799:down|Aoah,27052:up|Adipoq,11450:up|Ceacam1,26365:up|Ctla2a,13024:down|Serping1,12258:up|Cx3cr1,13051:down|Serpine2,20720:up|Cd109,235505:down
epidermis development	GO:0008544	1.80E-04	1.78E-03	Tnf,21926:up|Fgfr1,14182:down|Egfr,13649:down|Inhba,16323:down|Sfrp4,20379:up|Ptgs2,19225:down|Bmp4,12159:up|Vdr,22337:down|Mcoln3,171166:up|Tgfb2,21808:down|Hpse,15442:up|Cd109,235505:down|Errfi1,74155:down|Fgfr2,14183:up
developmental maturation	GO:0021700	1.80E-04	1.78E-03	Fam101b,76566:down|Pparg,19016:up|Fgfr1,14182:down|Aldh1a2,19378:up|Ptgs2,19225:down|Ednrb,13618:up|Grem1,23892:up|Igf1,16000:up|Epas1,13819:up|Lyl1,17095:up|Ret,19713:up|Vegfa,22339:down|Tgfb2,21808:down|Mmp2,17390:down
regulation of metal ion transport	GO:0010959	1.83E-04	1.80E-03	Vdr,22337:down|Cxcl12,20315:up|Htr2a,15558:down|Serpine2,20720:up|Fxyd1,56188:up|Glp1r,14652:down|Cd4,12504:up|Ccl2,20293:up|Stc1,20855:up|Ptgs2,19225:down|Fxyd2,11936:up|Ccl4,20303:up|Bmp4,12159:up|Igf1,16000:up|Tgfb2,21808:down|S100a1,20193:up
negative regulation of viral life cycle	GO:1903901	1.83E-04	1.80E-03	Ceacam1,26365:up|Tnf,21926:up|Ptx3,19288:up|Oasl1,231655:up|Slpi,20568:up|Oas3,246727:up|Ccl4,20303:up
blood coagulation	GO:0007596	1.86E-04	1.83E-03	Kng1,16644:up|Gas6,14456:up|C3,12266:up|Pdgfc,54635:down|Anxa8,11752:down|Tfpi2,21789:down|Ceacam1,26365:up|Serping1,12258:up|Hpse,15442:up|Serpine2,20720:up|F3,14066:up
organic acid metabolic process	GO:0006082	1.89E-04	1.85E-03	P4ha1,18451:down|Hpgd,15446:up|Aldh1a2,19378:up|C3,12266:up|Marc1,66112:up|Ddah1,69219:down|Fabp4,11770:up|Fam132b,227358:down|Htr2a,15558:down|Gfpt2,14584:down|Npl,74091:up|Cdo1,12583:up|Mamdc2,71738:down|Pparg,19016:up|Ggt5,23887:up|Akr1c18,105349:up|Lpl,16956:up|Sulf1,240725:down|Ptgs2,19225:down|Serinc2,230779:down|Adipoq,11450:up|Hal,15109:up|Igf1,16000:up|Chst11,58250:down|Crabp2,12904:down|Gatm,67092:down|Hpse,15442:up|Aldh1a3,56847:down|Prkar2b,19088:up|Enpp1,18605:down
platelet-derived growth factor receptor signaling pathway	GO:0048008	1.90E-04	1.86E-03	Vegfa,22339:down|Pdgfc,54635:down|Ptgir,19222:up|Plat,18791:down|F3,14066:up|Adipoq,11450:up
prostate glandular acinus morphogenesis	GO:0060526	1.94E-04	1.88E-03	Sfrp1,20377:up|Fgfr2,14183:up|Igf1,16000:up
regulation of branching involved in prostate gland morphogenesis	GO:0060687	1.94E-04	1.88E-03	Sfrp1,20377:up|Fgfr2,14183:up|Bmp4,12159:up
prostate epithelial cord arborization involved in prostate glandular acinus morphogenesis	GO:0060527	1.94E-04	1.88E-03	Sfrp1,20377:up|Fgfr2,14183:up|Igf1,16000:up
regulation of mesodermal cell fate specification	GO:0042661	1.94E-04	1.88E-03	Sfrp2,20319:up|Fgfr1,14182:down|Bmpr1a,12166:down
negative regulation of gastrulation	GO:2000542	1.94E-04	1.88E-03	Col5a2,12832:down|Sfrp2,20319:up|Col5a1,12831:down
inner ear development	GO:0048839	1.97E-04	1.90E-03	Cthrc1,68588:down|Cxcl14,57266:down|Fgfr1,14182:down|Col11a1,12814:down|Ptk7,71461:down|Wnt5a,22418:down|Igf1,16000:up|Bmp4,12159:up|Mcoln3,171166:up|Aldh1a3,56847:down|Fgfr2,14183:up
regulation of bone mineralization	GO:0030500	2.01E-04	1.94E-03	Fam101b,76566:down|Ank,11732:down|Gpm6b,14758:up|Bmpr1a,12166:down|Mgp,17313:up|Grem1,23892:up|Bmp4,12159:up
neuron projection development	GO:0031175	2.02E-04	1.95E-03	Adm,11535:up|Flrt2,399558:down|Lama2,16773:down|Efemp1,216616:up|Plxnd1,67784:up|Ptk7,71461:down|Dpysl3,22240:down|Sema7a,20361:down|Ankrd1,107765:down|Tnfrsf12a,27279:down|Cxcl12,20315:up|Postn,50706:down|Vegfa,22339:down|Mmp2,17390:down|Serpine2,20720:up|Plk2,20620:down|Cd24a,12484:down|Egfr,13649:down|Fgfr1,14182:down|Ncam1,17967:down|Sfrp1,20377:up|Wnt5a,22418:down|Bmp4,12159:up|Sfrp2,20319:up|Crabp2,12904:down|Tnc,21923:down|Ret,19713:up|Tgfb2,21808:down|Fgfr2,14183:up
regulation of homeostatic process	GO:0032844	2.03E-04	1.95E-03	Htr2b,15559:down|Inhba,16323:down|Ednrb,13618:up|Vegfa,22339:down|Htr2a,15558:down|Anpep,16790:up|Tnf,21926:up|Trf,22041:up|Glp1r,14652:down|Cd24a,12484:down|Egfr,13649:down|Cd4,12504:up|Ptgs2,19225:down|Tfrc,22042:up|Bmp4,12159:up|Ceacam1,26365:up|Tgfb2,21808:down|Tnfrsf11b,18383:up|Kitl,17311:up
regulation of neurogenesis	GO:0050767	2.04E-04	1.96E-03	Prl2c2,18811:up|Flrt2,399558:down|Efemp1,216616:up|Plxnd1,67784:up|Ptk7,71461:down|Dpysl3,22240:down|Ednrb,13618:up|Sema7a,20361:down|Ankrd1,107765:down|Tnfrsf12a,27279:down|Cxcl12,20315:up|Vegfa,22339:down|Cx3cr1,13051:down|Serpine2,20720:up|Pparg,19016:up|Plk2,20620:down|Trf,22041:up|Fgfr1,14182:down|Cd24a,12484:down|Sfrp1,20377:up|Wnt5a,22418:down|Bmpr1a,12166:down|Bmp4,12159:up|Col3a1,12825:down|Sfrp2,20319:up|Crabp2,12904:down|Ret,19713:up
formation of primary germ layer	GO:0001704	2.05E-04	1.96E-03	Col5a2,12832:down|Fgfr1,14182:down|Wnt5a,22418:down|Inhba,16323:down|Bmpr1a,12166:down|Bmp4,12159:up|Sfrp2,20319:up|Col5a1,12831:down
morphogenesis of an epithelial bud	GO:0060572	2.06E-04	1.96E-03	Sulf1,240725:down|Wnt5a,22418:down|Fgfr2,14183:up|Bmp4,12159:up
response to iron ion	GO:0010039	2.06E-04	1.96E-03	Trf,22041:up|Hmox1,15368:up|Tfrc,22042:up|Slc40a1,53945:up
regulation of hair follicle development	GO:0051797	2.06E-04	1.96E-03	Tnf,21926:up|Tgfb2,21808:down|Inhba,16323:down|Hpse,15442:up
mesodermal cell fate commitment	GO:0001710	2.06E-04	1.96E-03	Sfrp2,20319:up|Fgfr1,14182:down|Bmpr1a,12166:down|Bmp4,12159:up
retinoic acid metabolic process	GO:0042573	2.06E-04	1.96E-03	Crabp2,12904:down|Akr1c18,105349:up|Aldh1a2,19378:up|Aldh1a3,56847:down
hemostasis	GO:0007599	2.07E-04	1.97E-03	Kng1,16644:up|Gas6,14456:up|C3,12266:up|Pdgfc,54635:down|Anxa8,11752:down|Tfpi2,21789:down|Ceacam1,26365:up|Serping1,12258:up|Hpse,15442:up|Serpine2,20720:up|F3,14066:up
sensory organ development	GO:0007423	2.09E-04	1.98E-03	Cthrc1,68588:down|Col5a2,12832:down|Ptk7,71461:down|Inhba,16323:down|Aldh1a2,19378:up|Bnc2,242509:down|Ninj1,18081:up|Vegfa,22339:down|Cxcl14,57266:down|Col11a1,12814:down|Egfr,13649:down|Fgfr1,14182:down|Wnt5a,22418:down|Col8a1,12837:down|Bmp4,12159:up|Igf1,16000:up|Col5a1,12831:down|Mcoln3,171166:up|Tgfb2,21808:down|Aldh1a3,56847:down|Fgfr2,14183:up
regulation of acute inflammatory response	GO:0002673	2.14E-04	2.02E-03	Tnf,21926:up|Pparg,19016:up|Serping1,12258:up|C3,12266:up|Ptgs2,19225:down|Ednrb,13618:up

positive regulation of phagocytosis	GO:0050766	2.14E-04	2.02E-03	Tnf,21926:up|Pparg,19016:up|Gas6,14456:up|Ptx3,19288:up|Ccl2,20296:up|C3,12266:up
cellular response to drug	GO:0035690	2.14E-04	2.02E-03	Ankrd1,107765:down|Gas6,14456:up|Gstm1,14862:up|Tfrc,22042:up|Ccl4,20303:up|Adipoq,11450:up
protein kinase B signaling	GO:0043491	2.17E-04	2.04E-03	Tnf,21926:up|Gas6,14456:up|Akr1c18,105349:up|Egfr,13649:down|C1qtnf3,81799:down|Ccl12,20293:up|Igf1,16000:up|Hpse,15442:up|F3,14066:up|Igf2,16002:up
coagulation	GO:0050817	2.19E-04	2.05E-03	Kng1,16644:up|Gas6,14456:up|C3,12266:up|Pdgfc,54635:down|Anxa8,11752:down|Tfpi2,21789:down|Ceacam1,26365:up|Serping1,12258:up|Hpse,15442:up|Serpine2,20720:up|F3,14066:up
regulation of smooth muscle cell migration	GO:0014910	2.21E-04	2.07E-03	Has2,15117:down|Postn,50706:down|Bmpr1a,12166:down|Igfbp3,16009:up|F3,14066:up|Igf1,16000:up|Adipoq,11450:up
hair cycle process	GO:0022405	2.22E-04	2.07E-03	Tnf,21926:up|Egfr,13649:down|Inhba,16323:down|Ptgs2,19225:down|Hpse,15442:up|Tgfb2,21808:down|Fgfr2,14183:up|Cd109,235505:down
molting cycle process	GO:0022404	2.22E-04	2.07E-03	Tnf,21926:up|Egfr,13649:down|Inhba,16323:down|Ptgs2,19225:down|Hpse,15442:up|Tgfb2,21808:down|Fgfr2,14183:up|Cd109,235505:down
hair follicle development	GO:0001942	2.22E-04	2.07E-03	Tnf,21926:up|Egfr,13649:down|Inhba,16323:down|Ptgs2,19225:down|Hpse,15442:up|Tgfb2,21808:down|Fgfr2,14183:up|Cd109,235505:down
negative regulation of hemopoiesis	GO:1903707	2.24E-04	2.08E-03	Fstl3,83554:down|Tmem176a,66058:up|Clec2d,93694:up|Lrrc17,74511:down|Tmem176b,65963:up|Sfrp1,20377:up|Bmp4,12159:up|Adipoq,11450:up|Ceacam1,26365:up
positive regulation of cell projection organization	GO:0031346	2.27E-04	2.11E-03	Prl2c2,18811:up|Efemp1,216616:up|Ptk7,71461:down|Dpysl3,22240:down|Sema7a,20361:down|Ankrd1,107765:down|Tnfrsf12a,27279:down|Cxcl12,20315:up|Vegfa,22339:down|Serpine2,20720:up|Cd24a,12484:down|Fgfr1,14182:down|Wnt5a,22418:down|Bmp4,12159:up|Crabp2,12904:down|Ret,19713:up
positive regulation of homeostatic process	GO:0032846	2.28E-04	2.11E-03	Tnf,21926:up|Trf,22041:up|Egfr,13649:down|Cd24a,12484:down|Inhba,16323:down|Cd4,12504:up|Ptgs2,19225:down|Tfrc,22042:up|Ednrb,13618:up|Bmp4,12159:up|Ceacam1,26365:up|Tgfb2,21808:down
single-organism cellular process	GO:0044763	2.28E-04	2.11E-03	Fstl3,83554:down|Igfbp4,16010:up|Adm,11535:up|Flrt2,399558:down|Tmem176b,65963:up|Plxnd1,67784:up|Sfrp4,20379:up|Ltbp2,16997:down|Serpinh1,12406:down|Lyl1,17095:up|Gstt1,14871:up|Blvrb,233016:up|Igfbp3,16009:up|Ibsp,15891:up|Plk2,20620:down|Cd24a,12484:down|Mme,17380:up|Il1rn,16181:up|Maob,109731:up|Bmp4,12159:up|Hpse,15442:up|Medag,70717:down|Ptgir,19222:up|Fgfr2,14183:up|Thbs2,21826:down|Htr2b,15559:down|Dram1,71712:up|Ccl6,20305:up|Gjb5,14622:down|Aspn,66695:down|Mt2,17750:up|Cxcr6,80901:down|Fam132b,227358:down|Nabp1,109019:down|Mmp2,17390:down|Slc40a1,53945:up|Egfr,13649:down|Alpl,11647:up|Tfrc,22042:up|Sncg,20618:up|Ccl8,20307:up|Serpina3g,20715:up|Rspo2,239405:up|Syt13,80976:up|Mt1,17748:up|Col5a2,12832:down|Aldh1a2,19378:up|Ccl2,20296:up|Ednrb,13618:up|Ampd3,11717:up|Mmp13,17386:up|Htra3,78558:down|Tnfrsf12a,27279:down|Uchl5,56207:up|Sord,20322:up|Clec4n,56620:up|Shroom4,208431:down|Enpp3,209558:down|Cdo1,12583:up|Dok2,13449:up|Mamdc2,71738:down|Csrp2,13008:down|Hvcn1,74096:up|Ncam1,17967:down|Ccl4,20303:up|Chst11,58250:down|Vegfb,22340:up|Aldh1a3,56847:down|Plpp3,67916:up|Mrc2,17534:down|Lrrc17,74511:down|Lama2,16773:down|Ccl9,20308:up|Ptk7,71461:down|Inhba,16323:down|Crlf1,12931:down|Plod2,26432:down|Vegfa,22339:down|Gfpt2,14584:down|Serpine2,20720:up|Cbr2,12409:up|Nxn,18230:down|Igf2,16002:up|Kdelr3,105785:down|Tmem176a,66058:up|Tnf,21926:up|Col1a2,12843:down|Fxyd1,56188:up|Fgfr1,14182:down|Lpl,16956:up|Sulf1,240725:down|Susd2,71733:up|Cd4,12504:up|Col3a1,12825:down|Emb,13723:up|Uck2,80914:down|Enpp1,18605:down|Clec2d,93694:up|Hpgd,15446:up|C3,12266:up|Enpp5,83965:up|Ankrd1,107765:down|Cx3cr1,13051:down|Cd109,235505:down|Ggt5,23887:up|Loxl2,94352:down|Loxl3,16950:down|Trf,22041:up|Cxcl14,57266:down|Ceacam10,26366:up|Wnt5a,22418:down|Stc1,20855:up|Mark1,226778:up|Adipoq,11450:up|Hal,15109:up|Igf1,16000:up|Crabp2,12904:down|Cxcl9,17329:up|Tgfb2,21808:down|F3,14066:up|Clec4e,56619:up|Cthrc1,68588:down|Kng1,16644:up|Prl2c2,18811:up|Efemp1,216616:up|Dpysl3,22240:down|Saa3,20210:up|Vdr,22337:down|Pdia5,72599:down|Fabp4,11770:up|Cxcl12,20315:up|Htr2a,15558:down|Slc16a4,229699:down|Smoc2,64074:down|Gas6,14456:up|Col11a1,12814:down|Sfrp1,20377:up|C1qtnf3,81799:down|Tgfbr3,21814:up|Fxyd2,11936:up|Serinc2,230779:down|Tll1,21892:down|Ceacam1,26365:up|Sfrp2,20319:up|Pxdn,69675:down|Tnfrsf11b,18383:up|Prkar2b,19088:up|Npnt,114249:up|S100a1,20193:up|Ly9,17085:up|Ccl7,20306:up|Pdzrn3,55983:down|Pdgfc,54635:down|Mgp,17313:up|Figf,14205:up|Sema7a,20361:down|Epas1,13819:up|Slc29a1,63959:up|Marc1,66112:up|Gpm6b,14758:up|Ddah1,69219:down|Postn,50706:down|Npl,74091:up|Man2a2,140481:up|Akr1c18,105349:up|Fabp5,16592:up|Ccl12,20293:up|Bmpr1a,12166:down|Wisp1,22402:down|Ank,11732:down|Tnc,21923:down|Gatm,67092:down|Ret,19713:up|Sdf2l1,64136:up|Pdxk,216134:up|Creb3l1,26427:down|Il1rl1,17082:down|Kitl,17311:up|Fam101b,76566:down|P4ha1,18451:down|Hp,15439:up|Aoah,27052:up|Rasl11a,68895:up|Plat,18791:down|Ccl3,20302:up|Ctla2a,13024:down|Srpx2,68792:down|Prelp,116847:up|Adamts12,239337:down|Lrrc15,74488:down|Anpep,16790:up|Pparg,19016:up|Glp1r,14652:down|Lmcd1,30937:up|Tnfrsf23,79201:down|Has2,15117:down|Hmox1,15368:up|Ptgs2,19225:down|Kcnj15,16516:down|Grem1,23892:up|Mcoln3,171166:up|Col5a1,12831:down|Errfi1,74155:down|Tnfsf9,21950:up
ion transport	GO:0006811	2.29E-04	2.12E-03	Sfrp4,20379:up|Gpm6b,14758:up|Steap1,70358:down|Hvcn1,74096:up|Trf,22041:up|Ccl12,20293:up|Stc1,20855:up|Il1rn,16181:up|Ccl4,20303:up|Maob,109731:up|Bmp4,12159:up|Igf1,16000:up|Ank,11732:down|Mcoln2,68279:up|Tgfb2,21808:down|Htr2b,15559:down|Vdr,22337:down|Fam132b,227358:down|Cxcl12,20315:up|Htr2a,15558:down|Serpine2,20720:up|Slc16a4,229699:down|Slc40a1,53945:up|Pparg,19016:up|Gas6,14456:up|Tnf,21926:up|Fxyd1,56188:up|Glp1r,14652:down|Fgfr1,14182:down|Cd4,12504:up|Ptgs2,19225:down|Tfrc,22042:up|Sncg,20618:up|Fxyd2,11936:up|Kcnj15,16516:down|Serinc2,230779:down|Mcoln3,171166:up|Emb,13723:up|Enpp1,18605:down|S100a1,20193:up
embryonic organ development	GO:0048568	2.36E-04	2.18E-03	Cthrc1,68588:down|Adm,11535:up|Ptk7,71461:down|Gjb5,14622:down|Aldh1a2,19378:up|Epas1,13819:up|Vegfa,22339:down|Col11a1,12814:down|Egfr,13649:down|Fgfr1,14182:down|Bmpr1a,12166:down|Wnt5a,22418:down|Bmp4,12159:up|Chst11,58250:down|Tbx15,21384:down|Tgfb2,21808:down|Aldh1a3,56847:down|Fgfr2,14183:up|Kitl,17311:up
regulation of protein localization to nucleus	GO:1900180	2.39E-04	2.20E-03	Tnf,21926:up|Gas6,14456:up|Egfr,13649:down|C1qtnf3,81799:down|Bmpr1a,12166:down|Ptgs2,19225:down|Grem1,23892:up|Igf1,16000:up|Bmp4,12159:up|Ceacam1,26365:up|Sfrp2,20319:up|Tgfb2,21808:down
regulation of protein kinase B signaling	GO:0051896	2.39E-04	2.20E-03	Tnf,21926:up|Gas6,14456:up|Akr1c18,105349:up|Egfr,13649:down|C1qtnf3,81799:down|Igf1,16000:up|Hpse,15442:up|F3,14066:up|Igf2,16002:up
non-canonical Wnt signaling pathway	GO:0035567	2.40E-04	2.21E-03	Cthrc1,68588:down|Sfrp2,20319:up|Sfrp1,20377:up|Ptk7,71461:down|Wnt5a,22418:down|Sfrp4,20379:up
cardiac muscle cell proliferation	GO:0060038	2.40E-04	2.21E-03	Fgfr1,14182:down|Ncam1,17967:down|Tgfb2,21808:down|Bmpr1a,12166:down|Tgfbr3,21814:up|Fgfr2,14183:up

<table>
<tr><td>positive regulation of macromolecule metabolic process</td><td>GO:0010604</td><td>2.47E-04</td><td>2.26E-03</td><td>Fstl3,83554:down|Igfbp4,16010:up|Sfrp4,20379:up|C3,12266:up|Lyl1,17095:up|Igfbp3,16009:up|Plk2,20620:down|Trf,22041:up|Cd24a,12484:down|Wnt5a,22418:down|Il1rn,16181:up|Adipoq,11450:up|Bmp4,12159:up|Igf1,16000:up|Tgfb2,21808:down|Fgfr2,14183:up|F3,14066:up|Htr2b,15559:down|Prl2c2,18811:up|Ccl6,20305:up|Vdr,22337:down|Htr2a,15558:down|Slc40a1,53945:up|Gas6,14456:up|Egfr,13649:down|Sfrp1,20377:up|C1qtnf3,81799:down|Tgfbr3,21814:up|Tfrc,22042:up|Ceacam1,26365:up|Ccl8,20307:up|Sfrp2,20319:up|Fam20a,208659:down|Npnt,114249:up|Tnfrsf11b,18383:up|Ice1,218333:down|Aldh1a2,19378:up|Ccl2,20296:up|Ccl7,20306:up|Ednrb,13618:up|Pdgfc,54635:down|Epas1,13819:up|Sema7a,20361:down|Sdpr,20324:down|Ccl12,20293:up|Bmpr1a,12166:down|Ccl4,20303:up|Tnc,21923:down|Ret,19713:up|Plpp3,67916:up|Creb3l1,26427:down|Kitl,17311:up|Ccl9,20308:up|Rasl11a,68895:up|Inhba,16323:down|Ccl3,20302:up|Crlf1,12931:down|Vegfa,22339:down|Igf2,16002:up|Pparg,19016:up|Tnf,21926:up|Glp1r,14652:down|Fgfr1,14182:down|Has2,15117:down|Cd4,12504:up|Grem1,23892:up</td></tr>
<tr><td>cellular response to lipid</td><td>GO:0071396</td><td>2.54E-04</td><td>2.32E-03</td><td>Mrc1,17533:up|Ptk7,71461:down|Inhba,16323:down|Ccl2,20296:up|Aldh1a2,19378:up|Ankrd1,107765:down|Vdr,22337:down|Mmp2,17390:down|Pparg,19016:up|Tnf,21926:up|Akr1c18,105349:up|Egfr,13649:down|Sfrp1,20377:up|Ccl12,20293:up|Bmp4,12159:up|Igf1,16000:up|Ret,19713:up|Ptgir,19222:up</td></tr>
<tr><td>skin epidermis development</td><td>GO:0098773</td><td>2.58E-04</td><td>2.35E-03</td><td>Tnf,21926:up|Egfr,13649:down|Inhba,16323:down|Ptgs2,19225:down|Hpse,15442:up|Tgfb2,21808:down|Fgfr2,14183:up|Cd109,235505:down</td></tr>
<tr><td>granulocyte migration</td><td>GO:0097530</td><td>2.58E-04</td><td>2.35E-03</td><td>Ccl9,20308:up|Ccl6,20305:up|Ccl7,20306:up|Ccl2,20296:up|Ccl12,20293:up|Ccl4,20303:up|Ccl3,20302:up|Ccl8,20307:up</td></tr>
<tr><td>response to copper ion</td><td>GO:0046688</td><td>2.62E-04</td><td>2.38E-03</td><td>Sord,20322:up|Loxl2,94352:down|Mt2,17750:up|Mt1,17748:up</td></tr>
<tr><td>negative regulation of biomineral tissue development</td><td>GO:0070168</td><td>2.62E-04</td><td>2.38E-03</td><td>Fam101b,76566:down|Gas6,14456:up|Aspn,66695:down|Grem1,23892:up</td></tr>
<tr><td>regulation of calcium ion transport</td><td>GO:0051924</td><td>2.62E-04</td><td>2.38E-03</td><td>Glp1r,14652:down|Ccl12,20293:up|Cd4,12504:up|Ptgs2,19225:down|Stc1,20855:up|Ccl4,20303:up|Igf1,16000:up|Bmp4,12159:up|Vdr,22337:down|Cxcl12,20315:up|Tgfb2,21808:down|S100a1,20193:up</td></tr>
<tr><td>response to biotic stimulus</td><td>GO:0009607</td><td>2.63E-04</td><td>2.38E-03</td><td>Adamts4,240913:down|Adm,11535:up|Hp,15439:up|Mrc1,17533:up|Ccl2,20296:up|Slpi,20568:up|Ednrb,13618:up|Ifit3,15959:up|Ankrd1,107765:down|Clec4n,56620:up|Oas3,246727:up|Tnf,21926:up|Lmcd1,30937:up|Ptx3,19288:up|Cd24a,12484:down|Wnt5a,22418:down|Cd4,12504:up|Ccl12,20293:up|Ptgs2,19225:down|Ceacam1,26365:up|Cxcl9,17329:up|Oasl1,231655:up|Ifit1,15957:up|Tnfrsf11b,18383:up|Ptgir,19222:up|Clec4e,56619:up</td></tr>
<tr><td>regulation of phagocytosis</td><td>GO:0050764</td><td>2.65E-04</td><td>2.40E-03</td><td>Tnf,21926:up|Pparg,19016:up|Gas6,14456:up|Ptx3,19288:up|Ccl2,20296:up|C3,12266:up|Adipoq,11450:up</td></tr>
<tr><td>negative regulation of defense response</td><td>GO:0031348</td><td>2.73E-04</td><td>2.47E-03</td><td>Pparg,19016:up|Clec2d,93694:up|C1qtnf3,81799:down|Aoah,27052:up|Adipoq,11450:up|Ceacam1,26365:up|Ctla2a,13024:down|Serping1,12258:up|Cx3cr1,13051:down|Igf2,16002:up</td></tr>
<tr><td>negative regulation of phosphorylation</td><td>GO:0042326</td><td>2.78E-04</td><td>2.51E-03</td><td>Flrt2,399558:down|Sfrp4,20379:up|Aspn,66695:down|Fabp4,11770:up|Igfbp3,16009:up|Cd109,235505:down|Lrrc15,74488:down|Sfrp1,20377:up|Grem1,23892:up|Adipoq,11450:up|Bmp4,12159:up|Igf1,16000:up|Ceacam1,26365:up|Sfrp2,20319:up|Prkar2b,19088:up|Plpp3,67916:up|Enpp1,18605:down|Errfi1,74155:down</td></tr>
<tr><td>limb development</td><td>GO:0060173</td><td>2.83E-04</td><td>2.54E-03</td><td>Fgfr1,14182:down|Wnt5a,22418:down|Bmpr1a,12166:down|Aldh1a2,19378:up|Grem1,23892:up|Bmp4,12159:up|Chst11,58250:down|Sfrp2,20319:up|Crabp2,12904:down|Rspo2,239405:up|Fgfr2,14183:up</td></tr>
<tr><td>appendage development</td><td>GO:0048736</td><td>2.83E-04</td><td>2.54E-03</td><td>Fgfr1,14182:down|Wnt5a,22418:down|Bmpr1a,12166:down|Aldh1a2,19378:up|Grem1,23892:up|Bmp4,12159:up|Chst11,58250:down|Sfrp2,20319:up|Crabp2,12904:down|Rspo2,239405:up|Fgfr2,14183:up</td></tr>
<tr><td>response to other organism</td><td>GO:0051707</td><td>2.89E-04</td><td>2.58E-03</td><td>Adamts4,240913:down|Adm,11535:up|Hp,15439:up|Mrc1,17533:up|Ccl2,20296:up|Slpi,20568:up|Ednrb,13618:up|Ifit3,15959:up|Ankrd1,107765:down|Clec4n,56620:up|Oas3,246727:up|Tnf,21926:up|Ptx3,19288:up|Lmcd1,30937:up|Cd24a,12484:down|Wnt5a,22418:down|Cd4,12504:up|Ccl12,20293:up|Ptgs2,19225:down|Cxcl9,17329:up|Oasl1,231655:up|Ifit1,15957:up|Tnfrsf11b,18383:up|Ptgir,19222:up|Clec4e,56619:up</td></tr>
<tr><td>response to external biotic stimulus</td><td>GO:0043207</td><td>2.89E-04</td><td>2.58E-03</td><td>Adamts4,240913:down|Adm,11535:up|Hp,15439:up|Mrc1,17533:up|Ccl2,20296:up|Slpi,20568:up|Ednrb,13618:up|Ifit3,15959:up|Ankrd1,107765:down|Clec4n,56620:up|Oas3,246727:up|Tnf,21926:up|Ptx3,19288:up|Lmcd1,30937:up|Cd24a,12484:down|Wnt5a,22418:down|Cd4,12504:up|Ccl12,20293:up|Ptgs2,19225:down|Cxcl9,17329:up|Oasl1,231655:up|Ifit1,15957:up|Tnfrsf11b,18383:up|Ptgir,19222:up|Clec4e,56619:up</td></tr>
<tr><td>neural crest cell development</td><td>GO:0014032</td><td>2.89E-04</td><td>2.58E-03</td><td>Htr2b,15559:down|Ret,19713:up|Bmpr1a,12166:down|Aldh1a2,19378:up|Ednrb,13618:up|Sema7a,20361:down|Kitl,17311:up</td></tr>
<tr><td>cardiac ventricle development</td><td>GO:0003231</td><td>2.90E-04</td><td>2.58E-03</td><td>Col11a1,12814:down|Ptk7,71461:down|Wnt5a,22418:down|Bmpr1a,12166:down|Tgfbr3,21814:up|Bmp4,12159:up|Sfrp2,20319:up|Tgfb2,21808:down|Fgfr2,14183:up</td></tr>
<tr><td>brain development</td><td>GO:0007420</td><td>2.90E-04</td><td>2.58E-03</td><td>Inhba,16323:down|Aldh1a2,19378:up|Sema7a,20361:down|Uchl5,56207:up|Shroom4,208431:down|Cxcl12,20315:up|Cx3cr1,13051:down|Serpine2,20720:up|Egfr,13649:down|Fgfr1,14182:down|Sfrp1,20377:up|Ncam1,17967:down|Wnt5a,22418:down|Bmpr1a,12166:down|Bmp4,12159:up|Igf1,16000:up|Col3a1,12825:down|Sfrp2,20319:up|Pcdh19,279653:down|Aldh1a3,56847:down|Fgfr2,14183:up</td></tr>
<tr><td>Wnt signaling pathway, planar cell polarity pathway</td><td>GO:0060071</td><td>2.92E-04</td><td>2.59E-03</td><td>Cthrc1,68588:down|Sfrp2,20319:up|Sfrp1,20377:up|Ptk7,71461:down|Wnt5a,22418:down</td></tr>
<tr><td>regulation of establishment of planar polarity</td><td>GO:0090175</td><td>2.92E-04</td><td>2.59E-03</td><td>Cthrc1,68588:down|Sfrp2,20319:up|Sfrp1,20377:up|Ptk7,71461:down|Wnt5a,22418:down</td></tr>
<tr><td>positive regulation of mesenchymal cell proliferation</td><td>GO:0002053</td><td>2.92E-04</td><td>2.59E-03</td><td>Fgfr1,14182:down|Wnt5a,22418:down|Vegfa,22339:down|Bmpr1a,12166:down|Fgfr2,14183:up</td></tr>
<tr><td>detection of mechanical stimulus involved in sensory perception</td><td>GO:0050974</td><td>2.92E-04</td><td>2.59E-03</td><td>Tnf,21926:up|Col11a1,12814:down|Htr2a,15558:down|Serpine2,20720:up|Igf1,16000:up</td></tr>
<tr><td>digestive tract morphogenesis</td><td>GO:0048546</td><td>3.01E-04</td><td>2.65E-03</td><td>Sfrp2,20319:up|Egfr,13649:down|Sfrp1,20377:up|Wnt5a,22418:down|Fgfr2,14183:up|Bmp4,12159:up</td></tr>
<tr><td>regulation of nitric oxide biosynthetic process</td><td>GO:0045428</td><td>3.01E-04</td><td>2.65E-03</td><td>Tnf,21926:up|Ptx3,19288:up|Ddah1,69219:down|Egfr,13649:down|Ptgs2,19225:down|Igf1,16000:up</td></tr>
<tr><td>regulation of cardiac muscle tissue growth</td><td>GO:0055021</td><td>3.01E-04</td><td>2.65E-03</td><td>Fgfr1,14182:down|Ncam1,17967:down|Bmpr1a,12166:down|Wisp1,22402:down|Fgfr2,14183:up|Igf1,16000:up</td></tr>
</table>

positive regulation of cellular component organization	GO:0051130	3.02E-04	2.66E-03	Ice1,218333:down|Flrt2,399558:down|Sfrp4,20379:up|Ccl2,20296:up|C3,12266:up|Sema7a,20361:down|Ankrd1,107765:down|Tnfrsf12a,27279:down|Igfbp3,16009:up|Trf,22041:up|Ptx3,19288:up|Cd24a,12484:down|Wnt5a,22418:down|Bmp4,12159:up|Igf1,16000:up|Crabp2,12904:down|Cxcl9,17329:up|Ret,19713:up|Tgfb2,21808:down|Thbs2,21826:down|Prl2c2,18811:up|Efemp1,216616:up|Ptk7,71461:down|Dpysl3,22240:down|Srpx2,68792:down|Cxcl12,20315:up|Plod2,26432:down|Vegfa,22339:down|Serpine2,20720:up|Igf2,16002:up|Pparg,19016:up|Gas6,14456:up|Tnf,21926:up|Lmcd1,30937:up|Fgfr1,14182:down|Has2,15117:down|Grem1,23892:up|Ccl8,20307:up
mesenchymal-epithelial cell signaling	GO:0060638	3.06E-04	2.67E-03	Tnc,21923:down|Fgfr1,14182:down|Wnt5a,22418:down
positive regulation of cell migration involved in sprouting angiogenesis	GO:0090050	3.06E-04	2.67E-03	Srpx2,68792:down|Vegfa,22339:down|Ptgs2,19225:down
convergent extension involved in axis elongation	GO:0060028	3.06E-04	2.67E-03	Sfrp2,20319:up|Sfrp1,20377:up|Wnt5a,22418:down
membranous septum morphogenesis	GO:0003149	3.06E-04	2.67E-03	Tgfb2,21808:down|Fgfr2,14183:up|Bmp4,12159:up
lung vasculature development	GO:0060426	3.06E-04	2.67E-03	Vegfa,22339:down|Errfi1,74155:down|Igf1,16000:up
single-organism nuclear import	GO:1902593	3.15E-04	2.73E-03	Tnf,21926:up|Gas6,14456:up|Akr1c18,105349:up|Egfr,13649:down|C1qtnf3,81799:down|Bmpr1a,12166:down|Ptgs2,19225:down|Grem1,23892:up|Igf1,16000:up|Bmp4,12159:up|Ceacam1,26365:up|Sfrp2,20319:up|Tgfb2,21808:down
protein import into nucleus	GO:0006606	3.15E-04	2.73E-03	Tnf,21926:up|Gas6,14456:up|Akr1c18,105349:up|Egfr,13649:down|C1qtnf3,81799:down|Bmpr1a,12166:down|Ptgs2,19225:down|Grem1,23892:up|Igf1,16000:up|Bmp4,12159:up|Ceacam1,26365:up|Sfrp2,20319:up|Tgfb2,21808:down
protein targeting to nucleus	GO:0044744	3.15E-04	2.73E-03	Tnf,21926:up|Gas6,14456:up|Akr1c18,105349:up|Egfr,13649:down|C1qtnf3,81799:down|Bmpr1a,12166:down|Ptgs2,19225:down|Grem1,23892:up|Igf1,16000:up|Bmp4,12159:up|Ceacam1,26365:up|Sfrp2,20319:up|Tgfb2,21808:down
signal transduction in absence of ligand	GO:0038034	3.16E-04	2.73E-03	Tnf,21926:up|Fgfr1,14182:down|Tgfb2,21808:down|Inhba,16323:down|Cx3cr1,13051:down|Kitl,17311:up|Igf1,16000:up
extrinsic apoptotic signaling pathway in absence of ligand	GO:0097192	3.16E-04	2.73E-03	Tnf,21926:up|Fgfr1,14182:down|Tgfb2,21808:down|Inhba,16323:down|Cx3cr1,13051:down|Kitl,17311:up|Igf1,16000:up
regulation of chemokine production	GO:0032642	3.16E-04	2.73E-03	Tnf,21926:up|Lpl,16956:up|C1qtnf3,81799:down|Postn,50706:down|Wnt5a,22418:down|Il1rl1,17082:down|Adipoq,11450:up
odontogenesis	GO:0042476	3.21E-04	2.76E-03	Inhba,16323:down|Bmpr1a,12166:down|Aspn,66695:down|Bmp4,12159:up|Rspo2,239405:up|Tnfrsf11b,18383:up|Fam20a,208659:down|Fgfr2,14183:up
leukocyte apoptotic process	GO:0071887	3.21E-04	2.76E-03	Gas6,14456:up|Cd24a,12484:down|Wnt5a,22418:down|Bmp4,12159:up|Ceacam1,26365:up|Cxcl12,20315:up|Tgfb2,21808:down|Kitl,17311:up
response to vitamin K	GO:0032571	3.26E-04	2.76E-03	Gas6,14456:up|Postn,50706:down
negative regulation of odontogenesis of dentin-containing tooth	GO:0042489	3.26E-04	2.76E-03	Rspo2,239405:up|Tnfrsf11b,18383:up
cellular response to vitamin K	GO:0071307	3.26E-04	2.76E-03	Gas6,14456:up|Postn,50706:down
lateral mesodermal cell fate specification	GO:0048377	3.26E-04	2.76E-03	Fgfr1,14182:down|Bmpr1a,12166:down
fibroblast growth factor receptor signaling pathway involved in orbitofrontal cortex development	GO:0035607	3.26E-04	2.76E-03	Fgfr1,14182:down|Fgfr2,14183:up
lateral mesodermal cell fate commitment	GO:0048372	3.26E-04	2.76E-03	Fgfr1,14182:down|Bmpr1a,12166:down
orbitofrontal cortex development	GO:0021769	3.26E-04	2.76E-03	Fgfr1,14182:down|Fgfr2,14183:up
regulation of lateral mesodermal cell fate specification	GO:0048378	3.26E-04	2.76E-03	Fgfr1,14182:down|Bmpr1a,12166:down
positive regulation of activation-induced cell death of T cells	GO:0070237	3.26E-04	2.76E-03	Ceacam1,26365:up|Tgfb2,21808:down
regulation of neuron projection development	GO:0010975	3.26E-04	2.76E-03	Efemp1,216616:up|Plxnd1,67784:up|Ptk7,71461:down|Dpysl3,22240:down|Sema7a,20361:down|Ankrd1,107765:down|Tnfrsf12a,27279:down|Cxcl12,20315:up|Vegfa,22339:down|Serpine2,20720:up|Plk2,20620:down|Cd24a,12484:down|Fgfr1,14182:down|Sfrp1,20377:up|Wnt5a,22418:down|Bmp4,12159:up|Sfrp2,20319:up|Crabp2,12904:down|Ret,19713:up
outflow tract septum morphogenesis	GO:0003148	3.27E-04	2.76E-03	Tgfb2,21808:down|Bmpr1a,12166:down|Fgfr2,14183:up|Bmp4,12159:up
heat generation	GO:0031649	3.27E-04	2.76E-03	Tnf,21926:up|Ptgs2,19225:down|Il1rn,16181:up|Ednrb,13618:up
nuclear import	GO:0051170	3.29E-04	2.77E-03	Tnf,21926:up|Gas6,14456:up|Akr1c18,105349:up|Egfr,13649:down|C1qtnf3,81799:down|Bmpr1a,12166:down|Ptgs2,19225:down|Grem1,23892:up|Igf1,16000:up|Bmp4,12159:up|Ceacam1,26365:up|Sfrp2,20319:up|Tgfb2,21808:down
mesenchymal cell proliferation	GO:0010463	3.35E-04	2.81E-03	Fgfr1,14182:down|Wnt5a,22418:down|Vegfa,22339:down|Bmpr1a,12166:down|Fgfr2,14183:up|Bmp4,12159:up

positive regulation of chemokine production	GO:0032722	3.35E-04	2.81E-03	Tnf,21926:up|Lpl,16956:up|Postn,50706:down|Wnt5a,22418:down|Il1rl1,17082:down|Adipoq,11450:up
epithelial tube branching involved in lung morphogenesis	GO:0060441	3.37E-04	2.83E-03	Tnf,21926:up|Tnc,21923:down|Rspo2,239405:up|Fgfr2,14183:up|Bmp4,12159:up
regulation of renal system process	GO:0098801	3.37E-04	2.83E-03	Gas6,14456:up|Stc1,20855:up|Ednrb,13618:up|Anpep,16790:up|Adipoq,11450:up
tube formation	GO:0035148	3.41E-04	2.86E-03	Cthrc1,68588:down|Adm,11535:up|Sfrp1,20377:up|Ptk7,71461:down|Wnt5a,22418:down|Grem1,23892:up|Bmp4,12159:up|Sfrp2,20319:up|Ret,19713:up|Vegfa,22339:down
cellular calcium ion homeostasis	GO:0006874	3.43E-04	2.87E-03	Htr2b,15559:down|Kng1,16644:up|Adm,11535:up|Ccl2,20296:up|Ednrb,13618:up|Ccl3,20302:up|Vdr,22337:down|Htr2a,15558:down|Fxyd1,56188:up|Glp1r,14652:down|Cd24a,12484:down|Cd4,12504:up|Stc1,20855:up|Bmp4,12159:up|Tgfb2,21808:down|Ptgir,19222:up
response to bacterium	GO:0009617	3.45E-04	2.87E-03	Adamts4,240913:down|Adm,11535:up|Hp,15439:up|Mrc1,17533:up|Ccl2,20296:up|Slpi,20568:up|Ednrb,13618:up|Ankrd1,107765:down|Tnf,21926:up|Cd24a,12484:down|Cd4,12504:up|Ccl12,20293:up|Wnt5a,22418:down|Ptgs2,19225:down|Cxcl9,17329:up|Ptgir,19222:up|Tnfrsf11b,18383:up|Clec4e,56619:up
divalent metal ion transport	GO:0070838	3.70E-04	3.08E-03	Htr2b,15559:down|Vdr,22337:down|Cxcl12,20315:up|Htr2a,15558:down|Slc40a1,53945:up|Gas6,14456:up|Trf,22041:up|Glp1r,14652:down|Cd4,12504:up|Ccl12,20293:up|Stc1,20855:up|Ptgs2,19225:down|Ccl4,20303:up|Bmp4,12159:up|Igf1,16000:up|Tgfb2,21808:down|S100a1,20193:up
endochondral bone morphogenesis	GO:0060350	3.72E-04	3.09E-03	Serpinh1,12406:down|Alpl,11647:up|Stc1,20855:up|Bnc2,242509:down|Mmp13,17386:up|Bmp4,12159:up
chemokine production	GO:0032602	3.74E-04	3.10E-03	Tnf,21926:up|Lpl,16956:up|C1qtnf3,81799:down|Postn,50706:down|Wnt5a,22418:down|Il1rl1,17082:down|Adipoq,11450:up
cellular transition metal ion homeostasis	GO:0046916	3.74E-04	3.10E-03	Trf,22041:up|Fam132b,227358:down|Hmox1,15368:up|Tfrc,22042:up|Mt2,17750:up|Slc40a1,53945:up|Mt1,17748:up
fat cell differentiation	GO:0045444	3.76E-04	3.11E-03	Pparg,19016:up|Sfrp1,20377:up|Wnt5a,22418:down|Ptgs2,19225:down|Igf1,16000:up|Adipoq,11450:up|Sfrp2,20319:up|Fabp4,11770:up|Vegfa,22339:down|Htr2a,15558:down|Medag,70717:down|Enpp1,18605:down
cell-cell adhesion	GO:0098609	3.78E-04	3.11E-03	Fstl3,83554:down|Ly9,17085:up|Ccl2,20296:up|Ctla2a,13024:down|Srpx2,68792:down|Cxcl12,20315:up|Vegfa,22339:down|Serpine2,20720:up|Igf2,16002:up|Tnf,21926:up|Egfr,13649:down|Cd24a,12484:down|Ncam1,17967:down|Has2,15117:down|Wnt5a,22418:down|Cd4,12504:up|Il1rn,16181:up|Tfrc,22042:up|Adipoq,11450:up|Bmp4,12159:up|Igf1,16000:up|Ceacam1,26365:up|Pcdh19,279653:down|Ret,19713:up|Tgfb2,21808:down|Cdsn,386463:up|Npnt,114249:up|Plpp3,67916:up|Tnfsf9,21950:up|Clec4e,56619:up
developmental process involved in reproduction	GO:0003006	3.78E-04	3.12E-03	Adm,11535:up|Inhba,16323:down|Gjb5,14622:down|Epas1,13819:up|Vdr,22337:down|Cxcl12,20315:up|Vegfa,22339:down|Mmp2,17390:down|Serpine2,20720:up|Pparg,19016:up|Alpl,11647:up|Egfr,13649:down|Sfrp1,20377:up|Sulf1,240725:down|Wnt5a,22418:down|Bmpr1a,12166:down|Ptgs2,19225:down|Bmp4,12159:up|Igf1,16000:up|Sfrp2,20319:up|Tnc,21923:down|Fgfr2,14183:up|Kitl,17311:up
regulation of lymphocyte migration	GO:2000401	3.87E-04	3.18E-03	Cxcl14,57266:down|Cxcl12,20315:up|Wnt5a,22418:down|Ccl12,20293:up|Ccl4,20303:up
negative regulation of innate immune response	GO:0045824	3.87E-04	3.18E-03	Ceacam1,26365:up|Pparg,19016:up|Clec2d,93694:up|Serping1,12258:up|Igf2,16002:up
divalent inorganic cation transport	GO:0072511	3.94E-04	3.23E-03	Htr2b,15559:down|Vdr,22337:down|Cxcl12,20315:up|Htr2a,15558:down|Slc40a1,53945:up|Gas6,14456:up|Trf,22041:up|Glp1r,14652:down|Cd4,12504:up|Ccl12,20293:up|Stc1,20855:up|Ptgs2,19225:down|Ccl4,20303:up|Bmp4,12159:up|Igf1,16000:up|Tgfb2,21808:down|S100a1,20193:up
positive regulation of cardiac muscle cell differentiation	GO:2000727	4.03E-04	3.29E-03	Wisp1,22402:down|Grem1,23892:up|Igf1,16000:up|Bmp4,12159:up
positive regulation of lymphocyte apoptotic process	GO:0070230	4.03E-04	3.29E-03	Ceacam1,26365:up|Cd24a,12484:down|Wnt5a,22418:down|Tgfb2,21808:down
adaptive immune response based on somatic recombination of immune receptors built from immunoglobulin superfamily domains	GO:0002460	4.29E-04	3.50E-03	Tnf,21926:up|Cd24a,12484:down|Ly9,17085:up|Cd4,12504:up|C3,12266:up|C1rb,667277:up|Tfrc,22042:up|Serping1,12258:up|C1s1,50908:up|Vegfa,22339:down|C1s2,317677:up|Il1rl1,17082:down
salivary gland morphogenesis	GO:0007435	4.43E-04	3.61E-03	Tnf,21926:up|Fgfr1,14182:down|Egfr,13649:down|Plxnd1,67784:up|Fgfr2,14183:up
negative regulation of viral genome replication	GO:0045071	4.43E-04	3.61E-03	Ceacam1,26365:up|Tnf,21926:up|Oasl1,231655:up|Slpi,20568:up|Oas3,246727:up
negative regulation of protein modification process	GO:0031400	4.46E-04	3.63E-03	Flrt2,399558:down|Sfrp4,20379:up|Aspn,66695:down|Fabp4,11770:up|Igfbp3,16009:up|Cd109,235505:down|Lrrc15,74488:down|Nxn,18230:down|Tnf,21926:up|Sfrp1,20377:up|Grem1,23892:up|Adipoq,11450:up|Bmp4,12159:up|Igf1,16000:up|Ceacam1,26365:up|Sfrp2,20319:up|Prkar2b,19088:up|Plpp3,67916:up|Errfi1,74155:down|Enpp1,18605:down
diterpenoid biosynthetic process	GO:0016102	4.53E-04	3.65E-03	Akr1c18,105349:up|Aldh1a2,19378:up|Aldh1a3,56847:down
cellular response to interferon-alpha	GO:0035457	4.53E-04	3.65E-03	Gas6,14456:up|Ifit1,15957:up|Ifit3,15959:up
extracellular fibril organization	GO:0043206	4.53E-04	3.65E-03	Ltbp2,16997:down|Col5a1,12831:down|Col3a1,12825:down
regulation of alkaline phosphatase activity	GO:0010692	4.53E-04	3.65E-03	Tnf,21926:up|Tgfb2,21808:down|Npnt,114249:up
retinoic acid biosynthetic process	GO:0002138	4.53E-04	3.65E-03	Akr1c18,105349:up|Aldh1a2,19378:up|Aldh1a3,56847:down
regulation of fever generation	GO:0031620	4.53E-04	3.65E-03	Tnf,21926:up|Ptgs2,19225:down|Ednrb,13618:up
regulation of heart growth	GO:0060420	4.55E-04	3.67E-03	Fgfr1,14182:down|Ncam1,17967:down|Bmpr1a,12166:down|Wisp1,22402:down|Fgfr2,14183:up|Igf1,16000:up

negative regulation of transport	GO:0051051	4.84E-04	3.89E-03	Sfrp4,20379:up|Gpm6b,14758:up|Htr2a,15558:down|Serpine2,20720:up|Anpep,16790:up|Kdelr3,105785:down|Gas6,14456:up|Pparg,19016:up|Tnf,21926:up|Hmox1,15368:up|Sfrp1,20377:up|C1qtnf3,81799:down|Il1rn,16181:up|Stc1,20855:up|Ptgs2,19225:down|Adipoq,11450:up|Maob,109731:up|Tgfb2,21808:down|Enpp1,18605:down
negative regulation of molecular function	GO:0044092	4.88E-04	3.92E-03	Kng1,16644:up|Flrt2,399558:down|Hp,15439:up|Sfrp4,20379:up|Slpi,20568:up|Cst7,13011:up|Aspn,66695:down|Tfpi2,21789:down|Fabp4,11770:up|Vegfa,22339:down|Serpine2,20720:up|Cd109,235505:down|Lrrc15,74488:down|Tnf,21926:up|Gas6,14456:up|Pparg,19016:up|Ptx3,19288:up|Hmox1,15368:up|Sfrp1,20377:up|Adipoq,11450:up|Bmp4,12159:up|Igf1,16000:up|Ceacam1,26365:up|Sfrp2,20319:up|Serping1,12258:up|Serpina3g,20715:up|Tgfb2,21808:down|Prkar2b,19088:up|Errfi1,74155:down
hematopoietic stem cell proliferation	GO:0071425	4.91E-04	3.93E-03	Sfrp2,20319:up|Prg4,96875:down|Wnt5a,22418:down|Kitl,17311:up
negative regulation of proteolysis	GO:0045861	4.94E-04	3.96E-03	Kng1,16644:up|Gas6,14456:up|Slpi,20568:up|Cst7,13011:up|Plat,18791:down|Tfpi2,21789:down|Igf1,16000:up|Ctla2a,13024:down|Sfrp2,20319:up|Serping1,12258:up|Serpina3g,20715:up|Vegfa,22339:down|Serpine2,20720:up|Cd109,235505:down
regulation of peptidase activity	GO:0052547	5.00E-04	4.00E-03	Tnf,21926:up|Pparg,19016:up|Kng1,16644:up|Gas6,14456:up|Slpi,20568:up|Cst7,13011:up|Tfpi2,21789:down|Igf1,16000:up|Sfrp2,20319:up|Serping1,12258:up|Serpina3g,20715:up|Vegfa,22339:down|Serpine2,20720:up|Cd109,235505:down|F3,14066:up
regulation of protein localization	GO:0032880	5.04E-04	4.02E-03	Htr2b,15559:down|Ice1,218333:down|Ccl2,20296:up|Ccl3,20302:up|Mmp13,17386:up|Ankrd1,107765:down|Gpm6b,14758:up|Clec4n,56620:up|Postn,50706:down|Lrrc15,74488:down|Kdelr3,105785:down|Gas6,14456:up|Tnf,21926:up|Glp1r,14652:down|Egfr,13649:down|Lpl,16956:up|Sfrp1,20377:up|C1qtnf3,81799:down|Wnt5a,22418:down|Bmpr1a,12166:down|Ptgs2,19225:down|Grem1,23892:up|Adipoq,11450:up|Bmp4,12159:up|Igf1,16000:up|Ceacam1,26365:up|Sfrp2,20319:up|Tgfb2,21808:down|Il1rl1,17082:down|Clec4e,56619:up
negative regulation of anion transport	GO:1903792	5.05E-04	4.02E-03	Tnf,21926:up|Gpm6b,14758:up|Sfrp4,20379:up|Il1rn,16181:up|Maob,109731:up
cellular iron ion homeostasis	GO:0006879	5.05E-04	4.02E-03	Trf,22041:up|Fam132b,227358:down|Hmox1,15368:up|Tfrc,22042:up|Slc40a1,53945:up
cardiac chamber development	GO:0003205	5.20E-04	4.13E-03	Col11a1,12814:down|Plxnd1,67784:up|Ptk7,71461:down|Wnt5a,22418:down|Bmpr1a,12166:down|Tgfbr3,21814:up|Bmp4,12159:up|Sfrp2,20319:up|Tgfb2,21808:down|Fgfr2,14183:up
neuron development	GO:0048666	5.36E-04	4.25E-03	Cthrc1,68588:down|Adm,11535:up|Flrt2,399558:down|Lama2,16773:down|Efemp1,216616:up|Plxnd1,67784:up|Ptk7,71461:down|Dpysl3,22240:down|Ednrb,13618:up|Sema7a,20361:down|Ankrd1,107765:down|Tnfrsf12a,27279:down|Cxcl12,20315:up|Postn,50706:down|Vegfa,22339:down|Mmp2,17390:down|Serpine2,20720:up|Plk2,20620:down|Cd24a,12484:down|Egfr,13649:down|Fgfr1,14182:down|Ncam1,17967:down|Sfrp1,20377:up|Wnt5a,22418:down|Bmp4,12159:up|Sfrp2,20319:up|Crabp2,12904:down|Tnc,21923:down|Ret,19713:up|Tgfb2,21808:down|Fgfr2,14183:up
regulation of nucleocytoplasmic transport	GO:0046822	5.52E-04	4.37E-03	Tnf,21926:up|Gas6,14456:up|Egfr,13649:down|C1qtnf3,81799:down|Bmpr1a,12166:down|Ptgs2,19225:down|Grem1,23892:up|Igf1,16000:up|Bmp4,12159:up|Ceacam1,26365:up|Sfrp2,20319:up
artery morphogenesis	GO:0048844	5.53E-04	4.37E-03	Hpgd,15446:up|Vegfa,22339:down|Tgfb2,21808:down|Bmpr1a,12166:down|Col3a1,12825:down|Bmp4,12159:up
nitric oxide biosynthetic process	GO:0006809	5.53E-04	4.37E-03	Tnf,21926:up|Ptx3,19288:up|Ddah1,69219:down|Egfr,13649:down|Ptgs2,19225:down|Igf1,16000:up
molting cycle	GO:0042303	5.55E-04	4.38E-03	Tnf,21926:up|Egfr,13649:down|Inhba,16323:down|Ptgs2,19225:down|Hpse,15442:up|Tgfb2,21808:down|Fgfr2,14183:up|Cd109,235505:down
hair cycle	GO:0042633	5.55E-04	4.38E-03	Tnf,21926:up|Egfr,13649:down|Inhba,16323:down|Ptgs2,19225:down|Hpse,15442:up|Tgfb2,21808:down|Fgfr2,14183:up|Cd109,235505:down
artery development	GO:0060840	5.58E-04	4.39E-03	Hpgd,15446:up|Plxnd1,67784:up|Vegfa,22339:down|Tgfb2,21808:down|Bmpr1a,12166:down|Col3a1,12825:down|Bmp4,12159:up
temperature homeostasis	GO:0001659	5.73E-04	4.50E-03	Tnf,21926:up|Htr2a,15558:down|Ptgs2,19225:down|Il1rn,16181:up|Ednrb,13618:up
positive regulation of nitric oxide biosynthetic process	GO:0045429	5.73E-04	4.50E-03	Tnf,21926:up|Ptx3,19288:up|Ddah1,69219:down|Egfr,13649:down|Ptgs2,19225:down
cell-matrix adhesion	GO:0007160	5.75E-04	4.51E-03	Grem1,23892:up|Ninj1,18081:up|Col3a1,12825:down|Gpm6b,14758:up|Sned1,208777:up|Postn,50706:down|Hpse,15442:up|Vegfa,22339:down|Npnt,114249:up|Adamts12,239337:down
response to lipopolysaccharide	GO:0032496	5.75E-04	4.51E-03	Tnf,21926:up|Adm,11535:up|Mrc1,17533:up|Ccl2,20296:up|Ccl12,20293:up|Ptgs2,19225:down|Slpi,20568:up|Ednrb,13618:up|Ankrd1,107765:down|Cxcl9,17329:up|Tnfrsf11b,18383:up|Ptgir,19222:up
forebrain development	GO:0030900	5.90E-04	4.60E-03	Fgfr1,14182:down|Egfr,13649:down|Ncam1,17967:down|Wnt5a,22418:down|Inhba,16323:down|Bmpr1a,12166:down|Aldh1a2,19378:up|Sema7a,20361:down|Col3a1,12825:down|Bmp4,12159:up|Uchl5,56207:up|Cxcl12,20315:up|Aldh1a3,56847:down|Cx3cr1,13051:down|Fgfr2,14183:up
negative regulation of epithelial to mesenchymal transition	GO:0010719	5.91E-04	4.60E-03	Sfrp2,20319:up|Sfrp1,20377:up|Tgfb2,21808:down|Tgfbr3,21814:up
positive regulation of cardiac muscle cell proliferation	GO:0060045	5.91E-04	4.60E-03	Fgfr1,14182:down|Ncam1,17967:down|Bmpr1a,12166:down|Fgfr2,14183:up
branch elongation of an epithelium	GO:0060602	5.91E-04	4.60E-03	Tnc,21923:down|Wnt5a,22418:down|Fgfr2,14183:up|Bmp4,12159:up
regulation of reproductive process	GO:2000241	5.92E-04	4.60E-03	Sfrp1,20377:up|Sulf1,240725:down|Wnt5a,22418:down|Inhba,16323:down|Ednrb,13618:up|Plat,18791:down|Igf1,16000:up|Bmp4,12159:up|Fgfr2,14183:up
humoral immune response	GO:0006959	5.93E-04	4.61E-03	Tnf,21926:up|Adm,11535:up|C3,12266:up|C1rb,667277:up|Slpi,20568:up|Serping1,12258:up|C1s1,50908:up|C1s2,317677:up
negative regulation of viral process	GO:0048525	6.02E-04	4.67E-03	Ceacam1,26365:up|Tnf,21926:up|Ptx3,19288:up|Oasl1,231655:up|Slpi,20568:up|Oas3,246727:up|Ccl4,20303:up
negative regulation of hydrolase activity	GO:0051346	6.10E-04	4.73E-03	Tnf,21926:up|Kng1,16644:up|Gas6,14456:up|Ptx3,19288:up|Slpi,20568:up|Cst7,13011:up|Tfpi2,21789:down|Igf1,16000:up|Sfrp2,20319:up|Serping1,12258:up|Serpina3g,20715:up|Vegfa,22339:down|Tgfb2,21808:down|Serpine2,20720:up|Cd109,235505:down

regulation of cellular metabolic process	GO:0031323	6.35E-04	4.89E-03	Fstl3,83554:down|Igfbp4,16010:up|Adm,11535:up|Flrt2,399558:down|Tfec,21426:up|Sfrp4,20379:up|C3,12266:up|Ankrd1,107765:down|Lyl1,17095:up|Igfbp3,16009:up|Cd109,235505:down|Loxl2,94352:down|Plk2,20620:down|Loxl3,16950:down|Trf,22041:up|Cd24a,12484:down|Wnt5a,22418:down|Il1rn,16181:up|Maob,109731:up|Adipoq,11450:up|Bmp4,12159:up|Igf1,16000:up|Tgfb2,21808:down|Ptgir,19222:up|F3,14066:up|Fgfr2,14183:up|Htr2b,15559:down|Kng1,16644:up|Prl2c2,18811:up|Dram1,71712:up|Efemp1,216616:up|Ccl6,20305:up|Slpi,20568:up|Aspn,66695:down|Vdr,22337:down|Fabp4,11770:up|Fam132b,227358:down|Htr2a,15558:down|Oas3,246727:up|Slc40a1,53945:up|Gas6,14456:up|Egfr,13649:down|Sfrp1,20377:up|C1qtnf3,81799:down|Tgfbr3,21814:up|Tfrc,22042:up|Ceacam1,26365:up|Ccl8,20307:up|Sfrp2,20319:up|Serpina3g,20715:up|Fam20a,208659:down|Npnt,114249:up|Tnfrsf11b,18383:up|Prkar2b,19088:up|S100a1,20193:up|Ice1,218333:down|Ccl2,20296:up|Ccl7,20306:up|Pdgfc,54635:down|Cst7,13011:up|Ednrb,13618:up|Sema7a,20361:down|Epas1,13819:up|Htra3,78558:down|Uchl5,56207:up|Ddah1,69219:down|Sdpr,20324:down|Ptx3,19288:up|Akr1c18,105349:up|Bmpr1a,12166:down|Ccl12,20293:up|Ccl4,20303:up|Ret,19713:up|Prg4,96875:down|Plpp3,67916:up|Creb3l1,26427:down|Kitl,17311:up|Hp,15439:up|Ccl9,20308:up|Inhba,16323:down|Rasl11a,68895:up|Bnc2,242509:down|Plat,18791:down|Ccl3,20302:up|Tfpi2,21789:down|Ctla2a,13024:down|Srpx2,68792:down|Crlf1,12931:down|Plod2,26432:down|Vegfa,22339:down|Serpine2,20720:up|Lrrc15,74488:down|Nxn,18230:down|Igf2,16002:up|Pparg,19016:up|Tnf,21926:up|Glp1r,14652:down|Lmcd1,30937:up|Fgfr1,14182:down|Hmox1,15368:up|Sulf1,240725:down|Cd4,12504:up|Ptgs2,19225:down|Grem1,23892:up|Phf11d,219132:up|Tbx15,21384:down|Serping1,12258:up|Errfi1,74155:down|Enpp1,18605:down
positive regulation of hair follicle development	GO:0051798	6.38E-04	4.89E-03	Tnf,21926:up|Tgfb2,21808:down|Hpse,15442:up
prostatic bud formation	GO:0060513	6.38E-04	4.89E-03	Sulf1,240725:down|Wnt5a,22418:down|Bmp4,12159:up
terpenoid biosynthetic process	GO:0016114	6.38E-04	4.89E-03	Akr1c18,105349:up|Aldh1a2,19378:up|Aldh1a3,56847:down
lung-associated mesenchyme development	GO:0060484	6.38E-04	4.89E-03	Fgfr1,14182:down|Ptk7,71461:down|Fgfr2,14183:up
positive regulation of insulin-like growth factor receptor signaling pathway	GO:0043568	6.38E-04	4.89E-03	Igfbp4,16010:up|Igfbp3,16009:up|Igf1,16000:up
bone mineralization involved in bone maturation	GO:0035630	6.38E-04	4.89E-03	Fam101b,76566:down|Grem1,23892:up|Igf1,16000:up
negative regulation of protein autophosphorylation	GO:0031953	6.38E-04	4.89E-03	Errfi1,74155:down|Enpp1,18605:down|Adipoq,11450:up
protein modification process	GO:0036211	6.47E-04	4.94E-03	Igfbp4,16010:up|Flrt2,399558:down|Sfrp4,20379:up|C3,12266:up|Igfbp3,16009:up|Cd109,235505:down|Loxl2,94352:down|Plk2,20620:down|Trf,22041:up|Cd24a,12484:down|Wnt5a,22418:down|Mark1,226778:up|Il1rn,16181:up|Adipoq,11450:up|Bmp4,12159:up|Igf1,16000:up|Tgfb2,21808:down|Fgfr2,14183:up|Htr2b,15559:down|Efemp1,216616:up|Ccl6,20305:up|Aspn,66695:down|Saa3,20210:up|Fabp4,11770:up|Htr2a,15558:down|Gas6,14456:up|Egfr,13649:down|Sfrp1,20377:up|C1qtnf3,81799:down|Tgfbr3,21814:up|Ceacam1,26365:up|Ccl8,20307:up|Sfrp2,20319:up|Fam20a,208659:down|Npnt,114249:up|Tnfrsf11b,18383:up|Prkar2b,19088:up|Neurl3,214854:down|Ccl2,20296:up|Ccl7,20306:up|Ednrb,13618:up|Pdgfc,54635:down|Pdzrn3,55983:down|Sema7a,20361:down|Uchl5,56207:up|Ddah1,69219:down|Dok2,13449:up|Man2a2,140481:up|Mamdc2,71738:down|Ccl12,20293:up|Bmpr1a,12166:down|Ccl4,20303:up|Ret,19713:up|Vegfb,22340:up|Sdf2l1,64136:up|Plpp3,67916:up|Kitl,17311:up|P4ha1,18451:down|Ccl9,20308:up|Inhba,16323:down|Ccl3,20302:up|Crlf1,12931:down|Vegfa,22339:down|Lrrc15,74488:down|Igf2,16002:up|Nxn,18230:down|Tnf,21926:up|Fgfr1,14182:down|Cd4,12504:up|Grem1,23892:up|Col3a1,12825:down|Errfi1,74155:down|Enpp1,18605:down
cellular protein modification process	GO:0006464	6.47E-04	4.94E-03	Igfbp4,16010:up|Flrt2,399558:down|Sfrp4,20379:up|C3,12266:up|Igfbp3,16009:up|Cd109,235505:down|Loxl2,94352:down|Plk2,20620:down|Trf,22041:up|Cd24a,12484:down|Wnt5a,22418:down|Mark1,226778:up|Il1rn,16181:up|Adipoq,11450:up|Bmp4,12159:up|Igf1,16000:up|Tgfb2,21808:down|Fgfr2,14183:up|Htr2b,15559:down|Efemp1,216616:up|Ccl6,20305:up|Aspn,66695:down|Saa3,20210:up|Fabp4,11770:up|Htr2a,15558:down|Gas6,14456:up|Egfr,13649:down|Sfrp1,20377:up|C1qtnf3,81799:down|Tgfbr3,21814:up|Ceacam1,26365:up|Ccl8,20307:up|Sfrp2,20319:up|Fam20a,208659:down|Npnt,114249:up|Tnfrsf11b,18383:up|Prkar2b,19088:up|Neurl3,214854:down|Ccl2,20296:up|Ccl7,20306:up|Ednrb,13618:up|Pdgfc,54635:down|Pdzrn3,55983:down|Sema7a,20361:down|Uchl5,56207:up|Ddah1,69219:down|Dok2,13449:up|Man2a2,140481:up|Mamdc2,71738:down|Ccl12,20293:up|Bmpr1a,12166:down|Ccl4,20303:up|Ret,19713:up|Vegfb,22340:up|Sdf2l1,64136:up|Plpp3,67916:up|Kitl,17311:up|P4ha1,18451:down|Ccl9,20308:up|Inhba,16323:down|Ccl3,20302:up|Crlf1,12931:down|Vegfa,22339:down|Lrrc15,74488:down|Igf2,16002:up|Nxn,18230:down|Tnf,21926:up|Fgfr1,14182:down|Cd4,12504:up|Grem1,23892:up|Col3a1,12825:down|Errfi1,74155:down|Enpp1,18605:down
vascular endothelial growth factor receptor signaling pathway	GO:0048010	6.48E-04	4.94E-03	Sulf1,240725:down|Vegfa,22339:down|Ccl2,20296:up|Figf,14205:up|Bmp4,12159:up
negative regulation of smooth muscle cell proliferation	GO:0048662	6.48E-04	4.94E-03	Pparg,19016:up|Hmox1,15368:up|Ptgir,19222:up|Igfbp3,16009:up|Adipoq,11450:up
positive regulation of neurogenesis	GO:0050769	6.50E-04	4.95E-03	Prl2c2,18811:up|Ptk7,71461:down|Dpysl3,22240:down|Sema7a,20361:down|Ankrd1,107765:down|Tnfrsf12a,27279:down|Cxcl12,20315:up|Vegfa,22339:down|Cx3cr1,13051:down|Serpine2,20720:up|Pparg,19016:up|Trf,22041:up|Cd24a,12484:down|Fgfr1,14182:down|Wnt5a,22418:down|Bmp4,12159:up|Crabp2,12904:down|Ret,19713:up
hexose metabolic process	GO:0019318	6.60E-04	5.02E-03	Tnf,21926:up|Igfbp4,16010:up|Fabp5,16592:up|C1qtnf3,81799:down|Igf1,16000:up|Adipoq,11450:up|Sord,20322:up|Igfbp3,16009:up|Enpp1,18605:down|Igf2,16002:up|Man2a2,140481:up
cytosolic calcium ion homeostasis	GO:0051480	6.65E-04	5.04E-03	Htr2b,15559:down|Kng1,16644:up|Glp1r,14652:down|Fxyd1,56188:up|Adm,11535:up|Cd24a,12484:down|Cd4,12504:up|Ednrb,13618:up|Ccl3,20302:up|Bmp4,12159:up|Tgfb2,21808:down|Htr2a,15558:down|Ptgir,19222:up

regulation of blood pressure	GO:0008217	6.66E-04	5.04E-03	Pparg,19016:up|Gas6,14456:up|Glp1r,14652:down|Col1a2,12843:down|Hmox1,15368:up|Ptgs2,19225:down|Ednrb,13618:up|Adipoq,11450:up|Ddah1,69219:down|Postn,50706:down
pathway-restricted SMAD protein phosphorylation	GO:0060389	6.67E-04	5.04E-03	Tgfb2,21808:down|Inhba,16323:down|Bmpr1a,12166:down|Tgfbr3,21814:up|Grem1,23892:up|Bmp4,12159:up
mesoderm formation	GO:0001707	6.67E-04	5.04E-03	Sfrp2,20319:up|Fgfr1,14182:down|Wnt5a,22418:down|Inhba,16323:down|Bmpr1a,12166:down|Bmp4,12159:up
positive regulation of intracellular protein transport	GO:0090316	6.77E-04	5.10E-03	Tnf,21926:up|Gas6,14456:up|Ice1,218333:down|Egfr,13649:down|Bmpr1a,12166:down|Ccl2,20296:up|Ptgs2,19225:down|Grem1,23892:up|Igf1,16000:up|Bmp4,12159:up|Ceacam1,26365:up|Sfrp2,20319:up
negative regulation of phosphorus metabolic process	GO:0010563	6.92E-04	5.20E-03	Flrt2,399558:down|Sfrp4,20379:up|Aspn,66695:down|Fabp4,11770:up|Igfbp3,16009:up|Cd109,235505:down|Lrrc15,74488:down|Tnf,21926:up|Sfrp1,20377:up|Grem1,23892:up|Adipoq,11450:up|Bmp4,12159:up|Igf1,16000:up|Ceacam1,26365:up|Sfrp2,20319:up|Tgfb2,21808:down|Prkar2b,19088:up|Plpp3,67916:up|Errfi1,74155:down|Enpp1,18605:down
negative regulation of phosphate metabolic process	GO:0045936	6.92E-04	5.20E-03	Flrt2,399558:down|Sfrp4,20379:up|Aspn,66695:down|Fabp4,11770:up|Igfbp3,16009:up|Cd109,235505:down|Lrrc15,74488:down|Tnf,21926:up|Sfrp1,20377:up|Grem1,23892:up|Adipoq,11450:up|Bmp4,12159:up|Igf1,16000:up|Ceacam1,26365:up|Sfrp2,20319:up|Tgfb2,21808:down|Prkar2b,19088:up|Plpp3,67916:up|Errfi1,74155:down|Enpp1,18605:down
calcium ion transport	GO:0006816	6.95E-04	5.22E-03	Htr2b,15559:down|Gas6,14456:up|Glp1r,14652:down|Ccl12,20293:up|Cd4,12504:up|Ptgs2,19225:down|Stc1,20855:up|Ccl4,20303:up|Igf1,16000:up|Bmp4,12159:up|Vdr,22337:down|Cxcl12,20315:up|Tgfb2,21808:down|Htr2a,15558:down|S100a1,20193:up
epithelial tube formation	GO:0072175	6.98E-04	5.23E-03	Cthrc1,68588:down|Adm,11535:up|Sfrp1,20377:up|Ptk7,71461:down|Wnt5a,22418:down|Grem1,23892:up|Bmp4,12159:up|Sfrp2,20319:up|Ret,19713:up
embryonic epithelial tube formation	GO:0001838	6.98E-04	5.23E-03	Cthrc1,68588:down|Adm,11535:up|Sfrp1,20377:up|Ptk7,71461:down|Wnt5a,22418:down|Grem1,23892:up|Bmp4,12159:up|Sfrp2,20319:up|Ret,19713:up
positive regulation of DNA replication	GO:0045740	6.99E-04	5.23E-03	Fgfr1,14182:down|Egfr,13649:down|Pdgfc,54635:down|Grem1,23892:up|Kitl,17311:up|Igf1,16000:up|Bmp4,12159:up
type B pancreatic cell proliferation	GO:0044342	7.05E-04	5.25E-03	Igfbp4,16010:up|Sfrp1,20377:up|Igfbp3,16009:up|Errfi1,74155:down
regulation of osteoblast proliferation	GO:0033688	7.05E-04	5.25E-03	Cthrc1,68588:down|Sfrp1,20377:up|Hpse,15442:up|Grem1,23892:up
positive regulation of vascular endothelial growth factor production	GO:0010575	7.05E-04	5.25E-03	Sulf1,240725:down|Hpse,15442:up|C3,12266:up|Ptgs2,19225:down
regulation of leukocyte cell-cell adhesion	GO:1903037	7.16E-04	5.33E-03	Tnf,21926:up|Cd24a,12484:down|Has2,15117:down|Ccl2,20296:up|Cd4,12504:up|Tfrc,22042:up|Igf1,16000:up|Bmp4,12159:up|Ctla2a,13024:down|Ceacam1,26365:up|Cxcl12,20315:up|Igf2,16002:up|Tnfsf9,21950:up
ear development	GO:0043583	7.20E-04	5.35E-03	Cthrc1,68588:down|Cxcl14,57266:down|Fgfr1,14182:down|Col11a1,12814:down|Ptk7,71461:down|Wnt5a,22418:down|Igf1,16000:up|Bmp4,12159:up|Mcoln3,171166:up|Aldh1a3,56847:down|Fgfr2,14183:up
salivary gland development	GO:0007431	7.30E-04	5.41E-03	Tnf,21926:up|Fgfr1,14182:down|Egfr,13649:down|Plxnd1,67784:up|Fgfr2,14183:up
complement activation	GO:0006956	7.30E-04	5.41E-03	C1s1,50908:up|Serping1,12258:up|C1s2,317677:up|C3,12266:up|C1rb,667277:up
positive regulation of cytosolic calcium ion concentration	GO:0007204	7.33E-04	5.42E-03	Htr2b,15559:down|Kng1,16644:up|Glp1r,14652:down|Adm,11535:up|Cd24a,12484:down|Cd4,12504:up|Ednrb,13618:up|Ccl3,20302:up|Bmp4,12159:up|Tgfb2,21808:down|Htr2a,15558:down|Ptgir,19222:up
homeostasis of number of cells	GO:0048872	7.43E-04	5.48E-03	Cd24a,12484:down|Hmox1,15368:up|Inhba,16323:down|Tgfbr3,21814:up|Ccl2,20296:up|Ampd3,11717:up|Epas1,13819:up|Bmp4,12159:up|Ceacam1,26365:up|Vegfa,22339:down|Tgfb2,21808:down|Slc40a1,53945:up|Kitl,17311:up
regulation of hormone levels	GO:0010817	7.45E-04	5.50E-03	Adm,11535:up|Inhba,16323:down|Aldh1a2,19378:up|Htr2a,15558:down|Igf2,16002:up|Pparg,19016:up|Tnf,21926:up|Akr1c18,105349:up|Glp1r,14652:down|Egfr,13649:down|Fgfr1,14182:down|Sfrp1,20377:up|C1qtnf3,81799:down|Il1rn,16181:up|Adipoq,11450:up|Igf1,16000:up|Crabp2,12904:down|Aldh1a3,56847:down
regulation of leukocyte proliferation	GO:0070663	7.52E-04	5.54E-03	Cd24a,12484:down|Ccl12,20293:up|Cd4,12504:up|Tfrc,22042:up|Grem1,23892:up|Igf1,16000:up|Bmp4,12159:up|Ceacam1,26365:up|Igf2,16002:up|Kitl,17311:up|Tnfsf9,21950:up
response to abiotic stimulus	GO:0009628	7.66E-04	5.63E-03	Htr2b,15559:down|Tfec,21426:up|Ccl2,20296:up|Plat,18791:down|Epas1,13819:up|Ankrd1,107765:down|Nabp1,109019:down|Vegfa,22339:down|Htr2a,15558:down|Hspb7,29818:up|Mmp2,17390:down|Serpine2,20720:up|Pparg,19016:up|Tnf,21926:up|Loxl2,94352:down|Hvcn1,74096:up|Egfr,13649:down|Cd24a,12484:down|Col11a1,12814:down|Hmox1,15368:up|Mme,17380:up|Sfrp1,20377:up|Igf1,16000:up|Col3a1,12825:down|Sfrp2,20319:up|Tgfb2,21808:down
positive regulation of leukocyte cell-cell adhesion	GO:1903039	7.69E-04	5.65E-03	Tnf,21926:up|Cd24a,12484:down|Has2,15117:down|Ccl2,20296:up|Cd4,12504:up|Tfrc,22042:up|Igf1,16000:up|Ceacam1,26365:up|Igf2,16002:up|Tnfsf9,21950:up
renal system development	GO:0072001	7.70E-04	5.65E-03	Fgfr1,14182:down|Sfrp1,20377:up|Sulf1,240725:down|Wnt5a,22418:down|Aldh1a2,19378:up|Grem1,23892:up|Bmp4,12159:up|Adipoq,11450:up|Crlf1,12931:down|Ret,19713:up|Vegfa,22339:down|Npnt,114249:up|Fgfr2,14183:up
regulation of cellular component organization	GO:0051128	7.87E-04	5.77E-03	Igfbp4,16010:up|Ice1,218333:down|Flrt2,399558:down|Plxnd1,67784:up|Sfrp4,20379:up|Ccl2,20296:up|C3,12266:up|Sema7a,20361:down|Ankrd1,107765:down|Htra3,78558:down|Tnfrsf12a,27279:down|Gpm6b,14758:up|Postn,50706:down|Igfbp3,16009:up|Plk2,20620:down|Trf,22041:up|Ptx3,19288:up|Cd24a,12484:down|Ncam1,17967:down|Wnt5a,22418:down|Ccl12,20293:up|Wisp1,22402:down|Adipoq,11450:up|Bmp4,12159:up|Igf1,16000:up|Crabp2,12904:down|Cxcl9,17329:up|Ret,19713:up|Tgfb2,21808:down|Fgfr2,14183:up|Thbs2,21826:down|Prl2c2,18811:up|Efemp1,216616:up|Ptk7,71461:down|Inhba,16323:down|Dpysl3,22240:down|Ccl3,20302:up|Srpx2,68792:down|Plod2,26432:down|Cxcl12,20315:up|Vegfa,22339:down|Serpine2,20720:up|Lrrc15,74488:down|Igf2,16002:up|Pparg,19016:up|Gas6,14456:up|Tnf,21926:up|Lmcd1,30937:up|Fgfr1,14182:down|Egfr,13649:down|Has2,15117:down|Sfrp1,20377:up|Tgfbr3,21814:up|Grem1,23892:up|Ccl8,20307:up|Sfrp2,20319:up|Col5a1,12831:down|Enpp1,18605:down

neuron differentiation	GO:0030182	7.88E-04	5.77E-03	Adm,11535:up|Flrt2,399558:down|Plxnd1,67784:up|Aldh1a2,19378:up|Figf,14205:up|Ednrb,13618:up|Sema7a,20361:down|Ankrd1,107765:down|Tnfrsf12a,27279:down|Postn,50706:down|Plk2,20620:down|Cd24a,12484:down|Ncam1,17967:down|Wnt5a,22418:down|Bmp4,12159:up|Crabp2,12904:down|Tnc,21923:down|Ret,19713:up|Tgfb2,21808:down|Fgfr2,14183:up|Cthrc1,68588:down|Lama2,16773:down|Efemp1,216616:up|Ptk7,71461:down|Inhba,16323:down|Dpysl3,22240:down|Cxcl12,20315:up|Vegfa,22339:down|Serpine2,20720:up|Mmp2,17390:down|Egfr,13649:down|Fgfr1,14182:down|Sfrp1,20377:up|Sfrp2,20319:up|Mcoln3,171166:up|Rspo2,239405:up
bone resorption	GO:0045453	7.97E-04	5.80E-03	Ceacam1,26365:up|Trf,22041:up|Egfr,13649:down|Vegfa,22339:down|Tfrc,22042:up|Tnfrsf11b,18383:up
neuroblast proliferation	GO:0007405	7.97E-04	5.80E-03	Prl2c2,18811:up|Fgfr1,14182:down|Cd24a,12484:down|Vegfa,22339:down|Cx3cr1,13051:down|Fgfr2,14183:up
nitric oxide metabolic process	GO:0046209	7.97E-04	5.80E-03	Tnf,21926:up|Ptx3,19288:up|Ddah1,69219:down|Egfr,13649:down|Ptgs2,19225:down|Igf1,16000:up
response to glucocorticoid	GO:0051384	7.97E-04	5.80E-03	Tnf,21926:up|Alpl,11647:up|Il1rn,16181:up|Pappa,18491:down|Cdo1,12583:up|Bmp4,12159:up
lung morphogenesis	GO:0060425	7.97E-04	5.80E-03	Tnf,21926:up|Tnc,21923:down|Rspo2,239405:up|Fgfr2,14183:up|Igf1,16000:up|Bmp4,12159:up
vasoconstriction	GO:0042310	8.08E-04	5.87E-03	Htr2b,15559:down|Adm,11535:up|Egfr,13649:down|Htr2a,15558:down|Ptgs2,19225:down|Mmp2,17390:down|Ednrb,13618:up
memory	GO:0007613	8.09E-04	5.87E-03	Plk2,20620:down|Glp1r,14652:down|Ptgs2,19225:down|Il1rn,16181:up|Igf1,16000:up|Htr2a,15558:down|Cx3cr1,13051:down|Igf2,16002:up
glial cell migration	GO:0008347	8.19E-04	5.92E-03	Efemp1,216616:up|Tgfb2,21808:down|Ccl2,20296:up|Ccl12,20293:up|Ccl3,20302:up
positive regulation of kidney development	GO:0090184	8.19E-04	5.92E-03	Ret,19713:up|Vegfa,22339:down|Grem1,23892:up|Bmp4,12159:up|Adipoq,11450:up
regulation of lipid biosynthetic process	GO:0046890	8.19E-04	5.92E-03	Htr2b,15559:down|Tnf,21926:up|Akr1c18,105349:up|C3,12266:up|Igf1,16000:up|Vdr,22337:down|Ceacam1,26365:up|Htr2a,15558:down|Igf2,16002:up
regulation of hair cycle	GO:0042634	8.35E-04	6.02E-03	Tnf,21926:up|Tgfb2,21808:down|Inhba,16323:down|Hpse,15442:up
positive regulation of blood vessel endothelial cell migration	GO:0043536	8.35E-04	6.02E-03	Prl2c2,18811:up|Srpx2,68792:down|Vegfa,22339:down|Ptgs2,19225:down
positive regulation of activated T cell proliferation	GO:0042104	8.35E-04	6.02E-03	Cd24a,12484:down|Igf2,16002:up|Tnfsf9,21950:up|Igf1,16000:up
response to organonitrogen compound	GO:0010243	8.43E-04	6.07E-03	Htr2b,15559:down|Col5a2,12832:down|Pdgfc,54635:down|Htr2a,15558:down|Mmp2,17390:down|Igf2,16002:up|Cdo1,12583:up|Pparg,19016:up|Tnf,21926:up|Col1a2,12843:down|Egfr,13649:down|Ncam1,17967:down|Hmox1,15368:up|Sfrp1,20377:up|Adipoq,11450:up|Igf1,16000:up|Col3a1,12825:down|Gatm,67092:down|Serpina3g,20715:up|Enpp1,18605:down
negative regulation of protein kinase activity	GO:0006469	8.55E-04	6.14E-03	Flrt2,399558:down|Sfrp1,20377:up|Aspn,66695:down|Bmp4,12159:up|Adipoq,11450:up|Ceacam1,26365:up|Sfrp2,20319:up|Fabp4,11770:up|Prkar2b,19088:up|Lrrc15,74488:down|Errfi1,74155:down
lymphocyte mediated immunity	GO:0002449	8.55E-04	6.14E-03	Tnf,21926:up|Clec2d,93694:up|Cd24a,12484:down|C3,12266:up|C1rb,667277:up|Tfrc,22042:up|Ceacam1,26365:up|Serping1,12258:up|C1s1,50908:up|C1s2,317677:up|Igf2,16002:up
regulation of wound healing	GO:0061041	8.59E-04	6.16E-03	Kng1,16644:up|Ceacam1,26365:up|Tnfrsf12a,27279:down|Serping1,12258:up|Hpse,15442:up|Serpine2,20720:up|Plpp3,67916:up|Cd109,235505:down
positive regulation of chemokine secretion	GO:0090197	8.66E-04	6.16E-03	Lpl,16956:up|Postn,50706:down|Il1rl1,17082:down
cellular response to vitamin	GO:0071295	8.66E-04	6.16E-03	Vdr,22337:down|Gas6,14456:up|Postn,50706:down
skin morphogenesis	GO:0043589	8.66E-04	6.16E-03	Col1a2,12843:down|Cdsn,386463:up|Errfi1,74155:down
negative regulation of natural killer cell mediated cytotoxicity	GO:0045953	8.66E-04	6.16E-03	Ceacam1,26365:up|Clec2d,93694:up|Igf2,16002:up
positive regulation of hair cycle	GO:0042635	8.66E-04	6.16E-03	Tnf,21926:up|Tgfb2,21808:down|Hpse,15442:up
renal system process	GO:0003014	8.67E-04	6.16E-03	Gas6,14456:up|Sulf1,240725:down|Stc1,20855:up|Ednrb,13618:up|Anpep,16790:up|Bmp4,12159:up|Adipoq,11450:up
regulation of cell morphogenesis involved in differentiation	GO:0010769	8.67E-04	6.16E-03	Has2,15117:down|Plxnd1,67784:up|Sfrp1,20377:up|Wnt5a,22418:down|Tgfbr3,21814:up|Grem1,23892:up|Sema7a,20361:down|Tnfrsf12a,27279:down|Sfrp2,20319:up|Crabp2,12904:down|Ret,19713:up|Cxcl12,20315:up|Postn,50706:down|Vegfa,22339:down|Tgfb2,21808:down
regulation of calcium-mediated signaling	GO:0050848	8.69E-04	6.16E-03	Lmcd1,30937:up|Cd24a,12484:down|Ncam1,17967:down|Cd4,12504:up|Ccl4,20303:up|Igf1,16000:up
striated muscle cell proliferation	GO:0014855	8.69E-04	6.16E-03	Fgfr1,14182:down|Ncam1,17967:down|Tgfb2,21808:down|Bmpr1a,12166:down|Tgfbr3,21814:up|Fgfr2,14183:up
negative regulation of homeostatic process	GO:0032845	8.85E-04	6.27E-03	Htr2b,15559:down|Glp1r,14652:down|Cd24a,12484:down|Ceacam1,26365:up|Vegfa,22339:down|Tgfb2,21808:down|Htr2a,15558:down|Tnfrsf11b,18383:up|Anpep,16790:up|Kitl,17311:up
muscle tissue development	GO:0060537	9.06E-04	6.41E-03	Aldh1a2,19378:up|Ankrd1,107765:down|Vegfa,22339:down|Col11a1,12814:down|Fgfr1,14182:down|Ncam1,17967:down|Tgfbr3,21814:up|Bmpr1a,12166:down|Wnt5a,22418:down|Wisp1,22402:down|Grem1,23892:up|Bmp4,12159:up|Igf1,16000:up|Col3a1,12825:down|Tgfb2,21808:down|Fgfr2,14183:up
erythrocyte homeostasis	GO:0034101	9.12E-04	6.43E-03	Cd24a,12484:down|Hmox1,15368:up|Inhba,16323:down|Tgfbr3,21814:up|Ampd3,11717:up|Epas1,13819:up|Bmp4,12159:up|Vegfa,22339:down
multicellular organismal homeostasis	GO:0048871	9.20E-04	6.48E-03	Tnf,21926:up|Trf,22041:up|Egfr,13649:down|Ptgs2,19225:down|Tfrc,22042:up|Il1rn,16181:up|Ednrb,13618:up|Ampd3,11717:up|Epas1,13819:up|Ceacam1,26365:up|Vegfa,22339:down|Htr2a,15558:down|Tnfrsf11b,18383:up|Slc40a1,53945:up
regulation of myeloid cell differentiation	GO:0045637	9.27E-04	6.52E-03	Fstl3,83554:down|Tnf,21926:up|Clec2d,93694:up|Lrrc17,74511:down|Sfrp1,20377:up|Inhba,16323:down|Ccl3,20302:up|Adipoq,11450:up|Ceacam1,26365:up|Kitl,17311:up

organ formation	GO:0048645	9.46E-04	6.64E-03	Fgfr1,14182:down|Sulf1,240725:down|Wnt5a,22418:down|Bmpr1a,12166:down|Fgfr2,14183:up|Bmp4,12159:up
mesoderm morphogenesis	GO:0048332	9.46E-04	6.64E-03	Sfrp2,20319:up|Fgfr1,14182:down|Wnt5a,22418:down|Inhba,16323:down|Bmpr1a,12166:down|Bmp4,12159:up
leukocyte mediated immunity	GO:0002443	9.54E-04	6.65E-03	Tnf,21926:up|Clec2d,93694:up|Cd24a,12484:down|Hmox1,15368:up|Ccl2,20296:up|C3,12266:up|C1rb,667277:up|Tfrc,22042:up|Ceacam1,26365:up|C1s1,50908:up|Serping1,12258:up|C1s2,317677:up|Igf2,16002:up
negative regulation of endodermal cell differentiation	GO:1903225	9.65E-04	6.65E-03	Col5a2,12832:down|Col5a1,12831:down
proteoglycan catabolic process	GO:0030167	9.65E-04	6.65E-03	Hpse,15442:up|Adamts12,239337:down
pigment catabolic process	GO:0046149	9.65E-04	6.65E-03	Hmox1,15368:up|Blvrb,233016:up
ventricular zone neuroblast division	GO:0021847	9.65E-04	6.65E-03	Fgfr1,14182:down|Fgfr2,14183:up
regulation of transcription from RNA polymerase II promoter in response to iron	GO:0034395	9.65E-04	6.65E-03	Hmox1,15368:up|Slc40a1,53945:up
regulation of planar cell polarity pathway involved in axis elongation	GO:2000040	9.65E-04	6.65E-03	Sfrp2,20319:up|Sfrp1,20377:up
lateral mesodermal cell differentiation	GO:0048371	9.65E-04	6.65E-03	Fgfr1,14182:down|Bmpr1a,12166:down
negative regulation of planar cell polarity pathway involved in axis elongation	GO:2000041	9.65E-04	6.65E-03	Sfrp2,20319:up|Sfrp1,20377:up
lateral mesoderm morphogenesis	GO:0048369	9.65E-04	6.65E-03	Fgfr1,14182:down|Bmpr1a,12166:down
negative regulation of dendritic cell differentiation	GO:2001199	9.65E-04	6.65E-03	Tmem176a,66058:up|Tmem176b,65963:up
negative regulation of bone mineralization involved in bone maturation	GO:1900158	9.65E-04	6.65E-03	Fam101b,76566:down|Grem1,23892:up
heme catabolic process	GO:0042167	9.65E-04	6.65E-03	Hmox1,15368:up|Blvrb,233016:up
phospholipase C-activating serotonin receptor signaling pathway	GO:0007208	9.65E-04	6.65E-03	Htr2b,15559:down|Htr2a,15558:down
lateral mesoderm formation	GO:0048370	9.65E-04	6.65E-03	Fgfr1,14182:down|Bmpr1a,12166:down
telencephalon development	GO:0021537	9.68E-04	6.66E-03	Fgfr1,14182:down|Egfr,13649:down|Wnt5a,22418:down|Inhba,16323:down|Sema7a,20361:down|Col3a1,12825:down|Bmp4,12159:up|Uchl5,56207:up|Cxcl12,20315:up|Aldh1a3,56847:down|Cx3cr1,13051:down
regulation of odontogenesis	GO:0042481	9.80E-04	6.72E-03	Rspo2,239405:up|Tnfrsf11b,18383:up|Aspn,66695:down|Bmp4,12159:up
regulation of vascular permeability	GO:0043114	9.80E-04	6.72E-03	Ceacam1,26365:up|Adm,11535:up|Vegfa,22339:down|Ccl4,20303:up
regulation of neural precursor cell proliferation	GO:2000177	9.95E-04	6.81E-03	Prl2c2,18811:up|Trf,22041:up|Cd24a,12484:down|Wnt5a,22418:down|Vegfa,22339:down|Cx3cr1,13051:down|Igf1,16000:up
regulation of fibroblast proliferation	GO:0048145	1.06E-03	7.27E-03	Pparg,19016:up|Gas6,14456:up|Egfr,13649:down|Sfrp1,20377:up|Wnt5a,22418:down|Pdgfc,54635:down|Igf1,16000:up
positive regulation of T cell proliferation	GO:0042102	1.14E-03	7.64E-03	Ceacam1,26365:up|Cd24a,12484:down|Cd4,12504:up|Tfrc,22042:up|Igf2,16002:up|Tnfsf9,21950:up|Igf1,16000:up
regulation of chemokine secretion	GO:0090196	1.14E-03	7.64E-03	Lpl,16956:up|Postn,50706:down|Il1rl1,17082:down
chemokine secretion	GO:0090195	1.14E-03	7.64E-03	Lpl,16956:up|Postn,50706:down|Il1rl1,17082:down
negative regulation of natural killer cell mediated immunity	GO:0002716	1.14E-03	7.64E-03	Ceacam1,26365:up|Clec2d,93694:up|Igf2,16002:up
regulation of apoptotic process involved in morphogenesis	GO:1902337	1.14E-03	7.64E-03	Vdr,22337:down|Tgfb2,21808:down|Tgfbr3,21814:up
positive regulation of inflammatory response to antigenic stimulus	GO:0002863	1.14E-03	7.64E-03	Tnf,21926:up|Cd24a,12484:down|C3,12266:up
prostate glandular acinus development	GO:0060525	1.14E-03	7.64E-03	Sfrp1,20377:up|Fgfr2,14183:up|Igf1,16000:up
regulation of macrophage cytokine production	GO:0010935	1.14E-03	7.64E-03	Wnt5a,22418:down|Tgfb2,21808:down|Sema7a,20361:down

positive regulation of sprouting angiogenesis	GO:1903672	1.14E-03	7.64E-03	Srpx2,68792:down|Vegfa,22339:down|Ptgs2,19225:down
negative regulation of cell adhesion	GO:0007162	1.14E-03	7.64E-03	Cd24a,12484:down|Il1rn,16181:up|Bmp4,12159:up|Adipoq,11450:up|Ceacam1,26365:up|Tnc,21923:down|Cxcl12,20315:up|Postn,50706:down|Vegfa,22339:down|Serpine2,20720:up|Mmp2,17390:down
negative regulation of JNK cascade	GO:0046329	1.14E-03	7.64E-03	Ceacam1,26365:up|Sfrp2,20319:up|Sfrp1,20377:up|Sfrp4,20379:up
coronary vasculature morphogenesis	GO:0060977	1.14E-03	7.64E-03	Fgfr1,14182:down|Vegfa,22339:down|Tgfbr3,21814:up|Fgfr2,14183:up
positive regulation of lymphocyte migration	GO:2000403	1.14E-03	7.64E-03	Cxcl14,57266:down|Cxcl12,20315:up|Wnt5a,22418:down|Ccl4,20303:up
regulation of sprouting angiogenesis	GO:1903670	1.14E-03	7.64E-03	Ceacam1,26365:up|Srpx2,68792:down|Vegfa,22339:down|Ptgs2,19225:down
osteoblast proliferation	GO:0033687	1.14E-03	7.64E-03	Cthrc1,68588:down|Sfrp1,20377:up|Hpse,15442:up|Grem1,23892:up
regulation of immune effector process	GO:0002697	1.14E-03	7.64E-03	Tnf,21926:up|Clec2d,93694:up|Lmcd1,30937:up|Cd24a,12484:down|Hmox1,15368:up|Wnt5a,22418:down|Ccl2,20296:up|C3,12266:up|Tfrc,22042:up|Sema7a,20361:down|Ceacam1,26365:up|Serping1,12258:up|Tgfb2,21808:down|Ifit1,15957:up|Igf2,16002:up
calcium-mediated signaling	GO:0019722	1.15E-03	7.67E-03	Htr2b,15559:down|Tnf,21926:up|Lmcd1,30937:up|Cd24a,12484:down|Ncam1,17967:down|Cd4,12504:up|Ccl4,20303:up|Igf1,16000:up
stress-activated protein kinase signaling cascade	GO:0031098	1.19E-03	7.92E-03	Tnf,21926:up|Trf,22041:up|Sfrp1,20377:up|Wnt5a,22418:down|Sfrp4,20379:up|Il1rn,16181:up|Ceacam1,26365:up|Sfrp2,20319:up|Vegfa,22339:down|Tgfb2,21808:down|Errfi1,74155:down
adaptive immune response	GO:0002250	1.19E-03	7.94E-03	Tnf,21926:up|Cd24a,12484:down|Ly9,17085:up|Cd4,12504:up|C3,12266:up|C1rb,667277:up|Tfrc,22042:up|Clec4n,56620:up|Serping1,12258:up|C1s1,50908:up|Serpina3g,20715:up|Vegfa,22339:down|C1s2,317677:up|Il1rl1,17082:down
cytokine production involved in immune response	GO:0002367	1.21E-03	8.05E-03	Tnf,21926:up|Gas6,14456:up|Hmox1,15368:up|Wnt5a,22418:down|Tgfb2,21808:down|Sema7a,20361:down
regulation of epithelial cell apoptotic process	GO:1904035	1.21E-03	8.05E-03	Gas6,14456:up|Akr1c18,105349:up|Hmox1,15368:up|Sfrp4,20379:up|Igf1,16000:up|Bmp4,12159:up
negative regulation of immune effector process	GO:0002698	1.22E-03	8.06E-03	Ceacam1,26365:up|Tnf,21926:up|Clec2d,93694:up|Serping1,12258:up|Hmox1,15368:up|Tgfb2,21808:down|Igf2,16002:up
negative regulation of extrinsic apoptotic signaling pathway	GO:2001237	1.22E-03	8.06E-03	Tnf,21926:up|Sfrp2,20319:up|Tnfrsf23,79201:down|Hmox1,15368:up|Cx3cr1,13051:down|Igf1,16000:up|Bmp4,12159:up
fibroblast proliferation	GO:0048144	1.22E-03	8.06E-03	Pparg,19016:up|Gas6,14456:up|Egfr,13649:down|Sfrp1,20377:up|Wnt5a,22418:down|Pdgfc,54635:down|Igf1,16000:up
regulation of primary metabolic process	GO:0080090	1.23E-03	8.16E-03	Fstl3,83554:down|Igfbp4,16010:up|Adm,11535:up|Flrt2,399558:down|Tfec,21426:up|Sfrp4,20379:up|C3,12266:up|Ankrd1,107765:down|Lyl1,17095:up|Igfbp3,16009:up|Cd109,235505:down|Loxl2,94352:down|Plk2,20620:down|Loxl3,16950:down|Trf,22041:up|Cd24a,12484:down|Wnt5a,22418:down|Il1rn,16181:up|Maob,109731:up|Adipoq,11450:up|Bmp4,12159:up|Igf1,16000:up|Tgfb2,21808:down|Ptgir,19222:up|F3,14066:up|Fgfr2,14183:up|Htr2b,15559:down|Kng1,16644:up|Prl2c2,18811:up|Efemp1,216616:up|Ccl6,20305:up|Slpi,20568:up|Aspn,66695:down|Vdr,22337:down|Fabp4,11770:up|Fam132b,227358:down|Htr2a,15558:down|Oas3,246727:up|Slc40a1,53945:up|Gas6,14456:up|Egfr,13649:down|Sfrp1,20377:up|C1qtnf3,81799:down|Tgfbr3,21814:up|Tfrc,22042:up|Ceacam1,26365:up|Ccl8,20307:up|Sfrp2,20319:up|Serpina3g,20715:up|Fam20a,208659:down|Npnt,114249:up|Tnfrsf11b,18383:up|Prkar2b,19088:up|S100a1,20193:up|Ice1,218333:down|Ccl2,20296:up|Ccl7,20306:up|Pdgfc,54635:down|Cst7,13011:up|Ednrb,13618:up|Sema7a,20361:down|Epas1,13819:up|Htra3,78558:down|Uchl5,56207:up|Sdpr,20324:down|Ptx3,19288:up|Akr1c18,105349:up|Ccl12,20293:up|Bmpr1a,12166:down|Ccl4,20303:up|Ret,19713:up|Prg4,96875:down|Plpp3,67916:up|Creb3l1,26427:down|Kitl,17311:up|Ccl9,20308:up|Inhba,16323:down|Rasl11a,68895:up|Bnc2,242509:down|Plat,18791:down|Ccl3,20302:up|Tfpi2,21789:down|Ctla2a,13024:down|Crlf1,12931:down|Vegfa,22339:down|Serpine2,20720:up|Lrrc15,74488:down|Nxn,18230:down|Igf2,16002:up|Pparg,19016:up|Tnf,21926:up|Glp1r,14652:down|Lmcd1,30937:up|Fgfr1,14182:down|Has2,15117:down|Hmox1,15368:up|Sulf1,240725:down|Cd4,12504:up|Grem1,23892:up|Phf11d,219132:up|Tbx15,21384:down|Serping1,12258:up|Errfi1,74155:down|Enpp1,18605:down
ventricular cardiac muscle tissue morphogenesis	GO:0055010	1.26E-03	8.31E-03	Col11a1,12814:down|Tgfb2,21808:down|Bmpr1a,12166:down|Tgfbr3,21814:up|Fgfr2,14183:up
embryonic cranial skeleton morphogenesis	GO:0048701	1.26E-03	8.31E-03	Chst11,58250:down|Tbx15,21384:down|Tgfb2,21808:down|Fgfr2,14183:up|Bmp4,12159:up
branching involved in salivary gland morphogenesis	GO:0060445	1.32E-03	8.68E-03	Tnf,21926:up|Fgfr1,14182:down|Plxnd1,67784:up|Fgfr2,14183:up
endocytosis	GO:0006897	1.32E-03	8.68E-03	Htr2b,15559:down|Adm,11535:up|Mrc1,17533:up|Sfrp4,20379:up|Ccl2,20296:up|C3,12266:up|Vegfa,22339:down|Gas6,14456:up|Pparg,19016:up|Tnf,21926:up|Ptx3,19288:up|Trf,22041:up|Cd24a,12484:down|Wnt5a,22418:down|Tfrc,22042:up|Grem1,23892:up|Adipoq,11450:up|Mrc2,17534:down
activation of protein kinase activity	GO:0032147	1.32E-03	8.68E-03	Tnf,21926:up|Gas6,14456:up|Trf,22041:up|Egfr,13649:down|Wnt5a,22418:down|Pdgfc,54635:down|Igf1,16000:up|Bmp4,12159:up|Tgfb2,21808:down|Vegfa,22339:down
regulation of stress-activated MAPK cascade	GO:0032872	1.32E-03	8.68E-03	Tnf,21926:up|Trf,22041:up|Sfrp1,20377:up|Wnt5a,22418:down|Sfrp4,20379:up|Il1rn,16181:up|Ceacam1,26365:up|Sfrp2,20319:up|Vegfa,22339:down|Tgfb2,21808:down
cardiac chamber morphogenesis	GO:0003206	1.36E-03	8.88E-03	Col11a1,12814:down|Wnt5a,22418:down|Bmpr1a,12166:down|Tgfbr3,21814:up|Bmp4,12159:up|Sfrp2,20319:up|Tgfb2,21808:down|Fgfr2,14183:up
negative regulation of intracellular signal transduction	GO:1902532	1.38E-03	9.02E-03	Flrt2,399558:down|Sfrp4,20379:up|Aspn,66695:down|Cxcl12,20315:up|Serpine2,20720:up|Lrrc15,74488:down|Sfrp1,20377:up|C1qtnf3,81799:down|Adipoq,11450:up|Bmp4,12159:up|Igf1,16000:up|Ceacam1,26365:up|Sfrp2,20319:up|Tgfb2,21808:down|Creb3l1,26427:down|Errfi1,74155:down|Il1rl1,17082:down

regulation of stress-activated protein kinase signaling cascade	GO:0070302	1.38E-03	9.02E-03	Tnf,21926:up|Trf,22041:up|Sfrp1,20377:up|Wnt5a,22418:down|Sfrp4,20379:up|Il1rn,16181:up|Ceacam1,26365:up|Sfrp2,20319:up|Vegfa,22339:down|Tgfb2,21808:down
regulation of extrinsic apoptotic signaling pathway in absence of ligand	GO:2001239	1.39E-03	9.08E-03	Tnf,21926:up|Fgfr1,14182:down|Tgfb2,21808:down|Inhba,16323:down|Cx3cr1,13051:down
regulation of reactive oxygen species biosynthetic process	GO:1903426	1.41E-03	9.22E-03	Tnf,21926:up|Ptx3,19288:up|Ddah1,69219:down|Egfr,13649:down|Ptgs2,19225:down|Igf1,16000:up
receptor metabolic process	GO:0043112	1.42E-03	9.24E-03	Htr2b,15559:down|Tnf,21926:up|Pparg,19016:up|Adm,11535:up|Sfrp4,20379:up|Tfrc,22042:up|Grem1,23892:up|Adipoq,11450:up|Vegfa,22339:down
mesodermal cell fate specification	GO:0007501	1.46E-03	9.43E-03	Sfrp2,20319:up|Fgfr1,14182:down|Bmpr1a,12166:down
negative regulation of leukocyte mediated cytotoxicity	GO:0001911	1.46E-03	9.43E-03	Ceacam1,26365:up|Clec2d,93694:up|Igf2,16002:up
positive regulation of T cell apoptotic process	GO:0070234	1.46E-03	9.43E-03	Ceacam1,26365:up|Wnt5a,22418:down|Tgfb2,21808:down
regulation of vitamin metabolic process	GO:0030656	1.46E-03	9.43E-03	Vdr,22337:down|Tnf,21926:up|Akr1c18,105349:up
regulation of heat generation	GO:0031650	1.46E-03	9.43E-03	Tnf,21926:up|Ptgs2,19225:down|Ednrb,13618:up
retinal metabolic process	GO:0042574	1.46E-03	9.43E-03	Akr1c18,105349:up|Aldh1a2,19378:up|Aldh1a3,56847:down
regulation of odontogenesis of dentin-containing tooth	GO:0042487	1.46E-03	9.43E-03	Rspo2,239405:up|Tnfrsf11b,18383:up|Bmp4,12159:up
neural tube closure	GO:0001843	1.47E-03	9.48E-03	Cthrc1,68588:down|Sfrp2,20319:up|Adm,11535:up|Sfrp1,20377:up|Ptk7,71461:down|Wnt5a,22418:down|Bmp4,12159:up
positive regulation of Wnt signaling pathway	GO:0030177	1.47E-03	9.48E-03	Sfrp2,20319:up|Sfrp1,20377:up|Sulf1,240725:down|Wnt5a,22418:down|Rspo2,239405:up|Sfrp4,20379:up|Fgfr2,14183:up
positive regulation of transmembrane receptor protein serine/threonine kinase signaling pathway	GO:0090100	1.47E-03	9.48E-03	Sulf1,240725:down|Tgfb2,21808:down|Inhba,16323:down|Bmpr1a,12166:down|Tgfbr3,21814:up|Npnt,114249:up|Bmp4,12159:up
regulation of blood vessel size	GO:0050880	1.49E-03	9.56E-03	Htr2b,15559:down|Kng1,16644:up|Adm,11535:up|Egfr,13649:down|Tgfbr3,21814:up|Ptgs2,19225:down|Ednrb,13618:up|Htr2a,15558:down|Mmp2,17390:down
sensory perception of pain	GO:0019233	1.51E-03	9.69E-03	Tnf,21926:up|Fgfr1,14182:down|Ncam1,17967:down|Mme,17380:up|Ptgs2,19225:down|Il1rn,16181:up|Ednrb,13618:up|Htr2a,15558:down
regulation of keratinocyte differentiation	GO:0045616	1.52E-03	9.69E-03	Vdr,22337:down|Tgfb2,21808:down|Cd109,235505:down|Errfi1,74155:down
positive regulation of leukocyte apoptotic process	GO:2000108	1.52E-03	9.69E-03	Ceacam1,26365:up|Cd24a,12484:down|Wnt5a,22418:down|Tgfb2,21808:down
positive regulation of tissue remodeling	GO:0034105	1.52E-03	9.69E-03	Vdr,22337:down|Trf,22041:up|Egfr,13649:down|Tfrc,22042:up
negative regulation of cellular metabolic process	GO:0031324	1.52E-03	9.69E-03	Flrt2,399558:down|Sfrp4,20379:up|Ednrb,13618:up|Cst7,13011:up|Epas1,13819:up|Ankrd1,107765:down|Igfbp3,16009:up|Cd109,235505:down|Loxl2,94352:down|Loxl3,16950:down|Akr1c18,105349:up|Ptx3,19288:up|Wnt5a,22418:down|Ccl4,20303:up|Adipoq,11450:up|Bmp4,12159:up|Igf1,16000:up|Prg4,96875:down|Tgfb2,21808:down|Plpp3,67916:up|Fgfr2,14183:up|Htr2b,15559:down|Kng1,16644:up|Hp,15439:up|Slpi,20568:up|Aspn,66695:down|Plat,18791:down|Tfpi2,21789:down|Vdr,22337:down|Ctla2a,13024:down|Fabp4,11770:up|Vegfa,22339:down|Serpine2,20720:up|Lrrc15,74488:down|Nxn,18230:down|Pparg,19016:up|Gas6,14456:up|Tnf,21926:up|Lmcd1,30937:up|Fgfr1,14182:down|Egfr,13649:down|Hmox1,15368:up|Sfrp1,20377:up|C1qtnf3,81799:down|Grem1,23892:up|Ceacam1,26365:up|Sfrp2,20319:up|Serping1,12258:up|Serpina3g,20715:up|Prkar2b,19088:up|Errfi1,74155:down|Enpp1,18605:down|S100a1,20193:up
negative regulation of myeloid cell differentiation	GO:0045638	1.53E-03	9.69E-03	Fstl3,83554:down|Ceacam1,26365:up|Clec2d,93694:up|Lrrc17,74511:down|Sfrp1,20377:up|Adipoq,11450:up
regulation of cytokine-mediated signaling pathway	GO:0001959	1.53E-03	9.69E-03	Pparg,19016:up|Gas6,14456:up|Cd24a,12484:down|Wnt5a,22418:down|Il1rn,16181:up|Adipoq,11450:up
negative regulation of ossification	GO:0030279	1.53E-03	9.69E-03	Fam101b,76566:down|Tnf,21926:up|Fgfr1,14182:down|Sfrp1,20377:up|Enpp1,18605:down|Grem1,23892:up
cardiac ventricle morphogenesis	GO:0003208	1.53E-03	9.69E-03	Sfrp2,20319:up|Col11a1,12814:down|Tgfb2,21808:down|Bmpr1a,12166:down|Tgfbr3,21814:up|Fgfr2,14183:up
detection of mechanical stimulus	GO:0050982	1.53E-03	9.71E-03	Tnf,21926:up|Col11a1,12814:down|Htr2a,15558:down|Serpine2,20720:up|Igf1,16000:up
positive regulation of reactive oxygen species biosynthetic process	GO:1903428	1.53E-03	9.71E-03	Tnf,21926:up|Ptx3,19288:up|Ddah1,69219:down|Egfr,13649:down|Ptgs2,19225:down
establishment of planar polarity	GO:0001736	1.53E-03	9.71E-03	Cthrc1,68588:down|Sfrp2,20319:up|Sfrp1,20377:up|Ptk7,71461:down|Wnt5a,22418:down
regulation of tube size	GO:0035150	1.56E-03	9.85E-03	Htr2b,15559:down|Kng1,16644:up|Adm,11535:up|Egfr,13649:down|Tgfbr3,21814:up|Ptgs2,19225:down|Ednrb,13618:up|Htr2a,15558:down|Mmp2,17390:down

cardiac septum development	GO:0003279	1.57E-03	9.88E-03	Plxnd1,67784:up|Ptk7,71461:down|Wnt5a,22418:down|Tgfb2,21808:down|Bmpr1a,12166:down|Fgfr2,14183:up|Bmp4,12159:up
regulation of striated muscle cell differentiation	GO:0051153	1.57E-03	9.88E-03	Ccl8,20307:up|Cxcl9,17329:up|Cxcl12,20315:up|Wisp1,22402:down|Grem1,23892:up|Igf1,16000:up|Bmp4,12159:up
tube closure	GO:0060606	1.57E-03	9.88E-03	Cthrc1,68588:down|Sfrp2,20319:up|Adm,11535:up|Sfrp1,20377:up|Ptk7,71461:down|Wnt5a,22418:down|Bmp4,12159:up
protein import	GO:0017038	1.58E-03	9.95E-03	Tnf,21926:up|Gas6,14456:up|Akr1c18,105349:up|Egfr,13649:down|C1qtnf3,81799:down|Bmpr1a,12166:down|Ptgs2,19225:down|Grem1,23892:up|Igf1,16000:up|Bmp4,12159:up|Ceacam1,26365:up|Sfrp2,20319:up|Tgfb2,21808:down
regulation of striated muscle tissue development	GO:0016202	1.59E-03	1.00E-02	Fgfr1,14182:down|Ncam1,17967:down|Bmpr1a,12166:down|Wisp1,22402:down|Grem1,23892:up|Igf1,16000:up|Bmp4,12159:up|Fgfr2,14183:up
gliogenesis	GO:0042063	1.60E-03	1.00E-02	Pparg,19016:up|Trf,22041:up|Egfr,13649:down|Efemp1,216616:up|Ccl2,20296:up|Ccl12,20293:up|Ccl3,20302:up|Igf1,16000:up|Bmp4,12159:up|Tgfb2,21808:down|Plpp3,67916:up|Serpine2,20720:up
regulation of blood circulation	GO:1903522	1.62E-03	1.01E-02	Glp1r,14652:down|Fxyd1,56188:up|Adm,11535:up|Egfr,13649:down|Ptgs2,19225:down|Stc1,20855:up|Epas1,13819:up|Tgfb2,21808:down|Htr2a,15558:down|Mmp2,17390:down|S100a1,20193:up
striated muscle tissue development	GO:0014706	1.66E-03	1.04E-02	Fgfr1,14182:down|Col11a1,12814:down|Ncam1,17967:down|Wnt5a,22418:down|Bmpr1a,12166:down|Tgfbr3,21814:up|Aldh1a2,19378:up|Wisp1,22402:down|Grem1,23892:up|Igf1,16000:up|Bmp4,12159:up|Ankrd1,107765:down|Vegfa,22339:down|Tgfb2,21808:down|Fgfr2,14183:up
inner ear morphogenesis	GO:0042472	1.67E-03	1.04E-02	Cthrc1,68588:down|Fgfr1,14182:down|Col11a1,12814:down|Ptk7,71461:down|Wnt5a,22418:down|Aldh1a3,56847:down|Fgfr2,14183:up
establishment of tissue polarity	GO:0007164	1.69E-03	1.05E-02	Cthrc1,68588:down|Sfrp2,20319:up|Sfrp1,20377:up|Ptk7,71461:down|Wnt5a,22418:down
negative regulation of transforming growth factor beta receptor signaling pathway	GO:0030512	1.69E-03	1.05E-02	Htra3,78558:down|Chst11,58250:down|Tgfbr3,21814:up|Cd109,235505:down|Aspn,66695:down
positive regulation of neural precursor cell proliferation	GO:2000179	1.69E-03	1.05E-02	Prl2c2,18811:up|Trf,22041:up|Vegfa,22339:down|Cx3cr1,13051:down|Igf1,16000:up
neural precursor cell proliferation	GO:0061351	1.71E-03	1.06E-02	Prl2c2,18811:up|Trf,22041:up|Fgfr1,14182:down|Cd24a,12484:down|Wnt5a,22418:down|Igf1,16000:up|Vegfa,22339:down|Cx3cr1,13051:down|Fgfr2,14183:up
regulation of cardiac muscle cell differentiation	GO:2000725	1.74E-03	1.08E-02	Wisp1,22402:down|Grem1,23892:up|Igf1,16000:up|Bmp4,12159:up
regulation of endothelial cell differentiation	GO:0045601	1.74E-03	1.08E-02	Ceacam1,26365:up|Tnf,21926:up|Vegfa,22339:down|Bmp4,12159:up
monosaccharide metabolic process	GO:0005996	1.74E-03	1.08E-02	Tnf,21926:up|Igfbp4,16010:up|Fabp5,16592:up|C1qtnf3,81799:down|Igf1,16000:up|Adipoq,11450:up|Sord,20322:up|Igfbp3,16009:up|Enpp1,18605:down|Igf2,16002:up|Man2a2,140481:up
regulation of muscle organ development	GO:0048634	1.77E-03	1.09E-02	Fgfr1,14182:down|Ncam1,17967:down|Bmpr1a,12166:down|Wisp1,22402:down|Grem1,23892:up|Igf1,16000:up|Bmp4,12159:up|Fgfr2,14183:up
viral genome replication	GO:0019079	1.77E-03	1.09E-02	Ceacam1,26365:up|Tnf,21926:up|Gas6,14456:up|Oasl1,231655:up|Slpi,20568:up|Oas3,246727:up
immunoglobulin mediated immune response	GO:0016064	1.77E-03	1.09E-02	Tnf,21926:up|C1s1,50908:up|Serping1,12258:up|C1s2,317677:up|C3,12266:up|C1rb,667277:up|Tfrc,22042:up
endothelium development	GO:0003158	1.77E-03	1.09E-02	Ceacam1,26365:up|Tnf,21926:up|Vegfa,22339:down|Stc1,20855:up|Adamts12,239337:down|Slc40a1,53945:up|Bmp4,12159:up
regulation of leukocyte mediated immunity	GO:0002703	1.79E-03	1.10E-02	Tnf,21926:up|Clec2d,93694:up|Cd24a,12484:down|Hmox1,15368:up|Ccl2,20296:up|C3,12266:up|Tfrc,22042:up|Ceacam1,26365:up|Igf2,16002:up
regulation of cell-substrate adhesion	GO:0010810	1.79E-03	1.10E-02	Has2,15117:down|Col8a1,12837:down|Grem1,23892:up|Ninj1,18081:up|Gpm6b,14758:up|Vegfa,22339:down|Postn,50706:down|Npnt,114249:up|Smoc2,64074:down
negative regulation of kinase activity	GO:0033673	1.81E-03	1.11E-02	Flrt2,399558:down|Sfrp1,20377:up|Aspn,66695:down|Bmp4,12159:up|Adipoq,11450:up|Ceacam1,26365:up|Sfrp2,20319:up|Fabp4,11770:up|Prkar2b,19088:up|Lrrc15,74488:down|Errfi1,74155:down
anion transport	GO:0006820	1.81E-03	1.11E-02	Sfrp4,20379:up|Gpm6b,14758:up|Fam132b,227358:down|Cxcl12,20315:up|Htr2a,15558:down|Slc16a4,229699:down|Pparg,19016:up|Tnf,21926:up|Fxyd1,56188:up|Fgfr1,14182:down|Il1rn,16181:up|Stc1,20855:up|Sncg,20618:up|Serinc2,230779:down|Maob,109731:up|Ank,11732:down|Emb,13723:up|Enpp1,18605:down
positive regulation of cell adhesion mediated by integrin	GO:0033630	1.83E-03	1.12E-02	Cd24a,12484:down|Ret,19713:up|Tgfb2,21808:down
regulation of mesoderm development	GO:2000380	1.83E-03	1.12E-02	Sfrp2,20319:up|Fgfr1,14182:down|Bmpr1a,12166:down
vasculogenesis involved in coronary vascular morphogenesis	GO:0060979	1.83E-03	1.12E-02	Fgfr1,14182:down|Tgfbr3,21814:up|Fgfr2,14183:up
negative regulation of bone resorption	GO:0045779	1.83E-03	1.12E-02	Ceacam1,26365:up|Vegfa,22339:down|Tnfrsf11b,18383:up
negative regulation of cellular response to transforming growth factor beta stimulus	GO:1903845	1.85E-03	1.13E-02	Htra3,78558:down|Chst11,58250:down|Tgfbr3,21814:up|Cd109,235505:down|Aspn,66695:down
lung alveolus development	GO:0048286	1.85E-03	1.13E-02	Vegfa,22339:down|Errfi1,74155:down|Fgfr2,14183:up|Igf1,16000:up|Bmp4,12159:up
regulation of muscle tissue development	GO:1901861	1.86E-03	1.13E-02	Fgfr1,14182:down|Ncam1,17967:down|Bmpr1a,12166:down|Wisp1,22402:down|Grem1,23892:up|Igf1,16000:up|Bmp4,12159:up|Fgfr2,14183:up

positive regulation of immune effector process	GO:0002699	1.87E-03	1.14E-02	Tnf,21926:up|Cd24a,12484:down|Hmox1,15368:up|Wnt5a,22418:down|Ccl2,20296:up|C3,12266:up|Tfrc,22042:up|Sema7a,20361:down|Ceacam1,26365:up
regulation of JUN kinase activity	GO:0043506	1.90E-03	1.14E-02	Tnf,21926:up|Trf,22041:up|Sfrp2,20319:up|Sfrp1,20377:up|Wnt5a,22418:down|Il1rn,16181:up
receptor-mediated virion attachment to host cell	GO:0046813	1.91E-03	1.14E-02	Gas6,14456:up|Lrrc15,74488:down
positive regulation of phosphatidylinositol biosynthetic process	GO:0010513	1.91E-03	1.14E-02	Htr2b,15559:down|Htr2a,15558:down
response to prostaglandin D	GO:0071798	1.91E-03	1.14E-02	Akr1c18,105349:up|Ptgir,19222:up
porphyrin-containing compound catabolic process	GO:0006787	1.91E-03	1.14E-02	Hmox1,15368:up|Blvrb,233016:up
negative regulation of alkaline phosphatase activity	GO:0010693	1.91E-03	1.14E-02	Tnf,21926:up|Tgfb2,21808:down
tetrapyrrole catabolic process	GO:0033015	1.91E-03	1.14E-02	Hmox1,15368:up|Blvrb,233016:up
cellular response to cadmium ion	GO:0071276	1.91E-03	1.14E-02	Akr1c18,105349:up|Hmox1,15368:up
cellular response to prostaglandin D stimulus	GO:0071799	1.91E-03	1.14E-02	Akr1c18,105349:up|Ptgir,19222:up
positive regulation of ovulation	GO:0060279	1.91E-03	1.14E-02	Inhba,16323:down|Plat,18791:down
detoxification of copper ion	GO:0010273	1.91E-03	1.14E-02	Mt2,17750:up|Mt1,17748:up
negative regulation of branching involved in ureteric bud morphogenesis	GO:0090191	1.91E-03	1.14E-02	Grem1,23892:up|Bmp4,12159:up
stress response to copper ion	GO:1990169	1.91E-03	1.14E-02	Mt2,17750:up|Mt1,17748:up
peripheral nervous system axon regeneration	GO:0014012	1.91E-03	1.14E-02	Tnc,21923:down|Mmp2,17390:down
positive regulation of natural killer cell chemotaxis	GO:2000503	1.91E-03	1.14E-02	Cxcl14,57266:down|Ccl4,20303:up
ureter maturation	GO:0035799	1.91E-03	1.14E-02	Ret,19713:up|Aldh1a2,19378:up
single-organism transport	GO:0044765	1.94E-03	1.16E-02	Adm,11535:up|Sfrp4,20379:up|C3,12266:up|Ankrd1,107765:down|Trf,22041:up|Cd24a,12484:down|Wnt5a,22418:down|Stc1,20855:up|Il1rn,16181:up|Maob,109731:up|Adipoq,11450:up|Bmp4,12159:up|Igf1,16000:up|Mcoln2,68279:up|Tgfb2,21808:down|Fgfr2,14183:up|Clec4e,56619:up|Htr2b,15559:down|Vdr,22337:down|Fam132b,227358:down|Cxcl12,20315:up|Htr2a,15558:down|Slc16a4,229699:down|Slc40a1,53945:up|Gas6,14456:up|Egfr,13649:down|Sec23a,20334:down|Sfrp1,20377:up|C1qtnf3,81799:down|Tfrc,22042:up|Fxyd2,11936:up|Sncg,20618:up|Serinc2,230779:down|Ceacam1,26365:up|Sfrp2,20319:up|Syt13,80976:up|S100a1,20193:up|Ccl2,20296:up|Ednrb,13618:up|Mmp13,17386:up|Slc29a1,63959:up|Gpm6b,14758:up|Clec4n,56620:up|Steap1,70358:down|Postn,50706:down|Cdo1,12583:up|Ptx3,19288:up|Hvcn1,74096:up|Akr1c18,105349:up|Fabp5,16592:up|Ncam1,17967:down|Ccl12,20293:up|Bmpr1a,12166:down|Ccl4,20303:up|Ank,11732:down|Creb3l1,26427:down|Il1rl1,17082:down|Inhba,16323:down|Ccl3,20302:up|Vegfa,22339:down|Serpine2,20720:up|Anpep,16790:up|Pparg,19016:up|Tnf,21926:up|Fxyd1,56188:up|Glp1r,14652:down|Fgfr1,14182:down|Lpl,16956:up|Hmox1,15368:up|Cd4,12504:up|Ptgs2,19225:down|Grem1,23892:up|Kcnj15,16516:down|Mcoln3,171166:up|Emb,13723:up|Enpp1,18605:down
retinoic acid receptor signaling pathway	GO:0048384	1.98E-03	1.18E-02	Crabp2,12904:down|Akr1c18,105349:up|Aldh1a2,19378:up|Aldh1a3,56847:down
cellular response to organonitrogen compound	GO:0071417	1.99E-03	1.18E-02	Htr2b,15559:down|Col5a2,12832:down|Tnf,21926:up|Pparg,19016:up|Col1a2,12843:down|Egfr,13649:down|Sfrp1,20377:up|Pdgfc,54635:down|Col3a1,12825:down|Igf1,16000:up|Adipoq,11450:up|Mmp2,17390:down|Enpp1,18605:down|Igf2,16002:up
primary neural tube formation	GO:0014020	1.99E-03	1.18E-02	Cthrc1,68588:down|Sfrp2,20319:up|Adm,11535:up|Sfrp1,20377:up|Ptk7,71461:down|Wnt5a,22418:down|Bmp4,12159:up
B cell mediated immunity	GO:0019724	1.99E-03	1.18E-02	Tnf,21926:up|C1s1,50908:up|Serping1,12258:up|C1s2,317677:up|C3,12266:up|C1rb,667277:up|Tfrc,22042:up
protein activation cascade	GO:0072376	2.03E-03	1.20E-02	C1s1,50908:up|Serping1,12258:up|C1s2,317677:up|C3,12266:up|C1rb,667277:up
negative regulation of wound healing	GO:0061045	2.03E-03	1.20E-02	Ceacam1,26365:up|Kng1,16644:up|Serping1,12258:up|Serpine2,20720:up|Cd109,235505:down
epidermal cell differentiation	GO:0009913	2.04E-03	1.21E-02	Fgfr1,14182:down|Sfrp4,20379:up|Ptgs2,19225:down|Bmp4,12159:up|Vdr,22337:down|Mcoln3,171166:up|Tgfb2,21808:down|Cd109,235505:down|Errfi1,74155:down
cognition	GO:0050890	2.09E-03	1.24E-02	Plk2,20620:down|Glp1r,14652:down|Egfr,13649:down|Ncam1,17967:down|Ptgs2,19225:down|Il1rn,16181:up|Igf1,16000:up|Shroom4,208431:down|Htr2a,15558:down|Prkar2b,19088:up|Cx3cr1,13051:down|Igf2,16002:up
positive regulation of calcium ion transport	GO:0051928	2.11E-03	1.25E-02	Cxcl12,20315:up|Ccl12,20293:up|Cd4,12504:up|Stc1,20855:up|Ccl4,20303:up|S100a1,20193:up|Bmp4,12159:up
regulation of glucose metabolic process	GO:0010906	2.11E-03	1.25E-02	Igfbp4,16010:up|C1qtnf3,81799:down|Igfbp3,16009:up|Enpp1,18605:down|Igf2,16002:up|Igf1,16000:up|Adipoq,11450:up

sensory organ morphogenesis	GO:0090596	2.16E-03	1.27E-02	Cthrc1,68588:down|Col5a2,12832:down|Fgfr1,14182:down|Col11a1,12814:down|Ptk7,71461:down|Wnt5a,22418:down|Col8a1,12837:down|Bmp4,12159:up|Col5a1,12831:down|Vegfa,22339:down|Aldh1a3,56847:down|Fgfr2,14183:up
regulation of Wnt signaling pathway	GO:0030111	2.17E-03	1.28E-02	Cthrc1,68588:down|Sfrp1,20377:up|Sulf1,240725:down|Wnt5a,22418:down|Sfrp4,20379:up|Grem1,23892:up|Sfrp2,20319:up|Rspo2,239405:up|Plpp3,67916:up|Fgfr2,14183:up|Nxn,18230:down
regulation of response to cytokine stimulus	GO:0060759	2.19E-03	1.29E-02	Pparg,19016:up|Gas6,14456:up|Cd24a,12484:down|Wnt5a,22418:down|Il1rn,16181:up|Adipoq,11450:up
ventricular cardiac muscle tissue development	GO:0003229	2.22E-03	1.30E-02	Col11a1,12814:down|Tgfb2,21808:down|Bmpr1a,12166:down|Tgfbr3,21814:up|Fgfr2,14183:up
regulation of interleukin-1 production	GO:0032652	2.22E-03	1.30E-02	Ceacam1,26365:up|Gas6,14456:up|Wnt5a,22418:down|Errfi1,74155:down|Ccl3,20302:up
face development	GO:0060324	2.22E-03	1.30E-02	Wnt5a,22418:down|Tgfb2,21808:down|Aldh1a2,19378:up|Aldh1a3,56847:down|Mmp2,17390:down
cell fate commitment involved in formation of primary germ layer	GO:0060795	2.24E-03	1.31E-02	Sfrp2,20319:up|Fgfr1,14182:down|Bmpr1a,12166:down|Bmp4,12159:up
positive chemotaxis	GO:0050918	2.24E-03	1.31E-02	Cxcl12,20315:up|Wnt5a,22418:down|Vegfa,22339:down|Figf,14205:up
cell migration involved in sprouting angiogenesis	GO:0002042	2.24E-03	1.31E-02	Srpx2,68792:down|Vegfa,22339:down|Ptgs2,19225:down|Grem1,23892:up
negative regulation of cytokine-mediated signaling pathway	GO:0001960	2.24E-03	1.31E-02	Pparg,19016:up|Gas6,14456:up|Il1rn,16181:up|Adipoq,11450:up
regulation of retinoic acid receptor signaling pathway	GO:0048385	2.26E-03	1.31E-02	Crabp2,12904:down|Akr1c18,105349:up|Aldh1a3,56847:down
vitamin D metabolic process	GO:0042359	2.26E-03	1.31E-02	Vdr,22337:down|Tnf,21926:up|Fgfr1,14182:down
regulation of glomerulus development	GO:0090192	2.26E-03	1.31E-02	Ret,19713:up|Bmp4,12159:up|Adipoq,11450:up
ureter development	GO:0072189	2.26E-03	1.31E-02	Ret,19713:up|Aldh1a2,19378:up|Bmp4,12159:up
regulation of cell fate specification	GO:0042659	2.26E-03	1.31E-02	Sfrp2,20319:up|Fgfr1,14182:down|Bmpr1a,12166:down
embryonic skeletal system development	GO:0048706	2.27E-03	1.32E-02	Col11a1,12814:down|Sulf1,240725:down|Wnt5a,22418:down|Bmp4,12159:up|Chst11,58250:down|Tbx15,21384:down|Tgfb2,21808:down|Fgfr2,14183:up
regulation of intracellular protein transport	GO:0033157	2.30E-03	1.33E-02	Tnf,21926:up|Gas6,14456:up|Ice1,218333:down|Egfr,13649:down|C1qtnf3,81799:down|Bmpr1a,12166:down|Ccl2,20296:up|Ptgs2,19225:down|Grem1,23892:up|Igf1,16000:up|Bmp4,12159:up|Adipoq,11450:up|Ceacam1,26365:up|Sfrp2,20319:up
cellular protein metabolic process	GO:0044267	2.34E-03	1.35E-02	Igfbp4,16010:up|Flrt2,399558:down|Sfrp4,20379:up|C3,12266:up|Igfbp3,16009:up|Cd109,235505:down|Loxl2,94352:down|Plk2,20620:down|Trf,22041:up|Cd24a,12484:down|Fkbp14,231997:down|Wnt5a,22418:down|Mark1,226778:up|Il1rn,16181:up|Adipoq,11450:up|Bmp4,12159:up|Igf1,16000:up|Tgfb2,21808:down|F3,14066:up|Fgfr2,14183:up|Htr2b,15559:down|Kng1,16644:up|Efemp1,216616:up|Ccl6,20305:up|Slpi,20568:up|Aspn,66695:down|Saa3,20210:up|Pdia5,72599:down|Fabp4,11770:up|Htr2a,15558:down|Gas6,14456:up|Egfr,13649:down|Sfrp1,20377:up|C1qtnf3,81799:down|Tgfbr3,21814:up|Ceacam1,26365:up|Ccl8,20307:up|Sfrp2,20319:up|Serpina3g,20715:up|Fam20a,208659:down|Npnt,114249:up|Tnfrsf11b,18383:up|Prkar2b,19088:up|Neurl3,214854:down|Ccl2,20296:up|Ccl7,20306:up|Pdzrn3,55983:down|Pdgfc,54635:down|Cst7,13011:up|Ednrb,13618:up|Mmp13,17386:up|Sema7a,20361:down|Uchl5,56207:up|Ddah1,69219:down|Fkbp10,14230:down|Dok2,13449:up|Man2a2,140481:up|Mamdc2,71738:down|Ccl12,20293:up|Bmpr1a,12166:down|Ccl4,20303:up|Ret,19713:up|Vegfb,22340:up|Sdf2l1,64136:up|Plpp3,67916:up|Kitl,17311:up|P4ha1,18451:down|Ccl9,20308:up|Inhba,16323:down|Plat,18791:down|Ccl3,20302:up|Tfpi2,21789:down|Ctla2a,13024:down|Crlf1,12931:down|Vegfa,22339:down|Serpine2,20720:up|Adamts12,239337:down|Lrrc15,74488:down|Igf2,16002:up|Nxn,18230:down|Pparg,19016:up|Tnf,21926:up|Fgfr1,14182:down|Cd4,12504:up|Grem1,23892:up|Col3a1,12825:down|Serping1,12258:up|Errfi1,74155:down|Enpp1,18605:down
regulation of production of molecular mediator of immune response	GO:0002700	2.37E-03	1.37E-02	Ceacam1,26365:up|Tnf,21926:up|Hmox1,15368:up|Wnt5a,22418:down|Tgfb2,21808:down|Tfrc,22042:up|Sema7a,20361:down
positive regulation of leukocyte proliferation	GO:0070665	2.38E-03	1.38E-02	Cd24a,12484:down|Cd4,12504:up|Tfrc,22042:up|Igf1,16000:up|Ceacam1,26365:up|Igf2,16002:up|Kitl,17311:up|Tnfsf9,21950:up

biological_process	GO:0008150	2.41E-03	1.39E-02	Fstl3,83554:down|Igfbp4,16010:up|Adm,11535:up|Flrt2,399558:down|Tmem176b,65963:up|Plxnd1,67784:up|Tfec,21426:up|Sfrp4,20379:up|Ltbp2,16997:down|Serpinh1,12406:down|Lyl1,17095:up|Gstt1,14871:up|Blvrb,233016:up|Igfbp3,16009:up|Ibsp,15891:up|Plk2,20620:down|Cd24a,12484:down|Mme,17380:up|Fkbp14,231997:down|Il1rn,16181:up|Maob,109731:up|Bmp4,12159:up|Col12a1,12816:down|Oasl1,231655:up|Hpse,15442:up|Medag,70717:down|Ptgir,19222:up|Fgfr2,14183:up|Thbs2,21826:down|Htr2b,15559:down|Mfsd12,73822:up|Dram1,71712:up|Ccl6,20305:up|Gjb5,14622:down|Anxa8,11752:down|Slpi,20568:up|Aspn,66695:down|Mt2,17750:up|Cxcr6,80901:down|Sned1,208777:up|Fam132b,227358:down|Nabp1,109019:down|Phf11a,219131:up|Mmp2,17390:down|Oas3,246727:up|Pappa,18491:down|Slc40a1,53945:up|Egfr,13649:down|Alpl,11647:up|C1rb,667277:up|Tfrc,22042:up|Sncg,20618:up|Ccl8,20307:up|Gstm1,14862:up|Serpina3g,20715:up|Rspo2,239405:up|Fam20a,208659:down|Cdsn,386463:up|Syt13,80976:up|Mt1,17748:up|Col5a2,12832:down|Mrc1,17533:up|Ccl2,20296:up|Aldh1a2,19378:up|Ednrb,13618:up|Ampd3,11717:up|Mmp13,17386:up|Htra3,78558:down|Tnfrsf12a,27279:down|Uchl5,56207:up|Sord,20322:up|Clec4n,56620:up|Shroom4,208431:down|Steap1,70358:down|Enpp3,209558:down|Cdo1,12583:up|Dok2,13449:up|Mamdc2,71738:down|Csrp2,13008:down|Hvcn1,74096:up|Ptx3,19288:up|Ncam1,17967:down|Ccl4,20303:up|Chst11,58250:down|Tmeff1,230157:down|Vegfb,22340:up|Aldh1a3,56847:down|Plpp3,67916:up|Mrc2,17534:down|Lrrc17,74511:down|Lama2,16773:down|Ccl9,20308:up|Ptk7,71461:down|Inhba,16323:down|Gsta4,14860:up|Bnc2,242509:down|Tfpi2,21789:down|Crlf1,12931:down|C1s1,50908:up|Plod2,26432:down|Vegfa,22339:down|Gfpt2,14584:down|Serpine2,20720:up|Cbr2,12409:up|Nxn,18230:down|Igf2,16002:up|Kdelr3,105785:down|Tmem176a,66058:up|Tnf,21926:up|Col1a2,12843:down|Fxyd1,56188:up|Fgfr1,14182:down|Lpl,16956:up|Sulf1,240725:down|Susd2,71733:up|Cd4,12504:up|Col3a1,12825:down|Tbx15,21384:down|Serping1,12258:up|Emb,13723:up|Uck2,80914:down|Enpp1,18605:down|Clec2d,93694:up|Hpgd,15446:up|C3,12266:up|Enpp5,83965:up|Ankrd1,107765:down|Cx3cr1,13051:down|Cd109,235505:down|Ggt5,23887:up|Loxl2,94352:down|Loxl3,16950:down|Trf,22041:up|Cxcl14,57266:down|Adam12,11489:down|Ceacam10,26366:up|Wnt5a,22418:down|Mark1,226778:up|Stc1,20855:up|Col8a1,12837:down|Adipoq,11450:up|Hal,15109:up|Igf1,16000:up|Crabp2,12904:down|Mcoln2,68279:up|Cxcl9,17329:up|Tgfb2,21808:down|F3,14066:up|Clec4e,56619:up|Adamts4,240913:down|Cthrc1,68588:down|Kng1,16644:up|Prl2c2,18811:up|Efemp1,216616:up|Dpysl3,22240:down|Ninj1,18081:up|Saa3,20210:up|Gdpd1,66569:up|Vdr,22337:down|Pdia5,72599:down|Fabp4,11770:up|Cxcl12,20315:up|Htr2a,15558:down|Slc16a4,229699:down|Smoc2,64074:down|Gas6,14456:up|Col11a1,12814:down|Sec23a,20334:down|Sfrp1,20377:up|C1qtnf3,81799:down|Tgfbr3,21814:up|Fxyd2,11936:up|Serinc2,230779:down|Tll1,21892:down|Ceacam1,26365:up|Sfrp2,20319:up|Pxdn,69675:down|Pamr1,210622:up|Tnfrsf11b,18383:up|Prkar2b,19088:up|Npnt,114249:up|S100a1,20193:up|Ice1,218333:down|Ly9,17085:up|Neurl3,214854:down|Ccl7,20306:up|Pdzrn3,55983:down|Pdgfc,54635:down|Mgp,17313:up|Figf,14205:up|Cst7,13011:up|Ifit3,15959:up|Sema7a,20361:down|Epas1,13819:up|Slc29a1,63959:up|Marc1,66112:up|Gpm6b,14758:up|Ddah1,69219:down|Sdpr,20324:down|Fkbp10,14230:down|Postn,50706:down|Hspb7,29818:up|Npl,74091:up|Man2a2,140481:up|Akr1c18,105349:up|Fabp5,16592:up|Bmpr1a,12166:down|Ccl12,20293:up|Wisp1,22402:down|Klk1b11,16613:up|Ank,11732:down|Tnc,21923:down|Gatm,67092:down|Ret,19713:up|Prg4,96875:down|C1s2,317677:up|Pdxk,216134:up|Sdf2l1,64136:up|Ifit1,15957:up|Creb3l1,26427:down|Il1rl1,17082:down|Kitl,17311:up|Fam101b,76566:down|P4ha1,18451:down|Hp,15439:up|Aoah,27052:up|Rasl11a,68895:up|Plat,18791:down|Ccl3
positive regulation of homotypic cell-cell adhesion	GO:0034112	2.43E-03	1.40E-02	Cd24a,12484:down|Has2,15117:down|Ccl2,20296:up|Cd4,12504:up|Tfrc,22042:up|Igf1,16000:up|Ceacam1,26365:up|Igf2,16002:up|Tnfsf9,21950:up
regulation of carbohydrate metabolic process	GO:0006109	2.43E-03	1.40E-02	Igfbp4,16010:up|Has2,15117:down|C1qtnf3,81799:down|Igf1,16000:up|Adipoq,11450:up|Htr2a,15558:down|Igfbp3,16009:up|Igf2,16002:up|Enpp1,18605:down
regulation of protein targeting	GO:1903533	2.46E-03	1.42E-02	Tnf,21926:up|Gas6,14456:up|Egfr,13649:down|C1qtnf3,81799:down|Bmpr1a,12166:down|Ccl2,20296:up|Ptgs2,19225:down|Grem1,23892:up|Igf1,16000:up|Bmp4,12159:up|Ceacam1,26365:up|Sfrp2,20319:up
negative regulation of transferase activity	GO:0051348	2.46E-03	1.42E-02	Pparg,19016:up|Flrt2,399558:down|Sfrp1,20377:up|Aspn,66695:down|Bmp4,12159:up|Adipoq,11450:up|Ceacam1,26365:up|Sfrp2,20319:up|Fabp4,11770:up|Prkar2b,19088:up|Lrrc15,74488:down|Errfi1,74155:down
regulation of cell projection organization	GO:0031344	2.49E-03	1.43E-02	Prl2c2,18811:up|Efemp1,216616:up|Plxnd1,67784:up|Ptk7,71461:down|Dpysl3,22240:down|Sema7a,20361:down|Ankrd1,107765:down|Tnfrsf12a,27279:down|Cxcl12,20315:up|Vegfa,22339:down|Serpine2,20720:up|Plk2,20620:down|Cd24a,12484:down|Fgfr1,14182:down|Sfrp1,20377:up|Wnt5a,22418:down|Bmp4,12159:up|Sfrp2,20319:up|Crabp2,12904:down|Ret,19713:up
negative regulation of multi-organism process	GO:0043901	2.50E-03	1.43E-02	Tnf,21926:up|Ptx3,19288:up|Slpi,20568:up|Ccl4,20303:up|Igf1,16000:up|Ceacam1,26365:up|Oasl1,231655:up|Oas3,246727:up
regeneration	GO:0031099	2.50E-03	1.43E-02	Adm,11535:up|Tnc,21923:down|Cxcl12,20315:up|Hmox1,15368:up|Mmp2,17390:down|Ninj1,18081:up|Igf1,16000:up
negative regulation of stress-activated protein kinase signaling cascade	GO:0070303	2.52E-03	1.44E-02	Ceacam1,26365:up|Sfrp2,20319:up|Sfrp1,20377:up|Sfrp4,20379:up
regulation of collagen biosynthetic process	GO:0032965	2.52E-03	1.44E-02	Pparg,19016:up|Ccl2,20296:up|Errfi1,74155:down|Bmp4,12159:up
negative regulation of stress-activated MAPK cascade	GO:0032873	2.52E-03	1.44E-02	Ceacam1,26365:up|Sfrp2,20319:up|Sfrp1,20377:up|Sfrp4,20379:up
regulation of polysaccharide biosynthetic process	GO:0032885	2.52E-03	1.44E-02	Has2,15117:down|Enpp1,18605:down|Igf2,16002:up|Igf1,16000:up
proteolysis	GO:0006508	2.52E-03	1.44E-02	C3,12266:up|Cst7,13011:up|Mmp13,17386:up|Htra3,78558:down|Uchl5,56207:up|Cd109,235505:down|Ggt5,23887:up|Plk2,20620:down|Adam12,11489:down|Mme,17380:up|Igf1,16000:up|Klk1b11,16613:up|Ret,19713:up|C1s2,317677:up|Sdf2l1,64136:up|F3,14066:up|Adamts4,240913:down|Kng1,16644:up|Hp,15439:up|Slpi,20568:up|Plat,18791:down|Tfpi2,21789:down|Ctla2a,13024:down|C1s1,50908:up|Vegfa,22339:down|Serpine2,20720:up|Mmp2,17390:down|Adamts12,239337:down|Anpep,16790:up|Pappa,18491:down|Pparg,19016:up|Gas6,14456:up|Tnf,21926:up|C1rb,667277:up|Tll1,21892:down|Sfrp2,20319:up|Serping1,12258:up|Serpina3g,20715:up|Pamr1,210622:up
negative regulation of cell growth	GO:0030308	2.54E-03	1.45E-02	Pparg,19016:up|Sfrp1,20377:up|Wnt5a,22418:down|Inhba,16323:down|Grem1,23892:up|Sfrp2,20319:up|Tgfb2,21808:down|Serpine2,20720:up|Enpp1,18605:down
leukocyte proliferation	GO:0070661	2.54E-03	1.45E-02	Cd24a,12484:down|Ccl12,20293:up|Cd4,12504:up|Tfrc,22042:up|Grem1,23892:up|Igf1,16000:up|Bmp4,12159:up|Ceacam1,26365:up|Cxcl12,20315:up|Igf2,16002:up|Kitl,17311:up|Tnfsf9,21950:up

chordate embryonic development	GO:0043009	2.62E-03	1.49E-02	Cthrc1,68588:down|Adm,11535:up|Ptk7,71461:down|Gjb5,14622:down|Aldh1a2,19378:up|Epas1,13819:up|Vegfa,22339:down|Col11a1,12814:down|Egfr,13649:down|Fgfr1,14182:down|Sfrp1,20377:up|Sulf1,240725:down|Wnt5a,22418:down|Tgfbr3,21814:up|Bmpr1a,12166:down|Bmp4,12159:up|Igf1,16000:up|Chst11,58250:down|Sfrp2,20319:up|Tbx15,21384:down|Tgfb2,21808:down|Fgfr2,14183:up
negative regulation of MAPK cascade	GO:0043409	2.62E-03	1.49E-02	Sfrp1,20377:up|Sfrp4,20379:up|Igf1,16000:up|Bmp4,12159:up|Adipoq,11450:up|Ceacam1,26365:up|Sfrp2,20319:up|Errfi1,74155:down
regulation of cytokine production involved in immune response	GO:0002718	2.63E-03	1.49E-02	Tnf,21926:up|Hmox1,15368:up|Wnt5a,22418:down|Tgfb2,21808:down|Sema7a,20361:down
mesoderm development	GO:0007498	2.65E-03	1.50E-02	Sfrp2,20319:up|Fgfr1,14182:down|Wnt5a,22418:down|Vegfa,22339:down|Inhba,16323:down|Bmpr1a,12166:down|Bmp4,12159:up
regulation of extent of cell growth	GO:0061387	2.65E-03	1.50E-02	Tnfrsf12a,27279:down|Crabp2,12904:down|Plxnd1,67784:up|Cxcl12,20315:up|Wnt5a,22418:down|Vegfa,22339:down|Sema7a,20361:down
response to nitrogen compound	GO:1901698	2.67E-03	1.51E-02	Htr2b,15559:down|Col5a2,12832:down|Pdgfc,54635:down|Htr2a,15558:down|Mmp2,17390:down|Igf2,16002:up|Cdo1,12583:up|Pparg,19016:up|Tnf,21926:up|Col1a2,12843:down|Egfr,13649:down|Ncam1,17967:down|Hmox1,15368:up|Sfrp1,20377:up|Adipoq,11450:up|Igf1,16000:up|Col3a1,12825:down|Gatm,67092:down|Serpina3g,20715:up|Ifit1,15957:up|Enpp1,18605:down
vasculogenesis	GO:0001570	2.68E-03	1.51E-02	Ceacam1,26365:up|Adm,11535:up|Fgfr1,14182:down|Has2,15117:down|Tgfbr3,21814:up|Fgfr2,14183:up
cellular response to fibroblast growth factor stimulus	GO:0044344	2.68E-03	1.51E-02	Flrt2,399558:down|Fgfr1,14182:down|Sfrp1,20377:up|Sulf1,240725:down|Wnt5a,22418:down|Fgfr2,14183:up
muscle organ development	GO:0007517	2.72E-03	1.53E-02	Fgfr1,14182:down|Col11a1,12814:down|Ncam1,17967:down|Wnt5a,22418:down|Bmpr1a,12166:down|Tgfbr3,21814:up|Wisp1,22402:down|Grem1,23892:up|Col3a1,12825:down|Igf1,16000:up|Bmp4,12159:up|Ankrd1,107765:down|Tgfb2,21808:down|Fgfr2,14183:up
developmental cell growth	GO:0048588	2.72E-03	1.53E-02	Plxnd1,67784:up|Wnt5a,22418:down|Wisp1,22402:down|Sema7a,20361:down|Igf1,16000:up|Tnfrsf12a,27279:down|Crabp2,12904:down|Cxcl12,20315:up|Postn,50706:down|Vegfa,22339:down
positive regulation of intracellular transport	GO:0032388	2.75E-03	1.54E-02	Tnf,21926:up|Gas6,14456:up|Ice1,218333:down|Egfr,13649:down|Bmpr1a,12166:down|Ccl2,20296:up|Cd4,12504:up|Ptgs2,19225:down|Grem1,23892:up|Igf1,16000:up|Bmp4,12159:up|Ceacam1,26365:up|Sfrp2,20319:up
muscle system process	GO:0003012	2.75E-03	1.54E-02	Htr2b,15559:down|Fxyd1,56188:up|Lmcd1,30937:up|Sulf1,240725:down|Wisp1,22402:down|Ptgs2,19225:down|Stc1,20855:up|Ednrb,13618:up|Igf1,16000:up|Vegfb,22340:up|Htr2a,15558:down|Npnt,114249:up|Errfi1,74155:down
negative regulation of kidney development	GO:0090185	2.75E-03	1.54E-02	Grem1,23892:up|Bmp4,12159:up|Adipoq,11450:up
regulation of platelet-derived growth factor receptor signaling pathway	GO:0010640	2.75E-03	1.54E-02	Ptgir,19222:up|F3,14066:up|Adipoq,11450:up
fibril organization	GO:0097435	2.75E-03	1.54E-02	Ltbp2,16997:down|Col5a1,12831:down|Col3a1,12825:down
negative regulation of cell killing	GO:0031342	2.75E-03	1.54E-02	Ceacam1,26365:up|Clec2d,93694:up|Igf2,16002:up
central nervous system development	GO:0007417	2.75E-03	1.54E-02	Inhba,16323:down|Aldh1a2,19378:up|Sema7a,20361:down|Uchl5,56207:up|Shroom4,208431:down|Cxcl12,20315:up|Cx3cr1,13051:down|Serpine2,20720:up|Pparg,19016:up|Egfr,13649:down|Fgfr1,14182:down|Ncam1,17967:down|Sfrp1,20377:up|Wnt5a,22418:down|Bmpr1a,12166:down|Bmp4,12159:up|Igf1,16000:up|Col3a1,12825:down|Sfrp2,20319:up|Pcdh19,279653:down|Aldh1a3,56847:down|Plpp3,67916:up|Fgfr2,14183:up
learning or memory	GO:0007611	2.77E-03	1.54E-02	Plk2,20620:down|Glp1r,14652:down|Egfr,13649:down|Ncam1,17967:down|Ptgs2,19225:down|Il1rn,16181:up|Igf1,16000:up|Htr2a,15558:down|Prkar2b,19088:up|Cx3cr1,13051:down|Igf2,16002:up
negative regulation of growth	GO:0045926	2.77E-03	1.54E-02	Tnf,21926:up|Pparg,19016:up|Sfrp1,20377:up|Wnt5a,22418:down|Inhba,16323:down|Grem1,23892:up|Bmp4,12159:up|Sfrp2,20319:up|Tgfb2,21808:down|Serpine2,20720:up|Enpp1,18605:down
cation transport	GO:0006812	2.78E-03	1.55E-02	Htr2b,15559:down|Vdr,22337:down|Gpm6b,14758:up|Cxcl12,20315:up|Htr2a,15558:down|Serpine2,20720:up|Slc40a1,53945:up|Gas6,14456:up|Glp1r,14652:down|Hvcn1,74096:up|Fxyd1,56188:up|Trf,22041:up|Cd4,12504:up|Ccl12,20293:up|Ptgs2,19225:down|Stc1,20855:up|Tfrc,22042:up|Kcnj15,16516:down|Fxyd2,11936:up|Ccl4,20303:up|Sncg,20618:up|Serinc2,230779:down|Maob,109731:up|Bmp4,12159:up|Igf1,16000:up|Tgfb2,21808:down|S100a1,20193:up
regulation of cellular protein localization	GO:1903827	2.81E-03	1.56E-02	Ice1,218333:down|Ccl2,20296:up|Gpm6b,14758:up|Lrrc15,74488:down|Gas6,14456:up|Tnf,21926:up|Egfr,13649:down|C1qtnf3,81799:down|Bmpr1a,12166:down|Wnt5a,22418:down|Ptgs2,19225:down|Grem1,23892:up|Adipoq,11450:up|Bmp4,12159:up|Igf1,16000:up|Ceacam1,26365:up|Sfrp2,20319:up|Tgfb2,21808:down
ventricular septum morphogenesis	GO:0060412	2.83E-03	1.57E-02	Wnt5a,22418:down|Tgfb2,21808:down|Fgfr2,14183:up|Bmp4,12159:up
regulation of activated T cell proliferation	GO:0046006	2.83E-03	1.57E-02	Cd24a,12484:down|Igf2,16002:up|Tnfsf9,21950:up|Igf1,16000:up
neural crest cell migration	GO:0001755	2.86E-03	1.58E-02	Htr2b,15559:down|Ret,19713:up|Ednrb,13618:up|Sema7a,20361:down|Kitl,17311:up
positive regulation of epithelial cell differentiation	GO:0030858	2.86E-03	1.58E-02	Vdr,22337:down|Sfrp4,20379:up|Kitl,17311:up|Bmp4,12159:up|Adipoq,11450:up
regulation of transforming growth factor beta receptor signaling pathway	GO:0017015	2.86E-03	1.58E-02	Htra3,78558:down|Chst11,58250:down|Tgfbr3,21814:up|Npnt,114249:up|Cd109,235505:down|Aspn,66695:down
glucose metabolic process	GO:0006006	2.87E-03	1.59E-02	Tnf,21926:up|Igfbp4,16010:up|Fabp5,16592:up|C1qtnf3,81799:down|Igf1,16000:up|Adipoq,11450:up|Igfbp3,16009:up|Igf2,16002:up|Enpp1,18605:down
negative regulation of cell-cell adhesion	GO:0022408	2.88E-03	1.59E-02	Cd24a,12484:down|Il1rn,16181:up|Bmp4,12159:up|Adipoq,11450:up|Ceacam1,26365:up|Cxcl12,20315:up|Vegfa,22339:down|Serpine2,20720:up

regulation of homotypic cell-cell adhesion	GO:0034110	2.89E-03	1.59E-02	Cd24a,12484:down|Has2,15117:down|Ccl2,20296:up|Cd4,12504:up|Tfrc,22042:up|Igf1,16000:up|Bmp4,12159:up|Ctla2a,13024:down|Ceacam1,26365:up|Serpine2,20720:up|Igf2,16002:up|Tnfsf9,21950:up
regulation of synaptic transmission	GO:0050804	2.91E-03	1.61E-02	Tnf,21926:up|Plk2,20620:down|Lama2,16773:down|Egfr,13649:down|Ncam1,17967:down|Ccl2,20296:up|Ptgs2,19225:down|Sncg,20618:up|Adipoq,11450:up|Gpm6b,14758:up|Htr2a,15558:down|Serpine2,20720:up|Cx3cr1,13051:down
female sex differentiation	GO:0046660	2.95E-03	1.62E-02	Sfrp1,20377:up|Wnt5a,22418:down|Vegfa,22339:down|Inhba,16323:down|Mmp2,17390:down|Kitl,17311:up|Bmp4,12159:up
positive regulation of neuron differentiation	GO:0045666	2.95E-03	1.62E-02	Fgfr1,14182:down|Cd24a,12484:down|Ptk7,71461:down|Wnt5a,22418:down|Dpysl3,22240:down|Sema7a,20361:down|Bmp4,12159:up|Ankrd1,107765:down|Tnfrsf12a,27279:down|Crabp2,12904:down|Ret,19713:up|Cxcl12,20315:up|Vegfa,22339:down|Serpine2,20720:up
macromolecule modification	GO:0043412	2.98E-03	1.64E-02	Igfbp4,16010:up|Flrt2,399558:down|Sfrp4,20379:up|C3,12266:up|Gstt1,14871:up|Igfbp3,16009:up|Cd109,235505:down|Loxl2,94352:down|Plk2,20620:down|Trf,22041:up|Cd24a,12484:down|Wnt5a,22418:down|Mark1,226778:up|Il1rn,16181:up|Adipoq,11450:up|Bmp4,12159:up|Igf1,16000:up|Tgfb2,21808:down|Fgfr2,14183:up|Htr2b,15559:down|Efemp1,216616:up|Ccl6,20305:up|Aspn,66695:down|Saa3,20210:up|Fabp4,11770:up|Htr2a,15558:down|Gas6,14456:up|Egfr,13649:down|Sfrp1,20377:up|C1qtnf3,81799:down|Tgfbr3,21814:up|Ceacam1,26365:up|Ccl8,20307:up|Sfrp2,20319:up|Fam20a,208659:down|Npnt,114249:up|Tnfrsf11b,18383:up|Prkar2b,19088:up|Neurl3,214854:down|Ccl2,20296:up|Ccl7,20306:up|Ednrb,13618:up|Pdgfc,54635:down|Pdzrn3,55983:down|Sema7a,20361:down|Uchl5,56207:up|Ddah1,69219:down|Dok2,13449:up|Man2a2,140481:up|Mamdc2,71738:down|Ccl12,20293:up|Bmpr1a,12166:down|Ccl4,20303:up|Ret,19713:up|Vegfb,22340:up|Sdf2l1,64136:up|Plpp3,67916:up|Kitl,17311:up|P4ha1,18451:down|Ccl9,20308:up|Inhba,16323:down|Ccl3,20302:up|Crlf1,12931:down|Vegfa,22339:down|Lrrc15,74488:down|Igf2,16002:up|Nxn,18230:down|Tnf,21926:up|Fgfr1,14182:down|Cd4,12504:up|Grem1,23892:up|Col3a1,12825:down|Errfi1,74155:down|Enpp1,18605:down
embryo development ending in birth or egg hatching	GO:0009792	3.00E-03	1.65E-02	Cthrc1,68588:down|Adm,11535:up|Ptk7,71461:down|Gjb5,14622:down|Aldh1a2,19378:up|Epas1,13819:up|Vegfa,22339:down|Col11a1,12814:down|Egfr,13649:down|Fgfr1,14182:down|Sfrp1,20377:up|Sulf1,240725:down|Wnt5a,22418:down|Tgfbr3,21814:up|Bmpr1a,12166:down|Bmp4,12159:up|Igf1,16000:up|Chst11,58250:down|Sfrp2,20319:up|Tbx15,21384:down|Tgfb2,21808:down|Fgfr2,14183:up
positive regulation of peptidyl-serine phosphorylation	GO:0033138	3.05E-03	1.67E-02	Tnf,21926:up|Gas6,14456:up|Sfrp2,20319:up|Ret,19713:up|Wnt5a,22418:down|Vegfa,22339:down
receptor internalization	GO:0031623	3.05E-03	1.67E-02	Htr2b,15559:down|Adm,11535:up|Vegfa,22339:down|Sfrp4,20379:up|Tfrc,22042:up|Grem1,23892:up
cellular response to alcohol	GO:0097306	3.05E-03	1.67E-02	Vdr,22337:down|Egfr,13649:down|Inhba,16323:down|Ccl7,20306:up|Mmp2,17390:down|Bmp4,12159:up
response to fibroblast growth factor	GO:0071774	3.05E-03	1.67E-02	Flrt2,399558:down|Fgfr1,14182:down|Sfrp1,20377:up|Sulf1,240725:down|Wnt5a,22418:down|Fgfr2,14183:up
regulation of cellular response to transforming growth factor beta stimulus	GO:1903844	3.05E-03	1.67E-02	Htra3,78558:down|Chst11,58250:down|Tgfbr3,21814:up|Npnt,114249:up|Cd109,235505:down|Aspn,66695:down
synaptic transmission	GO:0007268	3.08E-03	1.68E-02	Htr2b,15559:down|Lama2,16773:down|Ccl2,20296:up|Plat,18791:down|Slc29a1,63959:up|Gpm6b,14758:up|Htr2a,15558:down|Cx3cr1,13051:down|Serpine2,20720:up|Tnf,21926:up|Plk2,20620:down|Cd24a,12484:down|Egfr,13649:down|Ncam1,17967:down|Ptgs2,19225:down|Sncg,20618:up|Adipoq,11450:up|Fgfr2,14183:up|Syt13,80976:up
regulation of kidney development	GO:0090183	3.10E-03	1.69E-02	Ret,19713:up|Vegfa,22339:down|Grem1,23892:up|Bmp4,12159:up|Adipoq,11450:up
negative regulation of muscle cell differentiation	GO:0051148	3.10E-03	1.69E-02	Tnf,21926:up|Prl2c2,18811:up|Cxcl14,57266:down|Ccl9,20308:up|Bmp4,12159:up
smooth muscle cell differentiation	GO:0051145	3.10E-03	1.69E-02	Adm,11535:up|Npnt,114249:up|Ednrb,13618:up|Fgfr2,14183:up|Bmp4,12159:up
cell adhesion involved in heart morphogenesis	GO:0061343	3.14E-03	1.69E-02	Flrt2,399558:down|Tgfb2,21808:down
positive regulation of glycogen (starch) synthase activity	GO:2000467	3.14E-03	1.69E-02	Igf2,16002:up|Adipoq,11450:up
regulation of bone mineralization involved in bone maturation	GO:1900157	3.14E-03	1.69E-02	Fam101b,76566:down|Grem1,23892:up
regulation of metanephric glomerulus development	GO:0072298	3.14E-03	1.69E-02	Ret,19713:up|Adipoq,11450:up
positive regulation of metanephric glomerulus development	GO:0072300	3.14E-03	1.69E-02	Ret,19713:up|Adipoq,11450:up
regulation of ovulation	GO:0060278	3.14E-03	1.69E-02	Inhba,16323:down|Plat,18791:down
positive regulation of peptidyl-tyrosine autophosphorylation	GO:1900086	3.14E-03	1.69E-02	Vegfa,22339:down|Grem1,23892:up
detoxification of inorganic compound	GO:0061687	3.14E-03	1.69E-02	Mt2,17750:up|Mt1,17748:up
midgut development	GO:0007494	3.14E-03	1.69E-02	Wnt5a,22418:down|Ednrb,13618:up

positive regulation of apoptotic cell clearance	GO:2000427	3.14E-03	1.69E-02	Ccl2,20296:up|C3,12266:up
regulation of dendritic cell differentiation	GO:2001198	3.14E-03	1.69E-02	Tmem176a,66058:up|Tmem176b,65963:up
stress-activated MAPK cascade	GO:0051403	3.15E-03	1.70E-02	Tnf,21926:up|Trf,22041:up|Sfrp1,20377:up|Wnt5a,22418:down|Sfrp4,20379:up|Il1rn,16181:up|Ceacam1,26365:up|Sfrp2,20319:up|Vegfa,22339:down|Tgfb2,21808:down
positive regulation of synaptic transmission	GO:0050806	3.16E-03	1.70E-02	Tnf,21926:up|Plk2,20620:down|Lama2,16773:down|Egfr,13649:down|Ccl2,20296:up|Ptgs2,19225:down|Serpine2,20720:up|Cx3cr1,13051:down
regulation of axon extension involved in axon guidance	GO:0048841	3.16E-03	1.70E-02	Plxnd1,67784:up|Cxcl12,20315:up|Wnt5a,22418:down|Vegfa,22339:down
positive regulation of phospholipase activity	GO:0010518	3.16E-03	1.70E-02	Htr2b,15559:down|Fgfr1,14182:down|Htr2a,15558:down|Fgfr2,14183:up
positive regulation of nitrogen compound metabolic process	GO:0051173	3.21E-03	1.72E-02	Fstl3,83554:down|Ice1,218333:down|Adm,11535:up|Pdgfc,54635:down|Epas1,13819:up|Ddah1,69219:down|Lyl1,17095:up|Sdpr,20324:down|Igfbp3,16009:up|Trf,22041:up|Ptx3,19288:up|Wnt5a,22418:down|Bmpr1a,12166:down|Maob,109731:up|Bmp4,12159:up|Igf1,16000:up|Ret,19713:up|Plpp3,67916:up|Ptgir,19222:up|Creb3l1,26427:down|Fgfr2,14183:up|Kitl,17311:up|Prl2c2,18811:up|Inhba,16323:down|Rasl11a,68895:up|Ccl3,20302:up|Vdr,22337:down|Vegfa,22339:down|Htr2a,15558:down|Igf2,16002:up|Slc40a1,53945:up|Pparg,19016:up|Tnf,21926:up|Glp1r,14652:down|Egfr,13649:down|Fgfr1,14182:down|Sfrp1,20377:up|Has2,15117:down|Tgfbr3,21814:up|Ptgs2,19225:down|Tfrc,22042:up|Grem1,23892:up|Sfrp2,20319:up|Npnt,114249:up
reactive oxygen species biosynthetic process	GO:1903409	3.25E-03	1.74E-02	Tnf,21926:up|Ptx3,19288:up|Ddah1,69219:down|Egfr,13649:down|Ptgs2,19225:down|Igf1,16000:up
response to estrogen	GO:0043627	3.25E-03	1.74E-02	Egfr,13649:down|Cd24a,12484:down|Sfrp1,20377:up|Hmox1,15368:up|Tgfb2,21808:down|Mmp2,17390:down
positive regulation of GTPase activity	GO:0043547	3.25E-03	1.74E-02	Ccl9,20308:up|Ccl6,20305:up|Wnt5a,22418:down|Ccl7,20306:up|Ccl2,20296:up|Ccl12,20293:up|Ccl4,20303:up|Ccl3,20302:up|Ccl8,20307:up
phosphate ion transport	GO:0006817	3.29E-03	1.75E-02	Ank,11732:down|Fgfr1,14182:down|Sfrp4,20379:up
ossification involved in bone maturation	GO:0043931	3.29E-03	1.75E-02	Fam101b,76566:down|Grem1,23892:up|Igf1,16000:up
bone maturation	GO:0070977	3.29E-03	1.75E-02	Fam101b,76566:down|Grem1,23892:up|Igf1,16000:up
activation of protein kinase B activity	GO:0032148	3.29E-03	1.75E-02	Gas6,14456:up|Wnt5a,22418:down|Igf1,16000:up
response to cadmium ion	GO:0046686	3.29E-03	1.75E-02	Sord,20322:up|Akr1c18,105349:up|Hmox1,15368:up
regulation of pathway-restricted SMAD protein phosphorylation	GO:0060393	3.35E-03	1.78E-02	Tgfb2,21808:down|Inhba,16323:down|Bmpr1a,12166:down|Grem1,23892:up|Bmp4,12159:up
positive regulation of fibroblast proliferation	GO:0048146	3.35E-03	1.78E-02	Gas6,14456:up|Egfr,13649:down|Wnt5a,22418:down|Pdgfc,54635:down|Igf1,16000:up
negative regulation of macromolecule metabolic process	GO:0010605	3.38E-03	1.80E-02	Flrt2,399558:down|Sfrp4,20379:up|Ednrb,13618:up|Cst7,13011:up|Epas1,13819:up|Ankrd1,107765:down|Igfbp3,16009:up|Cd109,235505:down|Loxl2,94352:down|Loxl3,16950:down|Ptx3,19288:up|Wnt5a,22418:down|Ccl4,20303:up|Adipoq,11450:up|Bmp4,12159:up|Igf1,16000:up|Prg4,96875:down|Tgfb2,21808:down|Plpp3,67916:up|Fgfr2,14183:up|Kng1,16644:up|Slpi,20568:up|Aspn,66695:down|Plat,18791:down|Tfpi2,21789:down|Vdr,22337:down|Ctla2a,13024:down|Fabp4,11770:up|Vegfa,22339:down|Serpine2,20720:up|Lrrc15,74488:down|Nxn,18230:down|Pparg,19016:up|Gas6,14456:up|Tnf,21926:up|Lmcd1,30937:up|Fgfr1,14182:down|Egfr,13649:down|Hmox1,15368:up|Sfrp1,20377:up|Tgfbr3,21814:up|Grem1,23892:up|Ceacam1,26365:up|Sfrp2,20319:up|Serping1,12258:up|Serpina3g,20715:up|Prkar2b,19088:up|Errfi1,74155:down|Enpp1,18605:down|S100a1,20193:up
positive regulation of biosynthetic process	GO:0009891	3.40E-03	1.81E-02	Fstl3,83554:down|Ice1,218333:down|Adm,11535:up|Ccl2,20296:up|Pdgfc,54635:down|Epas1,13819:up|Ddah1,69219:down|Lyl1,17095:up|Sdpr,20324:down|Trf,22041:up|Ptx3,19288:up|Wnt5a,22418:down|Bmpr1a,12166:down|Bmp4,12159:up|Igf1,16000:up|Ret,19713:up|Tgfb2,21808:down|Plpp3,67916:up|Ptgir,19222:up|Creb3l1,26427:down|Fgfr2,14183:up|Kitl,17311:up|Prl2c2,18811:up|Htr2b,15559:down|Inhba,16323:down|Rasl11a,68895:up|Ccl3,20302:up|Vdr,22337:down|Vegfa,22339:down|Htr2a,15558:down|Igf2,16002:up|Slc40a1,53945:up|Pparg,19016:up|Tnf,21926:up|Glp1r,14652:down|Egfr,13649:down|Fgfr1,14182:down|Sfrp1,20377:up|Has2,15117:down|Tgfbr3,21814:up|Ptgs2,19225:down|Grem1,23892:up|Sfrp2,20319:up|Npnt,114249:up
monocarboxylic acid metabolic process	GO:0032787	3.42E-03	1.81E-02	P4ha1,18451:down|Hpgd,15446:up|Aldh1a2,19378:up|C3,12266:up|Fabp4,11770:up|Fam132b,227358:down|Htr2a,15558:down|Cdo1,12583:up|Pparg,19016:up|Ggt5,23887:up|Akr1c18,105349:up|Lpl,16956:up|Ptgs2,19225:down|Adipoq,11450:up|Igf1,16000:up|Crabp2,12904:down|Aldh1a3,56847:down|Prkar2b,19088:up
cell morphogenesis involved in differentiation	GO:0000904	3.42E-03	1.81E-02	Fam101b,76566:down|Flrt2,399558:down|Lama2,16773:down|Plxnd1,67784:up|Sema7a,20361:down|Tnfsf12a,27279:down|Cxcl12,20315:up|Postn,50706:down|Vegfa,22339:down|Loxl2,94352:down|Loxl3,16950:down|Sfrp1,20377:up|Has2,15117:down|Wnt5a,22418:down|Tgfbr3,21814:up|Stc1,20855:up|Grem1,23892:up|Bmp4,12159:up|Sfrp2,20319:up|Crabp2,12904:down|Ret,19713:up|Tgfb2,21808:down|Fgfr2,14183:up
positive regulation of cellular protein localization	GO:1903829	3.46E-03	1.83E-02	Tnf,21926:up|Gas6,14456:up|Ice1,218333:down|Egfr,13649:down|Bmpr1a,12166:down|Ccl2,20296:up|Ptgs2,19225:down|Grem1,23892:up|Igf1,16000:up|Bmp4,12159:up|Ceacam1,26365:up|Sfrp2,20319:up|Tgfb2,21808:down
I-kappaB kinase/NF-kappaB signaling	GO:0007249	3.51E-03	1.85E-02	Tnf,21926:up|Plk2,20620:down|Hmox1,15368:up|C1qtnf3,81799:down|Ptgs2,19225:down|Grem1,23892:up|Saa3,20210:up|Adipoq,11450:up|Clec4n,56620:up|Il1rl1,17082:down
regulation of organ formation	GO:0003156	3.52E-03	1.85E-02	Fgfr1,14182:down|Sulf1,240725:down|Wnt5a,22418:down|Bmp4,12159:up
negative regulation of response to cytokine stimulus	GO:0060761	3.52E-03	1.85E-02	Pparg,19016:up|Gas6,14456:up|Il1rn,16181:up|Adipoq,11450:up

regulation of neuroblast proliferation	GO:1902692	3.52E-03	1.85E-02	Prl2c2,18811:up|Cd24a,12484:down|Vegfa,22339:down|Cx3cr1,13051:down
regulation of proteolysis	GO:0030162	3.58E-03	1.88E-02	Kng1,16644:up|C3,12266:up|Slpi,20568:up|Cst7,13011:up|Plat,18791:down|Tfpi2,21789:down|Ctla2a,13024:down|Uchl5,56207:up|Vegfa,22339:down|Serpine2,20720:up|Cd109,235505:down|Gas6,14456:up|Pparg,19016:up|Tnf,21926:up|Plk2,20620:down|Igf1,16000:up|Sfrp2,20319:up|Serping1,12258:up|Serpina3g,20715:up|F3,14066:up
regulation of T cell proliferation	GO:0042129	3.61E-03	1.90E-02	Cd24a,12484:down|Cd4,12504:up|Tfrc,22042:up|Igf1,16000:up|Bmp4,12159:up|Ceacam1,26365:up|Igf2,16002:up|Tnfsf9,21950:up
response to temperature stimulus	GO:0009266	3.63E-03	1.91E-02	Htr2b,15559:down|Tfec,21426:up|Hmox1,15368:up|Htr2a,15558:down|Hspb7,29818:up|Ccl2,20296:up|Igf1,16000:up
regulation of lymphocyte mediated immunity	GO:0002706	3.63E-03	1.91E-02	Ceacam1,26365:up|Tnf,21926:up|Clec2d,93694:up|Cd24a,12484:down|C3,12266:up|Tfrc,22042:up|Igf2,16002:up
T cell proliferation	GO:0042098	3.65E-03	1.92E-02	Cd24a,12484:down|Cd4,12504:up|Tfrc,22042:up|Igf1,16000:up|Bmp4,12159:up|Ceacam1,26365:up|Cxcl12,20315:up|Igf2,16002:up|Tnfsf9,21950:up
positive regulation of cytoplasmic transport	GO:1903651	3.72E-03	1.95E-02	Tnf,21926:up|Gas6,14456:up|Egfr,13649:down|Bmpr1a,12166:down|Cd4,12504:up|Ptgs2,19225:down|Grem1,23892:up|Igf1,16000:up|Bmp4,12159:up|Ceacam1,26365:up|Sfrp2,20319:up
Wnt signaling pathway	GO:0016055	3.74E-03	1.96E-02	Cthrc1,68588:down|Sfrp1,20377:up|Sulf1,240725:down|Ptk7,71461:down|Wnt5a,22418:down|Sfrp4,20379:up|Wisp1,22402:down|Mark1,226778:up|Grem1,23892:up|Sfrp2,20319:up|Rspo2,239405:up|Plpp3,67916:up|Nxn,18230:down|Fgfr2,14183:up
negative regulation of secretion	GO:0051048	3.77E-03	1.97E-02	Tnf,21926:up|Pparg,19016:up|Gas6,14456:up|Sfrp1,20377:up|Hmox1,15368:up|C1qtnf3,81799:down|Il1rn,16181:up|Maob,109731:up|Adipoq,11450:up|Anpep,16790:up
receptor-mediated endocytosis	GO:0006898	3.80E-03	1.98E-02	Htr2b,15559:down|Trf,22041:up|Adm,11535:up|Cd24a,12484:down|Mrc1,17533:up|Sfrp4,20379:up|Tfrc,22042:up|Grem1,23892:up|Vegfa,22339:down
regulation of interleukin-6 production	GO:0032675	3.82E-03	1.99E-02	Tnf,21926:up|Gas6,14456:up|Prg4,96875:down|C1qtnf3,81799:down|Wnt5a,22418:down|Figf,14205:up|Tnfsf9,21950:up
regulation of anatomical structure size	GO:0090066	3.86E-03	2.00E-02	Htr2b,15559:down|Kng1,16644:up|Adm,11535:up|Plxnd1,67784:up|Ednrb,13618:up|Sema7a,20361:down|Tnfrsf12a,27279:down|Cxcl12,20315:up|Vegfa,22339:down|Htr2a,15558:down|Mmp2,17390:down|Egfr,13649:down|Tgfbr3,21814:up|Wnt5a,22418:down|Ptgs2,19225:down|Crabp2,12904:down|Ret,19713:up
ovulation	GO:0030728	3.90E-03	2.00E-02	Inhba,16323:down|Ptgs2,19225:down|Plat,18791:down
response to interferon-alpha	GO:0035455	3.90E-03	2.00E-02	Gas6,14456:up|Ifit1,15957:up|Ifit3,15959:up
positive regulation of protein autophosphorylation	GO:0031954	3.90E-03	2.00E-02	Vegfa,22339:down|Pdgfc,54635:down|Grem1,23892:up
regulation of monocyte chemotaxis	GO:0090025	3.90E-03	2.00E-02	Cxcl12,20315:up|Ccl2,20296:up|Grem1,23892:up
protein heterotrimerization	GO:0070208	3.90E-03	2.00E-02	Col1a2,12843:down|C1qtnf6,72709:down|Adipoq,11450:up
regulation of heterotypic cell-cell adhesion	GO:0034114	3.90E-03	2.00E-02	Tnf,21926:up|Il1rn,16181:up|Adipoq,11450:up
negative regulation of smooth muscle cell migration	GO:0014912	3.90E-03	2.00E-02	Bmpr1a,12166:down|Igfbp3,16009:up|Adipoq,11450:up
positive regulation of epidermal cell differentiation	GO:0045606	3.90E-03	2.00E-02	Vdr,22337:down|Sfrp4,20379:up|Bmp4,12159:up
ovarian follicle development	GO:0001541	3.90E-03	2.00E-02	Vegfa,22339:down|Inhba,16323:down|Mmp2,17390:down|Kitl,17311:up|Bmp4,12159:up
morphogenesis of a polarized epithelium	GO:0001738	3.90E-03	2.00E-02	Cthrc1,68588:down|Sfrp2,20319:up|Sfrp1,20377:up|Ptk7,71461:down|Wnt5a,22418:down
regulation of lymphocyte apoptotic process	GO:0070228	3.90E-03	2.00E-02	Ceacam1,26365:up|Cd24a,12484:down|Wnt5a,22418:down|Tgfb2,21808:down|Bmp4,12159:up
regulation of collagen metabolic process	GO:0010712	3.90E-03	2.00E-02	Pparg,19016:up|Ccl2,20296:up|Errfi1,74155:down|Bmp4,12159:up
regulation of T cell apoptotic process	GO:0070232	3.90E-03	2.00E-02	Ceacam1,26365:up|Wnt5a,22418:down|Tgfb2,21808:down|Bmp4,12159:up
regulation of polysaccharide metabolic process	GO:0032881	3.90E-03	2.00E-02	Has2,15117:down|Enpp1,18605:down|Igf2,16002:up|Igf1,16000:up
metanephric nephron development	GO:0072210	3.90E-03	2.00E-02	Ret,19713:up|Grem1,23892:up|Bmp4,12159:up|Adipoq,11450:up
regulation of DNA replication	GO:0006275	3.94E-03	2.02E-02	Pparg,19016:up|Fgfr1,14182:down|Egfr,13649:down|Pdgfc,54635:down|Grem1,23892:up|Igf1,16000:up|Bmp4,12159:up|Kitl,17311:up
neural tube formation	GO:0001841	4.02E-03	2.06E-02	Cthrc1,68588:down|Sfrp2,20319:up|Adm,11535:up|Sfrp1,20377:up|Ptk7,71461:down|Wnt5a,22418:down|Bmp4,12159:up

regulation of macromolecule metabolic process	GO:0060255	4.05E-03	2.07E-02	Fstl3,83554:down|Igfbp4,16010:up|Flrt2,399558:down|Tfec,21426:up|Sfrp4,20379:up|C3,12266:up|Ankrd1,107765:down|Lyl1,17095:up|Igfbp3,16009:up|Cd109,235505:down|Loxl2,94352:down|Plk2,20620:down|Loxl3,16950:down|Trf,22041:up|Cd24a,12484:down|Wnt5a,22418:down|Il1rn,16181:up|Adipoq,11450:up|Bmp4,12159:up|Igf1,16000:up|Tgfb2,21808:down|F3,14066:up|Fgfr2,14183:up|Htr2b,15559:down|Kng1,16644:up|Prl2c2,18811:up|Efemp1,216616:up|Ccl6,20305:up|Slpi,20568:up|Aspn,66695:down|Vdr,22337:down|Fabp4,11770:up|Htr2a,15558:down|Oas3,246727:up|Slc40a1,53945:up|Gas6,14456:up|Egfr,13649:down|Sfrp1,20377:up|C1qtnf3,81799:down|Tgfbr3,21814:up|Tfrc,22042:up|Ceacam1,26365:up|Ccl8,20307:up|Sfrp2,20319:up|Serpina3g,20715:up|Fam20a,208659:down|Npnt,114249:up|Tnfrsf11b,18383:up|Prkar2b,19088:up|S100a1,20193:up|Ice1,218333:down|Aldh1a2,19378:up|Ccl2,20296:up|Ccl7,20306:up|Pdgfc,54635:down|Cst7,13011:up|Ednrb,13618:up|Sema7a,20361:down|Epas1,13819:up|Htra3,78558:down|Uchl5,56207:up|Sdpr,20324:down|Ptx3,19288:up|Ccl12,20293:up|Bmpr1a,12166:down|Ccl4,20303:up|Tnc,21923:down|Ret,19713:up|Prg4,96875:down|Plpp3,67916:up|Creb3l1,26427:down|Kitl,17311:up|Ccl9,20308:up|Inhba,16323:down|Rasl11a,68895:up|Bnc2,242509:down|Plat,18791:down|Ccl3,20302:up|Tfpi2,21789:down|Ctla2a,13024:down|Crlf1,12931:down|Vegfa,22339:down|Serpine2,20720:up|Lrrc15,74488:down|Nxn,18230:down|Igf2,16002:up|Pparg,19016:up|Tnf,21926:up|Glp1r,14652:down|Lmcd1,30937:up|Fgfr1,14182:down|Has2,15117:down|Hmox1,15368:up|Sulf1,240725:down|Cd4,12504:up|Grem1,23892:up|Phf11d,219132:up|Tbx15,21384:down|Serping1,12258:up|Errfi1,74155:down|Enpp1,18605:down
single-organism biosynthetic process	GO:0044711	4.09E-03	2.09E-02	Adm,11535:up|Ccl2,20296:up|Aldh1a2,19378:up|C3,12266:up|Ampd3,11717:up|Serpinh1,12406:down|Sord,20322:up|Cdo1,12583:up|Ggt5,23887:up|Akr1c18,105349:up|Fabp5,16592:up|Wnt5a,22418:down|Adipoq,11450:up|Bmp4,12159:up|Igf1,16000:up|Chst11,58250:down|Gatm,67092:down|Pdxk,216134:up|Aldh1a3,56847:down|Ptgir,19222:up|Htr2b,15559:down|Vdr,22337:down|Htr2a,15558:down|Igf2,16002:up|Tnf,21926:up|Pparg,19016:up|Fgfr1,14182:down|Lpl,16956:up|Has2,15117:down|C1qtnf3,81799:down|Ptgs2,19225:down|Ceacam1,26365:up|Col5a1,12831:down|Uck2,80914:down|Errfi1,74155:down|Enpp1,18605:down
homotypic cell-cell adhesion	GO:0034109	4.12E-03	2.11E-02	Ly9,17085:up|Ccl2,20296:up|Ctla2a,13024:down|Cxcl12,20315:up|Serpine2,20720:up|Igf2,16002:up|Cd24a,12484:down|Ncam1,17967:down|Has2,15117:down|Cd4,12504:up|Tfrc,22042:up|Bmp4,12159:up|Igf1,16000:up|Ceacam1,26365:up|Plpp3,67916:up|Tnfsf9,21950:up|Clec4e,56619:up
ovulation cycle	GO:0042698	4.14E-03	2.11E-02	Egfr,13649:down|Vegfa,22339:down|Inhba,16323:down|Mmp2,17390:down|Kitl,17311:up|Bmp4,12159:up
regulation of lipid metabolic process	GO:0019216	4.14E-03	2.11E-02	Htr2b,15559:down|Tnf,21926:up|Pparg,19016:up|Akr1c18,105349:up|C3,12266:up|Igf1,16000:up|Adipoq,11450:up|Vdr,22337:down|Ceacam1,26365:up|Fam132b,227358:down|Htr2a,15558:down|Igf2,16002:up
regulation of neuron differentiation	GO:0045664	4.18E-03	2.13E-02	Efemp1,216616:up|Plxnd1,67784:up|Ptk7,71461:down|Dpysl3,22240:down|Ednrb,13618:up|Sema7a,20361:down|Ankrd1,107765:down|Tnfrsf12a,27279:down|Cxcl12,20315:up|Vegfa,22339:down|Serpine2,20720:up|Plk2,20620:down|Cd24a,12484:down|Fgfr1,14182:down|Sfrp1,20377:up|Wnt5a,22418:down|Bmp4,12159:up|Sfrp2,20319:up|Crabp2,12904:down|Ret,19713:up
cardiac septum morphogenesis	GO:0060411	4.20E-03	2.14E-02	Wnt5a,22418:down|Tgfb2,21808:down|Bmpr1a,12166:down|Fgfr2,14183:up|Bmp4,12159:up
regulation of metabolic process	GO:0019222	4.21E-03	2.14E-02	Fstl3,83554:down|Igfbp4,16010:up|Adm,11535:up|Flrt2,399558:down|Tfec,21426:up|Sfrp4,20379:up|C3,12266:up|Ankrd1,107765:down|Lyl1,17095:up|Igfbp3,16009:up|Cd109,235505:down|Loxl2,94352:down|Plk2,20620:down|Loxl3,16950:down|Trf,22041:up|Cd24a,12484:down|Wnt5a,22418:down|Il1rn,16181:up|Maob,109731:up|Adipoq,11450:up|Bmp4,12159:up|Igf1,16000:up|Tgfb2,21808:down|Ptgir,19222:up|F3,14066:up|Fgfr2,14183:up|Htr2b,15559:down|Kng1,16644:up|Prl2c2,18811:up|Dram1,71712:up|Efemp1,216616:up|Ccl6,20305:up|Slpi,20568:up|Aspn,66695:down|Vdr,22337:down|Fabp4,11770:up|Fam132b,227358:down|Htr2a,15558:down|Oas3,246727:up|Slc40a1,53945:up|Gas6,14456:up|Egfr,13649:down|Sfrp1,20377:up|C1qtnf3,81799:down|Tgfbr3,21814:up|Tfrc,22042:up|Serinc2,230779:down|Ceacam1,26365:up|Ccl8,20307:up|Sfrp2,20319:up|Serpina3g,20715:up|Fam20a,208659:down|Npnt,114249:up|Tnfrsf11b,18383:up|Prkar2b,19088:up|S100a1,20193:up|Ice1,218333:down|Aldh1a2,19378:up|Ccl2,20296:up|Ccl7,20306:up|Pdgfc,54635:down|Cst7,13011:up|Ednrb,13618:up|Sema7a,20361:down|Epas1,13819:up|Htra3,78558:down|Uchl5,56207:up|Ddah1,69219:down|Sdpr,20324:down|Ptx3,19288:up|Akr1c18,105349:up|Bmpr1a,12166:down|Ccl12,20293:up|Ccl4,20303:up|Tnc,21923:down|Ret,19713:up|Prg4,96875:down|Plpp3,67916:up|Creb3l1,26427:down|Kitl,17311:up|Hp,15439:up|Ccl9,20308:up|Inhba,16323:down|Rasl11a,68895:up|Bnc2,242509:down|Plat,18791:down|Ccl3,20302:up|Tfpi2,21789:down|Ctla2a,13024:down|Srpx2,68792:down|Crlf1,12931:down|Plod2,26432:down|Vegfa,22339:down|Serpine2,20720:up|Lrrc15,74488:down|Nxn,18230:down|Igf2,16002:up|Pparg,19016:up|Tnf,21926:up|Glp1r,14652:down|Lmcd1,30937:up|Fgfr1,14182:down|Has2,15117:down|Hmox1,15368:up|Sulf1,240725:down|Cd4,12504:up|Ptgs2,19225:down|Grem1,23892:up|Phf11d,219132:up|Tbx15,21384:down|Serping1,12258:up|Errfi1,74155:down|Enpp1,18605:down
response to mechanical stimulus	GO:0009612	4.22E-03	2.14E-02	Ankrd1,107765:down|Tnf,21926:up|Col11a1,12814:down|Htr2a,15558:down|Mmp2,17390:down|Serpine2,20720:up|Igf1,16000:up
kidney development	GO:0001822	4.23E-03	2.14E-02	Fgfr1,14182:down|Sfrp1,20377:up|Sulf1,240725:down|Grem1,23892:up|Bmp4,12159:up|Adipoq,11450:up|Crlf1,12931:down|Ret,19713:up|Vegfa,22339:down|Npnt,114249:up|Fgfr2,14183:up
regulation of JNK cascade	GO:0046328	4.30E-03	2.18E-02	Tnf,21926:up|Trf,22041:up|Sfrp1,20377:up|Wnt5a,22418:down|Sfrp4,20379:up|Il1rn,16181:up|Ceacam1,26365:up|Sfrp2,20319:up
stem cell division	GO:0017145	4.32E-03	2.18E-02	Sfrp2,20319:up|Fgfr1,14182:down|Tgfb2,21808:down|Fgfr2,14183:up
regulation of cardiac muscle cell proliferation	GO:0060043	4.32E-03	2.18E-02	Fgfr1,14182:down|Ncam1,17967:down|Bmpr1a,12166:down|Fgfr2,14183:up
regulation of axon guidance	GO:1902667	4.32E-03	2.18E-02	Plxnd1,67784:up|Cxcl12,20315:up|Wnt5a,22418:down|Vegfa,22339:down
regulation of monooxygenase activity	GO:0032768	4.32E-03	2.18E-02	Vdr,22337:down|Htr2b,15559:down|Tnf,21926:up|Egfr,13649:down
metal ion transport	GO:0030001	4.32E-03	2.18E-02	Htr2b,15559:down|Vdr,22337:down|Cxcl12,20315:up|Htr2a,15558:down|Serpine2,20720:up|Slc40a1,53945:up|Gas6,14456:up|Trf,22041:up|Fxyd1,56188:up|Glp1r,14652:down|Cd4,12504:up|Ccl12,20293:up|Ptgs2,19225:down|Stc1,20855:up|Tfrc,22042:up|Kcnj15,16516:down|Fxyd2,11936:up|Ccl4,20303:up|Bmp4,12159:up|Igf1,16000:up|Tgfb2,21808:down|S100a1,20193:up
regulation of apoptotic signaling pathway	GO:2001233	4.36E-03	2.20E-02	Tnf,21926:up|Tnfrsf23,79201:down|Fgfr1,14182:down|Sfrp1,20377:up|Hmox1,15368:up|Inhba,16323:down|Igf1,16000:up|Bmp4,12159:up|Tnfrsf12a,27279:down|Sfrp2,20319:up|Cxcl12,20315:up|Tgfb2,21808:down|Cx3cr1,13051:down|Creb3l1,26427:down

single-organism localization	GO:1902578	4.36E-03	2.20E-02	Adm,11535:up|Sfrp4,20379:up|C3,12266:up|Ankrd1,107765:down|Trf,22041:up|Cd24a,12484:down|Wnt5a,22418:down|Stc1,20855:up|Il1rn,16181:up|Maob,109731:up|Adipoq,11450:up|Bmp4,12159:up|Igf1,16000:up|Mcoln2,68279:up|Tgfb2,21808:down|Fgfr2,14183:up|Clec4e,56619:up|Htr2b,15559:down|Vdr,22337:down|Fam132b,227358:down|Cxcl12,20315:up|Htr2a,15558:down|Slc16a4,229699:down|Slc40a1,53945:up|Gas6,14456:up|Egfr,13649:down|Sec23a,20334:down|Sfrp1,20377:up|C1qtnf3,81799:down|Tfrc,22042:up|Fxyd2,11936:up|Sncg,20618:up|Serinc2,230779:down|Ceacam1,26365:up|Sfrp2,20319:up|Syt13,80976:up|S100a1,20193:up|Ccl2,20296:up|Ednrb,13618:up|Mmp13,17386:up|Slc29a1,63959:up|Gpm6b,14758:up|Clec4n,56620:up|Steap1,70358:down|Postn,50706:down|Cdo1,12583:up|Ptx3,19288:up|Hvcn1,74096:up|Akr1c18,105349:up|Fabp5,16592:up|Ncam1,17967:down|Ccl12,20293:up|Bmpr1a,12166:down|Ccl4,20303:up|Ank,11732:down|Creb3l1,26427:down|Il1rl1,17082:down|Inhba,16323:down|Ccl3,20302:up|Vegfa,22339:down|Serpine2,20720:up|Lrrc15,74488:down|Anpep,16790:up|Kdelr3,105785:down|Pparg,19016:up|Tnf,21926:up|Fxyd1,56188:up|Glp1r,14652:down|Fgfr1,14182:down|Lpl,16956:up|Hmox1,15368:up|Cd4,12504:up|Ptgs2,19225:down|Grem1,23892:up|Kcnj15,16516:down|Mcoln3,171166:up|Emb,13723:up|Enpp1,18605:down
regulation of carbohydrate biosynthetic process	GO:0043255	4.38E-03	2.20E-02	Has2,15117:down|C1qtnf3,81799:down|Enpp1,18605:down|Igf2,16002:up|Igf1,16000:up|Adipoq,11450:up
Ras protein signal transduction	GO:0007265	4.45E-03	2.24E-02	Ccl9,20308:up|Ccl6,20305:up|Ccl7,20306:up|Ccl2,20296:up|Ccl3,20302:up|Dok2,13449:up|Col1a2,12843:down|Plk2,20620:down|Ccl12,20293:up|Wnt5a,22418:down|Ccl4,20303:up|Igf1,16000:up|Col3a1,12825:down|Ccl8,20307:up|Tgfb2,21808:down|Kitl,17311:up
regulation of cell morphogenesis	GO:0022604	4.49E-03	2.25E-02	Plxnd1,67784:up|Ccl2,20296:up|Ccl3,20302:up|Sema7a,20361:down|Tnfrsf12a,27279:down|Cxcl12,20315:up|Postn,50706:down|Vegfa,22339:down|Sfrp1,20377:up|Has2,15117:down|Ccl12,20293:up|Tgfbr3,21814:up|Wnt5a,22418:down|Grem1,23892:up|Sfrp2,20319:up|Crabp2,12904:down|Ret,19713:up|Tgfb2,21808:down
positive regulation of leukocyte activation	GO:0002696	4.50E-03	2.26E-02	Gas6,14456:up|Cd24a,12484:down|Wnt5a,22418:down|Ccl2,20296:up|Cd4,12504:up|Tfrc,22042:up|Igf1,16000:up|Ceacam1,26365:up|Igf2,16002:up|Il1rl1,17082:down|Tnfsf9,21950:up
vitamin metabolic process	GO:0006766	4.52E-03	2.26E-02	Vdr,22337:down|Tnf,21926:up|Akr1c18,105349:up|Fgfr1,14182:down|Pdxk,216134:up
regulation of viral genome replication	GO:0045069	4.52E-03	2.26E-02	Ceacam1,26365:up|Tnf,21926:up|Oasl1,231655:up|Slpi,20568:up|Oas3,246727:up
cellular response to organic cyclic compound	GO:0071407	4.55E-03	2.27E-02	Htr2b,15559:down|Tnf,21926:up|Pparg,19016:up|Akr1c18,105349:up|Egfr,13649:down|Alpl,11647:up|Sfrp1,20377:up|Inhba,16323:down|Ccl12,20293:up|Igf1,16000:up|Bmp4,12159:up|Adipoq,11450:up|Vdr,22337:down|Mmp2,17390:down|Ifit1,15957:up
regulation of cell activation	GO:0050865	4.55E-03	2.27E-02	Ccl2,20296:up|Ctla2a,13024:down|Serpine2,20720:up|Igf2,16002:up|Gas6,14456:up|Cd24a,12484:down|Hmox1,15368:up|Sfrp1,20377:up|Cd4,12504:up|Wnt5a,22418:down|Tfrc,22042:up|Bmp4,12159:up|Igf1,16000:up|Ceacam1,26365:up|Tnfsf9,21950:up|Il1rl1,17082:down
positive regulation of bone resorption	GO:0045780	4.57E-03	2.27E-02	Trf,22041:up|Egfr,13649:down|Tfrc,22042:up
negative regulation of cytokine production involved in immune response	GO:0002719	4.57E-03	2.27E-02	Tnf,21926:up|Hmox1,15368:up|Tgfb2,21808:down
regulation of macrophage derived foam cell differentiation	GO:0010743	4.57E-03	2.27E-02	Pparg,19016:up|Lpl,16956:up|Adipoq,11450:up
regulation of non-canonical Wnt signaling pathway	GO:2000050	4.57E-03	2.27E-02	Sfrp2,20319:up|Sfrp1,20377:up|Wnt5a,22418:down
positive regulation of bone remodeling	GO:0046852	4.57E-03	2.27E-02	Trf,22041:up|Egfr,13649:down|Tfrc,22042:up
SMAD protein import into nucleus	GO:0007184	4.57E-03	2.27E-02	Tgfb2,21808:down|Bmpr1a,12166:down|Bmp4,12159:up
positive regulation of myoblast fusion	GO:1901741	4.57E-03	2.27E-02	Ccl8,20307:up|Cxcl9,17329:up|Cxcl12,20315:up
regulation of I-kappaB kinase/NF-kappaB signaling	GO:0043122	4.59E-03	2.27E-02	Tnf,21926:up|Plk2,20620:down|Hmox1,15368:up|C1qtnf3,81799:down|Ptgs2,19225:down|Grem1,23892:up|Adipoq,11450:up|Clec4n,56620:up|Il1rl1,17082:down
positive regulation of reactive oxygen species metabolic process	GO:2000379	4.64E-03	2.27E-02	Tnf,21926:up|Akr1c18,105349:up|Ptx3,19288:up|Ddah1,69219:down|Egfr,13649:down|Ptgs2,19225:down
ear morphogenesis	GO:0042471	4.65E-03	2.27E-02	Cthrc1,68588:down|Fgfr1,14182:down|Col11a1,12814:down|Ptk7,71461:down|Wnt5a,22418:down|Aldh1a3,56847:down|Fgfr2,14183:up
virion attachment to host cell	GO:0019062	4.66E-03	2.27E-02	Gas6,14456:up|Lrrc15,74488:down
negative regulation of mesonephros development	GO:0061218	4.66E-03	2.27E-02	Grem1,23892:up|Bmp4,12159:up
cardiac muscle fiber development	GO:0048739	4.66E-03	2.27E-02	Vegfa,22339:down|Bmp4,12159:up
response to UV-A	GO:0070141	4.66E-03	2.27E-02	Egfr,13649:down|Mme,17380:up
positive regulation of axon extension involved in axon guidance	GO:0048842	4.66E-03	2.27E-02	Cxcl12,20315:up|Vegfa,22339:down
positive regulation of axon guidance	GO:1902669	4.66E-03	2.27E-02	Cxcl12,20315:up|Vegfa,22339:down

negative regulation of non-canonical Wnt signaling pathway	GO:2000051	4.66E-03	2.27E-02	Sfrp2,20319:up|Sfrp1,20377:up
forebrain ventricular zone progenitor cell division	GO:0021869	4.66E-03	2.27E-02	Fgfr1,14182:down|Fgfr2,14183:up
regulation of endodermal cell differentiation	GO:1903224	4.66E-03	2.27E-02	Col5a2,12832:down|Col5a1,12831:down
negative regulation of receptor biosynthetic process	GO:0010871	4.66E-03	2.27E-02	Pparg,19016:up|Adipoq,11450:up
negative regulation of bone development	GO:1903011	4.66E-03	2.27E-02	Fam101b,76566:down|Grem1,23892:up
stress response to metal ion	GO:0097501	4.66E-03	2.27E-02	Mt2,17750:up|Mt1,17748:up
adhesion of symbiont to host cell	GO:0044650	4.66E-03	2.27E-02	Gas6,14456:up|Lrrc15,74488:down
positive regulation of catecholamine metabolic process	GO:0045915	4.66E-03	2.27E-02	Maob,109731:up|Epas1,13819:up
regulation of phosphatidylinositol biosynthetic process	GO:0010511	4.66E-03	2.27E-02	Htr2b,15559:down|Htr2a,15558:down
regulation of peptidyl-tyrosine autophosphorylation	GO:1900084	4.66E-03	2.27E-02	Vegfa,22339:down|Grem1,23892:up
integrin biosynthetic process	GO:0045112	4.66E-03	2.27E-02	Col5a1,12831:down|Tgfb2,21808:down
positive regulation of dopamine metabolic process	GO:0045964	4.66E-03	2.27E-02	Maob,109731:up|Epas1,13819:up
cellular response to nitrogen compound	GO:1901699	4.66E-03	2.27E-02	Htr2b,15559:down|Col5a2,12832:down|Tnf,21926:up|Pparg,19016:up|Col1a2,12843:down|Egfr,13649:down|Sfrp1,20377:up|Pdgfc,54635:down|Col3a1,12825:down|Igf1,16000:up|Adipoq,11450:up|Mmp2,17390:down|Ifit1,15957:up|Enpp1,18605:down|Igf2,16002:up
NF-kappaB import into nucleus	GO:0042348	4.76E-03	2.30E-02	Tnf,21926:up|C1qtnf3,81799:down|Ptgs2,19225:down|Grem1,23892:up
activated T cell proliferation	GO:0050798	4.76E-03	2.30E-02	Cd24a,12484:down|Igf2,16002:up|Tnfsf9,21950:up|Igf1,16000:up
regulation of NF-kappaB import into nucleus	GO:0042345	4.76E-03	2.30E-02	Tnf,21926:up|C1qtnf3,81799:down|Ptgs2,19225:down|Grem1,23892:up
negative regulation of hemostasis	GO:1900047	4.76E-03	2.30E-02	Ceacam1,26365:up|Kng1,16644:up|Serping1,12258:up|Serpine2,20720:up
cell adhesion mediated by integrin	GO:0033627	4.76E-03	2.30E-02	Cd24a,12484:down|Ret,19713:up|Tgfb2,21808:down|Npnt,114249:up
negative regulation of blood coagulation	GO:0030195	4.76E-03	2.30E-02	Ceacam1,26365:up|Kng1,16644:up|Serping1,12258:up|Serpine2,20720:up
regulation of leukocyte activation	GO:0002694	4.77E-03	2.31E-02	Gas6,14456:up|Cd24a,12484:down|Sfrp1,20377:up|Hmox1,15368:up|Wnt5a,22418:down|Ccl2,20296:up|Cd4,12504:up|Tfrc,22042:up|Igf1,16000:up|Bmp4,12159:up|Ctla2a,13024:down|Ceacam1,26365:up|Igf2,16002:up|Il1rl1,17082:down|Tnfsf9,21950:up
lipid storage	GO:0019915	4.85E-03	2.34E-02	Tnf,21926:up|Pparg,19016:up|Lpl,16956:up|C3,12266:up|Enpp1,18605:down
interleukin-1 production	GO:0032612	4.85E-03	2.34E-02	Ceacam1,26365:up|Gas6,14456:up|Wnt5a,22418:down|Errfi1,74155:down|Ccl3,20302:up
interleukin-6 production	GO:0032635	4.88E-03	2.35E-02	Tnf,21926:up|Gas6,14456:up|Prg4,96875:down|C1qtnf3,81799:down|Wnt5a,22418:down|Figf,14205:up|Tnfsf9,21950:up
regulation of organ growth	GO:0046620	4.91E-03	2.37E-02	Fgfr1,14182:down|Ncam1,17967:down|Bmpr1a,12166:down|Wisp1,22402:down|Fgfr2,14183:up|Igf1,16000:up
production of molecular mediator of immune response	GO:0002440	5.08E-03	2.44E-02	Tnf,21926:up|Gas6,14456:up|Hmox1,15368:up|Wnt5a,22418:down|Tfrc,22042:up|Sema7a,20361:down|Ceacam1,26365:up|Tgfb2,21808:down
mammary gland development	GO:0030879	5.11E-03	2.46E-02	Vdr,22337:down|Wnt5a,22418:down|Vegfa,22339:down|Fgfr2,14183:up|Igf1,16000:up|Cdo1,12583:up|Bmp4,12159:up
protein localization to nucleus	GO:0034504	5.18E-03	2.49E-02	Tnf,21926:up|Gas6,14456:up|Akr1c18,105349:up|Egfr,13649:down|C1qtnf3,81799:down|Bmpr1a,12166:down|Ptgs2,19225:down|Grem1,23892:up|Igf1,16000:up|Bmp4,12159:up|Ceacam1,26365:up|Sfrp2,20319:up|Tgfb2,21808:down
smooth muscle contraction	GO:0006939	5.19E-03	2.49E-02	Htr2b,15559:down|Sulf1,240725:down|Htr2a,15558:down|Ptgs2,19225:down|Npnt,114249:up|Ednrb,13618:up
epithelial cell apoptotic process	GO:1904019	5.19E-03	2.49E-02	Gas6,14456:up|Akr1c18,105349:up|Hmox1,15368:up|Sfrp4,20379:up|Igf1,16000:up|Bmp4,12159:up
endoderm development	GO:0007492	5.20E-03	2.49E-02	Col5a2,12832:down|Col5a1,12831:down|Inhba,16323:down|Bmpr1a,12166:down|Bmp4,12159:up
ventricular septum development	GO:0003281	5.20E-03	2.49E-02	Ptk7,71461:down|Wnt5a,22418:down|Tgfb2,21808:down|Fgfr2,14183:up|Bmp4,12159:up
regulation of steroid biosynthetic process	GO:0050810	5.20E-03	2.49E-02	Vdr,22337:down|Tnf,21926:up|Akr1c18,105349:up|Igf2,16002:up|Igf1,16000:up

neuron projection extension involved in neuron projection guidance	GO:1902284	5.23E-03	2.49E-02	Plxnd1,67784:up|Cxcl12,20315:up|Wnt5a,22418:down|Vegfa,22339:down
regulation of transcription from RNA polymerase II promoter in response to stress	GO:0043618	5.23E-03	2.49E-02	Hmox1,15368:up|Vegfa,22339:down|Creb3l1,26427:down|Epas1,13819:up
negative regulation of leukocyte mediated immunity	GO:0002704	5.23E-03	2.49E-02	Ceacam1,26365:up|Clec2d,93694:up|Hmox1,15368:up|Igf2,16002:up
axon extension involved in axon guidance	GO:0048846	5.23E-03	2.49E-02	Plxnd1,67784:up|Cxcl12,20315:up|Wnt5a,22418:down|Vegfa,22339:down
negative regulation of peptidyl-tyrosine phosphorylation	GO:0050732	5.23E-03	2.49E-02	Sfrp2,20319:up|Sfrp1,20377:up|Errfi1,74155:down|Igf1,16000:up
retinoid metabolic process	GO:0001523	5.23E-03	2.49E-02	Crabp2,12904:down|Akr1c18,105349:up|Aldh1a2,19378:up|Aldh1a3,56847:down
regulation of metanephros development	GO:0072215	5.30E-03	2.51E-02	Ret,19713:up|Bmp4,12159:up|Adipoq,11450:up
post-anal tail morphogenesis	GO:0036342	5.30E-03	2.51E-02	Chst11,58250:down|Sfrp2,20319:up|Wnt5a,22418:down
cellular metabolic compound salvage	GO:0043094	5.30E-03	2.51E-02	Pdxk,216134:up|Uck2,80914:down|Ampd3,11717:up
positive regulation of NF-kappaB import into nucleus	GO:0042346	5.30E-03	2.51E-02	Tnf,21926:up|Ptgs2,19225:down|Grem1,23892:up
regulation of cell migration involved in sprouting angiogenesis	GO:0090049	5.30E-03	2.51E-02	Srpx2,68792:down|Vegfa,22339:down|Ptgs2,19225:down
viral life cycle	GO:0019058	5.51E-03	2.61E-02	Tnf,21926:up|Gas6,14456:up|Ptx3,19288:up|Slpi,20568:up|Ccl4,20303:up|Ceacam1,26365:up|Oasl1,231655:up|Lrrc15,74488:down|Oas3,246727:up
regulation of oxidoreductase activity	GO:0051341	5.57E-03	2.63E-02	Vdr,22337:down|Htr2b,15559:down|Tnf,21926:up|Egfr,13649:down|Hp,15439:up
carboxylic acid metabolic process	GO:0019752	5.70E-03	2.69E-02	P4ha1,18451:down|Hpgd,15446:up|Aldh1a2,19378:up|C3,12266:up|Ddah1,69219:down|Fabp4,11770:up|Fam132b,227358:down|Htr2a,15558:down|Gfpt2,14584:down|Npl,74091:up|Cdo1,12583:up|Pparg,19016:up|Ggt5,23887:up|Akr1c18,105349:up|Lpl,16956:up|Ptgs2,19225:down|Serinc2,230779:down|Adipoq,11450:up|Hal,15109:up|Igf1,16000:up|Crabp2,12904:down|Gatm,67092:down|Aldh1a3,56847:down|Prkar2b,19088:up
negative regulation of secretion by cell	GO:1903531	5.71E-03	2.69E-02	Tnf,21926:up|Pparg,19016:up|Gas6,14456:up|Sfrp1,20377:up|Hmox1,15368:up|C1qtnf3,81799:down|Il1rn,16181:up|Maob,109731:up|Adipoq,11450:up
regulation of cardiac muscle hypertrophy	GO:0010611	5.73E-03	2.69E-02	Lmcd1,30937:up|Wisp1,22402:down|Errfi1,74155:down|Igf1,16000:up
positive regulation of transcription factor import into nucleus	GO:0042993	5.73E-03	2.69E-02	Ceacam1,26365:up|Tnf,21926:up|Ptgs2,19225:down|Grem1,23892:up
positive regulation of axon extension	GO:0045773	5.73E-03	2.69E-02	Tnfrsf12a,27279:down|Cxcl12,20315:up|Vegfa,22339:down|Sema7a,20361:down
negative regulation of coagulation	GO:0050819	5.73E-03	2.69E-02	Ceacam1,26365:up|Kng1,16644:up|Serping1,12258:up|Serpine2,20720:up
regulation of cellular carbohydrate metabolic process	GO:0010675	5.73E-03	2.69E-02	Igfbp4,16010:up|C1qtnf3,81799:down|Igf1,16000:up|Adipoq,11450:up|Htr2a,15558:down|Igfbp3,16009:up|Igf2,16002:up|Enpp1,18605:down
regulation of viral life cycle	GO:1903900	5.87E-03	2.76E-02	Ceacam1,26365:up|Tnf,21926:up|Ptx3,19288:up|Oasl1,231655:up|Slpi,20568:up|Oas3,246727:up|Ccl4,20303:up
cell fate commitment	GO:0045165	5.90E-03	2.77E-02	Pparg,19016:up|Fgfr1,14182:down|Ly9,17085:up|Sfrp1,20377:up|Wnt5a,22418:down|Bmpr1a,12166:down|Epas1,13819:up|Bmp4,12159:up|Sfrp2,20319:up|Tgfb2,21808:down|Fgfr2,14183:up
fibroblast growth factor receptor signaling pathway	GO:0008543	5.95E-03	2.79E-02	Flrt2,399558:down|Fgfr1,14182:down|Sulf1,240725:down|Wnt5a,22418:down|Fgfr2,14183:up
negative regulation of leukocyte proliferation	GO:0070664	5.95E-03	2.79E-02	Ceacam1,26365:up|Cd24a,12484:down|Ccl12,20293:up|Grem1,23892:up|Bmp4,12159:up
negative regulation of synaptic transmission	GO:0050805	5.95E-03	2.79E-02	Plk2,20620:down|Gpm6b,14758:up|Htr2a,15558:down|Ptgs2,19225:down|Adipoq,11450:up
embryonic digit morphogenesis	GO:0042733	5.95E-03	2.79E-02	Chst11,58250:down|Sfrp2,20319:up|Wnt5a,22418:down|Bmpr1a,12166:down|Bmp4,12159:up
embryonic organ morphogenesis	GO:0048562	5.96E-03	2.79E-02	Cthrc1,68588:down|Fgfr1,14182:down|Col11a1,12814:down|Ptk7,71461:down|Wnt5a,22418:down|Aldh1a2,19378:up|Bmp4,12159:up|Chst11,58250:down|Tbx15,21384:down|Tgfb2,21808:down|Aldh1a3,56847:down|Fgfr2,14183:up
negative regulation of stem cell proliferation	GO:2000647	6.11E-03	2.84E-02	Cd24a,12484:down|Wnt5a,22418:down|Bmp4,12159:up
endochondral bone growth	GO:0003416	6.11E-03	2.84E-02	Stc1,20855:up|Bnc2,242509:down|Mmp13,17386:up
negative regulation of interleukin-1 production	GO:0032692	6.11E-03	2.84E-02	Ceacam1,26365:up|Gas6,14456:up|Errfi1,74155:down

positive regulation of positive chemotaxis	GO:0050927	6.11E-03	2.84E-02	Cxcl12,20315:up|Vegfa,22339:down|Figf,14205:up
response to prostaglandin	GO:0034694	6.11E-03	2.84E-02	Akr1c18,105349:up|Tgfbr3,21814:up|Ptgir,19222:up
paraxial mesoderm development	GO:0048339	6.11E-03	2.84E-02	Fgfr1,14182:down|Wnt5a,22418:down|Bmpr1a,12166:down
positive regulation of phospholipase C activity	GO:0010863	6.11E-03	2.84E-02	Htr2b,15559:down|Fgfr1,14182:down|Htr2a,15558:down
positive regulation of branching involved in ureteric bud morphogenesis	GO:0090190	6.11E-03	2.84E-02	Vegfa,22339:down|Grem1,23892:up|Bmp4,12159:up
regulation of myoblast fusion	GO:1901739	6.11E-03	2.84E-02	Ccl8,20307:up|Cxcl9,17329:up|Cxcl12,20315:up
female gonad development	GO:0008585	6.11E-03	2.84E-02	Sfrp1,20377:up|Vegfa,22339:down|Inhba,16323:down|Mmp2,17390:down|Kitl,17311:up|Bmp4,12159:up
regulation of axon extension	GO:0030516	6.11E-03	2.84E-02	Tnfrsf12a,27279:down|Plxnd1,67784:up|Cxcl12,20315:up|Wnt5a,22418:down|Vegfa,22339:down|Sema7a,20361:down
regulation of reactive oxygen species metabolic process	GO:2000377	6.19E-03	2.87E-02	Tnf,21926:up|Akr1c18,105349:up|Ptx3,19288:up|Egfr,13649:down|Hp,15439:up|Ptgs2,19225:down|Igf1,16000:up|Ddah1,69219:down
negative regulation of metabolic process	GO:0009892	6.20E-03	2.87E-02	Flrt2,399558:down|Sfrp4,20379:up|Ednrb,13618:up|Cst7,13011:up|Epas1,13819:up|Ankrd1,107765:down|Igfbp3,16009:up|Cd109,235505:down|Loxl2,94352:down|Loxl3,16950:down|Akr1c18,105349:up|Ptx3,19288:up|Wnt5a,22418:down|Ccl4,20303:up|Adipoq,11450:up|Bmp4,12159:up|Igf1,16000:up|Prg4,96875:down|Tgfb2,21808:down|Plpp3,67916:up|Fgfr2,14183:up|Htr2b,15559:down|Kng1,16644:up|Hp,15439:up|Slpi,20568:up|Aspn,66695:down|Plat,18791:down|Tfpi2,21789:down|Vdr,22337:down|Ctla2a,13024:down|Fabp4,11770:up|Vegfa,22339:down|Serpine2,20720:up|Lrrc15,74488:down|Nxn,18230:down|Pparg,19016:up|Gas6,14456:up|Tnf,21926:up|Lmcd1,30937:up|Fgfr1,14182:down|Egfr,13649:down|Hmox1,15368:up|Sfrp1,20377:up|C1qtnf3,81799:down|Tgfbr3,21814:up|Grem1,23892:up|Ceacam1,26365:up|Sfrp2,20319:up|Serping1,12258:up|Serpina3g,20715:up|Prkar2b,19088:up|Errfi1,74155:down|Enpp1,18605:down|S100a1,20193:up
peptidyl-amino acid modification	GO:0018193	6.21E-03	2.88E-02	P4ha1,18451:down|Efemp1,216616:up|Pdgfc,54635:down|Crlf1,12931:down|Vegfa,22339:down|Htr2a,15558:down|Igf2,16002:up|Mamdc2,71738:down|Gas6,14456:up|Tnf,21926:up|Plk2,20620:down|Fgfr1,14182:down|Egfr,13649:down|Cd24a,12484:down|Sfrp1,20377:up|Wnt5a,22418:down|Cd4,12504:up|Grem1,23892:up|Adipoq,11450:up|Igf1,16000:up|Sfrp2,20319:up|Ret,19713:up|Plpp3,67916:up|Errfi1,74155:down|Fgfr2,14183:up|Kitl,17311:up
patterning of blood vessels	GO:0001569	6.26E-03	2.89E-02	Sfrp2,20319:up|Plxnd1,67784:up|Cxcl12,20315:up|Vegfa,22339:down
diterpenoid metabolic process	GO:0016101	6.26E-03	2.89E-02	Crabp2,12904:down|Akr1c18,105349:up|Aldh1a2,19378:up|Aldh1a3,56847:down
brown fat cell differentiation	GO:0050873	6.26E-03	2.89E-02	Pparg,19016:up|Fabp4,11770:up|Ptgs2,19225:down|Adipoq,11450:up
positive regulation of DNA metabolic process	GO:0051054	6.34E-03	2.92E-02	Fgfr1,14182:down|Egfr,13649:down|Pdgfc,54635:down|Tfrc,22042:up|Grem1,23892:up|Igf1,16000:up|Bmp4,12159:up|Igfbp3,16009:up|Kitl,17311:up
cardiac muscle hypertrophy	GO:0003300	6.36E-03	2.92E-02	Htr2b,15559:down|Lmcd1,30937:up|Wisp1,22402:down|Errfi1,74155:down|Igf1,16000:up
positive regulation of canonical Wnt signaling pathway	GO:0090263	6.36E-03	2.92E-02	Sfrp2,20319:up|Sfrp1,20377:up|Rspo2,239405:up|Sfrp4,20379:up|Fgfr2,14183:up
canonical Wnt signaling pathway	GO:0060070	6.40E-03	2.92E-02	Cthrc1,68588:down|Sfrp1,20377:up|Ptk7,71461:down|Wnt5a,22418:down|Sfrp4,20379:up|Grem1,23892:up|Sfrp2,20319:up|Rspo2,239405:up|Plpp3,67916:up|Fgfr2,14183:up
negative regulation of Wnt signaling pathway	GO:0030178	6.42E-03	2.92E-02	Cthrc1,68588:down|Sfrp2,20319:up|Sfrp1,20377:up|Wnt5a,22418:down|Sfrp4,20379:up|Nxn,18230:down|Grem1,23892:up
cellular carbohydrate metabolic process	GO:0044262	6.43E-03	2.92E-02	Igfbp4,16010:up|Has2,15117:down|C1qtnf3,81799:down|Aoah,27052:up|Igf1,16000:up|Adipoq,11450:up|Sord,20322:up|Htr2a,15558:down|Igfbp3,16009:up|Enpp1,18605:down|Igf2,16002:up
negative regulation of canonical Wnt signaling pathway	GO:0090090	6.44E-03	2.92E-02	Cthrc1,68588:down|Sfrp2,20319:up|Sfrp1,20377:up|Wnt5a,22418:down|Sfrp4,20379:up|Grem1,23892:up
negative regulation of metanephros development	GO:0072217	6.44E-03	2.92E-02	Bmp4,12159:up|Adipoq,11450:up
nucleotide salvage	GO:0043173	6.44E-03	2.92E-02	Uck2,80914:down|Ampd3,11717:up
regulation of calcidiol 1-monooxygenase activity	GO:0060558	6.44E-03	2.92E-02	Vdr,22337:down|Tnf,21926:up
parturition	GO:0007567	6.44E-03	2.92E-02	Akr1c18,105349:up|Hpgd,15446:up
positive regulation of phospholipid biosynthetic process	GO:0071073	6.44E-03	2.92E-02	Htr2b,15559:down|Htr2a,15558:down
ferrous iron transport	GO:0015684	6.44E-03	2.92E-02	Trf,22041:up|Slc40a1,53945:up
bud elongation involved in lung branching	GO:0060449	6.44E-03	2.92E-02	Fgfr2,14183:up|Bmp4,12159:up
fibroblast growth factor production	GO:0090269	6.44E-03	2.92E-02	Fgfr1,14182:down|Ptgs2,19225:down

convergent extension involved in gastrulation	GO:0060027	6.44E-03	2.92E-02	Sfrp1,20377:up|Wnt5a,22418:down
epithelial cell proliferation involved in salivary gland morphogenesis	GO:0060664	6.44E-03	2.92E-02	Tnf,21926:up|Fgfr2,14183:up
positive regulation of chemokine (C-X-C motif) ligand 2 production	GO:2000343	6.44E-03	2.92E-02	Tnf,21926:up|Postn,50706:down
cellular response to zinc ion	GO:0071294	6.44E-03	2.92E-02	Hvcn1,74096:up|Mt1,17748:up
regulation of glycogen (starch) synthase activity	GO:2000465	6.44E-03	2.92E-02	Igf2,16002:up|Adipoq,11450:up
blood vessel maturation	GO:0001955	6.44E-03	2.92E-02	Lyl1,17095:up|Mmp2,17390:down
regulation of branching involved in lung morphogenesis	GO:0061046	6.44E-03	2.92E-02	Tnf,21926:up|Bmp4,12159:up
regulation of fibroblast growth factor production	GO:0090270	6.44E-03	2.92E-02	Fgfr1,14182:down|Ptgs2,19225:down
positive regulation of vasculogenesis	GO:2001214	6.44E-03	2.92E-02	Ceacam1,26365:up|Adm,11535:up
regulation of apoptotic cell clearance	GO:2000425	6.44E-03	2.92E-02	Ccl2,20296:up|C3,12266:up
positive regulation of cell activation	GO:0050867	6.62E-03	2.99E-02	Gas6,14456:up|Cd24a,12484:down|Wnt5a,22418:down|Ccl2,20296:up|Cd4,12504:up|Tfrc,22042:up|Igf1,16000:up|Ceacam1,26365:up|Igf2,16002:up|Il1rl1,17082:down|Tnfsf9,21950:up
positive regulation of lymphocyte proliferation	GO:0050671	6.71E-03	3.03E-02	Ceacam1,26365:up|Cd24a,12484:down|Cd4,12504:up|Tfrc,22042:up|Igf2,16002:up|Tnfsf9,21950:up|Igf1,16000:up
regulation of vasoconstriction	GO:0019229	6.78E-03	3.05E-02	Adm,11535:up|Egfr,13649:down|Htr2a,15558:down|Ptgs2,19225:down|Mmp2,17390:down
response to heat	GO:0009408	6.78E-03	3.05E-02	Tfec,21426:up|Hmox1,15368:up|Hspb7,29818:up|Ccl2,20296:up|Igf1,16000:up
regulation of epithelial to mesenchymal transition	GO:0010717	6.78E-03	3.05E-02	Sfrp2,20319:up|Sfrp1,20377:up|Tgfb2,21808:down|Tgfbr3,21814:up|Grem1,23892:up
leukocyte homeostasis	GO:0001776	6.78E-03	3.05E-02	Ceacam1,26365:up|Cd24a,12484:down|Tgfb2,21808:down|Ccl2,20296:up|Slc40a1,53945:up|Kitl,17311:up
development of primary female sexual characteristics	GO:0046545	6.78E-03	3.05E-02	Sfrp1,20377:up|Vegfa,22339:down|Inhba,16323:down|Mmp2,17390:down|Kitl,17311:up|Bmp4,12159:up
positive regulation of receptor-mediated endocytosis	GO:0048260	6.82E-03	3.06E-02	Trf,22041:up|Vegfa,22339:down|Sfrp4,20379:up|Grem1,23892:up
negative regulation of interleukin-6 production	GO:0032715	6.82E-03	3.06E-02	Tnf,21926:up|Gas6,14456:up|Prg4,96875:down|C1qtnf3,81799:down
regulation of muscle hypertrophy	GO:0014743	6.82E-03	3.06E-02	Lmcd1,30937:up|Wisp1,22402:down|Errfi1,74155:down|Igf1,16000:up
carbohydrate metabolic process	GO:0005975	6.86E-03	3.08E-02	Igfbp4,16010:up|Aoah,27052:up|Sord,20322:up|Htr2a,15558:down|Gfpt2,14584:down|Npl,74091:up|Igfbp3,16009:up|Igf2,16002:up|Man2a2,140481:up|Tnf,21926:up|Fabp5,16592:up|Has2,15117:down|C1qtnf3,81799:down|Adipoq,11450:up|Igf1,16000:up|Chst11,58250:down|Hpse,15442:up|Vegfb,22340:up|Sdf2l1,64136:up|Enpp1,18605:down
positive regulation of T cell activation	GO:0050870	6.95E-03	3.10E-02	Cd24a,12484:down|Ccl2,20296:up|Cd4,12504:up|Tfrc,22042:up|Igf1,16000:up|Ceacam1,26365:up|Igf2,16002:up|Tnfsf9,21950:up
cochlea morphogenesis	GO:0090103	6.98E-03	3.10E-02	Cthrc1,68588:down|Ptk7,71461:down|Wnt5a,22418:down
cellular response to nutrient	GO:0031670	6.98E-03	3.10E-02	Vdr,22337:down|Gas6,14456:up|Postn,50706:down
somatic stem cell division	GO:0048103	6.98E-03	3.10E-02	Fgfr1,14182:down|Tgfb2,21808:down|Fgfr2,14183:up
positive regulation of receptor internalization	GO:0002092	6.98E-03	3.10E-02	Vegfa,22339:down|Sfrp4,20379:up|Grem1,23892:up
regulation of transforming growth factor beta production	GO:0071634	6.98E-03	3.10E-02	Cd24a,12484:down|Tgfb2,21808:down|Ptgs2,19225:down
positive regulation of mesonephros development	GO:0061213	6.98E-03	3.10E-02	Vegfa,22339:down|Grem1,23892:up|Bmp4,12159:up
regulation of positive chemotaxis	GO:0050926	6.98E-03	3.10E-02	Cxcl12,20315:up|Vegfa,22339:down|Figf,14205:up
monovalent inorganic anion homeostasis	GO:0055083	6.98E-03	3.10E-02	Sfrp4,20379:up|Stc1,20855:up|Enpp1,18605:down
collagen catabolic process	GO:0030574	6.98E-03	3.10E-02	Mmp2,17390:down|Mmp13,17386:up|Mrc2,17534:down

macrophage derived foam cell differentiation	GO:0010742	6.98E-03	3.10E-02	Pparg,19016:up|Lpl,16956:up|Adipoq,11450:up
pharyngeal system development	GO:0060037	6.98E-03	3.10E-02	Tgfb2,21808:down|Bmpr1a,12166:down|Bmp4,12159:up
phosphatidylinositol 3-kinase signaling	GO:0014065	7.14E-03	3.16E-02	Htr2b,15559:down|Tgfb2,21808:down|Htr2a,15558:down|Pdgfc,54635:down|Serpine2,20720:up|Igf1,16000:up
positive regulation of cell-substrate adhesion	GO:0010811	7.14E-03	3.16E-02	Has2,15117:down|Vegfa,22339:down|Npnt,114249:up|Smoc2,64074:down|Col8a1,12837:down|Ninj1,18081:up
cell-cell adhesion via plasma-membrane adhesion molecules	GO:0098742	7.31E-03	3.23E-02	Ceacam1,26365:up|Pcdh19,279653:down|Cd24a,12484:down|Ret,19713:up|Tgfb2,21808:down|Il1rn,16181:up|Adipoq,11450:up
positive regulation of mononuclear cell proliferation	GO:0032946	7.31E-03	3.23E-02	Ceacam1,26365:up|Cd24a,12484:down|Cd4,12504:up|Tfrc,22042:up|Igf2,16002:up|Tnfsf9,21950:up|Igf1,16000:up
apoptotic signaling pathway	GO:0097190	7.33E-03	3.24E-02	Inhba,16323:down|Tnfrsf12a,27279:down|Vdr,22337:down|Cxcl12,20315:up|Cx3cr1,13051:down|Tnf,21926:up|Tnfrsf23,79201:down|Cd24a,12484:down|Fgfr1,14182:down|Hmox1,15368:up|Sfrp1,20377:up|Bmp4,12159:up|Igf1,16000:up|Sfrp2,20319:up|Tgfb2,21808:down|Tnfrsf11b,18383:up|Creb3l1,26427:down|Kitl,17311:up
regulation of extrinsic apoptotic signaling pathway via death domain receptors	GO:1902041	7.41E-03	3.27E-02	Sfrp2,20319:up|Tnfrsf23,79201:down|Sfrp1,20377:up|Hmox1,15368:up
mesenchyme morphogenesis	GO:0072132	7.41E-03	3.27E-02	Fgfr1,14182:down|Wnt5a,22418:down|Tgfb2,21808:down|Bmpr1a,12166:down
regulation of phospholipase activity	GO:0010517	7.41E-03	3.27E-02	Htr2b,15559:down|Fgfr1,14182:down|Htr2a,15558:down|Fgfr2,14183:up
regulation of DNA-templated transcription in response to stress	GO:0043620	7.41E-03	3.27E-02	Hmox1,15368:up|Vegfa,22339:down|Creb3l1,26427:down|Epas1,13819:up
carbohydrate biosynthetic process	GO:0016051	7.49E-03	3.30E-02	Has2,15117:down|C1qtnf3,81799:down|Igf1,16000:up|Adipoq,11450:up|Sord,20322:up|Chst11,58250:down|Igf2,16002:up|Enpp1,18605:down
positive regulation of I-kappaB kinase/NF-kappaB signaling	GO:0043123	7.63E-03	3.36E-02	Tnf,21926:up|Plk2,20620:down|Clec4n,56620:up|Hmox1,15368:up|Ptgs2,19225:down|Grem1,23892:up|Adipoq,11450:up
striated muscle hypertrophy	GO:0014897	7.68E-03	3.38E-02	Htr2b,15559:down|Lmcd1,30937:up|Wisp1,22402:down|Errfi1,74155:down|Igf1,16000:up
JNK cascade	GO:0007254	7.78E-03	3.42E-02	Tnf,21926:up|Trf,22041:up|Sfrp1,20377:up|Wnt5a,22418:down|Sfrp4,20379:up|Il1rn,16181:up|Ceacam1,26365:up|Sfrp2,20319:up
response to vitamin	GO:0033273	7.92E-03	3.46E-02	Vdr,22337:down|Gas6,14456:up|Postn,50706:down
foam cell differentiation	GO:0090077	7.92E-03	3.46E-02	Pparg,19016:up|Lpl,16956:up|Adipoq,11450:up
innervation	GO:0060384	7.92E-03	3.46E-02	Ret,19713:up|Sulf1,240725:down|Serpine2,20720:up
receptor biosynthetic process	GO:0032800	7.92E-03	3.46E-02	Tnf,21926:up|Pparg,19016:up|Adipoq,11450:up
positive regulation of myoblast differentiation	GO:0045663	7.92E-03	3.46E-02	Ccl8,20307:up|Cxcl9,17329:up|Igfbp3,16009:up
labyrinthine layer morphogenesis	GO:0060713	7.92E-03	3.46E-02	Adm,11535:up|Gjb5,14622:down|Fgfr2,14183:up
transforming growth factor beta production	GO:0071604	7.92E-03	3.46E-02	Cd24a,12484:down|Tgfb2,21808:down|Ptgs2,19225:down
regulation of interleukin-1 secretion	GO:0050704	7.92E-03	3.46E-02	Gas6,14456:up|Wnt5a,22418:down|Ccl3,20302:up
regulation of insulin-like growth factor receptor signaling pathway	GO:0043567	7.92E-03	3.46E-02	Igfbp4,16010:up|Igfbp3,16009:up|Igf1,16000:up
regulation of intracellular transport	GO:0032386	7.94E-03	3.47E-02	Ice1,218333:down|Ccl2,20296:up|Gas6,14456:up|Tnf,21926:up|Egfr,13649:down|Hmox1,15368:up|Cd4,12504:up|Bmpr1a,12166:down|C1qtnf3,81799:down|Ptgs2,19225:down|Grem1,23892:up|Adipoq,11450:up|Bmp4,12159:up|Igf1,16000:up|Ceacam1,26365:up|Sfrp2,20319:up|Tgfb2,21808:down
protein trimerization	GO:0070206	8.04E-03	3.51E-02	Col1a2,12843:down|C1qtnf3,81799:down|C1qtnf6,72709:down|Adipoq,11450:up
blood vessel endothelial cell migration	GO:0043534	8.16E-03	3.55E-02	Prl2c2,18811:up|Srpx2,68792:down|Vegfa,22339:down|Ptgs2,19225:down|Grem1,23892:up
positive regulation of axonogenesis	GO:0050772	8.16E-03	3.55E-02	Tnfrsf12a,27279:down|Crabp2,12904:down|Cxcl12,20315:up|Vegfa,22339:down|Sema7a,20361:down

organic substance metabolic process	GO:0071704	8.20E-03	3.57E-02	Fstl3,83554:down|Igfbp4,16010:up|Adm,11535:up|Flrt2,399558:down|Tfec,21426:up|Sfrp4,20379:up|Serpinh1,12406:down|Lyl1,17095:up|Gstt1,14871:up|Blvrb,233016:up|Igfbp3,16009:up|Plk2,20620:down|Cd24a,12484:down|Mme,17380:up|Fkbp14,231997:down|Il1rn,16181:up|Maob,109731:up|Bmp4,12159:up|Hpse,15442:up|Ptgir,19222:up|Fgfr2,14183:up|Htr2b,15559:down|Ccl6,20305:up|Slpi,20568:up|Aspn,66695:down|Fam132b,227358:down|Nabp1,109019:down|Mmp2,17390:down|Oas3,246727:up|Pappa,18491:down|Slc40a1,53945:up|Egfr,13649:down|C1rb,667277:up|Tfrc,22042:up|Ccl8,20307:up|Gstm1,14862:up|Serpina3g,20715:up|Fam20a,208659:down|Aldh1a2,19378:up|Ccl2,20296:up|Ednrb,13618:up|Ampd3,11717:up|Mmp13,17386:up|Htra3,78558:down|Uchl5,56207:up|Sord,20322:up|Enpp3,209558:down|Cdo1,12583:up|Dok2,13449:up|Mamdc2,71738:down|Ptx3,19288:up|Ccl4,20303:up|Chst11,58250:down|Vegfb,22340:up|Aldh1a3,56847:down|Plpp3,67916:up|Mrc2,17534:down|Ccl9,20308:up|Inhba,16323:down|Bnc2,242509:down|Tfpi2,21789:down|Crlf1,12931:down|C1s1,50908:up|Vegfa,22339:down|Gfpt2,14584:down|Cbr2,12409:up|Serpine2,20720:up|Nxn,18230:down|Igf2,16002:up|Tnf,21926:up|Fgfr1,14182:down|Lpl,16956:up|Sulf1,240725:down|Cd4,12504:up|Col3a1,12825:down|Tbx15,21384:down|Serping1,12258:up|Uck2,80914:down|Enpp1,18605:down|Hpgd,15446:up|C3,12266:up|Enpp5,83965:up|Ankrd1,107765:down|Cd109,235505:down|Ggt5,23887:up|Loxl2,94352:down|Trf,22041:up|Loxl3,16950:down|Adam12,11489:down|Wnt5a,22418:down|Mark1,226778:up|Adipoq,11450:up|Hal,15109:up|Igf1,16000:up|Crabp2,12904:down|Tgfb2,21808:down|F3,14066:up|Adamts4,240913:down|Kng1,16644:up|Prl2c2,18811:up|Efemp1,216616:up|Saa3,20210:up|Gdpd1,66569:up|Vdr,22337:down|Pdia5,72599:down|Fabp4,11770:up|Htr2a,15558:down|Gas6,14456:up|Col11a1,12814:down|Sfrp1,20377:up|C1qtnf3,81799:down|Tgfbr3,21814:up|Serinc2,230779:down|Tll1,21892:down|Ceacam1,26365:up|Sfrp2,20319:up|Pamr1,210622:up|Tnfrsf11b,18383:up|Prkar2b,19088:up|Npnt,114249:up|S100a1,20193:up|Ice1,218333:down|Neurl3,214854:down|Ccl7,20306:up|Pdzrn3,55983:down|Pdgfc,54635:down|Cst7,13011:up|Epas1,13819:up|Marc1,66112:up|Sema7a,20361:down|Ddah1,69219:down|Fkbp10,14230:down|Sdpr,20324:down|Npl,74091:up|Man2a2,140481:up|Akr1c18,105349:up|Fabp5,16592:up|Ccl12,20293:up|Bmpr1a,12166:down|Klk1b11,16613:up|Tnc,21923:down|Gatm,67092:down|Ret,19713:up|Prg4,96875:down|Sdf2l1,64136:up|Pdxk,216134:up|C1s2,317677:up|Creb3l1,26427:down|Kitl,17311:up|P4ha1,18451:down|Hp,15439:up|Aoah,27052:up|Rasl11a,68895:up|Ccl3,20302:up|Plat,18791:down|Ctla2a,13024:down|Adamts12,239337:down|Lrrc15,74488:down|Anpep,16790:up|Pparg,19016:up|Lmcd1,30937:up|Glp1r,14652:down|Hmox1,15368:up|Has2,15117:down|Ptgs2,19225:down|Grem1,23892:up|Phf11d,219132:up|Col5a1,12831:down|Errfi1,74155:down
embryonic skeletal system morphogenesis	GO:0048704	8.29E-03	3.60E-02	Chst11,58250:down|Tbx15,21384:down|Col11a1,12814:down|Tgfb2,21808:down|Fgfr2,14183:up|Bmp4,12159:up
nephron development	GO:0072006	8.29E-03	3.60E-02	Ret,19713:up|Sulf1,240725:down|Vegfa,22339:down|Npnt,114249:up|Grem1,23892:up|Bmp4,12159:up|Adipoq,11450:up
positive regulation of stress-activated MAPK cascade	GO:0032874	8.29E-03	3.60E-02	Ceacam1,26365:up|Tnf,21926:up|Trf,22041:up|Wnt5a,22418:down|Vegfa,22339:down|Tgfb2,21808:down|Il1rn,16181:up
regulation of cellular response to stress	GO:0080135	8.34E-03	3.61E-02	Hp,15439:up|Sfrp4,20379:up|Ankrd1,107765:down|Cxcl12,20315:up|Vegfa,22339:down|Tnf,21926:up|Trf,22041:up|Egfr,13649:down|Hmox1,15368:up|Sfrp1,20377:up|Bmpr1a,12166:down|Wnt5a,22418:down|Il1rn,16181:up|Ceacam1,26365:up|Sfrp2,20319:up|Sdf2l1,64136:up|Tgfb2,21808:down|Creb3l1,26427:down
regulation of axonogenesis	GO:0050770	8.36E-03	3.61E-02	Plxnd1,67784:up|Wnt5a,22418:down|Sema7a,20361:down|Tnfrsf12a,27279:down|Crabp2,12904:down|Ret,19713:up|Cxcl12,20315:up|Vegfa,22339:down
lung lobe development	GO:0060462	8.49E-03	3.61E-02	Fgfr2,14183:up|Igf1,16000:up
cell-cell adhesion mediated by integrin	GO:0033631	8.49E-03	3.61E-02	Cd24a,12484:down|Npnt,114249:up
positive regulation of fever generation	GO:0031622	8.49E-03	3.61E-02	Tnf,21926:up|Ptgs2,19225:down
response to follicle-stimulating hormone	GO:0032354	8.49E-03	3.61E-02	Inhba,16323:down|Tgfbr3,21814:up
negative regulation of glial cell apoptotic process	GO:0034351	8.49E-03	3.61E-02	Gas6,14456:up|Ccl12,20293:up
Wnt signaling pathway involved in somitogenesis	GO:0090244	8.49E-03	3.61E-02	Sfrp2,20319:up|Sfrp1,20377:up
negative regulation of osteoblast proliferation	GO:0033689	8.49E-03	3.61E-02	Sfrp1,20377:up|Grem1,23892:up
negative regulation by host of viral process	GO:0044793	8.49E-03	3.61E-02	Ceacam1,26365:up|Ptx3,19288:up
negative regulation of fibroblast apoptotic process	GO:2000270	8.49E-03	3.61E-02	Gas6,14456:up|Sfrp1,20377:up
glomerular visceral epithelial cell development	GO:0072015	8.49E-03	3.61E-02	Bmp4,12159:up|Adipoq,11450:up
negative regulation of heterotypic cell-cell adhesion	GO:0034115	8.49E-03	3.61E-02	Il1rn,16181:up|Adipoq,11450:up
olfactory bulb interneuron development	GO:0021891	8.49E-03	3.61E-02	Fgfr1,14182:down|Wnt5a,22418:down
cranial suture morphogenesis	GO:0060363	8.49E-03	3.61E-02	Fgfr2,14183:up|Bmp4,12159:up
regulation of activation-induced cell death of T cells	GO:0070235	8.49E-03	3.61E-02	Ceacam1,26365:up|Tgfb2,21808:down
regulation of cell-cell adhesion involved in gastrulation	GO:0070587	8.49E-03	3.61E-02	Il1rn,16181:up|Adipoq,11450:up

gastro-intestinal system smooth muscle contraction	GO:0014831	8.49E-03	3.61E-02	Htr2b,15559:down|Sulf1,240725:down
positive regulation of macrophage cytokine production	GO:0060907	8.49E-03	3.61E-02	Wnt5a,22418:down|Sema7a,20361:down
negative regulation of granulocyte differentiation	GO:0030853	8.49E-03	3.61E-02	Ceacam1,26365:up|Adipoq,11450:up
astrocyte cell migration	GO:0043615	8.49E-03	3.61E-02	Ccl12,20293:up|Ccl3,20302:up
negative regulation of mesenchymal cell proliferation	GO:0072201	8.49E-03	3.61E-02	Wnt5a,22418:down|Bmp4,12159:up
lung lobe morphogenesis	GO:0060463	8.49E-03	3.61E-02	Fgfr2,14183:up|Igf1,16000:up
glomerular epithelial cell development	GO:0072310	8.49E-03	3.61E-02	Bmp4,12159:up|Adipoq,11450:up
positive regulation of calcineurin-NFAT signaling cascade	GO:0070886	8.49E-03	3.61E-02	Lmcd1,30937:up|Igf1,16000:up
positive regulation of chemokine biosynthetic process	GO:0045080	8.49E-03	3.61E-02	Tnf,21926:up|Wnt5a,22418:down
low-density lipoprotein particle receptor biosynthetic process	GO:0045713	8.49E-03	3.61E-02	Pparg,19016:up|Adipoq,11450:up
positive regulation of protein kinase C signaling	GO:0090037	8.49E-03	3.61E-02	Wnt5a,22418:down|Vegfa,22339:down
hormone secretion	GO:0046879	8.58E-03	3.65E-02	Tnf,21926:up|Pparg,19016:up|Glp1r,14652:down|Adm,11535:up|Fgfr1,14182:down|Egfr,13649:down|Sfrp1,20377:up|C1qtnf3,81799:down|Inhba,16323:down|Il1rn,16181:up|Adipoq,11450:up|Htr2a,15558:down
positive regulation of stress-activated protein kinase signaling cascade	GO:0070304	8.64E-03	3.67E-02	Ceacam1,26365:up|Tnf,21926:up|Trf,22041:up|Wnt5a,22418:down|Vegfa,22339:down|Tgfb2,21808:down|Il1rn,16181:up
muscle hypertrophy	GO:0014896	8.66E-03	3.67E-02	Htr2b,15559:down|Lmcd1,30937:up|Wisp1,22402:down|Errfi1,74155:down|Igf1,16000:up
lymphocyte apoptotic process	GO:0070227	8.66E-03	3.67E-02	Ceacam1,26365:up|Cd24a,12484:down|Wnt5a,22418:down|Tgfb2,21808:down|Bmp4,12159:up
negative regulation of stem cell differentiation	GO:2000737	8.70E-03	3.68E-02	Sfrp2,20319:up|Sfrp1,20377:up|Tgfb2,21808:down|Tgfbr3,21814:up
developmental programmed cell death	GO:0010623	8.70E-03	3.68E-02	Vdr,22337:down|Tgfb2,21808:down|Tgfbr3,21814:up|Kitl,17311:up
nucleocytoplasmic transport	GO:0006913	8.90E-03	3.76E-02	Tnf,21926:up|Gas6,14456:up|Akr1c18,105349:up|Egfr,13649:down|C1qtnf3,81799:down|Bmpr1a,12166:down|Ptgs2,19225:down|Grem1,23892:up|Igf1,16000:up|Bmp4,12159:up|Ceacam1,26365:up|Sfrp2,20319:up|Tgfb2,21808:down
endodermal cell differentiation	GO:0035987	8.93E-03	3.76E-02	Col5a2,12832:down|Col5a1,12831:down|Inhba,16323:down
regulation of glucan biosynthetic process	GO:0010962	8.93E-03	3.76E-02	Enpp1,18605:down|Igf2,16002:up|Igf1,16000:up
regulation of phospholipase C activity	GO:1900274	8.93E-03	3.76E-02	Htr2b,15559:down|Fgfr1,14182:down|Htr2a,15558:down
chondroitin sulfate proteoglycan metabolic process	GO:0050654	8.93E-03	3.76E-02	Mamdc2,71738:down|Chst11,58250:down|Igf1,16000:up
regulation of glycogen biosynthetic process	GO:0005979	8.93E-03	3.76E-02	Enpp1,18605:down|Igf2,16002:up|Igf1,16000:up
dopamine secretion	GO:0014046	8.93E-03	3.76E-02	Cxcl12,20315:up|Htr2a,15558:down|Sncg,20618:up
regulation of dopamine secretion	GO:0014059	8.93E-03	3.76E-02	Cxcl12,20315:up|Htr2a,15558:down|Sncg,20618:up
regulation of branching involved in ureteric bud morphogenesis	GO:0090189	8.93E-03	3.76E-02	Vegfa,22339:down|Grem1,23892:up|Bmp4,12159:up
positive regulation of blood circulation	GO:1903524	9.12E-03	3.83E-02	Glp1r,14652:down|Adm,11535:up|Egfr,13649:down|Tgfb2,21808:down|Htr2a,15558:down|Ptgs2,19225:down
negative regulation of apoptotic signaling pathway	GO:2001234	9.13E-03	3.83E-02	Tnf,21926:up|Tnfrsf23,79201:down|Hmox1,15368:up|Igf1,16000:up|Bmp4,12159:up|Sfrp2,20319:up|Cxcl12,20315:up|Cx3cr1,13051:down|Creb3l1,26427:down
intracellular receptor signaling pathway	GO:0030522	9.31E-03	3.91E-02	Pparg,19016:up|Akr1c18,105349:up|Sfrp1,20377:up|Aldh1a2,19378:up|Igf1,16000:up|Vdr,22337:down|Crabp2,12904:down|Aldh1a3,56847:down
positive regulation of lipid metabolic process	GO:0045834	9.37E-03	3.93E-02	Htr2b,15559:down|Tnf,21926:up|Pparg,19016:up|Htr2a,15558:down|Igf2,16002:up|Igf1,16000:up|Adipoq,11450:up
terpenoid metabolic process	GO:0006721	9.40E-03	3.93E-02	Crabp2,12904:down|Akr1c18,105349:up|Aldh1a2,19378:up|Aldh1a3,56847:down
cellular polysaccharide biosynthetic process	GO:0033692	9.40E-03	3.93E-02	Has2,15117:down|Enpp1,18605:down|Igf2,16002:up|Igf1,16000:up

positive regulation of pathway-restricted SMAD protein phosphorylation	GO:0010862	9.40E-03	3.93E-02	Tgfb2,21808:down|Inhba,16323:down|Bmpr1a,12166:down|Bmp4,12159:up
nuclear transport	GO:0051169	9.53E-03	3.99E-02	Tnf,21926:up|Gas6,14456:up|Akr1c18,105349:up|Egfr,13649:down|C1qtnf3,81799:down|Bmpr1a,12166:down|Ptgs2,19225:down|Grem1,23892:up|Igf1,16000:up|Bmp4,12159:up|Ceacam1,26365:up|Sfrp2,20319:up|Tgfb2,21808:down
negative regulation of cell morphogenesis involved in differentiation	GO:0010771	9.56E-03	4.00E-02	Sfrp2,20319:up|Sfrp1,20377:up|Postn,50706:down|Wnt5a,22418:down|Tgfb2,21808:down|Tgfbr3,21814:up
heart contraction	GO:0060047	9.64E-03	4.03E-02	Glp1r,14652:down|Fxyd1,56188:up|Adm,11535:up|Stc1,20855:up|Epas1,13819:up|Vegfb,22340:up|Tgfb2,21808:down|S100a1,20193:up
cell activation	GO:0001775	9.73E-03	4.05E-02	Ly9,17085:up|Ccl2,20296:up|Ctla2a,13024:down|Lyl1,17095:up|Cxcl12,20315:up|Cx3cr1,13051:down|Serpine2,20720:up|Igf2,16002:up|Gas6,14456:up|Tnf,21926:up|Egfr,13649:down|Cd24a,12484:down|Hmox1,15368:up|Sfrp1,20377:up|Wnt5a,22418:down|Cd4,12504:up|Tfrc,22042:up|Bmp4,12159:up|Igf1,16000:up|Ceacam1,26365:up|Il1rl1,17082:down|Clec4e,56619:up|Tnfsf9,21950:up
extrinsic apoptotic signaling pathway via death domain receptors	GO:0008625	9.73E-03	4.05E-02	Tnf,21926:up|Sfrp2,20319:up|Tnfrsf23,79201:down|Sfrp1,20377:up|Hmox1,15368:up
cellular hormone metabolic process	GO:0034754	9.73E-03	4.05E-02	Crabp2,12904:down|Akr1c18,105349:up|Adm,11535:up|Aldh1a2,19378:up|Aldh1a3,56847:down
hormone transport	GO:0009914	9.95E-03	4.12E-02	Tnf,21926:up|Pparg,19016:up|Glp1r,14652:down|Adm,11535:up|Fgfr1,14182:down|Egfr,13649:down|Sfrp1,20377:up|C1qtnf3,81799:down|Inhba,16323:down|Il1rn,16181:up|Adipoq,11450:up|Htr2a,15558:down
regulation of canonical Wnt signaling pathway	GO:0060828	9.98E-03	4.12E-02	Cthrc1,68588:down|Sfrp1,20377:up|Wnt5a,22418:down|Sfrp4,20379:up|Grem1,23892:up|Sfrp2,20319:up|Rspo2,239405:up|Fgfr2,14183:up
response to hypoxia	GO:0001666	9.98E-03	4.12E-02	Loxl2,94352:down|Cd24a,12484:down|Hmox1,15368:up|Plat,18791:down|Epas1,13819:up|Tgfb2,21808:down|Vegfa,22339:down|Mmp2,17390:down
leukocyte cell-cell adhesion	GO:0007159	1.00E-02	4.12E-02	Tnf,21926:up|Cd24a,12484:down|Ly9,17085:up|Has2,15117:down|Ccl2,20296:up|Cd4,12504:up|Tfrc,22042:up|Igf1,16000:up|Bmp4,12159:up|Ctla2a,13024:down|Ceacam1,26365:up|Cxcl12,20315:up|Igf2,16002:up|Clec4e,56619:up|Tnfsf9,21950:up
regulation of excretion	GO:0044062	1.00E-02	4.12E-02	Stc1,20855:up|Ednrb,13618:up|Anpep,16790:up
positive regulation of syncytium formation by plasma membrane fusion	GO:0060143	1.00E-02	4.12E-02	Ccl8,20307:up|Cxcl9,17329:up|Cxcl12,20315:up
monocyte differentiation	GO:0030224	1.00E-02	4.12E-02	Pparg,19016:up|Vegfa,22339:down|Bmp4,12159:up
positive regulation of epithelial cell apoptotic process	GO:1904037	1.00E-02	4.12E-02	Akr1c18,105349:up|Sfrp4,20379:up|Bmp4,12159:up
regulation of vascular endothelial growth factor receptor signaling pathway	GO:0030947	1.00E-02	4.12E-02	Vegfa,22339:down|Figf,14205:up|Bmp4,12159:up
negative regulation of extrinsic apoptotic signaling pathway via death domain receptors	GO:1902042	1.00E-02	4.12E-02	Sfrp2,20319:up|Tnfrsf23,79201:down|Hmox1,15368:up
regulation of inflammatory response to antigenic stimulus	GO:0002861	1.00E-02	4.12E-02	Tnf,21926:up|Cd24a,12484:down|C3,12266:up
positive regulation of acute inflammatory response	GO:0002675	1.00E-02	4.12E-02	Tnf,21926:up|C3,12266:up|Ptgs2,19225:down
isoprenoid biosynthetic process	GO:0008299	1.00E-02	4.12E-02	Akr1c18,105349:up|Aldh1a2,19378:up|Aldh1a3,56847:down
regulation of mesonephros development	GO:0061217	1.00E-02	4.12E-02	Vegfa,22339:down|Grem1,23892:up|Bmp4,12159:up
multicellular organismal catabolic process	GO:0044243	1.00E-02	4.12E-02	Mmp2,17390:down|Mmp13,17386:up|Mrc2,17534:down
positive regulation of steroid biosynthetic process	GO:0010893	1.00E-02	4.12E-02	Tnf,21926:up|Igf2,16002:up|Igf1,16000:up
positive regulation of neuroblast proliferation	GO:0002052	1.00E-02	4.12E-02	Prl2c2,18811:up|Vegfa,22339:down|Cx3cr1,13051:down
regulation of hormone biosynthetic process	GO:0046885	1.00E-02	4.12E-02	Akr1c18,105349:up|Igf2,16002:up|Igf1,16000:up
carbohydrate derivative metabolic process	GO:1901135	1.01E-02	4.14E-02	Htr2b,15559:down|Adm,11535:up|Aoah,27052:up|Ampd3,11717:up|Vegfa,22339:down|Htr2a,15558:down|Gfpt2,14584:down|Npl,74091:up|Adamts12,239337:down|Man2a2,140481:up|Mamdc2,71738:down|Ptx3,19288:up|Akr1c18,105349:up|Col11a1,12814:down|Has2,15117:down|Sulf1,240725:down|Wnt5a,22418:down|Serinc2,230779:down|Igf1,16000:up|Chst11,58250:down|Vegfb,22340:up|Hpse,15442:up|Sdf2l1,64136:up|Ptgir,19222:up|Uck2,80914:down|Enpp1,18605:down
aorta development	GO:0035904	1.01E-02	4.15E-02	Plxnd1,67784:up|Tgfb2,21808:down|Bmpr1a,12166:down|Col3a1,12825:down

positive regulation of lipase activity	GO:0060193	1.01E-02	4.15E-02	Htr2b,15559:down|Fgfr1,14182:down|Htr2a,15558:down|Fgfr2,14183:up
lung epithelium development	GO:0060428	1.01E-02	4.15E-02	Errfi1,74155:down|Fgfr2,14183:up|Igf1,16000:up|Bmp4,12159:up
positive regulation of smooth muscle cell migration	GO:0014911	1.01E-02	4.15E-02	Has2,15117:down|Postn,50706:down|F3,14066:up|Igf1,16000:up
negative regulation of fat cell differentiation	GO:0045599	1.01E-02	4.15E-02	Wnt5a,22418:down|Vegfa,22339:down|Enpp1,18605:down|Adipoq,11450:up
regulation of multicellular organismal metabolic process	GO:0044246	1.01E-02	4.15E-02	Pparg,19016:up|Ccl2,20296:up|Errfi1,74155:down|Bmp4,12159:up
protein import into nucleus, translocation	GO:0000060	1.01E-02	4.15E-02	Tnf,21926:up|Gas6,14456:up|Akr1c18,105349:up|Igf1,16000:up
regulation of heart contraction	GO:0008016	1.01E-02	4.15E-02	Glp1r,14652:down|Fxyd1,56188:up|Adm,11535:up|Tgfb2,21808:down|Stc1,20855:up|S100a1,20193:up|Epas1,13819:up
regulation of glucose transport	GO:0010827	1.03E-02	4.21E-02	Tnf,21926:up|C3,12266:up|Enpp1,18605:down|Igf1,16000:up|Adipoq,11450:up
neural tube development	GO:0021915	1.03E-02	4.22E-02	Cthrc1,68588:down|Adm,11535:up|Sfrp1,20377:up|Ptk7,71461:down|Wnt5a,22418:down|Aldh1a2,19378:up|Bmp4,12159:up|Sfrp2,20319:up
regulation of cell size	GO:0008361	1.03E-02	4.22E-02	Plxnd1,67784:up|Wnt5a,22418:down|Sema7a,20361:down|Tnfrsf12a,27279:down|Crabp2,12904:down|Ret,19713:up|Cxcl12,20315:up|Vegfa,22339:down
synapse organization	GO:0050808	1.03E-02	4.22E-02	Flrt2,399558:down|Plxnd1,67784:up|Wnt5a,22418:down|Pdzrn3,55983:down|Sncg,20618:up|Srpx2,68792:down|Tnc,21923:down|Fgfr2,14183:up|Thbs2,21826:down
glycoprotein metabolic process	GO:0009100	1.05E-02	4.28E-02	Mamdc2,71738:down|Ptx3,19288:up|Col11a1,12814:down|Sulf1,240725:down|Igf1,16000:up|Chst11,58250:down|Hpse,15442:up|Vegfb,22340:up|Sdf2l1,64136:up|Adamts12,239337:down|Man2a2,140481:up
Rap protein signal transduction	GO:0032486	1.06E-02	4.31E-02	Plk2,20620:down|Ccl9,20308:up|Ccl6,20305:up|Wnt5a,22418:down|Ccl7,20306:up|Ccl2,20296:up|Ccl12,20293:up|Ccl4,20303:up|Ccl3,20302:up|Ccl8,20307:up
heart process	GO:0003015	1.07E-02	4.33E-02	Glp1r,14652:down|Fxyd1,56188:up|Adm,11535:up|Stc1,20855:up|Epas1,13819:up|Vegfb,22340:up|Tgfb2,21808:down|S100a1,20193:up
regulation of glial cell apoptotic process	GO:0034350	1.08E-02	4.33E-02	Gas6,14456:up|Ccl12,20293:up
positive regulation of steroid hormone biosynthetic process	GO:0090031	1.08E-02	4.33E-02	Igf2,16002:up|Igf1,16000:up
forebrain neuroblast division	GO:0021873	1.08E-02	4.33E-02	Fgfr1,14182:down|Fgfr2,14183:up
mammary gland formation	GO:0060592	1.08E-02	4.33E-02	Fgfr2,14183:up|Bmp4,12159:up
vitamin D biosynthetic process	GO:0042368	1.08E-02	4.33E-02	Vdr,22337:down|Tnf,21926:up
negative regulation of macrophage derived foam cell differentiation	GO:0010745	1.08E-02	4.33E-02	Pparg,19016:up|Adipoq,11450:up
regulation of vitamin D biosynthetic process	GO:0060556	1.08E-02	4.33E-02	Vdr,22337:down|Tnf,21926:up
negative regulation of collagen metabolic process	GO:0010713	1.08E-02	4.33E-02	Pparg,19016:up|Errfi1,74155:down
negative regulation of collagen biosynthetic process	GO:0032966	1.08E-02	4.33E-02	Pparg,19016:up|Errfi1,74155:down
negative regulation of vascular permeability	GO:0043116	1.08E-02	4.33E-02	Ceacam1,26365:up|Adm,11535:up
positive regulation of cell growth involved in cardiac muscle cell development	GO:0061051	1.08E-02	4.33E-02	Wisp1,22402:down|Igf1,16000:up
blood vessel endothelial cell proliferation involved in sprouting angiogenesis	GO:0002043	1.08E-02	4.33E-02	Vegfa,22339:down|Bmp4,12159:up
cell-cell adhesion involved in gastrulation	GO:0070586	1.08E-02	4.33E-02	Il1rn,16181:up|Adipoq,11450:up
negative regulation of interleukin-6 secretion	GO:1900165	1.08E-02	4.33E-02	Gas6,14456:up|C1qtnf3,81799:down
positive regulation of catenin import into nucleus	GO:0035413	1.08E-02	4.33E-02	Sfrp2,20319:up|Egfr,13649:down
establishment of epithelial cell apical/basal polarity	GO:0045198	1.08E-02	4.33E-02	Ptk7,71461:down|Wnt5a,22418:down
positive regulation of NFAT protein import into nucleus	GO:0051533	1.08E-02	4.33E-02	Ceacam1,26365:up|Tnf,21926:up
cellular response to X-ray	GO:0071481	1.08E-02	4.33E-02	Sfrp2,20319:up|Sfrp1,20377:up

regulation of neurological system process	GO:0031644	1.09E-02	4.36E-02	Tnf,21926:up|Trf,22041:up|Fgfr1,14182:down|Ncam1,17967:down|Ednrb,13618:up
positive regulation of lipid biosynthetic process	GO:0046889	1.09E-02	4.36E-02	Htr2b,15559:down|Tnf,21926:up|Htr2a,15558:down|Igf2,16002:up|Igf1,16000:up
odontogenesis of dentin-containing tooth	GO:0042475	1.09E-02	4.36E-02	Bmpr1a,12166:down|Rspo2,239405:up|Fam20a,208659:down|Tnfrsf11b,18383:up|Bmp4,12159:up
response to nutrient	GO:0007584	1.09E-02	4.36E-02	Vdr,22337:down|Gas6,14456:up|Gatm,67092:down|Postn,50706:down
T cell apoptotic process	GO:0070231	1.09E-02	4.36E-02	Ceacam1,26365:up|Wnt5a,22418:down|Tgfb2,21808:down|Bmp4,12159:up
multi-organism process	GO:0051704	1.09E-02	4.37E-02	Adm,11535:up|Hpgd,15446:up|Mrc1,17533:up|Ccl2,20296:up|Ednrb,13618:up|Ifit3,15959:up|Ankrd1,107765:down|Clec4n,56620:up|Akr1c18,105349:up|Ptx3,19288:up|Cd24a,12484:down|Wnt5a,22418:down|Ccl12,20293:up|Ccl4,20303:up|Bmp4,12159:up|Igf1,16000:up|Cxcl9,17329:up|Oasl1,231655:up|Ifit1,15957:up|Ptgir,19222:up|Clec4e,56619:up|Kitl,17311:up|Adamts4,240913:down|Hp,15439:up|Inhba,16323:down|Slpi,20568:up|Plat,18791:down|Vdr,22337:down|Cxcl12,20315:up|Mmp2,17390:down|Serpine2,20720:up|Lrrc15,74488:down|Oas3,246727:up|Gas6,14456:up|Tnf,21926:up|Lmcd1,30937:up|Cd4,12504:up|Ptgs2,19225:down|Ceacam1,26365:up|Tnfrsf11b,18383:up
positive regulation of cellular biosynthetic process	GO:0031328	1.10E-02	4.41E-02	Fstl3,83554:down|Ice1,218333:down|Adm,11535:up|Pdgfc,54635:down|Epas1,13819:up|Ddah1,69219:down|Lyl1,17095:up|Sdpr,20324:down|Trf,22041:up|Ptx3,19288:up|Wnt5a,22418:down|Bmpr1a,12166:down|Bmp4,12159:up|Igf1,16000:up|Ret,19713:up|Plpp3,67916:up|Ptgir,19222:up|Creb3l1,26427:down|Fgfr2,14183:up|Kitl,17311:up|Prl2c2,18811:up|Htr2b,15559:down|Rasl11a,68895:up|Inhba,16323:down|Ccl3,20302:up|Vdr,22337:down|Vegfa,22339:down|Htr2a,15558:down|Igf2,16002:up|Slc40a1,53945:up|Pparg,19016:up|Tnf,21926:up|Glp1r,14652:down|Egfr,13649:down|Fgfr1,14182:down|Sfrp1,20377:up|Tgfbr3,21814:up|Ptgs2,19225:down|Grem1,23892:up|Sfrp2,20319:up|Npnt,114249:up
response to decreased oxygen levels	GO:0036293	1.11E-02	4.41E-02	Loxl2,94352:down|Cd24a,12484:down|Hmox1,15368:up|Plat,18791:down|Epas1,13819:up|Tgfb2,21808:down|Vegfa,22339:down|Mmp2,17390:down
single-organism behavior	GO:0044708	1.11E-02	4.41E-02	Plk2,20620:down|Glp1r,14652:down|Egfr,13649:down|Ncam1,17967:down|Ptgs2,19225:down|Il1rn,16181:up|Sncg,20618:up|Igf1,16000:up|Srpx2,68792:down|Cxcl12,20315:up|Htr2a,15558:down|Prkar2b,19088:up|Serpine2,20720:up|Cx3cr1,13051:down|Igf2,16002:up
replacement ossification	GO:0036075	1.12E-02	4.43E-02	Alpl,11647:up|Mmp13,17386:up|Bmp4,12159:up
regulation of fibroblast growth factor receptor signaling pathway	GO:0040036	1.12E-02	4.43E-02	Sulf1,240725:down|Wnt5a,22418:down|Fgfr2,14183:up
regulation of humoral immune response	GO:0002920	1.12E-02	4.43E-02	Tnf,21926:up|Serping1,12258:up|C3,12266:up
embryonic placenta morphogenesis	GO:0060669	1.12E-02	4.43E-02	Adm,11535:up|Gjb5,14622:down|Fgfr2,14183:up
detoxification	GO:0098754	1.12E-02	4.43E-02	Tnf,21926:up|Mt2,17750:up|Mt1,17748:up
endochondral ossification	GO:0001958	1.12E-02	4.43E-02	Alpl,11647:up|Mmp13,17386:up|Bmp4,12159:up
cardiac epithelial to mesenchymal transition	GO:0060317	1.12E-02	4.43E-02	Has2,15117:down|Tgfb2,21808:down|Tgfbr3,21814:up
negative regulation of production of molecular mediator of immune response	GO:0002701	1.12E-02	4.43E-02	Tnf,21926:up|Hmox1,15368:up|Tgfb2,21808:down
positive regulation of binding	GO:0051099	1.14E-02	4.52E-02	Cthrc1,68588:down|Plk2,20620:down|Plxnd1,67784:up|Wnt5a,22418:down|Tgfb2,21808:down|Igf1,16000:up|Bmp4,12159:up
regulation of Ras protein signal transduction	GO:0046578	1.14E-02	4.52E-02	Ccl9,20308:up|Ccl6,20305:up|Wnt5a,22418:down|Ccl7,20306:up|Ccl2,20296:up|Ccl12,20293:up|Ccl4,20303:up|Ccl3,20302:up|Col3a1,12825:down|Igf1,16000:up|Ccl8,20307:up|Tgfb2,21808:down|Kitl,17311:up
regulation of blood coagulation	GO:0030193	1.15E-02	4.54E-02	Ceacam1,26365:up|Kng1,16644:up|Serping1,12258:up|Hpse,15442:up|Serpine2,20720:up
regulation of hemostasis	GO:1900046	1.15E-02	4.54E-02	Ceacam1,26365:up|Kng1,16644:up|Serping1,12258:up|Hpse,15442:up|Serpine2,20720:up
positive regulation of adaptive immune response based on somatic recombination of immune receptors built from immunoglobulin superfamily domains	GO:0002824	1.15E-02	4.54E-02	Tnf,21926:up|Cd24a,12484:down|Cd4,12504:up|C3,12266:up|Tfrc,22042:up
regulation of transcription factor import into nucleus	GO:0042990	1.15E-02	4.54E-02	Ceacam1,26365:up|Tnf,21926:up|C1qtnf3,81799:down|Ptgs2,19225:down|Grem1,23892:up
protein autophosphorylation	GO:0046777	1.17E-02	4.61E-02	Fgfr1,14182:down|Egfr,13649:down|Pdgfc,54635:down|Grem1,23892:up|Adipoq,11450:up|Vegfa,22339:down|Errfi1,74155:down|Fgfr2,14183:up|Enpp1,18605:down
regulation of blood vessel endothelial cell migration	GO:0043535	1.17E-02	4.62E-02	Prl2c2,18811:up|Srpx2,68792:down|Vegfa,22339:down|Ptgs2,19225:down
negative regulation of leukocyte apoptotic process	GO:2000107	1.17E-02	4.62E-02	Gas6,14456:up|Cxcl12,20315:up|Kitl,17311:up|Bmp4,12159:up

<table>
<tr><td>regulation of T cell activation</td><td>GO:0050863</td><td>1.18E-02</td><td>4.65E-02</td><td>Cd24a,12484:down|Ccl2,20296:up|Cd4,12504:up|Tfrc,22042:up|Igf1,16000:up|Bmp4,12159:up|Ctla2a,13024:down|Ceacam1,26365:up|Igf2,16002:up|Tnfsf9,21950:up</td></tr>
<tr><td>regulation of ion homeostasis</td><td>GO:2000021</td><td>1.18E-02</td><td>4.66E-02</td><td>Htr2b,15559:down|Glp1r,14652:down|Cd4,12504:up|Ednrb,13618:up|Bmp4,12159:up|Htr2a,15558:down|Tgfb2,21808:down|Anpep,16790:up</td></tr>
<tr><td>regulation of binding</td><td>GO:0051098</td><td>1.19E-02</td><td>4.69E-02</td><td>Cthrc1,68588:down|Plk2,20620:down|Plxnd1,67784:up|Hmox1,15368:up|Wnt5a,22418:down|Tgfbr3,21814:up|Slpi,20568:up|Igf1,16000:up|Bmp4,12159:up|Adipoq,11450:up|Tgfb2,21808:down</td></tr>
<tr><td>glucose transport</td><td>GO:0015758</td><td>1.20E-02</td><td>4.71E-02</td><td>Tnf,21926:up|Fabp5,16592:up|C3,12266:up|Enpp1,18605:down|Igf1,16000:up|Adipoq,11450:up</td></tr>
<tr><td>cellular polysaccharide metabolic process</td><td>GO:0044264</td><td>1.21E-02</td><td>4.76E-02</td><td>Has2,15117:down|Aoah,27052:up|Enpp1,18605:down|Igf2,16002:up|Igf1,16000:up</td></tr>
<tr><td>monoamine transport</td><td>GO:0015844</td><td>1.21E-02</td><td>4.76E-02</td><td>Gpm6b,14758:up|Cxcl12,20315:up|Htr2a,15558:down|Sncg,20618:up|Maob,109731:up</td></tr>
<tr><td>pancreas development</td><td>GO:0031016</td><td>1.21E-02</td><td>4.76E-02</td><td>Wnt5a,22418:down|Aldh1a2,19378:up|Igf2,16002:up|Igf1,16000:up|Bmp4,12159:up</td></tr>
<tr><td>regulation of DNA metabolic process</td><td>GO:0051052</td><td>1.23E-02</td><td>4.83E-02</td><td>Pparg,19016:up|Fgfr1,14182:down|Egfr,13649:down|Pdgfc,54635:down|Tfrc,22042:up|Grem1,23892:up|Igf1,16000:up|Bmp4,12159:up|Adipoq,11450:up|Ankrd1,107765:down|Igfbp3,16009:up|Kitl,17311:up</td></tr>
<tr><td>negative regulation of leukocyte migration</td><td>GO:0002686</td><td>1.24E-02</td><td>4.85E-02</td><td>Cxcl12,20315:up|Ccl12,20293:up|Grem1,23892:up</td></tr>
<tr><td>heart trabecula morphogenesis</td><td>GO:0061384</td><td>1.24E-02</td><td>4.85E-02</td><td>Tgfb2,21808:down|Bmpr1a,12166:down|Tgfbr3,21814:up</td></tr>
<tr><td>modification by symbiont of host morphology or physiology</td><td>GO:0044003</td><td>1.24E-02</td><td>4.85E-02</td><td>Ceacam1,26365:up|Ptx3,19288:up|Cd4,12504:up</td></tr>
<tr><td>positive regulation of cartilage development</td><td>GO:0061036</td><td>1.24E-02</td><td>4.85E-02</td><td>Loxl2,94352:down|Wnt5a,22418:down|Bmp4,12159:up</td></tr>
<tr><td>primary metabolic process</td><td>GO:0044238</td><td>1.24E-02</td><td>4.86E-02</td><td>Fstl3,83554:down|Igfbp4,16010:up|Adm,11535:up|Flrt2,399558:down|Tfec,21426:up|Sfrp4,20379:up|Serpinh1,12406:down|Lyl1,17095:up|Gstt1,14871:up|Igfbp3,16009:up|Plk2,20620:down|Cd24a,12484:down|Mme,17380:up|Fkbp14,231997:down|Il1rn,16181:up|Maob,109731:up|Bmp4,12159:up|Hpse,15442:up|Ptgir,19222:up|Fgfr2,14183:up|Htr2b,15559:down|Ccl6,20305:up|Slpi,20568:up|Aspn,66695:down|Fam132b,227358:down|Nabp1,109019:down|Mmp2,17390:down|Oas3,246727:up|Pappa,18491:down|Slc40a1,53945:up|Egfr,13649:down|C1rb,667277:up|Tfrc,22042:up|Ccl8,20307:up|Serpina3g,20715:up|Fam20a,208659:down|Aldh1a2,19378:up|Ccl2,20296:up|Ednrb,13618:up|Ampd3,11717:up|Mmp13,17386:up|Htra3,78558:down|Uchl5,56207:up|Sord,20322:up|Enpp3,209558:down|Cdo1,12583:up|Dok2,13449:up|Mamdc2,71738:down|Ptx3,19288:up|Ccl4,20303:up|Chst11,58250:down|Vegfb,22340:up|Aldh1a3,56847:down|Plpp3,67916:up|Ccl9,20308:up|Inhba,16323:down|Bnc2,242509:down|Tfpi2,21789:down|Crlf1,12931:down|C1s1,50908:up|Vegfa,22339:down|Gfpt2,14584:down|Cbr2,12409:up|Serpine2,20720:up|Nxn,18230:down|Igf2,16002:up|Tnf,21926:up|Fgfr1,14182:down|Lpl,16956:up|Sulf1,240725:down|Cd4,12504:up|Col3a1,12825:down|Tbx15,21384:down|Serping1,12258:up|Uck2,80914:down|Enpp1,18605:down|Hpgd,15446:up|C3,12266:up|Enpp5,83965:up|Ankrd1,107765:down|Cd109,235505:down|Ggt5,23887:up|Loxl2,94352:down|Trf,22041:up|Loxl3,16950:down|Adam12,11489:down|Wnt5a,22418:down|Mark1,226778:up|Adipoq,11450:up|Hal,15109:up|Igf1,16000:up|Crabp2,12904:down|Tgfb2,21808:down|F3,14066:up|Adamts4,240913:down|Kng1,16644:up|Prl2c2,18811:up|Efemp1,216616:up|Saa3,20210:up|Gdpd1,66569:up|Vdr,22337:down|Pdia5,72599:down|Fabp4,11770:up|Htr2a,15558:down|Gas6,14456:up|Col11a1,12814:down|Sfrp1,20377:up|C1qtnf3,81799:down|Tgfbr3,21814:up|Serinc2,230779:down|Tll1,21892:down|Ceacam1,26365:up|Sfrp2,20319:up|Pamr1,210622:up|Tnfrsf11b,18383:up|Prkar2b,19088:up|Npnt,114249:up|S100a1,20193:up|Ice1,218333:down|Neurl3,214854:down|Ccl7,20306:up|Pdzrn3,55983:down|Pdgfc,54635:down|Cst7,13011:up|Epas1,13819:up|Sema7a,20361:down|Ddah1,69219:down|Fkbp10,14230:down|Sdpr,20324:down|Npl,74091:up|Man2a2,140481:up|Akr1c18,105349:up|Fabp5,16592:up|Ccl12,20293:up|Bmpr1a,12166:down|Klk1b11,16613:up|Gatm,67092:down|Ret,19713:up|Prg4,96875:down|Sdf2l1,64136:up|C1s2,317677:up|Creb3l1,26427:down|Kitl,17311:up|P4ha1,18451:down|Hp,15439:up|Aoah,27052:up|Rasl11a,68895:up|Ccl3,20302:up|Plat,18791:down|Ctla2a,13024:down|Adamts12,239337:down|Lrrc15,74488:down|Anpep,16790:up|Pparg,19016:up|Lmcd1,30937:up|Glp1r,14652:down|Hmox1,15368:up|Has2,15117:down|Ptgs2,19225:down|Grem1,23892:up|Phf11d,219132:up|Errfi1,74155:down</td></tr>
<tr><td>syncytium formation by plasma membrane fusion</td><td>GO:0000768</td><td>1.25E-02</td><td>4.90E-02</td><td>Ccl8,20307:up|Cxcl9,17329:up|Cxcl12,20315:up|Cd109,235505:down</td></tr>
<tr><td>regulation of protein binding</td><td>GO:0043393</td><td>1.26E-02</td><td>4.93E-02</td><td>Cthrc1,68588:down|Plk2,20620:down|Plxnd1,67784:up|Wnt5a,22418:down|Tgfbr3,21814:up|Slpi,20568:up|Bmp4,12159:up|Adipoq,11450:up</td></tr>
<tr><td>signal release</td><td>GO:0023061</td><td>1.27E-02</td><td>4.94E-02</td><td>Tnf,21926:up|Pparg,19016:up|Glp1r,14652:down|Adm,11535:up|Fgfr1,14182:down|Egfr,13649:down|Sfrp1,20377:up|C1qtnf3,81799:down|Inhba,16323:down|Il1rn,16181:up|Sncg,20618:up|Adipoq,11450:up|Htr2a,15558:down|Syt13,80976:up</td></tr>
<tr><td>hormone metabolic process</td><td>GO:0042445</td><td>1.27E-02</td><td>4.96E-02</td><td>Crabp2,12904:down|Akr1c18,105349:up|Adm,11535:up|Aldh1a2,19378:up|Aldh1a3,56847:down|Igf2,16002:up|Igf1,16000:up</td></tr>
<tr><td>positive regulation of protein binding</td><td>GO:0032092</td><td>1.28E-02</td><td>4.96E-02</td><td>Cthrc1,68588:down|Plk2,20620:down|Plxnd1,67784:up|Wnt5a,22418:down|Bmp4,12159:up</td></tr>
<tr><td>synaptic transmission, glutamatergic</td><td>GO:0035249</td><td>1.28E-02</td><td>4.96E-02</td><td>Egfr,13649:down|Htr2a,15558:down|Ptgs2,19225:down|Serpine2,20720:up|Plat,18791:down</td></tr>
<tr><td>response to ketone</td><td>GO:1901654</td><td>1.28E-02</td><td>4.96E-02</td><td>Gas6,14456:up|Postn,50706:down|Tgfb2,21808:down|Tgfbr3,21814:up|Bmp4,12159:up</td></tr>
<tr><td>transcription factor import into nucleus</td><td>GO:0042991</td><td>1.28E-02</td><td>4.96E-02</td><td>Ceacam1,26365:up|Tnf,21926:up|C1qtnf3,81799:down|Ptgs2,19225:down|Grem1,23892:up</td></tr>
<tr><td>ovulation cycle process</td><td>GO:0022602</td><td>1.28E-02</td><td>4.96E-02</td><td>Vegfa,22339:down|Inhba,16323:down|Mmp2,17390:down|Kitl,17311:up|Bmp4,12159:up</td></tr>
<tr><td>maintenance of location</td><td>GO:0051235</td><td>1.28E-02</td><td>4.98E-02</td><td>Kdelr3,105785:down|Htr2b,15559:down|Tnf,21926:up|Pparg,19016:up|Glp1r,14652:down|Lpl,16956:up|Cd4,12504:up|C3,12266:up|Tgfb2,21808:down|Htr2a,15558:down|Enpp1,18605:down</td></tr>
<tr><td>leukocyte activation</td><td>GO:0045321</td><td>1.29E-02</td><td>4.99E-02</td><td>Ly9,17085:up|Ccl2,20296:up|Ctla2a,13024:down|Lyl1,17095:up|Cxcl12,20315:up|Cx3cr1,13051:down|Igf2,16002:up|Gas6,14456:up|Cd24a,12484:down|Hmox1,15368:up|Sfrp1,20377:up|Wnt5a,22418:down|Cd4,12504:up|Tfrc,22042:up|Bmp4,12159:up|Igf1,16000:up|Ceacam1,26365:up|Il1rl1,17082:down|Tnfsf9,21950:up|Clec4e,56619:up</td></tr>
</table>

gonad development	GO:0008406	1.30E-02	5.06E-02	Sfrp1,20377:up|Wnt5a,22418:down|Inhba,16323:down|Bmp4,12159:up|Sfrp2,20319:up|Vegfa,22339:down|Mmp2,17390:down|Kitl,17311:up
calcium ion transport into cytosol	GO:0060402	1.31E-02	5.06E-02	Htr2b,15559:down|Glp1r,14652:down|Tgfb2,21808:down|Htr2a,15558:down|Cd4,12504:up|Bmp4,12159:up
hexose transport	GO:0008645	1.31E-02	5.06E-02	Tnf,21926:up|Fabp5,16592:up|C3,12266:up|Enpp1,18605:down|Igf1,16000:up|Adipoq,11450:up
axon development	GO:0061564	1.32E-02	5.10E-02	Flrt2,399558:down|Lama2,16773:down|Plxnd1,67784:up|Wnt5a,22418:down|Sema7a,20361:down|Tnfrsf12a,27279:down|Crabp2,12904:down|Tnc,21923:down|Ret,19713:up|Cxcl12,20315:up|Vegfa,22339:down|Tgfb2,21808:down|Mmp2,17390:down|Fgfr2,14183:up
eye development	GO:0001654	1.32E-02	5.10E-02	Col5a2,12832:down|Egfr,13649:down|Inhba,16323:down|Aldh1a2,19378:up|Col8a1,12837:down|Ninj1,18081:up|Bmp4,12159:up|Col5a1,12831:down|Vegfa,22339:down|Tgfb2,21808:down|Aldh1a3,56847:down|Fgfr2,14183:up
calcium ion import	GO:0070509	1.32E-02	5.11E-02	Htr2b,15559:down|Glp1r,14652:down|Cxcl12,20315:up|Tgfb2,21808:down|Htr2a,15558:down|Ccl12,20293:up|Stc1,20855:up
regulation of vasculogenesis	GO:2001212	1.33E-02	5.11E-02	Ceacam1,26365:up|Adm,11535:up
regulation of cytokine secretion involved in immune response	GO:0002739	1.33E-02	5.11E-02	Tnf,21926:up|Wnt5a,22418:down
induction of positive chemotaxis	GO:0050930	1.33E-02	5.11E-02	Cxcl12,20315:up|Figf,14205:up
regulation of branching involved in salivary gland morphogenesis	GO:0060693	1.33E-02	5.11E-02	Tnf,21926:up|Fgfr1,14182:down
exocrine pancreas development	GO:0031017	1.33E-02	5.11E-02	Igf2,16002:up|Igf1,16000:up
positive regulation of SMAD protein import into nucleus	GO:0060391	1.33E-02	5.11E-02	Bmpr1a,12166:down|Bmp4,12159:up
surfactant homeostasis	GO:0043129	1.33E-02	5.11E-02	Vegfa,22339:down|Epas1,13819:up
regulation of chemokine (C-X-C motif) ligand 2 production	GO:2000341	1.33E-02	5.11E-02	Tnf,21926:up|Postn,50706:down
negative regulation of multicellular organismal metabolic process	GO:0044252	1.33E-02	5.11E-02	Pparg,19016:up|Errfi1,74155:down
positive regulation of endothelial cell chemotaxis	GO:2001028	1.33E-02	5.11E-02	Fgfr1,14182:down|Vegfa,22339:down
regulation of leukocyte mediated cytotoxicity	GO:0001910	1.34E-02	5.14E-02	Ceacam1,26365:up|Clec2d,93694:up|Ccl2,20296:up|Igf2,16002:up
negative regulation of reproductive process	GO:2000242	1.34E-02	5.14E-02	Sulf1,240725:down|Wnt5a,22418:down|Igf1,16000:up|Bmp4,12159:up
leukocyte aggregation	GO:0070486	1.34E-02	5.14E-02	Cd24a,12484:down|Ly9,17085:up|Has2,15117:down|Ccl2,20296:up|Cd4,12504:up|Tfrc,22042:up|Igf1,16000:up|Bmp4,12159:up|Ctla2a,13024:down|Ceacam1,26365:up|Cxcl12,20315:up|Igf2,16002:up|Clec4e,56619:up|Tnfsf9,21950:up
response to oxygen levels	GO:0070482	1.35E-02	5.15E-02	Loxl2,94352:down|Cd24a,12484:down|Hmox1,15368:up|Plat,18791:down|Epas1,13819:up|Tgfb2,21808:down|Vegfa,22339:down|Mmp2,17390:down
regulation of peptidyl-serine phosphorylation	GO:0033135	1.36E-02	5.21E-02	Tnf,21926:up|Gas6,14456:up|Sfrp2,20319:up|Ret,19713:up|Wnt5a,22418:down|Vegfa,22339:down
placenta development	GO:0001890	1.37E-02	5.21E-02	Pparg,19016:up|Adm,11535:up|Egfr,13649:down|Gjb5,14622:down|Ptgs2,19225:down|Fgfr2,14183:up|Epas1,13819:up
phagocytosis	GO:0006909	1.37E-02	5.21E-02	Tnf,21926:up|Pparg,19016:up|Gas6,14456:up|Ptx3,19288:up|Ccl2,20296:up|C3,12266:up|Adipoq,11450:up
cartilage development involved in endochondral bone morphogenesis	GO:0060351	1.37E-02	5.21E-02	Serpinh1,12406:down|Stc1,20855:up|Mmp13,17386:up
negative regulation of protein maturation	GO:1903318	1.37E-02	5.21E-02	Ctla2a,13024:down|Serping1,12258:up|Serpine2,20720:up
regulation of glycogen metabolic process	GO:0070873	1.37E-02	5.21E-02	Enpp1,18605:down|Igf2,16002:up|Igf1,16000:up
regulation of syncytium formation by plasma membrane fusion	GO:0060142	1.37E-02	5.21E-02	Ccl8,20307:up|Cxcl9,17329:up|Cxcl12,20315:up
aorta morphogenesis	GO:0035909	1.37E-02	5.21E-02	Tgfb2,21808:down|Bmpr1a,12166:down|Col3a1,12825:down
negative regulation of DNA biosynthetic process	GO:2000279	1.37E-02	5.21E-02	Ankrd1,107765:down|Pparg,19016:up|Adipoq,11450:up
negative regulation of protein processing	GO:0010955	1.37E-02	5.21E-02	Ctla2a,13024:down|Serping1,12258:up|Serpine2,20720:up
lipid metabolic process	GO:0006629	1.39E-02	5.26E-02	Htr2b,15559:down|Adm,11535:up|Hpgd,15446:up|Aoah,27052:up|Aldh1a2,19378:up|C3,12266:up|Gdpd1,66569:up|Vdr,22337:down|Fabp4,11770:up|Fam132b,227358:down|Htr2a,15558:down|Igf2,16002:up|Pparg,19016:up|Tnf,21926:up|Ggt5,23887:up|Akr1c18,105349:up|Fgfr1,14182:down|Fabp5,16592:up|Lpl,16956:up|Ptgs2,19225:down|Il1rn,16181:up|Serinc2,230779:down|Adipoq,11450:up|Igf1,16000:up|Ceacam1,26365:up|Crabp2,12904:down|Aldh1a3,56847:down|Plpp3,67916:up|Prkar2b,19088:up

regulation of multi-organism process	GO:0043900	1.41E-02	5.37E-02	Tnf,21926:up|Lmcd1,30937:up|Ptx3,19288:up|Inhba,16323:down|Slpi,20568:up|Ednrb,13618:up|Plat,18791:down|Ccl4,20303:up|Igf1,16000:up|Ceacam1,26365:up|Oasl1,231655:up|Ifit1,15957:up|Oas3,246727:up
negative regulation of vasculature development	GO:1901343	1.42E-02	5.37E-02	Sulf1,240725:down|Ccl2,20296:up|Cx3cr1,13051:down|Thbs2,21826:down|Bmp4,12159:up
regulation of coagulation	GO:0050818	1.42E-02	5.37E-02	Ceacam1,26365:up|Kng1,16644:up|Serping1,12258:up|Hpse,15442:up|Serpine2,20720:up
columnar/cuboidal epithelial cell differentiation	GO:0002065	1.42E-02	5.37E-02	Fgfr1,14182:down|Mcoln3,171166:up|Wnt5a,22418:down|Ednrb,13618:up|Fgfr2,14183:up|Kitl,17311:up|Bmp4,12159:up
regulation of hormone secretion	GO:0046883	1.42E-02	5.37E-02	Tnf,21926:up|Pparg,19016:up|Glp1r,14652:down|Fgfr1,14182:down|Egfr,13649:down|Sfrp1,20377:up|C1qtnf3,81799:down|Inhba,16323:down|Adipoq,11450:up|Htr2a,15558:down
cytosolic calcium ion transport	GO:0060401	1.42E-02	5.37E-02	Htr2b,15559:down|Glp1r,14652:down|Tgfb2,21808:down|Htr2a,15558:down|Cd4,12504:up|Bmp4,12159:up
monosaccharide transport	GO:0015749	1.42E-02	5.37E-02	Tnf,21926:up|Fabp5,16592:up|C3,12266:up|Enpp1,18605:down|Igf1,16000:up|Adipoq,11450:up
syncytium formation	GO:0006949	1.43E-02	5.41E-02	Ccl8,20307:up|Cxcl9,17329:up|Cxcl12,20315:up|Cd109,235505:down
mammary gland morphogenesis	GO:0060443	1.43E-02	5.41E-02	Vdr,22337:down|Wnt5a,22418:down|Fgfr2,14183:up|Bmp4,12159:up
development of primary sexual characteristics	GO:0045137	1.43E-02	5.41E-02	Sfrp1,20377:up|Wnt5a,22418:down|Inhba,16323:down|Bmp4,12159:up|Sfrp2,20319:up|Vegfa,22339:down|Mmp2,17390:down|Kitl,17311:up
transport	GO:0006810	1.48E-02	5.57E-02	Adm,11535:up|Sfrp4,20379:up|C3,12266:up|Ankrd1,107765:down|Trf,22041:up|Cd24a,12484:down|Wnt5a,22418:down|Stc1,20855:up|Il1rn,16181:up|Maob,109731:up|Adipoq,11450:up|Bmp4,12159:up|Igf1,16000:up|Crabp2,12904:down|Mcoln2,68279:up|Tgfb2,21808:down|Fgfr2,14183:up|Clec4e,56619:up|Htr2b,15559:down|Mfsd12,73822:up|Vdr,22337:down|Fabp4,11770:up|Fam132b,227358:down|Cxcl12,20315:up|Htr2a,15558:down|Slc16a4,229699:down|Slc40a1,53945:up|Gas6,14456:up|Egfr,13649:down|Sec23a,20334:down|Sfrp1,20377:up|C1qtnf3,81799:down|Tfrc,22042:up|Sncg,20618:up|Fxyd2,11936:up|Serinc2,230779:down|Ceacam1,26365:up|Sfrp2,20319:up|Syt13,80976:up|S100a1,20193:up|Ice1,218333:down|Mrc1,17533:up|Ccl2,20296:up|Ednrb,13618:up|Mmp13,17386:up|Slc29a1,63959:up|Gpm6b,14758:up|Clec4n,56620:up|Steap1,70358:down|Postn,50706:down|Cdo1,12583:up|Ptx3,19288:up|Hvcn1,74096:up|Akr1c18,105349:up|Fabp5,16592:up|Ncam1,17967:down|Ccl12,20293:up|Bmpr1a,12166:down|Ccl4,20303:up|Ank,11732:down|Creb3l1,26427:down|Mrc2,17534:down|Il1rl1,17082:down|Inhba,16323:down|Ccl3,20302:up|Vegfa,22339:down|Serpine2,20720:up|Anpep,16790:up|Kdelr3,105785:down|Pparg,19016:up|Tnf,21926:up|Fxyd1,56188:up|Glp1r,14652:down|Fgfr1,14182:down|Lpl,16956:up|Hmox1,15368:up|Cd4,12504:up|Ptgs2,19225:down|Grem1,23892:up|Kcnj15,16516:down|Mcoln3,171166:up|Emb,13723:up|Enpp1,18605:down
negative regulation of inflammatory response	GO:0050728	1.48E-02	5.58E-02	Ctla2a,13024:down|Pparg,19016:up|C1qtnf3,81799:down|Aoah,27052:up|Cx3cr1,13051:down|Adipoq,11450:up
regulation of adaptive immune response based on somatic recombination of immune receptors built from immunoglobulin superfamily domains	GO:0002822	1.48E-02	5.58E-02	Tnf,21926:up|Cd24a,12484:down|Cd4,12504:up|C3,12266:up|Tfrc,22042:up|Il1rl1,17082:down
regulation of amine transport	GO:0051952	1.49E-02	5.60E-02	Tnf,21926:up|Cxcl12,20315:up|Htr2a,15558:down|Il1rn,16181:up|Sncg,20618:up
regulation of cell-matrix adhesion	GO:0001952	1.49E-02	5.60E-02	Gpm6b,14758:up|Postn,50706:down|Vegfa,22339:down|Grem1,23892:up|Ninj1,18081:up
regulation of small GTPase mediated signal transduction	GO:0051056	1.51E-02	5.65E-02	Ccl9,20308:up|Ccl6,20305:up|Wnt5a,22418:down|Ccl7,20306:up|Ccl2,20296:up|Ccl12,20293:up|Ccl4,20303:up|Ccl3,20302:up|Col3a1,12825:down|Igf1,16000:up|Ccl8,20307:up|Tgfb2,21808:down|Kitl,17311:up
regulation of keratinocyte proliferation	GO:0010837	1.51E-02	5.65E-02	Vdr,22337:down|Has2,15117:down|Cd109,235505:down
protein kinase C signaling	GO:0070528	1.51E-02	5.65E-02	Htr2b,15559:down|Wnt5a,22418:down|Vegfa,22339:down
negative regulation of lymphocyte mediated immunity	GO:0002707	1.51E-02	5.65E-02	Ceacam1,26365:up|Clec2d,93694:up|Igf2,16002:up
apoptotic process involved in morphogenesis	GO:0060561	1.51E-02	5.65E-02	Vdr,22337:down|Tgfb2,21808:down|Tgfbr3,21814:up
single-organism carbohydrate metabolic process	GO:0044723	1.55E-02	5.79E-02	Igfbp4,16010:up|Aoah,27052:up|Sord,20322:up|Htr2a,15558:down|Igfbp3,16009:up|Igf2,16002:up|Man2a2,140481:up|Tnf,21926:up|Fabp5,16592:up|Has2,15117:down|C1qtnf3,81799:down|Adipoq,11450:up|Igf1,16000:up|Chst11,58250:down|Vegfb,22340:up|Sdf2l1,64136:up|Enpp1,18605:down
positive regulation of lymphocyte activation	GO:0051251	1.56E-02	5.82E-02	Gas6,14456:up|Cd24a,12484:down|Ccl2,20296:up|Cd4,12504:up|Tfrc,22042:up|Igf1,16000:up|Ceacam1,26365:up|Igf2,16002:up|Tnfsf9,21950:up
endothelial cell differentiation	GO:0045446	1.56E-02	5.84E-02	Ceacam1,26365:up|Tnf,21926:up|Vegfa,22339:down|Stc1,20855:up|Bmp4,12159:up
positive regulation of adaptive immune response	GO:0002821	1.56E-02	5.84E-02	Tnf,21926:up|Cd24a,12484:down|Cd4,12504:up|C3,12266:up|Tfrc,22042:up

positive regulation of nucleobase-containing compound metabolic process	GO:0045935	1.59E-02	5.84E-02	Fstl3,83554:down|Ice1,218333:down|Adm,11535:up|Pdgfc,54635:down|Epas1,13819:up|Lyl1,17095:up|Sdpr,20324:down|Igfbp3,16009:up|Trf,22041:up|Wnt5a,22418:down|Bmpr1a,12166:down|Bmp4,12159:up|Igf1,16000:up|Ret,19713:up|Plpp3,67916:up|Ptgir,19222:up|Creb3l1,26427:down|Fgfr2,14183:up|Kitl,17311:up|Prl2c2,18811:up|Rasl11a,68895:up|Inhba,16323:down|Ccl3,20302:up|Vdr,22337:down|Vegfa,22339:down|Htr2a,15558:down|Igf2,16002:up|Slc40a1,53945:up|Pparg,19016:up|Tnf,21926:up|Glp1r,14652:down|Egfr,13649:down|Fgfr1,14182:down|Sfrp1,20377:up|Tgfbr3,21814:up|Tfrc,22042:up|Grem1,23892:up|Sfrp2,20319:up|Npnt,114249:up
regulation of vesicle-mediated transport	GO:0060627	1.60E-02	5.84E-02	Tnf,21926:up|Pparg,19016:up|Gas6,14456:up|Trf,22041:up|Ptx3,19288:up|Ncam1,17967:down|Hmox1,15368:up|Wnt5a,22418:down|Sfrp4,20379:up|Ccl2,20296:up|C3,12266:up|Grem1,23892:up|Adipoq,11450:up|Vegfa,22339:down
regulation of tumor necrosis factor production	GO:0032680	1.61E-02	5.84E-02	Gas6,14456:up|Ccl2,20296:up|Errfi1,74155:down|Ccl4,20303:up|Ccl3,20302:up|Adipoq,11450:up
axon extension	GO:0048675	1.61E-02	5.84E-02	Tnfrsf12a,27279:down|Plxnd1,67784:up|Cxcl12,20315:up|Wnt5a,22418:down|Vegfa,22339:down|Sema7a,20361:down
regulation of chronic inflammatory response	GO:0002676	1.61E-02	5.84E-02	Tnf,21926:up|Cx3cr1,13051:down
negative regulation of JUN kinase activity	GO:0043508	1.61E-02	5.84E-02	Sfrp2,20319:up|Sfrp1,20377:up
nucleoside triphosphate catabolic process	GO:0009143	1.61E-02	5.84E-02	Enpp1,18605:down|Enpp3,209558:down
olfactory bulb interneuron differentiation	GO:0021889	1.61E-02	5.84E-02	Fgfr1,14182:down|Wnt5a,22418:down
positive regulation of DNA-dependent DNA replication	GO:2000105	1.61E-02	5.84E-02	Fgfr1,14182:down|Bmp4,12159:up
regulation of type B pancreatic cell proliferation	GO:0061469	1.61E-02	5.84E-02	Sfrp1,20377:up|Errfi1,74155:down
negative regulation of tumor necrosis factor-mediated signaling pathway	GO:0010804	1.61E-02	5.84E-02	Gas6,14456:up|Adipoq,11450:up
pulmonary valve morphogenesis	GO:0003184	1.61E-02	5.84E-02	Tgfb2,21808:down|Bmp4,12159:up
response to lithium ion	GO:0010226	1.61E-02	5.84E-02	Pparg,19016:up|Fabp4,11770:up
positive regulation of humoral immune response	GO:0002922	1.61E-02	5.84E-02	Tnf,21926:up|C3,12266:up
cellular response to lithium ion	GO:0071285	1.61E-02	5.84E-02	Pparg,19016:up|Fabp4,11770:up
regulation of chemokine biosynthetic process	GO:0045073	1.61E-02	5.84E-02	Tnf,21926:up|Wnt5a,22418:down
polarized epithelial cell differentiation	GO:0030859	1.61E-02	5.84E-02	Ptk7,71461:down|Wnt5a,22418:down
negative regulation of vasoconstriction	GO:0045906	1.61E-02	5.84E-02	Adm,11535:up|Mmp2,17390:down
ferric iron transport	GO:0015682	1.61E-02	5.84E-02	Trf,22041:up|Tfrc,22042:up
regulation of transcription from RNA polymerase II promoter in response to oxidative stress	GO:0043619	1.61E-02	5.84E-02	Hmox1,15368:up|Epas1,13819:up
negative regulation of gluconeogenesis	GO:0045721	1.61E-02	5.84E-02	C1qtnf3,81799:down|Adipoq,11450:up
anatomical structure regression	GO:0060033	1.61E-02	5.84E-02	Bmpr1a,12166:down|Ninj1,18081:up
chemokine biosynthetic process	GO:0042033	1.61E-02	5.84E-02	Tnf,21926:up|Wnt5a,22418:down
chemokine (C-X-C motif) ligand 2 production	GO:0072567	1.61E-02	5.84E-02	Tnf,21926:up|Postn,50706:down
negative regulation of platelet aggregation	GO:0090331	1.61E-02	5.84E-02	Ceacam1,26365:up|Serpine2,20720:up
regulation of cAMP-dependent protein kinase activity	GO:2000479	1.61E-02	5.84E-02	Prkar2b,19088:up|Adipoq,11450:up
regulation of sequestering of triglyceride	GO:0010889	1.61E-02	5.84E-02	Pparg,19016:up|Lpl,16956:up
trivalent inorganic anion homeostasis	GO:0072506	1.61E-02	5.84E-02	Sfrp4,20379:up|Enpp1,18605:down
pulmonary valve development	GO:0003177	1.61E-02	5.84E-02	Tgfb2,21808:down|Bmp4,12159:up

<table>
<tr><td>positive regulation of heat generation</td><td>GO:0031652</td><td>1.61E-02</td><td>5.84E-02</td><td>Tnf,21926:up|Ptgs2,19225:down</td></tr>
<tr><td>adhesion of symbiont to host</td><td>GO:0044406</td><td>1.61E-02</td><td>5.84E-02</td><td>Gas6,14456:up|Lrrc15,74488:down</td></tr>
<tr><td>phosphate ion homeostasis</td><td>GO:0055062</td><td>1.61E-02</td><td>5.84E-02</td><td>Sfrp4,20379:up|Enpp1,18605:down</td></tr>
<tr><td>modulation by virus of host process</td><td>GO:0019054</td><td>1.61E-02</td><td>5.84E-02</td><td>Ceacam1,26365:up|Cd4,12504:up</td></tr>
<tr><td>regulation of cholesterol storage</td><td>GO:0010885</td><td>1.61E-02</td><td>5.84E-02</td><td>Pparg,19016:up|Lpl,16956:up</td></tr>
<tr><td>Peyer's patch development</td><td>GO:0048541</td><td>1.61E-02</td><td>5.84E-02</td><td>Ceacam1,26365:up|Ret,19713:up</td></tr>
<tr><td>mesenchymal to epithelial transition involved in metanephros morphogenesis</td><td>GO:0003337</td><td>1.61E-02</td><td>5.84E-02</td><td>Grem1,23892:up|Bmp4,12159:up</td></tr>
<tr><td>regulation of complement activation</td><td>GO:0030449</td><td>1.61E-02</td><td>5.84E-02</td><td>Serping1,12258:up|C3,12266:up</td></tr>
<tr><td>positive regulation of myeloid leukocyte cytokine production involved in immune response</td><td>GO:0061081</td><td>1.61E-02</td><td>5.84E-02</td><td>Wnt5a,22418:down|Sema7a,20361:down</td></tr>
<tr><td>trivalent inorganic cation transport</td><td>GO:0072512</td><td>1.61E-02</td><td>5.84E-02</td><td>Trf,22041:up|Tfrc,22042:up</td></tr>
<tr><td>regulation of synaptic transmission, glutamatergic</td><td>GO:0051966</td><td>1.63E-02</td><td>5.84E-02</td><td>Egfr,13649:down|Htr2a,15558:down|Ptgs2,19225:down|Serpine2,20720:up</td></tr>
<tr><td>glomerulus development</td><td>GO:0032835</td><td>1.63E-02</td><td>5.84E-02</td><td>Ret,19713:up|Sulf1,240725:down|Bmp4,12159:up|Adipoq,11450:up</td></tr>
<tr><td>positive regulation of extrinsic apoptotic signaling pathway</td><td>GO:2001238</td><td>1.63E-02</td><td>5.84E-02</td><td>Tnfrsf12a,27279:down|Sfrp1,20377:up|Tgfb2,21808:down|Inhba,16323:down</td></tr>
<tr><td>axis specification</td><td>GO:0009798</td><td>1.64E-02</td><td>5.84E-02</td><td>Sfrp1,20377:up|Wnt5a,22418:down|Bmpr1a,12166:down|Stc1,20855:up|Bmp4,12159:up</td></tr>
<tr><td>response to nicotine</td><td>GO:0035094</td><td>1.65E-02</td><td>5.84E-02</td><td>Tnf,21926:up|Hmox1,15368:up|Igf2,16002:up</td></tr>
<tr><td>interleukin-1 secretion</td><td>GO:0050701</td><td>1.65E-02</td><td>5.84E-02</td><td>Gas6,14456:up|Wnt5a,22418:down|Ccl3,20302:up</td></tr>
<tr><td>apoptotic cell clearance</td><td>GO:0043277</td><td>1.65E-02</td><td>5.84E-02</td><td>Gas6,14456:up|Ccl2,20296:up|C3,12266:up</td></tr>
<tr><td>regulation of cell adhesion mediated by integrin</td><td>GO:0033628</td><td>1.65E-02</td><td>5.84E-02</td><td>Cd24a,12484:down|Ret,19713:up|Tgfb2,21808:down</td></tr>
<tr><td>lactation</td><td>GO:0007595</td><td>1.65E-02</td><td>5.84E-02</td><td>Vdr,22337:down|Vegfa,22339:down|Cdo1,12583:up</td></tr>
<tr><td>tumor necrosis factor production</td><td>GO:0032640</td><td>1.67E-02</td><td>5.84E-02</td><td>Gas6,14456:up|Ccl2,20296:up|Errfi1,74155:down|Ccl4,20303:up|Ccl3,20302:up|Adipoq,11450:up</td></tr>
<tr><td>reactive oxygen species metabolic process</td><td>GO:0072593</td><td>1.69E-02</td><td>5.84E-02</td><td>Tnf,21926:up|Akr1c18,105349:up|Ptx3,19288:up|Egfr,13649:down|Hp,15439:up|Ptgs2,19225:down|Igf1,16000:up|Pxdn,69675:down|Ddah1,69219:down</td></tr>
<tr><td>neuron projection extension</td><td>GO:1990138</td><td>1.69E-02</td><td>5.84E-02</td><td>Tnfrsf12a,27279:down|Plxnd1,67784:up|Cxcl12,20315:up|Postn,50706:down|Wnt5a,22418:down|Vegfa,22339:down|Sema7a,20361:down</td></tr>
<tr><td>regulation of lymphocyte activation</td><td>GO:0051249</td><td>1.72E-02</td><td>5.84E-02</td><td>Gas6,14456:up|Cd24a,12484:down|Sfrp1,20377:up|Ccl2,20296:up|Cd4,12504:up|Tfrc,22042:up|Igf1,16000:up|Bmp4,12159:up|Ctla2a,13024:down|Ceacam1,26365:up|Igf2,16002:up|Tnfsf9,21950:up</td></tr>
<tr><td>regulation of innate immune response</td><td>GO:0045088</td><td>1.72E-02</td><td>5.84E-02</td><td>Tnf,21926:up|Pparg,19016:up|Clec2d,93694:up|Wnt5a,22418:down|Ceacam1,26365:up|Serping1,12258:up|Mmp2,17390:down|Igf2,16002:up</td></tr>
<tr><td>positive regulation of reproductive process</td><td>GO:2000243</td><td>1.73E-02</td><td>5.84E-02</td><td>Wnt5a,22418:down|Inhba,16323:down|Ednrb,13618:up|Plat,18791:down</td></tr>
<tr><td>establishment of localization</td><td>GO:0051234</td><td>1.74E-02</td><td>5.84E-02</td><td>Adm,11535:up|Sfrp4,20379:up|C3,12266:up|Ankrd1,107765:down|Trf,22041:up|Cd24a,12484:down|Wnt5a,22418:down|Stc1,20855:up|Il1rn,16181:up|Maob,109731:up|Adipoq,11450:up|Bmp4,12159:up|Igf1,16000:up|Crabp2,12904:down|Mcoln2,68279:up|Tgfb2,21808:down|Fgfr2,14183:up|Clec4e,56619:up|Htr2b,15559:down|Mfsd12,73822:up|Vdr,22337:down|Fabp4,11770:up|Fam132b,227358:down|Cxcl12,20315:up|Htr2a,15558:down|Slc16a4,229699:down|Slc40a1,53945:up|Gas6,14456:up|Egfr,13649:down|Sec23a,20334:down|Sfrp1,20377:up|C1qtnf3,81799:down|Tfrc,22042:up|Sncg,20618:up|Fxyd2,11936:up|Serinc2,230779:down|Ceacam1,26365:up|Sfrp2,20319:up|Npnt,114249:up|Syt13,80976:up|S100a1,20193:up|Ice1,218333:down|Mrc1,17533:up|Ccl2,20296:up|Ednrb,13618:up|Mmp13,17386:up|Slc29a1,63959:up|Gpm6b,14758:up|Clec4n,56620:up|Steap1,70358:down|Postn,50706:down|Cdo1,12583:up|Ptx3,19288:up|Hvcn1,74096:up|Akr1c18,105349:up|Fabp5,16592:up|Ncam1,17967:down|Ccl12,20293:up|Bmpr1a,12166:down|Ccl4,20303:up|Ank,11732:down|Creb3l1,26427:down|Mrc2,17534:down|Il1rl1,17082:down|Inhba,16323:down|Ccl3,20302:up|Vegfa,22339:down|Serpine2,20720:up|Lrrc15,74488:down|Anpep,16790:up|Kdelr3,105785:down|Pparg,19016:up|Tnf,21926:up|Fxyd1,56188:up|Glp1r,14652:down|Fgfr1,14182:down|Lpl,16956:up|Hmox1,15368:up|Cd4,12504:up|Ptgs2,19225:down|Grem1,23892:up|Kcnj15,16516:down|Mcoln3,171166:up|Emb,13723:up|Enpp1,18605:down</td></tr>
<tr><td>regulation of tumor necrosis factor superfamily cytokine production</td><td>GO:1903555</td><td>1.74E-02</td><td>5.84E-02</td><td>Gas6,14456:up|Ccl2,20296:up|Errfi1,74155:down|Ccl4,20303:up|Ccl3,20302:up|Adipoq,11450:up</td></tr>
</table>

<table>
<tr><td>sulfur compound metabolic process</td><td>GO:0006790</td><td>1.74E-02</td><td>5.84E-02</td><td>Mamdc2,71738:down|Ggt5,23887:up|Sulf1,240725:down|Igf1,16000:up|Chst11,58250:down|Gstm1,14862:up|Gstt1,14871:up|Hpse,15442:up|Enpp1,18605:down|Cdo1,12583:up</td></tr>
<tr><td>metabolic process</td><td>GO:0008152</td><td>1.75E-02</td><td>5.84E-02</td><td>Fstl3,83554:down|Igfbp4,16010:up|Adm,11535:up|Flrt2,399558:down|Tfec,21426:up|Sfrp4,20379:up|Serpinh1,12406:down|Lyl1,17095:up|Gstt1,14871:up|Blvrb,233016:up|Igfbp3,16009:up|Plk2,20620:down|Cd24a,12484:down|Mme,17380:up|Fkbp14,231997:down|Il1rn,16181:up|Maob,109731:up|Bmp4,12159:up|Hpse,15442:up|Ptgir,19222:up|Fgfr2,14183:up|Htr2b,15559:down|Dram1,71712:up|Ccl6,20305:up|Slpi,20568:up|Aspn,66695:down|Mt2,17750:up|Fam132b,227358:down|Nabp1,109019:down|Mmp2,17390:down|Oas3,246727:up|Pappa,18491:down|Slc40a1,53945:up|Egfr,13649:down|Alpl,11647:up|C1rb,667277:up|Tfrc,22042:up|Ccl8,20307:up|Gstm1,14862:up|Serpina3g,20715:up|Fam20a,208659:down|Mt1,17748:up|Aldh1a2,19378:up|Ccl2,20296:up|Ednrb,13618:up|Ampd3,11717:up|Mmp13,17386:up|Htra3,78558:down|Uchl5,56207:up|Sord,20322:up|Steap1,70358:down|Enpp3,209558:down|Cdo1,12583:up|Dok2,13449:up|Mamdc2,71738:down|Ptx3,19288:up|Ccl4,20303:up|Chst11,58250:down|Vegfb,22340:up|Aldh1a3,56847:down|Plpp3,67916:up|Mrc2,17534:down|Ccl9,20308:up|Inhba,16323:down|Gsta4,14860:up|Bnc2,242509:down|Tfpi2,21789:down|Crlf1,12931:down|C1s1,50908:up|Plod2,26432:down|Vegfa,22339:down|Gfpt2,14584:down|Serpine2,20720:up|Cbr2,12409:up|Nxn,18230:down|Igf2,16002:up|Tnf,21926:up|Fgfr1,14182:down|Lpl,16956:up|Sulf1,240725:down|Cd4,12504:up|Col3a1,12825:down|Tbx15,21384:down|Serping1,12258:up|Uck2,80914:down|Enpp1,18605:down|Hpgd,15446:up|C3,12266:up|Enpp5,83965:up|Ankrd1,107765:down|Cd109,235505:down|Ggt5,23887:up|Loxl2,94352:down|Trf,22041:up|Loxl3,16950:down|Adam12,11489:down|Wnt5a,22418:down|Mark1,226778:up|Adipoq,11450:up|Hal,15109:up|Igf1,16000:up|Crabp2,12904:down|Tgfb2,21808:down|F3,14066:up|Adamts4,240913:down|Kng1,16644:up|Prl2c2,18811:up|Efemp1,216616:up|Saa3,20210:up|Gdpd1,66569:up|Vdr,22337:down|Pdia5,72599:down|Fabp4,11770:up|Htr2a,15558:down|Gas6,14456:up|Col11a1,12814:down|Sfrp1,20377:up|C1qtnf3,81799:down|Tgfbr3,21814:up|Serinc2,230779:down|Tll1,21892:down|Ceacam1,26365:up|Sfrp2,20319:up|Pxdn,69675:down|Pamr1,210622:up|Tnfrsf11b,18383:up|Prkar2b,19088:up|Npnt,114249:up|S100a1,20193:up|Ice1,218333:down|Neurl3,214854:down|Ccl7,20306:up|Pdzrn3,55983:down|Pdgfc,54635:down|Cst7,13011:up|Sema7a,20361:down|Epas1,13819:up|Marc1,66112:up|Ddah1,69219:down|Sdpr,20324:down|Fkbp10,14230:down|Npl,74091:up|Man2a2,140481:up|Akr1c18,105349:up|Fabp5,16592:up|Ccl12,20293:up|Bmpr1a,12166:down|Klk1b11,16613:up|Tnc,21923:down|Gatm,67092:down|Ret,19713:up|Prg4,96875:down|Sdf2l1,64136:up|Pdxk,216134:up|C1s2,317677:up|Creb3l1,26427:down|Kitl,17311:up|P4ha1,18451:down|Hp,15439:up|Aoah,27052:up|Rasl11a,68895:up|Plat,18791:down|Ccl3,20302:up|Ctla2a,13024:down|Srpx2,68792:down|Adamts12,239337:down|Lrrc15,74488:down|Anpep,16790:up|Pparg,19016:up|Lmcd1,30937:up|Glp1r,14652:down|Hmox1,15368:up|Has2,15117:down|Ptgs2,19225:down|Grem1,23892:up|Phf11d,219132:up|Col5a1,12831:down|Errfi1,74155:down</td></tr>
<tr><td>palate development</td><td>GO:0060021</td><td>1.80E-02</td><td>5.84E-02</td><td>Wnt5a,22418:down|Inhba,16323:down|Bmpr1a,12166:down|Tgfbr3,21814:up|Bnc2,242509:down</td></tr>
<tr><td>amine transport</td><td>GO:0015837</td><td>1.80E-02</td><td>5.84E-02</td><td>Tnf,21926:up|Cxcl12,20315:up|Htr2a,15558:down|Il1rn,16181:up|Sncg,20618:up</td></tr>
<tr><td>spleen trabecula formation</td><td>GO:0060345</td><td>1.81E-02</td><td>5.84E-02</td><td>Slc40a1,53945:up</td></tr>
<tr><td>CMP metabolic process</td><td>GO:0046035</td><td>1.81E-02</td><td>5.84E-02</td><td>Uck2,80914:down</td></tr>
<tr><td>negative regulation of metanephric comma-shaped body morphogenesis</td><td>GO:2000007</td><td>1.81E-02</td><td>5.84E-02</td><td>Bmp4,12159:up</td></tr>
<tr><td>positive regulation of activation of membrane attack complex</td><td>GO:0001970</td><td>1.81E-02</td><td>5.84E-02</td><td>C3,12266:up</td></tr>
<tr><td>regulation of protein kinase D signaling</td><td>GO:1903570</td><td>1.81E-02</td><td>5.84E-02</td><td>Vegfa,22339:down</td></tr>
<tr><td>pentitol metabolic process</td><td>GO:0019519</td><td>1.81E-02</td><td>5.84E-02</td><td>Sord,20322:up</td></tr>
<tr><td>negative regulation of branching involved in lung morphogenesis</td><td>GO:0061048</td><td>1.81E-02</td><td>5.84E-02</td><td>Tnf,21926:up</td></tr>
<tr><td>bud dilation involved in lung branching</td><td>GO:0060503</td><td>1.81E-02</td><td>5.84E-02</td><td>Bmp4,12159:up</td></tr>
<tr><td>mesodermal cell fate determination</td><td>GO:0007500</td><td>1.81E-02</td><td>5.84E-02</td><td>Bmp4,12159:up</td></tr>
<tr><td>canonical Wnt signaling pathway involved in positive regulation of endothelial cell migration</td><td>GO:0044328</td><td>1.81E-02</td><td>5.84E-02</td><td>Plpp3,67916:up</td></tr>
<tr><td>intermediate mesoderm formation</td><td>GO:0048391</td><td>1.81E-02</td><td>5.84E-02</td><td>Bmp4,12159:up</td></tr>
<tr><td>dichloromethane metabolic process</td><td>GO:0018900</td><td>1.81E-02</td><td>5.84E-02</td><td>Gstt1,14871:up</td></tr>
<tr><td>L-xylitol catabolic process</td><td>GO:0051160</td><td>1.81E-02</td><td>5.84E-02</td><td>Sord,20322:up</td></tr>
<tr><td>positive regulation of T cell homeostatic proliferation</td><td>GO:0042103</td><td>1.81E-02</td><td>5.84E-02</td><td>Cd24a,12484:down</td></tr>
<tr><td>hematopoietic stem cell migration to bone marrow</td><td>GO:0097241</td><td>1.81E-02</td><td>5.84E-02</td><td>Gas6,14456:up</td></tr>
<tr><td>paraxial mesoderm structural organization</td><td>GO:0048352</td><td>1.81E-02</td><td>5.84E-02</td><td>Bmpr1a,12166:down</td></tr>
</table>

regulation of renal albumin absorption	GO:2000532	1.81E-02	5.84E-02	Adipoq,11450:up
negative regulation of hepatocyte growth factor receptor signaling pathway	GO:1902203	1.81E-02	5.84E-02	Adamts12,239337:down
fibroblast growth factor receptor signaling pathway involved in positive regulation of cell proliferation in bone marrow	GO:0035604	1.81E-02	5.84E-02	Fgfr2,14183:up
positive regulation of chemokine (C-C motif) ligand 2 secretion	GO:1904209	1.81E-02	5.84E-02	Postn,50706:down
negative regulation of hydrogen peroxide catabolic process	GO:2000296	1.81E-02	5.84E-02	Hp,15439:up
regulation of oligodendrocyte apoptotic process	GO:1900141	1.81E-02	5.84E-02	Gas6,14456:up
renal system segmentation	GO:0061150	1.81E-02	5.84E-02	Bmp4,12159:up
convergent extension involved in somitogenesis	GO:0090246	1.81E-02	5.84E-02	Sfrp1,20377:up
chemokine receptor transport within lipid bilayer	GO:0033606	1.81E-02	5.84E-02	Cd24a,12484:down
motogenic signaling involved in postnatal olfactory bulb interneuron migration	GO:0021837	1.81E-02	5.84E-02	Fgfr1,14182:down
negative regulation of sodium-dependent phosphate transport	GO:2000119	1.81E-02	5.84E-02	Sfrp4,20379:up
negative regulation of chronic inflammatory response to non-antigenic stimulus	GO:0002881	1.81E-02	5.84E-02	Cx3cr1,13051:down
intermediate mesodermal cell differentiation	GO:0048392	1.81E-02	5.84E-02	Bmp4,12159:up
negative regulation of canonical Wnt signaling pathway involved in controlling type B pancreatic cell proliferation	GO:2000080	1.81E-02	5.84E-02	Sfrp1,20377:up
tooth eruption	GO:0044691	1.81E-02	5.84E-02	Fam20a,208659:down
BMP signaling pathway involved in nephric duct formation	GO:0071893	1.81E-02	5.84E-02	Bmp4,12159:up
cellular response to chromate	GO:0071247	1.81E-02	5.84E-02	Mt1,17748:up
pyridoxal 5'-phosphate salvage	GO:0009443	1.81E-02	5.84E-02	Pdxk,216134:up
pentitol catabolic process	GO:0019527	1.81E-02	5.84E-02	Sord,20322:up
regulation of erythrocyte clearance	GO:0034106	1.81E-02	5.84E-02	Cd24a,12484:down
regulation of renal phosphate excretion	GO:1903402	1.81E-02	5.84E-02	Stc1,20855:up
L-cysteine catabolic process to pyruvate	GO:0019450	1.81E-02	5.84E-02	Cdo1,12583:up
specification of ureteric bud anterior/posterior symmetry by BMP signaling pathway	GO:0072101	1.81E-02	5.84E-02	Bmp4,12159:up
induction by virus of host cell-cell fusion	GO:0006948	1.81E-02	5.84E-02	Cd4,12504:up
pancreatic trypsinogen secretion	GO:1990747	1.81E-02	5.84E-02	Mmp13,17386:up
pulmonary artery endothelial tube morphogenesis	GO:0061155	1.81E-02	5.84E-02	Bmp4,12159:up

negative regulation of metanephric mesenchymal cell migration	GO:2000590	1.81E-02	5.84E-02	Adipoq,11450:up
iron cation export	GO:1903414	1.81E-02	5.84E-02	Slc40a1,53945:up
enteric smooth muscle cell differentiation	GO:0035645	1.81E-02	5.84E-02	Ednrb,13618:up
CMP salvage	GO:0006238	1.81E-02	5.84E-02	Uck2,80914:down
renal phosphate excretion	GO:0044722	1.81E-02	5.84E-02	Stc1,20855:up
ossification involved in bone remodeling	GO:0043932	1.81E-02	5.84E-02	Cthrc1,68588:down
negative regulation of oligodendrocyte apoptotic process	GO:1900142	1.81E-02	5.84E-02	Gas6,14456:up
sorbitol catabolic process	GO:0006062	1.81E-02	5.84E-02	Sord,20322:up
protein deamination	GO:0018277	1.81E-02	5.84E-02	Loxl2,94352:down
negative regulation of erythrocyte aggregation	GO:0034119	1.81E-02	5.84E-02	Cd24a,12484:down
lipopolysaccharide metabolic process	GO:0008653	1.81E-02	5.84E-02	Aoah,27052:up
negative regulation of retinoic acid biosynthetic process	GO:1900053	1.81E-02	5.84E-02	Akr1c18,105349:up
BMP signaling pathway involved in ureter morphogenesis	GO:0061149	1.81E-02	5.84E-02	Bmp4,12159:up
nucleus accumbens development	GO:0021768	1.81E-02	5.84E-02	Aldh1a3,56847:down
L-cysteine catabolic process to pyruvate, using cysteine dioxygenase	GO:0019451	1.81E-02	5.84E-02	Cdo1,12583:up
coronal suture morphogenesis	GO:0060365	1.81E-02	5.84E-02	Fgfr2,14183:up
regulation of pancreatic trypsinogen secretion	GO:1904242	1.81E-02	5.84E-02	Mmp13,17386:up
negative regulation of long-term synaptic potentiation	GO:1900272	1.81E-02	5.84E-02	Cx3cr1,13051:down
negative regulation of osteoclast proliferation	GO:0090291	1.81E-02	5.84E-02	Grem1,23892:up
response to serotonin	GO:1904014	1.81E-02	5.84E-02	Htr2b,15559:down
negative regulation of erythrocyte clearance	GO:0034107	1.81E-02	5.84E-02	Cd24a,12484:down
specification of ureteric bud anterior/posterior symmetry	GO:0072100	1.81E-02	5.84E-02	Bmp4,12159:up
canonical Wnt signaling pathway involved in positive regulation of cell-cell adhesion	GO:0044329	1.81E-02	5.84E-02	Plpp3,67916:up
positive regulation of mitotic cell cycle DNA replication	GO:1903465	1.81E-02	5.84E-02	Fgfr1,14182:down
regulation of metanephric comma-shaped body morphogenesis	GO:2000006	1.81E-02	5.84E-02	Bmp4,12159:up
positive regulation of trophectodermal cell proliferation	GO:1904075	1.81E-02	5.84E-02	Igf1,16000:up
polysaccharide localization	GO:0033037	1.81E-02	5.84E-02	Chst11,58250:down
positive regulation of renal albumin absorption	GO:2000534	1.81E-02	5.84E-02	Adipoq,11450:up
stromal-epithelial cell signaling involved in prostate gland development	GO:0044345	1.81E-02	5.84E-02	Sfrp1,20377:up
response to luteinizing hormone	GO:0034699	1.81E-02	5.84E-02	Tgfbr3,21814:up

negative regulation of metanephric S-shaped body morphogenesis	GO:2000005	1.81E-02	5.84E-02	Bmp4,12159:up
ureter epithelial cell differentiation	GO:0072192	1.81E-02	5.84E-02	Bmp4,12159:up
positive regulation of pancreatic trypsinogen secretion	GO:1904244	1.81E-02	5.84E-02	Mmp13,17386:up
metanephric comma-shaped body morphogenesis	GO:0072278	1.81E-02	5.84E-02	Bmp4,12159:up
cellular response to serotonin	GO:1904015	1.81E-02	5.84E-02	Htr2b,15559:down
UMP salvage	GO:0044206	1.81E-02	5.84E-02	Uck2,80914:down
pyrimidine nucleotide salvage	GO:0032262	1.81E-02	5.84E-02	Uck2,80914:down
intermediate mesoderm morphogenesis	GO:0048390	1.81E-02	5.84E-02	Bmp4,12159:up
regulation of transcription from RNA polymerase II promoter involved in mesonephros development	GO:0061216	1.81E-02	5.84E-02	Bmp4,12159:up
L-xylitol metabolic process	GO:0051164	1.81E-02	5.84E-02	Sord,20322:up
mesoderm structural organization	GO:0048338	1.81E-02	5.84E-02	Bmpr1a,12166:down
C21-steroid hormone catabolic process	GO:0008208	1.81E-02	5.84E-02	Akr1c18,105349:up
erythrocyte clearance	GO:0034102	1.81E-02	5.84E-02	Cd24a,12484:down
chemokine receptor transport out of membrane raft	GO:0032600	1.81E-02	5.84E-02	Cd24a,12484:down
detection of oxidative stress	GO:0070994	1.81E-02	5.84E-02	Adipoq,11450:up
uridine transport	GO:0015862	1.81E-02	5.84E-02	Slc29a1,63959:up
posterior midgut development	GO:0007497	1.81E-02	5.84E-02	Ednrb,13618:up
insulin catabolic process	GO:1901143	1.81E-02	5.84E-02	Ceacam1,26365:up
regulation of chemokine (C-C motif) ligand 2 secretion	GO:1904207	1.81E-02	5.84E-02	Postn,50706:down
negative regulation of transforming growth factor beta3 production	GO:0032913	1.81E-02	5.84E-02	Cd24a,12484:down
seminal vesicle epithelium development	GO:0061108	1.81E-02	5.84E-02	Serpine2,20720:up
inorganic diphosphate transport	GO:0030505	1.81E-02	5.84E-02	Enpp1,18605:down
negative regulation of branch elongation involved in ureteric bud branching by BMP signaling pathway	GO:0072097	1.81E-02	5.84E-02	Bmp4,12159:up
response to chromate	GO:0046687	1.81E-02	5.84E-02	Mt1,17748:up
cellular response to amine stimulus	GO:0071418	1.81E-02	5.84E-02	Htr2b,15559:down
hyaloid vascular plexus regression	GO:1990384	1.81E-02	5.84E-02	Ninj1,18081:up
hexitol catabolic process	GO:0019407	1.81E-02	5.84E-02	Sord,20322:up
neural plate mediolateral regionalization	GO:0021998	1.81E-02	5.84E-02	Bmpr1a,12166:down
negative regulation of branch elongation involved in ureteric bud branching	GO:0072096	1.81E-02	5.84E-02	Bmp4,12159:up

positive regulation of cell proliferation involved in outflow tract morphogenesis	GO:1901964	1.81E-02	5.84E-02	Bmp4,12159:up
regulation of cardiac muscle fiber development	GO:0055018	1.81E-02	5.84E-02	Bmp4,12159:up
negative regulation of pancreatic stellate cell proliferation	GO:2000230	1.81E-02	5.84E-02	Pparg,19016:up
protein transport out of membrane raft	GO:0032599	1.81E-02	5.84E-02	Cd24a,12484:down
negative regulation of natural killer cell chemotaxis	GO:2000502	1.81E-02	5.84E-02	Ccl12,20293:up
BMP signaling pathway involved in renal system segmentation	GO:0061151	1.81E-02	5.84E-02	Bmp4,12159:up
pyrimidine ribonucleotide salvage	GO:0010138	1.81E-02	5.84E-02	Uck2,80914:down
negative regulation of cell proliferation involved in heart morphogenesis	GO:2000137	1.81E-02	5.84E-02	Bmp4,12159:up
renal albumin absorption	GO:0097018	1.81E-02	5.84E-02	Adipoq,11450:up
progesterone catabolic process	GO:0006709	1.81E-02	5.84E-02	Akr1c18,105349:up
regulation of retinoic acid biosynthetic process	GO:1900052	1.81E-02	5.84E-02	Akr1c18,105349:up
growth plate cartilage axis specification	GO:0003421	1.81E-02	5.84E-02	Stc1,20855:up
negative regulation of dermatome development	GO:0061185	1.81E-02	5.84E-02	Sfrp2,20319:up
negative regulation of isoprenoid metabolic process	GO:0045827	1.81E-02	5.84E-02	Akr1c18,105349:up
regulation of metanephric S-shaped body morphogenesis	GO:2000004	1.81E-02	5.84E-02	Bmp4,12159:up
endothelin receptor signaling pathway	GO:0086100	1.81E-02	5.84E-02	Ednrb,13618:up
negative regulation of cellular response to hepatocyte growth factor stimulus	GO:2001113	1.81E-02	5.84E-02	Adamts12,239337:down
anterior/posterior pattern specification involved in ureteric bud development	GO:0072099	1.81E-02	5.84E-02	Bmp4,12159:up
fibroblast growth factor receptor signaling pathway involved in hemopoiesis	GO:0035603	1.81E-02	5.84E-02	Fgfr2,14183:up
regulation of trophectodermal cell proliferation	GO:1904073	1.81E-02	5.84E-02	Igf1,16000:up
positive regulation of protein kinase D signaling	GO:1903572	1.81E-02	5.84E-02	Vegfa,22339:down
regulation of metanephric glomerular visceral epithelial cell development	GO:2000477	1.81E-02	5.84E-02	Adipoq,11450:up
ferrous iron transmembrane transport	GO:1903874	1.81E-02	5.84E-02	Slc40a1,53945:up
positive regulation of cardiac muscle fiber development	GO:0055020	1.81E-02	5.84E-02	Bmp4,12159:up
negative regulation of mesenchymal cell proliferation involved in ureter development	GO:0072200	1.81E-02	5.84E-02	Bmp4,12159:up
ferrous iron export	GO:1903988	1.81E-02	5.84E-02	Slc40a1,53945:up

negative regulation of renal phosphate excretion	GO:1903403	1.81E-02	5.84E-02	Stc1,20855:up
negative regulation of T cell homeostatic proliferation	GO:0046014	1.81E-02	5.84E-02	Cd24a,12484:down
mesenchymal-epithelial cell signaling involved in prostate gland development	GO:0060739	1.81E-02	5.84E-02	Tnc,21923:down
fibroblast growth factor receptor signaling pathway involved in negative regulation of apoptotic process in bone marrow	GO:0035602	1.81E-02	5.84E-02	Fgfr2,14183:up
positive regulation of metanephric glomerular visceral epithelial cell development	GO:2000478	1.81E-02	5.84E-02	Adipoq,11450:up
CMP biosynthetic process	GO:0009224	1.81E-02	5.84E-02	Uck2,80914:down
regulation of cellular response to hepatocyte growth factor stimulus	GO:2001112	1.81E-02	5.84E-02	Adamts12,239337:down
macrophage differentiation	GO:0030225	1.81E-02	5.84E-02	Vegfa,22339:down|Bmp4,12159:up|Adipoq,11450:up
iron ion transport	GO:0006826	1.81E-02	5.84E-02	Trf,22041:up|Tfrc,22042:up|Slc40a1,53945:up
positive regulation of B cell mediated immunity	GO:0002714	1.81E-02	5.84E-02	Tnf,21926:up|C3,12266:up|Tfrc,22042:up
fat-soluble vitamin metabolic process	GO:0006775	1.81E-02	5.84E-02	Vdr,22337:down|Tnf,21926:up|Fgfr1,14182:down
positive regulation of immunoglobulin mediated immune response	GO:0002891	1.81E-02	5.84E-02	Tnf,21926:up|C3,12266:up|Tfrc,22042:up
regulation of muscle system process	GO:0090257	1.83E-02	5.91E-02	Fxyd1,56188:up|Lmcd1,30937:up|Wisp1,22402:down|Ptgs2,19225:down|Stc1,20855:up|Igf1,16000:up|Npnt,114249:up|Errfi1,74155:down
regulation of lymphocyte proliferation	GO:0050670	1.83E-02	5.91E-02	Cd24a,12484:down|Cd4,12504:up|Tfrc,22042:up|Igf1,16000:up|Bmp4,12159:up|Ceacam1,26365:up|Igf2,16002:up|Tnfsf9,21950:up
positive regulation of JUN kinase activity	GO:0043507	1.84E-02	5.93E-02	Tnf,21926:up|Trf,22041:up|Wnt5a,22418:down|Il1rn,16181:up
cellular carbohydrate biosynthetic process	GO:0034637	1.84E-02	5.93E-02	Has2,15117:down|Enpp1,18605:down|Igf2,16002:up|Igf1,16000:up
polysaccharide biosynthetic process	GO:0000271	1.84E-02	5.93E-02	Has2,15117:down|Enpp1,18605:down|Igf2,16002:up|Igf1,16000:up

cellular process	GO:0009987	1.87E-02	6.02E-02	Fstl3,83554:down|Igfbp4,16010:up|Adm,11535:up|Flrt2,399558:down|Tmem176b,65963:up|Plxnd1,67784:up|Tfec,21426:up|Sfrp4,20379:up|Ltbp2,16997:down|Serpinh1,12406:down|Lyl1,17095:up|Gstt1,14871:up|Blvrb,233016:up|Igfbp3,16009:up|Ibsp,15891:up|Plk2,20620:down|Cd24a,12484:down|Mme,17380:up|Fkbp14,231997:down|Il1rn,16181:up|Maob,109731:up|Bmp4,12159:up|Oasl1,231655:up|Hpse,15442:up|Medag,70717:down|Ptgir,19222:up|Fgfr2,14183:up|Thbs2,21826:down|Htr2b,15559:down|Dram1,71712:up|Ccl6,20305:up|Gjb5,14622:down|Slpi,20568:up|Aspn,66695:down|Mt2,17750:up|Cxcr6,80901:down|Fam132b,227358:down|Nabp1,109019:down|Mmp2,17390:down|Oas3,246727:up|Slc40a1,53945:up|Egfr,13649:down|Alpl,11647:up|Tfrc,22042:up|Sncg,20618:up|Ccl8,20307:up|Gstm1,14862:up|Serpina3g,20715:up|Rspo2,239405:up|Fam20a,208659:down|Syt13,80976:up|Mt1,17748:up|Col5a2,12832:down|Mrc1,17533:up|Aldh1a2,19378:up|Ccl2,20296:up|Ednrb,13618:up|Ampd3,11717:up|Mmp13,17386:up|Htra3,78558:down|Tnfrsf12a,27279:down|Uchl5,56207:up|Sord,20322:up|Clec4n,56620:up|Shroom4,208431:down|Enpp3,209558:down|Cdo1,12583:up|Dok2,13449:up|Mamdc2,71738:down|Csrp2,13008:down|Hvcn1,74096:up|Ptx3,19288:up|Ncam1,17967:down|Ccl4,20303:up|Chst11,58250:down|Vegfb,22340:up|Aldh1a3,56847:down|Plpp3,67916:up|Mrc2,17534:down|Lrrc17,74511:down|Lama2,16773:down|Ccl9,20308:up|Ptk7,71461:down|Inhba,16323:down|Bnc2,242509:down|Tfpi2,21789:down|Crlf1,12931:down|Plod2,26432:down|Vegfa,22339:down|Gfpt2,14584:down|Serpine2,20720:up|Cbr2,12409:up|Nxn,18230:down|Igf2,16002:up|Kdelr3,105785:down|Tmem176a,66058:up|Tnf,21926:up|Col1a2,12843:down|Fxyd1,56188:up|Fgfr1,14182:down|Lpl,16956:up|Sulf1,240725:down|Susd2,71733:up|Cd4,12504:up|Col3a1,12825:down|Tbx15,21384:down|Serping1,12258:up|Emb,13723:up|Uck2,80914:down|Enpp1,18605:down|Clec2d,93694:up|Hpgd,15446:up|C3,12266:up|Enpp5,83965:up|Ankrd1,107765:down|Cx3cr1,13051:down|Cd109,235505:down|Ggt5,23887:up|Loxl2,94352:down|Loxl3,16950:down|Trf,22041:up|Cxcl14,57266:down|Ceacam10,26366:up|Wnt5a,22418:down|Stc1,20855:up|Mark1,226778:up|Col8a1,12837:down|Adipoq,11450:up|Hal,15109:up|Igf1,16000:up|Crabp2,12904:down|Cxcl9,17329:up|Tgfb2,21808:down|F3,14066:up|Clec4e,56619:up|Cthrc1,68588:down|Kng1,16644:up|Prl2c2,18811:up|Efemp1,216616:up|Dpysl3,22240:down|Ninj1,18081:up|Saa3,20210:up|Vdr,22337:down|Pdia5,72599:down|Fabp4,11770:up|Cxcl12,20315:up|Htr2a,15558:down|Slc16a4,229699:down|Smoc2,64074:down|Gas6,14456:up|Col11a1,12814:down|Sfrp1,20377:up|C1qtnf3,81799:down|Tgfbr3,21814:up|Fxyd2,11936:up|Serinc2,230779:down|Tll1,21892:down|Ceacam1,26365:up|Sfrp2,20319:up|Pxdn,69675:down|Tnfrsf11b,18383:up|Prkar2b,19088:up|Npnt,114249:up|S100a1,20193:up|Ice1,218333:down|Ly9,17085:up|Neurl3,214854:down|Ccl7,20306:up|Pdzrn3,55983:down|Pdgfc,54635:down|Mgp,17313:up|Figf,14205:up|Cst7,13011:up|Ifit3,15959:up|Sema7a,20361:down|Epas1,13819:up|Slc29a1,63959:up|Marc1,66112:up|Gpm6b,14758:up|Ddah1,69219:down|Sdpr,20324:down|Fkbp10,14230:down|Postn,50706:down|Npl,74091:up|Man2a2,140481:up|Akr1c18,105349:up|Fabp5,16592:up|Bmpr1a,12166:down|Ccl12,20293:up|Wisp1,22402:down|Ank,11732:down|Tnc,21923:down|Gatm,67092:down|Ret,19713:up|Prg4,96875:down|Sdf2l1,64136:up|Pdxk,216134:up|Ifit1,15957:up|Creb3l1,26427:down|Il1rl1,17082:down|Kitl,17311:up|Fam101b,76566:down|P4ha1,18451:down|Hp,15439:up|Aoah,27052:up|Rasl11a,68895:up|Plat,18791:down|Ccl3,20302:up|Ctla2a,13024:down|Srpx2,68792:down|Prelp,116847:up|Adamts12,239337:down|Lrrc15,74488:down|Anpep,16790:up|Pparg,19016:up|Glp1r,14652:down|Lmcd1,30937:up|Tnfrsf23,79201:down|Has2,15117:down|Hmox1,15368:up|C1qtnf6,72709:down|Ptgs2,19225:down|Kcnj15,16516:down|Grem1,23892:up|Phf11d,219132:up|Col5a1,12831:down|Mcoln3,171166:up|Errfi1,74155:dow
phosphatidylinositol-mediated signaling	GO:0048015	1.88E-02	6.02E-02	Htr2b,15559:down|Tgfb2,21808:down|Htr2a,15558:down|Pdgfc,54635:down|Serpine2,20720:up|Igf1,16000:up
tumor necrosis factor superfamily cytokine production	GO:0071706	1.88E-02	6.02E-02	Gas6,14456:up|Ccl2,20296:up|Errfi1,74155:down|Ccl4,20303:up|Ccl3,20302:up|Adipoq,11450:up
regulation of steroid metabolic process	GO:0019218	1.89E-02	6.02E-02	Vdr,22337:down|Tnf,21926:up|Akr1c18,105349:up|Igf2,16002:up|Igf1,16000:up
negative regulation of platelet-derived growth factor receptor signaling pathway	GO:0010642	1.91E-02	6.02E-02	Ptgir,19222:up|Adipoq,11450:up
positive regulation of vascular permeability	GO:0043117	1.91E-02	6.02E-02	Vegfa,22339:down|Ccl4,20303:up
chemical homeostasis within a tissue	GO:0048875	1.91E-02	6.02E-02	Vegfa,22339:down|Epas1,13819:up
positive regulation of non-canonical Wnt signaling pathway	GO:2000052	1.91E-02	6.02E-02	Sfrp1,20377:up|Wnt5a,22418:down
lactate transport	GO:0015727	1.91E-02	6.02E-02	Emb,13723:up|Slc16a4,229699:down
plasma membrane lactate transport	GO:0035879	1.91E-02	6.02E-02	Emb,13723:up|Slc16a4,229699:down
response to gonadotropin	GO:0034698	1.91E-02	6.02E-02	Inhba,16323:down|Tgfbr3,21814:up
divalent inorganic anion homeostasis	GO:0072505	1.91E-02	6.02E-02	Sfrp4,20379:up|Enpp1,18605:down
activation-induced cell death of T cells	GO:0006924	1.91E-02	6.02E-02	Ceacam1,26365:up|Tgfb2,21808:down
regulation of Wnt signaling pathway, planar cell polarity pathway	GO:2000095	1.91E-02	6.02E-02	Sfrp2,20319:up|Sfrp1,20377:up
regulation of transcription from RNA polymerase II promoter involved in heart development	GO:1901213	1.91E-02	6.02E-02	Grem1,23892:up|Bmp4,12159:up
regulation of phospholipid biosynthetic process	GO:0071071	1.91E-02	6.02E-02	Htr2b,15559:down|Htr2a,15558:down
positive regulation of monocyte chemotaxis	GO:0090026	1.91E-02	6.02E-02	Cxcl12,20315:up|Ccl2,20296:up

positive regulation of glomerulus development	GO:0090193	1.91E-02	6.02E-02	Ret,19713:up|Adipoq,11450:up
epithelial cell proliferation involved in lung morphogenesis	GO:0060502	1.91E-02	6.02E-02	Fgfr2,14183:up|Bmp4,12159:up
regulation of neuron maturation	GO:0014041	1.91E-02	6.02E-02	Ret,19713:up|Ednrb,13618:up
cholesterol storage	GO:0010878	1.91E-02	6.02E-02	Pparg,19016:up|Lpl,16956:up
positive regulation of vascular endothelial growth factor receptor signaling pathway	GO:0030949	1.91E-02	6.02E-02	Vegfa,22339:down|Bmp4,12159:up
cytokine secretion involved in immune response	GO:0002374	1.91E-02	6.02E-02	Tnf,21926:up|Wnt5a,22418:down
negative regulation of keratinocyte proliferation	GO:0010839	1.91E-02	6.02E-02	Vdr,22337:down|Cd109,235505:down
cartilage morphogenesis	GO:0060536	1.91E-02	6.02E-02	Rspo2,239405:up|Stc1,20855:up
fat-soluble vitamin biosynthetic process	GO:0042362	1.91E-02	6.02E-02	Vdr,22337:down|Tnf,21926:up
striatum development	GO:0021756	1.91E-02	6.02E-02	Inhba,16323:down|Aldh1a3,56847:down
lactate transmembrane transport	GO:0035873	1.91E-02	6.02E-02	Emb,13723:up|Slc16a4,229699:down
mucosal-associated lymphoid tissue development	GO:0048537	1.91E-02	6.02E-02	Ceacam1,26365:up|Ret,19713:up
regulation of protein activation cascade	GO:2000257	1.91E-02	6.02E-02	Serping1,12258:up|C3,12266:up
negative regulation of leukocyte chemotaxis	GO:0002689	1.91E-02	6.02E-02	Ccl12,20293:up|Grem1,23892:up
trachea morphogenesis	GO:0060439	1.91E-02	6.02E-02	Rspo2,239405:up|Bmp4,12159:up
low-density lipoprotein receptor particle metabolic process	GO:0032799	1.91E-02	6.02E-02	Pparg,19016:up|Adipoq,11450:up
positive regulation of metanephros development	GO:0072216	1.91E-02	6.02E-02	Ret,19713:up|Adipoq,11450:up
modulation by symbiont of host cellular process	GO:0044068	1.91E-02	6.02E-02	Ceacam1,26365:up|Cd4,12504:up
neuroblast division	GO:0055057	1.91E-02	6.02E-02	Fgfr1,14182:down|Fgfr2,14183:up
neuronal stem cell division	GO:0036445	1.91E-02	6.02E-02	Fgfr1,14182:down|Fgfr2,14183:up
chemokine metabolic process	GO:0050755	1.91E-02	6.02E-02	Tnf,21926:up|Wnt5a,22418:down
paraxial mesoderm morphogenesis	GO:0048340	1.91E-02	6.02E-02	Wnt5a,22418:down|Bmpr1a,12166:down
muscle contraction	GO:0006936	1.93E-02	6.08E-02	Htr2b,15559:down|Fxyd1,56188:up|Sulf1,240725:down|Ptgs2,19225:down|Stc1,20855:up|Ednrb,13618:up|Vegfb,22340:up|Htr2a,15558:down|Npnt,114249:up
sex differentiation	GO:0007548	1.93E-02	6.08E-02	Sfrp1,20377:up|Wnt5a,22418:down|Inhba,16323:down|Bmpr1a,12166:down|Bmp4,12159:up|Sfrp2,20319:up|Vegfa,22339:down|Mmp2,17390:down|Kitl,17311:up
regulation of mononuclear cell proliferation	GO:0032944	1.94E-02	6.11E-02	Cd24a,12484:down|Cd4,12504:up|Tfrc,22042:up|Igf1,16000:up|Bmp4,12159:up|Ceacam1,26365:up|Igf2,16002:up|Tnfsf9,21950:up
positive regulation of nuclear division	GO:0051785	1.95E-02	6.14E-02	Tnf,21926:up|Wnt5a,22418:down|Igf2,16002:up|Igf1,16000:up
dendritic cell differentiation	GO:0097028	1.97E-02	6.18E-02	Tmem176a,66058:up|Tmem176b,65963:up|Tnfsf9,21950:up
regulation of natural killer cell mediated cytotoxicity	GO:0042269	1.97E-02	6.18E-02	Ceacam1,26365:up|Clec2d,93694:up|Igf2,16002:up
apoptotic process involved in development	GO:1902742	1.97E-02	6.18E-02	Vdr,22337:down|Tgfb2,21808:down|Tgfbr3,21814:up
cellular response to estrogen stimulus	GO:0071391	1.97E-02	6.18E-02	Egfr,13649:down|Sfrp1,20377:up|Mmp2,17390:down
positive regulation of steroid metabolic process	GO:0045940	1.97E-02	6.18E-02	Tnf,21926:up|Igf2,16002:up|Igf1,16000:up
regulation of interferon-gamma production	GO:0032649	1.98E-02	6.21E-02	Tnf,21926:up|Gas6,14456:up|Wnt5a,22418:down|Il1rl1,17082:down|Tnfsf9,21950:up

inositol lipid-mediated signaling	GO:0048017	2.03E-02	6.35E-02	Htr2b,15559:down|Tgfb2,21808:down|Htr2a,15558:down|Pdgfc,54635:down|Serpine2,20720:up|Igf1,16000:up
single-organism catabolic process	GO:0044712	2.05E-02	6.44E-02	Hp,15439:up|Sfrp4,20379:up|Mmp13,17386:up|Enpp5,83965:up|Sord,20322:up|Ddah1,69219:down|Plod2,26432:down|Htr2a,15558:down|Mmp2,17390:down|Blvrb,233016:up|Npl,74091:up|Enpp3,209558:down|Cdo1,12583:up|Tnf,21926:up|Akr1c18,105349:up|Lmcd1,30937:up|Hmox1,15368:up|Lpl,16956:up|Adipoq,11450:up|Hal,15109:up|Igf1,16000:up|Pxdn,69675:down|Hpse,15442:up|Sdf2l1,64136:up|Enpp1,18605:down|Mrc2,17534:down
oxidation-reduction process	GO:0055114	2.06E-02	6.45E-02	P4ha1,18451:down|Hpgd,15446:up|Aldh1a2,19378:up|Marc1,66112:up|Sord,20322:up|Plod2,26432:down|Steap1,70358:down|Blvrb,233016:up|Cbr2,12409:up|Nxn,18230:down|Igf2,16002:up|Cdo1,12583:up|Pparg,19016:up|Loxl2,94352:down|Akr1c18,105349:up|Loxl3,16950:down|Hmox1,15368:up|Ptgs2,19225:down|Adipoq,11450:up|Igf1,16000:up|Maob,109731:up|Pxdn,69675:down|Aldh1a3,56847:down|Enpp1,18605:down
branching involved in ureteric bud morphogenesis	GO:0001658	2.07E-02	6.47E-02	Vegfa,22339:down|Npnt,114249:up|Grem1,23892:up|Bmp4,12159:up
regulation of protein processing	GO:0070613	2.07E-02	6.47E-02	Ctla2a,13024:down|Serping1,12258:up|C3,12266:up|Serpine2,20720:up
sensory perception of mechanical stimulus	GO:0050954	2.13E-02	6.65E-02	Tnf,21926:up|Fgfr1,14182:down|Col11a1,12814:down|Mcoln3,171166:up|Htr2a,15558:down|Serpine2,20720:up|Igf1,16000:up
positive regulation of bone mineralization	GO:0030501	2.14E-02	6.65E-02	Gpm6b,14758:up|Bmpr1a,12166:down|Bmp4,12159:up
positive regulation of BMP signaling pathway	GO:0030513	2.14E-02	6.65E-02	Sulf1,240725:down|Tgfbr3,21814:up|Bmp4,12159:up
positive regulation of myotube differentiation	GO:0010831	2.14E-02	6.65E-02	Ccl8,20307:up|Cxcl9,17329:up|Cxcl12,20315:up
heart valve morphogenesis	GO:0003179	2.14E-02	6.65E-02	Tgfb2,21808:down|Bmpr1a,12166:down|Bmp4,12159:up
regulation of natural killer cell mediated immunity	GO:0002715	2.14E-02	6.65E-02	Ceacam1,26365:up|Clec2d,93694:up|Igf2,16002:up
positive regulation of Ras protein signal transduction	GO:0046579	2.14E-02	6.65E-02	Col3a1,12825:down|Kitl,17311:up|Igf1,16000:up
endoderm formation	GO:0001706	2.14E-02	6.65E-02	Col5a2,12832:down|Col5a1,12831:down|Inhba,16323:down
insulin-like growth factor receptor signaling pathway	GO:0048009	2.14E-02	6.65E-02	Igfbp4,16010:up|Igfbp3,16009:up|Igf1,16000:up
olfactory lobe development	GO:0021988	2.14E-02	6.65E-02	Fgfr1,14182:down|Wnt5a,22418:down|Sema7a,20361:down
cell projection organization	GO:0030030	2.15E-02	6.67E-02	Cthrc1,68588:down|Prl2c2,18811:up|Adm,11535:up|Flrt2,399558:down|Lama2,16773:down|Efemp1,216616:up|Plxnd1,67784:up|Ptk7,71461:down|Dpysl3,22240:down|Sema7a,20361:down|Ankrd1,107765:down|Tnfrsf12a,27279:down|Cxcl12,20315:up|Postn,50706:down|Vegfa,22339:down|Mmp2,17390:down|Serpine2,20720:up|Plk2,20620:down|Cd24a,12484:down|Egfr,13649:down|Fgfr1,14182:down|Ncam1,17967:down|Sfrp1,20377:up|Wnt5a,22418:down|Bmp4,12159:up|Sfrp2,20319:up|Crabp2,12904:down|Tnc,21923:down|Ret,19713:up|Tgfb2,21808:down|Fgfr2,14183:up
nucleoside phosphate catabolic process	GO:1901292	2.16E-02	6.72E-02	Htr2a,15558:down|Enpp1,18605:down|Enpp3,209558:down|Enpp5,83965:up|Igf1,16000:up
small GTPase mediated signal transduction	GO:0007264	2.18E-02	6.76E-02	Ccl9,20308:up|Ccl6,20305:up|Rasl11a,68895:up|Ccl7,20306:up|Ccl2,20296:up|Ccl3,20302:up|Dok2,13449:up|Col1a2,12843:down|Plk2,20620:down|Hmox1,15368:up|Wnt5a,22418:down|Ccl12,20293:up|Ccl4,20303:up|Igf1,16000:up|Col3a1,12825:down|Ccl8,20307:up|Tgfb2,21808:down|Kitl,17311:up
regulation of protein maturation	GO:1903317	2.19E-02	6.78E-02	Ctla2a,13024:down|Serping1,12258:up|C3,12266:up|Serpine2,20720:up
regulation of organic acid transport	GO:0032890	2.19E-02	6.78E-02	Tnf,21926:up|Fxyd1,56188:up|Fam132b,227358:down|Il1rn,16181:up
establishment of apical/basal cell polarity	GO:0035089	2.23E-02	6.83E-02	Ptk7,71461:down|Wnt5a,22418:down
ventricular trabecula myocardium morphogenesis	GO:0003222	2.23E-02	6.83E-02	Tgfb2,21808:down|Bmpr1a,12166:down
craniofacial suture morphogenesis	GO:0097094	2.23E-02	6.83E-02	Fgfr2,14183:up|Bmp4,12159:up
branching involved in labyrinthine layer morphogenesis	GO:0060670	2.23E-02	6.83E-02	Adm,11535:up|Fgfr2,14183:up
iron ion import	GO:0097286	2.23E-02	6.83E-02	Trf,22041:up|Tfrc,22042:up
positive regulation of osteoblast proliferation	GO:0033690	2.23E-02	6.83E-02	Cthrc1,68588:down|Hpse,15442:up
glomerular visceral epithelial cell differentiation	GO:0072112	2.23E-02	6.83E-02	Bmp4,12159:up|Adipoq,11450:up
renal filtration cell differentiation	GO:0061318	2.23E-02	6.83E-02	Bmp4,12159:up|Adipoq,11450:up
dopamine biosynthetic process	GO:0042416	2.23E-02	6.83E-02	Tgfb2,21808:down|Epas1,13819:up

regulation of SMAD protein import into nucleus	GO:0060390	2.23E-02	6.83E-02	Bmpr1a,12166:down|Bmp4,12159:up
eyelid development in camera-type eye	GO:0061029	2.23E-02	6.83E-02	Egfr,13649:down|Inhba,16323:down
glial cell apoptotic process	GO:0034349	2.23E-02	6.83E-02	Gas6,14456:up|Ccl12,20293:up
glomerular epithelial cell differentiation	GO:0072311	2.23E-02	6.83E-02	Bmp4,12159:up|Adipoq,11450:up
enteric nervous system development	GO:0048484	2.23E-02	6.83E-02	Ret,19713:up|Ednrb,13618:up
negative regulation of androgen receptor signaling pathway	GO:0060766	2.23E-02	6.83E-02	Sfrp1,20377:up|Igf1,16000:up
metanephric glomerulus development	GO:0072224	2.23E-02	6.83E-02	Ret,19713:up|Adipoq,11450:up
prostate gland growth	GO:0060736	2.23E-02	6.83E-02	Fgfr2,14183:up|Igf1,16000:up
G-protein coupled receptor internalization	GO:0002031	2.23E-02	6.83E-02	Htr2b,15559:down|Adm,11535:up
regulation of cytoplasmic transport	GO:1903649	2.23E-02	6.84E-02	Tnf,21926:up|Gas6,14456:up|Egfr,13649:down|C1qtnf3,81799:down|Bmpr1a,12166:down|Cd4,12504:up|Ptgs2,19225:down|Grem1,23892:up|Igf1,16000:up|Bmp4,12159:up|Ceacam1,26365:up|Sfrp2,20319:up|Tgfb2,21808:down
response to alkaloid	GO:0043279	2.26E-02	6.91E-02	Tnf,21926:up|Ncam1,17967:down|Hmox1,15368:up|Htr2a,15558:down|Igf2,16002:up
polysaccharide metabolic process	GO:0005976	2.26E-02	6.91E-02	Has2,15117:down|Aoah,27052:up|Enpp1,18605:down|Igf2,16002:up|Igf1,16000:up
cardiac muscle cell development	GO:0055013	2.31E-02	7.06E-02	Vegfa,22339:down|Wisp1,22402:down|Igf1,16000:up|Bmp4,12159:up
SMAD protein signal transduction	GO:0060395	2.31E-02	7.06E-02	Trf,22041:up|Tgfb2,21808:down|Inhba,16323:down|Bmp4,12159:up
negative regulation of MAP kinase activity	GO:0043407	2.31E-02	7.06E-02	Sfrp2,20319:up|Sfrp1,20377:up|Bmp4,12159:up|Adipoq,11450:up
inner ear receptor cell differentiation	GO:0060113	2.31E-02	7.06E-02	Cthrc1,68588:down|Fgfr1,14182:down|Mcoln3,171166:up|Bmp4,12159:up
embryonic forelimb morphogenesis	GO:0035115	2.31E-02	7.06E-02	Crabp2,12904:down|Rspo2,239405:up|Aldh1a2,19378:up
specification of organ identity	GO:0010092	2.31E-02	7.06E-02	Fgfr1,14182:down|Fgfr2,14183:up|Bmp4,12159:up
macromolecule metabolic process	GO:0043170	2.34E-02	7.14E-02	Fstl3,83554:down|Igfbp4,16010:up|Adm,11535:up|Flrt2,399558:down|Tfec,21426:up|Sfrp4,20379:up|Serpinh1,12406:down|Lyl1,17095:up|Gstt1,14871:up|Igfbp3,16009:up|Plk2,20620:down|Cd24a,12484:down|Mme,17380:up|Fkbp14,231997:down|Il1rn,16181:up|Bmp4,12159:up|Hpse,15442:up|Fgfr2,14183:up|Htr2b,15559:down|Ccl6,20305:up|Slpi,20568:up|Aspn,66695:down|Nabp1,109019:down|Mmp2,17390:down|Oas3,246727:up|Pappa,18491:down|Slc40a1,53945:up|Egfr,13649:down|C1rb,667277:up|Tfrc,22042:up|Ccl8,20307:up|Serpina3g,20715:up|Fam20a,208659:down|Aldh1a2,19378:up|Ccl2,20296:up|Ednrb,13618:up|Mmp13,17386:up|Htra3,78558:down|Uchl5,56207:up|Dok2,13449:up|Mamdc2,71738:down|Ptx3,19288:up|Ccl4,20303:up|Chst11,58250:down|Vegfb,22340:up|Plpp3,67916:up|Mrc2,17534:down|Ccl9,20308:up|Inhba,16323:down|Bnc2,242509:down|Tfpi2,21789:down|Crlf1,12931:down|C1s1,50908:up|Vegfa,22339:down|Serpine2,20720:up|Nxn,18230:down|Igf2,16002:up|Tnf,21926:up|Fgfr1,14182:down|Sulf1,240725:down|Cd4,12504:up|Col3a1,12825:down|Tbx15,21384:down|Serping1,12258:up|Enpp1,18605:down|C3,12266:up|Ankrd1,107765:down|Cd109,235505:down|Ggt5,23887:up|Loxl2,94352:down|Trf,22041:up|Loxl3,16950:down|Adam12,11489:down|Wnt5a,22418:down|Mark1,226778:up|Adipoq,11450:up|Igf1,16000:up|Tgfb2,21808:down|F3,14066:up|Adamts4,240913:down|Prl2c2,18811:up|Kng1,16644:up|Efemp1,216616:up|Saa3,20210:up|Vdr,22337:down|Pdia5,72599:down|Fabp4,11770:up|Htr2a,15558:down|Gas6,14456:up|Col11a1,12814:down|Sfrp1,20377:up|Tgfbr3,21814:up|C1qtnf3,81799:down|Tll1,21892:down|Ceacam1,26365:up|Sfrp2,20319:up|Pamr1,210622:up|Tnfrsf11b,18383:up|Prkar2b,19088:up|Npnt,114249:up|S100a1,20193:up|Ice1,218333:down|Neurl3,214854:down|Ccl7,20306:up|Cst7,13011:up|Pdgfc,54635:down|Pdzrn3,55983:down|Epas1,13819:up|Sema7a,20361:down|Ddah1,69219:down|Fkbp10,14230:down|Sdpr,20324:down|Man2a2,140481:up|Ccl12,20293:up|Bmpr1a,12166:down|Klk1b11,16613:up|Tnc,21923:down|Ret,19713:up|Prg4,96875:down|Sdf2l1,64136:up|C1s2,317677:up|Creb3l1,26427:down|Kitl,17311:up|P4ha1,18451:down|Hp,15439:up|Aoah,27052:up|Rasl11a,68895:up|Ccl3,20302:up|Plat,18791:down|Ctla2a,13024:down|Adamts12,239337:down|Lrrc15,74488:down|Anpep,16790:up|Pparg,19016:up|Lmcd1,30937:up|Glp1r,14652:down|Hmox1,15368:up|Has2,15117:down|Grem1,23892:up|Phf11d,219132:up|Col5a1,12831:down|Errfi1,74155:down
regulation of adaptive immune response	GO:0002819	2.34E-02	7.14E-02	Tnf,21926:up|Cd24a,12484:down|Cd4,12504:up|C3,12266:up|Tfrc,22042:up|Il1rl1,17082:down
regulation of Ran protein signal transduction	GO:0032015	2.37E-02	7.20E-02	Ccl9,20308:up|Ccl6,20305:up|Wnt5a,22418:down|Ccl7,20306:up|Ccl2,20296:up|Ccl12,20293:up|Ccl4,20303:up|Ccl3,20302:up|Ccl8,20307:up
regulation of GTPase activity	GO:0043087	2.37E-02	7.20E-02	Ccl9,20308:up|Ccl6,20305:up|Wnt5a,22418:down|Ccl7,20306:up|Ccl2,20296:up|Ccl12,20293:up|Ccl4,20303:up|Ccl3,20302:up|Ccl8,20307:up
positive regulation of macromolecule biosynthetic process	GO:0010557	2.41E-02	7.33E-02	Fstl3,83554:down|Ice1,218333:down|Ccl2,20296:up|Pdgfc,54635:down|Epas1,13819:up|Lyl1,17095:up|Sdpr,20324:down|Trf,22041:up|Wnt5a,22418:down|Bmpr1a,12166:down|Bmp4,12159:up|Igf1,16000:up|Ret,19713:up|Tgfb2,21808:down|Plpp3,67916:up|Creb3l1,26427:down|Fgfr2,14183:up|Kitl,17311:up|Prl2c2,18811:up|Rasl11a,68895:up|Inhba,16323:down|Ccl3,20302:up|Vdr,22337:down|Vegfa,22339:down|Igf2,16002:up|Slc40a1,53945:up|Tnf,21926:up|Pparg,19016:up|Glp1r,14652:down|Egfr,13649:down|Fgfr1,14182:down|Sfrp1,20377:up|Has2,15117:down|Tgfbr3,21814:up|Grem1,23892:up|Sfrp2,20319:up|Npnt,114249:up

Ran protein signal transduction	GO:0031291	2.42E-02	7.37E-02	Ccl9,20308:up|Ccl6,20305:up|Wnt5a,22418:down|Ccl7,20306:up|Ccl2,20296:up|Ccl12,20293:up|Ccl4,20303:up|Ccl3,20302:up|Ccl8,20307:up
regulation of Rap protein signal transduction	GO:0032487	2.42E-02	7.37E-02	Ccl9,20308:up|Ccl6,20305:up|Wnt5a,22418:down|Ccl7,20306:up|Ccl2,20296:up|Ccl12,20293:up|Ccl4,20303:up|Ccl3,20302:up|Ccl8,20307:up
forebrain neuron differentiation	GO:0021879	2.44E-02	7.40E-02	Fgfr1,14182:down|Wnt5a,22418:down|Inhba,16323:down|Fgfr2,14183:up
telencephalon cell migration	GO:0022029	2.44E-02	7.40E-02	Fgfr1,14182:down|Egfr,13649:down|Cxcl12,20315:up|Cx3cr1,13051:down
regulation of cell killing	GO:0031341	2.44E-02	7.40E-02	Ceacam1,26365:up|Clec2d,93694:up|Ccl2,20296:up|Igf2,16002:up
zymogen activation	GO:0031638	2.46E-02	7.45E-02	Tnf,21926:up|Pparg,19016:up|Serpine2,20720:up|Plat,18791:down|F3,14066:up
response to alcohol	GO:0097305	2.48E-02	7.51E-02	Vdr,22337:down|Egfr,13649:down|Inhba,16323:down|Ccl7,20306:up|Mmp2,17390:down|Cdo1,12583:up|Bmp4,12159:up
regulation of Rab protein signal transduction	GO:0032483	2.48E-02	7.51E-02	Ccl9,20308:up|Ccl6,20305:up|Wnt5a,22418:down|Ccl7,20306:up|Ccl2,20296:up|Ccl12,20293:up|Ccl4,20303:up|Ccl3,20302:up|Ccl8,20307:up
viral process	GO:0016032	2.49E-02	7.51E-02	Tnf,21926:up|Gas6,14456:up|Lmcd1,30937:up|Ptx3,19288:up|Cd4,12504:up|Slpi,20568:up|Ccl4,20303:up|Ifit3,15959:up|Ceacam1,26365:up|Cxcl9,17329:up|Oasl1,231655:up|Ifit1,15957:up|Lrrc15,74488:down|Oas3,246727:up
T cell activation	GO:0042110	2.49E-02	7.51E-02	Cd24a,12484:down|Ly9,17085:up|Ccl2,20296:up|Cd4,12504:up|Tfrc,22042:up|Igf1,16000:up|Bmp4,12159:up|Ctla2a,13024:down|Ceacam1,26365:up|Cxcl12,20315:up|Igf2,16002:up|Clec4e,56619:up|Tnfsf9,21950:up
T cell aggregation	GO:0070489	2.49E-02	7.51E-02	Cd24a,12484:down|Ly9,17085:up|Ccl2,20296:up|Cd4,12504:up|Tfrc,22042:up|Igf1,16000:up|Bmp4,12159:up|Ctla2a,13024:down|Ceacam1,26365:up|Cxcl12,20315:up|Igf2,16002:up|Clec4e,56619:up|Tnfsf9,21950:up
regulation of receptor internalization	GO:0002090	2.50E-02	7.51E-02	Vegfa,22339:down|Sfrp4,20379:up|Grem1,23892:up
positive regulation of multicellular organismal metabolic process	GO:0044253	2.50E-02	7.51E-02	Pparg,19016:up|Ccl2,20296:up|Bmp4,12159:up
dopamine metabolic process	GO:0042417	2.50E-02	7.51E-02	Tgfb2,21808:down|Maob,109731:up|Epas1,13819:up
glucan biosynthetic process	GO:0009250	2.50E-02	7.51E-02	Enpp1,18605:down|Igf2,16002:up|Igf1,16000:up
positive regulation of heart contraction	GO:0045823	2.50E-02	7.51E-02	Glp1r,14652:down|Adm,11535:up|Tgfb2,21808:down
forebrain neuron development	GO:0021884	2.50E-02	7.51E-02	Fgfr1,14182:down|Wnt5a,22418:down|Fgfr2,14183:up
T cell migration	GO:0072678	2.50E-02	7.51E-02	Cxcl12,20315:up|Wnt5a,22418:down|Ccl2,20296:up
negative regulation of cellular carbohydrate metabolic process	GO:0010677	2.50E-02	7.51E-02	C1qtnf3,81799:down|Enpp1,18605:down|Adipoq,11450:up
glycogen biosynthetic process	GO:0005978	2.50E-02	7.51E-02	Enpp1,18605:down|Igf2,16002:up|Igf1,16000:up
endocardial cushion development	GO:0003197	2.50E-02	7.51E-02	Tgfb2,21808:down|Bmpr1a,12166:down|Bmp4,12159:up
lipid biosynthetic process	GO:0008610	2.54E-02	7.61E-02	Htr2b,15559:down|Ggt5,23887:up|Tnf,21926:up|Akr1c18,105349:up|Fabp5,16592:up|Lpl,16956:up|Aldh1a2,19378:up|C3,12266:up|Ptgs2,19225:down|Igf1,16000:up|Vdr,22337:down|Ceacam1,26365:up|Htr2a,15558:down|Aldh1a3,56847:down|Igf2,16002:up
lymphocyte aggregation	GO:0071593	2.54E-02	7.61E-02	Cd24a,12484:down|Ly9,17085:up|Ccl2,20296:up|Cd4,12504:up|Tfrc,22042:up|Igf1,16000:up|Bmp4,12159:up|Ctla2a,13024:down|Ceacam1,26365:up|Cxcl12,20315:up|Igf2,16002:up|Clec4e,56619:up|Tnfsf9,21950:up
regulation of Ral protein signal transduction	GO:0032485	2.55E-02	7.61E-02	Ccl9,20308:up|Ccl6,20305:up|Wnt5a,22418:down|Ccl7,20306:up|Ccl2,20296:up|Ccl12,20293:up|Ccl4,20303:up|Ccl3,20302:up|Ccl8,20307:up
Ral protein signal transduction	GO:0032484	2.55E-02	7.61E-02	Ccl9,20308:up|Ccl6,20305:up|Wnt5a,22418:down|Ccl7,20306:up|Ccl2,20296:up|Ccl12,20293:up|Ccl4,20303:up|Ccl3,20302:up|Ccl8,20307:up
organic hydroxy compound transport	GO:0015850	2.56E-02	7.61E-02	Sncg,20618:up|Maob,109731:up|Adipoq,11450:up|Gpm6b,14758:up|Cxcl12,20315:up|Emb,13723:up|Htr2a,15558:down|Slc16a4,229699:down
chronic inflammatory response	GO:0002544	2.57E-02	7.61E-02	Tnf,21926:up|Cx3cr1,13051:down
negative regulation of fibroblast growth factor receptor signaling pathway	GO:0040037	2.57E-02	7.61E-02	Sulf1,240725:down|Wnt5a,22418:down
activation of phospholipase C activity	GO:0007202	2.57E-02	7.61E-02	Htr2b,15559:down|Htr2a,15558:down
positive regulation of hormone biosynthetic process	GO:0046886	2.57E-02	7.61E-02	Igf2,16002:up|Igf1,16000:up
regulation of monocyte chemotactic protein-1 production	GO:0071637	2.57E-02	7.61E-02	C1qtnf3,81799:down|Adipoq,11450:up
establishment of monopolar cell polarity	GO:0061162	2.57E-02	7.61E-02	Ptk7,71461:down|Wnt5a,22418:down

chondroitin sulfate proteoglycan biosynthetic process	GO:0050650	2.57E-02	7.61E-02	Chst11,58250:down|Igf1,16000:up
positive regulation of glycogen metabolic process	GO:0070875	2.57E-02	7.61E-02	Igf2,16002:up|Igf1,16000:up
white fat cell differentiation	GO:0050872	2.57E-02	7.61E-02	Pparg,19016:up|Fabp4,11770:up
regulation of endothelial cell chemotaxis	GO:2001026	2.57E-02	7.61E-02	Fgfr1,14182:down|Vegfa,22339:down
glycoprotein catabolic process	GO:0006516	2.57E-02	7.61E-02	Hpse,15442:up|Adamts12,239337:down
positive regulation of endothelial cell apoptotic process	GO:2000353	2.57E-02	7.61E-02	Akr1c18,105349:up|Bmp4,12159:up
regulation of response to tumor cell	GO:0002834	2.57E-02	7.61E-02	Ceacam1,26365:up|Cd24a,12484:down
endothelial tube morphogenesis	GO:0061154	2.57E-02	7.61E-02	Adamts12,239337:down|Bmp4,12159:up
detection of mechanical stimulus involved in sensory perception of pain	GO:0050966	2.57E-02	7.61E-02	Tnf,21926:up|Htr2a,15558:down
regulation of immune response to tumor cell	GO:0002837	2.57E-02	7.61E-02	Ceacam1,26365:up|Cd24a,12484:down
monocyte chemotactic protein-1 production	GO:0071605	2.57E-02	7.61E-02	C1qtnf3,81799:down|Adipoq,11450:up
metanephric renal vesicle morphogenesis	GO:0072283	2.57E-02	7.61E-02	Grem1,23892:up|Bmp4,12159:up
positive regulation of glycolytic process	GO:0045821	2.57E-02	7.61E-02	Htr2a,15558:down|Igf1,16000:up
positive regulation of glycogen biosynthetic process	GO:0045725	2.57E-02	7.61E-02	Igf2,16002:up|Igf1,16000:up
regulation of cell growth involved in cardiac muscle cell development	GO:0061050	2.57E-02	7.61E-02	Wisp1,22402:down|Igf1,16000:up
glomerular epithelium development	GO:0072010	2.57E-02	7.61E-02	Bmp4,12159:up|Adipoq,11450:up
regulation of lipase activity	GO:0060191	2.57E-02	7.61E-02	Htr2b,15559:down|Fgfr1,14182:down|Htr2a,15558:down|Fgfr2,14183:up
regulation of glucose import	GO:0046324	2.57E-02	7.61E-02	Tnf,21926:up|Enpp1,18605:down|Igf1,16000:up|Adipoq,11450:up
regulation of cell junction assembly	GO:1901888	2.57E-02	7.61E-02	Tnf,21926:up|Gpm6b,14758:up|Vegfa,22339:down|Grem1,23892:up
regulation of muscle cell apoptotic process	GO:0010660	2.57E-02	7.61E-02	Sfrp2,20319:up|Hmox1,15368:up|Igfbp3,16009:up|Igf1,16000:up
regulation of muscle adaptation	GO:0043502	2.57E-02	7.61E-02	Lmcd1,30937:up|Wisp1,22402:down|Errfi1,74155:down|Igf1,16000:up
cardiocyte differentiation	GO:0035051	2.60E-02	7.70E-02	Vegfa,22339:down|Tgfb2,21808:down|Wisp1,22402:down|Grem1,23892:up|Igf1,16000:up|Bmp4,12159:up
regulation of Cdc42 protein signal transduction	GO:0032489	2.61E-02	7.70E-02	Ccl9,20308:up|Ccl6,20305:up|Wnt5a,22418:down|Ccl7,20306:up|Ccl2,20296:up|Ccl12,20293:up|Ccl4,20303:up|Ccl3,20302:up|Ccl8,20307:up
striated muscle cell differentiation	GO:0051146	2.61E-02	7.70E-02	Wisp1,22402:down|Grem1,23892:up|Igf1,16000:up|Bmp4,12159:up|Ccl8,20307:up|Cxcl9,17329:up|Cxcl12,20315:up|Vegfa,22339:down|Igf2,16002:up
Rab protein signal transduction	GO:0032482	2.61E-02	7.70E-02	Ccl9,20308:up|Ccl6,20305:up|Wnt5a,22418:down|Ccl7,20306:up|Ccl2,20296:up|Ccl12,20293:up|Ccl4,20303:up|Ccl3,20302:up|Ccl8,20307:up
multi-organism cellular process	GO:0044764	2.62E-02	7.72E-02	Tnf,21926:up|Gas6,14456:up|Lmcd1,30937:up|Ptx3,19288:up|Cd4,12504:up|Slpi,20568:up|Ccl4,20303:up|Ifit3,15959:up|Ceacam1,26365:up|Cxcl9,17329:up|Oasl1,231655:up|Ifit1,15957:up|Lrrc15,74488:down|Oas3,246727:up
small molecule biosynthetic process	GO:0044283	2.62E-02	7.72E-02	Ggt5,23887:up|Tnf,21926:up|Akr1c18,105349:up|Fabp5,16592:up|Lpl,16956:up|C1qtnf3,81799:down|Aldh1a2,19378:up|Ptgs2,19225:down|Adipoq,11450:up|Vdr,22337:down|Sord,20322:up|Gatm,67092:down|Aldh1a3,56847:down|Cdo1,12583:up
organic hydroxy compound biosynthetic process	GO:1901617	2.63E-02	7.76E-02	Tnf,21926:up|Akr1c18,105349:up|Fabp5,16592:up|Wnt5a,22418:down|Epas1,13819:up|Vdr,22337:down|Pdxk,216134:up|Tgfb2,21808:down
negative regulation of nervous system development	GO:0051961	2.64E-02	7.79E-02	Tnf,21926:up|Plk2,20620:down|Cd24a,12484:down|Efemp1,216616:up|Wnt5a,22418:down|Bmpr1a,12166:down|Dpysl3,22240:down|Ednrb,13618:up|Col3a1,12825:down|Bmp4,12159:up
positive regulation of leukocyte mediated immunity	GO:0002705	2.67E-02	7.86E-02	Tnf,21926:up|Cd24a,12484:down|Ccl2,20296:up|C3,12266:up|Tfrc,22042:up
interferon-gamma production	GO:0032609	2.67E-02	7.86E-02	Tnf,21926:up|Gas6,14456:up|Wnt5a,22418:down|Il1rl1,17082:down|Tnfsf9,21950:up
ammonium transport	GO:0015696	2.67E-02	7.86E-02	Gpm6b,14758:up|Cxcl12,20315:up|Htr2a,15558:down|Sncg,20618:up|Maob,109731:up

neuromuscular junction development	GO:0007528	2.69E-02	7.88E-02	Tnc,21923:down|Pdzrn3,55983:down|Fgfr2,14183:up
positive regulation of biomineral tissue development	GO:0070169	2.69E-02	7.88E-02	Gpm6b,14758:up|Bmpr1a,12166:down|Bmp4,12159:up
mammary gland duct morphogenesis	GO:0060603	2.69E-02	7.88E-02	Vdr,22337:down|Wnt5a,22418:down|Fgfr2,14183:up
pituitary gland development	GO:0021983	2.69E-02	7.88E-02	Wnt5a,22418:down|Bmpr1a,12166:down|Bmp4,12159:up
regulation of lipid storage	GO:0010883	2.69E-02	7.88E-02	Pparg,19016:up|Lpl,16956:up|C3,12266:up
regulation of hormone metabolic process	GO:0032350	2.69E-02	7.88E-02	Akr1c18,105349:up|Igf2,16002:up|Igf1,16000:up
defense response to other organism	GO:0098542	2.71E-02	7.92E-02	Adamts4,240913:down|Tnf,21926:up|Lmcd1,30937:up|Adm,11535:up|Hp,15439:up|Cd4,12504:up|Slpi,20568:up|Ifit3,15959:up|Clec4n,56620:up|Cxcl9,17329:up|Oasl1,231655:up|Ifit1,15957:up|Oas3,246727:up|Clec4e,56619:up
cardiac cell development	GO:0055006	2.71E-02	7.92E-02	Vegfa,22339:down|Wisp1,22402:down|Igf1,16000:up|Bmp4,12159:up
ureteric bud morphogenesis	GO:0060675	2.71E-02	7.92E-02	Vegfa,22339:down|Npnt,114249:up|Grem1,23892:up|Bmp4,12159:up
Cdc42 protein signal transduction	GO:0032488	2.73E-02	8.00E-02	Ccl9,20308:up|Ccl6,20305:up|Wnt5a,22418:down|Ccl7,20306:up|Ccl2,20296:up|Ccl12,20293:up|Ccl4,20303:up|Ccl3,20302:up|Ccl8,20307:up
mesonephric tubule morphogenesis	GO:0072171	2.85E-02	8.32E-02	Vegfa,22339:down|Npnt,114249:up|Grem1,23892:up|Bmp4,12159:up
forebrain cell migration	GO:0021885	2.85E-02	8.32E-02	Fgfr1,14182:down|Egfr,13649:down|Cxcl12,20315:up|Cx3cr1,13051:down
regulation of Rac protein signal transduction	GO:0035020	2.87E-02	8.37E-02	Ccl9,20308:up|Ccl6,20305:up|Wnt5a,22418:down|Ccl7,20306:up|Ccl2,20296:up|Ccl12,20293:up|Ccl4,20303:up|Ccl3,20302:up|Ccl8,20307:up
positive regulation of gene expression	GO:0010628	2.89E-02	8.41E-02	Fstl3,83554:down|Ice1,218333:down|Sfrp4,20379:up|Aldh1a2,19378:up|C3,12266:up|Epas1,13819:up|Lyl1,17095:up|Sdpr,20324:down|Trf,22041:up|Wnt5a,22418:down|Bmpr1a,12166:down|Bmp4,12159:up|Igf1,16000:up|Tnc,21923:down|Ret,19713:up|Tgfb2,21808:down|Plpp3,67916:up|Creb3l1,26427:down|Fgfr2,14183:up|Prl2c2,18811:up|Rasl11a,68895:up|Inhba,16323:down|Ccl3,20302:up|Vdr,22337:down|Vegfa,22339:down|Igf2,16002:up|Slc40a1,53945:up|Pparg,19016:up|Gas6,14456:up|Tnf,21926:up|Glp1r,14652:down|Egfr,13649:down|Fgfr1,14182:down|Sfrp1,20377:up|Tgfbr3,21814:up|Grem1,23892:up|Sfrp2,20319:up|Npnt,114249:up
heterotypic cell-cell adhesion	GO:0034113	2.89E-02	8.41E-02	Tnf,21926:up|Il1rn,16181:up|Adipoq,11450:up
myoblast fusion	GO:0007520	2.89E-02	8.41E-02	Ccl8,20307:up|Cxcl9,17329:up|Cxcl12,20315:up
cochlea development	GO:0090102	2.89E-02	8.41E-02	Cthrc1,68588:down|Ptk7,71461:down|Wnt5a,22418:down
regulation of endothelial cell apoptotic process	GO:2000351	2.89E-02	8.41E-02	Gas6,14456:up|Akr1c18,105349:up|Bmp4,12159:up
dopamine transport	GO:0015872	2.89E-02	8.41E-02	Cxcl12,20315:up|Htr2a,15558:down|Sncg,20618:up
segmentation	GO:0035282	2.89E-02	8.42E-02	Sfrp2,20319:up|Sfrp1,20377:up|Wnt5a,22418:down|Bmpr1a,12166:down|Bmp4,12159:up
cellular response to steroid hormone stimulus	GO:0071383	2.92E-02	8.45E-02	Pparg,19016:up|Akr1c18,105349:up|Egfr,13649:down|Sfrp1,20377:up|Igf1,16000:up|Bmp4,12159:up|Vdr,22337:down|Mmp2,17390:down
regulation of steroid hormone biosynthetic process	GO:0090030	2.93E-02	8.45E-02	Igf2,16002:up|Igf1,16000:up
positive regulation of cofactor metabolic process	GO:0051194	2.93E-02	8.45E-02	Htr2a,15558:down|Igf1,16000:up
establishment or maintenance of monopolar cell polarity	GO:0061339	2.93E-02	8.45E-02	Ptk7,71461:down|Wnt5a,22418:down
lateral mesoderm development	GO:0048368	2.93E-02	8.45E-02	Fgfr1,14182:down|Bmpr1a,12166:down
positive regulation of coenzyme metabolic process	GO:0051197	2.93E-02	8.45E-02	Htr2a,15558:down|Igf1,16000:up
response to stilbenoid	GO:0035634	2.93E-02	8.45E-02	Ifit3,15959:up|Saa3,20210:up
negative regulation of platelet activation	GO:0010544	2.93E-02	8.45E-02	Ceacam1,26365:up|Serpine2,20720:up
positive regulation of cellular amine metabolic process	GO:0033240	2.93E-02	8.45E-02	Maob,109731:up|Epas1,13819:up
positive regulation of macrophage activation	GO:0043032	2.93E-02	8.45E-02	Wnt5a,22418:down|Il1rl1,17082:down
negative regulation of amino acid transport	GO:0051956	2.93E-02	8.45E-02	Tnf,21926:up|Il1rn,16181:up
forebrain morphogenesis	GO:0048853	2.93E-02	8.45E-02	Uchl5,56207:up|Wnt5a,22418:down

leukocyte migration involved in inflammatory response	GO:0002523	2.93E-02	8.45E-02	Cd24a,12484:down|Ccl2,20296:up
immune response to tumor cell	GO:0002418	2.93E-02	8.45E-02	Ceacam1,26365:up|Cd24a,12484:down
regulation of NFAT protein import into nucleus	GO:0051532	2.93E-02	8.45E-02	Ceacam1,26365:up|Tnf,21926:up
positive regulation of extrinsic apoptotic signaling pathway in absence of ligand	GO:2001241	2.93E-02	8.45E-02	Tgfb2,21808:down|Inhba,16323:down
positive regulation of ion transmembrane transport	GO:0034767	2.98E-02	8.58E-02	Fxyd1,56188:up|Cxcl12,20315:up|Ccl12,20293:up|Stc1,20855:up|S100a1,20193:up|Bmp4,12159:up
cellular response to molecule of bacterial origin	GO:0071219	2.98E-02	8.58E-02	Ankrd1,107765:down|Tnf,21926:up|Mrc1,17533:up|Wnt5a,22418:down|Ccl2,20296:up|Ccl12,20293:up
diencephalon development	GO:0021536	2.99E-02	8.62E-02	Ncam1,17967:down|Wnt5a,22418:down|Bmpr1a,12166:down|Bmp4,12159:up
isoprenoid metabolic process	GO:0006720	2.99E-02	8.62E-02	Crabp2,12904:down|Akr1c18,105349:up|Aldh1a2,19378:up|Aldh1a3,56847:down
regulation of viral process	GO:0050792	3.00E-02	8.65E-02	Tnf,21926:up|Lmcd1,30937:up|Ptx3,19288:up|Slpi,20568:up|Ccl4,20303:up|Ceacam1,26365:up|Oasl1,231655:up|Ifit1,15957:up|Oas3,246727:up
embryonic placenta development	GO:0001892	3.01E-02	8.65E-02	Adm,11535:up|Egfr,13649:down|Gjb5,14622:down|Fgfr2,14183:up|Epas1,13819:up
negative regulation of endopeptidase activity	GO:0010951	3.01E-02	8.65E-02	Gas6,14456:up|Sfrp2,20319:up|Vegfa,22339:down|Slpi,20568:up|Igf1,16000:up
positive regulation of cell morphogenesis involved in differentiation	GO:0010770	3.05E-02	8.76E-02	Tnfrsf12a,27279:down|Crabp2,12904:down|Has2,15117:down|Cxcl12,20315:up|Vegfa,22339:down|Tgfb2,21808:down|Sema7a,20361:down
regulation of synapse structure or activity	GO:0050803	3.07E-02	8.82E-02	Plk2,20620:down|Flrt2,399558:down|Ncam1,17967:down|Wnt5a,22418:down|Ptgs2,19225:down|Srpx2,68792:down|Serpine2,20720:up|Cx3cr1,13051:down|Thbs2,21826:down
regulation of interleukin-1 beta production	GO:0032651	3.10E-02	8.87E-02	Wnt5a,22418:down|Errfi1,74155:down|Ccl3,20302:up
hindlimb morphogenesis	GO:0035137	3.10E-02	8.87E-02	Bmpr1a,12166:down|Rspo2,239405:up|Bmp4,12159:up
positive regulation of oxidoreductase activity	GO:0051353	3.10E-02	8.87E-02	Vdr,22337:down|Htr2b,15559:down|Tnf,21926:up
positive regulation of interleukin-8 production	GO:0032757	3.10E-02	8.87E-02	Tnf,21926:up|Wnt5a,22418:down|Adipoq,11450:up
heart valve development	GO:0003170	3.10E-02	8.87E-02	Tgfb2,21808:down|Bmpr1a,12166:down|Bmp4,12159:up
mechanoreceptor differentiation	GO:0042490	3.14E-02	8.98E-02	Cthrc1,68588:down|Fgfr1,14182:down|Mcoln3,171166:up|Bmp4,12159:up
modification of morphology or physiology of other organism involved in symbiotic interaction	GO:0051817	3.14E-02	8.98E-02	Ceacam1,26365:up|Ptx3,19288:up|Cd4,12504:up|Ccl4,20303:up
cellular aldehyde metabolic process	GO:0006081	3.14E-02	8.98E-02	Akr1c18,105349:up|Pdxk,216134:up|Aldh1a2,19378:up|Aldh1a3,56847:down
regulation of ARF protein signal transduction	GO:0032012	3.14E-02	8.98E-02	Ccl9,20308:up|Ccl6,20305:up|Wnt5a,22418:down|Ccl7,20306:up|Ccl2,20296:up|Ccl12,20293:up|Ccl4,20303:up|Ccl3,20302:up|Ccl8,20307:up
ARF protein signal transduction	GO:0032011	3.14E-02	8.98E-02	Ccl9,20308:up|Ccl6,20305:up|Wnt5a,22418:down|Ccl7,20306:up|Ccl2,20296:up|Ccl12,20293:up|Ccl4,20303:up|Ccl3,20302:up|Ccl8,20307:up
liver development	GO:0001889	3.25E-02	9.20E-02	Ceacam1,26365:up|Hp,15439:up|Hmox1,15368:up|Tgfbr3,21814:up|Bmp4,12159:up
lymphocyte proliferation	GO:0046651	3.29E-02	9.20E-02	Cd24a,12484:down|Cd4,12504:up|Tfrc,22042:up|Igf1,16000:up|Bmp4,12159:up|Ceacam1,26365:up|Cxcl12,20315:up|Igf2,16002:up|Tnfsf9,21950:up
positive regulation of interleukin-6 production	GO:0032755	3.30E-02	9.20E-02	Tnf,21926:up|Wnt5a,22418:down|Figf,14205:up|Tnfsf9,21950:up
positive regulation of production of molecular mediator of immune response	GO:0002702	3.30E-02	9.20E-02	Ceacam1,26365:up|Wnt5a,22418:down|Tfrc,22042:up|Sema7a,20361:down
muscle cell apoptotic process	GO:0010657	3.30E-02	9.20E-02	Sfrp2,20319:up|Hmox1,15368:up|Igfbp3,16009:up|Igf1,16000:up
positive regulation of interleukin-1 secretion	GO:0050716	3.31E-02	9.20E-02	Wnt5a,22418:down|Ccl3,20302:up
positive regulation of nucleotide catabolic process	GO:0030813	3.31E-02	9.20E-02	Htr2a,15558:down|Igf1,16000:up
mesenchymal to epithelial transition	GO:0060231	3.31E-02	9.20E-02	Grem1,23892:up|Bmp4,12159:up

regulation of thymocyte apoptotic process	GO:0070243	3.31E-02	9.20E-02	Wnt5a,22418:down|Bmp4,12159:up
spongiotrophoblast layer development	GO:0060712	3.31E-02	9.20E-02	Adm,11535:up|Gjb5,14622:down
negative regulation of bone mineralization	GO:0030502	3.31E-02	9.20E-02	Fam101b,76566:down|Grem1,23892:up
regulation of transcription involved in cell fate commitment	GO:0060850	3.31E-02	9.20E-02	Pparg,19016:up|Bmp4,12159:up
NFAT protein import into nucleus	GO:0051531	3.31E-02	9.20E-02	Ceacam1,26365:up|Tnf,21926:up
positive regulation of interleukin-1 beta secretion	GO:0050718	3.31E-02	9.20E-02	Wnt5a,22418:down|Ccl3,20302:up
regulation of calcineurin-NFAT signaling cascade	GO:0070884	3.31E-02	9.20E-02	Lmcd1,30937:up|Igf1,16000:up
growth plate cartilage development	GO:0003417	3.31E-02	9.20E-02	Stc1,20855:up|Mmp13,17386:up
regulation of fibroblast apoptotic process	GO:2000269	3.31E-02	9.20E-02	Gas6,14456:up|Sfrp1,20377:up
regulation of immunoglobulin secretion	GO:0051023	3.31E-02	9.20E-02	Ceacam1,26365:up|Tnf,21926:up
hydrogen peroxide catabolic process	GO:0042744	3.31E-02	9.20E-02	Pxdn,69675:down|Hp,15439:up
type B pancreatic cell development	GO:0003323	3.31E-02	9.20E-02	Wnt5a,22418:down|Bmp4,12159:up
sulfur compound catabolic process	GO:0044273	3.31E-02	9.20E-02	Hpse,15442:up|Cdo1,12583:up
negative regulation of epithelial cell apoptotic process	GO:1904036	3.31E-02	9.20E-02	Gas6,14456:up|Hmox1,15368:up|Igf1,16000:up
keratinocyte proliferation	GO:0043616	3.31E-02	9.20E-02	Vdr,22337:down|Has2,15117:down|Cd109,235505:down
phenol-containing compound biosynthetic process	GO:0046189	3.31E-02	9.20E-02	Wnt5a,22418:down|Tgfb2,21808:down|Epas1,13819:up
positive regulation of small GTPase mediated signal transduction	GO:0051057	3.31E-02	9.20E-02	Col3a1,12825:down|Kitl,17311:up|Igf1,16000:up
regulation of neuron death	GO:1901214	3.34E-02	9.20E-02	Tnf,21926:up|Glp1r,14652:down|Hmox1,15368:up|Ccl12,20293:up|Ccl3,20302:up|Igf1,16000:up|Crlf1,12931:down|Vegfa,22339:down|Tgfb2,21808:down|Mt1,17748:up
positive regulation of ion transport	GO:0043270	3.36E-02	9.20E-02	Fxyd1,56188:up|Ccl12,20293:up|Cd4,12504:up|Stc1,20855:up|Ccl4,20303:up|Bmp4,12159:up|Fam132b,227358:down|Cxcl12,20315:up|S100a1,20193:up
nephron epithelium development	GO:0072009	3.37E-02	9.20E-02	Vegfa,22339:down|Npnt,114249:up|Grem1,23892:up|Bmp4,12159:up|Adipoq,11450:up
regulation of JAK-STAT cascade	GO:0046425	3.38E-02	9.20E-02	Flrt2,399558:down|Crlf1,12931:down|Ret,19713:up|Lrrc15,74488:down|Aspn,66695:down|Igf1,16000:up
cellular modified amino acid metabolic process	GO:0006575	3.41E-02	9.20E-02	P4ha1,18451:down|Ggt5,23887:up|Gstm1,14862:up|Ddah1,69219:down|Gstt1,14871:up|Gatm,67092:down|Serinc2,230779:down
regulation of cysteine-type endopeptidase activity involved in apoptotic process	GO:0043281	3.41E-02	9.20E-02	Tnf,21926:up|Pparg,19016:up|Gas6,14456:up|Sfrp2,20319:up|Vegfa,22339:down|F3,14066:up|Igf1,16000:up
mononuclear cell proliferation	GO:0032943	3.44E-02	9.20E-02	Cd24a,12484:down|Cd4,12504:up|Tfrc,22042:up|Igf1,16000:up|Bmp4,12159:up|Ceacam1,26365:up|Cxcl12,20315:up|Igf2,16002:up|Tnfsf9,21950:up
regulation of receptor-mediated endocytosis	GO:0048259	3.45E-02	9.20E-02	Trf,22041:up|Vegfa,22339:down|Sfrp4,20379:up|Grem1,23892:up
multi-multicellular organism process	GO:0044706	3.49E-02	9.20E-02	Vdr,22337:down|Akr1c18,105349:up|Hpgd,15446:up|Ptgs2,19225:down|Mmp2,17390:down|Serpine2,20720:up
keratinocyte differentiation	GO:0030216	3.50E-02	9.20E-02	Vdr,22337:down|Tgfb2,21808:down|Ptgs2,19225:down|Cd109,235505:down|Errfi1,74155:down
erythrocyte differentiation	GO:0030218	3.50E-02	9.20E-02	Vegfa,22339:down|Inhba,16323:down|Tgfbr3,21814:up|Epas1,13819:up|Bmp4,12159:up
cardiac muscle cell differentiation	GO:0055007	3.50E-02	9.20E-02	Vegfa,22339:down|Wisp1,22402:down|Grem1,23892:up|Igf1,16000:up|Bmp4,12159:up
detection of abiotic stimulus	GO:0009582	3.50E-02	9.20E-02	Tnf,21926:up|Col11a1,12814:down|Htr2a,15558:down|Serpine2,20720:up|Igf1,16000:up

regulation of biosynthetic process	GO:0009889	3.51E-02	9.20E-02	Fstl3,83554:down|Adm,11535:up|Tfec,21426:up|Sfrp4,20379:up|C3,12266:up|Ankrd1,107765:down|Lyl1,17095:up|Loxl2,94352:down|Loxl3,16950:down|Trf,22041:up|Wnt5a,22418:down|Adipoq,11450:up|Bmp4,12159:up|Igf1,16000:up|Tgfb2,21808:down|Ptgir,19222:up|Fgfr2,14183:up|Htr2b,15559:down|Prl2c2,18811:up|Efemp1,216616:up|Vdr,22337:down|Fabp4,11770:up|Htr2a,15558:down|Slc40a1,53945:up|Gas6,14456:up|Egfr,13649:down|Sfrp1,20377:up|C1qtnf3,81799:down|Tgfbr3,21814:up|Ceacam1,26365:up|Sfrp2,20319:up|Npnt,114249:up|S100a1,20193:up|Ice1,218333:down|Ccl2,20296:up|Ednrb,13618:up|Pdgfc,54635:down|Epas1,13819:up|Uchl5,56207:up|Htra3,78558:down|Ddah1,69219:down|Sdpr,20324:down|Ptx3,19288:up|Akr1c18,105349:up|Bmpr1a,12166:down|Ccl4,20303:up|Ret,19713:up|Prg4,96875:down|Plpp3,67916:up|Creb3l1,26427:down|Kitl,17311:up|Rasl11a,68895:up|Inhba,16323:down|Bnc2,242509:down|Ccl3,20302:up|Vegfa,22339:down|Igf2,16002:up|Tnf,21926:up|Pparg,19016:up|Lmcd1,30937:up|Glp1r,14652:down|Fgfr1,14182:down|Hmox1,15368:up|Has2,15117:down|Sulf1,240725:down|Ptgs2,19225:down|Grem1,23892:up|Phf11d,219132:up|Tbx15,21384:down|Enpp1,18605:down|Errfi1,74155:down
forelimb morphogenesis	GO:0035136	3.53E-02	9.20E-02	Crabp2,12904:down|Rspo2,239405:up|Aldh1a2,19378:up
negative regulation of osteoblast differentiation	GO:0045668	3.53E-02	9.20E-02	Tnf,21926:up|Fgfr1,14182:down|Sfrp1,20377:up
tumor necrosis factor-mediated signaling pathway	GO:0033209	3.53E-02	9.20E-02	Tnf,21926:up|Gas6,14456:up|Adipoq,11450:up
endothelial cell apoptotic process	GO:0072577	3.53E-02	9.20E-02	Gas6,14456:up|Akr1c18,105349:up|Bmp4,12159:up
cellular response to retinoic acid	GO:0071300	3.53E-02	9.20E-02	Ret,19713:up|Ptk7,71461:down|Aldh1a2,19378:up
positive regulation of nucleic acid-templated transcription	GO:1903508	3.55E-02	9.20E-02	Fstl3,83554:down|Ice1,218333:down|Epas1,13819:up|Lyl1,17095:up|Sdpr,20324:down|Trf,22041:up|Wnt5a,22418:down|Bmpr1a,12166:down|Bmp4,12159:up|Igf1,16000:up|Ret,19713:up|Plpp3,67916:up|Creb3l1,26427:down|Fgfr2,14183:up|Prl2c2,18811:up|Rasl11a,68895:up|Inhba,16323:down|Ccl3,20302:up|Vdr,22337:down|Vegfa,22339:down|Igf2,16002:up|Slc40a1,53945:up|Tnf,21926:up|Pparg,19016:up|Glp1r,14652:down|Egfr,13649:down|Fgfr1,14182:down|Sfrp1,20377:up|Tgfbr3,21814:up|Grem1,23892:up|Sfrp2,20319:up|Npnt,114249:up
positive regulation of transcription, DNA-templated	GO:0045893	3.55E-02	9.20E-02	Fstl3,83554:down|Ice1,218333:down|Epas1,13819:up|Lyl1,17095:up|Sdpr,20324:down|Trf,22041:up|Wnt5a,22418:down|Bmpr1a,12166:down|Bmp4,12159:up|Igf1,16000:up|Ret,19713:up|Plpp3,67916:up|Creb3l1,26427:down|Fgfr2,14183:up|Prl2c2,18811:up|Rasl11a,68895:up|Inhba,16323:down|Ccl3,20302:up|Vdr,22337:down|Vegfa,22339:down|Igf2,16002:up|Slc40a1,53945:up|Tnf,21926:up|Pparg,19016:up|Glp1r,14652:down|Egfr,13649:down|Fgfr1,14182:down|Sfrp1,20377:up|Tgfbr3,21814:up|Grem1,23892:up|Sfrp2,20319:up|Npnt,114249:up
epithelial cell development	GO:0002064	3.56E-02	9.20E-02	Tnf,21926:up|Fgfr1,14182:down|Wnt5a,22418:down|Stc1,20855:up|Grem1,23892:up|Bmp4,12159:up|Adipoq,11450:up|Fgfr2,14183:up
chemoattraction of axon	GO:0061642	3.58E-02	9.20E-02	Wnt5a,22418:down
halogenated hydrocarbon metabolic process	GO:0042197	3.58E-02	9.20E-02	Gstt1,14871:up
positive regulation of protein activation cascade	GO:2000259	3.58E-02	9.20E-02	C3,12266:up
negative regulation of natural killer cell mediated cytotoxicity directed against tumor cell target	GO:0002859	3.58E-02	9.20E-02	Ceacam1,26365:up
modulation by host of viral catalytic activity	GO:0044867	3.58E-02	9.20E-02	Ptx3,19288:up
negative regulation by host of viral exo-alpha-sialidase activity	GO:0044869	3.58E-02	9.20E-02	Ptx3,19288:up
regulation of sodium-dependent phosphate transport	GO:2000118	3.58E-02	9.20E-02	Sfrp4,20379:up
cloaca development	GO:0035844	3.58E-02	9.20E-02	Bmp4,12159:up
pyridoxal phosphate metabolic process	GO:0042822	3.58E-02	9.20E-02	Pdxk,216134:up
mesenchymal cell differentiation involved in lung development	GO:0060915	3.58E-02	9.20E-02	Fgfr2,14183:up
monocyte homeostasis	GO:0035702	3.58E-02	9.20E-02	Ccl2,20296:up
negative regulation of dendritic cell apoptotic process	GO:2000669	3.58E-02	9.20E-02	Gas6,14456:up
cellular response to jasmonic acid stimulus	GO:0071395	3.58E-02	9.20E-02	Akr1c18,105349:up
erythrocyte aggregation	GO:0034117	3.58E-02	9.20E-02	Cd24a,12484:down
sorbitol metabolic process	GO:0006060	3.58E-02	9.20E-02	Sord,20322:up
positive regulation of blood microparticle formation	GO:2000334	3.58E-02	9.20E-02	Tnf,21926:up

aorta smooth muscle tissue morphogenesis	GO:0060414	3.58E-02	9.20E-02	Col3a1,12825:down
negative regulation of immune response to tumor cell	GO:0002838	3.58E-02	9.20E-02	Ceacam1,26365:up
creatine biosynthetic process	GO:0006601	3.58E-02	9.20E-02	Gatm,67092:down
blood microparticle formation	GO:0072564	3.58E-02	9.20E-02	Tnf,21926:up
negative regulation of bone trabecula formation	GO:1900155	3.58E-02	9.20E-02	Grem1,23892:up
hypophysis morphogenesis	GO:0048850	3.58E-02	9.20E-02	Wnt5a,22418:down
negative regulation of chondrocyte development	GO:0061182	3.58E-02	9.20E-02	Fam101b,76566:down
positive regulation of T cell mediated immune response to tumor cell	GO:0002842	3.58E-02	9.20E-02	Cd24a,12484:down
positive regulation of fibroblast growth factor production	GO:0090271	3.58E-02	9.20E-02	Ptgs2,19225:down
pyrimidine nucleoside transport	GO:0015864	3.58E-02	9.20E-02	Slc29a1,63959:up
renal protein absorption	GO:0097017	3.58E-02	9.20E-02	Adipoq,11450:up
regulation of exo-alpha-sialidase activity	GO:1903015	3.58E-02	9.20E-02	Ptx3,19288:up
mating plug formation	GO:0042628	3.58E-02	9.20E-02	Serpine2,20720:up
vitamin B6 metabolic process	GO:0042816	3.58E-02	9.20E-02	Pdxk,216134:up
regulation of transcription from RNA polymerase II promoter involved in kidney development	GO:0060994	3.58E-02	9.20E-02	Bmp4,12159:up
modulation by host of viral exo-alpha-sialidase activity	GO:0044866	3.58E-02	9.20E-02	Ptx3,19288:up
regulation of mesenchymal stem cell proliferation	GO:1902460	3.58E-02	9.20E-02	Bmp4,12159:up
cellular response to cisplatin	GO:0072719	3.58E-02	9.20E-02	Hmox1,15368:up
lateral sprouting involved in mammary gland duct morphogenesis	GO:0060599	3.58E-02	9.20E-02	Wnt5a,22418:down
canonical Wnt signaling pathway involved in positive regulation of wound healing	GO:0044330	3.58E-02	9.20E-02	Plpp3,67916:up
post-mating behavior	GO:0045297	3.58E-02	9.20E-02	Serpine2,20720:up
canonical Wnt signaling pathway involved in regulation of type B pancreatic cell proliferation	GO:0044343	3.58E-02	9.20E-02	Sfrp1,20377:up
mesodermal to mesenchymal transition involved in gastrulation	GO:0060809	3.58E-02	9.20E-02	Wnt5a,22418:down
negative regulation of secondary metabolite biosynthetic process	GO:1900377	3.58E-02	9.20E-02	Wnt5a,22418:down
activation of plasma proteins involved in acute inflammatory response	GO:0002541	3.58E-02	9.20E-02	F3,14066:up
cloacal septation	GO:0060197	3.58E-02	9.20E-02	Bmp4,12159:up
negative regulation of L-glutamate transport	GO:0002037	3.58E-02	9.20E-02	Tnf,21926:up
cervix development	GO:0060067	3.58E-02	9.20E-02	Wnt5a,22418:down

BMP signaling pathway involved in heart induction	GO:0003130	3.58E-02	9.20E-02	Bmp4,12159:up
metanephric glomerular visceral epithelial cell differentiation	GO:0072248	3.58E-02	9.20E-02	Adipoq,11450:up
response to cisplatin	GO:0072718	3.58E-02	9.20E-02	Hmox1,15368:up
positive regulation of adiponectin secretion	GO:0070165	3.58E-02	9.20E-02	C1qtnf3,81799:down
endodermal-mesodermal cell signaling	GO:0003133	3.58E-02	9.20E-02	Bmp4,12159:up
metanephric glomerular epithelial cell development	GO:0072313	3.58E-02	9.20E-02	Adipoq,11450:up
regulation of hydrogen peroxide catabolic process	GO:2000295	3.58E-02	9.20E-02	Hp,15439:up
endocardial cushion fusion	GO:0003274	3.58E-02	9.20E-02	Tgfb2,21808:down
regulation of bone trabecula formation	GO:1900154	3.58E-02	9.20E-02	Grem1,23892:up
granulocyte colony-stimulating factor signaling pathway	GO:0038158	3.58E-02	9.20E-02	Ceacam1,26365:up
bile acid signaling pathway	GO:0038183	3.58E-02	9.20E-02	Vdr,22337:down
planar cell polarity pathway involved in pericardium morphogenesis	GO:0061354	3.58E-02	9.20E-02	Wnt5a,22418:down
positive regulation of translational initiation by iron	GO:0045994	3.58E-02	9.20E-02	Tnf,21926:up
response to lead ion	GO:0010288	3.58E-02	9.20E-02	Ncam1,17967:down
neural crest cell fate commitment	GO:0014034	3.58E-02	9.20E-02	Sfrp1,20377:up
fibroblast growth factor receptor signaling pathway involved in mammary gland specification	GO:0060595	3.58E-02	9.20E-02	Fgfr2,14183:up
cellular response to UV-A	GO:0071492	3.58E-02	9.20E-02	Mme,17380:up
regulation of pancreatic stellate cell proliferation	GO:2000229	3.58E-02	9.20E-02	Pparg,19016:up
immune complex clearance by monocytes and macrophages	GO:0002436	3.58E-02	9.20E-02	Ccl2,20296:up
response to jasmonic acid	GO:0009753	3.58E-02	9.20E-02	Akr1c18,105349:up
negative regulation of interferon-gamma-mediated signaling pathway	GO:0060336	3.58E-02	9.20E-02	Pparg,19016:up
metanephric glomerular visceral epithelial cell development	GO:0072249	3.58E-02	9.20E-02	Adipoq,11450:up
alditol catabolic process	GO:0019405	3.58E-02	9.20E-02	Sord,20322:up
positive regulation of immune complex clearance by monocytes and macrophages	GO:0090265	3.58E-02	9.20E-02	Ccl2,20296:up
regulation of blood microparticle formation	GO:2000332	3.58E-02	9.20E-02	Tnf,21926:up
regulation of T cell mediated immune response to tumor cell	GO:0002840	3.58E-02	9.20E-02	Cd24a,12484:down
T cell mediated immune response to tumor cell	GO:0002424	3.58E-02	9.20E-02	Cd24a,12484:down

positive regulation of dopamine biosynthetic process	GO:1903181	3.58E-02	9.20E-02	Epas1,13819:up
chlorinated hydrocarbon metabolic process	GO:0042196	3.58E-02	9.20E-02	Gstt1,14871:up
regulation of erythrocyte aggregation	GO:0034118	3.58E-02	9.20E-02	Cd24a,12484:down
vacuolar phosphate transport	GO:0007037	3.58E-02	9.20E-02	Fgfr1,14182:down
negative regulation of melanin biosynthetic process	GO:0048022	3.58E-02	9.20E-02	Wnt5a,22418:down
planar cell polarity pathway involved in gastrula mediolateral intercalation	GO:0060775	3.58E-02	9.20E-02	Wnt5a,22418:down
pancreatic stellate cell proliferation	GO:0072343	3.58E-02	9.20E-02	Pparg,19016:up
negative regulation by host of symbiont catalytic activity	GO:0052403	3.58E-02	9.20E-02	Ptx3,19288:up
chronic inflammatory response to non-antigenic stimulus	GO:0002545	3.58E-02	9.20E-02	Cx3cr1,13051:down
helper T cell enhancement of adaptive immune response	GO:0035397	3.58E-02	9.20E-02	Cd4,12504:up
negative regulation of platelet-derived growth factor receptor-alpha signaling pathway	GO:2000584	3.58E-02	9.20E-02	Adipoq,11450:up
positive regulation of platelet-derived growth factor receptor signaling pathway	GO:0010641	3.58E-02	9.20E-02	F3,14066:up
sesquiterpenoid catabolic process	GO:0016107	3.58E-02	9.20E-02	Akr1c18,105349:up
regulation of platelet-derived growth factor receptor-alpha signaling pathway	GO:2000583	3.58E-02	9.20E-02	Adipoq,11450:up
negative regulation of exo-alpha-sialidase activity	GO:1903016	3.58E-02	9.20E-02	Ptx3,19288:up
seminal vesicle development	GO:0061107	3.58E-02	9.20E-02	Serpine2,20720:up
helper T cell extravasation	GO:0035684	3.58E-02	9.20E-02	Ccl2,20296:up
negative regulation of complement activation, lectin pathway	GO:0001869	3.58E-02	9.20E-02	Serping1,12258:up
farnesol catabolic process	GO:0016488	3.58E-02	9.20E-02	Akr1c18,105349:up
negative regulation of natural killer cell mediated immune response to tumor cell	GO:0002856	3.58E-02	9.20E-02	Ceacam1,26365:up
regulation of mitotic cell cycle DNA replication	GO:1903463	3.58E-02	9.20E-02	Fgfr1,14182:down
L-cysteine catabolic process to taurine	GO:0019452	3.58E-02	9.20E-02	Cdo1,12583:up
positive regulation of protein kinase C activity	GO:1900020	3.58E-02	9.20E-02	Wnt5a,22418:down
intermediate mesoderm development	GO:0048389	3.58E-02	9.20E-02	Bmp4,12159:up
sesquiterpenoid metabolic process	GO:0006714	3.58E-02	9.20E-02	Akr1c18,105349:up
negative regulation of interleukin-1-mediated signaling pathway	GO:2000660	3.58E-02	9.20E-02	Il1rn,16181:up

negative regulation of mast cell cytokine production	GO:0032764	3.58E-02	9.20E-02	Hmox1,15368:up
regulation of canonical Wnt signaling pathway involved in controlling type B pancreatic cell proliferation	GO:2000079	3.58E-02	9.20E-02	Sfrp1,20377:up
negative regulation of lymphocyte chemotaxis	GO:1901624	3.58E-02	9.20E-02	Ccl12,20293:up
regulation of protein kinase C activity	GO:1900019	3.58E-02	9.20E-02	Wnt5a,22418:down
endodermal-mesodermal cell signaling involved in heart induction	GO:0003134	3.58E-02	9.20E-02	Bmp4,12159:up
negative regulation of catalytic activity in other organism involved in symbiotic interaction	GO:0052199	3.58E-02	9.20E-02	Ptx3,19288:up
divalent metal ion export	GO:0070839	3.58E-02	9.20E-02	Slc40a1,53945:up
regulation of sphingolipid mediated signaling pathway	GO:1902068	3.58E-02	9.20E-02	Plpp3,67916:up
farnesol metabolic process	GO:0016487	3.58E-02	9.20E-02	Akr1c18,105349:up
negative regulation of response to interferon-gamma	GO:0060331	3.58E-02	9.20E-02	Pparg,19016:up
positive regulation of complement activation	GO:0045917	3.58E-02	9.20E-02	C3,12266:up
positive regulation of CD4-positive, alpha-beta T cell proliferation	GO:2000563	3.58E-02	9.20E-02	Ceacam1,26365:up
esophagus smooth muscle contraction	GO:0014846	3.58E-02	9.20E-02	Sulf1,240725:down
mediolateral intercalation	GO:0060031	3.58E-02	9.20E-02	Wnt5a,22418:down
regulation of immune complex clearance by monocytes and macrophages	GO:0090264	3.58E-02	9.20E-02	Ccl2,20296:up
pattern specification involved in mesonephros development	GO:0061227	3.58E-02	9.20E-02	Bmp4,12159:up
planar cell polarity pathway involved in outflow tract morphogenesis	GO:0061347	3.58E-02	9.20E-02	Wnt5a,22418:down
metanephric S-shaped body morphogenesis	GO:0072284	3.58E-02	9.20E-02	Bmp4,12159:up
planar cell polarity pathway involved in cardiac muscle tissue morphogenesis	GO:0061350	3.58E-02	9.20E-02	Wnt5a,22418:down
heme oxidation	GO:0006788	3.58E-02	9.20E-02	Hmox1,15368:up
metanephric glomerular epithelial cell differentiation	GO:0072312	3.58E-02	9.20E-02	Adipoq,11450:up
squamous basal epithelial stem cell differentiation involved in prostate gland acinus development	GO:0060529	3.58E-02	9.20E-02	Fgfr2,14183:up
negative regulation of tooth mineralization	GO:0070171	3.58E-02	9.20E-02	Aspn,66695:down
branch elongation involved in salivary gland morphogenesis	GO:0060667	3.58E-02	9.20E-02	Fgfr2,14183:up
positive regulation of prolactin secretion	GO:1902722	3.58E-02	9.20E-02	Egfr,13649:down
regulation of exocyst assembly	GO:0001928	3.58E-02	9.20E-02	Ncam1,17967:down
mammary gland bud formation	GO:0060615	3.58E-02	9.20E-02	Fgfr2,14183:up

fructose biosynthetic process	GO:0046370	3.58E-02	9.20E-02	Sord,20322:up
positive regulation of chronic inflammatory response to antigenic stimulus	GO:0002876	3.58E-02	9.20E-02	Tnf,21926:up
heparan sulfate proteoglycan catabolic process	GO:0030200	3.58E-02	9.20E-02	Hpse,15442:up
regulation of complement activation, lectin pathway	GO:0001868	3.58E-02	9.20E-02	Serping1,12258:up
negative regulation by host of viral glycoprotein metabolic process	GO:0044871	3.58E-02	9.20E-02	Ptx3,19288:up
planar cell polarity pathway involved in ventricular septum morphogenesis	GO:0061348	3.58E-02	9.20E-02	Wnt5a,22418:down
positive regulation of serine phosphorylation of STAT3 protein	GO:1903263	3.58E-02	9.20E-02	Ret,19713:up
chemokine (C-C motif) ligand 2 secretion	GO:0035926	3.58E-02	9.20E-02	Postn,50706:down
taurine biosynthetic process	GO:0042412	3.58E-02	9.20E-02	Cdo1,12583:up
regulation of chronic inflammatory response to non-antigenic stimulus	GO:0002880	3.58E-02	9.20E-02	Cx3cr1,13051:down
positive regulation of mesenchymal stem cell proliferation	GO:1902462	3.58E-02	9.20E-02	Bmp4,12159:up
negative regulation of cardiac muscle hypertrophy in response to stress	GO:1903243	3.58E-02	9.20E-02	Errfi1,74155:down
modulation by host of viral glycoprotein metabolic process	GO:0044870	3.58E-02	9.20E-02	Ptx3,19288:up
positive regulation of platelet-derived growth factor production	GO:0090362	3.58E-02	9.20E-02	Ptgs2,19225:down
growth plate cartilage morphogenesis	GO:0003422	3.58E-02	9.20E-02	Stc1,20855:up
negative regulation of response to tumor cell	GO:0002835	3.58E-02	9.20E-02	Ceacam1,26365:up
regulation of dopamine biosynthetic process	GO:1903179	3.58E-02	9.20E-02	Epas1,13819:up
negative regulation of protein import into nucleus, translocation	GO:0033159	3.58E-02	9.20E-02	Gas6,14456:up
positive regulation of parathyroid hormone secretion	GO:2000830	3.58E-02	9.20E-02	Fgfr1,14182:down
regulation of translational initiation by iron	GO:0006447	3.58E-02	9.20E-02	Tnf,21926:up
melanocyte proliferation	GO:0097325	3.58E-02	9.20E-02	Wnt5a,22418:down
pyridoxal phosphate biosynthetic process	GO:0042823	3.58E-02	9.20E-02	Pdxk,216134:up
planar cell polarity pathway involved in cardiac right atrium morphogenesis	GO:0061349	3.58E-02	9.20E-02	Wnt5a,22418:down
anterior/posterior pattern specification involved in kidney development	GO:0072098	3.58E-02	9.20E-02	Bmp4,12159:up
regulation of lymphocyte differentiation	GO:0045619	3.60E-02	9.23E-02	Ctla2a,13024:down|Gas6,14456:up|Cd24a,12484:down|Sfrp1,20377:up|Tnfsf9,21950:up|Bmp4,12159:up
hepaticobiliary system development	GO:0061008	3.63E-02	9.30E-02	Ceacam1,26365:up|Hp,15439:up|Hmox1,15368:up|Tgfbr3,21814:up|Bmp4,12159:up
detection of external stimulus	GO:0009581	3.63E-02	9.30E-02	Tnf,21926:up|Col11a1,12814:down|Htr2a,15558:down|Serpine2,20720:up|Igf1,16000:up

negative regulation of protein serine/threonine kinase activity	GO:0071901	3.63E-02	9.30E-02	Sfrp2,20319:up|Sfrp1,20377:up|Prkar2b,19088:up|Bmp4,12159:up|Adipoq,11450:up
axonogenesis	GO:0007409	3.64E-02	9.33E-02	Flrt2,399558:down|Lama2,16773:down|Plxnd1,67784:up|Wnt5a,22418:down|Sema7a,20361:down|Tnfrsf12a,27279:down|Crabp2,12904:down|Ret,19713:up|Cxcl12,20315:up|Vegfa,22339:down|Tgfb2,21808:down|Fgfr2,14183:up
positive regulation of RNA biosynthetic process	GO:1902680	3.65E-02	9.33E-02	Fstl3,83554:down|Ice1,218333:down|Epas1,13819:up|Lyl1,17095:up|Sdpr,20324:down|Trf,22041:up|Wnt5a,22418:down|Bmpr1a,12166:down|Bmp4,12159:up|Igf1,16000:up|Ret,19713:up|Plpp3,67916:up|Creb3l1,26427:down|Fgfr2,14183:up|Prl2c2,18811:up|Rasl11a,68895:up|Inhba,16323:down|Ccl3,20302:up|Vdr,22337:down|Vegfa,22339:down|Igf2,16002:up|Slc40a1,53945:up|Tnf,21926:up|Pparg,19016:up|Glp1r,14652:down|Egfr,13649:down|Fgfr1,14182:down|Sfrp1,20377:up|Tgfbr3,21814:up|Grem1,23892:up|Sfrp2,20319:up|Npnt,114249:up
cellular metabolic process	GO:0044237	3.70E-02	9.40E-02	Fstl3,83554:down|Igfbp4,16010:up|Adm,11535:up|Flrt2,399558:down|Tfec,21426:up|Sfrp4,20379:up|Lyl1,17095:up|Gstt1,14871:up|Blvrb,233016:up|Igfbp3,16009:up|Plk2,20620:down|Cd24a,12484:down|Mme,17380:up|Fkbp14,231997:down|Il1rn,16181:up|Maob,109731:up|Bmp4,12159:up|Hpse,15442:up|Ptgir,19222:up|Fgfr2,14183:up|Htr2b,15559:down|Dram1,71712:up|Ccl6,20305:up|Slpi,20568:up|Aspn,66695:down|Fam132b,227358:down|Nabp1,109019:down|Oas3,246727:up|Slc40a1,53945:up|Egfr,13649:down|Alpl,11647:up|Tfrc,22042:up|Ccl8,20307:up|Gstm1,14862:up|Serpina3g,20715:up|Fam20a,208659:down|Aldh1a2,19378:up|Ccl2,20296:up|Ednrb,13618:up|Ampd3,11717:up|Mmp13,17386:up|Htra3,78558:down|Uchl5,56207:up|Sord,20322:up|Enpp3,209558:down|Cdo1,12583:up|Dok2,13449:up|Mamdc2,71738:down|Ptx3,19288:up|Ccl4,20303:up|Chst11,58250:down|Vegfb,22340:up|Aldh1a3,56847:down|Plpp3,67916:up|Ccl9,20308:up|Inhba,16323:down|Bnc2,242509:down|Tfpi2,21789:down|Crlf1,12931:down|Plod2,26432:down|Vegfa,22339:down|Gfpt2,14584:down|Cbr2,12409:up|Serpine2,20720:up|Nxn,18230:down|Igf2,16002:up|Tnf,21926:up|Fgfr1,14182:down|Lpl,16956:up|Sulf1,240725:down|Cd4,12504:up|Col3a1,12825:down|Tbx15,21384:down|Serping1,12258:up|Uck2,80914:down|Enpp1,18605:down|Hpgd,15446:up|C3,12266:up|Enpp5,83965:up|Ankrd1,107765:down|Cd109,235505:down|Ggt5,23887:up|Loxl2,94352:down|Trf,22041:up|Loxl3,16950:down|Wnt5a,22418:down|Mark1,226778:up|Adipoq,11450:up|Hal,15109:up|Igf1,16000:up|Crabp2,12904:down|Tgfb2,21808:down|F3,14066:up|Kng1,16644:up|Prl2c2,18811:up|Efemp1,216616:up|Saa3,20210:up|Vdr,22337:down|Pdia5,72599:down|Fabp4,11770:up|Htr2a,15558:down|Gas6,14456:up|Col11a1,12814:down|Sfrp1,20377:up|C1qtnf3,81799:down|Tgfbr3,21814:up|Serinc2,230779:down|Ceacam1,26365:up|Sfrp2,20319:up|Pxdn,69675:down|Tnfrsf11b,18383:up|Prkar2b,19088:up|Npnt,114249:up|S100a1,20193:up|Ice1,218333:down|Neurl3,214854:down|Ccl7,20306:up|Cst7,13011:up|Pdgfc,54635:down|Pdzrn3,55983:down|Epas1,13819:up|Marc1,66112:up|Sema7a,20361:down|Ddah1,69219:down|Fkbp10,14230:down|Sdpr,20324:down|Npl,74091:up|Man2a2,140481:up|Akr1c18,105349:up|Fabp5,16592:up|Ccl12,20293:up|Bmpr1a,12166:down|Gatm,67092:down|Ret,19713:up|Prg4,96875:down|Sdf2l1,64136:up|Pdxk,216134:up|Creb3l1,26427:down|Kitl,17311:up|P4ha1,18451:down|Hp,15439:up|Aoah,27052:up|Rasl11a,68895:up|Ccl3,20302:up|Plat,18791:down|Ctla2a,13024:down|Srpx2,68792:down|Adamts12,239337:down|Lrrc15,74488:down|Anpep,16790:up|Pparg,19016:up|Lmcd1,30937:up|Glp1r,14652:down|Hmox1,15368:up|Has2,15117:down|Ptgs2,19225:down|Grem1,23892:up|Phf11d,219132:up|Errfi1,74155:down
cellular monovalent inorganic anion homeostasis	GO:0030320	3.70E-02	9.40E-02	Stc1,20855:up|Enpp1,18605:down
response to phenylpropanoid	GO:0080184	3.70E-02	9.40E-02	Ifit3,15959:up|Saa3,20210:up
cellular response to monoamine stimulus	GO:0071868	3.70E-02	9.40E-02	Htr2b,15559:down|Adipoq,11450:up
tooth mineralization	GO:0034505	3.70E-02	9.40E-02	Fam20a,208659:down|Aspn,66695:down
cardioblast differentiation	GO:0010002	3.70E-02	9.40E-02	Tgfb2,21808:down|Grem1,23892:up
response to tumor cell	GO:0002347	3.70E-02	9.40E-02	Ceacam1,26365:up|Cd24a,12484:down
cellular response to prostaglandin stimulus	GO:0071379	3.70E-02	9.40E-02	Akr1c18,105349:up|Ptgir,19222:up
positive regulation of hormone metabolic process	GO:0032352	3.70E-02	9.40E-02	Igf2,16002:up|Igf1,16000:up
cellular anion homeostasis	GO:0030002	3.70E-02	9.40E-02	Stc1,20855:up|Enpp1,18605:down
regulation of bone development	GO:1903010	3.70E-02	9.40E-02	Fam101b,76566:down|Grem1,23892:up
cardiac left ventricle morphogenesis	GO:0003214	3.70E-02	9.40E-02	Sfrp2,20319:up|Tgfb2,21808:down
plasminogen activation	GO:0031639	3.70E-02	9.40E-02	Serpine2,20720:up|Plat,18791:down
nitric oxide mediated signal transduction	GO:0007263	3.70E-02	9.40E-02	Mt2,17750:up|Mt1,17748:up
regulation of protein kinase C signaling	GO:0090036	3.70E-02	9.40E-02	Wnt5a,22418:down|Vegfa,22339:down
serotonin receptor signaling pathway	GO:0007210	3.70E-02	9.40E-02	Htr2b,15559:down|Htr2a,15558:down
regulation of granulocyte differentiation	GO:0030852	3.70E-02	9.40E-02	Ceacam1,26365:up|Adipoq,11450:up
modulation by host of viral process	GO:0044788	3.70E-02	9.40E-02	Ceacam1,26365:up|Ptx3,19288:up
renal vesicle morphogenesis	GO:0072077	3.70E-02	9.40E-02	Grem1,23892:up|Bmp4,12159:up

positive regulation of transmembrane transport	GO:0034764	3.71E-02	9.41E-02	Fxyd1,56188:up|Cxcl12,20315:up|Ccl12,20293:up|Stc1,20855:up|S100a1,20193:up|Bmp4,12159:up
Rho protein signal transduction	GO:0007266	3.73E-02	9.45E-02	Col1a2,12843:down|Ccl9,20308:up|Ccl6,20305:up|Wnt5a,22418:down|Ccl7,20306:up|Ccl2,20296:up|Ccl12,20293:up|Ccl4,20303:up|Ccl3,20302:up|Col3a1,12825:down|Ccl8,20307:up
positive regulation of vasoconstriction	GO:0045907	3.76E-02	9.52E-02	Egfr,13649:down|Htr2a,15558:down|Ptgs2,19225:down
cGMP metabolic process	GO:0046068	3.76E-02	9.52E-02	Htr2b,15559:down|Wnt5a,22418:down|Vegfa,22339:down
negative regulation of calcium ion transport	GO:0051926	3.76E-02	9.52E-02	Tgfb2,21808:down|Ptgs2,19225:down|Stc1,20855:up
T cell homeostasis	GO:0043029	3.76E-02	9.52E-02	Ceacam1,26365:up|Cd24a,12484:down|Tgfb2,21808:down
forebrain generation of neurons	GO:0021872	3.78E-02	9.55E-02	Fgfr1,14182:down|Wnt5a,22418:down|Inhba,16323:down|Fgfr2,14183:up
vasodilation	GO:0042311	3.78E-02	9.55E-02	Kng1,16644:up|Adm,11535:up|Egfr,13649:down|Ednrb,13618:up
nephron tubule morphogenesis	GO:0072078	3.78E-02	9.55E-02	Vegfa,22339:down|Npnt,114249:up|Grem1,23892:up|Bmp4,12159:up
response to retinoic acid	GO:0032526	3.78E-02	9.55E-02	Pparg,19016:up|Ret,19713:up|Ptk7,71461:down|Aldh1a2,19378:up
carbohydrate transport	GO:0008643	3.82E-02	9.65E-02	Tnf,21926:up|Fabp5,16592:up|C3,12266:up|Enpp1,18605:down|Igf1,16000:up|Adipoq,11450:up
negative regulation of neurogenesis	GO:0050768	3.92E-02	9.88E-02	Plk2,20620:down|Cd24a,12484:down|Efemp1,216616:up|Wnt5a,22418:down|Bmpr1a,12166:down|Dpysl3,22240:down|Ednrb,13618:up|Col3a1,12825:down|Bmp4,12159:up
organic substance transport	GO:0071702	3.93E-02	9.91E-02	Ice1,218333:down|Ccl2,20296:up|C3,12266:up|Mmp13,17386:up|Slc29a1,63959:up|Ankrd1,107765:down|Gpm6b,14758:up|Clec4n,56620:up|Postn,50706:down|Akr1c18,105349:up|Cd24a,12484:down|Fabp5,16592:up|Wnt5a,22418:down|Bmpr1a,12166:down|Il1rn,16181:up|Maob,109731:up|Adipoq,11450:up|Bmp4,12159:up|Igf1,16000:up|Tgfb2,21808:down|Il1rl1,17082:down|Clec4e,56619:up|Htr2b,15559:down|Inhba,16323:down|Ccl3,20302:up|Fam132b,227358:down|Cxcl12,20315:up|Htr2a,15558:down|Slc16a4,229699:down|Kdelr3,105785:down|Pparg,19016:up|Gas6,14456:up|Tnf,21926:up|Fxyd1,56188:up|Glp1r,14652:down|Egfr,13649:down|Sec23a,20334:down|Lpl,16956:up|Sfrp1,20377:up|C1qtnf3,81799:down|Ptgs2,19225:down|Tfrc,22042:up|Sncg,20618:up|Grem1,23892:up|Serinc2,230779:down|Ceacam1,26365:up|Sfrp2,20319:up|Emb,13723:up|Enpp1,18605:down
secretion by tissue	GO:0032941	3.96E-02	9.97E-02	Vdr,22337:down|Vegfa,22339:down|Mmp13,17386:up|Cdo1,12583:up
lymphocyte activation	GO:0046649	3.99E-02	1.00E-01	Ly9,17085:up|Ccl2,20296:up|Ctla2a,13024:down|Lyl1,17095:up|Cxcl12,20315:up|Igf2,16002:up|Gas6,14456:up|Cd24a,12484:down|Sfrp1,20377:up|Cd4,12504:up|Tfrc,22042:up|Bmp4,12159:up|Igf1,16000:up|Ceacam1,26365:up|Tnfsf9,21950:up|Clec4e,56619:up
regulation of immunoglobulin mediated immune response	GO:0002889	4.00E-02	1.00E-01	Tnf,21926:up|C3,12266:up|Tfrc,22042:up
negative regulation of carbohydrate metabolic process	GO:0045912	4.00E-02	1.00E-01	C1qtnf3,81799:down|Enpp1,18605:down|Adipoq,11450:up
regulation of cell-substrate junction assembly	GO:0090109	4.00E-02	1.00E-01	Gpm6b,14758:up|Vegfa,22339:down|Grem1,23892:up
negative regulation of muscle cell apoptotic process	GO:0010656	4.00E-02	1.00E-01	Sfrp2,20319:up|Hmox1,15368:up|Igf1,16000:up
cellular extravasation	GO:0045123	4.00E-02	1.00E-01	Tnf,21926:up|Cxcl12,20315:up|Ccl2,20296:up
regulation of focal adhesion assembly	GO:0051893	4.00E-02	1.00E-01	Gpm6b,14758:up|Vegfa,22339:down|Grem1,23892:up
regulation of B cell mediated immunity	GO:0002712	4.00E-02	1.00E-01	Tnf,21926:up|C3,12266:up|Tfrc,22042:up
regulation of endopeptidase activity	GO:0052548	4.00E-02	1.01E-01	Tnf,21926:up|Pparg,19016:up|Gas6,14456:up|Slpi,20568:up|Igf1,16000:up|Sfrp2,20319:up|Vegfa,22339:down|F3,14066:up
regulation of nuclear division	GO:0051783	4.06E-02	1.02E-01	Tnf,21926:up|Wnt5a,22418:down|Igf2,16002:up|Fgfr2,14183:up|Igf1,16000:up|Bmp4,12159:up
Rac protein signal transduction	GO:0016601	4.08E-02	1.02E-01	Ccl9,20308:up|Ccl6,20305:up|Wnt5a,22418:down|Ccl7,20306:up|Ccl2,20296:up|Ccl12,20293:up|Ccl4,20303:up|Ccl3,20302:up|Ccl8,20307:up
axon guidance	GO:0007411	4.10E-02	1.02E-01	Flrt2,399558:down|Lama2,16773:down|Plxnd1,67784:up|Cxcl12,20315:up|Wnt5a,22418:down|Vegfa,22339:down|Tgfb2,21808:down
neuron projection guidance	GO:0097485	4.10E-02	1.02E-01	Flrt2,399558:down|Lama2,16773:down|Plxnd1,67784:up|Cxcl12,20315:up|Wnt5a,22418:down|Vegfa,22339:down|Tgfb2,21808:down
endocardial cushion formation	GO:0003272	4.12E-02	1.02E-01	Tgfb2,21808:down|Bmpr1a,12166:down
hair cycle phase	GO:0044851	4.12E-02	1.02E-01	Tgfb2,21808:down|Ptgs2,19225:down
cell aggregation	GO:0098743	4.12E-02	1.02E-01	Col11a1,12814:down|Tgfb2,21808:down
phasic smooth muscle contraction	GO:0014821	4.12E-02	1.02E-01	Htr2b,15559:down|Ednrb,13618:up
cartilage condensation	GO:0001502	4.12E-02	1.02E-01	Col11a1,12814:down|Tgfb2,21808:down
serotonin transport	GO:0006837	4.12E-02	1.02E-01	Gpm6b,14758:up|Maob,109731:up

vitamin biosynthetic process	GO:0009110	4.12E-02	1.02E-01	Vdr,22337:down|Tnf,21926:up
response to monoamine	GO:0071867	4.12E-02	1.02E-01	Htr2b,15559:down|Adipoq,11450:up
renal vesicle development	GO:0072087	4.12E-02	1.02E-01	Grem1,23892:up|Bmp4,12159:up
fibroblast apoptotic process	GO:0044346	4.12E-02	1.02E-01	Gas6,14456:up|Sfrp1,20377:up
negative regulation of epidermis development	GO:0045683	4.12E-02	1.02E-01	Tgfb2,21808:down|Inhba,16323:down
subpallium development	GO:0021544	4.12E-02	1.02E-01	Inhba,16323:down|Aldh1a3,56847:down
regulation of platelet aggregation	GO:0090330	4.12E-02	1.02E-01	Ceacam1,26365:up|Serpine2,20720:up
regulation of receptor biosynthetic process	GO:0010869	4.12E-02	1.02E-01	Pparg,19016:up|Adipoq,11450:up
dorsal/ventral axis specification	GO:0009950	4.12E-02	1.02E-01	Sfrp1,20377:up|Bmpr1a,12166:down
negative regulation of cysteine-type endopeptidase activity involved in apoptotic process	GO:0043154	4.13E-02	1.03E-01	Gas6,14456:up|Sfrp2,20319:up|Vegfa,22339:down|Igf1,16000:up
nephron epithelium morphogenesis	GO:0072088	4.13E-02	1.03E-01	Vegfa,22339:down|Npnt,114249:up|Grem1,23892:up|Bmp4,12159:up
negative regulation of angiogenesis	GO:0016525	4.13E-02	1.03E-01	Sulf1,240725:down|Ccl2,20296:up|Cx3cr1,13051:down|Thbs2,21826:down
somitogenesis	GO:0001756	4.13E-02	1.03E-01	Sfrp2,20319:up|Sfrp1,20377:up|Wnt5a,22418:down|Bmpr1a,12166:down
glucose import	GO:0046323	4.13E-02	1.03E-01	Tnf,21926:up|Enpp1,18605:down|Igf1,16000:up|Adipoq,11450:up
modification of morphology or physiology of other organism	GO:0035821	4.13E-02	1.03E-01	Ceacam1,26365:up|Ptx3,19288:up|Cd4,12504:up|Ccl4,20303:up
lymphocyte homeostasis	GO:0002260	4.13E-02	1.03E-01	Ceacam1,26365:up|Cd24a,12484:down|Tgfb2,21808:down|Slc40a1,53945:up
lymphocyte differentiation	GO:0030098	4.16E-02	1.03E-01	Gas6,14456:up|Cd24a,12484:down|Ly9,17085:up|Sfrp1,20377:up|Cd4,12504:up|Bmp4,12159:up|Ctla2a,13024:down|Lyl1,17095:up|Tnfsf9,21950:up|Clec4e,56619:up
positive regulation of nucleotide metabolic process	GO:0045981	4.18E-02	1.04E-01	Adm,11535:up|Wnt5a,22418:down|Htr2a,15558:down|Ptgir,19222:up|Igf1,16000:up
positive regulation of purine nucleotide metabolic process	GO:1900544	4.18E-02	1.04E-01	Adm,11535:up|Wnt5a,22418:down|Htr2a,15558:down|Ptgir,19222:up|Igf1,16000:up
glycosaminoglycan biosynthetic process	GO:0006024	4.24E-02	1.05E-01	Chst11,58250:down|Has2,15117:down|Igf1,16000:up
positive regulation of mitotic nuclear division	GO:0045840	4.24E-02	1.05E-01	Tnf,21926:up|Igf2,16002:up|Igf1,16000:up
viral entry into host cell	GO:0046718	4.24E-02	1.05E-01	Ceacam1,26365:up|Gas6,14456:up|Ptx3,19288:up
response to fatty acid	GO:0070542	4.24E-02	1.05E-01	Akr1c18,105349:up|Tgfbr3,21814:up|Ptgir,19222:up
inflammatory response to antigenic stimulus	GO:0002437	4.24E-02	1.05E-01	Tnf,21926:up|Cd24a,12484:down|C3,12266:up
ion transmembrane transport	GO:0034220	4.27E-02	1.06E-01	Htr2b,15559:down|Cxcl12,20315:up|Htr2a,15558:down|Slc16a4,229699:down|Slc40a1,53945:up|Gas6,14456:up|Fxyd1,56188:up|Hvcn1,74096:up|Glp1r,14652:down|Ccl12,20293:up|Stc1,20855:up|Fxyd2,11936:up|Kcnj15,16516:down|Bmp4,12159:up|Ank,11732:down|Tgfb2,21808:down|Emb,13723:up|S100a1,20193:up
nephron morphogenesis	GO:0072028	4.31E-02	1.06E-01	Vegfa,22339:down|Npnt,114249:up|Grem1,23892:up|Bmp4,12159:up
positive regulation of carbohydrate metabolic process	GO:0045913	4.31E-02	1.06E-01	Has2,15117:down|Htr2a,15558:down|Igf2,16002:up|Igf1,16000:up
negative regulation of cysteine-type endopeptidase activity	GO:2000117	4.31E-02	1.06E-01	Gas6,14456:up|Sfrp2,20319:up|Vegfa,22339:down|Igf1,16000:up
negative regulation of biosynthetic process	GO:0009890	4.33E-02	1.07E-01	Sfrp4,20379:up|Ednrb,13618:up|Epas1,13819:up|Ankrd1,107765:down|Vdr,22337:down|Fabp4,11770:up|Vegfa,22339:down|Gas6,14456:up|Pparg,19016:up|Tnf,21926:up|Loxl2,94352:down|Loxl3,16950:down|Akr1c18,105349:up|Lmcd1,30937:up|Fgfr1,14182:down|Egfr,13649:down|Hmox1,15368:up|Sfrp1,20377:up|C1qtnf3,81799:down|Wnt5a,22418:down|Ccl4,20303:up|Grem1,23892:up|Adipoq,11450:up|Bmp4,12159:up|Ceacam1,26365:up|Sfrp2,20319:up|Prg4,96875:down|Errfi1,74155:down|Enpp1,18605:down|Fgfr2,14183:up|S100a1,20193:up
negative regulation of gene expression	GO:0010629	4.37E-02	1.08E-01	Sfrp4,20379:up|Ednrb,13618:up|Epas1,13819:up|Ankrd1,107765:down|Vdr,22337:down|Ctla2a,13024:down|Fabp4,11770:up|Vegfa,22339:down|Serpine2,20720:up|Tnf,21926:up|Gas6,14456:up|Pparg,19016:up|Loxl2,94352:down|Loxl3,16950:down|Lmcd1,30937:up|Fgfr1,14182:down|Egfr,13649:down|Hmox1,15368:up|Sfrp1,20377:up|Wnt5a,22418:down|Tgfbr3,21814:up|Grem1,23892:up|Ccl4,20303:up|Adipoq,11450:up|Bmp4,12159:up|Igf1,16000:up|Sfrp2,20319:up|Serping1,12258:up|Tgfb2,21808:down|Fgfr2,14183:up|S100a1,20193:up
positive regulation of JNK cascade	GO:0046330	4.47E-02	1.10E-01	Ceacam1,26365:up|Tnf,21926:up|Trf,22041:up|Wnt5a,22418:down|Il1rn,16181:up

regulation of gliogenesis	GO:0014013	4.47E-02	1.10E-01	Pparg,19016:up|Trf,22041:up|Efemp1,216616:up|Serpine2,20720:up|Bmp4,12159:up
response to toxic substance	GO:0009636	4.47E-02	1.10E-01	Tnf,21926:up|Alpl,11647:up|Ccl4,20303:up|Mt2,17750:up|Mt1,17748:up
positive regulation of glucose transport	GO:0010828	4.49E-02	1.10E-01	C3,12266:up|Igf1,16000:up|Adipoq,11450:up
negative regulation of chemotaxis	GO:0050922	4.49E-02	1.10E-01	Wnt5a,22418:down|Ccl12,20293:up|Grem1,23892:up
regulation of adherens junction organization	GO:1903391	4.49E-02	1.10E-01	Gpm6b,14758:up|Vegfa,22339:down|Grem1,23892:up
regulation of sensory perception of pain	GO:0051930	4.49E-02	1.10E-01	Fgfr1,14182:down|Ncam1,17967:down|Ednrb,13618:up
regulation of sensory perception	GO:0051931	4.49E-02	1.10E-01	Fgfr1,14182:down|Ncam1,17967:down|Ednrb,13618:up
regulation of phosphatidylinositol 3-kinase signaling	GO:0014066	4.49E-02	1.10E-01	Tgfb2,21808:down|Pdgfc,54635:down|Serpine2,20720:up|Igf1,16000:up
positive regulation of lymphocyte mediated immunity	GO:0002708	4.49E-02	1.10E-01	Tnf,21926:up|Cd24a,12484:down|C3,12266:up|Tfrc,22042:up
cell morphogenesis	GO:0000902	4.54E-02	1.11E-01	Fam101b,76566:down|Flrt2,399558:down|Lama2,16773:down|Plxnd1,67784:up|Ccl2,20296:up|Ccl3,20302:up|Sema7a,20361:down|Tnfrsf12a,27279:down|Vdr,22337:down|Cxcl12,20315:up|Postn,50706:down|Vegfa,22339:down|Loxl2,94352:down|Loxl3,16950:down|Egfr,13649:down|Sfrp1,20377:up|Has2,15117:down|Wnt5a,22418:down|Ccl12,20293:up|Tgfbr3,21814:up|Stc1,20855:up|Grem1,23892:up|Bmp4,12159:up|Sfrp2,20319:up|Crabp2,12904:down|Ret,19713:up|Tgfb2,21808:down|Fgfr2,14183:up
negative regulation of glucose import	GO:0046325	4.55E-02	1.11E-01	Tnf,21926:up|Enpp1,18605:down
regulation of androgen receptor signaling pathway	GO:0060765	4.55E-02	1.11E-01	Sfrp1,20377:up|Igf1,16000:up
vascular smooth muscle contraction	GO:0014829	4.55E-02	1.11E-01	Htr2a,15558:down|Ednrb,13618:up
cardiac right ventricle morphogenesis	GO:0003215	4.55E-02	1.11E-01	Tgfb2,21808:down|Bmpr1a,12166:down
regulation of dopamine metabolic process	GO:0042053	4.55E-02	1.11E-01	Maob,109731:up|Epas1,13819:up
positive regulation of transcription from RNA polymerase II promoter in response to stress	GO:0036003	4.55E-02	1.11E-01	Vegfa,22339:down|Creb3l1,26427:down
regulation of interleukin-1 beta secretion	GO:0050706	4.55E-02	1.11E-01	Wnt5a,22418:down|Ccl3,20302:up
cellular response to estradiol stimulus	GO:0071392	4.55E-02	1.11E-01	Egfr,13649:down|Mmp2,17390:down
positive regulation of pri-miRNA transcription from RNA polymerase II promoter	GO:1902895	4.55E-02	1.11E-01	Bmpr1a,12166:down|Bmp4,12159:up
glomerular filtration	GO:0003094	4.55E-02	1.11E-01	Gas6,14456:up|Sulf1,240725:down
positive regulation of alpha-beta T cell proliferation	GO:0046641	4.55E-02	1.11E-01	Ceacam1,26365:up|Cd24a,12484:down
positive regulation of lipid storage	GO:0010884	4.55E-02	1.11E-01	Lpl,16956:up|C3,12266:up
regulation of neuron apoptotic process	GO:0043523	4.59E-02	1.12E-01	Tnf,21926:up|Glp1r,14652:down|Hmox1,15368:up|Ccl12,20293:up|Ccl3,20302:up|Crlf1,12931:down|Tgfb2,21808:down|Mt1,17748:up
positive regulation of immune response	GO:0050778	4.61E-02	1.13E-01	Tnf,21926:up|Cd24a,12484:down|Wnt5a,22418:down|Cd4,12504:up|C3,12266:up|C1rb,667277:up|Tfrc,22042:up|Sema7a,20361:down|Serping1,12258:up|C1s1,50908:up|C1s2,317677:up|Mmp2,17390:down
tissue homeostasis	GO:0001894	4.65E-02	1.13E-01	Ceacam1,26365:up|Trf,22041:up|Egfr,13649:down|Vegfa,22339:down|Tfrc,22042:up|Tnfrsf11b,18383:up|Epas1,13819:up
regulation of cysteine-type endopeptidase activity	GO:2000116	4.65E-02	1.13E-01	Tnf,21926:up|Pparg,19016:up|Gas6,14456:up|Sfrp2,20319:up|Vegfa,22339:down|F3,14066:up|Igf1,16000:up
regulation of Rho protein signal transduction	GO:0035023	4.65E-02	1.13E-01	Ccl9,20308:up|Ccl6,20305:up|Wnt5a,22418:down|Ccl7,20306:up|Ccl2,20296:up|Ccl12,20293:up|Ccl4,20303:up|Ccl3,20302:up|Col3a1,12825:down|Ccl8,20307:up
positive regulation of transcription from RNA polymerase II promoter	GO:0045944	4.66E-02	1.14E-01	Fstl3,83554:down|Prl2c2,18811:up|Inhba,16323:down|Ccl3,20302:up|Epas1,13819:up|Vdr,22337:down|Sdpr,20324:down|Vegfa,22339:down|Igf2,16002:up|Slc40a1,53945:up|Pparg,19016:up|Tnf,21926:up|Glp1r,14652:down|Egfr,13649:down|Fgfr1,14182:down|Wnt5a,22418:down|Bmpr1a,12166:down|Grem1,23892:up|Bmp4,12159:up|Igf1,16000:up|Sfrp2,20319:up|Npnt,114249:up|Creb3l1,26427:down|Fgfr2,14183:up
eye morphogenesis	GO:0048592	4.68E-02	1.14E-01	Col5a2,12832:down|Col5a1,12831:down|Vegfa,22339:down|Aldh1a3,56847:down|Col8a1,12837:down|Bmp4,12159:up
body fluid secretion	GO:0007589	4.68E-02	1.14E-01	Vdr,22337:down|Vegfa,22339:down|Mmp13,17386:up|Cdo1,12583:up

negative regulation of blood vessel morphogenesis	GO:2000181	4.68E-02	1.14E-01	Sulf1,240725:down|Ccl2,20296:up|Cx3cr1,13051:down|Thbs2,21826:down
renal tubule morphogenesis	GO:0061333	4.68E-02	1.14E-01	Vegfa,22339:down|Npnt,114249:up|Grem1,23892:up|Bmp4,12159:up
anion homeostasis	GO:0055081	4.75E-02	1.15E-01	Sfrp4,20379:up|Stc1,20855:up|Enpp1,18605:down
excretion	GO:0007588	4.75E-02	1.15E-01	Stc1,20855:up|Ednrb,13618:up|Anpep,16790:up
neuron projection regeneration	GO:0031102	4.75E-02	1.15E-01	Adm,11535:up|Tnc,21923:down|Mmp2,17390:down
negative regulation of cytokine secretion	GO:0050710	4.75E-02	1.15E-01	Tnf,21926:up|Gas6,14456:up|C1qtnf3,81799:down
regulation of catecholamine secretion	GO:0050433	4.75E-02	1.15E-01	Cxcl12,20315:up|Htr2a,15558:down|Sncg,20618:up
symbiosis, encompassing mutualism through parasitism	GO:0044403	4.76E-02	1.15E-01	Tnf,21926:up|Gas6,14456:up|Lmcd1,30937:up|Ptx3,19288:up|Cd4,12504:up|Slpi,20568:up|Ccl4,20303:up|Ifit3,15959:up|Ceacam1,26365:up|Cxcl9,17329:up|Oasl1,231655:up|Ifit1,15957:up|Lrrc15,74488:down|Oas3,246727:up
interspecies interaction between organisms	GO:0044419	4.76E-02	1.15E-01	Tnf,21926:up|Gas6,14456:up|Lmcd1,30937:up|Ptx3,19288:up|Cd4,12504:up|Slpi,20568:up|Ccl4,20303:up|Ifit3,15959:up|Ceacam1,26365:up|Cxcl9,17329:up|Oasl1,231655:up|Ifit1,15957:up|Lrrc15,74488:down|Oas3,246727:up
interaction with host	GO:0051701	4.88E-02	1.18E-01	Ceacam1,26365:up|Gas6,14456:up|Ptx3,19288:up|Cd4,12504:up
detection of stimulus involved in sensory perception	GO:0050906	4.93E-02	1.20E-01	Tnf,21926:up|Col11a1,12814:down|Htr2a,15558:down|Serpine2,20720:up|Igf1,16000:up
protein transport	GO:0015031	4.94E-02	1.20E-01	Ice1,218333:down|Ccl2,20296:up|Mmp13,17386:up|Ankrd1,107765:down|Gpm6b,14758:up|Clec4n,56620:up|Postn,50706:down|Akr1c18,105349:up|Cd24a,12484:down|Wnt5a,22418:down|Bmpr1a,12166:down|Il1rn,16181:up|Adipoq,11450:up|Bmp4,12159:up|Igf1,16000:up|Tgfb2,21808:down|Il1rl1,17082:down|Clec4e,56619:up|Htr2b,15559:down|Ccl3,20302:up|Kdelr3,105785:down|Tnf,21926:up|Pparg,19016:up|Gas6,14456:up|Glp1r,14652:down|Egfr,13649:down|Sfrp1,20377:up|Lpl,16956:up|Sec23a,20334:down|C1qtnf3,81799:down|Tfrc,22042:up|Ptgs2,19225:down|Sncg,20618:up|Grem1,23892:up|Ceacam1,26365:up|Sfrp2,20319:up
cellular response to type I interferon	GO:0071357	5.00E-02	1.20E-01	Wnt5a,22418:down|Ifit1,15957:up
chondroitin sulfate metabolic process	GO:0030204	5.00E-02	1.20E-01	Mamdc2,71738:down|Chst11,58250:down
regulation of catecholamine metabolic process	GO:0042069	5.00E-02	1.20E-01	Maob,109731:up|Epas1,13819:up
leukocyte tethering or rolling	GO:0050901	5.00E-02	1.20E-01	Tnf,21926:up|Cxcl12,20315:up
regulation of protein import into nucleus, translocation	GO:0033158	5.00E-02	1.20E-01	Gas6,14456:up|Igf1,16000:up
metanephric nephron morphogenesis	GO:0072273	5.00E-02	1.20E-01	Grem1,23892:up|Bmp4,12159:up
hair follicle maturation	GO:0048820	5.00E-02	1.20E-01	Tgfb2,21808:down|Ptgs2,19225:down
atrioventricular valve morphogenesis	GO:0003181	5.00E-02	1.20E-01	Tgfb2,21808:down|Bmpr1a,12166:down
renal sodium excretion	GO:0035812	5.00E-02	1.20E-01	Ednrb,13618:up|Anpep,16790:up
renal filtration	GO:0097205	5.00E-02	1.20E-01	Gas6,14456:up|Sulf1,240725:down
regulation of renal sodium excretion	GO:0035813	5.00E-02	1.20E-01	Ednrb,13618:up|Anpep,16790:up
morphogenesis of an endothelium	GO:0003159	5.00E-02	1.20E-01	Adamts12,239337:down|Bmp4,12159:up
regulation of tumor necrosis factor-mediated signaling pathway	GO:0010803	5.00E-02	1.20E-01	Gas6,14456:up|Adipoq,11450:up
pattern specification process	GO:0007389	5.00E-02	1.20E-01	Fgfr1,14182:down|Plxnd1,67784:up|Sfrp1,20377:up|Wnt5a,22418:down|Bmpr1a,12166:down|Aldh1a2,19378:up|Stc1,20855:up|Grem1,23892:up|Bmp4,12159:up|Sfrp2,20319:up|Cxcl12,20315:up|Vegfa,22339:down|Fgfr2,14183:up
entry into host	GO:0044409	5.02E-02	1.20E-01	Ceacam1,26365:up|Gas6,14456:up|Ptx3,19288:up
negative regulation of tumor necrosis factor production	GO:0032720	5.02E-02	1.20E-01	Gas6,14456:up|Errfi1,74155:down|Adipoq,11450:up
entry into host cell	GO:0030260	5.02E-02	1.20E-01	Ceacam1,26365:up|Gas6,14456:up|Ptx3,19288:up
entry into other organism involved in symbiotic interaction	GO:0051828	5.02E-02	1.20E-01	Ceacam1,26365:up|Gas6,14456:up|Ptx3,19288:up

movement in environment of other organism involved in symbiotic interaction	GO:0052192	5.02E-02	1.20E-01	Ceacam1,26365:up|Gas6,14456:up|Ptx3,19288:up
movement in host environment	GO:0052126	5.02E-02	1.20E-01	Ceacam1,26365:up|Gas6,14456:up|Ptx3,19288:up
regulation of protein tyrosine kinase activity	GO:0061097	5.02E-02	1.20E-01	Gas6,14456:up|Errfi1,74155:down|Grem1,23892:up
entry into cell of other organism involved in symbiotic interaction	GO:0051806	5.02E-02	1.20E-01	Ceacam1,26365:up|Gas6,14456:up|Ptx3,19288:up
leukocyte mediated cytotoxicity	GO:0001909	5.07E-02	1.20E-01	Ceacam1,26365:up|Clec2d,93694:up|Ccl2,20296:up|Igf2,16002:up
regulation of synapse assembly	GO:0051963	5.07E-02	1.20E-01	Srpx2,68792:down|Flrt2,399558:down|Wnt5a,22418:down|Thbs2,21826:down
cellular response to hormone stimulus	GO:0032870	5.08E-02	1.20E-01	Pparg,19016:up|Akr1c18,105349:up|Egfr,13649:down|Sfrp1,20377:up|Inhba,16323:down|Igf1,16000:up|Bmp4,12159:up|Adipoq,11450:up|Vdr,22337:down|Mmp2,17390:down|Ptgir,19222:up|Enpp1,18605:down|Igf2,16002:up
inorganic anion transport	GO:0015698	5.09E-02	1.20E-01	Ank,11732:down|Fxyd1,56188:up|Fgfr1,14182:down|Sfrp4,20379:up|Enpp1,18605:down
synapse assembly	GO:0007416	5.09E-02	1.20E-01	Srpx2,68792:down|Flrt2,399558:down|Plxnd1,67784:up|Wnt5a,22418:down|Thbs2,21826:down
negative regulation of cellular biosynthetic process	GO:0031327	5.12E-02	1.20E-01	Sfrp4,20379:up|Ednrb,13618:up|Epas1,13819:up|Ankrd1,107765:down|Vdr,22337:down|Fabp4,11770:up|Vegfa,22339:down|Gas6,14456:up|Pparg,19016:up|Tnf,21926:up|Loxl2,94352:down|Loxl3,16950:down|Akr1c18,105349:up|Lmcd1,30937:up|Fgfr1,14182:down|Egfr,13649:down|Hmox1,15368:up|Sfrp1,20377:up|C1qtnf3,81799:down|Wnt5a,22418:down|Ccl4,20303:up|Grem1,23892:up|Adipoq,11450:up|Bmp4,12159:up|Sfrp2,20319:up|Prg4,96875:down|Errfi1,74155:down|Enpp1,18605:down|Fgfr2,14183:up|S100a1,20193:up
establishment of localization in cell	GO:0051649	5.13E-02	1.20E-01	Ice1,218333:down|Adm,11535:up|Ccl2,20296:up|Mmp13,17386:up|Ankrd1,107765:down|Gpm6b,14758:up|Clec4n,56620:up|Postn,50706:down|Akr1c18,105349:up|Cd24a,12484:down|Ncam1,17967:down|Wnt5a,22418:down|Bmpr1a,12166:down|Il1rn,16181:up|Maob,109731:up|Adipoq,11450:up|Bmp4,12159:up|Igf1,16000:up|Tgfb2,21808:down|Creb3l1,26427:down|Fgfr2,14183:up|Il1rl1,17082:down|Clec4e,56619:up|Htr2b,15559:down|Inhba,16323:down|Ccl3,20302:up|Cxcl12,20315:up|Htr2a,15558:down|Serpine2,20720:up|Pparg,19016:up|Gas6,14456:up|Tnf,21926:up|Glp1r,14652:down|Egfr,13649:down|Fgfr1,14182:down|Sec23a,20334:down|Lpl,16956:up|Hmox1,15368:up|Sfrp1,20377:up|C1qtnf3,81799:down|Cd4,12504:up|Ptgs2,19225:down|Sncg,20618:up|Grem1,23892:up|Ceacam1,26365:up|Sfrp2,20319:up|Syt13,80976:up
organic cyclic compound catabolic process	GO:1901361	5.19E-02	1.20E-01	Akr1c18,105349:up|Hmox1,15368:up|Enpp5,83965:up|Igf1,16000:up|Hal,15109:up|Htr2a,15558:down|Igfbp3,16009:up|Blvrb,233016:up|Enpp1,18605:down|Enpp3,209558:down
cellular response to biotic stimulus	GO:0071216	5.22E-02	1.20E-01	Ankrd1,107765:down|Tnf,21926:up|Mrc1,17533:up|Wnt5a,22418:down|Ccl2,20296:up|Ccl12,20293:up
negative regulation of cell activation	GO:0050866	5.22E-02	1.20E-01	Ceacam1,26365:up|Cd24a,12484:down|Sfrp1,20377:up|Hmox1,15368:up|Serpine2,20720:up|Bmp4,12159:up
negative regulation of intracellular transport	GO:0032387	5.26E-02	1.20E-01	Gas6,14456:up|Hmox1,15368:up|C1qtnf3,81799:down|Tgfb2,21808:down|Adipoq,11450:up
glycosaminoglycan metabolic process	GO:0030203	5.27E-02	1.20E-01	Mamdc2,71738:down|Chst11,58250:down|Has2,15117:down|Igf1,16000:up
aminoglycan biosynthetic process	GO:0006023	5.29E-02	1.20E-01	Chst11,58250:down|Has2,15117:down|Igf1,16000:up
cerebral cortex cell migration	GO:0021795	5.29E-02	1.20E-01	Fgfr1,14182:down|Egfr,13649:down|Cx3cr1,13051:down
natural killer cell mediated cytotoxicity	GO:0042267	5.29E-02	1.20E-01	Ceacam1,26365:up|Clec2d,93694:up|Igf2,16002:up
negative regulation of tumor necrosis factor superfamily cytokine production	GO:1903556	5.29E-02	1.20E-01	Gas6,14456:up|Errfi1,74155:down|Adipoq,11450:up
modification by host of symbiont morphology or physiology	GO:0051851	5.29E-02	1.20E-01	Ceacam1,26365:up|Ptx3,19288:up|Ccl4,20303:up
glutathione metabolic process	GO:0006749	5.29E-02	1.20E-01	Ggt5,23887:up|Gstm1,14862:up|Gstt1,14871:up
immune complex clearance	GO:0002434	5.33E-02	1.20E-01	Ccl2,20296:up
positive regulation of hepatocyte differentiation	GO:0070368	5.33E-02	1.20E-01	Bmp4,12159:up
negative regulation of plasminogen activation	GO:0010757	5.33E-02	1.20E-01	Serpine2,20720:up
regulation of serine phosphorylation of STAT3 protein	GO:1903261	5.33E-02	1.20E-01	Ret,19713:up
single-organism reproductive behavior	GO:0044704	5.33E-02	1.20E-01	Serpine2,20720:up
cellular response to insulin-like growth factor stimulus	GO:1990314	5.33E-02	1.20E-01	Igf1,16000:up
UMP biosynthetic process	GO:0006222	5.33E-02	1.20E-01	Uck2,80914:down
Mullerian duct regression	GO:0001880	5.33E-02	1.20E-01	Bmpr1a,12166:down

cholangiocyte apoptotic process	GO:1902488	5.33E-02	1.20E-01	Igf1,16000:up
non-canonical Wnt signaling pathway involved in heart development	GO:0061341	5.33E-02	1.20E-01	Wnt5a,22418:down
pyrimidine ribonucleoside monophosphate metabolic process	GO:0009173	5.33E-02	1.20E-01	Uck2,80914:down
insulin receptor signaling pathway via phosphatidylinositol 3-kinase	GO:0038028	5.33E-02	1.20E-01	Igf2,16002:up
vitamin D3 metabolic process	GO:0070640	5.33E-02	1.20E-01	Fgfr1,14182:down
negative regulation of glomerular mesangial cell proliferation	GO:0072125	5.33E-02	1.20E-01	Bmp4,12159:up
regulation of platelet-derived growth factor production	GO:0090361	5.33E-02	1.20E-01	Ptgs2,19225:down
cyclooxygenase pathway	GO:0019371	5.33E-02	1.20E-01	Ptgs2,19225:down
Peyer's patch morphogenesis	GO:0061146	5.33E-02	1.20E-01	Ret,19713:up
glial cell-derived neurotrophic factor receptor signaling pathway	GO:0035860	5.33E-02	1.20E-01	Sulf1,240725:down
modulation by host of viral molecular function	GO:0044868	5.33E-02	1.20E-01	Ptx3,19288:up
positive regulation of CDP-diacylglycerol-serine O-phosphatidyltransferase activity	GO:1904219	5.33E-02	1.20E-01	Serinc2,230779:down
hexitol metabolic process	GO:0006059	5.33E-02	1.20E-01	Sord,20322:up
negative regulation of low-density lipoprotein particle receptor biosynthetic process	GO:0045715	5.33E-02	1.20E-01	Adipoq,11450:up
microglial cell activation involved in immune response	GO:0002282	5.33E-02	1.20E-01	Cx3cr1,13051:down
positive regulation of vitamin D biosynthetic process	GO:0060557	5.33E-02	1.20E-01	Tnf,21926:up
pyrimidine ribonucleoside monophosphate biosynthetic process	GO:0009174	5.33E-02	1.20E-01	Uck2,80914:down
positive regulation of lymphangiogenesis	GO:1901492	5.33E-02	1.20E-01	Vegfa,22339:down
activation of membrane attack complex	GO:0001905	5.33E-02	1.20E-01	C3,12266:up
olfactory pit development	GO:0060166	5.33E-02	1.20E-01	Aldh1a3,56847:down
positive regulation of vitamin D 24-hydroxylase activity	GO:0010980	5.33E-02	1.20E-01	Vdr,22337:down
type IIa hypersensitivity	GO:0001794	5.33E-02	1.20E-01	C3,12266:up
L-cysteine metabolic process	GO:0046439	5.33E-02	1.20E-01	Cdo1,12583:up
apoptotic process involved in outflow tract morphogenesis	GO:0003275	5.33E-02	1.20E-01	Tgfb2,21808:down
regulation of parathyroid hormone secretion	GO:2000828	5.33E-02	1.20E-01	Fgfr1,14182:down
regulation of activation of membrane attack complex	GO:0001969	5.33E-02	1.20E-01	C3,12266:up
mesenchymal cell proliferation involved in ureteric bud development	GO:0072138	5.33E-02	1.20E-01	Bmp4,12159:up

cellular response to temperature stimulus	GO:0071502	5.33E-02	1.20E-01	Htr2b,15559:down
positive regulation of type II hypersensitivity	GO:0002894	5.33E-02	1.20E-01	C3,12266:up
multi-organism membrane fusion	GO:0044800	5.33E-02	1.20E-01	Gas6,14456:up
regulation of chondrocyte development	GO:0061181	5.33E-02	1.20E-01	Fam101b,76566:down
fusion of virus membrane with host plasma membrane	GO:0019064	5.33E-02	1.20E-01	Gas6,14456:up
postnatal olfactory bulb interneuron migration	GO:0021827	5.33E-02	1.20E-01	Fgfr1,14182:down
transforming growth factor beta receptor complex assembly	GO:0007181	5.33E-02	1.20E-01	Tgfbr3,21814:up
positive regulation of cAMP-dependent protein kinase activity	GO:2000481	5.33E-02	1.20E-01	Adipoq,11450:up
specification of organ position	GO:0010159	5.33E-02	1.20E-01	Bmp4,12159:up
negative regulation of leukocyte tethering or rolling	GO:1903237	5.33E-02	1.20E-01	Cxcl12,20315:up
negative regulation of glomerulus development	GO:0090194	5.33E-02	1.20E-01	Bmp4,12159:up
modulation by host of symbiont catalytic activity	GO:0052422	5.33E-02	1.20E-01	Ptx3,19288:up
mammary gland bud morphogenesis	GO:0060648	5.33E-02	1.20E-01	Fgfr2,14183:up
positive regulation of serine C-palmitoyltransferase activity	GO:1904222	5.33E-02	1.20E-01	Serinc2,230779:down
negative regulation of synapse assembly	GO:0051964	5.33E-02	1.20E-01	Wnt5a,22418:down
metanephric glomerular epithelium development	GO:0072244	5.33E-02	1.20E-01	Adipoq,11450:up
regulation of transforming growth factor beta3 production	GO:0032910	5.33E-02	1.20E-01	Cd24a,12484:down
Wnt signaling pathway involved in digestive tract morphogenesis	GO:0044333	5.33E-02	1.20E-01	Wnt5a,22418:down
Wnt signaling pathway, calcium modulating pathway	GO:0007223	5.33E-02	1.20E-01	Wnt5a,22418:down
positive regulation of inorganic anion transmembrane transport	GO:1903797	5.33E-02	1.20E-01	Fxyd1,56188:up
regulation of retinal ganglion cell axon guidance	GO:0090259	5.33E-02	1.20E-01	Vegfa,22339:down
transforming growth factor beta3 production	GO:0032907	5.33E-02	1.20E-01	Cd24a,12484:down
extracellular polysaccharide biosynthetic process	GO:0045226	5.33E-02	1.20E-01	Has2,15117:down
positive regulation of keratinocyte apoptotic process	GO:1902174	5.33E-02	1.20E-01	Sfrp4,20379:up
regulation of apoptotic process involved in outflow tract morphogenesis	GO:1902256	5.33E-02	1.20E-01	Tgfb2,21808:down
extracellular polysaccharide metabolic process	GO:0046379	5.33E-02	1.20E-01	Has2,15117:down
regulation of chronic inflammatory response to antigenic stimulus	GO:0002874	5.33E-02	1.20E-01	Tnf,21926:up

positive regulation of type IIa hypersensitivity	GO:0001798	5.33E-02	1.20E-01	C3,12266:up
positive regulation of integrin biosynthetic process	GO:0045726	5.33E-02	1.20E-01	Tgfb2,21808:down
negative regulation of cholangiocyte apoptotic process	GO:1904193	5.33E-02	1.20E-01	Igf1,16000:up
planar cell polarity pathway involved in heart morphogenesis	GO:0061346	5.33E-02	1.20E-01	Wnt5a,22418:down
prostate gland stromal morphogenesis	GO:0060741	5.33E-02	1.20E-01	Igf1,16000:up
modulation of catalytic activity in other organism involved in symbiotic interaction	GO:0052203	5.33E-02	1.20E-01	Ptx3,19288:up
L-cysteine catabolic process	GO:0019448	5.33E-02	1.20E-01	Cdo1,12583:up
positive regulation of cell proliferation by VEGF-activated platelet derived growth factor receptor signaling pathway	GO:0038091	5.33E-02	1.20E-01	Vegfa,22339:down
regulation of osteoclast proliferation	GO:0090289	5.33E-02	1.20E-01	Grem1,23892:up
prostate epithelial cord elongation	GO:0060523	5.33E-02	1.20E-01	Fgfr2,14183:up
positive regulation of catagen	GO:0051795	5.33E-02	1.20E-01	Tgfb2,21808:down
positive regulation of cell-cell adhesion mediated by integrin	GO:0033634	5.33E-02	1.20E-01	Cd24a,12484:down
positive regulation of epithelial cell differentiation involved in kidney development	GO:2000698	5.33E-02	1.20E-01	Adipoq,11450:up
negative regulation of cardiac muscle adaptation	GO:0010616	5.33E-02	1.20E-01	Errfi1,74155:down
regulation of cholangiocyte apoptotic process	GO:1904192	5.33E-02	1.20E-01	Igf1,16000:up
regulation of cytotoxic T cell differentiation	GO:0045583	5.33E-02	1.20E-01	Tnfsf9,21950:up
regulation of monocyte aggregation	GO:1900623	5.33E-02	1.20E-01	Has2,15117:down
platelet-derived growth factor production	GO:0090360	5.33E-02	1.20E-01	Ptgs2,19225:down
ureter urothelium development	GO:0072190	5.33E-02	1.20E-01	Bmp4,12159:up
striatal medium spiny neuron differentiation	GO:0021773	5.33E-02	1.20E-01	Inhba,16323:down
positive regulation of transcription from RNA polymerase II promoter involved in smooth muscle cell differentiation	GO:2000721	5.33E-02	1.20E-01	Npnt,114249:up
deltoid tuberosity development	GO:0035993	5.33E-02	1.20E-01	Bmp4,12159:up
positive regulation of vitamin metabolic process	GO:0046136	5.33E-02	1.20E-01	Tnf,21926:up
regulation of vitamin D 24-hydroxylase activity	GO:0010979	5.33E-02	1.20E-01	Vdr,22337:down
cerebral cortex tangential migration using cell-cell interactions	GO:0021823	5.33E-02	1.20E-01	Fgfr1,14182:down
regulation of endothelial tube morphogenesis	GO:1901509	5.33E-02	1.20E-01	Adamts12,239337:down
mesenchymal stem cell proliferation	GO:0097168	5.33E-02	1.20E-01	Bmp4,12159:up
positive regulation of interleukin-18 production	GO:0032741	5.33E-02	1.20E-01	Tnf,21926:up

cysteine catabolic process	GO:0009093	5.33E-02	1.20E-01	Cdo1,12583:up
type II hypersensitivity	GO:0002445	5.33E-02	1.20E-01	C3,12266:up
regulation of cell proliferation involved in outflow tract morphogenesis	GO:1901963	5.33E-02	1.20E-01	Bmp4,12159:up
negative regulation of Wnt signaling pathway involved in dorsal/ventral axis specification	GO:2000054	5.33E-02	1.20E-01	Sfrp1,20377:up
protein kinase D signaling	GO:0089700	5.33E-02	1.20E-01	Vegfa,22339:down
regulation of type IIa hypersensitivity	GO:0001796	5.33E-02	1.20E-01	C3,12266:up
UMP metabolic process	GO:0046049	5.33E-02	1.20E-01	Uck2,80914:down
cardiac right atrium morphogenesis	GO:0003213	5.33E-02	1.20E-01	Wnt5a,22418:down
chemorepulsion of axon	GO:0061643	5.33E-02	1.20E-01	Wnt5a,22418:down
membrane fusion involved in viral entry into host cell	GO:0039663	5.33E-02	1.20E-01	Gas6,14456:up
polyprenol catabolic process	GO:0016095	5.33E-02	1.20E-01	Akr1c18,105349:up
epicardial cell to mesenchymal cell transition	GO:0003347	5.33E-02	1.20E-01	Tgfbr3,21814:up
positive regulation of production of miRNAs involved in gene silencing by miRNA	GO:1903800	5.33E-02	1.20E-01	Egfr,13649:down
oligodendrocyte apoptotic process	GO:0097252	5.33E-02	1.20E-01	Gas6,14456:up
regulation of type II hypersensitivity	GO:0002892	5.33E-02	1.20E-01	C3,12266:up
positive regulation of cytotoxic T cell differentiation	GO:0045585	5.33E-02	1.20E-01	Tnfsf9,21950:up
pilomotor reflex	GO:0097195	5.33E-02	1.20E-01	Npnt,114249:up
3'-phosphoadenosine 5'-phosphosulfate metabolic process	GO:0050427	5.33E-02	1.20E-01	Enpp1,18605:down
chronic inflammatory response to antigenic stimulus	GO:0002439	5.33E-02	1.20E-01	Tnf,21926:up
positive regulation of superoxide dismutase activity	GO:1901671	5.33E-02	1.20E-01	Tnf,21926:up
positive regulation of monocyte aggregation	GO:1900625	5.33E-02	1.20E-01	Has2,15117:down
substrate-dependent cell migration, cell attachment to substrate	GO:0006931	5.33E-02	1.20E-01	Tnfrsf12a,27279:down
positive regulation of calcidiol 1-monooxygenase activity	GO:0060559	5.33E-02	1.20E-01	Tnf,21926:up
regulation of CDP-diacylglycerol-serine O-phosphatidyltransferase activity	GO:1904217	5.33E-02	1.20E-01	Serinc2,230779:down
regulation of isoprenoid metabolic process	GO:0019747	5.33E-02	1.20E-01	Akr1c18,105349:up
VEGF-activated platelet-derived growth factor receptor signaling pathway	GO:0038086	5.33E-02	1.20E-01	Vegfa,22339:down
platelet-derived growth factor receptor-alpha signaling pathway	GO:0035790	5.33E-02	1.20E-01	Adipoq,11450:up

positive regulation of RNA metabolic process	GO:0051254	5.45E-02	1.22E-01	Fstl3,83554:down|Ice1,218333:down|Epas1,13819:up|Lyl1,17095:up|Sdpr,20324:down|Trf,22041:up|Wnt5a,22418:down|Bmpr1a,12166:down|Bmp4,12159:up|Igf1,16000:up|Ret,19713:up|Plpp3,67916:up|Creb3l1,26427:down|Fgfr2,14183:up|Prl2c2,18811:up|Rasl11a,68895:up|Inhba,16323:down|Ccl3,20302:up|Vdr,22337:down|Vegfa,22339:down|Igf2,16002:up|Slc40a1,53945:up|Tnf,21926:up|Pparg,19016:up|Glp1r,14652:down|Egfr,13649:down|Fgfr1,14182:down|Sfrp1,20377:up|Tgfbr3,21814:up|Grem1,23892:up|Sfrp2,20319:up|Npnt,114249:up
endothelial cell chemotaxis	GO:0035767	5.46E-02	1.22E-01	Fgfr1,14182:down|Vegfa,22339:down
trachea development	GO:0060438	5.46E-02	1.22E-01	Rspo2,239405:up|Bmp4,12159:up
smooth muscle tissue development	GO:0048745	5.46E-02	1.22E-01	Col3a1,12825:down|Bmp4,12159:up
thymocyte apoptotic process	GO:0070242	5.46E-02	1.22E-01	Wnt5a,22418:down|Bmp4,12159:up
positive regulation of interleukin-1 beta production	GO:0032731	5.46E-02	1.22E-01	Wnt5a,22418:down|Ccl3,20302:up
cell surface receptor signaling pathway involved in heart development	GO:0061311	5.46E-02	1.22E-01	Wnt5a,22418:down|Bmp4,12159:up
positive regulation of transforming growth factor beta receptor signaling pathway	GO:0030511	5.46E-02	1.22E-01	Tgfbr3,21814:up|Npnt,114249:up
positive regulation of monooxygenase activity	GO:0032770	5.46E-02	1.22E-01	Htr2b,15559:down|Tnf,21926:up
cellular response to interferon-beta	GO:0035458	5.46E-02	1.22E-01	Ifit1,15957:up|Ifit3,15959:up
positive regulation of cellular response to transforming growth factor beta stimulus	GO:1903846	5.46E-02	1.22E-01	Tgfbr3,21814:up|Npnt,114249:up
negative adaptation of signaling pathway	GO:0022401	5.46E-02	1.22E-01	Htr2b,15559:down|Adm,11535:up
desensitization of G-protein coupled receptor protein signaling pathway	GO:0002029	5.46E-02	1.22E-01	Htr2b,15559:down|Adm,11535:up
definitive hemopoiesis	GO:0060216	5.46E-02	1.22E-01	Lyl1,17095:up|Tgfbr3,21814:up
modulation by virus of host morphology or physiology	GO:0019048	5.46E-02	1.22E-01	Ceacam1,26365:up|Cd4,12504:up
immunoglobulin secretion	GO:0048305	5.46E-02	1.22E-01	Ceacam1,26365:up|Tnf,21926:up
calcineurin-NFAT signaling cascade	GO:0033173	5.46E-02	1.22E-01	Lmcd1,30937:up|Igf1,16000:up
cofactor catabolic process	GO:0051187	5.46E-02	1.22E-01	Hmox1,15368:up|Blvrb,233016:up
negative regulation of organic acid transport	GO:0032891	5.46E-02	1.22E-01	Tnf,21926:up|Il1rn,16181:up
cellular nitrogen compound catabolic process	GO:0044270	5.51E-02	1.23E-01	Hmox1,15368:up|Enpp5,83965:up|Igf1,16000:up|Hal,15109:up|Htr2a,15558:down|Igfbp3,16009:up|Blvrb,233016:up|Enpp1,18605:down|Enpp3,209558:down
regulation of symbiosis, encompassing mutualism through parasitism	GO:0043903	5.51E-02	1.23E-01	Tnf,21926:up|Lmcd1,30937:up|Ptx3,19288:up|Slpi,20568:up|Ccl4,20303:up|Ceacam1,26365:up|Oasl1,231655:up|Ifit1,15957:up|Oas3,246727:up
reproductive process	GO:0022414	5.55E-02	1.24E-01	Adm,11535:up|Hpgd,15446:up|Inhba,16323:down|Gjb5,14622:down|Ednrb,13618:up|Plat,18791:down|Epas1,13819:up|Vdr,22337:down|Cxcl12,20315:up|Vegfa,22339:down|Mmp2,17390:down|Serpine2,20720:up|Pparg,19016:up|Akr1c18,105349:up|Egfr,13649:down|Alpl,11647:up|Sfrp1,20377:up|Sulf1,240725:down|Wnt5a,22418:down|Bmpr1a,12166:down|Ptgs2,19225:down|Bmp4,12159:up|Igf1,16000:up|Sfrp2,20319:up|Tnc,21923:down|Fgfr2,14183:up|Kitl,17311:up
reproduction	GO:0000003	5.55E-02	1.24E-01	Adm,11535:up|Hpgd,15446:up|Inhba,16323:down|Gjb5,14622:down|Ednrb,13618:up|Plat,18791:down|Epas1,13819:up|Vdr,22337:down|Cxcl12,20315:up|Vegfa,22339:down|Mmp2,17390:down|Serpine2,20720:up|Pparg,19016:up|Akr1c18,105349:up|Egfr,13649:down|Alpl,11647:up|Sfrp1,20377:up|Sulf1,240725:down|Wnt5a,22418:down|Bmpr1a,12166:down|Ptgs2,19225:down|Bmp4,12159:up|Igf1,16000:up|Sfrp2,20319:up|Tnc,21923:down|Fgfr2,14183:up|Kitl,17311:up
regulation of peptide transport	GO:0090087	5.56E-02	1.24E-01	Tnf,21926:up|Pparg,19016:up|Glp1r,14652:down|Egfr,13649:down|Sfrp1,20377:up|C1qtnf3,81799:down|Ptgs2,19225:down|Grem1,23892:up
neuron death	GO:0070997	5.57E-02	1.24E-01	Tnf,21926:up|Glp1r,14652:down|Hmox1,15368:up|Ccl12,20293:up|Ccl3,20302:up|Igf1,16000:up|Crlf1,12931:down|Vegfa,22339:down|Tgfb2,21808:down|Mt1,17748:up
hormone biosynthetic process	GO:0042446	5.57E-02	1.24E-01	Akr1c18,105349:up|Igf2,16002:up|Igf1,16000:up
positive regulation of calcium ion import	GO:0090280	5.57E-02	1.24E-01	Cxcl12,20315:up|Ccl12,20293:up|Stc1,20855:up
negative regulation of JAK-STAT cascade	GO:0046426	5.57E-02	1.24E-01	Flrt2,399558:down|Lrrc15,74488:down|Aspn,66695:down

catecholamine secretion	GO:0050432	5.57E-02	1.24E-01	Cxcl12,20315:up|Htr2a,15558:down|Sncg,20618:up
regulation of mitotic nuclear division	GO:0007088	5.59E-02	1.25E-01	Tnf,21926:up|Igf2,16002:up|Fgfr2,14183:up|Igf1,16000:up|Bmp4,12159:up
negative regulation of neuron death	GO:1901215	5.62E-02	1.25E-01	Glp1r,14652:down|Crlf1,12931:down|Hmox1,15368:up|Vegfa,22339:down|Ccl12,20293:up|Mt1,17748:up|Igf1,16000:up
metanephros development	GO:0001656	5.69E-02	1.27E-01	Ret,19713:up|Grem1,23892:up|Bmp4,12159:up|Adipoq,11450:up
JAK-STAT cascade	GO:0007259	5.80E-02	1.29E-01	Flrt2,399558:down|Crlf1,12931:down|Ret,19713:up|Lrrc15,74488:down|Aspn,66695:down|Igf1,16000:up
interleukin-1 beta production	GO:0032611	5.86E-02	1.30E-01	Wnt5a,22418:down|Errfi1,74155:down|Ccl3,20302:up
natural killer cell mediated immunity	GO:0002228	5.86E-02	1.30E-01	Ceacam1,26365:up|Clec2d,93694:up|Igf2,16002:up
negative regulation of T cell proliferation	GO:0042130	5.86E-02	1.30E-01	Ceacam1,26365:up|Cd24a,12484:down|Bmp4,12159:up
labyrinthine layer development	GO:0060711	5.86E-02	1.30E-01	Adm,11535:up|Gjb5,14622:down|Fgfr2,14183:up
negative regulation of I-kappaB kinase/NF-kappaB signaling	GO:0043124	5.86E-02	1.30E-01	C1qtnf3,81799:down|Il1rl1,17082:down|Adipoq,11450:up
regulation of sequence-specific DNA binding transcription factor activity	GO:0051090	5.87E-02	1.30E-01	Tnf,21926:up|Pparg,19016:up|Gas6,14456:up|Hmox1,15368:up|Wnt5a,22418:down|Tgfbr3,21814:up|Sfrp4,20379:up|Grem1,23892:up|Vegfa,22339:down|Plpp3,67916:up
negative regulation of cell cycle	GO:0045786	5.87E-02	1.30E-01	Plk2,20620:down|Egfr,13649:down|Hpgd,15446:up|Inhba,16323:down|Susd2,71733:up|Ptgs2,19225:down|Bmp4,12159:up|Nabp1,109019:down|Tgfb2,21808:down|Fgfr2,14183:up
catechol-containing compound biosynthetic process	GO:0009713	5.93E-02	1.31E-01	Tgfb2,21808:down|Epas1,13819:up
regulation of protein localization to cell surface	GO:2000008	5.93E-02	1.31E-01	Tnf,21926:up|Gpm6b,14758:up
heparan sulfate proteoglycan metabolic process	GO:0030201	5.93E-02	1.31E-01	Sulf1,240725:down|Hpse,15442:up
negative regulation of glucose transport	GO:0010829	5.93E-02	1.31E-01	Tnf,21926:up|Enpp1,18605:down
physiological cardiac muscle hypertrophy	GO:0003301	5.93E-02	1.31E-01	Wisp1,22402:down|Igf1,16000:up
regulation of cell maturation	GO:1903429	5.93E-02	1.31E-01	Ret,19713:up|Ednrb,13618:up
regulation of cGMP metabolic process	GO:0030823	5.93E-02	1.31E-01	Wnt5a,22418:down|Vegfa,22339:down
catecholamine biosynthetic process	GO:0042423	5.93E-02	1.31E-01	Tgfb2,21808:down|Epas1,13819:up
cell growth involved in cardiac muscle cell development	GO:0061049	5.93E-02	1.31E-01	Wisp1,22402:down|Igf1,16000:up
antibacterial humoral response	GO:0019731	5.93E-02	1.31E-01	Adm,11535:up|Slpi,20568:up
physiological muscle hypertrophy	GO:0003298	5.93E-02	1.31E-01	Wisp1,22402:down|Igf1,16000:up
organ regeneration	GO:0031100	5.93E-02	1.31E-01	Cxcl12,20315:up|Hmox1,15368:up
regulation of smooth muscle cell differentiation	GO:0051150	5.93E-02	1.31E-01	Fgfr2,14183:up|Bmp4,12159:up
glycerolipid biosynthetic process	GO:0045017	5.94E-02	1.31E-01	Htr2b,15559:down|Fabp5,16592:up|Lpl,16956:up|Htr2a,15558:down|C3,12266:up
response to oxidative stress	GO:0006979	6.07E-02	1.34E-01	Tnf,21926:up|Akr1c18,105349:up|Hp,15439:up|Hmox1,15368:up|Ptgs2,19225:down|Epas1,13819:up|Bmp4,12159:up|Adipoq,11450:up|Pxdn,69675:down|Mmp2,17390:down
regulation of DNA biosynthetic process	GO:2000278	6.12E-02	1.35E-01	Ankrd1,107765:down|Pparg,19016:up|Grem1,23892:up|Adipoq,11450:up
neuroepithelial cell differentiation	GO:0060563	6.12E-02	1.35E-01	Fgfr1,14182:down|Mcoln3,171166:up|Ednrb,13618:up|Kitl,17311:up
epidermal growth factor receptor signaling pathway	GO:0007173	6.12E-02	1.35E-01	Ceacam1,26365:up|Egfr,13649:down|Efemp1,216616:up|Errfi1,74155:down
cyclic-nucleotide-mediated signaling	GO:0019935	6.15E-02	1.35E-01	Glp1r,14652:down|Ptgir,19222:up|Ednrb,13618:up
interaction with symbiont	GO:0051702	6.15E-02	1.35E-01	Ceacam1,26365:up|Ptx3,19288:up|Ccl4,20303:up
glandular epithelial cell differentiation	GO:0002067	6.15E-02	1.35E-01	Wnt5a,22418:down|Fgfr2,14183:up|Bmp4,12159:up
catechol-containing compound metabolic process	GO:0009712	6.15E-02	1.35E-01	Tgfb2,21808:down|Maob,109731:up|Epas1,13819:up
epithelial cell morphogenesis	GO:0003382	6.15E-02	1.35E-01	Stc1,20855:up|Grem1,23892:up|Bmp4,12159:up

regulation of endocrine process	GO:0044060	6.15E-02	1.35E-01	Fgfr1,14182:down|C1qtnf3,81799:down|Inhba,16323:down
catecholamine metabolic process	GO:0006584	6.15E-02	1.35E-01	Tgfb2,21808:down|Maob,109731:up|Epas1,13819:up
heterocycle catabolic process	GO:0046700	6.16E-02	1.35E-01	Hmox1,15368:up|Enpp5,83965:up|Igf1,16000:up|Hal,15109:up|Htr2a,15558:down|Igfbp3,16009:up|Blvrb,233016:up|Enpp1,18605:down|Enpp3,209558:down
camera-type eye development	GO:0043010	6.27E-02	1.38E-01	Egfr,13649:down|Inhba,16323:down|Aldh1a2,19378:up|Col8a1,12837:down|Ninj1,18081:up|Bmp4,12159:up|Vegfa,22339:down|Aldh1a3,56847:down|Fgfr2,14183:up
establishment of protein localization	GO:0045184	6.27E-02	1.38E-01	Ice1,218333:down|Ccl2,20296:up|Mmp13,17386:up|Ankrd1,107765:down|Gpm6b,14758:up|Clec4n,56620:up|Postn,50706:down|Akr1c18,105349:up|Cd24a,12484:down|Wnt5a,22418:down|Bmpr1a,12166:down|Il1rn,16181:up|Adipoq,11450:up|Bmp4,12159:up|Igf1,16000:up|Tgfb2,21808:down|Il1rl1,17082:down|Clec4e,56619:up|Htr2b,15559:down|Ccl3,20302:up|Lrrc15,74488:down|Kdelr3,105785:down|Tnf,21926:up|Pparg,19016:up|Gas6,14456:up|Glp1r,14652:down|Egfr,13649:down|Sfrp1,20377:up|Lpl,16956:up|Sec23a,20334:down|C1qtnf3,81799:down|Tfrc,22042:up|Ptgs2,19225:down|Sncg,20618:up|Grem1,23892:up|Ceacam1,26365:up|Sfrp2,20319:up|Npnt,114249:up
calcium ion transmembrane transport	GO:0070588	6.28E-02	1.38E-01	Htr2b,15559:down|Gas6,14456:up|Glp1r,14652:down|Tgfb2,21808:down|Htr2a,15558:down|S100a1,20193:up|Bmp4,12159:up
positive regulation of sequence-specific DNA binding transcription factor activity	GO:0051091	6.28E-02	1.38E-01	Tnf,21926:up|Pparg,19016:up|Wnt5a,22418:down|Vegfa,22339:down|Tgfbr3,21814:up|Plpp3,67916:up|Grem1,23892:up
positive regulation of myeloid cell differentiation	GO:0045639	6.35E-02	1.39E-01	Tnf,21926:up|Inhba,16323:down|Ccl3,20302:up|Kitl,17311:up
muscle adaptation	GO:0043500	6.35E-02	1.39E-01	Lmcd1,30937:up|Wisp1,22402:down|Errfi1,74155:down|Igf1,16000:up
cell maturation	GO:0048469	6.41E-02	1.40E-01	Pparg,19016:up|Fgfr1,14182:down|Ret,19713:up|Vegfa,22339:down|Ednrb,13618:up|Epas1,13819:up
positive regulation of collagen biosynthetic process	GO:0032967	6.42E-02	1.40E-01	Ccl2,20296:up|Bmp4,12159:up
reflex	GO:0060004	6.42E-02	1.40E-01	Aldh1a3,56847:down|Npnt,114249:up
positive regulation of T cell migration	GO:2000406	6.42E-02	1.40E-01	Cxcl12,20315:up|Wnt5a,22418:down
substrate-dependent cell migration	GO:0006929	6.42E-02	1.40E-01	Tnfrsf12a,27279:down|Fgfr1,14182:down
regulation of cellular extravasation	GO:0002691	6.42E-02	1.40E-01	Cxcl12,20315:up|Ccl2,20296:up
type B pancreatic cell differentiation	GO:0003309	6.42E-02	1.40E-01	Wnt5a,22418:down|Bmp4,12159:up
regulation of pri-miRNA transcription from RNA polymerase II promoter	GO:1902893	6.42E-02	1.40E-01	Bmpr1a,12166:down|Bmp4,12159:up
positive regulation of cardiac muscle hypertrophy	GO:0010613	6.42E-02	1.40E-01	Wisp1,22402:down|Igf1,16000:up
androgen metabolic process	GO:0008209	6.42E-02	1.40E-01	Akr1c18,105349:up|Adm,11535:up
adaptation of signaling pathway	GO:0023058	6.42E-02	1.40E-01	Htr2b,15559:down|Adm,11535:up
positive regulation of muscle hypertrophy	GO:0014742	6.42E-02	1.40E-01	Wisp1,22402:down|Igf1,16000:up
embryonic hemopoiesis	GO:0035162	6.42E-02	1.40E-01	Vegfa,22339:down|Kitl,17311:up
heart formation	GO:0060914	6.42E-02	1.40E-01	Bmpr1a,12166:down|Bmp4,12159:up
regulation of catenin import into nucleus	GO:0035412	6.42E-02	1.40E-01	Sfrp2,20319:up|Egfr,13649:down
organ induction	GO:0001759	6.42E-02	1.40E-01	Fgfr1,14182:down|Bmp4,12159:up
pericardium development	GO:0060039	6.42E-02	1.40E-01	Wnt5a,22418:down|Tgfbr3,21814:up
positive regulation of synaptic transmission, glutamatergic	GO:0051968	6.42E-02	1.40E-01	Egfr,13649:down|Ptgs2,19225:down
atrioventricular valve development	GO:0003171	6.42E-02	1.40E-01	Tgfb2,21808:down|Bmpr1a,12166:down
glandular epithelial cell development	GO:0002068	6.42E-02	1.40E-01	Wnt5a,22418:down|Bmp4,12159:up
protein targeting	GO:0006605	6.44E-02	1.40E-01	Tnf,21926:up|Gas6,14456:up|Akr1c18,105349:up|Egfr,13649:down|C1qtnf3,81799:down|Bmpr1a,12166:down|Ccl2,20296:up|Ptgs2,19225:down|Grem1,23892:up|Igf1,16000:up|Bmp4,12159:up|Ceacam1,26365:up|Sfrp2,20319:up|Tgfb2,21808:down
regulation of alcohol biosynthetic process	GO:1902930	6.45E-02	1.40E-01	Vdr,22337:down|Tnf,21926:up|Akr1c18,105349:up
negative regulation of cellular protein localization	GO:1903828	6.48E-02	1.41E-01	Gas6,14456:up|Gpm6b,14758:up|C1qtnf3,81799:down|Lrrc15,74488:down|Adipoq,11450:up

<table>
<tr><td>organic acid transport</td><td>GO:0015849</td><td>6.53E-02</td><td>1.42E-01</td><td>Tnf,21926:up|Pparg,19016:up|Fxyd1,56188:up|Il1rn,16181:up|Serinc2,230779:down|Fam132b,227358:down|Emb,13723:up|Slc16a4,229699:down</td></tr>
<tr><td>alpha-amino acid metabolic process</td><td>GO:1901605</td><td>6.56E-02</td><td>1.42E-01</td><td>P4ha1,18451:down|Ddah1,69219:down|Gatm,67092:down|Gfpt2,14584:down|Serinc2,230779:down|Hal,15109:up|Cdo1,12583:up</td></tr>
<tr><td>regulation of generation of precursor metabolites and energy</td><td>GO:0043467</td><td>6.57E-02</td><td>1.42E-01</td><td>Htr2a,15558:down|Enpp1,18605:down|Igf2,16002:up|Igf1,16000:up</td></tr>
<tr><td>protein maturation</td><td>GO:0051604</td><td>6.61E-02</td><td>1.43E-01</td><td>Tnf,21926:up|Pparg,19016:up|C3,12266:up|Plat,18791:down|Serpinh1,12406:down|Ctla2a,13024:down|Serping1,12258:up|Serpine2,20720:up|F3,14066:up</td></tr>
<tr><td>cellular lipid metabolic process</td><td>GO:0044255</td><td>6.62E-02</td><td>1.43E-01</td><td>Htr2b,15559:down|Hpgd,15446:up|Aoah,27052:up|Aldh1a2,19378:up|C3,12266:up|Fabp4,11770:up|Fam132b,227358:down|Htr2a,15558:down|Pparg,19016:up|Ggt5,23887:up|Akr1c18,105349:up|Fabp5,16592:up|Lpl,16956:up|Ptgs2,19225:down|Serinc2,230779:down|Adipoq,11450:up|Crabp2,12904:down|Aldh1a3,56847:down|Prkar2b,19088:up|Plpp3,67916:up</td></tr>
<tr><td>neuron apoptotic process</td><td>GO:0051402</td><td>6.65E-02</td><td>1.44E-01</td><td>Tnf,21926:up|Glp1r,14652:down|Hmox1,15368:up|Ccl12,20293:up|Ccl3,20302:up|Crlf1,12931:down|Tgfb2,21808:down|Mt1,17748:up</td></tr>
<tr><td>defense response to bacterium</td><td>GO:0042742</td><td>6.70E-02</td><td>1.44E-01</td><td>Adamts4,240913:down|Tnf,21926:up|Adm,11535:up|Hp,15439:up|Cd4,12504:up|Slpi,20568:up|Clec4e,56619:up</td></tr>
<tr><td>neurological system process</td><td>GO:0050877</td><td>6.75E-02</td><td>1.44E-01</td><td>Ednrb,13618:up|Epas1,13819:up|Shroom4,208431:down|Htr2a,15558:down|Cx3cr1,13051:down|Serpine2,20720:up|Igf2,16002:up|Tnf,21926:up|Plk2,20620:down|Trf,22041:up|Glp1r,14652:down|Col11a1,12814:down|Egfr,13649:down|Fgfr1,14182:down|Ncam1,17967:down|Mme,17380:up|Ptgs2,19225:down|Il1rn,16181:up|Igf1,16000:up|Mcoln3,171166:up|Aldh1a3,56847:down|Prkar2b,19088:up</td></tr>
<tr><td>cellular response to ketone</td><td>GO:1901655</td><td>6.76E-02</td><td>1.44E-01</td><td>Gas6,14456:up|Postn,50706:down|Bmp4,12159:up</td></tr>
<tr><td>cellular carbohydrate catabolic process</td><td>GO:0044275</td><td>6.76E-02</td><td>1.44E-01</td><td>Sord,20322:up|Htr2a,15558:down|Igf1,16000:up</td></tr>
<tr><td>macrophage activation</td><td>GO:0042116</td><td>6.76E-02</td><td>1.44E-01</td><td>Wnt5a,22418:down|Cx3cr1,13051:down|Il1rl1,17082:down</td></tr>
<tr><td>regulation of interleukin-8 production</td><td>GO:0032677</td><td>6.76E-02</td><td>1.44E-01</td><td>Tnf,21926:up|Wnt5a,22418:down|Adipoq,11450:up</td></tr>
<tr><td>establishment of protein localization to organelle</td><td>GO:0072594</td><td>6.84E-02</td><td>1.44E-01</td><td>Tnf,21926:up|Gas6,14456:up|Akr1c18,105349:up|Egfr,13649:down|C1qtnf3,81799:down|Bmpr1a,12166:down|Ptgs2,19225:down|Grem1,23892:up|Igf1,16000:up|Bmp4,12159:up|Ceacam1,26365:up|Sfrp2,20319:up|Tgfb2,21808:down</td></tr>
<tr><td>cellular response to lipopolysaccharide</td><td>GO:0071222</td><td>6.86E-02</td><td>1.44E-01</td><td>Ankrd1,107765:down|Tnf,21926:up|Mrc1,17533:up|Ccl2,20296:up|Ccl12,20293:up</td></tr>
<tr><td>extracellular matrix assembly</td><td>GO:0085029</td><td>6.93E-02</td><td>1.44E-01</td><td>Gpm6b,14758:up|Has2,15117:down</td></tr>
<tr><td>apoptotic nuclear changes</td><td>GO:0030262</td><td>6.93E-02</td><td>1.44E-01</td><td>Cd24a,12484:down|Igfbp3,16009:up</td></tr>
<tr><td>response to X-ray</td><td>GO:0010165</td><td>6.93E-02</td><td>1.44E-01</td><td>Sfrp2,20319:up|Sfrp1,20377:up</td></tr>
<tr><td>copulation</td><td>GO:0007620</td><td>6.93E-02</td><td>1.44E-01</td><td>Serpine2,20720:up|Ednrb,13618:up</td></tr>
<tr><td>positive regulation of collagen metabolic process</td><td>GO:0010714</td><td>6.93E-02</td><td>1.44E-01</td><td>Ccl2,20296:up|Bmp4,12159:up</td></tr>
<tr><td>regulation of superoxide metabolic process</td><td>GO:0090322</td><td>6.93E-02</td><td>1.44E-01</td><td>Tnf,21926:up|Egfr,13649:down</td></tr>
<tr><td>cellular component disassembly involved in execution phase of apoptosis</td><td>GO:0006921</td><td>6.93E-02</td><td>1.44E-01</td><td>Cd24a,12484:down|Igfbp3,16009:up</td></tr>
<tr><td>cellular zinc ion homeostasis</td><td>GO:0006882</td><td>6.93E-02</td><td>1.44E-01</td><td>Mt2,17750:up|Mt1,17748:up</td></tr>
<tr><td>catenin import into nucleus</td><td>GO:0035411</td><td>6.93E-02</td><td>1.44E-01</td><td>Sfrp2,20319:up|Egfr,13649:down</td></tr>
<tr><td>metanephric mesenchymal cell migration</td><td>GO:0035789</td><td>7.04E-02</td><td>1.44E-01</td><td>Adipoq,11450:up</td></tr>
<tr><td>regulation of branching involved in salivary gland morphogenesis by mesenchymal-epithelial signaling</td><td>GO:0060665</td><td>7.04E-02</td><td>1.44E-01</td><td>Fgfr1,14182:down</td></tr>
<tr><td>chain elongation of O-linked mannose residue</td><td>GO:0044845</td><td>7.04E-02</td><td>1.44E-01</td><td>Sdf2l1,64136:up</td></tr>
<tr><td>negative regulation of neurotransmitter uptake</td><td>GO:0051581</td><td>7.04E-02</td><td>1.44E-01</td><td>Gpm6b,14758:up</td></tr>
<tr><td>regulation of cardiac muscle hypertrophy in response to stress</td><td>GO:1903242</td><td>7.04E-02</td><td>1.44E-01</td><td>Errfi1,74155:down</td></tr>
<tr><td>negative regulation of B cell differentiation</td><td>GO:0045578</td><td>7.04E-02</td><td>1.44E-01</td><td>Sfrp1,20377:up</td></tr>
<tr><td>positive regulation of melanocyte differentiation</td><td>GO:0045636</td><td>7.04E-02</td><td>1.44E-01</td><td>Kitl,17311:up</td></tr>
</table>

positive regulation of sodium ion export from cell	GO:1903278	7.04E-02	1.44E-01	Fxyd1,56188:up
protection from natural killer cell mediated cytotoxicity	GO:0042270	7.04E-02	1.44E-01	Clec2d,93694:up
modification by host of symbiont molecular function	GO:0052428	7.04E-02	1.44E-01	Ptx3,19288:up
negative regulation of cholesterol storage	GO:0010887	7.04E-02	1.44E-01	Pparg,19016:up
cell wall macromolecule biosynthetic process	GO:0044038	7.04E-02	1.44E-01	Sdf2l1,64136:up
negative regulation of serotonin uptake	GO:0051612	7.04E-02	1.44E-01	Gpm6b,14758:up
positive regulation of hair follicle maturation	GO:0048818	7.04E-02	1.44E-01	Tgfb2,21808:down
positive regulation of DNA catabolic process	GO:1903626	7.04E-02	1.44E-01	Igfbp3,16009:up
axis elongation involved in somitogenesis	GO:0090245	7.04E-02	1.44E-01	Sfrp1,20377:up
regulation of serine C-palmitoyltransferase activity	GO:1904220	7.04E-02	1.44E-01	Serinc2,230779:down
regulation of dermatome development	GO:0061183	7.04E-02	1.44E-01	Sfrp2,20319:up
positive regulation of oligodendrocyte progenitor proliferation	GO:0070447	7.04E-02	1.44E-01	Trf,22041:up
ascending aorta morphogenesis	GO:0035910	7.04E-02	1.44E-01	Tgfb2,21808:down
cytotoxic T cell differentiation	GO:0045065	7.04E-02	1.44E-01	Tnfsf9,21950:up
regulation of mast cell proliferation	GO:0070666	7.04E-02	1.44E-01	Kitl,17311:up
tendon cell differentiation	GO:0035990	7.04E-02	1.44E-01	Bmp4,12159:up
sodium-dependent phosphate transport	GO:0044341	7.04E-02	1.44E-01	Sfrp4,20379:up
trachea cartilage morphogenesis	GO:0060535	7.04E-02	1.44E-01	Rspo2,239405:up
regulation of sodium ion export	GO:1903273	7.04E-02	1.44E-01	Fxyd1,56188:up
negative regulation of neuron maturation	GO:0014043	7.04E-02	1.44E-01	Ednrb,13618:up
regulation of serotonin uptake	GO:0051611	7.04E-02	1.44E-01	Gpm6b,14758:up
ascending aorta development	GO:0035905	7.04E-02	1.44E-01	Tgfb2,21808:down
response to heparin	GO:0071503	7.04E-02	1.44E-01	Sfrp1,20377:up
cellular lactam metabolic process	GO:0072338	7.04E-02	1.44E-01	Mme,17380:up
trophectodermal cell proliferation	GO:0001834	7.04E-02	1.44E-01	Igf1,16000:up
regulation of catagen	GO:0051794	7.04E-02	1.44E-01	Tgfb2,21808:down
modulation by symbiont of host molecular function	GO:0052055	7.04E-02	1.44E-01	Ptx3,19288:up
creatinine metabolic process	GO:0046449	7.04E-02	1.44E-01	Mme,17380:up
regulation of mast cell cytokine production	GO:0032763	7.04E-02	1.44E-01	Hmox1,15368:up
positive regulation of transcription from RNA polymerase II promoter involved in heart development	GO:1901228	7.04E-02	1.44E-01	Grem1,23892:up
progesterone secretion	GO:0042701	7.04E-02	1.44E-01	Inhba,16323:down
negative regulation of bicellular tight junction assembly	GO:1903347	7.04E-02	1.44E-01	Tnf,21926:up
positive regulation of mast cell proliferation	GO:0070668	7.04E-02	1.44E-01	Kitl,17311:up

ureter morphogenesis	GO:0072197	7.04E-02	1.44E-01	Bmp4,12159:up
tendon formation	GO:0035992	7.04E-02	1.44E-01	Bmp4,12159:up
positive regulation of humoral immune response mediated by circulating immunoglobulin	GO:0002925	7.04E-02	1.44E-01	Tnf,21926:up
regulation of hepatocyte differentiation	GO:0070366	7.04E-02	1.44E-01	Bmp4,12159:up
cellular response to heparin	GO:0071504	7.04E-02	1.44E-01	Sfrp1,20377:up
maintenance of blood-brain barrier	GO:0035633	7.04E-02	1.44E-01	Ptgs2,19225:down
dermatome development	GO:0061054	7.04E-02	1.44E-01	Sfrp2,20319:up
cell migration involved in metanephros development	GO:0035788	7.04E-02	1.44E-01	Adipoq,11450:up
positive regulation of mammary gland involution	GO:1903521	7.04E-02	1.44E-01	Vdr,22337:down
mannoprotein metabolic process	GO:0006056	7.04E-02	1.44E-01	Sdf2l1,64136:up
mast cell proliferation	GO:0070662	7.04E-02	1.44E-01	Kitl,17311:up
regulation of sodium ion export from cell	GO:1903276	7.04E-02	1.44E-01	Fxyd1,56188:up
regulation of hepatocyte growth factor receptor signaling pathway	GO:1902202	7.04E-02	1.44E-01	Adamts12,239337:down
cell proliferation involved in mesonephros development	GO:0061209	7.04E-02	1.44E-01	Bmp4,12159:up
hematopoietic stem cell migration	GO:0035701	7.04E-02	1.44E-01	Gas6,14456:up
pyrimidine-containing compound salvage	GO:0008655	7.04E-02	1.44E-01	Uck2,80914:down
cell wall glycoprotein biosynthetic process	GO:0031506	7.04E-02	1.44E-01	Sdf2l1,64136:up
mesenchymal cell proliferation involved in ureter development	GO:0072198	7.04E-02	1.44E-01	Bmp4,12159:up
negative regulation of apoptotic process in bone marrow	GO:0071866	7.04E-02	1.44E-01	Fgfr2,14183:up
cellular component macromolecule biosynthetic process	GO:0070589	7.04E-02	1.44E-01	Sdf2l1,64136:up
pyrimidine nucleoside salvage	GO:0043097	7.04E-02	1.44E-01	Uck2,80914:down
astrocyte activation	GO:0048143	7.04E-02	1.44E-01	Egfr,13649:down
comma-shaped body morphogenesis	GO:0072049	7.04E-02	1.44E-01	Bmp4,12159:up
IMP salvage	GO:0032264	7.04E-02	1.44E-01	Ampd3,11717:up
regulation of apoptotic process in bone marrow	GO:0071865	7.04E-02	1.44E-01	Fgfr2,14183:up
negative regulation by host of symbiont molecular function	GO:0052405	7.04E-02	1.44E-01	Ptx3,19288:up
vein smooth muscle contraction	GO:0014826	7.04E-02	1.44E-01	Ednrb,13618:up
opsonization	GO:0008228	7.04E-02	1.44E-01	Ptx3,19288:up
bud outgrowth involved in lung branching	GO:0060447	7.04E-02	1.44E-01	Tnc,21923:down
cell wall biogenesis	GO:0042546	7.04E-02	1.44E-01	Sdf2l1,64136:up

positive regulation of transcription from RNA polymerase II promoter involved in myocardial precursor cell differentiation	GO:0003257	7.04E-02	1.44E-01	Grem1,23892:up
regulation of dendritic cell apoptotic process	GO:2000668	7.04E-02	1.44E-01	Gas6,14456:up
apoptotic process involved in mammary gland involution	GO:0060057	7.04E-02	1.44E-01	Vdr,22337:down
positive regulation of apoptotic process involved in morphogenesis	GO:1902339	7.04E-02	1.44E-01	Vdr,22337:down
positive regulation of cell-cell adhesion mediated by cadherin	GO:2000049	7.04E-02	1.44E-01	Wnt5a,22418:down
negative regulation of mesodermal cell fate specification	GO:0042662	7.04E-02	1.44E-01	Sfrp2,20319:up
mesendoderm development	GO:0048382	7.04E-02	1.44E-01	Bmpr1a,12166:down
mammary gland specification	GO:0060594	7.04E-02	1.44E-01	Fgfr2,14183:up
regulation of mesenchymal cell proliferation involved in ureter development	GO:0072199	7.04E-02	1.44E-01	Bmp4,12159:up
positive regulation of pancreatic juice secretion	GO:0090187	7.04E-02	1.44E-01	Mmp13,17386:up
activation of transmembrane receptor protein tyrosine kinase activity	GO:0007171	7.04E-02	1.44E-01	Pdgfc,54635:down
regulation of interleukin-1-mediated signaling pathway	GO:2000659	7.04E-02	1.44E-01	Il1rn,16181:up
prostate gland morphogenetic growth	GO:0060737	7.04E-02	1.44E-01	Fgfr2,14183:up
positive regulation of apoptotic process involved in mammary gland involution	GO:0060058	7.04E-02	1.44E-01	Vdr,22337:down
cell wall mannoprotein biosynthetic process	GO:0000032	7.04E-02	1.44E-01	Sdf2l1,64136:up
positive regulation of sodium ion export	GO:1903275	7.04E-02	1.44E-01	Fxyd1,56188:up
regulation of superoxide dismutase activity	GO:1901668	7.04E-02	1.44E-01	Tnf,21926:up
positive regulation of cytokine secretion involved in immune response	GO:0002741	7.04E-02	1.44E-01	Wnt5a,22418:down
phosphate ion transmembrane transport	GO:0035435	7.04E-02	1.44E-01	Ank,11732:down
regulation of metanephric mesenchymal cell migration	GO:2000589	7.04E-02	1.44E-01	Adipoq,11450:up
negative regulation of mast cell apoptotic process	GO:0033026	7.04E-02	1.44E-01	Kitl,17311:up
regulation of testosterone biosynthetic process	GO:2000224	7.04E-02	1.44E-01	Akr1c18,105349:up
positive regulation of endothelial cell chemotaxis to fibroblast growth factor	GO:2000546	7.04E-02	1.44E-01	Fgfr1,14182:down
positive regulation of apoptotic DNA fragmentation	GO:1902512	7.04E-02	1.44E-01	Igfbp3,16009:up
ferrous iron import into cell	GO:0097460	7.04E-02	1.44E-01	Trf,22041:up

regulation of branch elongation involved in ureteric bud branching	GO:0072095	7.04E-02	1.44E-01	Bmp4,12159:up
regulation of T cell homeostatic proliferation	GO:0046013	7.04E-02	1.44E-01	Cd24a,12484:down
regulation of interleukin-18 production	GO:0032661	7.04E-02	1.44E-01	Tnf,21926:up
positive regulation of glomerular filtration	GO:0003104	7.04E-02	1.44E-01	Gas6,14456:up
regulation of phosphate transport	GO:0010966	7.04E-02	1.44E-01	Sfrp4,20379:up
regulation of L-glutamate transport	GO:0002036	7.04E-02	1.44E-01	Tnf,21926:up
Fc-gamma receptor signaling pathway	GO:0038094	7.04E-02	1.44E-01	Clec4e,56619:up
endodermal digestive tract morphogenesis	GO:0061031	7.04E-02	1.44E-01	Fgfr2,14183:up
dense core granule biogenesis	GO:0061110	7.04E-02	1.44E-01	Serpine2,20720:up
dendritic cell apoptotic process	GO:0097048	7.04E-02	1.44E-01	Gas6,14456:up
regulation of Wnt signaling pathway involved in dorsal/ventral axis specification	GO:2000053	7.04E-02	1.44E-01	Sfrp1,20377:up
positive regulation of mast cell chemotaxis	GO:0060754	7.04E-02	1.44E-01	Figf,14205:up
exocyst assembly	GO:0001927	7.04E-02	1.44E-01	Ncam1,17967:down
regulation of mammary gland involution	GO:1903519	7.04E-02	1.44E-01	Vdr,22337:down
myoblast fate commitment	GO:0048625	7.04E-02	1.44E-01	Epas1,13819:up
negative regulation of lymphocyte migration	GO:2000402	7.04E-02	1.44E-01	Ccl12,20293:up
regulation of ribonuclease activity	GO:0060700	7.04E-02	1.44E-01	Oas3,246727:up
negative regulation of macrophage cytokine production	GO:0010936	7.04E-02	1.44E-01	Tgfb2,21808:down
mannoprotein biosynthetic process	GO:0006057	7.04E-02	1.44E-01	Sdf2l1,64136:up
diencephalon morphogenesis	GO:0048852	7.04E-02	1.44E-01	Wnt5a,22418:down
spongiotrophoblast differentiation	GO:0060708	7.04E-02	1.44E-01	Gjb5,14622:down
purine ribonucleoside bisphosphate metabolic process	GO:0034035	7.04E-02	1.44E-01	Enpp1,18605:down
activation of MAPK activity	GO:0000187	7.04E-02	1.44E-01	Tnf,21926:up|Trf,22041:up|Wnt5a,22418:down|Igf1,16000:up
sulfur compound biosynthetic process	GO:0044272	7.04E-02	1.44E-01	Chst11,58250:down|Ggt5,23887:up|Igf1,16000:up|Cdo1,12583:up
regulation of myotube differentiation	GO:0010830	7.08E-02	1.45E-01	Ccl8,20307:up|Cxcl9,17329:up|Cxcl12,20315:up
regulation of immunoglobulin production	GO:0002637	7.08E-02	1.45E-01	Ceacam1,26365:up|Tnf,21926:up|Tfrc,22042:up
positive regulation of myeloid leukocyte differentiation	GO:0002763	7.08E-02	1.45E-01	Tnf,21926:up|Ccl3,20302:up|Kitl,17311:up
pigment metabolic process	GO:0042440	7.08E-02	1.45E-01	Hmox1,15368:up|Wnt5a,22418:down|Blvrb,233016:up
mucopolysaccharide metabolic process	GO:1903510	7.08E-02	1.45E-01	Mamdc2,71738:down|Chst11,58250:down|Has2,15117:down
aromatic compound catabolic process	GO:0019439	7.10E-02	1.45E-01	Hmox1,15368:up|Enpp5,83965:up|Igf1,16000:up|Hal,15109:up|Htr2a,15558:down|Igfbp3,16009:up|Blvrb,233016:up|Enpp1,18605:down|Enpp3,209558:down
fatty acid metabolic process	GO:0006631	7.16E-02	1.46E-01	Ggt5,23887:up|Pparg,19016:up|Hpgd,15446:up|Lpl,16956:up|C3,12266:up|Ptgs2,19225:down|Adipoq,11450:up|Fabp4,11770:up|Fam132b,227358:down|Prkar2b,19088:up
male sex differentiation	GO:0046661	7.25E-02	1.48E-01	Sfrp2,20319:up|Sfrp1,20377:up|Wnt5a,22418:down|Inhba,16323:down|Bmpr1a,12166:down
kidney morphogenesis	GO:0060993	7.28E-02	1.48E-01	Vegfa,22339:down|Npnt,114249:up|Grem1,23892:up|Bmp4,12159:up
negative regulation of sequestering of calcium ion	GO:0051283	7.28E-02	1.48E-01	Htr2b,15559:down|Glp1r,14652:down|Tgfb2,21808:down|Htr2a,15558:down

release of sequestered calcium ion into cytosol	GO:0051209	7.28E-02	1.48E-01	Htr2b,15559:down|Glp1r,14652:down|Tgfb2,21808:down|Htr2a,15558:down
response to peptide hormone	GO:0043434	7.32E-02	1.49E-01	Pparg,19016:up|Igf1,16000:up|Adipoq,11450:up|Serpina3g,20715:up|Gatm,67092:down|Igf2,16002:up|Enpp1,18605:down|Cdo1,12583:up
columnar/cuboidal epithelial cell development	GO:0002066	7.40E-02	1.51E-01	Fgfr1,14182:down|Wnt5a,22418:down|Bmp4,12159:up
extracellular matrix disassembly	GO:0022617	7.44E-02	1.51E-01	Tgfb2,21808:down|Mmp13,17386:up
regulation of B cell differentiation	GO:0045577	7.44E-02	1.51E-01	Cd24a,12484:down|Sfrp1,20377:up
positive regulation of ATP metabolic process	GO:1903580	7.44E-02	1.51E-01	Htr2a,15558:down|Igf1,16000:up
establishment of epithelial cell polarity	GO:0090162	7.44E-02	1.51E-01	Ptk7,71461:down|Wnt5a,22418:down
pri-miRNA transcription from RNA polymerase II promoter	GO:0061614	7.44E-02	1.51E-01	Bmpr1a,12166:down|Bmp4,12159:up
negative regulation of neural precursor cell proliferation	GO:2000178	7.44E-02	1.51E-01	Cd24a,12484:down|Wnt5a,22418:down
response to type I interferon	GO:0034340	7.44E-02	1.51E-01	Wnt5a,22418:down|Ifit1,15957:up
positive regulation of nucleoside metabolic process	GO:0045979	7.44E-02	1.51E-01	Htr2a,15558:down|Igf1,16000:up
negative regulation of cytokine biosynthetic process	GO:0042036	7.44E-02	1.51E-01	Prg4,96875:down|Errfi1,74155:down
zinc ion homeostasis	GO:0055069	7.44E-02	1.51E-01	Mt2,17750:up|Mt1,17748:up
melanocyte differentiation	GO:0030318	7.44E-02	1.51E-01	Ednrb,13618:up|Kitl,17311:up
metanephros morphogenesis	GO:0003338	7.44E-02	1.51E-01	Grem1,23892:up|Bmp4,12159:up
carboxylic acid biosynthetic process	GO:0046394	7.45E-02	1.51E-01	Ggt5,23887:up|Akr1c18,105349:up|Lpl,16956:up|Aldh1a2,19378:up|Ptgs2,19225:down|Gatm,67092:down|Aldh1a3,56847:down|Cdo1,12583:up
organic acid biosynthetic process	GO:0016053	7.45E-02	1.51E-01	Ggt5,23887:up|Akr1c18,105349:up|Lpl,16956:up|Aldh1a2,19378:up|Ptgs2,19225:down|Gatm,67092:down|Aldh1a3,56847:down|Cdo1,12583:up
regulation of sequestering of calcium ion	GO:0051282	7.52E-02	1.52E-01	Htr2b,15559:down|Glp1r,14652:down|Tgfb2,21808:down|Htr2a,15558:down
nephron tubule development	GO:0072080	7.52E-02	1.52E-01	Vegfa,22339:down|Npnt,114249:up|Grem1,23892:up|Bmp4,12159:up
calcium ion transmembrane import into cytosol	GO:0097553	7.52E-02	1.52E-01	Htr2b,15559:down|Glp1r,14652:down|Tgfb2,21808:down|Htr2a,15558:down
calcium ion import into cytosol	GO:1902656	7.52E-02	1.52E-01	Htr2b,15559:down|Glp1r,14652:down|Tgfb2,21808:down|Htr2a,15558:down
peptidyl-serine modification	GO:0018209	7.59E-02	1.54E-01	Mamdc2,71738:down|Tnf,21926:up|Gas6,14456:up|Plk2,20620:down|Wnt5a,22418:down|Sfrp2,20319:up|Ret,19713:up|Vegfa,22339:down
peptide transport	GO:0015833	7.60E-02	1.54E-01	Tnf,21926:up|Pparg,19016:up|Glp1r,14652:down|Egfr,13649:down|Sfrp1,20377:up|C1qtnf3,81799:down|Ptgs2,19225:down|Il1rn,16181:up|Grem1,23892:up
hormone-mediated signaling pathway	GO:0009755	7.75E-02	1.57E-01	Vdr,22337:down|Pparg,19016:up|Sfrp1,20377:up|Igf1,16000:up|Bmp4,12159:up|Adipoq,11450:up
female pregnancy	GO:0007565	7.77E-02	1.57E-01	Vdr,22337:down|Hpgd,15446:up|Ptgs2,19225:down|Mmp2,17390:down
sequestering of calcium ion	GO:0051208	7.77E-02	1.57E-01	Htr2b,15559:down|Glp1r,14652:down|Tgfb2,21808:down|Htr2a,15558:down
ERBB signaling pathway	GO:0038127	7.77E-02	1.57E-01	Ceacam1,26365:up|Egfr,13649:down|Efemp1,216616:up|Errfi1,74155:down
phenol-containing compound metabolic process	GO:0018958	7.77E-02	1.57E-01	Wnt5a,22418:down|Tgfb2,21808:down|Maob,109731:up|Epas1,13819:up
neuron projection morphogenesis	GO:0048812	7.80E-02	1.57E-01	Flrt2,399558:down|Lama2,16773:down|Egfr,13649:down|Plxnd1,67784:up|Wnt5a,22418:down|Sema7a,20361:down|Tnfrsf12a,27279:down|Crabp2,12904:down|Ret,19713:up|Cxcl12,20315:up|Postn,50706:down|Vegfa,22339:down|Tgfb2,21808:down|Fgfr2,14183:up
regulation of stem cell differentiation	GO:2000736	7.85E-02	1.58E-01	Sfrp2,20319:up|Sfrp1,20377:up|Tgfb2,21808:down|Tgfbr3,21814:up|Grem1,23892:up
negative regulation of macromolecule biosynthetic process	GO:0010558	7.93E-02	1.60E-01	Sfrp4,20379:up|Ednrb,13618:up|Epas1,13819:up|Ankrd1,107765:down|Vdr,22337:down|Fabp4,11770:up|Vegfa,22339:down|Gas6,14456:up|Pparg,19016:up|Tnf,21926:up|Loxl2,94352:down|Loxl3,16950:down|Lmcd1,30937:up|Fgfr1,14182:down|Egfr,13649:down|Hmox1,15368:up|Sfrp1,20377:up|Wnt5a,22418:down|Ccl4,20303:up|Grem1,23892:up|Adipoq,11450:up|Bmp4,12159:up|Sfrp2,20319:up|Prg4,96875:down|Errfi1,74155:down|Enpp1,18605:down|Fgfr2,14183:up|S100a1,20193:up
endocardial cushion morphogenesis	GO:0003203	7.97E-02	1.60E-01	Tgfb2,21808:down|Bmpr1a,12166:down
positive regulation of protein tyrosine kinase activity	GO:0061098	7.97E-02	1.60E-01	Gas6,14456:up|Grem1,23892:up

peptide catabolic process	GO:0043171	7.97E-02	1.60E-01	Mmp13,17386:up|Anpep,16790:up
enteroendocrine cell differentiation	GO:0035883	7.97E-02	1.60E-01	Wnt5a,22418:down|Bmp4,12159:up
heme metabolic process	GO:0042168	7.97E-02	1.60E-01	Hmox1,15368:up|Blvrb,233016:up
activation of JUN kinase activity	GO:0007257	7.97E-02	1.60E-01	Trf,22041:up|Wnt5a,22418:down
regulation of alpha-beta T cell proliferation	GO:0046640	7.97E-02	1.60E-01	Ceacam1,26365:up|Cd24a,12484:down
regulation of nitric-oxide synthase activity	GO:0050999	7.97E-02	1.60E-01	Htr2b,15559:down|Egfr,13649:down
positive regulation of calcium ion transmembrane transporter activity	GO:1901021	7.97E-02	1.60E-01	S100a1,20193:up|Bmp4,12159:up
interleukin-1 beta secretion	GO:0050702	7.97E-02	1.60E-01	Wnt5a,22418:down|Ccl3,20302:up
branching involved in mammary gland duct morphogenesis	GO:0060444	7.97E-02	1.60E-01	Vdr,22337:down|Wnt5a,22418:down
establishment or maintenance of epithelial cell apical/basal polarity	GO:0045197	7.97E-02	1.60E-01	Ptk7,71461:down|Wnt5a,22418:down
regulation of macrophage activation	GO:0043030	7.97E-02	1.60E-01	Wnt5a,22418:down|Il1rl1,17082:down
aminoglycan metabolic process	GO:0006022	8.02E-02	1.61E-01	Mamdc2,71738:down|Chst11,58250:down|Has2,15117:down|Igf1,16000:up
somite development	GO:0061053	8.02E-02	1.61E-01	Sfrp2,20319:up|Sfrp1,20377:up|Wnt5a,22418:down|Bmpr1a,12166:down
endocrine hormone secretion	GO:0060986	8.06E-02	1.62E-01	Fgfr1,14182:down|C1qtnf3,81799:down|Inhba,16323:down
positive regulation of gliogenesis	GO:0014015	8.06E-02	1.62E-01	Pparg,19016:up|Trf,22041:up|Serpine2,20720:up
negative regulation of cellular component organization	GO:0051129	8.12E-02	1.63E-01	Tnf,21926:up|Plk2,20620:down|Efemp1,216616:up|Sfrp1,20377:up|Wnt5a,22418:down|Tgfbr3,21814:up|Dpysl3,22240:down|Igf1,16000:up|Bmp4,12159:up|Adipoq,11450:up|Sfrp2,20319:up|Postn,50706:down|Tgfb2,21808:down|Lrrc15,74488:down|Fgfr2,14183:up
negative regulation of RNA biosynthetic process	GO:1902679	8.22E-02	1.65E-01	Sfrp4,20379:up|Ednrb,13618:up|Epas1,13819:up|Ankrd1,107765:down|Vdr,22337:down|Fabp4,11770:up|Vegfa,22339:down|Gas6,14456:up|Pparg,19016:up|Tnf,21926:up|Loxl2,94352:down|Loxl3,16950:down|Lmcd1,30937:up|Fgfr1,14182:down|Hmox1,15368:up|Sfrp1,20377:up|Wnt5a,22418:down|Ccl4,20303:up|Grem1,23892:up|Adipoq,11450:up|Bmp4,12159:up|Sfrp2,20319:up|Fgfr2,14183:up|S100a1,20193:up
negative regulation of leukocyte cell-cell adhesion	GO:1903038	8.28E-02	1.66E-01	Ceacam1,26365:up|Cd24a,12484:down|Cxcl12,20315:up|Bmp4,12159:up
negative regulation of developmental growth	GO:0048640	8.28E-02	1.66E-01	Sfrp2,20319:up|Sfrp1,20377:up|Wnt5a,22418:down|Bmp4,12159:up
interleukin-8 production	GO:0032637	8.39E-02	1.67E-01	Tnf,21926:up|Wnt5a,22418:down|Adipoq,11450:up
negative regulation of cell division	GO:0051782	8.39E-02	1.67E-01	Susd2,71733:up|Fgfr2,14183:up|Bmp4,12159:up
negative regulation of ERK1 and ERK2 cascade	GO:0070373	8.39E-02	1.67E-01	Errfi1,74155:down|Igf1,16000:up|Adipoq,11450:up
cell-cell junction organization	GO:0045216	8.47E-02	1.67E-01	Ceacam1,26365:up|Tnf,21926:up|Gpm6b,14758:up|Vegfa,22339:down|Tgfb2,21808:down|Grem1,23892:up
negative regulation of interferon-gamma production	GO:0032689	8.51E-02	1.67E-01	Gas6,14456:up|Il1rl1,17082:down
positive regulation of osteoclast differentiation	GO:0045672	8.51E-02	1.67E-01	Tnf,21926:up|Ccl3,20302:up
inositol phosphate-mediated signaling	GO:0048016	8.51E-02	1.67E-01	Lmcd1,30937:up|Igf1,16000:up
antimicrobial humoral response	GO:0019730	8.51E-02	1.67E-01	Adm,11535:up|Slpi,20568:up
regulation of myeloid cell apoptotic process	GO:0033032	8.51E-02	1.67E-01	Kitl,17311:up|Adipoq,11450:up
response to zinc ion	GO:0010043	8.51E-02	1.67E-01	Hvcn1,74096:up|Mt1,17748:up
regulation of T cell migration	GO:2000404	8.51E-02	1.67E-01	Cxcl12,20315:up|Wnt5a,22418:down
acylglycerol homeostasis	GO:0055090	8.51E-02	1.67E-01	Lpl,16956:up|C1qtnf3,81799:down
triglyceride homeostasis	GO:0070328	8.51E-02	1.67E-01	Lpl,16956:up|C1qtnf3,81799:down
positive regulation of interleukin-1 production	GO:0032732	8.51E-02	1.67E-01	Wnt5a,22418:down|Ccl3,20302:up

cellular component morphogenesis	GO:0032989	8.52E-02	1.67E-01	Fam101b,76566:down|Flrt2,399558:down|Lama2,16773:down|Plxnd1,67784:up|Ccl2,20296:up|Ccl3,20302:up|Sema7a,20361:down|Tnfrsf12a,27279:down|Vdr,22337:down|Cxcl12,20315:up|Postn,50706:down|Vegfa,22339:down|Loxl2,94352:down|Loxl3,16950:down|Egfr,13649:down|Sfrp1,20377:up|Has2,15117:down|Wnt5a,22418:down|Ccl12,20293:up|Tgfbr3,21814:up|Stc1,20855:up|Grem1,23892:up|Bmp4,12159:up|Sfrp2,20319:up|Crabp2,12904:down|Ret,19713:up|Tgfb2,21808:down|Fgfr2,14183:up
regulation of cellular biosynthetic process	GO:0031326	8.53E-02	1.67E-01	Fstl3,83554:down|Adm,11535:up|Tfec,21426:up|Sfrp4,20379:up|C3,12266:up|Ankrd1,107765:down|Lyl1,17095:up|Loxl2,94352:down|Loxl3,16950:down|Trf,22041:up|Wnt5a,22418:down|Adipoq,11450:up|Bmp4,12159:up|Igf1,16000:up|Ptgir,19222:up|Fgfr2,14183:up|Htr2b,15559:down|Prl2c2,18811:up|Efemp1,216616:up|Vdr,22337:down|Fabp4,11770:up|Htr2a,15558:down|Slc40a1,53945:up|Gas6,14456:up|Egfr,13649:down|Sfrp1,20377:up|C1qtnf3,81799:down|Tgfbr3,21814:up|Sfrp2,20319:up|Npnt,114249:up|S100a1,20193:up|Ice1,218333:down|Ednrb,13618:up|Pdgfc,54635:down|Epas1,13819:up|Uchl5,56207:up|Htra3,78558:down|Ddah1,69219:down|Sdpr,20324:down|Ptx3,19288:up|Akr1c18,105349:up|Bmpr1a,12166:down|Ccl4,20303:up|Ret,19713:up|Prg4,96875:down|Plpp3,67916:up|Creb3l1,26427:down|Kitl,17311:up|Rasl11a,68895:up|Inhba,16323:down|Bnc2,242509:down|Ccl3,20302:up|Vegfa,22339:down|Igf2,16002:up|Tnf,21926:up|Pparg,19016:up|Lmcd1,30937:up|Glp1r,14652:down|Fgfr1,14182:down|Hmox1,15368:up|Sulf1,240725:down|Ptgs2,19225:down|Grem1,23892:up|Phf11d,219132:up|Tbx15,21384:down|Enpp1,18605:down|Errfi1,74155:down
cell killing	GO:0001906	8.54E-02	1.67E-01	Ceacam1,26365:up|Clec2d,93694:up|Ccl2,20296:up|Igf2,16002:up
renal tubule development	GO:0061326	8.54E-02	1.67E-01	Vegfa,22339:down|Npnt,114249:up|Grem1,23892:up|Bmp4,12159:up
positive regulation of hemopoiesis	GO:1903708	8.66E-02	1.67E-01	Tnf,21926:up|Gas6,14456:up|Inhba,16323:down|Ccl3,20302:up|Tnfsf9,21950:up|Kitl,17311:up
organophosphate catabolic process	GO:0046434	8.70E-02	1.67E-01	Htr2a,15558:down|Enpp1,18605:down|Enpp3,209558:down|Enpp5,83965:up|Igf1,16000:up
positive regulation of B cell apoptotic process	GO:0002904	8.72E-02	1.67E-01	Cd24a,12484:down
atrial septum primum morphogenesis	GO:0003289	8.72E-02	1.67E-01	Tgfb2,21808:down
bone marrow development	GO:0048539	8.72E-02	1.67E-01	Lrrc17,74511:down
mammary gland branching involved in pregnancy	GO:0060745	8.72E-02	1.67E-01	Vdr,22337:down
ureter smooth muscle cell differentiation	GO:0072193	8.72E-02	1.67E-01	Bmp4,12159:up
positive regulation of MAPKKK cascade by fibroblast growth factor receptor signaling pathway	GO:0090080	8.72E-02	1.67E-01	Fgfr1,14182:down
negative regulation of hair follicle development	GO:0051799	8.72E-02	1.67E-01	Inhba,16323:down
positive regulation of protein kinase A signaling	GO:0010739	8.72E-02	1.67E-01	Adipoq,11450:up
positive regulation of integrin activation	GO:0033625	8.72E-02	1.67E-01	Cd24a,12484:down
9-cis-retinoic acid metabolic process	GO:0042905	8.72E-02	1.67E-01	Aldh1a2,19378:up
regulation of inorganic anion transmembrane transport	GO:1903795	8.72E-02	1.67E-01	Fxyd1,56188:up
positive regulation of nuclear cell cycle DNA replication	GO:0010571	8.72E-02	1.67E-01	Fgfr1,14182:down
endocardial cushion to mesenchymal transition	GO:0090500	8.72E-02	1.67E-01	Has2,15117:down
negative regulation of hydrogen peroxide metabolic process	GO:0010727	8.72E-02	1.67E-01	Hp,15439:up
osteoclast fusion	GO:0072675	8.72E-02	1.67E-01	Cd109,235505:down
positive regulation of retinoic acid receptor signaling pathway	GO:0048386	8.72E-02	1.67E-01	Aldh1a3,56847:down
positive regulation of brown fat cell differentiation	GO:0090336	8.72E-02	1.67E-01	Ptgs2,19225:down
skeletal muscle satellite cell maintenance involved in skeletal muscle regeneration	GO:0014834	8.72E-02	1.67E-01	Igf1,16000:up
regulation of mast cell chemotaxis	GO:0060753	8.72E-02	1.67E-01	Figf,14205:up
negative regulation by host of viral genome replication	GO:0044828	8.72E-02	1.67E-01	Ceacam1,26365:up

serotonin uptake	GO:0051610	8.72E-02	1.67E-01	Gpm6b,14758:up
negative regulation of tumor necrosis factor biosynthetic process	GO:0042536	8.72E-02	1.67E-01	Errfi1,74155:down
regulation of cardiac muscle adaptation	GO:0010612	8.72E-02	1.67E-01	Errfi1,74155:down
negative regulation of cytokine secretion involved in immune response	GO:0002740	8.72E-02	1.67E-01	Tnf,21926:up
urinary bladder development	GO:0060157	8.72E-02	1.67E-01	Wnt5a,22418:down
positive regulation of cell proliferation involved in heart morphogenesis	GO:2000138	8.72E-02	1.67E-01	Bmp4,12159:up
modulation of molecular function in other organism involved in symbiotic interaction	GO:0052205	8.72E-02	1.67E-01	Ptx3,19288:up
apoptotic process in bone marrow	GO:0071839	8.72E-02	1.67E-01	Fgfr2,14183:up
positive regulation of alkaline phosphatase activity	GO:0010694	8.72E-02	1.67E-01	Npnt,114249:up
ureter smooth muscle development	GO:0072191	8.72E-02	1.67E-01	Bmp4,12159:up
T cell homeostatic proliferation	GO:0001777	8.72E-02	1.67E-01	Cd24a,12484:down
parathyroid hormone secretion	GO:0035898	8.72E-02	1.67E-01	Fgfr1,14182:down
cell migration involved in kidney development	GO:0035787	8.72E-02	1.67E-01	Adipoq,11450:up
positive regulation of branching involved in lung morphogenesis	GO:0061047	8.72E-02	1.67E-01	Bmp4,12159:up
regulation of secondary metabolite biosynthetic process	GO:1900376	8.72E-02	1.67E-01	Wnt5a,22418:down
pulmonary artery morphogenesis	GO:0061156	8.72E-02	1.67E-01	Bmp4,12159:up
ferrous iron import	GO:0070627	8.72E-02	1.67E-01	Trf,22041:up
positive regulation of histone deacetylase activity	GO:1901727	8.72E-02	1.67E-01	Vegfa,22339:down
vascular wound healing	GO:0061042	8.72E-02	1.67E-01	Hpse,15442:up
negative regulation of monocyte chemotaxis	GO:0090027	8.72E-02	1.67E-01	Grem1,23892:up
direct ossification	GO:0036072	8.72E-02	1.67E-01	Mmp2,17390:down
nitrate metabolic process	GO:0042126	8.72E-02	1.67E-01	Marc1,66112:up
positive regulation of CD8-positive, alpha-beta T cell activation	GO:2001187	8.72E-02	1.67E-01	Ceacam1,26365:up
negative regulation of serotonin secretion	GO:0014063	8.72E-02	1.67E-01	Maob,109731:up
negative regulation of molecular function in other organism	GO:0044362	8.72E-02	1.67E-01	Ptx3,19288:up
regulation of melanin biosynthetic process	GO:0048021	8.72E-02	1.67E-01	Wnt5a,22418:down
positive regulation of cardioblast differentiation	GO:0051891	8.72E-02	1.67E-01	Tgfb2,21808:down
mesenchymal cell proliferation involved in lung development	GO:0060916	8.72E-02	1.67E-01	Fgfr2,14183:up
S-shaped body morphogenesis	GO:0072050	8.72E-02	1.67E-01	Bmp4,12159:up
NADH oxidation	GO:0006116	8.72E-02	1.67E-01	Cbr2,12409:up
intramembranous ossification	GO:0001957	8.72E-02	1.67E-01	Mmp2,17390:down
extracellular matrix constituent secretion	GO:0070278	8.72E-02	1.67E-01	Creb3l1,26427:down

negative regulation of glycogen biosynthetic process	GO:0045719	8.72E-02	1.67E-01	Enpp1,18605:down
mast cell apoptotic process	GO:0033024	8.72E-02	1.67E-01	Kitl,17311:up
negative regulation of myosin-light-chain-phosphatase activity	GO:0035509	8.72E-02	1.67E-01	Tnf,21926:up
positive regulation of hyaluronan biosynthetic process	GO:1900127	8.72E-02	1.67E-01	Has2,15117:down
epithelial cell proliferation involved in mammary gland duct elongation	GO:0060750	8.72E-02	1.67E-01	Wnt5a,22418:down
cell proliferation involved in outflow tract morphogenesis	GO:0061325	8.72E-02	1.67E-01	Bmp4,12159:up
tricuspid valve morphogenesis	GO:0003186	8.72E-02	1.67E-01	Bmpr1a,12166:down
positive regulation of cell proliferation in bone marrow	GO:0071864	8.72E-02	1.67E-01	Fgfr2,14183:up
branch elongation involved in ureteric bud branching	GO:0060681	8.72E-02	1.67E-01	Bmp4,12159:up
negative regulation of hepatocyte proliferation	GO:2000346	8.72E-02	1.67E-01	Ceacam1,26365:up
mast cell homeostasis	GO:0033023	8.72E-02	1.67E-01	Kitl,17311:up
negative regulation of oocyte development	GO:0060283	8.72E-02	1.67E-01	Igf1,16000:up
negative regulation of immature T cell proliferation in thymus	GO:0033088	8.72E-02	1.67E-01	Bmp4,12159:up
negative regulation of molecular function in other organism involved in symbiotic interaction	GO:0052204	8.72E-02	1.67E-01	Ptx3,19288:up
positive regulation of neuron maturation	GO:0014042	8.72E-02	1.67E-01	Ret,19713:up
modulation of molecular function in other organism	GO:0044359	8.72E-02	1.67E-01	Ptx3,19288:up
creatine metabolic process	GO:0006600	8.72E-02	1.67E-01	Gatm,67092:down
iron ion transmembrane transport	GO:0034755	8.72E-02	1.67E-01	Slc40a1,53945:up
negative regulation of cellular response to vascular endothelial growth factor stimulus	GO:1902548	8.72E-02	1.67E-01	Adamts12,239337:down
positive regulation of transcription from RNA polymerase II promoter in response to hypoxia	GO:0061419	8.72E-02	1.67E-01	Vegfa,22339:down
pyrimidine nucleoside monophosphate biosynthetic process	GO:0009130	8.72E-02	1.67E-01	Uck2,80914:down
neural crest formation	GO:0014029	8.72E-02	1.67E-01	Sfrp1,20377:up
dichotomous subdivision of terminal units involved in salivary gland branching	GO:0060666	8.72E-02	1.67E-01	Plxnd1,67784:up
positive regulation of endothelial cell chemotaxis by VEGF-activated vascular endothelial growth factor receptor signaling pathway	GO:0038033	8.72E-02	1.67E-01	Vegfa,22339:down

canonical Wnt signaling pathway involved in regulation of cell proliferation	GO:0044340	8.72E-02	1.67E-01	Sfrp1,20377:up
BMP signaling pathway involved in heart development	GO:0061312	8.72E-02	1.67E-01	Bmp4,12159:up
sclerotome development	GO:0061056	8.72E-02	1.67E-01	Sfrp2,20319:up
negative regulation of fibroblast growth factor production	GO:0090272	8.72E-02	1.67E-01	Fgfr1,14182:down
negative regulation of synaptic transmission, dopaminergic	GO:0032227	8.72E-02	1.67E-01	Ptgs2,19225:down
cellular response to nicotine	GO:0071316	8.72E-02	1.67E-01	Tnf,21926:up
positive regulation of fibroblast apoptotic process	GO:2000271	8.72E-02	1.67E-01	Sfrp1,20377:up
purine nucleotide salvage	GO:0032261	8.72E-02	1.67E-01	Ampd3,11717:up
negative regulation of sequestering of triglyceride	GO:0010891	8.72E-02	1.67E-01	Pparg,19016:up
positive regulation of store-operated calcium channel activity	GO:1901341	8.72E-02	1.67E-01	Bmp4,12159:up
positive regulation of neuroepithelial cell differentiation	GO:1902913	8.72E-02	1.67E-01	Kitl,17311:up
regulation of integrin biosynthetic process	GO:0045113	8.72E-02	1.67E-01	Tgfb2,21808:down
regulation of cell proliferation in bone marrow	GO:0071863	8.72E-02	1.67E-01	Fgfr2,14183:up
regulation of mast cell apoptotic process	GO:0033025	8.72E-02	1.67E-01	Kitl,17311:up
9-cis-retinoic acid biosynthetic process	GO:0042904	8.72E-02	1.67E-01	Aldh1a2,19378:up
negative regulation of cell proliferation involved in kidney development	GO:1901723	8.72E-02	1.67E-01	Bmp4,12159:up
intestine smooth muscle contraction	GO:0014827	8.72E-02	1.67E-01	Htr2b,15559:down
regulation of CD4-positive, alpha-beta T cell proliferation	GO:2000561	8.72E-02	1.67E-01	Ceacam1,26365:up
histidine catabolic process	GO:0006548	8.72E-02	1.67E-01	Hal,15109:up
CD4-positive, alpha-beta T cell proliferation	GO:0035739	8.72E-02	1.67E-01	Ceacam1,26365:up
thelarche	GO:0042695	8.72E-02	1.67E-01	Wnt5a,22418:down
mammary gland branching involved in thelarche	GO:0060744	8.72E-02	1.67E-01	Wnt5a,22418:down
terpenoid catabolic process	GO:0016115	8.72E-02	1.67E-01	Akr1c18,105349:up
nephric duct formation	GO:0072179	8.72E-02	1.67E-01	Bmp4,12159:up
monocyte aggregation	GO:0070487	8.72E-02	1.67E-01	Has2,15117:down
positive regulation of chronic inflammatory response	GO:0002678	8.72E-02	1.67E-01	Tnf,21926:up
positive regulation of phosphatidylinositol 3-kinase signaling	GO:0014068	8.74E-02	1.67E-01	Tgfb2,21808:down|Pdgfc,54635:down|Igf1,16000:up
activation of cysteine-type endopeptidase activity involved in apoptotic process	GO:0006919	8.74E-02	1.67E-01	Tnf,21926:up|Pparg,19016:up|F3,14066:up
regulation of cytokine biosynthetic process	GO:0042035	8.80E-02	1.68E-01	Tnf,21926:up|Prg4,96875:down|Wnt5a,22418:down|Errfi1,74155:down
interleukin-6 secretion	GO:0072604	9.05E-02	1.72E-01	Gas6,14456:up|C1qtnf3,81799:down

negative regulation of amine transport	GO:0051953	9.05E-02	1.72E-01	Tnf,21926:up|Il1rn,16181:up
positive regulation of cytokine-mediated signaling pathway	GO:0001961	9.05E-02	1.72E-01	Gas6,14456:up|Wnt5a,22418:down
positive regulation of response to cytokine stimulus	GO:0060760	9.05E-02	1.72E-01	Gas6,14456:up|Wnt5a,22418:down
hair follicle morphogenesis	GO:0031069	9.05E-02	1.72E-01	Tgfb2,21808:down|Fgfr2,14183:up
prostaglandin metabolic process	GO:0006693	9.05E-02	1.72E-01	Hpgd,15446:up|Ptgs2,19225:down
prostanoid metabolic process	GO:0006692	9.05E-02	1.72E-01	Hpgd,15446:up|Ptgs2,19225:down
response to interferon-beta	GO:0035456	9.05E-02	1.72E-01	Ifit1,15957:up|Ifit3,15959:up
motor neuron axon guidance	GO:0008045	9.05E-02	1.72E-01	Plxnd1,67784:up|Cxcl12,20315:up
protein processing	GO:0016485	9.07E-02	1.73E-01	Tnf,21926:up|Pparg,19016:up|C3,12266:up|Plat,18791:down|Ctla2a,13024:down|Serping1,12258:up|Serpine2,20720:up|F3,14066:up
regulation of calcium ion import	GO:0090279	9.07E-02	1.73E-01	Cxcl12,20315:up|Tgfb2,21808:down|Ccl12,20293:up|Stc1,20855:up
cerebral cortex development	GO:0021987	9.07E-02	1.73E-01	Fgfr1,14182:down|Egfr,13649:down|Cx3cr1,13051:down|Col3a1,12825:down
negative regulation of leukocyte activation	GO:0002695	9.14E-02	1.74E-01	Ceacam1,26365:up|Cd24a,12484:down|Sfrp1,20377:up|Hmox1,15368:up|Bmp4,12159:up
nucleoside phosphate metabolic process	GO:0006753	9.22E-02	1.75E-01	Htr2b,15559:down|Adm,11535:up|Wnt5a,22418:down|Ampd3,11717:up|Enpp5,83965:up|Igf1,16000:up|Vegfa,22339:down|Gfpt2,14584:down|Htr2a,15558:down|Cbr2,12409:up|Ptgir,19222:up|Uck2,80914:down|Enpp1,18605:down|Enpp3,209558:down
female gamete generation	GO:0007292	9.34E-02	1.77E-01	Inhba,16323:down|Ptgs2,19225:down|Plat,18791:down|Igf1,16000:up
steroid biosynthetic process	GO:0006694	9.37E-02	1.78E-01	Vdr,22337:down|Tnf,21926:up|Akr1c18,105349:up|Igf2,16002:up|Igf1,16000:up
cell division	GO:0051301	9.41E-02	1.79E-01	Htr2b,15559:down|Tnf,21926:up|Fgfr1,14182:down|Wnt5a,22418:down|Susd2,71733:up|Pdgfc,54635:down|Figf,14205:up|Igf1,16000:up|Bmp4,12159:up|Sfrp2,20319:up|Vegfa,22339:down|Tgfb2,21808:down|Vegfb,22340:up|Fgfr2,14183:up|Igf2,16002:up
monocarboxylic acid biosynthetic process	GO:0072330	9.43E-02	1.79E-01	Ggt5,23887:up|Akr1c18,105349:up|Lpl,16956:up|Aldh1a2,19378:up|Aldh1a3,56847:down|Ptgs2,19225:down
regulation of purine nucleotide metabolic process	GO:1900542	9.43E-02	1.79E-01	Adm,11535:up|Wnt5a,22418:down|Vegfa,22339:down|Htr2a,15558:down|Ptgir,19222:up|Igf1,16000:up
long-term synaptic potentiation	GO:0060291	9.45E-02	1.79E-01	Plk2,20620:down|Cx3cr1,13051:down|Serpine2,20720:up
response to cocaine	GO:0042220	9.61E-02	1.82E-01	Ncam1,17967:down|Htr2a,15558:down
embryonic hindlimb morphogenesis	GO:0035116	9.61E-02	1.82E-01	Rspo2,239405:up|Bmp4,12159:up
epithelial cell differentiation involved in kidney development	GO:0035850	9.61E-02	1.82E-01	Bmp4,12159:up|Adipoq,11450:up
positive regulation of smooth muscle contraction	GO:0045987	9.61E-02	1.82E-01	Ptgs2,19225:down|Npnt,114249:up
response to pain	GO:0048265	9.61E-02	1.82E-01	Ret,19713:up|Ednrb,13618:up
regulation of heart morphogenesis	GO:2000826	9.61E-02	1.82E-01	Tgfb2,21808:down|Bmp4,12159:up
negative regulation of lymphocyte proliferation	GO:0050672	9.81E-02	1.85E-01	Ceacam1,26365:up|Cd24a,12484:down|Bmp4,12159:up
negative regulation of mononuclear cell proliferation	GO:0032945	9.81E-02	1.85E-01	Ceacam1,26365:up|Cd24a,12484:down|Bmp4,12159:up
cell-substrate adherens junction assembly	GO:0007045	9.81E-02	1.85E-01	Gpm6b,14758:up|Vegfa,22339:down|Grem1,23892:up
focal adhesion assembly	GO:0048041	9.81E-02	1.85E-01	Gpm6b,14758:up|Vegfa,22339:down|Grem1,23892:up
positive regulation of interferon-gamma production	GO:0032729	9.81E-02	1.85E-01	Tnf,21926:up|Wnt5a,22418:down|Tnfsf9,21950:up
amide transport	GO:0042886	9.81E-02	1.85E-01	Tnf,21926:up|Pparg,19016:up|Glp1r,14652:down|Egfr,13649:down|Sfrp1,20377:up|C1qtnf3,81799:down|Ptgs2,19225:down|Il1rn,16181:up|Grem1,23892:up
central nervous system neuron differentiation	GO:0021953	9.82E-02	1.85E-01	Sfrp2,20319:up|Fgfr1,14182:down|Sfrp1,20377:up|Wnt5a,22418:down|Inhba,16323:down|Fgfr2,14183:up
positive regulation of leukocyte differentiation	GO:1902107	9.83E-02	1.85E-01	Tnf,21926:up|Gas6,14456:up|Ccl3,20302:up|Tnfsf9,21950:up|Kitl,17311:up

single organism reproductive process	GO:0044702	9.89E-02	1.87E-01	Adm,11535:up|Inhba,16323:down|Gjb5,14622:down|Ednrb,13618:up|Plat,18791:down|Epas1,13819:up|Vdr,22337:down|Cxcl12,20315:up|Vegfa,22339:down|Mmp2,17390:down|Serpine2,20720:up|Pparg,19016:up|Egfr,13649:down|Sfrp1,20377:up|Sulf1,240725:down|Wnt5a,22418:down|Bmpr1a,12166:down|Ptgs2,19225:down|Bmp4,12159:up|Igf1,16000:up|Sfrp2,20319:up|Tnc,21923:down|Fgfr2,14183:up|Kitl,17311:up
cellular component organization	GO:0016043	1.00E-01	1.89E-01	Igfbp4,16010:up|Adm,11535:up|Flrt2,399558:down|Plxnd1,67784:up|Sfrp4,20379:up|C3,12266:up|Ankrd1,107765:down|Ltbp2,16997:down|Serpinh1,12406:down|Igfbp3,16009:up|Ibsp,15891:up|Loxl2,94352:down|Plk2,20620:down|Loxl3,16950:down|Trf,22041:up|Cd24a,12484:down|Wnt5a,22418:down|Mark1,226778:up|Stc1,20855:up|Adipoq,11450:up|Bmp4,12159:up|Igf1,16000:up|Crabp2,12904:down|Cxcl9,17329:up|Tgfb2,21808:down|Hpse,15442:up|Fgfr2,14183:up|Thbs2,21826:down|Cthrc1,68588:down|Prl2c2,18811:up|Efemp1,216616:up|Dpysl3,22240:down|Vdr,22337:down|Cxcl12,20315:up|Mmp2,17390:down|Smoc2,64074:down|Gas6,14456:up|Egfr,13649:down|Col11a1,12814:down|Sfrp1,20377:up|C1qtnf3,81799:down|Tgfbr3,21814:up|Sncg,20618:up|Ceacam1,26365:up|Ccl8,20307:up|Sfrp2,20319:up|Pxdn,69675:down|Npnt,114249:up|Tnfrsf11b,18383:up|Syt13,80976:up|Col5a2,12832:down|Ice1,218333:down|Ccl2,20296:up|Pdzrn3,55983:down|Pdgfc,54635:down|Mgp,17313:up|Mmp13,17386:up|Sema7a,20361:down|Epas1,13819:up|Htra3,78558:down|Tnfrsf12a,27279:down|Gpm6b,14758:up|Shroom4,208431:down|Postn,50706:down|Ptx3,19288:up|Ncam1,17967:down|Ccl12,20293:up|Wisp1,22402:down|Tnc,21923:down|Ret,19713:up|Creb3l1,26427:down|Fam101b,76566:down|P4ha1,18451:down|Lama2,16773:down|Ptk7,71461:down|Inhba,16323:down|Ccl3,20302:up|Srpx2,68792:down|Plod2,26432:down|Vegfa,22339:down|Cbr2,12409:up|Serpine2,20720:up|Lrrc15,74488:down|Igf2,16002:up|Pparg,19016:up|Tnf,21926:up|Lmcd1,30937:up|Col1a2,12843:down|Fgfr1,14182:down|Lpl,16956:up|Hmox1,15368:up|Has2,15117:down|Sulf1,240725:down|C1qtnf6,72709:down|Grem1,23892:up|Col3a1,12825:down|Col5a1,12831:down|Enpp1,18605:down
steroid hormone mediated signaling pathway	GO:0043401	1.01E-01	1.89E-01	Vdr,22337:down|Pparg,19016:up|Sfrp1,20377:up|Igf1,16000:up|Bmp4,12159:up
regulation of cellular ketone metabolic process	GO:0010565	1.01E-01	1.89E-01	Pparg,19016:up|Akr1c18,105349:up|Fam132b,227358:down|C1qtnf3,81799:down|Adipoq,11450:up
myotube differentiation	GO:0014902	1.02E-01	1.89E-01	Ccl8,20307:up|Cxcl9,17329:up|Cxcl12,20315:up|Igf1,16000:up
positive regulation of cellular carbohydrate metabolic process	GO:0010676	1.02E-01	1.89E-01	Htr2a,15558:down|Igf2,16002:up|Igf1,16000:up
hexose biosynthetic process	GO:0019319	1.02E-01	1.89E-01	Sord,20322:up|C1qtnf3,81799:down|Adipoq,11450:up
catecholamine transport	GO:0051937	1.02E-01	1.89E-01	Cxcl12,20315:up|Htr2a,15558:down|Sncg,20618:up
regulation of neuron migration	GO:2001222	1.02E-01	1.89E-01	Flrt2,399558:down|Col3a1,12825:down
triglyceride biosynthetic process	GO:0019432	1.02E-01	1.89E-01	Lpl,16956:up|C3,12266:up
alpha-beta T cell proliferation	GO:0046633	1.02E-01	1.89E-01	Ceacam1,26365:up|Cd24a,12484:down
myeloid cell apoptotic process	GO:0033028	1.02E-01	1.89E-01	Kitl,17311:up|Adipoq,11450:up
positive regulation of cytokine production involved in immune response	GO:0002720	1.02E-01	1.89E-01	Wnt5a,22418:down|Sema7a,20361:down
cell junction organization	GO:0034330	1.02E-01	1.89E-01	Ceacam1,26365:up|Tnf,21926:up|Gpm6b,14758:up|Vegfa,22339:down|Tgfb2,21808:down|Grem1,23892:up
negative regulation of macrophage differentiation	GO:0045650	1.04E-01	1.89E-01	Adipoq,11450:up
positive regulation of pigment cell differentiation	GO:0050942	1.04E-01	1.89E-01	Kitl,17311:up
chondrocyte development involved in endochondral bone morphogenesis	GO:0003433	1.04E-01	1.89E-01	Serpinh1,12406:down
retinal blood vessel morphogenesis	GO:0061304	1.04E-01	1.89E-01	Vegfa,22339:down
positive regulation of dendritic cell chemotaxis	GO:2000510	1.04E-01	1.89E-01	Gas6,14456:up
adiponectin-activated signaling pathway	GO:0033211	1.04E-01	1.89E-01	Adipoq,11450:up
necroptotic signaling pathway	GO:0097527	1.04E-01	1.89E-01	Tnf,21926:up
prolactin secretion	GO:0070459	1.04E-01	1.89E-01	Egfr,13649:down
negative regulation of motor neuron apoptotic process	GO:2000672	1.04E-01	1.89E-01	Crlf1,12931:down
doxorubicin metabolic process	GO:0044598	1.04E-01	1.89E-01	Akr1c18,105349:up
activation of MAPKKK activity	GO:0000185	1.04E-01	1.89E-01	Tnf,21926:up
lung growth	GO:0060437	1.04E-01	1.89E-01	Rspo2,239405:up

negative regulation of chronic inflammatory response	GO:0002677	1.04E-01	1.89E-01	Cx3cr1,13051:down
negative regulation of glycogen metabolic process	GO:0070874	1.04E-01	1.89E-01	Enpp1,18605:down
cell proliferation in midbrain	GO:0033278	1.04E-01	1.89E-01	Wnt5a,22418:down
ductus arteriosus closure	GO:0097070	1.04E-01	1.89E-01	Hpgd,15446:up
snRNA transcription from RNA polymerase III promoter	GO:0042796	1.04E-01	1.89E-01	Ice1,218333:down
regulation of branching involved in mammary gland duct morphogenesis	GO:0060762	1.04E-01	1.89E-01	Wnt5a,22418:down
septum primum development	GO:0003284	1.04E-01	1.89E-01	Tgfb2,21808:down
regulation of hair follicle maturation	GO:0048819	1.04E-01	1.89E-01	Tgfb2,21808:down
regulation of melanocyte differentiation	GO:0045634	1.04E-01	1.89E-01	Kitl,17311:up
regulation of stem cell division	GO:2000035	1.04E-01	1.89E-01	Sfrp2,20319:up
L-serine transport	GO:0015825	1.04E-01	1.89E-01	Serinc2,230779:down
positive regulation of myeloid cell apoptotic process	GO:0033034	1.04E-01	1.89E-01	Adipoq,11450:up
ventricular compact myocardium morphogenesis	GO:0003223	1.04E-01	1.89E-01	Bmpr1a,12166:down
testosterone biosynthetic process	GO:0061370	1.04E-01	1.89E-01	Akr1c18,105349:up
cellular triglyceride homeostasis	GO:0035356	1.04E-01	1.89E-01	C1qtnf3,81799:down
mast cell cytokine production	GO:0032762	1.04E-01	1.89E-01	Hmox1,15368:up
apoptotic process involved in heart morphogenesis	GO:0003278	1.04E-01	1.89E-01	Tgfb2,21808:down
otic vesicle formation	GO:0030916	1.04E-01	1.89E-01	Fgfr2,14183:up
negative regulation of interleukin-6 biosynthetic process	GO:0045409	1.04E-01	1.89E-01	Prg4,96875:down
negative regulation of mast cell degranulation	GO:0043305	1.04E-01	1.89E-01	Hmox1,15368:up
N-acetylneuraminate catabolic process	GO:0019262	1.04E-01	1.89E-01	Npl,74091:up
positive regulation of thymocyte apoptotic process	GO:0070245	1.04E-01	1.89E-01	Wnt5a,22418:down
polyketide metabolic process	GO:0030638	1.04E-01	1.89E-01	Akr1c18,105349:up
cGMP-mediated signaling	GO:0019934	1.04E-01	1.89E-01	Ednrb,13618:up
pre-B cell differentiation	GO:0002329	1.04E-01	1.89E-01	Cd24a,12484:down
negative regulation of hair cycle	GO:0042636	1.04E-01	1.89E-01	Inhba,16323:down
negative regulation of transforming growth factor beta production	GO:0071635	1.04E-01	1.89E-01	Cd24a,12484:down
convergent extension involved in organogenesis	GO:0060029	1.04E-01	1.89E-01	Wnt5a,22418:down
cell proliferation in bone marrow	GO:0071838	1.04E-01	1.89E-01	Fgfr2,14183:up
multinuclear osteoclast differentiation	GO:0072674	1.04E-01	1.89E-01	Cd109,235505:down
regulation of adiponectin secretion	GO:0070163	1.04E-01	1.89E-01	C1qtnf3,81799:down
embryonic neurocranium morphogenesis	GO:0048702	1.04E-01	1.89E-01	Tgfb2,21808:down
primitive erythrocyte differentiation	GO:0060319	1.04E-01	1.89E-01	Vegfa,22339:down

negative regulation of immature T cell proliferation	GO:0033087	1.04E-01	1.89E-01	Bmp4,12159:up
branch elongation involved in mammary gland duct branching	GO:0060751	1.04E-01	1.89E-01	Wnt5a,22418:down
positive regulation of anion transmembrane transport	GO:1903961	1.04E-01	1.89E-01	Fxyd1,56188:up
heart induction	GO:0003129	1.04E-01	1.89E-01	Bmp4,12159:up
non-canonical Wnt signaling pathway via JNK cascade	GO:0038031	1.04E-01	1.89E-01	Wnt5a,22418:down
negative regulation of cAMP-dependent protein kinase activity	GO:2000480	1.04E-01	1.89E-01	Prkar2b,19088:up
definitive erythrocyte differentiation	GO:0060318	1.04E-01	1.89E-01	Tgfbr3,21814:up
substrate-dependent cerebral cortex tangential migration	GO:0021825	1.04E-01	1.89E-01	Fgfr1,14182:down
glomerulus vasculature morphogenesis	GO:0072103	1.04E-01	1.89E-01	Bmp4,12159:up
angiogenesis involved in coronary vascular morphogenesis	GO:0060978	1.04E-01	1.89E-01	Vegfa,22339:down
regulation of histone deacetylase activity	GO:1901725	1.04E-01	1.89E-01	Vegfa,22339:down
interleukin-18 production	GO:0032621	1.04E-01	1.89E-01	Tnf,21926:up
common myeloid progenitor cell proliferation	GO:0035726	1.04E-01	1.89E-01	Ceacam1,26365:up
metanephric collecting duct development	GO:0072205	1.04E-01	1.89E-01	Bmp4,12159:up
aortic valve morphogenesis	GO:0003180	1.04E-01	1.89E-01	Bmp4,12159:up
positive regulation of cell migration by vascular endothelial growth factor signaling pathway	GO:0038089	1.04E-01	1.89E-01	Vegfa,22339:down
response to glucagon	GO:0033762	1.04E-01	1.89E-01	Cdo1,12583:up
response to yeast	GO:0001878	1.04E-01	1.89E-01	Ptx3,19288:up
negative regulation of monocyte chemotactic protein-1 production	GO:0071638	1.04E-01	1.89E-01	C1qtnf3,81799:down
positive regulation of hematopoietic stem cell proliferation	GO:1902035	1.04E-01	1.89E-01	Kitl,17311:up
pyrimidine nucleoside monophosphate metabolic process	GO:0009129	1.04E-01	1.89E-01	Uck2,80914:down
aortic valve development	GO:0003176	1.04E-01	1.89E-01	Bmp4,12159:up
common-partner SMAD protein phosphorylation	GO:0007182	1.04E-01	1.89E-01	Bmp4,12159:up
tricuspid valve development	GO:0003175	1.04E-01	1.89E-01	Bmpr1a,12166:down
imidazole-containing compound catabolic process	GO:0052805	1.04E-01	1.89E-01	Hal,15109:up
positive regulation of synaptic plasticity	GO:0031915	1.04E-01	1.89E-01	Ptgs2,19225:down
aminoglycoside antibiotic metabolic process	GO:0030647	1.04E-01	1.89E-01	Akr1c18,105349:up
negative regulation of vitamin metabolic process	GO:0046137	1.04E-01	1.89E-01	Akr1c18,105349:up
negative regulation of protein activation cascade	GO:2000258	1.04E-01	1.89E-01	Serping1,12258:up
T cell extravasation	GO:0072683	1.04E-01	1.89E-01	Ccl2,20296:up

protein retention in ER lumen	GO:0006621	1.04E-01	1.89E-01	Kdelr3,105785:down
daunorubicin metabolic process	GO:0044597	1.04E-01	1.89E-01	Akr1c18,105349:up
peptidyl-proline hydroxylation to 4-hydroxy-L-proline	GO:0018401	1.04E-01	1.89E-01	P4ha1,18451:down
negative regulation of T-helper 1 type immune response	GO:0002826	1.04E-01	1.89E-01	Il1rl1,17082:down
adiponectin secretion	GO:0070162	1.04E-01	1.89E-01	C1qtnf3,81799:down
negative regulation of complement activation	GO:0045916	1.04E-01	1.89E-01	Serping1,12258:up
glomerular capillary formation	GO:0072104	1.04E-01	1.89E-01	Bmp4,12159:up
positive regulation of sequestering of triglyceride	GO:0010890	1.04E-01	1.89E-01	Lpl,16956:up
primary alcohol catabolic process	GO:0034310	1.04E-01	1.89E-01	Akr1c18,105349:up
osteoclast proliferation	GO:0002158	1.04E-01	1.89E-01	Grem1,23892:up
male gonad development	GO:0008584	1.05E-01	1.90E-01	Sfrp2,20319:up|Sfrp1,20377:up|Wnt5a,22418:down|Inhba,16323:down
development of primary male sexual characteristics	GO:0046546	1.05E-01	1.90E-01	Sfrp2,20319:up|Sfrp1,20377:up|Wnt5a,22418:down|Inhba,16323:down
negative regulation of homotypic cell-cell adhesion	GO:0034111	1.05E-01	1.90E-01	Ceacam1,26365:up|Cd24a,12484:down|Serpine2,20720:up|Bmp4,12159:up
nerve development	GO:0021675	1.06E-01	1.92E-01	Ret,19713:up|Sulf1,240725:down|Serpine2,20720:up
cholesterol homeostasis	GO:0042632	1.06E-01	1.92E-01	Cd24a,12484:down|Fabp4,11770:up|Lpl,16956:up
sterol homeostasis	GO:0055092	1.06E-01	1.92E-01	Cd24a,12484:down|Fabp4,11770:up|Lpl,16956:up
sequestering of metal ion	GO:0051238	1.08E-01	1.95E-01	Htr2b,15559:down|Glp1r,14652:down|Tgfb2,21808:down|Htr2a,15558:down
negative regulation of fibroblast proliferation	GO:0048147	1.08E-01	1.95E-01	Pparg,19016:up|Sfrp1,20377:up
granulocyte differentiation	GO:0030851	1.08E-01	1.95E-01	Ceacam1,26365:up|Adipoq,11450:up
cell proliferation in forebrain	GO:0021846	1.08E-01	1.95E-01	Fgfr1,14182:down|Fgfr2,14183:up
regulation of platelet activation	GO:0010543	1.08E-01	1.95E-01	Ceacam1,26365:up|Serpine2,20720:up
cardiac atrium morphogenesis	GO:0003209	1.08E-01	1.95E-01	Wnt5a,22418:down|Tgfb2,21808:down
activation of MAPKK activity	GO:0000186	1.08E-01	1.95E-01	Egfr,13649:down|Bmp4,12159:up
cellular response to fatty acid	GO:0071398	1.08E-01	1.95E-01	Akr1c18,105349:up|Ptgir,19222:up
negative regulation of B cell activation	GO:0050869	1.08E-01	1.95E-01	Cd24a,12484:down|Sfrp1,20377:up
negative regulation of intracellular steroid hormone receptor signaling pathway	GO:0033144	1.08E-01	1.95E-01	Sfrp1,20377:up|Igf1,16000:up
regulation of nucleotide metabolic process	GO:0006140	1.09E-01	1.96E-01	Adm,11535:up|Wnt5a,22418:down|Vegfa,22339:down|Htr2a,15558:down|Ptgir,19222:up|Igf1,16000:up
response to peptide	GO:1901652	1.09E-01	1.96E-01	Pparg,19016:up|Igf1,16000:up|Adipoq,11450:up|Serpina3g,20715:up|Gatm,67092:down|Igf2,16002:up|Enpp1,18605:down|Cdo1,12583:up
positive regulation of tumor necrosis factor production	GO:0032760	1.09E-01	1.97E-01	Ccl2,20296:up|Ccl4,20303:up|Ccl3,20302:up
positive regulation of neuron apoptotic process	GO:0043525	1.09E-01	1.97E-01	Tnf,21926:up|Tgfb2,21808:down|Ccl3,20302:up
positive regulation of synapse assembly	GO:0051965	1.09E-01	1.97E-01	Srpx2,68792:down|Flrt2,399558:down|Thbs2,21826:down
glucan metabolic process	GO:0044042	1.09E-01	1.97E-01	Enpp1,18605:down|Igf2,16002:up|Igf1,16000:up
cellular glucan metabolic process	GO:0006073	1.09E-01	1.97E-01	Enpp1,18605:down|Igf2,16002:up|Igf1,16000:up
glycogen metabolic process	GO:0005977	1.09E-01	1.97E-01	Enpp1,18605:down|Igf2,16002:up|Igf1,16000:up

regulation of smoothened signaling pathway	GO:0008589	1.09E-01	1.97E-01	Sfrp1,20377:up|Serpine2,20720:up|Fgfr2,14183:up
negative regulation of transcription, DNA-templated	GO:0045892	1.10E-01	1.98E-01	Sfrp4,20379:up|Ednrb,13618:up|Epas1,13819:up|Ankrd1,107765:down|Vdr,22337:down|Fabp4,11770:up|Vegfa,22339:down|Gas6,14456:up|Pparg,19016:up|Tnf,21926:up|Loxl2,94352:down|Loxl3,16950:down|Lmcd1,30937:up|Fgfr1,14182:down|Hmox1,15368:up|Sfrp1,20377:up|Wnt5a,22418:down|Grem1,23892:up|Adipoq,11450:up|Bmp4,12159:up|Sfrp2,20319:up|Fgfr2,14183:up|S100a1,20193:up
DNA replication	GO:0006260	1.10E-01	1.99E-01	Pparg,19016:up|Fgfr1,14182:down|Egfr,13649:down|Pdgfc,54635:down|Grem1,23892:up|Igf1,16000:up|Bmp4,12159:up|Kitl,17311:up
negative regulation of nucleic acid-templated transcription	GO:1903507	1.12E-01	2.01E-01	Sfrp4,20379:up|Ednrb,13618:up|Epas1,13819:up|Ankrd1,107765:down|Vdr,22337:down|Fabp4,11770:up|Vegfa,22339:down|Gas6,14456:up|Pparg,19016:up|Tnf,21926:up|Loxl2,94352:down|Loxl3,16950:down|Lmcd1,30937:up|Fgfr1,14182:down|Hmox1,15368:up|Sfrp1,20377:up|Wnt5a,22418:down|Grem1,23892:up|Adipoq,11450:up|Bmp4,12159:up|Sfrp2,20319:up|Fgfr2,14183:up|S100a1,20193:up
rhythmic process	GO:0048511	1.12E-01	2.02E-01	Pparg,19016:up|Egfr,13649:down|Inhba,16323:down|Igf1,16000:up|Bmp4,12159:up|Vegfa,22339:down|Mmp2,17390:down|Kitl,17311:up
activation of cysteine-type endopeptidase activity	GO:0097202	1.13E-01	2.03E-01	Tnf,21926:up|Pparg,19016:up|F3,14066:up
regulation of cellular response to insulin stimulus	GO:1900076	1.13E-01	2.03E-01	Pparg,19016:up|Enpp1,18605:down|Igf2,16002:up
ADP metabolic process	GO:0046031	1.13E-01	2.03E-01	Htr2a,15558:down|Ampd3,11717:up|Igf1,16000:up
alpha-amino acid catabolic process	GO:1901606	1.13E-01	2.03E-01	Ddah1,69219:down|Hal,15109:up|Cdo1,12583:up
positive regulation of tumor necrosis factor superfamily cytokine production	GO:1903557	1.13E-01	2.03E-01	Ccl2,20296:up|Ccl4,20303:up|Ccl3,20302:up
negative regulation of RNA metabolic process	GO:0051253	1.13E-01	2.03E-01	Sfrp4,20379:up|Ednrb,13618:up|Epas1,13819:up|Ankrd1,107765:down|Vdr,22337:down|Fabp4,11770:up|Vegfa,22339:down|Gas6,14456:up|Pparg,19016:up|Tnf,21926:up|Loxl2,94352:down|Loxl3,16950:down|Lmcd1,30937:up|Fgfr1,14182:down|Hmox1,15368:up|Sfrp1,20377:up|Wnt5a,22418:down|Ccl4,20303:up|Grem1,23892:up|Adipoq,11450:up|Bmp4,12159:up|Sfrp2,20319:up|Fgfr2,14183:up|S100a1,20193:up
gastrulation with mouth forming second	GO:0001702	1.13E-01	2.03E-01	Wnt5a,22418:down|Plpp3,67916:up
peptide cross-linking	GO:0018149	1.13E-01	2.03E-01	Mamdc2,71738:down|Col3a1,12825:down
olfactory bulb development	GO:0021772	1.13E-01	2.03E-01	Fgfr1,14182:down|Wnt5a,22418:down
negative regulation of signal transduction in absence of ligand	GO:1901099	1.13E-01	2.03E-01	Tnf,21926:up|Cx3cr1,13051:down
face morphogenesis	GO:0060325	1.13E-01	2.03E-01	Tgfb2,21808:down|Mmp2,17390:down
response to fungus	GO:0009620	1.13E-01	2.03E-01	Ptx3,19288:up|Clec4n,56620:up
regulation of glycolytic process	GO:0006110	1.13E-01	2.03E-01	Htr2a,15558:down|Igf1,16000:up
embryonic digestive tract development	GO:0048566	1.13E-01	2.03E-01	Aldh1a2,19378:up|Fgfr2,14183:up
negative regulation of extrinsic apoptotic signaling pathway in absence of ligand	GO:2001240	1.13E-01	2.03E-01	Tnf,21926:up|Cx3cr1,13051:down
glial cell differentiation	GO:0010001	1.15E-01	2.06E-01	Pparg,19016:up|Egfr,13649:down|Plpp3,67916:up|Serpine2,20720:up|Igf1,16000:up|Bmp4,12159:up
neuron migration	GO:0001764	1.15E-01	2.06E-01	Gas6,14456:up|Flrt2,399558:down|Cxcl12,20315:up|Mark1,226778:up|Col3a1,12825:down
ammonium ion metabolic process	GO:0097164	1.15E-01	2.06E-01	Akr1c18,105349:up|Fabp5,16592:up|Tgfb2,21808:down|Maob,109731:up|Epas1,13819:up
positive regulation of cell cycle	GO:0045787	1.16E-01	2.07E-01	Tnf,21926:up|Fgfr1,14182:down|Egfr,13649:down|Wnt5a,22418:down|Igf1,16000:up|Tgfb2,21808:down|Fgfr2,14183:up|Igf2,16002:up
organonitrogen compound catabolic process	GO:1901565	1.16E-01	2.08E-01	Ddah1,69219:down|Hmox1,15368:up|Blvrb,233016:up|Mmp13,17386:up|Anpep,16790:up|Hal,15109:up|Cdo1,12583:up
positive regulation of NF-kappaB transcription factor activity	GO:0051092	1.16E-01	2.08E-01	Tnf,21926:up|Wnt5a,22418:down|Tgfbr3,21814:up|Grem1,23892:up
dorsal/ventral pattern formation	GO:0009953	1.16E-01	2.08E-01	Sfrp1,20377:up|Bmpr1a,12166:down|Grem1,23892:up|Bmp4,12159:up
cell-substrate junction assembly	GO:0007044	1.17E-01	2.08E-01	Gpm6b,14758:up|Vegfa,22339:down|Grem1,23892:up
astrocyte differentiation	GO:0048708	1.17E-01	2.08E-01	Egfr,13649:down|Plpp3,67916:up|Serpine2,20720:up
mammary gland epithelium development	GO:0061180	1.17E-01	2.08E-01	Vdr,22337:down|Wnt5a,22418:down|Fgfr2,14183:up

hematopoietic progenitor cell differentiation	GO:0002244	1.18E-01	2.08E-01	Fstl3,83554:down|Sfrp1,20377:up|Vegfa,22339:down|Inhba,16323:down|Bmp4,12159:up
vesicle-mediated transport	GO:0016192	1.18E-01	2.08E-01	Htr2b,15559:down|Adm,11535:up|Mrc1,17533:up|Sfrp4,20379:up|Ccl2,20296:up|C3,12266:up|Vegfa,22339:down|Kdelr3,105785:down|Gas6,14456:up|Pparg,19016:up|Tnf,21926:up|Ptx3,19288:up|Trf,22041:up|Cd24a,12484:down|Sec23a,20334:down|Ncam1,17967:down|Hmox1,15368:up|Wnt5a,22418:down|Tfrc,22042:up|Grem1,23892:up|Adipoq,11450:up|Fgfr2,14183:up|Syt13,80976:up|Mrc2,17534:down
acylglycerol biosynthetic process	GO:0046463	1.19E-01	2.08E-01	Lpl,16956:up|C3,12266:up
neutral lipid biosynthetic process	GO:0046460	1.19E-01	2.08E-01	Lpl,16956:up|C3,12266:up
porphyrin-containing compound metabolic process	GO:0006778	1.19E-01	2.08E-01	Hmox1,15368:up|Blvrb,233016:up
epidermis morphogenesis	GO:0048730	1.19E-01	2.08E-01	Tgfb2,21808:down|Fgfr2,14183:up
cellular response to vascular endothelial growth factor stimulus	GO:0035924	1.19E-01	2.08E-01	Vegfa,22339:down|Adamts12,239337:down
cytokine biosynthetic process	GO:0042089	1.20E-01	2.08E-01	Tnf,21926:up|Prg4,96875:down|Wnt5a,22418:down|Errfi1,74155:down
positive regulation of extracellular matrix disassembly	GO:0090091	1.20E-01	2.08E-01	Tgfb2,21808:down
keratinocyte apoptotic process	GO:0097283	1.20E-01	2.08E-01	Sfrp4,20379:up
regulation of low-density lipoprotein particle receptor biosynthetic process	GO:0045714	1.20E-01	2.08E-01	Adipoq,11450:up
Wnt signaling pathway involved in dorsal/ventral axis specification	GO:0044332	1.20E-01	2.08E-01	Sfrp1,20377:up
positive regulation of cell maturation	GO:1903431	1.20E-01	2.08E-01	Ret,19713:up
positive regulation of developmental pigmentation	GO:0048087	1.20E-01	2.08E-01	Kitl,17311:up
positive regulation of keratinocyte migration	GO:0051549	1.20E-01	2.08E-01	Has2,15117:down
regulation of endothelial cell chemotaxis to fibroblast growth factor	GO:2000544	1.20E-01	2.08E-01	Fgfr1,14182:down
epithelial fluid transport	GO:0042045	1.20E-01	2.08E-01	Ednrb,13618:up
mitotic DNA replication	GO:1902969	1.20E-01	2.08E-01	Fgfr1,14182:down
cell chemotaxis to fibroblast growth factor	GO:0035766	1.20E-01	2.08E-01	Fgfr1,14182:down
positive regulation of cholesterol storage	GO:0010886	1.20E-01	2.08E-01	Lpl,16956:up
transferrin transport	GO:0033572	1.20E-01	2.08E-01	Tfrc,22042:up
epithelial-mesenchymal cell signaling	GO:0060684	1.20E-01	2.08E-01	Bmp4,12159:up
hormone catabolic process	GO:0042447	1.20E-01	2.08E-01	Akr1c18,105349:up
sulfur amino acid catabolic process	GO:0000098	1.20E-01	2.08E-01	Cdo1,12583:up
cellular phosphate ion homeostasis	GO:0030643	1.20E-01	2.08E-01	Enpp1,18605:down
regulation of keratinocyte apoptotic process	GO:1902172	1.20E-01	2.08E-01	Sfrp4,20379:up
commissural neuron axon guidance	GO:0071679	1.20E-01	2.08E-01	Vegfa,22339:down
lens induction in camera-type eye	GO:0060235	1.20E-01	2.08E-01	Bmp4,12159:up
vascular smooth muscle cell development	GO:0097084	1.20E-01	2.08E-01	Adm,11535:up
regulation of tooth mineralization	GO:0070170	1.20E-01	2.08E-01	Aspn,66695:down
pericardium morphogenesis	GO:0003344	1.20E-01	2.08E-01	Wnt5a,22418:down

positive regulation of deacetylase activity	GO:0090045	1.20E-01	2.08E-01	Vegfa,22339:down
negative regulation of cellular extravasation	GO:0002692	1.20E-01	2.08E-01	Cxcl12,20315:up
cellular trivalent inorganic anion homeostasis	GO:0072502	1.20E-01	2.08E-01	Enpp1,18605:down
trachea formation	GO:0060440	1.20E-01	2.08E-01	Bmp4,12159:up
chondroitin sulfate biosynthetic process	GO:0030206	1.20E-01	2.08E-01	Chst11,58250:down
regulation of keratinocyte migration	GO:0051547	1.20E-01	2.08E-01	Has2,15117:down
regulation of lymphangiogenesis	GO:1901490	1.20E-01	2.08E-01	Vegfa,22339:down
histidine metabolic process	GO:0006547	1.20E-01	2.08E-01	Hal,15109:up
artery smooth muscle contraction	GO:0014824	1.20E-01	2.08E-01	Htr2a,15558:down
endothelial cell chemotaxis to fibroblast growth factor	GO:0035768	1.20E-01	2.08E-01	Fgfr1,14182:down
optic cup morphogenesis involved in camera-type eye development	GO:0002072	1.20E-01	2.08E-01	Aldh1a3,56847:down
regulation of transcription from RNA polymerase II promoter involved in myocardial precursor cell differentiation	GO:0003256	1.20E-01	2.08E-01	Grem1,23892:up
outer ear morphogenesis	GO:0042473	1.20E-01	2.08E-01	Fgfr1,14182:down
cellular response to follicle-stimulating hormone stimulus	GO:0071372	1.20E-01	2.08E-01	Inhba,16323:down
regulation of cell-cell adhesion mediated by integrin	GO:0033632	1.20E-01	2.08E-01	Cd24a,12484:down
neural plate regionalization	GO:0060897	1.20E-01	2.08E-01	Bmpr1a,12166:down
regulation of microvillus assembly	GO:0032534	1.20E-01	2.08E-01	Prl2c2,18811:up
vitamin D receptor signaling pathway	GO:0070561	1.20E-01	2.08E-01	Vdr,22337:down
taurine metabolic process	GO:0019530	1.20E-01	2.08E-01	Cdo1,12583:up
ureteric bud formation	GO:0060676	1.20E-01	2.08E-01	Grem1,23892:up
regulation of cardioblast differentiation	GO:0051890	1.20E-01	2.08E-01	Tgfb2,21808:down
positive regulation of hypersensitivity	GO:0002885	1.20E-01	2.08E-01	C3,12266:up
regulation of myosin-light-chain-phosphatase activity	GO:0035507	1.20E-01	2.08E-01	Tnf,21926:up
positive regulation of cyclin-dependent protein serine/threonine kinase activity involved in G1/S transition of mitotic cell cycle	GO:0031659	1.20E-01	2.08E-01	Egfr,13649:down
mesenchymal cell differentiation involved in kidney development	GO:0072161	1.20E-01	2.08E-01	Bmp4,12159:up
negative regulation of apoptotic process involved in morphogenesis	GO:1902338	1.20E-01	2.08E-01	Tgfbr3,21814:up
negative regulation of mast cell activation involved in immune response	GO:0033007	1.20E-01	2.08E-01	Hmox1,15368:up
cellular response to cholesterol	GO:0071397	1.20E-01	2.08E-01	Inhba,16323:down

ureteric bud elongation	GO:0060677	1.20E-01	2.08E-01	Bmp4,12159:up
non-canonical Wnt signaling pathway via MAPK cascade	GO:0038030	1.20E-01	2.08E-01	Wnt5a,22418:down
renal system vasculature morphogenesis	GO:0061438	1.20E-01	2.08E-01	Bmp4,12159:up
positive regulation of epithelial cell proliferation involved in lung morphogenesis	GO:0060501	1.20E-01	2.08E-01	Fgfr2,14183:up
serine transport	GO:0032329	1.20E-01	2.08E-01	Serinc2,230779:down
regulation of cell-cell adhesion mediated by cadherin	GO:2000047	1.20E-01	2.08E-01	Wnt5a,22418:down
Type II pneumocyte differentiation	GO:0060510	1.20E-01	2.08E-01	Igf1,16000:up
dorsal aorta morphogenesis	GO:0035912	1.20E-01	2.08E-01	Bmpr1a,12166:down
negative regulation of protein localization to cell surface	GO:2000009	1.20E-01	2.08E-01	Gpm6b,14758:up
negative regulation of renal sodium excretion	GO:0035814	1.20E-01	2.08E-01	Anpep,16790:up
mesenchymal cell differentiation involved in renal system development	GO:2001012	1.20E-01	2.08E-01	Bmp4,12159:up
tonic smooth muscle contraction	GO:0014820	1.20E-01	2.08E-01	Htr2a,15558:down
Type I pneumocyte differentiation	GO:0060509	1.20E-01	2.08E-01	Igf1,16000:up
peptide cross-linking via chondroitin 4-sulfate glycosaminoglycan	GO:0019800	1.20E-01	2.08E-01	Mamdc2,71738:down
positive regulation of myoblast proliferation	GO:2000288	1.20E-01	2.08E-01	Igf1,16000:up
serine phosphorylation of STAT3 protein	GO:0033136	1.20E-01	2.08E-01	Ret,19713:up
cellular response to epinephrine stimulus	GO:0071872	1.20E-01	2.08E-01	Adipoq,11450:up
negative regulation of cell fate specification	GO:0009996	1.20E-01	2.08E-01	Sfrp2,20319:up
kidney vasculature morphogenesis	GO:0061439	1.20E-01	2.08E-01	Bmp4,12159:up
response to epinephrine	GO:0071871	1.20E-01	2.08E-01	Adipoq,11450:up
B cell chemotaxis	GO:0035754	1.20E-01	2.08E-01	Gas6,14456:up
maintenance of protein localization in endoplasmic reticulum	GO:0035437	1.20E-01	2.08E-01	Kdelr3,105785:down
mannose metabolic process	GO:0006013	1.20E-01	2.08E-01	Man2a2,140481:up
atrioventricular canal development	GO:0036302	1.20E-01	2.08E-01	Has2,15117:down
neuron-neuron synaptic transmission	GO:0007270	1.20E-01	2.09E-01	Egfr,13649:down|Htr2a,15558:down|Ptgs2,19225:down|Serpine2,20720:up|Plat,18791:down
regulation of ion transmembrane transport	GO:0034765	1.20E-01	2.09E-01	Hvcn1,74096:up|Fxyd1,56188:up|Ccl12,20293:up|Stc1,20855:up|Kcnj15,16516:down|Fxyd2,11936:up|Bmp4,12159:up|Cxcl12,20315:up|Tgfb2,21808:down|S100a1,20193:up
negative regulation of behavior	GO:0048521	1.21E-01	2.10E-01	Wnt5a,22418:down|Ccl12,20293:up|Grem1,23892:up
muscle cell development	GO:0055001	1.23E-01	2.13E-01	Adm,11535:up|Vegfa,22339:down|Wisp1,22402:down|Igf1,16000:up|Bmp4,12159:up
peptidyl-serine phosphorylation	GO:0018105	1.24E-01	2.16E-01	Tnf,21926:up|Gas6,14456:up|Plk2,20620:down|Sfrp2,20319:up|Ret,19713:up|Wnt5a,22418:down|Vegfa,22339:down
regulation of glial cell differentiation	GO:0045685	1.25E-01	2.17E-01	Pparg,19016:up|Serpine2,20720:up|Bmp4,12159:up
response to ammonium ion	GO:0060359	1.25E-01	2.17E-01	Htr2b,15559:down|Ncam1,17967:down|Htr2a,15558:down
monosaccharide biosynthetic process	GO:0046364	1.25E-01	2.17E-01	Sord,20322:up|C1qtnf3,81799:down|Adipoq,11450:up
positive regulation of anion transport	GO:1903793	1.25E-01	2.17E-01	Fxyd1,56188:up|Fam132b,227358:down|Cxcl12,20315:up
regulation of telomerase activity	GO:0051972	1.25E-01	2.17E-01	Pparg,19016:up|Grem1,23892:up

regulation of nucleotide catabolic process	GO:0030811	1.25E-01	2.17E-01	Htr2a,15558:down|Igf1,16000:up
cellular response to heat	GO:0034605	1.25E-01	2.17E-01	Tfec,21426:up|Hmox1,15368:up
positive regulation of phospholipid metabolic process	GO:1903727	1.25E-01	2.17E-01	Htr2b,15559:down|Htr2a,15558:down
regulation of myelination	GO:0031641	1.25E-01	2.17E-01	Tnf,21926:up|Trf,22041:up
positive regulation of vasodilation	GO:0045909	1.25E-01	2.17E-01	Adm,11535:up|Egfr,13649:down
proximal/distal pattern formation	GO:0009954	1.25E-01	2.17E-01	Aldh1a2,19378:up|Grem1,23892:up
regulation of synaptic plasticity	GO:0048167	1.25E-01	2.17E-01	Plk2,20620:down|Ncam1,17967:down|Ptgs2,19225:down|Cx3cr1,13051:down|Serpine2,20720:up
G-protein coupled receptor signaling pathway	GO:0007186	1.25E-01	2.17E-01	Htr2b,15559:down|Adm,11535:up|Ccl9,20308:up|Ccl6,20305:up|Ccl7,20306:up|Ccl2,20296:up|C3,12266:up|Ednrb,13618:up|Ccl3,20302:up|Cxcr6,80901:down|Htr2a,15558:down|Cx3cr1,13051:down|Akr1c18,105349:up|Glp1r,14652:down|Ccl12,20293:up|Ccl4,20303:up|Ccl8,20307:up|Cxcl9,17329:up|Ptgir,19222:up
post-embryonic development	GO:0009791	1.26E-01	2.17E-01	Chst11,58250:down|Vegfa,22339:down|Fgfr2,14183:up|Bmp4,12159:up
cellular response to stress	GO:0033554	1.26E-01	2.17E-01	Adm,11535:up|Tfec,21426:up|Sfrp4,20379:up|Epas1,13819:up|Ankrd1,107765:down|Uchl5,56207:up|Akr1c18,105349:up|Trf,22041:up|Wnt5a,22418:down|Bmpr1a,12166:down|Il1rn,16181:up|Bmp4,12159:up|Tnc,21923:down|Tgfb2,21808:down|Sdf2l1,64136:up|Creb3l1,26427:down|Hp,15439:up|Pdia5,72599:down|Cxcl12,20315:up|Plod2,26432:down|Nabp1,109019:down|Vegfa,22339:down|Mmp2,17390:down|Tnf,21926:up|Gas6,14456:up|Lmcd1,30937:up|Egfr,13649:down|Sfrp1,20377:up|Hmox1,15368:up|Ptgs2,19225:down|Ceacam1,26365:up|Sfrp2,20319:up|Errfi1,74155:down
negative regulation of mitotic cell cycle	GO:0045930	1.28E-01	2.21E-01	Plk2,20620:down|Egfr,13649:down|Nabp1,109019:down|Fgfr2,14183:up|Bmp4,12159:up
negative regulation of neuron apoptotic process	GO:0043524	1.28E-01	2.21E-01	Glp1r,14652:down|Crlf1,12931:down|Hmox1,15368:up|Ccl12,20293:up|Mt1,17748:up
cytokine metabolic process	GO:0042107	1.29E-01	2.22E-01	Tnf,21926:up|Prg4,96875:down|Wnt5a,22418:down|Errfi1,74155:down
purine ribonucleoside diphosphate metabolic process	GO:0009179	1.29E-01	2.22E-01	Htr2a,15558:down|Ampd3,11717:up|Igf1,16000:up
adherens junction assembly	GO:0034333	1.29E-01	2.22E-01	Gpm6b,14758:up|Vegfa,22339:down|Grem1,23892:up
purine nucleoside diphosphate metabolic process	GO:0009135	1.29E-01	2.22E-01	Htr2a,15558:down|Ampd3,11717:up|Igf1,16000:up
organic hydroxy compound metabolic process	GO:1901615	1.30E-01	2.24E-01	Tnf,21926:up|Akr1c18,105349:up|Fgfr1,14182:down|Fabp5,16592:up|Wnt5a,22418:down|Maob,109731:up|Epas1,13819:up|Vdr,22337:down|Sord,20322:up|Tgfb2,21808:down|Pdxk,216134:up
myeloid leukocyte activation	GO:0002274	1.31E-01	2.25E-01	Hmox1,15368:up|Wnt5a,22418:down|Cx3cr1,13051:down|Il1rl1,17082:down|Tnfsf9,21950:up
cellular response to oxidative stress	GO:0034599	1.31E-01	2.25E-01	Tnf,21926:up|Akr1c18,105349:up|Hp,15439:up|Hmox1,15368:up|Epas1,13819:up|Bmp4,12159:up
maternal process involved in female pregnancy	GO:0060135	1.32E-01	2.25E-01	Vdr,22337:down|Ptgs2,19225:down
negative regulation of cellular response to insulin stimulus	GO:1900077	1.32E-01	2.25E-01	Pparg,19016:up|Enpp1,18605:down
positive regulation of glucose metabolic process	GO:0010907	1.32E-01	2.25E-01	Igf2,16002:up|Igf1,16000:up
establishment or maintenance of bipolar cell polarity	GO:0061245	1.32E-01	2.25E-01	Ptk7,71461:down|Wnt5a,22418:down
regulation of amino acid transport	GO:0051955	1.32E-01	2.25E-01	Tnf,21926:up|Il1rn,16181:up
sleep	GO:0030431	1.32E-01	2.25E-01	Htr2a,15558:down|Slc29a1,63959:up
cardiac atrium development	GO:0003230	1.32E-01	2.25E-01	Wnt5a,22418:down|Tgfb2,21808:down
fatty acid derivative biosynthetic process	GO:1901570	1.32E-01	2.25E-01	Ggt5,23887:up|Ptgs2,19225:down
establishment or maintenance of apical/basal cell polarity	GO:0035088	1.32E-01	2.25E-01	Ptk7,71461:down|Wnt5a,22418:down
icosanoid biosynthetic process	GO:0046456	1.32E-01	2.25E-01	Ggt5,23887:up|Ptgs2,19225:down
axon regeneration	GO:0031103	1.32E-01	2.25E-01	Tnc,21923:down|Mmp2,17390:down
regulation of cardiac muscle cell apoptotic process	GO:0010665	1.32E-01	2.25E-01	Sfrp2,20319:up|Igfbp3,16009:up

brain morphogenesis	GO:0048854	1.32E-01	2.25E-01	Uchl5,56207:up|Wnt5a,22418:down
cellular response to corticosteroid stimulus	GO:0071384	1.32E-01	2.25E-01	Akr1c18,105349:up|Bmp4,12159:up
positive regulation of fatty acid metabolic process	GO:0045923	1.32E-01	2.25E-01	Pparg,19016:up|Adipoq,11450:up
pigment cell differentiation	GO:0050931	1.32E-01	2.25E-01	Ednrb,13618:up|Kitl,17311:up
regulation of fatty acid metabolic process	GO:0019217	1.33E-01	2.27E-01	Pparg,19016:up|Fam132b,227358:down|Adipoq,11450:up
carboxylic acid transport	GO:0046942	1.35E-01	2.27E-01	Tnf,21926:up|Pparg,19016:up|Fam132b,227358:down|Emb,13723:up|Il1rn,16181:up|Slc16a4,229699:down|Serinc2,230779:down
nitrogen compound transport	GO:0071705	1.35E-01	2.27E-01	Slc29a1,63959:up|Gpm6b,14758:up|Cxcl12,20315:up|Htr2a,15558:down|Pparg,19016:up|Tnf,21926:up|Glp1r,14652:down|Egfr,13649:down|Sfrp1,20377:up|C1qtnf3,81799:down|Il1rn,16181:up|Ptgs2,19225:down|Sncg,20618:up|Grem1,23892:up|Maob,109731:up|Serinc2,230779:down
negative regulation of protein secretion	GO:0050709	1.35E-01	2.27E-01	Tnf,21926:up|Gas6,14456:up|Sfrp1,20377:up|C1qtnf3,81799:down
positive regulation of endopeptidase activity	GO:0010950	1.35E-01	2.27E-01	Tnf,21926:up|Pparg,19016:up|Sfrp2,20319:up|F3,14066:up
negative regulation of epidermal growth factor-activated receptor activity	GO:0007175	1.36E-01	2.27E-01	Errfi1,74155:down
positive regulation of voltage-gated calcium channel activity	GO:1901387	1.36E-01	2.27E-01	S100a1,20193:up
regulation of hematopoietic stem cell proliferation	GO:1902033	1.36E-01	2.27E-01	Kitl,17311:up
positive regulation of acute inflammatory response to antigenic stimulus	GO:0002866	1.36E-01	2.27E-01	C3,12266:up
lacrimal gland development	GO:0032808	1.36E-01	2.27E-01	Fgfr2,14183:up
negative regulation of muscle adaptation	GO:0014745	1.36E-01	2.27E-01	Errfi1,74155:down
negative regulation by host of viral transcription	GO:0043922	1.36E-01	2.27E-01	Ccl4,20303:up
inflammatory response to wounding	GO:0090594	1.36E-01	2.27E-01	Hmox1,15368:up
regulation of dendritic cell chemotaxis	GO:2000508	1.36E-01	2.27E-01	Gas6,14456:up
regulation of interferon-gamma-mediated signaling pathway	GO:0060334	1.36E-01	2.27E-01	Pparg,19016:up
regulation of natural killer cell mediated cytotoxicity directed against tumor cell target	GO:0002858	1.36E-01	2.27E-01	Ceacam1,26365:up
glomerular basement membrane development	GO:0032836	1.36E-01	2.27E-01	Sulf1,240725:down
very-low-density lipoprotein particle remodeling	GO:0034372	1.36E-01	2.27E-01	Lpl,16956:up
regulation of motor neuron apoptotic process	GO:2000671	1.36E-01	2.27E-01	Crlf1,12931:down
cellular response to arsenic-containing substance	GO:0071243	1.36E-01	2.27E-01	Hmox1,15368:up
protein transport within lipid bilayer	GO:0032594	1.36E-01	2.27E-01	Cd24a,12484:down
regulation of CD8-positive, alpha-beta T cell activation	GO:2001185	1.36E-01	2.27E-01	Ceacam1,26365:up
ectopic germ cell programmed cell death	GO:0035234	1.36E-01	2.27E-01	Kitl,17311:up
negative regulation of pathway-restricted SMAD protein phosphorylation	GO:0060394	1.36E-01	2.27E-01	Grem1,23892:up
regulation of apoptotic DNA fragmentation	GO:1902510	1.36E-01	2.27E-01	Igfbp3,16009:up

anterior/posterior axis specification, embryo	GO:0008595	1.36E-01	2.27E-01	Wnt5a,22418:down
tripartite regional subdivision	GO:0007351	1.36E-01	2.27E-01	Wnt5a,22418:down
regulation of cardiac muscle cell membrane potential	GO:0086036	1.36E-01	2.27E-01	Fxyd1,56188:up
natural killer cell mediated immune response to tumor cell	GO:0002423	1.36E-01	2.27E-01	Ceacam1,26365:up
wound healing involved in inflammatory response	GO:0002246	1.36E-01	2.27E-01	Hmox1,15368:up
positive regulation of interleukin-8 biosynthetic process	GO:0045416	1.36E-01	2.27E-01	Tnf,21926:up
iron ion import into cell	GO:0097459	1.36E-01	2.27E-01	Trf,22041:up
dorsal aorta development	GO:0035907	1.36E-01	2.27E-01	Bmpr1a,12166:down
calcium-independent cell-cell adhesion via plasma membrane cell-adhesion molecules	GO:0016338	1.36E-01	2.27E-01	Ceacam1,26365:up
trachea cartilage development	GO:0060534	1.36E-01	2.27E-01	Rspo2,239405:up
negative regulation of mesoderm development	GO:2000381	1.36E-01	2.27E-01	Sfrp2,20319:up
polyprenol metabolic process	GO:0016093	1.36E-01	2.27E-01	Akr1c18,105349:up
regulation of cyclin-dependent protein serine/threonine kinase activity involved in G1/S transition of mitotic cell cycle	GO:0031657	1.36E-01	2.27E-01	Egfr,13649:down
regulation of transforming growth factor beta2 production	GO:0032909	1.36E-01	2.27E-01	Tgfb2,21808:down
positive regulation of synaptic transmission, cholinergic	GO:0032224	1.36E-01	2.27E-01	Lama2,16773:down
regulation of store-operated calcium channel activity	GO:1901339	1.36E-01	2.27E-01	Bmp4,12159:up
hindgut morphogenesis	GO:0007442	1.36E-01	2.27E-01	Wnt5a,22418:down
neuron cell-cell adhesion	GO:0007158	1.36E-01	2.27E-01	Ret,19713:up
paraxial mesoderm formation	GO:0048341	1.36E-01	2.27E-01	Wnt5a,22418:down
cellular response to vitamin D	GO:0071305	1.36E-01	2.27E-01	Vdr,22337:down
negative regulation of neuroblast proliferation	GO:0007406	1.36E-01	2.27E-01	Cd24a,12484:down
nucleoside salvage	GO:0043174	1.36E-01	2.27E-01	Uck2,80914:down
natural killer cell mediated cytotoxicity directed against tumor cell target	GO:0002420	1.36E-01	2.27E-01	Ceacam1,26365:up
ER-associated misfolded protein catabolic process	GO:0071712	1.36E-01	2.27E-01	Sdf2l1,64136:up
snRNA transcription from RNA polymerase II promoter	GO:0042795	1.36E-01	2.27E-01	Ice1,218333:down
post-embryonic camera-type eye development	GO:0031077	1.36E-01	2.27E-01	Vegfa,22339:down
regulation of humoral immune response mediated by circulating immunoglobulin	GO:0002923	1.36E-01	2.27E-01	Tnf,21926:up
cellular response to UV-B	GO:0071493	1.36E-01	2.27E-01	Mme,17380:up

cellular divalent inorganic anion homeostasis	GO:0072501	1.36E-01	2.27E-01	Enpp1,18605:down
negative regulation of smooth muscle cell apoptotic process	GO:0034392	1.36E-01	2.27E-01	Igf1,16000:up
catagen	GO:0042637	1.36E-01	2.27E-01	Tgfb2,21808:down
secondary heart field specification	GO:0003139	1.36E-01	2.27E-01	Bmp4,12159:up
negative regulation of myelination	GO:0031642	1.36E-01	2.27E-01	Tnf,21926:up
norepinephrine biosynthetic process	GO:0042421	1.36E-01	2.27E-01	Epas1,13819:up
positive regulation of protein localization to cell surface	GO:2000010	1.36E-01	2.27E-01	Tnf,21926:up
transforming growth factor beta2 production	GO:0032906	1.36E-01	2.27E-01	Tgfb2,21808:down
negative regulation of hormone metabolic process	GO:0032351	1.36E-01	2.27E-01	Akr1c18,105349:up
regulation of hyaluronan biosynthetic process	GO:1900125	1.36E-01	2.27E-01	Has2,15117:down
isoprenoid catabolic process	GO:0008300	1.36E-01	2.27E-01	Akr1c18,105349:up
regulation of serotonin secretion	GO:0014062	1.36E-01	2.27E-01	Maob,109731:up
regulation of plasminogen activation	GO:0010755	1.36E-01	2.27E-01	Serpine2,20720:up
regulation of pigment cell differentiation	GO:0050932	1.36E-01	2.27E-01	Kitl,17311:up
negative regulation of interleukin-1 secretion	GO:0050711	1.36E-01	2.27E-01	Gas6,14456:up
regulation of leukocyte tethering or rolling	GO:1903236	1.36E-01	2.27E-01	Cxcl12,20315:up
positive regulation of cAMP-mediated signaling	GO:0043950	1.36E-01	2.27E-01	Ptgir,19222:up
positive regulation of monocyte chemotactic protein-1 production	GO:0071639	1.36E-01	2.27E-01	Adipoq,11450:up
visceral serous pericardium development	GO:0061032	1.36E-01	2.27E-01	Tgfbr3,21814:up
regulation of natural killer cell mediated immune response to tumor cell	GO:0002855	1.36E-01	2.27E-01	Ceacam1,26365:up
regulation of follicle-stimulating hormone secretion	GO:0046880	1.36E-01	2.27E-01	Inhba,16323:down
negative regulation of keratinocyte differentiation	GO:0045617	1.36E-01	2.27E-01	Tgfb2,21808:down
IMP biosynthetic process	GO:0006188	1.36E-01	2.27E-01	Ampd3,11717:up
positive regulation of penile erection	GO:0060406	1.36E-01	2.27E-01	Ednrb,13618:up
tissue regeneration	GO:0042246	1.38E-01	2.29E-01	Ninj1,18081:up|Igf1,16000:up
regulation of sodium ion transmembrane transporter activity	GO:2000649	1.38E-01	2.29E-01	Fxyd1,56188:up|Fxyd2,11936:up
regulation of DNA-dependent DNA replication	GO:0090329	1.38E-01	2.29E-01	Fgfr1,14182:down|Bmp4,12159:up
developmental induction	GO:0031128	1.38E-01	2.29E-01	Fgfr1,14182:down|Bmp4,12159:up
negative regulation of mitotic nuclear division	GO:0045839	1.38E-01	2.29E-01	Fgfr2,14183:up|Bmp4,12159:up
hydrogen peroxide metabolic process	GO:0042743	1.38E-01	2.29E-01	Pxdn,69675:down|Hp,15439:up
positive regulation of immunoglobulin production	GO:0002639	1.38E-01	2.29E-01	Ceacam1,26365:up|Tfrc,22042:up
proteoglycan biosynthetic process	GO:0030166	1.38E-01	2.29E-01	Chst11,58250:down|Igf1,16000:up

regulation of striated muscle cell apoptotic process	GO:0010662	1.38E-01	2.29E-01	Sfrp2,20319:up|Igfbp3,16009:up
positive regulation of cation channel activity	GO:2001259	1.38E-01	2.29E-01	S100a1,20193:up|Bmp4,12159:up
T-helper 1 type immune response	GO:0042088	1.38E-01	2.29E-01	Vegfa,22339:down|Il1rl1,17082:down
activin receptor signaling pathway	GO:0032924	1.38E-01	2.29E-01	Fstl3,83554:down|Inhba,16323:down
camera-type eye morphogenesis	GO:0048593	1.38E-01	2.30E-01	Vegfa,22339:down|Aldh1a3,56847:down|Col8a1,12837:down|Bmp4,12159:up
inorganic ion transmembrane transport	GO:0098660	1.40E-01	2.32E-01	Htr2b,15559:down|Gas6,14456:up|Glp1r,14652:down|Fxyd1,56188:up|Kcnj15,16516:down|Fxyd2,11936:up|Bmp4,12159:up|Ank,11732:down|Tgfb2,21808:down|Htr2a,15558:down|S100a1,20193:up|Slc40a1,53945:up
negative regulation of neuron differentiation	GO:0045665	1.40E-01	2.33E-01	Plk2,20620:down|Cd24a,12484:down|Efemp1,216616:up|Wnt5a,22418:down|Dpysl3,22240:down|Ednrb,13618:up
nucleotide metabolic process	GO:0009117	1.41E-01	2.34E-01	Htr2b,15559:down|Adm,11535:up|Wnt5a,22418:down|Ampd3,11717:up|Enpp5,83965:up|Igf1,16000:up|Vegfa,22339:down|Gfpt2,14584:down|Htr2a,15558:down|Cbr2,12409:up|Ptgir,19222:up|Uck2,80914:down|Enpp1,18605:down
energy reserve metabolic process	GO:0006112	1.41E-01	2.35E-01	Enpp1,18605:down|Igf2,16002:up|Igf1,16000:up
protein complex localization	GO:0031503	1.42E-01	2.36E-01	Tnf,21926:up|C1qtnf3,81799:down|Ptgs2,19225:down|Grem1,23892:up
lipid localization	GO:0010876	1.42E-01	2.36E-01	Tnf,21926:up|Pparg,19016:up|Lpl,16956:up|Inhba,16323:down|C3,12266:up|Adipoq,11450:up|Fam132b,227358:down|Enpp1,18605:down
cellular component organization or biogenesis	GO:0071840	1.42E-01	2.36E-01	Igfbp4,16010:up|Adm,11535:up|Flrt2,399558:down|Plxnd1,67784:up|Sfrp4,20379:up|C3,12266:up|Ankrd1,107765:down|Ltbp2,16997:down|Serpinh1,12406:down|Igfbp3,16009:up|Ibsp,15891:up|Loxl2,94352:down|Plk2,20620:down|Loxl3,16950:down|Trf,22041:up|Cd24a,12484:down|Wnt5a,22418:down|Mark1,226778:up|Stc1,20855:up|Adipoq,11450:up|Bmp4,12159:up|Igf1,16000:up|Crabp2,12904:down|Cxcl9,17329:up|Tgfb2,21808:down|Hpse,15442:up|Fgfr2,14183:up|Thbs2,21826:down|Cthrc1,68588:down|Prl2c2,18811:up|Efemp1,216616:up|Dpysl3,22240:down|Vdr,22337:down|Cxcl12,20315:up|Mmp2,17390:down|Smoc2,64074:down|Gas6,14456:up|Egfr,13649:down|Col11a1,12814:down|Sfrp1,20377:up|C1qtnf3,81799:down|Tgfbr3,21814:up|Sncg,20618:up|Ceacam1,26365:up|Ccl8,20307:up|Sfrp2,20319:up|Pxdn,69675:down|Npnt,114249:up|Tnfrsf11b,18383:up|Syt13,80976:up|Col5a2,12832:down|Ice1,218333:down|Ccl2,20296:up|Pdzrn3,55983:down|Pdgfc,54635:down|Mgp,17313:up|Mmp13,17386:up|Sema7a,20361:down|Epas1,13819:up|Htra3,78558:down|Tnfrsf12a,27279:down|Gpm6b,14758:up|Shroom4,208431:down|Postn,50706:down|Ptx3,19288:up|Ncam1,17967:down|Ccl12,20293:up|Wisp1,22402:down|Tnc,21923:down|Ret,19713:up|Sdf2l1,64136:up|Creb3l1,26427:down|Fam101b,76566:down|P4ha1,18451:down|Lama2,16773:down|Ptk7,71461:down|Inhba,16323:down|Ccl3,20302:up|Srpx2,68792:down|Plod2,26432:down|Vegfa,22339:down|Cbr2,12409:up|Serpine2,20720:up|Lrrc15,74488:down|Igf2,16002:up|Pparg,19016:up|Tnf,21926:up|Lmcd1,30937:up|Col1a2,12843:down|Fgfr1,14182:down|Lpl,16956:up|Hmox1,15368:up|Has2,15117:down|Sulf1,240725:down|C1qtnf6,72709:down|Grem1,23892:up|Col3a1,12825:down|Col5a1,12831:down|Enpp1,18605:down
negative regulation of catabolic process	GO:0009895	1.43E-01	2.37E-01	Htr2b,15559:down|Tnf,21926:up|Egfr,13649:down|Hp,15439:up|Hmox1,15368:up|Serpine2,20720:up
auditory receptor cell differentiation	GO:0042491	1.44E-01	2.39E-01	Fgfr1,14182:down|Mcoln3,171166:up
androgen receptor signaling pathway	GO:0030521	1.44E-01	2.39E-01	Sfrp1,20377:up|Igf1,16000:up
regulation of oligodendrocyte differentiation	GO:0048713	1.44E-01	2.39E-01	Pparg,19016:up|Bmp4,12159:up
head morphogenesis	GO:0060323	1.44E-01	2.39E-01	Tgfb2,21808:down|Mmp2,17390:down
positive regulation of glial cell differentiation	GO:0045687	1.44E-01	2.39E-01	Pparg,19016:up|Serpine2,20720:up
regulation of cell cycle process	GO:0010564	1.44E-01	2.39E-01	Tnf,21926:up|Plk2,20620:down|Fgfr1,14182:down|Egfr,13649:down|Sfrp1,20377:up|Wnt5a,22418:down|Susd2,71733:up|Igf1,16000:up|Bmp4,12159:up|Fgfr2,14183:up|Igf2,16002:up
inorganic cation transmembrane transport	GO:0098662	1.44E-01	2.39E-01	Htr2b,15559:down|Gas6,14456:up|Glp1r,14652:down|Fxyd1,56188:up|Kcnj15,16516:down|Fxyd2,11936:up|Bmp4,12159:up|Tgfb2,21808:down|Htr2a,15558:down|S100a1,20193:up|Slc40a1,53945:up
cell morphogenesis involved in neuron differentiation	GO:0048667	1.44E-01	2.39E-01	Flrt2,399558:down|Lama2,16773:down|Plxnd1,67784:up|Wnt5a,22418:down|Sema7a,20361:down|Tnfrsf12a,27279:down|Crabp2,12904:down|Ret,19713:up|Cxcl12,20315:up|Vegfa,22339:down|Tgfb2,21808:down|Fgfr2,14183:up
positive regulation of peptidase activity	GO:0010952	1.45E-01	2.40E-01	Tnf,21926:up|Pparg,19016:up|Sfrp2,20319:up|F3,14066:up
anion transmembrane transport	GO:0098656	1.45E-01	2.40E-01	Ank,11732:down|Fxyd1,56188:up|Emb,13723:up|Slc16a4,229699:down
ribonucleoside diphosphate metabolic process	GO:0009185	1.46E-01	2.41E-01	Htr2a,15558:down|Ampd3,11717:up|Igf1,16000:up
negative regulation of lipid metabolic process	GO:0045833	1.46E-01	2.41E-01	Ceacam1,26365:up|Tnf,21926:up|Akr1c18,105349:up
regulation of sodium ion transport	GO:0002028	1.46E-01	2.41E-01	Fxyd1,56188:up|Serpine2,20720:up|Fxyd2,11936:up

organonitrogen compound metabolic process	GO:1901564	1.46E-01	2.42E-01	Adm,11535:up|Mmp13,17386:up|Ampd3,11717:up|Epas1,13819:up|Ddah1,69219:down|Gstt1,14871:up|Blvrb,233016:up|Cdo1,12583:up|Mamdc2,71738:down|Ggt5,23887:up|Fabp5,16592:up|Mme,17380:up|Wnt5a,22418:down|Maob,109731:up|Hal,15109:up|Igf1,16000:up|Chst11,58250:down|Gatm,67092:down|Tgfb2,21808:down|Pdxk,216134:up|Ptgir,19222:up|Htr2b,15559:down|P4ha1,18451:down|Gfpt2,14584:down|Htr2a,15558:down|Vegfa,22339:down|Cbr2,12409:up|Anpep,16790:up|Tnf,21926:up|Egfr,13649:down|Hmox1,15368:up|Has2,15117:down|Serinc2,230779:down|Gstm1,14862:up|Uck2,80914:down|Enpp1,18605:down
regulation of transmembrane transport	GO:0034762	1.47E-01	2.42E-01	Hvcn1,74096:up|Fxyd1,56188:up|Ccl12,20293:up|Stc1,20855:up|Kcnj15,16516:down|Fxyd2,11936:up|Bmp4,12159:up|Cxcl12,20315:up|Tgfb2,21808:down|S100a1,20193:up
regionalization	GO:0003002	1.47E-01	2.43E-01	Fgfr1,14182:down|Sfrp1,20377:up|Wnt5a,22418:down|Bmpr1a,12166:down|Aldh1a2,19378:up|Grem1,23892:up|Bmp4,12159:up|Sfrp2,20319:up|Fgfr2,14183:up
response to reactive oxygen species	GO:0000302	1.47E-01	2.43E-01	Tnf,21926:up|Akr1c18,105349:up|Hp,15439:up|Hmox1,15368:up|Mmp2,17390:down
steroid metabolic process	GO:0008202	1.48E-01	2.43E-01	Vdr,22337:down|Tnf,21926:up|Akr1c18,105349:up|Adm,11535:up|Fgfr1,14182:down|Igf2,16002:up|Igf1,16000:up
negative regulation of lymphocyte activation	GO:0051250	1.48E-01	2.43E-01	Ceacam1,26365:up|Cd24a,12484:down|Sfrp1,20377:up|Bmp4,12159:up
embryonic camera-type eye development	GO:0031076	1.50E-01	2.43E-01	Aldh1a2,19378:up|Aldh1a3,56847:down
amino sugar metabolic process	GO:0006040	1.50E-01	2.43E-01	Gfpt2,14584:down|Npl,74091:up
regulation of cellular carbohydrate catabolic process	GO:0043471	1.50E-01	2.43E-01	Htr2a,15558:down|Igf1,16000:up
embryo implantation	GO:0007566	1.50E-01	2.43E-01	Ptgs2,19225:down|Mmp2,17390:down
response to estradiol	GO:0032355	1.50E-01	2.43E-01	Egfr,13649:down|Mmp2,17390:down
regulation of carbohydrate catabolic process	GO:0043470	1.50E-01	2.43E-01	Htr2a,15558:down|Igf1,16000:up
positive regulation of glucose import	GO:0046326	1.50E-01	2.43E-01	Igf1,16000:up|Adipoq,11450:up
negative regulation of blood circulation	GO:1903523	1.50E-01	2.43E-01	Adm,11535:up|Mmp2,17390:down
positive regulation of organic acid transport	GO:0032892	1.50E-01	2.43E-01	Fxyd1,56188:up|Fam132b,227358:down
cell-cell signaling involved in cell fate commitment	GO:0045168	1.50E-01	2.43E-01	Fgfr1,14182:down|Bmp4,12159:up
positive regulation of cell-matrix adhesion	GO:0001954	1.50E-01	2.43E-01	Vegfa,22339:down|Ninj1,18081:up
multicellular organismal iron ion homeostasis	GO:0060586	1.51E-01	2.43E-01	Slc40a1,53945:up
development of secondary female sexual characteristics	GO:0046543	1.51E-01	2.43E-01	Wnt5a,22418:down
cellular response to ethanol	GO:0071361	1.51E-01	2.43E-01	Ccl7,20306:up
regulation of nuclear cell cycle DNA replication	GO:0033262	1.51E-01	2.43E-01	Fgfr1,14182:down
hepatocyte growth factor receptor signaling pathway	GO:0048012	1.51E-01	2.43E-01	Adamts12,239337:down
positive regulation of response to reactive oxygen species	GO:1901033	1.51E-01	2.43E-01	Tnf,21926:up
regulation of epithelial cell proliferation involved in lung morphogenesis	GO:2000794	1.51E-01	2.43E-01	Fgfr2,14183:up
complement activation, alternative pathway	GO:0006957	1.51E-01	2.43E-01	C3,12266:up
negative regulation of synaptic transmission, glutamatergic	GO:0051967	1.51E-01	2.43E-01	Htr2a,15558:down
negative regulation of activin receptor signaling pathway	GO:0032926	1.51E-01	2.43E-01	Fstl3,83554:down
positive regulation of myelination	GO:0031643	1.51E-01	2.43E-01	Trf,22041:up
progesterone metabolic process	GO:0042448	1.51E-01	2.43E-01	Akr1c18,105349:up
peptidyl-proline hydroxylation	GO:0019511	1.51E-01	2.43E-01	P4ha1,18451:down
snRNA transcription	GO:0009301	1.51E-01	2.43E-01	Ice1,218333:down

Bergmann glial cell differentiation	GO:0060020	1.51E-01	2.43E-01	Plpp3,67916:up
N-acetylneuraminate metabolic process	GO:0006054	1.51E-01	2.43E-01	Npl,74091:up
fructose metabolic process	GO:0006000	1.51E-01	2.43E-01	Sord,20322:up
tangential migration from the subventricular zone to the olfactory bulb	GO:0022028	1.51E-01	2.43E-01	Fgfr1,14182:down
Fc receptor signaling pathway	GO:0038093	1.51E-01	2.43E-01	Clec4e,56619:up
T-helper 17 cell lineage commitment	GO:0072540	1.51E-01	2.43E-01	Ly9,17085:up
sphingolipid mediated signaling pathway	GO:0090520	1.51E-01	2.43E-01	Plpp3,67916:up
negative regulation of glutamate secretion	GO:0014050	1.51E-01	2.43E-01	Il1rn,16181:up
endothelial cell morphogenesis	GO:0001886	1.51E-01	2.43E-01	Stc1,20855:up
positive regulation of transcription from RNA polymerase II promoter in response to endoplasmic reticulum stress	GO:1990440	1.51E-01	2.43E-01	Creb3l1,26427:down
hindgut development	GO:0061525	1.51E-01	2.43E-01	Wnt5a,22418:down
purine-containing compound salvage	GO:0043101	1.51E-01	2.43E-01	Ampd3,11717:up
neural plate pattern specification	GO:0060896	1.51E-01	2.43E-01	Bmpr1a,12166:down
negative regulation of sodium ion transport	GO:0010766	1.51E-01	2.43E-01	Serpine2,20720:up
response to mercury ion	GO:0046689	1.51E-01	2.43E-01	Gatm,67092:down
negative regulation of thymocyte apoptotic process	GO:0070244	1.51E-01	2.43E-01	Bmp4,12159:up
negative regulation of T cell differentiation in thymus	GO:0033085	1.51E-01	2.43E-01	Bmp4,12159:up
regulation of hypersensitivity	GO:0002883	1.51E-01	2.43E-01	C3,12266:up
regulation of blood vessel remodeling	GO:0060312	1.51E-01	2.43E-01	Ceacam1,26365:up
regulation of response to interferon-gamma	GO:0060330	1.51E-01	2.43E-01	Pparg,19016:up
cysteine metabolic process	GO:0006534	1.51E-01	2.43E-01	Cdo1,12583:up
sodium ion export from cell	GO:0036376	1.51E-01	2.43E-01	Fxyd1,56188:up
negative regulation of myeloid leukocyte mediated immunity	GO:0002887	1.51E-01	2.43E-01	Hmox1,15368:up
regulation of glial cell migration	GO:1903975	1.51E-01	2.43E-01	Efemp1,216616:up
otic vesicle morphogenesis	GO:0071600	1.51E-01	2.43E-01	Fgfr2,14183:up
renal system pattern specification	GO:0072048	1.51E-01	2.43E-01	Bmp4,12159:up
negative regulation of phosphatidylinositol 3-kinase signaling	GO:0014067	1.51E-01	2.43E-01	Serpine2,20720:up
regulation of production of miRNAs involved in gene silencing by miRNA	GO:1903798	1.51E-01	2.43E-01	Egfr,13649:down
insulin metabolic process	GO:1901142	1.51E-01	2.43E-01	Ceacam1,26365:up
regulation of removal of superoxide radicals	GO:2000121	1.51E-01	2.43E-01	Tnf,21926:up
germ cell migration	GO:0008354	1.51E-01	2.43E-01	Cxcl12,20315:up
pattern specification involved in kidney development	GO:0061004	1.51E-01	2.43E-01	Bmp4,12159:up
oligodendrocyte progenitor proliferation	GO:0070444	1.51E-01	2.43E-01	Trf,22041:up

primitive streak formation	GO:0090009	1.51E-01	2.43E-01	Wnt5a,22418:down
regulation of DNA catabolic process	GO:1903624	1.51E-01	2.43E-01	Igfbp3,16009:up
liver regeneration	GO:0097421	1.51E-01	2.43E-01	Hmox1,15368:up
complement activation, lectin pathway	GO:0001867	1.51E-01	2.43E-01	Serping1,12258:up
immature B cell differentiation	GO:0002327	1.51E-01	2.43E-01	Cd24a,12484:down
cellular response to sterol	GO:0036315	1.51E-01	2.43E-01	Inhba,16323:down
negative regulation of humoral immune response	GO:0002921	1.51E-01	2.43E-01	Serping1,12258:up
regulation of oligodendrocyte progenitor proliferation	GO:0070445	1.51E-01	2.43E-01	Trf,22041:up
triglyceride-rich lipoprotein particle remodeling	GO:0034370	1.51E-01	2.43E-01	Lpl,16956:up
eosinophil chemotaxis	GO:0048245	1.51E-01	2.43E-01	Ccl2,20296:up
ribonucleoside monophosphate biosynthetic process	GO:0009156	1.52E-01	2.44E-01	Htr2a,15558:down|Uck2,80914:down|Ampd3,11717:up|Igf1,16000:up
lipid homeostasis	GO:0055088	1.52E-01	2.44E-01	Cd24a,12484:down|Fabp4,11770:up|Lpl,16956:up|C1qtnf3,81799:down
regulation of synapse organization	GO:0050807	1.52E-01	2.44E-01	Srpx2,68792:down|Flrt2,399558:down|Wnt5a,22418:down|Thbs2,21826:down
negative regulation of protein transport	GO:0051224	1.52E-01	2.45E-01	Kdelr3,105785:down|Tnf,21926:up|Gas6,14456:up|Sfrp1,20377:up|C1qtnf3,81799:down|Adipoq,11450:up
cyclic nucleotide metabolic process	GO:0009187	1.53E-01	2.45E-01	Htr2b,15559:down|Adm,11535:up|Wnt5a,22418:down|Vegfa,22339:down|Ptgir,19222:up
ribonucleotide metabolic process	GO:0009259	1.54E-01	2.46E-01	Htr2b,15559:down|Adm,11535:up|Wnt5a,22418:down|Ampd3,11717:up|Igf1,16000:up|Htr2a,15558:down|Vegfa,22339:down|Ptgir,19222:up|Uck2,80914:down|Enpp1,18605:down
icosanoid metabolic process	GO:0006690	1.54E-01	2.47E-01	Ggt5,23887:up|Hpgd,15446:up|Ptgs2,19225:down
cellular amino acid catabolic process	GO:0009063	1.54E-01	2.47E-01	Ddah1,69219:down|Hal,15109:up|Cdo1,12583:up
fatty acid derivative metabolic process	GO:1901568	1.54E-01	2.47E-01	Ggt5,23887:up|Hpgd,15446:up|Ptgs2,19225:down
regulation of gluconeogenesis	GO:0006111	1.56E-01	2.50E-01	C1qtnf3,81799:down|Adipoq,11450:up
cardiac muscle cell apoptotic process	GO:0010659	1.56E-01	2.50E-01	Sfrp2,20319:up|Igfbp3,16009:up
tetrapyrrole metabolic process	GO:0033013	1.56E-01	2.50E-01	Hmox1,15368:up|Blvrb,233016:up
small molecule catabolic process	GO:0044282	1.57E-01	2.51E-01	Sord,20322:up|Akr1c18,105349:up|Ddah1,69219:down|Npl,74091:up|Hal,15109:up|Cdo1,12583:up|Adipoq,11450:up
phospholipase C-activating G-protein coupled receptor signaling pathway	GO:0007200	1.58E-01	2.53E-01	Htr2b,15559:down|Htr2a,15558:down|Ednrb,13618:up
multicellular organismal response to stress	GO:0033555	1.58E-01	2.53E-01	Ncam1,17967:down|Ret,19713:up|Ednrb,13618:up
substrate adhesion-dependent cell spreading	GO:0034446	1.58E-01	2.53E-01	Has2,15117:down|Sfrp1,20377:up|Postn,50706:down
in utero embryonic development	GO:0001701	1.61E-01	2.57E-01	Adm,11535:up|Fgfr1,14182:down|Egfr,13649:down|Gjb5,14622:down|Bmpr1a,12166:down|Tgfbr3,21814:up|Igf1,16000:up|Epas1,13819:up|Vegfa,22339:down|Fgfr2,14183:up
specification of symmetry	GO:0009799	1.62E-01	2.59E-01	Wnt5a,22418:down|Aldh1a2,19378:up|Grem1,23892:up|Bmp4,12159:up
nucleoside monophosphate biosynthetic process	GO:0009124	1.62E-01	2.59E-01	Htr2a,15558:down|Uck2,80914:down|Ampd3,11717:up|Igf1,16000:up
anterior/posterior pattern specification	GO:0009952	1.63E-01	2.59E-01	Sfrp2,20319:up|Sfrp1,20377:up|Wnt5a,22418:down|Bmpr1a,12166:down|Aldh1a2,19378:up|Bmp4,12159:up
central nervous system neuron development	GO:0021954	1.63E-01	2.59E-01	Fgfr1,14182:down|Wnt5a,22418:down|Fgfr2,14183:up
nucleotide catabolic process	GO:0009166	1.63E-01	2.59E-01	Htr2a,15558:down|Enpp5,83965:up|Igf1,16000:up
transition metal ion transport	GO:0000041	1.63E-01	2.59E-01	Trf,22041:up|Tfrc,22042:up|Slc40a1,53945:up
negative regulation of G-protein coupled receptor protein signaling pathway	GO:0045744	1.63E-01	2.59E-01	Htr2b,15559:down|Adm,11535:up

regulation of substrate adhesion-dependent cell spreading	GO:1900024	1.63E-01	2.59E-01	Has2,15117:down|Postn,50706:down
striated muscle cell apoptotic process	GO:0010658	1.63E-01	2.59E-01	Sfrp2,20319:up|Igfbp3,16009:up
regulation of cellular amine metabolic process	GO:0033238	1.63E-01	2.59E-01	Maob,109731:up|Epas1,13819:up
regulation of cardiac muscle cell contraction	GO:0086004	1.63E-01	2.59E-01	Fxyd1,56188:up|Stc1,20855:up
regulation of response to reactive oxygen species	GO:1901031	1.63E-01	2.59E-01	Tnf,21926:up|Hp,15439:up
sodium ion homeostasis	GO:0055078	1.63E-01	2.59E-01	Ednrb,13618:up|Anpep,16790:up
regulation of mitotic cell cycle	GO:0007346	1.64E-01	2.61E-01	Tnf,21926:up|Plk2,20620:down|Fgfr1,14182:down|Egfr,13649:down|Igf1,16000:up|Bmp4,12159:up|Nabp1,109019:down|Fgfr2,14183:up|Igf2,16002:up
positive regulation of mitotic cell cycle	GO:0045931	1.65E-01	2.61E-01	Tnf,21926:up|Fgfr1,14182:down|Igf2,16002:up|Igf1,16000:up
regulation of transcription from RNA polymerase II promoter	GO:0006357	1.66E-01	2.61E-01	Fstl3,83554:down|Ednrb,13618:up|Epas1,13819:up|Ankrd1,107765:down|Htra3,78558:down|Sdpr,20324:down|Wnt5a,22418:down|Bmpr1a,12166:down|Bmp4,12159:up|Igf1,16000:up|Creb3l1,26427:down|Fgfr2,14183:up|Prl2c2,18811:up|Inhba,16323:down|Ccl3,20302:up|Vdr,22337:down|Vegfa,22339:down|Igf2,16002:up|Slc40a1,53945:up|Tnf,21926:up|Pparg,19016:up|Lmcd1,30937:up|Glp1r,14652:down|Egfr,13649:down|Fgfr1,14182:down|Sfrp1,20377:up|Hmox1,15368:up|Sulf1,240725:down|Tgfbr3,21814:up|Grem1,23892:up|Sfrp2,20319:up|Npnt,114249:up|S100a1,20193:up
nucleobase-containing small molecule metabolic process	GO:0055086	1.66E-01	2.61E-01	Htr2b,15559:down|Adm,11535:up|Wnt5a,22418:down|Ampd3,11717:up|Enpp5,83965:up|Igf1,16000:up|Vegfa,22339:down|Gfpt2,14584:down|Htr2a,15558:down|Cbr2,12409:up|Ptgir,19222:up|Uck2,80914:down|Enpp1,18605:down|Enpp3,209558:down
catabolic process	GO:0009056	1.66E-01	2.61E-01	Sfrp4,20379:up|Mmp13,17386:up|Enpp5,83965:up|Uchl5,56207:up|Sord,20322:up|Ddah1,69219:down|Blvrb,233016:up|Npl,74091:up|Igfbp3,16009:up|Enpp3,209558:down|Cdo1,12583:up|Plk2,20620:down|Akr1c18,105349:up|Wnt5a,22418:down|Maob,109731:up|Adipoq,11450:up|Hal,15109:up|Igf1,16000:up|Sdf2l1,64136:up|Hpse,15442:up|Mrc2,17534:down|Htr2b,15559:down|Hp,15439:up|Dram1,71712:up|Plod2,26432:down|Htr2a,15558:down|Serpine2,20720:up|Mmp2,17390:down|Adamts12,239337:down|Anpep,16790:up|Tnf,21926:up|Lmcd1,30937:up|Egfr,13649:down|Lpl,16956:up|Hmox1,15368:up|Ceacam1,26365:up|Pxdn,69675:down|Enpp1,18605:down
determination of dorsal identity	GO:0048263	1.67E-01	2.61E-01	Grem1,23892:up
regulation of glomerular mesangial cell proliferation	GO:0072124	1.67E-01	2.61E-01	Bmp4,12159:up
positive regulation of heterotypic cell-cell adhesion	GO:0034116	1.67E-01	2.61E-01	Tnf,21926:up
positive regulation of interleukin-5 production	GO:0032754	1.67E-01	2.61E-01	Il1rl1,17082:down
leukotriene biosynthetic process	GO:0019370	1.67E-01	2.61E-01	Ggt5,23887:up
ketone catabolic process	GO:0042182	1.67E-01	2.61E-01	Akr1c18,105349:up
vagina development	GO:0060068	1.67E-01	2.61E-01	Wnt5a,22418:down
positive regulation of collateral sprouting	GO:0048672	1.67E-01	2.61E-01	Crabp2,12904:down
positive regulation of interleukin-8 secretion	GO:2000484	1.67E-01	2.61E-01	Wnt5a,22418:down
arginine catabolic process	GO:0006527	1.67E-01	2.61E-01	Ddah1,69219:down
positive regulation of T cell chemotaxis	GO:0010820	1.67E-01	2.61E-01	Wnt5a,22418:down
hemoglobin biosynthetic process	GO:0042541	1.67E-01	2.61E-01	Inhba,16323:down
positive regulation of immunoglobulin secretion	GO:0051024	1.67E-01	2.61E-01	Ceacam1,26365:up
cerebellar granular layer morphogenesis	GO:0021683	1.67E-01	2.61E-01	Serpine2,20720:up
negative regulation of substrate adhesion-dependent cell spreading	GO:1900025	1.67E-01	2.61E-01	Postn,50706:down
regulation of pancreatic juice secretion	GO:0090186	1.67E-01	2.61E-01	Mmp13,17386:up
chondrocyte differentiation involved in endochondral bone morphogenesis	GO:0003413	1.67E-01	2.61E-01	Serpinh1,12406:down
mammary gland involution	GO:0060056	1.67E-01	2.61E-01	Vdr,22337:down

regulation of integrin activation	GO:0033623	1.67E-01	2.61E-01	Cd24a,12484:down
hypersensitivity	GO:0002524	1.67E-01	2.61E-01	C3,12266:up
cerebral cortex tangential migration	GO:0021800	1.67E-01	2.61E-01	Fgfr1,14182:down
regulation of oocyte development	GO:0060281	1.67E-01	2.61E-01	Igf1,16000:up
positive regulation of keratinocyte proliferation	GO:0010838	1.67E-01	2.61E-01	Has2,15117:down
negative regulation of thymocyte aggregation	GO:2000399	1.67E-01	2.61E-01	Bmp4,12159:up
regulation of production of small RNA involved in gene silencing by RNA	GO:0070920	1.67E-01	2.61E-01	Egfr,13649:down
follicle-stimulating hormone secretion	GO:0046884	1.67E-01	2.61E-01	Inhba,16323:down
limb bud formation	GO:0060174	1.67E-01	2.61E-01	Fgfr2,14183:up
regulation of immature T cell proliferation in thymus	GO:0033084	1.67E-01	2.61E-01	Bmp4,12159:up
Wnt signaling pathway involved in heart development	GO:0003306	1.67E-01	2.61E-01	Wnt5a,22418:down
positive regulation of type I interferon-mediated signaling pathway	GO:0060340	1.67E-01	2.61E-01	Wnt5a,22418:down
4-hydroxyproline metabolic process	GO:0019471	1.67E-01	2.61E-01	P4ha1,18451:down
motor neuron apoptotic process	GO:0097049	1.67E-01	2.61E-01	Crlf1,12931:down
hepatocyte differentiation	GO:0070365	1.67E-01	2.61E-01	Bmp4,12159:up
imidazole-containing compound metabolic process	GO:0052803	1.67E-01	2.61E-01	Hal,15109:up
primitive hemopoiesis	GO:0060215	1.67E-01	2.61E-01	Vegfa,22339:down
luteinization	GO:0001553	1.67E-01	2.61E-01	Inhba,16323:down
positive regulation of cerebellar granule cell precursor proliferation	GO:0021940	1.67E-01	2.61E-01	Igf1,16000:up
positive regulation of macrophage derived foam cell differentiation	GO:0010744	1.67E-01	2.61E-01	Lpl,16956:up
negative regulation of leukocyte degranulation	GO:0043301	1.67E-01	2.61E-01	Hmox1,15368:up
embryonic viscerocranium morphogenesis	GO:0048703	1.67E-01	2.61E-01	Chst11,58250:down
urinary bladder smooth muscle contraction	GO:0014832	1.67E-01	2.61E-01	Htr2a,15558:down
regulation of secondary metabolic process	GO:0043455	1.67E-01	2.61E-01	Wnt5a,22418:down
positive regulation of transcription from RNA polymerase III promoter	GO:0045945	1.67E-01	2.61E-01	Ice1,218333:down
glomerulus morphogenesis	GO:0072102	1.67E-01	2.61E-01	Bmp4,12159:up
mast cell chemotaxis	GO:0002551	1.67E-01	2.61E-01	Figf,14205:up
sodium ion export	GO:0071436	1.67E-01	2.61E-01	Fxyd1,56188:up
positive regulation of DNA damage response, signal transduction by p53 class mediator	GO:0043517	1.67E-01	2.61E-01	Ankrd1,107765:down
negative regulation of cell maturation	GO:1903430	1.67E-01	2.61E-01	Ednrb,13618:up

negative regulation of mast cell activation	GO:0033004	1.67E-01	2.61E-01	Hmox1,15368:up
regulation of penile erection	GO:0060405	1.67E-01	2.61E-01	Ednrb,13618:up
cardiac muscle cell myoblast differentiation	GO:0060379	1.67E-01	2.61E-01	Grem1,23892:up
branchiomotor neuron axon guidance	GO:0021785	1.67E-01	2.61E-01	Plxnd1,67784:up
determination of dorsal/ventral asymmetry	GO:0048262	1.67E-01	2.61E-01	Grem1,23892:up
response to progesterone	GO:0032570	1.67E-01	2.61E-01	Tgfb2,21808:down
fructose 6-phosphate metabolic process	GO:0006002	1.67E-01	2.61E-01	Gfpt2,14584:down
righting reflex	GO:0060013	1.67E-01	2.61E-01	Aldh1a3,56847:down
phosphatidylserine metabolic process	GO:0006658	1.67E-01	2.61E-01	Serinc2,230779:down
negative regulation of neuron migration	GO:2001223	1.67E-01	2.61E-01	Col3a1,12825:down
transcription from RNA polymerase II promoter	GO:0006366	1.67E-01	2.61E-01	Fstl3,83554:down|Ice1,218333:down|Ednrb,13618:up|Epas1,13819:up|Ankrd1,107765:down|Htra3,78558:down|Sdpr,20324:down|Wnt5a,22418:down|Bmpr1a,12166:down|Bmp4,12159:up|Igf1,16000:up|Creb3l1,26427:down|Fgfr2,14183:up|Prl2c2,18811:up|Inhba,16323:down|Ccl3,20302:up|Vdr,22337:down|Vegfa,22339:down|Igf2,16002:up|Slc40a1,53945:up|Tnf,21926:up|Pparg,19016:up|Lmcd1,30937:up|Glp1r,14652:down|Egfr,13649:down|Fgfr1,14182:down|Sfrp1,20377:up|Hmox1,15368:up|Sulf1,240725:down|Tgfbr3,21814:up|Grem1,23892:up|Sfrp2,20319:up|Npnt,114249:up|S100a1,20193:up
homophilic cell adhesion via plasma membrane adhesion molecules	GO:0007156	1.67E-01	2.61E-01	Ceacam1,26365:up|Pcdh19,279653:down|Ret,19713:up
regulation of calcium ion transport into cytosol	GO:0010522	1.67E-01	2.61E-01	Tgfb2,21808:down|Cd4,12504:up|Bmp4,12159:up
DNA biosynthetic process	GO:0071897	1.69E-01	2.64E-01	Ankrd1,107765:down|Pparg,19016:up|Grem1,23892:up|Adipoq,11450:up
positive regulation of calcium ion transport into cytosol	GO:0010524	1.69E-01	2.64E-01	Cd4,12504:up|Bmp4,12159:up
positive regulation of wound healing	GO:0090303	1.69E-01	2.64E-01	Hpse,15442:up|Plpp3,67916:up
alcohol catabolic process	GO:0046164	1.69E-01	2.64E-01	Sord,20322:up|Akr1c18,105349:up
cell differentiation involved in kidney development	GO:0061005	1.69E-01	2.64E-01	Bmp4,12159:up|Adipoq,11450:up
endocrine pancreas development	GO:0031018	1.69E-01	2.64E-01	Wnt5a,22418:down|Bmp4,12159:up
biological phase	GO:0044848	1.69E-01	2.64E-01	Tgfb2,21808:down|Ptgs2,19225:down
neuron maturation	GO:0042551	1.69E-01	2.64E-01	Ret,19713:up|Ednrb,13618:up
negative regulation of lymphocyte differentiation	GO:0045620	1.69E-01	2.64E-01	Sfrp1,20377:up|Bmp4,12159:up
cardiac muscle cell contraction	GO:0086003	1.69E-01	2.64E-01	Fxyd1,56188:up|Stc1,20855:up
positive regulation of cellular catabolic process	GO:0031331	1.70E-01	2.65E-01	Tnf,21926:up|Plk2,20620:down|Lmcd1,30937:up|Hmox1,15368:up|Sfrp4,20379:up|Igf1,16000:up|Plod2,26432:down|Htr2a,15558:down|Igfbp3,16009:up
negative regulation of cellular macromolecule biosynthetic process	GO:2000113	1.71E-01	2.66E-01	Sfrp4,20379:up|Ednrb,13618:up|Epas1,13819:up|Ankrd1,107765:down|Vdr,22337:down|Fabp4,11770:up|Vegfa,22339:down|Gas6,14456:up|Pparg,19016:up|Tnf,21926:up|Loxl2,94352:down|Loxl3,16950:down|Lmcd1,30937:up|Fgfr1,14182:down|Egfr,13649:down|Hmox1,15368:up|Sfrp1,20377:up|Wnt5a,22418:down|Grem1,23892:up|Adipoq,11450:up|Bmp4,12159:up|Sfrp2,20319:up|Enpp1,18605:down|Fgfr2,14183:up|S100a1,20193:up
negative regulation of hormone secretion	GO:0046888	1.72E-01	2.67E-01	Pparg,19016:up|Sfrp1,20377:up|Adipoq,11450:up
positive regulation of catabolic process	GO:0009896	1.72E-01	2.68E-01	Tnf,21926:up|Plk2,20620:down|Lmcd1,30937:up|Hmox1,15368:up|Wnt5a,22418:down|Sfrp4,20379:up|Igf1,16000:up|Plod2,26432:down|Htr2a,15558:down|Igfbp3,16009:up
monocarboxylic acid transport	GO:0015718	1.73E-01	2.69E-01	Pparg,19016:up|Fam132b,227358:down|Emb,13723:up|Slc16a4,229699:down
ribose phosphate metabolic process	GO:0019693	1.74E-01	2.71E-01	Htr2b,15559:down|Adm,11535:up|Wnt5a,22418:down|Ampd3,11717:up|Igf1,16000:up|Htr2a,15558:down|Vegfa,22339:down|Ptgir,19222:up|Uck2,80914:down|Enpp1,18605:down
regulation of catabolic process	GO:0009894	1.75E-01	2.72E-01	Htr2b,15559:down|Dram1,71712:up|Hp,15439:up|Sfrp4,20379:up|Uchl5,56207:up|Plod2,26432:down|Htr2a,15558:down|Igfbp3,16009:up|Serpine2,20720:up|Tnf,21926:up|Lmcd1,30937:up|Plk2,20620:down|Egfr,13649:down|Hmox1,15368:up|Wnt5a,22418:down|Igf1,16000:up
regulation of glycoprotein metabolic process	GO:1903018	1.76E-01	2.73E-01	Ptx3,19288:up|Igf1,16000:up

<table>
<tr><td>inner ear receptor cell development</td><td>GO:0060119</td><td>1.76E-01</td><td>2.73E-01</td><td>Cthrc1,68588:down|Fgfr1,14182:down</td></tr>
<tr><td>peptidyl-tyrosine autophosphorylation</td><td>GO:0038083</td><td>1.76E-01</td><td>2.73E-01</td><td>Vegfa,22339:down|Grem1,23892:up</td></tr>
<tr><td>hair cell differentiation</td><td>GO:0035315</td><td>1.76E-01</td><td>2.73E-01</td><td>Fgfr1,14182:down|Mcoln3,171166:up</td></tr>
<tr><td>autonomic nervous system development</td><td>GO:0048483</td><td>1.76E-01</td><td>2.73E-01</td><td>Ret,19713:up|Ednrb,13618:up</td></tr>
<tr><td>chaperone-mediated protein folding</td><td>GO:0061077</td><td>1.76E-01</td><td>2.73E-01</td><td>Fkbp10,14230:down|Fkbp14,231997:down</td></tr>
<tr><td>positive regulation of muscle contraction</td><td>GO:0045933</td><td>1.76E-01</td><td>2.73E-01</td><td>Ptgs2,19225:down|Npnt,114249:up</td></tr>
<tr><td>negative regulation of lipid biosynthetic process</td><td>GO:0051055</td><td>1.76E-01</td><td>2.73E-01</td><td>Ceacam1,26365:up|Akr1c18,105349:up</td></tr>
<tr><td>pyridine-containing compound metabolic process</td><td>GO:0072524</td><td>1.76E-01</td><td>2.73E-01</td><td>Htr2a,15558:down|Pdxk,216134:up|Cbr2,12409:up|Igf1,16000:up</td></tr>
<tr><td>regulation of cyclic nucleotide metabolic process</td><td>GO:0030799</td><td>1.76E-01</td><td>2.73E-01</td><td>Adm,11535:up|Wnt5a,22418:down|Vegfa,22339:down|Ptgir,19222:up</td></tr>
<tr><td>positive regulation of cyclic nucleotide metabolic process</td><td>GO:0030801</td><td>1.76E-01</td><td>2.73E-01</td><td>Adm,11535:up|Wnt5a,22418:down|Ptgir,19222:up</td></tr>
<tr><td>limbic system development</td><td>GO:0021761</td><td>1.76E-01</td><td>2.73E-01</td><td>Fgfr1,14182:down|Aldh1a3,56847:down|Fgfr2,14183:up</td></tr>
<tr><td>cell fate specification</td><td>GO:0001708</td><td>1.76E-01</td><td>2.73E-01</td><td>Sfrp2,20319:up|Fgfr1,14182:down|Bmpr1a,12166:down</td></tr>
<tr><td>regulation of nitrogen compound metabolic process</td><td>GO:0051171</td><td>1.79E-01</td><td>2.77E-01</td><td>Fstl3,83554:down|Adm,11535:up|Tfec,21426:up|Sfrp4,20379:up|Ankrd1,107765:down|Lyl1,17095:up|Igfbp3,16009:up|Loxl2,94352:down|Loxl3,16950:down|Trf,22041:up|Wnt5a,22418:down|Maob,109731:up|Adipoq,11450:up|Bmp4,12159:up|Igf1,16000:up|Ptgir,19222:up|Fgfr2,14183:up|Prl2c2,18811:up|Efemp1,216616:up|Vdr,22337:down|Fabp4,11770:up|Htr2a,15558:down|Oas3,246727:up|Slc40a1,53945:up|Gas6,14456:up|Egfr,13649:down|Sfrp1,20377:up|Tgfbr3,21814:up|Tfrc,22042:up|Sfrp2,20319:up|Npnt,114249:up|S100a1,20193:up|Ice1,218333:down|Ednrb,13618:up|Pdgfc,54635:down|Epas1,13819:up|Uchl5,56207:up|Htra3,78558:down|Ddah1,69219:down|Sdpr,20324:down|Ptx3,19288:up|Bmpr1a,12166:down|Ccl4,20303:up|Ret,19713:up|Plpp3,67916:up|Creb3l1,26427:down|Kitl,17311:up|Rasl11a,68895:up|Inhba,16323:down|Bnc2,242509:down|Ccl3,20302:up|Vegfa,22339:down|Igf2,16002:up|Tnf,21926:up|Pparg,19016:up|Lmcd1,30937:up|Glp1r,14652:down|Fgfr1,14182:down|Hmox1,15368:up|Has2,15117:down|Sulf1,240725:down|Ptgs2,19225:down|Grem1,23892:up|Phf11d,219132:up|Tbx15,21384:down</td></tr>
<tr><td>positive regulation of multi-organism process</td><td>GO:0043902</td><td>1.80E-01</td><td>2.77E-01</td><td>Ceacam1,26365:up|Inhba,16323:down|Ednrb,13618:up|Plat,18791:down</td></tr>
<tr><td>adherens junction organization</td><td>GO:0034332</td><td>1.81E-01</td><td>2.77E-01</td><td>Gpm6b,14758:up|Vegfa,22339:down|Grem1,23892:up</td></tr>
<tr><td>negative regulation of intracellular protein transport</td><td>GO:0090317</td><td>1.81E-01</td><td>2.77E-01</td><td>Gas6,14456:up|C1qtnf3,81799:down|Adipoq,11450:up</td></tr>
<tr><td>negative regulation of establishment of protein localization to plasma membrane</td><td>GO:0090005</td><td>1.82E-01</td><td>2.77E-01</td><td>Lrrc15,74488:down</td></tr>
<tr><td>notochord morphogenesis</td><td>GO:0048570</td><td>1.82E-01</td><td>2.77E-01</td><td>Wnt5a,22418:down</td></tr>
<tr><td>nephric duct development</td><td>GO:0072176</td><td>1.82E-01</td><td>2.77E-01</td><td>Bmp4,12159:up</td></tr>
<tr><td>regulation of endothelial cell development</td><td>GO:1901550</td><td>1.82E-01</td><td>2.77E-01</td><td>Tnf,21926:up</td></tr>
<tr><td>cell-cell adhesion mediated by cadherin</td><td>GO:0044331</td><td>1.82E-01</td><td>2.77E-01</td><td>Wnt5a,22418:down</td></tr>
<tr><td>interferon-gamma-mediated signaling pathway</td><td>GO:0060333</td><td>1.82E-01</td><td>2.77E-01</td><td>Pparg,19016:up</td></tr>
<tr><td>positive regulation of dopamine secretion</td><td>GO:0033603</td><td>1.82E-01</td><td>2.77E-01</td><td>Cxcl12,20315:up</td></tr>
<tr><td>regulation of brown fat cell differentiation</td><td>GO:0090335</td><td>1.82E-01</td><td>2.77E-01</td><td>Ptgs2,19225:down</td></tr>
<tr><td>positive regulation of CREB transcription factor activity</td><td>GO:0032793</td><td>1.82E-01</td><td>2.77E-01</td><td>Vegfa,22339:down</td></tr>
<tr><td>regulation of T cell chemotaxis</td><td>GO:0010819</td><td>1.82E-01</td><td>2.77E-01</td><td>Wnt5a,22418:down</td></tr>
<tr><td>regulation of cellular response to vascular endothelial growth factor stimulus</td><td>GO:1902547</td><td>1.82E-01</td><td>2.77E-01</td><td>Adamts12,239337:down</td></tr>
<tr><td>protein nitrosylation</td><td>GO:0017014</td><td>1.82E-01</td><td>2.77E-01</td><td>Ddah1,69219:down</td></tr>
<tr><td>serine family amino acid catabolic process</td><td>GO:0009071</td><td>1.82E-01</td><td>2.77E-01</td><td>Cdo1,12583:up</td></tr>
</table>

heart field specification	GO:0003128	1.82E-01	2.77E-01	Bmp4,12159:up
enamel mineralization	GO:0070166	1.82E-01	2.77E-01	Fam20a,208659:down
glomerular mesangial cell proliferation	GO:0072110	1.82E-01	2.77E-01	Bmp4,12159:up
response to low-density lipoprotein particle	GO:0055098	1.82E-01	2.77E-01	Pparg,19016:up
positive regulation by host of viral process	GO:0044794	1.82E-01	2.77E-01	Ceacam1,26365:up
embryonic camera-type eye formation	GO:0060900	1.82E-01	2.77E-01	Aldh1a3,56847:down
macrophage activation involved in immune response	GO:0002281	1.82E-01	2.77E-01	Cx3cr1,13051:down
regulation of establishment of endothelial barrier	GO:1903140	1.82E-01	2.77E-01	Tnf,21926:up
nephric duct morphogenesis	GO:0072178	1.82E-01	2.77E-01	Bmp4,12159:up
chondrocyte proliferation	GO:0035988	1.82E-01	2.77E-01	Stc1,20855:up
urinary tract smooth muscle contraction	GO:0014848	1.82E-01	2.77E-01	Htr2a,15558:down
vocalization behavior	GO:0071625	1.82E-01	2.77E-01	Srpx2,68792:down
T-helper cell lineage commitment	GO:0002295	1.82E-01	2.77E-01	Ly9,17085:up
retina vasculature morphogenesis in camera-type eye	GO:0061299	1.82E-01	2.77E-01	Vegfa,22339:down
cellular response to gonadotropin stimulus	GO:0071371	1.82E-01	2.77E-01	Inhba,16323:down
IMP metabolic process	GO:0046040	1.82E-01	2.77E-01	Ampd3,11717:up
mesonephric tubule formation	GO:0072172	1.82E-01	2.77E-01	Grem1,23892:up
dichotomous subdivision of an epithelial terminal unit	GO:0060600	1.82E-01	2.77E-01	Plxnd1,67784:up
positive regulation of natural killer cell differentiation	GO:0032825	1.82E-01	2.77E-01	Gas6,14456:up
positive regulation of phagocytosis, engulfment	GO:0060100	1.82E-01	2.77E-01	Pparg,19016:up
negative regulation of telomerase activity	GO:0051974	1.82E-01	2.77E-01	Pparg,19016:up
mitral valve morphogenesis	GO:0003183	1.82E-01	2.77E-01	Bmpr1a,12166:down
collecting duct development	GO:0072044	1.82E-01	2.77E-01	Bmp4,12159:up
pyramidal neuron development	GO:0021860	1.82E-01	2.77E-01	Fgfr2,14183:up
positive regulation of urine volume	GO:0035810	1.82E-01	2.77E-01	Ednrb,13618:up
keratinocyte migration	GO:0051546	1.82E-01	2.77E-01	Has2,15117:down
immature T cell proliferation in thymus	GO:0033080	1.82E-01	2.77E-01	Bmp4,12159:up
regulation of hydrogen peroxide metabolic process	GO:0010310	1.82E-01	2.77E-01	Hp,15439:up
positive regulation of keratinocyte differentiation	GO:0045618	1.82E-01	2.77E-01	Vdr,22337:down
positive regulation of superoxide anion generation	GO:0032930	1.82E-01	2.77E-01	Egfr,13649:down
response to vitamin D	GO:0033280	1.82E-01	2.77E-01	Vdr,22337:down
actin crosslink formation	GO:0051764	1.82E-01	2.77E-01	Dpysl3,22240:down
diet induced thermogenesis	GO:0002024	1.82E-01	2.77E-01	Pparg,19016:up
positive regulation of interleukin-17 production	GO:0032740	1.82E-01	2.77E-01	Ly9,17085:up

regulation of myoblast proliferation	GO:2000291	1.82E-01	2.77E-01	Igf1,16000:up
androgen biosynthetic process	GO:0006702	1.82E-01	2.77E-01	Akr1c18,105349:up
positive regulation of renal sodium excretion	GO:0035815	1.82E-01	2.77E-01	Ednrb,13618:up
negative regulation of dendritic spine development	GO:0061000	1.82E-01	2.77E-01	Plk2,20620:down
modulation by host of viral genome replication	GO:0044827	1.82E-01	2.77E-01	Ceacam1,26365:up
regulation of neurotransmitter uptake	GO:0051580	1.82E-01	2.77E-01	Gpm6b,14758:up
neurotransmitter catabolic process	GO:0042135	1.82E-01	2.77E-01	Maob,109731:up
negative regulation of cell fate commitment	GO:0010454	1.82E-01	2.77E-01	Sfrp2,20319:up
glutathione biosynthetic process	GO:0006750	1.82E-01	2.77E-01	Ggt5,23887:up
response to axon injury	GO:0048678	1.82E-01	2.77E-01	Tnc,21923:down|Mmp2,17390:down
regulation of myeloid leukocyte mediated immunity	GO:0002886	1.82E-01	2.77E-01	Hmox1,15368:up|C3,12266:up
regulation of coenzyme metabolic process	GO:0051196	1.82E-01	2.77E-01	Htr2a,15558:down|Igf1,16000:up
regulation of sodium ion transmembrane transport	GO:1902305	1.82E-01	2.77E-01	Fxyd1,56188:up|Fxyd2,11936:up
positive regulation of blood pressure	GO:0045777	1.82E-01	2.77E-01	Glp1r,14652:down|Adipoq,11450:up
ribonucleotide biosynthetic process	GO:0009260	1.82E-01	2.77E-01	Htr2b,15559:down|Adm,11535:up|Htr2a,15558:down|Ptgir,19222:up|Uck2,80914:down|Ampd3,11717:up|Igf1,16000:up
response to virus	GO:0009615	1.82E-01	2.77E-01	Tnf,21926:up|Lmcd1,30937:up|Cxcl9,17329:up|Oasl1,231655:up|Ifit1,15957:up|Oas3,246727:up|Ifit3,15959:up
regulation of cell shape	GO:0008360	1.83E-01	2.78E-01	Vegfa,22339:down|Ccl2,20296:up|Ccl12,20293:up|Ccl3,20302:up
negative regulation of neuron projection development	GO:0010977	1.83E-01	2.78E-01	Plk2,20620:down|Efemp1,216616:up|Wnt5a,22418:down|Dpysl3,22240:down
unsaturated fatty acid metabolic process	GO:0033559	1.85E-01	2.81E-01	Ggt5,23887:up|Hpgd,15446:up|Ptgs2,19225:down
positive regulation of neuron death	GO:1901216	1.85E-01	2.81E-01	Tnf,21926:up|Tgfb2,21808:down|Ccl3,20302:up
T cell differentiation	GO:0030217	1.86E-01	2.82E-01	Ctla2a,13024:down|Ly9,17085:up|Cd4,12504:up|Clec4e,56619:up|Tnfsf9,21950:up|Bmp4,12159:up
cellular response to external stimulus	GO:0071496	1.87E-01	2.84E-01	Gas6,14456:up|Lmcd1,30937:up|Hmox1,15368:up|Sfrp4,20379:up|Tfrc,22042:up|Ankrd1,107765:down|Vdr,22337:down|Sfrp2,20319:up|Plod2,26432:down|Postn,50706:down
membrane protein proteolysis	GO:0033619	1.89E-01	2.85E-01	Tnf,21926:up|Ret,19713:up
cellular modified amino acid biosynthetic process	GO:0042398	1.89E-01	2.85E-01	Ggt5,23887:up|Gatm,67092:down
regulation of cofactor metabolic process	GO:0051193	1.89E-01	2.85E-01	Htr2a,15558:down|Igf1,16000:up
defense response to Gram-negative bacterium	GO:0050829	1.89E-01	2.85E-01	Adm,11535:up|Cd4,12504:up
protein localization to cell surface	GO:0034394	1.89E-01	2.85E-01	Tnf,21926:up|Gpm6b,14758:up
T cell selection	GO:0045058	1.89E-01	2.85E-01	Ly9,17085:up|Cd4,12504:up
regulation of actin filament-based movement	GO:1903115	1.89E-01	2.85E-01	Fxyd1,56188:up|Stc1,20855:up
unsaturated fatty acid biosynthetic process	GO:0006636	1.89E-01	2.85E-01	Ggt5,23887:up|Ptgs2,19225:down
anterior/posterior axis specification	GO:0009948	1.89E-01	2.85E-01	Wnt5a,22418:down|Bmp4,12159:up
cation transmembrane transport	GO:0098655	1.89E-01	2.86E-01	Htr2b,15559:down|Gas6,14456:up|Glp1r,14652:down|Fxyd1,56188:up|Kcnj15,16516:down|Fxyd2,11936:up|Bmp4,12159:up|Tgfb2,21808:down|Htr2a,15558:down|S100a1,20193:up|Slc40a1,53945:up
ribose phosphate biosynthetic process	GO:0046390	1.90E-01	2.86E-01	Htr2b,15559:down|Adm,11535:up|Htr2a,15558:down|Ptgir,19222:up|Uck2,80914:down|Ampd3,11717:up|Igf1,16000:up
pyruvate metabolic process	GO:0006090	1.90E-01	2.86E-01	Htr2a,15558:down|Igf1,16000:up|Cdo1,12583:up
negative regulation of T cell activation	GO:0050868	1.90E-01	2.86E-01	Ceacam1,26365:up|Cd24a,12484:down|Bmp4,12159:up

sensory perception	GO:0007600	1.93E-01	2.91E-01	Tnf,21926:up|Fgfr1,14182:down|Col11a1,12814:down|Ncam1,17967:down|Mme,17380:up|Ptgs2,19225:down|Il1rn,16181:up|Ednrb,13618:up|Igf1,16000:up|Epas1,13819:up|Mcoln3,171166:up|Htr2a,15558:down|Serpine2,20720:up
glycerophospholipid biosynthetic process	GO:0046474	1.94E-01	2.92E-01	Htr2b,15559:down|Fabp5,16592:up|Htr2a,15558:down
cAMP-mediated signaling	GO:0019933	1.95E-01	2.92E-01	Glp1r,14652:down|Ptgir,19222:up
cellular response to dsRNA	GO:0071359	1.95E-01	2.92E-01	Egfr,13649:down|Ifit1,15957:up
negative regulation of nuclear division	GO:0051784	1.95E-01	2.92E-01	Fgfr2,14183:up|Bmp4,12159:up
response to cAMP	GO:0051591	1.95E-01	2.92E-01	Cdo1,12583:up|Adipoq,11450:up
lipopolysaccharide-mediated signaling pathway	GO:0031663	1.95E-01	2.92E-01	Tnf,21926:up|Ccl12,20293:up
citrulline metabolic process	GO:0000052	1.97E-01	2.92E-01	Ddah1,69219:down
regulation of glomerular filtration	GO:0003093	1.97E-01	2.92E-01	Gas6,14456:up
pyramidal neuron differentiation	GO:0021859	1.97E-01	2.92E-01	Fgfr2,14183:up
angiogenesis involved in wound healing	GO:0060055	1.97E-01	2.92E-01	Hpse,15442:up
lens fiber cell development	GO:0070307	1.97E-01	2.92E-01	Fgfr2,14183:up
positive regulation of podosome assembly	GO:0071803	1.97E-01	2.92E-01	Tnf,21926:up
regulation of synaptic transmission, cholinergic	GO:0032222	1.97E-01	2.92E-01	Lama2,16773:down
negative regulation of release of sequestered calcium ion into cytosol	GO:0051280	1.97E-01	2.92E-01	Tgfb2,21808:down
regulation of immature T cell proliferation	GO:0033083	1.97E-01	2.92E-01	Bmp4,12159:up
regulation of centriole replication	GO:0046599	1.97E-01	2.92E-01	Plk2,20620:down
positive regulation of astrocyte differentiation	GO:0048711	1.97E-01	2.92E-01	Serpine2,20720:up
dendritic cell chemotaxis	GO:0002407	1.97E-01	2.92E-01	Gas6,14456:up
cellular response to hepatocyte growth factor stimulus	GO:0035729	1.97E-01	2.92E-01	Adamts12,239337:down
positive regulation of membrane protein ectodomain proteolysis	GO:0051044	1.97E-01	2.92E-01	Tnf,21926:up
positive regulation of transcription from RNA polymerase I promoter	GO:0045943	1.97E-01	2.92E-01	Rasl11a,68895:up
positive regulation of immune response to tumor cell	GO:0002839	1.97E-01	2.92E-01	Cd24a,12484:down
cellular response to platelet-derived growth factor stimulus	GO:0036120	1.97E-01	2.92E-01	Has2,15117:down
regulation of interleukin-8 biosynthetic process	GO:0045414	1.97E-01	2.92E-01	Tnf,21926:up
positive regulation of response to tumor cell	GO:0002836	1.97E-01	2.92E-01	Cd24a,12484:down
branching morphogenesis of a nerve	GO:0048755	1.97E-01	2.92E-01	Fgfr2,14183:up
blastoderm segmentation	GO:0007350	1.97E-01	2.92E-01	Wnt5a,22418:down
mitral valve development	GO:0003174	1.97E-01	2.92E-01	Bmpr1a,12166:down
insemination	GO:0007320	1.97E-01	2.92E-01	Serpine2,20720:up
development of secondary sexual characteristics	GO:0045136	1.97E-01	2.92E-01	Wnt5a,22418:down
glycoside metabolic process	GO:0016137	1.97E-01	2.92E-01	Akr1c18,105349:up
positive regulation of gene silencing by miRNA	GO:2000637	1.97E-01	2.92E-01	Egfr,13649:down

<table>
<tr><td>regulation of extracellular matrix disassembly</td><td>GO:0010715</td><td>1.97E-01</td><td>2.92E-01</td><td>Tgfb2,21808:down</td></tr>
<tr><td>UDP-N-acetylglucosamine metabolic process</td><td>GO:0006047</td><td>1.97E-01</td><td>2.92E-01</td><td>Gfpt2,14584:down</td></tr>
<tr><td>negative regulation of acute inflammatory response</td><td>GO:0002674</td><td>1.97E-01</td><td>2.92E-01</td><td>Pparg,19016:up</td></tr>
<tr><td>negative regulation of receptor binding</td><td>GO:1900121</td><td>1.97E-01</td><td>2.92E-01</td><td>Adipoq,11450:up</td></tr>
<tr><td>positive regulation of Rho protein signal transduction</td><td>GO:0035025</td><td>1.97E-01</td><td>2.92E-01</td><td>Col3a1,12825:down</td></tr>
<tr><td>regulation of gonadotropin secretion</td><td>GO:0032276</td><td>1.97E-01</td><td>2.92E-01</td><td>Inhba,16323:down</td></tr>
<tr><td>bronchus development</td><td>GO:0060433</td><td>1.97E-01</td><td>2.92E-01</td><td>Bmp4,12159:up</td></tr>
<tr><td>anagen</td><td>GO:0042640</td><td>1.97E-01</td><td>2.92E-01</td><td>Ptgs2,19225:down</td></tr>
<tr><td>AMP metabolic process</td><td>GO:0046033</td><td>1.97E-01</td><td>2.92E-01</td><td>Ampd3,11717:up</td></tr>
<tr><td>mast cell migration</td><td>GO:0097531</td><td>1.97E-01</td><td>2.92E-01</td><td>Figf,14205:up</td></tr>
<tr><td>regulation of transcription from RNA polymerase II promoter in response to hypoxia</td><td>GO:0061418</td><td>1.97E-01</td><td>2.92E-01</td><td>Vegfa,22339:down</td></tr>
<tr><td>cellular response to fluid shear stress</td><td>GO:0071498</td><td>1.97E-01</td><td>2.92E-01</td><td>Ptgs2,19225:down</td></tr>
<tr><td>lateral ventricle development</td><td>GO:0021670</td><td>1.97E-01</td><td>2.92E-01</td><td>Uchl5,56207:up</td></tr>
<tr><td>CD4-positive, alpha-beta T cell lineage commitment</td><td>GO:0043373</td><td>1.97E-01</td><td>2.92E-01</td><td>Ly9,17085:up</td></tr>
<tr><td>semaphorin-plexin signaling pathway involved in axon guidance</td><td>GO:1902287</td><td>1.97E-01</td><td>2.92E-01</td><td>Plxnd1,67784:up</td></tr>
<tr><td>response to hepatocyte growth factor</td><td>GO:0035728</td><td>1.97E-01</td><td>2.92E-01</td><td>Adamts12,239337:down</td></tr>
<tr><td>otic vesicle development</td><td>GO:0071599</td><td>1.97E-01</td><td>2.92E-01</td><td>Fgfr2,14183:up</td></tr>
<tr><td>regulation of cell cycle</td><td>GO:0051726</td><td>1.97E-01</td><td>2.93E-01</td><td>Hpgd,15446:up|Inhba,16323:down|Nabp1,109019:down|Igf2,16002:up|Tnf,21926:up|Plk2,20620:down|Egfr,13649:down|Fgfr1,14182:down|Sfrp1,20377:up|Susd2,71733:up|Wnt5a,22418:down|Ptgs2,19225:down|Bmp4,12159:up|Igf1,16000:up|Tgfb2,21808:down|Fgfr2,14183:up</td></tr>
<tr><td>cellular macromolecule metabolic process</td><td>GO:0044260</td><td>1.99E-01</td><td>2.95E-01</td><td>Fstl3,83554:down|Igfbp4,16010:up|Adm,11535:up|Flrt2,399558:down|Tfec,21426:up|Sfrp4,20379:up|C3,12266:up|Ankrd1,107765:down|Lyl1,17095:up|Gstt1,14871:up|Igfbp3,16009:up|Cd109,235505:down|Loxl2,94352:down|Plk2,20620:down|Loxl3,16950:down|Trf,22041:up|Cd24a,12484:down|Fkbp14,231997:down|Wnt5a,22418:down|Mark1,226778:up|Il1rn,16181:up|Igf1,16000:up|Adipoq,11450:up|Bmp4,12159:up|Tgfb2,21808:down|Hpse,15442:up|F3,14066:up|Fgfr2,14183:up|Htr2b,15559:down|Kng1,16644:up|Prl2c2,18811:up|Efemp1,216616:up|Ccl6,20305:up|Slpi,20568:up|Aspn,66695:down|Saa3,20210:up|Vdr,22337:down|Pdia5,72599:down|Fabp4,11770:up|Nabp1,109019:down|Htr2a,15558:down|Oas3,246727:up|Slc40a1,53945:up|Gas6,14456:up|Egfr,13649:down|Col11a1,12814:down|Sfrp1,20377:up|C1qtnf3,81799:down|Tgfbr3,21814:up|Tfrc,22042:up|Ceacam1,26365:up|Ccl8,20307:up|Sfrp2,20319:up|Serpina3g,20715:up|Fam20a,208659:down|Npnt,114249:up|Tnfrsf11b,18383:up|Prkar2b,19088:up|S100a1,20193:up|Ice1,218333:down|Neurl3,214854:down|Ccl2,20296:up|Ccl7,20306:up|Pdzrn3,55983:down|Pdgfc,54635:down|Cst7,13011:up|Ednrb,13618:up|Mmp13,17386:up|Sema7a,20361:down|Epas1,13819:up|Htra3,78558:down|Uchl5,56207:up|Ddah1,69219:down|Sdpr,20324:down|Fkbp10,14230:down|Man2a2,140481:up|Dok2,13449:up|Mamdc2,71738:down|Ptx3,19288:up|Bmpr1a,12166:down|Ccl12,20293:up|Ccl4,20303:up|Chst11,58250:down|Ret,19713:up|Vegfb,22340:up|Sdf2l1,64136:up|Plpp3,67916:up|Creb3l1,26427:down|Kitl,17311:up|P4ha1,18451:down|Ccl9,20308:up|Inhba,16323:down|Aoah,27052:up|Rasl11a,68895:up|Bnc2,242509:down|Plat,18791:down|Ccl3,20302:up|Tfpi2,21789:down|Ctla2a,13024:down|Crlf1,12931:down|Vegfa,22339:down|Serpine2,20720:up|Adamts12,239337:down|Lrrc15,74488:down|Nxn,18230:down|Igf2,16002:up|Pparg,19016:up|Tnf,21926:up|Glp1r,14652:down|Lmcd1,30937:up|Fgfr1,14182:down|Has2,15117:down|Hmox1,15368:up|Sulf1,240725:down|Cd4,12504:up|Grem1,23892:up|Col3a1,12825:down|Phf11d,219132:up|Tbx15,21384:down|Serping1,12258:up|Errfi1,74155:down|Enpp1,18605:down</td></tr>
<tr><td>regulation of systemic arterial blood pressure</td><td>GO:0003073</td><td>1.99E-01</td><td>2.95E-01</td><td>Gas6,14456:up|Ddah1,69219:down|Postn,50706:down</td></tr>
<tr><td>intracellular protein transport</td><td>GO:0006886</td><td>2.00E-01</td><td>2.97E-01</td><td>Ice1,218333:down|Ccl2,20296:up|Gas6,14456:up|Tnf,21926:up|Akr1c18,105349:up|Cd24a,12484:down|Egfr,13649:down|Sec23a,20334:down|C1qtnf3,81799:down|Bmpr1a,12166:down|Ptgs2,19225:down|Grem1,23892:up|Adipoq,11450:up|Bmp4,12159:up|Igf1,16000:up|Ceacam1,26365:up|Sfrp2,20319:up|Tgfb2,21808:down</td></tr>
<tr><td>response to glucose</td><td>GO:0009749</td><td>2.02E-01</td><td>2.99E-01</td><td>Gas6,14456:up|Glp1r,14652:down|Igf1,16000:up|Adipoq,11450:up</td></tr>
<tr><td>phosphatidylinositol biosynthetic process</td><td>GO:0006661</td><td>2.02E-01</td><td>2.99E-01</td><td>Htr2b,15559:down|Htr2a,15558:down</td></tr>
</table>

developmental pigmentation	GO:0048066	2.02E-01	2.99E-01	Ednrb,13618:up|Kitl,17311:up
secondary metabolic process	GO:0019748	2.02E-01	2.99E-01	Akr1c18,105349:up|Wnt5a,22418:down
positive regulation of tyrosine phosphorylation of STAT protein	GO:0042531	2.02E-01	2.99E-01	Crlf1,12931:down|Igf1,16000:up
regulation of cellular component size	GO:0032535	2.07E-01	3.07E-01	Plxnd1,67784:up|Wnt5a,22418:down|Sema7a,20361:down|Tnfrsf12a,27279:down|Crabp2,12904:down|Ret,19713:up|Cxcl12,20315:up|Vegfa,22339:down
carboxylic acid catabolic process	GO:0046395	2.07E-01	3.07E-01	Ddah1,69219:down|Npl,74091:up|Hal,15109:up|Cdo1,12583:up|Adipoq,11450:up
organic acid catabolic process	GO:0016054	2.07E-01	3.07E-01	Ddah1,69219:down|Npl,74091:up|Hal,15109:up|Cdo1,12583:up|Adipoq,11450:up
import into cell	GO:0098657	2.09E-01	3.09E-01	Trf,22041:up|Gpm6b,14758:up
morphogenesis of an epithelial sheet	GO:0002011	2.09E-01	3.09E-01	Col5a1,12831:down|Wnt5a,22418:down
mating	GO:0007618	2.09E-01	3.09E-01	Serpine2,20720:up|Ednrb,13618:up
regulation of vasodilation	GO:0042312	2.09E-01	3.09E-01	Adm,11535:up|Egfr,13649:down
striated muscle cell development	GO:0055002	2.09E-01	3.09E-01	Vegfa,22339:down|Wisp1,22402:down|Igf1,16000:up|Bmp4,12159:up
pyrimidine ribonucleotide biosynthetic process	GO:0009220	2.11E-01	3.09E-01	Uck2,80914:down
pyrimidine ribonucleoside biosynthetic process	GO:0046132	2.11E-01	3.09E-01	Uck2,80914:down
microvillus assembly	GO:0030033	2.11E-01	3.09E-01	Prl2c2,18811:up
positive regulation of transforming growth factor beta production	GO:0071636	2.11E-01	3.09E-01	Ptgs2,19225:down
serotonin secretion	GO:0001820	2.11E-01	3.09E-01	Maob,109731:up
regulation of ketone biosynthetic process	GO:0010566	2.11E-01	3.09E-01	Akr1c18,105349:up
interleukin-8 biosynthetic process	GO:0042228	2.11E-01	3.09E-01	Tnf,21926:up
negative regulation of epidermal cell differentiation	GO:0045605	2.11E-01	3.09E-01	Tgfb2,21808:down
regulation of cerebellar granule cell precursor proliferation	GO:0021936	2.11E-01	3.09E-01	Igf1,16000:up
nonribosomal peptide biosynthetic process	GO:0019184	2.11E-01	3.09E-01	Ggt5,23887:up
positive regulation of cholesterol efflux	GO:0010875	2.11E-01	3.09E-01	Adipoq,11450:up
magnesium ion homeostasis	GO:0010960	2.11E-01	3.09E-01	Egfr,13649:down
positive regulation of posttranscriptional gene silencing	GO:0060148	2.11E-01	3.09E-01	Egfr,13649:down
positive regulation of cardiac muscle cell apoptotic process	GO:0010666	2.11E-01	3.09E-01	Igfbp3,16009:up
embryonic skeletal joint morphogenesis	GO:0060272	2.11E-01	3.09E-01	Bmp4,12159:up
protein deglycosylation	GO:0006517	2.11E-01	3.09E-01	Man2a2,140481:up
response to cholesterol	GO:0070723	2.11E-01	3.09E-01	Inhba,16323:down
detection of temperature stimulus involved in sensory perception of pain	GO:0050965	2.11E-01	3.09E-01	Htr2a,15558:down
immature T cell proliferation	GO:0033079	2.11E-01	3.09E-01	Bmp4,12159:up
nucleoside transport	GO:0015858	2.11E-01	3.09E-01	Slc29a1,63959:up
cellular defense response	GO:0006968	2.11E-01	3.09E-01	Clec2d,93694:up
response to platelet-derived growth factor	GO:0036119	2.11E-01	3.09E-01	Has2,15117:down
positive regulation of macrophage chemotaxis	GO:0010759	2.11E-01	3.09E-01	Ccl2,20296:up

positive regulation of tyrosine phosphorylation of Stat5 protein	GO:0042523	2.11E-01	3.09E-01	Igf1,16000:up
hyaluronan biosynthetic process	GO:0030213	2.11E-01	3.09E-01	Has2,15117:down
response to lipoprotein particle	GO:0055094	2.11E-01	3.09E-01	Pparg,19016:up
semaphorin-plexin signaling pathway involved in neuron projection guidance	GO:1902285	2.11E-01	3.09E-01	Plxnd1,67784:up
positive regulation of insulin receptor signaling pathway	GO:0046628	2.11E-01	3.09E-01	Igf2,16002:up
regulation of acute inflammatory response to antigenic stimulus	GO:0002864	2.11E-01	3.09E-01	C3,12266:up
positive regulation of peptidyl-serine phosphorylation of STAT protein	GO:0033141	2.11E-01	3.09E-01	Ret,19713:up
regulation of timing of cell differentiation	GO:0048505	2.11E-01	3.09E-01	Serpine2,20720:up
positive regulation of sequestering of calcium ion	GO:0051284	2.11E-01	3.09E-01	Tgfb2,21808:down
renal absorption	GO:0070293	2.11E-01	3.09E-01	Adipoq,11450:up
I-kappaB phosphorylation	GO:0007252	2.11E-01	3.09E-01	Saa3,20210:up
cellular response to exogenous dsRNA	GO:0071360	2.11E-01	3.09E-01	Ifit1,15957:up
cerebellar granular layer development	GO:0021681	2.11E-01	3.09E-01	Serpine2,20720:up
regulation of epithelial cell differentiation involved in kidney development	GO:2000696	2.11E-01	3.09E-01	Adipoq,11450:up
thalamus development	GO:0021794	2.11E-01	3.09E-01	Ncam1,17967:down
polyol catabolic process	GO:0046174	2.11E-01	3.09E-01	Sord,20322:up
lung secretory cell differentiation	GO:0061140	2.11E-01	3.09E-01	Igf1,16000:up
positive regulation of striated muscle cell apoptotic process	GO:0010663	2.11E-01	3.09E-01	Igfbp3,16009:up
detection of temperature stimulus involved in sensory perception	GO:0050961	2.11E-01	3.09E-01	Htr2a,15558:down
positive regulation of action potential	GO:0045760	2.11E-01	3.09E-01	Tnf,21926:up
eosinophil migration	GO:0072677	2.11E-01	3.09E-01	Ccl2,20296:up
apoptotic DNA fragmentation	GO:0006309	2.11E-01	3.09E-01	Igfbp3,16009:up
cellular amino acid metabolic process	GO:0006520	2.13E-01	3.11E-01	P4ha1,18451:down|Ddah1,69219:down|Gatm,67092:down|Gfpt2,14584:down|Serinc2,230779:down|Hal,15109:up|Cdo1,12583:up
alcohol biosynthetic process	GO:0046165	2.13E-01	3.11E-01	Vdr,22337:down|Tnf,21926:up|Akr1c18,105349:up|Fabp5,16592:up
response to hexose	GO:0009746	2.13E-01	3.11E-01	Gas6,14456:up|Glp1r,14652:down|Igf1,16000:up|Adipoq,11450:up
immunoglobulin production	GO:0002377	2.13E-01	3.11E-01	Ceacam1,26365:up|Tnf,21926:up|Tfrc,22042:up
positive regulation of autophagy	GO:0010508	2.14E-01	3.12E-01	Plk2,20620:down|Lmcd1,30937:up|Plod2,26432:down|Hmox1,15368:up|Sfrp4,20379:up
inorganic anion transmembrane transport	GO:0098661	2.15E-01	3.14E-01	Ank,11732:down|Fxyd1,56188:up
adenylate cyclase-modulating G-protein coupled receptor signaling pathway	GO:0007188	2.17E-01	3.16E-01	Htr2b,15559:down|Glp1r,14652:down|Htr2a,15558:down|Ptgir,19222:up
response to monosaccharide	GO:0034284	2.17E-01	3.16E-01	Gas6,14456:up|Glp1r,14652:down|Igf1,16000:up|Adipoq,11450:up
pallium development	GO:0021543	2.17E-01	3.16E-01	Fgfr1,14182:down|Egfr,13649:down|Cx3cr1,13051:down|Col3a1,12825:down

endothelial cell development	GO:0001885	2.22E-01	3.23E-01	Tnf,21926:up|Stc1,20855:up
L-amino acid transport	GO:0015807	2.22E-01	3.23E-01	Tnf,21926:up|Serinc2,230779:down
organic acid transmembrane transport	GO:1903825	2.22E-01	3.23E-01	Emb,13723:up|Slc16a4,229699:down
regulation of nucleobase-containing compound metabolic process	GO:0019219	2.22E-01	3.23E-01	Fstl3,83554:down|Ice1,218333:down|Adm,11535:up|Tfec,21426:up|Sfrp4,20379:up|Pdgfc,54635:down|Ednrb,13618:up|Epas1,13819:up|Ankrd1,107765:down|Htra3,78558:down|Uchl5,56207:up|Lyl1,17095:up|Sdpr,20324:down|Igfbp3,16009:up|Loxl2,94352:down|Loxl3,16950:down|Trf,22041:up|Wnt5a,22418:down|Bmpr1a,12166:down|Ccl4,20303:up|Adipoq,11450:up|Bmp4,12159:up|Igf1,16000:up|Ret,19713:up|Plpp3,67916:up|Ptgir,19222:up|Creb3l1,26427:down|Fgfr2,14183:up|Kitl,17311:up|Prl2c2,18811:up|Efemp1,216616:up|Inhba,16323:down|Rasl11a,68895:up|Bnc2,242509:down|Ccl3,20302:up|Vdr,22337:down|Fabp4,11770:up|Vegfa,22339:down|Htr2a,15558:down|Oas3,246727:up|Igf2,16002:up|Slc40a1,53945:up|Pparg,19016:up|Gas6,14456:up|Tnf,21926:up|Glp1r,14652:down|Lmcd1,30937:up|Fgfr1,14182:down|Egfr,13649:down|Hmox1,15368:up|Sfrp1,20377:up|Sulf1,240725:down|Tgfbr3,21814:up|Tfrc,22042:up|Grem1,23892:up|Phf11d,219132:up|Sfrp2,20319:up|Tbx15,21384:down|Npnt,114249:up|S100a1,20193:up
nucleoside diphosphate metabolic process	GO:0009132	2.22E-01	3.24E-01	Htr2a,15558:down|Ampd3,11717:up|Igf1,16000:up
negative regulation of cytoplasmic transport	GO:1903650	2.22E-01	3.24E-01	Gas6,14456:up|C1qtnf3,81799:down|Tgfb2,21808:down
cardiac muscle contraction	GO:0060048	2.22E-01	3.24E-01	Fxyd1,56188:up|Vegfb,22340:up|Stc1,20855:up
single-organism carbohydrate catabolic process	GO:0044724	2.22E-01	3.24E-01	Sord,20322:up|Htr2a,15558:down|Igf1,16000:up
regulation of macromolecule biosynthetic process	GO:0010556	2.23E-01	3.24E-01	Fstl3,83554:down|Ice1,218333:down|Tfec,21426:up|Sfrp4,20379:up|Ccl2,20296:up|Pdgfc,54635:down|Ednrb,13618:up|Epas1,13819:up|Ankrd1,107765:down|Htra3,78558:down|Uchl5,56207:up|Lyl1,17095:up|Sdpr,20324:down|Loxl2,94352:down|Loxl3,16950:down|Trf,22041:up|Wnt5a,22418:down|Bmpr1a,12166:down|Ccl4,20303:up|Adipoq,11450:up|Bmp4,12159:up|Igf1,16000:up|Ret,19713:up|Prg4,96875:down|Tgfb2,21808:down|Plpp3,67916:up|Creb3l1,26427:down|Fgfr2,14183:up|Kitl,17311:up|Prl2c2,18811:up|Efemp1,216616:up|Inhba,16323:down|Rasl11a,68895:up|Bnc2,242509:down|Ccl3,20302:up|Vdr,22337:down|Fabp4,11770:up|Vegfa,22339:down|Igf2,16002:up|Slc40a1,53945:up|Pparg,19016:up|Gas6,14456:up|Tnf,21926:up|Glp1r,14652:down|Lmcd1,30937:up|Fgfr1,14182:down|Egfr,13649:down|Has2,15117:down|Hmox1,15368:up|Sfrp1,20377:up|Sulf1,240725:down|Tgfbr3,21814:up|Grem1,23892:up|Phf11d,219132:up|Sfrp2,20319:up|Tbx15,21384:down|Npnt,114249:up|Errfi1,74155:down|Enpp1,18605:down|S100a1,20193:up
regulation of muscle contraction	GO:0006937	2.24E-01	3.25E-01	Fxyd1,56188:up|Ptgs2,19225:down|Npnt,114249:up|Stc1,20855:up
biosynthetic process	GO:0009058	2.25E-01	3.25E-01	Fstl3,83554:down|Adm,11535:up|Tfec,21426:up|Sfrp4,20379:up|C3,12266:up|Ankrd1,107765:down|Serpinh1,12406:down|Lyl1,17095:up|Ggt5,23887:up|Loxl2,94352:down|Loxl3,16950:down|Trf,22041:up|Wnt5a,22418:down|Adipoq,11450:up|Bmp4,12159:up|Igf1,16000:up|Tgfb2,21808:down|Ptgir,19222:up|Fgfr2,14183:up|Htr2b,15559:down|Prl2c2,18811:up|Efemp1,216616:up|Vdr,22337:down|Fabp4,11770:up|Htr2a,15558:down|Slc40a1,53945:up|Gas6,14456:up|Egfr,13649:down|Sfrp1,20377:up|C1qtnf3,81799:down|Tgfbr3,21814:up|Ceacam1,26365:up|Sfrp2,20319:up|Npnt,114249:up|S100a1,20193:up|Ice1,218333:down|Aldh1a2,19378:up|Ccl2,20296:up|Pdgfc,54635:down|Ednrb,13618:up|Ampd3,11717:up|Epas1,13819:up|Htra3,78558:down|Sord,20322:up|Uchl5,56207:up|Ddah1,69219:down|Sdpr,20324:down|Cdo1,12583:up|Man2a2,140481:up|Ptx3,19288:up|Akr1c18,105349:up|Fabp5,16592:up|Bmpr1a,12166:down|Ccl4,20303:up|Chst11,58250:down|Gatm,67092:down|Ret,19713:up|Prg4,96875:down|Vegfb,22340:up|Sdf2l1,64136:up|Pdxk,216134:up|Aldh1a3,56847:down|Plpp3,67916:up|Creb3l1,26427:down|Kitl,17311:up|Rasl11a,68895:up|Inhba,16323:down|Bnc2,242509:down|Ccl3,20302:up|Vegfa,22339:down|Gfpt2,14584:down|Igf2,16002:up|Pparg,19016:up|Tnf,21926:up|Lmcd1,30937:up|Glp1r,14652:down|Fgfr1,14182:down|Hmox1,15368:up|Lpl,16956:up|Has2,15117:down|Sulf1,240725:down|Ptgs2,19225:down|Grem1,23892:up|Phf11d,219132:up|Tbx15,21384:down|Col5a1,12831:down|Uck2,80914:down|Errfi1,74155:down|Enpp1,18605:down
detection of mechanical stimulus involved in sensory perception of sound	GO:0050910	2.25E-01	3.25E-01	Col11a1,12814:down
kidney mesenchyme development	GO:0072074	2.25E-01	3.25E-01	Bmp4,12159:up
positive regulation of nitric-oxide synthase biosynthetic process	GO:0051770	2.25E-01	3.25E-01	Ccl2,20296:up
cell differentiation involved in metanephros development	GO:0072202	2.25E-01	3.25E-01	Adipoq,11450:up
regulation of microvillus organization	GO:0032530	2.25E-01	3.25E-01	Prl2c2,18811:up
renal system process involved in regulation of blood volume	GO:0001977	2.25E-01	3.25E-01	Gas6,14456:up
regulation of anion transmembrane transport	GO:1903959	2.25E-01	3.25E-01	Fxyd1,56188:up
decidualization	GO:0046697	2.25E-01	3.25E-01	Ptgs2,19225:down
sulfur amino acid biosynthetic process	GO:0000097	2.25E-01	3.25E-01	Cdo1,12583:up
glomerular mesangium development	GO:0072109	2.25E-01	3.25E-01	Bmp4,12159:up

negative regulation of calcium ion transport into cytosol	GO:0010523	2.25E-01	3.25E-01	Tgfb2,21808:down
lipoprotein localization	GO:0044872	2.25E-01	3.25E-01	Pparg,19016:up
regulation of development, heterochronic	GO:0040034	2.25E-01	3.25E-01	Serpine2,20720:up
regulation of cell proliferation involved in kidney development	GO:1901722	2.25E-01	3.25E-01	Bmp4,12159:up
cardiac conduction system development	GO:0003161	2.25E-01	3.25E-01	Bmpr1a,12166:down
lipoprotein transport	GO:0042953	2.25E-01	3.25E-01	Pparg,19016:up
penile erection	GO:0043084	2.25E-01	3.25E-01	Ednrb,13618:up
alpha-beta T cell lineage commitment	GO:0002363	2.25E-01	3.25E-01	Ly9,17085:up
positive regulation of fibroblast migration	GO:0010763	2.25E-01	3.25E-01	Fgfr1,14182:down
regulation of phagocytosis, engulfment	GO:0060099	2.25E-01	3.25E-01	Pparg,19016:up
leukotriene metabolic process	GO:0006691	2.25E-01	3.25E-01	Ggt5,23887:up
negative regulation of lipid storage	GO:0010888	2.25E-01	3.25E-01	Pparg,19016:up
pyrimidine ribonucleotide metabolic process	GO:0009218	2.25E-01	3.25E-01	Uck2,80914:down
T-helper 17 cell differentiation	GO:0072539	2.25E-01	3.25E-01	Ly9,17085:up
positive regulation of nitric-oxide synthase activity	GO:0051000	2.25E-01	3.25E-01	Htr2b,15559:down
GABAergic neuron differentiation	GO:0097154	2.25E-01	3.25E-01	Inhba,16323:down
regulation of peptidyl-serine phosphorylation of STAT protein	GO:0033139	2.25E-01	3.25E-01	Ret,19713:up
cell wall macromolecule metabolic process	GO:0044036	2.25E-01	3.25E-01	Sdf2l1,64136:up
positive regulation of execution phase of apoptosis	GO:1900119	2.25E-01	3.25E-01	Igfbp3,16009:up
myoblast proliferation	GO:0051450	2.25E-01	3.25E-01	Igf1,16000:up
nose development	GO:0043584	2.25E-01	3.25E-01	Aldh1a3,56847:down
T cell chemotaxis	GO:0010818	2.25E-01	3.25E-01	Wnt5a,22418:down
positive regulation of protein import into nucleus, translocation	GO:0033160	2.25E-01	3.25E-01	Igf1,16000:up
positive regulation of transcription regulatory region DNA binding	GO:2000679	2.25E-01	3.25E-01	Igf1,16000:up
negative regulation of oligodendrocyte differentiation	GO:0048715	2.25E-01	3.25E-01	Bmp4,12159:up
cellular response to misfolded protein	GO:0071218	2.25E-01	3.25E-01	Sdf2l1,64136:up
heart trabecula formation	GO:0060347	2.25E-01	3.25E-01	Tgfbr3,21814:up
defense response to virus	GO:0051607	2.26E-01	3.25E-01	Lmcd1,30937:up|Cxcl9,17329:up|Oasl1,231655:up|Ifit1,15957:up|Oas3,246727:up|Ifit3,15959:up
cellular response to abiotic stimulus	GO:0071214	2.27E-01	3.27E-01	Ankrd1,107765:down|Hvcn1,74096:up|Sfrp2,20319:up|Sfrp1,20377:up|Mme,17380:up
regulation of G-protein coupled receptor protein signaling pathway	GO:0008277	2.27E-01	3.27E-01	Htr2b,15559:down|Adm,11535:up|C3,12266:up
organophosphate metabolic process	GO:0019637	2.28E-01	3.28E-01	Htr2b,15559:down|Adm,11535:up|Ampd3,11717:up|Enpp5,83965:up|Vegfa,22339:down|Htr2a,15558:down|Gfpt2,14584:down|Cbr2,12409:up|Enpp3,209558:down|Fabp5,16592:up|Wnt5a,22418:down|Serinc2,230779:down|Igf1,16000:up|Pdxk,216134:up|Plpp3,67916:up|Ptgir,19222:up|Enpp1,18605:down|Uck2,80914:down

positive regulation of hormone secretion	GO:0046887	2.28E-01	3.28E-01	Glp1r,14652:down|Fgfr1,14182:down|Egfr,13649:down|C1qtnf3,81799:down
negative regulation of epithelial cell migration	GO:0010633	2.29E-01	3.28E-01	Tgfbr3,21814:up|Stc1,20855:up
negative regulation of autophagy	GO:0010507	2.29E-01	3.28E-01	Htr2b,15559:down|Hmox1,15368:up
organic hydroxy compound catabolic process	GO:1901616	2.29E-01	3.28E-01	Sord,20322:up|Akr1c18,105349:up
regulation of epidermal growth factor receptor signaling pathway	GO:0042058	2.29E-01	3.28E-01	Ceacam1,26365:up|Errfi1,74155:down
regulation of phospholipid metabolic process	GO:1903725	2.29E-01	3.28E-01	Htr2b,15559:down|Htr2a,15558:down
positive regulation of cell cycle process	GO:0090068	2.30E-01	3.31E-01	Tnf,21926:up|Fgfr1,14182:down|Wnt5a,22418:down|Igf2,16002:up|Igf1,16000:up
peptide hormone secretion	GO:0030072	2.32E-01	3.33E-01	Tnf,21926:up|Pparg,19016:up|Glp1r,14652:down|Egfr,13649:down|Sfrp1,20377:up|Il1rn,16181:up
response to extracellular stimulus	GO:0009991	2.34E-01	3.36E-01	Pparg,19016:up|Gas6,14456:up|Lmcd1,30937:up|Hmox1,15368:up|Sfrp4,20379:up|Tfrc,22042:up|Vdr,22337:down|Sfrp2,20319:up|Gatm,67092:down|Plod2,26432:down|Postn,50706:down
positive regulation of alpha-beta T cell activation	GO:0046635	2.35E-01	3.37E-01	Ceacam1,26365:up|Cd24a,12484:down
regulation of plasma lipoprotein particle levels	GO:0097006	2.35E-01	3.37E-01	Lpl,16956:up|Adipoq,11450:up
superoxide metabolic process	GO:0006801	2.35E-01	3.37E-01	Tnf,21926:up|Egfr,13649:down
negative regulation of peptide hormone secretion	GO:0090278	2.35E-01	3.37E-01	Pparg,19016:up|Sfrp1,20377:up
cellular response to ionizing radiation	GO:0071479	2.35E-01	3.37E-01	Sfrp2,20319:up|Sfrp1,20377:up
positive regulation of cysteine-type endopeptidase activity involved in apoptotic process	GO:0043280	2.37E-01	3.39E-01	Tnf,21926:up|Pparg,19016:up|F3,14066:up
purine ribonucleotide metabolic process	GO:0009150	2.37E-01	3.39E-01	Htr2b,15559:down|Adm,11535:up|Wnt5a,22418:down|Ampd3,11717:up|Igf1,16000:up|Htr2a,15558:down|Vegfa,22339:down|Ptgir,19222:up|Enpp1,18605:down
cellular catabolic process	GO:0044248	2.38E-01	3.40E-01	Sfrp4,20379:up|Mmp13,17386:up|Enpp5,83965:up|Uchl5,56207:up|Sord,20322:up|Ddah1,69219:down|Blvrb,233016:up|Npl,74091:up|Igfbp3,16009:up|Enpp3,209558:down|Cdo1,12583:up|Plk2,20620:down|Akr1c18,105349:up|Maob,109731:up|Adipoq,11450:up|Hal,15109:up|Igf1,16000:up|Sdf2l1,64136:up|Hpse,15442:up|Htr2b,15559:down|Hp,15439:up|Dram1,71712:up|Plod2,26432:down|Htr2a,15558:down|Adamts12,239337:down|Anpep,16790:up|Tnf,21926:up|Lmcd1,30937:up|Lpl,16956:up|Hmox1,15368:up|Pxdn,69675:down|Enpp1,18605:down
positive regulation of cell size	GO:0045793	2.40E-01	3.40E-01	Ret,19713:up
regulation of type I interferon-mediated signaling pathway	GO:0060338	2.40E-01	3.40E-01	Wnt5a,22418:down
positive regulation of cellular extravasation	GO:0002693	2.40E-01	3.40E-01	Ccl2,20296:up
female genitalia development	GO:0030540	2.40E-01	3.40E-01	Wnt5a,22418:down
regulation of developmental pigmentation	GO:0048070	2.40E-01	3.40E-01	Kitl,17311:up
pyrimidine nucleoside biosynthetic process	GO:0046134	2.40E-01	3.40E-01	Uck2,80914:down
calcium-dependent cell-cell adhesion via plasma membrane cell adhesion molecules	GO:0016339	2.40E-01	3.40E-01	Ceacam1,26365:up
embryonic skeletal joint development	GO:0072498	2.40E-01	3.40E-01	Bmp4,12159:up
negative regulation of neurotransmitter transport	GO:0051589	2.40E-01	3.40E-01	Gpm6b,14758:up
epithelial cell differentiation involved in prostate gland development	GO:0060742	2.40E-01	3.40E-01	Fgfr2,14183:up
regulation of superoxide anion generation	GO:0032928	2.40E-01	3.40E-01	Egfr,13649:down
amino sugar catabolic process	GO:0046348	2.40E-01	3.40E-01	Npl,74091:up

negative regulation of glycoprotein metabolic process	GO:1903019	2.40E-01	3.40E-01	Ptx3,19288:up
positive regulation of meiotic nuclear division	GO:0045836	2.40E-01	3.40E-01	Wnt5a,22418:down
negative regulation of interleukin-1 beta production	GO:0032691	2.40E-01	3.40E-01	Errfi1,74155:down
protein hydroxylation	GO:0018126	2.40E-01	3.40E-01	P4ha1,18451:down
cell wall organization or biogenesis	GO:0071554	2.40E-01	3.40E-01	Sdf2l1,64136:up
positive regulation of extracellular matrix organization	GO:1903055	2.40E-01	3.40E-01	Tgfb2,21808:down
CD4-positive or CD8-positive, alpha-beta T cell lineage commitment	GO:0043369	2.40E-01	3.40E-01	Ly9,17085:up
epithelial to mesenchymal transition involved in endocardial cushion formation	GO:0003198	2.40E-01	3.40E-01	Tgfb2,21808:down
negative regulation of phagocytosis	GO:0050765	2.40E-01	3.40E-01	Adipoq,11450:up
melanin biosynthetic process	GO:0042438	2.40E-01	3.40E-01	Wnt5a,22418:down
aldehyde biosynthetic process	GO:0046184	2.40E-01	3.40E-01	Pdxk,216134:up
response to sterol	GO:0036314	2.40E-01	3.40E-01	Inhba,16323:down
positive regulation of endothelial cell differentiation	GO:0045603	2.40E-01	3.40E-01	Bmp4,12159:up
norepinephrine metabolic process	GO:0042415	2.40E-01	3.40E-01	Epas1,13819:up
regulation of nuclease activity	GO:0032069	2.40E-01	3.40E-01	Oas3,246727:up
positive regulation of p38MAPK cascade	GO:1900745	2.40E-01	3.40E-01	Vegfa,22339:down
positive regulation of cGMP metabolic process	GO:0030825	2.40E-01	3.40E-01	Wnt5a,22418:down
telencephalon regionalization	GO:0021978	2.40E-01	3.40E-01	Bmp4,12159:up
positive regulation of histone deacetylation	GO:0031065	2.40E-01	3.40E-01	Vegfa,22339:down
regulation of cell proliferation involved in heart morphogenesis	GO:2000136	2.40E-01	3.40E-01	Bmp4,12159:up
pancreatic juice secretion	GO:0030157	2.40E-01	3.40E-01	Mmp13,17386:up
regulation of bicellular tight junction assembly	GO:2000810	2.40E-01	3.40E-01	Tnf,21926:up
response to UV-B	GO:0010224	2.40E-01	3.40E-01	Mme,17380:up
negative regulation of nucleobase-containing compound metabolic process	GO:0045934	2.40E-01	3.40E-01	Sfrp4,20379:up|Ednrb,13618:up|Epas1,13819:up|Ankrd1,107765:down|Vdr,22337:down|Fabp4,11770:up|Vegfa,22339:down|Gas6,14456:up|Pparg,19016:up|Tnf,21926:up|Loxl2,94352:down|Loxl3,16950:down|Lmcd1,30937:up|Fgfr1,14182:down|Hmox1,15368:up|Sfrp1,20377:up|Wnt5a,22418:down|Ccl4,20303:up|Grem1,23892:up|Adipoq,11450:up|Bmp4,12159:up|Sfrp2,20319:up|Fgfr2,14183:up|S100a1,20193:up
transmembrane transport	GO:0055085	2.40E-01	3.40E-01	Htr2b,15559:down|Ednrb,13618:up|Cxcl12,20315:up|Htr2a,15558:down|Slc16a4,229699:down|Slc40a1,53945:up|Gas6,14456:up|Fxyd1,56188:up|Hvcn1,74096:up|Glp1r,14652:down|Ccl12,20293:up|Stc1,20855:up|Kcnj15,16516:down|Fxyd2,11936:up|Bmp4,12159:up|Ank,11732:down|Tgfb2,21808:down|Emb,13723:up|S100a1,20193:up
cellular response to peptide hormone stimulus	GO:0071375	2.40E-01	3.41E-01	Pparg,19016:up|Enpp1,18605:down|Igf2,16002:up|Igf1,16000:up|Adipoq,11450:up
cellular response to extracellular stimulus	GO:0031668	2.42E-01	3.42E-01	Gas6,14456:up|Lmcd1,30937:up|Hmox1,15368:up|Sfrp4,20379:up|Tfrc,22042:up|Vdr,22337:down|Sfrp2,20319:up|Plod2,26432:down|Postn,50706:down
platelet aggregation	GO:0070527	2.42E-01	3.42E-01	Ceacam1,26365:up|Serpine2,20720:up
T cell differentiation involved in immune response	GO:0002292	2.42E-01	3.42E-01	Ly9,17085:up|Clec4e,56619:up
negative regulation of protein import into nucleus	GO:0042308	2.42E-01	3.42E-01	Gas6,14456:up|C1qtnf3,81799:down
muscle fiber development	GO:0048747	2.42E-01	3.42E-01	Vegfa,22339:down|Bmp4,12159:up

protein complex subunit organization	GO:0071822	2.42E-01	3.43E-01	Fam101b,76566:down|P4ha1,18451:down|Col5a2,12832:down|Ice1,218333:down|Pdgfc,54635:down|Dpysl3,22240:down|Mgp,17313:up|Serpinh1,12406:down|Shroom4,208431:down|Vegfa,22339:down|Cbr2,12409:up|Tnf,21926:up|Loxl2,94352:down|Plk2,20620:down|Trf,22041:up|Col1a2,12843:down|Cd24a,12484:down|Col11a1,12814:down|Ncam1,17967:down|Hmox1,15368:up|C1qtnf3,81799:down|Tgfbr3,21814:up|C1qtnf6,72709:down|Grem1,23892:up|Adipoq,11450:up|Col3a1,12825:down|Sfrp2,20319:up|Col5a1,12831:down|Tgfb2,21808:down
regulation of peptide hormone secretion	GO:0090276	2.47E-01	3.49E-01	Tnf,21926:up|Pparg,19016:up|Glp1r,14652:down|Egfr,13649:down|Sfrp1,20377:up
cell junction assembly	GO:0034329	2.48E-01	3.50E-01	Tnf,21926:up|Gpm6b,14758:up|Vegfa,22339:down|Grem1,23892:up
protein localization to organelle	GO:0033365	2.48E-01	3.51E-01	Kdelr3,105785:down|Tnf,21926:up|Gas6,14456:up|Akr1c18,105349:up|Egfr,13649:down|C1qtnf3,81799:down|Bmpr1a,12166:down|Ptgs2,19225:down|Grem1,23892:up|Igf1,16000:up|Bmp4,12159:up|Ceacam1,26365:up|Sfrp2,20319:up|Tgfb2,21808:down|Htr2a,15558:down
regulation of ATP metabolic process	GO:1903578	2.49E-01	3.51E-01	Htr2a,15558:down|Igf1,16000:up
body morphogenesis	GO:0010171	2.49E-01	3.51E-01	Tgfb2,21808:down|Mmp2,17390:down
negative regulation of peptide secretion	GO:0002792	2.49E-01	3.51E-01	Pparg,19016:up|Sfrp1,20377:up
regulation of insulin receptor signaling pathway	GO:0046626	2.49E-01	3.51E-01	Enpp1,18605:down|Igf2,16002:up
regulation of nucleoside metabolic process	GO:0009118	2.49E-01	3.51E-01	Htr2a,15558:down|Igf1,16000:up
regulation of ERBB signaling pathway	GO:1901184	2.49E-01	3.51E-01	Ceacam1,26365:up|Errfi1,74155:down
anatomical structure homeostasis	GO:0060249	2.50E-01	3.52E-01	Ceacam1,26365:up|Trf,22041:up|Egfr,13649:down|Vegfa,22339:down|Tfrc,22042:up|Tnfrsf11b,18383:up|Epas1,13819:up
endocrine process	GO:0050886	2.51E-01	3.53E-01	Fgfr1,14182:down|C1qtnf3,81799:down|Inhba,16323:down
intrinsic apoptotic signaling pathway in response to DNA damage	GO:0008630	2.51E-01	3.53E-01	Tnf,21926:up|Cxcl12,20315:up|Hmox1,15368:up
cellular response to insulin stimulus	GO:0032869	2.52E-01	3.53E-01	Pparg,19016:up|Enpp1,18605:down|Igf2,16002:up|Adipoq,11450:up
peptide secretion	GO:0002790	2.53E-01	3.53E-01	Tnf,21926:up|Pparg,19016:up|Glp1r,14652:down|Egfr,13649:down|Sfrp1,20377:up|Il1rn,16181:up
positive regulation of signal transduction by p53 class mediator	GO:1901798	2.53E-01	3.53E-01	Ankrd1,107765:down
regulation of mononuclear cell migration	GO:0071675	2.53E-01	3.53E-01	Ccl2,20296:up
positive regulation of cellular response to oxidative stress	GO:1900409	2.53E-01	3.53E-01	Tnf,21926:up
negative regulation of chemokine production	GO:0032682	2.53E-01	3.53E-01	C1qtnf3,81799:down
positive regulation of glycoprotein biosynthetic process	GO:0010560	2.53E-01	3.53E-01	Igf1,16000:up
detection of temperature stimulus	GO:0016048	2.53E-01	3.53E-01	Htr2a,15558:down
positive regulation of extrinsic apoptotic signaling pathway via death domain receptors	GO:1902043	2.53E-01	3.53E-01	Sfrp1,20377:up
atrial septum morphogenesis	GO:0060413	2.53E-01	3.53E-01	Tgfb2,21808:down
hematopoietic stem cell differentiation	GO:0060218	2.53E-01	3.53E-01	Sfrp1,20377:up
basement membrane organization	GO:0071711	2.53E-01	3.53E-01	Flrt2,399558:down
axonal fasciculation	GO:0007413	2.53E-01	3.53E-01	Wnt5a,22418:down
post-embryonic organ development	GO:0048569	2.53E-01	3.53E-01	Vegfa,22339:down
regulation of interleukin-8 secretion	GO:2000482	2.53E-01	3.53E-01	Wnt5a,22418:down
negative regulation of neurological system process	GO:0031645	2.53E-01	3.53E-01	Tnf,21926:up
positive regulation of protein export from nucleus	GO:0046827	2.53E-01	3.53E-01	Gas6,14456:up

negative regulation of interleukin-2 production	GO:0032703	2.53E-01	3.53E-01	Ceacam1,26365:up
regulation of hepatocyte proliferation	GO:2000345	2.53E-01	3.53E-01	Ceacam1,26365:up
cellular response to catecholamine stimulus	GO:0071870	2.53E-01	3.53E-01	Adipoq,11450:up
cell migration involved in gastrulation	GO:0042074	2.53E-01	3.53E-01	Wnt5a,22418:down
trophoblast giant cell differentiation	GO:0060707	2.53E-01	3.53E-01	Gjb5,14622:down
ectoderm development	GO:0007398	2.53E-01	3.53E-01	Bmpr1a,12166:down
response to muscle stretch	GO:0035994	2.53E-01	3.53E-01	Ankrd1,107765:down
negative regulation of cardiac muscle hypertrophy	GO:0010614	2.53E-01	3.53E-01	Errfi1,74155:down
behavioral response to cocaine	GO:0048148	2.53E-01	3.53E-01	Htr2a,15558:down
phosphatidylcholine biosynthetic process	GO:0006656	2.53E-01	3.53E-01	Fabp5,16592:up
regulation of interleukin-5 production	GO:0032674	2.53E-01	3.53E-01	Il1rl1,17082:down
DNA catabolic process, endonucleolytic	GO:0000737	2.53E-01	3.53E-01	Igfbp3,16009:up
interleukin-1-mediated signaling pathway	GO:0070498	2.53E-01	3.53E-01	Il1rn,16181:up
interleukin-5 production	GO:0032634	2.53E-01	3.53E-01	Il1rl1,17082:down
cell proliferation involved in heart morphogenesis	GO:0061323	2.53E-01	3.53E-01	Bmp4,12159:up
wound healing, spreading of epidermal cells	GO:0035313	2.53E-01	3.53E-01	Col5a1,12831:down
response to arsenic-containing substance	GO:0046685	2.53E-01	3.53E-01	Hmox1,15368:up
positive regulation of digestive system process	GO:0060456	2.53E-01	3.53E-01	Mmp13,17386:up
hemoglobin metabolic process	GO:0020027	2.53E-01	3.53E-01	Inhba,16323:down
negative regulation of protein tyrosine kinase activity	GO:0061099	2.53E-01	3.53E-01	Errfi1,74155:down
T-helper 17 type immune response	GO:0072538	2.53E-01	3.53E-01	Ly9,17085:up
regulation of podosome assembly	GO:0071801	2.53E-01	3.53E-01	Tnf,21926:up
pyrimidine nucleobase metabolic process	GO:0006206	2.53E-01	3.53E-01	Uck2,80914:down
negative regulation of phosphoprotein phosphatase activity	GO:0032515	2.53E-01	3.53E-01	Tnf,21926:up
gonadotropin secretion	GO:0032274	2.53E-01	3.53E-01	Inhba,16323:down
regulation of cellular catabolic process	GO:0031329	2.54E-01	3.54E-01	Htr2b,15559:down|Tnf,21926:up|Plk2,20620:down|Lmcd1,30937:up|Dram1,71712:up|Hp,15439:up|Hmox1,15368:up|Sfrp4,20379:up|Igf1,16000:up|Uchl5,56207:up|Plod2,26432:down|Htr2a,15558:down|Igfbp3,16009:up
regulation of RNA biosynthetic process	GO:2001141	2.54E-01	3.54E-01	Fstl3,83554:down|Ice1,218333:down|Tfec,21426:up|Sfrp4,20379:up|Ednrb,13618:up|Epas1,13819:up|Ankrd1,107765:down|Htra3,78558:down|Uchl5,56207:up|Lyl1,17095:up|Sdpr,20324:down|Loxl2,94352:down|Loxl3,16950:down|Trf,22041:up|Wnt5a,22418:down|Bmpr1a,12166:down|Ccl4,20303:up|Adipoq,11450:up|Bmp4,12159:up|Igf1,16000:up|Ret,19713:up|Plpp3,67916:up|Creb3l1,26427:down|Fgfr2,14183:up|Prl2c2,18811:up|Efemp1,216616:up|Inhba,16323:down|Rasl11a,68895:up|Bnc2,242509:down|Ccl3,20302:up|Vdr,22337:down|Fabp4,11770:up|Vegfa,22339:down|Igf2,16002:up|Slc40a1,53945:up|Pparg,19016:up|Gas6,14456:up|Tnf,21926:up|Glp1r,14652:down|Lmcd1,30937:up|Fgfr1,14182:down|Egfr,13649:down|Hmox1,15368:up|Sfrp1,20377:up|Sulf1,240725:down|Tgfbr3,21814:up|Grem1,23892:up|Phf11d,219132:up|Sfrp2,20319:up|Tbx15,21384:down|Npnt,114249:up|S100a1,20193:up

organic substance biosynthetic process	GO:1901576	2.55E-01	3.55E-01	Fstl3,83554:down|Adm,11535:up|Tfec,21426:up|Sfrp4,20379:up|C3,12266:up|Ankrd1,107765:down|Serpinh1,12406:down|Lyl1,17095:up|Ggt5,23887:up|Loxl2,94352:down|Loxl3,16950:down|Trf,22041:up|Wnt5a,22418:down|Adipoq,11450:up|Bmp4,12159:up|Igf1,16000:up|Tgfb2,21808:down|Ptgir,19222:up|Fgfr2,14183:up|Htr2b,15559:down|Prl2c2,18811:up|Efemp1,216616:up|Vdr,22337:down|Fabp4,11770:up|Htr2a,15558:down|Slc40a1,53945:up|Gas6,14456:up|Egfr,13649:down|Sfrp1,20377:up|C1qtnf3,81799:down|Tgfbr3,21814:up|Ceacam1,26365:up|Sfrp2,20319:up|Npnt,114249:up|S100a1,20193:up|Ice1,218333:down|Aldh1a2,19378:up|Ccl2,20296:up|Pdgfc,54635:down|Ednrb,13618:up|Ampd3,11717:up|Epas1,13819:up|Htra3,78558:down|Sord,20322:up|Uchl5,56207:up|Sdpr,20324:down|Cdo1,12583:up|Man2a2,140481:up|Akr1c18,105349:up|Fabp5,16592:up|Bmpr1a,12166:down|Ccl4,20303:up|Chst11,58250:down|Gatm,67092:down|Ret,19713:up|Prg4,96875:down|Vegfb,22340:up|Sdf2l1,64136:up|Pdxk,216134:up|Aldh1a3,56847:down|Plpp3,67916:up|Creb3l1,26427:down|Kitl,17311:up|Rasl11a,68895:up|Inhba,16323:down|Bnc2,242509:down|Ccl3,20302:up|Vegfa,22339:down|Gfpt2,14584:down|Igf2,16002:up|Pparg,19016:up|Tnf,21926:up|Lmcd1,30937:up|Glp1r,14652:down|Fgfr1,14182:down|Hmox1,15368:up|Lpl,16956:up|Has2,15117:down|Sulf1,240725:down|Ptgs2,19225:down|Grem1,23892:up|Phf11d,219132:up|Tbx15,21384:down|Col5a1,12831:down|Uck2,80914:down|Errfi1,74155:down|Enpp1,18605:down
execution phase of apoptosis	GO:0097194	2.55E-01	3.55E-01	Cd24a,12484:down|Igfbp3,16009:up
embryonic pattern specification	GO:0009880	2.55E-01	3.55E-01	Wnt5a,22418:down|Fgfr2,14183:up
positive regulation of lipid transport	GO:0032370	2.55E-01	3.55E-01	Fam132b,227358:down|Adipoq,11450:up
response to ethanol	GO:0045471	2.55E-01	3.55E-01	Ccl7,20306:up|Cdo1,12583:up
response to carbohydrate	GO:0009743	2.56E-01	3.55E-01	Gas6,14456:up|Glp1r,14652:down|Igf1,16000:up|Adipoq,11450:up
maintenance of cell number	GO:0098727	2.56E-01	3.55E-01	Fgfr1,14182:down|Sfrp1,20377:up|Bmpr1a,12166:down|Igf1,16000:up
nucleobase-containing compound catabolic process	GO:0034655	2.56E-01	3.55E-01	Htr2a,15558:down|Igfbp3,16009:up|Enpp1,18605:down|Enpp3,209558:down|Enpp5,83965:up|Igf1,16000:up
response to hydrogen peroxide	GO:0042542	2.56E-01	3.56E-01	Hp,15439:up|Hmox1,15368:up|Mmp2,17390:down
positive regulation of cation transmembrane transport	GO:1904064	2.56E-01	3.56E-01	Fxyd1,56188:up|S100a1,20193:up|Bmp4,12159:up
B cell differentiation	GO:0030183	2.56E-01	3.56E-01	Cd24a,12484:down|Lyl1,17095:up|Sfrp1,20377:up
carbohydrate catabolic process	GO:0016052	2.56E-01	3.56E-01	Sord,20322:up|Htr2a,15558:down|Igf1,16000:up
detection of stimulus	GO:0051606	2.57E-01	3.57E-01	Tnf,21926:up|Col11a1,12814:down|Htr2a,15558:down|Serpine2,20720:up|Igf1,16000:up
cellular response to peptide	GO:1901653	2.57E-01	3.57E-01	Pparg,19016:up|Enpp1,18605:down|Igf2,16002:up|Igf1,16000:up|Adipoq,11450:up
carbohydrate derivative biosynthetic process	GO:1901137	2.58E-01	3.58E-01	Htr2b,15559:down|Adm,11535:up|Has2,15117:down|Ampd3,11717:up|Igf1,16000:up|Chst11,58250:down|Htr2a,15558:down|Vegfb,22340:up|Sdf2l1,64136:up|Gfpt2,14584:down|Ptgir,19222:up|Uck2,80914:down|Man2a2,140481:up
regulation of autophagy	GO:0010506	2.58E-01	3.58E-01	Htr2b,15559:down|Plk2,20620:down|Lmcd1,30937:up|Dram1,71712:up|Plod2,26432:down|Hmox1,15368:up|Sfrp4,20379:up
G-protein coupled receptor signaling pathway, coupled to cyclic nucleotide second messenger	GO:0007187	2.60E-01	3.60E-01	Htr2b,15559:down|Glp1r,14652:down|Htr2a,15558:down|Ptgir,19222:up
cell cycle G1/S phase transition	GO:0044843	2.60E-01	3.60E-01	Plk2,20620:down|Egfr,13649:down|Inhba,16323:down|Susd2,71733:up
negative regulation of DNA metabolic process	GO:0051053	2.61E-01	3.62E-01	Ankrd1,107765:down|Pparg,19016:up|Adipoq,11450:up
regulation of smooth muscle contraction	GO:0006940	2.62E-01	3.63E-01	Ptgs2,19225:down|Npnt,114249:up
purine nucleotide metabolic process	GO:0006163	2.63E-01	3.64E-01	Htr2b,15559:down|Adm,11535:up|Wnt5a,22418:down|Ampd3,11717:up|Igf1,16000:up|Htr2a,15558:down|Vegfa,22339:down|Ptgir,19222:up|Enpp1,18605:down
negative regulation of cell projection organization	GO:0031345	2.63E-01	3.65E-01	Plk2,20620:down|Efemp1,216616:up|Wnt5a,22418:down|Dpysl3,22240:down
regulation of peptide secretion	GO:0002791	2.64E-01	3.65E-01	Tnf,21926:up|Pparg,19016:up|Glp1r,14652:down|Egfr,13649:down|Sfrp1,20377:up
glucose homeostasis	GO:0042593	2.64E-01	3.65E-01	Pparg,19016:up|Gas6,14456:up|C1qtnf3,81799:down|Igf1,16000:up|Adipoq,11450:up
cellular ketone metabolic process	GO:0042180	2.64E-01	3.65E-01	Pparg,19016:up|Akr1c18,105349:up|Fam132b,227358:down|C1qtnf3,81799:down|Adipoq,11450:up
carbohydrate homeostasis	GO:0033500	2.64E-01	3.65E-01	Pparg,19016:up|Gas6,14456:up|C1qtnf3,81799:down|Igf1,16000:up|Adipoq,11450:up
amelogenesis	GO:0097186	2.67E-01	3.65E-01	Fam20a,208659:down
response to fluid shear stress	GO:0034405	2.67E-01	3.65E-01	Ptgs2,19225:down
negative regulation of B cell proliferation	GO:0030889	2.67E-01	3.65E-01	Cd24a,12484:down
response to catecholamine	GO:0071869	2.67E-01	3.65E-01	Adipoq,11450:up

negative regulation of membrane potential	GO:0045837	2.67E-01	3.65E-01	Il1rn,16181:up
regulation of transcription from RNA polymerase I promoter	GO:0006356	2.67E-01	3.65E-01	Rasl11a,68895:up
negative regulation of release of cytochrome c from mitochondria	GO:0090201	2.67E-01	3.65E-01	Igf1,16000:up
positive regulation of response to oxidative stress	GO:1902884	2.67E-01	3.65E-01	Tnf,21926:up
modulation of transcription in other organism involved in symbiotic interaction	GO:0052312	2.67E-01	3.65E-01	Ccl4,20303:up
modulation by host of viral transcription	GO:0043921	2.67E-01	3.65E-01	Ccl4,20303:up
melanin metabolic process	GO:0006582	2.67E-01	3.65E-01	Wnt5a,22418:down
regulation of natural killer cell differentiation	GO:0032823	2.67E-01	3.65E-01	Gas6,14456:up
regulation of tyrosine phosphorylation of Stat5 protein	GO:0042522	2.67E-01	3.65E-01	Igf1,16000:up
positive regulation of protein processing	GO:0010954	2.67E-01	3.65E-01	C3,12266:up
arginine metabolic process	GO:0006525	2.67E-01	3.65E-01	Ddah1,69219:down
integrin activation	GO:0033622	2.67E-01	3.65E-01	Cd24a,12484:down
response to misfolded protein	GO:0051788	2.67E-01	3.65E-01	Sdf2l1,64136:up
retinal ganglion cell axon guidance	GO:0031290	2.67E-01	3.65E-01	Vegfa,22339:down
dendritic cell migration	GO:0036336	2.67E-01	3.65E-01	Gas6,14456:up
positive regulation of cholesterol transport	GO:0032376	2.67E-01	3.65E-01	Adipoq,11450:up
regulation of synaptic transmission, dopaminergic	GO:0032225	2.67E-01	3.65E-01	Ptgs2,19225:down
regulation of transcription from RNA polymerase III promoter	GO:0006359	2.67E-01	3.65E-01	Ice1,218333:down
negative regulation of smooth muscle contraction	GO:0045986	2.67E-01	3.65E-01	Ptgs2,19225:down
regulation of receptor binding	GO:1900120	2.67E-01	3.65E-01	Adipoq,11450:up
positive regulation of fatty acid oxidation	GO:0046321	2.67E-01	3.65E-01	Pparg,19016:up
positive regulation of sterol transport	GO:0032373	2.67E-01	3.65E-01	Adipoq,11450:up
negative regulation of myeloid cell apoptotic process	GO:0033033	2.67E-01	3.65E-01	Kitl,17311:up
regulation of smooth muscle cell apoptotic process	GO:0034391	2.67E-01	3.65E-01	Igf1,16000:up
regulation of membrane protein ectodomain proteolysis	GO:0051043	2.67E-01	3.65E-01	Tnf,21926:up
modulation by host of symbiont transcription	GO:0052472	2.67E-01	3.65E-01	Ccl4,20303:up
response to prostaglandin E	GO:0034695	2.67E-01	3.65E-01	Tgfbr3,21814:up
negative regulation of endoplasmic reticulum stress-induced intrinsic apoptotic signaling pathway	GO:1902236	2.67E-01	3.65E-01	Creb3l1,26427:down
smooth muscle cell apoptotic process	GO:0034390	2.67E-01	3.65E-01	Igf1,16000:up
regulation of protein kinase A signaling	GO:0010738	2.67E-01	3.65E-01	Adipoq,11450:up
positive regulation of proteolysis	GO:0045862	2.68E-01	3.67E-01	Tnf,21926:up|Pparg,19016:up|Plk2,20620:down|Sfrp2,20319:up|C3,12266:up|F3,14066:up
heart looping	GO:0001947	2.69E-01	3.67E-01	Wnt5a,22418:down|Aldh1a2,19378:up

regulation of calcium ion transmembrane transporter activity	GO:1901019	2.69E-01	3.67E-01	S100a1,20193:up|Bmp4,12159:up
cellular localization	GO:0051641	2.74E-01	3.74E-01	Ice1,218333:down|Adm,11535:up|Ccl2,20296:up|Mmp13,17386:up|Ankrd1,107765:down|Gpm6b,14758:up|Clec4n,56620:up|Postn,50706:down|Akr1c18,105349:up|Cd24a,12484:down|Ncam1,17967:down|Wnt5a,22418:down|Bmpr1a,12166:down|Il1rn,16181:up|Maob,109731:up|Adipoq,11450:up|Bmp4,12159:up|Igf1,16000:up|Tgfb2,21808:down|Creb3l1,26427:down|Fgfr2,14183:up|Il1rl1,17082:down|Clec4e,56619:up|Htr2b,15559:down|Inhba,16323:down|Ccl3,20302:up|Cxcl12,20315:up|Htr2a,15558:down|Serpine2,20720:up|Lrrc15,74488:down|Kdelr3,105785:down|Pparg,19016:up|Gas6,14456:up|Tnf,21926:up|Glp1r,14652:down|Egfr,13649:down|Fgfr1,14182:down|Sec23a,20334:down|Lpl,16956:up|Hmox1,15368:up|Sfrp1,20377:up|C1qtnf3,81799:down|Cd4,12504:up|Ptgs2,19225:down|Sncg,20618:up|Grem1,23892:up|Ceacam1,26365:up|Sfrp2,20319:up|Syt13,80976:up
leukocyte activation involved in immune response	GO:0002366	2.74E-01	3.75E-01	Ly9,17085:up|Hmox1,15368:up|Cx3cr1,13051:down|Tfrc,22042:up|Clec4e,56619:up
regulation of B cell proliferation	GO:0030888	2.76E-01	3.76E-01	Cd24a,12484:down|Tfrc,22042:up
cell activation involved in immune response	GO:0002263	2.78E-01	3.79E-01	Ly9,17085:up|Hmox1,15368:up|Cx3cr1,13051:down|Tfrc,22042:up|Clec4e,56619:up
establishment or maintenance of cell polarity	GO:0007163	2.80E-01	3.79E-01	Ptk7,71461:down|Wnt5a,22418:down|Mark1,226778:up|Igf1,16000:up
removal of superoxide radicals	GO:0019430	2.80E-01	3.79E-01	Tnf,21926:up
positive regulation of protein maturation	GO:1903319	2.80E-01	3.79E-01	C3,12266:up
regulation of interleukin-6 biosynthetic process	GO:0045408	2.80E-01	3.79E-01	Prg4,96875:down
steroid catabolic process	GO:0006706	2.80E-01	3.79E-01	Akr1c18,105349:up
regulation of macrophage differentiation	GO:0045649	2.80E-01	3.79E-01	Adipoq,11450:up
cellular response to dexamethasone stimulus	GO:0071549	2.80E-01	3.79E-01	Bmp4,12159:up
low-density lipoprotein particle clearance	GO:0034383	2.80E-01	3.79E-01	Adipoq,11450:up
lymphangiogenesis	GO:0001946	2.80E-01	3.79E-01	Vegfa,22339:down
regulation of collateral sprouting	GO:0048670	2.80E-01	3.79E-01	Crabp2,12904:down
cell proliferation in external granule layer	GO:0021924	2.80E-01	3.79E-01	Igf1,16000:up
negative regulation of NF-kappaB import into nucleus	GO:0042347	2.80E-01	3.79E-01	C1qtnf3,81799:down
regulation of B cell apoptotic process	GO:0002902	2.80E-01	3.79E-01	Cd24a,12484:down
fibrinolysis	GO:0042730	2.80E-01	3.79E-01	Serping1,12258:up
transepithelial transport	GO:0070633	2.80E-01	3.79E-01	Ednrb,13618:up
pyridine-containing compound biosynthetic process	GO:0072525	2.80E-01	3.79E-01	Pdxk,216134:up
peroxisome proliferator activated receptor signaling pathway	GO:0035357	2.80E-01	3.79E-01	Pparg,19016:up
negative regulation of viral entry into host cell	GO:0046597	2.80E-01	3.79E-01	Ptx3,19288:up
sensory perception of temperature stimulus	GO:0050951	2.80E-01	3.79E-01	Htr2a,15558:down
regulation of tumor necrosis factor biosynthetic process	GO:0042534	2.80E-01	3.79E-01	Errfi1,74155:down
tumor necrosis factor biosynthetic process	GO:0042533	2.80E-01	3.79E-01	Errfi1,74155:down
response to amine	GO:0014075	2.80E-01	3.79E-01	Htr2b,15559:down
neural nucleus development	GO:0048857	2.80E-01	3.79E-01	Aldh1a3,56847:down
positive regulation of glycoprotein metabolic process	GO:1903020	2.80E-01	3.79E-01	Igf1,16000:up
alditol metabolic process	GO:0019400	2.80E-01	3.79E-01	Sord,20322:up
regulation of urine volume	GO:0035809	2.80E-01	3.79E-01	Ednrb,13618:up

negative regulation of protein localization to plasma membrane	GO:1903077	2.80E-01	3.79E-01	Lrrc15,74488:down
positive regulation of translational initiation	GO:0045948	2.80E-01	3.79E-01	Tnf,21926:up
mononuclear cell migration	GO:0071674	2.80E-01	3.79E-01	Ccl2,20296:up
alditol phosphate metabolic process	GO:0052646	2.80E-01	3.79E-01	Serinc2,230779:down
toll-like receptor 3 signaling pathway	GO:0034138	2.80E-01	3.79E-01	Tnf,21926:up
lymph vessel morphogenesis	GO:0036303	2.80E-01	3.79E-01	Vegfa,22339:down
regulation of cAMP-mediated signaling	GO:0043949	2.80E-01	3.79E-01	Ptgir,19222:up
negative regulation of muscle hypertrophy	GO:0014741	2.80E-01	3.79E-01	Errfi1,74155:down
negative regulation of viral transcription	GO:0032897	2.80E-01	3.79E-01	Ccl4,20303:up
CD8-positive, alpha-beta T cell activation	GO:0036037	2.80E-01	3.79E-01	Ceacam1,26365:up
positive regulation of chondrocyte differentiation	GO:0032332	2.80E-01	3.79E-01	Loxl2,94352:down
pyrimidine ribonucleoside metabolic process	GO:0046131	2.80E-01	3.79E-01	Uck2,80914:down
cerebellar granule cell precursor proliferation	GO:0021930	2.80E-01	3.79E-01	Igf1,16000:up
carbohydrate derivative catabolic process	GO:1901136	2.81E-01	3.79E-01	Hpse,15442:up|Npl,74091:up|Adamts12,239337:down
establishment of cell polarity	GO:0030010	2.81E-01	3.79E-01	Ptk7,71461:down|Wnt5a,22418:down|Mark1,226778:up
regulation of tyrosine phosphorylation of STAT protein	GO:0042509	2.82E-01	3.81E-01	Crlf1,12931:down|Igf1,16000:up
glycolytic process	GO:0006096	2.82E-01	3.81E-01	Htr2a,15558:down|Igf1,16000:up
ADP phosphorylation	GO:0006757	2.82E-01	3.81E-01	Htr2a,15558:down|Igf1,16000:up
regulation of intracellular steroid hormone receptor signaling pathway	GO:0033143	2.82E-01	3.81E-01	Sfrp1,20377:up|Igf1,16000:up
positive regulation of cysteine-type endopeptidase activity	GO:2001056	2.86E-01	3.85E-01	Tnf,21926:up|Pparg,19016:up|F3,14066:up
monovalent inorganic cation transport	GO:0015672	2.86E-01	3.86E-01	Hvcn1,74096:up|Fxyd1,56188:up|Kcnj15,16516:down|Sncg,20618:up|Fxyd2,11936:up|Maob,109731:up|Gpm6b,14758:up|Cxcl12,20315:up|Htr2a,15558:down|Serpine2,20720:up
multicellular organism reproduction	GO:0032504	2.88E-01	3.89E-01	Egfr,13649:down|Sfrp1,20377:up|Inhba,16323:down|Ptgs2,19225:down|Ednrb,13618:up|Plat,18791:down|Igf1,16000:up|Bmp4,12159:up|Vdr,22337:down|Cxcl12,20315:up|Vegfa,22339:down|Mmp2,17390:down|Serpine2,20720:up|Kitl,17311:up
glutamine family amino acid metabolic process	GO:0009064	2.89E-01	3.90E-01	Ddah1,69219:down|Gfpt2,14584:down
gluconeogenesis	GO:0006094	2.89E-01	3.90E-01	C1qtnf3,81799:down|Adipoq,11450:up
negative regulation of nucleocytoplasmic transport	GO:0046823	2.89E-01	3.90E-01	Gas6,14456:up|C1qtnf3,81799:down
regulation of phosphatase activity	GO:0010921	2.90E-01	3.91E-01	Tnf,21926:up|Tgfb2,21808:down|Npnt,114249:up
cardiac muscle hypertrophy in response to stress	GO:0014898	2.93E-01	3.91E-01	Errfi1,74155:down
positive regulation of protein targeting to membrane	GO:0090314	2.93E-01	3.91E-01	Ccl2,20296:up
negative regulation of epidermal growth factor receptor signaling pathway	GO:0042059	2.93E-01	3.91E-01	Errfi1,74155:down
cellular response to superoxide	GO:0071451	2.93E-01	3.91E-01	Tnf,21926:up
negative regulation of cell junction assembly	GO:1901889	2.93E-01	3.91E-01	Tnf,21926:up
cell proliferation in hindbrain	GO:0021534	2.93E-01	3.91E-01	Igf1,16000:up
muscle hypertrophy in response to stress	GO:0003299	2.93E-01	3.91E-01	Errfi1,74155:down

negative regulation of growth of symbiont involved in interaction with host	GO:0044146	2.93E-01	3.91E-01	Tnf,21926:up
positive regulation of meiotic cell cycle	GO:0051446	2.93E-01	3.91E-01	Wnt5a,22418:down
regulation of regulatory T cell differentiation	GO:0045589	2.93E-01	3.91E-01	Ctla2a,13024:down
auditory receptor cell development	GO:0060117	2.93E-01	3.91E-01	Fgfr1,14182:down
positive regulation of catecholamine secretion	GO:0033605	2.93E-01	3.91E-01	Cxcl12,20315:up
type I interferon signaling pathway	GO:0060337	2.93E-01	3.91E-01	Wnt5a,22418:down
lymphoid progenitor cell differentiation	GO:0002320	2.93E-01	3.91E-01	Bmp4,12159:up
purine nucleoside bisphosphate metabolic process	GO:0034032	2.93E-01	3.91E-01	Enpp1,18605:down
serine phosphorylation of STAT protein	GO:0042501	2.93E-01	3.91E-01	Ret,19713:up
ribonucleoside bisphosphate metabolic process	GO:0033875	2.93E-01	3.91E-01	Enpp1,18605:down
cellular response to pH	GO:0071467	2.93E-01	3.91E-01	Hvcn1,74096:up
L-glutamate transport	GO:0015813	2.93E-01	3.91E-01	Tnf,21926:up
positive regulation of isotype switching	GO:0045830	2.93E-01	3.91E-01	Tfrc,22042:up
regulation of epidermal growth factor-activated receptor activity	GO:0007176	2.93E-01	3.91E-01	Errfi1,74155:down
positive regulation of oligodendrocyte differentiation	GO:0048714	2.93E-01	3.91E-01	Pparg,19016:up
regulation of endoplasmic reticulum unfolded protein response	GO:1900101	2.93E-01	3.91E-01	Sdf2l1,64136:up
nitric-oxide synthase biosynthetic process	GO:0051767	2.93E-01	3.91E-01	Ccl2,20296:up
positive regulation of focal adhesion assembly	GO:0051894	2.93E-01	3.91E-01	Vegfa,22339:down
positive regulation of fatty acid transport	GO:2000193	2.93E-01	3.91E-01	Fam132b,227358:down
nucleoside bisphosphate metabolic process	GO:0033865	2.93E-01	3.91E-01	Enpp1,18605:down
myeloid dendritic cell differentiation	GO:0043011	2.93E-01	3.91E-01	Tnfsf9,21950:up
regulation of nitric-oxide synthase biosynthetic process	GO:0051769	2.93E-01	3.91E-01	Ccl2,20296:up
negative regulation of growth of symbiont in host	GO:0044130	2.93E-01	3.91E-01	Tnf,21926:up
retina vasculature development in camera-type eye	GO:0061298	2.93E-01	3.91E-01	Vegfa,22339:down
regulation of cholesterol efflux	GO:0010874	2.93E-01	3.91E-01	Adipoq,11450:up
positive regulation of sodium ion transmembrane transport	GO:1902307	2.93E-01	3.91E-01	Fxyd1,56188:up
cellular response to oxygen radical	GO:0071450	2.93E-01	3.91E-01	Tnf,21926:up
multi-organism membrane organization	GO:0044803	2.93E-01	3.91E-01	Gas6,14456:up
interleukin-6 biosynthetic process	GO:0042226	2.93E-01	3.91E-01	Prg4,96875:down
response to dexamethasone	GO:0071548	2.93E-01	3.91E-01	Bmp4,12159:up

regulation of RNA metabolic process	GO:0051252	2.95E-01	3.94E-01	Fstl3,83554:down|Ice1,218333:down|Tfec,21426:up|Sfrp4,20379:up|Ednrb,13618:up|Epas1,13819:up|Ankrd1,107765:down|Htra3,78558:down|Uchl5,56207:up|Lyl1,17095:up|Sdpr,20324:down|Loxl2,94352:down|Loxl3,16950:down|Trf,22041:up|Wnt5a,22418:down|Bmpr1a,12166:down|Ccl4,20303:up|Adipoq,11450:up|Bmp4,12159:up|Igf1,16000:up|Ret,19713:up|Plpp3,67916:up|Creb3l1,26427:down|Fgfr2,14183:up|Prl2c2,18811:up|Efemp1,216616:up|Inhba,16323:down|Rasl11a,68895:up|Bnc2,242509:down|Ccl3,20302:up|Vdr,22337:down|Fabp4,11770:up|Vegfa,22339:down|Oas3,246727:up|Igf2,16002:up|Slc40a1,53945:up|Pparg,19016:up|Gas6,14456:up|Tnf,21926:up|Glp1r,14652:down|Lmcd1,30937:up|Fgfr1,14182:down|Egfr,13649:down|Hmox1,15368:up|Sfrp1,20377:up|Sulf1,240725:down|Tgfbr3,21814:up|Grem1,23892:up|Phf11d,219132:up|Sfrp2,20319:up|Tbx15,21384:down|Npnt,114249:up|S100a1,20193:up
regulation of cation transmembrane transport	GO:1904062	2.95E-01	3.94E-01	Fxyd1,56188:up|Tgfb2,21808:down|Fxyd2,11936:up|S100a1,20193:up|Bmp4,12159:up
regulation of B cell activation	GO:0050864	2.95E-01	3.94E-01	Cd24a,12484:down|Sfrp1,20377:up|Tfrc,22042:up
positive regulation of apoptotic signaling pathway	GO:2001235	2.96E-01	3.94E-01	Tnfrsf12a,27279:down|Sfrp1,20377:up|Tgfb2,21808:down|Inhba,16323:down
actin-mediated cell contraction	GO:0070252	2.96E-01	3.94E-01	Fxyd1,56188:up|Stc1,20855:up
positive regulation of cytokine biosynthetic process	GO:0042108	2.96E-01	3.94E-01	Tnf,21926:up|Wnt5a,22418:down
endoplasmic reticulum unfolded protein response	GO:0030968	2.96E-01	3.94E-01	Sdf2l1,64136:up|Creb3l1,26427:down
activation of immune response	GO:0002253	2.96E-01	3.94E-01	Tnf,21926:up|C1s1,50908:up|Serping1,12258:up|C1s2,317677:up|C3,12266:up|C1rb,667277:up
regulation of transcription, DNA-templated	GO:0006355	2.98E-01	3.97E-01	Fstl3,83554:down|Ice1,218333:down|Tfec,21426:up|Sfrp4,20379:up|Ednrb,13618:up|Epas1,13819:up|Ankrd1,107765:down|Htra3,78558:down|Uchl5,56207:up|Lyl1,17095:up|Sdpr,20324:down|Loxl2,94352:down|Loxl3,16950:down|Trf,22041:up|Wnt5a,22418:down|Bmpr1a,12166:down|Adipoq,11450:up|Bmp4,12159:up|Igf1,16000:up|Ret,19713:up|Plpp3,67916:up|Creb3l1,26427:down|Fgfr2,14183:up|Prl2c2,18811:up|Efemp1,216616:up|Inhba,16323:down|Rasl11a,68895:up|Bnc2,242509:down|Ccl3,20302:up|Vdr,22337:down|Fabp4,11770:up|Vegfa,22339:down|Igf2,16002:up|Slc40a1,53945:up|Pparg,19016:up|Gas6,14456:up|Tnf,21926:up|Glp1r,14652:down|Lmcd1,30937:up|Fgfr1,14182:down|Egfr,13649:down|Hmox1,15368:up|Sfrp1,20377:up|Sulf1,240725:down|Tgfbr3,21814:up|Grem1,23892:up|Sfrp2,20319:up|Phf11d,219132:up|Tbx15,21384:down|Npnt,114249:up|S100a1,20193:up
purine ribonucleotide biosynthetic process	GO:0009152	2.99E-01	3.98E-01	Htr2b,15559:down|Adm,11535:up|Htr2a,15558:down|Ptgir,19222:up|Ampd3,11717:up|Igf1,16000:up
glycerolipid metabolic process	GO:0046486	2.99E-01	3.98E-01	Htr2b,15559:down|Fabp5,16592:up|Lpl,16956:up|Htr2a,15558:down|C3,12266:up|Serinc2,230779:down
regulation of nucleic acid-templated transcription	GO:1903506	3.01E-01	4.00E-01	Fstl3,83554:down|Ice1,218333:down|Tfec,21426:up|Sfrp4,20379:up|Ednrb,13618:up|Epas1,13819:up|Ankrd1,107765:down|Htra3,78558:down|Uchl5,56207:up|Lyl1,17095:up|Sdpr,20324:down|Loxl2,94352:down|Loxl3,16950:down|Trf,22041:up|Wnt5a,22418:down|Bmpr1a,12166:down|Adipoq,11450:up|Bmp4,12159:up|Igf1,16000:up|Ret,19713:up|Plpp3,67916:up|Creb3l1,26427:down|Fgfr2,14183:up|Prl2c2,18811:up|Efemp1,216616:up|Inhba,16323:down|Rasl11a,68895:up|Bnc2,242509:down|Ccl3,20302:up|Vdr,22337:down|Fabp4,11770:up|Vegfa,22339:down|Igf2,16002:up|Slc40a1,53945:up|Pparg,19016:up|Gas6,14456:up|Tnf,21926:up|Glp1r,14652:down|Lmcd1,30937:up|Fgfr1,14182:down|Egfr,13649:down|Hmox1,15368:up|Sfrp1,20377:up|Sulf1,240725:down|Tgfbr3,21814:up|Grem1,23892:up|Sfrp2,20319:up|Phf11d,219132:up|Tbx15,21384:down|Npnt,114249:up|S100a1,20193:up
dicarboxylic acid transport	GO:0006835	3.02E-01	4.02E-01	Tnf,21926:up|Il1rn,16181:up
defense response to Gram-positive bacterium	GO:0050830	3.02E-01	4.02E-01	Tnf,21926:up|Adm,11535:up
positive regulation of cAMP biosynthetic process	GO:0030819	3.02E-01	4.02E-01	Adm,11535:up|Ptgir,19222:up
positive regulation of response to DNA damage stimulus	GO:2001022	3.02E-01	4.02E-01	Ankrd1,107765:down|Egfr,13649:down
cytoplasmic transport	GO:0016482	3.04E-01	4.02E-01	Htr2b,15559:down|Htr2a,15558:down|Gas6,14456:up|Tnf,21926:up|Akr1c18,105349:up|Glp1r,14652:down|Egfr,13649:down|Sec23a,20334:down|C1qtnf3,81799:down|Cd4,12504:up|Bmpr1a,12166:down|Ptgs2,19225:down|Grem1,23892:up|Bmp4,12159:up|Igf1,16000:up|Ceacam1,26365:up|Sfrp2,20319:up|Tgfb2,21808:down
anatomical structure arrangement	GO:0048532	3.06E-01	4.02E-01	Bmpr1a,12166:down
misfolded or incompletely synthesized protein catabolic process	GO:0006515	3.06E-01	4.02E-01	Sdf2l1,64136:up
glutamine family amino acid catabolic process	GO:0009065	3.06E-01	4.02E-01	Ddah1,69219:down
mannosylation	GO:0097502	3.06E-01	4.02E-01	Sdf2l1,64136:up
regulation of macrophage chemotaxis	GO:0010758	3.06E-01	4.02E-01	Ccl2,20296:up
vascular endothelial growth factor signaling pathway	GO:0038084	3.06E-01	4.02E-01	Vegfa,22339:down

interleukin-8 secretion	GO:0072606	3.06E-01	4.02E-01	Wnt5a,22418:down
inorganic cation import into cell	GO:0098659	3.06E-01	4.02E-01	Trf,22041:up
long term synaptic depression	GO:0060292	3.06E-01	4.02E-01	Plk2,20620:down
macromolecular complex remodeling	GO:0034367	3.06E-01	4.02E-01	Lpl,16956:up
embryonic digestive tract morphogenesis	GO:0048557	3.06E-01	4.02E-01	Fgfr2,14183:up
negative regulation of oxidoreductase activity	GO:0051354	3.06E-01	4.02E-01	Hp,15439:up
regulation of triglyceride biosynthetic process	GO:0010866	3.06E-01	4.02E-01	C3,12266:up
regulation of growth of symbiont in host	GO:0044126	3.06E-01	4.02E-01	Tnf,21926:up
positive regulation of adherens junction organization	GO:1903393	3.06E-01	4.02E-01	Vegfa,22339:down
inorganic ion import into cell	GO:0099587	3.06E-01	4.02E-01	Trf,22041:up
negative regulation of regulated secretory pathway	GO:1903306	3.06E-01	4.02E-01	Hmox1,15368:up
cell proliferation involved in kidney development	GO:0072111	3.06E-01	4.02E-01	Bmp4,12159:up
positive regulation of protein complex disassembly	GO:0043243	3.06E-01	4.02E-01	Tnf,21926:up
podosome assembly	GO:0071800	3.06E-01	4.02E-01	Tnf,21926:up
nephron tubule formation	GO:0072079	3.06E-01	4.02E-01	Grem1,23892:up
plasma lipoprotein particle remodeling	GO:0034369	3.06E-01	4.02E-01	Lpl,16956:up
microglial cell activation	GO:0001774	3.06E-01	4.02E-01	Cx3cr1,13051:down
N-glycan processing	GO:0006491	3.06E-01	4.02E-01	Man2a2,140481:up
protein-lipid complex remodeling	GO:0034368	3.06E-01	4.02E-01	Lpl,16956:up
negative regulation of ERBB signaling pathway	GO:1901185	3.06E-01	4.02E-01	Errfi1,74155:down
negative regulation of lipid catabolic process	GO:0050995	3.06E-01	4.02E-01	Tnf,21926:up
cardiac muscle adaptation	GO:0014887	3.06E-01	4.02E-01	Errfi1,74155:down
atrial septum development	GO:0003283	3.06E-01	4.02E-01	Tgfb2,21808:down
protein mannosylation	GO:0035268	3.06E-01	4.02E-01	Sdf2l1,64136:up
positive regulation of erythrocyte differentiation	GO:0045648	3.06E-01	4.02E-01	Inhba,16323:down
metanephric nephron epithelium development	GO:0072243	3.06E-01	4.02E-01	Adipoq,11450:up
negative regulation of calcium ion import	GO:0090281	3.06E-01	4.02E-01	Tgfb2,21808:down
modulation of growth of symbiont involved in interaction with host	GO:0044144	3.06E-01	4.02E-01	Tnf,21926:up
acute inflammatory response to antigenic stimulus	GO:0002438	3.06E-01	4.02E-01	C3,12266:up
GTP metabolic process	GO:0046039	3.06E-01	4.02E-01	Ampd3,11717:up
tyrosine phosphorylation of STAT protein	GO:0007260	3.09E-01	4.06E-01	Crlf1,12931:down|Igf1,16000:up
cellular response to unfolded protein	GO:0034620	3.09E-01	4.06E-01	Sdf2l1,64136:up|Creb3l1,26427:down
regulation of cardiac muscle contraction	GO:0055117	3.09E-01	4.06E-01	Fxyd1,56188:up|Stc1,20855:up

adenylate cyclase-inhibiting G-protein coupled receptor signaling pathway	GO:0007193	3.09E-01	4.06E-01	Htr2b,15559:down|Htr2a,15558:down
determination of heart left/right asymmetry	GO:0061371	3.09E-01	4.06E-01	Wnt5a,22418:down|Aldh1a2,19378:up
regulation of calcium ion transmembrane transport	GO:1903169	3.10E-01	4.07E-01	Tgfb2,21808:down|S100a1,20193:up|Bmp4,12159:up
plasma membrane organization	GO:0007009	3.15E-01	4.13E-01	Tnf,21926:up|Gas6,14456:up|Col5a1,12831:down|Tgfb2,21808:down|Lrrc15,74488:down|Adipoq,11450:up
regulation of T cell differentiation	GO:0045580	3.15E-01	4.13E-01	Ctla2a,13024:down|Tnfsf9,21950:up|Bmp4,12159:up
embryonic heart tube morphogenesis	GO:0003143	3.16E-01	4.13E-01	Wnt5a,22418:down|Aldh1a2,19378:up
cell redox homeostasis	GO:0045454	3.16E-01	4.13E-01	Pdia5,72599:down|Nxn,18230:down
lens development in camera-type eye	GO:0002088	3.16E-01	4.13E-01	Fgfr2,14183:up|Bmp4,12159:up
nucleoside triphosphate metabolic process	GO:0009141	3.17E-01	4.13E-01	Htr2a,15558:down|Enpp1,18605:down|Ampd3,11717:up|Enpp3,209558:down|Igf1,16000:up
aromatic compound biosynthetic process	GO:0019438	3.17E-01	4.13E-01	Fstl3,83554:down|Ice1,218333:down|Adm,11535:up|Tfec,21426:up|Sfrp4,20379:up|Ednrb,13618:up|Ampd3,11717:up|Epas1,13819:up|Ankrd1,107765:down|Htra3,78558:down|Uchl5,56207:up|Lyl1,17095:up|Sdpr,20324:down|Loxl2,94352:down|Loxl3,16950:down|Trf,22041:up|Wnt5a,22418:down|Bmpr1a,12166:down|Ccl4,20303:up|Adipoq,11450:up|Bmp4,12159:up|Igf1,16000:up|Ret,19713:up|Tgfb2,21808:down|Pdxk,216134:up|Plpp3,67916:up|Ptgir,19222:up|Creb3l1,26427:down|Fgfr2,14183:up|Htr2b,15559:down|Prl2c2,18811:up|Efemp1,216616:up|Inhba,16323:down|Rasl11a,68895:up|Bnc2,242509:down|Ccl3,20302:up|Vdr,22337:down|Fabp4,11770:up|Vegfa,22339:down|Htr2a,15558:down|Igf2,16002:up|Slc40a1,53945:up|Pparg,19016:up|Gas6,14456:up|Tnf,21926:up|Glp1r,14652:down|Lmcd1,30937:up|Fgfr1,14182:down|Egfr,13649:down|Hmox1,15368:up|Sfrp1,20377:up|Sulf1,240725:down|Tgfbr3,21814:up|Grem1,23892:up|Phf11d,219132:up|Sfrp2,20319:up|Tbx15,21384:down|Npnt,114249:up|Uck2,80914:down|S100a1,20193:up
purine nucleotide biosynthetic process	GO:0006164	3.18E-01	4.13E-01	Htr2b,15559:down|Adm,11535:up|Htr2a,15558:down|Ptgir,19222:up|Ampd3,11717:up|Igf1,16000:up
positive regulation of filopodium assembly	GO:0051491	3.18E-01	4.13E-01	Dpysl3,22240:down
T cell lineage commitment	GO:0002360	3.18E-01	4.13E-01	Ly9,17085:up
notochord development	GO:0030903	3.18E-01	4.13E-01	Wnt5a,22418:down
regulation of long-term synaptic potentiation	GO:1900271	3.18E-01	4.13E-01	Cx3cr1,13051:down
regulatory T cell differentiation	GO:0045066	3.18E-01	4.13E-01	Ctla2a,13024:down
mammary gland lobule development	GO:0061377	3.18E-01	4.13E-01	Vegfa,22339:down
response to antibiotic	GO:0046677	3.18E-01	4.13E-01	Alpl,11647:up
regulation of establishment or maintenance of cell polarity	GO:0032878	3.18E-01	4.13E-01	Igf1,16000:up
epithelial cell proliferation involved in liver morphogenesis	GO:0072575	3.18E-01	4.13E-01	Ceacam1,26365:up
hepatocyte proliferation	GO:0072574	3.18E-01	4.13E-01	Ceacam1,26365:up
respiratory burst	GO:0045730	3.18E-01	4.13E-01	Cd24a,12484:down
positive regulation of cellular response to insulin stimulus	GO:1900078	3.18E-01	4.13E-01	Igf2,16002:up
positive regulation of protein deacetylation	GO:0090312	3.18E-01	4.13E-01	Vegfa,22339:down
tongue development	GO:0043586	3.18E-01	4.13E-01	Bnc2,242509:down
tyrosine phosphorylation of Stat5 protein	GO:0042506	3.18E-01	4.13E-01	Igf1,16000:up
regulation of interleukin-17 production	GO:0032660	3.18E-01	4.13E-01	Ly9,17085:up
defense response to fungus	GO:0050832	3.18E-01	4.13E-01	Clec4n,56620:up
regulation of glutamate secretion	GO:0014048	3.18E-01	4.13E-01	Il1rn,16181:up
protein targeting to plasma membrane	GO:0072661	3.18E-01	4.13E-01	Gas6,14456:up

regulation of histone deacetylation	GO:0031063	3.18E-01	4.13E-01	Vegfa,22339:down
positive regulation of muscle cell apoptotic process	GO:0010661	3.18E-01	4.13E-01	Igfbp3,16009:up
negative regulation of intrinsic apoptotic signaling pathway in response to DNA damage	GO:1902230	3.18E-01	4.13E-01	Cxcl12,20315:up
mammary gland alveolus development	GO:0060749	3.18E-01	4.13E-01	Vegfa,22339:down
astrocyte development	GO:0014002	3.18E-01	4.13E-01	Egfr,13649:down
triglyceride catabolic process	GO:0019433	3.18E-01	4.13E-01	Lpl,16956:up
response to superoxide	GO:0000303	3.18E-01	4.13E-01	Tnf,21926:up
pyrimidine nucleotide biosynthetic process	GO:0006221	3.18E-01	4.13E-01	Uck2,80914:down
secondary metabolite biosynthetic process	GO:0044550	3.18E-01	4.13E-01	Wnt5a,22418:down
C21-steroid hormone metabolic process	GO:0008207	3.18E-01	4.13E-01	Akr1c18,105349:up
regulation of cellular component biogenesis	GO:0044087	3.21E-01	4.17E-01	Tnf,21926:up|Prl2c2,18811:up|Ice1,218333:down|Plk2,20620:down|Flrt2,399558:down|Cd24a,12484:down|Ncam1,17967:down|Wnt5a,22418:down|Dpysl3,22240:down|Grem1,23892:up|Srpx2,68792:down|Gpm6b,14758:up|Vegfa,22339:down|Thbs2,21826:down
integrin-mediated signaling pathway	GO:0007229	3.22E-01	4.18E-01	Sema7a,20361:down|Col3a1,12825:down
regulation of neurotransmitter transport	GO:0051588	3.22E-01	4.18E-01	Gpm6b,14758:up|Sncg,20618:up
negative regulation of protein localization to nucleus	GO:1900181	3.22E-01	4.18E-01	Gas6,14456:up|C1qtnf3,81799:down
sperm motility	GO:0030317	3.22E-01	4.18E-01	Sord,20322:up|Ceacam10,26366:up
cellular component disassembly	GO:0022411	3.24E-01	4.20E-01	Tnf,21926:up|Lmcd1,30937:up|Cd24a,12484:down|Sfrp4,20379:up|Mmp13,17386:up|Plod2,26432:down|Tgfb2,21808:down|Igfbp3,16009:up
regulation of neurotransmitter levels	GO:0001505	3.24E-01	4.20E-01	Gpm6b,14758:up|Syt13,80976:up|Sncg,20618:up|Maob,109731:up
purine nucleoside monophosphate biosynthetic process	GO:0009127	3.25E-01	4.21E-01	Htr2a,15558:down|Ampd3,11717:up|Igf1,16000:up
purine ribonucleoside monophosphate biosynthetic process	GO:0009168	3.25E-01	4.21E-01	Htr2a,15558:down|Ampd3,11717:up|Igf1,16000:up
protein heterooligomerization	GO:0051291	3.25E-01	4.21E-01	Col1a2,12843:down|C1qtnf6,72709:down|Adipoq,11450:up
RNA biosynthetic process	GO:0032774	3.27E-01	4.23E-01	Fstl3,83554:down|Ice1,218333:down|Tfec,21426:up|Sfrp4,20379:up|Ednrb,13618:up|Epas1,13819:up|Ankrd1,107765:down|Htra3,78558:down|Uchl5,56207:up|Lyl1,17095:up|Sdpr,20324:down|Loxl2,94352:down|Loxl3,16950:down|Trf,22041:up|Wnt5a,22418:down|Bmpr1a,12166:down|Ccl4,20303:up|Adipoq,11450:up|Bmp4,12159:up|Igf1,16000:up|Ret,19713:up|Plpp3,67916:up|Creb3l1,26427:down|Fgfr2,14183:up|Prl2c2,18811:up|Efemp1,216616:up|Inhba,16323:down|Rasl11a,68895:up|Bnc2,242509:down|Ccl3,20302:up|Vdr,22337:down|Fabp4,11770:up|Vegfa,22339:down|Igf2,16002:up|Slc40a1,53945:up|Pparg,19016:up|Gas6,14456:up|Tnf,21926:up|Glp1r,14652:down|Lmcd1,30937:up|Fgfr1,14182:down|Egfr,13649:down|Hmox1,15368:up|Sfrp1,20377:up|Sulf1,240725:down|Tgfbr3,21814:up|Grem1,23892:up|Phf11d,219132:up|Sfrp2,20319:up|Tbx15,21384:down|Npnt,114249:up|S100a1,20193:up
CD4-positive, alpha-beta T cell activation	GO:0035710	3.29E-01	4.25E-01	Ceacam1,26365:up|Ly9,17085:up
myeloid cell activation involved in immune response	GO:0002275	3.29E-01	4.25E-01	Hmox1,15368:up|Cx3cr1,13051:down
synaptic vesicle transport	GO:0048489	3.30E-01	4.25E-01	Cd24a,12484:down|Syt13,80976:up|Fgfr2,14183:up
establishment of synaptic vesicle localization	GO:0097480	3.30E-01	4.25E-01	Cd24a,12484:down|Syt13,80976:up|Fgfr2,14183:up
positive regulation of heart rate	GO:0010460	3.31E-01	4.25E-01	Adm,11535:up
secretory granule organization	GO:0033363	3.31E-01	4.25E-01	Serpine2,20720:up
negative regulation of response to reactive oxygen species	GO:1901032	3.31E-01	4.25E-01	Hp,15439:up
negative regulation of T cell apoptotic process	GO:0070233	3.31E-01	4.25E-01	Bmp4,12159:up
negative regulation of protein dephosphorylation	GO:0035308	3.31E-01	4.25E-01	Tnf,21926:up

regulation of cellular senescence	GO:2000772	3.31E-01	4.25E-01	Bmpr1a,12166:down
regulation of activin receptor signaling pathway	GO:0032925	3.31E-01	4.25E-01	Fstl3,83554:down
regulation of voltage-gated calcium channel activity	GO:1901385	3.31E-01	4.25E-01	S100a1,20193:up
microvillus organization	GO:0032528	3.31E-01	4.25E-01	Prl2c2,18811:up
negative regulation of cardiac muscle cell apoptotic process	GO:0010667	3.31E-01	4.25E-01	Sfrp2,20319:up
cellular response to epidermal growth factor stimulus	GO:0071364	3.31E-01	4.25E-01	Egfr,13649:down
uterus development	GO:0060065	3.31E-01	4.25E-01	Wnt5a,22418:down
response to dietary excess	GO:0002021	3.31E-01	4.25E-01	Pparg,19016:up
vascular smooth muscle cell differentiation	GO:0035886	3.31E-01	4.25E-01	Adm,11535:up
cellular detoxification	GO:1990748	3.31E-01	4.25E-01	Tnf,21926:up
positive regulation of DNA recombination	GO:0045911	3.31E-01	4.25E-01	Tfrc,22042:up
regulation of T-helper 1 type immune response	GO:0002825	3.31E-01	4.25E-01	Il1rl1,17082:down
response to oxygen radical	GO:0000305	3.31E-01	4.25E-01	Tnf,21926:up
neurotransmitter uptake	GO:0001504	3.31E-01	4.25E-01	Gpm6b,14758:up
middle ear morphogenesis	GO:0042474	3.31E-01	4.25E-01	Fgfr1,14182:down
protein kinase A signaling	GO:0010737	3.31E-01	4.25E-01	Adipoq,11450:up
negative regulation of calcium ion transmembrane transport	GO:1903170	3.31E-01	4.25E-01	Tgfb2,21808:down
nuclear DNA replication	GO:0033260	3.31E-01	4.25E-01	Fgfr1,14182:down
nucleotide biosynthetic process	GO:0009165	3.32E-01	4.26E-01	Htr2b,15559:down|Adm,11535:up|Htr2a,15558:down|Ptgir,19222:up|Uck2,80914:down|Ampd3,11717:up|Igf1,16000:up
cofactor metabolic process	GO:0051186	3.32E-01	4.26E-01	Akr1c18,105349:up|Hmox1,15368:up|Htr2a,15558:down|Pdxk,216134:up|Blvrb,233016:up|Cbr2,12409:up|Igf1,16000:up
negative regulation of nitrogen compound metabolic process	GO:0051172	3.34E-01	4.28E-01	Sfrp4,20379:up|Ednrb,13618:up|Epas1,13819:up|Ankrd1,107765:down|Vdr,22337:down|Fabp4,11770:up|Vegfa,22339:down|Gas6,14456:up|Pparg,19016:up|Tnf,21926:up|Loxl2,94352:down|Loxl3,16950:down|Lmcd1,30937:up|Fgfr1,14182:down|Egfr,13649:down|Hmox1,15368:up|Sfrp1,20377:up|Wnt5a,22418:down|Ccl4,20303:up|Grem1,23892:up|Adipoq,11450:up|Bmp4,12159:up|Sfrp2,20319:up|Fgfr2,14183:up|S100a1,20193:up
activation of mitophagy in response to mitochondrial depolarization	GO:0098779	3.35E-01	4.30E-01	Lmcd1,30937:up|Plod2,26432:down|Sfrp4,20379:up
response to mitochondrial depolarisation	GO:0098780	3.35E-01	4.30E-01	Lmcd1,30937:up|Plod2,26432:down|Sfrp4,20379:up
response to ionizing radiation	GO:0010212	3.35E-01	4.30E-01	Sfrp2,20319:up|Sfrp1,20377:up|Nabp1,109019:down
response to organophosphorus	GO:0046683	3.36E-01	4.30E-01	Cdo1,12583:up|Adipoq,11450:up
positive regulation of cAMP metabolic process	GO:0030816	3.36E-01	4.30E-01	Adm,11535:up|Ptgir,19222:up
regulation of cellular response to oxidative stress	GO:1900407	3.36E-01	4.30E-01	Tnf,21926:up|Hp,15439:up
nucleoside phosphate biosynthetic process	GO:1901293	3.38E-01	4.33E-01	Htr2b,15559:down|Adm,11535:up|Htr2a,15558:down|Ptgir,19222:up|Uck2,80914:down|Ampd3,11717:up|Igf1,16000:up
organic anion transport	GO:0015711	3.38E-01	4.33E-01	Tnf,21926:up|Pparg,19016:up|Fam132b,227358:down|Emb,13723:up|Il1rn,16181:up|Slc16a4,229699:down|Serinc2,230779:down
purine-containing compound biosynthetic process	GO:0072522	3.40E-01	4.36E-01	Htr2b,15559:down|Adm,11535:up|Htr2a,15558:down|Ptgir,19222:up|Ampd3,11717:up|Igf1,16000:up

cellular process involved in reproduction in multicellular organism	GO:0022412	3.40E-01	4.36E-01	Sfrp1,20377:up|Cxcl12,20315:up|Inhba,16323:down|Kitl,17311:up|Igf1,16000:up|Bmp4,12159:up
organic substance catabolic process	GO:1901575	3.41E-01	4.36E-01	Mmp13,17386:up|Enpp5,83965:up|Uchl5,56207:up|Sord,20322:up|Ddah1,69219:down|Htr2a,15558:down|Blvrb,233016:up|Npl,74091:up|Serpine2,20720:up|Igfbp3,16009:up|Adamts12,239337:down|Enpp3,209558:down|Cdo1,12583:up|Anpep,16790:up|Tnf,21926:up|Plk2,20620:down|Akr1c18,105349:up|Egfr,13649:down|Hmox1,15368:up|Lpl,16956:up|Wnt5a,22418:down|Adipoq,11450:up|Hal,15109:up|Igf1,16000:up|Ceacam1,26365:up|Hpse,15442:up|Sdf2l1,64136:up|Enpp1,18605:down
negative regulation of phosphatase activity	GO:0010923	3.42E-01	4.36E-01	Tnf,21926:up|Tgfb2,21808:down
fatty acid transport	GO:0015908	3.42E-01	4.36E-01	Pparg,19016:up|Fam132b,227358:down
liver morphogenesis	GO:0072576	3.43E-01	4.36E-01	Ceacam1,26365:up
B cell apoptotic process	GO:0001783	3.43E-01	4.36E-01	Cd24a,12484:down
prostaglandin biosynthetic process	GO:0001516	3.43E-01	4.36E-01	Ptgs2,19225:down
protein kinase C-activating G-protein coupled receptor signaling pathway	GO:0007205	3.43E-01	4.36E-01	Htr2b,15559:down
prostanoid biosynthetic process	GO:0046457	3.43E-01	4.36E-01	Ptgs2,19225:down
positive regulation of transcription from RNA polymerase II promoter involved in cellular response to chemical stimulus	GO:1901522	3.43E-01	4.36E-01	Vegfa,22339:down
lymph vessel development	GO:0001945	3.43E-01	4.36E-01	Vegfa,22339:down
positive regulation of neurological system process	GO:0031646	3.43E-01	4.36E-01	Trf,22041:up
glomerulus vasculature development	GO:0072012	3.43E-01	4.36E-01	Bmp4,12159:up
positive regulation of blood coagulation	GO:0030194	3.43E-01	4.36E-01	Hpse,15442:up
positive regulation of hemostasis	GO:1900048	3.43E-01	4.36E-01	Hpse,15442:up
cellular response to mechanical stimulus	GO:0071260	3.43E-01	4.36E-01	Ankrd1,107765:down
maternal placenta development	GO:0001893	3.43E-01	4.36E-01	Ptgs2,19225:down
fluid transport	GO:0042044	3.43E-01	4.36E-01	Ednrb,13618:up
lens morphogenesis in camera-type eye	GO:0002089	3.43E-01	4.36E-01	Bmp4,12159:up
regulation of gene silencing by miRNA	GO:0060964	3.43E-01	4.36E-01	Egfr,13649:down
renal system process involved in regulation of systemic arterial blood pressure	GO:0003071	3.43E-01	4.36E-01	Gas6,14456:up
T cell costimulation	GO:0031295	3.43E-01	4.36E-01	Cd24a,12484:down
positive regulation of peptidyl-threonine phosphorylation	GO:0010800	3.43E-01	4.36E-01	Wnt5a,22418:down
DNA catabolic process	GO:0006308	3.43E-01	4.36E-01	Igfbp3,16009:up
acidic amino acid transport	GO:0015800	3.43E-01	4.36E-01	Tnf,21926:up
glutamine metabolic process	GO:0006541	3.43E-01	4.36E-01	Gfpt2,14584:down
beta-amyloid metabolic process	GO:0050435	3.43E-01	4.36E-01	Mme,17380:up
centriole replication	GO:0007099	3.43E-01	4.36E-01	Plk2,20620:down
metanephric epithelium development	GO:0072207	3.43E-01	4.36E-01	Adipoq,11450:up
negative regulation of endothelial cell apoptotic process	GO:2000352	3.43E-01	4.36E-01	Gas6,14456:up
positive regulation of mitochondrion degradation	GO:1903599	3.45E-01	4.38E-01	Lmcd1,30937:up|Plod2,26432:down|Sfrp4,20379:up

cellular amine metabolic process	GO:0044106	3.45E-01	4.38E-01	Fabp5,16592:up|Maob,109731:up|Epas1,13819:up
positive regulation of ion transmembrane transporter activity	GO:0032414	3.49E-01	4.43E-01	S100a1,20193:up|Bmp4,12159:up
adenylate cyclase-activating G-protein coupled receptor signaling pathway	GO:0007189	3.49E-01	4.43E-01	Glp1r,14652:down|Ptgir,19222:up
nicotinamide nucleotide metabolic process	GO:0046496	3.50E-01	4.44E-01	Htr2a,15558:down|Cbr2,12409:up|Igf1,16000:up
striated muscle contraction	GO:0006941	3.50E-01	4.44E-01	Fxyd1,56188:up|Vegfb,22340:up|Stc1,20855:up
alpha-beta T cell activation	GO:0046631	3.50E-01	4.44E-01	Ceacam1,26365:up|Cd24a,12484:down|Ly9,17085:up
determination of bilateral symmetry	GO:0009855	3.50E-01	4.44E-01	Wnt5a,22418:down|Aldh1a2,19378:up|Grem1,23892:up
glycerophospholipid metabolic process	GO:0006650	3.53E-01	4.47E-01	Htr2b,15559:down|Fabp5,16592:up|Htr2a,15558:down|Serinc2,230779:down
negative regulation of smoothened signaling pathway	GO:0045879	3.55E-01	4.47E-01	Serpine2,20720:up
kidney vasculature development	GO:0061440	3.55E-01	4.47E-01	Bmp4,12159:up
maintenance of protein localization in organelle	GO:0072595	3.55E-01	4.47E-01	Kdelr3,105785:down
growth of symbiont involved in interaction with host	GO:0044116	3.55E-01	4.47E-01	Tnf,21926:up
positive regulation of CD4-positive, alpha-beta T cell activation	GO:2000516	3.55E-01	4.47E-01	Ceacam1,26365:up
superoxide anion generation	GO:0042554	3.55E-01	4.47E-01	Egfr,13649:down
centriole assembly	GO:0098534	3.55E-01	4.47E-01	Plk2,20620:down
neuron recognition	GO:0008038	3.55E-01	4.47E-01	Wnt5a,22418:down
negative regulation of muscle contraction	GO:0045932	3.55E-01	4.47E-01	Ptgs2,19225:down
growth of symbiont in host	GO:0044117	3.55E-01	4.47E-01	Tnf,21926:up
negative regulation of macroautophagy	GO:0016242	3.55E-01	4.47E-01	Hmox1,15368:up
lymphocyte costimulation	GO:0031294	3.55E-01	4.47E-01	Cd24a,12484:down
growth involved in symbiotic interaction	GO:0044110	3.55E-01	4.47E-01	Tnf,21926:up
positive regulation of natural killer cell activation	GO:0032816	3.55E-01	4.47E-01	Gas6,14456:up
epiboly involved in wound healing	GO:0090505	3.55E-01	4.47E-01	Col5a1,12831:down
negative regulation of striated muscle cell apoptotic process	GO:0010664	3.55E-01	4.47E-01	Sfrp2,20319:up
wound healing, spreading of cells	GO:0044319	3.55E-01	4.47E-01	Col5a1,12831:down
renal system vasculature development	GO:0061437	3.55E-01	4.47E-01	Bmp4,12159:up
positive regulation of myeloid leukocyte mediated immunity	GO:0002888	3.55E-01	4.47E-01	C3,12266:up
potassium ion import	GO:0010107	3.55E-01	4.47E-01	Kcnj15,16516:down
negative regulation of axon extension involved in axon guidance	GO:0048843	3.55E-01	4.47E-01	Wnt5a,22418:down
regulation of execution phase of apoptosis	GO:1900117	3.55E-01	4.47E-01	Igfbp3,16009:up
pyrimidine nucleoside metabolic process	GO:0006213	3.55E-01	4.47E-01	Uck2,80914:down
negative regulation of response to biotic stimulus	GO:0002832	3.55E-01	4.47E-01	Ceacam1,26365:up

macromitophagy	GO:0000423	3.55E-01	4.47E-01	Lmcd1,30937:up|Plod2,26432:down|Sfrp4,20379:up
positive regulation of JAK-STAT cascade	GO:0046427	3.55E-01	4.48E-01	Crlf1,12931:down|Igf1,16000:up
cellular amino acid biosynthetic process	GO:0008652	3.55E-01	4.48E-01	Gatm,67092:down|Cdo1,12583:up
regulation of ion transmembrane transporter activity	GO:0032412	3.57E-01	4.50E-01	Fxyd1,56188:up|Fxyd2,11936:up|S100a1,20193:up|Bmp4,12159:up
endocrine system development	GO:0035270	3.60E-01	4.53E-01	Wnt5a,22418:down|Bmpr1a,12166:down|Bmp4,12159:up
mitochondrion degradation by induced vacuole formation	GO:0035695	3.60E-01	4.53E-01	Lmcd1,30937:up|Plod2,26432:down|Sfrp4,20379:up
synaptic vesicle localization	GO:0097479	3.60E-01	4.53E-01	Cd24a,12484:down|Syt13,80976:up|Fgfr2,14183:up
negative regulation of binding	GO:0051100	3.60E-01	4.53E-01	Hmox1,15368:up|Slpi,20568:up|Adipoq,11450:up
pyridine nucleotide metabolic process	GO:0019362	3.60E-01	4.53E-01	Htr2a,15558:down|Cbr2,12409:up|Igf1,16000:up
cellular response to radiation	GO:0071478	3.60E-01	4.53E-01	Sfrp2,20319:up|Sfrp1,20377:up|Mme,17380:up
regulation of response to biotic stimulus	GO:0002831	3.61E-01	4.54E-01	Ceacam1,26365:up|Lmcd1,30937:up|Cd24a,12484:down|Ifit1,15957:up
response to dsRNA	GO:0043331	3.62E-01	4.55E-01	Egfr,13649:down|Ifit1,15957:up
protein localization	GO:0008104	3.64E-01	4.57E-01	Ice1,218333:down|Ccl2,20296:up|Mmp13,17386:up|Ankrd1,107765:down|Gpm6b,14758:up|Clec4n,56620:up|Postn,50706:down|Akr1c18,105349:up|Cd24a,12484:down|Wnt5a,22418:down|Bmpr1a,12166:down|Il1rn,16181:up|Adipoq,11450:up|Bmp4,12159:up|Igf1,16000:up|Tgfb2,21808:down|Il1rl1,17082:down|Clec4e,56619:up|Htr2b,15559:down|Ccl3,20302:up|Htr2a,15558:down|Lrrc15,74488:down|Kdelr3,105785:down|Tnf,21926:up|Pparg,19016:up|Gas6,14456:up|Glp1r,14652:down|Egfr,13649:down|Sfrp1,20377:up|Lpl,16956:up|Sec23a,20334:down|C1qtnf3,81799:down|Cd4,12504:up|Tfrc,22042:up|Ptgs2,19225:down|Sncg,20618:up|Grem1,23892:up|Ceacam1,26365:up|Sfrp2,20319:up|Npnt,114249:up
smoothened signaling pathway	GO:0007224	3.65E-01	4.58E-01	Sfrp1,20377:up|Serpine2,20720:up|Fgfr2,14183:up
organic cyclic compound biosynthetic process	GO:1901362	3.65E-01	4.58E-01	Fstl3,83554:down|Ice1,218333:down|Adm,11535:up|Tfec,21426:up|Sfrp4,20379:up|Ednrb,13618:up|Ampd3,11717:up|Epas1,13819:up|Ankrd1,107765:down|Htra3,78558:down|Uchl5,56207:up|Lyl1,17095:up|Sdpr,20324:down|Loxl2,94352:down|Loxl3,16950:down|Akr1c18,105349:up|Trf,22041:up|Wnt5a,22418:down|Bmpr1a,12166:down|Ccl4,20303:up|Adipoq,11450:up|Bmp4,12159:up|Igf1,16000:up|Ret,19713:up|Tgfb2,21808:down|Pdxk,216134:up|Plpp3,67916:up|Ptgir,19222:up|Creb3l1,26427:down|Fgfr2,14183:up|Htr2b,15559:down|Prl2c2,18811:up|Efemp1,216616:up|Inhba,16323:down|Rasl11a,68895:up|Bnc2,242509:down|Ccl3,20302:up|Vdr,22337:down|Fabp4,11770:up|Vegfa,22339:down|Htr2a,15558:down|Igf2,16002:up|Slc40a1,53945:up|Pparg,19016:up|Gas6,14456:up|Tnf,21926:up|Glp1r,14652:down|Lmcd1,30937:up|Fgfr1,14182:down|Egfr,13649:down|Hmox1,15368:up|Sfrp1,20377:up|Sulf1,240725:down|Tgfbr3,21814:up|Grem1,23892:up|Phf11d,219132:up|Sfrp2,20319:up|Tbx15,21384:down|Npnt,114249:up|Uck2,80914:down|S100a1,20193:up
nucleobase-containing compound biosynthetic process	GO:0034654	3.65E-01	4.58E-01	Fstl3,83554:down|Ice1,218333:down|Adm,11535:up|Tfec,21426:up|Sfrp4,20379:up|Ednrb,13618:up|Ampd3,11717:up|Epas1,13819:up|Ankrd1,107765:down|Htra3,78558:down|Uchl5,56207:up|Lyl1,17095:up|Sdpr,20324:down|Loxl2,94352:down|Loxl3,16950:down|Trf,22041:up|Wnt5a,22418:down|Bmpr1a,12166:down|Ccl4,20303:up|Adipoq,11450:up|Bmp4,12159:up|Igf1,16000:up|Ret,19713:up|Plpp3,67916:up|Ptgir,19222:up|Creb3l1,26427:down|Fgfr2,14183:up|Htr2b,15559:down|Prl2c2,18811:up|Efemp1,216616:up|Inhba,16323:down|Rasl11a,68895:up|Bnc2,242509:down|Ccl3,20302:up|Vdr,22337:down|Fabp4,11770:up|Vegfa,22339:down|Htr2a,15558:down|Igf2,16002:up|Slc40a1,53945:up|Pparg,19016:up|Gas6,14456:up|Tnf,21926:up|Glp1r,14652:down|Lmcd1,30937:up|Fgfr1,14182:down|Egfr,13649:down|Hmox1,15368:up|Sfrp1,20377:up|Sulf1,240725:down|Tgfbr3,21814:up|Grem1,23892:up|Phf11d,219132:up|Sfrp2,20319:up|Tbx15,21384:down|Npnt,114249:up|Uck2,80914:down|S100a1,20193:up
steroid hormone secretion	GO:0035929	3.66E-01	4.58E-01	Inhba,16323:down
positive regulation of TOR signaling	GO:0032008	3.66E-01	4.58E-01	Gas6,14456:up
regulation of protein targeting to membrane	GO:0090313	3.66E-01	4.58E-01	Ccl2,20296:up
negative regulation of receptor activity	GO:2000272	3.66E-01	4.58E-01	Errfi1,74155:down
negative regulation of axon guidance	GO:1902668	3.66E-01	4.58E-01	Wnt5a,22418:down
regulation of viral entry into host cell	GO:0046596	3.66E-01	4.58E-01	Ptx3,19288:up
positive regulation of G-protein coupled receptor protein signaling pathway	GO:0045745	3.66E-01	4.58E-01	C3,12266:up
response to epidermal growth factor	GO:0070849	3.66E-01	4.58E-01	Egfr,13649:down
primary alcohol metabolic process	GO:0034308	3.66E-01	4.58E-01	Akr1c18,105349:up
hyaluronan metabolic process	GO:0030212	3.66E-01	4.58E-01	Has2,15117:down

cellular response to alkaloid	GO:0071312	3.66E-01	4.58E-01	Tnf,21926:up
positive regulation of cell junction assembly	GO:1901890	3.66E-01	4.58E-01	Vegfa,22339:down
cellular response to interleukin-4	GO:0071353	3.66E-01	4.58E-01	Mrc1,17533:up
neurotransmitter metabolic process	GO:0042133	3.66E-01	4.58E-01	Maob,109731:up
quinone metabolic process	GO:1901661	3.66E-01	4.58E-01	Akr1c18,105349:up
epiboly	GO:0090504	3.66E-01	4.58E-01	Col5a1,12831:down
regulation of p38MAPK cascade	GO:1900744	3.66E-01	4.58E-01	Vegfa,22339:down
interleukin-17 production	GO:0032620	3.66E-01	4.58E-01	Ly9,17085:up
regulation of endoplasmic reticulum stress-induced intrinsic apoptotic signaling pathway	GO:1902235	3.66E-01	4.58E-01	Creb3l1,26427:down
positive regulation of telomerase activity	GO:0051973	3.66E-01	4.58E-01	Grem1,23892:up
negative regulation of exocytosis	GO:0045920	3.66E-01	4.58E-01	Hmox1,15368:up
multicellular organismal reproductive process	GO:0048609	3.67E-01	4.59E-01	Egfr,13649:down|Inhba,16323:down|Ptgs2,19225:down|Ednrb,13618:up|Plat,18791:down|Igf1,16000:up|Bmp4,12159:up|Vdr,22337:down|Cxcl12,20315:up|Vegfa,22339:down|Mmp2,17390:down|Serpine2,20720:up|Kitl,17311:up
macromolecule localization	GO:0033036	3.68E-01	4.60E-01	Ice1,218333:down|Ccl2,20296:up|C3,12266:up|Mmp13,17386:up|Ankrd1,107765:down|Gpm6b,14758:up|Clec4n,56620:up|Postn,50706:down|Akr1c18,105349:up|Cd24a,12484:down|Wnt5a,22418:down|Bmpr1a,12166:down|Il1rn,16181:up|Adipoq,11450:up|Bmp4,12159:up|Igf1,16000:up|Chst11,58250:down|Tgfb2,21808:down|Il1rl1,17082:down|Clec4e,56619:up|Htr2b,15559:down|Inhba,16323:down|Ccl3,20302:up|Fam132b,227358:down|Htr2a,15558:down|Lrrc15,74488:down|Kdelr3,105785:down|Pparg,19016:up|Gas6,14456:up|Tnf,21926:up|Glp1r,14652:down|Egfr,13649:down|Sec23a,20334:down|Sfrp1,20377:up|Lpl,16956:up|C1qtnf3,81799:down|Cd4,12504:up|Ptgs2,19225:down|Tfrc,22042:up|Sncg,20618:up|Grem1,23892:up|Ceacam1,26365:up|Sfrp2,20319:up|Npnt,114249:up|Enpp1,18605:down
heterocycle biosynthetic process	GO:0018130	3.68E-01	4.60E-01	Fstl3,83554:down|Ice1,218333:down|Adm,11535:up|Tfec,21426:up|Sfrp4,20379:up|Ednrb,13618:up|Ampd3,11717:up|Epas1,13819:up|Ankrd1,107765:down|Htra3,78558:down|Uchl5,56207:up|Lyl1,17095:up|Sdpr,20324:down|Loxl2,94352:down|Loxl3,16950:down|Trf,22041:up|Wnt5a,22418:down|Bmpr1a,12166:down|Ccl4,20303:up|Adipoq,11450:up|Bmp4,12159:up|Igf1,16000:up|Ret,19713:up|Pdxk,216134:up|Plpp3,67916:up|Ptgir,19222:up|Creb3l1,26427:down|Fgfr2,14183:up|Htr2b,15559:down|Prl2c2,18811:up|Efemp1,216616:up|Inhba,16323:down|Rasl11a,68895:up|Bnc2,242509:down|Ccl3,20302:up|Vdr,22337:down|Fabp4,11770:up|Vegfa,22339:down|Htr2a,15558:down|Igf2,16002:up|Slc40a1,53945:up|Pparg,19016:up|Gas6,14456:up|Tnf,21926:up|Glp1r,14652:down|Lmcd1,30937:up|Fgfr1,14182:down|Egfr,13649:down|Hmox1,15368:up|Sfrp1,20377:up|Sulf1,240725:down|Tgfbr3,21814:up|Grem1,23892:up|Phf11d,219132:up|Sfrp2,20319:up|Tbx15,21384:down|Npnt,114249:up|Uck2,80914:down|S100a1,20193:up
transcription, DNA-templated	GO:0006351	3.68E-01	4.60E-01	Fstl3,83554:down|Ice1,218333:down|Tfec,21426:up|Sfrp4,20379:up|Ednrb,13618:up|Epas1,13819:up|Ankrd1,107765:down|Htra3,78558:down|Uchl5,56207:up|Lyl1,17095:up|Sdpr,20324:down|Loxl2,94352:down|Loxl3,16950:down|Trf,22041:up|Wnt5a,22418:down|Bmpr1a,12166:down|Adipoq,11450:up|Bmp4,12159:up|Igf1,16000:up|Ret,19713:up|Plpp3,67916:up|Creb3l1,26427:down|Fgfr2,14183:up|Prl2c2,18811:up|Efemp1,216616:up|Inhba,16323:down|Rasl11a,68895:up|Bnc2,242509:down|Ccl3,20302:up|Vdr,22337:down|Fabp4,11770:up|Vegfa,22339:down|Igf2,16002:up|Slc40a1,53945:up|Pparg,19016:up|Gas6,14456:up|Tnf,21926:up|Glp1r,14652:down|Lmcd1,30937:up|Fgfr1,14182:down|Egfr,13649:down|Hmox1,15368:up|Sfrp1,20377:up|Sulf1,240725:down|Tgfbr3,21814:up|Grem1,23892:up|Sfrp2,20319:up|Phf11d,219132:up|Tbx15,21384:down|Npnt,114249:up|S100a1,20193:up
regulation of plasma membrane organization	GO:1903729	3.68E-01	4.60E-01	Tgfb2,21808:down|Lrrc15,74488:down
regulation of heart rate	GO:0002027	3.68E-01	4.60E-01	Adm,11535:up|Epas1,13819:up
regulation of response to oxidative stress	GO:1902882	3.68E-01	4.60E-01	Tnf,21926:up|Hp,15439:up
myeloid leukocyte mediated immunity	GO:0002444	3.68E-01	4.60E-01	Hmox1,15368:up|C3,12266:up
cell aging	GO:0007569	3.68E-01	4.60E-01	Prelp,116847:up|Bmpr1a,12166:down
response to insulin	GO:0032868	3.69E-01	4.61E-01	Pparg,19016:up|Enpp1,18605:down|Igf2,16002:up|Adipoq,11450:up
amine metabolic process	GO:0009308	3.70E-01	4.61E-01	Fabp5,16592:up|Maob,109731:up|Epas1,13819:up
nucleic acid-templated transcription	GO:0097659	3.71E-01	4.63E-01	Fstl3,83554:down|Ice1,218333:down|Tfec,21426:up|Sfrp4,20379:up|Ednrb,13618:up|Epas1,13819:up|Ankrd1,107765:down|Htra3,78558:down|Uchl5,56207:up|Lyl1,17095:up|Sdpr,20324:down|Loxl2,94352:down|Loxl3,16950:down|Trf,22041:up|Wnt5a,22418:down|Bmpr1a,12166:down|Adipoq,11450:up|Bmp4,12159:up|Igf1,16000:up|Ret,19713:up|Plpp3,67916:up|Creb3l1,26427:down|Fgfr2,14183:up|Prl2c2,18811:up|Efemp1,216616:up|Inhba,16323:down|Rasl11a,68895:up|Bnc2,242509:down|Ccl3,20302:up|Vdr,22337:down|Fabp4,11770:up|Vegfa,22339:down|Igf2,16002:up|Slc40a1,53945:up|Pparg,19016:up|Gas6,14456:up|Tnf,21926:up|Glp1r,14652:down|Lmcd1,30937:up|Fgfr1,14182:down|Egfr,13649:down|Hmox1,15368:up|Sfrp1,20377:up|Sulf1,240725:down|Tgfbr3,21814:up|Grem1,23892:up|Sfrp2,20319:up|Phf11d,219132:up|Tbx15,21384:down|Npnt,114249:up|S100a1,20193:up

DNA metabolic process	GO:0006259	3.73E-01	4.65E-01	Pparg,19016:up|Fgfr1,14182:down|Egfr,13649:down|Pdgfc,54635:down|Tfrc,22042:up|Grem1,23892:up|Igf1,16000:up|Bmp4,12159:up|Adipoq,11450:up|Ankrd1,107765:down|Uchl5,56207:up|Gstt1,14871:up|Nabp1,109019:down|Igfbp3,16009:up|Kitl,17311:up
nucleoside diphosphate phosphorylation	GO:0006165	3.75E-01	4.67E-01	Htr2a,15558:down|Igf1,16000:up
nucleotide phosphorylation	GO:0046939	3.75E-01	4.67E-01	Htr2a,15558:down|Igf1,16000:up
cellular response to topologically incorrect protein	GO:0035967	3.75E-01	4.67E-01	Sdf2l1,64136:up|Creb3l1,26427:down
regulation of alpha-beta T cell activation	GO:0046634	3.75E-01	4.67E-01	Ceacam1,26365:up|Cd24a,12484:down
cellular biosynthetic process	GO:0044249	3.77E-01	4.68E-01	Fstl3,83554:down|Adm,11535:up|Tfec,21426:up|Sfrp4,20379:up|C3,12266:up|Ankrd1,107765:down|Lyl1,17095:up|Ggt5,23887:up|Loxl2,94352:down|Loxl3,16950:down|Trf,22041:up|Wnt5a,22418:down|Adipoq,11450:up|Bmp4,12159:up|Igf1,16000:up|Tgfb2,21808:down|Ptgir,19222:up|Fgfr2,14183:up|Htr2b,15559:down|Prl2c2,18811:up|Efemp1,216616:up|Vdr,22337:down|Fabp4,11770:up|Htr2a,15558:down|Slc40a1,53945:up|Gas6,14456:up|Egfr,13649:down|Sfrp1,20377:up|C1qtnf3,81799:down|Tgfbr3,21814:up|Sfrp2,20319:up|Npnt,114249:up|S100a1,20193:up|Ice1,218333:down|Aldh1a2,19378:up|Pdgfc,54635:down|Ednrb,13618:up|Ampd3,11717:up|Epas1,13819:up|Htra3,78558:down|Uchl5,56207:up|Ddah1,69219:down|Sdpr,20324:down|Cdo1,12583:up|Man2a2,140481:up|Ptx3,19288:up|Akr1c18,105349:up|Fabp5,16592:up|Bmpr1a,12166:down|Ccl4,20303:up|Chst11,58250:down|Gatm,67092:down|Ret,19713:up|Prg4,96875:down|Vegfb,22340:up|Sdf2l1,64136:up|Pdxk,216134:up|Aldh1a3,56847:down|Plpp3,67916:up|Creb3l1,26427:down|Kitl,17311:up|Rasl11a,68895:up|Inhba,16323:down|Bnc2,242509:down|Ccl3,20302:up|Vegfa,22339:down|Igf2,16002:up|Pparg,19016:up|Tnf,21926:up|Lmcd1,30937:up|Glp1r,14652:down|Fgfr1,14182:down|Hmox1,15368:up|Lpl,16956:up|Has2,15117:down|Sulf1,240725:down|Ptgs2,19225:down|Grem1,23892:up|Phf11d,219132:up|Tbx15,21384:down|Uck2,80914:down|Errfi1,74155:down|Enpp1,18605:down
regulation of transmembrane transporter activity	GO:0022898	3.78E-01	4.68E-01	Fxyd1,56188:up|Fxyd2,11936:up|S100a1,20193:up|Bmp4,12159:up
regulation of fatty acid transport	GO:2000191	3.78E-01	4.68E-01	Fam132b,227358:down
skeletal muscle tissue regeneration	GO:0043403	3.78E-01	4.68E-01	Igf1,16000:up
positive regulation of alcohol biosynthetic process	GO:1902932	3.78E-01	4.68E-01	Tnf,21926:up
natural killer cell differentiation	GO:0001779	3.78E-01	4.68E-01	Gas6,14456:up
necroptotic process	GO:0070266	3.78E-01	4.68E-01	Tnf,21926:up
regulation of isotype switching	GO:0045191	3.78E-01	4.68E-01	Tfrc,22042:up
pyrimidine-containing compound biosynthetic process	GO:0072528	3.78E-01	4.68E-01	Uck2,80914:down
positive regulation of coagulation	GO:0050820	3.78E-01	4.68E-01	Hpse,15442:up
collateral sprouting	GO:0048668	3.78E-01	4.68E-01	Crabp2,12904:down
negative regulation of dendrite development	GO:2000171	3.78E-01	4.68E-01	Plk2,20620:down
regulation of gene silencing by RNA	GO:0060966	3.78E-01	4.68E-01	Egfr,13649:down
transcription from RNA polymerase III promoter	GO:0006383	3.78E-01	4.68E-01	Ice1,218333:down
positive regulation of tyrosine phosphorylation of Stat3 protein	GO:0042517	3.78E-01	4.68E-01	Crlf1,12931:down
positive regulation of receptor activity	GO:2000273	3.78E-01	4.68E-01	Grem1,23892:up
ventricular system development	GO:0021591	3.78E-01	4.68E-01	Uchl5,56207:up
negative regulation of adaptive immune response based on somatic recombination of immune receptors built from immunoglobulin superfamily domains	GO:0002823	3.78E-01	4.68E-01	Il1rl1,17082:down
calcium ion-dependent exocytosis of neurotransmitter	GO:0048791	3.78E-01	4.68E-01	Syt13,80976:up
positive regulation of phosphatase activity	GO:0010922	3.78E-01	4.68E-01	Npnt,114249:up
guanosine-containing compound metabolic process	GO:1901068	3.78E-01	4.68E-01	Ampd3,11717:up

water-soluble vitamin metabolic process	GO:0006767	3.78E-01	4.68E-01	Pdxk,216134:up
regulation of posttranscriptional gene silencing	GO:0060147	3.78E-01	4.68E-01	Egfr,13649:down
cellular response to cAMP	GO:0071320	3.78E-01	4.68E-01	Adipoq,11450:up
positive regulation of cellular component biogenesis	GO:0044089	3.79E-01	4.69E-01	Tnf,21926:up|Ice1,218333:down|Flrt2,399558:down|Cd24a,12484:down|Dpysl3,22240:down|Srpx2,68792:down|Vegfa,22339:down|Thbs2,21826:down
alcohol metabolic process	GO:0006066	3.79E-01	4.69E-01	Vdr,22337:down|Tnf,21926:up|Sord,20322:up|Akr1c18,105349:up|Fgfr1,14182:down|Fabp5,16592:up
neuron fate commitment	GO:0048663	3.81E-01	4.71E-01	Tgfb2,21808:down|Bmp4,12159:up
T cell activation involved in immune response	GO:0002286	3.81E-01	4.71E-01	Ly9,17085:up|Clec4e,56619:up
regulation of multicellular organism growth	GO:0040014	3.81E-01	4.71E-01	Igf2,16002:up|Fgfr2,14183:up
regulation of striated muscle contraction	GO:0006942	3.81E-01	4.71E-01	Fxyd1,56188:up|Stc1,20855:up
positive regulation of cyclic nucleotide biosynthetic process	GO:0030804	3.81E-01	4.71E-01	Adm,11535:up|Ptgir,19222:up
cyclic purine nucleotide metabolic process	GO:0052652	3.84E-01	4.74E-01	Htr2b,15559:down|Adm,11535:up|Ptgir,19222:up
positive regulation of protein complex assembly	GO:0031334	3.86E-01	4.76E-01	Tnf,21926:up|Ice1,218333:down|Cd24a,12484:down|Vegfa,22339:down
lens fiber cell differentiation	GO:0070306	3.89E-01	4.78E-01	Fgfr2,14183:up
neuromuscular synaptic transmission	GO:0007274	3.89E-01	4.78E-01	Cd24a,12484:down
negative regulation of endothelial cell proliferation	GO:0001937	3.89E-01	4.78E-01	Sulf1,240725:down
negative chemotaxis	GO:0050919	3.89E-01	4.78E-01	Wnt5a,22418:down
regulation of DNA damage response, signal transduction by p53 class mediator	GO:0043516	3.89E-01	4.78E-01	Ankrd1,107765:down
nucleotide-sugar metabolic process	GO:0009225	3.89E-01	4.78E-01	Gfpt2,14584:down
response to interleukin-4	GO:0070670	3.89E-01	4.78E-01	Mrc1,17533:up
forebrain regionalization	GO:0021871	3.89E-01	4.78E-01	Bmp4,12159:up
production of miRNAs involved in gene silencing by miRNA	GO:0035196	3.89E-01	4.78E-01	Egfr,13649:down
negative regulation of cell-matrix adhesion	GO:0001953	3.89E-01	4.78E-01	Postn,50706:down
negative regulation of potassium ion transport	GO:0043267	3.89E-01	4.78E-01	Htr2a,15558:down
blastocyst growth	GO:0001832	3.89E-01	4.78E-01	Igf1,16000:up
cyclic nucleotide biosynthetic process	GO:0009190	3.89E-01	4.78E-01	Htr2b,15559:down|Adm,11535:up|Ptgir,19222:up
purine-containing compound metabolic process	GO:0072521	3.93E-01	4.83E-01	Htr2b,15559:down|Adm,11535:up|Wnt5a,22418:down|Ampd3,11717:up|Igf1,16000:up|Htr2a,15558:down|Vegfa,22339:down|Ptgir,19222:up|Enpp1,18605:down
negative regulation of sequence-specific DNA binding transcription factor activity	GO:0043433	3.94E-01	4.83E-01	Gas6,14456:up|Hmox1,15368:up|Sfrp4,20379:up
negative regulation of dephosphorylation	GO:0035305	3.94E-01	4.83E-01	Tnf,21926:up|Tgfb2,21808:down
amino acid transport	GO:0006865	3.99E-01	4.88E-01	Tnf,21926:up|Il1rn,16181:up|Serinc2,230779:down
dorsal/ventral neural tube patterning	GO:0021904	4.00E-01	4.88E-01	Bmp4,12159:up
production of small RNA involved in gene silencing by RNA	GO:0070918	4.00E-01	4.88E-01	Egfr,13649:down
embryonic camera-type eye morphogenesis	GO:0048596	4.00E-01	4.88E-01	Aldh1a3,56847:down

neutral lipid catabolic process	GO:0046461	4.00E-01	4.88E-01	Lpl,16956:up
plasma lipoprotein particle clearance	GO:0034381	4.00E-01	4.88E-01	Adipoq,11450:up
mammary gland epithelial cell proliferation	GO:0033598	4.00E-01	4.88E-01	Wnt5a,22418:down
pyrimidine nucleotide metabolic process	GO:0006220	4.00E-01	4.88E-01	Uck2,80914:down
transcription from RNA polymerase I promoter	GO:0006360	4.00E-01	4.88E-01	Rasl11a,68895:up
positive T cell selection	GO:0043368	4.00E-01	4.88E-01	Ly9,17085:up
regulation of extracellular matrix organization	GO:1903053	4.00E-01	4.88E-01	Tgfb2,21808:down
regulation of thymocyte aggregation	GO:2000398	4.00E-01	4.88E-01	Bmp4,12159:up
synaptic vesicle endocytosis	GO:0048488	4.00E-01	4.88E-01	Cd24a,12484:down
glutamate secretion	GO:0014047	4.00E-01	4.88E-01	Il1rn,16181:up
inner ear receptor stereocilium organization	GO:0060122	4.00E-01	4.88E-01	Cthrc1,68588:down
dsRNA fragmentation	GO:0031050	4.00E-01	4.88E-01	Egfr,13649:down
regulation of cell aging	GO:0090342	4.00E-01	4.88E-01	Bmpr1a,12166:down
acylglycerol catabolic process	GO:0046464	4.00E-01	4.88E-01	Lpl,16956:up
cGMP biosynthetic process	GO:0006182	4.00E-01	4.88E-01	Htr2b,15559:down
cortical actin cytoskeleton organization	GO:0030866	4.00E-01	4.88E-01	Tnf,21926:up
regulation of T cell differentiation in thymus	GO:0033081	4.00E-01	4.88E-01	Bmp4,12159:up
regulation of meiotic nuclear division	GO:0040020	4.00E-01	4.88E-01	Wnt5a,22418:down
regulation of cation channel activity	GO:2001257	4.01E-01	4.89E-01	S100a1,20193:up|Bmp4,12159:up
single-organism cellular localization	GO:1902580	4.02E-01	4.90E-01	Ccl2,20296:up|Lrrc15,74488:down|Gas6,14456:up|Tnf,21926:up|Akr1c18,105349:up|Cd24a,12484:down|Egfr,13649:down|Hmox1,15368:up|C1qtnf3,81799:down|Bmpr1a,12166:down|Ptgs2,19225:down|Grem1,23892:up|Adipoq,11450:up|Bmp4,12159:up|Igf1,16000:up|Ceacam1,26365:up|Sfrp2,20319:up|Tgfb2,21808:down|Fgfr2,14183:up|Syt13,80976:up
response to radiation	GO:0009314	4.03E-01	4.91E-01	Pparg,19016:up|Sfrp2,20319:up|Egfr,13649:down|Sfrp1,20377:up|Mme,17380:up|Nabp1,109019:down|Col3a1,12825:down
phospholipid biosynthetic process	GO:0008654	4.04E-01	4.92E-01	Htr2b,15559:down|Fabp5,16592:up|Htr2a,15558:down
cellular response to hypoxia	GO:0071456	4.07E-01	4.96E-01	Vegfa,22339:down|Epas1,13819:up
oligodendrocyte differentiation	GO:0048709	4.07E-01	4.96E-01	Pparg,19016:up|Bmp4,12159:up
positive regulation of transporter activity	GO:0032411	4.07E-01	4.96E-01	S100a1,20193:up|Bmp4,12159:up
embryonic heart tube development	GO:0035050	4.07E-01	4.96E-01	Wnt5a,22418:down|Aldh1a2,19378:up
fatty acid biosynthetic process	GO:0006633	4.09E-01	4.98E-01	Ggt5,23887:up|Lpl,16956:up|Ptgs2,19225:down
aging	GO:0007568	4.09E-01	4.98E-01	Ncam1,17967:down|Prelp,116847:up|Bmpr1a,12166:down
insulin secretion	GO:0030073	4.10E-01	4.99E-01	Tnf,21926:up|Glp1r,14652:down|Sfrp1,20377:up|Il1rn,16181:up
cytoplasmic sequestering of protein	GO:0051220	4.11E-01	4.99E-01	Kdelr3,105785:down
response to food	GO:0032094	4.11E-01	4.99E-01	Pparg,19016:up
positive regulation of substrate adhesion-dependent cell spreading	GO:1900026	4.11E-01	4.99E-01	Has2,15117:down
positive regulation of NIK/NF-kappaB signaling	GO:1901224	4.11E-01	4.99E-01	Tnf,21926:up
mating behavior	GO:0007617	4.11E-01	4.99E-01	Serpine2,20720:up

regulation of protein export from nucleus	GO:0046825	4.11E-01	4.99E-01	Gas6,14456:up
cell differentiation involved in embryonic placenta development	GO:0060706	4.11E-01	4.99E-01	Gjb5,14622:down
regulation of the force of heart contraction	GO:0002026	4.11E-01	4.99E-01	Adm,11535:up
NADH metabolic process	GO:0006734	4.11E-01	4.99E-01	Cbr2,12409:up
negative regulation of adaptive immune response	GO:0002820	4.11E-01	4.99E-01	Il1rl1,17082:down
negative regulation of glial cell differentiation	GO:0045686	4.11E-01	4.99E-01	Bmp4,12159:up
sodium ion transmembrane transport	GO:0035725	4.13E-01	5.01E-01	Fxyd1,56188:up|Fxyd2,11936:up
response to calcium ion	GO:0051592	4.13E-01	5.01E-01	Akr1c18,105349:up|Egfr,13649:down
negative regulation of protein binding	GO:0032091	4.13E-01	5.01E-01	Slpi,20568:up|Adipoq,11450:up
response to purine-containing compound	GO:0014074	4.13E-01	5.01E-01	Cdo1,12583:up|Adipoq,11450:up
actin filament-based process	GO:0030029	4.14E-01	5.02E-01	Fam101b,76566:down|Tnf,21926:up|Fxyd1,56188:up|Trf,22041:up|Sfrp1,20377:up|Ptk7,71461:down|Dpysl3,22240:down|Stc1,20855:up|Gpm6b,14758:up|Shroom4,208431:down|Tgfb2,21808:down
establishment of protein localization to plasma membrane	GO:0090002	4.18E-01	5.07E-01	Tnf,21926:up|Gas6,14456:up|Lrrc15,74488:down
sensory perception of sound	GO:0007605	4.18E-01	5.07E-01	Fgfr1,14182:down|Col11a1,12814:down|Mcoln3,171166:up
response to nutrient levels	GO:0031667	4.19E-01	5.08E-01	Pparg,19016:up|Gas6,14456:up|Lmcd1,30937:up|Hmox1,15368:up|Sfrp4,20379:up|Vdr,22337:down|Gatm,67092:down|Plod2,26432:down|Postn,50706:down
positive regulation of nucleotide biosynthetic process	GO:0030810	4.20E-01	5.08E-01	Adm,11535:up|Ptgir,19222:up
actin filament-based movement	GO:0030048	4.20E-01	5.08E-01	Fxyd1,56188:up|Stc1,20855:up
cellular response to decreased oxygen levels	GO:0036294	4.20E-01	5.08E-01	Vegfa,22339:down|Epas1,13819:up
positive regulation of purine nucleotide biosynthetic process	GO:1900373	4.20E-01	5.08E-01	Adm,11535:up|Ptgir,19222:up
positive regulation of T cell mediated immunity	GO:0002711	4.22E-01	5.09E-01	Cd24a,12484:down
sulfur amino acid metabolic process	GO:0000096	4.22E-01	5.09E-01	Cdo1,12583:up
eye photoreceptor cell development	GO:0042462	4.22E-01	5.09E-01	Vegfa,22339:down
peptidyl-proline modification	GO:0018208	4.22E-01	5.09E-01	P4ha1,18451:down
regulation of mast cell degranulation	GO:0043304	4.22E-01	5.09E-01	Hmox1,15368:up
ketone biosynthetic process	GO:0042181	4.22E-01	5.09E-01	Akr1c18,105349:up
p38MAPK cascade	GO:0038066	4.22E-01	5.09E-01	Vegfa,22339:down
positive regulation of smoothened signaling pathway	GO:0045880	4.22E-01	5.09E-01	Sfrp1,20377:up
regulation of mast cell activation involved in immune response	GO:0033006	4.22E-01	5.09E-01	Hmox1,15368:up
regulation of astrocyte differentiation	GO:0048710	4.22E-01	5.09E-01	Serpine2,20720:up
embryonic axis specification	GO:0000578	4.22E-01	5.09E-01	Wnt5a,22418:down
positive regulation of interleukin-12 production	GO:0032735	4.22E-01	5.09E-01	Tnfsf9,21950:up
mRNA stabilization	GO:0048255	4.22E-01	5.09E-01	Vegfa,22339:down
oxidoreduction coenzyme metabolic process	GO:0006733	4.23E-01	5.10E-01	Htr2a,15558:down|Cbr2,12409:up|Igf1,16000:up
glycoprotein biosynthetic process	GO:0009101	4.24E-01	5.11E-01	Chst11,58250:down|Sdf2l1,64136:up|Vegfb,22340:up|Man2a2,140481:up|Igf1,16000:up
negative regulation of intrinsic apoptotic signaling pathway	GO:2001243	4.26E-01	5.13E-01	Cxcl12,20315:up|Creb3l1,26427:down

regulation of transporter activity	GO:0032409	4.27E-01	5.14E-01	Fxyd1,56188:up|Fxyd2,11936:up|S100a1,20193:up|Bmp4,12159:up
regulation of response to DNA damage stimulus	GO:2001020	4.28E-01	5.15E-01	Ankrd1,107765:down|Egfr,13649:down|Cxcl12,20315:up
vesicle docking involved in exocytosis	GO:0006904	4.32E-01	5.19E-01	Ncam1,17967:down
regulation of viral transcription	GO:0046782	4.32E-01	5.19E-01	Ccl4,20303:up
cell cycle DNA replication	GO:0044786	4.32E-01	5.19E-01	Fgfr1,14182:down
regulation of sterol transport	GO:0032371	4.32E-01	5.19E-01	Adipoq,11450:up
lung epithelial cell differentiation	GO:0060487	4.32E-01	5.19E-01	Igf1,16000:up
regulation of intrinsic apoptotic signaling pathway in response to DNA damage	GO:1902229	4.32E-01	5.19E-01	Cxcl12,20315:up
RNA stabilization	GO:0043489	4.32E-01	5.19E-01	Vegfa,22339:down
myeloid dendritic cell activation	GO:0001773	4.32E-01	5.19E-01	Tnfsf9,21950:up
regulation of fatty acid oxidation	GO:0046320	4.32E-01	5.19E-01	Pparg,19016:up
regulation of cholesterol transport	GO:0032374	4.32E-01	5.19E-01	Adipoq,11450:up
regulation of fibroblast migration	GO:0010762	4.32E-01	5.19E-01	Fgfr1,14182:down
hindbrain development	GO:0030902	4.38E-01	5.25E-01	Aldh1a2,19378:up|Serpine2,20720:up|Igf1,16000:up
B cell proliferation	GO:0042100	4.38E-01	5.25E-01	Cd24a,12484:down|Tfrc,22042:up
pigmentation	GO:0043473	4.38E-01	5.25E-01	Ednrb,13618:up|Kitl,17311:up
positive regulation of lymphocyte differentiation	GO:0045621	4.38E-01	5.25E-01	Gas6,14456:up|Tnfsf9,21950:up
glycosyl compound metabolic process	GO:1901657	4.40E-01	5.27E-01	Akr1c18,105349:up|Htr2a,15558:down|Uck2,80914:down|Enpp1,18605:down|Ampd3,11717:up|Igf1,16000:up
G1/S transition of mitotic cell cycle	GO:0000082	4.42E-01	5.28E-01	Plk2,20620:down|Egfr,13649:down|Inhba,16323:down
regulation of dephosphorylation	GO:0035303	4.42E-01	5.28E-01	Tnf,21926:up|Tgfb2,21808:down|Npnt,114249:up
plasma lipoprotein particle organization	GO:0071827	4.43E-01	5.28E-01	Lpl,16956:up
phosphatidylcholine metabolic process	GO:0046470	4.43E-01	5.28E-01	Fabp5,16592:up
negative regulation of T cell differentiation	GO:0045581	4.43E-01	5.28E-01	Bmp4,12159:up
phospholipid dephosphorylation	GO:0046839	4.43E-01	5.28E-01	Plpp3,67916:up
neutral amino acid transport	GO:0015804	4.43E-01	5.28E-01	Serinc2,230779:down
regulation of filopodium assembly	GO:0051489	4.43E-01	5.28E-01	Dpysl3,22240:down
lung cell differentiation	GO:0060479	4.43E-01	5.28E-01	Igf1,16000:up
regulation of transcription regulatory region DNA binding	GO:2000677	4.43E-01	5.28E-01	Igf1,16000:up
intestinal absorption	GO:0050892	4.43E-01	5.28E-01	Vdr,22337:down
synaptic transmission, dopaminergic	GO:0001963	4.43E-01	5.28E-01	Ptgs2,19225:down
negative regulation of insulin receptor signaling pathway	GO:0046627	4.43E-01	5.28E-01	Enpp1,18605:down
regulation of centrosome duplication	GO:0010824	4.43E-01	5.28E-01	Plk2,20620:down
serine family amino acid metabolic process	GO:0009069	4.43E-01	5.28E-01	Cdo1,12583:up
phagocytosis, engulfment	GO:0006911	4.43E-01	5.28E-01	Pparg,19016:up
protein localization to cytoskeleton	GO:0044380	4.43E-01	5.28E-01	Htr2a,15558:down
cell projection morphogenesis	GO:0048858	4.43E-01	5.28E-01	Flrt2,399558:down|Lama2,16773:down|Egfr,13649:down|Plxnd1,67784:up|Wnt5a,22418:down|Sema7a,20361:down|Tnfrsf12a,27279:down|Crabp2,12904:down|Ret,19713:up|Cxcl12,20315:up|Postn,50706:down|Vegfa,22339:down|Tgfb2,21808:down|Fgfr2,14183:up

<table>
<tr><td>regulation of gene expression</td><td>GO:0010468</td><td>4.47E-01</td><td>5.33E-01</td><td>Fstl3,83554:down|Ice1,218333:down|Tfec,21426:up|Sfrp4,20379:up|Aldh1a2,19378:up|C3,12266:up|Ednrb,13618:up|Epas1,13819:up|Ankrd1,107765:down|Htra3,78558:down|Uchl5,56207:up|Lyl1,17095:up|Sdpr,20324:down|Loxl2,94352:down|Loxl3,16950:down|Trf,22041:up|Wnt5a,22418:down|Bmpr1a,12166:down|Ccl4,20303:up|Adipoq,11450:up|Bmp4,12159:up|Igf1,16000:up|Tnc,21923:down|Ret,19713:up|Tgfb2,21808:down|Plpp3,67916:up|Creb3l1,26427:down|Fgfr2,14183:up|Prl2c2,18811:up|Efemp1,216616:up|Inhba,16323:down|Rasl11a,68895:up|Bnc2,242509:down|Ccl3,20302:up|Vdr,22337:down|Ctla2a,13024:down|Fabp4,11770:up|Vegfa,22339:down|Serpine2,20720:up|Igf2,16002:up|Slc40a1,53945:up|Pparg,19016:up|Gas6,14456:up|Tnf,21926:up|Glp1r,14652:down|Lmcd1,30937:up|Fgfr1,14182:down|Egfr,13649:down|Hmox1,15368:up|Sfrp1,20377:up|Sulf1,240725:down|Tgfbr3,21814:up|Grem1,23892:up|Phf11d,219132:up|Sfrp2,20319:up|Tbx15,21384:down|Serping1,12258:up|Npnt,114249:up|S100a1,20193:up</td></tr>
<tr><td>response to unfolded protein</td><td>GO:0006986</td><td>4.50E-01</td><td>5.36E-01</td><td>Sdf2l1,64136:up|Creb3l1,26427:down</td></tr>
<tr><td>regulation of hematopoietic progenitor cell differentiation</td><td>GO:1901532</td><td>4.53E-01</td><td>5.38E-01</td><td>Vegfa,22339:down</td></tr>
<tr><td>energy homeostasis</td><td>GO:0097009</td><td>4.53E-01</td><td>5.38E-01</td><td>Ampd3,11717:up</td></tr>
<tr><td>cellular response to glucocorticoid stimulus</td><td>GO:0071385</td><td>4.53E-01</td><td>5.38E-01</td><td>Bmp4,12159:up</td></tr>
<tr><td>embryonic eye morphogenesis</td><td>GO:0048048</td><td>4.53E-01</td><td>5.38E-01</td><td>Aldh1a3,56847:down</td></tr>
<tr><td>protein localization to endoplasmic reticulum</td><td>GO:0070972</td><td>4.53E-01</td><td>5.38E-01</td><td>Kdelr3,105785:down</td></tr>
<tr><td>negative regulation of transcription factor import into nucleus</td><td>GO:0042992</td><td>4.53E-01</td><td>5.38E-01</td><td>C1qtnf3,81799:down</td></tr>
<tr><td>homeostasis of number of cells within a tissue</td><td>GO:0048873</td><td>4.53E-01</td><td>5.38E-01</td><td>Vegfa,22339:down</td></tr>
<tr><td>regulation of tyrosine phosphorylation of Stat3 protein</td><td>GO:0042516</td><td>4.53E-01</td><td>5.38E-01</td><td>Crlf1,12931:down</td></tr>
<tr><td>negative regulation of lymphocyte apoptotic process</td><td>GO:0070229</td><td>4.53E-01</td><td>5.38E-01</td><td>Bmp4,12159:up</td></tr>
<tr><td>ncRNA transcription</td><td>GO:0098781</td><td>4.53E-01</td><td>5.38E-01</td><td>Ice1,218333:down</td></tr>
<tr><td>membrane hyperpolarization</td><td>GO:0060081</td><td>4.53E-01</td><td>5.38E-01</td><td>Adipoq,11450:up</td></tr>
<tr><td>semaphorin-plexin signaling pathway</td><td>GO:0071526</td><td>4.53E-01</td><td>5.38E-01</td><td>Plxnd1,67784:up</td></tr>
<tr><td>cerebellar cortex morphogenesis</td><td>GO:0021696</td><td>4.53E-01</td><td>5.38E-01</td><td>Serpine2,20720:up</td></tr>
<tr><td>ribonucleoside monophosphate metabolic process</td><td>GO:0009161</td><td>4.55E-01</td><td>5.40E-01</td><td>Htr2a,15558:down|Uck2,80914:down|Ampd3,11717:up|Igf1,16000:up</td></tr>
<tr><td>regulation of DNA binding</td><td>GO:0051101</td><td>4.56E-01</td><td>5.42E-01</td><td>Hmox1,15368:up|Igf1,16000:up</td></tr>
<tr><td>triglyceride metabolic process</td><td>GO:0006641</td><td>4.56E-01</td><td>5.42E-01</td><td>Lpl,16956:up|C3,12266:up</td></tr>
<tr><td>cellular response to oxygen levels</td><td>GO:0071453</td><td>4.56E-01</td><td>5.42E-01</td><td>Vegfa,22339:down|Epas1,13819:up</td></tr>
<tr><td>lymphocyte activation involved in immune response</td><td>GO:0002285</td><td>4.57E-01</td><td>5.42E-01</td><td>Ly9,17085:up|Tfrc,22042:up|Clec4e,56619:up</td></tr>
<tr><td>cellular response to nutrient levels</td><td>GO:0031669</td><td>4.59E-01</td><td>5.44E-01</td><td>Vdr,22337:down|Gas6,14456:up|Lmcd1,30937:up|Plod2,26432:down|Hmox1,15368:up|Postn,50706:down|Sfrp4,20379:up</td></tr>
<tr><td>learning</td><td>GO:0007612</td><td>4.61E-01</td><td>5.46E-01</td><td>Glp1r,14652:down|Ptgs2,19225:down|Prkar2b,19088:up</td></tr>
<tr><td>stem cell maintenance</td><td>GO:0019827</td><td>4.61E-01</td><td>5.46E-01</td><td>Fgfr1,14182:down|Sfrp1,20377:up|Bmpr1a,12166:down</td></tr>
<tr><td>regulation of cellular macromolecule biosynthetic process</td><td>GO:2000112</td><td>4.61E-01</td><td>5.46E-01</td><td>Fstl3,83554:down|Ice1,218333:down|Tfec,21426:up|Sfrp4,20379:up|Pdgfc,54635:down|Ednrb,13618:up|Epas1,13819:up|Ankrd1,107765:down|Htra3,78558:down|Uchl5,56207:up|Lyl1,17095:up|Sdpr,20324:down|Loxl2,94352:down|Loxl3,16950:down|Trf,22041:up|Wnt5a,22418:down|Bmpr1a,12166:down|Adipoq,11450:up|Bmp4,12159:up|Igf1,16000:up|Ret,19713:up|Plpp3,67916:up|Creb3l1,26427:down|Fgfr2,14183:up|Kitl,17311:up|Prl2c2,18811:up|Efemp1,216616:up|Inhba,16323:down|Rasl11a,68895:up|Bnc2,242509:down|Ccl3,20302:up|Vdr,22337:down|Fabp4,11770:up|Vegfa,22339:down|Igf2,16002:up|Slc40a1,53945:up|Pparg,19016:up|Gas6,14456:up|Tnf,21926:up|Glp1r,14652:down|Lmcd1,30937:up|Fgfr1,14182:down|Egfr,13649:down|Hmox1,15368:up|Sfrp1,20377:up|Sulf1,240725:down|Tgfbr3,21814:up|Grem1,23892:up|Phf11d,219132:up|Sfrp2,20319:up|Tbx15,21384:down|Npnt,114249:up|Enpp1,18605:down|S100a1,20193:up</td></tr>
<tr><td>platelet activation</td><td>GO:0030168</td><td>4.63E-01</td><td>5.46E-01</td><td>Ceacam1,26365:up|Serpine2,20720:up</td></tr>
<tr><td>regulation of lipid transport</td><td>GO:0032368</td><td>4.63E-01</td><td>5.46E-01</td><td>Fam132b,227358:down|Adipoq,11450:up</td></tr>
<tr><td>digestive system process</td><td>GO:0022600</td><td>4.63E-01</td><td>5.46E-01</td><td>Vdr,22337:down|Mmp13,17386:up</td></tr>
<tr><td>positive regulation of embryonic development</td><td>GO:0040019</td><td>4.63E-01</td><td>5.46E-01</td><td>Igf1,16000:up</td></tr>
</table>

myotube cell development	GO:0014904	4.63E-01	5.46E-01	Igf1,16000:up
bicellular tight junction assembly	GO:0070830	4.63E-01	5.46E-01	Tnf,21926:up
regulation of protein deacetylation	GO:0090311	4.63E-01	5.46E-01	Vegfa,22339:down
positive regulation of epithelial to mesenchymal transition	GO:0010718	4.63E-01	5.46E-01	Tgfb2,21808:down
protein-lipid complex subunit organization	GO:0071825	4.63E-01	5.46E-01	Lpl,16956:up
viral transcription	GO:0019083	4.63E-01	5.46E-01	Ccl4,20303:up
snRNA metabolic process	GO:0016073	4.63E-01	5.46E-01	Ice1,218333:down
programmed necrotic cell death	GO:0097300	4.63E-01	5.46E-01	Tnf,21926:up
cellular response to ammonium ion	GO:0071242	4.63E-01	5.46E-01	Htr2b,15559:down
regulation of peptidyl-threonine phosphorylation	GO:0010799	4.63E-01	5.46E-01	Wnt5a,22418:down
negative regulation of response to endoplasmic reticulum stress	GO:1903573	4.63E-01	5.46E-01	Creb3l1,26427:down
protein targeting to membrane	GO:0006612	4.68E-01	5.53E-01	Gas6,14456:up|Ccl2,20296:up
synaptic transmission, cholinergic	GO:0007271	4.72E-01	5.56E-01	Lama2,16773:down
tyrosine phosphorylation of Stat3 protein	GO:0042503	4.72E-01	5.56E-01	Crlf1,12931:down
membrane protein ectodomain proteolysis	GO:0006509	4.72E-01	5.56E-01	Tnf,21926:up
synaptic vesicle recycling	GO:0036465	4.72E-01	5.56E-01	Cd24a,12484:down
xenobiotic metabolic process	GO:0006805	4.72E-01	5.56E-01	Gstt1,14871:up
cortical cytoskeleton organization	GO:0030865	4.72E-01	5.56E-01	Tnf,21926:up
regulation of cell cycle G1/S phase transition	GO:1902806	4.74E-01	5.58E-01	Egfr,13649:down|Susd2,71733:up
regulation of cAMP biosynthetic process	GO:0030817	4.74E-01	5.58E-01	Adm,11535:up|Ptgir,19222:up
organophosphate biosynthetic process	GO:0090407	4.76E-01	5.60E-01	Htr2b,15559:down|Adm,11535:up|Fabp5,16592:up|Ampd3,11717:up|Igf1,16000:up|Pdxk,216134:up|Htr2a,15558:down|Ptgir,19222:up|Uck2,80914:down
nucleoside monophosphate metabolic process	GO:0009123	4.78E-01	5.63E-01	Htr2a,15558:down|Uck2,80914:down|Ampd3,11717:up|Igf1,16000:up
intracellular steroid hormone receptor signaling pathway	GO:0030518	4.80E-01	5.65E-01	Sfrp1,20377:up|Igf1,16000:up
insulin receptor signaling pathway	GO:0008286	4.80E-01	5.65E-01	Enpp1,18605:down|Igf2,16002:up
nucleobase metabolic process	GO:0009112	4.82E-01	5.65E-01	Uck2,80914:down
negative regulation of DNA binding	GO:0043392	4.82E-01	5.65E-01	Hmox1,15368:up
positive regulation of DNA repair	GO:0045739	4.82E-01	5.65E-01	Egfr,13649:down
negative regulation of endothelial cell migration	GO:0010596	4.82E-01	5.65E-01	Stc1,20855:up
positive regulation of leukocyte mediated cytotoxicity	GO:0001912	4.82E-01	5.65E-01	Ccl2,20296:up
carbohydrate derivative transport	GO:1901264	4.82E-01	5.65E-01	Slc29a1,63959:up
establishment of endothelial barrier	GO:0061028	4.82E-01	5.65E-01	Tnf,21926:up
regulation of natural killer cell activation	GO:0032814	4.82E-01	5.65E-01	Gas6,14456:up
cell part morphogenesis	GO:0032990	4.84E-01	5.68E-01	Flrt2,399558:down|Lama2,16773:down|Egfr,13649:down|Plxnd1,67784:up|Wnt5a,22418:down|Sema7a,20361:down|Tnfrsf12a,27279:down|Crabp2,12904:down|Ret,19713:up|Cxcl12,20315:up|Postn,50706:down|Vegfa,22339:down|Tgfb2,21808:down|Fgfr2,14183:up
ribonucleoside biosynthetic process	GO:0042455	4.85E-01	5.68E-01	Htr2a,15558:down|Uck2,80914:down|Igf1,16000:up

regulation of mitochondrion degradation	GO:1903146	4.89E-01	5.73E-01	Lmcd1,30937:up|Plod2,26432:down|Sfrp4,20379:up
protein localization to plasma membrane	GO:0072659	4.90E-01	5.74E-01	Tnf,21926:up|Gas6,14456:up|Lrrc15,74488:down|Adipoq,11450:up
nitrogen compound metabolic process	GO:0006807	4.90E-01	5.74E-01	Fstl3,83554:down|Adm,11535:up|Tfec,21426:up|Sfrp4,20379:up|Enpp5,83965:up|Ankrd1,107765:down|Lyl1,17095:up|Gstt1,14871:up|Blvrb,233016:up|Igfbp3,16009:up|Ggt5,23887:up|Loxl2,94352:down|Loxl3,16950:down|Trf,22041:up|Mme,17380:up|Wnt5a,22418:down|Maob,109731:up|Adipoq,11450:up|Bmp4,12159:up|Hal,15109:up|Igf1,16000:up|Tgfb2,21808:down|Ptgir,19222:up|Fgfr2,14183:up|Htr2b,15559:down|Prl2c2,18811:up|Efemp1,216616:up|Vdr,22337:down|Fabp4,11770:up|Nabp1,109019:down|Htr2a,15558:down|Oas3,246727:up|Slc40a1,53945:up|Gas6,14456:up|Egfr,13649:down|Sfrp1,20377:up|Tgfbr3,21814:up|Tfrc,22042:up|Serinc2,230779:down|Sfrp2,20319:up|Gstm1,14862:up|Npnt,114249:up|S100a1,20193:up|Ice1,218333:down|Pdgfc,54635:down|Ednrb,13618:up|Ampd3,11717:up|Mmp13,17386:up|Epas1,13819:up|Marc1,66112:up|Htra3,78558:down|Uchl5,56207:up|Ddah1,69219:down|Sdpr,20324:down|Enpp3,209558:down|Cdo1,12583:up|Mamdc2,71738:down|Ptx3,19288:up|Akr1c18,105349:up|Fabp5,16592:up|Bmpr1a,12166:down|Ccl4,20303:up|Chst11,58250:down|Gatm,67092:down|Ret,19713:up|Pdxk,216134:up|Plpp3,67916:up|Creb3l1,26427:down|Kitl,17311:up|P4ha1,18451:down|Inhba,16323:down|Rasl11a,68895:up|Bnc2,242509:down|Ccl3,20302:up|Vegfa,22339:down|Gfpt2,14584:down|Cbr2,12409:up|Igf2,16002:up|Anpep,16790:up|Pparg,19016:up|Tnf,21926:up|Lmcd1,30937:up|Glp1r,14652:down|Fgfr1,14182:down|Hmox1,15368:up|Has2,15117:down|Sulf1,240725:down|Ptgs2,19225:down|Grem1,23892:up|Phf11d,219132:up|Tbx15,21384:down|Uck2,80914:down|Enpp1,18605:down
cellular response to calcium ion	GO:0071277	4.91E-01	5.74E-01	Akr1c18,105349:up
glycerolipid catabolic process	GO:0046503	4.91E-01	5.74E-01	Lpl,16956:up
positive regulation of multicellular organism growth	GO:0040018	4.91E-01	5.74E-01	Igf2,16002:up
cholesterol efflux	GO:0033344	4.91E-01	5.74E-01	Adipoq,11450:up
positive regulation of cyclin-dependent protein serine/threonine kinase activity	GO:0045737	4.91E-01	5.74E-01	Egfr,13649:down
negative regulation of epithelial cell differentiation	GO:0030857	4.91E-01	5.74E-01	Tgfb2,21808:down
negative regulation of axon extension	GO:0030517	4.91E-01	5.74E-01	Wnt5a,22418:down
regulation of glycoprotein biosynthetic process	GO:0010559	4.91E-01	5.74E-01	Igf1,16000:up
striated muscle adaptation	GO:0014888	4.91E-01	5.74E-01	Errfi1,74155:down
regulation of receptor activity	GO:0010469	4.92E-01	5.75E-01	Errfi1,74155:down|Grem1,23892:up
blastocyst development	GO:0001824	4.92E-01	5.75E-01	Tgfbr3,21814:up|Igf1,16000:up
nucleoside biosynthetic process	GO:0009163	4.94E-01	5.77E-01	Htr2a,15558:down|Uck2,80914:down|Igf1,16000:up
regulation of protein catabolic process	GO:0042176	4.94E-01	5.77E-01	Uchl5,56207:up|Tnf,21926:up|Plk2,20620:down|Egfr,13649:down|Wnt5a,22418:down|Serpine2,20720:up
ATP biosynthetic process	GO:0006754	4.98E-01	5.81E-01	Htr2a,15558:down|Igf1,16000:up
mitotic cell cycle checkpoint	GO:0007093	4.98E-01	5.81E-01	Plk2,20620:down|Nabp1,109019:down
cellular response to glucose stimulus	GO:0071333	4.98E-01	5.81E-01	Gas6,14456:up|Igf1,16000:up
negative regulation of endocytosis	GO:0045806	5.01E-01	5.83E-01	Adipoq,11450:up
positive regulation of B cell proliferation	GO:0030890	5.01E-01	5.83E-01	Tfrc,22042:up
photoreceptor cell development	GO:0042461	5.01E-01	5.83E-01	Vegfa,22339:down
positive regulation of cyclin-dependent protein kinase activity	GO:1904031	5.01E-01	5.83E-01	Egfr,13649:down
response to exogenous dsRNA	GO:0043330	5.01E-01	5.83E-01	Ifit1,15957:up
negative regulation of Ras protein signal transduction	GO:0046580	5.01E-01	5.83E-01	Tgfb2,21808:down
regulation of CD4-positive, alpha-beta T cell activation	GO:2000514	5.01E-01	5.83E-01	Ceacam1,26365:up
reproductive behavior	GO:0019098	5.01E-01	5.83E-01	Serpine2,20720:up
multi-organism reproductive behavior	GO:0044705	5.01E-01	5.83E-01	Serpine2,20720:up

response to pH	GO:0009268	5.01E-01	5.83E-01	Hvcn1,74096:up
positive regulation of response to biotic stimulus	GO:0002833	5.01E-01	5.83E-01	Cd24a,12484:down
regulation of granulocyte chemotaxis	GO:0071622	5.01E-01	5.83E-01	Ccl2,20296:up
regulation of digestive system process	GO:0044058	5.01E-01	5.83E-01	Mmp13,17386:up
glycosyl compound biosynthetic process	GO:1901659	5.03E-01	5.85E-01	Htr2a,15558:down|Uck2,80914:down|Igf1,16000:up
protein folding	GO:0006457	5.03E-01	5.85E-01	Pdia5,72599:down|Fkbp10,14230:down|Fkbp14,231997:down
regulation of insulin secretion	GO:0050796	5.07E-01	5.90E-01	Tnf,21926:up|Glp1r,14652:down|Sfrp1,20377:up
cellular response to monosaccharide stimulus	GO:0071326	5.09E-01	5.91E-01	Gas6,14456:up|Igf1,16000:up
cellular response to hexose stimulus	GO:0071331	5.09E-01	5.91E-01	Gas6,14456:up|Igf1,16000:up
fatty acid oxidation	GO:0019395	5.09E-01	5.91E-01	Pparg,19016:up|Adipoq,11450:up
regulation of erythrocyte differentiation	GO:0045646	5.10E-01	5.91E-01	Inhba,16323:down
regulation of phosphoprotein phosphatase activity	GO:0043666	5.10E-01	5.91E-01	Tnf,21926:up
negative regulation of small GTPase mediated signal transduction	GO:0051058	5.10E-01	5.91E-01	Tgfb2,21808:down
negative regulation of mitochondrion organization	GO:0010823	5.10E-01	5.91E-01	Igf1,16000:up
positive regulation of sodium ion transport	GO:0010765	5.10E-01	5.91E-01	Fxyd1,56188:up
cellular response to xenobiotic stimulus	GO:0071466	5.10E-01	5.91E-01	Gstt1,14871:up
negative regulation of cell cycle G1/S phase transition	GO:1902807	5.10E-01	5.91E-01	Susd2,71733:up
negative regulation of response to extracellular stimulus	GO:0032105	5.10E-01	5.91E-01	Hmox1,15368:up
negative regulation of response to nutrient levels	GO:0032108	5.10E-01	5.91E-01	Hmox1,15368:up
adult behavior	GO:0030534	5.12E-01	5.93E-01	Cxcl12,20315:up|Htr2a,15558:down|Sncg,20618:up
B cell activation	GO:0042113	5.13E-01	5.95E-01	Cd24a,12484:down|Lyl1,17095:up|Sfrp1,20377:up|Tfrc,22042:up
negative regulation of cellular catabolic process	GO:0031330	5.16E-01	5.98E-01	Htr2b,15559:down|Hp,15439:up|Hmox1,15368:up
negative regulation of cell cycle process	GO:0010948	5.16E-01	5.98E-01	Susd2,71733:up|Fgfr2,14183:up|Bmp4,12159:up
cellular protein localization	GO:0034613	5.17E-01	5.98E-01	Ice1,218333:down|Ccl2,20296:up|Gpm6b,14758:up|Htr2a,15558:down|Lrrc15,74488:down|Kdelr3,105785:down|Gas6,14456:up|Tnf,21926:up|Akr1c18,105349:up|Cd24a,12484:down|Egfr,13649:down|Sec23a,20334:down|C1qtnf3,81799:down|Wnt5a,22418:down|Cd4,12504:up|Bmpr1a,12166:down|Ptgs2,19225:down|Grem1,23892:up|Adipoq,11450:up|Bmp4,12159:up|Igf1,16000:up|Ceacam1,26365:up|Sfrp2,20319:up|Tgfb2,21808:down
regulation of mast cell activation	GO:0033003	5.18E-01	5.98E-01	Hmox1,15368:up
regulation of meiotic cell cycle	GO:0051445	5.18E-01	5.98E-01	Wnt5a,22418:down
heterophilic cell-cell adhesion via plasma membrane cell adhesion molecules	GO:0007157	5.18E-01	5.98E-01	Cd24a,12484:down
regulation of gene silencing	GO:0060968	5.18E-01	5.98E-01	Egfr,13649:down
fibroblast migration	GO:0010761	5.18E-01	5.98E-01	Fgfr1,14182:down
regulation of centrosome cycle	GO:0046605	5.18E-01	5.98E-01	Plk2,20620:down
cell fate determination	GO:0001709	5.18E-01	5.98E-01	Bmp4,12159:up
regulation of leukocyte degranulation	GO:0043300	5.18E-01	5.98E-01	Hmox1,15368:up

negative regulation of DNA replication	GO:0008156	5.18E-01	5.98E-01	Pparg,19016:up
regulation of NIK/NF-kappaB signaling	GO:1901222	5.18E-01	5.98E-01	Tnf,21926:up
clathrin-mediated endocytosis	GO:0072583	5.18E-01	5.98E-01	Cd24a,12484:down
spleen development	GO:0048536	5.18E-01	5.98E-01	Slc40a1,53945:up
regulation of release of cytochrome c from mitochondria	GO:0090199	5.18E-01	5.98E-01	Igf1,16000:up
arachidonic acid metabolic process	GO:0019369	5.18E-01	5.98E-01	Ptgs2,19225:down
positive regulation of macroautophagy	GO:0016239	5.18E-01	5.98E-01	Hmox1,15368:up
oocyte development	GO:0048599	5.18E-01	5.98E-01	Igf1,16000:up
regulation of triglyceride metabolic process	GO:0090207	5.18E-01	5.98E-01	C3,12266:up
negative regulation of gliogenesis	GO:0014014	5.18E-01	5.98E-01	Bmp4,12159:up
acylglycerol metabolic process	GO:0006639	5.20E-01	6.00E-01	Lpl,16956:up|C3,12266:up
adult locomotory behavior	GO:0008344	5.20E-01	6.00E-01	Cxcl12,20315:up|Sncg,20618:up
response to topologically incorrect protein	GO:0035966	5.20E-01	6.00E-01	Sdf2l1,64136:up|Creb3l1,26427:down
cAMP biosynthetic process	GO:0006171	5.20E-01	6.00E-01	Adm,11535:up|Ptgir,19222:up
lipid oxidation	GO:0034440	5.20E-01	6.00E-01	Pparg,19016:up|Adipoq,11450:up
neurotransmitter transport	GO:0006836	5.21E-01	6.00E-01	Gpm6b,14758:up|Syt13,80976:up|Sncg,20618:up
positive regulation of innate immune response	GO:0045089	5.21E-01	6.00E-01	Tnf,21926:up|Wnt5a,22418:down|Mmp2,17390:down
ribonucleoside metabolic process	GO:0009119	5.21E-01	6.00E-01	Htr2a,15558:down|Uck2,80914:down|Enpp1,18605:down|Ampd3,11717:up|Igf1,16000:up
single-organism intracellular transport	GO:1902582	5.22E-01	6.01E-01	Htr2b,15559:down|Ccl2,20296:up|Htr2a,15558:down|Gas6,14456:up|Tnf,21926:up|Akr1c18,105349:up|Glp1r,14652:down|Cd24a,12484:down|Egfr,13649:down|Fgfr1,14182:down|Sec23a,20334:down|Hmox1,15368:up|C1qtnf3,81799:down|Cd4,12504:up|Bmpr1a,12166:down|Ptgs2,19225:down|Grem1,23892:up|Bmp4,12159:up|Igf1,16000:up|Ceacam1,26365:up|Sfrp2,20319:up|Tgfb2,21808:down
apoptotic mitochondrial changes	GO:0008637	5.26E-01	6.06E-01	Cd24a,12484:down|Igf1,16000:up
eye photoreceptor cell differentiation	GO:0001754	5.27E-01	6.06E-01	Vegfa,22339:down
pigment biosynthetic process	GO:0046148	5.27E-01	6.06E-01	Wnt5a,22418:down
protein O-linked glycosylation	GO:0006493	5.27E-01	6.06E-01	Vegfb,22340:up
negative regulation of response to oxidative stress	GO:1902883	5.27E-01	6.06E-01	Hp,15439:up
genitalia development	GO:0048806	5.27E-01	6.06E-01	Wnt5a,22418:down
negative regulation of cellular response to oxidative stress	GO:1900408	5.27E-01	6.06E-01	Hp,15439:up
neutral lipid metabolic process	GO:0006638	5.32E-01	6.11E-01	Lpl,16956:up|C3,12266:up
negative regulation of transcription from RNA polymerase II promoter	GO:0000122	5.32E-01	6.11E-01	Tnf,21926:up|Pparg,19016:up|Lmcd1,30937:up|Fgfr1,14182:down|Ednrb,13618:up|Epas1,13819:up|Bmp4,12159:up|Ankrd1,107765:down|Vdr,22337:down|Vegfa,22339:down|Fgfr2,14183:up|S100a1,20193:up
cellular macromolecule localization	GO:0070727	5.34E-01	6.13E-01	Ice1,218333:down|Ccl2,20296:up|Gpm6b,14758:up|Htr2a,15558:down|Lrrc15,74488:down|Kdelr3,105785:down|Gas6,14456:up|Tnf,21926:up|Akr1c18,105349:up|Cd24a,12484:down|Egfr,13649:down|Sec23a,20334:down|C1qtnf3,81799:down|Wnt5a,22418:down|Cd4,12504:up|Bmpr1a,12166:down|Ptgs2,19225:down|Grem1,23892:up|Adipoq,11450:up|Bmp4,12159:up|Igf1,16000:up|Ceacam1,26365:up|Sfrp2,20319:up|Tgfb2,21808:down
necrotic cell death	GO:0070265	5.36E-01	6.13E-01	Tnf,21926:up
isotype switching	GO:0045190	5.36E-01	6.13E-01	Tfrc,22042:up
regulation of action potential	GO:0098900	5.36E-01	6.13E-01	Tnf,21926:up
negative regulation of cell-substrate adhesion	GO:0010812	5.36E-01	6.13E-01	Postn,50706:down

cerebellum morphogenesis	GO:0021587	5.36E-01	6.13E-01	Serpine2,20720:up
somatic diversification of immunoglobulins involved in immune response	GO:0002208	5.36E-01	6.13E-01	Tfrc,22042:up
cellular senescence	GO:0090398	5.36E-01	6.13E-01	Bmpr1a,12166:down
apical junction assembly	GO:0043297	5.36E-01	6.13E-01	Tnf,21926:up
negative regulation of striated muscle tissue development	GO:0045843	5.36E-01	6.13E-01	Bmp4,12159:up
positive regulation of amine transport	GO:0051954	5.36E-01	6.13E-01	Cxcl12,20315:up
somatic recombination of immunoglobulin genes involved in immune response	GO:0002204	5.36E-01	6.13E-01	Tfrc,22042:up
regulation of interleukin-12 production	GO:0032655	5.36E-01	6.13E-01	Tnfsf9,21950:up
filopodium assembly	GO:0046847	5.36E-01	6.13E-01	Dpysl3,22240:down
mast cell degranulation	GO:0043303	5.36E-01	6.13E-01	Hmox1,15368:up
mast cell activation involved in immune response	GO:0002279	5.36E-01	6.13E-01	Hmox1,15368:up
negative regulation of insulin secretion	GO:0046676	5.36E-01	6.13E-01	Sfrp1,20377:up
actin cytoskeleton organization	GO:0030036	5.36E-01	6.13E-01	Fam101b,76566:down|Tnf,21926:up|Trf,22041:up|Sfrp1,20377:up|Ptk7,71461:down|Dpysl3,22240:down|Gpm6b,14758:up|Shroom4,208431:down|Tgfb2,21808:down
positive regulation of peptide hormone secretion	GO:0090277	5.37E-01	6.14E-01	Glp1r,14652:down|Egfr,13649:down
DNA-dependent DNA replication	GO:0006261	5.37E-01	6.14E-01	Fgfr1,14182:down|Bmp4,12159:up
phosphatidylinositol metabolic process	GO:0046488	5.42E-01	6.20E-01	Htr2b,15559:down|Htr2a,15558:down
cellular response to carbohydrate stimulus	GO:0071322	5.42E-01	6.20E-01	Gas6,14456:up|Igf1,16000:up
locomotory behavior	GO:0007626	5.43E-01	6.21E-01	Mcoln3,171166:up|Cxcl12,20315:up|Aldh1a3,56847:down|Sncg,20618:up
viral gene expression	GO:0019080	5.44E-01	6.21E-01	Ccl4,20303:up
oocyte differentiation	GO:0009994	5.44E-01	6.21E-01	Igf1,16000:up
long-chain fatty acid transport	GO:0015909	5.44E-01	6.21E-01	Pparg,19016:up
neural tube patterning	GO:0021532	5.44E-01	6.21E-01	Bmp4,12159:up
NIK/NF-kappaB signaling	GO:0038061	5.44E-01	6.21E-01	Tnf,21926:up
mast cell mediated immunity	GO:0002448	5.44E-01	6.21E-01	Hmox1,15368:up
positive regulation of cell killing	GO:0031343	5.44E-01	6.21E-01	Ccl2,20296:up
positive regulation of DNA binding	GO:0043388	5.44E-01	6.21E-01	Igf1,16000:up
regulation of cAMP metabolic process	GO:0030814	5.48E-01	6.25E-01	Adm,11535:up|Ptgir,19222:up
multi-organism reproductive process	GO:0044703	5.49E-01	6.25E-01	Akr1c18,105349:up|Hpgd,15446:up|Inhba,16323:down|Ptgs2,19225:down|Ednrb,13618:up|Plat,18791:down|Igf1,16000:up|Bmp4,12159:up|Vdr,22337:down|Cxcl12,20315:up|Mmp2,17390:down|Serpine2,20720:up|Kitl,17311:up
multicellular organism growth	GO:0035264	5.51E-01	6.28E-01	Igf2,16002:up|Fgfr2,14183:up|Igf1,16000:up
phospholipid metabolic process	GO:0006644	5.51E-01	6.28E-01	Htr2b,15559:down|Fabp5,16592:up|Htr2a,15558:down|Plpp3,67916:up|Serinc2,230779:down
water homeostasis	GO:0030104	5.52E-01	6.28E-01	Igf1,16000:up
negative regulation of muscle organ development	GO:0048635	5.52E-01	6.28E-01	Bmp4,12159:up
pyrimidine-containing compound metabolic process	GO:0072527	5.52E-01	6.28E-01	Uck2,80914:down
negative regulation of response to DNA damage stimulus	GO:2001021	5.52E-01	6.28E-01	Cxcl12,20315:up

regulation of T cell mediated immunity	GO:0002709	5.52E-01	6.28E-01	Cd24a,12484:down
NAD metabolic process	GO:0019674	5.52E-01	6.28E-01	Cbr2,12409:up
positive regulation of dephosphorylation	GO:0035306	5.52E-01	6.28E-01	Npnt,114249:up
purine ribonucleoside triphosphate biosynthetic process	GO:0009206	5.53E-01	6.29E-01	Htr2a,15558:down|Igf1,16000:up
establishment of vesicle localization	GO:0051650	5.55E-01	6.31E-01	Cd24a,12484:down|Syt13,80976:up|Fgfr2,14183:up
purine nucleoside triphosphate biosynthetic process	GO:0009145	5.59E-01	6.35E-01	Htr2a,15558:down|Igf1,16000:up
regulation of cyclic nucleotide biosynthetic process	GO:0030802	5.59E-01	6.35E-01	Adm,11535:up|Ptgir,19222:up
positive regulation of peptide secretion	GO:0002793	5.59E-01	6.35E-01	Glp1r,14652:down|Egfr,13649:down
negative regulation of muscle tissue development	GO:1901862	5.61E-01	6.36E-01	Bmp4,12159:up
positive regulation of response to nutrient levels	GO:0032109	5.61E-01	6.36E-01	Hmox1,15368:up
interleukin-12 production	GO:0032615	5.61E-01	6.36E-01	Tnfsf9,21950:up
response to xenobiotic stimulus	GO:0009410	5.61E-01	6.36E-01	Gstt1,14871:up
T-helper cell differentiation	GO:0042093	5.61E-01	6.36E-01	Ly9,17085:up
positive regulation of response to extracellular stimulus	GO:0032106	5.61E-01	6.36E-01	Hmox1,15368:up
germ cell development	GO:0007281	5.62E-01	6.37E-01	Cxcl12,20315:up|Kitl,17311:up|Igf1,16000:up|Bmp4,12159:up
positive regulation of protein catabolic process	GO:0045732	5.64E-01	6.39E-01	Tnf,21926:up|Plk2,20620:down|Wnt5a,22418:down
cellular glucose homeostasis	GO:0001678	5.64E-01	6.39E-01	Gas6,14456:up|Igf1,16000:up
ribonucleoside triphosphate biosynthetic process	GO:0009201	5.64E-01	6.39E-01	Htr2a,15558:down|Igf1,16000:up
myelination	GO:0042552	5.64E-01	6.39E-01	Tnf,21926:up|Trf,22041:up
nucleoside metabolic process	GO:0009116	5.64E-01	6.39E-01	Htr2a,15558:down|Uck2,80914:down|Enpp1,18605:down|Ampd3,11717:up|Igf1,16000:up
regulation of interleukin-2 production	GO:0032663	5.69E-01	6.43E-01	Ceacam1,26365:up
somatic stem cell maintenance	GO:0035019	5.69E-01	6.43E-01	Sfrp1,20377:up
hindbrain morphogenesis	GO:0021575	5.69E-01	6.43E-01	Serpine2,20720:up
CD4-positive, alpha-beta T cell differentiation involved in immune response	GO:0002294	5.69E-01	6.43E-01	Ly9,17085:up
regulation of signal transduction by p53 class mediator	GO:1901796	5.69E-01	6.43E-01	Ankrd1,107765:down
maintenance of protein location in cell	GO:0032507	5.69E-01	6.44E-01	Kdelr3,105785:down|Cd4,12504:up
axon ensheathment	GO:0008366	5.74E-01	6.49E-01	Tnf,21926:up|Trf,22041:up
ensheathment of neurons	GO:0007272	5.74E-01	6.49E-01	Tnf,21926:up|Trf,22041:up
cellular response to reactive oxygen species	GO:0034614	5.74E-01	6.49E-01	Tnf,21926:up|Akr1c18,105349:up
immunoglobulin production involved in immunoglobulin mediated immune response	GO:0002381	5.76E-01	6.51E-01	Tfrc,22042:up
alpha-beta T cell activation involved in immune response	GO:0002287	5.76E-01	6.51E-01	Ly9,17085:up

<table>
<tr><td>somatic recombination of immunoglobulin gene segments</td><td>GO:0016447</td><td>5.76E-01</td><td>6.51E-01</td><td>Tfrc,22042:up</td></tr>
<tr><td>alpha-beta T cell differentiation involved in immune response</td><td>GO:0002293</td><td>5.76E-01</td><td>6.51E-01</td><td>Ly9,17085:up</td></tr>
<tr><td>membrane invagination</td><td>GO:0010324</td><td>5.76E-01</td><td>6.51E-01</td><td>Pparg,19016:up</td></tr>
<tr><td>digestion</td><td>GO:0007586</td><td>5.79E-01</td><td>6.54E-01</td><td>Vdr,22337:down|Mmp13,17386:up</td></tr>
<tr><td>establishment of protein localization to membrane</td><td>GO:0090150</td><td>5.80E-01</td><td>6.54E-01</td><td>Tnf,21926:up|Gas6,14456:up|Ccl2,20296:up|Lrrc15,74488:down</td></tr>
<tr><td>cellular lipid catabolic process</td><td>GO:0044242</td><td>5.80E-01</td><td>6.54E-01</td><td>Akr1c18,105349:up|Lpl,16956:up|Adipoq,11450:up</td></tr>
<tr><td>vesicle docking</td><td>GO:0048278</td><td>5.84E-01</td><td>6.58E-01</td><td>Ncam1,17967:down</td></tr>
<tr><td>regulation of establishment of protein localization to plasma membrane</td><td>GO:0090003</td><td>5.84E-01</td><td>6.58E-01</td><td>Lrrc15,74488:down</td></tr>
<tr><td>gene silencing by miRNA</td><td>GO:0035195</td><td>5.84E-01</td><td>6.58E-01</td><td>Egfr,13649:down</td></tr>
<tr><td>determination of left/right symmetry</td><td>GO:0007368</td><td>5.84E-01</td><td>6.58E-01</td><td>Wnt5a,22418:down|Aldh1a2,19378:up</td></tr>
<tr><td>ATP metabolic process</td><td>GO:0046034</td><td>5.84E-01</td><td>6.58E-01</td><td>Htr2a,15558:down|Ampd3,11717:up|Igf1,16000:up</td></tr>
<tr><td>macromolecule biosynthetic process</td><td>GO:0009059</td><td>5.88E-01</td><td>6.62E-01</td><td>Fstl3,83554:down|Tfec,21426:up|Sfrp4,20379:up|Ankrd1,107765:down|Serpinh1,12406:down|Lyl1,17095:up|Loxl2,94352:down|Loxl3,16950:down|Trf,22041:up|Wnt5a,22418:down|Adipoq,11450:up|Bmp4,12159:up|Igf1,16000:up|Tgfb2,21808:down|Fgfr2,14183:up|Prl2c2,18811:up|Efemp1,216616:up|Vdr,22337:down|Fabp4,11770:up|Slc40a1,53945:up|Gas6,14456:up|Egfr,13649:down|Sfrp1,20377:up|Tgfbr3,21814:up|Sfrp2,20319:up|Npnt,114249:up|S100a1,20193:up|Ice1,218333:down|Ccl2,20296:up|Ednrb,13618:up|Pdgfc,54635:down|Epas1,13819:up|Uchl5,56207:up|Htra3,78558:down|Sdpr,20324:down|Man2a2,140481:up|Bmpr1a,12166:down|Ccl4,20303:up|Chst11,58250:down|Ret,19713:up|Vegfb,22340:up|Sdf2l1,64136:up|Prg4,96875:down|Plpp3,67916:up|Creb3l1,26427:down|Kitl,17311:up|Rasl11a,68895:up|Inhba,16323:down|Bnc2,242509:down|Ccl3,20302:up|Vegfa,22339:down|Igf2,16002:up|Tnf,21926:up|Pparg,19016:up|Lmcd1,30937:up|Glp1r,14652:down|Fgfr1,14182:down|Hmox1,15368:up|Has2,15117:down|Sulf1,240725:down|Grem1,23892:up|Phf11d,219132:up|Col5a1,12831:down|Tbx15,21384:down|Enpp1,18605:down|Errfi1,74155:down</td></tr>
<tr><td>lipid modification</td><td>GO:0030258</td><td>5.88E-01</td><td>6.62E-01</td><td>Pparg,19016:up|Plpp3,67916:up|Adipoq,11450:up</td></tr>
<tr><td>intracellular transport</td><td>GO:0046907</td><td>5.90E-01</td><td>6.64E-01</td><td>Htr2b,15559:down|Ice1,218333:down|Ccl2,20296:up|Htr2a,15558:down|Gas6,14456:up|Tnf,21926:up|Glp1r,14652:down|Akr1c18,105349:up|Cd24a,12484:down|Egfr,13649:down|Fgfr1,14182:down|Sec23a,20334:down|Hmox1,15368:up|C1qtnf3,81799:down|Cd4,12504:up|Bmpr1a,12166:down|Ptgs2,19225:down|Grem1,23892:up|Adipoq,11450:up|Bmp4,12159:up|Igf1,16000:up|Ceacam1,26365:up|Sfrp2,20319:up|Tgfb2,21808:down</td></tr>
<tr><td>regulation of purine nucleotide biosynthetic process</td><td>GO:1900371</td><td>5.94E-01</td><td>6.68E-01</td><td>Adm,11535:up|Ptgir,19222:up</td></tr>
<tr><td>neurotransmitter secretion</td><td>GO:0007269</td><td>5.94E-01</td><td>6.68E-01</td><td>Syt13,80976:up|Sncg,20618:up</td></tr>
<tr><td>negative regulation of protein catabolic process</td><td>GO:0042177</td><td>5.94E-01</td><td>6.68E-01</td><td>Egfr,13649:down|Serpine2,20720:up</td></tr>
<tr><td>autophagy</td><td>GO:0006914</td><td>5.96E-01</td><td>6.70E-01</td><td>Htr2b,15559:down|Plk2,20620:down|Lmcd1,30937:up|Dram1,71712:up|Plod2,26432:down|Hmox1,15368:up|Sfrp4,20379:up</td></tr>
<tr><td>mitochondrion degradation</td><td>GO:0000422</td><td>5.97E-01</td><td>6.70E-01</td><td>Lmcd1,30937:up|Plod2,26432:down|Sfrp4,20379:up</td></tr>
<tr><td>ethanolamine-containing compound metabolic process</td><td>GO:0042439</td><td>5.99E-01</td><td>6.72E-01</td><td>Fabp5,16592:up</td></tr>
<tr><td>somatic diversification of immunoglobulins</td><td>GO:0016445</td><td>5.99E-01</td><td>6.72E-01</td><td>Tfrc,22042:up</td></tr>
<tr><td>negative regulation of blood pressure</td><td>GO:0045776</td><td>5.99E-01</td><td>6.72E-01</td><td>Adipoq,11450:up</td></tr>
<tr><td>DNA damage response, signal transduction by p53 class mediator</td><td>GO:0030330</td><td>5.99E-01</td><td>6.72E-01</td><td>Ankrd1,107765:down</td></tr>
<tr><td>regulation of nucleotide biosynthetic process</td><td>GO:0030808</td><td>5.99E-01</td><td>6.72E-01</td><td>Adm,11535:up|Ptgir,19222:up</td></tr>
<tr><td>protein stabilization</td><td>GO:0050821</td><td>6.04E-01</td><td>6.77E-01</td><td>Plpp3,67916:up|Igf1,16000:up</td></tr>
<tr><td>maintenance of protein location</td><td>GO:0045185</td><td>6.04E-01</td><td>6.77E-01</td><td>Kdelr3,105785:down|Cd4,12504:up</td></tr>
<tr><td>sodium ion transport</td><td>GO:0006814</td><td>6.04E-01</td><td>6.78E-01</td><td>Fxyd1,56188:up|Serpine2,20720:up|Fxyd2,11936:up</td></tr>
<tr><td>negative regulation of cation transmembrane transport</td><td>GO:1904063</td><td>6.06E-01</td><td>6.78E-01</td><td>Tgfb2,21808:down</td></tr>
</table>

negative regulation of reactive oxygen species metabolic process	GO:2000378	6.06E-01	6.78E-01	Hp,15439:up
multi-organism metabolic process	GO:0044033	6.06E-01	6.78E-01	Ccl4,20303:up
posttranscriptional gene silencing	GO:0016441	6.06E-01	6.78E-01	Egfr,13649:down
posttranscriptional gene silencing by RNA	GO:0035194	6.06E-01	6.78E-01	Egfr,13649:down
interleukin-2 production	GO:0032623	6.06E-01	6.78E-01	Ceacam1,26365:up
vesicle localization	GO:0051648	6.08E-01	6.81E-01	Cd24a,12484:down|Syt13,80976:up|Fgfr2,14183:up
response to UV	GO:0009411	6.09E-01	6.81E-01	Egfr,13649:down|Mme,17380:up
potassium ion transport	GO:0006813	6.12E-01	6.85E-01	Htr2a,15558:down|Kcnj15,16516:down|Fxyd2,11936:up
cerebellar cortex development	GO:0021695	6.13E-01	6.85E-01	Serpine2,20720:up
photoreceptor cell differentiation	GO:0046530	6.13E-01	6.85E-01	Vegfa,22339:down
protein export from nucleus	GO:0006611	6.13E-01	6.85E-01	Gas6,14456:up
histone deacetylation	GO:0016575	6.13E-01	6.85E-01	Vegfa,22339:down
nucleoside triphosphate biosynthetic process	GO:0009142	6.19E-01	6.90E-01	Htr2a,15558:down|Igf1,16000:up
monovalent inorganic cation homeostasis	GO:0055067	6.19E-01	6.90E-01	Ednrb,13618:up|Anpep,16790:up
positive regulation of organelle assembly	GO:1902117	6.20E-01	6.91E-01	Tnf,21926:up
respiratory gaseous exchange	GO:0007585	6.20E-01	6.91E-01	Chst11,58250:down
regulation of lipid catabolic process	GO:0050994	6.20E-01	6.91E-01	Tnf,21926:up
negative regulation of axonogenesis	GO:0050771	6.20E-01	6.91E-01	Wnt5a,22418:down
cell cycle arrest	GO:0007050	6.23E-01	6.94E-01	Tgfb2,21808:down|Inhba,16323:down
regulation of translational initiation	GO:0006446	6.27E-01	6.98E-01	Tnf,21926:up
release of cytochrome c from mitochondria	GO:0001836	6.27E-01	6.98E-01	Igf1,16000:up
regulation of neurotransmitter secretion	GO:0046928	6.27E-01	6.98E-01	Sncg,20618:up
positive regulation of DNA biosynthetic process	GO:2000573	6.27E-01	6.98E-01	Grem1,23892:up
regulation of dendritic spine development	GO:0060998	6.27E-01	6.98E-01	Plk2,20620:down
macromolecule glycosylation	GO:0043413	6.28E-01	6.98E-01	Sdf2l1,64136:up|Vegfb,22340:up|Man2a2,140481:up
protein glycosylation	GO:0006486	6.28E-01	6.98E-01	Sdf2l1,64136:up|Vegfb,22340:up|Man2a2,140481:up
maintenance of location in cell	GO:0051651	6.33E-01	7.03E-01	Kdelr3,105785:down|Cd4,12504:up
cAMP metabolic process	GO:0046058	6.33E-01	7.03E-01	Adm,11535:up|Ptgir,19222:up
regulation of DNA recombination	GO:0000018	6.34E-01	7.04E-01	Tfrc,22042:up
negative regulation of protein complex disassembly	GO:0043242	6.34E-01	7.04E-01	Tnf,21926:up
positive regulation of stem cell differentiation	GO:2000738	6.34E-01	7.04E-01	Tgfb2,21808:down
centrosome duplication	GO:0051298	6.34E-01	7.04E-01	Plk2,20620:down
regulation of excitatory postsynaptic membrane potential	GO:0060079	6.34E-01	7.04E-01	Slc29a1,63959:up
actin filament bundle organization	GO:0061572	6.37E-01	7.07E-01	Fam101b,76566:down|Dpysl3,22240:down
somatic cell DNA recombination	GO:0016444	6.41E-01	7.10E-01	Tfrc,22042:up
membrane docking	GO:0022406	6.41E-01	7.10E-01	Ncam1,17967:down

cellular response to UV	GO:0034644	6.41E-01	7.10E-01	Mme,17380:up
somatic diversification of immune receptors via germline recombination within a single locus	GO:0002562	6.41E-01	7.10E-01	Tfrc,22042:up
regulation of defense response to virus	GO:0050688	6.42E-01	7.11E-01	Lmcd1,30937:up|Ifit1,15957:up
visual perception	GO:0007601	6.46E-01	7.16E-01	Col11a1,12814:down|Epas1,13819:up
purine ribonucleoside triphosphate metabolic process	GO:0009205	6.46E-01	7.16E-01	Htr2a,15558:down|Ampd3,11717:up|Igf1,16000:up
intrinsic apoptotic signaling pathway in response to endoplasmic reticulum stress	GO:0070059	6.47E-01	7.16E-01	Creb3l1,26427:down
CD4-positive, alpha-beta T cell differentiation	GO:0043367	6.47E-01	7.16E-01	Ly9,17085:up
regulation of mRNA stability	GO:0043488	6.47E-01	7.16E-01	Vegfa,22339:down
mast cell activation	GO:0045576	6.47E-01	7.16E-01	Hmox1,15368:up
lysosome localization	GO:0032418	6.47E-01	7.16E-01	Hmox1,15368:up
membrane depolarization	GO:0051899	6.51E-01	7.20E-01	Slc29a1,63959:up|Adipoq,11450:up
cholesterol transport	GO:0030301	6.54E-01	7.22E-01	Adipoq,11450:up
leukocyte degranulation	GO:0043299	6.54E-01	7.22E-01	Hmox1,15368:up
cellular nitrogen compound biosynthetic process	GO:0044271	6.54E-01	7.23E-01	Fstl3,83554:down|Ice1,218333:down|Adm,11535:up|Tfec,21426:up|Sfrp4,20379:up|Ednrb,13618:up|Ampd3,11717:up|Epas1,13819:up|Ankrd1,107765:down|Htra3,78558:down|Uchl5,56207:up|Ddah1,69219:down|Lyl1,17095:up|Sdpr,20324:down|Ggt5,23887:up|Loxl2,94352:down|Loxl3,16950:down|Trf,22041:up|Ptx3,19288:up|Wnt5a,22418:down|Bmpr1a,12166:down|Ccl4,20303:up|Adipoq,11450:up|Bmp4,12159:up|Igf1,16000:up|Ret,19713:up|Pdxk,216134:up|Plpp3,67916:up|Ptgir,19222:up|Creb3l1,26427:down|Fgfr2,14183:up|Htr2b,15559:down|Prl2c2,18811:up|Efemp1,216616:up|Inhba,16323:down|Rasl11a,68895:up|Bnc2,242509:down|Ccl3,20302:up|Vdr,22337:down|Fabp4,11770:up|Vegfa,22339:down|Htr2a,15558:down|Igf2,16002:up|Slc40a1,53945:up|Pparg,19016:up|Gas6,14456:up|Tnf,21926:up|Glp1r,14652:down|Lmcd1,30937:up|Fgfr1,14182:down|Egfr,13649:down|Hmox1,15368:up|Sfrp1,20377:up|Sulf1,240725:down|Tgfbr3,21814:up|Ptgs2,19225:down|Grem1,23892:up|Phf11d,219132:up|Sfrp2,20319:up|Tbx15,21384:down|Npnt,114249:up|Uck2,80914:down|S100a1,20193:up
dephosphorylation	GO:0016311	6.55E-01	7.23E-01	Tnf,21926:up|Alpl,11647:up|Tgfb2,21808:down|Npnt,114249:up|Plpp3,67916:up
lipid transport	GO:0006869	6.56E-01	7.24E-01	Pparg,19016:up|Fam132b,227358:down|Inhba,16323:down|Adipoq,11450:up
purine ribonucleoside metabolic process	GO:0046128	6.56E-01	7.24E-01	Htr2a,15558:down|Enpp1,18605:down|Ampd3,11717:up|Igf1,16000:up
organelle disassembly	GO:1903008	6.57E-01	7.25E-01	Lmcd1,30937:up|Plod2,26432:down|Sfrp4,20379:up
ribonucleoside triphosphate metabolic process	GO:0009199	6.57E-01	7.25E-01	Htr2a,15558:down|Ampd3,11717:up|Igf1,16000:up
glycosylation	GO:0070085	6.57E-01	7.25E-01	Sdf2l1,64136:up|Vegfb,22340:up|Man2a2,140481:up
sterol transport	GO:0015918	6.60E-01	7.27E-01	Adipoq,11450:up
peripheral nervous system development	GO:0007422	6.60E-01	7.27E-01	Ednrb,13618:up
ER-associated ubiquitin-dependent protein catabolic process	GO:0030433	6.60E-01	7.27E-01	Sdf2l1,64136:up
regulation of organelle assembly	GO:1902115	6.64E-01	7.31E-01	Tnf,21926:up|Plk2,20620:down
regulation of cell projection assembly	GO:0060491	6.64E-01	7.31E-01	Prl2c2,18811:up|Dpysl3,22240:down
sensory perception of light stimulus	GO:0050953	6.64E-01	7.31E-01	Col11a1,12814:down|Epas1,13819:up
purine ribonucleoside monophosphate metabolic process	GO:0009167	6.64E-01	7.31E-01	Htr2a,15558:down|Ampd3,11717:up|Igf1,16000:up
somatic diversification of immune receptors	GO:0002200	6.66E-01	7.33E-01	Tfrc,22042:up
neuromuscular process controlling balance	GO:0050885	6.66E-01	7.33E-01	Aldh1a3,56847:down

oligosaccharide metabolic process	GO:0009311	6.66E-01	7.33E-01	Man2a2,140481:up
purine nucleoside monophosphate metabolic process	GO:0009126	6.68E-01	7.34E-01	Htr2a,15558:down|Ampd3,11717:up|Igf1,16000:up
purine nucleoside metabolic process	GO:0042278	6.69E-01	7.35E-01	Htr2a,15558:down|Enpp1,18605:down|Ampd3,11717:up|Igf1,16000:up
lipid catabolic process	GO:0016042	6.72E-01	7.38E-01	Tnf,21926:up|Akr1c18,105349:up|Lpl,16956:up|Adipoq,11450:up
activation of GTPase activity	GO:0090630	6.72E-01	7.38E-01	Wnt5a,22418:down
mitotic cell cycle process	GO:1903047	6.73E-01	7.39E-01	Tnf,21926:up|Plk2,20620:down|Fgfr1,14182:down|Egfr,13649:down|Inhba,16323:down|Igf1,16000:up|Bmp4,12159:up|Nabp1,109019:down|Fgfr2,14183:up|Igf2,16002:up
intrinsic apoptotic signaling pathway	GO:0097193	6.75E-01	7.41E-01	Tnf,21926:up|Cxcl12,20315:up|Hmox1,15368:up|Creb3l1,26427:down
purine nucleoside triphosphate metabolic process	GO:0009144	6.75E-01	7.41E-01	Htr2a,15558:down|Ampd3,11717:up|Igf1,16000:up
protein oligomerization	GO:0051259	6.75E-01	7.42E-01	Col1a2,12843:down|Hmox1,15368:up|C1qtnf3,81799:down|C1qtnf6,72709:down|Dpysl3,22240:down|Cbr2,12409:up|Adipoq,11450:up
regulation of membrane potential	GO:0042391	6.77E-01	7.43E-01	Tnf,21926:up|Fxyd1,56188:up|Il1rn,16181:up|Slc29a1,63959:up|Adipoq,11450:up
coenzyme metabolic process	GO:0006732	6.78E-01	7.44E-01	Htr2a,15558:down|Pdxk,216134:up|Cbr2,12409:up|Igf1,16000:up
response to endoplasmic reticulum stress	GO:0034976	6.81E-01	7.47E-01	Pdia5,72599:down|Sdf2l1,64136:up|Creb3l1,26427:down
regulation of protein localization to plasma membrane	GO:1903076	6.84E-01	7.49E-01	Lrrc15,74488:down
macroautophagy	GO:0016236	6.86E-01	7.52E-01	Lmcd1,30937:up|Plod2,26432:down|Hmox1,15368:up|Sfrp4,20379:up
regulation of protein complex assembly	GO:0043254	6.88E-01	7.53E-01	Tnf,21926:up|Ice1,218333:down|Cd24a,12484:down|Ncam1,17967:down|Vegfa,22339:down
purine nucleoside biosynthetic process	GO:0042451	6.89E-01	7.54E-01	Htr2a,15558:down|Igf1,16000:up
purine ribonucleoside biosynthetic process	GO:0046129	6.89E-01	7.54E-01	Htr2a,15558:down|Igf1,16000:up
regulation of RNA stability	GO:0043487	6.90E-01	7.55E-01	Vegfa,22339:down
cellular aromatic compound metabolic process	GO:0006725	6.93E-01	7.58E-01	Fstl3,83554:down|Adm,11535:up|Tfec,21426:up|Sfrp4,20379:up|Enpp5,83965:up|Ankrd1,107765:down|Lyl1,17095:up|Gstt1,14871:up|Blvrb,233016:up|Igfbp3,16009:up|Loxl2,94352:down|Loxl3,16950:down|Trf,22041:up|Wnt5a,22418:down|Maob,109731:up|Adipoq,11450:up|Bmp4,12159:up|Hal,15109:up|Igf1,16000:up|Tgfb2,21808:down|Ptgir,19222:up|Fgfr2,14183:up|Htr2b,15559:down|Prl2c2,18811:up|Efemp1,216616:up|Vdr,22337:down|Fabp4,11770:up|Nabp1,109019:down|Htr2a,15558:down|Oas3,246727:up|Slc40a1,53945:up|Gas6,14456:up|Egfr,13649:down|Sfrp1,20377:up|Tgfbr3,21814:up|Tfrc,22042:up|Sfrp2,20319:up|Npnt,114249:up|S100a1,20193:up|Ice1,218333:down|Pdgfc,54635:down|Ednrb,13618:up|Ampd3,11717:up|Epas1,13819:up|Htra3,78558:down|Uchl5,56207:up|Sdpr,20324:down|Enpp3,209558:down|Bmpr1a,12166:down|Ccl4,20303:up|Ret,19713:up|Pdxk,216134:up|Plpp3,67916:up|Creb3l1,26427:down|Kitl,17311:up|Rasl11a,68895:up|Inhba,16323:down|Bnc2,242509:down|Ccl3,20302:up|Gfpt2,14584:down|Vegfa,22339:down|Cbr2,12409:up|Igf2,16002:up|Anpep,16790:up|Tnf,21926:up|Pparg,19016:up|Lmcd1,30937:up|Glp1r,14652:down|Fgfr1,14182:down|Hmox1,15368:up|Sulf1,240725:down|Grem1,23892:up|Phf11d,219132:up|Tbx15,21384:down|Uck2,80914:down|Enpp1,18605:down
endomembrane system organization	GO:0010256	6.94E-01	7.59E-01	Tnf,21926:up|Gas6,14456:up|Col5a1,12831:down|Tgfb2,21808:down|Serpine2,20720:up|Lrrc15,74488:down|Adipoq,11450:up
positive regulation of viral process	GO:0048524	6.95E-01	7.59E-01	Ceacam1,26365:up
synaptic vesicle exocytosis	GO:0016079	6.95E-01	7.59E-01	Syt13,80976:up
protein deacetylation	GO:0006476	6.95E-01	7.59E-01	Vegfa,22339:down
B cell activation involved in immune response	GO:0002312	6.95E-01	7.59E-01	Tfrc,22042:up
regulation of TOR signaling	GO:0032006	6.95E-01	7.59E-01	Gas6,14456:up
natural killer cell activation	GO:0030101	6.95E-01	7.59E-01	Gas6,14456:up
signal transduction in response to DNA damage	GO:0042770	6.95E-01	7.59E-01	Ankrd1,107765:down
organonitrogen compound biosynthetic process	GO:1901566	7.01E-01	7.64E-01	Htr2b,15559:down|Adm,11535:up|Ampd3,11717:up|Epas1,13819:up|Htr2a,15558:down|Cdo1,12583:up|Tnf,21926:up|Ggt5,23887:up|Fabp5,16592:up|Egfr,13649:down|Has2,15117:down|Igf1,16000:up|Chst11,58250:down|Gatm,67092:down|Pdxk,216134:up|Tgfb2,21808:down|Ptgir,19222:up|Uck2,80914:down
regulation of postsynaptic membrane potential	GO:0060078	7.01E-01	7.64E-01	Slc29a1,63959:up
regulation of release of sequestered calcium ion into cytosol	GO:0051279	7.01E-01	7.64E-01	Tgfb2,21808:down

regulation of protein dephosphorylation	GO:0035304	7.01E-01	7.64E-01	Tnf,21926:up
alpha-amino acid biosynthetic process	GO:1901607	7.01E-01	7.64E-01	Gatm,67092:down
regulation of DNA repair	GO:0006282	7.01E-01	7.64E-01	Egfr,13649:down
DNA recombination	GO:0006310	7.01E-01	7.65E-01	Uchl5,56207:up|Nabp1,109019:down|Tfrc,22042:up
regulation of intrinsic apoptotic signaling pathway	GO:2001242	7.04E-01	7.68E-01	Cxcl12,20315:up|Creb3l1,26427:down
negative regulation of ion transmembrane transport	GO:0034766	7.06E-01	7.69E-01	Tgfb2,21808:down
regulation of Notch signaling pathway	GO:0008593	7.06E-01	7.69E-01	Postn,50706:down
cell-cell junction assembly	GO:0007043	7.06E-01	7.69E-01	Tnf,21926:up
energy derivation by oxidation of organic compounds	GO:0015980	7.08E-01	7.71E-01	Enpp1,18605:down|Igf2,16002:up|Igf1,16000:up
generation of precursor metabolites and energy	GO:0006091	7.09E-01	7.72E-01	Htr2a,15558:down|Enpp1,18605:down|Igf2,16002:up|Igf1,16000:up
heterocycle metabolic process	GO:0046483	7.10E-01	7.73E-01	Fstl3,83554:down|Adm,11535:up|Tfec,21426:up|Sfrp4,20379:up|Enpp5,83965:up|Ankrd1,107765:down|Lyl1,17095:up|Gstt1,14871:up|Blvrb,233016:up|Igfbp3,16009:up|Loxl2,94352:down|Loxl3,16950:down|Trf,22041:up|Mme,17380:up|Wnt5a,22418:down|Adipoq,11450:up|Bmp4,12159:up|Hal,15109:up|Igf1,16000:up|Ptgir,19222:up|Fgfr2,14183:up|Htr2b,15559:down|Prl2c2,18811:up|Efemp1,216616:up|Vdr,22337:down|Fabp4,11770:up|Nabp1,109019:down|Htr2a,15558:down|Oas3,246727:up|Slc40a1,53945:up|Gas6,14456:up|Egfr,13649:down|Sfrp1,20377:up|Tgfbr3,21814:up|Tfrc,22042:up|Sfrp2,20319:up|Npnt,114249:up|S100a1,20193:up|Ice1,218333:down|Pdgfc,54635:down|Ednrb,13618:up|Ampd3,11717:up|Epas1,13819:up|Htra3,78558:down|Uchl5,56207:up|Sdpr,20324:down|Enpp3,209558:down|Bmpr1a,12166:down|Ccl4,20303:up|Ret,19713:up|Pdxk,216134:up|Plpp3,67916:up|Creb3l1,26427:down|Kitl,17311:up|P4ha1,18451:down|Rasl11a,68895:up|Inhba,16323:down|Bnc2,242509:down|Ccl3,20302:up|Gfpt2,14584:down|Vegfa,22339:down|Cbr2,12409:up|Igf2,16002:up|Tnf,21926:up|Pparg,19016:up|Lmcd1,30937:up|Glp1r,14652:down|Fgfr1,14182:down|Hmox1,15368:up|Sulf1,240725:down|Grem1,23892:up|Phf11d,219132:up|Tbx15,21384:down|Uck2,80914:down|Enpp1,18605:down
ER to Golgi vesicle-mediated transport	GO:0006888	7.12E-01	7.74E-01	Sec23a,20334:down
positive regulation of B cell activation	GO:0050871	7.12E-01	7.74E-01	Tfrc,22042:up
centrosome cycle	GO:0007098	7.12E-01	7.74E-01	Plk2,20620:down
fatty acid beta-oxidation	GO:0006635	7.12E-01	7.74E-01	Adipoq,11450:up
neuropeptide signaling pathway	GO:0007218	7.17E-01	7.80E-01	Glp1r,14652:down
cellular response to starvation	GO:0009267	7.18E-01	7.81E-01	Gas6,14456:up|Lmcd1,30937:up|Plod2,26432:down|Hmox1,15368:up|Sfrp4,20379:up
positive regulation of T cell differentiation	GO:0045582	7.22E-01	7.84E-01	Tnfsf9,21950:up
long-chain fatty acid metabolic process	GO:0001676	7.22E-01	7.84E-01	Ptgs2,19225:down
positive regulation of proteasomal ubiquitin-dependent protein catabolic process	GO:0032436	7.22E-01	7.84E-01	Plk2,20620:down
protein N-linked glycosylation	GO:0006487	7.22E-01	7.84E-01	Man2a2,140481:up
cellular response to light stimulus	GO:0071482	7.27E-01	7.89E-01	Mme,17380:up
protein deacylation	GO:0035601	7.27E-01	7.89E-01	Vegfa,22339:down
skeletal muscle cell differentiation	GO:0035914	7.27E-01	7.89E-01	Ankrd1,107765:down
macromolecule deacylation	GO:0098732	7.32E-01	7.93E-01	Vegfa,22339:down
glial cell development	GO:0021782	7.32E-01	7.93E-01	Egfr,13649:down
oogenesis	GO:0048477	7.32E-01	7.93E-01	Igf1,16000:up
positive regulation of insulin secretion	GO:0032024	7.32E-01	7.93E-01	Glp1r,14652:down
regulation of cyclin-dependent protein serine/threonine kinase activity	GO:0000079	7.32E-01	7.93E-01	Egfr,13649:down
regulation of mitochondrion organization	GO:0010821	7.34E-01	7.94E-01	Lmcd1,30937:up|Plod2,26432:down|Sfrp4,20379:up|Igf1,16000:up

T cell mediated immunity	GO:0002456	7.37E-01	7.97E-01	Cd24a,12484:down
dendritic spine development	GO:0060996	7.37E-01	7.97E-01	Plk2,20620:down
regulation of cyclin-dependent protein kinase activity	GO:1904029	7.37E-01	7.97E-01	Egfr,13649:down
protein localization to membrane	GO:0072657	7.37E-01	7.97E-01	Tnf,21926:up|Gas6,14456:up|Ccl2,20296:up|Lrrc15,74488:down|Adipoq,11450:up
negative regulation of transmembrane transport	GO:0034763	7.42E-01	8.01E-01	Tgfb2,21808:down
polyol metabolic process	GO:0019751	7.42E-01	8.01E-01	Sord,20322:up
DNA modification	GO:0006304	7.42E-01	8.01E-01	Gstt1,14871:up
liposaccharide metabolic process	GO:1903509	7.42E-01	8.01E-01	Aoah,27052:up
regulation of potassium ion transport	GO:0043266	7.42E-01	8.01E-01	Htr2a,15558:down
regulation of protein complex disassembly	GO:0043244	7.42E-01	8.01E-01	Tnf,21926:up
organic cyclic compound metabolic process	GO:1901360	7.42E-01	8.01E-01	Fstl3,83554:down|Adm,11535:up|Tfec,21426:up|Sfrp4,20379:up|Enpp5,83965:up|Ankrd1,107765:down|Lyl1,17095:up|Gstt1,14871:up|Blvrb,233016:up|Igfbp3,16009:up|Loxl2,94352:down|Loxl3,16950:down|Trf,22041:up|Mme,17380:up|Wnt5a,22418:down|Maob,109731:up|Adipoq,11450:up|Bmp4,12159:up|Hal,15109:up|Igf1,16000:up|Tgfb2,21808:down|Ptgir,19222:up|Fgfr2,14183:up|Htr2b,15559:down|Prl2c2,18811:up|Efemp1,216616:up|Vdr,22337:down|Fabp4,11770:up|Nabp1,109019:down|Htr2a,15558:down|Oas3,246727:up|Slc40a1,53945:up|Gas6,14456:up|Egfr,13649:down|Sfrp1,20377:up|Tgfbr3,21814:up|Tfrc,22042:up|Sfrp2,20319:up|Npnt,114249:up|S100a1,20193:up|Ice1,218333:down|Pdgfc,54635:down|Ednrb,13618:up|Ampd3,11717:up|Epas1,13819:up|Htra3,78558:down|Uchl5,56207:up|Sdpr,20324:down|Enpp3,209558:down|Akr1c18,105349:up|Bmpr1a,12166:down|Ccl4,20303:up|Ret,19713:up|Pdxk,216134:up|Plpp3,67916:up|Creb3l1,26427:down|Kitl,17311:up|P4ha1,18451:down|Rasl11a,68895:up|Inhba,16323:down|Bnc2,242509:down|Ccl3,20302:up|Vegfa,22339:down|Gfpt2,14584:down|Cbr2,12409:up|Igf2,16002:up|Tnf,21926:up|Pparg,19016:up|Lmcd1,30937:up|Glp1r,14652:down|Fgfr1,14182:down|Hmox1,15368:up|Sulf1,240725:down|Grem1,23892:up|Phf11d,219132:up|Tbx15,21384:down|Uck2,80914:down|Enpp1,18605:down
cell cycle checkpoint	GO:0000075	7.44E-01	8.04E-01	Plk2,20620:down|Nabp1,109019:down
TOR signaling	GO:0031929	7.46E-01	8.05E-01	Gas6,14456:up
localization within membrane	GO:0051668	7.46E-01	8.05E-01	Cd24a,12484:down
regulation of pH	GO:0006885	7.46E-01	8.05E-01	Ednrb,13618:up
ERAD pathway	GO:0036503	7.46E-01	8.05E-01	Sdf2l1,64136:up
actin filament organization	GO:0007015	7.49E-01	8.08E-01	Fam101b,76566:down|Trf,22041:up|Shroom4,208431:down|Dpysl3,22240:down
mitotic spindle organization	GO:0007052	7.51E-01	8.09E-01	Plk2,20620:down
negative regulation of protein ubiquitination	GO:0031397	7.51E-01	8.09E-01	Nxn,18230:down
peptidyl-threonine phosphorylation	GO:0018107	7.51E-01	8.09E-01	Wnt5a,22418:down
cell cycle phase transition	GO:0044770	7.51E-01	8.09E-01	Plk2,20620:down|Egfr,13649:down|Inhba,16323:down|Susd2,71733:up
positive regulation of histone modification	GO:0031058	7.55E-01	8.13E-01	Vegfa,22339:down
positive regulation of proteasomal protein catabolic process	GO:1901800	7.55E-01	8.13E-01	Plk2,20620:down
response to starvation	GO:0042594	7.56E-01	8.13E-01	Gas6,14456:up|Lmcd1,30937:up|Plod2,26432:down|Hmox1,15368:up|Sfrp4,20379:up
proton transport	GO:0015992	7.60E-01	8.17E-01	Hvcn1,74096:up
regulation of macroautophagy	GO:0016241	7.60E-01	8.17E-01	Hmox1,15368:up
T cell differentiation in thymus	GO:0033077	7.60E-01	8.17E-01	Bmp4,12159:up
thymocyte aggregation	GO:0071594	7.60E-01	8.17E-01	Bmp4,12159:up
actin cytoskeleton reorganization	GO:0031532	7.60E-01	8.17E-01	Ptk7,71461:down

cellular nitrogen compound metabolic process	GO:0034641	7.60E-01	8.17E-01	Fstl3,83554:down|Adm,11535:up|Tfec,21426:up|Sfrp4,20379:up|Enpp5,83965:up|Ankrd1,107765:down|Lyl1,17095:up|Gstt1,14871:up|Blvrb,233016:up|Igfbp3,16009:up|Ggt5,23887:up|Loxl2,94352:down|Loxl3,16950:down|Trf,22041:up|Mme,17380:up|Wnt5a,22418:down|Maob,109731:up|Adipoq,11450:up|Bmp4,12159:up|Hal,15109:up|Igf1,16000:up|Ptgir,19222:up|Fgfr2,14183:up|Htr2b,15559:down|Prl2c2,18811:up|Efemp1,216616:up|Vdr,22337:down|Fabp4,11770:up|Nabp1,109019:down|Htr2a,15558:down|Oas3,246727:up|Slc40a1,53945:up|Gas6,14456:up|Egfr,13649:down|Sfrp1,20377:up|Tgfbr3,21814:up|Tfrc,22042:up|Gstm1,14862:up|Sfrp2,20319:up|Npnt,114249:up|S100a1,20193:up|Ice1,218333:down|Pdgfc,54635:down|Ednrb,13618:up|Ampd3,11717:up|Mmp13,17386:up|Epas1,13819:up|Htra3,78558:down|Uchl5,56207:up|Ddah1,69219:down|Sdpr,20324:down|Enpp3,209558:down|Ptx3,19288:up|Fabp5,16592:up|Bmpr1a,12166:down|Ccl4,20303:up|Ret,19713:up|Pdxk,216134:up|Plpp3,67916:up|Creb3l1,26427:down|Kitl,17311:up|Rasl11a,68895:up|Inhba,16323:down|Bnc2,242509:down|Ccl3,20302:up|Vegfa,22339:down|Gfpt2,14584:down|Cbr2,12409:up|Igf2,16002:up|Anpep,16790:up|Pparg,19016:up|Tnf,21926:up|Lmcd1,30937:up|Glp1r,14652:down|Fgfr1,14182:down|Hmox1,15368:up|Sulf1,240725:down|Ptgs2,19225:down|Grem1,23892:up|Phf11d,219132:up|Tbx15,21384:down|Uck2,80914:down|Enpp1,18605:down
hydrogen transport	GO:0006818	7.64E-01	8.21E-01	Hvcn1,74096:up
regulation of G1/S transition of mitotic cell cycle	GO:2000045	7.64E-01	8.21E-01	Egfr,13649:down
gene silencing by RNA	GO:0031047	7.64E-01	8.21E-01	Egfr,13649:down
peptidyl-threonine modification	GO:0018210	7.69E-01	8.25E-01	Wnt5a,22418:down
Notch signaling pathway	GO:0007219	7.71E-01	8.27E-01	Hp,15439:up|Postn,50706:down
xenophagy	GO:0098792	7.73E-01	8.28E-01	Lmcd1,30937:up
toll-like receptor signaling pathway	GO:0002224	7.73E-01	8.28E-01	Tnf,21926:up
positive regulation of chromatin modification	GO:1903310	7.73E-01	8.28E-01	Vegfa,22339:down
regulated secretory pathway	GO:0045055	7.74E-01	8.29E-01	Hmox1,15368:up|Syt13,80976:up
cerebellum development	GO:0021549	7.77E-01	8.32E-01	Serpine2,20720:up
cellular biogenic amine metabolic process	GO:0006576	7.77E-01	8.32E-01	Fabp5,16592:up
double-strand break repair via homologous recombination	GO:0000724	7.77E-01	8.32E-01	Nabp1,109019:down
recombinational repair	GO:0000725	7.77E-01	8.32E-01	Nabp1,109019:down
positive regulation of translation	GO:0045727	7.77E-01	8.32E-01	Tnf,21926:up
associative learning	GO:0008306	7.81E-01	8.35E-01	Glp1r,14652:down
negative regulation of protein modification by small protein conjugation or removal	GO:1903321	7.81E-01	8.35E-01	Nxn,18230:down
regulation of actin filament-based process	GO:0032970	7.84E-01	8.38E-01	Fxyd1,56188:up|Gpm6b,14758:up|Tgfb2,21808:down|Stc1,20855:up
alpha-beta T cell differentiation	GO:0046632	7.85E-01	8.38E-01	Ly9,17085:up
fatty acid catabolic process	GO:0009062	7.85E-01	8.38E-01	Adipoq,11450:up
coenzyme biosynthetic process	GO:0009108	7.85E-01	8.38E-01	Pdxk,216134:up
chloride transport	GO:0006821	7.89E-01	8.42E-01	Fxyd1,56188:up
positive regulation of mitochondrion organization	GO:0010822	7.89E-01	8.42E-01	Lmcd1,30937:up|Plod2,26432:down|Sfrp4,20379:up
multi-organism behavior	GO:0051705	7.93E-01	8.45E-01	Serpine2,20720:up
regulation of proteasomal protein catabolic process	GO:0061136	7.97E-01	8.50E-01	Uchl5,56207:up|Plk2,20620:down
mitotic cell cycle	GO:0000278	7.98E-01	8.51E-01	Tnf,21926:up|Plk2,20620:down|Fgfr1,14182:down|Egfr,13649:down|Inhba,16323:down|Igf1,16000:up|Bmp4,12159:up|Nabp1,109019:down|Fgfr2,14183:up|Igf2,16002:up
regulation of exocytosis	GO:0017157	8.00E-01	8.52E-01	Ncam1,17967:down|Hmox1,15368:up
nucleic acid phosphodiester bond hydrolysis	GO:0090305	8.00E-01	8.53E-01	Igfbp3,16009:up
circadian rhythm	GO:0007623	8.05E-01	8.57E-01	Pparg,19016:up|Igf1,16000:up
metencephalon development	GO:0022037	8.07E-01	8.59E-01	Serpine2,20720:up

<table>
<tr><td>cell recognition</td><td>GO:0008037</td><td>8.07E-01</td><td>8.59E-01</td><td>Wnt5a,22418:down</td></tr>
<tr><td>protein deubiquitination</td><td>GO:0016579</td><td>8.07E-01</td><td>8.59E-01</td><td>Uchl5,56207:up</td></tr>
<tr><td>response to light stimulus</td><td>GO:0009416</td><td>8.08E-01</td><td>8.59E-01</td><td>Pparg,19016:up|Egfr,13649:down|Mme,17380:up</td></tr>
<tr><td>positive regulation of proteolysis involved in cellular protein catabolic process</td><td>GO:1903052</td><td>8.11E-01</td><td>8.62E-01</td><td>Plk2,20620:down</td></tr>
<tr><td>acid secretion</td><td>GO:0046717</td><td>8.11E-01</td><td>8.62E-01</td><td>Il1rn,16181:up</td></tr>
<tr><td>mitotic nuclear division</td><td>GO:0007067</td><td>8.16E-01</td><td>8.67E-01</td><td>Tnf,21926:up|Igf2,16002:up|Fgfr2,14183:up|Igf1,16000:up|Bmp4,12159:up</td></tr>
<tr><td>positive regulation of cellular amide metabolic process</td><td>GO:0034250</td><td>8.18E-01</td><td>8.68E-01</td><td>Tnf,21926:up</td></tr>
<tr><td>positive regulation of defense response to virus by host</td><td>GO:0002230</td><td>8.18E-01</td><td>8.68E-01</td><td>Lmcd1,30937:up</td></tr>
<tr><td>cell cycle process</td><td>GO:0022402</td><td>8.20E-01</td><td>8.70E-01</td><td>Tnf,21926:up|Plk2,20620:down|Fgfr1,14182:down|Egfr,13649:down|Sfrp1,20377:up|Wnt5a,22418:down|Inhba,16323:down|Susd2,71733:up|Igf1,16000:up|Bmp4,12159:up|Nabp1,109019:down|Tgfb2,21808:down|Fgfr2,14183:up|Igf2,16002:up</td></tr>
<tr><td>peptide metabolic process</td><td>GO:0006518</td><td>8.20E-01</td><td>8.70E-01</td><td>Ggt5,23887:up|Tnf,21926:up|Egfr,13649:down|Mme,17380:up|Mmp13,17386:up|Igf1,16000:up|Gstm1,14862:up|Gstt1,14871:up|Anpep,16790:up</td></tr>
<tr><td>sexual reproduction</td><td>GO:0019953</td><td>8.23E-01</td><td>8.73E-01</td><td>Inhba,16323:down|Ptgs2,19225:down|Ednrb,13618:up|Plat,18791:down|Igf1,16000:up|Bmp4,12159:up|Cxcl12,20315:up|Serpine2,20720:up|Kitl,17311:up</td></tr>
<tr><td>centrosome organization</td><td>GO:0051297</td><td>8.24E-01</td><td>8.73E-01</td><td>Plk2,20620:down</td></tr>
<tr><td>gamete generation</td><td>GO:0007276</td><td>8.24E-01</td><td>8.73E-01</td><td>Cxcl12,20315:up|Inhba,16323:down|Ptgs2,19225:down|Plat,18791:down|Kitl,17311:up|Igf1,16000:up|Bmp4,12159:up</td></tr>
<tr><td>posttranscriptional regulation of gene expression</td><td>GO:0010608</td><td>8.25E-01</td><td>8.74E-01</td><td>Tnf,21926:up|Egfr,13649:down|Vegfa,22339:down|Igf1,16000:up</td></tr>
<tr><td>nucleobase-containing compound metabolic process</td><td>GO:0006139</td><td>8.27E-01</td><td>8.76E-01</td><td>Fstl3,83554:down|Adm,11535:up|Tfec,21426:up|Sfrp4,20379:up|Enpp5,83965:up|Ankrd1,107765:down|Lyl1,17095:up|Gstt1,14871:up|Igfbp3,16009:up|Loxl2,94352:down|Loxl3,16950:down|Trf,22041:up|Wnt5a,22418:down|Adipoq,11450:up|Bmp4,12159:up|Igf1,16000:up|Ptgir,19222:up|Fgfr2,14183:up|Htr2b,15559:down|Prl2c2,18811:up|Efemp1,216616:up|Vdr,22337:down|Fabp4,11770:up|Nabp1,109019:down|Htr2a,15558:down|Oas3,246727:up|Slc40a1,53945:up|Gas6,14456:up|Egfr,13649:down|Sfrp1,20377:up|Tgfbr3,21814:up|Tfrc,22042:up|Sfrp2,20319:up|Npnt,114249:up|S100a1,20193:up|Ice1,218333:down|Ednrb,13618:up|Pdgfc,54635:down|Ampd3,11717:up|Epas1,13819:up|Uchl5,56207:up|Htra3,78558:down|Sdpr,20324:down|Enpp3,209558:down|Bmpr1a,12166:down|Ccl4,20303:up|Ret,19713:up|Plpp3,67916:up|Creb3l1,26427:down|Kitl,17311:up|Rasl11a,68895:up|Inhba,16323:down|Bnc2,242509:down|Ccl3,20302:up|Gfpt2,14584:down|Vegfa,22339:down|Cbr2,12409:up|Igf2,16002:up|Tnf,21926:up|Pparg,19016:up|Lmcd1,30937:up|Glp1r,14652:down|Fgfr1,14182:down|Hmox1,15368:up|Sulf1,240725:down|Grem1,23892:up|Phf11d,219132:up|Tbx15,21384:down|Enpp1,18605:down|Uck2,80914:down</td></tr>
<tr><td>monocarboxylic acid catabolic process</td><td>GO:0072329</td><td>8.27E-01</td><td>8.76E-01</td><td>Adipoq,11450:up</td></tr>
<tr><td>translational initiation</td><td>GO:0006413</td><td>8.27E-01</td><td>8.76E-01</td><td>Tnf,21926:up</td></tr>
<tr><td>pattern recognition receptor signaling pathway</td><td>GO:0002221</td><td>8.34E-01</td><td>8.82E-01</td><td>Tnf,21926:up</td></tr>
<tr><td>cellular macromolecule biosynthetic process</td><td>GO:0034645</td><td>8.36E-01</td><td>8.83E-01</td><td>Fstl3,83554:down|Ice1,218333:down|Tfec,21426:up|Sfrp4,20379:up|Pdgfc,54635:down|Ednrb,13618:up|Epas1,13819:up|Ankrd1,107765:down|Htra3,78558:down|Uchl5,56207:up|Lyl1,17095:up|Sdpr,20324:down|Man2a2,140481:up|Loxl2,94352:down|Loxl3,16950:down|Trf,22041:up|Wnt5a,22418:down|Bmpr1a,12166:down|Adipoq,11450:up|Bmp4,12159:up|Igf1,16000:up|Chst11,58250:down|Ret,19713:up|Sdf2l1,64136:up|Vegfb,22340:up|Plpp3,67916:up|Creb3l1,26427:down|Fgfr2,14183:up|Kitl,17311:up|Prl2c2,18811:up|Efemp1,216616:up|Inhba,16323:down|Rasl11a,68895:up|Bnc2,242509:down|Ccl3,20302:up|Vdr,22337:down|Fabp4,11770:up|Vegfa,22339:down|Igf2,16002:up|Slc40a1,53945:up|Pparg,19016:up|Gas6,14456:up|Tnf,21926:up|Glp1r,14652:down|Lmcd1,30937:up|Fgfr1,14182:down|Egfr,13649:down|Has2,15117:down|Hmox1,15368:up|Sfrp1,20377:up|Sulf1,240725:down|Tgfbr3,21814:up|Grem1,23892:up|Phf11d,219132:up|Sfrp2,20319:up|Tbx15,21384:down|Npnt,114249:up|Enpp1,18605:down|S100a1,20193:up</td></tr>
<tr><td>regulation of regulated secretory pathway</td><td>GO:1903305</td><td>8.37E-01</td><td>8.84E-01</td><td>Hmox1,15368:up</td></tr>
<tr><td>establishment of organelle localization</td><td>GO:0051656</td><td>8.38E-01</td><td>8.85E-01</td><td>Cd24a,12484:down|Hmox1,15368:up|Syt13,80976:up|Fgfr2,14183:up</td></tr>
<tr><td>protein catabolic process</td><td>GO:0030163</td><td>8.38E-01</td><td>8.85E-01</td><td>Tnf,21926:up|Plk2,20620:down|Egfr,13649:down|Wnt5a,22418:down|Ceacam1,26365:up|Uchl5,56207:up|Hpse,15442:up|Sdf2l1,64136:up|Serpine2,20720:up|Adamts12,239337:down</td></tr>
<tr><td>regulation of circadian rhythm</td><td>GO:0042752</td><td>8.40E-01</td><td>8.86E-01</td><td>Pparg,19016:up</td></tr>
<tr><td>negative regulation of cell cycle phase transition</td><td>GO:1901988</td><td>8.40E-01</td><td>8.86E-01</td><td>Susd2,71733:up</td></tr>
<tr><td>innate immune response-activating signal transduction</td><td>GO:0002758</td><td>8.40E-01</td><td>8.86E-01</td><td>Tnf,21926:up</td></tr>
<tr><td>membrane fusion</td><td>GO:0061025</td><td>8.40E-01</td><td>8.86E-01</td><td>Gas6,14456:up|Syt13,80976:up</td></tr>
</table>

regulation of defense response to virus by host	GO:0050691	8.43E-01	8.89E-01	Lmcd1,30937:up
action potential	GO:0001508	8.45E-01	8.91E-01	Tnf,21926:up
regulation of response to nutrient levels	GO:0032107	8.45E-01	8.91E-01	Hmox1,15368:up
regulation of response to extracellular stimulus	GO:0032104	8.45E-01	8.91E-01	Hmox1,15368:up
sensory perception of smell	GO:0007608	8.45E-01	8.91E-01	Igf1,16000:up
microtubule organizing center organization	GO:0031023	8.45E-01	8.91E-01	Plk2,20620:down
regulation of proteolysis involved in cellular protein catabolic process	GO:1903050	8.47E-01	8.92E-01	Uchl5,56207:up|Plk2,20620:down
single-organism membrane organization	GO:0044802	8.49E-01	8.94E-01	Tnf,21926:up|Gas6,14456:up|Cd24a,12484:down|Ccl2,20296:up|Adipoq,11450:up|Col5a1,12831:down|Tgfb2,21808:down|Lrrc15,74488:down|Syt13,80976:up
protein homooligomerization	GO:0051260	8.51E-01	8.96E-01	Hmox1,15368:up|Dpysl3,22240:down|Adipoq,11450:up
signal transduction by p53 class mediator	GO:0072331	8.51E-01	8.96E-01	Ankrd1,107765:down
positive regulation of cellular protein catabolic process	GO:1903364	8.54E-01	8.98E-01	Plk2,20620:down
mitotic cell cycle phase transition	GO:0044772	8.54E-01	8.98E-01	Plk2,20620:down|Egfr,13649:down|Inhba,16323:down
regulation of translation	GO:0006417	8.56E-01	9.00E-01	Tnf,21926:up|Egfr,13649:down|Igf1,16000:up
protein modification by small protein removal	GO:0070646	8.56E-01	9.00E-01	Uchl5,56207:up
membrane organization	GO:0061024	8.58E-01	9.01E-01	Tnf,21926:up|Pparg,19016:up|Gas6,14456:up|Cd24a,12484:down|Ccl2,20296:up|Adipoq,11450:up|Col5a1,12831:down|Tgfb2,21808:down|Lrrc15,74488:down|Syt13,80976:up
activation of innate immune response	GO:0002218	8.59E-01	9.02E-01	Tnf,21926:up
secondary alcohol metabolic process	GO:1902652	8.59E-01	9.02E-01	Fgfr1,14182:down
neuromuscular process	GO:0050905	8.62E-01	9.03E-01	Aldh1a3,56847:down
sphingolipid metabolic process	GO:0006665	8.69E-01	9.10E-01	Serinc2,230779:down
positive regulation of organelle organization	GO:0010638	8.71E-01	9.12E-01	Tnf,21926:up|Lmcd1,30937:up|Wnt5a,22418:down|Sfrp4,20379:up|Igf1,16000:up|Plod2,26432:down|Vegfa,22339:down|Igf2,16002:up
calcium ion-dependent exocytosis	GO:0017156	8.71E-01	9.12E-01	Syt13,80976:up
regulation of protein stability	GO:0031647	8.73E-01	9.13E-01	Plpp3,67916:up|Igf1,16000:up
negative regulation of organelle organization	GO:0010639	8.75E-01	9.15E-01	Fgfr2,14183:up|Igf1,16000:up|Bmp4,12159:up
regulation of cell cycle phase transition	GO:1901987	8.78E-01	9.18E-01	Egfr,13649:down|Susd2,71733:up
nuclear export	GO:0051168	8.81E-01	9.20E-01	Gas6,14456:up
protein tetramerization	GO:0051262	8.81E-01	9.20E-01	Cbr2,12409:up
actin filament bundle assembly	GO:0051017	8.81E-01	9.20E-01	Dpysl3,22240:down
RNA metabolic process	GO:0016070	8.81E-01	9.20E-01	Fstl3,83554:down|Ice1,218333:down|Tfec,21426:up|Sfrp4,20379:up|Ednrb,13618:up|Epas1,13819:up|Ankrd1,107765:down|Htra3,78558:down|Uchl5,56207:up|Lyl1,17095:up|Sdpr,20324:down|Loxl2,94352:down|Loxl3,16950:down|Trf,22041:up|Wnt5a,22418:down|Bmpr1a,12166:down|Ccl4,20303:up|Adipoq,11450:up|Bmp4,12159:up|Igf1,16000:up|Ret,19713:up|Plpp3,67916:up|Creb3l1,26427:down|Fgfr2,14183:up|Prl2c2,18811:up|Efemp1,216616:up|Inhba,16323:down|Rasl11a,68895:up|Bnc2,242509:down|Ccl3,20302:up|Vdr,22337:down|Fabp4,11770:up|Vegfa,22339:down|Oas3,246727:up|Igf2,16002:up|Slc40a1,53945:up|Pparg,19016:up|Gas6,14456:up|Tnf,21926:up|Glp1r,14652:down|Lmcd1,30937:up|Fgfr1,14182:down|Egfr,13649:down|Hmox1,15368:up|Sfrp1,20377:up|Sulf1,240725:down|Tgfbr3,21814:up|Grem1,23892:up|Phf11d,219132:up|Sfrp2,20319:up|Tbx15,21384:down|Npnt,114249:up|S100a1,20193:up
feeding behavior	GO:0007631	8.83E-01	9.22E-01	Glp1r,14652:down
spindle organization	GO:0007051	8.85E-01	9.24E-01	Plk2,20620:down
regulation of cellular protein catabolic process	GO:1903362	8.85E-01	9.24E-01	Uchl5,56207:up|Plk2,20620:down
immune response-regulating cell surface receptor signaling pathway	GO:0002768	8.87E-01	9.26E-01	Clec4e,56619:up

cofactor biosynthetic process	GO:0051188	8.89E-01	9.27E-01	Pdxk,216134:up
macromolecular complex subunit organization	GO:0043933	8.90E-01	9.28E-01	Fam101b,76566:down|P4ha1,18451:down|Col5a2,12832:down|Ice1,218333:down|Pdgfc,54635:down|Dpysl3,22240:down|Mgp,17313:up|Serpinh1,12406:down|Shroom4,208431:down|Vegfa,22339:down|Cbr2,12409:up|Tnf,21926:up|Loxl2,94352:down|Plk2,20620:down|Col1a2,12843:down|Trf,22041:up|Cd24a,12484:down|Col11a1,12814:down|Ncam1,17967:down|Hmox1,15368:up|Lpl,16956:up|C1qtnf3,81799:down|Tgfbr3,21814:up|C1qtnf6,72709:down|Grem1,23892:up|Adipoq,11450:up|Col3a1,12825:down|Sfrp2,20319:up|Col5a1,12831:down|Tgfb2,21808:down
regulation of histone modification	GO:0031056	8.91E-01	9.29E-01	Vegfa,22339:down
nuclear division	GO:0000280	8.92E-01	9.30E-01	Tnf,21926:up|Wnt5a,22418:down|Igf2,16002:up|Fgfr2,14183:up|Igf1,16000:up|Bmp4,12159:up
negative regulation of translation	GO:0017148	8.93E-01	9.30E-01	Egfr,13649:down
regulation of cellular amide metabolic process	GO:0034248	8.95E-01	9.32E-01	Tnf,21926:up|Egfr,13649:down|Igf1,16000:up
protein complex disassembly	GO:0043241	8.99E-01	9.35E-01	Tnf,21926:up
vesicle fusion	GO:0006906	9.01E-01	9.37E-01	Syt13,80976:up
immune response-regulating signaling pathway	GO:0002764	9.01E-01	9.37E-01	Tnf,21926:up|Clec4e,56619:up
cytoskeleton organization	GO:0007010	9.01E-01	9.37E-01	Fam101b,76566:down|Tnf,21926:up|Plk2,20620:down|Trf,22041:up|Sfrp1,20377:up|Ptk7,71461:down|Ccl2,20296:up|Ccl12,20293:up|Dpysl3,22240:down|Mark1,226778:up|Gpm6b,14758:up|Shroom4,208431:down|Tgfb2,21808:down
exocytosis	GO:0006887	9.01E-01	9.37E-01	Ncam1,17967:down|Hmox1,15368:up|Syt13,80976:up
retina development in camera-type eye	GO:0060041	9.02E-01	9.38E-01	Vegfa,22339:down
cellular response to DNA damage stimulus	GO:0006974	9.03E-01	9.39E-01	Ankrd1,107765:down|Uchl5,56207:up|Tnf,21926:up|Egfr,13649:down|Cxcl12,20315:up|Hmox1,15368:up|Nabp1,109019:down
organelle membrane fusion	GO:0090174	9.04E-01	9.39E-01	Syt13,80976:up
cellular potassium ion transport	GO:0071804	9.06E-01	9.41E-01	Kcnj15,16516:down
potassium ion transmembrane transport	GO:0071805	9.06E-01	9.41E-01	Kcnj15,16516:down
protein complex biogenesis	GO:0070271	9.07E-01	9.42E-01	Tnf,21926:up|Ice1,218333:down|Col1a2,12843:down|Cd24a,12484:down|Ncam1,17967:down|Hmox1,15368:up|C1qtnf3,81799:down|Tgfbr3,21814:up|C1qtnf6,72709:down|Pdgfc,54635:down|Dpysl3,22240:down|Mgp,17313:up|Adipoq,11450:up|Vegfa,22339:down|Cbr2,12409:up
protein complex assembly	GO:0006461	9.07E-01	9.42E-01	Tnf,21926:up|Ice1,218333:down|Col1a2,12843:down|Cd24a,12484:down|Ncam1,17967:down|Hmox1,15368:up|C1qtnf3,81799:down|Tgfbr3,21814:up|C1qtnf6,72709:down|Pdgfc,54635:down|Dpysl3,22240:down|Mgp,17313:up|Adipoq,11450:up|Vegfa,22339:down|Cbr2,12409:up
negative regulation of cellular amide metabolic process	GO:0034249	9.08E-01	9.42E-01	Egfr,13649:down
nucleic acid metabolic process	GO:0090304	9.08E-01	9.42E-01	Fstl3,83554:down|Ice1,218333:down|Tfec,21426:up|Sfrp4,20379:up|Pdgfc,54635:down|Ednrb,13618:up|Epas1,13819:up|Ankrd1,107765:down|Htra3,78558:down|Uchl5,56207:up|Lyl1,17095:up|Gstt1,14871:up|Sdpr,20324:down|Igfbp3,16009:up|Loxl2,94352:down|Loxl3,16950:down|Trf,22041:up|Wnt5a,22418:down|Bmpr1a,12166:down|Ccl4,20303:up|Adipoq,11450:up|Bmp4,12159:up|Igf1,16000:up|Ret,19713:up|Plpp3,67916:up|Creb3l1,26427:down|Fgfr2,14183:up|Kitl,17311:up|Prl2c2,18811:up|Efemp1,216616:up|Inhba,16323:down|Rasl11a,68895:up|Bnc2,242509:down|Ccl3,20302:up|Vdr,22337:down|Fabp4,11770:up|Nabp1,109019:down|Vegfa,22339:down|Oas3,246727:up|Igf2,16002:up|Slc40a1,53945:up|Pparg,19016:up|Gas6,14456:up|Tnf,21926:up|Glp1r,14652:down|Lmcd1,30937:up|Fgfr1,14182:down|Egfr,13649:down|Hmox1,15368:up|Sfrp1,20377:up|Sulf1,240725:down|Tgfbr3,21814:up|Tfrc,22042:up|Grem1,23892:up|Phf11d,219132:up|Sfrp2,20319:up|Tbx15,21384:down|Npnt,114249:up|S100a1,20193:up
regulation of dendrite development	GO:0050773	9.09E-01	9.43E-01	Plk2,20620:down
regulation of chromatin modification	GO:1903308	9.09E-01	9.43E-01	Vegfa,22339:down
regulation of microtubule cytoskeleton organization	GO:0070507	9.13E-01	9.46E-01	Plk2,20620:down
positive regulation of chromosome organization	GO:2001252	9.13E-01	9.46E-01	Vegfa,22339:down
nucleobase-containing compound transport	GO:0015931	9.14E-01	9.48E-01	Slc29a1,63959:up
macromolecular complex disassembly	GO:0032984	9.16E-01	9.49E-01	Tnf,21926:up
regulation of proteasomal ubiquitin-dependent protein catabolic process	GO:0032434	9.19E-01	9.52E-01	Plk2,20620:down
regulation of chromatin organization	GO:1902275	9.19E-01	9.52E-01	Vegfa,22339:down

gene silencing	GO:0016458	9.20E-01	9.52E-01	Egfr,13649:down
organelle localization	GO:0051640	9.26E-01	9.56E-01	Cd24a,12484:down|Hmox1,15368:up|Syt13,80976:up|Fgfr2,14183:up
membrane lipid metabolic process	GO:0006643	9.30E-01	9.60E-01	Serinc2,230779:down
organelle fission	GO:0048285	9.30E-01	9.60E-01	Tnf,21926:up|Wnt5a,22418:down|Igf2,16002:up|Fgfr2,14183:up|Igf1,16000:up|Bmp4,12159:up
vesicle organization	GO:0016050	9.32E-01	9.61E-01	Serpine2,20720:up|Syt13,80976:up
double-strand break repair	GO:0006302	9.32E-01	9.62E-01	Nabp1,109019:down
macromolecule catabolic process	GO:0009057	9.39E-01	9.68E-01	Tnf,21926:up|Plk2,20620:down|Egfr,13649:down|Wnt5a,22418:down|Ceacam1,26365:up|Uchl5,56207:up|Hpse,15442:up|Sdf2l1,64136:up|Igfbp3,16009:up|Serpine2,20720:up|Adamts12,239337:down
regulation of actin cytoskeleton organization	GO:0032956	9.41E-01	9.69E-01	Gpm6b,14758:up|Tgfb2,21808:down
mitochondrion organization	GO:0007005	9.43E-01	9.71E-01	Lmcd1,30937:up|Cd24a,12484:down|Plod2,26432:down|Sfrp4,20379:up|Igf1,16000:up|Epas1,13819:up
regulation of microtubule-based process	GO:0032886	9.44E-01	9.72E-01	Plk2,20620:down
skeletal muscle tissue development	GO:0007519	9.44E-01	9.72E-01	Ankrd1,107765:down
cellular amide metabolic process	GO:0043603	9.45E-01	9.72E-01	Ggt5,23887:up|Tnf,21926:up|Egfr,13649:down|Mme,17380:up|Mmp13,17386:up|Igf1,16000:up|Gstm1,14862:up|Gstt1,14871:up|Anpep,16790:up
single-organism membrane fusion	GO:0044801	9.48E-01	9.75E-01	Syt13,80976:up
meiotic nuclear division	GO:0007126	9.50E-01	9.76E-01	Wnt5a,22418:down
skeletal muscle organ development	GO:0060538	9.50E-01	9.76E-01	Ankrd1,107765:down
regulation of organelle organization	GO:0033043	9.50E-01	9.77E-01	Tnf,21926:up|Plk2,20620:down|Lmcd1,30937:up|Wnt5a,22418:down|Sfrp4,20379:up|Igf1,16000:up|Bmp4,12159:up|Gpm6b,14758:up|Plod2,26432:down|Vegfa,22339:down|Tgfb2,21808:down|Fgfr2,14183:up|Igf2,16002:up
organelle fusion	GO:0048284	9.51E-01	9.77E-01	Syt13,80976:up
gene expression	GO:0010467	9.53E-01	9.79E-01	Fstl3,83554:down|Ice1,218333:down|Tfec,21426:up|Sfrp4,20379:up|Aldh1a2,19378:up|C3,12266:up|Ednrb,13618:up|Epas1,13819:up|Ankrd1,107765:down|Htra3,78558:down|Serpinh1,12406:down|Uchl5,56207:up|Lyl1,17095:up|Sdpr,20324:down|Loxl2,94352:down|Loxl3,16950:down|Trf,22041:up|Wnt5a,22418:down|Bmpr1a,12166:down|Ccl4,20303:up|Adipoq,11450:up|Bmp4,12159:up|Igf1,16000:up|Tnc,21923:down|Ret,19713:up|Tgfb2,21808:down|Plpp3,67916:up|Creb3l1,26427:down|F3,14066:up|Fgfr2,14183:up|Prl2c2,18811:up|Efemp1,216616:up|Inhba,16323:down|Rasl11a,68895:up|Bnc2,242509:down|Plat,18791:down|Ccl3,20302:up|Vdr,22337:down|Ctla2a,13024:down|Fabp4,11770:up|Vegfa,22339:down|Serpine2,20720:up|Igf2,16002:up|Slc40a1,53945:up|Pparg,19016:up|Gas6,14456:up|Tnf,21926:up|Glp1r,14652:down|Lmcd1,30937:up|Fgfr1,14182:down|Egfr,13649:down|Hmox1,15368:up|Sfrp1,20377:up|Sulf1,240725:down|Tgfbr3,21814:up|Grem1,23892:up|Phf11d,219132:up|Sfrp2,20319:up|Tbx15,21384:down|Serping1,12258:up|Npnt,114249:up|S100a1,20193:up
meiotic cell cycle process	GO:1903046	9.56E-01	9.82E-01	Wnt5a,22418:down
proteasomal protein catabolic process	GO:0010498	9.57E-01	9.83E-01	Uchl5,56207:up|Plk2,20620:down|Sdf2l1,64136:up
cell cycle	GO:0007049	9.60E-01	9.85E-01	Hpgd,15446:up|Inhba,16323:down|Nabp1,109019:down|Igf2,16002:up|Tnf,21926:up|Plk2,20620:down|Egfr,13649:down|Fgfr1,14182:down|Sfrp1,20377:up|Susd2,71733:up|Wnt5a,22418:down|Ptgs2,19225:down|Bmp4,12159:up|Igf1,16000:up|Tgfb2,21808:down|Fgfr2,14183:up
regulation of mitotic cell cycle phase transition	GO:1901990	9.63E-01	9.88E-01	Egfr,13649:down
DNA repair	GO:0006281	9.64E-01	9.88E-01	Uchl5,56207:up|Egfr,13649:down|Nabp1,109019:down
regulation of protein ubiquitination	GO:0031396	9.65E-01	9.89E-01	Nxn,18230:down
regulation of gene expression, epigenetic	GO:0040029	9.67E-01	9.90E-01	Egfr,13649:down
sensory perception of chemical stimulus	GO:0007606	9.68E-01	9.91E-01	Igf1,16000:up
Golgi vesicle transport	GO:0048193	9.68E-01	9.91E-01	Sec23a,20334:down
regulation of cytoskeleton organization	GO:0051493	9.70E-01	9.94E-01	Plk2,20620:down|Gpm6b,14758:up|Tgfb2,21808:down
protein dephosphorylation	GO:0006470	9.74E-01	9.96E-01	Tnf,21926:up
immune response-activating signal transduction	GO:0002757	9.76E-01	9.99E-01	Tnf,21926:up
dendrite development	GO:0016358	9.76E-01	9.99E-01	Plk2,20620:down

regulation of protein modification by small protein conjugation or removal	GO:1903320	9.77E-01	9.99E-01	Nxn,18230:down
meiotic cell cycle	GO:0051321	9.78E-01	9.99E-01	Wnt5a,22418:down
macromolecular complex assembly	GO:0065003	9.81E-01	1.00E+00	Tnf,21926:up|Ice1,218333:down|Col1a2,12843:down|Cd24a,12484:down|Ncam1,17967:down|Hmox1,15368:up|C1qtnf3,81799:down|Tgfbr3,21814:up|C1qtnf6,72709:down|Pdgfc,54635:down|Dpysl3,22240:down|Mgp,17313:up|Adipoq,11450:up|Vegfa,22339:down|Cbr2,12409:up
proteasome-mediated ubiquitin-dependent protein catabolic process	GO:0043161	9.82E-01	1.00E+00	Plk2,20620:down|Sdf2l1,64136:up
vacuolar transport	GO:0007034	9.82E-01	1.00E+00	Fgfr1,14182:down
cellular component assembly	GO:0022607	9.84E-01	1.00E+00	Prl2c2,18811:up|Ice1,218333:down|Flrt2,399558:down|Plxnd1,67784:up|Pdgfc,54635:down|Dpysl3,22240:down|Mgp,17313:up|Srpx2,68792:down|Gpm6b,14758:up|Vegfa,22339:down|Cbr2,12409:up|Tnf,21926:up|Col1a2,12843:down|Plk2,20620:down|Cd24a,12484:down|Hmox1,15368:up|Has2,15117:down|Ncam1,17967:down|C1qtnf3,81799:down|Wnt5a,22418:down|Tgfbr3,21814:up|C1qtnf6,72709:down|Grem1,23892:up|Adipoq,11450:up|Thbs2,21826:down
histone modification	GO:0016570	9.85E-01	1.00E+00	Loxl2,94352:down|Vegfa,22339:down
cell projection assembly	GO:0030031	9.86E-01	1.00E+00	Prl2c2,18811:up|Dpysl3,22240:down
cellular protein complex assembly	GO:0043623	9.86E-01	1.00E+00	Ncam1,17967:down|Tgfbr3,21814:up|Pdgfc,54635:down
peptide biosynthetic process	GO:0043043	9.87E-01	1.00E+00	Ggt5,23887:up|Tnf,21926:up|Egfr,13649:down|Igf1,16000:up
regulation of chromosome organization	GO:0033044	9.88E-01	1.00E+00	Vegfa,22339:down
cellular component biogenesis	GO:0044085	9.90E-01	1.00E+00	Prl2c2,18811:up|Ice1,218333:down|Flrt2,399558:down|Plxnd1,67784:up|Pdgfc,54635:down|Dpysl3,22240:down|Mgp,17313:up|Srpx2,68792:down|Gpm6b,14758:up|Vegfa,22339:down|Cbr2,12409:up|Serpine2,20720:up|Tnf,21926:up|Plk2,20620:down|Col1a2,12843:down|Cd24a,12484:down|Hmox1,15368:up|Has2,15117:down|Ncam1,17967:down|C1qtnf3,81799:down|Wnt5a,22418:down|Tgfbr3,21814:up|C1qtnf6,72709:down|Grem1,23892:up|Adipoq,11450:up|Sdf2l1,64136:up|Thbs2,21826:down
proteolysis involved in cellular protein catabolic process	GO:0051603	9.93E-01	1.00E+00	Uchl5,56207:up|Plk2,20620:down|Sdf2l1,64136:up|Adamts12,239337:down
cellular macromolecule catabolic process	GO:0044265	9.93E-01	1.00E+00	Uchl5,56207:up|Plk2,20620:down|Sdf2l1,64136:up|Hpse,15442:up|Igfbp3,16009:up|Adamts12,239337:down
ubiquitin-dependent protein catabolic process	GO:0006511	9.93E-01	1.00E+00	Uchl5,56207:up|Plk2,20620:down|Sdf2l1,64136:up
modification-dependent protein catabolic process	GO:0019941	9.94E-01	1.00E+00	Uchl5,56207:up|Plk2,20620:down|Sdf2l1,64136:up
modification-dependent macromolecule catabolic process	GO:0043632	9.95E-01	1.00E+00	Uchl5,56207:up|Plk2,20620:down|Sdf2l1,64136:up
microtubule cytoskeleton organization	GO:0000226	9.95E-01	1.00E+00	Plk2,20620:down|Mark1,226778:up
cellular protein catabolic process	GO:0044257	9.95E-01	1.00E+00	Uchl5,56207:up|Plk2,20620:down|Sdf2l1,64136:up|Adamts12,239337:down
amide biosynthetic process	GO:0043604	9.95E-01	1.00E+00	Ggt5,23887:up|Tnf,21926:up|Egfr,13649:down|Igf1,16000:up
protein ubiquitination	GO:0016567	9.96E-01	1.00E+00	Neurl3,214854:down|Pdzrn3,55983:down|Nxn,18230:down
translation	GO:0006412	9.96E-01	1.00E+00	Tnf,21926:up|Egfr,13649:down|Igf1,16000:up
covalent chromatin modification	GO:0016569	9.97E-01	1.00E+00	Loxl2,94352:down|Vegfa,22339:down
protein modification by small protein conjugation or removal	GO:0070647	9.98E-01	1.00E+00	Uchl5,56207:up|Neurl3,214854:down|Pdzrn3,55983:down|Nxn,18230:down
protein modification by small protein conjugation	GO:0032446	9.98E-01	1.00E+00	Neurl3,214854:down|Pdzrn3,55983:down|Nxn,18230:down
chromatin modification	GO:0016568	9.99E-01	1.00E+00	Loxl2,94352:down|Vegfa,22339:down
ncRNA metabolic process	GO:0034660	9.99E-01	1.00E+00	Ice1,218333:down
organelle assembly	GO:0070925	9.99E-01	1.00E+00	Tnf,21926:up|Plk2,20620:down
chromatin organization	GO:0006325	9.99E-01	1.00E+00	Loxl2,94352:down|Vegfa,22339:down
microtubule-based process	GO:0007017	######	1.00E+00	Plk2,20620:down|Mark1,226778:up

cellular macromolecular complex assembly	GO:0034622	######	1.00E+00	Ncam1,17967:down|Tgfbr3,21814:up|Pdgfc,54635:down
single-organism organelle organization	GO:1902589	######	1.00E+00	Fam101b,76566:down|Ptk7,71461:down|Sfrp4,20379:up|Dpysl3,22240:down|Gpm6b,14758:up|Shroom4,208431:down|Plod2,26432:down|Vegfa,22339:down|Igf2,16002:up|Tnf,21926:up|Loxl2,94352:down|Trf,22041:up|Lmcd1,30937:up|Plk2,20620:down|Sfrp1,20377:up|Mark1,226778:up|Bmp4,12159:up|Igf1,16000:up|Tgfb2,21808:down|Fgfr2,14183:up|Syt13,80976:up
chromosome organization	GO:0051276	######	1.00E+00	Loxl2,94352:down|Vegfa,22339:down
RNA processing	GO:0006396	######	1.00E+00	Egfr,13649:down
organelle organization	GO:0006996	######	1.00E+00	Fam101b,76566:down|Ptk7,71461:down|Sfrp4,20379:up|Ccl2,20296:up|Dpysl3,22240:down|Epas1,13819:up|Gpm6b,14758:up|Shroom4,208431:down|Plod2,26432:down|Vegfa,22339:down|Serpine2,20720:up|Igf2,16002:up|Tnf,21926:up|Loxl2,94352:down|Plk2,20620:down|Trf,22041:up|Lmcd1,30937:up|Cd24a,12484:down|Sfrp1,20377:up|Wnt5a,22418:down|Ccl12,20293:up|Mark1,226778:up|Bmp4,12159:up|Igf1,16000:up|Tgfb2,21808:down|Fgfr2,14183:up|Syt13,80976:up
